# Deciphering viral presences: two novel partial giant viruses detected in marine metagenome and in a mine drainage metagenome

**DOI:** 10.1186/s12985-018-0976-9

**Published:** 2018-04-10

**Authors:** Julien Andreani, Jonathan Verneau, Didier Raoult, Anthony Levasseur, Bernard La Scola

**Affiliations:** Aix-Marseille Université, IRD, APHM, MEPHI, IHU Méditerranée Infection, 19-21 Bd Jean Moulin, 13005 Marseille, France

**Keywords:** Pithovirus, Cedratvirus, Orpheovirus, Mine drainage metagenome, Marine metagenome, MG-digger, Misidentifiedvirus

## Abstract

Nucleo-cytoplasmic large DNA viruses are doubled stranded DNA viruses capable of infecting eukaryotic cells. Since the discovery of Mimivirus and Pandoravirus, there has been no doubt about their extraordinary features compared to “classic” viruses. Recently, we reported the expansion of the proposed family *Pithoviridae*, with the description of Cedratvirus and Orpheovirus, two new viruses related to Pithoviruses. Studying the major capsid protein of Orpheovirus, we detected a homologous sequence in a mine drainage metagenome. The in-depth exploration of this metagenome, using the MG-Digger program, enabled us to retrieve up to 10 contigs with clear evidence of viral sequences. Moreover, phylogenetic analyses further extended our screening with the discovery in another marine metagenome of a second virus closely related to Orpheovirus IHUMI-LCC2. This virus is a misidentified virus confused with and annotated as a Rickettsiales bacterium. It presents a partial genome size of about 170 kbp.

**Electronic supplementary material:**

The online version of this article (10.1186/s12985-018-0976-9) contains supplementary material, which is available to authorized users.

## Main text

Metagenomic analysis is a powerful method to detect micro-organisms in their ecosystems. These micro-organisms belong to all the components of the tree of life but also to those of the viral world. The commitment to be totally independent from the culture process constitutes an extremely important part of its success [1, 2]. These techniques have pushed researchers to explore different environments, or microbiota as in the Human Microbiome Project [3], and sometimes multiplying samples collection in various environments such as the international space station or as the Permafrost in “omics” study [4, 5]. Nevertheless, a wide part of these metagenomic results are still unknown in database, and is referred to as “dark matter” [6]. On the opposite side, the culture tools and notably some culturomics studies allows us to describe and characterize isolates, which is impossible by the use of metagenomic analysis alone [7]. This confirms the extreme complementary of both methods.

On the other hand, giant viruses are double stranded DNA viruses described as potentially comparable to certain bacteria by their genomic length and their particle size [8, 9]. Based on the reconstructions of ancestral sequences of viral RNA polymerase II subunits as baits, successful studies were carried out to identify individual sequences as well as partial or near complete genomes [10–13]. Finally, each new viral description which came from culture isolation allows us to retrospectively better understand unknown old or new metagenomic reports and finally permits to better decipher the “dark matter”. Recently, we characterised a new giant virus named Orpheovirus and its growth on the amoeba *Vermamoeba vermiformis* [14]. The gene ORPV_1034 was predicted to encode a 587 amino-acid viral major capsid protein (MCP). With the goal of investigating homologies in database to increase efficiency of our annotation we used, with standard parameter (i.e. e-value inclusion threshold 1e-3), an online software (HHblits) able to detect homologies and structure. The result showed 65 hits but only 2 proteins matched with a special interest (Additional file 1). The probability obtained was at 97,72% with a length of 302 amino-acids between Orpheovirus and *Pithovirus sibericum* and it was at 97,04% with alignments of 237 amino-acids between Orpheovirus and unknown sequence present in a metagenome. Then, using PSI-BLAST against metagenomes database, we also detected homologies between Orpheovirus and Cedratvirus A11 (55% coverage, 29%identity) as well as with *Pithovirus sibericum* (41% coverage, 21% identity). PSI-BLAST against metagenomes database of the Orpheovirus’s MCP detected an homology with the same protein detected by HHblits (47% coverage, 24%identity) with the sequence EQD26795.1 and also, with 2 additional proteins issued from different marine metagenomes [15, 16]. We observed that the best hit with this viral capsid came from an acid mine drainage metagenome located in Spain.

We decided to investigate this pyrite mine using MG-Digger program [17] with updated giant viruses database (e.g Pithovirus massiliensis LC8, Pacmanvirus A23, Cedratvirus A11, Cedratvirus lausannensis, Orpheovirus IHUMI-LCC2, Kaumoebavirus and Klosneuvirinae). Projects have been previously registered [18] under the IDs PRJNA193663 sample (B1A), PRJNA193664 (sample B1B) and PRJNA193665 (sample B2A) at NCBI. This Whole Genome Shotgun project has been deposited under the accession AUZX00000000- AUZY00000000-AUZZ00000000. The parameters used for MG-Digger were fixed with an e-value cut-off of 10^− 3^ and no limit of both coverage and identity percent are defined to detect giant viruses-like sequences in the alignment of the BLAST options. All contigs available in the mine drainage metagenome represent 41,233 contigs. The MG-Digger program accepts protein or nucleic acid sequences in input [17] and in this case, we used nucleic acid contigs in input against updated database.

We detected and revealed 10 contigs from the series AUZX and AUZY as showing best hit informing the viral presence (samples B1A and B1B) (Table 1, Additional file 2) with no doubt that their origins are viral. Indeed, they presented a score higher than 60 for all queries with an average coverage of 79,2% (ranging from 53 to 99%), e-value inferior at 10^− 7^ and identity with an average of about 38% (ranging from 27% to 48%). Each contig detected encode for one putative protein. To complete the annotation, all proteins corresponding to each viral contig were downloaded and all annotations were confirmed by DELTA-BLASTP program [19]. Six viral contigs retrieved as best hit proteins from the members of the proposed family *Pithoviridae* (Pithoviruses and Cedratviruses) and Orpheovirus. Detailed annotation of these 6 proteins encoded by these contigs revealed 1 DNA polymerase B family, 1 DNA topoisomerase IIA, 1 mRNA capping enzyme, 1, ribonucleoside-diphosphate reductase, 1 Very Early Transcription Factor (VETF) and the previously found MCP. Moreover, we found 2 putative Ankyrin-repeat proteins matching with Mimiviruses, and one hypothetical protein and one protein coding for D5 helicase primase protein present best hits with Iridoviruses (Table 1, Additional file 2).

**Table 1:** Resume of 10 best hit obtained in the mine metagenome using our viral database. Contigs in the first column were used to the blastx, equivalent proteins are indicated in the fourth column, in the middle (second and third columns) we added the annotation and the best hit obtained with MG-Digger. Blast results are available in supplementary data

Contig query	Match as best hit	Annotation	Protein accession member
AUZX01005870.1 contig18488	*Acanthamoeba polyphaga mimivirus*	ankyrin repeat protein	EQD66250.1
AUZX01007984.1 contig21683	*Acanthamoeba polyphaga mimivirus*	ankyrin repeat protein	EQD57210.1
AUZX01014511.1 contig07869	*Cedratvirus A11*	7-methylguanosine mRNA capping	EQD31999.1
AUZX01014088.1 contig07382	*Cedratvirus A11*	DNA topoisomerase IIA	EQD33330.1
AUZX01005811.1 contig18401	*Invertebrate iridovirus 25*	hypothetical protein IIV25_070R	EQD66514.1
AUZY01012469.1 contig09137	*Orpheovirus IHUMI-LCC2*	DNA polymerase B family	EQD29730.1
AUZY01012943.1 contig09710	*Pithovirus sibericum*	Ribonucleoside-diphosphate reductase large subunit	EQD27148.1
AUZX01011676.1 contig04641	*Pithovirus massiliensis*	Pithovirus-massiliensis_169 VETF early transcription factor large subunit	EQD42498.1
AUZY01013064.1 contig09855	*Pithovirus massiliensis*	Pithovirus-massiliensis_152 Major capsid protein	EQD26795.1
AUZY01002937.1 contig14672	*Cherax quadricarinatus iridovirus*	037 L D5 primase-helicase	EQD71007.1

Subsequently, phylogenetic analyses were performed at first glance on these predicted proteins (data not shown). Indeed, we retrieved homologous sequences by Blast protein strategy with Orpheovirus, Cedratviruses, Pithoviruses and some of the 9 contigs and also with a Rickettsiales bacterium present in the NCBI database (belonging to another marine metagenome from Atlantic North under the accession number: NVVL00000000.1).

Then, this marine metagenome for which the annotation was performed by automatic pipeline, was further investigated. Amongst 111 contigs, all genes and proteins were predicted de novo using GenemarkS [20], with a blastp analysis of the 1947 predicted proteins followed by GC% assignment of putative viral contigs (Additional files 3 and 4). Indeed, we used an e-value cut-off at 10^− 2^ and our results highlight that some proteins have a best hit (Additional file 3) with viral strains notably with proteins previously described as fundamental proteins in Nucleo-Cytoplasmic-Large DNA viruses (e.g DNA-directed RNA polymerase subunit RPB2, Flap endonuclease 1, transcription initiation factor TFIIB, DNA polymerase delta catalytic subunit). Finally, we identified 15 viral contigs totalizing 177,601 base pairs with a GC % close to 33%. Initially annotated as a Rickettsiales hit, we propose to re-annotate these viral sequences like a Misidentifiedvirus. On the other hand, true Rickettsiales contigs present a GC% more variable around 40% and we identified 11 contigs that could potentially be a ciliate protist close to *Oxytricha spp*. (Additional file 5). This ciliate could be the host of the Misidentifiedvirus as previously described for others protists [12].

**Fig. 1:**
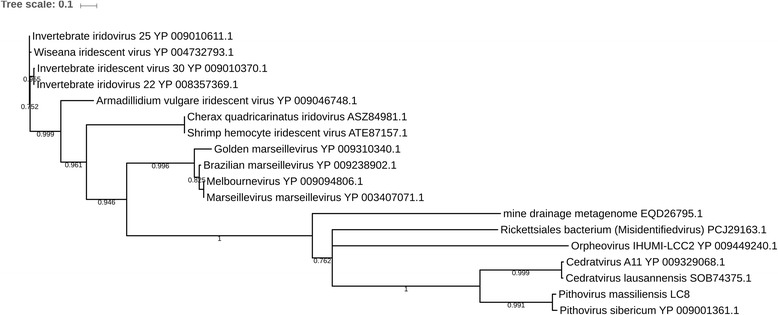
Phylogenetic tree based on the viral major capsid protein. Alignment was performed using Muscle in the MEGA6.0 software. MVIEW [26] was used to visualized the alignment, and after we performed a maximum likelihood tree on the 423 positions with 1000 bootstrap replication with FastTree program (JTT model) with standard parameter. ITolV3 online [22] was used to visualized the tree. Branches under 0.5 as bootstrap value were deleted

For all phylogenetic analyses we used Muscle algorithm [21] to perform alignment. Alignments were curated manually and finally, FastTree program was used to build maximum-likelihood trees with standard program (Jones-Taylor-Thornton model). Then, ITolV3 online was used to visualized trees [22]. Phylogenetic analysis based on the MCP anchors these 2 different viruses from marine and mine metagenomes closely-related to Orpheovirus (Fig. 1, Additional file 6). DNA topoisomerase II, VETF, and mRNA capping enzyme trees confirmed these results (Additional file 7). Nevertheless, due to the partial sequences for the mine drainage, their exact position could not definitively be determined.

Retrieving best hit virus constitute an unambiguous evidence for viral presence, especially when we found structural gene described like hallmark genes in Nucleo-cytoplasmic Large DNA viruses [23, 24]. Altogether, these data confirm a viral presence in this mine pyrite and a Misidentifiedvirus in the marine metagenome as two putative viruses possessing a close relationship with the proposed *Pithoviridae* and *Orpheoviridae* families. The rapid expansion of gigantic dsDNA reports and their genomic descriptions permit to evaluate and re-evaluate published and novel metagenomes. MG-digger program is a functional and appropriate tool to investigate them. Nevertheless, it is limited by the detection of best hit that it confers its confidence. Finally, a complementary method is emerging promoted by the next-generation sequencing. Indeed, the metagenomic binning approach represent a major step in the genome reconstruction from different metagenomes [12]. Such additional approaches could be implemented in future automatic pipelines. There is no doubt that the development of new programs [25] associated with efficient new viral isolation would enable further discoveries and start filling the knowledge gap in the current dark matter content.

## Additional files

Additional file 1: Data sheet about HHblits results obtained for the predicted protein ORPV_1034. 65 hits are mentioned with their probabilities, alignments, e-value and target length. (XLSX 24 kb)

Additional file 2: MG-digger results. The NCBI databases available are up to October 2017. Data sheet contains all queries matching with each identified subjects. We added annotation in the column F. BLAST scores are indicate in columns from G to S. (XLSX 13 kb)

Additional file 3: Blastp result obtained using de novo predicted proteins for the marine metagenome NVVL00000000.1. Data sheet represent all blastp results obtained and extracted specific blast for each contig. We added the annotation of the Misidentifiedvirus in the column H of the datasheet named “Genermarks-111contig”. (XLSX 14868 kb)

Additional file 4: Marine metagenome proteins predicted. GenemarkS was used to predict de novo proteins from 111 “Rickettsiales Bacterium” contigs. (PDF 2040 kb)

Additional file 5: List of NVVL00000000.1 contigs. We identified 15 and 11 contigs among 111 “Rickettsiales” contigs as viruses and as ciliate protist. (PDF 60 kb)

Additional file 6: Protein alignment of Major capsid protein. This alignment was done MUSCLE with standard parameter and was used to build the tree in the Fig. 1. (PDF 104 kb)

Additional file 7: Additional phylogenetic trees. Maximum likelihood trees were added based on VETF, mRNA capping enzyme and DNA topoisomerase II proteins. (PDF 744 kb)

## Abbreviations

MCP: Major capsid protein
VETF: Very Early Transcription Factor
