# Supplementary material for: Deciphering viral presences: two novel partial giant viruses detected in marine metagenome and in a mine drainage metagenome
Source: Virol J. 2018 Apr 10;15:66. doi: 10.1186/s12985-018-0976-9 (PMC5891951; doi:10.1186/s12985-018-0976-9)
Supplement: Supplementary file 4 — Marine metagenome proteins predicted. GenemarkS was used to predict de novo proteins from 111 “Rickettsiales Bacterium” contigs. (PDF 2040 kb) [file 12985_2018_976_MOESM4_ESM.pdf]

>gene_1|GeneMark.hmm|56_aa|+|2|172 >NVVL01000001.1 Rickettsiales bacterium isolate NORP64 Contig_source1382A_460, whole genome shotgun sequence
VNIFAFSMLFILPLIKTGIYFIVLYLYPPKNKHFSSLPLKPEHIRSSFCQQLDKKN

>gene_2|GeneMark.hmm|82_aa|+|231|479 >NVVL01000001.1 Rickettsiales bacterium isolate NORP64 Contig_source1382A_460, whole genome shotgun sequence
MKKWQLQNIKAKLIALVKQASRGKPEQTTAKERAEIVILPAKRYVDLTSSKISLVEFLRN
SPLYGAELDLERNQTPCRDIEL

>gene_3|GeneMark.hmm|98_aa|+|482|778 >NVVL01000001.1 Rickettsiales bacterium isolate NORP64 Contig_source1382A_460, whole genome shotgun sequence
MLQSRANTQKLLHDIKNNGMPKIALKVKAGARQNSINGIIEIDNKPYLKLSIKSSPEKGK
ANKMIIDFLAKEWGMRKSDLTIISGKTSQYKILHIKEP

>gene_4|GeneMark.hmm|196_aa|-|780|1370 >NVVL01000001.1 Rickettsiales bacterium isolate NORP64 Contig_source1382A_460, whole genome shotgun sequence
MTDFSIEDTHPGKIIAGVDEVGRGPLAGPVVTAAVIVDRAVVIEGIKDSKKLSKKKREEL
FEKISLHYKCSVGIISPAEIDRINILEATKKACQMAVRSLEIEPDIVLVDGNMKFSDERF
CSIIKGDDKSVSIAAASIIAKVTRDRMMEELGVEFPEYKWAQNSGYGTKDHIDAIIKHGA
TPHHRRSFRVRGFNYS

>gene_5|GeneMark.hmm|242_aa|-|1363|2091 >NVVL01000001.1 Rickettsiales bacterium isolate NORP64 Contig_source1382A_460, whole genome shotgun sequence
MKFSHLSRIYSTDSIAIGQTITIEGEDFHYLKSVIRLKLGATFRMFNENDGEFMLKITQI
GRSSIIANVESLLRLPALQKDLTLAICVIKQDRMIEAIKAAVQIGVTRIVPVISERVQHR
QNKRDKILKSIIQSTEQSERFKPAEFTEEITLKELCAMDDIEQIIWANETESEGVTIKNI
KQIKGRPCVLIGCEGGFSDAEIQMLKSHDHVQSVSLGHSVLRAEIAAIYALSCTSMMRGV
ND

>gene_6|GeneMark.hmm|48_aa|+|2113|2259 >NVVL01000001.1 Rickettsiales bacterium isolate NORP64 Contig_source1382A_460, whole genome shotgun sequence
MLKKDKSDNTPTNEESSSKNTDEEFGGQNGPDPTRYGDWEKNGRVSDF

>gene_7|GeneMark.hmm|60_aa|-|2350|2532 >NVVL01000001.1 Rickettsiales bacterium isolate NORP64 Contig_source1382A_460, whole genome shotgun sequence
MGVSLVQLLVILLIILLLFGAGKLPRVMSELGKGLKSFKSSMNEDDSKDDSNDDNKNKKG

>gene_8|GeneMark.hmm|431_aa|+|2767|4062 >NVVL01000001.1 Rickettsiales bacterium isolate NORP64 Contig_source1382A_460, whole genome shotgun sequence
MTSKQTPAEKELFMTMAERKIYNKWRLRILISIILGYGTYYLCRLNFSIIMPAYIEEFSY
SKTQLGWVITITSIVYGVGKFVNGYFSDKSNARYFMPAGLILSAIITFILGFTNSLVFLG
IFLIFNYWFQSMGWPPAARMLTHWFAPQELGTKWALGAASHQIGGALTLIFCGYLVANFG
WRYAFFIPGIIAFIVGVILFSALRDSPKELGLPPVEAYKGDEDFKEDTSENHLGSMEIFL
KVFANRNIWYICFANMCVYFIRSGIVVWAPLFLIEFKDFSLVDSGWQVAAYEFAGLPGGF
MAGWISDKLFGGKRGFVGIMFMSLLALALYTFWKLPAEHIYLSASSLACIGFFLSGPQIL
VGLASADFASKKAVGTANGWVGTVGQLGASLSGIGVGLLTQYYGWDAAFMAFIGAAILGA
VFFSLTLKNRA

>gene_9|GeneMark.hmm|178_aa|+|4300|4836 >NVVL01000001.1 Rickettsiales bacterium isolate NORP64 Contig_source1382A_460, whole genome shotgun sequence
MKTSAKQTGFILLGLALIAMIFTLTQMSSATAAKKNGKKFGDWVVSCSPANKKTKTPAVC
VLTQQINMSKDDKQQTVALFQIGYFGKKKDLKLVQTLPFGISIEAGTSIISSKKLIAPGK
YTTCLASGCQAVASISKKDLKTMTSNSENAVAFMSLNGKQITFPISMKGIERGLKYIK

>gene_10|GeneMark.hmm|274_aa|+|5125|5949 >NVVL01000001.1 Rickettsiales bacterium isolate NORP64 Contig_source1382A_460, whole genome shotgun sequence
MQRIFTALFVILTGNVSLASKPEPWQLYLQEAATPVMEELTAFHDFMMIIITGVTLFVFF
LIIYVVLRFNAKANPVPAKFSSNLTVEIAWTLIPVIILIIIAVPSFRILKMAEHTPDSDV
TVKVVGSQWFWTYSYPDHGIEFDSYMIQDEDLLPGQLRLLEVDNRIIVPQGKVVKFLITA
ADVIHSFAVPALGIKLDAVPGRTNETWAKIDKIGVYYGQCSELCGVNHGFMPIAVEVVSQ
KDYDIWVESAKVKFASHNSIKPDAIKMAANIKSE

>gene_11|GeneMark.hmm|524_aa|+|5961|7535 >NVVL01000001.1 Rickettsiales bacterium isolate NORP64 Contig_source1382A_460, whole genome shotgun sequence
MTDHAHHTQTGWRRWLLSTNHKDIGTMYITLAVVGGLVGGLFSVLFRMQLAEPGSNFLGG
DHQFFNVLITAHALIMVFFMIMPVLFGGFGNWFVPLLIGAPDMAFPRMNNISFWLLVPAF
LLLILSAMVDGGVGTGWTLYPPLSGLLGHPGAAVDIAIFSLHLAGISSILGALNMITTIL
NMRAPGMTLLKMPLFVWSILVTAALLILVLPVLAGAITMLLTDRNLGTHFFTPEGGGDPV
LFQHLFWFFGHPEVYVIVLPGFGIVSQIISTFSRKPIFGYGAMVASMVIIGFLGILVWAH
HMFTVGLSYTALVYFTAATMIIAVPTGIKIFSWIATMWGGSITFETPMLFAIGFIVIFTF
GGVTGVVLANSALGRIMHDTYYIVAHFHYTMSLGALFTAFAGFYYWFGKMSGKVYPEWMG
KMHFTLTFIGVNLTFFPQHFLGLAGMPRRIPDYPDAFAGWNYVSSIGSYISYFAALFFVF
IIFYTLKYGKKCPPNQWGEGATTLEWSVDSPAPHHTFETPPTVK

>gene_12|GeneMark.hmm|313_aa|+|7535|8476 >NVVL01000001.1 Rickettsiales bacterium isolate NORP64 Contig_source1382A_460, whole genome shotgun sequence
MTKSTTGHTNSEMGASVGDYFSLMKPRVMSLVVFTAFCGLWLAPGDIHPLIAFVAILCIT
LGAGASAAINMWYDRDIDAIMTRTQKRPIITGAILPDEALSFGIMSGFMSVFLMALCVNL
VASALLALTILYYIFIYTIWLKRTSIQNVVIGGASGALPPMIGWAAVTGDVSLESFTLFA
IIFIWTPPHSWALALFRLQDYRNCAVPMMPVIKGESYTKKQILLYSILMIFVVMMPYFLG
MANILYLGISLILGAGFLYYVAALFQTNENQETETHGAGTQGAETQGAKALFWYSIFYLF
TIFLSLLFLKLPT

>gene_13|GeneMark.hmm|448_aa|+|8581|9927 >NVVL01000001.1 Rickettsiales bacterium isolate NORP64 Contig_source1382A_460, whole genome shotgun sequence
MKKKIIALVGRPNVGKSTLFNRLSTRKKAIVHDRPGVTRDRKYSDAKLGPMEFTVVDTPG
LEEAEEGKLEHRMMQQTMVAIAEADLVCLVIDNEAGITPADKFFAEFIRKYSKKQILIAN
KSERKFNHDSEYYKIGFGTPVPVSAEHGQGLAYLYEAIAEQIDTEDGEEISDPMSGDCLQ
IVISGRPNAGKSTFVNAIVGDERMLTGPEAGLTRESVEIAWEYKGQRVKLIDTAGLRKRA
NVKQSLEKLSTGDAIGSIKFANTVILMIDATIALEHQDLTIANYVIDQGRSLVIAINKWD
LIRKKREYEEEFEYKLQKYLPQVQGVPVVYLSSTDNVKTLEVLDKCLDIYKLWNKKISTS
KLNDWLSFTLEKHPLPIQKKLGRRVRIKYITQTKTRPPTFKLFTSDPGSITEAYRKYLVN
SLRNDFDLPGVPIRLQFIKSDNPYAKKK

>gene_14|GeneMark.hmm|220_aa|+|9927|10589 >NVVL01000001.1 Rickettsiales bacterium isolate NORP64 Contig_source1382A_460, whole genome shotgun sequence
MKSVLLTRSDTENDLLMLRLQESGYETLSCNLIKYELQKVDEAGFKRFKQIIVTSSFAAN
NAPPAHSKGVSAWAVGKKSAKILASKGYEIRFVAPSAESLKDQFVEDTNKPTLYLSSNYI
TIDMPFFVTRCIFYKVTYQDSLTEQQIARYKNGVDYILLYSENSAKTFLKLVKENNLLHC
LENSTIITISSKISKVLADYFKTSVVCNGTDLILEYLKSK

>gene_15|GeneMark.hmm|342_aa|+|10586|11614 >NVVL01000001.1 Rickettsiales bacterium isolate NORP64 Contig_source1382A_460, whole genome shotgun sequence
MTQPKNTKNKKKLDDENVPAKNSPGGQAKKSEEVLEAEVLGEKALEVELLEEDSAQKLEP
RKINYFKKFLLAATILLSWVFVLKQLYPDFLEHFLEDEKAVIITEQPQPESELVKDALNS
SYDPGAEPPLESFAPNNLIEDINEIILPEEEADEENPYEQLVETQCRLHVKLATTKLRDR
LLNNLDDYRLYLANMNQLVIKFKSDKRYEFELKKLSARPLPSYIRDIVSMLELYDRQLAA
NIAKAKANLDGKAGRDGRAALNDSGSVGAEPDLGKDTDLGEMLGLEKKIDLGILSKFIKI
EKISALSEEQKNLKREIEGQLRVLVQYIYSSELQDKFINRVR

>gene_16|GeneMark.hmm|355_aa|+|11615|12682 >NVVL01000001.1 Rickettsiales bacterium isolate NORP64 Contig_source1382A_460, whole genome shotgun sequence
MIRLLILCVIFVLFYIGIGAMEHYDSALKFTALGYHIETTLFAFTAAFVIIQLITMICLK
LVYLMFNLPQIIRNEWQRSSIQKLNRKLLNTLAELLMGNKKKSLGLASKLISSLNDKNKN
VIHLILAELSDNPGKREEYLRNLTTEKHYSIYALKRLAEISYKNMHYKQAEDYALKAFNE
DDTDTELMLSLIRIYASLKTWQRLVFVVSKLQRADTKLLQKHSGEIAEYYYLAAKDYVQE
ESDDEALNYLESALELNPCHMPALNLFTKLAVNMKNSSSALKILKSAFAATPSFELAKMY
ANSAHSSSDVVYGTLANLATPAKYPELFLALATYLNLPDQIVKIKEASLSSYAPV

>gene_17|GeneMark.hmm|354_aa|+|12669|13733 >NVVL01000001.1 Rickettsiales bacterium isolate NORP64 Contig_source1382A_460, whole genome shotgun sequence
MHPSDDRNINIKLDGRQLELLKIFEQIAGEIKPNNFASRFKRLFSAGGHIRGIYLHGSVG
RGKTLLMNMFYDQISLPKEMIHFQKFMQDVHTKMHQLQSASTNRVVQDLAADIASRAQVI
CMDEFEIKDISDAMIIMRLFKYLAKKNVFIFLTTNTLPDNLYKDGLQRESFLPFISMLKR
HFQILHLDTEKDYRYNAVSHFENRVLYPASRETAQEIQGIKEKLSEQAVFSKTDLSVFGR
DISFNQAHQNILFTNFKELFERDLGYVDYVNICENFEIIVLESVRKIEEDEQDIITRFIN
FIDNAYFCRILLFIEIKCKPEEIYPRGRKTAEFERTISRLNEMNTKNYLQREIS

>gene_18|GeneMark.hmm|270_aa|+|13730|14542 >NVVL01000001.1 Rickettsiales bacterium isolate NORP64 Contig_source1382A_460, whole genome shotgun sequence
MNFDKPKFITFEGGEGSGKSTQSKMLHEYLRARHIKSIHTREVGGTPEAEQIRNLLVYSE
LLPVSELMLVMAARYEHINNVIIPALQDGNWVICDRFVDSTACYQSGESGLTIDDIYELH
DQLMKANYKNSAGADGASAHSISAHRTGPYNTSAHGTSPYSTSARVFDTDISNFQNITSS
QNSSPSPENMGIMPDMTFFMDIPPQIGLRRAGARGDVNKFEGKNIDFHMKIYERFKKLAT
EHEDRIISIPCENLSIDGIHKEVLNKIFKV

>gene_19|GeneMark.hmm|675_aa|+|14543|16570 >NVVL01000001.1 Rickettsiales bacterium isolate NORP64 Contig_source1382A_460, whole genome shotgun sequence
VMLYKSINIFLLFASLCITINSSEAKIMKEDQLIPREVFFGNPDRIAARLSNDGQNISFL
APKDGVLNVWVAPGGQISEARVVTDEKKRGIRSYFWAKDNAHIIYAQDKKGDENWRLYSV
NINTLEQKDLTPTDGVRASVLKLSYKFPSQMLIQLNDRVPEYFDIYKVDINTGARELVYE

NTGQYASFTADDDFNIRAGYKMLPSGEGEIYLFENGNPEAPKLFQKIAIEDMLTTSTLHI
SHDGTKLFMINSSGRNTSSLVEVDLATHASKVLHTDSKADVSDYLINTKTKMVQAAATNY
MRKEWTIFEPSIASDLEHLKTIEDADVEITSRTLEDDQWIVVFLKSDGPYKYYSYNRTTK
EASFLFVSNSKQEGQNFAKMHPVKITSRDGLDLISYLTVPRWLDNGSGIPSKPVPLVLNV
HGGPNARDSWGFSPEDQWLANRGYAVLNVNYRGSTGFGKAFINAGDGEWARKMQDDLADA
VNWAVDNNITKRDKVVIMGGSYGGYAALVGMSMTPDMYVAGIDIVGPSNLETLLKSVPPY
WKPQIAHLVKIIGASPETEEGRKFLKERSPLTYAHQIKKPLMIVQGANDPRVKQAESDQI
VAEMKKHAIPAMYLLYPDEGHGLARPENRLSMYAYAEMFLANAAGGRVTPHDSSFPDSSV
EMREGGDINWTREKN

>gene_20|GeneMark.hmm|116_aa|+|16657|17007 >NVVL01000001.1 Rickettsiales bacterium isolate NORP64 Contig_source1382A_460, whole genome shotgun sequence
MSDSYVDYILDLLSPYGNITAKRMFGGHGIYKDSVIIALIINEELYFKADGTNKEQYEKL
GSSPFIYEAKGKPVVISYWQVPLEILEDTSKLPVWMEQSFEISLKILQEKNRKKKL

>gene_21|GeneMark.hmm|65_aa|+|17138|17335 >NVVL01000001.1 Rickettsiales bacterium isolate NORP64 Contig_source1382A_460, whole genome shotgun sequence
MASQTLFIGPRGQITLPKKIRNLFKSDAVVLELVDNEHVMISPVPDVGGAIFNYAKKNRF
NFRRS

>gene_22|GeneMark.hmm|136_aa|+|17386|17796 >NVVL01000001.1 Rickettsiales bacterium isolate NORP64 Contig_source1382A_460, whole genome shotgun sequence
MKKYIADTNFILRYLLADNQESYHQTKIIFEQARIGKCQIEIEQSVFAEVIFVLSSFYEV
PRLEIVKILKSLLSYKGIKVDSDIYSVALDVYLEHNIHIVDSIIAVKTLTSGDELLTFDK
KLQNVILKKLNSENNK

>gene_23|GeneMark.hmm|134_aa|-|17903|18307 >NVVL01000001.1 Rickettsiales bacterium isolate NORP64 Contig_source1382A_460, whole genome shotgun sequence
MPTIDKKKNVILFIDDEKICHTLVELIIPNFTDFKLVGAFDGEGAIELAARYANSLCLVI
SDVILPDMTGYDIFKTLKQNDKFAKLPFIFQTGLGSQEEELKKNINEGVQILYKPYNQTD
LLAAIDKACPKEER

>gene_24|GeneMark.hmm|246_aa|-|18322|19062 >NVVL01000001.1 Rickettsiales bacterium isolate NORP64 Contig_source1382A_460, whole genome shotgun sequence
MSKNSDSSVLEERDSSSNMDEVSFLLKIGHELKTPIHGIGGITKFLNSNWESLDDKTRRE
CIVKISEASDSLNDLLDSLLNRTDGKNTIELSLKKSNLIEVTKLATSECRNFYVQEKDIK
ISFKTSLDEFFITFDQLWYQRLLINLICNAINYSDKGEIVVEVAKRAIDGEDKCIISVKD
EGFGIPESELKSIFEPFNRSSRKTELKKGIGLGLAICREIVDAHKGTIRAVNNEGAGATI
EIILSI

>gene_25|GeneMark.hmm|296_aa|+|19328|20218 >NVVL01000001.1 Rickettsiales bacterium isolate NORP64 Contig_source1382A_460, whole genome shotgun sequence
MNNTRIIIFTFILFIFSFARGDIHQASSVLEINNRLLELLEDNNSKKTLIILPVQNFLLK
RTNPYFNIEDRNLRPLIERVLKKVKLSRETYFEELLLTEYENKPADPFIKDFIHNLQNKK
LPLIAITDSSSGGFNKIPFLEVWISNRLLKEGIDISKSPLGEKQIIFNKNFKQTKGSYPT
FYRGLLSSNMHGADNMYEHILAFMLIEKLKWMPDTIYIIHTEENTIKLLQQQFKHIDPDV
RVIGFHYLPPQNKLMNKALSNISPQKFMQFWEGVITKLNNIKLKHSAKGAKDPYEQ

>gene_26|GeneMark.hmm|283_aa|+|20208|21059 >NVVL01000001.1 Rickettsiales bacterium isolate NORP64 Contig_source1382A_460, whole genome shotgun sequence
MNNKFTNNTLILILFWVTSLCSANIANAQLIKTYSVNSSSMAQIETWADKDTIIFITLDN
TLTIPNSLMFSYNSEANRTFIQNLASLGKQMPAYNKIIATWYQQRKIKLVEPQWAGLITR
LKAKGATVYALCSMPIELVNIEEKRYKELKDLGIILTSTVNQKEKLQIGKNSSWPARFYK
GIIFTAEGGPSIALMEFLKITNISVKKMAIIGNIEYKLKELEQILRTFDMEYYNILYLGA
RELATNPDPELIKFQQQELVNNGVWMEDAAALRARAARLNPAQ

>gene_27|GeneMark.hmm|336_aa|+|21099|22109 >NVVL01000001.1 Rickettsiales bacterium isolate NORP64 Contig_source1382A_460, whole genome shotgun sequence
MLQNYLQKYFTKAFYPTPHRNWYKEKGYVGADSSLQFADFLTTQKKNKTSKFHEEFKSLD
ITRQYVNSGEHKLDSVILTPYKSISRNKPGEGLYFVMFQGRGEYYESRFRDMALQAKETG
ASVVGFNPKGFHASSGKTQKLYDIVEDGIAIIGFLLEKGIPSNRIIMQGNSLGAGVQEMV
SEHYAHVKNIRFRQINSNSFKNLASVLAYHYKAPILERVFSVILKYAGWEISVGAEFYKT
GPYRCLLRRKGDRTIGGFAEYNAMVNHQEDYENCSKDYRETNKWLNEHNQLIYTGRSKKD
PHALSLHHFAIKGEGKRSVYYLINRYLAASNTIVVI

>gene_28|GeneMark.hmm|161_aa|-|22256|22741 >NVVL01000001.1 Rickettsiales bacterium isolate NORP64 Contig_source1382A_460, whole genome shotgun sequence
MAKESEITNESNIVNIAGKSNLAGRNAAIMFHKPYMETAFYEKQHLETECAQLGVTIINA
IYNEDKDAFVAYKKLLPYIQQENDNSKPLLLFANAKNLVLPENRMSKIILSVLIDLKEIE
LYLYNQGFNMKNGKSELALEPCTAKGILHIKQPDPSLGVFI

>gene_29|GeneMark.hmm|84_aa|-|22959|23213 >NVVL01000001.1 Rickettsiales bacterium isolate NORP64 Contig_source1382A_460, whole genome shotgun sequence
METEEAPQKTRLKNSPHRVRYPNGIARGGALLLYNNHRINLYSTILFKLLNTLAILVDSA
TGMTKSVILEFESFFASGYLSAIK

>gene_30|GeneMark.hmm|851_aa|-|23312|25867 >NVVL01000001.1 Rickettsiales bacterium isolate NORP64 Contig_source1382A_460, whole genome shotgun sequence
MVGKMLNNKSMRIMLLTTLFFNTHAAIAAPSDPEGTSTSEASDSEGSASSLLNSSDNDSD
DTASVHSEASDLGANYTLIDKKLTFTTDRFGFQEENFADDYDPIQEISVGEGQSLSIEGN
IGDNYGDDYEAQKEHYTQILEENEDFKEAIELDEVKLTGIEEITGTGNVVFTGNNVVNCW
LGNEDEYLESIETQGDTIFIKAIYAKHLTFQYETMMFSRHINTEHITLNQAELTFKQGAD
RAIKITGVGTVIYHKGSDLGAITLTVDEDGYSGELVLMATEDNTEIYEMTANITARLEQD
ENATLNYNGFELNNLDAENKSTASPAVSEHDNDSTDVDDEHLSSSSSESDSDDDDVAEVP
IAIPAAPVQEAKIDNAPAPARHSSPSSEVSDNRPPSSEVSDNRSPSTSNEESLPQIIKNC
LNYSNKDDPAQNKTITTETPEEQQTKAANEAIYQVAGREISNIDKNIKTKEDIVQFVAKV
TTQQARDLSPNTQKVIYTIAEDDKTAEVRKKEHTATAHFTKAQKEASQLVVQSVISETTA
RIANISLNSQPNFISNLASNITGTAAGDSHKIEKGVWISGMLAKNTQKENKNHSAYNAKI
YGGTIGADAQVTEHTLIGAFYSNITSAFTFKGAAKGSKLDAKSHVFGIYGDTAFTDNLSA
QMILAYGQTTIDNRRKDGTNTAISKFKNAVYSGDLNLTYNKYLANNVSIIPTMGIRYSYF
ADEAYKESKAGLNNLSYGKKSSSNLEAMFGAKLIWQKQVLSNLILVPSIKASVHQVLSEQ
SSKVKTTLAWGEEYFSSDIVKKDKKADKTRFNIGAGLLAKIDNVDLSMDYSLSIRKQYTG
HQGSVKIRINF

>gene_31|GeneMark.hmm|66_aa|-|26034|26234 >NVVL01000001.1 Rickettsiales bacterium isolate NORP64 Contig_source1382A_460, whole genome shotgun sequence
MLNLLFNRRIEALMIFIFCRNNELNRYISKAKKHEGSSLNELFIYKLANFYYRHANNYYT
LIVVLN

>gene_32|GeneMark.hmm|884_aa|+|26442|29096 >NVVL01000001.1 Rickettsiales bacterium isolate NORP64 Contig_source1382A_460, whole genome shotgun sequence
LKEVFMETTTNLEDFLSAPETYNVLDLSNQNITPAEFIRLFTALKDTNNSTLTSLNCSGN
KLSSAAIMILADWLGGQNTALNSLNMSRCYLGSKAITTVVNALEGGGSVKELILCTNAIG
DETASLITETLTRCESLTVLDLEGNQISEQGSAALCGKLAKLGDNLKILNQLGEQGVIDI
FPVQVEAAEIKGAEVDVLAKMAEVYEGVQGNSELCLVVIGNTGAGKSTFVNYLAGNRLQV
TQDPESKKLLVDRHPEDTPLAGITINHTSASGTKIPYQCSVNIDEGEAEPINITIWDCPG
NNPSSDANIEGTISGFTFDRLFEATKQVKLLLVVPEFDITHGRGNGFVNAIARLTEEFSP
RVLDAVSLVVTRVTEQRNEANIKYLVREVFNENDTLRGFADDHPIKGVIKSLLQDSSTIH
IFPKFAEPAEEIQPDMELIASIHASGDYLHEPNAQDVRVSEGVKGYSENILRELVYNNLN
MMLHVISQALVNPTDCRSSDPEKNAFLRNYESIKPLVPESYKHFAPMLPLEENQFLTPNP
DHPGAAEERHFTDLDQLSELKDILWQFKNGDFLSLREGMNAIASVLSSFEKSIDVANRDT
KDVLLQYAYALHQQLNCLEFISGMGAEPFLAAEPLALQQYMTQCYNGICRNLEAGVRALE
IEPDGGSQAYYDQAISYLTKYSDCEPVIAQAYMFKAQLEESENANVAVGYYAQAIDIDAA
PADACEKLGDLLFKSGDLPNAVKAYSALHYVSKIDLCFATLQAQPTSTENAALMLSIANY
RSQWKEGEIVYLNTLNANHSSEFFAQVKGAVTLHMRDSHLCEVLNSGKFYNFAEVNEEFR
ASLLLAYPDEVAPEPGVEAFLVPVELEDVPGIIGAPAAGEEEVA

>gene_33|GeneMark.hmm|41_aa|+|29303|29428 >NVVL01000001.1 Rickettsiales bacterium isolate NORP64 Contig_source1382A_460, whole genome shotgun sequence
MSREGVPYIVLPISAGQVFSILVKIDDAKLLRVNLSGVRSV

>gene_34|GeneMark.hmm|207_aa|+|29577|30200 >NVVL01000001.1 Rickettsiales bacterium isolate NORP64 Contig_source1382A_460, whole genome shotgun sequence
MSRGIFATTKGSVSADQVRATIEKFSQGKEHYIGGFHEALRSCFDSKNIDIITSAFRNEK
TVSVQCGSEVWLLEMAPPPPLYPPGSENNPIASHVTMPALESLIANFARNSTLQALGFEK
AMEDYFNVSDLQNIISILLNGNDVYIICARGRILQLTEENLSPPRHLLEEPVAVEELTDS
PDSPGSPDFSDFSDSAKYLGGESDFVF

>gene_35|GeneMark.hmm|100_aa|+|30254|30556 >NVVL01000001.1 Rickettsiales bacterium isolate NORP64 Contig_source1382A_460, whole genome shotgun sequence
LFEGFTSRGNYYGSEFIENMQSYFEKEDLEKLASILWDLKEKAYVMCSCGEVLCLGADIV
SLPALSPLLAGDAEEMEEHLNTDGKPDSLVAGDGVAAFVI

>gene_36|GeneMark.hmm|67_aa|-|30734|30937 >NVVL01000001.1 Rickettsiales bacterium isolate NORP64 Contig_source1382A_460, whole genome shotgun sequence
MAVQISVHGGNGEKAIRDLKRKMQRELVFRRMKMSRFYEPPSAKRVREKQEAERRIRKNN
RRRRLES

>gene_37|GeneMark.hmm|236_aa|+|30967|31677 >NVVL01000001.1 Rickettsiales bacterium isolate NORP64 Contig_source1382A_460, whole genome shotgun sequence
MLKYPLIIYIVSLHVKKKGRVGAKMTDISRKQAERRLKFFAALAKLLATNMWSEALLGKV

EESCAFDKGYNYALFPEGVSQIAVSFESWQDEQMLSALSKTEKPAKIRECIARALEIRIM
DVVHKNVAINNSGFFMMPQNILEGNKAAFRTCDLIWKYAGDQSADFNYYTKRGLLLSVYK
SASAFYFADNSKDHEKTKIFIKNALDNIVNIASLKSRIKLPKIPKMEDLPILRLFS

>gene_38|GeneMark.hmm|1056_aa|+|31739|34909 >NVVL01000001.1 Rickettsiales bacterium isolate NORP64 Contig_source1382A_460, whole genome shotgun sequence
MTEQKYYPEAKSTVSFPEIENEILKYWKENKIFQKSIDNRESVIDGKKNEFVFYDGPPFA
NGLPHYGHLLTGFVKDAYARYQTTKGKKVERRFGWDCHGLPAEMGAEKELGFSGRIAIAE
YGVDKFNDHCRSSVMKYSKTWEQYVDRQARWVDFEKSYKTMDKNFMESVLWAFKELYKKG
LVYESMRVMPYSWACETPLSNFETRLDNSYRERADKAVTVSFELAQKPAGAPDGCSEYRI
LAWTTTPWTLPSNLALAVGGEMEYACVVKDDVCYIIASFAVNSYAKELGVSEGDEFEVLK
GSDLAGLSYKPLFDYFKDNAKSFEIFTAEFVVEGDGTGIVHMAPGFGEDDQVVCEEKGIK
LVCPVDNAGKFTSEVTDFAGMQVFDANDKIIIHLKEQGNWLKTEQYIHNYPHCWRTDTPL
IYKAVPSWYIKVSQFKDRMVELNQEINWIPGNIKDGMFGKWLENARDWSISRNRFWGTPI
PIWRSDDSAYPRIDVYGSIEELEKDFGVKVEDLHKPFIDDLTRPNPDDPTGKSTMRRVGD
VFDCWFESGSMPYGQAHYPFENKEWFEDHFPADFIVEYSAQTRGWFYTLMVLSTALFDRP
PFLNCICHGVILDSTGQKLSKRLNNYADPLELFEKYGADATRATMLSRGVVKGQELMIDK
EGKMVFDTLRLFIKPIWNSFHFFCLYANSDGIKAEQSFESDNVLDRYILSKLKLATSAIE
NNMDAFDSQGAYGAISDFFEVLNNWYIRRSRSRFWKAEKDQDKISAYNTLYTCLETMSRA
MSSLVPLISEHIYLAITEKSESNMSVHLTDFPVLDEIQIDEGLVETMDRVLDICSTALFI
RNSENIRVRQPLASLKIVVDNSSVFKDFEDLIKDEINVKSIEYNSDLANYADKKLSIKFP
VLGARLPAKMKEIIVASKQGKWEYDGTSLTVAGEELLSGEYSLVLQPKKECGGKSLSDNQ
GMIVLDLEITKELADEGVARDIIRLVQQARKDAEFDVSDRIKLELKSDFDLSGILAVHAG
FISEQTLSELAYGISEDYKVETSLQDKNLEIRLQRL

>gene_39|GeneMark.hmm|448_aa|+|35179|36525 >NVVL01000001.1 Rickettsiales bacterium isolate NORP64 Contig_source1382A_460, whole genome shotgun sequence
MKKIFLLPLFLICVPTMSSAITLEQALVSGYNHDEVLKINKTDFLQEIEEFPRALAEFMP
RITAGIDFSHTRRKSKNSSLEHKGSHTTQSVTLNQPIFNGGASLAKLKAARFAFRASMGK
YYAAEQDQILKEIDAYLSCAATREKYAISKMSVKSNKTQLEAMKEKFKLGESTETEVAAS
EEGLATAESNQSIAYANYEASRAEFFRIFGVEAAGIKMPALSEGLPKSLEEVLKEALAKN
YSIDQARYSSQASKSSEYASKGVLLPSVSLSVRGERIKFKKQDPARNRSNNNSVTSTISV
KVPILEKGGFEYSEVRRAKHQTRKTIIALNNTIKTIKSQGRASWSFLSANKLRIKSAAKA
VKAAEIAYDGVQQEEMLGSKTIIDVLRTEGRLNDARSNLVEAKKALVLAGYRIQVLMGKL
TAKSMNLKVEHFDPEAEFKRIKFRIIGF

>gene_40|GeneMark.hmm|129_aa|+|36528|36917 >NVVL01000001.1 Rickettsiales bacterium isolate NORP64 Contig_source1382A_460, whole genome shotgun sequence
VLQEIAKEGITKEGTASGEGEASLGEVLKSIRGAIDNPSPNLSFESDDDFYGDDEGYGVL
ELTNLVSDADLARTPHKNTGNAQHIGQELLEQTLEKVMRPLVKEWLQKHLPRVVEKVVTQ
ETRRVMPKS

>gene_41|GeneMark.hmm|851_aa|+|36955|39510 >NVVL01000001.1 Rickettsiales bacterium isolate NORP64 Contig_source1382A_460, whole genome shotgun sequence
MARNLLLFLSILLLSWSYSLAGNKPNPLMGTPFMGTPLTEGTLEGVPLAEGALEGRALVA
SLLTTPPQVADPIPMTHPVAPAPFAGTLLTGILLADASPNGVIFDGVMVEKDAIKEDNLK
EANLEKGSVKKASPLQDPGASGELAVASIEGAGGEAALPSMPESSIAEKSESFLAKGEEK

SFFAKIKSFFGFDDEKTQQPESKSAQGAKAQLARAGSAGGRGAESLFAPLEPGMDDGDAG
LVTSQPVGANDEFISGELPQIPSGFSQMPADSGQIDSGDELRLPDGFNEIDEDFSTVEAS
SSSSMPGSGEPDFEALNSAGSGLAGSSLAGAGSTDPASAATSFAGSNNRDAGNISLSKED
SKPEMSLAIPTEFEEYATGATGGALGDAAKNELDTQKETLGQKQNQTQESLQRQSSKLFQ
PSEQAVMPANPGASSAEQMGKLTTKSAINSAAEQSAQVVTPANSGTSSVEQMGKLTTKSA
INSAAEQSEQALTPANSGASSIEQMGKLTTKSAINSAAEQSEQAVTPANSGVSSAEQMGK
LTTKSAVNSAAEQSSQVVTPANPGASSAEQVGKLTTKPAGKQVAKSDDKLEAKQPTTPVT
PVKPAGKLPNASTGKEPLPTPSYAKELTLPPREETATSKYEAELELRQSAKSELPHISPE
ELSLAQDAQEAKPDPVQLKFIANEAQLLVLPNDDIVLGELTEDAKLDLIDLRLYVDLFWE
EYRKSKNEEKRAEIDNFVDNYDANFNKTASLHGENHAYEGLTEAFKAIRNDNILSLISIL
NSYPLLQLSDGNGNTLLHAAARVGNYPAAKLLVLKGVDIFAKNRLHQTALSIARELSHDE
IVTLLKSAGLK

>gene_42|GeneMark.hmm|1154_aa|+|39654|43118 >NVVL01000001.1 Rickettsiales bacterium isolate NORP64 Contig_source1382A_460, whole genome shotgun sequence
MESDQYYLFKDRRFLPIFIVQFCGALNDNILKNALIILITFKLANELTLSSYTLIMLAHM
LFTLPFVLFASISGQLADKYERSTIVKIIKFVEIGIVIASSYGFYTTNLPILFGCIAMMG
THSTFFGPIKYSVLPDHLRKEELLGANGFIEAGTFLSILLGTMMGGFYNFSNILVISLAL
IVSVVGFVASLFMPKSNNFNPDVKINLNILQETVNMVKYAATKNQVYMAILGISWFWFIA
ATILAQIPLLTKDILGADESVASLFLVTFSVGVGVGSFLCNKIFNNTITTKYIFLATMGI
SVCIIDLFFASSIAAVSDEPEQLKTISEFLTRGHRWRIVIDLFLLSAISGLYVVPLYTVM
QYFSARSHRSRVISANNLINSFFMMGSTITLSVLRYFDFSIPSVILFVAICNTMVAIYIY
RLVPDSQIIPRKVWTFISKIIFGIMYKIEIKGLENYKKAGKKTIIIANHLSFVEPALIAV
YVEEDLQFAINTAIAKEWWVKPFLKIVKTYPVEPNNPMAIKRLIEEVKKDKKIAIFPEGK
ITTTGSLMKVYEGCGMVADKADAAILPIRVDGTQFTSFSRVSGFLGGKFSLRRKVTITIL
PPVKITPPEGAVSRERRKYIRQGLYDIMSDMTFESSEYNITLFQAMINASKTYSSNLKVM
HDVEHNKANYRSILIKSFILSELLCVDTKPGEKLGLMLPNMVGTAITFWGMQAAGRVPAM
INFTSGSANVISSCNTAQVKILYTSKKFIEKAELESLVKAVSEAGIKVVYMENLRKRVSI
GLKLKALVASMFPQRYYDSLNQDTSASSTAVILFTAGTEGRPKAVALSHKNLLANTAQAL
ARIDFNPRDIAFNTLPMFHSFGLMASLVMSLNGIKTFFYPSPLHYRIIPEMIYDVNATIL
FATDTFLAGYGAHAHPYDFHTIRYVIAGAEALKERTRKLWSDKFGIRIFEGYGVTEAAPI
ISANTPMHNKPGTVGRLMPKIEYFLQKVDGIKEGGKLCVKGANIMQGYISPDNPGVIVPP
VVEKLGEGWYDTGDIVSVDEERYITILGRQKRFAKIAGEMISLAGVESLLIKLDPEAEHA
AVSIADEKKGEQIILFSTGKDITRERILQTCKQNQYSELYLPRVIIPIKEIPMMVTGKRN
YRKMTEMAKKKVGE

>gene_43|GeneMark.hmm|77_aa|+|43108|43341 >NVVL01000001.1 Rickettsiales bacterium isolate NORP64 Contig_source1382A_460, whole genome shotgun sequence
LESRDIQVSVESSIGAIISPLISALFASNPNNDYFFLTKGEIVAIGLLCFVRIIDDFDFS
NFSKISKQWDLNLLIAI

>gene_44|GeneMark.hmm|92_aa|-|43460|43738 >NVVL01000001.1 Rickettsiales bacterium isolate NORP64 Contig_source1382A_460, whole genome shotgun sequence
LDNFSNSAMLSQEGFVVESLHETIVNDARKESSLTTKLLIILSIGLLFWVKIIQQLSISR
DRMIGENNKSPNSYQGNRIKILRELLPMIYLI

>gene_45|GeneMark.hmm|320_aa|+|43791|44753 >NVVL01000001.1 Rickettsiales bacterium isolate NORP64 Contig_source1382A_460, whole genome shotgun sequence
VGVTQDLTKAFEWYRKAADQGHAAAQNNLGAKYLNGEGVTQDFSKAVNWFRRSADQGHGA
AQYNLGVLYANGDGVAQDLTKAFEWYRKAADQGDADAQYSLGFQYDKGEGIIQDLTKAFE
WYRKAADQGNAFAQNNLGAKYLNGEGVAQDLTKAFEWYRKAADQGCTLAQHNLGLQYANG
EGVAQDLTKAVNWFSKAADQGHGAAQYNLGVLYANGDGVAQDLTKAFEWYRKAADQGYAD
AQHILGVLYVNGEGVAQDFTKAAKWFRKATDQGHADAQHILGILYTEGKGVTQDLTKAFE
WFTKAADQGHAAAQDNLKLK

>gene_46|GeneMark.hmm|451_aa|-|44841|46196 >NVVL01000001.1 Rickettsiales bacterium isolate NORP64 Contig_source1382A_460, whole genome shotgun sequence
MTATILVVDDNEQNIKLLEAKLVSEYYTVLTAENARGAIEILKKNKVDIILLDVMMPEMD
GFTACREIKSNKDTAHIPIVIVTALHGVDDRVKGLEAGADELLTKPIDDVALFARVKSLA
RMKAAMDELTLRNSAITELGGKTIKLKTSFANNKILLLDDDLIQAKNIKSKLTSLTQQIQ
IISSAADIAGLGSFVPDLVIISCQLSDQDPLRISAMLRAKPSFKNIVLMLLSEEENISIV
IKGMEIGINDYFVYPVTKEELQARIRTQLRRKQYQDDLRLELEENIDLSTKDGLTGVFNR
RYFDIHIKHLVQLAQNSRHDICMMMFDLDDFKKVNDTYGHQAGDAILREFSKSLKSSFRA
TDLVARYGGEEFIVLLSDVGIKKATQIAEKTRAAIAKIDFTIPGETKTLKQFTSIGVAKF
NPNDSIESFIRRVDKALYNAKESGKNCVIAG

>gene_47|GeneMark.hmm|124_aa|-|46199|46573 >NVVL01000001.1 Rickettsiales bacterium isolate NORP64 Contig_source1382A_460, whole genome shotgun sequence
MPKVFAIEDNELNLKLFRDLLKIQKCDIILSDDGVGVLETAIEEQPDLILMDIQLGDING
MDLIRDLKMHEKTKHIPIIAITALAMKNDELQILEAGCAMYLAKPVAINDFFKATSKFLN
LVDE

>gene_48|GeneMark.hmm|159_aa|-|46697|47176 >NVVL01000001.1 Rickettsiales bacterium isolate NORP64 Contig_source1382A_460, whole genome shotgun sequence
MIQISCYLTELEALPKVFCGLAEKCYYSDMRTLVLTENDSFSTSLDRVLWTYSKKHFIPH
AMSGDPQPEDQPILITSAFEHSNNAEIVIFVNATESMILEAAAAGSAVRLQNMKKILFLF
DDAQKMQSSEINNILKKSSLGEFELKSFAKTSQGTWKTI

>gene_49|GeneMark.hmm|328_aa|-|47298|48284 >NVVL01000001.1 Rickettsiales bacterium isolate NORP64 Contig_source1382A_460, whole genome shotgun sequence
MIRSMAGSYNMRLIYNFFFIIIFLPAMSFAATAAGAGDGTIAEAGAKILEAGAATIRTNV
KILVTDATTLGTDTKVIEADAIIVESASGTNARGIAATDKVSAADAEVTATAKVSAAAAS
EPMAGAQQGGRINSDKQIVKARHIEECIGKTDGATEQIARFSVKAAVVDVESILEHSFAI
QHIKKSITTINNKIQEELSVKDIELKHIESDLIKQRGTLDRKKFELKVAEFNKKVSMVQR
EKQKKKSALERAHAEAIALVQKNMNLVISELSKKHGFNMVFPSMQLLFVSHDLNITLEVI
TKLNKRLKKVDIKYELYRDNKSPEDTDS

>gene_50|GeneMark.hmm|762_aa|-|48309|50597 >NVVL01000001.1 Rickettsiales bacterium isolate NORP64 Contig_source1382A_460, whole genome shotgun sequence
VKNIFNIALFLMAIILSSSAMAAKINKINVRGNKRIEQSTIVDYLNVRVGDHYSEAKKNE
MIKNLYATSLFETINIAFHKGALKVSVQETPFISRVVFAGNYKVRTKILLNEIYTAPGES
LRKAKIRTDVEKIKEIYKRSGRFSVHVSSKIEPLENNRVKVIFEIDEGPKTGIKNIYFAG
NSHYKDSELKSIVLTKESRWFRFLETNDTYDPDRIEFDKQLLKRFYNSVGFADFRVISVT

SDLLPTKEGFALTYSIEEGEKYKFGKITINNKLDTIDDSEVLKFVDAKKEGETFNLSAMQ
RMAEKVSKYLGGKGYPQVEVHPEVTPNRASGTVDVVIVVDQASKIFINKINIEGNLKTED
SVIRRQLQIAEGDVFNRSKLERGQRNIRNLDYFGKFNLGMSQTKKQDRYDINIDVEEKST
ASFGFDLGYNTTGGMFGKISFLERNLIGSGRQFNAGVQIGKKSTHYYTGLTDPSFLGKDL
SLGGTLFKSDDGTGSGFENGKQNYSSSTIGTRINLGYNITDDLFHGIEYGIKKSELKAPG
KTASIFIKEQMGKFVTSEISHSLTYDKTDSRILPKNGYLLTAEQAFAGIGGDTNYLKHEL
SGKAFKSFVDNKYTLKFSAAGGHIKGLKGKKVRIPDRFTVGDYNLRGFASGGIGPRDVAT
KESINGQKYYTLSTELSFPLGLPEEFNMTGSIFIDAGALWDADSTAATSKGLYNTKTLRS
SYGFGVLWITRIAPIRLDWGFPIKKEKFDETQVFHIRFSTHL

>gene_51|GeneMark.hmm|100_aa|-|50850|51152 >NVVL01000001.1 Rickettsiales bacterium isolate NORP64 Contig_source1382A_460, whole genome shotgun sequence
MTKYYDPITPGEYLEHEFLEPLGLTQSQISRDIDVPISRINGIVKGSRAITADTAIRLGI
YFKTSAQMWLNLQAQYDLKIAQRKNLASIQNRVQVFKAVA

>gene_52|GeneMark.hmm|92_aa|-|51164|51442 >NVVL01000001.1 Rickettsiales bacterium isolate NORP64 Contig_source1382A_460, whole genome shotgun sequence
MIKSFKCKHTKNLFEDIRGKKFSSIAKFAKMRLTSLSIACSLNDLKSPPSNRLEALKGDR
KNQYSIRINKQYRLCFSWQRGDAYDVEVVDYH

>gene_53|GeneMark.hmm|290_aa|+|51904|52776 >NVVL01000001.1 Rickettsiales bacterium isolate NORP64 Contig_source1382A_460, whole genome shotgun sequence
MKKLSFQQIILELQRYWQDYGCAILQPYDVEMGAGTFHPATVLRALDKKHWNVAFVQPSR
RPTDSRYAQHPNRMQHYYQFQVILKPSPDNIQELYLKSLEHLGINTKKHDVRFVEDDWES
PTLGAAGLGWEVWCNGMEVSQFTYMQQIGGIECRPVAGELTYGLERLALYIQGVDAVRDL
DWNGQTGDRALTYGEVDFHAEKQFSKFNLELADTEILFRQFADAEKQCKALIKEDLPIPA
YDYCIKASHLFNLLCARKAVSVTERAAYIARVRNLAKLSCQKWVEMEGEL

>gene_54|GeneMark.hmm|677_aa|+|52780|54813 >NVVL01000001.1 Rickettsiales bacterium isolate NORP64 Contig_source1382A_460, whole genome shotgun sequence
MSELLLELFSEEIPAMMQKKASSGYKEIFSKYFAANEIAFDEIDVYAGPRRITIHIQGVA
DILPACEKELKGPKISAPQQAIEGFCRSNNITPDELSVQEIKGQQFYIYKEKTPERCSRD
ILLSTLSTPISEYVWPKSMYWGEYKIKWVRPLRNILCVFDGEVIPFEYGHLVANNKCYGH
RFMSPKECVVTNFAEYKKALEENFVILSSDERREYISQGTKKIASKLGLVVKEDAALLEE
VSGLVEHAEVMVGKIDEKFLSVPSEILVDSMKIHQKYFSLFDKDGKFAPYFLFVSNIRSE
DPDIVVSGNEKVLSARLADALYFYNQDLKITLEERALRLEKVIFHAKLGSLKDKTLRLAE
IVRFVSPKSKEAAAAALLCKSDIVSEVVDEFPSLQGIMGYYYAKAESKSEELALAIRDHY
KPQGAADQCPSGEGAILALADKIDTLCGLMLAGEKPSGSKDPFALRRQAVGIVRIILENK
LSVDLAELVDFVIAKYQDSLLDIKPDSALNAKKQVLLFLEERVRHFFKGEYNHELIASLL
DFELEPDLLALQMKLSALKSFVTSGAGDDLLVAYKRANNIIGGAKLTGQVDASAFVLEDE
RALFACINDYSDKITLALGDSNYSESLKLLANLRKPIADFFDNVMVKDKDPLIANNRLLL
LDQTKKIFNKLAKFEKF

>gene_55|GeneMark.hmm|334_aa|+|54813|55817 >NVVL01000001.1 Rickettsiales bacterium isolate NORP64 Contig_source1382A_460, whole genome shotgun sequence
MSQIEKAAKIIRAGGLVAFPTETVYGLGADAGSQDACLGIFKAKGRPSHNPLIVHAASPQ
QASSFAVFGADAQKLMKIWPAPLTLVLPIREGAKLAPCVTAGLETVAVRVPSDARARSLI

EASGCPIAAPSANKSGRLSSTTYEHVQKNFGDSVFIVQDDGVKSCGLESTIIDLSTDTPT
ILRYGFITPELLSEALGKEVAIASKLSKIKAPGMLLKHYAPKTKIRLEAAALNPGEVGLD
FGNSCLGSGSLRNEGFRNGSMSSEGGSSDSVSSAGSLNLSPSGDLSEAASNLFDYLHRLD
EFALAHSIKTIAIAHIPNESVGLAINDRLSRAGE

>gene_56|GeneMark.hmm|176_aa|+|56102|56632 >NVVL01000001.1 Rickettsiales bacterium isolate NORP64 Contig_source1382A_460, whole genome shotgun sequence
MNKIYGVVFTILTGMIVCNIFFHVVLGAHFTGYYDLVGFMAATGVLISFVLAETEFSHIK
VDLIKYRDTKALKLLRTSLIFIVYTCFACAQYMAIYDSFFRSINNVMIEVTTYALYFISS
ICFTLLSVVSGYRVVKLIISKDGKIEEGRLEKIAEGRMSEVVESKEKEQQKREFQK

>gene_57|GeneMark.hmm|434_aa|+|56751|58055 >NVVL01000001.1 Rickettsiales bacterium isolate NORP64 Contig_source1382A_460, whole genome shotgun sequence
MLSGFWLFCALIIFLLLRFPVFIALILSGSLGYIWEVGLNPWWHWASFSPTNLVSQYHYA
AIPLFLLMGQIASAGNITKDLFVFTDNIARKTRGGVAILTILSCGVFGSLCGSSLATAAT
MTPIAYKEMKSRNYSKELILGTLAAGGTLGVLVPPSIVLVIYSIIAEESIALLSIAAIVP
AILAFLGYVCAIKYFIYKYPNEVPELLPKRQKTEYGLKAVFSLCSILLLFFVMLAGLYKG
IFMQAESAAIGVAGVFAIVAASGNLSKLNWPKIFTESAKTTASIMAIFIGAEIFSVALTA
FDFPKELAEYVMTYREYPLGIITIMIAALLVLGCFMDGIAIMLLIVPLFLPIVKELDLWI
APENIGIWFGIIILMVVEIGLITPPFGLNLFVIKSIEPELEQASNFKSVKYFVAAELVRM
SLLVLIPGIMWCVA

>gene_58|GeneMark.hmm|156_aa|-|58142|58612 >NVVL01000001.1 Rickettsiales bacterium isolate NORP64 Contig_source1382A_460, whole genome shotgun sequence
MKILGIDPALTALGWGVILSESPKIIYVDSGLIKTKPATPLHFRLANIADSIEEAIDLHQ
PDVIAMEETFVNKNAVSSLKLGYVRGALMALIGKKNIPFYEYKPNNIKKTITGVGHADKE
QVKHMVQMIISGTNPKISLDESDALAIAYTCLANQR

>gene_59|GeneMark.hmm|1023_aa|-|58612|61683 >NVVL01000001.1 Rickettsiales bacterium isolate NORP64 Contig_source1382A_460, whole genome shotgun sequence
MNLSVLCIKRPVFTIVLSLVIMSLGAVFFNKLQVRGTPNISPAIITVNSYYDGADALYME
KQITAPIEKALKTLKNLKSMSSSSSVSQSNIMLEFELDTDIEVALNDVRSMMPPANYFPD
DMGAPSISKMDSDSWPTLWISVTSSMHDDLELTRIVNNQIKAPLEKLSTVGKSRIYGGSY
YSMHIEPINTKMFQHKLTPIDLEAAIRSQNKDYPAGSIKTDSRSFSLKLAGSLNKPEEFE
DIIVKKYPDGTIIKLKDVAHVRLKPYESDMSMRYNGNQSLAIGLIKQSTANIIELSESVI
AELPKIVKTLPPSVKIEIAFDGAVSVKASIRAVYFTIFEAVLLVGFVIYLFLGSLRITMI
PLVTIPISLIGTFSAMYMLGFTINTFTLLAMILAVGLVVDDAIVMLENIFRHSHELGKPP
MVAAIDASKEISFAVVAMTMTLAAVFLPIGLIDGFLGKLFVEFAWTLAFCVLFSGFVALT
LTPMMTSRMIKQETSPKLKFLQSFDSYLLRVQDGYIGGLIYAMANKKKFYAVCAGSIVAL
IASFVYIDKTFVPKEDQGYLIVSFKGVEGASTQESLKTIIEAEKTFSKYEDIQGFLTIAG
DGSGDQGFAFVPLNDWSTRSNSQDELADKLNKDFAKIPGMSIFAISPQPFGGGGSNSPVN
FILQTSLEYSDLDQVAQKFVDQMKENKMFENISKDFKASTPTLDIVVNREKAYRYGVDID
TIGKTVQYLIAGKNVGDFRMGNDIYNVVLRYNKKDRNNPSDLNKIYVKTKDNELLPLETV
ASLIETITVKTYKHYNNSKAIRITSDMSKDTSLGDAAKIIEEMAENIIDPNTTQLKFSGQ
IQQMNESGGDTLITFLFALLFIYLILSAQFESFSDSLLILLAVPFSMTGGVLTLLIFGDS
INMYSNIGLITLIGLVTKNSIMLVEFTNQLRETGKNIHDAVIEAAKLRLRPILMTSIATI
CGAIPLAFASGAGAASRSSIGLVVVGGMLVGTLFTIFVIPLLYETFKKEKDLREERSLGE

GNS

>gene_60|GeneMark.hmm|376_aa|-|61680|62810 >NVVL01000001.1 Rickettsiales bacterium isolate NORP64 Contig_source1382A_460, whole genome shotgun sequence
LERLLVVQNIFRVFLLPFLILAQALMLASCSKEIEDSPPVLVKPAKIATHDFHEKFIAIG
QFKYESSKTYYAKIAGTVDSIPTHQGERILKGDILITIDGEIAKTLKNQADASFISAQSS
YERELSLYKKKIIKTEALDRSKVALETAKANRASAMKKYDNMVIKAPFDGYVGVVRARVG
DYLKIGDYLFSFIENGDKTVFIELPEILHGQIDKTTEVFVTDTKGTKSSGRIVAVSDYLS
NRGTITTKLLFPLSAAIVHGSYVETEIVFNKHSAMGVPEKAVLKNSTGNFIYKITSDNVI
KQVYVGVGSRTDGIIEISSEEITEGDLIVIEGLTKVSDKSKIELIKEDSGTVSSKTTDSK
AVDSKAVDSKAMGSSQ

>gene_61|GeneMark.hmm|117_aa|-|62960|63313 >NVVL01000001.1 Rickettsiales bacterium isolate NORP64 Contig_source1382A_460, whole genome shotgun sequence
MSFSKFHIELDNDLAWKLVLLFSAMLLILGTAESAFATENKDVIGETLCRLADTLRGSVA
KAVATMAIFSVGVGLFLGKLNWGVAAATAVGIAIMFSAPRLISFISGVDYGTKCLVD

>gene_62|GeneMark.hmm|385_aa|-|63564|64721 >NVVL01000001.1 Rickettsiales bacterium isolate NORP64 Contig_source1382A_460, whole genome shotgun sequence
MSGSENNNLEIVIQTKDLARALGFASSVVEKRNVMMELGNIKLVAKGGTLEIGATDMDLY
LNQNIGAEIIREGGTTVSTQTLSEVIRKIPDDEITIKENVLANKLEIIGKNCHFELLTLP
TEKFPEMEDIDTESSLSISCADLARIIEYTNFAMSSEETRYNLNGIYLHVKGAEFCSAAT
DGHRLAVASVPLSNKSEEFGVIVPSKTVTELLKITKDGQNIHSEMRIFLSSNKVKFECNN
LVLISKLIDGVFPDYSTFIPADNESKLTVSKKLIASAIDRVATITVDKFRAVKLAIDNDS
IKITASGEAKGAANEMVAFSDEKKDYCNFSGESLSVGFNPRYISDVFSSLTEEQVELYFK
DEFSPVLIKTAQNPKDSFVIMPVKI

>gene_63|GeneMark.hmm|474_aa|-|64767|66191 >NVVL01000001.1 Rickettsiales bacterium isolate NORP64 Contig_source1382A_460, whole genome shotgun sequence
MQQSNHVNLSQKDDSYYAHLWRDVSKDLTSHYGKDLYRNWFSKINFLESQGGKNILLSAP
SNFIRDWIKSNYFNTLTEIWKRHDSAIKTIDIVTKEIPADQKNAANTPHTPNISGAAGIA
NSATNVLENNNDILSSLDTRFTFDNFVVGPPNEFPYAAAMAVAESDNAVVKSNPLFLYGG
VGLGKTHLMHAIAWHCHEKNPNRKVLYMSAEKFMYRFIKALRSKDVMAFKDEFRSVDVLM
IDDIQFICGKESTQEEFFHTFNAIIDNNKQMVISCDRSPTDLDGVEDRIKSRLGWGLVAD
VHSTTYELRLGILESKLEKMDIQISPHVVDFLASKITSNVRELEGALNKVIAHSTLVGRE
VTLESTQDILRDLLRSNERIITVQEIQKKVAGRYNIRVVEMSSARRLRSIARPRQIAMYL
SKILTSKSLADIGAAFGKKDHTTVMHAVKKVGELMLKDSDFHEEITLLTRILQS

>gene_64|GeneMark.hmm|89_aa|+|66343|66612 >NVVL01000001.1 Rickettsiales bacterium isolate NORP64 Contig_source1382A_460, whole genome shotgun sequence
MNIKNKDQLIKKLLYQSCNRGCKETDLIIGQFARQNLDNMSSDELAIFEKILQLADGDIY
DWYTKKKPVPAENKSEIMVQILNFEPAQI

>gene_65|GeneMark.hmm|426_aa|-|66671|67951 >NVVL01000001.1 Rickettsiales bacterium isolate NORP64 Contig_source1382A_460, whole genome shotgun sequence
MKGMNDEKPVKQKAKRAKKSETQVKTISKTQTDPITDLQAAITAELQKPQTALVTQSGQP
QTALVTQLQAPQINLPPIPQAESTTRIQMNKRSPHDELFKSLLGNLDSAKDFLTHHLPAD

FRKLVDLSQITVDKESFVEPNLKKRFSDIIYKVKTIDNKDAFIMILCEHQSSVDNLIALR
LDKYRNLLLEREVVGKGKQTLPLVAQVVVYNGRDKYTAPLSLWEMFEDPALAKELMGGEY
KLINLQSMSDDEIRERNYVGMMEYFMKYAFKQDQIEVWKRFLKIFGRHLIMQDKDNNYLY
MKAFFCYTSRKLEDSSKDLLEELLVNELLKYEGGQPVRSIADAYIDEGIQEGINIASANM
AIILQESEARGEAREARGEATGKMQSLCQTALNMLEQGLGVNLTSKVTGLSEEKIKKLEE
LEFSKV

>gene_66|GeneMark.hmm|53_aa|-|68052|68213 >NVVL01000001.1 Rickettsiales bacterium isolate NORP64 Contig_source1382A_460, whole genome shotgun sequence
VTGEPKDSGVGTSRTQQKYKMPWDLLWILRSYDCTYVDNSSRSKLAKPSVKKY

>gene_67|GeneMark.hmm|981_aa|+|68310|71255 >NVVL01000001.1 Rickettsiales bacterium isolate NORP64 Contig_source1382A_460, whole genome shotgun sequence
MANSIAMYTNASETISGNVAAEKACGYKRLENVFTTSFIKTADGGRVSVNTEIKKTISQQ
GKLQSTGNNTDIAIDGSGMIPVTTSGNDMIKYTRSGNFRPDELGNLKNGANQLLMAWPLD
KEGKIPANSSLSSSLLPVNFANTKGTPIATSIVSIAMNLNAGEEAMRGNGVTAKPQTLGA
NKTAGDAGILSPDQLGSSGLEIGDRFKFTSTPPGTEKSVIFGGVVVSNTPSNATPIFGAT
SANMTFDFTGGAITNDQKLIIEVPGVGKYSFRPAAVGNAQRKEFSNIYELADAINNINSL
NARIGENGQLYVGPTDARKGLNFSEEGGGTLVSSFGFSNLAPSAVGEYRFNSLYTLRDAV
NRDAADYSLKATIEDGAIQITSLLSTTAFTVEGESLGVHSISSAILNPAGTEKGRATVFI
SAPGSNLSEGDFVRLENVGGGIIDGLYSVSGAGANGFNVFIPDNNPLVFPALLAAGQIAL
GAAALWQRAPGEKESITAGTITGVAGANITIDGGAAHGLANDDVIYVSGGTFFEPGAGGN
NITLPEGYYVVSGVAGNNFNVIATAIAAGAIAPGFNGVDLQFRKIGNDGAAYPNAATSGT
FNSRIFNTNAIATNTITYHVGNNHTYAVGDIISLTGLGGPQLIDGITIDDNIAYKITAIG
VGFVTFEVDATAGVAAAGATDITYADLAFVNTRTNNFSRFFEYFGIDQTKSQYDPTYNAL
NEDKNLSGGANASFTYPLSVYDSLGSPYRLMLRFAKLGNNEWTVELSAQKDEETGTYDIT
NIKPGSNGLIRSGILRFDSHGMLQGTPSGFEDPVVVQRNNGSAPSEIVIDWENKLSAIKS
GTVSQTKNDNNVEIIQSDGQGAGNLVKLQISPEGYIVGTFDSGETRNLYQIPVALFPNLN
GLEAGSSDTYEVNFRSGLAKYYVPGKSGAGKTIGSALEESNVDSTGELLKMKELSSAIAL
NARMASSKHQSNKQILDELRS

>gene_68|GeneMark.hmm|47_aa|+|71358|71501 >NVVL01000001.1 Rickettsiales bacterium isolate NORP64 Contig_source1382A_460, whole genome shotgun sequence
LVVCDKVARVSCKLRFFELACGFAAIADARIQQNNMKFNFRKNYYDE

>gene_69|GeneMark.hmm|1557_aa|+|71491|76164 >NVVL01000001.1 Rickettsiales bacterium isolate NORP64 Contig_source1382A_460, whole genome shotgun sequence
MMNKSSFLKKLLTTASVVVIAANAGSAFGEQKQTKEGEVAAIIDVVPGAGKNIQNSALED
AQFNNKDSLLVKAGTKKVTVSDARVIASIGLNGENAVELALEEDVSVSSVVDLSESGKKL
SVNVGAGKTFTLNKDLEGKFNLGGEGAADIEIAGGNFSALDNIMLGTGAGGGVGTLNIDL
IMTVDGEVDAAVAGSGLVHVNDDVTFANLIGKTNAVSIEVADKKKVTLQKKSNFTTIDMK
GESEFVVENGVELTATAFTVEADKATVHFEGNSDVTVAKMQGAGTDGFKLVEIGGAVNFK
TAAEYKAKETKLKGDSASAEFSNKAALTLETDFTTDTDKKGKVVFAEGSEVTFTGSIGAA
GAAFELVEVGKDGRLILANKDAKLYATSVETAVNETGEFILQGEGAELHANVGSSAKKFK
NFGIAMTAAESTVSLSNHIYVSGAVALSTNGKNNTLVMKEGAKIVGDIVSEDGKGTLLVE
GVAAIDGFVGLDAATEKYTVNKQEINGGTIKALRFGKDGVLTVSKNRVNTREIDFTAGGI
LNLTEDKVANFANGLNIKGLGSITVNKATKGKEVKVETKLGESKDKMFTLFAATGGADIE

FTKSSYIKKVAIGAATLKLGEIDGKFLIGELVHSGDAPTLSITQNTTLLEGTNLSSGAKK
LEAIQFETDKNLIVGKGVNLHTNKGFIAKGNNDLTFEGGSIVDSVIGTKDSKFNSVTITE
AGDISFLKEIFLTNNLTVDKAGTTSFGGDVTLTDLILTEGTANINANLTANQIKTNAVDK
TTLNFSSASKAVIDSKVGDGVTVFKEVVLNGTDIEFKKTFNTKSLNFKKGLTATLSGLQE
DGLKNTKITTEDAHKGILVLNKDNNQQFDEEVGTKDKHFGIIKLVADNTNIITINNNFYS
ALHTATAEKGRVNIIKEDAVIGSLGEASDELENVTFTDSASVLDGVWAKDIEIATGKKAT
LSYIVASSNEFKLNKDSEAVFTGKSGKLDATVVASADGFGKMTFSQTLSMITKSIGTSAA
KIAEVTFTGDADSRVTVLSADIFANEINVGKKHTIMLKNAIEMGGKTTFQSESITEINAY
KLTLSSGDSALEEGAEIAFIFNDVDDHGQISVKGATTKLDAKDKTIDVVVIDDKSALPVS
EEEYELFITENNGTLTMDGLKMNVKHEGNDFVQWTQKDAAITLLRNNVAKEKLVLVTDKN
TAEAYGDATIKGDARDLVTALGNMTTEERQKTLDSLTDLAKATTSVTSNTIAGTQNAASH
ALQRRVDQLGISSGSDSSSAYGAWFSPFYGTTTQKAVKGAAGYKGSNYGGTIGGDLQTDS
GLVLGLTASYAKTKLKHKDAKSGDTTDVRSFIFGAYAAKQITDNWFVQGDVSFGTNKFKN
TENRFLSKVLTEKAKSKYSVNSFSLGMLAGYSYKIADAVVTPVAGLSFSRVGGVSYKEKG
TSFINRDVKKTAVNVLEPMIGFSAHMTTDMDGIAITPEFHTSVKYDVIKSDAKATVTHSG
LNKKILYKSKKAHQAKFNIGVGVNAVAGIYEYGASYDLDIAKKSLSHQGSLKVRVNF

>gene_70|GeneMark.hmm|370_aa|+|76355|77467 >NVVL01000001.1 Rickettsiales bacterium isolate NORP64 Contig_source1382A_460, whole genome shotgun sequence
MEKIKLAEYIWLDGTKPTSQIRSKSRVVSLLDSPNLNSPDLDSPDLDSPDSDSLNLDDFP
EWSFDGSSTNQAVGEDSDCILKPVNFVKDPLSETDGYLVLCEVFNPDGSVHSSNSRAILR
KALENGGADEDVWLGFEQEYTMFKKNIPLGWPEHGFPGIQGPYYCGVGSEQIFGRDLARR
HQTACIEAGIMYYGSNAEVMPGQWEYQIGYRGIDSEDAGALNISDHTWIARWLMHRLSEE
YNIHISHENKPIKGDWNGAGMHANFSTKNMRDPQKGRATIKNALIQLESTHQEHIKHYGE
RLEERLTGKHETSSISTFSAGNANRGSSIRIPRPVADKGYGYIEDRRPGANADPYMVAAL
LISTICSVSK

>gene_71|GeneMark.hmm|489_aa|+|78202|79671 >NVVL01000001.1 Rickettsiales bacterium isolate NORP64 Contig_source1382A_460, whole genome shotgun sequence
MKNLESLKGHHNSTKLETTDLSAVPSVEEQGRSQGSGSGESNNNWSWRSFSWQGMVLGGV
LAFSVYNFWKAVENRAKSREYKALEQLKSNLKKLSEECSDNNTTIEKLWQNMSASSFIKM
LLDDIKKNYNSDPSTLAKILSDLEVNYLVYIIASYCNNYLYKCGHTGREEVRAELEFARE
LLASKCNQTLDDLNAAALRGHPNFSELYIQITHILGRTFLYRDNTEADIENGIRYFEEVC
SFGEQYQQMGGQKLFYLLLSARNIGFMKLCQAEFRENEPIDEPTCSTKKASLRSVIEEFK
QLKQDAEEYTEFTFKDGELTRETIIPANDAYNRLECAQRIMVCYYNLFLYGAREDELADD
MREIKDLIGTKHTEEHSMAADIAHLRQTNLKKYADILNMFAKLWSALEGLALPEEVVDMH
VNELREIIIGQVDSKLTSREEILQVAKDMSVKGSFPNLEALELLESCNSDQFEQLVDDET
PLAGEGESA

>gene_72|GeneMark.hmm|220_aa|+|80302|80964 >NVVL01000001.1 Rickettsiales bacterium isolate NORP64 Contig_source1382A_460, whole genome shotgun sequence
MKLSEKACNQDEKFVVALDGPSASGKGAIGSALAQEFGLVYVQSSIVYRGLAYICLQKGI
SSGNTHAIIKLSETEDVISEVRSVDLNFEKIGEFASEISVLPEVRRNLGEYLTHLIKTTP
RIVMEGRDIGTIVAPNADLKIFITANVEIRAERRFKQLILEGKECTLGAVLASLRGRDAR
DSSRSVAPLEAASDALVVDTSSLSPEEVVSKIKDFVACRV

>gene_73|GeneMark.hmm|572_aa|+|81067|82785 >NVVL01000001.1 Rickettsiales bacterium isolate NORP64 Contig_source1382A_460, whole genome shotgun sequence
MTIKKRFIPQLADLTPSTEDFSELLKSVDTSHIKEGSVVKGQVVEVGSEVIIVDVGLKNE
GRIAVSEFQTAKGQALPNVGDVVDVYIERVASRRGTVLSREKAIREESWVKLEEVFKKEE
HATGTIFGRVKGGFTVDLSGVVAFLPGSQVDVRPIKDPTSIMDIPQPFQILKMDRKLGNI
VVSRKAILEESRSEAREEMLSKIKEGDVLEGVVKNITDYGAFIDLGSVDGLLHVTDISWS
RINHPSEVLTFGQDIKVMVIKYNAESKRISLGMKQLEANPWDDVVNEFPLGKTMSGKITN
IADYGGFVELKEGIEGLVHSSEIAWGKGNQNPKKLLSVGQEVEFVILDVDTERHRISLSI
KKCQDNPLIKFAEKNPVGTIIKAPIRNITDFGMFVAIDERNDGMIHESDLSWEKSGEDLL
KNYQKGDEVECKVLNIDIDKDRVALGIKQLTDDPYEGSTDTFKKNMVVTCEVISIDDDGI
EVSVEGKAQGYVKKFDLSSERYEQKPDRFTEGDRVDAKIISINKETRKLGLSIKALEIEE
RAKVIKEYGSTDSGATLGDILGAALGEDSKKK

>gene_74|GeneMark.hmm|101_aa|+|82963|83268 >NVVL01000001.1 Rickettsiales bacterium isolate NORP64 Contig_source1382A_460, whole genome shotgun sequence
MTKKIMFVEDLQNGWKLYGKMGNGSQLSCDRSRKLNKQVGWFVGFIQKNDQSIAFAYLIA
DDSPEKSYASLRAKAAAKEKLIAMTNYPKSHSFSLLVGNGS

>gene_75|GeneMark.hmm|442_aa|+|83488|84816 >NVVL01000001.1 Rickettsiales bacterium isolate NORP64 Contig_source1382A_460, whole genome shotgun sequence
LINYLYSKAHGGEFILRMDDTDLVRSKDEYKENIKRDLKWLGLEWDSSFNQLSNLEEYEQ
AKAKLIAKGRLYPCYETPEELDIKRKLQLSRGKPPIYDRAALDLSPEQIAIYEKQGRTPH
YRFLVESAPIIWDDMVKGTVKYHGSNLSDPVIIRADGSMTYMLCSTIDDINFNITHVIRG
EDHVSNTAIQIQMFEALGAGGSGELVPSKLAPNELAPSKLVPGKSVPVFGHLSLVKAKDE
KISKRDGGFEISALRGEGVEPMALNSFLGLIGTSVQMMPHKNMRELIEKFDITTYSKSPT
TYIRGELDLINHKLILTYDFADIQDYLAQNGLQQINEKFWLAVRPNLQKLSDIKEWWRIC
HEITPAISKAPELMKIASDALPEEITDATWKEWTKAIMAASGKKGKELFMPLRLALTGME
TGPELKNLLPLLSRQEILKRLR

>gene_76|GeneMark.hmm|948_aa|-|84998|87844 >NVVL01000001.1 Rickettsiales bacterium isolate NORP64 Contig_source1382A_460, whole genome shotgun sequence
MTNNKKTLNKILSSCCIAALISVNGTNVAYGGGKNTFRSEFLDQIRGGFQLKEASQQKGD
REPSEAFDGLGKAISNSFKMIGDDVDNSDADDSDWSDSDDSDSDNHDRRPAKNLLGKAIG
EYVESKASPQQEATTTQTRISDVEQELKAIDNITFVRGNQIFHNNGRQVDGKTFGDHAGF
IEAYKESTKKLLREKLAILNAQKNVTLPKNTHSAQTTEVEIKTPAVPPVDDHKAESINDL
LRFAMMNRQVIQTGGKPPVVPSVNDSKMEFTNNLMPQQPITMEHLEAKTVEEAKKVTFAP
ALEILHAEALCAEQADDLMLQRPITMEYLEADTVEEVKKVTFAPALEGPTQVTFEARRAM
LNSIHNLRKDQEPEESTVSIKQDISDLQAEIARLAEEIKIAKAAGEERAAKLRSIVVSSP
EHRNNMIQLHKEIKNAYDAKKRAEKEGVGEIPEDFAQMWDDIDPSTDTDEEILDNFEGMW
SRLEASALEESPTEKLKRVVAKMSKSTNSKMYAQMQAAKLATEAVARFTAKKRAAEKASR
VAAARLAAAKLAAEKFVQNARSAALGKGNTSDPRLGLVVVAREKFDQATSNLTLTKKSKA
NLKEKMKTALHATDDLGQMANTLDNATNHVEKAVFSAQAAKAHAAAGNVNMTLTAESANT
NAASRLAYAYAAGDDDTGFTHGLWVKGFVSKGSLKDQYKMTNNGFTIGSDIEINDDTILG
IAFTLPKTKVNYDKKFAMNSKISSHVGTIYGSHNFANHVFVNGKFSYGKGTVKTDRKVNG
GIAKSKFKPTIMNIELDSGRSFMLGDGYSITPKIGCGYSAYSAPAYKETGASNDKISAAK
RTAFNLKAGISLAKSIAFESGIMLKPEIHAYVEHEVGVKNKNQTIKHDGIWDIESKLHNA
KNKAVKINLGSSLKVSANERVDCDFGYDFNKKAKFSSHTGYLRLRLNF

>gene_77|GeneMark.hmm|42_aa|-|88129|88257 >NVVL01000001.1 Rickettsiales bacterium isolate NORP64 Contig_source1382A_460, whole genome shotgun sequence
MIFKSQHELWENILYIVHWHIAPKISSTRLFCQKFEAIMNIN

>gene_78|GeneMark.hmm|192_aa|+|88865|89443 >NVVL01000001.1 Rickettsiales bacterium isolate NORP64 Contig_source1382A_460, whole genome shotgun sequence
MTQRYFSNMTSKVLVASPFAMRGTMFHESVIYVLEHRPEGAIGFIVNKPIINATPLSEVV
KKLDLDIDADNLKLAPHVGGPMTEKGGFFLHSSEYTKNIVSTHAGSGLSVSSSDVLLRDI
AKGVGPEYALFVLGYTFWEEGEMELEFENNLWIATEANRELIFSPDSGLKWGKTFQNLGI
SSGGFAPMMANC

>gene_79|GeneMark.hmm|246_aa|+|89499|90239 >NVVL01000001.1 Rickettsiales bacterium isolate NORP64 Contig_source1382A_460, whole genome shotgun sequence
MDFNPEPEGSLSLLGKWRAHSGRNNTHELLAILKGLSAGQIGKIMKVSEKIAKQNHQRFQ
TYENLPEKPAIFAYVGDVYKKMEPGTFSLEDLEFAQDNLRIISAFYGLVRPMDKIKAYRL
EMVNKLPGAPVKSLALMWQEDIAQMINEEIKTHPNKYVINLASDEYSAAIDAASLDAKMV
NIHFREIRDGVLRNIAINSKRARGRMAGYIIKGRIGSPEELKSVSALHYAFDAGLSDESN
FFFVNS

>gene_80|GeneMark.hmm|882_aa|-|90335|92983 >NVVL01000001.1 Rickettsiales bacterium isolate NORP64 Contig_source1382A_460, whole genome shotgun sequence
MSKLTTEQIRTTFIDYFKNNGHTHVPSSSLVPHNDPSLMFVNSGMVQFKDVFTGAEKRDY
TRATSSQKSVRAGGKHNDLENVGYTARHHTFFEMLGNFSFGDYFKEEAIYYTWNLLTKEF
GLDKSKLYITIYHDDDEAYSYWNKIAGFGDDRIIRIATNDNFWSMGDTGPCGPCSEVFYD
HGEHIAGGLPGTPEEDGDRFIEIWNLVFMQFEQINKDTRIALPSKSVDTGMGLERVSALI
QGVHNNYDIDMFRDIISHTESILKVAAEGEALFSYRVIADHLRSASFLIADGVMPSNEGR
GYVLRRIMRRAMRHAHQLGGKDPVMYKLLPKLVDIFGHTFPEIKRAEEFISSVLKQEEER
FKTTLDRGLKLLEEEASSIANSGSLPGNVAFKLYDTYGFPLDLTEDILKKRNIKVDIAGF
DKNMQEQKERARKAWNGSGESKTDSIWFDIMSEFGATEFLGYSLNSAEGKVFTLVQNGKR
IKQISEKGQKFTLISNQTPFYGESGGQMGDIGMISSSSCKIKVVNSLKYLGKVIGHICEL
EEGLISQNDEIKLTINVTHRNHLRMHHTATHILHAVLRDVLGKHITQKGSLVAEDKFRFD
ISHPAALTKAEIKLVEEKVNHIIMENSQVNTDLMSTDKAIESGAMALFGEKYDSEVRVVS
FGTGAEEKTPYSLELCGGTHVKRTGDIGAFKITYEGAIAAGVRRIEAVCGKFALASIAKD
EELLGNVMKSIKSNKADLLQKLTGLIDSKKDLEKQLENLHMDRLMLSDSEINDSATKIKE
ARVIYRQIDGVSPKMIRMAAENLAKKHDDLVIIYTSKSGAKLSVLIAVSKPLSEKHNAGT
IAKELAPLIGGSGGGKPTLAQIGGGDVGKLQQLKDIIMRMLG

>gene_81|GeneMark.hmm|516_aa|+|93170|94720 >NVVL01000001.1 Rickettsiales bacterium isolate NORP64 Contig_source1382A_460, whole genome shotgun sequence
MSSLSSSKLGFLVNILNNLFHKRNLIILIGKNGITVCALKHGKLQNSLFVDLEDKSYMRK
IGNFLKKYKKFYVVFLLDHKDCKLKHEMTPVLTSMLKPNQLDEFISNEYDPEDIVAYNVY
DIDYQNGEVWNSCIASVPFNKHISKLLEYILNHSFKYSGMYFLSLEFETIINRILKKSDN
TNYANHLQIFAVVTQSSGIRVVVKYKKDIMAEETIEYPAGKSDMYLLGTIEQAVSDKVLF
YKEYIKKMGLETCVILLVEDVLVKKFSKLEFDNCTTVIVSRDEISNARKYREERFQDTVL
MELFNNFNSCIAFNKPLGAITKMTFINRVFFKPVIFFLLLGLAALVSIKYQTILVKKEIR
ELNQKYYTLSEEYREVQKQHPDIENITTLVDLYNLQNLINKKMVSPTHLKTMLIKSYRKN

VKITSLQWKVLNPTKIELSENQLRITMKIVYKGDANSMITGIKMLNEYVDDLRAIFPKHN
FIYRRDAEEILQIANRITIPASVIIEGNTEQVKNAI

>gene_82|GeneMark.hmm|210_aa|+|94710|95342 >NVVL01000001.1 Rickettsiales bacterium isolate NORP64 Contig_source1382A_460, whole genome shotgun sequence
MLYKLIIHLKNIIFFKVIIYVIGLVLLFVLIPIFQESYEQALQRKEKSTKFLKSTALKLD
SIMDFEDKILGVNNDYLRLIKNSGKIGCAQRAKLLKNLKPLSEKYKLFEPMNISLLRYVG
HESAFKTNSNIKINLYVLEVNFTAANYNIILRILRDIYRMLPKRSNVADISVNKVEGLTP
DIISKLNYDRAPGELDVKIKIYLREIVYEK

>gene_83|GeneMark.hmm|393_aa|+|95332|96513 >NVVL01000001.1 Rickettsiales bacterium isolate NORP64 Contig_source1382A_460, whole genome shotgun sequence
MKNDIILPPGTDSRPFQEQFNALFADNLSENLKNSIAMLLAIDYAARLSSKYKIEEHAAI
CARRLKQYIGQKLVLGEEFSSVMTFFAVALTPEEWQEDMDQSRREKSQTKEALIDSIITA
QFGSLNSSQKELVRMFLQDSLSSKRSGNFEGEGTLKGEDGPERDGKLKEEGHLKGMANLA
RDGRRLSQNNAKFTQENNIFHQKNNAFINFIIANSDLFQSIIISAQRNEKRDKEILEFTK
MHITKIMNYSRQLERKISSIKNIVEKSVFSASVLVSASIGLVIGGLALPLLVVPAIAAAV
KYSPAIGQKLSSSIVKNSSLIQEETEGLNKLKLNSLSALNKGQNQGKSLEHSGLKEDMKD
LIQDIITKQVPKRSEQPASGGDSSKKNKLDKMR

>gene_84|GeneMark.hmm|149_aa|-|96859|97308 >NVVL01000001.1 Rickettsiales bacterium isolate NORP64 Contig_source1382A_460, whole genome shotgun sequence
MKHSAVETLVGFLVILVATGFFIFAYNTSNVAKAANSYAIFANFQNIDGITDGADVKLSG
IKVGYVENVTLEEETYFATVKLRIDSNVDIPKDSRAIVSTSGLLGGKYIRINPGPSDYNI
PDSGKIIFTQSALNIEDLIAKLMYSLTSK

>gene_85|GeneMark.hmm|114_aa|-|97326|97670 >NVVL01000001.1 Rickettsiales bacterium isolate NORP64 Contig_source1382A_460, whole genome shotgun sequence
MSFIDNFCIRFFAKHVGSDRFGNKYYLGQKKNYLGKAKRFVTYNGQPNGAKVPPDWHRWL
HYSSDDLPEMQDAIKLQRMDAGGRICDIKYADISSASSSKSSSTYSSWSPNQKR

>gene_86|GeneMark.hmm|160_aa|-|97671|98153 >NVVL01000001.1 Rickettsiales bacterium isolate NORP64 Contig_source1382A_460, whole genome shotgun sequence
MTAPAMSASSTSLPFVEIYTDGACSGNPGPGGWGALLIFEDTQKEIFGYDIETTNNKMEI
FAAISALNILKKSCRVNLYTDSKYVQKGITEWIHNWQRNGWRKSDNKPVKNVELWQSLQQ
KIEKHEINWCWVKGHSNNKGNDIADRLAVEGRELAKRKVK

>gene_87|GeneMark.hmm|164_aa|-|98159|98653 >NVVL01000001.1 Rickettsiales bacterium isolate NORP64 Contig_source1382A_460, whole genome shotgun sequence
MLGIVHKVLWSLQNEGMVNDQCFYVSFNTTSLNVIMSERLKQRYPEEITIVLQYQFRDLI
VFDNKFTVNMSFDGIPETIEVPFDSLTGFVDVNANFSLKFSRIAENAPSVVDEFAGRANI
SKLKKHPSPSVLKVTPVKSTKEPKKKTAASVIQLDQFRKKHSDK

>gene_88|GeneMark.hmm|36_aa|-|98810|98920 >NVVL01000001.1 Rickettsiales bacterium isolate NORP64 Contig_source1382A_460, whole genome shotgun sequence
VLSKEIIFTQYDTAGSFDLGSKKVNANDNNRYAVAA

>gene_89|GeneMark.hmm|465_aa|-|99159|100556	>NVVL01000001.1 Rickettsiales bacterium isolate NORP64 Contig_source1382A_460, whole genome shotgun sequence
MLKKINDFVVKSLSFLALLAIVSCNDSTTKSSCDNIPELEKLSNQGEVVSQANLASCYYK
GEGVAKDFDKAVLWYRKAADQGYADAQNSLGNRYYNGEGVDKDFTKAVLWYRKAADQENA
DAQHNLGNRYYNGEGVAQDLSKAVLWHRKAADQGNATAQNSLASCYYEGKGVAQDFTKAV
LWHRKAADQGYAAAQNNLGYLYYKGEGVAQDFTKAAKWHRKAADQGNASAQNNLGNRYSN
GEGLAQDLSKAILWYKKAADQGNSFAQNSLGVQYAKGEGVTKDLFKAVQYFTKAADQGNA
AAQNNLKRKLLQFASPFGCKIGITTFEEVEKMYKLTQSGSNKWSGGMMYNITPITQVDFT
GINNINLIFDDNKIIQGLIAKFDKNKFHDLFLFLESKYNIVSKQIPYVGNKSAKFSMING
TIELESMHLSGHTSLIFTTNKLDKSFKKGTQLEKTLKTQKEKSML

>gene_90|GeneMark.hmm|959_aa|-|100672|103551	>NVVL01000001.1 Rickettsiales bacterium isolate NORP64 Contig_source1382A_460, whole genome shotgun sequence
MFGTDVGVDMAIVIGFLILTLVVGFGQSGKIENIKEYALGGRNFSTGALIATVVASWVSG
SGFSIVTTKTYYGGGYHLVATFGMMVSFLITAFYFVPRMGEFLGKTSIAEAMGELYGEKI
RVIVAISGIIASSGYIAVQFKVFGDIVGYFSNSDRTLSIIITGLAVTAYSAYGGVRSVTF
TDILQFFTFCFAIPLIGIMIWNKLYMVDSFNMMTALEVDKFNYKKILDVGNPQFWEIIPL
MIYFALPGMKPSIFQRISMGTNLAQIKKVFVVSAFLIILVQIATAWIAFLVFSANPNIEQ
GELFGYIVDNYTYTGLKGLFIVCVIAMTMSTADTEINISSVLFSNDACAALNITPKSRLL
LSKIFSLFLGICSIFVALSTQDLLQIILSTASFYMPVVSVLFILTIAGFRTSPKSALIGM
SAGFFTVIIWRLAKIEMNPIAVAMFVNLVFLMGSHYLLDQKGGWVGIKDKSFLEAQKLES
IQKRQQFFESIKNFNLVDFYKKHAASDNLTYVGFGIYCAIYTITTMHSTEVNLLQDNGQI
ILAIYQIMMVTGIVMAMYPLWPLNITNNIKERIIQTWWPIAIFYMMIFFSTFFVLVNGAN
HLQPVVFAINMIIAILILGWKTASVFIVGGVYLAVQFYKYYMGIDYVDISIGSPQFILMY
SLVLIGTILIIFLKPKQEHLEKTEHQVGSLAGEVGSLTTEVNTLDTKVVGLNEKVVHYSA
RATDNAREVERLGATSQKILNNVSHELRLPVGNVINFASMMHDGLEKFSKEQLKTISKEV
YQNSNRLSTMILNMLDLATLDVKKIELMKRKVNFSKLVEERVESCRNIYVDSKPLKFKIN
IEPGVIAPIDRNYIRQTIDNLVINAITFSETGQITISVAKQANFVTFTVQDQGIGIPASE
IYDVFTPFKMASNTETKAEGRGVGLALCKAAVEAHGGKIKVESHGVGVLFRVVLPLNSE

>gene_91|GeneMark.hmm|536_aa|-|103855|105465	>NVVL01000001.1 Rickettsiales bacterium isolate NORP64 Contig_source1382A_460, whole genome shotgun sequence
MLKKLLFCITLILLYPLSCAELQRTGQPSYGASLKAQNDDLEYLGIFSPKVNFDYQAPKL
QEMLAKRSSYKNLITRLYLTFIYWDQNRNSIDATNESQELFKMLLAKIPDANYCYSYRSD
ASTEEMFFRWFINKPFVWHVERYEIFKSADLEGKTYQLPLWLVLKYPQIVKNDAYRKPLK
VNTPYKIESLPEFTALESTLKRMTEGRWETTSRITHYASNVNDIGAQHEYIKRYMNINIL
RILNYNSNHTTEQNLKPIKFWGGKGLWNSLVYKDFVVSFEKTVIALQKFYQSTPYIIGYE
QLNRNPLISEQLIRKILSSLVYDYAPIDDSDFGVYMMETGDEKSYYKIMKSVLGSSSSHN
SNNSAGLKKADSRASNMFLSRAILNDFSAETIELIIRLYGGVNSCNYSETPLMNAASRPE
ILRLLIKNGAKIDTVNTFGKTALFYAIQFGDYEAVKILIDHGADVNKALYNLETFNNLSD
NYELEQFFMLEYVADFTPLIYAKKYASKDVQNLLLKHKATLGKVDLPLIKEWINKK

>gene_92|GeneMark.hmm|111_aa|-|105617|105952	>NVVL01000001.1 Rickettsiales bacterium isolate NORP64 Contig_source1382A_460, whole genome shotgun sequence
LIKVIFCYNMPGKLRSYAKSSCARIFRSKLSCAADLRLNSFLVYPYGEVAEWLKAPAWKA
CLRAIVTRVRIPSSPPAFALASFSWQASFKFTRAKAARRSFSEGGLYPFYV

>gene_93|GeneMark.hmm|272_aa|-|106022|106840 >NVVL01000001.1 Rickettsiales bacterium isolate NORP64 Contig_source1382A_460, whole genome shotgun sequence
MKNIMTKISKKLLEGKKGIITGVANNMSISWAIAQLAKAHGAEIALTYQGEVLEKRIFPL
AEEIGCDFVQECDVTDEASMDRLFAAVKEKWGKIDFILHGIAFADKNELKGRYIDTSLPN
FLNSMNISCYSLTALAKRAEPLMNEGGSILTLTYYGAEKVIPYYNVMGLAKSALETSVKY
LAHDMGGNNIRVNAISAGPIRTLASSGIGDFRSMLKTHAATSPLKRNTTQQDVAGSALYL
MSDLSSGVTGEIHYVDSGYNIMGMNTPPKETK

>gene_94|GeneMark.hmm|502_aa|+|106903|108411 >NVVL01000001.1 Rickettsiales bacterium isolate NORP64 Contig_source1382A_460, whole genome shotgun sequence
MFLTRKVSFIAGIICAASFAPVFFTPGIFALSVLCAQVSRANGVGQAAGFGYAFGFGFFL
ASLYWICFGVAVYIDQFWWAIPFALFGLPAFLALLIAFQAAIAWQFRKCHIYKYHLYHFF
FCLIWIAMEWFISWGFTGFPWCYIGYAFSVSDKMIQASSIFGILGLSFAAVYIGSTLYSK
KFLILRLLTGGAICIALFAFGHYRLEQNPTVLSDVKIRIVQPSIPQTAKWDPDIFWGNLD
KQIAMSQKPGSPDIILWSEAALTVPYYYAPVHNALMSVFTKEGQVLLSGGVSDNGKQGAD
YEVYSSLFAIDSAGENLFDYHKSHLVPFGEYMPGRKYIPLKKITHGSIDYTPGTRKTVYL
QLFNLSVQPLICYESIFFQEVKISNSDADIVINVTNDAWYGNSSGPYQHFEISRIRCIEN
GLPMVRSANNGISAIMDPMGRVLAKLELNQVGILDGYIPLKLVLPTIYSEWGKWGVCSWV
SLVLILQSMIYSFFFCLVKRRI

>gene_95|GeneMark.hmm|103_aa|+|108501|108809 >NVVL01000001.1 Rickettsiales bacterium isolate NORP64 Contig_source1382A_460, whole genome shotgun sequence
MSDKKETKGRADNVDRLVSHRLKMRRMMLGLSQNDLGKAVDVSIQQVQKYEKATNRISSG
KLFAFSKFLKVPVAYFYDQSDDSGNIIGSVLAEEASSYSSDLK

>gene_96|GeneMark.hmm|180_aa|-|1|540 >NVVL01000002.1 Rickettsiales bacterium isolate NORP64 Contig_source1382A_922, whole genome shotgun sequence
MFEKPLISFDFAIKFLLKSKEDYEIIEGFLSALFVANNYKPVKITALLESESNKEAAHLK
KSVADLIVEDTDGNKFIVEIERSFTPNFTHKACFNTARLISDSLSTSQDYSKIQKIFHIS
LLYFETAGMEKPIYHGKTIVHEVDTEHPINIDIMNKGIATFENKNIFPEYFFISIPRFND

>gene_97|GeneMark.hmm|334_aa|-|929|1933 >NVVL01000002.1 Rickettsiales bacterium isolate NORP64 Contig_source1382A_922, whole genome shotgun sequence
MSLLNAKSIYKPFAYPWAYEAWHTQQKIHWLPEEVPLADDVKDWNYNLSAGEKHLLTQIF
RFFTQADIEVNNCYMKHYSQVFQPTEVQMMLAAFSNTETIHIAAYSHLLDTIGMKESEYQ
AFMKYKEMKDKYDYMQKFGVETKEDIATTLAVFGAFTEGLQLFASFAILLNFPRFNKMKG
MGQIVTWSVRDETLHTNSIITLFKTFVQENPEVVWNEKLRSRLYEACATIVHFEDAFIELA
FEVGGIEGLTAREVRQYIRYIADRRLMQLGLKEIYLVDSNPLPWMDEMMNGIEHANFFEA
RATEYTKAATTGTWDDVFTNIDKGKAAGKGQTAG

>gene_98|GeneMark.hmm|620_aa|-|1991|3853 >NVVL01000002.1 Rickettsiales bacterium isolate NORP64 Contig_source1382A_922, whole genome shotgun sequence
MTMIKKKKFEIKIDRSRDDLLTDFGKAMLKDRYLLKGEDYQDLFARVSSYYADSQEHADA
MYKYMSKLWFMPATPVLSNGGTNRGLPISCFLNETKDSLEGIVDLWNENIWLASRGGGIG
SYWGNVRSINELVRGNGKTSGIVPFLKVQDSMTLAISQGSLRRGSSAVYLPMDHPEITEF
IDLRRPTGGDVNRRALNIHHGVVIPDAFMEAVENNEEWKLISPHTKKVIESVSARELWSK

LLTTRIETGEPYLLFIDSVNNNIPEHHKKLGLKVKTSNLCAEITLPTGVDHLGNQRTAVC
CLSSLNVEYYQEWKDDAEFIPNIMRFLDNVLEDFIESAPESMSRAVYSAKRERSVGLGIM
GFHSFLQSQNVPIESVMSKVWNKKIFEHIEEAANASSVMLAEERGACLDAAEVGAKERFS
NKTSIAPTASISIICGNSSPGIEPYAANSFTQKTLSGSFTVTNKHLVKLLQDKGFNNEQV
WSSISTHEGSVQHLNFLTDHEKDVFKTAYEIDQNWLIELAAERTPYISQSQSLNIFIPGN
VSKQYLHDIHFKAWKKKIKSLYYARSTSIQRADKVSHTVVQAQEMIDLEKKNNNEPSKLA
KSALPPERIENNYDECLSCQ

>gene_99|GeneMark.hmm|263_aa|+|4233|5024 >NVVL01000002.1 Rickettsiales bacterium isolate NORP64 Contig_source1382A_922, whole genome shotgun sequence
MLEFINLFAKIILEFSHESLIVPLIIIGYIWIDKKTFFHGICLVLISMIFNAGLKATFQI
PLSPALGKEGFAFPSGHMQTSIVLYGFLYKFSKNTIIKVSLVILLTLIAMSLIYFGYHNI
YDILGALFFGLMLIFGYVSIKSKMPESRLFLAIILFTTCCMLYIRLIYQISPHLWMAYYA
LFGLLVSEYFFDNKQIKLDAKKSKIIATIFCFSSLFLTNILFALLPNALSFLQSLRWFLI
GFFIPFSVFLSYSIHCKGKAGDS

>gene_100|GeneMark.hmm|848_aa|-|5154|7700 >NVVL01000002.1 Rickettsiales bacterium isolate NORP64 Contig_source1382A_922, whole genome shotgun sequence
MKTLLKLTLWLTLLALSLSGVMYAANNGQLNAATKHALRYYLKSQGVKAKFSDFQFKDGV
LTSSVLRLRKGSATVRITNFKLQTSLSGNWSAPIFSAKLNPAQTTVLGQNDVQILNTEIS
YEARMNLLSGDKDYNFSVSNIKIDKFKDIDNNPLETGQAYYKYSSHDGEQIIDCKVQFGS
TTYLNITNLDNNKIQIEAKNIPLMFYKLVQKLSPEHGLVNFFESYIKNGHVTEASLVLDL
AAETISEDSLFGNVALRGVDYQYSKEFPTISKMDINLDVKGGSLVFAIDRGYSSSILLYD
GVIKMDWLGAEKTVLFITAKGKGDVLGLVDFISESTHAETSKANIDFRKLKGEAFIDIDV
KVPLKTSSVNIYNISADIQNVSLGIFKDSVHLKETTLSGRFDGDQVTIRGDGELNGFKSE
LDFVYNIEDESGTGIGAEVAVGAKDKLEFDHKLDIKTYFKSAAKMSGEDQKIGFISLMGG
DSVVDFHYTNKDSKGFINIESDISDLDLYLDKLGIHKKKNDKANLTVNGSFDDPTTGVFQ
FSITGENKLSMSGEIAMENEEFTIDIKELKHKKTDLSGKIISKGDLLSTELHGEALDLSS
ADMMQLLEKEQDDGGVVLKLSIKKVYLKDNIRLDDLEVDLECVAIQCTSGTISAMIGTKQ
IEIFVTTDNGKEKWLIRSGNAGASLRGFGAYDAMRSGALSIDLTTDREMVKSGEIIPILS
GTFEIKRFVLGNTPTMARIVSFVSLPGMVNAILGNKDIVFSSMSGNFSFKNSILTIENST
AKGPFFGFSLKGTVNIADRIMDINGHVTPSLYGVSSIVGAIPLIGRIFTGHKQQRGLISA
SYNIQDSY

>gene_101|GeneMark.hmm|433_aa|+|7879|9180 >NVVL01000002.1 Rickettsiales bacterium isolate NORP64 Contig_source1382A_922, whole genome shotgun sequence
MKIAFIGTGYVGLVSGVMMSHLGHDVVCLDVDENKINKLKNKEAPIYEAGLDEYLAKYAN
SPALQFVLGYDATIQDTDCLFVTVGTPPKEDGDADLSGIFDAVQNACKYVRSECVIVLKS
TVPPGTCAQVREFMQNQGKTIHIVSNPEFLREGSAIKDFLEPDRIVIGAESEHAFEVMRQ
IYKPLTDAGVRLIETDLATSELIKYASNMFLANKIAFINEMADLCEVIGADISTLSTGVG
TDKRIGEAFLKAGPGFGGSCFPKDILALQQLSKKVDSDFLVLDAVIKANSNRPGRMLKKI
SKAIGGDINGKKFAVLGLTYKAETDDLRSSPAIDLINLLQEKGGHVVAYDPEGMVNADKY
FRKLDCASSAMSAIEGADAIIIATEWEELKKIDLEQAKRVARKPVIIDLRNILCPKEVLS
KGFEYYSIGRKNY

>gene_102|GeneMark.hmm|1149_aa|+|9198|12647 >NVVL01000002.1 Rickettsiales bacterium isolate NORP64 Contig_source1382A_922, whole genome shotgun sequence

MTNKFIHFRVQSSYSMLESTIKIPQLVELTKNNWMGAVCLADRGNLFASLEFALEASKAG
VQPIHGAILNILFSNKGKDDFAEIVLIAKNDAGYKNLLKLASYTFTKNDRKTCNHITFDD
LEKHNEGLIVLSAYTAGIIGKFLLQNNLPSAKNTAKKLQQLFGDRFYFEIMRHGSEKEKR
IEDEYLQIANEFGIPLLATNQVLFSDVSMHDAHDVLLCISEGVVQEVQDRKRVSNNCYFK
STAEMIALFKDLPEAIENTVYLAQRCSIMAETRPPSLPKFSNGEISEEVLIRDQAKAGLE
EKLAQKIDKEHIKKADQAPLRKAYFERLEYELGIICRMDFSGYFLIVSDFIRWSKENGIA
VGPGRGSGAGSIVAWVLKITDLDPIEFGLLFERFLNPERVSMPDFDIDFCQERREEVISY
VRSKYGNERVGQIITFGKLQAKAAIKDVARVLGLNYSTADYLTELVPFNAVNPVTLSQAI
ADVAELKGAARGKGLYNLEGDNELIKQVLSTALTLEGLQRHSSTHAAGVVIANQDMTQIV
PVYKDVNADMLISQYSMKYCELAGLVKFDFLGLQTLTVITKTIKLLEDSGVVLDIEKIPF
DDKKTFKMLANGLSTGVFQFESIGMKSSLRRLKPDNINDIIALGALYRPGPMDNIPTYIA
CKHGEQEVDYLHPLLESTLKDTYGVIIYQEQVMEIARKLAGYSAGTADLLRRAMGKKVKA
EMDAQEEIFVKGALANKVSESDAKSIFATVAKFAGYGFNKAHAAAYGVISYQTAYLKANF
PVEFLIAALNLDIDSSDKISIFLQEAKNANINVIAPDINESSGLFEIKNDNDNAKKSITF
ALGAIKSVTVNFGNAASAVRMEKGNFKSIIDFAERIDPKLLNKRLLESTIKAGCFDKLHP
NRNSLLQSVPKIMAYSAAYHLEQKSHQISLISVGSNGNDVLVDAEPLSASEIAYAEFDVM
GLFLKNHPLSGLASSLKKCSIKDTDYIKNELPQGSHKLKLAGIIVKKDARMSKRGLFVTL
IISDPKGIFEVTIYNEDVFKNYAHLINVKEPVIIYCDILKDKGGLRTTALRFSSINDELQ
GIQYNLTLSPKKPQDVEKIMELLNSRISQDKSNSSISVLLPVEEEFVAKITMPPCFRLNE
EDAMKLSEF

>gene_103|GeneMark.hmm|300_aa|-|12830|13732 >NVVL01000002.1 Rickettsiales bacterium isolate NORP64 Contig_source1382A_922, whole genome shotgun sequence
MDIINPRVDLAFKKIFGVEENKDLLISLINSIVGKEDQVADVTLLNPYNPQNFRQDKLSI
LDIKARGIDGKRFNIEIQISDEADYDKRALFYWAKLYTEQLQVAEDYSELSKAIGIHILN
FTSIPSSERYHNTFHITEKTSGELYFKDLELHTIELKKFTPDSKEDLSHIVAKVKNSLDM
WIAFLTRHDLLKADNLPPELNNKELKKALNVLNVMNFTEEERGAYEDHLKWLRVEANSLK
KAEAKGHEKGLEKGLDEGRKLEKIEMAKTMLVQGLDIKLISKISKLSIEEIEDLRSYEVT

>gene_104|GeneMark.hmm|52_aa|-|13810|13968 >NVVL01000002.1 Rickettsiales bacterium isolate NORP64 Contig_source1382A_922, whole genome shotgun sequence
VILKTLLAGLRSSAGGAIIKGLCKSAGWDEDTYLDYINSVFLMRLPWVALLL

>gene_105|GeneMark.hmm|483_aa|-|13965|15416 >NVVL01000002.1 Rickettsiales bacterium isolate NORP64 Contig_source1382A_922, whole genome shotgun sequence
MSVPPVRSIPLFIGRENELERLGALHKKRAPGLVVIKGRRRVGKSRLINFFASGRVKNKL
WDFAGLAPQDGMDDQTQRDHFARQLAAHLKLPPFTFQDWSDAFDHLSNHIKAGDIILLDE
ISWMGAKDPSFISKLKAWWDKQQLPIMVVFCGSVSSWIEENILKSTAFFGRINLTITLAP
LPIDEANKLLRASGFKGSDYDAYKLLSILGGIPWYLEQITPGQSADDLIKQLCFEKDSLL
VLEFDRIFHDLFNGKGVSYKKILDALKDGMKTLADIRTIIEFPHSGTLSSLMEHLIIAGF
VQKQNLWSFKTTKPLKQSLYRICDPYMRFYLKLIEPQRNKIDLGAFQNTAIAQLPGFEAH
IGFQLEQLLLQNRSLLLKAIGINPADIVADGPFRQSKTASKTGCQIDYLIQTTTKNLFIC
EFKFKRRELGLDIITQLQEKANALKVPRGFATIPVLFHIGGVSSNVATDDYFYRIIDIAD
FLQ

>gene_106|GeneMark.hmm|340_aa|-|15558|16580 >NVVL01000002.1 Rickettsiales bacterium isolate NORP64 Contig_source1382A_922, whole genome shotgun sequence

MISLNKDQIKKLLDIIAFSDASSCLSRASDIVINNTDIGFSLDITGITHAEAELIRQKAI
KELKNINNIGNINIVLTSSAPIKQGAKLGTKPGKSPSREKPKLHIDGVDQVIMVASGKGG
VGKSTISALLGSKLAADGMRVGIIDADIYGPSMPNIFNLSGKPELDGNKMIPLKNHGISI
NSIGFLTAPSASISWRGPMASKALYQLLSLTKWGQLDYLIIDTPPGTGDIHLSLLQNYII
DKVFMITTPQKISEIDVSRAISLYQKFNIPISGIIENMSYYHEPISGKKIKLFSGSAGEE
ISKKHNIPMLAQLPISPGLSTACDKGESLQPYAWLLDGVV

>gene_107|GeneMark.hmm|382_aa|+|16564|17712 >NVVL01000002.1 Rickettsiales bacterium isolate NORP64 Contig_source1382A_922, whole genome shotgun sequence
LFKLIILAIFYKLTILVYEFSLFFTNSNLVLIIMINKIYGQFFSKSPWDDNGQEDNIFTQ
KRKPQFNFDNFQFDFSPKLILIGLAALIAVWLASGIYEVKEGEQAIVIRFGKYHRSGEPG
LNYHFPNPIEKIIVAEVDKSNRLEIGYRSTGRARVGGAVSARDIASESLMLTGDENIMEL
NIDVMWHIKDLSHYVFNISDPEDTVRAAAQSAIREVIASIDITSVLSNKKQAIADRMEVL
IQEILDQYESGVTIEQVKLLRAEPPKEVIAAYRDVQTAKADKENAINKAEAYQNDILPRA
RGESAKIVQAAEGYKVEVVSKAKGDASRFNAVYKQYVLGKSVTKSRLYLETIEDILQDAS
ITIVGSGGVLPHMAINKDNLIK

>gene_108|GeneMark.hmm|287_aa|+|17722|18585 >NVVL01000002.1 Rickettsiales bacterium isolate NORP64 Contig_source1382A_922, whole genome shotgun sequence
MNMNKLYITIFAAFAALVLLAGSLFTVDQRQTAVVFQFGEAVRVIEEPGLNIKIPLVQNV
QYLDRRILNVVAEEKELTASDEKRIIVDAFAKYKIIDTVKFIKTVHTYAGANLRLNKILE
SSMRTVIGRFPLVTLLTEQRSDLMLEIRDLVHRETEPFGIQVIDVRILKSDLPHENSEAI
YNRMQTERYKEAKRIRAEGAEESARIKSKADKESQIILATAYMEAQKAKGQGDYEAARLY
NLAYSKDPEFYKFYRSLVAYKASLSKDSTQFILSPKSAFFKYLQIGK

>gene_109|GeneMark.hmm|491_aa|+|18589|20064 >NVVL01000002.1 Rickettsiales bacterium isolate NORP64 Contig_source1382A_922, whole genome shotgun sequence
MRYLRALFLILITTLHFGAANGGALATAPVTTPAPALSPAHITSFADIVEPLMPTVVNVY
TVRYNEQLKNRKAGLPEIFPFEKFNSFFEKYNVPFSFDDLYSRPEAMSLGSGFIIDEEGY
IITNDHVVAGADEIYVKLTDNIEIPAKIVGTDAKTDLALLKIETKKKLPFAVFADSNRTR
IGDVVIAIGNPLGFGGTVTTGIISSKGRDLGEDLVDDFIQTDAAINAGNSGGPLYNIEGK
VIGMNTSIADGGGGTNIGIGFAIPANTVQDIMKQLREKGKIRRGRLNVNIQEVTTELAEA
LSLKDENGVLIVDVNIGGVGDKAGLQRGDVITKFNDDIVLDSRKLKIFVADTNIGEEIKL
TVIRGQNPVILTAKMVEFKKPSAAEIDSNVSTMQESGVTFSNLSPKLITKFGLPPESKGV
LVASMQAQEASNELKIGDLVMSIDQESINNIEQFKQIYDKMGKTGKKNVVLLVKRIIRRK
ESTMFAAFPIK

>gene_110|GeneMark.hmm|279_aa|+|20086|20925 >NVVL01000002.1 Rickettsiales bacterium isolate NORP64 Contig_source1382A_922, whole genome shotgun sequence
MTEEINAMEEANEVSSRDEISGRDDIAVLSFYSFVNMESPEIMQQKILLLAKKKCIKGTV
LVAHEGFNGSISGEEEKLHWLIAEIAKITGADDVSSKVNYCAEQPFSKMKVKIKKEIVAL
KFGDLDVENLKGDYIESADWSEFIKRSDVVTVDTRNDYEVKVGTFKGAIDPKTEVFSQMP
QWTKDNMGLLKDKKVAMFCTGGIRCEKSTALLKDLGVSEVYHLKGGILQYLEDTKNESGA
WQGECFVFDDRGAVSANLSPSDGFWVGEGQTAKGVSSSK

>gene_111|GeneMark.hmm|204_aa|+|20922|21536 >NVVL01000002.1 Rickettsiales bacterium isolate NORP64 Contig_source1382A_922, whole genome shotgun sequence

MILQREKICAIISDNSIISSAICENTTCQIDVVSSDYESFIDSKNKLDLLILDKDIYPHK
LPLHRINCLINLTDQKFSDSEIRIAKPLKLYDLLGIIASNMREEYFFCCINHEWIYHERR
ARFISASREISLTDKENALVARLLSDKNFSAKKDSLKTSLWNYHQDSESTTVDTHLYKLK
QKLPAGMVEIRQSECLFKFEKVVF

>gene_112|GeneMark.hmm|58_aa|-|21736|21912 >NVVL01000002.1 Rickettsiales bacterium isolate NORP64 Contig_source1382A_922, whole genome shotgun sequence
VEYTDPKVPEEKKKELAQVMQDHMSKEDRVKFMKTVKDSYQEEAVTKRNTQIASQQIC

>gene_113|GeneMark.hmm|244_aa|-|22174|22908 >NVVL01000002.1 Rickettsiales bacterium isolate NORP64 Contig_source1382A_922, whole genome shotgun sequence
MIDYTKSFSAKESKGFDAGLRSYMLKIYNYMTLALLLTGAMAFGTLNFPPLMNIMYNIGA
NGEFMGVSTAGMVITFSPILIALYFFMGMGKMSVQSSRALFWVYAALMGMSLSHLGLVYT
GQSLTRTFFICSAVFGSVSIYGYTTKKDLTSMGSFLMMGLIGLIVVSVINIFLQSPAIEF
ATSFIGVAVFIGLTAWDTQKLKAMYYASGGGEMGQKMAIRGAFTLYLDFINLFLYLLRFF
GDRR

>gene_114|GeneMark.hmm|288_aa|-|22963|23829 >NVVL01000002.1 Rickettsiales bacterium isolate NORP64 Contig_source1382A_922, whole genome shotgun sequence
MPNKTSQNKLIALFRLTRFHLPTGYLLAFFPAAFGLLLAYEETPNLNYLPLFFVGSILAR
SAGCVINDILDKDIDRLVARTQDRPIASGIITVTQAVIFLVLLFTGCLAILLSLSITSIA
LGIIAFLMILLYPIMKRITYFPQAFLGVTFNMGCLIGYAAVKDEVSYESMLLYIACGFWT
FGYDTIYAFMDIKDDKKIGVWSSAIFFEDKPYKLIIAGFYVAFFVLFSIATQDSFGMYSL
CTISFAVMATLWGIASLDVKDPKNCLARFKANNYLGFLLFFVMLLENL

>gene_115|GeneMark.hmm|841_aa|+|23891|26416 >NVVL01000002.1 Rickettsiales bacterium isolate NORP64 Contig_source1382A_922, whole genome shotgun sequence
MTDFPKKYKFNDHEKKWQAFWQEQGIYLWDKSEARDNTFVVDTPPPTVSGQLHIGHVYSY
THTDFIVRYKRMKGLNIFYPMGFDDNGLPTERLVEKKKKVKASQMKREEFVAICEDVVES
EEEKFRDLFNSIALSVDWNIEFRTISKTSCKISQMSFLDLMNKEEIYRDSQPMLWDPVDQ
TALAQADIEDQEKTTFMNDIQFTTEDGDKITIATTRPELLAACVSVFFHPDDARYNKLAG
KMAISPLFGVRVPLLADDMVDPEKGTGLVMCCTFGDQTDILWWRKHKLPTKIIFTKWGTI
DGITFDDTCINKQQAEKFGAEITGLKIKAAREKMIDILKAEGLLLAQTERTQTVKCAERS
GAPLEILTTPQWFIKSVAHKEALAKRSDELNWYPQSMKVKLDKWIEGVALDWCISRQRYF
GVPFPVWYSKRPGEEGKPIFAKLEQLPIDPALDLPEGYLRDEVEADKDVMDTWSTSSVSP
QLSSHGIADGFMVDQKRHENLFPADLRPQAHEILRTWGYYTMLKSHLHEGTLPWKNIMIS
GWCLAEDKSKMSKSKGNIIAPETLLEQHGSDVIRYWAASSRLGADTAYSEDVMKNGKRLV
NKLWNAAKFVSQHFDKIPDSDKNLELGALKGKISCDFDKYFLLKLSELVQEASADFENYE
YALAMAKTEQFFWSLFCDNYLEITKSRAYDEESENPEGALSARLALYHGLKVLLKLFAPF
VPHITEELHQLLYSKESIHTRHSWPKLDINFDEFAPHESESLIEVLDLVRKIKAKDNLSI
KAPIEYIEIEGAKLSDELVSDLKNVTSSAKIKFVDRLSSNAQELGSDKLKINVKYDESGA
A

>gene_116|GeneMark.hmm|61_aa|-|26900|27085 >NVVL01000002.1 Rickettsiales bacterium isolate NORP64 Contig_source1382A_922, whole genome shotgun sequence
LFIKLAKTPGAQSVIVLIPMPAQMPTMMMISKNAEITTISGLKFISASAFKVKPKILKIR
S

>gene_117|GeneMark.hmm|926_aa|-|27076|29856 >NVVL01000002.1 Rickettsiales bacterium isolate NORP64 Contig_source1382A_922, whole genome shotgun sequence
MEVKMTKQMDVSKDRTDNKRPVEVLGNVEQPPSKKIKRDIDEMIKLYKTAFAFYRDKEPK
KVYEILEKMHIRDLYQLNPVMTTEMYSVLAIKVSNYETKNHCLAKIKVGLSTIDPRALDA
KNMLKVALLLERCIQQLSTHFNDSDNILRTTQLLDEYSALMRNLEPGALNDQPTLVIAQY
GTLAQCYALLNARESVLLVCEQLFNLCREAMVKISVVPESAWLLRSAVYLVRALSTCESS
GRKKLVDDNIETVIQILSFVSAFDRIDEKTALMSLEIINKLMIICSKTNAIISSTYSSNF
LEREFAVIHFAEALTSIIHQLADKANTSRVLTKTSDVMHAMFGHVMFLEVDHGVIAQYVN
CWQQSLQKGTLDNPGSILDQVSSAINTALRSNQTVSIEPLLDLAWLLIQTTASNKITAGE
TLYTLYSYLDMYLQAGQTEIFLNHFTAMLQMHIQSVQTSKDYYGLNAQFETFMSLCHLQN
DRNMLLPLLQQLDEAFVEKLTNNPTQRLSHYFITSAIQYWNLGQHELAFGHLSRAMDVIE
ITNARNEKVFCIFFLEFCRLYSIQSGDHMFSDKFNKYHEIYTKLPARERQADLRTQVNDI
LAIINADTVDASSPLLSMRYEDNGSIIIDRALIETVSSFIKFKHNKGISFNHAPDHSRYA
TLQDISANDREEIQRIAREMHRQIDDTLCVLNQGSSEQWQEFAQCARWICPQLWVALRVN
DANQLEWGRASWRIQVIFEQLITYSMMNHDITGLKKISDILFTRLTLKNQWKNNSLLECL
EHTIDAFIFCQEGMEAARCIQTIVQTPQLADERIDKLTIKLLALMPLSRPDEEDVQLLLL
PQAVREYEILNNFLDRSDVLNCVEDRQVNNALVTQYGHISHMLYQSLLSILLHDPTLPSE
ISYILADYTYDSDGYNLAGALPSVCL

>gene_118|GeneMark.hmm|287_aa|-|30539|31402 >NVVL01000002.1 Rickettsiales bacterium isolate NORP64 Contig_source1382A_922, whole genome shotgun sequence
MNNLQAKLDQLKWLKSSGVEYFCSTEKDSKNSLISELISNSSDKVEINEPKPVKSTNSVQ
STKPLKSVIQNIMKETKTTNTPNSNILAARALADKAENLAQLKEYVENFEGCALKKFAAN
TVFADGVQDAKILLIGEAPGATEDEQGIPFCGESGKLLDKMLASIGICRTKNAYITNTIF
WRPPANRRPTTEEIDICKPFVEKHIALMKPKLIILVGGTATASLLGKSEGISHIRKNSYS
YTNQYISDPIHTTALFHPAYLLRQPMQKKTTWYDLLKIKDLLKKLGV

>gene_119|GeneMark.hmm|322_aa|+|31424|32392 >NVVL01000002.1 Rickettsiales bacterium isolate NORP64 Contig_source1382A_922, whole genome shotgun sequence
MSKYTYTIPENLNGERVDKALSTLCQETSRSQIQKAIKNEKLTLNDQIISNLSARVKEND
QVEIIIIEEITSELLPANIPLDIVYEDDDMLVINKSSNMTVHPGAGAHADTLVNALLYHT
NHLSDVGGEVRPGIVHRLDKTTSGLMVVAKNNQAHVHLAAQIESRELTRMYKALVWGLIK
PTSGTIDKPIGRSRADRKKMSTFKTGGKHAVTHYVTKKILHGGLFSFVECKLETGRTHQI
RVHLSHMGHSIVGDQTYGNNSRKISGCPESLRPALLAMDHQALHSFYISFIHPKSGDRME
FTKEIPLDYNKLIESILNSQST

>gene_120|GeneMark.hmm|247_aa|+|32405|33148 >NVVL01000002.1 Rickettsiales bacterium isolate NORP64 Contig_source1382A_922, whole genome shotgun sequence
MHKCKIFLVVIFSLILSSCSEERDENVWVVGTSADNPPYEYMHDGRITGFDIDLMKQIAG
HLGKDIKFKNMDFHGLIAALTTKNLDMIISGLSITPERLRRVDFSVPYTDAKIAVLYYTR
DRFQKTSDLTGKAIGAQLGTVWSLIAHDLSSQHRFSIKSLANNLLLVEELKNKQLDAMIL
EESQAARFISIYPQFSSFNLEQYSSAFAVALQKKSPHTKNINHAIKALRSNGTIEALARK
WGIIGAE

>gene_121|GeneMark.hmm|157_aa|+|33135|33608 >NVVL01000002.1 Rickettsiales bacterium isolate NORP64 Contig_source1382A_922, whole genome shotgun sequence

LVQNEEFKLAVGKFPTGVTVITTNQGSKLYGFTANSFASVSLEPHLISFCLNKEAYSFDA
FSDAEYFSISILSSAQADISGHFARHSADKFKGVDYELGKHSNSPLIQGAICFIECKKYN
IFECGDHSIFIGEVIKTQIDEDKSPLLYFSKSYLEIK

>gene_122|GeneMark.hmm|247_aa|+|33605|34348 >NVVL01000002.1 Rickettsiales bacterium isolate NORP64 Contig_source1382A_922, whole genome shotgun sequence
MSIIKIGLSGANGRMGRAIQNLLLDKTDKFELTAKITSSSSELDLSKACLDSDIIIDFSS
SAILPSLVKAAAQHGAKLVVGTTGHAQSHFADLKDLAKTNAILYASNTSIGANLIAMLAA
KSAKILKGYDVEIIEAHHKHKKDAPSGTALMIGQKIADAMNINFKDNAVFDRASKGKRID
GEIGFASIRGGGIFGENEIMFAADDELVSISCRALSRDAFASGALIAAEWLSAQKAGFYS
MEDVLEF

>gene_123|GeneMark.hmm|1027_aa|-|34821|37904 >NVVL01000002.1 Rickettsiales bacterium isolate NORP64 Contig_source1382A_922, whole genome shotgun sequence
MKRALIILLLIFQSQSAYASFWDSIGRCLKDPCNCGEKPITEYWGKQVIRPSVKKNKNCP
PWDKYDGRSRYNCLMKKAMPGTFIESYKNLCGEKSPSSSYKTPRIRVRGQQCNALKCWST
STSLDWDGDCVTLAGRYGLPLYRMCARIALPANTQTTPSIKADPGYTDGKHLDSKGKTQD
DKYPVGKDGAPIKLSRPKLCLYLDPAFLSLESGLQADVMDIDPNWQPFHKTEAVHPIIQI
IKTFFKVFKDSASGLASAISDLTASLGSSSDIATQSLSKAFEFLGTLIGIFGNAIEKLLD
EVGAINRAVSSTKYGCVELPLGPMPPPFCSQISYFQLATLQKICQLDKDGKYEKSTNSSK
CLVSAVRNNFVRNSVRVGYDHLVPLCKPGESPSHGTCAEINNLASTLTNAKALHEITKKR
DIIKHCSAASSGELCAQTSIKHRCSVTEHGCRDGFRIVYAKKIGHNITPEPYFYDDLPNC
TNNSPSACQVIWGINTAEFADISLVFPKVSNSANIVSAIKHSFRLFDYGLDTDGSTPPKE
NKFHATIVQQSHENQDYNFVQEPNQICLFGNKRMIDCTERVPAPQPLLSCNNVFGTNCKS
SYFSPQATITYRSDDGRERSKQDVTSTLVVPETVYQSGGNRKAVAYLAGTQFEALVTDDN
YAMNPFMNAPPLKDPTSILGTYLDNILPISLNPPYARNPYAVYMSGLEYISNKYHLGGKY
ICVRNKNMRQCPDDIHMCVLAKLANKNTVKCSIFYKSTENHPGLSMCSPDQARNPSCGVI
DFMPKISGGRVNIKKCADGALCYESDAELCTVGMSAAERSIPGAQFGETLATNQHYNPFI
KDARGIVRIRKYNQNSLRLRTKTLRELGACTLISPDPRQDTCSAQNNFSQDNGFASWTSV
KVGEISTGTCKAGMKLFNPANPLKRLCIPVSEPAPPRFALEPLYRMKGTPPKKEYTNTKC
VFDGCKVEGGFNAYWPAVKSGELSLGRCNLGYYRVSTPQRRCVQKSPGVFVLSFPDRRKG
YCDRLGK

>gene_124|GeneMark.hmm|818_aa|-|37935|40391 >NVVL01000002.1 Rickettsiales bacterium isolate NORP64 Contig_source1382A_922, whole genome shotgun sequence
MSSKASDNSLYNDSAADFVPVACHYDAHTLLTKNGELLQTIQIHGLLSEKISKNLFNLRE
VVRNSISSTIECSKYAFWIHTVREKADLDDPAEYPNFFSANVHDIWRRKNYWHDKFVNKL
YITIVHKGTELKLKNLNSFINSFFQRRISSSEEESLEQACGELHKTVDSILSGLSEYGAE
KLGTLIEGEECYSQPISFYKHIMHLSNKDSTMPTQDISSVLASHQYVAQSDRMEVIGGGE
QKQVAIISIKEYQEISSDALDKFMQTPVEMIATEIFYFVKRSDVVADFTVQNDTLNISGD
SELRKWKGLDKIVDAEDNGIRFCHQQISFMVIGEDKETLDRDAIRASESLAQIGIVHIRE
DINLEKLFWSQLPANFSFLSRMKPTILDNTAALASLHNFPMGNQYNPWGRATTLLRTEKG
TPYFMNFHDQTGKGSTCIFGNRKSGRTTLLNFLLSEMDKYRPSIMYITDDMDSGLYIKAK
EGRWFQREKNIINPLICEDTPENRVFMLEFFKIISKHYFDPLTNAEMLLLTTLSETVFTL
PGQERSLAELLKSLKESDKGGKTLKKRLAAYAENGIYHGVFEREEAINIEEGEIVAMNLQ
NFDDAAYSKAHYPKEKRLIEQFEYDLNAMRAVKAGIVLAAQNIMKVIGDKPKIFVMDNLA
EIINLKHYSFLMSTLMNNMHAMNGIFVSTVNTDKLYSLYGTKLTQNANSKTSQDWIRELN

TSFFLPSELSVAGLEEILDLDSAELRKLARLTPASRMFLIRQDGTTITSELSIGGLIGLT
SLLCSGEQERELYREIIKNEGNDTTEKWVQALCEACEN

>gene_125|GeneMark.hmm|284_aa|-|40655|41509 >NVVL01000002.1 Rickettsiales bacterium isolate NORP64 Contig_source1382A_922, whole genome shotgun sequence
MSKNQTIIIKKVKKIQGGGHHGGAWKVAYADFVTAMMAFFLLLWLVSISDKATLQGVAQY
FTPTESISDKAGLGFGGGTDNNLTEGQLSEYAAASSLIYGSPSKGHRVDAAKAPTSMSDV
EKEHFISIMQSIQKSTELQKYSENIHIDLTNEGLRIQIMDSDNRPMFKPDTTELQPYMLK
IIDIIGKMVSTQPNYISITGHTASVPEDAEDDIDFWYISSERANKIRQYLVENLINKNQV
VKLVGKSDREPFDPKDPFSVRNIRVGITLLNNSSVSAYQKSAPR

>gene_126|GeneMark.hmm|289_aa|-|41519|42388 >NVVL01000002.1 Rickettsiales bacterium isolate NORP64 Contig_source1382A_922, whole genome shotgun sequence
MFYIIGVVIVFGSVLGGYTMHGGNLAVLNQPNEVVIIVGSAIGSVVIGFPMSYLVQSMKG
MKHFFTAMPYNKKDYMELLLFSFNTFKLMKIKGMLEIESHIESPEESELFKLAPVLKKDH
FILDFMADNLRLMTMGMDNPYQFEDVMDREIDLYKEHTSVPGDLFTNLGDAFPALGIVAA
VLGVITTMGSIMEPPDVLGALIGAALVGTFLGILVAYGFASPMGHFLHKYGAYQVKFADC
AKVGFVSYLNGNPPIIVIEFMRKAIPEHYRPSFEELDVYINENAMKMMG

>gene_127|GeneMark.hmm|158_aa|-|42429|42905 >NVVL01000002.1 Rickettsiales bacterium isolate NORP64 Contig_source1382A_922, whole genome shotgun sequence
LSESNFNNKFMRAALIEAEKAYAAGEIPVGCVIVCKDTGQIIARAHNIMQKNNNPNMHAE
IIAINHACGQLGNKSLSNCDLYVTLEPCTMCASAISNARLACLYYGASDPKQGAVEHGVR
FYTSNSCFHRPEIYYGLYCEESEELMKGFFSQLRKSKI

>gene_128|GeneMark.hmm|299_aa|-|42892|43791 >NVVL01000002.1 Rickettsiales bacterium isolate NORP64 Contig_source1382A_922, whole genome shotgun sequence
MFKLDENDLIRSASYASVVIAIIIMVVKMYGWLATDSQSILASLVDSFLDISSSLINLIA
IRVSLMPADDTYRFGYDKFQDLAIFSQSIFFFASSLFTMFSSGKALYIGAVPANVALGAN
VMYICIFLTFILVLYQSYVIRHTNSRIIAADKLHYFSDFLTNIAIVISLYLSATFWYLDA
LAGIGVSFYIMFVSYQLFRDAIHNLADEEFETEDRDRIIKIISSFSETKGIHELKTRSAG
SKPFIQFHLELDGSLSLLEAHYISDRISDALIKEFPKAEITIHQDPEGFEHEVNYVERI

>gene_129|GeneMark.hmm|155_aa|+|43894|44361 >NVVL01000002.1 Rickettsiales bacterium isolate NORP64 Contig_source1382A_922, whole genome shotgun sequence
MKSISKLFLLIFTIVSLQSCQNMNKQGGGTLIGGAAGALLGAQFGKGSGKLVAVGIGALA
GALVGGQVGKSMDEYDKKLLENSSRQALEFSPSGRPVEWKNPDSGNRGSITPTKTFRERG
RYCREYTQEVIVGGEKQKAYGKACRQPDGNWQIIK

>gene_130|GeneMark.hmm|232_aa|+|44567|45265 >NVVL01000002.1 Rickettsiales bacterium isolate NORP64 Contig_source1382A_922, whole genome shotgun sequence
MNTLVEFKSLSKSFDKKILIDNVSFKLNKGEITTLIGQNGAGKTTLAKIILGLEGYENGE
VIIQKGLKIGYVPQKLDFGFNMPITSSGLLEILAPKYSKSDFSKFAEFINLDSIRKKDIS
EISGGQLQKLIIAATLLSRPDIIILDEPTQFLDVASQQEFYKIIARLKVDLGLTVFMISH
DLFTVMKNSDQVICLNKHVCCSGKPAEIDSNSDFKNALSEIGVYIHNHDHKH

>gene_131|GeneMark.hmm|296_aa|+|45272|46162 >NVVL01000002.1 Rickettsiales bacterium isolate NORP64 Contig_source1382A_922, whole genome shotgun sequence
MLENTIEYQINFWQAIAAHYIFPEGGFAFTTKMSGADTNLLILTKQDLQTLKSILPEVTS
FFEKHQVPWGINIVDNPHSAEIISFLEEKHFSKIYTQHQMETKLASLPQTPGSPLVREVG
DGDLEMLKDWTKPVVSGFKIQGSKDADLYYKLNEIALSREDNILKHFVLYDGAQPLSSAT
LSIHGDVARLDNVSTMVEAQRKGNGRALVEYCISKARALGCKYIIFESSERGINLYRKMG
FKEVAMSCVYSASKSSEFSANDPSACSASDSLVCSANESSESSASNPLPRNGSKSS

>gene_132|GeneMark.hmm|255_aa|+|46159|46926 >NVVL01000002.1 Rickettsiales bacterium isolate NORP64 Contig_source1382A_922, whole genome shotgun sequence
MIIILAISVLIGLIFAPLGCLVLWKRYVYFGDGLAHASMLAAALSLLAGIPIFYAGILNT
LLFALLIFKLKHKSGNNAAIGLTSSIMLSLALILSYIFPGEFNFSSLLFGDIVAATGDDV
LILCALLASVLVFLALTYRDLILIILSKDVARSKNIKVQALELVFLSILSFAVLSTIKIV
GALLVTSLILIPAMTARIVSTSPMMMIGFAIFFAQLMNFAGIMLSFYADIPFAPIIILCG
GGIYVMVLLWKNCRT

>gene_133|GeneMark.hmm|203_aa|-|46938|47549 >NVVL01000002.1 Rickettsiales bacterium isolate NORP64 Contig_source1382A_922, whole genome shotgun sequence
MSDFLTFLTGVGIGAGLIIAIGAQNAFVLKKGITKSHIFIVAITCSVIDAVLIIFGICGL
GDFISENEQLLQITKYGGAVFLFYYGARSFYSSIYLTHSLADEAQSKAGDLKKTILTLLA
LSLLNPHVYLDTIVLLGSIGAQMEEGERLYFALGAVCASFMWFFSLAYGAGHLSRFFKSS
ASWKILDACIGVIMWSIAVSLFV

>gene_134|GeneMark.hmm|446_aa|-|47652|48992 >NVVL01000002.1 Rickettsiales bacterium isolate NORP64 Contig_source1382A_922, whole genome shotgun sequence
MKNFALIILLPLFLVSCDNFGTTRVKNLIDLTPRLEVESAQKIKFSNARELLLDEKKAKS
FKLAKREIIARPAIAKGVMYSVDGKGYVSAFSLKEQKILWSSDIAKGELDRSFSAGGVLF
SDDKLYVTNSSRFLVILDAKTGHEIIRKEFPDILRAKPIMATDRLLLVQTISNQLLAYDI
VSSKLVWLHEGGIETISTRSQAAPKVYNGHAIVSYSSGEVVYLDVTNGKEKWVYNLSTID
DIGFPSFDPSVIVTEPIIHKNYVYFATSNAKLVKLDLSNGAPAWIRDADDIQSMSLINNT
LFVTNNARQAAALSSHNGKVLWVGNMISEKDRSGKRPKTAFFQAPFVSKTPNGFAVNIIA
SNGELYQFATGLPGTGLPGADLSGIDKSGTDKSGTDKSGTGKPALSVPGQLPVQPKIIKI
VKGVMYHWISCCNGKMYLMTNRKVQY

>gene_135|GeneMark.hmm|216_aa|-|49077|49727 >NVVL01000002.1 Rickettsiales bacterium isolate NORP64 Contig_source1382A_922, whole genome shotgun sequence
VADLLDEVLHDARYEKNLLLFRKFLPFVIAVSVIIAISIASYGWYQNKISKHNREIGDVF
VSIMSTKHDKKSSMVDSLHELVASGDNKQIELAELKIAGELINSKNKKPALEKLEKIISN
KGYYEVTTSFARLLWVGVVLDQKKVSDANKIKAIDYLQYFSKDTQVFFASATLMKALFYK
KNAQNDLAIEYANKLLQLKDASLVIREQARAILANI

>gene_136|GeneMark.hmm|234_aa|-|49775|50479 >NVVL01000002.1 Rickettsiales bacterium isolate NORP64 Contig_source1382A_922, whole genome shotgun sequence
MIFNRKMTRKILLILCIAFSAGGAIADSKSYGPIIHRQSKQIQELKDRISGLENLIKDLQ
EDLIQSGIVTKQFSANLEGAGSKDFIQPATLPARKNFFTDKENSSQPKDRSDYDYALAAL
KDSKYLDAEGQFASFIRNYPTHRLQSNATFWYAETFYRRGLFSKAAINYLRSYKKYPKGA
KAPDALLKLAYSLASLNKGKEACSMLKKLDLEFPKRPIGSIKRTMEAKTKFLCN

>gene_137|GeneMark.hmm|484_aa|-|50533|51987 >NVVL01000002.1 Rickettsiales bacterium isolate NORP64 Contig_source1382A_922, whole genome shotgun sequence
VNIVANIILYRYFMIEEVLIKQAITQNLFVADSYKKHIWNHRKPAMAQVQQTSQGFLRNK
EFIEFASASLEFLRNTNADISIFNKFGTQFFSNHQQKMVKVEKNDHLFTHEKILLYLDEY
FLKEYTPDNAFAEAYMGRTVSSLIPRAILTEKNDIKYEKSFITTYVPIINDSNGNFSVDG
VMKITRDITNEWTNVIYLERRIFSAFLIVFLVFFAIVLYNTSRAQRIINKQIETNKALVE
EKAKAEMENSAKTDFLANISHELRTPLNAIIGFSEIILAQTYGKIANDQYEDYVGDINAS
GKHLLGVINDILDLSKASANKLTIEEAELDLSKLMLSSMRFVKPRADKANIQLVEQIPSQ
HIVIIADQKRLKQALLNLLSNAVKFTPAGGVVTLSVKKDVIKKLVYITVEDTGIGMNDKD
IPKALAPFGQVDNSTSRKYDGTGLGLPLTKKLVELMNGSFDLQSIPNEGTKITITFKYSG
IIEL

>gene_138|GeneMark.hmm|250_aa|-|52252|53004 >NVVL01000002.1 Rickettsiales bacterium isolate NORP64 Contig_source1382A_922, whole genome shotgun sequence
MKFVKSVKTIFFIAILSITSISFASENYASANEMTEENDYDANYYEDTTALLFKIRGFYM
RAPSKLESLPKPQAGTSAPSFIAQNGFGFDTSTTYFFNNNLAAEVSLGLARLQLKNSLVN
EFAKAFGTDPASSSKNNIFLVPLAITAQYHIAPFGAIRPYVGAGYHATYMHLRSKAMKVS
SGHGAVLQAGVDIAFQDDTFFTLDIKQYFLRSNLTFKKDFLSPGIASAASVKSRVKWDPL
VISLGFGFKL

>gene_139|GeneMark.hmm|292_aa|+|53254|54132 >NVVL01000002.1 Rickettsiales bacterium isolate NORP64 Contig_source1382A_922, whole genome shotgun sequence
METKLRSYFIGVSWFILSLVSSSINDVLAKYLGMRLHSYEITCFRFAFGTLTLLPFIMYY
GTSTLKTSRPTVHFFRGLLLFLGIAGWTYGLTLAPVTTATIVTLTMPVFVLVLGVFFLSE
NIIWQRWLVTLVAFCGLVVALNPNADDFNPEVLIFVFAALAFSSLDIVNKIFVVKESMIS
MLFYSAIITALLAVPFAYQYWITPTTEELALLFVLGASANLILFFLLKAFAVADATALAP
YRYIELLISATIAYIVFTEIPAESTVWGALIVIPSTLFIIYSEKQELSKKTN

>gene_140|GeneMark.hmm|425_aa|+|54197|55474 >NVVL01000002.1 Rickettsiales bacterium isolate NORP64 Contig_source1382A_922, whole genome shotgun sequence
MNKRKLILTSGVANSFEWYDYALIGNFAPILGKKFFPDADPSAALLQVFLVFAVGYLMRP
IGGVFFGIIGDKFGRKSALSTAIMCMAVPTAAIGFLPTYETLGISATILMVIFRMLQGLS
MGGALTGSIAFVIEHSPKEHRGALGSVAMSSICIGILLGSLVAFATQNLFTTEQFEDWAW
RVPFIIGIAVYFAGIYIKNNTKETPLFEEAKAHCEIAKTPLRTAFRYHWFDMLMSFFINA
TGSVIFYIEAIYLASYLKINRGFPESDVGILITCCYIIMIFVTLFAGWLSDKIGRRKIFI
LNLLFIIAVIPFLMEVMENGTFTAVIGAQVIIAIIAATYIGPEPALQAEFYPTSIRNTAL
SISYNTGTSVFGGTAPYIMESLVQNTNTITSSVYYIITCAFVSLAALYFYKDKSSYDRAK
AIDDT

>gene_141|GeneMark.hmm|1024_aa|+|55519|58593 >NVVL01000002.1 Rickettsiales bacterium isolate NORP64 Contig_source1382A_922, whole genome shotgun sequence
MTAKIFVTKLVDEKEVVKKLLPLSRFHKAEQRLVESHGKKLVEKIKSDRHFSIEEFLHRY
SLSDAEGVAVLSLAESLLRIPDNKVGMELISDKLSNKHWESYLFKRTKSFKTFLAAFGLY
FSGKFTDLVKSEGIISNLFERAGRRTFINIIKSSILFLSQEFVFAENMEEAIEKSSGRKY
AGNDFSFDLLGESSRTRAQATIYYREYKRAIKLISEAFPYNGEKLEERPNLSVKLTALHQ
RFELEKFDDLEKEFLPQIIDLVCEAKKNNLTITFDAEESSRIDVYLLFLTKLIRHPSFKD

YDGISFVVQSYQTRCYEIVEYILSLAKELGKKIPIRLVKGAYWDTEIKHAQELGLEFYPV
FTRKEYTDMAYIACARKILENDEFLYPQFATHNALTAASIIELAGDKEYEFQKLFGMGNA
LHSELANHRKVRIYAPVGNTKDLLAYLMRRMLENGASANFVSKVNSTEISMNELVHNVSD
NALHLLDLNNKITLPENIYPNRKNAMGYDLGYKTNYDYIQSKVASYFDNIYNVGSIIKGK
EIIHTKRGREKFCPAKKAEKFSSISSSTESEIKEAIDVAKSAFDGWSAVETRRRAQIIEN
IAYSLEENKFELYALLIKEGGKSIHDAINEVIEAIDFCRYYASQAQIIMQEKKLPGPTGE
SNVLSMHGRGVFLCVSPWNFPLAIFIGQIVAALAAGNSVVAKPSGNTPVVANFTVKLMHK
AGIPTSVLQLIITGGSTFNKYAISDERIAGVAFTGSCQVAKEINRMLANRESGIVPFIAE
TGGQNAMIVDSSALLEQVTDDVLTSAFYSAGQRCSALRMLYVQEEIYNDLLEMIKGATES
IKIGDTSDFSIDTGAVINEAAKQKLEEHVKNMRGKGFKIFEHPHLGKLEDGHYFYPHIIE
VKSINDIPDENFGAILHIAKYKSKHLDKVIDDINNYGFGLTFGIHSRIEEKIEYIRSKVK
MGNIYANRSITGAKVESQPFGGEGKSGTGFKAGGANYLLRFMTERTTTIDMTAFGGNVEL
LSQN

>gene_142|GeneMark.hmm|950_aa|+|58976|61828 >NVVL01000002.1 Rickettsiales bacterium isolate NORP64 Contig_source1382A_922, whole genome shotgun sequence
MFGIEVGVDMAIVVVFLLINLGVGLYYGQGVKNIKDYALGGRNFSTATLTATVIASWIGG
DNFSLYLSESYTEGLYYILSSFAALGSFLIIGYVYAPRMGEFLGDLSIAESMGKLYGKHA
RLMTSLVGFILIITMVSLQFKIASTLLSHLFGVSGVYTTIASGVIVILYSSLGGVKSVNF
TDIVQFFTFGTIIPVIALIVWSGVDDTETILHTIENNPLFDINLLLDYNTPRFWSFLTLM
LLFLIPGFGPSTFQRMSMSRDTQQMRKSFIYAAVAVMTVQLLISWISILILSKEPNLDSA
NLFAYILEEYSYTGLKGIFIVGVLTMSMSTADSHLNSGAVMFSYDIANTLNLIKKGQELL
VSRISTVVIGSIALFLAIKFDNLFDLFIFGYSFYMPVVTAPFTLAVLGFRTTSRSALAGM
ISGFLVALGWMFLDTEVDSVIPGLLANIVVMFLYHYLFKQPGGWTKKHSAKTTCSMKQCF
KNYLCKIKEFDVLAYCKQNLPKNESLYIVFAVFAIVSIFSTSYSIPTEIRNQYPSLIHGL
YFSTLVVAVSFLTFPMYLKPLCSKNVLSILWAFGLFYILVFVVGLLVIISNFGQLQLMMF
ILSIMVLSLLTRWQMALFLMVTGVISAIQFFKYYAGVENFTSDFGSLEFKVTYVMLLIGS
ILIAFFKPKQEHLEQTEHKVGTLETEATTKDGKITILNKEVGHYSERVSDQAKEIERLGA
TAQKILNNVNHELRLPVGNVMNFAEMLRDGLGKFSDEQLKMISEEVYTNSNRLSSMILNM
LDLATLNAKKIELKKEKVNFSKLVHERLQNCRDIYAQDKKALKFHLNVEDDLMVFADINY
IRQMIDNLVINAINFSEKGTIAISVDREKYFVSFAIRDEGKGIPKTEIYDIFTPFKMGSN
TESKAEGRGVGLALCKSVIEAHGGEISAESRPIGALFRVFLPYGEVKEGK

>gene_143|GeneMark.hmm|176_aa|+|62018|62548 >NVVL01000002.1 Rickettsiales bacterium isolate NORP64 Contig_source1382A_922, whole genome shotgun sequence
MTKLRVLTVPDPILRQKALPVRSVDDEILKLMDDMLEVMYDGGIGLAANQVGSLKRVLVV
DIRENSYDEEVENADLYPLCIANPEIIESSEEMEELEEGCLSLPDQRIMVIRPVSIKLRY
LDRDNKQQELFADGLLARVIQHEMDHLDGKLLVDYLSKMKKDIAMRKLTKLKKISA

>gene_144|GeneMark.hmm|313_aa|+|62586|63527 >NVVL01000002.1 Rickettsiales bacterium isolate NORP64 Contig_source1382A_922, whole genome shotgun sequence
MGTDEFAVPTLEYLANLKDPNDKYSYSRAHRISGVFTPPPKPKGRGMKLTKSPVHEAALA
HEIPVYNHTSLKTYAVAELIDFIDADVIIVAAYGLIVPKNILSAKKYGSLNIHPSSLPKY
RGAAPIQRTIMAGESISGISIIQMDEGVDTGDVILHEPVAFDARDNFKQTYDKCARISAV
LMAKVLSEIDTLPRVKQPEEGVSYAPKLTKEEGQINWNDDSFKIDCMVRGMNPWPGVYFH
HEGKMIKILSAIYNKDSHDYAPGQIINDDFEVACGSGTLRIESLKPEGKPEMSAKDYLRG
AVSMSKGTILSSK

>gene_145|GeneMark.hmm|340_aa|+|63528|64550 >NVVL01000002.1 Rickettsiales bacterium isolate NORP64 Contig_source1382A_922, whole genome shotgun sequence
MQKTLQISKTTMKKTALSGIQTSGHLHLGNYLGSIKNWLSMQDEYDCYFFLADLHSITVD
ISPEALRASTMNSAAIYLASGIDPKKSTIFKQSSVKEHSELAWMLNCITPIGWLKRMTQF
KDKAGKKQDSASTGLFTYPILMAADILLYNADVVPVGDDQKQHLELARDIAGALNRKFGQ
EVLKVPEPMIQGAATRVMSLRDGTKKMSKSDESEASRINLTDSADVIMKKLKKSKTDSIA
EITYNKADRPEVANLINIFSALTEQSKEAILGEYEGKGFAEFKTDLAEVIIAKLAPINAE
YQRIMQDPAYINQTLEDGAARARSVARRTCDAVAQGFGLV

>gene_146|GeneMark.hmm|292_aa|-|65068|65946 >NVVL01000002.1 Rickettsiales bacterium isolate NORP64 Contig_source1382A_922, whole genome shotgun sequence
MKNAAVPYDSIYRMNSNLSLLGNAFRPLNLDTILDSLDLSTKQYKELGQRVLQHSMIDSL
LEVYKILGYEDWEAARGLRLSSVPIGLRALEEIQHKDNAVTPEQHPMLCASILRKASNIA
GDCGKTKSSLDVDSPLIPRVDAGVNNIMNAYKDEVLQMMVSMSFSEELQQAMGNIFFKAF
GGEEVSGNNLVSFANPVALLKAQGCKVFAHQHEYDSWIVEQQVLEAAQQQALEAAQALEA
AQQALEAQQALEAAQQALVAPPEQEQEQGLLGFLSGVSGVSGVLAWWYGDDA

>gene_147|GeneMark.hmm|424_aa|+|66154|67428 >NVVL01000002.1 Rickettsiales bacterium isolate NORP64 Contig_source1382A_922, whole genome shotgun sequence
MALFINFIPILLFVGIFFGSGLYFSFMGVDNAFYQVPPTAAILPSIIVAWLMNRNLEKGA
MPSFLDGARHPDIIIMCIIFLLAGALSSVTQAIGSVQSTVNFALSIINPEYLLIGFFIVS
ALISTAIGTSLGTVAALSSLMVELATKGAFSVEMGAATLVGGAIFGDNLSVISDTTIASV
TSQGADLKEKLKLNVKLALPAAAVTIMIMLSIGGEVNITEAGGDYSLILILPYIVLITLA
VMGISVFAVLIIALALAGAIGIIYADYSLIAYSQDISAGFHDMHEIMLLAILVGGLSGLS
NHKAKAIVEALTKLLPKDAGRKSAELLIAFMVSVFDALLALNTVAIIFCGEFAKEISLKY
KIKPHESAVWLDVFSCVIQGIIPYGAQILLVCAIAEISPLVVIGKVYYCYVLAGVTLSFI
LLRK

>gene_148|GeneMark.hmm|392_aa|+|67602|68780 >NVVL01000002.1 Rickettsiales bacterium isolate NORP64 Contig_source1382A_922, whole genome shotgun sequence
MSKIRKITQRFSATFPNATSLIKPPLPLFERGPWEHLTHAGVPHGQFWSQDYVGLENTHS
KMHPLKWAFLRGDRKDARELLASGENPNIPIAQQTTPLHIACEIGDVEMVHAALAAGADV
NAQDAKLNTPLLIAFRYPNYHILKTLLEARADPFYKGEHYQTFLHAWAASASSLGAGEFS
IIMELIQNLTGTDLAAQYVNAVDLFGRTPMHGASMAGNTEAACQLAEIGGYIDITDRGGR
TPTHMAAYNLHLKTIKALAQLGATHNPLDTDGKHPIYAVLLRLMQDHSKEDLKQAIEIIR
MLAPGDDINAITAGKTYLQRATEMQNLLLVKALLELGADPSILSNDGSWSDYFFGESVTP
TETATKTGNIAILLALQEAQETGLSGDTDGDS

>gene_149|GeneMark.hmm|114_aa|+|68975|69319 >NVVL01000002.1 Rickettsiales bacterium isolate NORP64 Contig_source1382A_922, whole genome shotgun sequence
MARVTVEDCVEKIPNRFELVVLASRRTKDIAAGFPLTIDRDNDKNPVVSLREIAADKIDI
DLLREKQIANLQKNNQVDDSSKENLYAQTKESDEEGLDLEESDIFSDDVALDED

>gene_150|GeneMark.hmm|149_aa|-|69427|69876 >NVVL01000002.1 Rickettsiales bacterium isolate NORP64 Contig_source1382A_922, whole genome shotgun sequence
MKNRPNIRIKQLSFWNVWLWMAYLVIRCRVYVGEFKRPLFREYSSLDLGADHFVLLKGNK

VIGIARIIYKQEMVEFGRIAIRPSSRGQGYGKTFIDLLISEVKDRDKVRIISLFAADHRL
VKFYRHFDFLENGEVYFGMLPFMNMIKFL

>gene_151|GeneMark.hmm|992_aa|-|69873|72851	>NVVL01000002.1 Rickettsiales bacterium isolate NORP64 Contig_source1382A_922, whole genome shotgun sequence
MQNKPCNPRVMRLLLVFGILLTIKCRYMLFGVDLAIVISFLVITLLIGLSHRRGIKSIQD
YALGGRNFSTGALVSTTIATWVGGGFFFIVLSKSYSDGLYFLFAASGMVTSFLITAFVFI
PRMGEFLGKTSIAEAMGDLYGKNIRIVTAGAGIIGDIGALAIQFKAFGDIFSYFLGMPSY
ISTIGAGVVVTIYSAFGGIRAVTFTDVLQLSTFCIAIPLVGVMIWDQLNLEGFNIINIAI
SEKFNYKEVFSFSNANFLQAIPLMLYFAIPSLKPAICQRIVIGSNVAQVKKAFIISAAIL
IFMLFIMAWISFLLFSVNPNLKSDQLLGFILDNYTYLGLKQLILVSVMAMAMSTADSVIH
VSSTLFTNDICTPLKIGVKRELVFTKIFAGLLGVVAIILALVTQDLLSILLIASSFYMPV
VTAPLIFSILGFRSSTKSVAIGMGAGFITIVLWKIAGIKMNGIVIATVVNLIFLLGSHYL
LKQKGGWVGIKDRSYLDELKIEKEAIRAKISKRLREFSFTSYIKNISPISEFAYMRLGIY
FVIYTFTTMYSIDTGVVTNDIQIILAIYQIMMACGILFVTYPLWSHTLENKFPLQVSWFV
SVFTILIFFSSFFVMVGNFTPxxFxxFxxNxIxAxxxxxWxxAxxxIxTGxxxxxxxxKx
YMxIDYIDISIGSPQFVxMYILMLxGTVMIIFxKPQQEHQKQTEHKVGExSxEVGxLVTV
xSxRDxQISTLDTKVxGLEERVDHYTERTxNxxQExxRLGAxAQRIxxNxNxExRLPVGN
VxxFxEMLHDGLGKLSDEQLKMLSKEVYKNSNxxxxMIxNMxDLATxQAKKIExQIKLAN
FxELVEERxAxCxKxYVEEKPxxFIIKIEKNVMVxMDENYIRQxIDNLVINAINFSEKxK
ILVxVxxQTNFVTFTIKDQGIGIPLxELYDVFTPFKMGSxTxxKAEGRGxGLALCKxxVN
xHxGxVKxESSxAGVLFRVVLPLASDRGRPRS

>gene_152|GeneMark.hmm|50_aa|+|72846|72998	>NVVL01000002.1 Rickettsiales bacterium isolate NORP64 Contig_source1382A_922, whole genome shotgun sequence
LHKCEDASTNNDPGYSREPGLNGGFLRLKLWIITTQKAPALTMCSVYFYN

>gene_153|GeneMark.hmm|257_aa|-|73120|73893	>NVVL01000002.1 Rickettsiales bacterium isolate NORP64 Contig_source1382A_922, whole genome shotgun sequence
MSGHNYYNLLDENGVSHKYAIPINSLETVLGQGVTYRLQSDDLAYEAKRHLFDTEVADLV
VVYHEYQEVKNTDIYKTDTVCSGAVLDNERSIAWEFLINATIYNSSTAAYWAVHTMLSGN
GAERNLFLEKFLIALAKENASTPAEFALLAREAAPSTFFAKLAKTCADHTANITNLVGEI
NFTYGDVIALFQVYREVLKCNSDYDGSFDNFYPEHCGRASGMFINSIKFISLQTDSGDMD
IVDLIGVEETDCCCVIQ

>gene_154|GeneMark.hmm|368_aa|-|73910|75016	>NVVL01000002.1 Rickettsiales bacterium isolate NORP64 Contig_source1382A_922, whole genome shotgun sequence
MANNSEKTLKNIQHQLDTRLNDVQTDAVVAMIRSFQKDLANMNVQDLASLKSAISSFRAS
TLSPEVAGELELLLTSITEFSAKLEAEKMIEERIIQEELKHARNSGVSAEEVREYERNEK
LAGVIAVRVDDYFKYKEELFSEQRRELDFLNALSSGTETSAEDKCLYLGDCNSAGLRTKM
GLAERRQIDRQDQAMEMWRHLPSVNEHNQRKIDAVDASLQANISQPVREHLEQERKQLQT
TLERGQQALEGIDKLLAGESKILDKRLKMMKLGILPKKGGEQFLKNHYLVFEEESRVNPN
ATGVERCKQLAKAADIDLDALKSEAELLRSARRTGRQVRGSSENSKTANQSASVVSGLSI
KKVKPKSR

>gene_155|GeneMark.hmm|489_aa|-|466|1935 >NVVL01000003.1 Rickettsiales bacterium isolate NORP64 Contig_source1382A_1001, whole genome shotgun sequence
MLKKINNFLVKSISFLALLTIISCNDATANPSCANIKELEKLSNQGDATSQADLALCYYD
GEGIAQDFSKAAKWHRKAADQGDAIAQYNLGVQYANGEGVTKDFTQAVKWFKKAADQGDT
TAQYNLGVQYANGEGVTKDFTQAVKWYRKAADQGYADAQYNLGILYANGEGVTKDFTQAV
KWFKKAADQGYADAQYNLGILYVNGEGVTKDFTQVVKWYRKAADQGDAFAQYNLGVQYAK
GEGITKDFTKAVKWYRKAADQGHADAQNNLGVLYTNGEGVTKDFTQAVKWFRKTADQGHA
FAQYNLGVRYAKGEGVAKDFTQAAKWYRKAAEQGCALAQNNLGIQYVNGEGVTKDFTKAA
KWLKKAVDQGYALAQNNLGVRYVNGEGVTKDFTQAVKWFRKAAKQGYALAQNNLGVQYAK
GEGVAKDFTQALKWFRKAADQGNADAQNNLGVLYTAGKGVTKDFTKATKWYRKAADQGNA
TAQKNLKLK

>gene_156|GeneMark.hmm|890_aa|-|2092|4764 >NVVL01000003.1 Rickettsiales bacterium isolate NORP64 Contig_source1382A_1001, whole genome shotgun sequence
MRIEDFKEKYNYPAATPVMRQYLDIKFSNIECLLLFRMGDFYELFYEDAITASRTLGIAL
TKRGKSGERAIEMCGVPHHALENYLNKLLEEGFKVAICDQMETPEEAKKRGGYKAIVNRS
ITRIITQGTILEESLLDAKEPNYLVSIAIDKKGNAGISSVDLSTSEISVISVPEREIINE
LARIRPKEILLSEQYRSGELASVIGTQLDMRISFQVDSFFSAKKCEKNILDFYQISDLAA
IGQLTMEQISAVGSIIEYISLTQKANLPKLPKPKILNYNKFMSIDASTRRNLEINAVLSG
GVRGSLLSCIDNSVTKTGSRLLNSYLSAPLIEIDAINKRLSVTKFFYDDIPLTEIIRKSL
KQTGDLERCLTRLNMGRGTPKDLLSIKYTIEIAEQIQAEFVAKKGVTLPENIEKITQPLL
GMSSLHDLIAESIREDAPNIISNGGIINRNYHPKVAELHDLIENGQDAVEKLRDKYRQET
GIDTLKINNNNVIGLFIDITARHADKILGENFIHRQTTANSIRYTTKELQELESNMVNAK
LLVVSLEQEIYAKICMELVDNQQLLLALAGSISLLDVYCNFAIIAHENNYCAPELSDDMR
FEIKAGRHPVIENILKKSAESFMHNDSHLSMDERVWLITGPNMSGKSTYLRQNALITILA
QIGSYVPASSAKIGVVDKIFSRIGAGDDLNKGQSTFMLEMLETSAILAQATHKSLIILDE
VGRGTSTYDGVAIAWSVLEHIHDKLRARCMFATHYHELVAMEDILPALANYTVAIDDSDG
RILFLHKIKKGSADKSYGVHVAELAGLPKSVIRKATDLLKKFEKDSVKSSKELMRGESYN
MNLFDMASENAPPSKYQELAEELSTLNPDKLSPREALDILYKLKETANSA

>gene_157|GeneMark.hmm|227_aa|-|4816|5499 >NVVL01000003.1 Rickettsiales bacterium isolate NORP64 Contig_source1382A_1001, whole genome shotgun sequence
MTSSGYRGKFTRVKTAKRRKPSSTRWLKRQLNDPYVAKAQLDGYRSRAAYKLIEINEKFN
ILKAGANVVDLGAAPGGWSQVAAKIIRSDAASAKNKLIAIDLLPIGNIAGVISFEKDFFE
DGAKEMITDALDGRLADIVLSDMASNTTGHSPTDHLRIMDLCENALVFALTILKPGGHFI
AKIFRGGAEGDFLSMVKQNFKKVKHFKPDSSRKESSEFYLIALERKE

>gene_158|GeneMark.hmm|158_aa|-|5503|5979 >NVVL01000003.1 Rickettsiales bacterium isolate NORP64 Contig_source1382A_1001, whole genome shotgun sequence
MLKAQLNTKSISRIAAIQTMYQYEDGENDIDIETLLLKLIDFYKNEDIKSDYELDSKSKL
KPRPSYNHLKELVKFSHENLEEIDAIIETHLTKEWTLQKMPKLLTATLRVAICEIKYFPE
TPRKVIINEYTDIASDMLDEGEIGFVNSVLDHYSTKKI

>gene_159|GeneMark.hmm|243_aa|-|6121|6852 >NVVL01000003.1 Rickettsiales bacterium isolate NORP64 Contig_source1382A_1001, whole genome shotgun sequence
MNQTTFPFSSGNDYDPNEYIESSSNKAAYNALMHWPEFAGVEPYARVLIIEGAKSSGKTF
LARRWAAKSGALFIKKMHELTENIIANHSAFIIDDFDESWEEEKLLHHFNIIHEHSKYLL

ITTRKLHEASLRETSLRERSLHEASLPEIKLPDLSSRLNASNKISIELPDDELLKILIFK
LFSNRSVVIGADVVEYLIKNLAREFQVIINSAEAINKFALENKRKITIPLVKQVLLAEKI
QIA

>gene_160|GeneMark.hmm|347_aa|-|6868|7911 >NVVL01000003.1 Rickettsiales bacterium isolate NORP64 Contig_source1382A_1001, whole genome shotgun sequence
MNKVIFWCASLLLIGGLMYVLSSTIAPFLVALMFAYLLQPSIETNCKRFNLGRPIVTFGV
FILFLSSFVTVIVLVVPIIFKQFAMFLGKIPQYKDNFEASVTSWSARLNDIDPELADKVS
DSLQSFINTAFSMFASLANHLWHYTLATINFFTFVALMPIILYYFLRDWPKIIKAIESML
PKHGKSKIREIFTSINQLLSAYIRGQLNICLMLALYYIIGLEIIGVDFALLLGVLSGFLI
IIPFIGALVSFLLVMVSCYFSFGYGVELLYVTLLFFIGHSVEGYVLAPKIIGDRIGLHPV
WIIFAVFACGSLFGILGVMFAIPIAGIVKVFLSHLIGYYKSTQIYKN

>gene_161|GeneMark.hmm|406_aa|-|7912|9132 >NVVL01000003.1 Rickettsiales bacterium isolate NORP64 Contig_source1382A_1001, whole genome shotgun sequence
VKTQRVQNHIYNNKAKSTSLAPAADAAQAANLACPSSLAHCFLLIVFALCFCPPQSFANT
GVISEIFIEASGNNKHEARIKAHELGMQRALFLVADKINIPTDDLSPVPYEDLRQVFKPI
TIADELSLSEKYIATVTYSYDQGKLYNLLLEYGNEEINDLFYEALIIPVLKQGITLNVWN
DDKKWHDIWGEHRDSLNEYKIFYPEKTLYLENKINSANLRELNYDDFTKIFHGKLFKKVI
IISGEFFTNRRTAKSLLRVRHYAHGQEEESMITEKDYDLKDWSDIANTIDLIVTKLIDSE
GKTRTTEPEEMAENEDVFHNPMADLKQIMMDIDVFDQEELDLVTAKLAAVKEIESFKISH
SYESRYKILIYTSSSEYALAEGLYLNGLSYKIHGNLYNLIDIKKGG

>gene_162|GeneMark.hmm|251_aa|-|9150|9905 >NVVL01000003.1 Rickettsiales bacterium isolate NORP64 Contig_source1382A_1001, whole genome shotgun sequence
MEKEDNGSILDDVFEKINKGEAPGEFVDFVDNLENPTFVSESENAPAEGAGAENTDKTES
AYQNENASENVSSNQNEGARQAESSYQSEETTQNQPDARTREELLAEIDSLKDKVLRGLA
ESENIRTRAAKSSQEAREYAIFSFAKDLVPVIDNLSRALEHLPENLSDDTKNIVEGVKMT
KQELESAFKKHSLDSISPHSGDKFDYNLHHAISQIVTEDQEEGTIVNTMQMGYKIKNRLI
RPAAVVVAKGS

>gene_163|GeneMark.hmm|866_aa|-|10081|12681 >NVVL01000003.1 Rickettsiales bacterium isolate NORP64 Contig_source1382A_1001, whole genome shotgun sequence
MSKVEVIMAEVITDKAKTPEVTMAEVITDNDATTSITPFLIDDTMKTMSKNFLAQAQKVS
EGFLFIKNQLLECPPEQLEISLHQFNVASSNVAGSLGIRAMVQDGNLQTLILLAKKLPDI
IKSMFGIEASFLIETLNFKKPPPTDIEPAQDVADRELARPSNTEPTTAERLPKNVEDLLT
YLSYTVRFHDSEILFSARQKECVKLSFLHIAAIIGNKIILKHLLTVGREEGATNGMSSTT
RVDYKHNNVIHYLELWGHKDVEKALLGDFSSELNPLRGHANSSGNKPKAITPSNAYAAFK
KNIMSRVQGYEPLKVLTTEILMAPQEETPPMELEQKVSPDMPQVQLEASTTAPVIITESS
IISYYFRMLLSDNVEESCKLLPAVANGLVALLNNLTNDINNSTNRIIAHNYLCYLGKIIS
IYGQTEGKLLDKVTNLCAAWGNHEPLAQFWILSAEKESFTPRDRVKYMLKAQSAIEGCTP
ETKVNFYKLRSSLGTNEHASNNALKKALKIIEDNGLQPNLTELAQIIPFQIQYCIQHPNT
PGSSIDKALELARLMDDKFQTELGCSLNLYCLYVNLMLGDDNAKHTVKQAISEMDVSTEH
QTAQKLYAPIVHEISKKILISDLPENSRDLDTKLIMIALNILLGAAKYNEEGLELAQTYS
QKLAEHPQYADEQSAAASEYPATDIGISSYLYLFKKLLTNTSDEPISQSALDTPPNLSRAH
RELCEEVSKLIQFVRSLSGATRLPTYLDHLSTTGSAAFNDLLGNSNPDYESSLPEPQNNL
ETAIDNSVQRFQGLLLNILENPDWYVIPNELLRVNDSLSSEYEAPPSAPPEELELIQLPN

PDVPCAGDAAAGAGSDSDTGSDSVSV

>gene_164|GeneMark.hmm|612_aa|-|13005|14843 >NVVL01000003.1 Rickettsiales bacterium isolate NORP64 Contig_source1382A_1001, whole genome shotgun sequence
MTHSGTNSAITSDNYQNLYSSMQAERKSPRDYDQGGDNIEKALESMESARKFAIALLMAS
AKNMGIPGDGDSDSNQASEMMQISRGIAEMQATEAEILATKALIEVQKNPAVNLMDLKDK
IVDYDDSTRNFQGKPISFSYDISHSEKSESALIANIFTIRDAQGNTVKTVSQPGKAGSHE
FIWDGTDNKDQKVPNSEYSISVESSGHKIVNGTRIPFAVEATTNLSGIVESIKIEKGVAN
SIVINGKPIMRDQISGVREPEEVEADLSLTPDLIGKYARLDLDRAQVKDGSMDVYFHNHT
EHSGVLSVQIYDENDKLLKTITSTEQAGSGMGKLTLDDVGLENGDYNLKIHLRSEQLDQD
IRLNDSIIDVAVAGINPREGNFMSARENSFSPHNIQFIVGNYLNPIQQRQAEYTNSEIVY
RDDGFIFKGGNPMEFSSVAKKPQEDGSILSHVDMRIYSADGNSDLVAIVRGEYDLYSMLD
SDSAAIVDNWDEQDPIAKNRFIEDGLLDGGELHLEEEYENSYKDNVVSVDFTWNGEFMGA
DDLHASEGQKFQRTTTPIYIKEDGTIYGGETMIPTKNAFALSVDQAEGELTLNLANGQKI
PEELVMDSRKLS

>gene_165|GeneMark.hmm|544_aa|-|14859|16493 >NVVL01000003.1 Rickettsiales bacterium isolate NORP64 Contig_source1382A_1001, whole genome shotgun sequence
MSAAVLEREVILPTRHKSGGNSASYTRKNESGEFKNLLETRTNASTSRSSSHERKAPRET
TREAEPLQTTSSEKTAETDFENTSATPSPEQLPMLSTSQHARGKEDALEEEPDLLEDDDL
ENTELQNAGLGNNDLENNTLKDTEEQLAQEAQNHPEFTLASAAAEPATPSLHNNAADALN
AANMQQAQLSDGEIARAQHSKATTHIAEIAENIDITSLKLGSVDQATEAQRNQKASEAIN
LTELTGLKINKVPSQNQPAPANPSGDAEPILSPKLLLAEESSRGQTVLSQISSNTRPSRS
EQAPIASSAAETALPSAVKPELANLNSNGGEAGTSARKKPATQLPTMTEKSENVFQISFQ
DNIPRNTNALSNALGANITHTHPPKPQAQIALAVNAAASSGTNGAKEITINLFPDALGAV
KVEILSAIGQDGVSKIQSIKIIADRRETLEILEKSRIDLEKSLKEVTDTKEEASLEFEMN
QQGEQQRGGYFESLEERANWMSKFADITSEDAQDSDPRNIPNELLTSIPASTGYVTKYSV
NIMV

>gene_166|GeneMark.hmm|218_aa|+|16779|17435 >NVVL01000003.1 Rickettsiales bacterium isolate NORP64 Contig_source1382A_1001, whole genome shotgun sequence
MSAQLIELVIFAAIAFFIINKLIAILGSTSDDDPTKRNSFFGENNSMKDVTDTGTASTGK
ERATILRPIFAKKKKIELGTLIVAENEKDIQAGLSELLEKLPAFNIKSFVKNVKSAFQMI
IEASNEEDDSNLKELVDARYTEAFKAIASSYGEYSAKNELTAQISEIFMFGNNAFVKMLF
SGKNITSKTSDMNEEWTFSKSTLNSSPVWQLTNIDRPQ

>gene_167|GeneMark.hmm|79_aa|+|17616|17855 >NVVL01000003.1 Rickettsiales bacterium isolate NORP64 Contig_source1382A_1001, whole genome shotgun sequence
MKYPFLLMIKFYQFFISPWLGKSCRFYPSCSEYAKDAIIMRGVLYGLYLSAIRILKCQPF
SDGGYDPVPEKRSSRNGSE

>gene_168|GeneMark.hmm|605_aa|+|17938|19755 >NVVL01000003.1 Rickettsiales bacterium isolate NORP64 Contig_source1382A_1001, whole genome shotgun sequence
MKGVEIVKSYLANAPESPGIYQMLDEDKNIIYIGKAKNIKNRLSQYTLKLSVKNTAMVAL
IRHIEYSITESESAALLLEGQLIKKFKPKFNILLKDDKSFPYIKLRMDHDYPQLLKFRGR
NLTGGKFFGPFASAMQVDVTLSELQKIFKLRSCSDSYFTNRARPCLQYQIKRCYAPCVNK
ISKNEYDDLVRQAQAFLEGKNKKLQEMLSAKMEQLSADMKFEQAAEIRDRIKAISYVQLK

SGGAHSLPDADVIALASHNGEFCVQLFIYRAHQPCGNQAYFPAHTERGESAGNVLSSFLM
QLYQNKTPPKQILLSHEIDDIKMHEQALKKLHGNSVTISVPKHGGRHKIMENALRNAELA
LEKHLKISAKNTNALEAVQELFSLPELPRRIEVYDNSHIQGAFPVGAMVVAGKDGFEKKE
YRLFNIKESDQTSHGGDDYAMLREVLTRRIKRLKSEPHRKPDLMIIDGGRGHMGVVMEVM
AKLGTSLPFICMSKGALRNSGREQFHRPGHDTFTLDKNLDVMKYLQILRDEVHNFAIKSH
RNKRAKSIRASSLDQIDGIGESRKKALLNYFGSFKAISGATISELKKIDGISDDLAKSIY
EALRS

>gene_169|GeneMark.hmm|1005_aa|+|19895|22912 >NVVL01000003.1 Rickettsiales bacterium isolate NORP64 Contig_source1382A_1001, whole genome shotgun sequence
MSKDNSENAIDTPSIFDVAIERRALSLSEIQAFSEFAQSLRDKNSDASISLAKYVRETFF
KGQNIVVVPDLTGLELEDLNLKNVDFSGSVIRNTRFTRCDLTDSVFCDTDLNGAFFDSTK
LKNIDFRGANLVNCEFGESYGHLAPSEMDEFSSGIKFSITADLGRRYADIKSNNHRLLER
QNLRAAKQGEIDRAYSELTFVQRVRSLSNASTGYDYYDNLTAELANMQKGLYPKEYIMHE
SFQRVFGSQGSYFDPCYMRGSGATERGQEKRYIRTDRAEMEAYLAELKSNPKLILNDFVR
QRHNAAYPNEMIDDAVIVHADCSSRVGLDNEWILADFSGLNFTGANLQSVNFAGANFTGE
NSEGTVFTKANISNSSFEGAILDNAQFIDTTAIDANFFDADIAAEITGSDFTRAYMANSR
AHTPNFGDAEVASGTGANAGVRIKESIFNFADISNANWDGITIEDSTFNYANLEGVSLAS
ATIRQTKMQHANLSRAILKEATLIEVDLENARLEQVNAFKVTIQKSVLRNISAREINLSE
ADLDEFCTLDGADLTQAIMKKAKADKVSFINAQMSGIQASEASFQGANFQNAQLRFAQMQ
EAKLNDAKAHNVDMTGADLTYIEAQRADFANANLEAIQASRANLTDAVMEGADIRAACFR
NALLERVNLHKAKINPLTDISNSNIDGASGTLETEEGENISIKDKKTQDDLVHSAKETGM
FRRVAGGLLSVVAWGAKKAAPYMRQIFSSKTGKIAGAIILGAALAVGAGWGLAAIGAATG
IIVGGAAIAGLVGAGIGWKSGNKAAQNFGITGAIATGFGAYYGGAPGAAMAATASVAANV
IVRGAVGRSIDEGIGDGVEYAANRLDDLSSNIGLSREQQLALKAHQQAEMHYIHPQEIDS
PVRSSAQGSLWRDQYNNERIKQAARSKQASPSASPPPPHSDSAKSAEAESSSGQGAAANA
ESQPKSQPQPQEPPLAKASKKVSHVERTAKKEVDKKESKISRNTG

>gene_170|GeneMark.hmm|212_aa|-|23073|23711 >NVVL01000003.1 Rickettsiales bacterium isolate NORP64 Contig_source1382A_1001, whole genome shotgun sequence
MKLLSGNALYGIQKTSLEKDISKVISNVKISAAEIADSASEQALKIKSGVKSSTYNNNYK
GRQNSSGSKLSGFVGSYGNKPFSVLNRNFAELNQTIKTDYETGIRQIMASLGVEDSASKL
AINSAILTLETALNKGAIAAFAIAAPAPSTDQEFALKTSITNSALLAVSDSDENEGGNTG
HGSWSGEEDAPEENKDDKPTDDSNDDELNILV

>gene_171|GeneMark.hmm|248_aa|-|24088|24834 >NVVL01000003.1 Rickettsiales bacterium isolate NORP64 Contig_source1382A_1001, whole genome shotgun sequence
MMALVLGLTAIMHEYTGGALLLTGAILSVIYCAANWWSDVITEGRVGKHHTNEVQMGLRI
GMSIFILSEIMFFFAFFFSIFYVSIFPTGIMDGLWVISEGSWPPEGIKTFDPWDIPFINT
LILLLSGTSVTWAHHAIKNNDQKNSVIALGITILLGMSFSMLQAYEYYHARFGLSDGVYA
ANFYLATGFHGVHIIVGTIFLIVCFFRARKGHFLSGNGHLGFEFAAWYWHFVDVVWLFLF
VFVYVLGR

>gene_172|GeneMark.hmm|185_aa|-|24964|25521 >NVVL01000003.1 Rickettsiales bacterium isolate NORP64 Contig_source1382A_1001, whole genome shotgun sequence
MGAKSNKDIVIAVLCLLLSMMILTLASVPIYNLFCKATGYGGTTQRVNPYALVAKGKRKI
LVEFDANIDSKLPWRFIPKHRSAEVVTGENTLIFYESENLSDHDIIGTSIYNVTPFKAGK

YFVKIHCFCFEEQLLRAGQKVLMPVSFFVDPAIEDDPEMDDVNRITLSYSFFKVRDLPDA
KNNGL

>gene_173|GeneMark.hmm|244_aa|-|25522|26256 >NVVL01000003.1 Rickettsiales bacterium isolate NORP64 Contig_source1382A_1001, whole genome shotgun sequence
MKNKLIIQNISKSYDKQAVLRGVSYHLEQGEAVGLFGPNGAGKTTLFSITIGLVKPDLGQ
ILLNQQDITNLPIYLRGRLGIAYLPQEPSVFKGLTVQDNIKAILEIAYTDEDEIVSKTEE
LLDEFSISHLKNKFAGTLSGGERRRLEIARALATKPNFIMLDEPLAGIDPLAVRDVRQLV
SHLKDRNIGVLITDHNVRDTLDIVDRAYIVYDGKILMEGKPEDIISSNKVREVYLGQTFS
KKGL

>gene_174|GeneMark.hmm|144_aa|-|26259|26693 >NVVL01000003.1 Rickettsiales bacterium isolate NORP64 Contig_source1382A_1001, whole genome shotgun sequence
MSLILKRLVIFLIYISPLSYHALSDERLRIDSDNLTIKKDDSSASFTGSVEASFNDYKLT
TSKLTILYGTINGKKDIVKIVIPSKLKLIKPCSEEIAIADRGIFDNKTKKLTLTGNVMLQ
QKDNILATDKLVYSGNFKAINHKK

>gene_175|GeneMark.hmm|209_aa|-|26709|27338 >NVVL01000003.1 Rickettsiales bacterium isolate NORP64 Contig_source1382A_1001, whole genome shotgun sequence
MPNTDKILARIKLLKYLFLSAGICVLIGILATIYGKDMFDSSHANTNSTNSLALRPKLSK
NHTLSINHSIFEGVSKDLTPYKIIAENVTRNSDNKYQLNRVEGDCSLKAGGLKMRAAAGT
IDMSTKLVTLKQDVYVTLNENTIFNSDEIQISLNNKDISSNSAVSVTLKDSNIKADKFKA
LGSSDDIKFEGNIKFESDVKSFSFKATNK

>gene_176|GeneMark.hmm|325_aa|-|27339|28316 >NVVL01000003.1 Rickettsiales bacterium isolate NORP64 Contig_source1382A_1001, whole genome shotgun sequence
MKHTHMKHTHMKHTDIAHNVITSQGDALAVLAKNIPADFAPLIDCILALKGRVILVGMGK
SGYIAKKIAASLASTGTPAFFTHPAEASHGDLGMITKADLVIMLSNSGETKELFDTIDYC
KRYNIKIASITMKEGSTLAKNSDFLLKLPTQKESSAIAAPTTSALMTLSLGDSIVTALHE
AKDFTKEDFKNFHPGGKIGANLLKVQDIMNSGEQLPVVSGGDAFADVIIKMNSKCLGCAV
IVDESKTMLGIITDGDLRRHIGGDLSKISAKDVMTPSFQSISPNALATEALFIMNDKSIT
ALPVLEDGLLVGVVHIHDILKAGVS

>gene_177|GeneMark.hmm|197_aa|-|28322|28915 >NVVL01000003.1 Rickettsiales bacterium isolate NORP64 Contig_source1382A_1001, whole genome shotgun sequence
LPDDFDLSGDLAIDTETMGLMLHRDRLCVLQISTGDGDACLVQFDGKDYSAPNLKKILLD
ENRCKIFHFARFDLAAIKKYLELDLTNIFCTKIASKLVRTYTGHHGLKDLCKEYLGVTLS
KQQQSSYWGTKDLSSHQKAYAASDVVYLHSLRAILVERLIDTGRTEIAHKLFEFLPTRAN
LDLIGWNEIDIFSHMST

>gene_178|GeneMark.hmm|126_aa|-|28965|29345 >NVVL01000003.1 Rickettsiales bacterium isolate NORP64 Contig_source1382A_1001, whole genome shotgun sequence
MNVERFKVKAGTYKLVLMRYRAPRSFNFTRKTLSNALILLMLLYFVFHSIYGSRGILAYF
TLQAELESSHKKLELLRAERLEVENRTKLLRPGSLDRDMLDEKTRNVLGLSSPKEKLFKT
IKTEDK

>gene_179|GeneMark.hmm|234_aa|-|29360|30064 >NVVL01000003.1 Rickettsiales bacterium isolate NORP64 Contig_source1382A_1001, whole genome shotgun sequence
MNMTKMINMLKTTKIAKNKEHIMQRILSGIAIGLIFIIAIFLYRPLFTILIYLIAALMLL
EWFNMTNKSASHLYAGLPIVALPIASLLIISDIDDDGWLLFSFFCLVWSVDTMAMVGGKL
IGGMKLAPSLSPNKTVSGLCIGVLSAMIVVNILSALPSYQLPYMLPKSMIYLNIIYMLLG
VIAQLSDLFISYFKRHFSIKDSGTIIPGHGGVLDRFDSIILTAPLVALYLISNL

>gene_180|GeneMark.hmm|227_aa|-|30071|30754 >NVVL01000003.1 Rickettsiales bacterium isolate NORP64 Contig_source1382A_1001, whole genome shotgun sequence
MNHNLNHLAIIIDGHARWAKQHGKTKVEGHRQGAQTIKSLLPHISASGIKHLTLYAFSSE
NWSRPAEEVSVLIKLLSHYVTTETGTLNDNNIRLKIIGDLDKLSPSLQKQIKTIVEKTEK
NSKMILTIAFSYGGRGEIARACQKAIDSGATNITEDSFKEFLYDPKMPDVDLLIRTSGAH
RISNFLLWQTAYAELYFIDKFWPDFNEKDLAVAIENYSQRERTFGTR

>gene_181|GeneMark.hmm|442_aa|-|30766|32094 >NVVL01000003.1 Rickettsiales bacterium isolate NORP64 Contig_source1382A_1001, whole genome shotgun sequence
MFFIPSNIKILNNIKKLLPKSLLIRFMLIIMVPTLIAQLLAVYLFYQRHWYNVTRHTSSL
VTTEISSLLSENYETTPNKQHSYQNLSYIFIPTAKLPAQQKKYAKELEIFKLSLQQRISQ
EIVLISKDNGRNYLYLQIGDNVLKIELPYKSLIHPSTRIFVLWIFFLTILLLSVSLIFSR
NQINSIINLTNAVESYGIGEKTKIYKPSGASEVRRAGLAFLKMKDRIDRQTAKRTQMLAM
ISHDLKTPLTRMKLQAEMMENPEEKEELMHDIDSMKHMISSYLDFAHGEGGENFQKIDLN
QWVPEYLNQKWSSENISVNLGDIGIFAQIKPHSFERALSNIISNALKYSTKIKISVSLSS
EKENAMIIIEDNGKGIKEHERKLVFKPFYRADKSRSLGDLSSVGLGLAITKEIINGHYGT
IILDTSIELGGLLVKIKLPIKT

>gene_182|GeneMark.hmm|225_aa|-|32078|32755 >NVVL01000003.1 Rickettsiales bacterium isolate NORP64 Contig_source1382A_1001, whole genome shotgun sequence
MNKKPHILIVDDDSRILKLLKKFLSQNDFLVSTTISASGGLELLSNFTYDLIILDVMMPE
ITGLDFASKIKNAGNTIPIVMLTALSEPEHRIKGLEAGASDYVTKPFEPRELVLRINNLI
NARTQYEKEKQSVRFGNNNYDLTTKELTQNNQTIKLSSSEQQLLDILLESSNETISRTEL
SEKIGVLNSRSVDVQIVRLRNKIETDPKNPKFLKTIRNQGYVLYT

>gene_183|GeneMark.hmm|291_aa|+|32845|33720 >NVVL01000003.1 Rickettsiales bacterium isolate NORP64 Contig_source1382A_1001, whole genome shotgun sequence
MDFKSSKKAADFIWINGEFIPWEDAYIHVLTHSLHYAGSVFEGERSYGGKIFKLMEHTKR
LINSAEDMGLEVNYSVAEINEATEELLKKNNLENAYIRPLVWRGAGALGVYGENMVSNLL
IMAQNSNHPFKNGARLVLSKWRKPTIDAMPPQSKSSAHYAMQIVSQQMAKQQGYDDALLL
DQYDDIAECTTTNIFFGKGRELVTPIADRFLNGITRQTVIEMARAMGIRVREERLTLEDA
EKYDTCFVTGTATEIKGVSSINYDYKKLEFPNDNMVKDLQEEFAKIVGKQI

>gene_184|GeneMark.hmm|296_aa|+|33717|34607 >NVVL01000003.1 Rickettsiales bacterium isolate NORP64 Contig_source1382A_1001, whole genome shotgun sequence
MTSKNIFKGVLTALITPFKEGVVDKGVLENMIEMQIDAAIHALVVGGSTGEGGLLPDDEY
YNLIEIAVKCSAGRIPIIAGISSISTRAALEKVLRLNQIGVDGIMSTVPHYVKPEQDGIQ
MHFEAVASTSKSPVMIYIHPSRTGSEISDETLLNLAKQKNILALKDAGDDIERPLRILPK
LMHENFTMLTGDDSKMLAYTANGGDGCVSVLANILPDKCVEIYDLHASYEALVVQREVTP
LLNALAAESNPICIKYAASKLNLCRDEESYTLPLTKARAETREAIDLALRELGLPR

>gene_185|GeneMark.hmm|153_aa|+|34641|35102 >NVVL01000003.1 Rickettsiales bacterium isolate NORP64 Contig_source1382A_1001, whole genome shotgun sequence
MEEYKKIVANNKRAQFEYFIEERMEAGIVLTGSEVKSIRGGGVAIADSHAAFSDGELYLY
NCHIAEYEKANRFNHETRRPRKLLLRGKEIQKILGKIKLKGYTLMALSVYFNSKNMIKIE
LGLAKGKKQHDKRQTIKENDWKREQGRIMRDKK

>gene_186|GeneMark.hmm|307_aa|+|35102|36025 >NVVL01000003.1 Rickettsiales bacterium isolate NORP64 Contig_source1382A_1001, whole genome shotgun sequence
MNISPDYLIERKRSKKQLSNWKIIALILLAALLFSSFGKLPASKNFAAGGFQSKDYIASV
QIENVIEEDNLRIRKFESIEKDDSIKALIVNINSPGGSVVGSEKIYNVLRKISKTKPVVV
IMGSVAASGGYLASLGGDYIIAHNGTLTGSIGVIMQSAEVTKLAEMVGVKFENFKSGELK
ASPNPTEETSPEARQATMDSIYEVYDYFVELVAARRNLDVEYVKKIADGRVYSGRQALDL
KLVDAIGDEDSAVKWLQEEKGIPFGAEVVDVKIRPRDKLIEILLEDLQGCVSGFFSALGS
KSFKSVI

>gene_187|GeneMark.hmm|83_aa|+|36063|36314 >NVVL01000003.1 Rickettsiales bacterium isolate NORP64 Contig_source1382A_1001, whole genome shotgun sequence
MATKKDLVGRVLQKLDYLSPEDVDYAVDSVLDCLKAELALENRVEIRGFGSLSIRKRSHP
KKDELYNSVYYRMSKNIQERLND

>gene_188|GeneMark.hmm|166_aa|+|36307|36807 >NVVL01000003.1 Rickettsiales bacterium isolate NORP64 Contig_source1382A_1001, whole genome shotgun sequence
MTNLKIGDIAPNFSITDNAVADTSVVAKDGSATTLSDFAGKYVVLYFYPKDDTPGCTVQA
RGFNSLLKEFNDLGAEIIGISKDDLKSHEKFTNKYDLQFPLVPDTDMEICKKYGVWVEKS
MFGKKYMGINRESFLIDPEGKIVHIWPKVSVLKHAAEVLAKLKEFV

>gene_189|GeneMark.hmm|181_aa|-|37266|37811 >NVVL01000003.1 Rickettsiales bacterium isolate NORP64 Contig_source1382A_1001, whole genome shotgun sequence
MEKSYSDVIKFWFEEIEPIQRWKKDPNFDAMITQKFGHVHTQASKGVLTPWRVNALGALA
EIIILDQFSRNIHRDTPAAFANDQLALRLAEDAIEQKYDQELEVSHRSFLYMPFMHSEEQ
KIHNRAMELFVQDGLEGAYDFEKKHKVIIDRFGRYPHRNEILGRVSTPEEIEFLKQDGSS
F

>gene_190|GeneMark.hmm|308_aa|-|37867|38793 >NVVL01000003.1 Rickettsiales bacterium isolate NORP64 Contig_source1382A_1001, whole genome shotgun sequence
MEFLAVIGFILAILAWGGVKIVPQQQAWIVETLGKFDQILGPGLNFLIPFVQKVSYKHTL
KEVAVDVPRQSAITKDNVTLDIDGILYMRIIDAKAASYGVSDPHYALTQLAQTNMRSQIG
RLTLDKTFEERENLNANIVNSINEASSTWGIQCMRYEIKDITPPHSVLQAMELQVAAERQ
KRAEILESEGKRESQINIAEADKREVVLKSEAALTDQINRAKGEAEAIVAVANATAKGIS
TIAVSIQEQGGSDAVSLRIAEQYIDAFKELAKESNTILLPANVNDVGSISSIVAQGTSIF
DAIRQQKK

>gene_191|GeneMark.hmm|156_aa|-|38824|39294 >NVVL01000003.1 Rickettsiales bacterium isolate NORP64 Contig_source1382A_1001, whole genome shotgun sequence
MIFEYLTNNTHTLWLLLGAIFIVLEVTAITGIGFLFAGLAAISLGGIIQFDLITSNDPVL
EIAIFLLLSFVWASILWIPFKRFKHSRTNKPFSNIVGDIAIIESSTLAPGKIGQVKWSGT

IMRAALDSKGTAVLTKGDQVVIKALNGNILIVESIN

>gene_192|GeneMark.hmm|233_aa|+|39736|40437 >NVVL01000003.1 Rickettsiales bacterium isolate NORP64 Contig_source1382A_1001, whole genome shotgun sequence
MKPLYKSLRESIESGDYFQDSRNWYKYKYIYPFAQRSLVLILSAIIFVIFFGVVLDLYAL
LPIVSQVNYSIATDYSQNSNAKIIHANRVPNSPLISIADIMLRNYVKNREHYSYSGLKKQ
FIFVKNNSTRIVFRRFYNSMNIDNPLSPVMRYQKTIQRQAMIISTHHPFAGKSIVKFATI
AQSPSRAHIENIVWQATISYEIDKINTASPSGTRFNFTVTDYKLKLLENKNAK

>gene_193|GeneMark.hmm|254_aa|+|40441|41205 >NVVL01000003.1 Rickettsiales bacterium isolate NORP64 Contig_source1382A_1001, whole genome shotgun sequence
MENGIMKQIIILAILLVSASSVFAIRESRPTSIDSRIRVMVYNPNDVFKFTGYYGYQASI
ELASGEEVVSISMGDTTSWQIVPAGNRIFIKPMEQDATTNMTLITNKRTYYFELYAEETL
DIRDPAMVFNVKFLYPDEEEEDHIRTFASASQAEPSLNHPEKFNFNYSISGNESIAPIKI
FDDGEFTYLQFRDKNNEIPAIFAVDKDLRESMVNFRLDPNNRNLIIVEQVFHKLALRSGQ
NITCIFNEAFRQGY

>gene_194|GeneMark.hmm|89_aa|-|41569|41838 >NVVL01000003.1 Rickettsiales bacterium isolate NORP64 Contig_source1382A_1001, whole genome shotgun sequence
MCSKSDHRSHKGLNNRVENAHQPTRKKEKITIKFKSPQGVQNTLSLMGKIRNIFSVDVGR
YTQNATNQRSAFNKAKSIWDDAAQGLLAA

>gene_195|GeneMark.hmm|109_aa|-|41948|42277 >NVVL01000003.1 Rickettsiales bacterium isolate NORP64 Contig_source1382A_1001, whole genome shotgun sequence
MYINKAPKRHRFPVSIISHVVWLYHRFNHSDRDIKEQMAYREIILSHETIRSWCYKFSSH
FKDVIKKQERNTKDKWHLDEMTIRINGEYFVLWRAVDSDGYELDISTET

>gene_196|GeneMark.hmm|115_aa|-|42362|42709 >NVVL01000003.1 Rickettsiales bacterium isolate NORP64 Contig_source1382A_1001, whole genome shotgun sequence
MTKKIAKKSEDINSKLIEELLSKSDPGELFGKEGMFSQLKKQIVEKILESEIEHELGYSK
HSKMSKSNSNRRNGSYEKTVLDDEGYKIPIEIPRDREGEYEPKLIPKGVRRAPSS

>gene_197|GeneMark.hmm|90_aa|-|42751|43023 >NVVL01000003.1 Rickettsiales bacterium isolate NORP64 Contig_source1382A_1001, whole genome shotgun sequence
MNYTPRLNLPLIHSGQAQKEITHNEALSMLDVLINPAQKIGQGWRFVTPSKWLEVTLDRD
GSKHRFNGEYWAPSSMGVCHKLCVSCKKIQ

>gene_198|GeneMark.hmm|82_aa|+|43097|43345 >NVVL01000003.1 Rickettsiales bacterium isolate NORP64 Contig_source1382A_1001, whole genome shotgun sequence
MTHVILSNIVASISELKTNPMSTVRSAMGKPLAILNRNQPVFYCVPADLYEAMLELIDDV
ELAQIVTQRASEKEIAVDINDL

>gene_199|GeneMark.hmm|58_aa|+|43335|43511 >NVVL01000003.1 Rickettsiales bacterium isolate NORP64 Contig_source1382A_1001, whole genome shotgun sequence
MTYKLTFLESAKKEWRKLSPDIQVQFKKKLLNRLSEPKVSHLIIPHLFVLKLYLIFSF

>gene_200|GeneMark.hmm|1239_aa|-|43708|47427 >NVVL01000003.1 Rickettsiales bacterium isolate NORP64 Contig_source1382A_1001, whole genome shotgun sequence
MGSFASMALPIAGGFGGFMVGGVGTAIMGANLGSAIGGMFASGKSRTVRLPSQNGARLGD
LRVQISSYGEVIPKLYGTMRLAGNVIWSTDLKETEHVHTQSFEQSSGGKGGGGRRTVTTT
QNSYSYTYSVTLAIAICGGEVDEISRIWADSKLISAETLQTNQGKFNIYLGTEDQLPDDI
IARHKNGAEFPAYRGLCYVVIEDFPLEKYGNRIPNFSFEVRRTVRFVPAVEDKVRELILI
PGSGEFVYADQVCAKKRQFPYGGTYGHSVINMHNYDGKPDMLLALDQMQNTLPNLEWVAL
VVTWFATSTNAGNCRIVPKVEFKDEEAIIEPQEWGVAGFTRETAEEVLRFDERTPTYGGT
PSDHTITAICKELKSRGLNVMLYPMIFVDELEPHPKPWRGRIRANSAIEIDHWFNGGSGY
NRFIKHYADLTCGIVNAFVIGSEMIGLTGFTDRPGSYPATAQFIKSAEMIKDRVSNNTIV
TYAADWSEYHHTEGGWFNMDSLWASKAIDVVAIDAYFPLTKDLPQSEITEELIQAGWESG
EGWDYYCDEDRTRHPFHGAKYAWKNLEHWWSTKHTNPDGNITEWQPRMKPVWFTEFGFPS
VDACSNQPNVFYDSSSIESCFPRGSLGRVDFLAQRQALNASLDYLESRTKRNGLDQLVPK
RFVWTWDARPFPAWPDNKRAWQDGDLWKTGHWVNGKFGQSSLAAIVADLLCEVGLSAADY
DTSRLIDQVDGYIISQHITAREAIEHLQAAYFFDVALSDGILKFVPRIGRTNVAEITESE
LVSNNNQDINETIEITIAQELELPEKINITHIDKGREYDTATTSSKRQTVSSKEQVNIIL
PISMNQQQAKQIADITLYNAWQERKNYQLTLPPKYGYLEPTDIITVQTGNITHQMRIVKT
DMQRTAQMRIVATNYNANMYDFHSSIQGEYEYQEYDFPLPVAKTKIELIDTPPLPSDQTT
ATVRIAVIPEHENWNGVAIYKTTNDGYDYKLLTTTNIASITGVSLNKLPPAASTIFDYRS
EIIVQLPYGELNSVIELVLLNGANAALIGNELIQFQHAELIADHQYKLTKLLRGRQGTEH
AIDTHSEGERFILLNEGILEGKVSHDMIGRSVLYKPVSIGSTLAETESVEFTHYAIHLKP
FAPVHVTAEHVENNIIKLSWIRRARTNHQWRDNVDIPLGEEYEKYEIDIKTSDGKLITTI
ESTEPAALYDRDQSQDNLTATIYQISSIVGRGYGTRIEV

>gene_201|GeneMark.hmm|156_aa|-|47448|47918 >NVVL01000003.1 Rickettsiales bacterium isolate NORP64 Contig_source1382A_1001, whole genome shotgun sequence
MKTKLSQKIVTAARSWLGTRYHHQGRLKKTSKHKGGVDCVGLVMGVVAELNITGAGGKNL
IELDRTDYSAHPDGRSLVEFLDMHLQKTNLKKIKDGDILLFKLFKHPQHVGIATLGKIRG
LGVIHCYSASGSVVEHILSPAWLRMIVGVYRFKLDK

>gene_202|GeneMark.hmm|284_aa|-|47920|48774 >NVVL01000003.1 Rickettsiales bacterium isolate NORP64 Contig_source1382A_1001, whole genome shotgun sequence
MRQLTQELTAHLSAEVTTLASCWRIERTDGLVLGFTDHDRPLMIDGVKYDSIAGFTPTNV
ESGSDMAVDNLDLAGQTFPSKITEIDLLAGKYDFAKLEVFLVNYNKPNTGKLIQKRGLLG
EVTIKKQLFYAEVRGLTQFLSQTMCESYSPHCRATLGDARCKFNLDQGEYTVRAAITEII
NNQTFRASILNHENEWFKDGYLIWESGNNIATKMEVKEFANHMVTLALPMPFAMHESDEF
SIIAGCNKSSNCCKQKFNNIINFRGEPDLPGIDKLMRTAGTALK

>gene_203|GeneMark.hmm|201_aa|-|48771|49376 >NVVL01000003.1 Rickettsiales bacterium isolate NORP64 Contig_source1382A_1001, whole genome shotgun sequence
MPSFIEAQFPAGISYGSKGGAMFSTDIVSTFSGHEQRNINWHQARARYDVASGVKTPEQW
QELIAFFRTRRGRAVGFRYKDWSDYQGVNQHLGTGDGRKTIFQMVKHYVSGDAVYSRIIN
KPVCNDFCKIYIDSILYEDGINIDFTTGQVEFRGAPRIGEEITADFEFDVAVRFDTDHLD
LSMDGFNTGSWQNIPLIEIRV

>gene_204|GeneMark.hmm|599_aa|-|49386|51185 >NVVL01000003.1 Rickettsiales bacterium isolate NORP64 Contig_source1382A_1001, whole genome shotgun sequence

MAATKEVRVTFRGVDKTKQAFGRIKKNFKDMEKLSSKVTRGFGGIGMAIGAAFGTRALKK
IIDTGDQIGKLSLRLGATTEGLSELKYAAEMTGVEFNSLSTGLQRMTRRVSEAALGTGSA
KDALIELGLSAEHMNSLKPEQQFELIANAMSEVTNESDKVRLAMKLFDTEGVALLQTMEH
GAEGIRKLREEARKLGLSLSGEDTKAMEAFNDSLGKLQAVLTGLLTSVLLPLLPAITAFF
EAIKDGNPVLTFLITALASLAGLRLAAWFAAAALSVKTFTLALMANPIGLVAVSISMAVA
ALVSLTRWFRKSTDAGAEHNKILQQAKLVSDEIVIANQKQFEALKKNNEAHLASKNILKK
TSAPLSKHNQQLEKMNKLLKEGANNQNLHNNAMEQSVHQLEQTKTINADVFSTIETHSKR
AADRMIDNLANCAFGAKTHAGSMKSTFKDLFKSLQSDILKTTLQRGLFGASAGGMSGMGG
MGGGGLLGGLFSALSGGAGSGAGGGLGGLLGALGNFGGFFAKGGTARAGKAHIVGDGGEP
ELFIPSTTGSITPFSKLSGGSNVTVNMHIQTPDIASFNHSRGQITADMARQISRAGRNL

>gene_205|GeneMark.hmm|119_aa|-|51330|51689 >NVVL01000003.1 Rickettsiales bacterium isolate NORP64 Contig_source1382A_1001, whole genome shotgun sequence
LIFKMNNRIIDRVKAHFDAKETKIIEVAEWGEGEESLYIHATPLTLAQKNRLYKMAKDDD
LGLMVEALIMKAKDSEGESLFSRSDKPDLMRGCDPDILIRVANAIMGDADDDITPIKKN

>gene_206|GeneMark.hmm|146_aa|-|51686|52126 >NVVL01000003.1 Rickettsiales bacterium isolate NORP64 Contig_source1382A_1001, whole genome shotgun sequence
MATHAGSEGIVKVGDNQIAEVKSWSLEEVSDTVDASVIGTAWRKHQATIKSWSGSIEAFW
DEADAAGQGGLLIGSEIELNLYPEGVADGRRYFTGTAIVTGISRQGAFDGIVESSFTFQG
NDELFERSHNDGGAVIIDGVAAGDED

>gene_207|GeneMark.hmm|147_aa|-|52156|52599 >NVVL01000003.1 Rickettsiales bacterium isolate NORP64 Contig_source1382A_1001, whole genome shotgun sequence
MVHARTNIRRAFIDLLIGATDAGARIYDSRIYTLEKRSLPEIIVFSDHEEIITDTISWPR
SQERSLRVSVECYVKSGNEVSSAVDNISAQVESLILASSNLGGICKDCRLEATDISLNND
GEQPVALASLVFAVLYRTLENSPEIIV

>gene_208|GeneMark.hmm|112_aa|-|52627|52965 >NVVL01000003.1 Rickettsiales bacterium isolate NORP64 Contig_source1382A_1001, whole genome shotgun sequence
MLRALQLLGLDFMFREDLTEFLDPEQGFAVNGTVIQADGMQYVLTGILSSDYVDIDSGMA
GVAGSSPCFECREDDISRIRYDDSLQVSGKHFRIKAIKPDGTGWAVLVLEEQ

>gene_209|GeneMark.hmm|709_aa|-|52934|55063 >NVVL01000003.1 Rickettsiales bacterium isolate NORP64 Contig_source1382A_1001, whole genome shotgun sequence
MTIYMNININIEAERKTMTQMTNNTEVKAEMPYRNDKLKQTQNIDLLYIDDDNLALIKNE
LSESREENPEENLGERSFYLSFSSEDPYERSFGIEILDHGEDCVDLSWLRSGRAPLLLDH
DHKKQIGIIESAEITSDLRGRAKVRFGRSVLAQSVYQDVIDGIRGNISVGYKYKRSDLVL
SEKGEEGKPDTYRVSKWEPLEVSLVSIPADQTVGIGRSDNARKSKKPKKSSKKPEQEISN
NETNIIYKVRSDMEPNKTEAKITEASAMQASEMRTSAMQTLPMQALSNIDAGFNPEQIRQ
DETTRISEILALGDKHNMQERAMEFIKTGRTIDNFRRQILDTLSTSSAINTARQENPIIG
MNHKEIGSFSIIKAIRAVISGNWRGAELEKEASETVSKQVGREPASFFVPLDIMAEQRSM
QIRNLEKITNVAGGYLVGTDYMSGSFIELLRSKMLVKQMGAKVMSGLHGDVAIPKQTGGA
TTYWVSEGGAPDHSQQSFGQVTLSPRSIAAFTDFTRKLVLQSSPDIETLIRNDLATVIAL
EIDRVAISGSGNGAEPLGILNTDGIGEVAINADPAHADAISWGKIVDLESNIAAQNADIG
TLGYLCNANMRGLLKQKEKANDTAQFIWENSGEQGFGMLNGYRVGTSNQVPANSLLFGNF
GDLIIGQWGVMDVLVDPYTLGTSGGIRIRVMQDVDLAVRHAESFAVARA

>gene_210|GeneMark.hmm|525_aa|-|54993|56570 >NVVL01000003.1 Rickettsiales bacterium isolate NORP64 Contig_source1382A_1001, whole genome shotgun sequence
MKLFNFFRKSSKAQTRNMSQRKYAAASINRLNADWVTSVLSADSELYNDLRTLRSRSREL
CMNNDYARRFLKRTGVNVIGATGIRLQVKAKDNSGKLLREINSHIEEQFHLWAKKGNCSV
CGKLSWVDSQKLFLESVARDGEVIVRLVKGFDNDFGFALQFLEADHLDEDLNMPLDNGGY
IRMGIEFNKWNRPVAYHLLSHHPSEVFHDKTSYEKKYQRIPASEIIHGFLIDRPSQSRGV
PWMHTAMPRLRMLAGYEEAELVAARVGASKMGFFVSPDGTGYAGSEHENDSPIMEADPGT
FEQLPSGMDVRMFDPNHPAGNFASFEKAILRGVASGLDISYNTLANDLEDVNFSSIRHGS
LEDRDSWKVLQNWVIEHFCRPVFEAWLLMSITSGKIEISATDFDKFNKPVWRARGWAWVD
PLKENHANQIAINQKTKTRAQVAADQGMDVEELFQQLAFEDELAKEYGLDLNTARYREDR
DRDKQLDRSSLDGDSKLDNNSENDNIYEYKYKHRSREKDHDTDDE

>gene_211|GeneMark.hmm|183_aa|-|56584|57135 >NVVL01000003.1 Rickettsiales bacterium isolate NORP64 Contig_source1382A_1001, whole genome shotgun sequence
MKKYNIEPASFTAGETVTWKKWIAGFESASGWELVYLMRSQSDDSAIEFAAEIEDDHHLV
TLASNETINYKTGQYWWQCFARRAGEQHLIAEGSMFVKPNFSQMDRVDGRSHVKKTLDAL
EAMIQGKASRDQLSYSIAGRSLSRLSPSELLQWRDQYKAEYVLTRRKSGDLNDQVIKIKF
GRV

>gene_212|GeneMark.hmm|34_aa|-|57176|57280 >NVVL01000003.1 Rickettsiales bacterium isolate NORP64 Contig_source1382A_1001, whole genome shotgun sequence
MNHDSGEPASDITKEVNRDIDLYMKYGEKYLSNT

>gene_213|GeneMark.hmm|634_aa|-|57345|59249 >NVVL01000003.1 Rickettsiales bacterium isolate NORP64 Contig_source1382A_1001, whole genome shotgun sequence
MTNALVKRISKQAAPPPRLKVSQWADQYRQLSRESSAEAGKWSTDRAPYQRDIMDAVCDP
SVEIVVVMSSAQVGKTEIINNIVGYYIHQDPSPMLVVQPTEKLAESWSTDRLAPMLRDSE
VFHNLVKDPRSRDSGNKILYKRFTGGHITMAGSNSPSSLASRPVRLVLCDEVDRYPASAG
SEGDPVNLAKKRATTFWNRKIVLTSTPTIKDLSRIEAAYLQSDQRRYYVPCTSCGEYQTL
KWGQIKWKKSTCEDSATKKSKDQPETAYYVCEVNGCILRDTDKAAMLSAGEWRAENEFSN
IAGFHLNELYSPWVSWERIVAEFLKAKLMPETLKTWINTTLGETWEEQGESVDENSLLSR
KENWGDLAPSGVVIVTAGVDVQDDRLEIEIVGFGKGQESWSLDYRVIHGDPAREDVWNDL
DNILEQTIKHESGINLRIACTCVDSGGHRTQSVYAYCKKRQLRRVFAIKGSGVPAKPIIS
RPTMSNRMRVKLFSVGTDTAKEMIYSRLKITEPGAGYCHFPARYDEQYFKQLTAEKVVTR
YHKGFPVRKWEKPAGRRNEALDCRVYALAALHIINPDLELLAKKMKIEGSNDKQHQQQND
YNQRKARSWEVTRGISHQGSRGGTGGRSSFIKNW

>gene_214|GeneMark.hmm|162_aa|-|59218|59706 >NVVL01000003.1 Rickettsiales bacterium isolate NORP64 Contig_source1382A_1001, whole genome shotgun sequence
MQTYSISTISKLLNLSERRVQQLSKDGIIPKAERGKYDLVSSVQGYVGYLYARAFGKNTA
SIDAHAEKARLLKAQATKAELELDILKDKYIETEEVEFLYGGLVLVFRSKMLSMPSKLVR
RLAAVGSDFARIEKILEDEIYEALTELSKYGDHDKCISKKNK

>gene_215|GeneMark.hmm|347_aa|-|59710|60753 >NVVL01000003.1 Rickettsiales bacterium isolate NORP64 Contig_source1382A_1001, whole genome shotgun sequence
MGLSTNISWNLSDLEKIESNGYKVFSCFSCAGGSSMGYELAGYDVLGNVEIDPKMMRLYL

LNLKPKYPFMMGIEQFNHLADKDLPKELFDLDILDGSPPCSSFSVAGVRESKWGKKTAFR
EGQKEQVLDDLFFHFINTASKLKPKVVIAENVKGLIIGKARGYVKQIIELFNKTGYKVQL
FLLDSSTMGVPQKRERTFFVARRDDLNLPELKLSFSETAISTSKALQDVDWRNNKITWLT
ENTKLLWQKTKLGAPLSEAHPKGSRFNEKKLNPNKPATTLTTHCPPLHWALPRRITVSEA
IRLQTFPDDYNFANRDGFYPIGMSVPPFMMQRLANEVQKQWLDNLEK

>gene_216|GeneMark.hmm|167_aa|-|60744|61247 >NVVL01000003.1 Rickettsiales bacterium isolate NORP64 Contig_source1382A_1001, whole genome shotgun sequence
MKIQLINISKLIPYAQNPRHNQLAIAKVAASIKEFGWQQPIVVDGNMTIIAGHTRYMAAT
QLDLKKVPVYVASSLTAAQVQAYRIADNRLHEDSSWDDELLKIELDELKDDSTDLELIGF
NAMELEKLIGDGKTDISNSKSSASREIKLSEFQEFAHQCPKCSFEWD

>gene_217|GeneMark.hmm|194_aa|-|61441|62025 >NVVL01000003.1 Rickettsiales bacterium isolate NORP64 Contig_source1382A_1001, whole genome shotgun sequence
MQISVSSNIKQIAKQLDHMQRRQLPFATSTALNKIAIAAQSSITKAIPFIFNNRKKWWGK
NQPTGIKVKFANKYELVSAVYTRAYFANIQEEGGIKTPRSGHKLAIPASGAPRKFRKSNA
LKKEQGNSRIFKSKSGKSILRRIGKNSIQKLYTLTDKAHIKPRFGFQKTAHKVFIRHFDK
IFQRQLDRALKTAS

>gene_218|GeneMark.hmm|148_aa|-|62040|62486 >NVVL01000003.1 Rickettsiales bacterium isolate NORP64 Contig_source1382A_1001, whole genome shotgun sequence
MATTREEKSNSRLMQLFAPALVGADQANIAAIDVSGFESVLVIANIGAAGDDLNQNNYIE
IELEESDNNVDFTHCSDESMQDAVAGTNTGTIAHLEAIDGAGISIMAAYKGQMRYIRPVI
RMTGNHGTGTIIGVSAILRGTKYRPVAA

>gene_219|GeneMark.hmm|167_aa|-|62710|63213 >NVVL01000003.1 Rickettsiales bacterium isolate NORP64 Contig_source1382A_1001, whole genome shotgun sequence
MKMNTAKLNTLKLQTAKPQTTKLPTPNKNDKRPIAKFHRSWLQDTLSGTPLEKHRGINQT
QYAAADRLSSNYQRIFSNGGWSPEEVQVKQDYFRSNGVERKAESIINHNRIFKQLSKNSQ
AIIEHFCLNEQPIRAYELKQIPNWPKGAGTARLREGLDELVEIYKNS

>gene_220|GeneMark.hmm|50_aa|-|63408|63560 >NVVL01000003.1 Rickettsiales bacterium isolate NORP64 Contig_source1382A_1001, whole genome shotgun sequence
MRAQIKRKSVIIIAALLQNSLLQMGENAIIKRLLCHIVTGIQGFLFLHLQ

>gene_221|GeneMark.hmm|771_aa|-|63544|65859 >NVVL01000003.1 Rickettsiales bacterium isolate NORP64 Contig_source1382A_1001, whole genome shotgun sequence
MSNIIQMVPNALPQRDQLQTFFYVLFGYSEGLIPCRSFPESGTDNSQRPHNIWIDADELV
MDKAVTFAKWTAREKVAFYVIPGIVSKQGQAGSDDIQQMQVLLIDIDSGDTEEKLKTLTS
AIGQPTMIVESGGITPEGSAKLHIYWQLERPAAGDDLQRLIKLRYRIALAAGGDTHFKSA
HQPIRVAGSIYHKKGACKLVKIRSYEPLEYNLEELIKSAEKLPKCKEEDSSNPLDYNNTT
PVFKDLLISKVYEGGESEQSRFSHLQRVTGFWLRRLHEGAITQQQAWEEITGYNAANVVP
AWDEKKLRQLVDGLWRKHVKKHGEAAESGEYPDKPEQKTQESTPVKPAKVASYSLGTFLN
DTSSLPEDIIAPRILTPSGMFVFGGAPKVGKSDFLLSLFTHMAAGKEFIGFKPPRPLKIF
YFQAEIGYHYLRERLQNMQLSDEIIKLAQDNLHITPNTKLLLNEPGIEAVAAQVKSVFQD
ETDIIAIDPIRNVFDGGRDGATENENDAMLFFLQKRIEPLRDMINPNAGIILAHHTKKLS
HKQFDEEPFAAFSGASSLRGYYSSGAILRKSELDDNERELIFELRNGAHIPTKILNKKDG

AWVEEDSLNKRIAYKTNGTTYDMERERRIMLITKLLQSQASKGKFYLMNQFAQKYQNHKD
LGGRRSIYDDCSIAATKGDIKFFNNPKDYNPKISNPEKGFGFMCTEHMKIHISKTVDSDT
GEIVENNEIILPTHYKNEGDGRKAEMKYPEIWDVDSEENNTINEVENAGSN

>gene_222|GeneMark.hmm|262_aa|-|65929|66717 >NVVL01000003.1 Rickettsiales bacterium isolate NORP64 Contig_source1382A_1001, whole genome shotgun sequence
LGVDVMLDYNHRSSKSSAPINDQLNILIDDALVKIRDTQPKRRYLGASQLGVPCSRLLQY
EQSGDGKDPGKGFSGQTLRIFAAGHVFEELAIKWLRDAGFELLTNQEDGSQFGFSCAGGN
LAGHVDGIITRAPKALGLAVPALWEMKSMNNKSWKETVRKGLVVSKPVYAAQVALYQAYM
EESVTGISKNPALFTAINKDTAELYHELIPFDGELAQRMSDKAANIIRATKAGEMLPRIA
SSSEYYECKMCAYQQRCWGDRQ

>gene_223|GeneMark.hmm|179_aa|-|66708|67247 >NVVL01000003.1 Rickettsiales bacterium isolate NORP64 Contig_source1382A_1001, whole genome shotgun sequence
MQFNDFNNAETSNFDLIKKGTIAKVQVKIKPGGYNDDEHGWTGAYVTKNEASDACYLNCE
FTVLNGEHQGRKVWSLIGLYSSKNDNRWGNIGRGFIRAILNSSRGYSNTDESEKASQARK
INSLAELDGIEFIAKIDVEIDRDGNDKNVIKTAIDPSHKAYLASEQRQAGSAVDNAVWG

>gene_224|GeneMark.hmm|299_aa|-|67237|68136 >NVVL01000003.1 Rickettsiales bacterium isolate NORP64 Contig_source1382A_1001, whole genome shotgun sequence
MNKLPIISAGERLSETRGIKGCILGKAGIGKTSLLWTLPVESTLFLDLEAGDLAVAGWGG
DAIRLRTWSECRDFAVYLGGGNPALRDDQPYSKAHYDAVCKRFGSASSIEKYETIFIDSI
TVAGRLCFNWSKNQPQSYSDRTGKEDIRAAYGLHGREMISWITHLQHSRDRNIWFVGILQ
EKLDEFNRKVFSMQIDGSKTELELPGIVDQVITMAEIAGDDERPYRAFVNHTINPYGYPA
KDRSGCLRVLEEAHLGRLMEKIKRAQQDGKNLTYDMAKESVNNLNKQSNKQNKENNYAI

>gene_225|GeneMark.hmm|171_aa|-|68129|68644 >NVVL01000003.1 Rickettsiales bacterium isolate NORP64 Contig_source1382A_1001, whole genome shotgun sequence
MDDINYRNDDIAKMTTKEVLHIPIGTVSNYSASLLYDLHKEAVAEFEDARKTRQWIESAI
GMKYEEKVRAKRVRLEKDSGIVHIEDDEFQITNDAPKKVEWDQRKLRGIMAGLIANGVDL
DELVETVYKIPERRFAECSSILQGSFAAARRVRLGKESYKLSRLETGGDHE

>gene_226|GeneMark.hmm|86_aa|+|68904|69164 >NVVL01000003.1 Rickettsiales bacterium isolate NORP64 Contig_source1382A_1001, whole genome shotgun sequence
LNEKTMKLVTWMKLSNLTQGQLAKLINASPQRIHKYLYGGAIPKEKAIHEIYIKTLGSVR
PEDFYKETSDIFEQELLKKQAIDGEA

>gene_227|GeneMark.hmm|117_aa|+|69169|69522 >NVVL01000003.1 Rickettsiales bacterium isolate NORP64 Contig_source1382A_1001, whole genome shotgun sequence
MNKKPTILTIDLGTQTGWAVQAQTGKISSGTESFHRTRFEGGGMLFLKFKRFLTDLKNRY
AGLDYVYFEEVRRHLGTDAAHIYGGFLGQLTAWCEHHNIAHPCWNHQEAHSWQRQCL

>gene_228|GeneMark.hmm|105_aa|+|69655|69972 >NVVL01000003.1 Rickettsiales bacterium isolate NORP64 Contig_source1382A_1001, whole genome shotgun sequence
MITTPSKPADIKQLNTAIDQLNEFLLSKDYITLLQQQYLSPETVAKIMEVKTSLLSNWRV
TGGGPSYIKLGSGSRGLVRYPLLGQAGLIHYMQQKLQNSSSDDKI

>gene_229|GeneMark.hmm|399_aa|+|70047|71246 >NVVL01000003.1 Rickettsiales bacterium isolate NORP64 Contig_source1382A_1001, whole genome shotgun sequence
MIKTDMKNIKASNKFKITKKLLDDLPSSNKRYYIWDSELPNFGVAVQKTGYKSYFLDYTT
QDQKRRRIKIAIVGEITPDEARKKAYKHVLLISQGSDPLDGKQLKNDEPTFKDVAEEYIE
KHAIPFKKATSIAGDREMLRKHILPFFEKISIKEVKRADIKEFHYQLREKKYTANRCVQL
LSKMMNLCEEWGYRPDGSNPCKGLKKYKEDAKERFLTLEEIKRLQLALDRFHESNQxPYF
PPLIRLLLLTGxRLSEILKCKWEYINFERKVIELPDSKTGKKEIILCDRSMTILNNIKHI
PNNPYIIVSSKKEGTHFNNPRKSWAKLLRIAGLEGLRLHDLRHTHASIALMQNIPLEVIG
KRLGHKTIQTTMRYAHLADSHLRKATDIVSNALGQAMGG

>gene_230|GeneMark.hmm|51_aa|-|71380|71535 >NVVL01000003.1 Rickettsiales bacterium isolate NORP64 Contig_source1382A_1001, whole genome shotgun sequence
MGSNTESKAEGRGVGLALCKAVIEAHGGEISAESRPVGALFRVCLPYGKGV

>gene_231|GeneMark.hmm|165_aa|-|220|717 >NVVL01000004.1 Rickettsiales bacterium isolate NORP64 Contig_source1382A_1352, whole genome shotgun sequence
MEIPTLKELQIEVTNNPAADIAQREKFVPCRSRKSTPDGGGYGTGRRKNSIARVWVKPGK
GIVTVNKKLCAEYFSRESYVTSIMQPFLDTNTNGQFDVVCTVKGGGSTGQAGAILNGIAR
ALDAISEDYHSVLRKNGFLTRDSRIVERKKYGRRKARKKAQFSKR

>gene_232|GeneMark.hmm|155_aa|-|724|1191 >NVVL01000004.1 Rickettsiales bacterium isolate NORP64 Contig_source1382A_1352, whole genome shotgun sequence
VKTYSAKPSEIEKNWLVIDAKDLVLGRLASQVAILLRGKHKPMFTPHMDCGDNVVIINAQ
HIHLSGKKGDRKDGKFYYRHTGHPGGIKETTAGKVLAGKYPERVVEMAVQRMISRNKMGR
KQFGNLHVYAGSEHPHTAQQPQVFDVADRNSKNKK

>gene_233|GeneMark.hmm|437_aa|-|1287|2600 >NVVL01000004.1 Rickettsiales bacterium isolate NORP64 Contig_source1382A_1352, whole genome shotgun sequence
MNNISLKKSILLLSLANLFFFFQFILRLSAGVFREDIMQRFAVDVASFGTLAGYYYLGYA
GAQIPLGIMLDRLSFRFVTSLAIAITSIGTLLFATAMDWGMLVFARFLIGIGSGVAFISI
VKIIKTCFDPKHTSMLIGFSFTCGLLGAVFGVIPMRILFDNLGYEDTFYALAIFGFFIAA
IMFIFAKTGNEDRAGNEDEKNNSSVRDSLKSVMSLMLNPTILIIGISGGLMVGALEGFAD
VWALSFFHHVFAMSRAESAAVTSSVYIGMCVGGPLLAMFANILRSTNLMIFITGMLTIIV
FMILFYVPSLSVYSATILMFFLGILCCYQALVFITTTKVVSESSAALAIAVVQCINMSFG
HFFHSVIANIFVKSWDGMIGESGGAIYTLENYIYALAIIPIGCGVGQFGFVYLSWRAKKR
GSESAVSSQLCNKMSRS

>gene_234|GeneMark.hmm|373_aa|-|2816|3937 >NVVL01000004.1 Rickettsiales bacterium isolate NORP64 Contig_source1382A_1352, whole genome shotgun sequence
MSKQEISDYIRDAGFFKDLSKDQLAIITDISEIESYANSEVVFKEGEKSRDLFLIISGGV
DLYKLSQHKYVFFTSLDTNNFIGEMAFVDGSPRSAKVISNNDTKLMKISYAFLEKNHPKL
LNILMPKIARIIMERLRKSTSTHVQTIEKNLQQFKIQNEFGYFFIAIIMAFATSAFMNKI
LEHFNIYEPGYSTLLYSWAYLITVSAPFLALLCIFKYKLSTLGVTSKNLRVSLVESVIVG
SIAILLLFGTFSVLGIEVDILGDVDYRENVSSTFFTLKLITYMIHSVLQEFVARGLLQHS
IQRFLRDRRGIKSVLITSTLFGVGHLHITISVAAMTFAVSVLLGLLFLRHKNIWGISLVH
FAMGVVGIVSGVI

>gene_235|GeneMark.hmm|825_aa|-|4040|6517 >NVVL01000004.1 Rickettsiales bacterium isolate NORP64 Contig_source1382A_1352, whole genome shotgun sequence
MNLSSFITNIPSNALSVEYDLLFVLAAYFVSICFSYVMLDIADIVQKNGDENKNSWFFFG
AITMGIGTWSMHFIWHKSIQIHMSFEVFHAVESLIFTIFASLITLFILRVSNNKLRYIFA
GISVGITIIMTHFICMEGMHQEMNIVYLPNLVILSVMISSLGTMAALWMMQDNIKIGWQF
IVKVLGALLMGGSICVAYYVGTEATIFMPMHNNMHPMEGSQAQAWMTPAIITLTLIIMGI
FIALSMYKLVLATSVLLKNEEIARRKEANLRLEIKVKERTAQVEVALEKAKLANRAKSTF
VANMSHELRTPLNAIIGLSELLVEELEEAKDKEYLEPMRRINSAGRHLLGLISDILDLSK
IEAGKVELFIEEFSIKDILEEVRVIEDPIAKKNKDKLIFDYSKGDIVIKNDITKVKQILV
NLISNACKFTTDGNVTLTVLHDKKSDRIQFIVKDTGIGVTKEQKAKIFSAFSQADSSTTK
KFGGTGLGLSITKKICELMGGTISIGGESGKGTVFTVDLPVSIEGVITKGTNEGANAEVN
VDAHISKIKSHQSKDLKILVIEDNDVERDLVSRYLVSAGYKVSIANNGEDGLKLASSPPM
PDVIILDILLPGMNGWHVLKTLKSNPDTWGINVIVTTVLEEKNKGYTMGVSDYIVKPIDK
KQLLGAISRYVLTNKLSNDDLGKVLIVDDDPDARNILKNMLTKYKVKFDEAVDGKKALES
IKKSKPDLIILDLIMPVMDGFAVVDTLKTSKKWSDIPIIINTSKRLDEEDRKKLDGGVIN
ILQKTKQDKDSMFKLIVELVRNHKDHKDHKDHGGNKGSKDHKGNK

>gene_236|GeneMark.hmm|527_aa|+|6784|8367 >NVVL01000004.1 Rickettsiales bacterium isolate NORP64 Contig_source1382A_1352, whole genome shotgun sequence
LPTGLALLIVNIHRDDFIMSEEVSSSISLFSKIRHSKARQMLWPIRSYELKRFLPMAFLM
LFILLNQNLVRVVKDGFVVTMIGTEVLSFIKLWVEMPIGVLFVVMYTRLCNFMTTEQIFR
IIVSIFLGFFLLFAFVIFPNKELFHPDPLIVQHYIEMFPHMKWMIVMWGKWSFVLFYVMG
ELWPMIVFSLFFWQLANKITNTQDAKRFYIFFGLIGQTNMMISGTIIVYFTQSQHFLLPF
FANITDSSEIILKSLTILVMISGVICLMLHRYIEARNINTLKGIEFKNERTDLLKLSLKE
SVKMVFTSKYLAIICILLVSYSTSVNLIEGLWMSRVKALHPETKDFIAYQGNVLYWTGIS
TLIFIFIGSLVIRRFGWFWGAVITPSMIFIAGSMFFTFVLLEDQLDIIFAGLTSVAPLGI
IVFIGGLQNVLGKGAKYSLFDATKEMVYIPLDKEMKTKGKAAVDIIGAKLGKSIGATVQM
LTFTIFPAANHEDIAGFLMVVFVIVCMMWIYGVKLLNREYHQLLSRA

>gene_237|GeneMark.hmm|313_aa|+|8415|9356 >NVVL01000004.1 Rickettsiales bacterium isolate NORP64 Contig_source1382A_1352, whole genome shotgun sequence
MTKHWSFQMALDLFNQPFMQLLYEAQTIHRANFDSNKLQISTLLSIKTGSCPENCSYCPQ
SAHYNTGLKKEPLINIKEVIESAKRAKEAGSTRFCMGAAWRGPHDRDLALVCDMIKEVKN
LELETCVTLGLLKEHQATMLKEAGLDFYNHNIDSSKEFYSQIITTRCFEDRIKTLDHVRN
AGIKVCCGGIIGMGETNEDRIKMLIMLANFKDPPESVPINKLIKIPGTPLENVEDVDPFD
FIRTIAMARILMPKSYVRIAAGRENMSDEMQALCFFAGANSVFYGEKLLTAENPVPEKDH
LLFERLGMSKLPA

>gene_238|GeneMark.hmm|481_aa|+|9559|11004 >NVVL01000004.1 Rickettsiales bacterium isolate NORP64 Contig_source1382A_1352, whole genome shotgun sequence
MSIVPITVAYGDGIGSEIMSAVMYILKESEAKIRVETIEIGEKLYKKNYSSGIANDTWES
LERTRVLLKAPITTPQGGGYKSLNVTLRKALGLYANVRPAMSYHPFVNTLHPKLDVVVVR
ENEEDLYAGIEYRHTRNMRESVKLISRSGCEKIIRYAFEYAIKNNRHKVTGFTKDNIMKF
SEGIFHQVFDEIAKEYPTIENEHYIVDIGTARLATKPEIFDVVVTSNLFGDIISDITAEI
SGSVGLAGSANIGKDYAMFEAIHGSAPDIAGQNIANPSGLLNGAIMMLVHIGQGEIASNI
ENAWKKTLEDGIHTVDIYDEKTSSKKVGTKEFAEEVVSRFGQKPSKLKIATYSNAPAASS

QEFSYNIDMSEEKKLVGVDIFVDIHANSADEVASKMGVIDSGSLNLKTISCKGLKLWPRD
DEYLTESDHWCCRFMPESGAITHTQIAALMTKLSEANIDFIKSENLYEFDGNRGYSLAQG
E

>gene_239|GeneMark.hmm|46_aa|-|11071|11211 >NVVL01000004.1 Rickettsiales bacterium isolate NORP64 Contig_source1382A_1352, whole genome shotgun sequence
LGALVFIEFDDSADCWGYMKNRYNQLRFLMRFSLEATLADQKYGVF

>gene_240|GeneMark.hmm|666_aa|-|11227|13227 >NVVL01000004.1 Rickettsiales bacterium isolate NORP64 Contig_source1382A_1352, whole genome shotgun sequence
MSRQVHQLRGRSGMPLHYFFSPQEIVKIWFSGNPEEQKTIKECLFSKKDTSNIGHYRDQE
EAKARGIFAQQCIAQSRVVSSQPNVVSSKIEKVTTNLFRFLYVDDEVPLEEDREFLEKRL
SVFTQIYGRSVGADKTQYAKVINGLIKLLLIHPRNNNKDFVDLLVKGAKCLVDIDKLMLI
SMLNELATLYLTQLDHAGAVVFAKVAYTLSGDFDVTIVKGNGDVIDVTIDKGNAAYNFAL
ALKYIKPHKSLKLLKEALIFLPEDADVKNEIAIVTQILQGNEEVLFSMPVNVVDNIEGQG
FWQKALYAVYDLMQNKDSSNRFILYNLTVILKILYKNEDWEQAQKFIEIYLVNLPNPAED
HSFIILLYYEYLVYEMNGESDSASAIMERMSSFPKIAESVADRANILHGVKSESGHDYVS
ALEYFEKIKHAATKNCYVAACEVKRGEYSEELTQEYHKKQQECYEQQRAAVLAESILGTT
IQEESPSWLVGEASYHASQDNVILIETACHLPDYYAVISPKLELDEVSYAQYLSALQGGV
VTHSNGQNGIKTLRKKVLEIKIAADSRLVTSCKYQNDAEEVLVVFDHICKNHEALQRYIN
KSKGITTQMVPSAIPLDLDSLDLGLDFRGELPRPDEDELKMPPPAVVQENPEDAVVVAIG
DSEQPT

>gene_241|GeneMark.hmm|312_aa|-|13451|14389 >NVVL01000004.1 Rickettsiales bacterium isolate NORP64 Contig_source1382A_1352, whole genome shotgun sequence
MGMLIDGKWTDQNQSTSADGKFVRKQSSFRDFIGSKRFPAEANRYHLYISHACPWAHRAV
VFRKLKGLESIISLSVVNSFMGENGWELGEGSDPINGKKYMHEIYTLADPSYTGRVSVPV
LWDKQEGTIVNNESSEIMRMFGSEFDAITKNSKDYYPENLRAEIDEVNEFVYENINNGVY
KTGFATAQLVYETEVAKLFAALDVLEKRLSQHRYLVGNVLTEADWRLFTTLLRFDLVYFG
HFKCNIRRIADYPNLSNHLRDLYQTDGVSDTVNIEHIKTHYYTSHIAINPMGIVPAGPEF
NCSTPHDRKRFG

>gene_242|GeneMark.hmm|356_aa|-|14418|15488 >NVVL01000004.1 Rickettsiales bacterium isolate NORP64 Contig_source1382A_1352, whole genome shotgun sequence
MQTDIAIIGSGPVGIFAAFQAGLLGMKSCIIDALDSPGGQCSALYPEKPIFDIPAHPEIL
AQGLVDNLVEQAAQFDPVYLLSRQVTSMQVAGMRGAGMSDAGVHDAGMHDAGGPDADGCF
ELETSKGDHVRAKAVIIAAGAGCFGYNRPPLADIKSYEDKSVFYFVDKRDKFASRKILIA
GGGDSALDWAISLASIAEVSLVHRRAKFRAAPSSVAKIHELADAGKLELITGFQLEGLSG
KDGVLERVTLADLDGNKKHVDADILLPFFGLSQDLGPLLEMGLDIKGHNIKANYPHFETN
IPGVYAVGDVATYEGKLKLILSGFSEAASALHHAYARVFDGKALHFEYSTSKVGKK

>gene_243|GeneMark.hmm|245_aa|-|15880|16617 >NVVL01000004.1 Rickettsiales bacterium isolate NORP64 Contig_source1382A_1352, whole genome shotgun sequence
MRVLLIEDDSSTARSIELALAAEGVVVDRAEVGTDGFELARLYEYCAVILDLMLPDIDGY
EVLLRLRSAKIKTPVLILSGLSSVDQKIKGLGFGADDYLTKPFNKDELIARIQAIVRRSK
GHSESVVRFDKVAINLDTRVVEVDGNQVHLTNKEYAILELLAMRKGTVLTKETFLNHLYS
NLDEPEIKIIDVFVCKLRKKLSNASGGVNYIETVWGRGYMLKDYDAPTSNLLAEEYAKTD

SPAKA

>gene_244|GeneMark.hmm|152_aa|-|16814|17272 >NVVL01000004.1 Rickettsiales bacterium isolate NORP64 Contig_source1382A_1352, whole genome shotgun sequence
MSTQAPNNEDMPHIAVNAQYIKDFSFENPGAPGTLSGFEEAPQIDLALDLNIQKMPEDNY
YEVEIAINAKAVIKKEVLFIVDLKYAGVFTLINIPEEQLEMLLAVHCPAIIFPYARKIIA
DATQDGGFQPLMIDPVDFGVLYNKKMTEGRES

>gene_245|GeneMark.hmm|188_aa|-|17324|17890 >NVVL01000004.1 Rickettsiales bacterium isolate NORP64 Contig_source1382A_1352, whole genome shotgun sequence
MSIMSDKWIREKSLKEAMISPFVGEQVKVAESGKIISYGLSSYGYDARVAREFKIFTNIN
TAIVDPKNFDNDSLVTKETDVCIIPPNSFALAHTVEYFKIPSDVLVICVGKSTYARCGII
VNVTPLEPGWEGHVTLEFSNTTPLPAKIYAGEGACQFLFLKGNEQCSVSYADRAGKYMGQ
KGVTLPRT

>gene_246|GeneMark.hmm|434_aa|+|18172|19476 >NVVL01000004.1 Rickettsiales bacterium isolate NORP64 Contig_source1382A_1352, whole genome shotgun sequence
MKNSKKLLLGTATALLSFSAGAMEMEKPSGTPVVSDLTVKISAFSHFQTGMRKQKNLTEK
EKNVSAKRKTFAFNSESAMSLEVSNYVNDFKYGGKIVLVPTNKRKGGSFNGSHIFTETNY
GRFEAGSPLPASYKMQVDGHDIAAAPATNWSRYVDFSTEDMMQKQSIEPSFAVVSDFFLD
SKLATKLDNRNYSSEPARSISYYTPKFALGQSSKVQVGVSYAPDSSNTGAEKHTKVSSGL
SIRNINKDGTDRFEIDSSVRDAVSAGITLEQNFSDGIDLKVGLTGEYANASGKATKFEND
VEKESYKLSPLRTFNIGAILNVGSMSYAGSFGSLGKSLTTPEFHKTGRSTKYYTGTVAYK
QGPFAASVSYFKSNQYENTVDAISIGTHYVMAPGFKPYVQISTFRLKGKPEFYPKLEKKK
TRGTVALIGVKLSL

>gene_247|GeneMark.hmm|409_aa|-|19796|21025 >NVVL01000004.1 Rickettsiales bacterium isolate NORP64 Contig_source1382A_1352, whole genome shotgun sequence
VKNYLSISEVKVGLNFDKKIIPVGRLVAQKHKVYFEYATSFIEKGMEISPLKLPLQAGVS
VFDRNLFEGLPGVFNDSLPDGWGRLLFDRFMRTQSVLPEELTVLDRLAHTGNHGMGALVY
EPDHSPEKTGDIINLNVLAAEAQEVLAGSAGDVLQNLLALNGSSAGARPKALIGVDASRE
NISHGFCQLQEGQEDWLVKFANSQDGNDSGAIEYVYALMALEAGVDMPDVHLFPGDKNAG
YFAVKRFDREGTKRIHMHTACGLLHSDFRTPSLDYRDLIALTGMLTRDACQVEKMFRLAV
FNVLAHNRDDHAKNFSFLMKESGDWKLSPAYDLTFSAGLWGEQSTMVVGEGKNPNIDHLI
KLGLEAKLSQRNIDSIIEQTKTALAQWKNLAKQYGVSQLNINLITTKIS

>gene_248|GeneMark.hmm|102_aa|-|21022|21330 >NVVL01000004.1 Rickettsiales bacterium isolate NORP64 Contig_source1382A_1352, whole genome shotgun sequence
MVSLITPFEAQKKLAEAVQARRLGMELTQEGLAARSGVALPTLRKFEQKGLISLESFLKI
LMVLGGLDDVVQAITPEKTAFSSIDDVLKANDKPTRQRGRRK

>gene_249|GeneMark.hmm|1376_aa|-|21656|25786 >NVVL01000004.1 Rickettsiales bacterium isolate NORP64 Contig_source1382A_1352, whole genome shotgun sequence
MSQRNFYSQLGNTQDFDQIRINIASPEQIRSWSFGEITKPETINYRTFKPEKNGLFCSRI
FGPVKDFECLCGKYKRMKYRGITCEKCGVEVTTASVRRERMGHIELAAPVAHIWFLKALP
SRISTLLDITMKDLEKILYFENYVVVEPGMSPLQKGDLLSEDGFVKAQDEYGEDNFVAMI
GAEVVRRMLSELDLEELSAQLKAELAETSSETKKKKIIKRLKLVENFLTSGNKPEWMVMT

VLPVIPPEIRPLVMLDGGRFATSDLNELYRRVINRNNRLKRLLELKAPDIIIRNEKRMLQ
ESVDALFDNGRRGKVVKNANKRPFKSLSDMLKGKQGRFRQNLLGKRVDYSGRSVIVVGPE
MKLHQCGLPKKMALELFKPFIYSKLELYGIATTIKAAKKIVEAEKVEVWDILEEVIREHP
VLLNRAPTLHRLGIQAFEPLLIEGKAIQLHPLVCAAFNADFDGDTMSVHIPLSIEAQLEA
RVLMMSTNNILSPANGKPIISADKDVVLGLYYLTQMLDGDIGDGMAFNDIKEIEFALFEK
VVTLHTKIKYRIEMLNADGEFVETLVDTTPGRVIMGQLLPKSKELDFKLLNKEMNKKNIS
SAIDSIYRYCGQKATVIFADKIMELGFKHACQSGISFGMDDMVVPSAKTKIIKDASAVVG
EFEKQYLEGLITYGEKFNKVVDAWTACTDKVAASMMEEISRPSEGKKTKQEEFNSIHMMA
VSGARGSPAQIRQLAGMRGLMTKPSGEIIETPIKSNFKEGLTVREYFNSTHGARKGLADT
ALKTANSGYLTRRLVDVAQDCIITDDDCGTEKGIEIKTIIDGGEVIVSLSDQILGRTVSA
DTHHPVTGELLVAACGLIDEEKLDTIESSGLDLIHVRSVLTCETKFGICARCYGRDLASG
TLVSKGEAVGVIAAQSIGEPGTQLTMRTFHIGGAATKGTEVSSVSASYEAKVKVIGRNLV
TNSVGAQIVMGRSCEVLLLDVKGAERARYKIPYGSKLMIDDGCKVEKGQMIAEWDPYSIP
VITEKSGKVLFVDLVEGVSIRTVVDDATGISSKVVIESRQYSKGAELAPRIQLVDAKGKS
VEVLEGVEVKHYLSVGAILSVEDGTEVAAGDILARITRESSKTRDITGGLPRVAELVEAR
RPKDHAVIAKLDGRIEFGKDYKSKRRVVLHPADGSDVVEYMLPKGRHVVVSEGDFVKRGD
MIINGNPVLQDILKVSGVEALAHYMVSEIQAVYRLQGVKIDDKHIEIIIKQMLQKVEVTH
SGSTTLLPEELIDLRTLNEINDKIVKEGGEPARYEPVLQGITKASLQTKSFISAASFQET
TKVLTEASVAGKVDTLKGLKENVIVGRLIPAGTGFYMSMAKKEAARKDAIIEKSKA

>gene_250|GeneMark.hmm|1392_aa|-|25902|30080 >NVVL01000004.1 Rickettsiales bacterium isolate NORP64 Contig_source1382A_1352, whole genome shotgun sequence
MHRCYDEIHSQYTSVIDIEISGDLMPQPLQTSKRIRKNFGKIKLVASIPNLIEIQKKSYD
AGFLQIDSPGDKRENKGLHSVLASIFPVQDQSNTATLEFVKYSFDAPKYDVEECIQRGVS
FVAPLKVTLRLSVWDVDETTGAREIKGIKEQEVYMGDIPFMTKTGTFVINGTERVVVSQM
HRSPGGFFYHDDGKTHSSGKFLYSARIIPYRGSWLDIEFDAKDLMYIRIDRKRKLPVTTL
LMAIGMGSKEILEHYYDTETYDYGKKGWVVDFDPARVLSHRLVNDLVDADTSEQLLEAGQ
KITPRLAKKFAENGLKKVLCQDDLLIGSFVGKEMVDAASGEVLAELGEQITEELLGKLLS
GGAKSVSVLKVANQGDAYIRNTLFADKNQSQDAALVDIFRVLRPGEPATVEAGQALFESL
FFDPNRYDLSEVGRIKINSRLNIDIPLENTCLTKEDIKSIIRVLIDLKDGKNSVDDIDHL
GNRRVRSVGELVENQFRIGLVRMEKSILERMGAVDIDTVMPHDLVNSKSLALVVKEFFNT
SQLSQFMDQTNPLSEVTHKRRMSALGPGGVSRDRVGFEVRDVHPTHYGRVCPIETPEGQN
IGLINSMAVYARVNKYGFIESPYRRVVDGFVTDEVIYLSAIDEGKYKIAQADMEIGAKGK
IMADMVSCRFETGNFVSVVPAEIDFVDVTPMQIVSVAASLIPFLEHDDANRALMGSNMQR
QAVPLIGAEAPFVGTGMESIVARDSGAAIMALNDGVVIEVDATRVVVQTVKPKEGGAPEV
DIYTLMKFRKSNHNTCINQKPLVSVGQKVSRGEIIVDGPAIDQGEIALGRNTLVAFIPWR
GYNFEDAILISERIVKDDVFTSIHIEEFEIVARDTRLGPEEITRDMPNVGEESMRNLDEV
GIVHVGAEVKPGDILVGKVTPKSESPVTPEEKLLRAIFGEKASDVRDTSLYVPPGASGTV
VEVRVLSRRGVEKDERAVAIEKLQIEKLAKDRDDESGIIDHFVFMRMEKVLEGEVVITGP
KGVKPGSVIDSDLLSGLSRGQYWQITVENNSVMDEIEQIKQQYDEKKDQINRHFNTKVER
LQSGDDLPQGALKVVKVFIATKHKLQPGDKMAGRHGNKGVISRIMPEEDMPFLEDGTVLD
VVLNPLGLPSRMNVGQILETHLGWAAKNLGKNVGEMLEKYSNNDIDIDSMKDFVSKIYKG
SDVAKQVKDLSKEDFIEFCGTMKDGVYFSTPVFDGAKVEGVKSMLELAGQNSSGQVRVMD
GRTGEYFDRSITVGYKYILKLHHLVSNKIHARSIGPYSLVTQQPLGGKSHFGGQRFGEME
VWALEAYGAAFTLQELLTVKSDDVSGRIKIYENIVKGDSNFTSGVPESFNVMIKEFRSLC
LNVQLEKGSKSE

>gene_251|GeneMark.hmm|123_aa|-|30387|30758 >NVVL01000004.1 Rickettsiales bacterium isolate NORP64 Contig_source1382A_1352, whole genome shotgun sequence
MADITKIAEDLSSLTVMEAADLSKMLEEKWGVSAAAPVAAAAAPAAGGAAAEEQTEFDVI
LASIGDKKIEVIKAVRVITALGLKEAKGLVDGAPSPIKQAVSKSEADEIKAKLEAAGATA
EVK

>gene_252|GeneMark.hmm|169_aa|-|30791|31300 >NVVL01000004.1 Rickettsiales bacterium isolate NORP64 Contig_source1382A_1352, whole genome shotgun sequence
VLRSNKKNCVTELEEIYKISNSVIVTHYHGLTVTEVTKLRRSLRENGASFKVVKNTLSKI
AALNSKFAGDPEMFAGPTAIAYSEDPVAAAKGVVEFAKSNDNLKIIGGVVNDKILDISAI
QQLAKLPSLDALRGKIIGVLQAPATNLARVTQAPASALARVLQAYADKN

>gene_253|GeneMark.hmm|227_aa|-|31315|31998 >NVVL01000004.1 Rickettsiales bacterium isolate NORP64 Contig_source1382A_1352, whole genome shotgun sequence
MGKKIAAVRKEIESGKEYSLKDAISVIKKNSFANFKETLDVAINLNVDPRHSDQMVRGMV
SLPSGSGKDVKVAVICKDDKVAEAKGAGADMAGSVDIIEEIKSGKINFDVCIATPDMMGV
VGQVARILGPKGMMPNPKLGTVTTDVATAVKNAKSGQVEYRVEKAGVIHAGIGKIDFTEG
DLLKNAESFIGSVIKAKPSGVKGIYMKSIFLSSTMGPSLKVDLSTIS

>gene_254|GeneMark.hmm|143_aa|-|32024|32455 >NVVL01000004.1 Rickettsiales bacterium isolate NORP64 Contig_source1382A_1352, whole genome shotgun sequence
MAKKISGYIKLTIPSGKANPAPPVGPALGQKGVNIMEFCKAFNAATSSFEPGTPVPVVIT
VFVDKSFTFITKTPPASFYLKKFAKVKKGAGATKKEAYVGKVTMADCEEIAKIKMEDLNA
NDLAAAAKIIAGSAESMGIEVVK

>gene_255|GeneMark.hmm|176_aa|-|32501|33031 >NVVL01000004.1 Rickettsiales bacterium isolate NORP64 Contig_source1382A_1352, whole genome shotgun sequence
MAKWYILHAVSGAEKSIQKMIEDQVVKKKMSDLFEEVVVPVVEVPEVKRGKKILVEKKFM
PGYILIKMKMTDESWHLVKSVPKITGFLGSKTTPQPLSDAEAEAIFRKVAAESEQASASS
LYNAGDKVQVIDGPFDSFSGTIESVDGEGQKMKISISIFGKATPIELSFSQVKKND

>gene_256|GeneMark.hmm|107_aa|-|33043|33366 >NVVL01000004.1 Rickettsiales bacterium isolate NORP64 Contig_source1382A_1352, whole genome shotgun sequence
LHLREKYDRSVAQLVERRSPKPKVAGSIPVTPATLLIDVGCMFKGLKSYKFFEQVKQETK
KVVWPEKKELTTSALIVVVSVFLFSIVSLIVDYGILNLVQMLLNIGK

>gene_257|GeneMark.hmm|692_aa|-|33424|35502 >NVVL01000004.1 Rickettsiales bacterium isolate NORP64 Contig_source1382A_1352, whole genome shotgun sequence
MSEKKLKLSDVRNIGICAHIDAGKTTTTERILYYTGKSHNIGEVHDGGATMDWMEQEQER
GITITSAATTCFWKEKRINIIDTPGHVDFTIEVERSLRVLDGAITVFDGVAGVEPQSETV
WRQADKYHVPRMCFVNKLDRAGANFYRCVDMIVDRLGANPLVIQLPIGIEDEFLGVVDLV
KMKAITWSSEGLGAEFTESEIPADLKEKAEEYRAKLLDMAAEIDDAIMEKYLDGAELTED
EIKFCIRKGTIAGKFVPITCGSAFKNKGVQPLLDAVIDYLPSPEDIPAIKAIDSKTGEEK
IVKSSEEGEFSGLAFKVMTDPFVGSLTFVRIYSGRITTGSSVINTVKNQKERVGRMLLMH
ANDREDIKEACAGDIVAIAGLKNTTTGDTLCSANAGVVLERMEFPEPVIQLAVEPKSTAD
QERMGFALAKLASEDPSFRVSSSENGQTNIAGMGELHLEILVDRMRREFNVEANVGAPEV
AYRETITQAHEVNYTHKKQSGGAGQFARVKIIFEPGESGSGFEFVSKVVGGSVPKEFIPG

VEKGIKSIMATGVVAGYPMIDFKATLIDGAYHDVDSSVLAFEIAAKAAFREGMPQAGAKL
LEPVMKVEVITPEDYMGDIIGDLNSRRGSVQSMEPRGNAQIIIAHVPLSEMFGYVNTLRS
MSQGRAQYSMEFDKYAHVPTHVVDNILSKQGG

>gene_258|GeneMark.hmm|160_aa|-|35509|35991 >NVVL01000004.1 Rickettsiales bacterium isolate NORP64 Contig_source1382A_1352, whole genome shotgun sequence
MSRRHAAVKRVVKPDTKYKSVLLSKFINNLMKDGKKSIAEKIVYDAMERVEKKHGANPLE
TFNESINNVKPYVEVSSVRVGGANYQVPSPVDERRGYALAIRWLVDAAQKRSGRSMVEKL
AEELFDAANSRGVAIKKREDTHKMAEANKAFAHFAQRQQR

>gene_259|GeneMark.hmm|123_aa|-|36027|36398 >NVVL01000004.1 Rickettsiales bacterium isolate NORP64 Contig_source1382A_1352, whole genome shotgun sequence
MPTNNQLVRFGRKSKVRKTNSPALRSNPFLKGVCVIVKTVTPKKPNSALRKIARVRLSNG
EYVNAYIPGEGHNLQEHSAVLVRGGRVPDLPGVKYHIVRGALDTQGVKGRKQARSRYGVS
AKA

>gene_260|GeneMark.hmm|214_aa|+|36658|37302 >NVVL01000004.1 Rickettsiales bacterium isolate NORP64 Contig_source1382A_1352, whole genome shotgun sequence
MYEIFYSLFGYNKQLFLTINHYTNIGALPQVLQAISSVFFIANFAIGYIAACVYVYYKSK
KAPNQEAYFTPIYNELVRLGACYAMFGFVFAGLKFSVNLQRPFCSLPPADFITIADTSLE
RCLSSFPSAHTGLAILVAYCFWRHMNPALKILACLVAMAVATSRVTLAMHYPADIIYSII
ATLISILIGNNIYALLKNLVVRPIGKMIARLLFN

>gene_261|GeneMark.hmm|281_aa|-|37334|38179 >NVVL01000004.1 Rickettsiales bacterium isolate NORP64 Contig_source1382A_1352, whole genome shotgun sequence
MGLIAGLYIVSTPIGNLGDISLRALETLKSSDVILCEDTRVSGKLLAKHGIKASLKVYND
NSDNSLRRTIKGYIAKGMAVSLISDAGTPLISDPGYKLVRELKADGHHIDAIPGACAAIT
ALTLSGIATDRFLFAGFLPKTKEKKKKAFEEFADLNATIVFYETANRIVSSLEVAIEVFG
DREANIGRELTKLYQESRSEKLSVLLEYYKNNPPKGEIVFTISPKSEDELSIDDIKDELS
AKLSRGESARTASDEVFSKYKGRISRKEVYKLANQLKNEVK

>gene_262|GeneMark.hmm|380_aa|+|38205|39347 >NVVL01000004.1 Rickettsiales bacterium isolate NORP64 Contig_source1382A_1352, whole genome shotgun sequence
MHNIKQKFLKYIFSFVCFAFLGSCSQTGDYFKAGNAADIAILLPMSGVEAEMGMEYAKMI
KMGIKDSAASKIKVTIYDSANQEALEDSLDKILNRGTDIIIGPIYSGPTKIVAEKAGSKG
IITISFSNNPTLAEQNLFIYGHAPMRQLEQITNYFLDNGHKNYIALLPSGKHSNVVSKIL
REMIIDGDGILSRLELYGASPEDIKSSVKIVSETASQLNESDLNLTQPVILIADDPVTLE
MVYANIRKYGLDKKAIIAGDSRINIDSPFNTNISFTGTLGVANEKLAARARKAGVRHISF
MHALAYDAGKMTGKYIGKRYNKEEFLTRMNSKEEFIGIAGRIHFIDSIAQRKYDVIRKEN
GIYVGDVKRGYGRSSPVHSR

>gene_263|GeneMark.hmm|419_aa|+|39598|40857 >NVVL01000004.1 Rickettsiales bacterium isolate NORP64 Contig_source1382A_1352, whole genome shotgun sequence
MRKIVISGMIGNALEWYDYALYAQFSYIIASKFFPPSEFTDILTFAVFACGFVVRPIGGI
LFGHIGDRYGRKMALVLGILLMAIPTAGIGLLPSYDSIGIAAPIILVLIRLVQGFSLGGE
FSGCIAYIVEHSPADKRGLAGSASFVSMCLGMLLGLIVANVFTYVLSEEDLLSWGWRVPF
VAGLFIGLVGLYIRSHLSESPIYKAAKAQGALSRTPLRETLTKYWREVLIAITIYINVTA

PFYTTTVFIKNYMQTLGYANEQGSLVCALILITLIITFPISAHISDKIGRKPVIVWASVA
LVIMIYPIFLALHSMNYVIAILSQVLFAAIVGVYMGPVPTLLVELFPTRVRFTGVAISYN
LSAAIFGGTAPMVGAALYQFTGEQISLAYYLTGLAFFCLCTLYFYKETYRNDLAGDYNT

>gene_264|GeneMark.hmm|224_aa|+|40854|41528 >NVVL01000004.1 Rickettsiales bacterium isolate NORP64 Contig_source1382A_1352, whole genome shotgun sequence
MNDNIFEVPEGENKVLMHSCCAPCSGPLIEKIVNDGIDLTIFFYNPNIHPKKEYEIRKQE
NIRFAEKLGLAFVDADYDVQNWFSRAKGMERDPERGRRCTMCFDMRFERTALYAKENDFK
IFTSSLGISRWKNMEQINECGVRAASHYPGTTYWTFNWRKNGGSARMYELAKEEEFYKQE
YCGCIYSLRDTNDWRVKNDRSEIAIGEEFYREKLQNTEKTSQNS

>gene_265|GeneMark.hmm|333_aa|+|41530|42531 >NVVL01000004.1 Rickettsiales bacterium isolate NORP64 Contig_source1382A_1352, whole genome shotgun sequence
MSDSHDFPTTDKPHKMNNSSSSDKPPQMSNSSSSDNLRKTSGWLNIYKPRGISSAKAVSL
IKRAFRGSKIGHTGTLDLEAEGVLPIAIGEATKLVSILIDAKKRYNFTVKFGAKTDTADS
AGKVIETSNHIPSEEECHKICSQFIGKVEQIPPAYSALKVNGQRAYKLARAGEEVKLAKR
CITIYDLKCTSYDHEAQTASYVCDCSKGTYIRTLAEDISLSLQSLGFVLQLRRLKVGMFD
AASAVDISDYMTIDFAEATAFLQNKCLKIEDVLDDIPVLEASESQTQKIRFGQNCQFEDD
QDYDLVWVRHNSRIVAIGSLLSNNFKSSRVFNL

>gene_266|GeneMark.hmm|91_aa|+|42542|42817 >NVVL01000004.1 Rickettsiales bacterium isolate NORP64 Contig_source1382A_1352, whole genome shotgun sequence
MTMSITQKRKTDLIKEYATVANDTGSVEVQCAVLTERIKNLTDHLKENFKDFSSRRGLLI
LVGRRRSLLSYLKKQSTDRYAVLIAKLGLRK

>gene_267|GeneMark.hmm|141_aa|+|43150|43575 >NVVL01000004.1 Rickettsiales bacterium isolate NORP64 Contig_source1382A_1352, whole genome shotgun sequence
MLSTWLFAAGITVHCLIGCVIGEIIGLSIGVTMQLHPVATMALATILAFITGMLLATTSV
VRNKRTTYMLAIRIVWLGEVVSISVMEIVMNGVDYYIGGVGAQSITDPIFWKGLLFAIPA
GYFAALPVNYYLIKKNLKKCH

>gene_268|GeneMark.hmm|358_aa|-|43723|44799 >NVVL01000004.1 Rickettsiales bacterium isolate NORP64 Contig_source1382A_1352, whole genome shotgun sequence
MKINKPINLRSDTTTPQTQNIIDAMTSTPVEDAAYGEDMATNELSEYCKELFQVEDAVFL
TGGMLANRLAIASQTSPGDEVITHHNYHINFFDSAGNAKINNVVLNCNHNPTGVLSAHDV
EEAINSKPRYHMCSQVKLITIENSINGFNGKIYPFGKQVELYKYTKDRDINLHLDGARIF
NAHIETKIPLAEYAKHTDTMSFCFAKGLGAPFGSMLLGRKEVIEKVRIMQIWLGSGYHQI
GYYANAAKYALQNNIPRLSDDNKNTKILADKIKSLPHIKLILPYPETNILTFSIKELGVS
NDIFLDECKKLGLLLFPWLEGQIRAVVHLAVSHSDIKQAAKIIETVILDTLPLTKVRG

>gene_269|GeneMark.hmm|590_aa|-|44810|46582 >NVVL01000004.1 Rickettsiales bacterium isolate NORP64 Contig_source1382A_1352, whole genome shotgun sequence
VTETRLYNFRKVFAFIIKLLPPFKFNIFVMLVVAMIWAFDLSFRQYIIKNILNGLVGSEG
VGVAEQIWQPACMYIFMALLITTTFRAYGYFVDVRMCPALRQNIAKFAFQKLLTHSHVYY
QSNFAGSLTHKVNNLTDSIIELVKLSIDRFFGCSLALILSIYTLFLVNINFALVTLIWAG
ISTIMSIFYFQKLSDLSRDYSASSASVSANIADSLINITTIRLFHSRFSEQYKLFKVCRK
KLIAERKFNWTFFLIWCIHGYSFNFLQIISLYFLIDGYQAGTITIGDFALVIGLNLEIVD

FLNRLVKELALFSDHYGRVVDALSTIFIDSELQDQIDAKPLKVASGKISFDKISFAYEGK
KPLFDNFSVTINPKQKVGLVGYSGSGKSSFINLILRLFDLDAGKIIIDGQSITEVKQHSL
RQKLAVVPQDLILFHNTLIANIRYGKFDASDEEIIRASRFAGIHEFISGLPCGYDTIVGE
KGIKLSGGERQRVLIARAFIKNAPILLLDEATNQLDSITEQEIQNSLFKLMEDKTTIVIA
HRLSTLLHMDRILVFEQGKIVQDGTHDELIVQGGLYRKLWNAQSNDMIRY

>gene_270|GeneMark.hmm|340_aa|-|46601|47623 >NVVL01000004.1 Rickettsiales bacterium isolate NORP64 Contig_source1382A_1352, whole genome shotgun sequence
LKKMNFLDQDGFVVIKNLIPKEAIESCTYHIKDAINQASAELKVDVADYLHATGRWATKS
PITRIAANIVDQDIEARLAELCSSKIIQKKSNVICKTADLVDAIPFHQDISYSRRDPYHF
SVWLALNDVDKNSGALQVIKNSHTLPIEPAADFWSPYFSDKSNQEKGKAQSIEVSAGDAI
IFDSRLWHGSNKNFAGKDRFAYVTRWSIPGKGYPDIPEIVAEKFGMFNCGHMTDEILQKS
LLLYEKQPPEYELPTKEKLVDKWIDILQKKGDVLNKVGALNEGSSLNIDSSLAIQHLKSL
RVLHLAHDAHDAGNISGEVYKNLWFSLLSPLGKKIQLLKI

>gene_271|GeneMark.hmm|260_aa|-|47620|48402 >NVVL01000004.1 Rickettsiales bacterium isolate NORP64 Contig_source1382A_1352, whole genome shotgun sequence
MQNMQYEKTSSQLNLLEQMQNIKISPCRSFHTLAGSPIYQARFRAVEKFHKPGFAPVYDK
DSDRNNKDSGYHIDFTGKALYKRRFTKVFGFYCDRATVADQNEYFHIDITGSKAHDHSYQ
WAGNYQEDVCVVRQNNQFFHVDMEGREIYSEKYDYAGDFKDDIAVIYRNGQASHINNEGR
LIHDRWYKKLGVFHKGYANAEDNNGWFHINREGKAIYEQRYKMVEPFYNGLAKVETFDGH
LGQIDVMGRMLVNIYTHKID

>gene_272|GeneMark.hmm|755_aa|-|48363|50630 >NVVL01000004.1 Rickettsiales bacterium isolate NORP64 Contig_source1382A_1352, whole genome shotgun sequence
MLSQKLADRHEVQVFARYEDSFLADFHYRTELDSLDPRILLNLVNIPLTKYRYRFVNEQV
DIRFQKILEEFKPDIVHFGHLNHLSLSLPSIAAKLNIPTIYTLHDFWLMCARGRFIQRNS
KQILQPCDCQEDQKCAEQCYSGYFTGDESSAETDLAYWQQWIATRMQHTKGIIEHIDHFI
SPSKFLMNKFITDFDIPQDKLSYLDYGFDLERLSARKRQKDKQFVFGYIGTHTPEKGVDL
LLRAFENITSDAHLRIWGAEREETKALKQISNQLPDHIRDKIEWMGSYKNENIVSDVFNH
LDSIIVPSIWGENSPLVIHEAQQLRIPVITSDHGGMAEYVRDGVNGLLFKHRDESDLTAK
MQKLSGDKNLYQKLTQTGYLYSKDGQIPEIGDHVAAVEKLYISAAKKKGKQISTKPGPWR
ITFDTNPDYCNFACVMCECFSPYSNVKEDKKARGIKPKIMPIATIRKIIEEASNTPLREI
IPSTMGEPLMYKHFDEIINMCHEFGLKMNLTTNGSFVVKGARKWAELLVPILSDIKISWN
GATKETHEKIMKGSKWEDVTRNLQTFLEVRDEYLEKTGKTCSVTLQLTFLESNLMELYDL
VKMAINLGVDRIKGHHLWAHFEEIKDLSMRKNSDSIRRWNKEVNRIYELRDQMLLPSGAK
IKLENFTILENDGVKDLAPGGACPFLGKEAWVNPEGKFSPCCAPDELRESLGVFGNVNDT
KIGDIWQGDKYRDLQKNYLNYDLCKTCNMRKPLVS

>gene_273|GeneMark.hmm|337_aa|-|50605|51618 >NVVL01000004.1 Rickettsiales bacterium isolate NORP64 Contig_source1382A_1352, whole genome shotgun sequence
MKKILITGGAGLIGSALNKELVGQGYGVVSCDIRFNDNPLSFFSQEIKPLLAQCSGIIHL
AAISRVIHGEEHPELCQEVNVDGTIKLLEFYKDLPHKPWFIYGSSREVYGQQETLPVSEN
APLKPVNNYARGKVLIEQQVSGLREFGFNTAILRFSNVYGGMLDHYDRVIPAFCLKALRG
EQIRIEGKECVFDFTHLEDVVGGIALAVGVLQKGAPLESAIHFTGGRGCNLEELANMILK
ITESNSQINYYPARNFDVGRFYGDYSMATKLLGWRPQYSLEEGLHKFIYDIQHHSVNCPY
NVGMRVYENIESYSWLPTILQCGIRNIHSDALAETGR

>gene_274|GeneMark.hmm|904_aa|-|52269|54983 >NVVL01000004.1 Rickettsiales bacterium isolate NORP64 Contig_source1382A_1352, whole genome shotgun sequence
MSTLSFSTEYVHGSYEETKVPSGDACGSNQLPQLEWPLPLTSGLSLSITDQEWSELNLLA
ANQRWEQLKAALEEKGRAEKMFPKWIKVLTILLNRTYLQKDLDEGLNALVAQRDYQQIDI
ELIKQLASMGSDVNYTFRGQIPDSALQIAVRSGNVSLIKTLLESGEERAVVKLNKYNENN
KNEHILYSAIQLGDLELVIYLLQYNILDSCQIACRKDYDKDTLVDCAILSDLEKALQIIP
RLFAAGYSFGSVENLMQHGELDYLDSKILLDLLKWGLSVDDGVKYGMTLFLEDRDEGYDP
KPFRMYAIKMLQFFLGKGGKIPEELSASLDERLQKLVKIAKIADDLYSGNPLSAPLSLED
IELKLKLKEATEALFQVLALPEDTDQTQALDLARALRGQDLGSFTRNIDLTNTGRVLDLD
LAIALRGQDLGSFTRNIDQTQALDLTNTGRALDLRQMEKAVKTIAKTIAKTLALARVLKY
TDETLEYTDETLEYTAKALKYADRALKDADRALKDAYRVLALKDAYRVLALKDAHRALED
AYRALEDVYQALEYTDRAKALKYAAKDLQYAVLALVLKDADQALVIKCVDQALDAALALK
ESDSKLIVPRLLRLWSRDGQSEYPTFDEFINWCEGQSLQLIFGGLPLTLRNAQSDLMFLE
DWIIQKLVTGQTNSMPLIVSLQNCKNSAEPVLDRRIINKYIEIFPTLEPLIEQCIQTGVL
TYSSLSMEQPNDSVGSKRLREEVPHQRVVRRKLNPDDGSLVNQSPVEEMLEILQRGIGSY
LVSNKPEVLATLSMHQEARTLLDKVVNNCLPCNLKTAILDLWCDLQKAIYSPSADTLEWA
GSKIQESQYENAGLKDENARLQGENARLQERLKALLEPQLDDVHMHEAPDLIGSSDTPEA
PDFL

>gene_275|GeneMark.hmm|102_aa|-|55175|55483 >NVVL01000004.1 Rickettsiales bacterium isolate NORP64 Contig_source1382A_1352, whole genome shotgun sequence
MSLKKGRAYKPIVRIEFTRIISDKSTAETRYYITSLPAKALYISKAIRSHGGGENSLHWV
LDVIFNEDQSRIRKGHAPANISAVRHIALNILKSAQKLCWRH

>gene_276|GeneMark.hmm|117_aa|-|55906|56259 >NVVL01000004.1 Rickettsiales bacterium isolate NORP64 Contig_source1382A_1352, whole genome shotgun sequence
MTRAKAGKISKNRHKKIIKMAKGYRGRAKNCFKIAIEKVEKALQYAYRDRRNRKRDFRGL
WIQRINAAVRQHDMIYSTFMGGMKKAGIEVDRKMLAEMAVNDPASFTHIVEQVKKAQ

>gene_277|GeneMark.hmm|66_aa|-|56284|56484 >NVVL01000004.1 Rickettsiales bacterium isolate NORP64 Contig_source1382A_1352, whole genome shotgun sequence
MPKMKTHSGAKKRFKLTASGKVKSAQAGKQHFMRRRTKSQLRNQRGTAVLCEPDAKRLKK
IFLPNG

>gene_278|GeneMark.hmm|391_aa|+|56904|58079 >NVVL01000004.1 Rickettsiales bacterium isolate NORP64 Contig_source1382A_1352, whole genome shotgun sequence
MEELFALKNVYELYTGHRVLVTLFIMLTGFYPMLMIMKKIISPRVDKIIKTRSKNSTRII
EKHRITARLIQVFIAVYLMFWGDILDDLTLMSESLIRIKDSAISIYLVTTISSVLLTAIN
IGVDLHGSRKTFNHTAIELHTHILKIFITACASLSIISVILGISISSLFTSLGAAAALLT
FVFKDTVLGLLASVQLTFQNIIRIGDWVSLPSYGADGDIEKITITVVVIRNFDKTCTTVP
TSAFLNTGVKNWRTMFESGGRRIKRAISLDMDTIKICSQEHLDKFRKMPCMTDFAKNNKH
MFDAKNETTNITMFRYYVEEYLKSHQDIHQEGFTFLVRQLNPTPTGIPLELYVFTKDTAW
ANYEAIQASIFDHLLGGLAKFELRAFQTVIR

>gene_279|GeneMark.hmm|643_aa|-|58182|60113 >NVVL01000004.1 Rickettsiales bacterium isolate NORP64 Contig_source1382A_1352, whole genome shotgun sequence

MKNKLLYLLVALFLACPVSYARMQTLQDAALQYDFYNRDTKIHKDGTHESIVEIQITLLR
EQAREFVAKYTMGYNSDSSSVQILEAKTIFQGKEYKVTDGMIEDKPVANNRAGFDEIKQI
AISFPKPEVGATIYLKYKEITNKVLIDKEFFAKYAFGTGGYWKKSNVTINSEIPLLVELN
DPYKKLLLTTENPADKKGYFHKINLKLLSPLINMPINEVAGSALNHKRITYVSLASLDKW
SNLGDKLAVKYNEVLEQSLPDVFLDIAKAAEKEGVAKKEGALEKEEDIVARINKITTMLS
KKVQYCGDWRSINGRLVPQDLAKVAEKQLGDCKDFSTITVKILRHLGYEAHVALVLRGAG
MQAYDEKFPSMSNFNHAILKVISKGGKTYWIDPTNVVSMADGIFPDIAGKSSLVLARGGA
SYEEIPDVAEAHAKTIIVNAVSLDNVGSSNIQLLGEDAMSLTGARLYASAQHIEDWVFKN
FFSVFIPKEARISSSIPNLNSRVVTPITISLGYKNPSMFHKTNIGLAYRLEGGLSLGADM
LDMDIKGSVNDLYLGNPRTMQRTTIFKNNKIRNLKELNFNFSSAHLTLARECVIKGEDSI
ITDKMITHKAWMENSYIKSAEFKKLQDIFRENRNISVVMVKDE

>gene_280|GeneMark.hmm|462_aa|-|124|1512 >NVVL01000005.1 Rickettsiales bacterium isolate NORP64 Contig_source1382A_1394, whole genome shotgun sequence
MAEYRVETDSIGEIKVESNYYWGAQTQRSIGNFKIADQKMPKEMIRALAILKRSAAKVHS
EIGALDIHIAAAIIESADRVLAGEFDDNFPLVVWQTGSGTQSNMNMNEVLAGIANEKLTG
EKGGKSPVHPNDHVNMGQSSNDVFPTAMHIATVLQSNELLIPALEKMHKSLDNKAKDWDG
IVKIGRTHLQDATPVSLGQEFSGYAYQIKYSLERVGHSLARVHYLAQGGTAVGTGINCKA
GFAEKFAEEVSKFTGYKFQTAPNKFEALACNDALVEFSGTLNSIAVSMMKIANDIRLLGS
GPRCGLGELNLPANEPGSSIMPGKVNPTQCEAMTMVAAQVMGNHVAVSIGGSNGHFELNV
FKPLIIQNVTHSIRLLSDAMNSLVSNCIDGIEPNIERINDLRDKSLMLVTALNPHIGYDN
AAKIAKKAFIEGTSLKEAAIASGVISSEDFDKYVVPQDMIGK

>gene_281|GeneMark.hmm|207_aa|-|1522|2145 >NVVL01000005.1 Rickettsiales bacterium isolate NORP64 Contig_source1382A_1394, whole genome shotgun sequence
MTRTIELSQALSAKLCHDLAGSIGAIDNCLSMICGQDTSIAKQAKVIASEESINLVSHLR
LLRAAYGASAEEEEVSVISIMKLLTDFFEDKKTELNLHFEKGLIFLDTQIAKAVMCLIVI
AAESGAMRGVLDVHVNNINASEFKIISRSKILKLKEDNAQILEGDLDAPIDVGNCREHYI
GRICAQNGYIVSVNQTDELIEYNLKKK

>gene_282|GeneMark.hmm|468_aa|-|2162|3568 >NVVL01000005.1 Rickettsiales bacterium isolate NORP64 Contig_source1382A_1394, whole genome shotgun sequence
LFRFERIVIMENFDVVVIGGGPGGYVAAIRAAQLGQKTALIEKEHLGGICLNWGCIPTKA
LLKSAELYQKMKHSEEYGISAGNVGFDLQKIVKRSRDISGKLVGGIAGLLKKNKVTVIEG
AAKIESPTQISVDGKTTVKAKNIIIATGARARVLEGFEPNGGNIWSYREALVPKTTPKSM
VVVGSGAIGVEFASFYNAIGVDVTVLEAADRILPVEDSEISSMARKAFESKGIKFKTGVV
LKEQKSGKNSVEIIFEHGGKKEVIKSEILLMAVGISGNVENIGLEKIKVKLDRGHIVTNG
LMQTSQKGVYAIGDVAGAPWLAHKASHEGVIAAEMIAGKKPHQITKTNIPGCTYSSPQIA
SVGLTEEAAKADGREIKIGRFPFYGNAKAMILGDGDGMIKTIFDAKTGEMLGSHMIGSDV
TELIQGYVVAKEMEGTEEDLMNTIFPHPTLSEMMYESVLQAYNKQIHI

>gene_283|GeneMark.hmm|416_aa|-|3561|4811 >NVVL01000005.1 Rickettsiales bacterium isolate NORP64 Contig_source1382A_1394, whole genome shotgun sequence
MPIKILMPALSPTMEHGNLAKWTKKEGDSVKAGEVIAEIETDKATMEVEAVDEGVLAKIM
VPEGAQEVPVNSLIGVLLEEDEDKAALAAFIEKESAAGTVASEPKKEEKPAPAASIPAAS
PSSAAPKTEMVRPAGARIFASPLAKRIAKMEGIALANVTGSGPHGRIIKADLLNIGTKRG

TISRHKEEYRLVPNNNIRKIIAKRLVESKQTVPHFYLSNECMMDSVLEIRAQLNASFGDD
KSKKLSVNDFVILATAKALMDIPEANASWSDDAIMMYNNVDISIAVAIDGGLITPVIRNA
DQKEITTISSEMKDLAKRARANSLKPEEFQGGGFSISNLGMYGVKSFNAIINPPQGCILA
VGMSSKRAVVINDKIETRMIMDVNLSCDHRVVDGAVGALFLAAFKKYIESPILMFV

>gene_284|GeneMark.hmm|333_aa|-|4861|5862 >NVVL01000005.1 Rickettsiales bacterium isolate NORP64 Contig_source1382A_1394, whole genome shotgun sequence
VKDKRFMKEITVREALRDAMTEEMRRDEKVFVMGEEVAEYQGAYKITQGMLEEFGAKRVI
DTPITEHGFTGLAVGAAFGGLRPIVEFMTFNFAMQAFDQIINSAAKTNYMSGGQIGCPIV
FRGANGAAARVGAQHSQNFAATYAHVPGLKVIAPYSAEDAKGLLISAIRDDNPVIFLENE
IMYGRSFETPEIVEPIEIGKAKILREGEDVTILAFSIQVGMALAAADILAEAGISAEVID
LRTIKPLDEEAIIESVVKTNRLVVVEEGWFFGGIGATISAIVMREAFDHLDAPVEVVAGK
DVPLPYAENLEKMSLPTVEDIVEAAKQACYREM

>gene_285|GeneMark.hmm|325_aa|-|5859|6836 >NVVL01000005.1 Rickettsiales bacterium isolate NORP64 Contig_source1382A_1394, whole genome shotgun sequence
MFEQKKYKLKPEEYVDSFEKMLLIRRFEEKCGQFYGMGLIGGFCHLYIGQEAVIAGITLA
KDEKDSTITSYRDHGHIIMSGADPKYVMAELMGRSSGCSRGKGGSMHLFNKDGKFYGGNG
IVGAQVPIGTGLGFAEQYNGTNNLCFTFFGDGATNQGQVYEAFNMAALWKLPVIYVIENN
EYSMGTSLERSTAMTDLYRKGESFNIPGFQADGMDIDAVYNATIQATEFVRGGNGPVILE
VKTYRYRGHSMSDPAQYRSKEEVEEYKQRDPVESAKKIILDNDYASADQVKEIEKRVKAK
IAEVAEFATSEPFPSEDELYTDIFR

>gene_286|GeneMark.hmm|116_aa|+|7039|7389 >NVVL01000005.1 Rickettsiales bacterium isolate NORP64 Contig_source1382A_1394, whole genome shotgun sequence
MYPAADISKARDFYETILGLEVGNIFKHPNDGFWIEYDLPEGGCLAISNLDTGRTPSPNS
GGAIAFEVEDLDIIIADLKSKDVQFKLEKFTTEMCSIAVILDPEGNAITLHQLKRK

>gene_287|GeneMark.hmm|78_aa|+|7401|7637 >NVVL01000005.1 Rickettsiales bacterium isolate NORP64 Contig_source1382A_1394, whole genome shotgun sequence
MKKILIIASSLILLASCSNKAKNSLGITETLPDEYQVTRNKSLEIPPHYHLNAPVNDNKN
TGKKKLSEAENKLLKEIK

>gene_288|GeneMark.hmm|432_aa|-|7665|8963 >NVVL01000005.1 Rickettsiales bacterium isolate NORP64 Contig_source1382A_1394, whole genome shotgun sequence
MAGSRARHVIKESHPDWKAFKGRAARDVLFSRFAYKQHDAQMQELADKMPDGYEMFLMTD
HLDDTKEASFRCAAFMNWQTKEVVFATAGTRLGRDVSKMGSDLFDDALLAASQKPRKLNP
AQVLNDMILDSLGAEASEYKFHYTGHSLGAAMAEMQAVDMDIKLTQRDLKAGEEQISAVT
FENPGTKKILSGMYKDAGLPAESAENLRLYEFNNRPNMINSLNEQAGEVYYVIEPNSQKEN
PSLIQMVIEFVTKKDKETSPFVGWVISLLEKGCDSYVKNNPLAGQVIGLLEQGLNLYSDH
KLDNFDEVFVQKEGRVTKNGELITVEEAYSGVKPMEYDEQIASRVVQLKKEEGDTGKQEY
AMNHINPETGEVSRILCGREELEVAVALIQSEIGNAMQNIIARKAPTALKSKEESRFELP
SAAEIVKDGLER

>gene_289|GeneMark.hmm|137_aa|-|9148|9561 >NVVL01000005.1 Rickettsiales bacterium isolate NORP64 Contig_source1382A_1394, whole genome shotgun sequence
MKKMFAGLLFVVTCVFSMSSVLAGDVVDSTGKERKAIFKSKAKESVQAGSKARSVVFPCK

IKNKLLVKTKHPVINHKKEEEDDEFSEDKKLDELNSEEETKAKTKANTQVKGKTKPKITT
KTTTKIKTAKAEPEATK

>gene_290|GeneMark.hmm|1074_aa|-|9743|12967 >NVVL01000005.1 Rickettsiales bacterium isolate NORP64 Contig_source1382A_1394, whole genome shotgun sequence
MASDSDSNAEGTLSIEEDLNSLNFQLEGVASLPSELPEVKNVRRREVPTGVGGEYDAGSS
FFPESYAPQSVPLDNLLVALETIGNIKNGNLEPSVVLTTFAELARLGKRSQAYVDELSGD
QADFDLSIREFQTQIVFLERRLDGALEKKGALETVITRAQFKLGDLQQSFTEEQEKNLAL
GVRIQDLGGRLRKYESTYDDSAPGLSRNSSVADYDVSLRDDLRRSEVQVISLKNSNADLL
IRAEFAEEECARQQDKIQAQSQEIKDQNQKIQDQNQKIQDQNQEIEDQSQKIERYEISNG
ILQADLKERERITNSVSVELKELKASIDSGGNTRRDSQPMLDMLGHLVNRFEVDGKNGAP
SSDNGVDRLAVTAEAGQRRSSLADILVAEDLQQDRQNSTIIADLRDQLKVAVAENQGLSQ
LLSQLSEATSEGDLAKLSSGSGLDVVASIAGKLSSVYALETRIDTLGRENNDLRGKVFTL
EDAADLVADEHRKQLEGLSAQHTAELEAARSRVSELEQTAETYGADMAALREQLAAAQRQ
ATEATEAEAEAAQKRVGELEQAAGQLENLRGQLAEAKEELEKKETALGELCNQLATARRE
AWEATQRATGAEEAQCVAELAALQAANDKTTAERRLVFLRAEVGEYKQVIAGQREVMQES
RAKLEEAVYEAGCYRIAKDETFALLKDSWKEKDELERQLEEFQWIADEARQAALDAQLEE
AARQALAVAVAAAALAAAQQRADTEAAHRVEAERLLNEALMTPEIREALRRLIEARARRE
AEAQQRREAEEEQRRIAAEAAEEQPEAPEQQVAEQQVAEPDVAVEALSEPRVLTLKVALP
DGVELKEGLEVPGNAQKFVPNVVFEYVEQFAHIDALPSVDQGVSLQAQQQQQPIKGGVLF
EEGIPPAQPPAEFVSDDEVVAKSGKKIAAERLEAMKQAASATASDATSEHGGDVALNGSG
AMLLAQQEVATEGSFKEAVDEQLLGTPAAKQSFAAQVVENNEDGLVGIMDTLMRAVSMMP
SPSFGSQPIHSQEANKRRNQDEKRSASPGDASSAKKKPAAKDKKPESESPAPSA

>gene_291|GeneMark.hmm|606_aa|-|13661|15481 >NVVL01000005.1 Rickettsiales bacterium isolate NORP64 Contig_source1382A_1394, whole genome shotgun sequence
MHKILQFTQINNFSVMTKQLTSEPALSLGSTLRKCAYFLFRKQKLQSLFLVFLIVLSGII
PSVDSLFLQNITDAIEGFSDKDLQQTNLVEMLFKWVIIYALWWEGLNILWRVYDYAYLKI
MPKIKAQVIDEFYDHIQHHSHEFFQNNLAGDISNRITEASRSLEMVFAFANEKIIRKLSV
ILFAMLTLYSVHIIIASIFLAWVVLFVGSSLLFARTINNYSTIYGKDKALVAGKIVDSIS
NISAIRMFASHRFERRYITTHLTSLVKSDQSMQWFMFKLRYFLGTSCTLMICAMIFYIIT
LRSNLDISIGQCVLVITLCLAVIADTWDLTQEFGDLFEQVGSFNQSMSLLEKCAITDHPD
AKPLIVKEPRIEFQNVTFKYYNNSNSFNNKSLIITPCERIGLVGFSGSGKSTFTSLISRL
YEIESGKIMIDGQDISKVSLETLRQNISIIPQEPILFHRSIRENIAYGKLGASDEEIRQA
AKSAHIDEFITNLPKGYDTICGERGNNFSGGQRQRIVIARALLKEAPILILDEATNSLDT
HTEKLIQSSLQQLMEGKTVLVIAHRLSTLLHMDRILVFDKGHVVEDGTHAKLRKNADLYK
TLWDNY

>gene_292|GeneMark.hmm|222_aa|-|15581|16249 >NVVL01000005.1 Rickettsiales bacterium isolate NORP64 Contig_source1382A_1394, whole genome shotgun sequence
LNCALGFPGATVSICSISQKVFMSRKRRVDNALEDSSRPHKKQIVQGQQDHPDQSSPSAI
QDASNSSDSLTYLIDQLFAEPILGSTRYSKEKKTSYMEIVQKYFPDKTLNPVALEPPLEV
LRVSLSEQIEPPKAPLVPRAIPELEPEPEPAPAAPSVPVIKCSFPLDAREFFVFDEGVHG
EPSEAPSSSDFIFWQFEYYCFAEPSVDRLLVGVLDHTDFPWF

>gene_293|GeneMark.hmm|399_aa|-|16407|17606 >NVVL01000005.1 Rickettsiales bacterium isolate NORP64 Contig_source1382A_1394, whole genome shotgun sequence

LQPNKKLVQVSNQAFLATPDFPTFLDFSVLSGSPGLPGCMSFPGYAGFPGFPDCPVPPGL
TAPPASHSDGNSVSQAAEHSAKPDLGPPALPSISKKHNSSELNHAISSYLKSAVEISNEL
ETALRQRLNGTDRDSNACKKPLIYLLSEGANKWDQNKGLVIKLLAEGDKIDELNNRGIIS
SGVINSEITALMHTCGAGSSRINLEAFKFLLSQDADITACNYRGWNVVDYIIRGRFVILA
FEALLDHCREHKKFPVSLDLIESCLNNFSIREKNKSKCIKVLKKYLTKNKADLSQNDFER
ALEMVKSPSFEHAEPEVDEVAELSEASGAPPDSFCLTPVSPEDSKSSFCLTPVSPEAQLE
SLFTPHVPPCFNQVIAPPALISAGMLGLSDPLSIFEFLQ

>gene_294|GeneMark.hmm|599_aa|-|17837|19636	>NVVL01000005.1 Rickettsiales bacterium isolate NORP64 Contig_source1382A_1394, whole genome shotgun sequence
MTPIFYIKGGILNFGDKNIFSELELYVSKGDRICLVGKNGCGKSSLMKIIDGEYELDSGE
LFQDPVVNVGYLKQDVKERPECTIYDFVMSGFKDPENNKYHADIILKQMEIDGTLMFNNC
SGGQVRRVCLAKTLIDKPDILLLDEPTNHLDIKAIEWLETYLKGYEGAVICISHDRMFQE
NITNKVWWIDRGVLRKSNKGFKHYDQWHQEIIESEEAVLHKMNRKLNAEKSWLQGGVTGR
RKRNVKRLANLQSLRENLQEHQSRLTNAKARLKIELAEEMKKTKFIIEANNISYNFPGKR
LFNNFTFKVKKGEKIGVIGSNGSGKSTLIRILTKELEPQEGKVKHGTNIDITYLDQHRTE
LNPKHSLKQTLCPTGGDQIFLQNKTMHVAGYLKQFMFDPKTLDSKVSTLSGGESNRLLLA
KILMNPGNFLILDEPTNDLDMDSLEMLLEILAEYKGTLIIVSHDRDFLERLVTRTLVFAQ
NEVIDLYGGYEDYLKYHRKEEKISKPKKVSTPATQEKSSKPQRLSYKYARLLEVLPEEIE
QIEQRIVTIENKLAEPGFYNADNRQFSLLTKELEKIKLELDVKMEQWLEVEAMQGDISE

>gene_295|GeneMark.hmm|434_aa|+|19790|21094	>NVVL01000005.1 Rickettsiales bacterium isolate NORP64 Contig_source1382A_1394, whole genome shotgun sequence
MSNMELAEFAAEELDKNPFIEDDNITVESSEPKKEQNDKEKICQTELRQTDLGPTTVRGK
DYSSQDYLANIATEKSLKEHITEQINLSFNDSKDKIIAYYLLDSMLSTGYLGITLNEAAI
MLRCDEKIIVRVLKTLQNFDPVGIFARNLRECLTIQLQNQEDTDYALLTMVQNIDLIATG
NFKKLAKLCGIDIANIGEMIKKIKSLNPKPANGFFFEQTSYKIPDVILTFTNDEHVKLET
NTESMPRLRVNLEYYLKIKNTVEKKEERHFAKTEVESANAIVKSIRQRSQTILKISKAIT
EQQMDFFTRGVMYLKPMTLNEIAAITGFNESTISRSTANKYISTPTGIFELKYFFSSSLG
TTRSASDNVSSTKVKEIIKQIIASEEDGEILSDDEISEQLSKFNISIARRTVAKYREAIG
LPSSSIRKRNKQMA

>gene_296|GeneMark.hmm|1387_aa|-|21201|25364	>NVVL01000005.1 Rickettsiales bacterium isolate NORP64 Contig_source1382A_1394, whole genome shotgun sequence
VGDGDGGSVIIGQSEAPDLNDVGEPEKGLYWEHHALPSQDKEGDGIPQSYGSLCLKVKEI
VGNDLDEDSIKNLSNSCISIDLGDLVDVASLAEGVNGLISLLYEYKMTEHFPALLQLCEE
PVLPDFGSYSLASTPAGSTAPSRQGSVRISSLSAQSSVRDLAEMFSGTACSTMNSQTGGA
YDPFQPNDSFVADNATFSTRFYKDIRSSEHILTEDDIDVLTKKLESEDLDYGYVFDLLTE
KLELPYNQQDFSIVQSYSTLIITCITELAKSNGLQTGDFEQIKAFLLKGVVSLESIVESL
LSLEPCGNSKLYNYMETLLFDNKQYITEKKAHTMFCAMQEYNNQCIAHKQPVIHIPESLL
NKLSDENFNHYLSTGLSRSSISIDEVRKQNFDQGRFYNIIAYALNTANVSSGSLNVSDLA
AKWHLSSGEIFAIMELCSKVEDSEYDDVLELPKLMKCLLRAHKLKDWCDLAQAAVALPLG
ENQLDQRILSLLIHSSPTEEILREVATKLPEICCDDRMRAELEKQGISLTDFMWLLNNGR
LDCNGDLVFASSYYEGLSKDGLSKLLKLSIGPVDFSWSERLDGEIMESALFNLDDAIDLP
PKLKPAFEARKHARDLFSKIRDIEGDKDSQSSLEISKWLQGGNVKALSKKLYDVAFLKRI
PLSEVCDLLPTIGIDDRIQLVLKINQTISPEDMILLLGAYDCNVTAHIEDLEGKNLLEYM
VARMFHGEDFKVNLLFALATVDLAIPNAPLNLNQDQLGYVTGMILNSGQDAVSAADTLMT

LAKNSGIDGLYAVLWGCGKRDYVMSKIDEANHQTILDELISRVSKERIASVLNEFSPHLR
EVQTAYEALIRDKAGDYTRAAGVLYEVYTRCVDTENLEELEEKILESLGTFAKPVVAPIP
GYKVLQKLRDQINANRAKRNQDQTGDEESGFDSKQLSTLEGLDSDDAKMWEVPQTTFGTS
DEPPESWSGGFSLLDESLDELDATDDESESGYSSYEPSDEDSGADADVESDSDRDYPDDD
FEDDQLGQFFPPGKAPTAPPPLLLAHGAQGVGVANPPPLPEFPPAKKVEIPPAPPPHVPP
HVPPQVQPPEVPPQVPPQVPPPHAPAPQVLPLPQRPELKFPELHHLPAPVVEFAHEYKVE
GGESGSGGPRGPESETVEPKIVTLLKQQVKQGAGSLILEPDTGSLVASLFEVSAAHEAGF
GFDSAARYKAELSFSSLASPLKNLEQVTLEKTVRRAEEIVPEEEKQKAAALPANFAVEAD
KSVDMKTPGKERLKDIEDPLLLKEEVLPALLLGVSAMKPGEEQPEDSEEEDLTGKILLER
KQSVSDLEYAIALIQSLLAGRSPPPSTPKTLGERRKFGLFNWLLSMLMMFINIGLIAMSF
KKAAKGR

>gene_297|GeneMark.hmm|660_aa|-|25939|27921 >NVVL01000005.1 Rickettsiales bacterium isolate NORP64 Contig_source1382A_1394, whole genome shotgun sequence
MNNNTKNILVWIAIFVVMITTISALQDDNTRIGTERLAFSDFLNRVDERQVNSVVIQGRW
VRGSLSDGTAFTTYAPDYPGLIDKLSMNGVYIDVSPPDTKMNSLFSIFVSWFPMLLLIGV
WVFFMKQMQGGGKGLGFGRSKAKLISDKGNKITFKNVAGIDEAKEELAEIVDFLRNPGKF
QSLGARIPKGCLLVGPPGTGKTLLARAIAGEAKVPFFSISGSDFVEMFVGVGASRVRDMF
EQGKKNAPCIIFIDEIDAVGRHRGVGLGGGNDEREQTLNQMLVEMDGFESNEGVVIIAAT
NRPDVLDPALLRPGRFDRQITVSNPNINGREEILKVHAKKIKCAKDVEFRVVARGTPGFS
GAQLANLINEAALSAARLGKKIVDMTDFDEAKDKILMGPERKSMAMTEEEKNGTAYHEGG
HALVSLYCPSLDPIHKATIIPRGRALGMVMSLPEKDQISVTCEKIKAQIAMALGGRVAEE
VIFGSENVTTGASSDIKAATSYATHMVTEVGLSAKVGPIFHGRSHEDNYIAGAGDKTRSE
KTSELIDSEISRIIKEGYETATRILTDHVDQLHILAKALMEYETLSGKQIKHLLLGNPID
TEETPEFPAGLKKKPKSKAKVAARSASSAGDVMGVVEKALERKGRSLKKPAPVPKKGAKE

>gene_298|GeneMark.hmm|432_aa|-|27934|29232 >NVVL01000005.1 Rickettsiales bacterium isolate NORP64 Contig_source1382A_1394, whole genome shotgun sequence
MPSTRFDDKITNSLGTIPPGKIAIAVSGGSDSVALMHLASIWARGAGVQLVILSVDHNLR
AGSKDEIRYIAKLARDLGHEFYPLSWDCGGSKVALQERARQGRYDLMTSKCHDLGIDILL
TAHHMDDMFETYLMRKSKKSSILGLSSTDEFFHNNIRILRPLSGCSKAELVHYLEQNNIG
WLEDESNRSDAYERNRIRKQLALYTRPEKLELAQEIAGVNNQASVLNERLIIAMAELLSI
NEYGFAMIDLAGLKKERCDIQIPILNYILTVISGKTATPRFRSIRKLLAKLAHNEKIDSS
LHGCILKETSKGLLIYREYSAIDKIKMPVDFAKNNYWDGRFEIISPQSCDGYYIDCLKFS
DYIELKDKINLKNLAKISDNNHKSILFTLSAIRRLEKIVAIPNISYYDSSGLEISFEVIF
RPSFISRFTHFL

>gene_299|GeneMark.hmm|524_aa|-|29376|30950 >NVVL01000005.1 Rickettsiales bacterium isolate NORP64 Contig_source1382A_1394, whole genome shotgun sequence
MTKETQNTYDETPYHSYPYAQSSPERLATLGALFGMDAPKIETARVLELGCAEGGNLIPH
AMHNPKGEYVGVDLSKVQIDAGIKNVKALGLKNVDLKHCSIMDIDKSFGKFDYIVCHGVL
SWVPDVVREKIFKVVNENLTENGIAYISYNTLPGWNMVRTIRDMMLYHSKNFQDPNEKVT
QSRALLEFVKDSLKNADTPYAKTLTKEAELLAKQGDHYLRHDHLEDENKQYYFNEFMAEA
GKNGMQYLSDCSLSSMYLGNMGKEIAEKLKDLNDIVRTEQYMDFITNRRFRSTLLCHKGV
KLNRALNNNDAKKFALSFNITPEKSLKDIKLASKDPLKFYFKGNKEQYITTSSPWLKAIL
YTFIENGGYPLKFDTIIEKANKKFKTDSKAQIEADLLKNVMNLVIKGYIDISLIERTSDK
VKVDKPKISDLAFYQANNTNNTWVTNLYHAPVGINLFDKFALKYMDGKNTKQQILELLIK

DVKDGKINMSKDKKKIEDPAQIKKELIAHLGHTVNRLTTQGLFV

>gene_300|GeneMark.hmm|249_aa|-|30982|31731 >NVVL01000005.1 Rickettsiales bacterium isolate NORP64 Contig_source1382A_1394, whole genome shotgun sequence
MIKSIKQSGIVALLSAVTLLSSAAHANDEISADDRYFYIGTEYGISDPVVNKFQDKDTKT
DVEMKRSTMYGGRIGYSFYPGMMIEISATHQPKYKLGYVLHPDTLGLPKAVPGTTDMSTN
VFTANLIYELPKKFAQLQPYVIFGAGVSRISIKSASSSVPGPQGNMEIFRIKNQTANYFT
YQLGVGVTRDLGANLSLDLGARVQVVNNLKLKYDALDKITHVVKAQKPIKKTIAVGEFTI
GLTFKIPVK

>gene_301|GeneMark.hmm|428_aa|+|32241|33527 >NVVL01000005.1 Rickettsiales bacterium isolate NORP64 Contig_source1382A_1394, whole genome shotgun sequence
MIFIYNLLSLLLFPIYLLVLLVRLCKKKENLKSILQRHSLLMPSRPDGQLVWIHAASVGE
SMVALTLIKAISTEHPKMNFLVTTGTLSSAGLLANWLPSNAQHQGAQHQDAQHQFVPIDN
ILIVRKFLKHWRPKLGIFIESEFWPCLINESAKNFDLILANARLSDRSFARWQKRRGIFN
FITSKFKTILVQSKTDLDKYQNLGCGRAINLGNLKFANKELEVDTEKLASLKSIFGDKKI
FLASSTHKEDEEVTLKIIKSLKQKNIDYYPIIILRHPERVGELAKACAQLGLKFSIRSKS
PAPSLGDDLYIVDSFGELGLFYSLAYISFIGGSFKHGGHNLVEPAYFDNVILLGPDMSNF
QNIADDMTSHKAAIQVRNAEELAEQIEFFLDEKNSKERSLYSANAQNFVDSRKETLNNYL
TAIDKFLK

>gene_302|GeneMark.hmm|333_aa|+|33658|34659 >NVVL01000005.1 Rickettsiales bacterium isolate NORP64 Contig_source1382A_1394, whole genome shotgun sequence
MIKLLYPKFWSKRGLISFALIPFSWIYQLLGVLRKKFASPIRLSQMVICVGNMSVGGTGK
TQITLWLAKVFRKKNIKFVIVTKGYGSSLRGAKLVTPTDCAADVGDESILLQEIAPVIAA
KTVHAALPIIRELNPDVVIFDDGMQNPHFIKNLNILVIDSARAAGNWRIFPAGPLRENVN
SAINRSDIIIMIGSKPCNDFELVQAVTSSQKTIFNAGMNLTSLHDASKQYYAFTAIGDPE
RFYRLLAENNLSILGKRSFPDHHNYTPEEIEELKNIAKENDCSLITTKKDYVKISDNNGN
GDSGDIICADVALDFENEANLLDKINEKLHKKS

>gene_303|GeneMark.hmm|292_aa|+|34634|35512 >NVVL01000005.1 Rickettsiales bacterium isolate NORP64 Contig_source1382A_1394, whole genome shotgun sequence
MKSYIKKAKHIVEYVLFTLITSALRLLSIDNSANLCSFIARKIGPSLKATSVARKNLQRI
LGNEVDTDKTIDDLWDNYGRYIGEFPFINKMTDQEIEQRVKCIGIEKLKHFQDNKQPFLL
LLAHQANWEFIIRRAKDLYPQFGVVYRKANNPYVNKTILKERNNDSIIMIEKGANGTRSL
IKAIKSKMSIAMLVDQKMNEGINVPFFGHDAMTAPAIARLSLQYDYPIVPCQMIRIDGTS
SFELVIHSPLEYDATGDTQKDCYDIMLMINQTIEGWIRQKPAQWFWFHNRWK

>gene_304|GeneMark.hmm|381_aa|+|35576|36721 >NVVL01000005.1 Rickettsiales bacterium isolate NORP64 Contig_source1382A_1394, whole genome shotgun sequence
MINKDVLPILDKLISFQSVTPKGEDALQYIAELLSKLGFECHLKNFGSGAKETSNLYAVL
GDCAPNICFAGHVDVVPPMSLDLWNSDPFKMTVKDDIIYGRGTVDMKGALACGLAATLEF
FKQHKPTGSISFLLTSDEEGDATYGTKMMLEHIKNYTPKIDLCLLGEPTTKKTIGDTIKI
GRRGSVNFALTVSGKQGHVAYPEKAINPIPIMTNILKDLSEKEFDSGSEFFQKSNLEITS
VDTGNQVTNVIVQSLSAKFNIRFNEKHTAASLNSEIEQIVKKHCDNYDLQYSSSSAPFIQ
KYSERMKKFTQIVQDNCGVIPDVETGGGTSDARFIHPFAEVVEFGLNCDPAHKINEHTKI
SDLQILYNVYYDSLVEFLTQR

>gene_305|GeneMark.hmm|439_aa|+|36946|38265	>NVVL01000005.1 Rickettsiales bacterium isolate NORP64 Contig_source1382A_1394, whole genome shotgun sequence
MNIAELKKDKTNYHAKVTIPNKEIEELIDKELSKAAKTAKMDGFRVGKVPVSVLRKKYAP
SIRSDVMREKINAAIDETIKKGSLSIAGNPSIEDLLNEEGKDLEFTLKFELLPEISLPDF
KKFSIEKPKLEISEKDIDKQIEELAGYSKTYDTEVKAKAKKGDQVTLDAVGYVDGKAFDG
GKLEGHKLVLGSNSFIPGFEDQLIGSKTGDDVSVKVDFPKEYHSKDLAGKPSEFKVKVIA
VHKESKVKIDDEFAKKFQCDTLDKLKEQISKNMQLSFAEPIHTMMKMKLFDQLESGLKFD
APKSLLEKEKSMIKSQIESMGSEDPETKDMSEKEKEAYFDRLASRRVRIGLMIAEYVKKN
QLQIAEEDIRQAIMAQARNYPGQEQQVIEFYQKDRNAFESLKGPILEEKGVKAIFEKEVT
LKEKTYSKEKLEKLLQEEV

>gene_306|GeneMark.hmm|74_aa|+|38566|38790	>NVVL01000005.1 Rickettsiales bacterium isolate NORP64 Contig_source1382A_1394, whole genome shotgun sequence
MQNFIKLLAVSLLLTGCYGTSYDKNPSPEMKKSSGTKASSSASKHISNHASRVASTAASR
AAAVTRAANKISRP

>gene_307|GeneMark.hmm|182_aa|+|39070|39618	>NVVL01000005.1 Rickettsiales bacterium isolate NORP64 Contig_source1382A_1394, whole genome shotgun sequence
LLIYKMETSLARKFSVVLAVFIAFVSNASAATRSRPGHLPLRSADAREYRTHKTNDLFKS
YLELSSFRHKIIAQNLANVNTPGYKADEVSMPVQYSDLAGSGQVVRKIGMIRTSNRHIAS
KKGAAGRFSSHKLHDPYEIKKNGNNVSLSQQIGKLSENKNDYAAVVKGYATTNSLFSSAI
GK

>gene_308|GeneMark.hmm|164_aa|+|39624|40118	>NVVL01000005.1 Rickettsiales bacterium isolate NORP64 Contig_source1382A_1394, whole genome shotgun sequence
MKKLFVFIILAWVTLACTTLGCTSTALADDLKKAASIASYGTRFQAERIKIAAENIANES
STAALPGGDPYRRKVLFAENRYSKRLGTHVIRTKKIGFDKSAFITKYDPHHPAADSEGMV
KYPNINRIIERADASEAQRGYEANLSVIEMTNSLRQKTIEAMGR

>gene_309|GeneMark.hmm|144_aa|+|40121|40555	>NVVL01000005.1 Rickettsiales bacterium isolate NORP64 Contig_source1382A_1394, whole genome shotgun sequence
MAVDSVSLSAQNSYMQVQNRPKLAPINTQMSQAVDFHRMVDQQFNSFSKMSPAQILAHIQ
GVRGSGASSTDFQAREQSVASTITKTIRETVGKQEEVTRKSLIGEASMVELLTATTEAKN
LMDATVKVRDEFLKAFDKVINMSM

>gene_310|GeneMark.hmm|60_aa|-|40623|40805	>NVVL01000005.1 Rickettsiales bacterium isolate NORP64 Contig_source1382A_1394, whole genome shotgun sequence
MFGIEVGVDMAIVVVFLLINLGVGLYYGQGVKNIKDYALGGRNFSTATLTATVIASWTVF

>gene_311|GeneMark.hmm|113_aa|-|40916|41257	>NVVL01000005.1 Rickettsiales bacterium isolate NORP64 Contig_source1382A_1394, whole genome shotgun sequence
MPDQPFSRSSLPNRHCDRIIFIAYNKLLPHMKKESDDSPLLILLTSARNLFLIENMMPKA
ILMTLIDLKEIELHLYNHGFNIKNGKSELTLKPYTANGIVRIKQSASSLGLSI

>gene_312|GeneMark.hmm|287_aa|-|41447|42310	>NVVL01000005.1 Rickettsiales bacterium isolate NORP64 Contig_source1382A_1394, whole genome shotgun sequence

MLSNNREKNNREKWQVELEELRQEWNLSWEGDPMETPTSIIIFARYKNQPAALKISNSAS
EHTSAVLKHYNGYGAARILKNRGRITLMKRCVPGVSLKDLTLNNEDEKATHILCDVIEKL
HSNVEFEGNYRFTHEFTQFFDHYLNSGDKQIPTKLIEKASAIFADLKDSKEKQILLHGDL
HHENIIYDQKQGWLAIDPKGFVGEACYEVGAFLRNPIDSEICLSHNIIKRRIDIICTRLG
YDKLRVLSLAFAQSILAAVQFIEDGYKYENALQVAKNLEAQELHLRV

>gene_313|GeneMark.hmm|212_aa|-|42324|42962 >NVVL01000005.1 Rickettsiales bacterium isolate NORP64 Contig_source1382A_1394, whole genome shotgun sequence
MRTIISIFLIFSLWAPASYAGNADTKKLGEYIENLINEGHKMFNNKKMSQTDRYHKTTKL
IEGHLYLDWMAKYTLGRHRRTIDKKKISEFSATYAKFIVKVYADLSVHYNGEKARLRKIK
QIDDNLFIVNTVIIKPADQDTIKIDYLVHEINETGDDPYRVGDIITEGISILNSQQAEFN
SVISTRGIDALIVDLQERINRAPALAPAGSDV

>gene_314|GeneMark.hmm|256_aa|-|43142|43912 >NVVL01000005.1 Rickettsiales bacterium isolate NORP64 Contig_source1382A_1394, whole genome shotgun sequence
MRLSIRSIFNPILLALCCIIANNTYAAKSSDKADNYSYNYASINNKCQVYDPYESFNRKI
FMINGILDAFTLRPIAKIYGKLTPDYTKSRVGSFLKNINEPLSTVNYGAQGNAGGIFKTF
WRFAINSTFGVLGMFDVASKVGLKVEPQTFGSTLAHYGMGAGPYIVLPIFGGMGARDVMD
PLISGRMNPIKYLMHSDFKYSITGTSVVHNRDEIMPFTDHVSKNSPDPYIAIRNAILSQR
EDKMVYQDGFKCPVVK

>gene_315|GeneMark.hmm|342_aa|-|43922|44950 >NVVL01000005.1 Rickettsiales bacterium isolate NORP64 Contig_source1382A_1394, whole genome shotgun sequence
MIRNLFSSACSAIKSFFSGLMQIPAWIRQQITCARGNFASLRRKLNDLTGSNMELGIYHL
YEQNFYDAIFRFKMVDKFLKPGDAAANYWLGWVYLLRREHKKSIICLSKADKADEVGLLP
FVKSIDSVSIIPDAICALHRDIIANKFTDKIPVGKVSTDKTNSKTENLPKSLVLALNEAI
TELPDKYSILELGSNIGLLSYEINKRMQESYDLTGVEASSRMINLQQEESYDKTIHSSVN
SFLSKCSDQYDVIVSLEGLAFVAKLEDIFSKIHSILKPNGYFAFSIPVLDKNIFLDKSLE
FAYNKPDIEHQLKKNGFNIVSISEINLEIKNNYSIFVCTKRI

>gene_316|GeneMark.hmm|400_aa|+|45062|46264 >NVVL01000005.1 Rickettsiales bacterium isolate NORP64 Contig_source1382A_1394, whole genome shotgun sequence
MSLIATRLKKVKPSPTLAVTAKAKELRARGINIISLAAGEPDFDTPESIKAAAIQGIKDG
ATKYTNVSGTPELKKAIIDKFKRDNNLDYTPDEVIVGTGGKQVIYNLFMATLDEGDEVII
PAPYWVSYPDMVLLAGGTPVAVSSDMETGFRARPADIEAAITGKTKWLILNSPSNPSGAT
YTASELEELAAIMRKNPKLHVMCDDMYEHIIFDEFTFNTLATIAPDLKERIFIVNGVSKA
YSMTGWRIGYGAGDKDIVKAMSIIQSQSTSNPSSISQIAATEALNGVQDFIEVNARNFQE
KRDLVLSKINAIDGLDCYKSEGAFYLFPKCSDLFGKKTPNGDVLSSSNDVATYLLEIANV
AIVPGIAFGLEGYFRISYATSRELLTDACDRISKAVMQLS

>gene_317|GeneMark.hmm|224_aa|+|46284|46958 >NVVL01000005.1 Rickettsiales bacterium isolate NORP64 Contig_source1382A_1394, whole genome shotgun sequence
MIKRSFKHFLKNSALVHELSACILFLYLKLVYITTSWEFVWEDGISPEIIRKEDGILFAM
WHNRLAFGMYIYKDYENVYALVSSHTDGKLITDVIRKMKYSVIEGSTNRNPTGAVRGIIK
AITNGGQVVITPDGPRGPVYKINSNITKLGHKYSKAVIPVSCMASKYFRLKSWDGMMIPK
PFGRVVVTIARPLPLSGDDEKDSKSLEEALTKLSAQASCELQGI

>gene_318|GeneMark.hmm|506_aa|+|47171|48691 >NVVL01000005.1 Rickettsiales bacterium isolate NORP64 Contig_source1382A_1394, whole genome shotgun sequence
MATPSEKLAESLTVLKQLQEDGNVAIRSTNLARTHRERLLKNGFLQEIMKGWYITTNPSE
RAGDSTAWYTSFWSFCSSYLQERFGENWCLSPEQSLLLHVANRTVPKQLLVRSDKAHNSV
IQLPYATSILEICANQPDPHIIVQDGLRIYSIQYALISCPASFYQQNPTDVRAALTMIRD
ASEILELLIEGSHTTIAGRLAGAFRNIGQERIADDIITTMRSIGYDVREQDPFEKKTPSL
ILNTAEHSPYVNRLKLMWQEMREPIIEIFPKSLGLTQNIDTYLQDIEANYTTDAYHSLSI
EGYKVSPELITKVHTGNWNPGKEDKQDYNALAARGYWQAYRSVRESLIAVLNGENSGDVA
GRDHRNWYREMFLPSVTAGIIKPIDLAGYRSAPVYIRQSMHVPPAKSIIRELLPAFFDLL
RTETEASVRIVLGHFLFVYIHPYMDGNGRIGRFLMNVMCASGGYPWLTVPVEKRDLYMNT
LEKASTSSDIKPFAHFIGSLVEGKSG

>gene_319|GeneMark.hmm|709_aa|-|48990|51119 >NVVL01000005.1 Rickettsiales bacterium isolate NORP64 Contig_source1382A_1394, whole genome shotgun sequence
MYNVFFQPVKTLIPMKEMAAKALNRLGIRNLRDLVFYKPTSYNISDISSDFTKIRDGILI
QAEVTIEAIDRPLSRRSPIKIYTSNDTGSLVLVFFNKIPPFIYSKIRVGNKCIVSGRVQR
FDGFYQITHPDFIFKQTLSAPVQPVYPLTYGIVNKQLYGYIMDGIKILETAINARISFKK
FDDENEKIYIENLLFEIKKLHLIGAPSDSSEIDRSIEESLAKLAEKELFAGQVALAKLKR
KELIVKGRSFPVSEKIKQQVLNKLGFQLTEAQEEAIREIESNQASNHQMMRLLQGDVGSG
KTLVALLLMINVAASGAQSALMVPTDLLSMQHFAFFKQALDGTSINPAVLTGKSTAKERR
LIQAGLESGEIDILIGTHALFQSTVEYKDLGFVVIDEQHRFGVEQRLELIAKASHPDVLV
MTATPIPRSLTLTMFGDMSVSHVRTKPKNRLPIITTIISGTKKPALIESLQKKLTLGEKI
YWVCPLIDQNDKSLEKDEGSSRTGVAKAGSDAGSDAAYASVVERFAELNAAYPGLASMLH
GKMKSAEKDEIMQKFKNGEIKILVATTVIEVGIDVPDATLIIIENAEKFGLAQLHQLRGR
VGRGELQSHSMLMYNHKRLSNTSKKRLEIMRKSNDGFYISEQDLLLRGGGEILGTKQSGE
PDFFFADLGRDLKILIQANKSAANAEFSEFVEFQTQLFARDREELAQSG

>gene_320|GeneMark.hmm|264_aa|-|51139|51933 >NVVL01000005.1 Rickettsiales bacterium isolate NORP64 Contig_source1382A_1394, whole genome shotgun sequence
MIKQHLEDHYNIGKLYNSYLISSDNIEMALAEVEGFVSTYLLEKTGESLRGNADYMIVQK
ESKTTKNIAVDQIRAMQKFLYKTSIVSGVKIAVIYAADQMNLNSANSCLKILEDTTANTH
IFLLTENASALLPTIRSRCALINHHYDDGAKYSIDERFVKPLLKSTPLSERLAFIAEFSV
KDRGLWIEFSSAAESLVAKFSMQFAGVASDLSSLEREVFAQFKSDSPEYLQMKYDQIGEI
IKNTNNFDLDLRASCALLIDKFRS

>gene_321|GeneMark.hmm|460_aa|-|51930|53312 >NVVL01000005.1 Rickettsiales bacterium isolate NORP64 Contig_source1382A_1394, whole genome shotgun sequence
MTVRTRFAPSPTGYLHIGGARTALFNYLFAKHHGGEFLLRVEDTDAARQADSAKQAILDS
MEWLGLVPDEEIVYQTNNAARHVEVARALVDAGKAYYCFTPQAEIVAMREAAYAAKEHFI
FHSPWRDASAESYPKDVKPVVRLKAPEDGETIVDDLLQGKVVVQNSHLDDMVLLRGDGTP
TYMLAVVVDDHDMGITHIIRGDDHLNNASRQQLIYVAMGWDVPKMVHIPLIHGTDGAKLS
KRHGALGASEYKDMGYLPEALNNYLLRLGWSHGDDEIISRAQAIEWFGVGGMGKSPSRID
FDKMKHLNATYMRAMDNEALSEMVFSNIDEPISEESCGYIRLGMESLKVRAELVTDLCDL
AGMYIVDRPLVISAEAKEIIRESDVSLIDQVSKGLGELKEFDKDNIQATLKEIAKQNDLK
LGGLMKYVRAFIAGRTASPSIFEMIAILGKDNSIARLKNR

>gene_322|GeneMark.hmm|84_aa|-|53512|53766 >NVVL01000005.1 Rickettsiales bacterium isolate NORP64 Contig_source1382A_1394, whole genome shotgun sequence
MIYEIENLQKARGVLSGVDGGVILSNPQGSTRYYGMRVIDHIFQTLKQEFPTKIEGFIVN
ADDDYSAFTTAHALGYRTICYSKK

>gene_323|GeneMark.hmm|171_aa|-|53785|54300 >NVVL01000005.1 Rickettsiales bacterium isolate NORP64 Contig_source1382A_1394, whole genome shotgun sequence
MTHGGKRPKAGRPRGQGRYGTTTKAIRVPEYLADEVIDFALNRGYKIPLLRANIRVGSPS
LVADHIEDTVDMNSLLIKNPETTFCMRAEGLSMSDARIYDGDILLVDSSITPVDGKMVVA
SIDSALTVKPLSYIDGKPYLLPENPSLDPIPVLENPELHIWGVVTNVLKSL

>gene_324|GeneMark.hmm|78_aa|-|54391|54627 >NVVL01000005.1 Rickettsiales bacterium isolate NORP64 Contig_source1382A_1394, whole genome shotgun sequence
MSEVIAVDQLRQYISRIEKLESDKADIADDIKQVFDEAKANGFDTKIMKQVMKLRKLDKE
SLAEQEAILDLYRAALDL

>gene_325|GeneMark.hmm|178_aa|+|54832|55368 >NVVL01000005.1 Rickettsiales bacterium isolate NORP64 Contig_source1382A_1394, whole genome shotgun sequence
MLLLMVVFALLQVGLVWAVIASKRKVDTKNMQLIQFYLDTKFLCKGLIDSLKFPESLTFC
NRFTSELKEYFNLEEIIVIDSVDMPANNANTVLKDRVIEFIEKNMDHIARGLHDFKFTTF
ILELDGKEYVLHISRILSKGEGDGFIICIELAPSLLSKNEIATLETCINILRTRLVYE

>gene_326|GeneMark.hmm|278_aa|+|55446|56282 >NVVL01000005.1 Rickettsiales bacterium isolate NORP64 Contig_source1382A_1394, whole genome shotgun sequence
MTQKTPLPLGQKTEYISQYDNSLLFSISRSIARDEIGLSTLLPFAGMDIWNCYEVSWLSP
SGKPNVRIMRFVVAADSEYLIESKSLKLYLNSLNNSKFRSEAELLTIIERDLSAAANSEV
HATFHRLDSFNEQSLATFSGINLDDLDVEISDYNVNSALLTLAHGNERASETLYSNLLKS
NCLVTQQPDWASVQIQYSGRKISHESLLKYLISFRNHNEFHEQCVEHIFNDIMQKCSPDS
LTVHAKYTRRGGVDINPYRSTKNPVETVDVNLSRDARQ

>gene_327|GeneMark.hmm|193_aa|-|56458|57039 >NVVL01000005.1 Rickettsiales bacterium isolate NORP64 Contig_source1382A_1394, whole genome shotgun sequence
MNNTKMRKIIVGISGSSGAIYGVRLLEMLKELGIESHLIISKAAHLTIAAETDHSPAEIK
KLASYSYNPADIGARISSGSFRTDGMIIAPCSMKTLSAIANGYEDNLIARAASVVMKEQK
KLALMTRETPLHAIHLENMLKLSRAGVAIVPPVPAFYNKPSTIDEIVTHSVVRVLDLFDI
ETSAIVRWNGIAP

>gene_328|GeneMark.hmm|274_aa|+|57443|58267 >NVVL01000005.1 Rickettsiales bacterium isolate NORP64 Contig_source1382A_1394, whole genome shotgun sequence
MTTTRIPDEVITELTNQIAVLKQELTRRDDFFTYIHHEINGLIHNIHFTSDVLSNSWDKL
RPDHIKEYIDIIFESAEYLKITSTDLFDAARFNQDDLQRSFAKVDLAELIIDIIERCNKI
FLVQKDLQIIFKNTKNKPVWVNGDDKKLRQVLYNLLVNAIKYSKRGTINVSLESAHQEGA
PQEGTHKNGTHQDGAPAWQVNIQDQGMGIPESDLESVFTPYFRVTKNNKTIPGYGLGLSF
CKRLIDAHSGQIWVRNNQNDVGVTFSFQIPAFAT

>gene_329|GeneMark.hmm|170_aa|-|58408|58920 >NVVL01000005.1 Rickettsiales bacterium isolate NORP64 Contig_source1382A_1394, whole genome shotgun sequence

LLYDSGKHEMASPKKFTPAERKGKAKASYKEWFGQAKEFLIGSLKLRPSENAIGRNIRAF
MLHQACENAFIASSLVFKDDRRKLHDLVELEKGVALVEPEFLRSFPKETKRDKHIFDLIK
KAYIDARYKKYYEVDEEELTEMAEMVKNFHNLVEKLCLRKIESFVENNEA

>gene_330|GeneMark.hmm|80_aa|+|3|245 >NVVL01000006.1 Rickettsiales bacterium isolate NORP64 Contig_source1382A_1705, whole genome shotgun sequence
RFLHGGTNEVKEQREVPFMIWFSDKYKAAYPEKWAAVQSFRGKDISHDYVFHSILDCIGI
ESDAINKSLSVCHRKKDDKK

>gene_331|GeneMark.hmm|520_aa|+|287|1849 >NVVL01000006.1 Rickettsiales bacterium isolate NORP64 Contig_source1382A_1705, whole genome shotgun sequence
MFNFIQKQLDIKIIKTAFVFAVIYCVLFNSPVLIYKFGFYKANMWTAPLEIAKDFIYLVV
TLFAFFFGLSVHRVIFIAGAIILFVTGAIASYYLFFFSIAPTLNIMPTIFGTHATEVYEL
VSLRLVGWVLFSLAVCLYGIRHFNVKTSDSFLTFILSAVCLFYVAGNVTSPKFSFLKTYF
PVQYLHNSYVFFFGHSKNLTKEDISLKYSFKDSSDDGVLGVLVIGESARHDRLGINGYER
KTTPNLAKMDVVSLKAQSCATSTYYSVPCMLSRLNMKDMDLVDSETTFLSVLTRLGFNTI
WLGSQSITKGYRNRKGGSFYYETDFHMIPGGSLVLRPNALDVTLIPYMEQNLDSTKKQFV
VLHSTGSHWNYSKRHTKEFAKFTPAIDSSLIKKDAAGCSLEALNNSYDNSILYTDFFLSR
VIAKIKDRKSFLIYSSDHGTSLGENGRFLHGGEMHLKEQREVPFIIWFSDKYKAAHPKKW
AAVQSLRGKDISHDYLFHSILDCIGIESDAIDKSLSVCKK

>gene_332|GeneMark.hmm|34_aa|-|1891|1995 >NVVL01000006.1 Rickettsiales bacterium isolate NORP64 Contig_source1382A_1705, whole genome shotgun sequence
LSLKAYSFEFIYELIADNLSASQDYTKVKTRYAA

>gene_333|GeneMark.hmm|384_aa|+|1997|3151 >NVVL01000006.1 Rickettsiales bacterium isolate NORP64 Contig_source1382A_1705, whole genome shotgun sequence
MKFYIIAGETSGDYIGGLLIDAIKAERRGSASDGAEDEFFGIGGNNMRKAGLDSLFPIDQ
INIMGFFEILPQILRIKKLIDLAARDIIRKNPQILVTIDAPGFTFRVAKKVRELAPEIKL
VHIVAPSVWAYKPGRAAKYAKLYDHLLTLLPFEPQYFTKLGLESTCIGHPILEQTFFKKS
PALKAEMGCDKDAKLIAITPGSRKGEIARHMPVIRQACDLLACTHKIKAIFVQPDDSNIH
QISSFLSGAKFDYSFSTDRLKAFAASDCALAKSGTNSLEIAASKTPMIVGYKMHPMSFFL
LKMMIKVKYASLINIIPNREIIPEYIQSEFNKDNIIFALSQLINDKKRAQDQVKGAEKIL
ESIGFSNGEKPSEIAAKIIIKMIS

>gene_334|GeneMark.hmm|681_aa|+|3151|5196 >NVVL01000006.1 Rickettsiales bacterium isolate NORP64 Contig_source1382A_1705, whole genome shotgun sequence
MIAHYLSKKLQDEYHNLNLWYFVSFIFGIILFFKFTPIFTLNTIFTILFGTIILSRIFYK
RDLLLFFLSACILSSSAGMLTSYVRMSSVSASTNPIKRPMISRLTGEVERIKPTLQGMQV
TIKNVVLERNITKKRGKNLKKIRIHISNKLSRKLGHNDKLRFKAKLFPLQTSVLPGTYDF
GFYMYMSGIEASGYALTPPEILSKNESTGSKYIQTIRNKVYHRLIQILGGDNGNFAAAIL
IGETKAIPKTISENMRNSGVAHVLSVSGLHLSLVAMLFFVSCRVILNCSNYFAYNTNIKI
IAASASIIGSFMYLQISGSNIAATRAFIMSSVFIISLMLGRSPEPLRSIMIAAFLILLFF
PEYIFHPSFQLSFAAVLCLISGYELYIKYENVINSAKGIFFSIKFYVYGNIYSSLLASIA
TAPFVIFHFYKFATYSVLMNLIAVPLMSFFMMPLGLISMLLMPFGADEFPLKALGFFISI
VTSSAAYITALPASVWAWGHISPMSLAVFSLCFFWVCLWKTKWRLAGLAVMFLSLFMMNN

TKKPDFIYDHTLKAIGLNSQRLGSPKLGSPRLEFQIYVHDKMPKFTSNYWVSWYGAKEAS
ITRKKIAAKDLIFKLPSGKTVSLNYWRCTYADIQIITSKKLKCDNGGQVISNKELWKHKQ
ILLYCDEKECRVQYREQNRWW

>gene_335|GeneMark.hmm|98_aa|+|5391|5687 >NVVL01000006.1 Rickettsiales bacterium isolate NORP64 Contig_source1382A_1705, whole genome shotgun sequence
MSKLPEPESESIEEFARLLSKQEGHSILKHTKLAAPILTVSSVETPPVETPPVDILNYTA
LFDTYAFNVIPKDKTPHHISAIGHAEEVPVTGDPTEAE

>gene_336|GeneMark.hmm|497_aa|-|5872|7365 >NVVL01000006.1 Rickettsiales bacterium isolate NORP64 Contig_source1382A_1705, whole genome shotgun sequence
MTDSELPPFKSKLRNIFWPIHKKELAKFIPMSALMFCVLFNQNVLRILKDSILISEVSAE
ITSFAKVYCVTPMAAIFVVIYMKLINHLPFSKIFYYLISVFTAFFILFAFILYPNVDFFH
MDGEKLSLYMQAYPHFKWYIALIGNWSYVLFYAFSELWPNVFYVLLFWQIANDITSTSEA
KRFYLLFALFGNSALVVVGLMMMHLSSDNSIIHTLFTVSNSKTMITKVSIAMVAVSSVCA
CLLVRYISNVIMKDPALYTEAERSTKPKLGIIDSFRYIARSKYLWLMLICTASFGLSMNL
IEAVWKAKIKELYPTVNSYMAFNSMYILWTGVTIMIMTIIGNNIMRNSSWFVAAVITPLI
VLITGTVFFLLVVFDNNVMSFFEGAILMSPLALAVFVGTVQNVLSKGSKYSIWDTSRQML
YIPLDQELKTKGKAAVDVISPKIGKAANGLIQSMIFTILPMATYTSIAPTLMVIFIVVCV
MWIYAIRKIYFEYQKIV

>gene_337|GeneMark.hmm|395_aa|+|7726|8913 >NVVL01000006.1 Rickettsiales bacterium isolate NORP64 Contig_source1382A_1705, whole genome shotgun sequence
MFDFQEEQCKRYKSEYGKFIKSREVESYKKLSKDIGYKSQIASLVYCGGAYKNTHATSSQ
GQDVPKDNPAKDIITKENSPTGNPSIDNLAPANLARNNSAKGESVFLLIPSIFNSPEILF
LGDKNSFTKNLSTKNNLYLASWSQQTAIQLTMENYVNEVQKMISFLHSLGHKKINLIGHC
IGGNMCLAASSLQGINQSAEQSANQDAGQIESLTLLTCPWDYSHFKKWVYLYDIMGFDTH
LKGINFIPKIYIQMMFFLMFPFQFKEKLEKYFALEQAHEKQSFLQIEAWLQSGNIISKSI
YAEIMQDFCRKNILQNNEWLIGGELITPEKLDIPTCIIYAKDDQIAPHSSIAPLQKRIKS
STLIETEGGHIGYLVKGCAYLQTKYNEWIVENNLR

>gene_338|GeneMark.hmm|391_aa|+|8919|10094 >NVVL01000006.1 Rickettsiales bacterium isolate NORP64 Contig_source1382A_1705, whole genome shotgun sequence
MQEAYIVSAKRTAIGGFLGSISSVPAPVLGAEIINAILRASKIDPSIIDEVILGQVITGG
SGQNPARQSVIKAGLPIEIPAMTINKVCGSGLKAVALAASAIRAGDGEVFIAGGQENMSL
GMHGSYMRGGNKFGDTKLTDFMMYDGLTDAFSKEMMGITAENIATKFTISRKCQDELALS
SQSKALAAQKDGKFQDEIVPIEVKHRKETVIFAEDEGIRPETTLEKLSKLKPAFKEDGTV
TAGNSSTINDGAACLLVVSEAALKKYNLTPLARIVSYAQCGVDPALMGTGPVPASKKALS
AAGWNPLDLDLIEANEAFAVQAEYVNRQMGWDVAKLNVNGGAIALGHPIGASGARILVTL
LHEMKRSGAKKALASLCIGGGMGIAMCLEGM

>gene_339|GeneMark.hmm|94_aa|+|10429|10713 >NVVL01000006.1 Rickettsiales bacterium isolate NORP64 Contig_source1382A_1705, whole genome shotgun sequence
MSQFVKNILTKEPGSLLTQIVDMLSSVDDISIEISDSGVASISKEKSKFVIIEKETIKLA
DKGGVFTPLKESIKTQDDFLKAATEAYWIVAKKK

>gene_340|GeneMark.hmm|195_aa|-|10824|11411 >NVVL01000006.1 Rickettsiales bacterium isolate NORP64 Contig_source1382A_1705, whole genome shotgun sequence
MAKIIFLNGASSSGKTTIARLLQEKLSEPYMHIGIDSLIDMMPDKINNWVDANPDSVQGF
YWQKSLDKNGHNLAHITSGSYAKKVNSTFQDIVVLFANNGHNIIVDDVCIPPKHYDLWKQ
KLYEFQVYYIMVNAEISDLETREKSRGDRTIGSARAQNMLVHKDKIYDLKINTSKNTLEE
CAQKIADFIRSYEVT

>gene_341|GeneMark.hmm|139_aa|-|11421|11840 >NVVL01000006.1 Rickettsiales bacterium isolate NORP64 Contig_source1382A_1705, whole genome shotgun sequence
MAKDVVGDKFDLFLYAVGKGDIETVTLFTDKTNAEIISALVVAAQNNRAEMVRLLLKNLA
LKNLDQDLSSISDIDITPEVWKQIQDFANDKDWEIVVSEDHASHLAGRPVLGSTYWGESS
WWGIVIECFSSIFPDENSP

>gene_342|GeneMark.hmm|232_aa|-|11904|12602 >NVVL01000006.1 Rickettsiales bacterium isolate NORP64 Contig_source1382A_1705, whole genome shotgun sequence
MSDESMLALSLKGVSKTYMQGGSLIDVLKNVDLEVRTGQMVAIIGASGSGKSTLLHIAGL
LDSADKGSVSIGAEQIKNLRNANRVRLNNMGFIYQYHHLLRDFTAQENAAMPLFISGVNK
QEAMERAGSILSDLGLAERLGNMPGELSGGEQQRVAVARALINNPKIILADEPTGNLDKS
TADEVFAMFLERAEKFGSAVVMVSHNLELASRMHKIYRLECGLLEEECARNK

>gene_343|GeneMark.hmm|414_aa|-|12595|13839 >NVVL01000006.1 Rickettsiales bacterium isolate NORP64 Contig_source1382A_1705, whole genome shotgun sequence
MLSNFISILSLRYFSAKKNEKFISIISAISLAGIAIGVAALIIVMSVMNGFHIELTGNII
GLNGDIKITPVGKTIENHQEIIKKLSAKSFVTKVTPVVQGQALVLGKTANSGVLIKGIDL
VDLKDKGSILGNVHSGSFEDYYGDNAVAVGSGLAGTIGVYARSKIKMISPVILPTLFGSM
PRAKDFKVVSVFSTDLYDYDSATMLMPISAAQKFLGLGEGINLLEINVDDSEKAKAYARE
LQKELGANIYVTSWLEEQKYFLDALEVERSVMFMILSLIMIVAAFNIISSLFMIVKDKTK
DIAILRTIGASTKQIMCIFIINGMMTGFIGTALGVISGLLFSYNIENIRKLLESISGSRL
FDPAIYFLYTLPSVVRFGDVVFVASLALGISFLATIYPAYKAAILNPVEAMRYE

>gene_344|GeneMark.hmm|433_aa|-|13829|15130 >NVVL01000006.1 Rickettsiales bacterium isolate NORP64 Contig_source1382A_1705, whole genome shotgun sequence
MLLSQYFLPVLKEQPSEAQVASHSLMLKAGMIRQSSSGIYSWLPLGLKVLRNIENIIRFN
MDASGFVEILMPCVQDGDLWRESGRLDVYGEEMLRFKDRHGHDLLFGPTNEEVITDIIRN
SIQSYRDLPKVLYQIQWKFRDEIRPRFGVMRGREFLMKDAYSIDLDEESAIKTYDQIFAA
YMRTFKDLGLNVIPLAADSGEIGGSMSHEFHVVAETGESKIYYDKKFDIIGEDLMGDMNQ
RKSLYAATEEKHDPKTCPIPESELSVKRGIEVGHIFNFADKYSKPMKAMVNDNNGKQIPV
HMGAYGIGVSRLVAAIIESSHDDKGIIWPISVAPFKVSVINVNIKDELSCSLAGNIYNML
RSKGIDVLYDDTNARTGAKFATHDLIGSPWQIIVGPKKAASGLVELKHRASGEVDEVSPD
EAILMVVNSLNVK

>gene_345|GeneMark.hmm|189_aa|-|15237|15806 >NVVL01000006.1 Rickettsiales bacterium isolate NORP64 Contig_source1382A_1705, whole genome shotgun sequence
VTKPTKDQAEQAVKLLLEYIGEDVNREGLKETPARVVRSYAELFAGYALPIEDVLSKTFT
DVSEYNDVVLLKKIKFTSLCEHHMLPFYGTVDIAYIPGGEVLGISKLARLVDMFSKRLQI
QERMTFEIANSLHSHLNPGGVAVRVSAVHSCMSARGVAKNGAVMDTTYFTGEYHEHAEKR
KEFLDSIRG

>gene_346|GeneMark.hmm|462_aa|-|15825|17213 >NVVL01000006.1 Rickettsiales bacterium isolate NORP64 Contig_source1382A_1705, whole genome shotgun sequence
MKNNTNLHAILWLKTGITALAIAGLYSIILVLLRTPGLNQLFTDKSIFKSALVIHVNLSV
LVWLLAITCIIWSISKVRSGFESLFTYIAIAGMALMALSPLYPESVPIMNNYVPMLENIF
FAIGLGLFGTAILCFAIQTILTSFMGDAFSDYGERIIAITKITSAMMYVAVWICFILSFI
GVSALSEIVPLDIEFFYEMLFWSGGHLLQFIYTQILMIVLLILAEGWRGGRIGSHAEYEL
ILVLNFVLSLAVFLGHAYYDIADGLFKEFFTRHMIYSGGVAPVIFIAMLIAEIVQKRTPN
VPTFIPAAFVSSVLLFLSGGAIGAVISGVNVTIPAHYHGSIVGISVAFMGLSYMLCFQKN
VAGSLTEKGGVISFIIPNSRSDNTKSQRSASWQIYTVTIGQILHIGGLALAGGYGVMRKT
PGEEIALSAKVYMGMVGAGGLLAIIGGLMFVYICARKLFYRE

>gene_347|GeneMark.hmm|314_aa|-|17451|18395 >NVVL01000006.1 Rickettsiales bacterium isolate NORP64 Contig_source1382A_1705, whole genome shotgun sequence
MFEKPLISFDFAIKFLLKSKEDYEIIEGFLSALFVANNYKPVKITALLESESNKEAAHLK
KSVADLIVEDTDGNKFIVEIERSFTPNFTHKACFNTARLISDSISTSQDYSKIQKIFHIS
LLYFETAGMEKPIYHGKTIIHEVDTEHPIDIDIMNKGIATFENKNIFPEYFFISIPRFND
QIRSEMDEWLYMMKNSEVKADFKSPYMARVAKRLKVLRMTDEERIEYHKYLKESAVQEDI
LNAANSKGLAEGLQKGMSQGLEKGRKEGREEGREEGAKLTKIEVAKTMLVKGLDINLISQ
ISELSIDEIMELKN

>gene_348|GeneMark.hmm|213_aa|-|18477|19118 >NVVL01000006.1 Rickettsiales bacterium isolate NORP64 Contig_source1382A_1705, whole genome shotgun sequence
MPYAFRYRVVEAFVASFLYLIETICGIKYRVEGLEKLPADGKPYLLLSNHQSFWENCIFL
MLLPKHSWVIKRELLNMPCVGNGIRSVNPIAIDRSSNRSVMYILNEGAKKFKQGISLVIF
PESTRVSVHKNARFKASAARLALNTKVPVVLVALNSGLVWPKGIWLKRPGTITIKIIESM
SPEQVAKYDTRELTDYIQKKITTEKNALVKAGV

>gene_349|GeneMark.hmm|310_aa|-|19351|20283 >NVVL01000006.1 Rickettsiales bacterium isolate NORP64 Contig_source1382A_1705, whole genome shotgun sequence
MHKNFPQYASEAGMLSIAKLASKHGIIPKKKYGQNFIFDETLCDRIVRAAEIGNDSVVLE
VGPGTGGLTRSILKTSPAALYAIETDSRCLPLLEEIKLSYPALEVLNQDALKVKLEDVIS
AVSAGGEEGSEAETSSKSKPSLEIATDKLDIVANLPYNVGSQLLVDWLCQIEHVASMTLM
LQKEVVDRITSESSKRSYGRLSIICQLVCDVHKCFDVSPKAFVPQPKIWSSIVRLVPKKD
RPTKEILLLIETITRHAFSGRRKMIKSSLKNLSPQIGDILDDLGIANTLRAENLSPEDYL
RIACHEGCRY

>gene_350|GeneMark.hmm|300_aa|-|20309|21211 >NVVL01000006.1 Rickettsiales bacterium isolate NORP64 Contig_source1382A_1705, whole genome shotgun sequence
MRKLLSLFIVLLSSSIFTSSAYAGYPDIVALVNDQPITKYDFQSRRKMAIALNNIDVSDP
AVSRKLSRDILNILIDEALLDQHMEKVSGKVTEQEVDDSITVIEQRNKMPKNGLKMFLKS
KGVEMSSFRKQVKGELIKRNIVQALSGGVSVSPMELDVALIHSGNQVFDVEAWVFTSKNH
DEKAQKQMHGLQKRFKGCDKVEDAMYAEFADAEKFDRKLHDLPVKTQAVVFDTKASSFSN
LYSEGDKFKTVFVCRKESAVSSADLNKIKSFLSNKKMSQKAAKFFKDLRAKAYIKNMISG

>gene_351|GeneMark.hmm|695_aa|-|21232|23319 >NVVL01000006.1 Rickettsiales bacterium isolate NORP64 Contig_source1382A_1705, whole genome shotgun sequence

MFSGLSFDKQPISKNVSMKADSVFYDASTDIITAKGNIFVSMGGYSLYADQMNYDSKRDV
IFAEGNVRVVDIDGKVIYGEHVVLKDKLKRGVIKDFIAKLEKNSVITANSAIRLSKNKYV
MKGSVFTSCKMTCNRKPIWQLNAAHVGVDLDKHEMTYKHVFFEVYGIPIMYFPYFQHSTP
NAPAKSGLLTPKIKHGDFMLPFYFRVKPNLDLTLSPRTSGKYTIFETELRHKVKYGGYEV
LGSYGNVEFKKTTATNITKTTKTGRYHLFATGSFVNNDIHYGFDIKRASDKAYLTNYHKI
YDSYLTSKIYANKVNERNYFSLEGGYFQSLKSETSNLSTPLVLPSVRTQNVYAIDEEESL
LLNVRSDNIAHRGSKGSQLARSSLDLELMKNIISDGGHMFSFSLANRADLYWVSFVDNKA
KKAKKEKEEILYRTIPEFRTKWRYPLARSFAAGFNVKIEPTAMVVFGRRYKSEFEKFAQI
DSPKNELSENNIFNANRYSGVDFHDYGTRFSYGANLSLASESIYVDAFLGQLLQKNNIKN
ENNSEYVGSIAIDIEDDFELFYRFRKDKKLSPILDEVGASIALERFTSKAVYTKLQNISR
YFAKDGFEFERNKASQVSFDMDYNLVNNLWVGFSSKLDVSSKSIKMLTRSIRVTYSFDCV
SINAVITNNSLLDASRGVKKTNDISFQVGLLGINM

>gene_352|GeneMark.hmm|445_aa|+|23447|24784 >NVVL01000006.1 Rickettsiales bacterium isolate NORP64 Contig_source1382A_1705, whole genome shotgun sequence
MLEDSLTNDISQSEFSVSEISSKIKSLLESNLGTVRIKGEISGLKIAASGHGYFSLKDTN
AVLAAICWRHALARMNFKLEEGLEVIVTGKITAYAGQSKYQISAEKIEPAGIGAFIKILK
ERRLKLQKEGLFAKELKQKLPFLPKKIGVITSISGAVIKDIIHRISDRCGTNIIIWPVAV
QGSASASEVTKAIDGFNKMAQKPDLLIIARGGGSIEDLWSFNEEIVIRAAHASKIPIISA
IGHETDYTLLDLAADMRAPTPTAAAEFAVPVLLDLRYTLKSLYGRISNRFMELVRYYSQA
LGNHTRALGRVSSDINNHTQRIDDLSFRLIASLPNLLKQKQNALQYFPLSRLKPLKILKY
KYLQYKNCADNLLRKEDSLLSEPEHRLKISLSLLASLDYNNVLKRGYAMIKNKDGTIISS
INEVKKSDNLKLKMQDGEIAVTCNN

>gene_353|GeneMark.hmm|35_aa|-|24841|24948 >NVVL01000006.1 Rickettsiales bacterium isolate NORP64 Contig_source1382A_1705, whole genome shotgun sequence
MSGEYLEHEFLEARSAGLEYLVFLEIFLSRFKFLD

>gene_354|GeneMark.hmm|601_aa|-|25057|26862 >NVVL01000006.1 Rickettsiales bacterium isolate NORP64 Contig_source1382A_1705, whole genome shotgun sequence
MKKFFLTTLLMAAPIAYGEGVVLSYEGNNQFEVSNQNGLITGIAEAKLLFPAGSQLKFTH
EEDYLRDVIAASSYDLSNPNSSMLVQKLNLEIQSIIDSWKSAKFTEEQREKILKTSYNAT
QKKLKEDKVINLANLDHKVYWTCLIEITKELFKKDPKTEDANALLAKNAEKLLARVQIDL
LRLEIKGQEIQNIITKAEDGYFDPATFQQALDNVHAGSAVIEQRVLNSYRQKQSTQETFV
EKLKDLADEKDPHKKQAYLDNINANIDLYVNPDIKAGDNPDELPAIQQPEETADAMTKMM
ATTFETVNQVAGLVSSRVMNKSGIASGNAFESYGLWLKGLYSRGEQKAYKGKNGYKFNQK
SVIIGADIGDESLVGFAYSFSKSDVKNQASSKNKDDISTHMGTIYGKYALTNQVFISGQV
QLGTSTIKIKRDTGINTISTAKPKASIVSGRTEFGYSFAASQNLLFIPTIGVAYSSIKVD
GYQEKGGTNRSINKMTTKRTSALAGVTAQYVHDTGSVTLLPELHFNVEYAFNAKNSDTVI
KFGSLSPITTPSEKPTKVRYNFGTSIRIINLGAFEGSVGYDLGAAKKFVSHTGTIKLRVN
M

>gene_355|GeneMark.hmm|142_aa|-|27045|27473 >NVVL01000006.1 Rickettsiales bacterium isolate NORP64 Contig_source1382A_1705, whole genome shotgun sequence
LIGILSILSFCLFGVYIILSNLTDNIVFFHPPSEISKIQPGATKARVGGIVRAGSILRIS
AKKISFIITDHIADLRIEYEGVLPALFREGQGIVAEGMLAPKPSPGSALELAPDTIFIAS
KLLAKHDENYTPPEIKRISRQE

>gene_356|GeneMark.hmm|51_aa|-|27475|27630 >NVVL01000006.1 Rickettsiales bacterium isolate NORP64 Contig_source1382A_1705, whole genome shotgun sequence
MITYLISAYSFTFAILGYCLAKTILDHRATNANAANEVTSKRVRGEDKKKK

>gene_357|GeneMark.hmm|92_aa|-|27979|28257 >NVVL01000006.1 Rickettsiales bacterium isolate NORP64 Contig_source1382A_1705, whole genome shotgun sequence
MNKGEFVSFMAAKHGCTKADAEKSIDMFTSSVIGALGEGGEVSLVGFGNFSVSDVAARAG
RNPRTGDPIQISAYKQPKFKVGQKLKDACNNR

>gene_358|GeneMark.hmm|148_aa|-|28392|28838 >NVVL01000006.1 Rickettsiales bacterium isolate NORP64 Contig_source1382A_1705, whole genome shotgun sequence
MNSFQETRLVPYKADLIHEIIMGIEQYPEFLPWCDKASILERHDEFITAELGVNFKGFSE
GYVSEVRSSKTDDGYVVSVRAISGIFKHLENKWSIKNVNNGSEVSFSIDFEFKSKILDGV
MGMIFSLAIEKIIVSFESRAEELSRKLV

>gene_359|GeneMark.hmm|299_aa|-|28860|29759 >NVVL01000006.1 Rickettsiales bacterium isolate NORP64 Contig_source1382A_1705, whole genome shotgun sequence
MTEETTLKRPDWIRVKAPNSKEYQETKKLIKDLKLNTVCEEAACPNIGECWSKKHATVMI
LGSVCTRACAFCNVKTGRPDLLDPHEPERLAIAVGKMGLEHVVVTSVDRDDLPDGGAAHF
AECIKEIRKTAPNTTIEILTPDFLKKDGALEVVVAAKPDVFNHNIETVPSLYKTIRPSAR
YFHSLKILNDVKKLDPEIFTKSGIMLGLGETTEEVLQVIDDLRAADVDFMTIGQYLQPTK
KHAKLDRYVTPEEFTYLGRLAKTKGFLMVSSSPLTRSSYHAGDDFKKMRLARQKQLELI

>gene_360|GeneMark.hmm|360_aa|-|29756|30838 >NVVL01000006.1 Rickettsiales bacterium isolate NORP64 Contig_source1382A_1705, whole genome shotgun sequence
MKKVYLESVELGNYRNFADLSLDFAQGMNIIKGPNGCGKTNILESVSFLSPGKGLKSANF
DDICKFTHNSWSSNFHLQSKMGRAEISTSFSENERSRKINYNGSKLSGSELTNLLNVIWL
TPQMEGLFLGGASARRRFLDRIVYNFDAKHAKNLKAYEHFMRQRNQALLQGDIYEQASWL
TKLEEHMAIEASAIDASRREVAFLMQESIDKLETEFPKAQLEVMKLVENQQDTEGFLEYY
TGALAASRKKDSYSGRTSFGVHKSDLLVFHKAHNAPAALCSTGEQKALLISIMLASIAAL
LENSNTTPILLLDELFIHLDEERKKHLSEYITSSKLQTFITTTDIVGIEDLAKNAHIIDL

>gene_361|GeneMark.hmm|309_aa|-|30869|31798 >NVVL01000006.1 Rickettsiales bacterium isolate NORP64 Contig_source1382A_1705, whole genome shotgun sequence
MPKPSEKFMPKPSDNEAPSGEEDPSRAIARSLKSTFKSFYEKVKSKIKLKSQATHIAKNS
LATTTGKHIATTLSEGQIAGNIKGFTEKVVEDILIPRSDIISASTDVTLQELSKIVIEHT
HTRTLIYEKELDNIVGFVHIKDLFEVIANSREFNLKSLTRKHIIAPCSLQLADLLAQMQK
KRTHIAVVVDEYGGTDGIVTIEDLIEELVGEIDDEHDTDHNENSFELIKPGLLVTSARAE
VSELERFLGVKFKKSDDEFITIGGLLMAKSGHVPAKGVVMNITDNIIAEVLERTPRTIKK
IKITYDKDQ

>gene_362|GeneMark.hmm|171_aa|-|31815|32330 >NVVL01000006.1 Rickettsiales bacterium isolate NORP64 Contig_source1382A_1705, whole genome shotgun sequence
MKINVEIARESQDREQRSFLNEEFIAEITERVLSRYPAFAQSGEFELSILLTDNSKMKSL
NKEFREKDKATNVLSFPDEDLDWRNIDKINKADKTHLDSSEVFLGDVAFGYQIISEEAQE
KSISFQDHFTHLLIHAILHLIGYDHIEDEDAKAMETLETELLQSLNIASPY

>gene_363|GeneMark.hmm|341_aa|-|32395|33420 >NVVL01000006.1 Rickettsiales bacterium isolate NORP64 Contig_source1382A_1705, whole genome shotgun sequence
MQAVAYNPGQLNCYCCFMKKFIFSAIIFLVLLLLTWIGALYFVLLKTDIATVPVNKTTYE
LSQGEYEYKPLTALVSYGAGRPVFLSNQFTLAESAINKGIEHIYLYGRHNIDEEFYNKHK
KTLEQPRGAGYWLWKPYFIWKTMEQLPEDSVIIYADSGIVFSKPPAETLIPFLDKEYDII
FSSHGHIPSLGIFMKKEAQIILGIDKNEKILNSPEIWADFMIIKNNASSKKFIKKWLKLC
ENPDLLTDSPFDPDLQDKNLRLHLHDQALLTFLVATNPQRKMVIKNLRERSGFGIHNFHR
HPEDKYTSPKFHIAGIKKKIANLLYNNSILQKIREMQDTIQ

>gene_364|GeneMark.hmm|539_aa|+|33612|35231 >NVVL01000006.1 Rickettsiales bacterium isolate NORP64 Contig_source1382A_1705, whole genome shotgun sequence
MTATLEEIAKSDKIKELYDEMPYASCPFDYNKPEHLCTIATLFGMSPPALENARILELGC
SDGGNSFRFAEIYPNSYTLGVDLSEVEIKNGQDILSQLKLKNMELKAMSITDLDESYGKF
DYIICHGVFSWVPDFVRDSILEVSKKLLTENGLAFVSYNTLPGWNMVNTVRELMLYHSAG
FDNIQDKILQSRAALNFLQSSLDGQDTPHAKFMGEMAKHMSEQKDYYLRHEYLGDENKAF
YFHDVIDMVRAHGLEYLGDADIQKMYTGTLPPEVAEKLNSAGDVVKVGQYIDFIQNTQFR
STILCHQGASIMRDISADLLKKLYFYCTITSKPTEEGLAPNIVDDSHVNFYLDNVESNTL
GTASPAIKAVFTTFAQNRGNALSFDELAGGANALVPEVSLELIQAEILNNFMHLIFSGHI
KIISNKPANIYEISDKPKISALSRFQIDRPSTDGVYWITNALNAIVAFKIQHIKLIKSLD
GTKTIKQLQKQTLESFKKGEIVASENNEKIEDEARLKPIADNLVNNTLGALRQNFCLIA

>gene_365|GeneMark.hmm|173_aa|+|35312|35833 >NVVL01000006.1 Rickettsiales bacterium isolate NORP64 Contig_source1382A_1705, whole genome shotgun sequence
MFIDKIPAKTKNGDFNVIIEIPMNDNPVKYEFDKESGAIMVDRFMQVAMSYPCNYGFIPH
TLSDDGDPADVLVMSQHPIIAGAVVKVRPVGVLMMEDESGMDEKILAVPISKLDVTFDSV
KDIGDVSDMLKQRIHHFFEHYKKLEKNKWVKIVGWENAEKSLQIIEEAITRAG

>gene_366|GeneMark.hmm|52_aa|-|35914|36072 >NVVL01000006.1 Rickettsiales bacterium isolate NORP64 Contig_source1382A_1705, whole genome shotgun sequence
LLNKELSEIYICSFGVVGLEALRVALEFLLPGLEYSLFLIFFHLFFNSKILI

>gene_367|GeneMark.hmm|204_aa|+|36288|36902 >NVVL01000006.1 Rickettsiales bacterium isolate NORP64 Contig_source1382A_1705, whole genome shotgun sequence
MPEKKDTTQDDINPHLRSRFELEMSSILEDFSSDQDLDKSMERIFQCSNTPDELESKLTI
LLSKYAENLVSQKEMQATHEIQDVVQREITKFCCNLTCNLGKGETHSSRDSLDKRCILTI
ESRKNLQREIQNFTIYLLYKVLNPKRISGETAKGNYINNLIARGKNVAGKYTGGQEKDLK
KYTDREINAMQKQAALARKGSFSR

>gene_368|GeneMark.hmm|640_aa|-|36924|38846 >NVVL01000006.1 Rickettsiales bacterium isolate NORP64 Contig_source1382A_1705, whole genome shotgun sequence
MICLNCNLSFAKENSKSKLFKLMKQHKWEKSYKLASKIGDPALGKIVLSQQYLDNKYKKN
SFEDIAKFLRANPRWPQNYLLRLRLEGRIEENTDKKLLANWFKRNRPLTGRGYKYHALTA
SFYEKNPDKLSTIIKNGWRYGSFKAEGQREYYKKFKKHLTENDHVRKIDNDLWEGNVSAA
KRSLHLVSPGYKKSFNAQIAFVQKKKKAERFFRRVSAKYYTPGLVYRYLSSKKTKPPSAS
KIDKLIKMVKSDKRYAGRFWALQSYLARESLEKKRYRDAYKIASGHFTVNAGNRSDAEFL
SGWIALSFLKNSKLALKHFRNFNRVVKTPISKSRGIYWLGRAHEKGRDIEKAQRLYNLAA

DKYPYTFYGQIAMVELKRAKMHLPPAINLEKYKKSSLAYLKNHEIARATQIVTMYGSNSL
SEKYIDAAVSGCKNTAEVMNMVTALKKAKNIHYMVWAAKAAVRKHVFIKNHAYPTPYKVS
KFPLETPLIYSIIRQESVFDKRAISSAKAMGLMQLIEPTACMTARSIKIRCRISKLTTDI
NYNMRLGSNYLGQLVKQYSGSYVLAMAAYNAGPPKLNKRLKIYGDPRKMKTLRQVVDWLE
LFPFPETRNYMQRSLEHLQIYRNIINKDNDFRLERDLLVK

>gene_369|GeneMark.hmm|315_aa|+|38921|39868 >NVVL01000006.1 Rickettsiales bacterium isolate NORP64 Contig_source1382A_1705, whole genome shotgun sequence
MNRAFIFPGQGSQSISMGKDFYDSEQSAKQAFQSVDDVLGYSLTDIIFNGTSAELAITTN
TQPALMVTSMAILRVLEEKSGKKIDQLCKLVAGHSLGEYSALCASGALSLEDTAKLLQIR
ASSMQKASPKGEGGMAACIGIGRAALQDMMGSLISEGVCQIANDNVEGQIVISGHEENID
HMIACLKDTGYRAIKLNVSAPFHCSLIKNAEEPMRAALDNATFNALQVPIISNVSAELVD
DTDVIKQNLVTQICGTVRWRETMDKMAAMGISELVEIGSGRVLAGLAKKSPHEFTVHNIA
SMEDLSKFLRSYEVS

>gene_370|GeneMark.hmm|491_aa|+|40495|41970 >NVVL01000006.1 Rickettsiales bacterium isolate NORP64 Contig_source1382A_1705, whole genome shotgun sequence
MGLDIKAPENVILKPIITVFGVGGAGGNAVNNMIKSGLKGANFVVANTDAQALTHSEALN
KIQLGATSTKGLGAGASPEVGKKAALESESEIRSYLEGSNMLFITAGMGGGTGTGASPVI
AKIARELGILTVGVVTKPFLFEGSRRMRVAEKGLADLQKEVDTLIVIPNQNLFRIANENT
TFQEAFEMADEVLHAGVRGVTDLITSPGLVNLDFADIETVMGEKGKAMMGTGEASGEERA
IKAAEQAIANPLLDQNSMCGASGVLINITGGKDMTLYEVDSAANRIREEIGDIDANIIFG
STFNPELDGIIRVSVVATGIEGTGLTHKNREEEEALEIAKNQQVTRQTQEDQAEGNLSGR
NLAEGNPASEEFASGGVASVSVSDVQPPSYNEYDDKISEGEAELQKKLEQLARQMDWNKH
QQISENPQSSAPSPAPLPRKPSLLRRMWNSLKAADSEAVPHAHTPNSEQAAMQNQADKTP
EIQEIPRFRKE

>gene_371|GeneMark.hmm|307_aa|+|41998|42921 >NVVL01000006.1 Rickettsiales bacterium isolate NORP64 Contig_source1382A_1705, whole genome shotgun sequence
MKINSQKSSKQTHKYIKHIELAPYNPDWPKLYESEAARIKQALGDNYIAIHHVGSTSVPS
LIAKPKIDIIAVVVDLQRSIKTLEQIGAEYRGEYNIPGRYFFRKRGGIDVNLHGDIDVNL
HIYEKDHPEIELNLMFRDYLRTHPAARDQYALLKQKLLQDKSSFEKNHRTFTEYTMRKGD
FIREILKDAGFSSVRIIRCSDQTEWSAAKKLRKQYFTNIKSNDPNDLESCYLNAQNAPSA
PAARRAFMDEDEHLALYKGPEIIGYSHIHLPGNNKPVIRILIIDENHHGQGFEEKFLTLI
REFIIAS

>gene_372|GeneMark.hmm|522_aa|+|43139|44707 >NVVL01000006.1 Rickettsiales bacterium isolate NORP64 Contig_source1382A_1705, whole genome shotgun sequence
MTDISGSKQTSSKSSEIPLSPLSKYAKFLDYIWPIERAELPKFLSITLLMFCILSIQNLI
RAMKDSVINTMIGVETSSFLKFWGVMPAAILITIIYVKLVSVMRGENIFYLMMSSFLGFF
LLFAFYLFPNHEMIHLNPETTQALIASYPNFKWFILLLSNWSFSLFYVIAELWPSAVFAL
LFWQFVNKVTTVEESKRFYPIFGLLGQTGLYLCGTFLANLLVINTYFTDLFTLEESQSVV
SIKIVMILVGILGVVALATFWVLNHKILDISTTENLQFRVKKNQITLKESLKIIVQSRYI
RLIATLLVCYGIAINLVEGPWKAGALKVYTTPTEWAAFTGNYLKYTGVLTVLFTIIGSNL
VRRLGWFSAAIITPIMVLVTGLAFFCIANFKFSETLIIMYFSFSDPVMLAIVIGAMQNVL
SKSSKYTLFDSTKEMSYVPLDDQLKTRGKAAADMIGTKLGKSASALLTSLIFVIIPTATY
ASISTFLMWIFIGICGVWIWAVIELNKEYKIACKNKGDETIY

>gene_373|GeneMark.hmm|195_aa|+|44859|45446 >NVVL01000006.1 Rickettsiales bacterium isolate NORP64 Contig_source1382A_1705, whole genome shotgun sequence
MRARMALHYSDIAYDLREVDLKNKPQAMLDISPKGTVPVLQLTSGEIIEESLEIMHYAIA
QNDPESICAVSKETDLEIQVLIQKNDIEFAKLLRKYKYFEKHKEETQESYRKQIEDLFLI
KYENMLEGNDFLFGKKSLADIATLPFIRQFALVDEDWFFESRYKNLIRWLNSFICNENFK
SVIMAKHPTWKESSC

>gene_374|GeneMark.hmm|187_aa|-|45694|46257 >NVVL01000006.1 Rickettsiales bacterium isolate NORP64 Contig_source1382A_1705, whole genome shotgun sequence
MLLIVGLGNLGTKYQNTRHNVGFMAVDLISDRYNLDWSTKSKFDADIASGECELGKIILC
KPNTYMNLSGISVQKIKSFYNIPISDIIVIHDDIDVLLEKTKYKLGGGAGGHNGLKSIDS
AIGKDYHRIRVGIGRPENPNHDVSDYVLGKFGAREEISIMQQLTKLEESIKLLADKDTDK
FKAAISK

>gene_375|GeneMark.hmm|216_aa|-|46259|46909 >NVVL01000006.1 Rickettsiales bacterium isolate NORP64 Contig_source1382A_1705, whole genome shotgun sequence
MSEILALTAEARKELGKGASRALRKRGMVPITIYGAGKKPVSLSVQEKELTKYYRKPQYI
SQLIEFRIGKEKYTVLPKAIELHPTTEMVRHADFVFLEKEMQKMQVPVVYTNKDVCVGIK
RGGYFNTARRLITILCPIANLPRSIEIDTSELEISASIQAKDAPLPKGAKLLGDPNFVIA
SIIGKRGKSDDLGDDDGAPAEGGGDAAKAEDSKEAK

>gene_376|GeneMark.hmm|310_aa|+|47190|48122 >NVVL01000006.1 Rickettsiales bacterium isolate NORP64 Contig_source1382A_1705, whole genome shotgun sequence
MAIFSLVFFKVLSSLLSVLMGFLSGKYSDVQKESIASLLFFFVAPIVFFAIPTSANLTIE
SLGITLLTFSLCTMLGFFSYWLYGKVWQDSHRNILALSAGTGNGGYVVLPIATALFDDHT
LSIFALGLIGISIYEASIGCYFCARSMGTFRQSVLRVARLPLLNAFFLGCLMSLSGFHLP
DFFDEFVNNMKGAFSILGMVVIGLALSNIEKFKFDMKFTSAAFASKFLFYPILFNAFILL
DKYVLGWYDVNDYNALQLLCIAPMATNTIVLASLYKIHPEKVATAVLLSLLFVLLYMPVM
ATLFIEGVVV

>gene_377|GeneMark.hmm|319_aa|+|48137|49096 >NVVL01000006.1 Rickettsiales bacterium isolate NORP64 Contig_source1382A_1705, whole genome shotgun sequence
VYKDMTKSTTIKTKFLVIGSGPAGLTAAIYAARAAMEPTLIHGMQPGGQMTITTDVENYP
GFAETIQGPWLMQEMEKQAEKVGTKLVHDYVERVDLSSRPFKAYTSSGDVYEAESMVVST
GAQAKWLGLPSETKFQGFGVSGCATCDGFFFKGKEVVVIGGGNTAVEEALYLTNHASKVH
LVHRRDSLKAERILQDRLLKHEKISMIWDSTLEEVVGTEDPKSVTGVRLKNIKTGDVTEL
PCEGVFIAIGHKPNTDLFKDQLDMDKEGYLITQPGSTATNIQGVYAAGDVQDKIYRQAIT
AAGTGCMAALDAAKFLDGE

>gene_378|GeneMark.hmm|484_aa|+|49100|50554 >NVVL01000006.1 Rickettsiales bacterium isolate NORP64 Contig_source1382A_1705, whole genome shotgun sequence
MSKPEGIITEDSLTRTLPANFRAEQMLLGAVLINSDLTEQVNEFLKAEHFHEPLHQKIYK
GIETLIEKGLSATPITVKSMLGNDPLYIEAKGDEYLIKLTTIAMVVINPRDYGKIIYELA
IKRHLIAIGEDIVNDSYNSTLEQTASEQLEQAEGKLYQLASEGMVDKGFAPLKDSVADSM
ASINRAMKNSEHITGISTGLIDLDTMLSGFHKSDLVIIAGRPSMGKTAFAINFALNACKA
LRRRTKEGEKPPSVGFFSLEMSSEQLAARLLSMMARVDSTNLRNGMVSEEHYNDLRKASA

ELSEMPFFIDDTPALSIASVRAKARKLKRKNNLGIIFIDYLQLLRGTGKSDNRVLEISEI
TQGLKAIAKELDIPVIALSQLSRAVEQRPDKRPMLSDLRESGSIEQDADVVMFLYREEYY
LKRSEPPSSDPKYAAWQENLDKFHNIAEILIAKHRNGAVGNVRLHYLEQFSSIGNLDKAR
MQNF

>gene_379|GeneMark.hmm|349_aa|+|50573|51622 >NVVL01000006.1 Rickettsiales bacterium isolate NORP64 Contig_source1382A_1705, whole genome shotgun sequence
MITIKPIIFGVAGTSLLPEEAQLFRDYPPAGFILFSRNIESKAQLTSLTRSLKNLYTERD
IPIFIDQEGGRVARIKPPIASKLYPTGKYFADLYDVDKSQAKDAICANYAAIMSELKELG
IDSPCAPVCDILYAGASDVIGDRCLGANADKVVDLCKSAISGIDKSSGIPFIKHIPGHGR
ATVDSHHDLPVVTTPLAELESTDFKVFKELSGEGVWGMTAHIIYTALDDSAPATCSRIMI
DYIRSEIGFNGVLVSDDIGMYALHGEVGQKHSAIKKILKELENIAETKNIEDIEDLEKIK
VYNKKLEEINPEFLESLAKVTRMSLSAGCDLILHCSGDIQEMRAVCEAL

>gene_380|GeneMark.hmm|278_aa|-|2|835 >NVVL01000007.1 Rickettsiales bacterium isolate NORP64 Contig_source1382A_2565, whole genome shotgun sequence
MSEVIEEVKEAKEAAIGGDATVVEEFAVSEESVLSEESIVEQENVATNALKESEESFKTP
APPADRPKSTSKNNKKRSPHDKLFKAIFGNEASARGILKKHLPEDLKKHLDLDHLKVERD
TFVSEDLKESMSDIVYKIKTKNQKKDGDVILFLCEHQSSNDKKMMFRLWKYAISAWEPYL
ARNEPLPIIAPLVLYNGKRKYSSYRRFWDLFTNSKLAEKIMAGKCQLIDLQSMPDEDILA
DKEFGLFKFFMKNIKRDNGIELWRECFKKFQEHIKLDK

>gene_381|GeneMark.hmm|86_aa|-|1378|1638 >NVVL01000007.1 Rickettsiales bacterium isolate NORP64 Contig_source1382A_2565, whole genome shotgun sequence
MDQGFVAEVSREAIYVLISMSAPILGVALAVGLMISLFQALTQIQETTLTFVPKIIAVYF
SMILILPYMFSKLKIFVDHIMQHIVG

>gene_382|GeneMark.hmm|249_aa|-|1668|2417 >NVVL01000007.1 Rickettsiales bacterium isolate NORP64 Contig_source1382A_2565, whole genome shotgun sequence
MNKFYLIFLSIFILTSCSDTIERLKRVGKAPEFENIELPTVEEDEDEIERRAERLKLQHA
HMQKTNSLWQPGSTRFFRDSRAWKVGDIIRVVVQIQDNASLNNSTNQKRTGSDTLGIPKL
FGKEKAIKANLSSVADMESLVSTKTTRNHNGSGSISRKEDIKTEIAAVVVKVLPNGNLVV
QGHQEVRVNYELREIKVAGIIRPKDITSDNAIRTNQMAEARISYGGRGIMSDVQTPRVGS
QVLDIVSPF

>gene_383|GeneMark.hmm|237_aa|-|2410|3123 >NVVL01000007.1 Rickettsiales bacterium isolate NORP64 Contig_source1382A_2565, whole genome shotgun sequence
MKLYKHALQLLLLQILLVLIASPAFSFGRDDFVAQTIYDLLQEKSQDKRIIFEPKYSSGK
KMDAIKTHLEDIKTITLGKFEPSRSSFRAIIHYNNGKSDVLSGRYESYILVPVAARYIKF
GSVIQSIDFTTKKTRLVSLAQGYATEESEIIGMQSKKYINIGHMFELRELARPLVIKTND
PVNITYSVSSIHLKTVGVAMGSGSVGDMLRVKNTSSGAILLGQIINKNTVQIGGNNE

>gene_384|GeneMark.hmm|262_aa|-|3350|4138 >NVVL01000007.1 Rickettsiales bacterium isolate NORP64 Contig_source1382A_2565, whole genome shotgun sequence
MTIALHTAVTGAEAQQTRINIIAGNVAAVNVDGYKKQEASFADIFYTNLKKAGIIENSEA
SPRPIGAQIGMGVKLVGTYRNLAQGPLNQTFRALDMAIAGGGYFAITLPNGRVGYTRAGN

FQKDAQTGNIVTSKGNPLTNGIAIPEGINVEDIQISDNGLITAQDPDNPVGLIEIGQVEL
FTFPNESGLEAIGDNLLVETVGSGEAVNIDDLTGRIKQRFLEGSNVQAVEELTKLIEAQR
AYELNTRVISTQDKIMETANKI

>gene_385|GeneMark.hmm|265_aa|-|4323|5120 >NVVL01000007.1 Rickettsiales bacterium isolate NORP64 Contig_source1382A_2565, whole genome shotgun sequence
MESFTTKASKLFCLIFLSGLLGFAQCACANIASYISLSSQIARQTQLDVIANNTANANTV
GFEQDNLLLRPVPLKQDKKKDISFVWAETTYRSGDDGPLRRTNRPTDLAIAGSGYFKLRT
PKGDRYTLDGSMIINNQGILVNHSGFPYLSIEGAEIEIPNEWISIDISQNGVIFVDEEEV
GQIGVFQFESADPVIKEGKNLYAIQGKDRVLEDFTVISGALRGSNVNATLAMAKMVEMQR
AFGLVSGLVSKIEEVERSVISKLAK

>gene_386|GeneMark.hmm|207_aa|+|5290|5913 >NVVL01000007.1 Rickettsiales bacterium isolate NORP64 Contig_source1382A_2565, whole genome shotgun sequence
MAEKKGENEEEPLTPDEAKDGAKGGDKGAKGEDGENAEDPASAKKKKIKKILIILAPILI
IALGTLYFFLVIKAPKHDDAAALDAHSAERGDGHGGGHGANPIKAKVNTYLDIDPITVSL
LSTRIKKEYLKLDLTLRLSSEHDSTTLETKMPIIKDALITFLKTLRKSDFNSSSSTIYLK
EEISKRINKIAAPIMIKEVLFQEITVN

>gene_387|GeneMark.hmm|318_aa|+|5920|6876 >NVVL01000007.1 Rickettsiales bacterium isolate NORP64 Contig_source1382A_2565, whole genome shotgun sequence
MAEKEKKPDDKETKNKNNNNDDAHGSGVKEMLEQSLQSYDKLPMLEIIFEKFIRKLSTAF
RNFTSEPMNISISESTSMRFGNYFESLKNPSSIVVFKAVEWDNLGLLILENDMVFSFVDL
LLGGKKNTVQNKKEDKERILTSIEQGLARQIAELTLLELGQSFDQVTPTTFAFDRLEINP
SFATIARPGDAIIVLKIKVEVDEQVKNLELVIPYKTIEPVKEQMQQIFLGDKFGADHEWE
DMLSDSIYGVELPIEVIINNKTSTVEEICKLKIGDTIVMDHAEDKDVIVRSGQIPIFTGK
IGKVDNKVAINLKNLYEE

>gene_388|GeneMark.hmm|184_aa|+|6882|7436 >NVVL01000007.1 Rickettsiales bacterium isolate NORP64 Contig_source1382A_2565, whole genome shotgun sequence
MLLLDFLLLIMVTICVGYCWVLNRRIHDLQNSRIEFARMIKELNASIVKAENNVNEMTEL
SKVTSTEIKVVVEEAKEATLELAAITEMANQLSEQLSAQFSFDDSKEEEEADSEANSLHS
KESEKFTDEDLAVPAGKAEGNGKSYTNDLKNFITNIVTKKPEDQGVNLNQMNYYDTLRKI
NAHK

>gene_389|GeneMark.hmm|218_aa|+|7433|8089 >NVVL01000007.1 Rickettsiales bacterium isolate NORP64 Contig_source1382A_2565, whole genome shotgun sequence
MKASLFSPILILLIITLFIKASSLFGRIQEQTSYALDTSATSMLVNTAYAGGHKKKAPPP
EPKKMLPAMPKKAKKSTGTSMVNNLTRSEVNLLKELSKRRDKIDKEKKALKLREQVLKET
ENKIDKKVTELQALQSQLEALMKKYEKKEHGKILSLVKIYETMKPKDAAKIFNELEMPVL
LRLVSNMKEVRVAPVIAGMNPSKARELSIELAKQKPIE

>gene_390|GeneMark.hmm|261_aa|-|8363|9148 >NVVL01000007.1 Rickettsiales bacterium isolate NORP64 Contig_source1382A_2565, whole genome shotgun sequence
MDKKVDMNREISMNRKKVSINRMVTMKKQIFAFIAALIIAAPFSGYACDSKKEGTYLNIS
ASTHSTAGNDLLVAKLRFEAEGDDAKALQNTVNKVMKLALAKVKKLKDADTSTVSSVYKS
SSRGKKDAPRKIIWHANQTMTIKGKAFEEILTLTGKLQNMGLTLRSLHYVTSLEKEEEIR

DSLMEKAIVKLITKSKRVAVAMGKKDIEIKSINVDAPHFPMNPAYGQEVSLMGASSKNAP
SASPGTNRVTMTVRASVLIKD

>gene_391|GeneMark.hmm|555_aa|-|9176|10843 >NVVL01000007.1 Rickettsiales bacterium isolate NORP64 Contig_source1382A_2565, whole genome shotgun sequence
MLTSLHIKDFILIEKLDLDFNEGFCVITGETGAGKSILLDSILFCFGGKTSTSLVRPGAD
SCSVAISFASSDAAAKYLSDIEIEAGSEIVIKRVQNVQGRKKFFINDQLVSAKATGQLFG
CLLELHGQHNHTKLVTPAAHLEILDEYGGFSAELRELAGSYRNWQDLEKEIKQLAKDREA
LAEEIEYLAHSCEELQKSQVQLGEEDSLADMKRKLQNRDQEIAKINDILRDLDSSSIEQI
VARSQRSIDSFGEHDLLEKVNSNLEIMYDKIEDSKTILTQILQEFQNIDQSAEEVNDRLY
EIRTLARKHSCAPDDLPDFLAKSEEQLERLEARLANSANTQNQAKQAKVLYFEQATSLSQ
KRSAAARELAEKTTKELGSLEMKKAIFKVEVNSPGAPGAPGASEKVATLKGIDSVRFIAS
TNPGMAPEPIDKIASGGELSRFMLALRVALFDKAAKQTIIFDEIDVGISGSVADSMGERL
KLLSSVVQIIIITHQPQVAGKADQHILVEKTQHDTHTSVVAGNLGAEGRSLELARMISGK
RITKTSIEAAKELII

>gene_392|GeneMark.hmm|247_aa|-|10884|11627 >NVVL01000007.1 Rickettsiales bacterium isolate NORP64 Contig_source1382A_2565, whole genome shotgun sequence
MNYIKSLSLIALASFALVSCKSKPKDTDAITPAAEIYQAGVGMLAEKQYKNAALEFEKIF
FQHPGNEITPRAELMQAYSLYLDGEYDEAVDVLEIFIKLHPRHSDIAYAYYLKALSNYTQ
ISNVKLDQSRTRYAREGLREVIKRFPGTKYATDAALKIDLVNDHLAGKEMMVGRYYLNKK
NPIAAIKRFQTVIDKYDTTSHTPEALHRLVESNLMLGLTEEAKKYAAVLNHNYHDSQWYG
YSYNLLK

>gene_393|GeneMark.hmm|454_aa|+|11809|13173 >NVVL01000007.1 Rickettsiales bacterium isolate NORP64 Contig_source1382A_2565, whole genome shotgun sequence
MLHVFNIMKELFSRVFTALLVIQLYIFGACAGDINYTYSKDQGLYVNFDDLGKVKIGADV
ECGFVRDGALYASFGVEAQIFKCSKVVLSFDGITNNAEEAGNWFFKLDVAPLYESIKVEK
KERKIYKKDSKGNDIDGNNHYVDWYKNLGSRLYYLGNIPLIEAYIGYKNTQHQIKLGRVK
TIMGLADEEVFFGDDAKFAPMGHWLTRDLFSGIDYGFSHSLADINVALYSGGNPTKGYSY
YLGGVETPNLKSNNTPSFAGKVKLKYSEFLGEDIDGYVFASYLSNMTGSTWDNETQDGKR
NASVIAYGATFKFNTGNKYLNSIGFFGQYNQYISGLREKGSQNDGSKKFKDIKQSGYFIG
TELKGFDDKISLGAAYEKIDRFDYNLFQYYNFADNNPFKNSKQTSLILQAKYNISSIVSL
VMAYHKISNKALFASDILDTRKTNRMKIGLRVAL

>gene_394|GeneMark.hmm|164_aa|-|13429|13923 >NVVL01000007.1 Rickettsiales bacterium isolate NORP64 Contig_source1382A_2565, whole genome shotgun sequence
MTKEDKLIFVGVISSAHGIKGDVLVKSYTDPAQNLLSTNLVNENGEVVALKFLREKAKGS
LVCKFSGSNDRNHAELLKGTKLYCQREDFSQTLDEEEFYFEDLKNLDVRDDSGNKIGVVS
NVANYGAGDIIEIRFDSDSKEEMFSFTKEFFPHVAKDHVVFAKG

>gene_395|GeneMark.hmm|865_aa|+|14007|16604 >NVVL01000007.1 Rickettsiales bacterium isolate NORP64 Contig_source1382A_2565, whole genome shotgun sequence
MKKLLISIVIITGLIFALITASVSITDFSRVYQDFTKSARIDMPAMEKSKFRIKKFPIPY
IIIDEINQKDNIKLKNIEVHFTPLSLLMFDPKIAEIKIESAIKYINHDDVNFLTHHEFIA
ELISEGALSLQAKIGRLTFIESDGDSPLEIEDFIYESADGITHFHGKIDKSSHITGSFAQ
SGDEVMFKLNMDDYHYNVSIEEKYKNAALESGKVKIVTDRLSTNLSNLIPDVNKIAHMLS

SSENVTITFDIKPTQNHTEFKNILITSSFLSGKGEAILSTDVKHASVVDFSFDQIDLAAW
SKDRAPEMKSKDNRGEEEHHHTIYRKFDFNHNLKLNITANSVKLNEHSTLSKVNIAANIG
EGALLIEDFSGKIDQEGKFNITGGVVQNSFRSLFNGKIKLHHKDLNDFAELFYGKESLAK
NAIPFYLSSDIKLSSVDLSLRNLLLKTNEAEMIGHISTKFIGNNTRTNATVKFSSMSLDD
NNFPVMPGMLQKFISFADGMKKPDYLSKFTPIRQINSISNYDLSFDKLTYNGRLYENVRF
NLELSPSRASLEQLVIHSGDEFIDTTLTLETNDIKPTLTITIHNAYARANFLSAPSLLAL
KNRILEHFDLSKINIIMDCSFKKLYDDNVVLEKVLFQAKSNKNLLRISKFSANLFGGRLQ
SSGSILLEPYTLNFVYALNSAQIDEIAKLFPKNFLNIGGIMSANGAWSTRGEKPNQLLYN
FYTKSDIIAKNIKINNFSIDDFVKKINLPSYNRANLSKDIKKALLTGSTTILDLKTSVEM
TKGILKTTSAAFKTNYSAGSSAALFNIYDFNLDSSSIFSFYLTAPKNSSASNAEPTQIGI
TAKGQLFSPKKEATYNKLEKALKAR

>gene_396|GeneMark.hmm|380_aa|+|16799|17941 >NVVL01000007.1 Rickettsiales bacterium isolate NORP64 Contig_source1382A_2565, whole genome shotgun sequence
MTENDNQDTDLMVAEAEQTEEDAKVEASMAIIKAVKTAEAAELAKSRPPAEDTALAELVE
PESHEEEEGEYLSHDPLICGTLAVKSHAIEFLQNYLPAKVQESVDLSTINVEPESYVEDT
LRKKMSDLVYSVKTKDGRDAFIYVALEHQSTVDRMISFRLQKYMLLLWERHKKNKIPGKL
PIIYPLVLYNGKEKYTAPLNFWELFEDPVLAKEVMGGDYTLIDLQSMHDNEINYNNVTSL
VLSVMKHIHDDDTLNMLDLLFGRCKVALSLDKKEDYLLLRMVIEYTNPKVPVEKRKRLEK
VIKNHLSKKEGEIAVKTIKDSYKEEGRVEGRVEGAEHKSLQIATNLLNQSLDKSFVASVT
GLPISEVTQLQSNIHSAGSS

>gene_397|GeneMark.hmm|429_aa|-|18081|19370 >NVVL01000007.1 Rickettsiales bacterium isolate NORP64 Contig_source1382A_2565, whole genome shotgun sequence
MAGKEMTEEFAELKINNETYKLPIRKSSIGPDVVDVTKLYAETGYFTYDPGFMSTASCTS
KITFIDGGKGVLRHRGYEIGDLANNKDFLEVSYLLLKGDLPNAKEYKGFKDEIIKHSLVN
EQIRNIFGAFRHSAHPMAVVLSVVGSLSAFYHDTIDMNTAEGRELCAIRMIAKMPTISAM
TYKYSLGQAFVYPDDELPFAENFLHMMFATPNKKYKVNPVLAKALDKIFILHADHEQNAS
TSTVRTSGSSGSNPFASVCAGITSLWGPAHGGANEAVINMLREIGTIKNIPKFIEKAKSK
EDPFRLMGFGHRVYKNYDPRAAVLRDTCKEVLAELGQLKGNPTLEIAMELERIALNDQYF
IDRKLYPNVDFYSGIIYQAMGIPSQMFTVLFALARTSGWISQWKEMSEDVEFKITRPRQL
YTGHVSRKV

>gene_398|GeneMark.hmm|447_aa|-|19881|21224 >NVVL01000007.1 Rickettsiales bacterium isolate NORP64 Contig_source1382A_2565, whole genome shotgun sequence
MSRRKVEDSAEANTEVIEKLVAKTRENSEHDNPFPNQLGGDGGNGGKVSFNSSEDAVSTP
LPPLTFATFNACTDTRVEEGSNNITHSEWRAEARMPKVLETLDKLGTDIIAIQELRNCPK
AKPPFNSTGPVNDHLEHSGYTTSVTFYRDGFQSEGASRYILAFKESRFTHIETETKYFTQ
SPDKFTDDYFASTYTTLNGKQEHVEAVKDHNFGMFFERGIQVTRLFDKETRETLCIFNVH
YPIPKDLRIKASELLRDFAQAEQELHPNIKIIIAGDFNSFPNWGGEEQMKPLIEAGFVDA
LTDATFPNETPADSTFYAFPYDLSDKQQKLNAAIESWKEQGLSTAELALNIDNFYLEEGR
KSCEIIDHILVLQQEGSMQNTVVQVQPLFDNFTGYEPEAIGEYVFSHAHGGDSELGPAFA
SDHMPVSTTIVWDREVPALGDNSGGDF

>gene_399|GeneMark.hmm|141_aa|+|21722|22147 >NVVL01000007.1 Rickettsiales bacterium isolate NORP64 Contig_source1382A_2565, whole genome shotgun sequence
MTQAQIPQILSQHLNTLSLERNVAAFDDSLTPNIDISGDYNTEAIDTSKWEVTVDLRVEM

SYKNGLALSIKLQYVATIDATDVSDADLDIFLYLEYPPMLLLELRKVVADTTMAAGVHQV
MLDPWDLSRQFAAELEGNKNG

>gene_400|GeneMark.hmm|345_aa|+|22153|23190 >NVVL01000007.1 Rickettsiales bacterium isolate NORP64 Contig_source1382A_2565, whole genome shotgun sequence
MSSEKPGWVKHTWKTLQRSFTLPSIMLKGAAGGNIALNVASVAKTYGRFDTATAINYLNE
QTSGMQPKKKRRIKWGKIAHDVIVPTRLVAAGFSALSLQAKKAEIGTNSAFARGGLRAIR
LLSGIAELALHAPELATTPIAHLARATAKAANKGRLCVARKLLSENTTQDEPEQRPNVEI
KGTSLASAAAIRRKRPPRMQRRQVSFSESEMDYSDSDEDFSSDEDLSFDPDIQEEAPAIT
ERAYHPALDSDNTSKVDLAETKAAPAQDSGATTRRITSSDKETSSLDKEIVSPAIITSDS
KKPALAEIKTILAQHVSNETKIKSPGITPFTRREHKGKTDGTPDR

>gene_401|GeneMark.hmm|936_aa|+|23333|26143 >NVVL01000007.1 Rickettsiales bacterium isolate NORP64 Contig_source1382A_2565, whole genome shotgun sequence
VTKDLERTGFLFGSNSVFIEELYQKYLEDPASIDPSWIEFFSTQTEFTPLKTTSKIIIKD
APKTQNKATSTSAPASALKEDSLRAKFMIMAYREHGHYLANLDPLNLETKKTREELKLTL
EDFGFTKDSLANEIDVTGELSGVSACTVGELVSILDRTYSGSLAAEFSHVESSGEKEWLY
EQLENANTHPSFSLEDKKKFLQDLVEVEGFEQYLHTKFSGAKRFSIEGGDASIVCLDRVI
ELSAASGAEEAVLGLSHRGRLSTLTKVMGKPYCAVLSEFMGTSAFPTNLNISGDVKYHMG
FSSDKVTRNGHKIHLSLTPNPSHLEAVNPVVAGKVRAKQDCMGRDRSKVVGILVHGDAAF
CGQGIVAESLVMSGLVPYEVGGIFHLVINNQIGFTANANDSRPGRYCTEVAKMVGAPILH
VNGDDIESVLLATKIACDYRTKFGRDVVVDIVCYRKYGHNEGDEPMYTQGQMYNIIKTKQ
TPAAIYAEKLVSQALVDAEYYPGLKQTFKTLLDSEFAQVEEYKPRAQWLEGHWSGFERSN
DAKLTTGIKQAELKKLCKSLCTIEDSFPLNKKLAKLFASRVESVTNKNTLDWASAEQLAF
GTLLKNNIPIRMTGQDVGRGTFSHRHAVLHSQIDKSIYEPLNNISDDQAFFEIADSNLSE
YGVLGFEYGYSLVNPKHLVIWEGQFGDFCNGAQTIFDQFISSAETKWLRLSGLVCLLPHG
FEGQGPEHSSARLERFLQLCAEENMQVAYPTTPASIFHLLRRQVMRNFRKPLMIMSPKSL
LRHKLAVSDIKDLDEGTSFEPVIDEIDADVSAKKVKKVIFCSGKVYYDLLEKRRASNIND
IAIVRLEQLYPFEQDLIVKIIKKYSNASTYVWCQEEPKNMGAWSYIRPYMEASLEQTGTK
KMIRYVGREEAASPAVGYLYLHNKQQESLVNKALGI

>gene_402|GeneMark.hmm|424_aa|+|26133|27407 >NVVL01000007.1 Rickettsiales bacterium isolate NORP64 Contig_source1382A_2565, whole genome shotgun sequence
LEYSKERKIMSVEIIVPALGESVSEATIAKWYKKPGDGVKVDELLLELETEKVTLEVNST
AAGIIESIEVGEDDTVEVGQVLGKIKAGAAPAESAGGTSAGASTQATTAAPAPAPQSGKS
VDPAKTMPPSVQKLVTENNLSPANIPATGKGGRINKGDVLAALNNPAAGAASSAGVASSA
GAGASTAPIADEKPVQRVRMSRLRKTIAHRLKESQNTAAILSTFNEIDMSNVIALRKKHK
DAFQEKHGAKLGFMSFFVKAVISALKAVPSVNAEMEGDDIIYKHYYDIGVAVGTEQGLVV
PVVKGADKMSFSGIEQEIVSLATRAREGKLAMSDLTGGTFSITNGGVYGSLLSTPIINPP
QTGIIGLHNMQQRPIAVDGKVEIRSMMYIALSYDHRVIDGKDAVTFLIKVKDAIENPERL
LLDL

>gene_403|GeneMark.hmm|234_aa|+|27535|28239 >NVVL01000007.1 Rickettsiales bacterium isolate NORP64 Contig_source1382A_2565, whole genome shotgun sequence
MSKSHIFLPIMAVVLFIILLWSFGFSSYFNIDTLRENQLFLKNHVANNFLSTFAIYFLLY
CAIVGFSIPIATLMTLVGGFLFGQTIGTIAVVTAASLGGCIIFSGVRLVSKNITNKRLGK
WIKQMQQGFAENAFLYMLTVRLIPIFPFVIVNLVAGILQIRFRDFFLGTLLGIIPSSFIY

VSIGVSLQELLARPDFSLYGLLEPRFFLALTALGILALLPVIYRKRRKADASED

>gene_404|GeneMark.hmm|62_aa|-|28321|28509 >NVVL01000007.1 Rickettsiales bacterium isolate NORP64 Contig_source1382A_2565, whole genome shotgun sequence
LQVLSLSVKMALSLGVMFMKKNPKLTAIDIANYFLVLVDREAGDVITHLKLLRLQEGVRI
FC

>gene_405|GeneMark.hmm|463_aa|-|28691|30082 >NVVL01000007.1 Rickettsiales bacterium isolate NORP64 Contig_source1382A_2565, whole genome shotgun sequence
MVQLIEFSYLISAICFIFALKALSSPRSARSGSIVGMIGMGVALIATFNLPSFAQKIPLM
IAISSGGIVGAIIARKIPMTEMPQLIAGFHSFVGLAAVLVAYTVLLSPAHFGIGVSGDLP
VGALIEMSLGAAIGAITFSGSIIAFGKLQGLIGSRPIKFFAQQYVCLLLSLSIIALIYCF
TQSESLLIFTLLVTVSLVVGVLLIVPIGGADMPVVISMLNSYSGWAAVGIGFTLGNSLLI
ITGALVGASGAILSHIMCHAMNRSLFNVIFGAFMKARESSGDSVEDDRIAKISSAEDAAF
LMQQADSVIIIPGYGMAVAQSQHAIKEMTDILRRSGVRVRFAIHPVAGRMPGHMNVLLAE
ANIDYEDVLELDEINNDFSSTDVALVIGANDITNPSAKTDKTSPIYGMPILDVEAAKTVL
FIKRSMAPGYAGVQNELFFRDNTLMLFGDAKKMVENIVKSMEA

>gene_406|GeneMark.hmm|132_aa|-|30094|30492 >NVVL01000007.1 Rickettsiales bacterium isolate NORP64 Contig_source1382A_2565, whole genome shotgun sequence
MREQLPSAIKEAAEISQKAAELTEKLRDISLHAPEVTGHVVDPLVFAVTIFALSCFIGYY
VVWKVTPSLHTPLMSITNAISGIIIGALISASSAEFGFSSALGFIAAFFAAINIFGGFI
VTERMLEMFKKK

>gene_407|GeneMark.hmm|375_aa|-|30497|31624 >NVVL01000007.1 Rickettsiales bacterium isolate NORP64 Contig_source1382A_2565, whole genome shotgun sequence
MKIAAIKERVKGENRVAITPDTAKLFTQKGYEVFVEQGIGDKANFSDLEYEKAGATVSAI
PLEILSDADIILKVQATELVDEFNEVAMAKAGALIIGLLSPYTNSKYLKKSLEKKLSTIA
MELVPRVTKAQNMDVLSSQSNLAGYRAVIEAAYCYDRAFPMMMTAAGTISPAKTLILGVG
VAGLQAIATAKRLGSIVSAYDVRAATKEQVESLGAKFVYPSESGSDAEDKSGYAKEVGED
FAMIQKRFLSEIIAGFDIVISTAQIPGKRAPMLLTKDMVAKMKPGSVIVDMATSSGGNIE
SSSADKIVSKKGVKIIGWTNMAAKIANDSSKLYAKNLYNLLNYAIKSGEFDFKDDLVEEM
LIGKNGMIINKKFKV

>gene_408|GeneMark.hmm|229_aa|+|31721|32410 >NVVL01000007.1 Rickettsiales bacterium isolate NORP64 Contig_source1382A_2565, whole genome shotgun sequence
MQNKTTKSHFALWQAIIALMIILNIQTSSAKDIAGGNIVSGNISKKIKSYISNIGSIAVE
FKQTDQTGSKAFGMLIIDKPYKFRCNYYRPFPIVIVGNKNYVSVYDYEMGHLSRIKTRDN
IFNFLLVDQIDFDSHFEVLLAKEHGNNYIIRLKHNNLNKVSEISFNKKSKHITGMKIFED
NNVITLTFGKTVQIKDAARKLFTMKDPDIFGKPERFNRAKLEKNFKKAS

>gene_409|GeneMark.hmm|522_aa|+|32600|34168 >NVVL01000007.1 Rickettsiales bacterium isolate NORP64 Contig_source1382A_2565, whole genome shotgun sequence
MLKSDSTYIPFARKYRPSDFSELLGQEVLVKTLTYCIKNHRLAQAHLLTGIRGVGKTSSA
RIIAKTVNCTELKTSDEQITPCNKCKNCESFNNHNHPDIIEIDAASRTGVDDIREIIESS
EYRPLIGKYKLFIIDEVHMLSKSAFNALLKIVEEPPEHVIFIFATTEVQKIPLTVISRCQ
RYDLRRLTFDEINILIKRIAKQEKIKIDDDALKIIAIKSEGSARDATSMLDQAASYVLNI

GDSEIITAENISKMLGLLQTSTMVKLTQLIIANDPASAIALLEEIYSNASNLEYFVQTMS
DFMAELSKSKVITTYHNPLYQSYSKEIADILVGTSLSRLSILWQIFSKGTQEIKLSHNEL
ITAEMLIIKAIYACNLPSAEAIMDASKASGHVPASSKTSPALPDIKQNEEENIFNFLKYC
SKQKEMEIYYWLLNDMELKNFSDGKMEIAAREANAFSGKIESLLKDWSDSDWKIAFSKQS
DIVSLKDRMMEKVKNAGDYQVIKTHFPNANISDIILRSYEAT

>gene_410|GeneMark.hmm|105_aa|+|34244|34561 >NVVL01000007.1 Rickettsiales bacterium isolate NORP64 Contig_source1382A_2565, whole genome shotgun sequence
MDINQLMKQAQEMQKQMQSMQEDMAAQEFEGKSGGGLVTITMTGNGEMKGVSIDPTLLKT
DEKEILEDLIVAAFNEAKTKADEKSKDSMSGAFGDLGNLPSGLKF

>gene_411|GeneMark.hmm|199_aa|+|34585|35184 >NVVL01000007.1 Rickettsiales bacterium isolate NORP64 Contig_source1382A_2565, whole genome shotgun sequence
MQKITGPDQLIYLFSKLPGLGARSARRIVLHLLQDKDLRLSGLIDGLQDAAERMKNCKIC
GNIDYTELCSICSDETRDGSIIAVVETVAELWAVERSEIFKGRYHVLGSTLSGSTIKNPN
ILKLPELLDRCARNNVRELILATNSTLDGQTTSYFIAEYFKDSNIDISRLASGIPVGGEL
DYLDEGTLSAALESRKSFD

>gene_412|GeneMark.hmm|147_aa|+|35519|35962 >NVVL01000007.1 Rickettsiales bacterium isolate NORP64 Contig_source1382A_2565, whole genome shotgun sequence
MLKNIVIALFAAVLLSGCSSSTGRYGSETQKHITEFEKVVGDRVHFALNSSSLSPMAKKT
LMRQAAWLKNHTAFKIRVEGHCDERGTTEINIALGESRADTVKRFLVSKGVSANSIDTIS
YGKERPAVIGNNESAWKMNRRSVTSIR

>gene_413|GeneMark.hmm|44_aa|-|36042|36176 >NVVL01000007.1 Rickettsiales bacterium isolate NORP64 Contig_source1382A_2565, whole genome shotgun sequence
LNRHALGATDFESVVYTNSTTEANIQEKYSGRIIVQTYPASQEQ

>gene_414|GeneMark.hmm|872_aa|-|36356|38974 >NVVL01000007.1 Rickettsiales bacterium isolate NORP64 Contig_source1382A_2565, whole genome shotgun sequence
PEKAVVPEQPTPAEPESSDVSLPVGDDASSDPSVGQPNMQHDESKHDDIVDSKNDDIVDV
TKPSTSEFSETPQPRLRPAQPSVASEPEEGTEISDLPQPWEEEGALAELKSNPQDGSGSN
DDAEVEAEPAIIPGFSPPDGKTPGGEEIAILPEEVREGAALDTGGAIPQPPEVFDKEILD
GARELSDLMPEVKNSPEREPAVAEEEAEIDVSSEDASTQDIHTRALKYFDNDHAIKRRIR
NLETRARVLKYLDISDSWEELEVYLDEAIDDLEEIGDYLEELDNPSERSQTILDKLEKFQ
GDLEATYEAFREEHSSLNPNEIFSDELKRLNHLKTKHLSQNEMDALTSAIEHVTEAQSAY
SDLEQYIQNLNEEHQKSADEISQALQLTEQESSLHMLATEDKIELTGEALKRTTQEAVEV
FEIMQLAMDRFNAVMERDESNIAELTERLALRKQKFPLHQKAGAESRERAIDAKHEMMRE
LEAAKAAQAPRETGSEARKQARKNAALGNEEWRGQREEHAEALEKRIREAREPREASREI
GKVNESPNLSSEALKKQRSEAHAEVRAKIDRRAQELDGETQERNQIEEFLKMQSGLQHLE
TKKLIEEGKANIGTYQQEDEAYELAARNREEAIEQKSPPIPEPLNFPESPPPDRSAPLNI
NHDRFASQMEELKQRRDVARGLDEARADLSELSSPQGESGAREIPEDLHNNYEQLDELIT
KSEQLSIPNKYDAYTMYGQNVPKDRLEEIREATVAQIDQRMTAIEDIQRTLDNAQNAEFQ
EEHTAPKIQLSAAAPEETKSPAFRESGALSSSEAPQALAHSANADHTEKVIGRVTVKVRA
SLEAVRHLEPVTVASPSRGRGSGAEQGAQQSK

>gene_415|GeneMark.hmm|503_aa|-|83|1594 >NVVL01000008.1 Rickettsiales bacterium isolate NORP64 Contig_source1382A_2665, whole genome shotgun sequence
MGVVERRGGTLLSDYVRSDEKVTIQCENNHEFEMTPSDVKRGRRGRWCPKCSGCCPIQAE
ERLKTVILERGGTLLSNYVKSLEKVTIQCENDHQFKMTPSSVKSKQWCPKCSGQCPIQAE
ERLKAVILERGGILLSDYVKDNEKVTIQCENGHEFEMIPNSVKSGHWCPKCSGCCPIQAE
ERLKAVILERGGTVLSNYVKSLEKVTIQCENGHEFRITPSSVKSGRWCPKCSGKCPIQAE
ERLKTVILERGGTLLSNYVKDNEKVTIQCENNHKFDMTPNSVKSGCWCPKCSGHCPIQAE
EKLKTVILERGGTLLSDYVKGKEKVTIQCENGHEFMMPPSDVKSGCWCPICNESKGERKC
RVFLEKMNIEYVREATCKYLGILEMSRRRYDFYVPIYKLLIEYDGVQHFEDIAFFDNVPL
KERQRIDERKTHVALEYGYNILRIDYTNLSTRQIERTLELTFEYINKGMVNKPSVFLTDI
FKYSYLNFKECMWIIPETVDNEI

>gene_416|GeneMark.hmm|485_aa|-|1728|3185 >NVVL01000008.1 Rickettsiales bacterium isolate NORP64 Contig_source1382A_2665, whole genome shotgun sequence
MEFVQSKYHNLSVNVAEGLGASSSDYVVTNDVLTILCKNGHEFEMTPSNVKSGHWCPKCL
ATRAEEKLKAVILERGGILLSDYVRNYDRVTIQCENNHEFRMTPNSVKSGRWCPKCSGWC
PIQAEERLKAVILERGGILLSDYVKNKEKVTIQCENGHKIEMTPNNVRCGQWCPKCSGWC
REYPNNCPIQAEKRLKTVILERGGTLLSDCIRNNDMVTIQCEKGHEFEMTPSSVKRGYWC
PKCSGKCPIQAEERLKTVILERGGILLSDYVKNKEKVTIQCENNHKFEATPSNVKSGYWC
PKCSATRAEEKLKAVILERGGILLSNYVSIHKKLTIQCENGHKFVMTPSTVESGCWCYGC
LGDSRTQNEEKLEAVILERGGTLLSDYFRIHDMVTIQCKNGHKFKMTPHRVKCGYWCPKC
SDNCYTQSEKKFKAMILERGGTLLSDHVGIHRNVNIQCENGHMFKMHPYGVKSGRWCSEC
QNPKQ

>gene_417|GeneMark.hmm|206_aa|-|3529|4149 >NVVL01000008.1 Rickettsiales bacterium isolate NORP64 Contig_source1382A_2665, whole genome shotgun sequence
MSGYVLYIDGMVGSGKTSLCKRIKAFLVSEESQCKLFSRPINTKLLNLFISNPKNYAFAL
ELSCLTNNRNVRENALSFASEGGIAIVDRSVYGDIACATLQYGSGNISTNEWAICVDTFE
GIKEIEVPNEIEELYVYLNTNTTDALNNIYNRGIDSERKYNAVYLERFGPIYFEVTKNIK
VHKTGEIPMNFEEVRRLLRSFGIPSN

>gene_418|GeneMark.hmm|260_aa|+|4217|4999 >NVVL01000008.1 Rickettsiales bacterium isolate NORP64 Contig_source1382A_2665, whole genome shotgun sequence
MFRGNHKKSLLVGLNYEGTDCELGGCENDVLNIKSMLILDFGYKNGDIKVLMDKDIKIYN
QKTTECKKENTIIRELKYMFKGCKAGDSFYFHYSGHGTSVPDNDGDEEDGMDEAIVLVIE
NKDYIIRDDDINEVLMSLPKGVSVVLVFDCCMSGTICDLQYQYDSNTKTTHLVNDKVIKA
NVVMLSGCKDSQTSSDVYLPLRDDYFGALTTAFTEIVRLDKDISWEFLVEMLRNLLLERG
HSQIPQLSSSNGETFNKIMT

>gene_419|GeneMark.hmm|374_aa|+|5045|6169 >NVVL01000008.1 Rickettsiales bacterium isolate NORP64 Contig_source1382A_2665, whole genome shotgun sequence
MSEQLDDLIFTLKKKIRVNHKSLTKVKNTTMLLNSLKDLRDMVGLSELKESISLQTTMLI
ELMGSDDELGMLNVILYGPPGVGKTKVAIILSRIWLSLGYLDDNGTTKEMIDTSGKYSQK
LDTKSFVIPLLLLGGYIWAMCKYAYGIVGLLILPIIFLLVLLAMVGYSYSSSFIGENKIL
DDQIVIVSRRDFVGDYVGQTAGKTRALLNKNLGKVIFIDEAYSLINGPMDSFGKEALTTL
NLHMSMHPRRCVVIFAGYEKELKNGVLKEQPGLSRRCMWNFRCKGYNGEELSEIFFRQMD
KDGWIVTDKYDVIGLIENNIKLFSSYGGDTEKLGFYSKLDASRNSFHSDKSSNKTLNINN

IKIGLKYLRNNKNM

>gene_420|GeneMark.hmm|244_aa|+|6262|6996 >NVVL01000008.1 Rickettsiales bacterium isolate NORP64 Contig_source1382A_2665, whole genome shotgun sequence
MSKIVVSDPEKTSDGVSYEIRLPQCSTKPTSAVKLTRNLRLLQCKIVPIRTLKVVRPKYT
NEYKSSRTSWRLYKVSLSEGKGNVNISAKFQEGIEPTVVRLRTMTATPKASRIEEDGNEY
SNVDIETLTPKNNFFGNSAIVLTIETPFTFPKGNVSLKNRKTFGGFDSHGYKVTPRGKNN
RTTKVPVDIARVERSIGNANENNDFYSTKEIREIAEDASIKVGAQATKGHIIKLILENFN
TRTS

>gene_421|GeneMark.hmm|1313_aa|+|6993|10934 >NVVL01000008.1 Rickettsiales bacterium isolate NORP64 Contig_source1382A_2665, whole genome shotgun sequence
MSSKVTRIKEPSKMSLSDRIKRYEVAKMEYGSYDERTGKTLPVRYVSNFEGVFMRFGDII
LGQYRKGYNLKHAIALAFEYNKYMDLNDVYIQCYLSFIEAKLSSSDALKYINEYLVGRKE
VQMTKEDMERKIDIWDDSNLDIDGFVSRDYSAYTVIGTIMDSVKFDTSDITYTDVFTTYK
INGKVLSYYPTSANGPRIFDNMYVSEEIPYIRYKSNKDHHKIYSMDMGIDLDRLIGNKNL
STDKIFFMLLLGGPKSNSNSYAKVTIDLVSSTLELNISKSGLQSEEILKRLEKYLTCKLS
KLSSGRAKGVFYMYGDITYIREIFLDIIMTDLDGILFMNEVNMSYPDRVRLNMFYRELDK
KEPIVVEDNYIRNLSSVSFSITQMVSIGTDDKVESDGSVSRKLKPGHRYIKVDISMAATP
EMAEEFKELMSYVFEYYKSQYETLFKLYSSIVPDINEEYILTRRLATRIQRLKSIAPEIF
IKGYARMCQGGNRQPELLNKTVLPYVYDVARKAMKLHNSNQYPDREDEDLVRTFDYISQR
YNGLKLEELEDPEVVFSENVNRKVKSDNINKSIGLYTGGRKLSKFPKESIKGRKNYRFFC
PDAKYKHPTILNNTLENSDVYDKLPCCALSDQSLKTSNSLYREYYEGIKVKKSKKNKPKI
SSLKTLDVGRFGNLVKEMRDVLKSVSNKYNYFRYGVVRSKNSFVHSLLPLYLSDDDNYSE
ASFTEKEKMVVDFRLALAKSVNHSVYKQELYDRSGKEIKDMIEDTNSYFDPDLFYRGIEE
FLGLNVYVFEYDTQKKEDERSFLSSPRFAKYHSRLFRDRKCIIILKHWGSISDRRVFPQC
ELVNGNNKTGSVYVFDREFNRNMYKLFLNVNSNVMWGVENGRKVVRKNWYSRVNYLDIAK
GAMGQVIDMYGKCRAIIMKTRGGNITMVTPPTQPHDLIEYKNIDLGSITSVRDVFRQRPN
SVDIDGDNIVGLWYSCMDVPNAIYFPVVRTQITSEYRGYEIGPSSPINMRVENTTQGTGN
MNIEKLGTSIARVNTIRKTIKIFLQLVTWIYLIYLRNGNTLENLIKRLVVLDTKRRHMDS
YDLYNGDKLPLTLPDVVSIKSAYEYIDNNSRGFVGKGTNKYTFLMYSKKMREYVIYFLNS
QIKKSMGLLVNIPTEIIGVYVSSEDFKKQKNSRLFTNNFQMKMWSRAQSRRKYKKIYTKL
DYEKYDGTEKVIYMNHNGDIYTIQNINENMFGSNIGSAMSVANTWKFRGINTGQATPITN
LKHPHVLYEISKSGILEIVEDNSESSDVPLELLRYYRYNNTKSYAAMLYMSSG

>gene_422|GeneMark.hmm|1082_aa|+|10990|14238 >NVVL01000008.1 Rickettsiales bacterium isolate NORP64 Contig_source1382A_2665, whole genome shotgun sequence
MFNDKKSNIVSKKSRNVEVEARFNEYSMGRGVSVHTFQRVLDILGPNYEETNTIDYIQNI
NDGVGSYGGIRKTTQDGKKDVWITKSGTSVFKRNDYNVKLSMSTEIPTTEPEEFTHNISR
EKKRFSIDLVHSKVDMTIIYTTNDKGSRSGPEYEIELELSDFDRFDEFGTYCFYVLSLIQ
DTPYVYTKKEYNDVILGLNNSLGSGITNAGNRKNIIDSRMLTKPRNLHLSDMVYGGLVGN
NITRYNVLYKADGKRKLFVTHGNSVWLLMYPSFVSKIGNFDGTVPNIGGFILDGELIESG
NDDDNRFLIFDVLSVPVEGIPSTKIQILPFSHRMHNMGENIEMLNKLDTLTTLRMKYFKQ
ITDVSSFYKIMNEMESERGNLDYETDGYVFVPEIIHRSVSKFSSVNQYLPTRKTHNRNLK
KTADLCKYKPPNMITIDFAVYRNSDGLIELKVMDGDISTMFEPKYPLAFDGLTMVDHDNE
ITANISDGTIVEYKWDTRMLVPVRIRSDKVRANSKAIAYGDNWNMMIIPLKLSTMLGKDI
TLMSKYHNSIKSKIYNKSSKSFKLMPTLLDIGSGKGGDIGKWNGKFAKVIAVEPNKEYII

EFSSRCHSIMGFVPQVIENIRDIKKIDSSKRLILINAKGENHELITKVVKKYTGGKVDVI
SSMMSLSFFWENSNIFTNLMKTIRNNLSDSGEFIFTTIDGDKILETFKPPIRVPTDEISN
MLKLNDENITMEFNGNYINVDYKGTIVSKQKEWLVHLSDLMLELKRYKITDVSDMNHERF
VSPGANSIIKLYTSGKFVPMKATKINFKGKKIVKRVVRKNVGVGMEFVPKKTNEKAEIKT
KIIETSEIPIIIYRIKEKGSELYPMFTTLNTKTGVGHGIDSTSEVVLTIDGTDVIKVTRI
SSNRTNSFFDCILKMAYHEYASNSDYIGRMNALDNIKRDVISILGSPSKGGTQYDFVLDG
ILPFYAQKEKEGNIYVDRLGNIISYDLAKMQKAMTESTTLSDLYYETVAGFLLVDLYVLD
MNKEIDLLYFPYSQVKEKSVVIMSNSDGRCEGIGIKNKNNLIQSTFSKESDFIRMLRETS
DF

>gene_423|GeneMark.hmm|205_aa|+|14287|14904 >NVVL01000008.1 Rickettsiales bacterium isolate NORP64 Contig_source1382A_2665, whole genome shotgun sequence
MLSKSKEIVKLPWTRSSVYRLKTIGDGSCFFHALSLSYYLPYISNISNGTKFNRRQFVKD
LRLDLSNRLASKVDKFDKNSKTFYEYLSRGKLHEMSLVLDKYKLYNMQEELKSNSPVDNT
YNEFISEILDKDIYLIDIAKMDVYITGNDMDLLYKGRDSIVIGIIGNHYELIGTMNNLGI
MSTLFSSENKFISDIKNRMKIILGV

>gene_424|GeneMark.hmm|269_aa|+|15005|15814 >NVVL01000008.1 Rickettsiales bacterium isolate NORP64 Contig_source1382A_2665, whole genome shotgun sequence
MSGGDESENLSFSVTFFDGYSFRNMVEYIRSGNKVGYFIFSKTLITYIEQDNTGIVLNKI
TIKTDELVEYVCNSETPIYVGLNFDELRSFTKPIKKKDSIKLYKIKNKPTVYIEVLSGSN
SHRTENVASIRISNIEKNVVYTLPESTNSKASCVISSTNFSLMCSGMSTIKSGFVEITNY
SKGMKFKACVGTGLSERLTTFGIINEEDRVSESDLQISMLTIKYLSKLPTLCNTGTMKLY
TNLVGCLLIRSHIGTFGEIDVYIKLKEKK

>gene_425|GeneMark.hmm|258_aa|+|15844|16620 >NVVL01000008.1 Rickettsiales bacterium isolate NORP64 Contig_source1382A_2665, whole genome shotgun sequence
MDTERKLTKYTVFCETEKSWVSTGYLEEVPTVCPNNNLHKIETVTILDTVDTSLTKVVED
TSGTQGYFKISGFSFDVDAGVGVITDHHIIFNYPIKVYNLMVLPKSNNIGDSLSVSIKDV
HVGLLVGDAGKGVISADVSVGDSVIGVDDITLGVVKLGFLVKLGDPTKENLGECISIDKD
NKTITVSNNTSATYSTGDKILITIVKINNVEISTNDVLVFGAGKLGGMLVPTKTTMTVQY
TNTSTEAKRVVGFFEHDY

>gene_426|GeneMark.hmm|320_aa|+|16837|17799 >NVVL01000008.1 Rickettsiales bacterium isolate NORP64 Contig_source1382A_2665, whole genome shotgun sequence
MKNFDEMVATTITAITVISSDVNIGNLFPVIPIKRDVKSKAKGKKIIIIGTGDYGDIVSC
RHDGTERGISKDSGFFRNAISLYISCGTKYVSVKLTSKSIQMCGLKSTEMMKQISEIVVN
LINGVQSNISAITDDKDMGKRILDWFTENVSVANGKLRICKCPKFIENCAYEFLKSLMSD
YVTVADYILHVKWVTTMENVVNEKLLIVSMKTSMVNYNYGLGFKIDRIRVRDLLRANPPF
GARYINSIDHYVSVTYPYVIEDGKSENIRKKSKERNHTFLIYASGNTTQSGPHPALVKEA
YNLFKKIITENEESIICAGV

>gene_427|GeneMark.hmm|452_aa|+|18029|19387 >NVVL01000008.1 Rickettsiales bacterium isolate NORP64 Contig_source1382A_2665, whole genome shotgun sequence
MGNCKSYPEPDTSNICTKDSIGSGDTTDHTLKHFCAPINGVLGGEDIRREYCGRIGSGEW
EYQGEGGSCNYNDCNKYQDVDSGCCSGCCGISGRKVLCKRKSYLGDPLSCCLKDYAFDEN
DSKCFNEGKTCFPENRDITSSQCDELVQDYCTGVDLDEDDTSWIDRWGNGSKCIYSLNRN

LFGSSGVNVNITSGGYISPVFFNSVSGFESSSKLIDKTFKKYASQGFEVGTLPGLSGYNE
FQNILFKICESSPGICQTGLNNLCVTKTTEDLTRNVELIPWCGCYMPNGEYQKYIDTFQI
NKECTPICARTGNIKLVDGDGITPVECSNDICVIDNVSIEISNSEVGDISFSQMCGGCSG
SNVNHKEGERNLGSATCSCVISDLDINASNSSIGGINLSQYCGKKTKCYSDDVEIDCDTK
KVKKPNKIIYGIVVSVIVLVIITILILISIIK

>gene_428|GeneMark.hmm|156_aa|-|19449|19919 >NVVL01000008.1 Rickettsiales bacterium isolate NORP64 Contig_source1382A_2665, whole genome shotgun sequence
MGIELFPYETVAKMESVNFVLDKGAVLPVRGSKYSAGLDIFSNKDIQVIPPKSRIVVKTG
VKVAYIPKNHYIRIAPRSGLSVKEWLDVGAGVVDEDYRGYIGVVIHNHKPTAFTVKPNMR
ISQLVITPVSYASSISVDEATETERGESGFGSTGIF

>gene_429|GeneMark.hmm|204_aa|+|20149|20763 >NVVL01000008.1 Rickettsiales bacterium isolate NORP64 Contig_source1382A_2665, whole genome shotgun sequence
MLLNKIIEAKGGLLFDDYVEVGGNIKVRCHLGHVFKTSTHWVSTGNWCPHCEVSDMEWKT
LYIIKIKGGFLINKYGDNNLMLYIRCSNNHSFDISVDGVNSGFWCTICAHNLYMSDNVIS
RVRAEICRRNGIFLHLDNMNRSDVFVICEEGHKFKINMERLEMGSWCNVPHKSKNITKVN
PNCRHATTKRYLWRPQHPQKDITL

>gene_430|GeneMark.hmm|488_aa|+|20830|22296 >NVVL01000008.1 Rickettsiales bacterium isolate NORP64 Contig_source1382A_2665, whole genome shotgun sequence
MFGNLSLGTYIFNQRTLKFHSKKNMSLGKVIGSGAYGIVSEIKINGKMYAVKRNTVSVYS
DGISCIREYDMLLRIGDHPNINKMVYHTTSKISDEGLGEELREGNIHFIFDKCQYDILED
DSLIDNEDEIRKVIKSMLSALKYLHDNGIVHRDIKPSNILKSYTGEYELCDFGMSLYLTK
QTEMSNSVTTILYRAPEMFLSQKRYSFNSDIWSVGCLMYELLTGERLIKRIPNGVKDFAQ
VVCKIIPNAFNYLKGFVDIEMYDSETFECKLNNYDKETVDLLTKMLEVDPSKRIDASEAL
NHKYFDEKDELIDIAFCSNKIEQNIEISHKYVGISKTIKLMVKNKTYKPHSNIRSVFHCL
DIYQRYMHWRKLNGKSLKGVSLLCLYTCMYISFKYFSPIEFQYTISDIFIVPKTMCENDI
IDIESEIVTKALTGKLYNKTPLECVNDVKSHEDTILTEDEQILLFNLFVDNVGEMSSMEI
AEKFLDVL

>gene_431|GeneMark.hmm|325_aa|+|22331|23308 >NVVL01000008.1 Rickettsiales bacterium isolate NORP64 Contig_source1382A_2665, whole genome shotgun sequence
MLKLLLVGDPHFKKTNLDDMDILSFDVIKYIEEYKPDAVVIMGDLLHTHESINSIVLCKC
IEFLKQIALNVKLYVIIGNHDRINNSDFMSDIHPFTAVDMWENAKVISKTEKVTLVVRDK
EYKILMVPYVYPGRFAEAINTVDNPLDVTCVLCHQEFFGCKMGGIISAVGDKWNSNNPLV
ISGHIHDYQRLGGNIIYVGSPIQHSFGSSNCGKISLFEFSDKVKETRLDINVKKRKTIYV
DCVGMKSLKNITNSKYHIRIVIKDTKANIDSMMRTTKLPNVKITVKVVDEVFLSANGGNF
EEILIKEMGDNLPEISWYNYLSKGN

>gene_432|GeneMark.hmm|1312_aa|+|23345|27283 >NVVL01000008.1 Rickettsiales bacterium isolate NORP64 Contig_source1382A_2665, whole genome shotgun sequence
MEGISPKDLILPPPYALSPSSLAKEQFEKVINEKLRSSVPGDMAVTTDFEKVRNERHRDL
MSSVVYEGFAQDSIESYNNWIGGSLSKQITNRSILLPNGDYIRFKDVSIYPPTMTIKTVG
PDGQETSSEKIMYPSTAREMSKTYSLSVYARLEQIDSHGNVVGILHKDQDKLIPLCSIPL
MLGSRYCSLYGMSDDQKLRVFECPHDPLGYFIINGGEKLLLLRDQLRVAKPLVYIDTNRN
KIVICRITSWTLSGTTVVDIHKNKSKHLYEINLYFLGNKRGVTGTICVMQIMRILWEMSG

GSNPDAFSFIEDVVKFTKSKWRDTIRRELYVSAVYTNSQPDDIATVMKSKGKRVYTQENL
KEFNEIVNSDIMKGRKLKHAQTSLKEREEQFEDTKRGIYNDLFVHIDEYVESDEYSTFDE
LNAIKIRRGSLKKYYLLCCMVARLAEYKVCLRPLDDRDDWENKQITLPGKTLEKRFWLVW
RKSILAKMRKRAKDMHVDVNNIKRVIKHGNVTNKLIKSFTANEWTHTKGNDYGKESVSVE
LDRTSHMSVHSQHGKINTPTNRRAKQKAIRQIIESQIGFVGLAETPEGQQCGLVKQKAIT
CMVSQDRNPEIVRNFLVTKQLLSNVYDVTNENMGPCMHDGNFIGWCNVGETRKELLEAKR
NGTLKHDTSITIDGVVLYVYSNAGRPTRPLLVVGKNGKTVIENKNKWGESFRELTRNGSI
EYVDPSEQNKFILADTLGTLVSKSERDSTLANLQRDLEKIRSGNPLYRNVKKIDFNDNPY
EEKVNLTELRILKEINIIKEYEIRSSNLKFDYCELDPNALLGVNASLIPLPNHDPAARNS
YQCHMGKQSLGIFHSNQDMRFDTSSRALISPRGGLFNTQSHNWVGLDDMPSGDMVICAIM
TQNGENQEDGISFSKAAIEAGLFRYTINKKHESVLKSDTSKGITEVFGVPVKRDGHPEGA
YDFLDENGIAKVGSMLKRGHCIIGKTRIRQMPDGTVMEKDASVYYVERGSEGIVDMVISPT
PLYGGGKVIKVNIRSTRVPIVGDKFASRHAQKGVISSMVNKEDLPFTSDGITPDVIINPH
AIPGRMTIGKIIEIITSKVTATTGEKFDATAFRNFDADEIANELRKNGYSGSGKEIMYNG
ITGEPFEVMIFIGPCLYQSLKHFVADKISARGKGPLDKSTRQPIGGRSRGGGCRVGEMER
DAFIAHGAANSLHERISISSDLFTGVFCKYCGSFATSRVPLDMYGESGSTSKSVNICPRC
SQSDGIVACNISSGFYLLARTLSAAALDIVFDLRPETYSEEISRVRNIGILK

>gene_433|GeneMark.hmm|211_aa|-|27301|27936 >NVVL01000008.1 Rickettsiales bacterium isolate NORP64 Contig_source1382A_2665, whole genome shotgun sequence
MNFSKEEILVVTETTKTLVVPKGIKKLICSDIGLETLILNEGIVYVDCSCNRLTELIVPD
SVITLSCYRNLLTKLIATNNVTSLSCSNNLLTELIVPNSVTTLHCYRNKITKLIIPSSVK
YVICLCNPLEDLSGYNELIVPKVVSLYTMCHNAITSQDNIPSFFDLVDKVTCCSCKNKVV
EDMSYIQFKGCNAGYVRKIIKCRKCYLDMGF

>gene_434|GeneMark.hmm|300_aa|-|28080|28982 >NVVL01000008.1 Rickettsiales bacterium isolate NORP64 Contig_source1382A_2665, whole genome shotgun sequence
MNFSKEETLVVTKTTKTLIVPKGIKKLVCSDIGLETLILNEEVVYVDCSHNKLTVLTVPN
SVTYLKCCNNKLTVLIVPDGVTYLDCSCNRLTDLIVPDSVIHLKCYYNQLTILIVPDRVT
SLDCYGNQLTELIVPNSVTNLDCSCNRLAKLILPDCVTSLDCSCNKLTKLTVPNSVTYLI
CHNNQLAKLILPNAVVNLDCSYNELIELIVPGSVTFLDCFGNLLEDLSKYNKLIKPKVVS
LYDMCHVMVNENNIPRCFSKVYKVTCDGCGDKVVKDSAYTLCEGNREGYIRKTIKCKKCF

>gene_435|GeneMark.hmm|414_aa|-|29208|30452 >NVVL01000008.1 Rickettsiales bacterium isolate NORP64 Contig_source1382A_2665, whole genome shotgun sequence
MHFSKEEILVVTAITKTLIVPKGIKKLVCNRIRLETLILNEEVEYVSCSHNELTELIVPN
GVTYLNCSVNKLTKLIVPNGATDLNCSGNKLTVLIIPNSVTRLDCSYNKLTELIVPDGVT
HLDCYYNELTILIIPNSVTRLDCYGNRLTVLIIPNSVTRLDCHGNKLTKLIVPDSVTYLD
CSGNKLTELIVPNGVSHLDCSGNKLTELIVPDGVTHLDCYYNELTRLTVPICVTHLDCYG
NGLTVLIVHNSVTHLNCHGNKLTKLIVPDSVTFLNCSYNELTKLIVPNSVIYLNCYRNHL
TKLIVPNSTTFLDCHGNKLTVLTVSVNLVDLDCSSNLLEDLSKYNELIKPKVVSLYAMCC
EIVNEKNIPRCFSKVYKVTCDECKNKVINDSAYISCKGYVGGYIIKTIKCKKCF

>gene_436|GeneMark.hmm|167_aa|-|30614|31117 >NVVL01000008.1 Rickettsiales bacterium isolate NORP64 Contig_source1382A_2665, whole genome shotgun sequence
MNFSKEEILVVTEATKTLVVPKGIKKVVCSEIGLETLILNEEVEYVDCSYNKITKLLVTN
NVARLYCHDNKLTELTIPHSVARLYCHDNLFEDLSKYSEVIKPKVVSLYAMCHEIVKEKN

VPECFDGVNKVTCDECEDKVIKDLVYVSYRGHRDGYIRKTIQCKKCF

>gene_437|GeneMark.hmm|330_aa|-|31261|32253 >NVVL01000008.1 Rickettsiales bacterium isolate NORP64 Contig_source1382A_2665, whole genome shotgun sequence
MGIKNFTSIFKYSSNSKTIKTFDHFSDTNFAIDAHNVIYAKFKTSVKKYIDIICLDDCID
IGVIIDMTFESMMTYVKPMMAKNITPIFIFDGVPPECKRKTNDKRKAERVKYELLAKEAK
SNIDFTDIVTIENYKKYIIRSIYPDRDVMKIIMDKLQSVKIPTIQAKGEAEKLCSSLCRE
GICSAVISNDSDSLVFSAHTMIKSISRKGLECISLSDILTDLDITFDQFVDICIMSGCDY
NSNIPGIGICKSIKLIKEYKSIDNLPEKLGNIILDKSILNYVECREQFGYTESNLLISDD
CTLFDCGCENGIQQTNKLNIPFIITKLVIV

>gene_438|GeneMark.hmm|270_aa|+|32279|33091 >NVVL01000008.1 Rickettsiales bacterium isolate NORP64 Contig_source1382A_2665, whole genome shotgun sequence
MYNHNNSKSGKCQEVRYVDGGIMKTKRGLCKGNIKGVKEVPIRKVKTLNDCVPFVKDICE
IVLEEELLDIFVKHGISEEVITLLAHGLGEYATGERLSKLEHISKMYDILKTKNKYICNN
CNGGYTFEYEVHSDIDDNVDKHGYQLDSQEDLQYIKSDVEMDIVIPYEINTNDEIVSFDV
SINLFKCPIYIKSDGLNIGYPNVQSDEKNKFILGFDSEKSSGNVTAYLEDDFLVITSFDV
TLGTYSPDNITLTFDPQDNMTLDIPLQVDA

>gene_439|GeneMark.hmm|351_aa|-|33160|34215 >NVVL01000008.1 Rickettsiales bacterium isolate NORP64 Contig_source1382A_2665, whole genome shotgun sequence
MDKWEPTVLVLGSGGMKGFMEIGALVYMSKIGKLNKIDTYVGCSIGAVICLLRICDYDYT
EIMKIAYNVDVSKFININLNTIVENMGLINSNDVVSKLETLVESQYGMVPTLEQLYITTG
KILTIVTYNMTKSRTEFISRDNMPDISCVDAVMLSCNIPFIFQTIKINGDIYVDGGIGNP
YPVDIYDNNKTKILGMYIAEPKPKETTTGFILNLIHCQSREITSRIIKGSSDKCKHIKLI
SPTTDTTGLSFTHEIRTDLIMIGWNAAIKATRGEVCEVEMEDLMSSIIDNSGEEYYTDPG
DSKNTNNKIISDSQSKISVKISNKSLKVLENRKCLDLLNSDPQDNTCQRYI

>gene_440|GeneMark.hmm|220_aa|-|34273|34935 >NVVL01000008.1 Rickettsiales bacterium isolate NORP64 Contig_source1382A_2665, whole genome shotgun sequence
MEEHDALDQVKSDEYDYDSDETSTFDENDNAFSSSDVSEEEFQEYHPTTTKSESSRRLSL
EAEEEEMLEGTDIDVKVEYVELELDDTLVETKEKEHRKWAPVIKTPHSNEKIKYIGLGES
SSENKHSSVDVQETTKRGIKSPITKIKELPQNLISKNLGETDKHQNLRIDLASHIYNTNN
ISPETAMDISKCVISKVESGTIYSDGIERIIDKINKSMVL

>gene_441|GeneMark.hmm|197_aa|+|34924|35517 >NVVL01000008.1 Rickettsiales bacterium isolate NORP64 Contig_source1382A_2665, whole genome shotgun sequence
MFFHLYKMENKLDIISVVLRIVVLFKVMESLQKPGVILAGSAVLVTVASTVYFGRAVGVL
NSSVRDVEAQMEAFNKELESIKGFTIPKSIIEEGFRNFKNVLSEINSSVELLSEKLDNNM
HSISEMQDYMREDGKTIKEFVKLPRQTNRRARFEYRPDNRYMERGGRPEYKRKYKNVGAD
EYDVENAMERIRKSQGI

>gene_442|GeneMark.hmm|280_aa|-|35576|36418 >NVVL01000008.1 Rickettsiales bacterium isolate NORP64 Contig_source1382A_2665, whole genome shotgun sequence
MEHKNKVTLENFNSSNVVIGTHEKKTFKGKDGKEDGDYITIPISYKYDGNTPTKGINLEL
PLVTCYVKKGKFGYQMSIKLDRTGKVYNVIKALESINISVGRMANTKRGAFSLKEEINLN
EIYKCNFKDHIYRPRDDNTYEKLEVDPTLYLPVKSWSVFAIPVEQNGKIVSRDIKKENLE

NIKLEGHPLIKVVSMYMGGGKFSLQLYLSCIIITKVTKVSEVELMSDILGNVSGDAMVEL
GNNLAELGIPLMTDNPLNTEEDEKEEEGGDDIKAIEYKDL

>gene_443|GeneMark.hmm|412_aa|-|36468|37706 >NVVL01000008.1 Rickettsiales bacterium isolate NORP64 Contig_source1382A_2665, whole genome shotgun sequence
MNSAVDNTWLFKVKQQSGEDTKVIPGSNLVLFTDGNMSCVDGEDSVIISGRSVIGNYTND
INIKYIHTKVLSHFDNFSTNKLTSELEDLKAMTLTNMGIFVKVKERISAIEEEMATLSNF
KKEEYLKKSLRILNEYDLVGPFIKHINFDNSEVEIDPMFGVRHNIIYKYLDLVKPYIFVD
VVRNVKVSNKCKSCKNCLDNLIPQDGTLTCNVCYANIPYVTKTQTCSYNVTPSNHNNYKD
KDNFEKAFNRYQGKQVDILPSNIVSVLDEYFKSVNIMIGDDVKKMSKAKIKKETSRTMMF
NALKCIGLSSHYEDINLICHIYWNWILPDVSDLEDTIMKDYDDSQEVFMSIKGEDRRSCL
NVQYRLFRHLQIRGHECNHEDFKTITTGETFRYHQIMWEAICKELGWVYMDL

>gene_444|GeneMark.hmm|77_aa|+|3|236 >NVVL01000009.1 Rickettsiales bacterium isolate NORP64 Contig_source1382A_3623, whole genome shotgun sequence
LYIYNSMYFRASLLFYLGLVFSGSSSWLVFGFSVTSLNLGFFKFSRFPAKSKFPLIFSLQ
AFFWLCAVFSTSCLGHY

>gene_445|GeneMark.hmm|1598_aa|+|606|5402 >NVVL01000009.1 Rickettsiales bacterium isolate NORP64 Contig_source1382A_3623, whole genome shotgun sequence
MERKDKNKRSSLAKLLVTASAVAMIAGSAGSAMAVALNVNADVPDLAVASNIGLSAPFAN
GDDLTLLGAFNITTANNVVIQAINVGLFTGQTFTVGTGHTVGLTAGTMDMNVILQGVGSV
FNIGVRDVTENVTVNGGAFTARHLTGTGAGNGDLTVTAGTAAVQNVAGFVNVSGGVVTSA
GTVGGTLSVTGDGQFDGAAGAASDVAGIVTINTTSAAMNTIKDAALAVNLEAGKLTMRHA
AADVTVTGGELTMGNAATNVIVGGGKIIDFGVITGNLTFTADAEVTTAAAAGSKVTGNVT
TATDGQGTIKFTANGLEVAGVIGADGAALKLVEVSGTQQLDLTQAAAVTHYAKEFNVTAV
GDNIRVADGASLNGNVTGLGFVDFRGAGSTTGNLGTNAAALTKVVAGSNAVGIVNIGAGD
HFVTAFESKNNGASGFTFADGANVTGKIDNTQGNANGAIIFEGKSTVTGTIGGAGGVVAF
ATVSVNGDAGSVVEVNDDVSATAITIKAGTFELKDTANARIITAANGITFAANGKLKITA
PQAVVNTHGVAADIITGAVNQGNIEVTGSAIFGDIGVVAGNGLASLEATKGTIELVAGVG
GAGAGVHVKDVKLNEVVLKLNNAAATYKLGSVELEADNKSTLEVNDNSTIIAIDTAAGLS
FGEEGKVLKALNFSANAITLTLGNGVSIYAKDLTGTVVGNINTATITTQGNNTLSFVNDN
ANRLAAINVNGNAGEVTRLLGATFVDGNVLLGANTTLEIDSSITTDVAANGGGIIGNAGS
NGTVRFINEKGDKIEVTGAVGAGANTVEGIEFAGGDVKFNSKIEVAANSGFTFTAGADGE
TTIEFVAATDVDNNTFTVADTADSAAKRTVILAKAVNTFGQEIAKSKDKAITFQMSNNVN
VIVVTADAAGARFTSSDGKGDLNLNKADVTIKSVGTVGNTINGLTFTENATVSEATIAKT
TTVVAGKVANFNESLVGETAALGDGSIANFADGFILDSKVDGAADNDGILNFAGKASINK
NIGTAHRVNLINFSDDNKEVRLATDIITSRVVNIRKGTVALDQNVSMNGTINAVESTLDL
GEHSLTIENGEALTFTGENTLKIKVIEVKDTIVSGSIRGTGEVVLADANTKLHFVPVDES
KSRPSNGAERKFTLIENVQLANFDLDKVDITTAIPGALVYGKELDGTTLYGSQKDNAKGY
LQDLVKDNSSVGIPEGMLARIEKHTAAPEGTAGYDVSGVMLDIAEHFVSDSAAAAARVTE
LDNRQNTTAAATAEVIQGASSAATSSVAGRVSSIARAPGASVQRNVAYANGEGVAAGDDH
ARFGAWFSPFYNQTTQKHVKGSAGYKSKITGLSLGFDAKANDDLSVGVALTGSTATVNHK
DKKSGDKTTVKSLMFSVYGMQQITDNWYTTGVATFGTNSVKNSEKRVISATSYDTARGKF
TSMSFSGEMMFGYNHVIDDITITPTFGLRYGRVNSTGYKETGSSQNLEVSMKATNSFEAI
AGVKVAAGSYEMNGAMLMPEIHASINHDFAAKTKKADAKLGGSGELGYAGKQAIKTTYEV

GFGVNAEYGMMEYGFGYDAQFAKKRIGHQGTMKVRVNF

>gene_446|GeneMark.hmm|641_aa|+|5650|7575 >NVVL01000009.1 Rickettsiales bacterium isolate NORP64 Contig_source1382A_3623, whole genome shotgun sequence
MLQAFCRLVVYFSLLIGFMTNIFGIIIKTFKAKTSPWVSMIFSKPLLPAQAFGIGVCIIL
INLLFSPSVSFASKRDALRIEIKIEKDHRVKAPISELTKNLEKIAKIYKTSNQKSYWESE
GKGALHKFLRSRGYYSATVNSEISGKNTNSITFHIEPNERYSLSNIGIKFTTGSNLNIKI
PAIADLKLKKGTPAIAEDIFSTQKEILEYVEEHNCLLSLSVSHQAVINHFDRKLELVFFI
DAGSSAKIGEVSFQGLDKIHPDYARKLTKLKSDQCFKTSLIKAARGRLQKSGLFASTKPT
IPKTTNKDGSVPVIFNIVERKSRSVLGGLSYGTDLGFGATLGWEHRNFLGQGELVRANLF
GNEREQILELNYVKPFFKRDDQTLRLGLRLENKKSKAFEHKEGAVSAFLERKLSEYWIGG
FGARFSHARVQKKSDNSKEKLSLLSLPLFFIKDTRDNILNSTRGYEVRLEGAPYFGVSSK
QKPFFKTTISGAAYVKPSDSRLVTAFRGSIGSILGVRSASVPIAEKFYVGGGGSVRGYEH
QMAGDLDSSKRPLGGRSFVEGSLELRFHFAEKMGLVAFFDGGRAYSAASPDFGKKLLYGA
GVGFRYYTEFAPIRVDIAFPIKKRQGVDKSYQIYFGLGQSF

>gene_447|GeneMark.hmm|1172_aa|+|7575|11093 >NVVL01000009.1 Rickettsiales bacterium isolate NORP64 Contig_source1382A_3623, whole genome shotgun sequence
MKNFWRILGGMIILLVLLAVSASVWLKTNGAREKVQQLVVEAIKEQTGFRASIEGMNLSF
PLIADIERVSLSKGGEEILRIKGFHINILPSLFSLWEISVWSVSAKELHFLKKVGAGGFV
PEEKGSGDIGGASEKLGQGSGTGFFAPDIMVRKVEAENIILAPQFTGIEKEVVLSLGGSL
SFDMGAKELEFVASSSVTLLAGEALDENLVTIIGSYNVGAGDVRIKLFEVVSGYIDARGE
FELRVREERVLGEMKYSTSRLGGVLSEYIEGTKSAISGIVKISGSPDNARVSAQGNVLID
FGENEYVRLSEIDWKSEMLFSKGAMDGSLTMNQGAVHVNGGIAYKDNKFYVKNMKAVAPG
AEEVINLVFNFENSILTGDVRSEDRYLLESAKYFPFLKSGSLALKTVFYDNNNRQGASVK
GQIKQLSTKFGDCGLINLDLNSSDLWNGQIADSNVSLKFLEVGTLMVQSLKLRASSGGQG
GVLASSSGGNHIRLSGEIGCEHGCFVDLKFASRVSAPAGAGWSVAIDEISGMVGDAQVKN
SGVILLGGGKGSAFEINDLKIGRGTFNASGKISDRGKASAGGVSGLVVMSDVPMNALSEI
LPEGYQDAKANGRIELSGSGDAPVLKGRINIDGLLLPGLEKDAKPGKGVGLRIVANLTSD
RTILSGRVLRLEEGSSSGGGSKILSSKTFSSKAPSSKFLSSKAPSAGEEIARLKIDLPAK
FTMVPFEYSIDDKKNFNASLFSKNFDIMALVPAYPGHEMEGDINADIVATGTLSNPSFKG
GAELLRGEYRYRQHGVWLGGVSGKLAFNGGQVLVGKMSARDESGNMIAASGELSLMGAHK
FKVEINTEKIKLISNAYLHGEMSGRLVLSGDKEQASAVGEFSLDSMEIKIPEHFTQHISE
INIIEPETEIDAIRAKVQGERGKGRGVEPEKDDSYKLKLDIKVKADKRLYIRGWGVDALL
RGGLHITGFAHDPAAYGRVRVVRGKYQEFGKILDIKNGELTFDGSIPPSPYLNIVGISEI
GSHEIRLRLSGSILNPDISIESTPAMSGEEALSMLLFGEKAENISTFQALQLADSLRRLA
GHGGGFDPLGLGRKILGVDDISFKTDANDPKKTSVGVGKYLTDRVYFEIEKGRQESGTRT
KIEVQISPKISIKSVQEEEGNNSVGVNWRFDY

>gene_448|GeneMark.hmm|61_aa|+|11174|11359 >NVVL01000009.1 Rickettsiales bacterium isolate NORP64 Contig_source1382A_3623, whole genome shotgun sequence
MYNDKIRQNKYFYIKILWYYTEKKVPAERKEELIKIILDELPDNAGEEVVKSVADIYYRR
R

>gene_449|GeneMark.hmm|57_aa|+|11331|11504 >NVVL01000009.1 Rickettsiales bacterium isolate NORP64 Contig_source1382A_3623, whole genome shotgun sequence
LQIFIIEEGREEAVIKRNTQIVTNMLEQHFDLKHIASVTGLSASEIEELQNSAHNSH

>gene_450|GeneMark.hmm|74_aa|-|11799|12023 >NVVL01000009.1 Rickettsiales bacterium isolate NORP64 Contig_source1382A_3623, whole genome shotgun sequence
MILKERNVLLGAIYKNNNVNKERKEIIQNVLRKIKSQTRLCTFMDHFVKDIIAELVITSV
FLCAGEKCRAILMT

>gene_451|GeneMark.hmm|90_aa|+|12380|12652 >NVVL01000009.1 Rickettsiales bacterium isolate NORP64 Contig_source1382A_3623, whole genome shotgun sequence
VCGHQARLATLDLNLELELDKLGHCCYHESELGFDCVLTSCFDYLSVFLIYVYLKYKITN
IFNKACEYYSHLHIFFSIKTKLTPSFSIFK

>gene_452|GeneMark.hmm|472_aa|+|12658|14076 >NVVL01000009.1 Rickettsiales bacterium isolate NORP64 Contig_source1382A_3623, whole genome shotgun sequence
MTTQTQTQTTQTKPATQRKEINKRPSRPVRKTPGRKPVRRDVEEDTPTVPDNGVKVALKE
LKQKSPEDLQVQAESLGIENVSSLLKQELIFAILKKIVEQGGQISADGVLEVLPDGFGFL
RSPDANYYAGSDDIYVSPNQIRRFGLKKGDTVEGQIRAPKSGERYFALLKVHKVNFEDND
HAYHRVHFDNLTPLYPEEKLSLEMQGKGHDFSTRTIEIVAPMGKGQRALIVAPPRVGKTV
LLQNIAHAITTNNPEVYVIVLLIDERPEEVTDMKRSVNCEVVSSTFDEPATRHVQLAEMV
IEKAKRLVEHKKDVVILLDSLTRLARAYNTVVPSSGKVLTGGVDANALQRPKRFFGAARN
IENGGSLTIIATALIDTGSRMDEVIFEEFKGTGNSEIVLDRKVADKRIYPAMDLTKSGTR
KEELLVDRATLSKMWVLRRIINPMGTIDSMEFLLSKFKETKDNAEFFSSMNS

>gene_453|GeneMark.hmm|318_aa|+|14108|15064 >NVVL01000009.1 Rickettsiales bacterium isolate NORP64 Contig_source1382A_3623, whole genome shotgun sequence
MQKRPKIALIGGGNIGGTLAHLVAKDALGDVVLFDLTEGKAKGKALDIAQGATIENSDVN
MEGTNRYEDIAGSDVVIVTAGIPRKPGMSRDDLVATNANVMKTVGAGIKQYAPDAFVIVI
TNPLDAMVYVLQKASGLPAKKVVGMAGVLDSARFNCFLAEEFKVSVENVNSFVLGGHGDS
MVPLVRYSTISGIPVPDMIKMGWSTQARIDAIVKRTRGGGGEIVSLLETGSAFYAPATSA
IEMAKSYLYDKRKIMPCAAYLNGEYGESDIYVGVPVVIGKNGVEKIVEIELNSDEKAEFK
KSVDGVKELVGSIQMSLA

>gene_454|GeneMark.hmm|372_aa|+|15392|16510 >NVVL01000009.1 Rickettsiales bacterium isolate NORP64 Contig_source1382A_3623, whole genome shotgun sequence
MSSPDVLSFWTTSIKNTISMIKKSNNFTVRSLVLLALLTIILCNDSNAKSSCDNIAELEK
LSNQGDVISQNNLASCYYVGEGVSKDITKAIKWYRKAADQGNVYAQRRLGSLYIMGAGVS
KDITKAIKWYRKAADQGDAFSQDTLGVQYYSGSGVTKDLTKAVSWYRKAADQGYAAAQNT
LGTLYFNGEGVTKDFTQAVLWYRRAADQGHVPSQAILGVLYANGLGVTKDFTKAVSWYRK
AADRGHAFAQNSLGDLYIKGKGVAKDFTKAVLWYRKAADQWDAAGQNNLGNLYAKGEGVA
KDLAKAVLLYRKAADQGYAAGQNNLGNLYAKGEGVAKDLTKAAKWFRKAADQGSVLAQNN
LRTCLKALSERH

>gene_455|GeneMark.hmm|716_aa|-|16697|18847 >NVVL01000009.1 Rickettsiales bacterium isolate NORP64 Contig_source1382A_3623, whole genome shotgun sequence
MKEKPNLQENTITQPQPQSQPKTQGTPPIPKKVDHEFELHGTKISDEYAWLRADGWPDKI
TDKEVLSYLEEENEYFNNFITPLSAQKEEFFEELKGRIKLEDQSTYTKKDDYYYYSRTEA
DQDYTIYCRKHGSIDAPEEILLDVNELAKGKDFTALGSFSISPDHKLMAYSVDFTGGERY
DIRVYDLEAKTFLSDQIENTIGSVVWHERLSGFFYSPTDEKWRRDKVKYHALGTNSDEDK

IILHEPDPLYNVSASKSSSREYILINVSGHDSNEISYIKMDDDSLTPKMLKAREEQILHS
VDHNADYFYLSTNKGAKNFHILRAKSAKYSENSDWDVYIAERKDAYLSSFDITQNYLMMN
YLMMNYRANALPEIVIRELRSGVEKTIGFPDSAYTAGAYSTNFKEDDIRIGYSSLSRPSS
VYNYVFASEQLHLLKTQEIPSGFTPEEYEVERIFAHTDGVQVPVTLFYKKSLFKKDGSNP
LYLYGYGSYGISVPPSFRNSAISLVNRGFVYAIAHIRGGDDRGHDWYEAAKFLTKKRTFS
DFIASAETLIEEGYTSKGNIVIAGGSAGGMLIGNVVNQRPELFKAAIAHVPFVDVLNTML
DEDLPLTPGEYKEWGNPKDKEYFDYIKSYSPYDNVLAQSYPHIFITSGLSDPRVGYWEGA
KWAARLRDRKVGNSLVFSKTNMSFGHSGASGRFDYLKEAADDLVFILEMFKRPQGM

>gene_456|GeneMark.hmm|149_aa|-|18915|19364 >NVVL01000009.1 Rickettsiales bacterium isolate NORP64 Contig_source1382A_3623, whole genome shotgun sequence
MKCPFCQSANISVKDSRETDNGRTIRRRRHCLSCKGRFTTFERIQTRKLFVVKRSGTKRP
FDSSKILNSITTALRKRNFTSDQIDELTNKITLKIESSINKEIPSRTIGKLIMQELAKID
QVAYIRFASVYKDFATAQDFAKFISKIKK

>gene_457|GeneMark.hmm|419_aa|-|19361|20620 >NVVL01000009.1 Rickettsiales bacterium isolate NORP64 Contig_source1382A_3623, whole genome shotgun sequence
MTNQELSSTDPEIFAAIESERKRQETYIELIASENFVSKAVLEAQGSILTNKYAEGYPSK
RYYCGCDEVDKVEEIARDRLKQLFNCSYANVQPHSGSQANQAVYLALLNPGDTILGMSLD
CGGHLTHGAAPNISGKWFNPVFYNVDPETHLIDYDEVERMALEHKPKLIIAGYSCYSRGL
DYARFREIADKVGAYLMADIAHIAGIIAAGEHPSPFPHAHVATSTTHKTLRGSRGGLIMS
DEEEIGKLMNKAVFPGLQGGPLMHIIAAKAVCFKEALNDDFKIYIKQVIANAKILASTLQ
ERGYPVLTGGTDNHIVVLDLRKHGLTGKLASASLDRAGMTSNKNTVPFDDTSPFVTSGVR
LGSAACTTRGFREEEFRIVGNMIADLLDSLKESEENAATEQAIFAKAVELNSKFPVQAI

>gene_458|GeneMark.hmm|140_aa|-|20617|21039 >NVVL01000009.1 Rickettsiales bacterium isolate NORP64 Contig_source1382A_3623, whole genome shotgun sequence
MMKCFIKIIKSFIKISWILIMTAFVLWYIPNNYAEIKYSILDDVAYDDSNFLRHYVNKMD
IKCQDKTYKFRGDHFDEMVREIKNNPSIAEYKTVQFDTGYLNRRQNKLFVRMLNSFKCSA
DNQYQSSYELYSPDKEQEKK

>gene_459|GeneMark.hmm|215_aa|-|21040|21687 >NVVL01000009.1 Rickettsiales bacterium isolate NORP64 Contig_source1382A_3623, whole genome shotgun sequence
MTTDIKKPCECPEIAALSGKAKKLMVMLHGVGSDGNDLISLVPFLQDHLPDYHFIAPDGI
EAYDMAPFGRQWFSLQDRDPQVIGGLVSENAPKAYQIIAKKQQELGLSNADTTLFGFSQG
TMLGVYMTLMQEAPFAAMLAFSGRLITPMLLQNTQTPICIVHGEQDDVVPAQESDNMAKY
CSENNIEHRQIMVPNLGHSIDASGIEFALNFLKGE

>gene_460|GeneMark.hmm|212_aa|-|21688|22326 >NVVL01000009.1 Rickettsiales bacterium isolate NORP64 Contig_source1382A_3623, whole genome shotgun sequence
MQMNSKTIDKIFQSFYNATQKPQTELNYSNNFTLAIAVLLSAQATDVSVNKATKDLFAKY
DTPEGILSLGEDGLKPYIKTIGLYNSKAKNVIALCRILIDNYNSEIPSEFDQLIKLPGIG
RKTANVVLSCAFGKVTIAVDTHVYRVSRRLGLSSGTNVGKVEEDLLANVPTKWQKHAHHW
LILHGRYICKARKPLCSECKITEYCEYYKELK

>gene_461|GeneMark.hmm|128_aa|+|22547|22933 >NVVL01000009.1 Rickettsiales bacterium isolate NORP64 Contig_source1382A_3623, whole genome shotgun sequence

MSGEEIFDLIKKQISDDNVVLYMKGTADFPQCGFSAMVAGILQTLGIKFKDINILSDENL
RQGVKDFSDWPTIPQLYINKEFVGGCDIVREMYENGELSDLLKKSQNKGAQQLDEETESS
AGAVTEKG

>gene_462|GeneMark.hmm|306_aa|+|22974|23894 >NVVL01000009.1 Rickettsiales bacterium isolate NORP64 Contig_source1382A_3623, whole genome shotgun sequence
MSESAISIRNLVKSYDQNVTKAVDDLDLTIPKGSIFGLLGSNGAGKSTLINILAGTVVKN
SGVVKVMGVDLDELPKKVSSLIGVVPQEIVFDSFFPIYQALEFTAGYYGIRPNFRKTEEI
LRALSLWDKRDVMPQKLSGGMKRRFLIAKAMVHSPEVLILDEPTAGVDIELRTQLWNYIK
ELNKNGVTIIITTHYLAEAQELCDEIAFIDKGKIVKQDSKQNLLEDLSDRHVDVEFSENV
TLKELSLLSKIKPEMILNNKFRFHLTSYENNYSKLLTELSSLGKEIKDLEVSQPDLEDVF
HQIVGR

>gene_463|GeneMark.hmm|295_aa|+|23968|24855 >NVVL01000009.1 Rickettsiales bacterium isolate NORP64 Contig_source1382A_3623, whole genome shotgun sequence
MIEILTNRAVLHVRGGDAGKFLQALVSNDVVGKIYSYNYFLTNQGRYLFDFFVYKETDSS
YFIDLHEKDLVGLIKKLTLYKLRSDVEIQDRSGEYKILYSNDKLKTSAETDAETGVELES
NAKLETGAKFSLQDPRFDEMGFRSLIQIENINYPPEKYGAIYNSDKYHCAIADGRPDLIY
DRSIPIEYGAEELLGIDYRKGCYIGQEVISRAKYQGVVRKKIFKLSFDKELSGDFCGTDV
TDLEGKKLGIVCSGYKNQVIALLREENYLGLAEKLSIVNGETAEILIPPWRTKAI

>gene_464|GeneMark.hmm|428_aa|+|24834|26120 >NVVL01000009.1 Rickettsiales bacterium isolate NORP64 Contig_source1382A_3623, whole genome shotgun sequence
MENQSNMRLLIIGDLNSEIKIAIDIAKSKNAKVMMVGSCDEGIDFMCMGKGADLVLISVQ
FDIKSLTKAMKNEKISTPVVAYGVQCSPKEAVLAIKSGAKEFLPLPPDERLIAAIFAALS
DDSVQIIAESKPMMDAMSVADKIAASDANVLITGQSGTGKEVFSSYIHSKSKRKDKAFVP
VNCAAIPENLLESELFGHEKGAFTGALSRRIGKFEESSGGTLLLDEISEMDVRLQAKLLR
AIQEKEIDRVGGAKPIKVDLRVIATSNRDLKKEIAAGRFREDLYFRLNVINIELPSLKER
EGDIKLLSDYFIEKHVKNNGLAPKTISKEAIEKMLTYSWPGNVRELENVLHRAVLMSASS
QIKPDDIQIEEERADAGNLESGSGMPSMEKQMVFNTLGYCLGNMSKAANILGVSIDILKE
KLEHYTKE

>gene_465|GeneMark.hmm|280_aa|-|26312|27154 >NVVL01000009.1 Rickettsiales bacterium isolate NORP64 Contig_source1382A_3623, whole genome shotgun sequence
MVTHAKDKENSISVNFWGVRGSRSCCGADFMEFGGSTSCISLEHKGTHYIFDAGSGIVSL
GKKFKSMKKAHLFLSHAHYDHVNGMSFFDPLWNPNLTLDIYAGVLAPYGGTEAFFKKYLF
SHPLFPVKPSDIRAHLTYHNFTPPASLTLEDAELKTIYLNHPGKASGYALYIGGRKFCYI
LDTETSGESFIDAICDFVKDADICVYDSTFIDAEKEEYKGWGHSTWQDAVSIAARANVTR
LFLTHHAPFRTDQELRHIENEARKIFPNTHAAREGEQVLL

>gene_466|GeneMark.hmm|355_aa|-|27160|28227 >NVVL01000009.1 Rickettsiales bacterium isolate NORP64 Contig_source1382A_3623, whole genome shotgun sequence
MYTLDKYKDQLSILVVDDNEDNLFTLTRRLKKEGYTNYITVNNGKDALARTKDSKIDLVL
LDLLMPDINGDEVLKQIKSDKATNAIMVLMISGDDRTENIAECIRLGAEDFLQKPFNVDI
LKARIGGCLRKKYYAEQEKDYLEKIEFEKQQSVQLLEATFPGKIIEELTKNGKVAPLLYK
NTAVIFTDISGFTNYCSKHSPKEVFDNMQAYVNLCEKLSEKHGLEKIKTIGDAFMATGGM
FVKTANPVLASAAFALDLLKERKSLPTLWDIHIGIDYGDVIAGIIGHSKYLFDVWGDTVN

VAARIQDVAKANSVCISKNGWEKIKNKSTGKSIGKFDLKGKGEMELYEVTAIKTG

>gene_467|GeneMark.hmm|121_aa|-|28241|28606 >NVVL01000009.1 Rickettsiales bacterium isolate NORP64 Contig_source1382A_3623, whole genome shotgun sequence
MIKILYVEDNEDNVYMLRMRLEKSGYEVIIAENGLIGVEKTRSEKPDLVIMDVGLPVMDG
HEAIKILKADEATKSIPIIILTAHALSTDVKKAKDVGADGFETKPLNAASLIEKIEKLTK
K

>gene_468|GeneMark.hmm|111_aa|+|28668|29003 >NVVL01000009.1 Rickettsiales bacterium isolate NORP64 Contig_source1382A_3623, whole genome shotgun sequence
VYLFVKIEGVSIDDYVTSEAQIGNMRWDDFRINFFLSNIARHAILVRTSFHRGIMSARAI
LSSKGQIVTQEPIGRNLSDFFGKGKALIQETEPQKENVDDLIAQAVMKNDR

>gene_469|GeneMark.hmm|322_aa|-|29095|30063 >NVVL01000009.1 Rickettsiales bacterium isolate NORP64 Contig_source1382A_3623, whole genome shotgun sequence
MFEKPLISFDYAIKFLLKNKEGYEIIEGFISALFAAENYKPVKITALLESESNKERADFK
RSIADLVVEDTDGNKYIVEIERSFTTNFMHKACFNSSRLIADNLSVSQDYTNIKKIFHIS
LLYFETEEMQKPIYHGKTVIHEVDTAHPIDLRVMNQGMVTFENHNVFPEYFFISIPRFNx
xINQEIDEWLYVMKNAEVKKDFKSPYMAKxxERLSVLKMDDEERNQYYKYLKESVNKEDT
LTAALEKGIKKGMKQGREEGREEGMEKGLEKGMEKGLEKGLEKGMEKVAIAMLSRNKPLD
EISEFTGLSSQQLLTLQRSLNI

>gene_470|GeneMark.hmm|190_aa|-|2|571 >NVVL01000010.1 Rickettsiales bacterium isolate NORP64 Contig_source1382A_7217, whole genome shotgun sequence
MLGSTIYDSYTQSTHSCIDLHKNFDQGNPLYEYEEGKGPYMRYTSTPNQRNIIKDLDNID
IPDDIKELANNMYKEINPPTKRNISRSYMLVYFIICAYDKLNRVYDPKFLCKKMGINVKS
LNKALKLDTKYKMGNFCIDRSCIDYIPMYYELMGLSGKGLLVAKDIATQIHKDDKIRRIF
PQNVAKGIIY

>gene_471|GeneMark.hmm|444_aa|+|663|1997 >NVVL01000010.1 Rickettsiales bacterium isolate NORP64 Contig_source1382A_7217, whole genome shotgun sequence
MSKKYLFSTLEVRKGKLCASQENRKINKNKSLVIRLLNKDLSFILIKKIFIGGRVKIKRC
GAIHSYIIKGEYIYLPYNYAKRIVGLEYVNKKYINIELSMNVKLRPKQIEARDTAMVYLK
KTGSVTLALYTGFGKSIVSLNLAIALGVPILIICIGNILSNGWLNLLNKYTDARSWLVGS
ELTESVNIIVCPMSKMHMLSEEYLSNFGTVIIDEVHEMCTKIRIHKLLNLTPRYIIACSA
TPDKDDGSDIMYKYICGDHRVTVKDNRPLTIIKFNTGMHPDPNLKWEDLLTYLYHSNKRN
NLVLDIVNNNPKKKILILTWRVSHVNILYDLLSINGKSVDFLHSSKTEYNDSDVLVGTVS
KIGTGFDEENICNNFKGKRLNMVIFLSTSKSKNLITQAIGRVRRADNPSVVFFCDNSKII
RRHWTLLRKVFPNASYEHVKIFDQ

>gene_472|GeneMark.hmm|437_aa|-|2071|3384 >NVVL01000010.1 Rickettsiales bacterium isolate NORP64 Contig_source1382A_7217, whole genome shotgun sequence
MTPGNVSNLESDGWSSFQKRLHTIESKGETIPSKFSHQATKISWSTPVVREVASRNTRSG
STYEADMNCDFLSNSYMTLDLPQITVGDEYLESVQVCWTPNIFHNIIESGGLYMNGYKYQ
NVDKYWLDIHSQLITTSSESKRNAYMKNIGNKAALCDWSCNIEGDELFLFIPWFFTENSV
LSLPLLLIKDSVKFEFKFNLNFSSLIRMRIKGENGNWECIKYDSKYLKGINDKQRIPSPQ

MFAKYLMILESERKWRLSESSHKIYARDMVRVSSMVVREGGDRVDININCSNPITHIYWT
AENMEQNKLNNLSNYTTSGDESGKNPIHKRSMSYGPLERDEELSGGHFEYADPYMSLAQL
PKSSGYNSSSYSDTTKSINADTGISTSGKNSTMALWIKSETDQQKYRVNCYLEHMIEITY
SKKDKCSISSSQVRAYK

>gene_473|GeneMark.hmm|483_aa|+|3606|5057 >NVVL01000010.1 Rickettsiales bacterium isolate NORP64 Contig_source1382A_7217, whole genome shotgun sequence
VKYSKTTPQEFAKRVNKEKRDVTLLLSIQKILINKGSVEITIDLRDGSEYKGDFNDKTFM
MLRKNHRESLDGFKDMFKRNRVKKKKYSLNNGFKAPMYLNSDITEFLVQGNEKGLFGYAY
LDTSVGGLLSETIVVQDDGDDEGNSSPSDRNSILSSAIFTSIMAAYLTTNELSDRSTTNK
GLTRGDKGYKGNHYGADVHMNNHFRKIFKATNEKVNATYNEILSEYKSLAVGEGVDKRVK
YLRSIGGDFAVGELKRHGYTGKFEPFVTSKRKFDENDFQFSRIQTFTSVGKLSRDDVKGK
LGDVRFGIMYPPKDTLRSILDSISPEINNYIKQVKDSNKRNDDSYTMTQVNHGYVPASEK
YDAEKMMDILDAAVKRLTYIKKPSDGSNPNKWDKEEMLYQSVSMRLLTEIAQLVISRTLE
VMRIMKVLGLSMEQIKDIVSGGRVIQSVGLSGTRGTKTVSTRKVRKGGKVRQPKKPRPRK
RNK

>gene_474|GeneMark.hmm|139_aa|+|5116|5535 >NVVL01000010.1 Rickettsiales bacterium isolate NORP64 Contig_source1382A_7217, whole genome shotgun sequence
MNVSKLGIVNLVLIVLIIVFVVTSSSMFLSHKNSVQNYGIAFKIQEGVTTGTTDSMVTGG
NNMYIANSTDPLTLTLTGNNDHLEGRTVGIKNNTDNNVTLKGQINGITLDDGGLTLIVEK
YKTAMFISKDRKGTYLRTQ

>gene_475|GeneMark.hmm|316_aa|+|5611|6561 >NVVL01000010.1 Rickettsiales bacterium isolate NORP64 Contig_source1382A_7217, whole genome shotgun sequence
MSSIINNKFGKDVNVKFVTSVSNSGLLVSIKIIDNVRGEIVLSDEDEYFLGEILKNVLSK
NKHHSLLISPNTHIKYGNSYIPSEYIHSALSKITLSKVYDSYTEEGMYISLKHINNMIDV
SSIYIKLFNDVVSKIKTLYKNNILFDIQNKIAEMWGFVKLPNNLTKVNLDERIYNRVELA
KNIKSGEDVILDVEVGSDNAVRHEISRNIKFNFVFFGDILRIYIHMRKDISNVLNEISKI
NNDTHGRIMIIKETLFDNAKDKYDIAKSLVDNVEMYKLSNEYIISISLPKCLGNVDGRDI
LREINKKYAMKRISHL

>gene_476|GeneMark.hmm|491_aa|+|6563|8038 >NVVL01000010.1 Rickettsiales bacterium isolate NORP64 Contig_source1382A_7217, whole genome shotgun sequence
MKGIFIEGEIGEEYYTKSKYLYKGIVELTLNDKYKGALMSERIIQISTSYIGHIEDIESI
KEIIINMCSKLLHSDVEEVTTTKTYSKNGIMTTFYPICKGRPLYSLDYSRFVRNCNIKEF
MGYTYIGNANEMRYIMDNLQDKYRELITESYLLGYCKPYIKEICGLGLGRSDPPYGQINT
IIKNKIQLLRENRIVVMPRMTTDNNIKLYPEIAKAFGIEIVSDALTVPIAFKFERFKEIT
LQSWLQESAIQIVNNNIPIYTIDFMTLPKFSHSLKSHTSKVRSSIESTSNIPSIVVGEVT
NIYKEILPIEFIKDFSIKTDAITGRFSSIKQINRFVDALHSFVDGTEIAIGKNTKRFDYR
NVKPVNSNVVYIEERDLDVEIKSIYDLTGTVLYSVHVKIHYDDYFGNEYVEFHLFDIPMIR
SDQSFSDLFTLVTELWETGMFLSTWCKFNIIRTGEMSRYPVYIFFDSVTLDSLVDEHETA
RTECDGNTKLI

>gene_477|GeneMark.hmm|231_aa|-|8360|9055 >NVVL01000010.1 Rickettsiales bacterium isolate NORP64 Contig_source1382A_7217, whole genome shotgun sequence
MDGGIYLSIFVTFILFALILLYFHTTNISTSRTLCPVGKCKTNILSGVKQCPDSKARILV

TPSTEVCNSPSTCESNKTPFALKKDGSTNADGVCDEGDVCRCLQKPRCANHITSYFTAEK
GTPSLGILPQRIVFNQVYSYTDISGKYNIKRPLEYINTLTDFCTIPNSWVSDDRIWPNNC
VLGTLVSIPDEPDTFNKSKISITPMGCVIGNGDECPDKFPYWDKDKISCAS

>gene_478|GeneMark.hmm|182_aa|-|9080|9628 >NVVL01000010.1 Rickettsiales bacterium isolate NORP64 Contig_source1382A_7217, whole genome shotgun sequence
MADIENQINVHIRNCGTDSGTHSFTSSDSFEDITENITEDIVEGLEDIIVDDVKEMIKAL
RKKIFLCDIKAKTYRISSNFLAILIILMASTLGAYESFQTEKNIPVITISFAIATLKVLH
QYINTGNRGTFYKVMHIKFRKMFTNATYALETLDNNDELLEFAKILRNDIDNLDLSLYGA
PK

>gene_479|GeneMark.hmm|245_aa|+|9693|10430 >NVVL01000010.1 Rickettsiales bacterium isolate NORP64 Contig_source1382A_7217, whole genome shotgun sequence
MESVIPLTAFSDKLSLCFDAGVYLNLDNMTLSKISLKYTNEVYKVTSNRSSTFLKYIALI
KDNRCVFDLIKSNNPNISNIFGFLWNDEKKVNKVVMDRKSIWTKYCGRSMDGKCFCCKMG
ISYWDWNESCLIPVSCGGSTTEENIRITCFRCHKASSTYVHLAEYMLHMKYDIPKSVPSE
VINLYKSIVSICESGEHKIDHLVKDKRISLIESAEFKTLLWDLNLDTRFITYNTLCEIFN
RFRSD

>gene_480|GeneMark.hmm|131_aa|+|10993|11388 >NVVL01000010.1 Rickettsiales bacterium isolate NORP64 Contig_source1382A_7217, whole genome shotgun sequence
MNHKNKLDPFSHAKLLLDLELKAFFPGKTEELAADLLPGDSGGGMRALMTMNPKLLAATL
SLIISGYRVDEVSVGDGQMEIIYNYLYGKGSGEIANEKKGKTKTSLMRYARAIKHYRDSL
LYYEESDDSSS

>gene_481|GeneMark.hmm|636_aa|+|11473|13383 >NVVL01000010.1 Rickettsiales bacterium isolate NORP64 Contig_source1382A_7217, whole genome shotgun sequence
MQDVFAFEYSGVNISLDPSEYSNSERPEVVSSDNVIISPIGSEDDKKIVLELLPMVSVER
ACNLQDCMRIGRSLHVCYDGSVEGLSKWVDFVSNDDILDEEDCNMEWRHMFDSKSMGLRN
IGYYAELDSPLEYDEWNNIRFCKQLELALGLAHTDVARLVWLTCWTSYLCTRIDGNGWYY
YDGNKWVTMDKGVKLRKYVSNEFRSLLERKRTNLSRQLEGPGTPSKKLGERALDNISALI
CKLGITAYKTCVVKELAEFFYDETFMERLDTNMHYTGLSNGVIEICKGEAFIRIGMPGDY
ISKSTNIRMPSDITYESGPVVECMTWFKKCFCDNELVRYFLRMSASLLMGGNQDKLIMFF
LGAKGDNSKTTIKKLFEKSFGDYCATFNPNLFTKEMSNSGPSPELEKGKGTKVAFINEPS
PDSPWGSSLIKTISGGDRYFARKNRDDGGYIESSFMLIILANMIPPMSPEDAVKNRLKSL
PFDSTFVNDPPESEEERWERRIFKGEKNFSNRFNVLAPYFLWIIASMYTSYHKHGLKEPT
QVIESTKQYWSDNDIYEIYIRQCLHRVTIDDTDEIDTNIKLLVVDAYTKFTQWMTSYSSH
IRVPSRSVFINEMKKKYRLGNPIRDEWVGFTIKCEI

>gene_482|GeneMark.hmm|191_aa|-|13425|14000 >NVVL01000010.1 Rickettsiales bacterium isolate NORP64 Contig_source1382A_7217, whole genome shotgun sequence
LEFHLPFSFSAIYVLLPSNIFYMLNVYVDIINMEIARHKTSKVDEIIYLISEKIEKPSGL
GYLMGSGLNLSKGFILNAISSTSSTKRLTMVGDKYQIEPNIHISPTLVYQGDNDYSKMLS
NSGDYLVVIRNNDSKGISNKIKNMSFGVWVLSYKDALRVASEVIRLGFILTEKKMATLDE
MRSLDVDFITK

>gene_483|GeneMark.hmm|290_aa|+|13982|14854 >NVVL01000010.1 Rickettsiales bacterium isolate NORP64 Contig_source1382A_7217, whole genome shotgun sequence
MGDEIPTNPKSPRDETIITIDGKDVDRDGVDKIELIDPLFPNFEEVVDKIEFTEEVPELA
VDKEVLDLAVKKIEFVDDTHELPKSLEDMSYEEKLDTKNELLVKIRILRKTYPEYDIPMC
STKCVSEIKKIYDKWLRQAVVEKNAGDYKIYLVLFWLVIELIGTKVLGLNFSGFTLNQFN
LVNKYETLLLELGEKSYSDISAEWSPEVRLIFLSMTNAIIFTIFKYISSSLGVETANAIQ
SAFNGLFTKTKSGKGETDVAGMIANFGSMFTGVSNTKTNTKPSRVPKYSG

>gene_484|GeneMark.hmm|206_aa|-|15339|15959 >NVVL01000010.1 Rickettsiales bacterium isolate NORP64 Contig_source1382A_7217, whole genome shotgun sequence
MCELYWPHTERKKEDFMKSISHHVIRIVSIDPGVVNFCFRIEDRYNNGNVVTLAYTNIGL
DNNNDKFVYLSKFLDKYTNEYAKTNIFVIEKQLYFSVGAFSVMHHVLSYFIKETKNKSNY
PLIYLANASIKWNGITDKKPKNVKKWSIGKAIELLELREDTWSLRILDSAKKKDDLSDVV
TQAEGIVRKLGLNYHITRAKIHLVCI

>gene_485|GeneMark.hmm|135_aa|-|15992|16399 >NVVL01000010.1 Rickettsiales bacterium isolate NORP64 Contig_source1382A_7217, whole genome shotgun sequence
MYIVILLLVYILYLFYLTNNGNVKLAKLSHLLNIIINKSKIQKYSYTLSESNVSSFVLNK
KDIYVVLQRNGIMYDDNTIIGVLLHEYSHIVCSDLENNGHTDLFNKIESVLITSANDLGV
YDSTLGVDDTYPCVK

>gene_486|GeneMark.hmm|147_aa|+|3|446 >NVVL01000011.1 Rickettsiales bacterium isolate NORP64 Contig_source1382A_8423, whole genome shotgun sequence
NRSRVKENKAVLPTLSRVVINKVDAVSVPTIPSTERIESRSSHSQPQLTKETPKHRFPKW
ISPTNQMNTRTRRRENDLLKRKNVKVSFKPDVPIMRDMIRNAPTDQRKKNIVKRAPSPPT
VRVLSKRKNKLDSGLKKVKPKKPKYII

>gene_487|GeneMark.hmm|82_aa|+|454|702 >NVVL01000011.1 Rickettsiales bacterium isolate NORP64 Contig_source1382A_8423, whole genome shotgun sequence
MERVQMHPEDRASFRNEKSRAVRMCILQYKCSIACIIVFFLVIELLLSFGGELLFVLKDE
ETSNKIFKMFLFRNCSRKIEKD

>gene_488|GeneMark.hmm|434_aa|-|1514|2818 >NVVL01000011.1 Rickettsiales bacterium isolate NORP64 Contig_source1382A_8423, whole genome shotgun sequence
MAQTKPLPIGEFEWLTDEDREEEKWLEYEQEDEYGFIVEVDLDYPDYLHGHHNSFPLAVE
RVKIDESMLSEYAADCHKLLRGGREKYSAEKLTATFNPRKKYVTHIFNLQTYLKLGMELT
KVHRVLRFRQAPFLKPYIEFCTAKRAASTSKFQENLQKFFANAVFGKTIENVRSHSVCKF
VRNKKSCGRYIASPRFKHFKIISENCAIVFLSQSQVKLNKPIAVGFTILELAKEFMYSQY
YNTIKPIFNNQCEVIMHDTDSLLMRVSTPQAEDNIEKIKHILDFSKYPKTHPKYNIINKN
RLGFFKDEVAGNHITEVCALRSKVYSLQIDEGDTKVKTENRCKGIRKGYSKKIPFAAFKK
CVLSITNHRTTQYNIMSRNHNVTTNKVNKLSFSSFCDKRSLAICGIHSYPYGSILIKKNY
CYLCVRDNVIVKRL

>gene_489|GeneMark.hmm|822_aa|-|2882|5350 >NVVL01000011.1 Rickettsiales bacterium isolate NORP64 Contig_source1382A_8423, whole genome shotgun sequence
MPLCDLCGIVFSRAYHLKRHTERVHKFGQRVFQCAICNAIFTKRETLQKHREQHIPTTLF

IRYQSAFKRKLASYRKIYPRMVKTYEKAWQLDHVSLNKLISFHLETDKFFKVYISTTIEF
VKYELDDETPEDNIELHLTSNTYAVNNARSARTFLRNARRNIAERVDDMLTHGSNWRVDE
VLYTDIHFAAFNPIAGSCGMLSINNLKQIKKFPKDSKKGANQNDCFYEAIAYHFTKSKNY
KVLQAFIRKHINRNVKTPIAVKDIDRFLRGNKQLNIAINVVYEEDDEEIYPIYTNSEVKD
EDHTITLFLYNTEVDGVLVRHYTHVEDINRLLRRKYNNGRKSYGNALHCFNCLQPFFTRN
ALSNHRELCLTNKTQRIELPQEDSVMQFEHHTRKFKVPIIGFFDFESSLVAPEHSCDKCK
VGISACKHKTVVEHLQIPNTYSIVIVDWTDTVIHTNTYSGEDCMEKFFDELLTLEPRLTA
VVDEAKALHGELNMSKEDEVMFQSATACNICDKVFMHDEMRHRDHCHLTGVFLGAAHVAC
NVNRVVPSTFVLMSHNFTGFDSHFMVKSMTEDKRLNSLKALPYNTEKFRTLSFNNYTFVD
SIAFLPSSLEQLVRDLTTTECQFNILDQVQLYPNDRSDLKKLFLRKNVFPYEFNTSLAKL
RATKNIPAKEDFSSKLSNSVISDEDYAHATKVWEEFECENMDEYCKMYCLSDTALLAxVM
LRFRNEVMQDHGLDCCHYISLPQLAFDCMLKMTGVKIGLISDPDMYLFIEANIRGGSSYV
NQRYSKSGLQDGGEEQEIGLPHAREEYIEHVYVDAINLYGEL

>gene_490|GeneMark.hmm|441_aa|+|5603|6928 >NVVL01000011.1 Rickettsiales bacterium isolate NORP64 Contig_source1382A_8423, whole genome shotgun sequence
MEKGFGGLLENETDETTGSLDFYAGVKYENSVVSGTIETFKPLSGTVAPFQFQMTDRGRG
SYIDLARSRLYMELQVVTDKGKPVTTDLDYSIVNDIGNALWSTVQFELADRACSELTQEE
YNYHSLIQKLLSYSSTSVAGHLKASRFHVDTANNYDKVEQRQNEGYDQRKKFCEESNVFQ
VIGPLCAGFCQMSRYLPASLRPQITLVRASSDDFVILHKGDAKLKIVVKAAELLVPYITI
SDTMQNRIESSLMKQPMIFPFTKTIIKTKQIPTGNLSAEIENLFTGLLPTSIIVGMVRAD
SRSGRSELNPFRFQPFNVGEVSLYANGVQYPRGAFTPSFDEKMYMKEYLALFDNTGIGSE
DDTNLITTQQFEDGMTLFAFDLSPDGCNLYHNHSPPLEGNIELKIKFKSALAQPITVFVF
GNIQSALSVDKNNKTVVLTGI

>gene_491|GeneMark.hmm|166_aa|+|6933|7433 >NVVL01000011.1 Rickettsiales bacterium isolate NORP64 Contig_source1382A_8423, whole genome shotgun sequence
MAWQEKIVRKAYPVISSEGLTNIFVENYLAEHCHTFRGVFSADRIPNILALEKRFSIVVN
LSNYGEIGSHFIAIIVFEDHVIYIDVLGEECTNKHIKKYLDYLRKPIQSNIRKIQSNTSR
CCGFFAIVYVMYFERPTVIEIVFHRGEQNLYRNDDLCIQYIIALRQ

>gene_492|GeneMark.hmm|86_aa|-|7527|7787 >NVVL01000011.1 Rickettsiales bacterium isolate NORP64 Contig_source1382A_8423, whole genome shotgun sequence
VRDFSRAGPTLPRDKFFLGEKHSLQIGSVKFPKGRGYSFDAVVFTRDGGVDSEGRQKKPF
SFNLPAKLILPLHRAVTEIVTQAGLI

>gene_493|GeneMark.hmm|250_aa|-|7883|8635 >NVVL01000011.1 Rickettsiales bacterium isolate NORP64 Contig_source1382A_8423, whole genome shotgun sequence
LHICYIVENKPTAPTLTPDASDIESCREGEDEYDSDPDFWQSGQNLDNVLAASEGEEEEV
EEEEEEEEEEEEEEEHDAVENVVTPRTSGGVGRRVALKPAIKRVRDQADREGDAAVKSPT
GANLLRRSVSVFQKGKDDDHPKEREVACGAASEVGSPKSSQIIASSAPPPPKPKKRRVAG
REGAGGSTDNGERHTGAGRSKKLKFGGLSIDSPPNLGGSVGGGDVEPSCSDNALDTPTGG
LKKQKIKGEY

>gene_494|GeneMark.hmm|191_aa|+|9258|9833 >NVVL01000011.1 Rickettsiales bacterium isolate NORP64 Contig_source1382A_8423, whole genome shotgun sequence
MSGEPTLKNIKLHFYIDIISLEKKLSKVECSVVYCNNFAVLRSKFTYIVFYGVNFVNTTG

VKSFGEIEASISHFCNLIQADRVTIEKYTIDNITWSGVYRSQIDLINLKREVINSKEDIR
VIYNPFKFPGASFRYNKTLTREKSPTLILFTRGTYTIVGSKCIEDAMMIYANIKTFLRMI
DREKSFAQTAG

>gene_495|GeneMark.hmm|211_aa|+|9836|10471 >NVVL01000011.1 Rickettsiales bacterium isolate NORP64 Contig_source1382A_8423, whole genome shotgun sequence
LGPLLQADLYCTHQSMNRCQAQCDVMEFIKNVCDRSHICTRISEYAYEYYLRLKLLLAAS
FTMEETAAFAIFESCNRHDSPRSSEEICQHSGVATSRLFKLENSLNINTSNTSAKHFLER
FCGNLDLDFTDEVQIGRVLGNNLYALRRMQPNCLVAAVIYFHCIVNNVNKSLKEICEACN
VSTGNTRRIAGLIREPYYGEGFKSKMENDFV

>gene_496|GeneMark.hmm|123_aa|+|10528|10899 >NVVL01000011.1 Rickettsiales bacterium isolate NORP64 Contig_source1382A_8423, whole genome shotgun sequence
MYIARGRYIQRGRGWGGVFKSIFRTIMPLVSKIGKSIAASPTAMRVANTAKQSIANSALS
VAADAISGENVGESLERNWKRTRQDLGNSLAPPPRAKKAKKKKKKTKFTARASKYRRDRD
IFD

>gene_497|GeneMark.hmm|363_aa|+|10902|11993 >NVVL01000011.1 Rickettsiales bacterium isolate NORP64 Contig_source1382A_8423, whole genome shotgun sequence
MSRKRVESNYVKLRHPTAFSGKSKLSQYYKNTAKESEIDNALSSNYSYSLHRQYKRPRQY
NPFFIYKKRQQLQIDLLETSKLSASNDGVRYLLTAIDCFSRKAWIEPIINKEKNTVLAAL
RRILEESGPFETAYVDHGSEFISNVMKRFFIAENIKVTYTRSEIKAAIVERFNRTIQTLI
YQYLTEYETLRYVDVLQDLLYSYNDRAHRSIANLSPNQAELEENKNKVVNALNKHYTKAT
RLRNAKKKRQFKVGDMVRLKLIGAKFVRGYHLQFTLELFKIEKIEHRMPIKTYVVRSMDT
NEVIEGSYYPEELQLVTNGGIFKVEKVMKKRTRRGVREELVRWEGFGPDHDSWNKVSDVA
RRF

>gene_498|GeneMark.hmm|583_aa|+|12000|13751 >NVVL01000011.1 Rickettsiales bacterium isolate NORP64 Contig_source1382A_8423, whole genome shotgun sequence
MSSFYVILSSAYSTKEHPSNALSKFVNIFCHPLDLKENDWKVAVQSVSFDSVFYSNHGVM
KICADVALVPYLTNETIITVTVPFQNESLDIKTPTDLYNYLFPFVKQFNRESVKKLDLYI
QQNNDVNSSSYQTFSTEDLAKAPYVLMTLEKNQIKIEGVYCYIKMWTVLARWMKLNVIHG
YNGEYVVLILNRDSAYITKPASEYDKPKKPELISVKMSEVKHSLSKENNQIISTHPFMDE
HNPYGNYYFEVTRKEYHSLTHINIPQLSVELLDENNKQLHLLPGQATHIKLKFKRMNSST
FVLRLGSSDNTSIYPDNNAASFKIQLPTYIDLKHQYEVALLSITHPQKLNYASVADEHNF
WIELEYLYEGETIIRKFDASPCLKDVDNKKDNKSFFRSLYKMLNDRITIEHRQKVRFTHV
EEDSNTVLASSLDTTTRVSILLAHVLGKHNISHTLYDEIKTSKNRNNMGEGANIILGDSI
VYSTGFDLFRLLPHCMLLYASCINPVHYGGHLAQILKFIPLHLVNKTLELQHSYESTHLD
YQTVNQNTLNQLQFELRTIDGKLVGFEGSQNTFINLIFRRIIE

>gene_499|GeneMark.hmm|107_aa|+|3|326 >NVVL01000012.1 Rickettsiales bacterium isolate NORP64 Contig_source1382A_8430, whole genome shotgun sequence
GVGNIYAAESLFMSKINPLTKANTLGAGAVSALVKSIKRVLQKAIYAGGTTLKDFLGGDS
KPGYFKQELQVYGRKGLPCYICKTPIESLKQAGRATFFCPSCQQNKD

>gene_500|GeneMark.hmm|428_aa|+|633|1919 >NVVL01000012.1 Rickettsiales bacterium isolate NORP64 Contig_source1382A_8430, whole genome shotgun sequence
LKKALLIEEKARVNIIMSARSFKVVVLLMLFLIIVLFLIINTKTICNEKYWEIARTKKQI
ANIEICTKEGNVDAAILASQMYGKGDTIKRDHDKAIDIIQKFADSGNSAALIQIADLYSC
YNKYRNFNKAEKWYKEAINNGDNYSEKAKIALAKFYLRKYFNTKDSKYIGLYIDHLKEIA
NNGNIDAILLLAAEYKEGSYIGKDYYTSLNWLEKAAERNSIRAYRSIGNYYANGFFGEAN
YKEAKHWFQKAAKSGDNVAASFLKSNPTPFGLEITKATITDFKRKFRKHNKIDAPNFING
GYTYLVSPKYIPLDDVKKEVVFVFDKNSILDSVLIVFDKRKYGEIQTHLKSKYAPIVKGA
QNIFMHSLTEVRLLNGYVFYSQLNTDGYNSKVCTLIYESRAFSIKLYERDRFLRKRQQDI
KKQKMNLL

>gene_501|GeneMark.hmm|90_aa|-|2220|2492 >NVVL01000012.1 Rickettsiales bacterium isolate NORP64 Contig_source1382A_8430, whole genome shotgun sequence
MDSFNVSALNSLQDILFALTQHNEELRNAAWQHLISWHNQYAVKLWFSFGDDTYERTVKM
LDKLRKTNIEPPYHAWNDLPNIMKTIQKKK

>gene_502|GeneMark.hmm|126_aa|+|2555|2935 >NVVL01000012.1 Rickettsiales bacterium isolate NORP64 Contig_source1382A_8430, whole genome shotgun sequence
MGFAVCPLYIYQRFPYLIFIMLSIISLSCGGQNYNKYYISTILAAIVLAAYHTSIEWGIF
EPSSLCKPLVYIDADFSVSDFKKMLYSQKIGTCSKPALVIFGLSMTEWNLLLNLVLLPIF
IKYRRL

>gene_503|GeneMark.hmm|83_aa|+|3163|3414 >NVVL01000012.1 Rickettsiales bacterium isolate NORP64 Contig_source1382A_8430, whole genome shotgun sequence
MHSITYTALRKSLSSVLDTLENNHVAYQVTRKNHKNMIILTEEDYNSKQETLYLLSNPAN
AARIRESIKQANNSEFVEVDLDD

>gene_504|GeneMark.hmm|88_aa|+|3407|3673 >NVVL01000012.1 Rickettsiales bacterium isolate NORP64 Contig_source1382A_8430, whole genome shotgun sequence
MTRKIKWTVNAAEDLAYWKKYDLNKYNRIKLLVKNIQETPITGIGKPEALKHALAGLWSR
RINQEHRLIYCITAKEIIIYNCRFHYKK

>gene_505|GeneMark.hmm|74_aa|+|3859|4083 >NVVL01000012.1 Rickettsiales bacterium isolate NORP64 Contig_source1382A_8430, whole genome shotgun sequence
MSLDKRGDYWMDKAVQIWLKNYALTAEIARLEQQKGLTPDPTIDSWVEQVVDIQLENTVL
AAEIARLEDCMPSN

>gene_506|GeneMark.hmm|84_aa|-|4236|4490 >NVVL01000012.1 Rickettsiales bacterium isolate NORP64 Contig_source1382A_8430, whole genome shotgun sequence
MKKFTIYTTNVCPYCVRAKALFDRKGFAYDELNAEDAKIREEMLAKSGGMRTVPQIFLGH
IHIGGCDELYALEKDGKLDGMVEG

>gene_507|GeneMark.hmm|661_aa|+|4728|6713 >NVVL01000012.1 Rickettsiales bacterium isolate NORP64 Contig_source1382A_8430, whole genome shotgun sequence
MSSFKIKSKYKPNGDQPKAIASLIKGLEAKEKSQMLLGITGSGKTFTMANVIEQTGRPAL
IMVHNKTLAAQIYAEMKDLFPDNAVEYFVSYYDYYQPEAYIARTDTFIEKDSSINEQIDM
LRHSATRSLMERRDVIVVSSVSCIYGLGSPELYSQMTLKLEAGKEYKRKDFLNQLIDLQY

DRNDIAFERGSFRVVGGNIDIFPSHYSTRAWNLSFFGDELEYIREFDPLTGEKFRKFDAA
VLYASSHFVTPEAVIKKSIVEIEDELQERLAVMKDMGNSLEAHRLNQRTQYDMEMLQATG
SCKGIENYSRFFTGRKAGMPPPTLFEYLPPDALLFVDESHVMVPQIRAMYNGDRARKSSL
VEHGFRLPSALDNRPLKFEEWLDYRPQTIFVSATPAAFELEETQGEVVELIIRPTGLLDP
ECIVRPASTQVEDLIGEIKSTIKRGFRILVTTLTKKMAEDLSAYLQELGHKVSYLHSEIH
TLERVEIIRSLRLGEIDILIGVNLLREGLDIPECALVAILDADKEGFLRSETSLVQTIGR
AARNSEGRVLLYADKMTNSLNKALAETDRRRKIQREYNKANGIIPQTISSKIHSVMELKK
VEDIAGGTEEAKIILSDPAKLQKHIAKLRREMCQAAKDLEFEKAAKLRDQMEVLKQAALE
M

>gene_508|GeneMark.hmm|426_aa|-|6845|8125 >NVVL01000012.1 Rickettsiales bacterium isolate NORP64 Contig_source1382A_8430, whole genome shotgun sequence
MSVPSVNASSMDVSSAQASPAQASPAQVSSAQVSSAQVLPSNMPPISIYIHWPFCLSLCP
YCDFNSHISGSIDHEQWLTAYKAEIDYFAHKISGRKVRSIFFGGGTPSLMKASVVEAIIQ
KIGDLAKITDDTEITLEANPTSYETQKFEEFKSAGVNRVSIGVQSLRQEGLQLLGRKHSA
KEAMHAIESASKLFARYSFDLIYAIPDQTLEEWQDDLKAAMSLAGGHISLYQLTIEKGTP
FYKLFHDGKLHLPSNDISSNMYEWTNEYLLENGYRRYEISNYAKQTHESIHNLCYWNYNE
YIGIGAGAHSRLHNDNMEIQAIMMTHAPAKWLSSVMKNGHGIQTSCNLSRSEVIDEMLLM
GTRLETGILEDNFQRVTGMNFESVLNLKTVKHYIEQDLVSLDNGRLKLTDRGLMLHNYLV
PRMIKT

>gene_509|GeneMark.hmm|260_aa|-|8122|8904 >NVVL01000012.1 Rickettsiales bacterium isolate NORP64 Contig_source1382A_8430, whole genome shotgun sequence
MKIITWNINSVRLRIKLLEKLCIEQNPDIICLQETKTEDKTFPLEAIKALGYEHVVYSGE
KSYNGVAIISKQPISKSFSLSLYNDHKRHICAQIGDIEVHNFYVPAGGDEPDIELNPKYK
HKLEYIDLMHKWLASNKSKSDKIILVGDLNIAPHEHDVWSSRQLRNVISHTEPERTALLA
LQDSLEFIDTGRYFVNMSEKLYSWWSYRNRDWTKSNRGRRLDHIWVTQPLKDNLKSINSF
QEARSWEKPSDHAPFILDML

>gene_510|GeneMark.hmm|94_aa|-|8947|9231 >NVVL01000012.1 Rickettsiales bacterium isolate NORP64 Contig_source1382A_8430, whole genome shotgun sequence
MRQIEYSSAFKKDYRRESKGKHQKTIDAKFKYVISSLIEDKILAEKYRDHDLLGDWLGYR
ECHISPDLLLIYRKTGKDFIRLARIGSHSKLFKK

>gene_511|GeneMark.hmm|88_aa|-|9218|9484 >NVVL01000012.1 Rickettsiales bacterium isolate NORP64 Contig_source1382A_8430, whole genome shotgun sequence
MSVANTYVRARIDSVTKERATEALHAMGLSVSDAIRLLMLRIAQEHRLPFEIKVPNQATK
EAIGELESNKGTKVSNIQTLMASLHETD

>gene_512|GeneMark.hmm|464_aa|-|9591|10985 >NVVL01000012.1 Rickettsiales bacterium isolate NORP64 Contig_source1382A_8430, whole genome shotgun sequence
MMRFFKHMRAGLKLLCTLCKNQILTYPPDKLKLPRHLKVFGTICGIIFYPKGLFIRPDKT
YGERFSNCLYALGSIYIKFGQTLSTRPDLIGAEMAESLRYLQDRLPPFDSAIVIERIETQ
FGKKISELFDEFEEKPVAAASIAQVHKARLKSGELVAVKILRPDIAKKYNDDIDFLEYCA
ALITKMVKSSKRLKLTEVIGIFRRSMEFELDLRSEAAAASRIADNFTGDDSLCVPKIHWN
LTTNDIITLEWIDGVSIYDKEAIIALGHDPKEIAAKIAVIFFNQAYRDGFFHADLHPGNI
FVCKDGRVALVDFGIVGILPENDRLAIAEILFAFLNRDYALIAKVHHRIGYIPSDTNLEY

FAQSCRAIAEPIIGLAIKEVSIARLLSHLFKVTEDFGMETQPQLLLLQKTMVVVEGIGQS
LDEDINMWQLAEPWIKKWAAKNITPEAKILRAVKKIINDLLDRA

>gene_513|GeneMark.hmm|269_aa|-|10995|11804 >NVVL01000012.1 Rickettsiales bacterium isolate NORP64 Contig_source1382A_8430, whole genome shotgun sequence
VDEKDKKDEKTFDEKSLDGKNLDKKSFGFEKVTSKQKRSLVDNIFSDVSAKYDLMNDLMS
LGVHRLWKNEFCKMIPNLDSKIIDVAGGTGDISLRIKENAKNQNKDPHIVVCDINPDMLE
VCRARAIDNNILTNFDILTCDAEKLPFPDNSFDYYTIAFGIRNMISIEKTLAEAYRVLKP
TGKFLCLEFSKVQNDVVRPLYDFYSFKLIPSIGQSIANNKDAYKYLAESIRMFPDQEDFK
TMIQKSGFSDVNYKNLSFGIAAIHYGFKN

>gene_514|GeneMark.hmm|265_aa|+|11905|12702 >NVVL01000012.1 Rickettsiales bacterium isolate NORP64 Contig_source1382A_8430, whole genome shotgun sequence
MSFKIVTWNVNSIKVRLPHLLEFIKEHDPDIICLQELKCQTENFPSEELSHLPYNLYVEG
QKTYNGVAILSKIQADEVKTSFPNNPASEQARFIEIALNTPMGYGRVICLYAPNGGEVDS
DKFSMKLKFYDAFTDYIEQIKSFEEKLYICADYNIAPFDIDVYSPQKLQDSTCCTYVEKQ
KLRTILNSDFVDSFRAKYPDKQEFSWWDYRGGSFEQNKGMRIDTIISSSNAISDLVDCTI
DYKTRGKHKPSDHAPVIAEYLSEAK

>gene_515|GeneMark.hmm|224_aa|+|12834|13508 >NVVL01000012.1 Rickettsiales bacterium isolate NORP64 Contig_source1382A_8430, whole genome shotgun sequence
MNNKLLQFLCIIIASFAQLFLCANTIADEGSTQKEPELGKVEEALGVKRGECKNPPCSTD
IDSLIDRLMVEDVKKQESLEMLRRLKEKNKKNKFNSLVEMNTAKIMVLNKITSKSRQETL
KLREVAFFGNLSIELHRCVKNTDPLNGNSLMLLTILDNHLEDDNLSVFHGWVLSSNPSIS
TLEHAIYEIIPLDCFMVEEKRPVDPEDEEPLDLDRGKPLDNNNN

>gene_516|GeneMark.hmm|207_aa|+|13624|14247 >NVVL01000012.1 Rickettsiales bacterium isolate NORP64 Contig_source1382A_8430, whole genome shotgun sequence
MHRLEEKRHFIDSIEENIKNQAVPIQSFPESAAPLYSKVTLSGTFLTDKSVYLYGRRSAS
PEKDGYYLLTPFAADNGEVYFVSRGWAPQSVKDKMEAESYTRKTIEAMVMSGEKKNFFVP
ENDQKNNIWFTLDLDMAKKLLRVSVADFYLMQINSQNLPKGAKPLATTYLNKVHNNHLEY
AITWYSLAACLFVMFMIYGRKPEESST

>gene_517|GeneMark.hmm|510_aa|+|2|1534 >NVVL01000013.1 Rickettsiales bacterium isolate NORP64 Contig_source1382A_8708, whole genome shotgun sequence
LLEKCVNIVESQYGKNSSERARILHLFGLIYYNLFDYHKAITVFKKVVHIKKTIFGTGSL
KFNESLLHLAMISSDIGQHFKATELLEPAITVIQNHYGENHVKTADALMHLAKALYAVGN
YARSEKLLRKALSLYTDSFSSEHLITAEVYHIMSSIYRQKGYYKESIDMLNKALKIRRRE
YGDDNVLVAELKVKLAHAYRHIGEYNTALPLLKSAISVYKNKYGINNMNTAWAMTYLGHL
FWDSGKINDSIQLLTQASEIMTKNYGEENLYTAWSFLRLGYILTATGSYDKAREFIQKGY
KIYGKYYKYPHIEKAWSDAYIGSFYNKTNKLTLALSHLKKALEIYQKQFGKNHKRTNWIK
IMLADNYSRRGDFAKALKLSEEAYESYRLHYGEQHSKTALSLQAVGRIYFAHGLSKGEKL
VRESLKILESQNSSKLYSTFSIMADIAFKKAQTHKRNGKMIKAQHYYRKSQEYLDQSLKI
AKRNLLSDSEHIKFLEQKSAKLASMNFLDN

>gene_518|GeneMark.hmm|324_aa|+|1554|2528	>NVVL01000013.1 Rickettsiales bacterium isolate NORP64 Contig_source1382A_8708, whole genome shotgun sequence
MWSKIVKNLIRAAICTLFGISANATTLNPNIITHNVGLSSVDMEHEIERVQHQDNLLFSV
EKTLSLLAQLNEFDLGCFLLRNKGLNGYWTSYLILQDKGQFDSPLEDWLIKKAPTVLATR
ERFYIFQDKAKSIIESKSSNEQINIASIPCGVLSDLLTMKFQTKAKVKFTGIDLDETSIK
MAKINAENFPKMHQTQILKRDAWNLGLKDEFDIIMSNGLNIYEKDEDRLVALYKEFYKSL
KTGGKLITSFLTYPPAFSKDSPWKNVILEDAQKQIALFRDILGAKWMVYRTEKEVRQQLS
EAGFVVEEVIYDSKHIFPTIIAKK

>gene_519|GeneMark.hmm|124_aa|-|2730|3104	>NVVL01000013.1 Rickettsiales bacterium isolate NORP64 Contig_source1382A_8708, whole genome shotgun sequence
LENIKMKKLHKALENARHFGKKQFIGLKHKVGVSNDFQHGMETVAEQVKHVNKLLSITED
NLSYLNRLVDYINDCIDRIVETLSNIIDILNEFFDDVLDNNCINNLDDFLEEAADILGEY
EISD

>gene_520|GeneMark.hmm|75_aa|+|3620|3847 >NVVL01000013.1 Rickettsiales bacterium isolate NORP64 Contig_source1382A_8708, whole genome shotgun sequence
MGVDGSFAECKAYSPAFCFGLRVGAHLERGEDVVELAKRDANPLVLDGECDEIRSSLLGV
LQETPICKFCADVGF

>gene_521|GeneMark.hmm|549_aa|-|3844|5493	>NVVL01000013.1 Rickettsiales bacterium isolate NORP64 Contig_source1382A_8708, whole genome shotgun sequence
MSTDSESRYSQSLMAFNFSPVSRDDAPQRARYNEYKEAISQEKLATTLLCQGKTIKSLTP
IELAALISFAVSNNYIDEIARLVSEVGIDVNTLYRDVDYDNLMKSTDFVLSPRVSRSVSC
LLYNAYRLENSEDAGRHFGMGYVSKVIMGFNIDVSALSEYDKMLILEESMRYEDLADISD
ILRVELPELSVDNKKLLLILAITNIAPIRGILTLLGIEDINELSPNLISAFLHCASMHSI
LKYVIDTWEVKLQVFIDDVEALYLPDTHISFGFAPEMILSHLSYAEFLQIELKLQDYYLL
HSSEHSVGRPEWLPNIIEQRHLEQCLVKSNKVTPEINVYCSWRVSFITQCIETKNLQAFC
QILTPRIDASSWNEEGDCTDHSLCVLLVEIAQCAMREEQAPQLLMLGGAVFDNMMRACKN
ACYYEDPYNERWDITLEMVINQVIVGEGGLSQLLLFLRENDVFAELNLLDSQDNLESISS
VEMRKFVELWEYSMQHLFFNQLLDQYLSCETNDKSELKNALLATSFGKIFQDDDDLSGSS
SGLCQSDHP

>gene_522|GeneMark.hmm|39_aa|+|6149|6268 >NVVL01000013.1 Rickettsiales bacterium isolate NORP64 Contig_source1382A_8708, whole genome shotgun sequence
MAAGSRWTIEECFEMAKGEVGLDEYEVRSFRGIAVSHRQ

>gene_523|GeneMark.hmm|108_aa|-|6341|6667	>NVVL01000013.1 Rickettsiales bacterium isolate NORP64 Contig_source1382A_8708, whole genome shotgun sequence
MTTEQTLAEEISEGLAEAIKFAKGEHVEGCVTYMFNDQEICPRDIRQSLHLTRERFHEWF
VCKVSNQRNWETGLRKPSEVTLAFYSMIKENPSQVYKMLHHSTIPKNG

>gene_524|GeneMark.hmm|72_aa|-|6673|6891 >NVVL01000013.1 Rickettsiales bacterium isolate NORP64 Contig_source1382A_8708, whole genome shotgun sequence
VELAESGKSDGKLNIEEKAVVLGLSTNYYDKKNVLFLLATYSKNKKEDLSAKEQKQISNL
TKELIMSFNQNK

>gene_525|GeneMark.hmm|355_aa|-|7489|8556 >NVVL01000013.1 Rickettsiales bacterium isolate NORP64 Contig_source1382A_8708, whole genome shotgun sequence
MVSTIDENNTVHVQIGRLKGRDLAIALKSKEVVVALGDSLTTHLDRLEGIYLAITLKSKE
VVAALGDSLTTHLDRLEGRDLVNALKNKALVAALGDSLTTHLDRLEGDDLVNALESDAIV
VALGDSLTTHLDRLEGYDLVNALESDAIVAALGDSFTIQIGRLEGGYLIYALKSKKVVVA
LGDSLTIQIGRLEGRDLVNALESKEVVAALGDSLTMHLGRLEGDDLVNALKALMAALGDS
FAEYAATLSQHKWYECAKDLNLPKEVMLAVHDFGEVNNNWNPILKTDVLRNFSSIAPATA
IAIATATDKDLKKLCPVSDLTQIIMSFVHLNCPQNDQVDILALEESDSGTGGSNS

>gene_526|GeneMark.hmm|164_aa|+|8704|9198 >NVVL01000013.1 Rickettsiales bacterium isolate NORP64 Contig_source1382A_8708, whole genome shotgun sequence
MAIEIQSIKDLLTIVDNLTKTLEQLRKGDTLVVWKLDRLGRSLRHLIELINELEAKEIKF
RSIQDGIDTSNSIGQFFFHITGAFAELERNLIKERTKAGLAAARARGRKGGRKPSLNKQQ
TQMMLELYQARSASIMEICDQFKISRKTFYRYVEKAKIMADANP

>gene_527|GeneMark.hmm|1212_aa|+|9754|13392 >NVVL01000013.1 Rickettsiales bacterium isolate NORP64 Contig_source1382A_8708, whole genome shotgun sequence
MSKSSNVGAEQNMPAVVPNKAFWYQGHHIVKLWEKYLTSKNDSISFLSARDGEVAGEDNV
SSLFNGMSLNYNFAIVLQGNNLYRNSSDQTITLCGEVADRDVASITRRMEAIIEQIRSYN
QSHPEAAITNHIIIFPYHRNPGHWNLGTVELTFTSNDDNDEQINARISIYEPFGGVSTGY
DHLASSLSDLNIVFEQNKLSATKQQYNGTSCGAITAENAKLFLAQSLDDQSGDLLERTYK
AGAEELRRQHIAEIDDKAFNQTQRDNQAYAVGGDRSIEEAHTATQSLADLIAHADRDWVK
QAIRHLLNSEDDNIRRDNIDLFKQFLLQQQYNEDDQYSKIAGMLLEADGRFKEGAMDVIA
AVVFREDNSQFPTFSATNSSARHETTVTLELSSDYDNLDTEAKVILDSVKKVSLDDLIIQ
GTYSFVPSKPTARALFKNTDTEETIIFGASDNLLFYIASLENGFEVIPRIAFGIRKVLDS
HGNNILHHAVFRQDLNLVTAILHRAMLDGIISDLVNHRNHDGTNPLGMAFLSTDSNNLGG
IKIATIFAGIFAYDITGYLNNKVHMDYTTSTTIGGTTLLHNVMLAASKCKGQEFRKALSE
FFKVLSKRMHDDKDCFNPNYPTMNPGSLGELENGKKLDSKKLDGHMISSSKLLELKFKSQ
GIKLLCKKYFDFILASYEVDQFDDDSGDSADEDSPAYTYKQMLLDFNRFIGNSIKGHTRE
KSHANFQRTFDAYDSDFAFFSDAMEGLSITEDDPWNNFWLKLNSISADSTESAEYKEVID
SMYVPLFHGVPFMHSQYSNFQRREVAKKIFAINRKLLGRFDDSMTEVEYTLTPEEEILIG
IHSRTATASAGMQSLYEIMVASPEDLQRLNTIDENLWGYFNRHDLPKKFKIAMKDYVFNF
ASSPIRGFWENNNNDGAPHSDSELPPSDDEIVKYRFPVIATSKAPDHAIRFAIGRNVEAA
RGEIRMQPEYIDGFPTHRLAGLLYVTMHKLSDLLEARGNHTMIDVNRRVKSGDIDIPTEN
IEKDPDFLRFKNQLECDFLGKISSDTVVAVIPVIYPNIHDGDGFESGYHEAVFGLNSSAT
RIYVTSPVKIRGDLSNQPNPKIMSGSSNIVGFGKVIIPTMVALANGILTAIAEKYGYFLC
AIRDDNELVPYKVKFDERKKKGAGQLSEAQLDIKNSGDIPQAIRLWQSIADQTEEDSDVA
EIGSMLSNAHID

>gene_528|GeneMark.hmm|106_aa|-|13615|13935 >NVVL01000013.1 Rickettsiales bacterium isolate NORP64 Contig_source1382A_8708, whole genome shotgun sequence
VINAINFSEKGIIAISVDREKYFASFAIRDEGKGIPKTEIYDIFTPFKMGSNTESKAAGR
GVGLALCKAVIEAHDGEIKAESRPVGALFRVCLPYAGALQNGTKSF

>gene_529|GeneMark.hmm|158_aa|+|494|970 >NVVL01000014.1 Rickettsiales bacterium isolate NORP64 Contig_source1382A_8750, whole genome shotgun sequence

VTPSSLIAAKASPRCLKNNYKIHMFNISPLQFYTVLGLLLFSFLSTPAYANGGVQTTLET
DEAPGSLGDIPESDFFEFQDEPLEGPLTLPDWFKLSFLELSNDIIDLKENNKNGLIVYFG
QKHCAYCxAHLEHNWQAPFIVLFLSKIDKKRQYHIILL

>gene_530|GeneMark.hmm|380_aa|+|985|2127 >NVVL01000014.1 Rickettsiales bacterium isolate NORP64 Contig_source1382A_8750, whole genome shotgun sequence
MNKQSGKYLTQKIGGEEYKAYIPFKLPPGGGIDLQKLHPYLEKATCAIAELNGAAKFLPN
HSLFIYMYVRREALLSSQIEGTQSSFSDLMLFENDQKPNISLDDVEEVSNYVKAINYGLE
RIKNGFPLSLRLLREIHEILLSGIRGSLKSPGEFRRSQNWIGGTRPGNAFFVPAPPEQMM
DCLYDLEKFLHDDSVPTLIKAGLAHVQFETIHPFLDGNGRLGRLLIILLLHAEGMIESPI
LYLSMYLKQNRETYYSLLQEVRTRGAWEAWLEFFLQGVIAASKDGIQTAQEINALFKADF
AKIESLGQIKFSCKRVLEHLKHLPQISVRHLSQTLAITPPTARNCLNKMVSLGILEETTG
KKRDKIYVYCKYLTLLEGEV

>gene_531|GeneMark.hmm|271_aa|+|2400|3215 >NVVL01000014.1 Rickettsiales bacterium isolate NORP64 Contig_source1382A_8750, whole genome shotgun sequence
MFLIIGKVIVMGDIVDRKALKYSTASGEKNISSPKEPVEGYDIWESGKVWGSIDVWKDSD
QDIQRSLADESNSDVDVLLLILDLRHNIISGNIEYVEYFLGCIAPHSISPYANLVMYSAK
SEEMQKYLLEYCEMHKLDGRKKLFAYFLCKSLLKNDIESAKSLLKHCYGDNEIDYVPVLD
IIWNILRYTPANGASSLDYSLAVDAILYILETHADFPLDYTQTIIDVKRYDGVLGYQKGL
LEGQVLENSESWNNESEMVGQGSTESMESLD

>gene_532|GeneMark.hmm|107_aa|+|3592|3915 >NVVL01000014.1 Rickettsiales bacterium isolate NORP64 Contig_source1382A_8750, whole genome shotgun sequence
MAKEDKADGFEPWIMGGFLNILDRDISTKGYPDSGMSNEDALLFLLKGDNLSPETVQFFN
GLVDQSPESNDERAHFILFGENSTEVRDVELEPEPECNFDWSFTFDF

>gene_533|GeneMark.hmm|792_aa|-|4068|6446 >NVVL01000014.1 Rickettsiales bacterium isolate NORP64 Contig_source1382A_8750, whole genome shotgun sequence
MIFTLSWLKQFLDTDATLEQITNTLTMTGLELEDLVDKSKELEAFKVAKIIETEPHPDAD
KLKLCKVETDSKTLQIVCGAPNARAGIKVVLASIGTVIPSGNFKIKKSQIRGIESSGMMC
SADELNIPGDSSGIIELPDDAVIGDAAAKYLGLDDPIIHINITPNRADALGVYGIARDLA
ASGLGTLKKLGNPEFNQEFETKTTLNVRGSEACPLFAIREIKDLENKESPTWLKNLLDNI
GVGSISPVVDVTNYISYSFGQPMHAYDADKISDNIIVETLSSDEKFKALNEKEYELKTGD
IVIRDEKNIHCLGGIIGGENSACDKNTKRIILEAASFDAEHIAKTGRRLIIDTDSRYRFE
RNVDREFTHMALDFATDLILSICNGVASKMVSSGDPALPVRTIDFPAGFLESRTNISLAP
QEIEDILCKLGFICTNIGESIKITIPSWRYDVSIKEDIVEEIVRIYGYDKIPETPLPEAP
IARIIPRDQRRVSDIKRLLASSGYDEIVTWSFMDSEKAKSFTTIKEELTLLNPISSDLDY
MRPSILPNLLRVANNNINRSFHDLSLFEVGPIFEDTGHKVITHASGIRTGNIAPKNSHEA
CRVFDLFDVKADIAAILESAGLDINKCQIRQNAPDYYHPTRSASICLGKNVLGYFGQVHP
LILKEFEIDADVIAFEMNISALPFGKEKYGKKPELVRSDYQAITRDYAFVVDKDQPVGEM
LSFIKNADKKRVKSVNLFDIYSGTHIDADKKSVALSVDIQDDAKTLTESDIETINKAIID
GIEKKFSATLRG

>gene_534|GeneMark.hmm|346_aa|-|6443|7483 >NVVL01000014.1 Rickettsiales bacterium isolate NORP64 Contig_source1382A_8750, whole genome shotgun sequence
MLEEILQSADEKINSAKNSKELQDIKVEFLGKSSLINQEMKKLGKAAPEERKELGQKINS

VKQKIQELIDAQSILLQKKELTERFASEKLDLTTPARPRATGSIHPITQAMEELIQVFSK
YGFDIKDGPSIEDDWYNFTALNIDENHPARQMHDTFYLKKTSEDSKLLRTHTSPIQIRTM
QNEKPPYRFIAPGRTYRSDSDMTHTPMFHQIEGLVIDKNIHMGHLKHVIIDFIRSFFEQS
NIEVQFRPSFFPFTEPSAEVDIRMNKDDKWLEVLGCGMVHPNVLKNVGIDPEEYQGFAFG
LGVERFAMLKYGIKDLRQFFEGDMRWLNHYNFKTLDIPTLAGGLTR

>gene_535|GeneMark.hmm|87_aa|+|7742|8005 >NVVL01000014.1 Rickettsiales bacterium isolate NORP64 Contig_source1382A_8750, whole genome shotgun sequence
MVFSKVVFENLNVTQLKRLIIMATGTVKWFNNTKGYGFIAPDDGTKDVFVHISALEKAGM
SGLEDGQKIKFEVVNEKGRDSAGNLEV

>gene_536|GeneMark.hmm|38_aa|+|8601|8717 >NVVL01000014.1 Rickettsiales bacterium isolate NORP64 Contig_source1382A_8750, whole genome shotgun sequence
MVFLLRAIRDQKSYPPTVAAALEFLRLPALSIPDTDYG

>gene_537|GeneMark.hmm|413_aa|+|8790|10031 >NVVL01000014.1 Rickettsiales bacterium isolate NORP64 Contig_source1382A_8750, whole genome shotgun sequence
MEKFSEAGIPEPLLHRLTEMGFTTPTPIQAEAIPHALEGRDILGSAQTGTGKTGAFGIPL
VTHLMNNAKGSALVLLPTRELAVQVMKALEQFTGRGKISTALLIGGEDMQRQYRQLNAKP
RLVVGTPGRINDHLTRKSLKLNDTDFLALDEVDRMLDMGFGIQLDAIAKHLTAKRQTLMF
SATLPKNIQKLSAKYLNDPVRISVGATHAPAANVKQEHVKLTDSEKYPRLLDEISEREGS
IIVFVKTKYSADGMAKKLRKNGHKVDALHGDLRQNKRSSVILAFRQQKYRILIATDVAAR
GLDIPHIQHVVNYDLPQNPEDYIHRIGRTARAGAEGNALSFLSPADGIKWRAIQRLLNPD
LKDNSGGEGRKKSGRKPFKGKPGAGRSGTGKPGQRRPGQNKPGQNWKGRKKAA

>gene_538|GeneMark.hmm|402_aa|+|10600|11808 >NVVL01000014.1 Rickettsiales bacterium isolate NORP64 Contig_source1382A_8750, whole genome shotgun sequence
MIDRKVRYLIEKALSRQPAVAIIGARQVGKTTLALDIAKGRTSLYLDLESYIDREKLQDP
IFFFDQYPDDLIILDEIHRMPELFQTLRGVIDSDRRKGKKTGRFLILGSAGLDLLKQSGE
SLAGRIGYVDMTPLTALEIDPNDVVKLWVRGGFPESFLGSSSEDSFLIRKDFIRTYLERE
IPQFAPRFPVETLERLWMMLAHCSGGMLNSSKLASNLSISTKTVSFYIDLLVDLLLFRRL
KPFHANIGKRLVKAPKIYIRDSGILHATLGITEYNALAGNPIVGASWEGFVIENILSVVQ
AGTRASFYRTSAGAEIDLVLEMPNNDIWAIEIKSGLTAKPSKGFYNAIEDIKATHRFVVY
SGKTQYPISENIQAISLIGIMKLLEATSAGLKDPASSAFGGG

>gene_539|GeneMark.hmm|613_aa|-|11961|13802 >NVVL01000014.1 Rickettsiales bacterium isolate NORP64 Contig_source1382A_8750, whole genome shotgun sequence
MKNKDQPFREQISFEISDLETYVEGVAHDAEITPLANWMTLKSQIYTEGLKEFHVSLNKT
PPMTIEQIREQHSKALLHDLEMITKRQALVEYNIPPFQEAFYKEINKPNDRSEEYNNFVI
ACSEIQIDAHFIERVSAQALLKLNENNVTVKILQEHIDRELSNYLEEHKRETARDLSRTA
ILLKTENGMVRGSFVEEIMTSKEINLTGEQREFIISSWQQGLFDLGWAASCVSVLHKAHP
DMENIWGMDTPRDRTSIFIDTSDGEVKILNRSKIILRSQDDPEGELKPYLEMLASVNITG
LTSNEFIPGCATAPPEIRFDINRINENLEFTIPENLTLSKTSNPLDAYTRLRKDTIEEYF
DSIKNDRPISSFAWEGLKNIMGDAESIKYAATHIHPKSEKLKATLLKKINILADEASNHP
VNFSDLSTASIAFCSTESDKEKQEEFTSKLLASCSTHQTITTLGDREIDSMEEAVVSIMT
PLLDYSNEDSLRKNAKKIIRESSSKLGIKFSWSQRWEQLKDNTSNLFTTLTGGTNSAHEF
MHNPENARIIQNINASLSKRSGKKFTPHAPVTTPPRKRFEPHPPETKPPVEVSPPNPTPS

VVHKKKTNSGRKH

>gene_540|GeneMark.hmm|307_aa|-|3|923 >NVVL01000015.1 Rickettsiales bacterium isolate NORP64 Contig_source1382A_8784, whole genome shotgun sequence
MKHIHIQDCSVPSLFQEAPPADKQLTDAELAGAVVAARGRGVLFSMACQQNETPEHDFLK
EVNVLIRAEYKAAVKWCMSHGTCGVSGETLDWVAELATIFITGNNTRHVAKSFHPYREIY
NPGEEAQIFALFSEMENSPPGELISKFLKIFDVANAAQIVEAVIDSIAADEDLASIIRAG
LSNAGDDEITHGLVEEIIIKILIYSQSDSTIRGANAYLLQQVIEKHPTCADGIREVYMAQ
EAMHSAAANMVPYLQEPLLDNKQLADAVVAARGKGVFFTRAYQDIPPIELGSWTEVNALI
RAEYEAS

>gene_541|GeneMark.hmm|91_aa|-|1113|1388 >NVVL01000015.1 Rickettsiales bacterium isolate NORP64 Contig_source1382A_8784, whole genome shotgun sequence
MAATGRDALRMLAKTEYNLIFIQKTPDIALQIRSSETELNREPAIISVLIPRTPVKQDPK
YLDAGIDYVLENPLIGDKIEEVLEKFGLGGG

>gene_542|GeneMark.hmm|115_aa|-|2207|2554 >NVVL01000015.1 Rickettsiales bacterium isolate NORP64 Contig_source1382A_8784, whole genome shotgun sequence
MAVKIRLARGGAKKRPYYRVIVADERAPRDGAFIEKLGTYNPLLKRDDEDRVSLKADRIE
HWLARGAQPTERVARFIKEAGIKLPDSILKKMEIKQKAQTTKPSKKELKEQAKAK

>gene_543|GeneMark.hmm|57_aa|-|2566|2739 >NVVL01000015.1 Rickettsiales bacterium isolate NORP64 Contig_source1382A_8784, whole genome shotgun sequence
VSKKKSKNLLVKLVSTADTGYYLVKKRNPKTLTEKLSFRKYDPKVRKHVEFKEEKIK

>gene_544|GeneMark.hmm|505_aa|-|2855|4372 >NVVL01000015.1 Rickettsiales bacterium isolate NORP64 Contig_source1382A_8784, whole genome shotgun sequence
MLLRSGIVIAFFTMLSRLFGLARELFIAATFGTSGIADSVNVAFKLPNLFRRIFGEGALS
AVFIPMFNEKLITSKDEALKFSGEVFALLLLILTVLTIILQISMPYMMLIIAPGFFNDPE
KYELTTTLCQITIPYLIFVSITALFGGMLNSVKKFAAFAFVPIIMSICVIGITYVFQEKH
SPHFSVAYALVIAGIFQVAFMYVCLKRAGLTFPFLFHPKDREVKKLLKNMGPATLGSGAQ
QLNLFLSQSIASFLPGAVSILSYAERLYQLPLSLIGITFGTLLLPELSRIYKQKDFAKAN
TLQNNSIKIALAISIPAMFGLFVLSAPIIHIIYEHGAFTPADTALTANALAAFSLGLPAF
VVAKIITPIFYANLDTKTPLRITVYSLLLNTILNVILMIPFGHIGIAIGSSIASWFNVWM
LARHSGKYGGFKIHPDIKLFSAKIVLSSSCMLASIVTFNYYYGYLFYSKSFLIKASALTG
VVVLGVFVFVLSSYFLKIHTKLLER

>gene_545|GeneMark.hmm|55_aa|+|4371|4538 >NVVL01000015.1 Rickettsiales bacterium isolate NORP64 Contig_source1382A_8784, whole genome shotgun sequence
MSFKFMFLESVSNLVFCTIFSRNQGISRLFLEGFLGKMDGWMVIAIKIRSLSLSL

>gene_546|GeneMark.hmm|68_aa|-|4670|4876 >NVVL01000015.1 Rickettsiales bacterium isolate NORP64 Contig_source1382A_8784, whole genome shotgun sequence
MTTGIVKWFNPTKGYGFIAPEDGSPDVFVHISAVESAGFNNLNEGEKLEFEVSDNKGKQS
ATNLKSLG

>gene_547|GeneMark.hmm|303_aa|-|5049|5960 >NVVL01000015.1 Rickettsiales bacterium isolate NORP64 Contig_source1382A_8784, whole genome shotgun sequence
MQIRIGTRKSKLALIQTNMVIRVIERHFPGAECIIVPMVTTGDKITDRNLYDIGGKALFL
KELEEALLANEIDLAVHSLKDVPGILPEGLLVAAVLEREDARDCFISADYKSIQELPHGS
VIGTSSVRRKIIIQQERPDLKLVQFRGNINTRLDKLKNSDIDATILAAAGLIRSSLFDPA
YCHMIETSQMLPAAGQGIIGIEIRDDDALTREICAKINHLPTWSVAQAERSFLAYLDASC
RTPLSAYAVLSGGVILARYMLSDDDGRFVFHHEEEAEEEHASDMGESAGEKLLARLKAAV
MQQ

>gene_548|GeneMark.hmm|318_aa|-|5997|6953 >NVVL01000015.1 Rickettsiales bacterium isolate NORP64 Contig_source1382A_8784, whole genome shotgun sequence
MIKFDREKDKVAIIAPASGANDDDGVLDVEKSMEKLGVAMNLFQNAGFRCEYDDKIFAGD
SLGYFAAPRAERLRQLKNALEDPEVKIISAFRGGYGCSEIVFDCLDVNPSGAKILIGFSD
ITVLHFLFNQHYNFPSIHTLVNDSYKHMFGDVLSVLSGGDITLDLTPLNDAATSATDALV
PSTTTAISAPAISAEMLGGNMTLICSLLGTKLHPNISGKILFIEDVNEKGYRIHRSLMHM
HNAGLFKEAAAVIFGDFTKADKHAEATLRVFSAEYLGGIAAYKTTGIGHGRVSHPITIGG
SGKIEGNQLVVSSSFELI

>gene_549|GeneMark.hmm|604_aa|-|7026|8840 >NVVL01000015.1 Rickettsiales bacterium isolate NORP64 Contig_source1382A_8784, whole genome shotgun sequence
MTDHKFIRNFSIIAHIDHGKSTLADRLIEFCGGVQKREMSQQILDSMDIERERGITIKAQ
TVRLEYKAKDGNIYHLNLMDTPGHVDFSYEVSRCLAACEGSLLVVDASQGVEAQTLANVY
QAMDNNHDIVPVLNKVDLPAAEPDQVKEQIEDVIGIDASNALLISAKTGLGIEDVLEAIV
HRLPPPKNEFVEKESPDALKALLVDSWYDQYLGVVILIRVFQGSLKKGMKVKMLSTNKTY
NIDNVGYFTPKKVMCDSLEQGQVGFFTASIKKVTDCKVGDTITENGEEVQMLTGFKPNLP
VVFCGLYPTDASEFETLRDSLEKLHLNDASLEFETETSTALGFGFRCGFLGLLHLEIVQE
RLDREFDLDLITTAPSVIYKVNLTNGKQMEVHNPADFPDVQTIDSIHEPWIKATIMVPEE
YLGSVLALCTDKRGVQVDMKYIAGRIMLIYRLPLNEIVFDFYDRLKSCTKGYASFDWEMD
GYEVSNLVKLSILVNSEPVDALSTIVHKSRAESRGRDLCNRLKDLIPRQLFQIAVQAAIG
GKIIARENVKAMRKDVLAKCYGGDVSRKRKLLEKQKAGKKRMRNVGNVEIPQSAFIAALR
VGDS

>gene_550|GeneMark.hmm|351_aa|+|8976|10031 >NVVL01000015.1 Rickettsiales bacterium isolate NORP64 Contig_source1382A_8784, whole genome shotgun sequence
LGGLFDPVSLKQRLDELEEISLDKDLWNDPKKASTLLKEKSLLEKTLGLYNHINDGLAEN
IELASIAEEENDTKMADQIQKDLEAIAKSAEKFETECLFSGEADNNNCFLEINAGAGGTE
SHDWALMMMRMYMRFGERMGFKTEIINIINGEDAGIKSCCIKFKGDMAYGWLKTESGVHR
LVRISPFNSAGKRMTSFASCWVYPEVDDNIDIVIEDKDLRIDTYRASGAGGQHVNTTDSA
VRITHIPTNIVVQSQNDRSQHRNKAEAMQMLKARLFDLELKKKTEESDKQNAAKTDNGWG
HQIRSYVLQPYQMVKDLRTNYETSNSAKVLDGDLEKFVSASLAANANKING

>gene_551|GeneMark.hmm|443_aa|+|10090|11421 >NVVL01000015.1 Rickettsiales bacterium isolate NORP64 Contig_source1382A_8784, whole genome shotgun sequence
MKEKRIGIFGLGVTGESVFTTLDGADKVDGTDKEIICWDDSEDNRNRFQTKFEHSTLAPL
SSPEWSKLDTIIISPGISKLHEVFSLAASHNILISSDVQLFIDNNPDSELIIITGTNGKS
TTTALIGHILTCSGIDHDIGGNIGPAVLSLPKNKKYYVLELSSFQIDLLKKFEPAISVLL
NLTPDHIDRHGSFDKYCAAKARIFDNDGLKIIGTNSDKSKELYEKLKKTGDKKIVPISSD

EGINCTSDTITDNFFDRKSYHMETLVNLTGVHNCENIAAAFAVCRAMGLKAGEIIQHIKS
FGGLKHRMQFLGNIENISFYNDSKATNTNSAASSISSLDNVLWLAGGVFKEDNLKPLGNS
IKNIKKAYLFGESKLLFAEYLEGIAEYKIYDNMEKAFYSAVRDAKIEGESANILLAPACT
SLDQFKNFEDRGERFIQLFEAIK

>gene_552|GeneMark.hmm|371_aa|+|11422|12537 >NVVL01000015.1 Rickettsiales bacterium isolate NORP64 Contig_source1382A_8784, whole genome shotgun sequence
MYGFGKNNFVQRWWRGVDQQLIIALTILIAFSLMLVTTTSMAIAEKIGLRDNYFSLRQVI
YLFISAGLLILFSSFDKKWIKRIGILGFLVSIVMMILIKFYGYEVKGARRWISIAGFSYQ
ASEFLKPFFWIVTGWILSLKYHEDFPSFTVCAALYIIVASLLITQPDFGMLVLVTAVFGT
QLFVAGLPLIWILFASITGFLGVISAYLLLPHVASRINSFLDPSESNYQVSKSILAFEQG
GLYGKGPGEGAIKQYLPDSHTDFIFAVAGEEFGGIICMIIVLVFAFIVLRTLINLRTEDN
KFVQLAAIGIITQFGLQATINMGVTLNLLPTKGMTLPFVSYGGSSTLSIAIAIGMLLGLT
KKQALFSRNRM

>gene_553|GeneMark.hmm|376_aa|+|12628|13758 >NVVL01000015.1 Rickettsiales bacterium isolate NORP64 Contig_source1382A_8784, whole genome shotgun sequence
MKKEKTILLAAGGTGGHLFPAIALSEELSKHNSNILECNILECNIHLITDLRCKKYLTPK
LPFKAHIVDLHLNLNGIFAKLKSAWKIVAACYRSFFLIRKIKPDVIIGFGGYPTFPSMLI
CKLMHIPIIVQEQNSILGKSNHFFAKSAKLIALSYSNTSNIDESFKSRICISGDLIRSSI
LKLAAKKGSNAKKFRSKRFHIFIVGGSQGARIFSELIPPALALLKKQHPGADLFITQQAP
KTEQPRIEALYDELNIKYELAEFFHNISEIYDKAQLVIARSGAGTIAELAATGLPAIFIP
FPSAAQNHQYFNAKSIEDMGAGWCFVQEEVSAPILAAKLSELLEDRSLLEDASRKLLARK
TDGSKYLADTILKIIQ

>gene_554|GeneMark.hmm|726_aa|-|1|2178 >NVVL01000016.1 Rickettsiales bacterium isolate NORP64 Contig_source1382A_9413, whole genome shotgun sequence
VFGILLTGKCRYMLFDIDVIIVAAFLIATLVIGLGHGGDVKTIKDYALGGRNFSTGALVA
TVVATWISGSGFAITISRTYYDGLYYLVATFGMMISFLITAFYFVPRMGEFLGKTSIADA
MGDLYGKKVREISAISGIIASAGHIAVQFKVLGDIVSYFTDSNSTLTIIVAGIAVTAYSA
YGGIKSVTFTDVLQFFTFCFAIPLIGMMIWNKLYLVDSFNMSHALQIDKFNYEKVLDLKN
HRFWEMIPILIYFSLPGMKPSLFQRISMGKNLAQIKKVFIISAFLIIFVKIAIAWIPFLV
FNANPNIEQGQLFGYIVDNYTYTGLKGVFVICVIAMSMSTADTEINISSVLFANDACSGF
QISPDKRLLLSKIFALGLGIISTFVALSTADLLDIILSTASFYMPIVSTPFMLTVFGFRS
TGKSVLIGMGGGFITVVAWKAMGIQANPIAIAMLVNLIFLMGSHYLLGQTGGWVGIKDRS
FLEKQKDENIRKRREIFEKLKTFNFFDFCKKYAVASNITYVGLGIYFVFYTITTMYSIEA
TLLQGRGQIIFAIYQIMMVTGTAMAMYPIWPVSIKSSLKELIIQTWWPIAIFYMLILFST
FFVLVNKENHLQFVIFAINMIIAIIVLGWRTASIFIATGVYLSVQFYKYYMGIDYIDISI
GSPQFVLMYILMLIGTVTIIFLKPQQEHQKQTEHRVGELSDEVGTLVTVVSSRDSQIITL
DTKVSG

>gene_555|GeneMark.hmm|81_aa|+|2218|2463 >NVVL01000016.1 Rickettsiales bacterium isolate NORP64 Contig_source1382A_9413, whole genome shotgun sequence
LHKCEHASTNNDPRYSRKLGLDGGFSMPQTLDYYNSKSFRFGRELSSFLQLFEAAHAVRR
GIYPRFQGGKSKIRGILGLRT

>gene_556|GeneMark.hmm|222_aa|-|2567|3235 >NVVL01000016.1 Rickettsiales bacterium isolate NORP64 Contig_source1382A_9413, whole genome shotgun sequence
MEEEQRYSKLNRTFARRIGKSLSNLNKDLLSGELPKYSYSSEKLLEANRAGKKTFLEIGF
GMGEHFLSQLANHPDAVHIGVEVYLNGVANVLKQLEHNCAELMLWPDDIDLVLEDMPEAS
IDGIYVLFPDPWHKRRYLKKRMFNKERVASFGSKLKAGGFIIFASDIEDYFDSACDILRE
DAGFVIEGDDFSSEHAGYVQTKYHQKAIREGRRPQFVKAIKR

>gene_557|GeneMark.hmm|277_aa|-|3328|4161 >NVVL01000016.1 Rickettsiales bacterium isolate NORP64 Contig_source1382A_9413, whole genome shotgun sequence
MTDKRDRAAKWFETLRNNLCSAFEEIEQDFARDKDISPEKFKQTPWNREGGGGGVMSVMH
GNVFEKVGVNISTVQGEFSEDFRKKIPGGEDDPRFFATGVSLVAHMCSPLVPAMHFNTRY
IETKKGWFGGGGDLTPMYEDALETAQFHESFKTACDKHDEGYYPRFKKECDEYFYLKHRK
EPRGVGGIFYDYLSNNFEDDFAFTKDVGLAILDVYPKIVKNKMHKPWSKEEREHQLIKRG
RYVEFNLLYDRGTTFGLSTGGNVEAILMSMPPEVKWP

>gene_558|GeneMark.hmm|111_aa|-|4204|4539 >NVVL01000016.1 Rickettsiales bacterium isolate NORP64 Contig_source1382A_9413, whole genome shotgun sequence
MKSFRLGIFAEYIAMLLYAVRFYGILHHRMKTYVGEVDLVIARGRQLVFVEVKARKNGIC
EGIVSSSQQNRITKAAELFISRNSRFAGYDVRFDLVVIEPYKWPVIIQNAW

>gene_559|GeneMark.hmm|423_aa|-|4555|5826 >NVVL01000016.1 Rickettsiales bacterium isolate NORP64 Contig_source1382A_9413, whole genome shotgun sequence
MNKRRSIFLSAISGNVLEYYDFTVYAVFSLAIGRAFFPGDSEIVQVFSSLAVFAVGFVTR
PIGGIIFGYIADKHGRRVSLITSMLGMTIPTFAIGLIPDYSEIGHYAPMILVMMRLLQGL
CISGEGAGAAIFILEHYQNLRPGFTAGVVHASNIAGTLMATLVGIALATFMPDSEFAWRF
AFILGGVMGIIGFYFRLRVSETPIFIMLAEKKKTLKAPFIHVIKTAWRSMLITFCLGGFA
SSVVYLIKTYINIFHCNVLHFDDVTARAFLAYSSVIMLLTMPLSGHISDSIGRFRTITIS
SIAVLVLALPCFMLLSCTDMLRQMLALTMLAVLGGMVAGTAYIFIISLFTPEQRFSGVAF
SYNLGIAMFGGTSAAISRWLLEVTGLHYAPAFYIMLTATMFLTATYFLRHTIRELLDANL
KNK

>gene_560|GeneMark.hmm|219_aa|-|5947|6606 >NVVL01000016.1 Rickettsiales bacterium isolate NORP64 Contig_source1382A_9413, whole genome shotgun sequence
MSVSKIGAGHTHAAEDIQVEHDHIPANGQAEHRLVHPRVAVLHNPLHTFGLDLGTVTLLS
MYTAFSSGTLQQQIDRLVESEEWDVIISLFENAVDNIDSPALKYLLPCIEANPQALGYII
AWIDDYGHGDFSVLSRIRDNSSDPSGVGAHASGKLAVMHMEALQEEKFDMLVDLLRLVVG
ATDGVDSPTLVKLESELPEDPPIAIAGEALIESTDTPIN

>gene_561|GeneMark.hmm|401_aa|-|6806|8011 >NVVL01000016.1 Rickettsiales bacterium isolate NORP64 Contig_source1382A_9413, whole genome shotgun sequence
MVMFKDLNPISVFVLIVYPLTIITLAVLYALNNSLGWFELGLFFAGYYISTISVGVGLHR
LWAHDTFKTNKFVEFVLMLLSAGTLQGPVLSWASNHFEHHTYTDTEKDPHTPLKYKSRVK
GFWWSHMGWMLYGTGSYQSVNRVTMLKLGKNKLLRWQFKNYWQLGLLMNLLVPFMLGYAI
GGNLTSACAGILFIGVGRALQQQTTFFVNSLCHFAGTHKYVSGTAGDVWWLAICLLGENW
HNFHHAFPSDYRNGSRWYQFDVHKWIIYLLSKCGMAWDLKVTTEVRINAKMTQAAKALSK
LQTEKLESLNKTINDLSVIHQETLEKLELSSVKAKIKLKKSFAKMQENIKTLKLQLDQQF
KELKAPISDKVVSLITKKARKLESAMQKLCREFEQSNMQAA

>gene_562|GeneMark.hmm|250_aa|+|8364|9116 >NVVL01000016.1 Rickettsiales bacterium isolate NORP64 Contig_source1382A_9413, whole genome shotgun sequence
MAGHSKFKNIQHRKGAQDKKRAKVFTKLVREIITAAKIGGVDINNNPRLRTAIVGAKSQN
LPKDRIEKALAKASDPSDTDNYTEMRYEGFVPGGIAIIVETLTDNKNRTVSDVRSAFTKH
GGNLGESGSVSFMFDRVGKIEYQASIASADEVMESAIEAGADDAESTDSEHIIYTNVESF
ASCLEYLSDKYGAPLASEIEWRPQNTIMLEDLERAEKLLKLIDALEESDDVQSVFGNYEL
SEEIFNKLSS

>gene_563|GeneMark.hmm|246_aa|+|9127|9867 >NVVL01000016.1 Rickettsiales bacterium isolate NORP64 Contig_source1382A_9413, whole genome shotgun sequence
MNKQIIILAAGHGTRMKSNLPKVMHDVDGKPMIEHVITNCYKASDDLVLVHSHHLQPYLD
QFTDRCKLANQKEQLGTAHAVAAAKDLFANEKYIGVIYGDNPLITDEIINDLFTHLEQTG
SKAVTLAFEYAKPNQYGRIVTDAEGNFERIVETKFANEEERKITLCNSGIMAFAPGILEE
YIDKCLAPNPEHPEREFYLTEIIEICSLAGEKVSYFKSGNPNLVVGVNTQDELVNANSII
QNHKEG

>gene_564|GeneMark.hmm|923_aa|+|10011|12782 >NVVL01000016.1 Rickettsiales bacterium isolate NORP64 Contig_source1382A_9413, whole genome shotgun sequence
MEKTAEKKPVLIIDGYGFIFRAYHVQPPLTSPAGAPVGALYGFVSMLLKLINDFKPEHAV
IVLDHGGKNFRHDLYKEYKANRPPVPDDLIAQLKLVRTAAEALNFTCLSKQGFEADDIIA
TLATKATSIKRNALIISSDKDLMQLVNEHVRMYDPAKAKHLTDEDIFNKFGVPPSKVREA
QSLIGDASDNIPGVAGIGVKTAALLINEFGDLDGVLASIDKIKSKRQQNLLTEYRDNALL
SWKLVGLDCAVEIDQDIENFHWAPPKAEEITAFLNEYGFKSLNKRIENLFNIKSLEPSAQ
ESKANKQRPERANITQGSIVRIENPAQLEPLFKEIEICGMLAVNIHQNGTEHELVLCAGA
DLYIVPYIAESPVALDLFSYTNSKSSVPNFNKQIHELLENKAIKKITYNLKELLKLCDCK
IQSAEDLQLMDYVLSAGGKSKNLAEIIEFYTHNKPGDNGTNQPSADETSEPGDLATSNLG
KKTTGKAAGGGGASITSYFIACYKALDCALIQDKTLYLYKSIDLPLCHILRNMEKEGVKI
NTQHLVRLSGEFAQKISKLEKEIFAITGKEFNIASPKQLGQILFDEMKLPFAKMTGKTKS
YSTNAEILEKLQDDGHKIGGLLLEYRHFTKLKNTYTDALPKQTDPRSGRIHTTFLQALTS
TGRLSSANPNVQNIPTRTPEGSSIREAFVASEGNKLISADYSQVELRILSHIANIEPLKQ
AFRENRDIHAQTASQVFELPLDKITPEIRRSAKAINFGIIYGMSAFGLARGLAISKQEAA
DYIEKYFKEYPGIQKYMSETIIAAKENSFVANMLGRKCFLPSINNKNHTLRSFGERAAIN
APMQSLASDIVKIAMIKLDQEFTKRSMKTKMILQIHDELIFESPSDEVESATKLIKSIME
GAFSLDVNLDAGISSGSNWREIH

>gene_565|GeneMark.hmm|75_aa|-|115|342 >NVVL01000017.1 Rickettsiales bacterium isolate NORP64 Contig_source1382A_10295, whole genome shotgun sequence
MSSRKEEISVVTTSNKTLIVSEEIKKTVCSDIGLETLILNDGIGYVDRSYNKLTELIMPG
SATHLDCSSNQSHLL

>gene_566|GeneMark.hmm|263_aa|+|920|1711 >NVVL01000017.1 Rickettsiales bacterium isolate NORP64 Contig_source1382A_10295, whole genome shotgun sequence
MHSSYYLTGEIEGKIYLVYNKNNIPYVLSDTSDGLVFDPRMTNPLELTISLSNNGMFIEL
ESGHLGSINNIATNVTNRELLLENSPNVNSDNSILSGVCYTISNSNGLLLSFQIYEPDLN
TLVDGIATEILKIGNDPIINLITNCIRFLPLTYYIQGSCLSIDNTDMKSEKSWVTEIGTP

ILGFTNIDECKVGIKYKYCGRNIGCNLLCKGPCKNNTLCFYNNKVDGYSCKSKDKGNDGK
TIIIIILIILLVVSFVFLVIRRV

>gene_567|GeneMark.hmm|276_aa|-|1891|2721 >NVVL01000017.1 Rickettsiales bacterium isolate NORP64 Contig_source1382A_10295, whole genome shotgun sequence
LDFGELTEKITMDGYSTVTHNAMFWLDSKTEKKITKNIKYKFFYDASKTTIDTYWKRIIL
RMSKGSFPKWFSFYDGNLVYRHRKIHEQISITKLCPIICSVECIDFIRSKTSMMSKMDKM
ELDSARKKHTNIKTWKCVKSAKFRTSLINDFIKRTVRENNCNFREMKGLYSLLNISVELK
HIGEEDIVIVDNRIDNIKGLFLSDDGFKITGTHEKQKQKPFIKSDIYMSPKHKRKNVKNV
KNIVDLWKDNMIKMCDDIQDGTIIICENGNIPLILL

>gene_568|GeneMark.hmm|69_aa|-|2787|2996 >NVVL01000017.1 Rickettsiales bacterium isolate NORP64 Contig_source1382A_10295, whole genome shotgun sequence
MEGGSVVILVGSVILLIYIIFVFICYQQKKFIFSPYKQPKLENGFQPNGKVTKLTPIEKM
NRANRILGN

>gene_569|GeneMark.hmm|778_aa|-|3063|5399 >NVVL01000017.1 Rickettsiales bacterium isolate NORP64 Contig_source1382A_10295, whole genome shotgun sequence
MLSEDKILNNAIAIAKGTETKALRILADSMGHSTTSYNDHKMAGIFLMECVLREGPKNIG
EYLEVMSHRLNKRTLSFMVSHSTIIDKELKKNYLVDLNFDWISASILVRSYLSRPQYGSA
VFETPSIMRMRIAIQMCSHEGIAKVLDAYGEMNDKLYMPASPTMFNAGMMSGQMSSCFIM
SSQDTSESILDNVKYTSLVSKHNGGYGLDVSRLRHSAIGSVGRSSGIIPMIKMYDSSTST
FGQTGKRAGAGTISCRLHHIDIVQFINVRSNEGSHTNRVFTVGTAIYTPWVFWERVRSNG
IWSMFCPNLTKSLNDIYGIDFMKEYERIENSNVDRKTIKARVLLSMISESKRKCGYPYIL
NGDSCNYKSNQKHLGYIRSCNLCTEIIQYAPEDEITSCNLHSITLPSHMKDGTFDYSALC
LTAAMCVNNLNNIIDNNFYVLGDKTKTNNLDNRPLGIGIQGFFDVVYLLDLLPNSNEVIE
LNKKIFACIYFSVLAASVDLAIKYGKCKGHDGSPMSEGKLQFDLWAEEYKLLKDNKLIED
SVRKEEDDIPLSPSEWGQLEIVLSNGEVIENTWESLRIFIIKYGLRNTLLTTIAPTATSA
QLMGSCEQVECHQANIYSRILHTGNVTVINKYMYRDLSDIGVWSNEISDLIKADGGSISK
LEAYVKSSIEDYENVIDMHRLRYVVKKYKTMYELSQKFMINLMADRSRYIDQSSSLNIYM
GDPTTSDVTTAILYANAKGLKTIMYYLRRKAANKQPMITINSKIKTFVDGYKECLSCQ

>gene_570|GeneMark.hmm|356_aa|-|5462|6532 >NVVL01000017.1 Rickettsiales bacterium isolate NORP64 Contig_source1382A_10295, whole genome shotgun sequence
MKPNTPDVFSRGYSVFLDSTKISSDDYITLMLIISKPDIPTYLSSLCTIKGDIPNDIYDK
KVIEYAKNKISEFVGYDVGEINTTVNSKRLYNLLKDIEHKKEWKSYSMLLKFFGIHSPCV
NVDMIKTLDRFGYDRKYRDYINVDKFTALYYMDRTHKNFANSLVMSYRGCEILPHQSEFL
VAVIKHGIVLQALELGLDWSFIEQMSGILKKSDTKKCNEQVASPGKGDFSKIFNLPYGFA
MTVSFAYSINSWYALENKIRELEWKVLRIPRLGNHTSIIENSGISLYEDLCESKTLYSLV
NLLMSSKNKVHKILLYDIENELHSKSFICDIQNKTMLSELSVLDMASELTNGGELG

>gene_571|GeneMark.hmm|109_aa|+|6519|6848 >NVVL01000017.1 Rickettsiales bacterium isolate NORP64 Contig_source1382A_10295, whole genome shotgun sequence
VLGFMSFYKFIFKLKMSSKQSLILWAVIFAISYYVFRYKRIRNLSAVAISSFIALLVSFM
FYVPTTPTDIFESDGMNALYAFVASITYIFLIFYLLLKATSDVESPMSH

>gene_572|GeneMark.hmm|117_aa|+|7042|7395 >NVVL01000017.1 Rickettsiales bacterium isolate NORP64 Contig_source1382A_10295, whole genome shotgun sequence
MGDVEILELTVGTPQFKDTVKKHGDLVDFISWYPTFILFTMDSWNGNKLNGVVYCGKLVG
DNISFDNTLKFDPKYTNMVDWVIKSKSVLESKSQLPFEDDEVVYEPGYSSVVIDNDY

>gene_573|GeneMark.hmm|113_aa|+|7446|7787 >NVVL01000017.1 Rickettsiales bacterium isolate NORP64 Contig_source1382A_10295, whole genome shotgun sequence
MTQLKDECKTFGIYSSIEALLLKDSFDPSVDKVNKYMELDEMVAMLSGLPLEHGNIIYAL
MIHYNNTHEKSISVPLYNGNRLSPKRGMSFSLDDIPKGLILIISLYLKCITDL

>gene_574|GeneMark.hmm|114_aa|-|7782|8126 >NVVL01000017.1 Rickettsiales bacterium isolate NORP64 Contig_source1382A_10295, whole genome shotgun sequence
MSNTTETRWFKIFVSVITAFVVGISIANVVYYNRIRTGTCNAITRGEADAMFWINIIVSI
SSGLLFVWSLWRLVFSKDLRSQITHGISGHAKSERSGLERKHFKGLTNFSSDEL

>gene_575|GeneMark.hmm|398_aa|+|8341|9537 >NVVL01000017.1 Rickettsiales bacterium isolate NORP64 Contig_source1382A_10295, whole genome shotgun sequence
LNQKVSNKKKELYIMEETQGSEFLYPSLEPTSKVGPNKLWHYTVELEARSKKHMVYNQIL
LLGDETSRGYYYVSNVVLGGSANNWKAKSGKFNSTGDELLGRLERHSYVYSPSLRIAGPR
TVITDIFREPGKFGLPSGFDAFVYDSEIQETGGRYLKSYIKEIENKRDASKGEKIPESAL
TNASYLNEIHKALKAAKMGKKSKSHKNSNKMSLGAKYMEIITINKTDEKKRTLDVTKADW
EKSTGFLKRVKPVTDNSKAGAGSTYKQTIHIISETQDNYERALETLIDDVNLDATVKAYI
KEHLDAAIRDWDAWKSRKGKKSKKVPKSSTLSAPRAYSRVKTGRGPVKVDFPEVVVDFSE
SEEEDESEEEDGVTLDGDGDSEEEEEEVVEVDEGNIFN

>gene_576|GeneMark.hmm|249_aa|+|9706|10455 >NVVL01000017.1 Rickettsiales bacterium isolate NORP64 Contig_source1382A_10295, whole genome shotgun sequence
MNFVKGESDDMLMEATSTLSKYRNCFDRSTDSDEIYHVSPFVKLLEKYKRKIITLHLSSD
SYSNDMWLSNTSEILQYGCNTGISSDIRIYISYLYNLSSHENKKIIMLKLYRTLMAACKS
SFNENNIDDNDIQDLQKSIEYLQATKSPSNIAETLHIPSNKFDPKEMIENLLGSIGDLDL
SDPGQCMKTLMSMMGNMGIDVETTMKIISGMSGIDKGTESDIKEILDNGGEIKAILGAND
TDDVTDENI

>gene_577|GeneMark.hmm|145_aa|-|10436|10873 >NVVL01000017.1 Rickettsiales bacterium isolate NORP64 Contig_source1382A_10295, whole genome shotgun sequence
MSTKMSLVSSIHRASDPRIFGPGLWFAIHTLSFDLNEKSDELKFSAMLSKLINNLKCPKC
KKHAIAYLKRKPITKRQNAKHLGEDVSVFRWTWIFHNYVNSILGKPLVSFIDAFDMFRNN
TNVCSKGCGSGKSNFRGITPIYSHQ

>gene_578|GeneMark.hmm|262_aa|-|10882|11670 >NVVL01000017.1 Rickettsiales bacterium isolate NORP64 Contig_source1382A_10295, whole genome shotgun sequence
VHILKEPNYQFRFFTINIDYKMSCTHCKKYEYDCDSQGYKKQCCEGITKVIAYAGEKYLC
GYKQEWSCDRKPKCKLAVVTCMDSRLDVPRLLGLCPGDCDIIRNAGGVVTDDVVRSLVVS
RELNGTVEILLIGVLDTVQTVYDGNTLASELEDDLCQSPEFLFETFSSAEKKLKEGVMRL
LRNPFIKGKECIKAYFYVTCDCTLGDNDYVAGELVPVCLDDLKEKACLHPKEKCKPQPCE
YKDTKQCHSSHHYQHDTSCHDF

>gene_579|GeneMark.hmm|276_aa|+|540|1370 >NVVL01000018.1 Rickettsiales bacterium isolate NORP64 Contig_source1382A_10570, whole genome shotgun sequence
MPELPEVETLKCYLEGHIIGETIASLEARRDNLRYELSPYLEQHAVLGVVSGVKRRAKFL
IMELSNGYMLIFHLGMSGRLTMQSVDYQPQKHDHIIISFQSGDQLVFNDARRFGMVYSCR
ADMLSEQSFLNRFGQEPLEEGFDACYLLKQLAAKKSPIKTAIMDNKIVVGVGNIYAAESL
FMSKINPLTKANTLGAGAVSALVKSIKRVLQKAIHAGGTTLKDFLGGDSKPGYFKQELQV
YGRKGLPCYICKTPIESVKQAGRATFFCPNCQQNKD

>gene_580|GeneMark.hmm|205_aa|+|1448|2065 >NVVL01000018.1 Rickettsiales bacterium isolate NORP64 Contig_source1382A_10570, whole genome shotgun sequence
MRKKKLKIKQSTINHMLKFFSFSKAPVRSILAVLTLGIGIGVAYEEMFGIGTWHSFHPKT
DKLNVCFTPPSGCGDLIAGQIVSAKSSIYMQAYGLTSKPIIQQLKLARGRGVTVRILLDG
GNLSDNESIYKELKAAHITVGFDKMSGIAHNKVIIIDKKKVITGSFNFTGAADYRNAENV
LLIDDKKIAKQYLQNWESRNKASKK

>gene_581|GeneMark.hmm|129_aa|-|2092|2481 >NVVL01000018.1 Rickettsiales bacterium isolate NORP64 Contig_source1382A_10570, whole genome shotgun sequence
LIIYSVLIIFMQFPAVSKKIHFSFSTAITALGISLILLAMPYLFYIEYLYGAAIWCSLLA
FEEMFAPYIDYYAAKHNAILTKEVSISIGGGLSVFWMRYFHDPTILIMFSLLLLLLAHYI
LALRIRNSS

>gene_582|GeneMark.hmm|888_aa|+|3046|5712 >NVVL01000018.1 Rickettsiales bacterium isolate NORP64 Contig_source1382A_10570, whole genome shotgun sequence
MNKLIYHFGGGDAEGDMTMRDILGGKGANLAQMCKLGLPIPPGFTISAELCQIYAKRESP
LTPNTEKELRKSIAKLEEETDKKFGSNDNPLLVSVRSGAKFSMPGMMDTILNLGMNDDVA
KALAARTDNPRFAYDSYRRFLEMYGSVVLEISSHLFEELLDDHKANHDVMRDSDLTVEML
QEIIEKYKAIILKYTGRPFESDPFKQMKHAVSAVMKSWMSPRAVVYRKINNLTDAIGTAV
NIQSMVFGNKGENSATGVIFTRCPSTGDNSLFGEFLVNAQGEDVVAGIRTPSPILAVAGG
KGDSMEEKMPKMVRELSDICQKLESYFRDMQDIEFTIEEGKLYILQTRNGKRAAASAVKI
AVDMVGEGVISKQEAIMRIDPESLNQLLHTAIDYSNSPEVIATGLPASPGACTGIAVFSS
GEAEELSHHHKVILVRNDTSPEDIKGMHVSVGMLTARGGMTSHAAVVARGMGTPCVCGMR
GVVVNEAAQTLTTPEGRTIKQGDTITIDGSSGKVMVGDVKLVYPEFSDEFKTILEWADSE
RTLQVRANAETPLDAKVAIKFGASGIGLCRTEHMFFDSNKILIVREMIIAMDYEGRMRAI
RRLQPLQIADFKALFEVMEGKPVNIRLLDPPLHEFLPTTENDKSELAKLLNVSSSVIEHR
LQTLHELNPMLGHRGCRLGISYPEIYIMQVRSILQAMHELDVEKGIKSNLELMIPLISEV
KELERIKEYVVKTIGEMEAEYSTKFDIKLGTMIELPRAALTADKIAKHVEFFSFGSNDLT
QTTFGMSRDDIGSFLPDYIEQKILTHDPFVRIDEEGVGQLIDIAIKRGKKAKPGLKMGIC
GEHAADPESIDFFYKIGMDYISCSPYRVPIARFAAARSKIKLSKGWNR

>gene_583|GeneMark.hmm|358_aa|+|5709|6785 >NVVL01000018.1 Rickettsiales bacterium isolate NORP64 Contig_source1382A_10570, whole genome shotgun sequence
MSLRDNLNKILAKHQELSDKLASGIVGEEFVKAAKEYSGLETVVEKITKYNKALSDLEDA
KEMLLEEGLDAETKDMVEAELRELGAAVPKLEREVKIALLPKDAADGKGAIIEVRGGTGG
EEAALFASDLFDMYQRYAAIKGWKFEILSISEIGVGGYKDASALIKGDGVFSRLKFESGV
HRVQRVPKTESSGRIHTSAATVAVLPEAEEVDVKIEDKDLRIDTYRASGAGGQHVNTTDS
AVRITHIPTGVVVAIQDEKSQHKNKAKALKILRSRIYEEERRALDAERATSRKGQIGSGD

RSERIRTYNFPQGRISDHRINLTLYKIDEVLREGRLDEFIDALISDDEAKRLAEVSDY

>gene_584|GeneMark.hmm|45_aa|-|6779|6916 >NVVL01000018.1 Rickettsiales bacterium isolate NORP64 Contig_source1382A_10570, whole genome shotgun sequence
VQPARGSSSELIVAAALVQCVATPTARLGSPRAQLPQAQASPSHQ

>gene_585|GeneMark.hmm|165_aa|-|7040|7537 >NVVL01000018.1 Rickettsiales bacterium isolate NORP64 Contig_source1382A_10570, whole genome shotgun sequence
MQEHSTVVVEEQKSMLIENLTVLGSCTITGSFEIRDIRFQSFSTLTVVSETETCLLQNKT
STSVSPNISFNSETLVLLSNIPTNAQTITAEHHEALLAYLQHPEMLKLFNPTADMISGIN
DYTDEHLMEILSNTLLLAESTPVALCRIIGEYLLTEEVAIAPEFE

>gene_586|GeneMark.hmm|172_aa|+|7536|8054 >NVVL01000018.1 Rickettsiales bacterium isolate NORP64 Contig_source1382A_10570, whole genome shotgun sequence
MYKDVSSLGSFIVKVTSLEMLSLPDISGFFLKKLKLSVSRVTELMVVKSSFVVSALQASP
AAKGKLNTTEDVSSPTISTIPTMLDLVLDIIHFPFSITYLQQCAVTLDKISIFLNFVNLS
FLIKIKKTFKNLKSLQKFVSVRSYVCRPKIGGYFRPADVASNLQGNIILNKH

>gene_587|GeneMark.hmm|433_aa|+|8114|9415 >NVVL01000018.1 Rickettsiales bacterium isolate NORP64 Contig_source1382A_10570, whole genome shotgun sequence
MSNIVNDSKNFFKVALLASLGSGLEYYDFVIYGMMVKYIGDIFFPNHLEFISIMQAFSVF
AVGYLIRPVGGVIFGMIADSYGRKKSFILVMLTMALSTLAIGLLPTYEQAGIIAPILLVI
CRLFQGVSFGAELPGAATIVSEFAPKGRLGRLSSLVLSSTSLGSLLAVFTLSMLASNFTY
VEITNNIWRIPFIIGGVLAVISFYIRNNITETPEFMAARRRQTGTQAGQPGVLTPLKLIL
SENMANIAIGFGLSFFLATLVIMNLYFPVYISKYFNYNLPDIYHAMTISMMASFILILFF
GVISDYVSKFNMMLLTLTLCAASLPFSYELLSRGGEGDLQIFFIIHQIWIASFFTSYIPI
LPRLFTTNVRYTGVALSYNIAFSMASLIPVICAYFFQEQESPFFLVGFIIIAIAMAMAAI
IALIFRGKGEYSY

>gene_588|GeneMark.hmm|727_aa|-|9458|11641 >NVVL01000018.1 Rickettsiales bacterium isolate NORP64 Contig_source1382A_10570, whole genome shotgun sequence
ISCNDATANPSCANIKELEKLSNQGDALAQTYLALCYYDGEGIAQDFAKAAKWHKKAADQ
GNALAQYNLGIQYFNGKGIAKDVTKAVNWFKKAADQGYTDAQYDLGVLYAKGKGVTKDFT
QAVKWYKKAAEQGDDFAQYNLGILYFKGEGVTKDFTQAAKWYKKAADQGCAIAQYNLGVL
YVIGKGVTKDFTQAVKWYKKAADQGYALAQYNLGVQYANGEGVAKDFTKAAKWYRKAADQ
GNADAQCSLGIQYVNGEEATKDFTKAAKWYRKAADQGHVLAQNNLGTLYFNGEGVNKDFT
KAAKCFRKAADQGNALAQYNLGVRYFNGEGVTKDFAKAAKCFRKAADQGNALAQYNLGTL
YFNGEGVTKDFTQAAKWFRKAADQGNIFAQNSLGIRYVNGEGVAKDFTQAIKWFRKAADQ
GYALAQHILGDLYLKGEGVTQDFTQAAKWYRKAAEQGHAAAQYNLGARYDKGEGVTQDFT
KSAKWYRKAAEQGHAFAQYNLGDQYAKGEGITKDFTKAAKWFRKAADQGNIFAQSSLGAR
YLKGEGVAKDVTKAAKWYRKASDQGCALAQNNLGAIYLKGDGVAKDFTKAVKWFRKAAEQ
GYALAQCNLGARYAKGEGVAQNFTQAAKWYRKAAEQGYADAQNNLATQYAKGDGVAKDFT
KAAKWYKKAAKQGDADAQYNLGVLYFRGEGVTQDFTKTIKWFRKAAEQGHAAAQKNLKLK
RPSARAV

>gene_589|GeneMark.hmm|262_aa|+|40|828 >NVVL01000019.1 Rickettsiales bacterium isolate NORP64 Contig_source1382A_11027, whole genome shotgun sequence
MNFSKEETLIVTKTTKTLIVPKGIKKLICIKISLETLILNEGIVYVDCGFNQLTVLTVPN
SVIHLNCSYNHLTALIVPNSVIRFNCSGNQLPKLIVPNGVIRFNCSGNQLTILIVPNGVT
YLDCSNNELTELTVPNSVTRLICSDNKLTILFVSNSVTLLYCSNNQLTQLIVPNSVTDLS
CHGNLLEDLSKYSELIKPKVVSLYAMCHGIVNENNIPKCFSKVDKVTCDGCKNKVVKDLV
YTSYKGYRDGYIRKTIICKKCL

>gene_590|GeneMark.hmm|340_aa|+|856|1878 >NVVL01000019.1 Rickettsiales bacterium isolate NORP64 Contig_source1382A_11027, whole genome shotgun sequence
LYSKCVSYNIVFLFCVHYDQMDNAFSRTKPHRFLMCTDGLIDLNAIGLQNYILVNPKNKI
DMISKYKIAQSCINKMDKNNRFPEVVVKYSSCNDNDIDLDFKYSITQWVIISEGAFYGKC
CCGDKMALSYNMLNFYNGNVLVVCYPCIKKFCTNFHKTSIDLLKSVVRNCISCNKMSPFC
DIKGGMCRNCRGRKQKGLGICATCLSEGVPYQHKRCSSCYNSNNKSFIELQTLEKSIADV
LIDTKSLCTSVCSSCSRSVYKEYTFKPLCKDCYCDPYKETKPCSICFKEMNFIPRWNKLC
NSCLDVRCFICESPYDRGNSNLHMCSKCKLVSDEFEKSYL

>gene_591|GeneMark.hmm|1002_aa|-|1950|4958 >NVVL01000019.1 Rickettsiales bacterium isolate NORP64 Contig_source1382A_11027, whole genome shotgun sequence
MINDYQTYCYFEMPLKTVSGVPIKWSYEMANIFYKKLSIILKNSKHSPVKFEFKFMKTFY
YYQPKATKPIITMWFNSTEAMKHCVNLTRMKKSDGNVYVKGIGMLKCSVWENNVNIVKKM
LIDKKCKHSQWVKVLAAKVVNVDKKISKIEREFVASFSDLKVLPECDTKGLMTHPSILVF
DVECYSDRYNSMPKSRNPLHACFMISVIFQKMGLCDTRERYMLTLGSVGEIKGTNVITFR
FDQEKELLLKFIELISTLKPNIISGHNIFGFDWKYIYGRCKVTGVNAKEWDVISLLKNKK
SKYVKNEWDSSGTGTVNMEYIRSPGFINIDTLYISRREFKLRKYTLESVSQHLLGRGKHD
VSPKQMFIAYEENMLSEKLMEKIKCGSGYHKNIARCVIDEVLSRFKKNSEELTKVAKYCI
EDSELVIDILEKMNCWISMIETSNAVGVSIFSIYTRGQQHRVLSKVLDTSHSMGIVVDSK
ESTIGKWNGGHVAKPITGLHDYVCCHDFKSLYPMIIIYKNICHSTLVLDKSIPDSDCNIC
EWVDSNGKKWKFRYIKQEIYKGVLPTLERSLLSARQNVKDMLKVEKDSIYRIVLDKRQAG
IKVTCNSVFGSLGVRVGGKLSLPEGAMSITAMGRNDIITCNNYLENKGFVVIYNDTDSTF
SKYKNTNDKLTQVEIYRLAVQWGKDMAKELTKLFPAPMSMELEKVGRMFSIGKKMYTFRE
VCMYGTNFGEFKHNDDIMFKGDVIARREASPWKRETYKKVLIMILDRVPFKTIMKSYITN
MYNVFKGDVEWECLLMSRSRRSGIYKSKSAFMKVFSDRLSTIGINVEKGDRLEYLILEGK
GNLGSRAITSDLYKEMTKTGNMIESFSVTCDIDKSYYIEDSENSVMGIIGIAFSDELHKI
TESNNLEDCMLVINEIKKRYNQLSNKIDAMLSTNDPEYVLTVLNKSNAKIVTECRRAKVS
GRYIFNNRLTSKPATKLSKAYKLSEDNLEKTYFNMMCVLDKL

>gene_592|GeneMark.hmm|257_aa|+|5229|6002 >NVVL01000019.1 Rickettsiales bacterium isolate NORP64 Contig_source1382A_11027, whole genome shotgun sequence
MYSVVAKGNCLSKINDIVWAKGAVILNEFTRKSLLCIKCKNTHEFTISIKDVLLGKWCTS
CITCRIQGNQNLMNKRQLVCYKKYSNILISRNGEMISTYNNGKTKVRVRCSSNHVTDLYV
SQIKNKSWCIICKNKRYTGKYKYIRASTTKPKTNILLSKFVNTDVPLVIKCKHGHEFTNT
ANDMISKACPCCSDEFGTMDNEISIVENINRRGGILLSPYVSENVILCLQCEKTHTFKLS
PINIRKGHWCGDCEKGK

>gene_593|GeneMark.hmm|185_aa|+|6361|6918 >NVVL01000019.1 Rickettsiales bacterium isolate NORP64 Contig_source1382A_11027, whole genome shotgun sequence

MTNFQLKYFREFAIMAFERKCELISTYINTNSTIRIKCKCGYTMNIRPKTIRTGKWCRVC
ERRSANRMMNAHPLNNIYFSRDKKDTRGDQVEEMFTLVMGDQVEEIDTNSSEGINDAKGS
IITREYTNKEMFIVENINRRGGILLSPYSNEKSLVSIQCTNRHEFEMYPMNIRRGYWCKV
CEKGK

>gene_594|GeneMark.hmm|175_aa|+|7049|7576 >NVVL01000019.1 Rickettsiales bacterium isolate NORP64 Contig_source1382A_11027, whole genome shotgun sequence
MEFKTEEILIVNKRMETLVVPFGIKKVICVSLGLKVLVLSDACVHVDCSNNNLGFLHIPP
NIVYLDCNNNKIRFLNLRDRKHIEYIDCYDNELDDIKVYSDHLEHLCCCTNNIKKITLSY
SKVLTVNYDGTTLTTTNTYNDNTLKTKVCSIENPHSEKLSNIFISSKNKTKVILS

>gene_595|GeneMark.hmm|116_aa|+|7661|8011 >NVVL01000019.1 Rickettsiales bacterium isolate NORP64 Contig_source1382A_11027, whole genome shotgun sequence
LTASSYDVKIETFTFVNEDKALVRSIRKLGAFCKDVESFHKTYVPLVHGIDSLLITLKYD
YVTENNGTHVNASFVLNIGCIYISCGGVLKVDLESIKQDITAMFEFRYDVVAKQVY

>gene_596|GeneMark.hmm|257_aa|-|8041|8814 >NVVL01000019.1 Rickettsiales bacterium isolate NORP64 Contig_source1382A_11027, whole genome shotgun sequence
MGANQVNVSKSTQELQNDILQVTEENCHAKCTNYDGGGTVIIDGTNIKGDLDIVQQCTAS
ASCTMNQTLQSQVEDIMSSISKQSQKSEQSFINFTISNQADVSEIKQNITNSITQIMQSS
CQATSDNIQKDNIVILRNDTVGGDFTLGQKGSASSNCILNNVAKQVLFNKQQAKQDQSQT
AENVLAIIVVAIVIGIIALGLFALFFFGKKILGSSSEILESPNSGEKGKVGNTKKKSSGL
FSKIGGLLEADPELLLL

>gene_597|GeneMark.hmm|690_aa|+|8866|10938 >NVVL01000019.1 Rickettsiales bacterium isolate NORP64 Contig_source1382A_11027, whole genome shotgun sequence
MSRKVRIVGKVSKVVISRKSFDIISQKSSFEYYCTHDFDYLPLMVGDAVIVKGDYVDSVL
TLDQYPMVVPGENKEAVVGYMRKGFRYKVGVSKIENIVSEVSSKYGSCIDYISNESQSYK
IETTKTIFSYEKTSYSYVNSVRVSDILSSGNEGHDKNMIKWWYNNITLRRLYLLGLNKRT
INEVEDYSLNQMYKILLDNPLRIVELDVPLCIKILKRTENSVTKKQIHQGKIARLIHKYR
KDGWSGVPMSKVKLSCSDIDEHTKALVTDYKIVIDESSDTEIIYFCHQLKLENKVASEFN
KRIKEKTLNIDVKYRMDTLDVDQKKAIRMAINNQISIITGPAGSGKTIVIGEIINNLRRL
DISYEVVSFTGKAVARLQEVTDGKEALTMHRLMARGGPFKHLIIDEASMVSTKLMGTFLS
GCGGNYKITLVGDVNQLLPIGWGSMFSESIKSMCIETTRLVNNYRSDDDGININSTNIIK
DKGIDIKSYENFKLINGDMSDIDKVLKQMEDVEDFDKNNVTVLTPYNKNLENINKKLRTK
FTNNTSDMFEEGDRVMMTQNNYEINVMNGEEGTVTGKYADYITVKFGGTTHKFVLESRKE
SYKDLVTTGSLVMSYGMTIHKSQGSEWKYVICYIGESYANSSFLSKNLLYTCITRAMKAI
WVIGSIGDFRLGCSRDPSYRHDILHQRLSS

>gene_598|GeneMark.hmm|122_aa|+|183|551 >NVVL01000020.1 Rickettsiales bacterium isolate NORP64 Contig_source1382A_11070, whole genome shotgun sequence
MIFVLLYIEYSSHDCARGKQCSHNIIDNDNVGDSDIDKIVEMVKKNTVYTIWRQSVIVGL
ISSLLVPYYTHQSLPSFHEWTGILLLVSLSTYLSSSWIWSHFSSPNSKEIIKRLESLSQI
VG

>gene_599|GeneMark.hmm|500_aa|-|594|2096 >NVVL01000020.1 Rickettsiales bacterium isolate NORP64 Contig_source1382A_11070, whole genome shotgun sequence
MSKYLDPDEINDAGIIFIQRNDPISTLAMSISKEKYSSMGFYYKTNASGRSETKVLYVEV
LKMHRPKLGESGATIEDLVSDDLVTLLSIKKLRPSIVDGVIDEIETRKIQEYFRIAIAKV
TSNESKNTESELVYQLLGYRDKSDVKCLSIMEMVNEVVKLMGRSSDVPLQSSKQKLGIKT
FHNKSKIDGGIEGKFGLISMFGSSFSQSESDTNKPIHSYSKDNELFGELLNIKLPDRIPI
DVKLAKIREIKIYQPILIKMSTIFIDMLLNNQRFFDTLSKGIVQDNVSIEIVRGFSDDFT
KLFTLISQSVKSGSLEYSKLLECSSIHNDRKNILGKGCESLIQTTELPELVSNILINVNV
KCGNNSRRKLVSSLSNLYDHVSYIKNSVDCDSECLVDINLLSSIVNNLMEVSSLRRPRIC
NISGDFERECKINVDNRCGTIYPMTMSSGKKIVLNTRYPNLLCYSKKELSEILRSLDNVS
VEDEDMDSIRAMLTDILASS

>gene_600|GeneMark.hmm|171_aa|+|2259|2774 >NVVL01000020.1 Rickettsiales bacterium isolate NORP64 Contig_source1382A_11070, whole genome shotgun sequence
MQVPHVDRFKKNVHDMVDLIGDIIELSEQGNKRNNKITLNVAGLFIKSYDKEKLIDHFIL
ESYKHWETIHKRDETFFLKNAISVFGKLPEDNVNTFKKLFEADGDISDEDKGAIWDFFIS
LVKICIKYIHSVRLPKTVLVGGEERNVYSNKKYSSVNLFSFSTLYDIKLVW

>gene_601|GeneMark.hmm|792_aa|+|2768|5146 >NVVL01000020.1 Rickettsiales bacterium isolate NORP64 Contig_source1382A_11070, whole genome shotgun sequence
VVGNYEWYIFIWDYQMTTVTKNDRKLRTVISAVENGISINGTTIDIGTPGITKIHKALIS
QQNGVHSDTLVQYYSRDDFSPLDIASCYSVENSVAILNHLVSDNVNEILSRTNIYPSLNE
LEDIMSGNTYITMDVTSFMKDNKYVGINKLKVVTSGDPQRNWDTIFGTAYGSNIFVLHTH
IYDNVLIGMIAKSMNKFVSIVVVVPAKHLEKYKKGATGSLPSTTSRPRVVKTKPKPKRVV
KTKPKPKRVVKPKRVVKTKPKPKRVVKPKRVVKTKPKPKRVVKTKPKPKRVVKPKKVVKT
EPKHTRAVEVSANIETHAINVRKMYEESLGAGGDGKFTELYKYYTLNIEDIFKPENGAIR
AFGVDSKPLVNSSDILDTLHGNLNVIVLIFLTTIGMVLKKDKTTIENGKDKHKKMRLDLY
FGDDKKQTTKRMINLLHIGMKTQNSMVEAHKPGAWEDFVKINESFRVLLRNLMGKLTLKY
ESDDDEYESNDEYESDGKALKLKEYASVIIGLFKGNKASKYSQIYEYFEANIDLMFLGDN
FGEGDLSGVLDALGSNINNITIIFLATIGMVFTKNKATIEDGRDRNKIKRMEKYISSSSA
KKTPEIIYSIYEGISNMNNKLAEEFGKYNPNKSWTVLPSVVVLLKQLHGKVTVKYLIGDF
EEKLFLHVEKLNDAQIVEDNCAEDAGDMFEYIDANIKSIANGSRVISVANRYSDKEIQNK
IFKVLKSVETISEYFFKSIRGGLDDEADLRVFSEFMRLHKELIGSFPNSLRNTNKFQLFV
EFAENFTKKFQT

>gene_602|GeneMark.hmm|123_aa|+|5165|5536 >NVVL01000020.1 Rickettsiales bacterium isolate NORP64 Contig_source1382A_11070, whole genome shotgun sequence
MSKSPNPLKIISKDLTDVMGLGNYITFDGMAILTFDMLVQWWTIYVSSNELMDDKGNIFP
NSIVSYVMDFRHPRNSFNIIKLYDRLNNLTESFDGTLTDSQIKDIKDYEKYLEELYESYT
PKL

>gene_603|GeneMark.hmm|452_aa|-|5518|6876 >NVVL01000020.1 Rickettsiales bacterium isolate NORP64 Contig_source1382A_11070, whole genome shotgun sequence
MIRDKISEILGVGNDVWMIYDYIQEDGLYMLGMNKTSDVNMYGFVEGLVVDINYMKIICS
SDGYKEIFMLNEITPDAIGNIDLVSVFGTKKTVNVKGLQVEMYPDGFDIRVFKYRGIVYH
ISNTKLSLNYSECYNKNSYDDLYNYFEIPDSDILEDRCYKFRIVSKDTLHVSKERINEPY
MVYCGVLGEQFPKLNTSKYDRVFDTESLSVEDTNDYLFSGYYEIDDSLDVRLLPGERVQI

KYTNKYYILESESYAWRKYVRGYEKDLYERMLQLLDVTHADTTINNPITNYPKMSTWDVK
GIMDEVSNKNIITWPVGDSYIPKSSEYKFYNVWAAFLMSVPLHSQANVSKFYNKIINDMS
NVIKWIKELSVGKIPDMSENSSRYQTDCRNLDFNVKNILSSSKDKALDIKDMYVVESFQD
RINKFIELEVLLSGSALSSLIKDMNCNYSLGV

>gene_604|GeneMark.hmm|366_aa|+|6989|8089 >NVVL01000020.1 Rickettsiales bacterium isolate NORP64 Contig_source1382A_11070, whole genome shotgun sequence
MDQNNSIRLWNIEFDSKCLVTKYGMLNGKIQSASVEIYSKCNRTLDEQALLEASNKYNKK
IRNGYGVDVNACINSCNTLQLANKYIIPGSLTAKGNAIASNVKCFPVLLQPKIDGIRCKA
FMKGGNLMLMSRNNKKHSFLDHIKEDVRRIISLVGNRTLDGELYNGDMSFVNIQSSIMCS
KTKSPSNEKIQYWIFDVVDYTLDVKSRLKLLDKAKVVKSKHIVFVPSIEAVSHEDISKKH
RGYVIDGYEGTMIRHIAKEGSKYHKMSLYRGKRNNNLLKHKDFLDEEGEVISISEGRGKF
KGAAIIRLIDPRGNELEVTPKGTFEHKTELYTNRNKYIGKLYKYKYQGLSEYGIPRFPIG
LYFIEK

>gene_605|GeneMark.hmm|92_aa|+|8108|8386 >NVVL01000020.1 Rickettsiales bacterium isolate NORP64 Contig_source1382A_11070, whole genome shotgun sequence
MERQNLSSLSRGQNATGMYGGNTNMFVLWFVLLTAVVWFLLYTIKPSFIQKKDSSGETTG
ESNNGKAFLWSLGISAVLLFILWAIKQSSMSY

>gene_606|GeneMark.hmm|179_aa|+|8425|8964 >NVVL01000020.1 Rickettsiales bacterium isolate NORP64 Contig_source1382A_11070, whole genome shotgun sequence
MSYTLPIITSIDDRPLHHTVRKSDTLYDNNTTKLIQCVYLFSTVLWILLVKSLGIYNGEY
MGLLFLSIPVIVYMINYVGCKEITKDVEQHMFKGNFLSFGYLIVVIFMNWNSSIERTKFF
KTLVISIILLMLSLVDLWVCESLLILSSSLKSIFQTASLGLLSYSLYMYYLDNRNKTMA

>gene_607|GeneMark.hmm|356_aa|+|9178|10248 >NVVL01000020.1 Rickettsiales bacterium isolate NORP64 Contig_source1382A_11070, whole genome shotgun sequence
MNFSKEETLVVTEATKTLIVPKGIKKLVCSGIGLETLILNEGIVIVDCQNNKLTKLTVPN
SVTRLYCFNNKLTELTVPDSVTDLYCSDNKLTELTVPGVTRLDCSYNHLTKLIVHNGVTD
LDCSGNRLTILIVPNSVTYLNCSDNKLTELIVPNSVTRLYCSYNELSKLTVPNGVILLHC
SCNHLTKLIVHNGVTDLYCYGNKLAKLTVPNGVIYLNCHSNVLTVLIVPNSVTHLYCFNN
KLTELTVPDSVIRLHCSSNLLTELIVPNSVTCLNCYGNLLEDLREYSELIKPRVVSLYTM
CYGIVNENNVPKFFSKVDKVTCDECGDKVIKDLAYILCKGNVEGYIRKTIICKKCF

>gene_608|GeneMark.hmm|205_aa|+|10371|10988 >NVVL01000020.1 Rickettsiales bacterium isolate NORP64 Contig_source1382A_11070, whole genome shotgun sequence
MNFSKEETLIVTKATKTLIVPKGIKKLFCVFVGLETLILNEGIVFVECCHNKLTTLIIPN
SVTRLDCYDNKLTVLIIPNSVTHLRCSCNKLTILIVPNDVTYLNCCYNELIELIVPNSVT
DLDCSGNLLEDLREYSELIKPRVVSLYAMCYGIVNEKDIPKFFSKVDKVTCDGCKNKVIK
DLAYILCKGNVEGYIRKTIKCKKCF

>gene_609|GeneMark.hmm|978_aa|+|1|2937 >NVVL01000021.1 Rickettsiales bacterium isolate NORP64 Contig_source1382A_13643, whole genome shotgun sequence
NFSFEVLRKPACSHKSASVEDLVTSMIMIPGSGEYVVDTKIQQKTILTPHGAEIETTDVN
SHNYHKIANSVHSLNQLQQICKNTEWVSPVVCWFGDNINARDCLIKPAVEFKDTKVAYSE

EWRVGGYNRETAYEITKDAFDRPLYGGSVNDASVVRYLKELKSRNLKTMFYPMFFLDVPQ
KPWRGHMTTEPEYVRDFFQKEHGYNDFILHYAELARDHVDAFVIGSELIGLTSIRSGDNF
PAVDELVALAQKVKQIMGDKVLVTYAADWSEYHHTTGGWFNLDPLWASSGIDFVGIDAYF
PVAPAAGSVITKEELEAGWNSGEGYDYYIDQSDDSKHPLAPEYAWKNLRYWWENPHKNPD
GALTEWVPRSKPIWFTEFGFPSINQATNQPNVFFDPRCIDGGVPKGSNGNIDFTIQRRAI
KAFIEYWKTQEYIGQMFLWTWDARPYPAWPHMRVWRDGNLWEKGHWVNNKFGTSNLGAIL
LEISLRSQINLDHVDVSTLDDTIEGFVLSNQMTAINAIDMLRASYFFDICGANQEMISFI
KRGSARELSVSSSECLKLSDNSFIEEIEIPKEVTLDKVDLYFIDGSKEYSTNYIYVNNET
NSYTDKATLRTPLVITEAEAKIMGELLLENASIEDKIISFILNKQDFKLKPTDFVSFKHA
GREYSIRVINTEIHGNQMIVTGIVDYRDFYLSVASAKNQLTLEYEHSEDSNLVILDLPFI
FNNARAPYLAAYLCNNASAPLYSKLPHDLHGNWSRIASLEPTNALGTLVEFIQPRHVNMF
MIDETSKLIVKGRRLEKYALGAQQLAMIGGEVITFRHIEKLQDGLYKISYLTRGGMGTEN
LMTNHAPGEDFVIINAGMGMNMISVSKKLIGKPVIFRACSIEKSMIYENKAQSPLPPFVT
YEQISGHELHIKWVTRSRHYNQWDEPAGAEDSSFTVKLHLNANGDAAEYQSLTWEIIIAI
SALDLSAGYSVDIIKGGN

>gene_610|GeneMark.hmm|245_aa|+|2960|3697 >NVVL01000021.1 Rickettsiales bacterium isolate NORP64 Contig_source1382A_13643, whole genome shotgun sequence
MQTNNFDMELMVPGQENKDVTFNESLLKIDSFLNLSVNGFRDEVPDEVPLGEKYIITNGE
YQHHICYSSHESKGVQYFRPKQGMVVFSVQAHSFMLYHEHNWVEIMQAAHEPPREDGAMV
GGAVDAAVVADDADAVNVGGAYVDDNFTGIDDEFIAPPNKKHFSLYLNSNTTLNFDNVLF
SEVTILIKQCYNAARSLTWPHNILWPDGSAHIMSEAPNAIDIVKLYRMPESTHFLGKIIG
QNYQF

>gene_611|GeneMark.hmm|148_aa|+|3792|4238 >NVVL01000021.1 Rickettsiales bacterium isolate NORP64 Contig_source1382A_13643, whole genome shotgun sequence
MVTLLASIAGFLTSIIPEIIKYFKDMNDKEHELSILEIQMQYNRDNDARSLEEIYVARDI
LEQASLYATFESKIWWVDMLNGSVRPVLAYSFFIMYVAVKYLQYNAISASAHIVEHIEII
WNIDDQAIFASVVSFYYGQRTFRRSYRT

>gene_612|GeneMark.hmm|154_aa|+|4201|4665 >NVVL01000021.1 Rickettsiales bacterium isolate NORP64 Contig_source1382A_13643, whole genome shotgun sequence
MGNEHSGGLIVHRCITKEGIELIKIFEGFSAKPYICSGGHLTIGYGHKLLPDEPTEEISQ
AEGILLLEQDLFRFERAVVRYIDNPLNNNQFAALVSFSFNLGAAALQRSTLRQKINYGLY
NEAANEFPKWVYADGRKIAGLVHRRIVEQELFLL

>gene_613|GeneMark.hmm|194_aa|-|4690|5274 >NVVL01000021.1 Rickettsiales bacterium isolate NORP64 Contig_source1382A_13643, whole genome shotgun sequence
MPIGRTNSRIASDFILSGIVVKKELSDNITQTLQGGLALKSYAAIWENFNHEQVGNIVEA
FVNHGGKEVWNLMPALLTPNSTQEQIGVIFSPKYQSLEFIRICAVVATILPPSPLKSYAL
QQAQIILARDHSAFEEFEALSNQVALLGASKEMAHVIFQFVVGDVGIRDEVHELIDLELE
QLQPAGVVIEEVAE

>gene_614|GeneMark.hmm|59_aa|-|5772|5951 >NVVL01000021.1 Rickettsiales bacterium isolate NORP64 Contig_source1382A_13643, whole genome shotgun sequence
LLLEDYSSFFELHNHLPVNQGALASKTVSLKKAEQEVAIILSSSNPEETIQNRVAYKAS

>gene_615|GeneMark.hmm|94_aa|-|6080|6364 >NVVL01000021.1 Rickettsiales bacterium isolate NORP64 Contig_source1382A_13643, whole genome shotgun sequence
MGNNSQLRDTASSNAESVVKPGTVTTELDRASLRKSVGDDLPGDSKNEDNEPPLLKNLSP
FEQAIYNLNQRENTELGDLLEVSGQMTIEGTSGE

>gene_616|GeneMark.hmm|689_aa|-|6846|8915 >NVVL01000021.1 Rickettsiales bacterium isolate NORP64 Contig_source1382A_13643, whole genome shotgun sequence
LRKFKQASIVGTSGIGKTQLIRTFAYENRSNYKVIWFVDCNLNLNQEFIKLAKELNKNNG
ANIKEGGNLVKEKVLEYLQKKDNWLLIFDNLKIGENKKVQDFINWEHNGHVIFASQDKEN
LPYSIELTKFNRNDSIILANNILESSSSEQEEFLAKSFNGYPILIVQAAQLLNQVPGLNF
KTYKKQVLRSSNKLKLNIALAISEFTPTAVNLLYKMALINNQAFSKDFLSYITDNKTSLE
DDIFQISKFALISNISSSEQNSIFEMHDIIAQTIIDMNGRKKSQDVLEKLISNLLKSVPR
SVTDFHVFRSGKTIFENFKVISRYTEEYPISLFGTMGLSLYLMTQYNNYSDYEAAEELVK
WFNEREDINKFNPSLMNNDERARYADFLQSLARHYRNRHFDFDKSMKYAMKAKEVYKNVE
GYESIKADLFYQLASNEIKVGNMKSAEQYIVKLKNTPFINNVESMLFFLKGDYESALNRM
SLLIKIRLGKIKADDLVLTSNYLLRAKILNFLGRYQEAYDQAKQIYNMHKFKKKEDHLIF
GRIFAELARSELGLGKIADALEHIKKSISILKNMQQEKAQKARFLESAYLARSYVVQGEI
FMKQNCFKDAIEFYRAAQKIYFYLYKNNMAHIEHVSYLHLQGAKAACRAGDLYHYKTFGE
PQVRGFGQEHPNTIAMFKYCGNYNMNLWQ

>gene_617|GeneMark.hmm|243_aa|-|747|1478 >NVVL01000022.1 Rickettsiales bacterium isolate NORP64 Contig_source1382A_13955, whole genome shotgun sequence
MNFSKEETLxVTEETKTLIVPKGIKKLVCSDIGLETLILNEEVVYVDCPYNKLTELIVPN
GVTFLNCSHNQLSLLIVPNSVTHLDCSNNKLTILIVPNNTTHLRCYDNKLTTLIIHKHMT
RLNCSHNKLTELIVPNSVTYLGCSGNRLTELIVPTSVVRLFCFYNLLEDLSKYNELIKPK
VVSLYDMCHAMVNENNIPKCFSKVDKVTCDGCKSKVIKDSAYILYEGYHEGYIRKTIKCK
KCF

>gene_618|GeneMark.hmm|391_aa|-|1605|2780 >NVVL01000022.1 Rickettsiales bacterium isolate NORP64 Contig_source1382A_13955, whole genome shotgun sequence
MRRGMQDTGIYIVRCYTCMKVLSPYYDTYSDLIGDGVSPRTAIRLIKLGNIINVGVESEI
TGEYITKLFEANDLDITRYNRLIKGTTPEEALELMKLSHILDIDHGSSSNEYIAGLFRFN
NVFGRYKEYIRISNGLNEFDVLDLMGLSDKFKESRFTVNNMVNFFSSIDSMKRYEDYLKI
LAGLNASEIMKLMGLGYVLDSNYKHKNTNSYVKRLLVSSGMPGKYKQYIEIVNGTKVHKL
LKLLGLEYILDTSPGCIITSDYVTKLFKENKVEYAFDSYIECISGEMNTRKALDILGVKR
ICCRMRVMSQAKIPLGRALNRDDGRVYETKPLEISRAFSNSSRMTNKSKLLEKFSKINVP
LFSDDPPVRNANMAGKIRTPREGTLREFKTN

>gene_619|GeneMark.hmm|227_aa|+|2852|3535 >NVVL01000022.1 Rickettsiales bacterium isolate NORP64 Contig_source1382A_13955, whole genome shotgun sequence
MVSVEYVMIVIVALIVLVLVGLLIYHFAFRKHGNIEGEPCSKDPCAAKHYCGGDTKCHSG
ISGGGSGMECSISAHCSSGLTCVDDKCGTGSSQDNHSGSFTNSSITAIIDGEKRYLTLSP
ATNDDDEAVSIWEKDEPNHKYSYSSSTNELSSSDSKLMVNSFGFIKEGPAAKFYFISSGG
EMRMEDAFANILSLDTRDNTAVFKDPPRYTSQSTGPTVKGIVIKVDK

>gene_620|GeneMark.hmm|367_aa|+|3573|4676 >NVVL01000022.1 Rickettsiales bacterium isolate NORP64 Contig_source1382A_13955, whole genome shotgun sequence
MENDDVIDYIYDLPLEMSCKIVEKITEIADVIKILEDERMGPLMMEYGRTLRQLGGFKMY
NINLIQGMRMLSYVDENILISVKTVLEANMLSTLAPGVSVTIVILSDNFTESLEIAKAVV
DNYLTKSITKGSVHRNMRIAFRNIDFDISDDSGGDFILINKNKMLIGTSLYRDVALVALS
IIKNLDIQMVSVDHENIKGLSLIVGNEIYLNTDMIYDFSRSDLGCLDQSNSPKLKDTILG
FNGTYSEFPDRLLTIYMYNNGLFLHREETDEFLLTSDTLIHRMIERDEYINGNKNKGKID
TTLNIWSSSKNYESLMDIFAKTIFVNSKDIMIKELTLIKIPLEFLNAHEMTDDSIHIEQS
YLEIFEG

>gene_621|GeneMark.hmm|632_aa|+|4711|6609 >NVVL01000022.1 Rickettsiales bacterium isolate NORP64 Contig_source1382A_13955, whole genome shotgun sequence
VIFKENSVSKMEDINIFDGRCKTKHHLLRILELVKTKDLVSIMNPIGSGKDIYIPLILTK
IGKVLVSFNTSRELSLNCKYLSKYTDVGRLSDESTYNEKTSVICGTSSEVRDLVSSNSSI
YFSREIIIIGGLDTSLVNNIITVAIWRKKIKSLSSTSPRMVLSNTSPSNMLYKSAADYNR
HTLVDVSSYTVSVIENFDITYDDSYGPGSYMLLTKIAYILSNVYENVTNKSTLVFLTDLP
RINRLVDLISEVVNASIVTNPVDEPGDESEHNGNVVYIVDDMSSSSPYIGNVDFVIDSMS
RTRGREVVYIDKRTAKMRASRLNTTKSPVCHRMCTKRVFDLLNGWDDSKMSSDDMYDLIV
GYKENGIDLSKINNQNGKSITEANNLSFILGSLTSRDEITEKGKFIRESHMSRRCSSMLW
ECMKKEYSKYPIYACVCLISLIDSYEHGYFKYPKRLDGERTVDYFNAVTTFKRRHHAKFF
GYSDVGTLLIMWLDLLNNSIDRESVKIWALENKLNMENIINVYDSTNKIMESIGGEHIKY
FEIKDVIDRMRPIIINAYTDRCLFLENGNNTKYSDNYGNNYFYDKLFNYNTMIVNPPKQL
VCLYFDDTKIGPGRIMKLSLNADDYVIKYFEH

>gene_622|GeneMark.hmm|300_aa|-|6639|7541 >NVVL01000022.1 Rickettsiales bacterium isolate NORP64 Contig_source1382A_13955, whole genome shotgun sequence
MNFSKEETLIVTEATKTLIVPKGIKKLVCTFVGLETLILNEGIEYVDCSSNQLTELTVPD
SVIRLYCSSNELTKLIVPSSVTRLKCFNNKLTELIVPNNVIHLYCSRNKLTKLIVPNGVT
CLYCSHNKLTELIVPNNVTCLYCPYNRLTKLIVSDNVTCLYCPYNRLTELTAPDSVIRLN
CSYNQLTELTVPNSAIRLYCSYNLLTKLTVPNGVTCLTCHDNLLEDLSKYNELIKPKVVS
LYDMCYGMVNENNIPKCFSKVDKVTCDECNDKVIKDLAYTRYKGNVEGYIRKTIKCKKCF

>gene_623|GeneMark.hmm|319_aa|-|7643|8602 >NVVL01000022.1 Rickettsiales bacterium isolate NORP64 Contig_source1382A_13955, whole genome shotgun sequence
MNFSKEEILIVVGTTKTLIVPKGIKKLICSKIGLETLILNEGIVYVDCSRNKLTILIVPD
SVIDLNCSDNKLTVLIVPDGVTYLYCLSNKLTELIVPDGVTCLYCSNNKLAKLIVPDGVT
RLYCSSNELTILIVPNGVTHLDCSNNKLTKLIVPKSVTFLYCYGNQLTELTVPDGVTFLN
CCRNHLTILIVPDNVTNLDCSFNKITELIVTDGVNYLVCSFNKLTELIVTNGVDYLVCYD
NLLEDLSKYSELTKPKVVSLYAMCYEIVNENNIPKCFSKVDKVTCDECRDYVIKDSVYIS
YKGHHEGYIRKTIKCKECF

>gene_624|GeneMark.hmm|28_aa|+|1|87 >NVVL01000023.1 Rickettsiales bacterium isolate NORP64 Contig_source1382A_14627, whole genome shotgun sequence
LSQFGTMLLKGFLLPEIQDYRLFIYLLR

>gene_625|GeneMark.hmm|65_aa|-|84|281 >NVVL01000023.1 Rickettsiales bacterium isolate NORP64 Contig_source1382A_14627, whole genome shotgun sequence
MRIVDDAAEPCTQAQMEDELKSAPVASLSIHQPTTAVTFSPEQVLQGMAARLANANITLG
TASSS

>gene_626|GeneMark.hmm|395_aa|+|791|1978 >NVVL01000023.1 Rickettsiales bacterium isolate NORP64 Contig_source1382A_14627, whole genome shotgun sequence
MAIFSPHTSIVQMSRREIARDYLKKGRVVYSKIHVPAGILEDSGKGLDAGIGRKPGVYDG
SARYMKSAELRALNEELAKENARAIWKNPELVFEYLPLDNPVFTKEEIGAALLEALHKDL
VIGEVADEKLIVGLEKNLARRCMVLEARIFSSDTLSLVTKCDLRGRALYTQTKRVELEER
YTKTIKELHGRSNHSLYLQDVDLDHLSIREWLAKHARSGILRFTQKLEQKIGLKLNVASS
KQRVLSPEQRKAVVGILNGKDLSLLQGLPGSGKTMLAGVIARQYKKAGKKVIGITPSDAA
ALALAKETGIECRNAILWRKIWLDEKWRGRYGEFQPGLRMDYYKEDKFQSKISNLTKNHV
VIMDESSRMDLINMDFILSEVRKAGAKIIMLGDNN

>gene_627|GeneMark.hmm|311_aa|+|1994|2929 >NVVL01000023.1 Rickettsiales bacterium isolate NORP64 Contig_source1382A_14627, whole genome shotgun sequence
MAGGFKKSIDICGSETLTEVRRQNNPADREAVKLLSQYRTHDALTMFEETGSIIIEETEL
AADSRLVDDYVEAYTKKAEELDRSNHRVADSLVVCSHTNASVVKFNSEIRERLKQNGILK
YSKVGIVGLFRRKGGVVNVGNKRMELLPGEQIVFTGNLNYEESSDVFNSEVGTVLSVNWV
DKQGFGYIAVLVNKADGSLEQVVLDTKKLSEYPGSILDYGYAVTAYKLQGATIDQTFIRH
GSNIGYETFNVMMSRHRENVWLYADRKTLHDALYATLDKNIVKARYRYNLEAKPGQNLDM
LGLKSHIINSL

>gene_628|GeneMark.hmm|1779_aa|-|2986|8325 >NVVL01000023.1 Rickettsiales bacterium isolate NORP64 Contig_source1382A_14627, whole genome shotgun sequence
FNEQDESVKTLLNGGDTDISGLEGYSGYEKICELRDGLLNSSDQLNQESVEPYEIITAAQ
TGEDKLLSAFVSKELQINYDSDDSDTPEDEDERYDALMCDALWRAAFNEQDESVKTLLNG
GDTDISGLEGYSGYKKILQLKEAMSSQRQQVLEQLRIDLFTAARSGRNLELLSELVQGKL
GFSESELQKVIEDTIEIAAFNSQQGALIRLLEGGNIDVSSFREHMIWDAIQQALDKVDEE
QSVAGNEIHTTLRQDAVTQPTPTGLDISTMNLSNLVIPTLDEFKQHPHPEGVDPALLQQY
GHEAKIPEELLAASSPASSAVPSSAVDFATTPAQISSDPAEEKQKTLALIRDYYQRGLEL
YHSGDSANLALAAQELAQALRPTLSKPSMRSLHVNIADVKEELLDSMYHLGKIYLADSSY
PDHHSKAAAIFQYCAGFARKHFTEEYFAQGQGGVLTIATSADDRKEFPSAEAAAEYFTNQ
AYLVENQFLVTLHSNMPAVTLINAEDTKAADLYCSSYHQQISGYKDSLAGIRFSTQDKLE
EISDLTVVDIAQRAAEVAKIYSDVTRFFVRHERDVLHQDAQHQAAPQQAAPPAFVQQLLI
ECYEQLGAPPEGCEYAIVSLGSLASGTMTPWSGLEFAILINEDNESYREYFRNLTKLLHI
KVINLGETPLRNVEIESLNNFKTANEADEWFQDEVMISGFNFDGSDWHARKLPFSQQGQQ
AENELDIEITIISDKPDYELILTPGQLAMLQREVGHSLSLTEPEHDLADSTPPDSDTEST
PGSESTESWVSSDERLVQALRSISLIDGSTRVDYETGQEFRTGQGLLDSYRAHLRDPEIL
APQILQERAMHILQEDVSKFSLKLSDEKKGNLVDVKKDFHRLGEVIQALSNYYNILAKDD
SPAITIWEMIDVMEIRDNPILSPVGAQHLREALGIATELELSSYPNNEAQGEWMSTYVPT
VENLSKEQRQQLVQQTFHIKDTSILHHFYYVMSKVRTIMDGFSKEESREVAERTLVTNDL
FDASNYNKGMVHARFIEYDQALGYLKIAKTEAPESFELLKNLFVLHQKMKATEDALAVAE
KMLRLSQAQHPDNPNHPDLAASYNSLGNAYDLKGNYDKAIEYHDLALTIRLQAYANSLSH
PEIAASYNNLGNAYNAKGEYGQAIIYHKQALAIRQEAYVSNPNHPDLATSFNNLGNAYER
KGEYDQAILYHEQALGVMQKAYTSTPHHPDLATSFNNLGNAYEKKGEYDQAIKYHDLALT

IRLQAYANSPSHPDIAMSYNNLGNVYDSKGEYGQAIIYHKLALAIKLEAYASTPNHPDIA
ISHNNLGNAYYSKGEYDQAYEHAGKAVEIYFRNHHQQHLAIAKSVYDQIVLQLGNLALLD
DSAAAAIEYYQQIDPNYEHIVFESEEFVRLQFEHAAMAYSSNALQSALNCQLVLVKIDPG
LRHGNHYHNLASYYAYLGHIEKANITFTDALAQPPAKVTGRLHVEYAQFLIVNRDNEALA
TSVQEISPHLHAAISSDNMSDLPYGRAEKDSVCEILQNLLSQRNAAIQVNPKVLAYYLLI
KYPECMQETNTPDPLLKALRSHCDNLQDEISFTLLADAYRSVGNEELAGQYTEQAALIVI
VDDLITGKIKLTTDGGDIEPAMVQEFARGLGVTGSSAVSEGRLDDAAKSYAKLFKIYHEI
LTGDLKGFQAGANSLATIALLCNHKDLFLHLKQKFGAEINRELVELSGLDLLPLEQQIPT
QLGIPHQAMQSDETLIQSPAEEEASSALGGANSSADDEG

>gene_629|GeneMark.hmm|29_aa|+|2|91 >NVVL01000024.1 Rickettsiales bacterium isolate NORP64 Contig_source1382A_15885, whole genome shotgun sequence
IVLRTISILNLNNFKSILVDFKSILNSFK

>gene_630|GeneMark.hmm|59_aa|+|437|616 >NVVL01000024.1 Rickettsiales bacterium isolate NORP64 Contig_source1382A_15885, whole genome shotgun sequence
MNQDFSVSIFYGDDQGRVAFLTGPTPTTKKPNVARKIVRRIDTDAGGSRVAQFYAKMNL

>gene_631|GeneMark.hmm|170_aa|-|835|1347 >NVVL01000024.1 Rickettsiales bacterium isolate NORP64 Contig_source1382A_15885, whole genome shotgun sequence
VHWDERGLQRMHCVRLIPRQVHWEERGLQRMHWVRLIPRQVHWDERGLIVRRVHWVRLIP
RQVHWFANNSTAGTDTHTGAMLAATTRARRSILVTQTTRQGIDQARSLSTASGKNTENIA
STTKFQIQGVDVGSLSSLFGLDMDESIPGTRKVVGGHVDSVETKPTMSKI

>gene_632|GeneMark.hmm|503_aa|-|2103|3614 >NVVL01000024.1 Rickettsiales bacterium isolate NORP64 Contig_source1382A_15885, whole genome shotgun sequence
MKKCYQLEKKVLKPFWNTQCLEISKLLWYPENTDLQDLQDLQDLEHISSSKLSSVSSNQM
MENSSFWITKMKNQKMKNWQTTYSLSSTSLTVDKWEKEDIELKSKMIRIYPTKKQTKILD
DWRHTNRYVYNKTIAYTRNNPSEKINFQSFRNSLVTNTIKDVDDKRSQNPNVKLWEMKTP
KEIRAYAVKDVVTAYKTAWSNLKNRNISHFKIGFRKKKSPTSSMTIQKQSIDFKNNYFYI
YNTFFSGSGISIGKRTLKKIKNDFKDGFKVEHDSKIVYKHGKYYLLIPIDISKKDTNKKI
KKNGVIALDPGTRTFMTGYSTSEVIECSRKADIFKKLKNKISILQENRKYKTIQKYYIRI
GNLVDDLHWRTIKYLTDNYDEILLPSFESQELVKKNKNKKVREQLLNLKHYQFKRRLICK
SNMLGNVKVYIVGEEYTSKTCGRCGALNNNLGSKEIFKCVKDDCNFKTGRDVNGARNILL
KHLDVCPRSTQVGSTQDKFASKS

>gene_633|GeneMark.hmm|143_aa|+|3779|4210 >NVVL01000024.1 Rickettsiales bacterium isolate NORP64 Contig_source1382A_15885, whole genome shotgun sequence
MSNTVEMSSKNDEMSNEIVALSNGNCDLWNRINDLSSKNDEMSNEIVALSNGNCDSWNRI
NDLSSKNDELSSKVNDLSSKVNDLSSKVNDLSSKVNDLSNDNSVLCDKNAALMRKISSLD
ESVNSLKRSSNSDGERVSKKIRI

>gene_634|GeneMark.hmm|117_aa|+|4362|4715 >NVVL01000024.1 Rickettsiales bacterium isolate NORP64 Contig_source1382A_15885, whole genome shotgun sequence
MVKTIVSLAVKCATSKICELDAKCRIKTRINKKNIETSLIKYVEKNSETFAGPIDDFAWS
RDEIAYEIAKNNFPFHALFSDECGEFGPFEDTDEVEDFIGEIIEKQFFKGQCYIDSD

>gene_635|GeneMark.hmm|135_aa|+|4758|5165 >NVVL01000024.1 Rickettsiales bacterium isolate NORP64 Contig_source1382A_15885, whole genome shotgun sequence
MNFTFFKKKEPLFIKLTMDKFESNFSSTKEEKIDGIVTNTIYEFFISTCEIPKWKQCQQQ
KLYVKNTDLYPQILVFYDDKIKQCSVPYWESSTILTYQKNVKTIKVVLVFQSHYTKDVVA
CETDYRPEESSSDFV

>gene_636|GeneMark.hmm|435_aa|+|5393|6700 >NVVL01000024.1 Rickettsiales bacterium isolate NORP64 Contig_source1382A_15885, whole genome shotgun sequence
MAVSQMTIYNIANAAVAEFQTMVGNIIPEGLKHKCMKFPDCNIDVYAPTLFCNDHAATMY
LGQTTCNIPGCKNKPLVGFPVCEESRHMRFLTVRLMAKQVKIIREYLKTAEENEDTCAMT
QEQAAVIESLKNELTKKDEHIDALTLEKSGAEANLLFQNAETIKQVKSLEDDLSNAERRV
FLRHLKYTDDIGSIVDEHKKDIAKKNVEIENFKNMAKNNNTEEIDDLKESLEYFKMRESE
LSIKCTHLATEKKKMTGAYNDATGVNQAIYGRIRDFQTEDNLKTATINAQNEKISTLKRK
FAQDFSNLERKFAQDFSNLEKVQEMALNKMASNDVELHRENDKIKTVNDAMETDLNCFKA
ECETLRSTTDAISEERDALFEECENYRIIMASMKTVFQGFARPATIERPKKRTRVIVEDD
SDDEIVSELVIDETV

>gene_637|GeneMark.hmm|55_aa|-|7420|7587 >NVVL01000024.1 Rickettsiales bacterium isolate NORP64 Contig_source1382A_15885, whole genome shotgun sequence
LQIARFQDIWELPIYNGFYFPKQLIFAACTTYSTADNENRSDPTYSDFDQNHNDS

>gene_638|GeneMark.hmm|621_aa|+|2|1867 >NVVL01000025.1 Rickettsiales bacterium isolate NORP64 Contig_source1382A_16436, whole genome shotgun sequence
LLISRLFAFLLGGGAIMFSLVETDLLSIIVLANSFYYPIVTPPFLLTVFGFRSSSKSVLI
GMIAGLATSLIWKMLPIEFGSFSQKMIGVLFAMLMNTVFLLASHYVLKQKGGWVGIKDSS
YLDEQKIIRREKWAALVSKARNFNPINYIKALPVADDSRYVTLGAYFLVFTFTSIYSTHA
EMLKANKAIITTIYKTMIISSTMMGMYLIWPLSIKTEIKQMVMRVWYPLAIFYMLIFFSI
FFVLLNKDNNLHFVLFAVNMIVSIVLLGWQMASVMIVVGMYMAVQFYKYYAGIEYLDISL
GSPQFILLYSLVFIGAALVIFFRPQQERQKQTEHKVGELLDEVGTLVTVVSSRDSQISTL
DTKVSGLEERVDHYTERTTNQAQEIERLGSTAQRILNNVNHELRLPVGNVMNFAEILHDG
LGKLSDEQLKMLSKEVYKNSNRLSTMILNMLDLATLQAKKIELQIKLANFSELVEERLAA
CRKIYVEEKPLKFIIKIEKNVMVxMDENYIRQTIDNLVINAINFSEKGKILVxVAKQTNF
VTFTIKDQGIGIPLSELYDVFTPFKMGSNTETKAEGRGVGLALCKAAVNAHGGEVKVESS
GAGVLFRVVLPLASDRGRPRS

>gene_639|GeneMark.hmm|514_aa|+|2625|4169 >NVVL01000025.1 Rickettsiales bacterium isolate NORP64 Contig_source1382A_16436, whole genome shotgun sequence
MQEKQMTVMTKSLTGAPTAKDIDWNSIPWNKANSEVKRLQMRIAKAERDGKMGKVKVLQR
LLTCSFYAKCLAVKRVVINKGAKTPGVDGIIWSSSNQKIQAVLSLKRRGYKPQALRRIYV
PKKQRGKLRPISIPTMKDKAMQALWLMALIPIAEERADPNSYGFRPKRSTQDARGQCFNA
LSKGKSAKWVFEGDIHSCFDKISHQWLIENIPMDKVILKKFLKAGYMEKQRLYPTNAGTG
QGSIISPTLAVMAxSGIEQKVRNSxKRVRxKEKINFISYADxFVITxDTPExLxxKVIPI
VTExLQEVxLELSxEKSKITHIDDGFNFLGFxVRKYQGVLLIKPAKENVKAVLAEIRSTI
KANYPAKTENLIYLLNPKIRGWANYFRGSVSSKTYSYVDEMIYRMLKAWMLRRHSNKSKS
WINRKYFKRRGFSNWNFYATVKDKQGDQTPLYLYKASETPIRRHIKIRAAAHPYNPEFNE

YFKFRESTSKTRITNKNPDFLLDYKLLGDNVAHT

>gene_640|GeneMark.hmm|640_aa|-|4241|6163 >NVVL01000025.1 Rickettsiales bacterium isolate NORP64 Contig_source1382A_16436, whole genome shotgun sequence
MASDPPASDPTRVDPLDQDGTLKVDLCDGLEFRLEFESADDIPKVVATPPEKPVFQTRHE
ATGGLDLLEIPFDSPEIYLDLSADASAGDAMEEEFRASLGRADSLFGSLRDAIGDYDIEE
DEPPLVMNGEKPTCEVLELPHPSPELNDETIALDMLKMAQSMQERFERIRLTLTEDIFNS
REGEREDACRLSRLEAEIKFSAAEETMRHDSEMRFRNMWDIIDGIIDDSLEEEREYSARL
SRLEKDIKSSTAQEIMRDNAQVASEYAEPFVQRHETWEDWSSEEDTPALSTIEERDELPK
FQPLSDEPTFSDDELETIEFSSSAPSSINVAYEEERSNYERELNASDFVLPLDEEIQTPE
KVIQHTIGSDSKYTNPFTQGIHDSDYDGDSEYFKEEYTPDLEQPDPLDLLEDELVEPNNP
SPAVEELIIIEEEPEILAYSPPSALVDLTHHAPHNEPVDYKMDLNVPDFILQPDEKTKIA
ASNNDLPANTEQLFQEAEDDISIFSLIDEVILPESDIIGEISQDKPTEVKPSITPSQTAS
INDAPLAKKAATDEVVVNNIQKVSEDAKTKPKIPMEQEINLGTKQEEAEERNAYNTWLIQ
KETEHMVSAIGLMRRFIMPRFNTKCRVKSTLLKIPPLSAE

>gene_641|GeneMark.hmm|67_aa|-|6370|6573 >NVVL01000025.1 Rickettsiales bacterium isolate NORP64 Contig_source1382A_16436, whole genome shotgun sequence
LQEIMPFPAKNLELVELHNFADQQQAAAAIFEYIEVFYNKIRLHSTIRYLAPAQFEDNER
ILRKSVL

>gene_642|GeneMark.hmm|114_aa|-|6715|7059 >NVVL01000025.1 Rickettsiales bacterium isolate NORP64 Contig_source1382A_16436, whole genome shotgun sequence
MTKLIDMINSFKDNGIEFISLENNIDTTTPMGMLLFTMCTAFSEMERVLISERVKAGLDA
ARYKDRKGGRPKALTPDKLKRLKLLKKSQAFSVTDMCNTVKIIRSVYYRPSIIT

>gene_643|GeneMark.hmm|822_aa|-|1|2466 >NVVL01000026.1 Rickettsiales bacterium isolate NORP64 Contig_source1382A_17624, whole genome shotgun sequence
MFGIEIGLDLAVVIGFLLINLGVGLYYGQGVKNIKDYALGGRDFSTPALVATIVATWVSG
SYFVITVSEAYREGMWFVVAGMGEVFSLLLTAFVLGPKIGKFLGNLSVAETMGVLFGRYA
RIITAIASIAKTIGYTTAQIKIFSIVLSHILGTDSMLTMLFSSTIIITYSAFGGVKSVAF
TDVIQFITFGAFIPVCALFIWKIFGDTELLRNTLEHSSNFDYTQLTNIENPKFWTYLFLF
IYFTVPQLNPTMFQRMLMGKDANQVKRSFAIAAIIILVINLVASLIGIIIYANNPDLEPN
NIFIQIVDDYFSREVKAVALISIIAMIMSTTDSWINSGAVIFVHDLCKPLNIKIRNELLL
SRIFTVCFGVISVFLALYVTRLFDLVLLTSNFYMPIVIPVLVLSLLGFRSTERAYLISVV
CAIIVIISWKYFVTFKVDSIVPAMIVNLVSFLLAHYLLGEPGGWVGNANEPKAKEIKVTQ
RISEWFARLPARIHQFNLLEYCKSRLPKSSAVFNYFPFIVAMTMATTMSIESDLYNSHLL
LINSLQILVLTIATAFFCRSLWPQILKEKYLGLIWCVSIFTGLAVISSFLVLLSKFSHMS
LAILTIHLTTISLFVxWRSAIVMISxGLWISFFFYERYxxEMVxGEIYDLKLKMIYVxxM
VAGFAxTIxRSKQExLEQxEHxxxTLETEVTTQDxKITxxNKExxHYxxRVSxQAREIEx
xxxTAQKILxNVNHELxxPVGNVMxFAEMMRDGLGTFSDEQxxMISEEVYTNSNRLSxMI
LNMxDLAxxNAKKIELKKEEVNFSKLVHERFENCRDVYAQDK

>gene_644|GeneMark.hmm|161_aa|-|2577|3062 >NVVL01000026.1 Rickettsiales bacterium isolate NORP64 Contig_source1382A_17624, whole genome shotgun sequence
MAKESEITNESNIVNITDNSNLAGRRAAIMFHRPYMETVFYEKLHLEVECKQLGITIVDT

IYNDDKDAFIAYNKLRPHRKKESDESKPLILLASARNLLSSRSLLPKIILSALIELKEID
LYLYNHGFNIKNGKSELTLKPCNVKGILNMKKSASNLGLFV

>gene_645|GeneMark.hmm|854_aa|+|3675|6239 >NVVL01000026.1 Rickettsiales bacterium isolate NORP64 Contig_source1382A_17624, whole genome shotgun sequence
MNINQNLINLRLTSIYKADSLWPYVNLYKRGAKFMSIFKRFEEQAARRAVEAERRKIAGI
ATIEAEKVGSALRSEQSSAISLAQRASDAYTDREVTKAKQSISSLQSGLSSAVSRLSREE
ASTRQSNQSISQLQAKWAAVERAEQEVAELRAAAEEAGRMELAAIEQQEQNAAQLVLQQE
LELQAARQIAPYEPNIQPAVIFPAEDIEGKFSAEHSEIIQTISSRICEGNDRIAKEKHPQ
AILLIGNTGAGKSTLAHALSGRGLQGIVEDETGELLIDALHPTEDIVIGHQMTSETTIPN
KCRNGETVVWDCPGSNDTQVDQEFPNIFYIKRLLEMSQAVRLVLVVPEPNIVGDKGSKFV
ETIKYFMNSFDAAMLGNSVSLVVSKVAPHKRIDHIKNSIDKVLTQNQQVTDEMKAFVGTL
LESSSVHLFYRPRDEGVMPSQANLLAAIEADGDYVVSDKNLAKLTVSQPAQEYSHDLLQT
VSGNFNNIVNLMINAVADSTNCMRADDGNLFTKNHAMIARWVPDQMSYDDLRAREELYFL
NLDMLTALKGLFESPVITFEEGVAVLRGAMQIFERFVDNDEAKQQMQEYAYVLNQQFEYI
KFFSEFIEEPEAQIYDLNALQAVIAQCWENIATNFEYQVKTIELEPGQGAAYYKKAIFYL
DQCDHPVCATTIAQAHMRIAELVVGDDVPAAIEHYVAAIGYDKTLAEVYEQLGQLFQREG
EYSKAIECWKVVGKAYAIKNSFDAWLENAPENVYAIMMVRGGYCEYKGRVDDAVSWYNNA
ASVAVNLEAAKQASSESERTLSNAKNIRDEGAARVREMMDSNFCNYSMVTPEFVESIADG
IVIAGGAFEADVVV

>gene_646|GeneMark.hmm|129_aa|-|6387|6776 >NVVL01000026.1 Rickettsiales bacterium isolate NORP64 Contig_source1382A_17624, whole genome shotgun sequence
MTKEIKILSVNRYPLNTTLTRLWKKNFNCTIDEAPTGRDALKMFTKTEYNLMFIRELPDM
LSTDIALQIRSSETELNREPAIISVLIPRTPANQDPKYLDAGIDYVLEDPLIGDKIEEVV
KKFGLGGDW

>gene_647|GeneMark.hmm|57_aa|+|3|176 >NVVL01000027.1 Rickettsiales bacterium isolate NORP64 Contig_source1382A_18017, whole genome shotgun sequence
EQFCGQAKILLDLSGRNIHKIALKNGQRIYVVPEAWYMAAEDLMREKKTQEEGGSDE

>gene_648|GeneMark.hmm|325_aa|+|169|1146 >NVVL01000027.1 Rickettsiales bacterium isolate NORP64 Contig_source1382A_18017, whole genome shotgun sequence
MSDIKVFDENEEEPENGICTRWVEIGALFILCEFVVFSVLLMTKEGALIVDIASMWVVFL
LLFLAWVVVLGFVFGVWGLEKAEALSFGWGFVLWLSVVLMVLLAFATQEGAGLRRQEDKE
KQHLQQVTTQLEEATAKYVPWLFNVEEKDIQHKKSYVVDVLENLSSQDLLDDWYNSNSAK
QFVLQKTKVDAKAQRVELTGLEFGAGHASDTPWQQRIYVIHFIQQDGRWLIRNILDKRLE
EHFKRVDELKEQGTQESETQESEIKQPAQTGFLTPGELYQSIKDLLGFVSRQRYLFAQNN
NTNLGYSISLDILYLEKSRDLDLKI

>gene_649|GeneMark.hmm|134_aa|+|1339|1743 >NVVL01000027.1 Rickettsiales bacterium isolate NORP64 Contig_source1382A_18017, whole genome shotgun sequence
MIKKMAISSQAASSNAASSQATSGCTTGGEAAVNKKGEKELRYKQLLALGAGLGVLALIP
EMALAANFNLNAGVTAATTPLITALTEHWGKGVLISGAGNAIIGEGDGWQRATRAVKGCI
AGGAAVLALIAMLG

>gene_650|GeneMark.hmm|98_aa|+|1736|2032 >NVVL01000027.1 Rickettsiales bacterium isolate NORP64 Contig_source1382A_18017, whole genome shotgun sequence
LVKAMNERVFTKCLNHERKYWNRSLGSIVLGVLMFALVGLWQGLMWGLGSGAIGFVVGGM
ISKHWYLGLIQRKLYWNLPFAKVTVDRNTPDSSDRELL

>gene_651|GeneMark.hmm|190_aa|+|2074|2646 >NVVL01000027.1 Rickettsiales bacterium isolate NORP64 Contig_source1382A_18017, whole genome shotgun sequence
MNQEIVRLNIETIARQRNVFLLLAMLAISCSLALSVKLVSTHDRVILVPGLNQEVWTSGK
GVSSSYLEEVSAMYLPLLLDLDAASIEWKKEHLMPYVSQSDPSYMKNLGEYFARVKARYK
KFTLSTHFALKSLESDQVNLTVKASGQLISRFGERGFKTNPAEYMLSFEWVNGRLLLREF
AAVSKEKEEK

>gene_652|GeneMark.hmm|252_aa|+|2648|3406 >NVVL01000027.1 Rickettsiales bacterium isolate NORP64 Contig_source1382A_18017, whole genome shotgun sequence
MIRIKGCMLGLIMLLYLVCAGGAMAGDAHIPSEYKFITVSYGKPTKITLSSNGINRINFA
EQRVVQIVGESQKFTKLLTSHGADLFLTSKLSGEAGCENISVVLSDGRVIDFVIKIEKSD
VPTIIKFNFSSLVFQRHEMVLAASKMIRAMREERVGKYYVQDVDKHFKLPELLAGVGVQV
VANYRYLKLAGVVLKVTPSNGGIEISENDFHQMFEKVISTRIGKTQGVGTRGVPVRISKP
EFVYVVYERSEE

>gene_653|GeneMark.hmm|447_aa|+|3403|4746 >NVVL01000027.1 Rickettsiales bacterium isolate NORP64 Contig_source1382A_18017, whole genome shotgun sequence
MSNLLTSLKEKAIKAGVRIKQLAGRGTGLPVPGLPGLNGESARGNNKILRKKQRGVVVVS
VCIVLVLIGGVKSYWESSQESKRLAKTKTSETGRRASGSEVLKIELADKALDPELHWRNF
YEELRRSDRLEFEKRLKEIEKSQEAVLSKAKKAIDTELAGTKRKLMLAHQELEDAGMELT
RVARMEQERINGAPPHMETLLEAEYFEDEIEFFRPKSAKNYVPEGTYFTGYLLGGIMVST
ALNTAADNATPVSFRLKHRGNLSKLNKTDISKCRIMGSAYGDLSSERAIIRLEKMICEKD
GFYTASRVAGQIFGADGFNGIKGTVISTASKHIKNAMIGGIISGLSSSAKGQDGMSILSG
GLTSTQKKGFKDLAGGGLLKGVGNAGDKLAEYYLKQAEAMSPVLTIPSGARVNLQITKGF
FMGEKTTHRRIKNGRKGAKQIKKEQKR

>gene_654|GeneMark.hmm|120_aa|+|4743|5105 >NVVL01000027.1 Rickettsiales bacterium isolate NORP64 Contig_source1382A_18017, whole genome shotgun sequence
MSILKTSIRVVCVGISMMLLSSCSSYKSSFSCGDAAGVHCTSMDCVDRLISSGEIEQYHN
KARLKGAHKKWKKSKGNAAKRSIAHENMDLPRVKTPRVKSQGLKTPRVKASLVKPLHGGV

>gene_655|GeneMark.hmm|514_aa|+|5105|6646 >NVVL01000027.1 Rickettsiales bacterium isolate NORP64 Contig_source1382A_18017, whole genome shotgun sequence
MLAIEKHLSKFADYCGLKTGGERGCDPMEEFSAKIDAATGGATPLADVLLYDSFDDDSGL
AFHKDGSCGFFFEISPLVGSSAGLEKNLTLFFNDELPEGGYLQFLIIAGHDTSSILDMWE
SGRTCGGPFLERITAYRRDFIEKCGRDFENAGDGRLARNFRTFVTYSTNDSGTEKGLKKI
IKFKRKLENKLRAEKFSPVLCKADDLLQINREIMQMDINLDGATKPARHNMARHDILNSL
SNQMLSPLSPVRIEEEQINHLATGLVTKVFHPSELPESFSLSRMISLLGEGDKTVPARFI
ISYTIANNLGASGTSAMKDQGNRSIHASEMSYTRNDLVAREEARDWREVLAIHKKGEQFL
TESMQVSITAPKEEIEIAEEVLKSLWNAQDWKLAISGNIQLLGMLSMLPMMQASYYQSLK
FFKLTRVAMSGEVVAKLPLQGEWKGVWKSGVLLMGRRGQLFCWNPFERVGGGGNYNCTIV
APSGAGKSFNLAETATSMIAQDVAMFIMDIGGSY

>gene_656|GeneMark.hmm|629_aa|+|2|1891 >NVVL01000028.1 Rickettsiales bacterium isolate NORP64 Contig_source1382A_18079, whole genome shotgun sequence
HDICSIIDIKEENKLKISRIFAIFISVAAVLMALLLKNLIDLILLAANFYLPIVTAPLLL
AIAGFRTSERSVLIAMFSAGVTVIFWKWQLQAITGVDSVLPGMVMHIIALFAAHYLLGED
GGWTGGGTSSGAGNSFGTGRDGTIGTGIGEDNKRILSRRERVQMAWQELKDFNILSYCNK
RLPKSWIIYISFSFIVFMTIVPIVSIEKDLYNMHTGLISSLQAIVFAIGTAFFCRGFWPK
SIKEKYLGLIWYVSIFTGLALISSFLVLLSKFSHMSLTILTIHLIMISLLVGWRMSLVMI
PAGLWISFSFYEGYIGEMVTGEIYDLKLKTIYVLVVVTGFAATIIRNRQEHLEQTEHKVG
ALETEAITKDTKITTLNKKVCHYSERVSDQAREIERLGATAQKILNNVNHELRLPVGNVM
NFAEILRDGLEKYDKEHLKMLSDEVYQNSNRLSSMILNMLDLATLNAKKIELKKKEKINFS
KLVHERFENCRNIYAQDKKALKFHLNIEDNVIVFADINYIRQMIDNLVINAINFSEQGTI
AISVNSEEYFVSFTIKDKGKGIPKTEIYDIFTPFKMGSNTESKAEGRGVGLALCKAVIEA
HDGEIKAESRPVGALFRVCLPYGELKEGK

>gene_657|GeneMark.hmm|77_aa|-|1975|2208 >NVVL01000028.1 Rickettsiales bacterium isolate NORP64 Contig_source1382A_18079, whole genome shotgun sequence
VRQNQESEELTNLIQKHFGFLTDEIVDVLFEMEARDTDAGGVLTCSQSEEFALKFDDILS
QSFHLFSSDLKGRAYSQ

>gene_658|GeneMark.hmm|107_aa|+|2260|2583 >NVVL01000028.1 Rickettsiales bacterium isolate NORP64 Contig_source1382A_18079, whole genome shotgun sequence
MLYNNTPFEKTLENTPDELYEWIDEVKNTFLNKFKNIELIAQKDFETLTKVVDANNRKEQ
AKHIAKTKYPKLVFAMLDAKDFRSMIWKMLEPSKESPFRLRKNPVTV

>gene_659|GeneMark.hmm|341_aa|+|2637|3662 >NVVL01000028.1 Rickettsiales bacterium isolate NORP64 Contig_source1382A_18079, whole genome shotgun sequence
MTAKNSNKINKNKKQSKSTNLAKAEIEKPKKLKHDEFMKETLGRRLTAIDFVKDHLPAKM
QEQLDLSTLEVEKETHVEKSLKKRMSDLVYSVKTKKGDDAYIYMVLEHQSSVDRDMALRL
QRYQLLLCQRHRDNKEPGKLPIIFPLVIYHGKRKYTAPLSFWELFDDPEYAKEIMAGNYT
LIDLRAMSDDDINYDRHHSLAFAAMKHIYDRDTLKMLDQLLANCKIAVSLDKREKYLFLS
LIIEYTSPKVPAEKREELAQVIQGHMSKEDGVKFMKTVRDSYIDEGYVIGEKKGAEQKSI
QIATNLLNQNLDQKFVASVTGLSAHEILKLKSSAHNSNDKS

>gene_660|GeneMark.hmm|91_aa|+|3686|3961 >NVVL01000028.1 Rickettsiales bacterium isolate NORP64 Contig_source1382A_18079, whole genome shotgun sequence
MNKAEFVSHIAKQYNCSKVRADEIINMFTSSVIDALGSGKEVSLAGFGHFTVSPVAARTG
RNPRTGEPLQIKAYNQPKFKSGQRMKDACNR

>gene_661|GeneMark.hmm|152_aa|+|4242|4700 >NVVL01000028.1 Rickettsiales bacterium isolate NORP64 Contig_source1382A_18079, whole genome shotgun sequence
MSTSKLQNVGVFILLLIFISWAVYADESAATIQKATKHAASSDKEIHLAYLQCYALQEND
KKQCHQLITQQFNLDREPSSQKYWLYYSYEAERLGFQWFLNSHNKSCAKIDHGVLFNQAT
NSYDVICADGNKFKMSFSYKDNSWTLLEWLIV

>gene_662|GeneMark.hmm|201_aa|-|4697|5302 >NVVL01000028.1 Rickettsiales bacterium isolate NORP64 Contig_source1382A_18079, whole genome shotgun sequence
MPLSKLGANLKLLRESAGLSRNHIERNYKIPTGTVLAWESNKCLDIKLTKLMNYLDVYKK
LGFSVTIDSLLSEKQFSPVSCKLSILNNFLVSSFGIDLLQKIPNILFYSDRDENILYLNP
LYHNTDSLDVKERMNANTLTAGQMLLKANNEYRAQIDEVLKGKTVNAEYHHAGDHMLKIQ
LQPNIYTHTNKTIGFVATIVN

>gene_663|GeneMark.hmm|100_aa|+|5539|5841 >NVVL01000028.1 Rickettsiales bacterium isolate NORP64 Contig_source1382A_18079, whole genome shotgun sequence
MDNNQTYHLDAPALSTHMNSLVVQAFRELGDSLADNLAFLEIEVLEGEQNCNVRTAYMRI
KDKIAENLDNILLRHSLAIIDEIRKEAVFIDTICSTEREF

>gene_664|GeneMark.hmm|131_aa|-|5933|6328 >NVVL01000028.1 Rickettsiales bacterium isolate NORP64 Contig_source1382A_18079, whole genome shotgun sequence
MHKQELATTRESLQLLLAKEDIDMCVNRVHSSSSTAEYKYCSADLITSKLLETEAPALEE
WESLSVLPAFNSKDSFVRSMIHTHIAKYCTFLVGILYELQSSKNLAILSVKFKQELAEAN
FHKANPGSQSE

>gene_665|GeneMark.hmm|482_aa|+|3|1451 >NVVL01000029.1 Rickettsiales bacterium isolate NORP64 Contig_source1382A_18225, whole genome shotgun sequence
EMEIIKAENSLYESGLGAVRGKRALVVIMFAQKFAKTRNRIGQTEWEGSAKLLADDAKVS
VSTVRNTIDYLADHGFMNWELGLLQFQNGDIKGQHHSYYYPTERMTRLIDQVDPIKSAER
AADDGMLQAHVTVLNYGEEIDQNDLFSYWEQTAETIGGQNIDHTCLESRSKDIYPTPTVI
KPQRKHIGNKQMRRNEAIPVQATLAPAPLRDTKTSKLKPNDISDMPNTKTVAFPFQGKKV
LEMHSTQKRRDSADLLELEDHEINLGNMPIAADGMEARELILIGLEALIISLALRQKDPE
IISGYDFECLENAPISTLAKAVFNSAYLALGANIITSGAFAKAVKSSGTITILALLDTTL
KKISRTAIPIKNPFGYWRSTVKNSIESADPITGLGSAIKTHSAVVAADLRDFLTKTSALL
ATEWVNGQAERSAEEILEAHKDIRQKRGDWSDTMPYIDHVKMAFENRTVQNRTLFFPVGL
MF

>gene_666|GeneMark.hmm|68_aa|-|1523|1729 >NVVL01000029.1 Rickettsiales bacterium isolate NORP64 Contig_source1382A_18225, whole genome shotgun sequence
MKKTRTTPDRKKQILFKPMKYWRKKRGFSQKEFAESINKPAAHVSEFETGKRSVTEVWIT
SICKSAEH

>gene_667|GeneMark.hmm|135_aa|-|1803|2210 >NVVL01000029.1 Rickettsiales bacterium isolate NORP64 Contig_source1382A_18225, whole genome shotgun sequence
MLKYYSLENHTDLKKQIMAIKRASKINSKFKTFERMKFWRKQRGFSQTEFARKISKPQPH
VSQLESGNRGVTDDWMHLYADALNVSPIVFFEAGIDPISEKISADVAQHVKKLTHPQKIK
FYMTVLELVNQPIEK

>gene_668|GeneMark.hmm|112_aa|+|2615|2953 >NVVL01000029.1 Rickettsiales bacterium isolate NORP64 Contig_source1382A_18225, whole genome shotgun sequence
MYPSVTFRFRHGSNLAEKLNAEDNKSKVIKQALQQYYNAGPTTDPEIPAVWQSVNRKLSG
VSTNINQIAYAFNRSSKKNVEHLPPVESLLELNKQVSNIISELERLTIESAV

>gene_669|GeneMark.hmm|587_aa|+|2950|4713 >NVVL01000029.1 Rickettsiales bacterium isolate NORP64 Contig_source1382A_18225, whole genome shotgun sequence
MSKRPRNPWEQRLRAPTYRQQKVTIPQKFEKSKEVVFKVLSHSKTIRGVVNSIAYNADHG
GQDEENSMFDKNGVQLTPEEITKRIDNWNLIRDGYENFSKKAREATPVELREMPDDEKYY
LRQSTHFMISFPHEHNELTDDKIHKIVQDAIRPMTDAGHHALYAIHRHQNKPHAHVVMKY
RGYNNKRLELNPHQIQKIREHIVEVAKEQNVELTATRRRDRVQSLEKRLPSIENYKAAYL
YREMLKQKKDFDKAGLSAEDINLALSKINERRDQIKKYKKSDKIYNALGKKIQKLEAKHE
KKPSDKLRQNIAGLAVQRDGHKVAIDAEMNIRAQRVNKPEDLVYSAIDKSQLKEVKKTLL
GDYEAVTQNINDLKLTRRHLENVAPEMLKQSKGDFVESKSHFDIVLENRNITRASLIKRQ
APNWYASHGLEYELRRSGATNTDVPNPYPNVRLPKLAMKTNQELKKFTEANYMDPEAARK
SFLIMAADNSRTAFWYANKCGEIFGELKPDAKRIEISSRDIRLSKDWKDQAKVSIKSAYS
NVSDNIESYHQEIADKLSRNNENVNQVGYLMNSVEKEGLTKAVYRSG

>gene_670|GeneMark.hmm|217_aa|+|4942|5595 >NVVL01000029.1 Rickettsiales bacterium isolate NORP64 Contig_source1382A_18225, whole genome shotgun sequence
MILTMLNNIKILVVVTISILGISLASAKAETVIIDGTLSYWYEGSSRHSKYKLIQEDYPR
INNEGYLLPPKRTLIDEDGNEVPYSLEKHLERIEIIMEERRSYNFEIVCDLDVARHKMFV
EDPQAYAGMLRNKQHPCMESPGHISAHRIKSAKHIKFVLDFIDEKEIHVGGLNRSHTTFF
DDLAIVSLGDLRAGVNFSIKNEEGFLKDRQVNLKIRE

>gene_671|GeneMark.hmm|227_aa|-|5789|6472 >NVVL01000029.1 Rickettsiales bacterium isolate NORP64 Contig_source1382A_18225, whole genome shotgun sequence
LAIDDYLAIPFAVLMFIIVVMTVEINTDGDITGVLTAFRDIGLRLGLATIANGEVVDFES
GASSAGIGALLCKISDVYDKNFWELTTTLWESISFYEIGKMFNIMFLTTPYFILDKLLLI
TLLIEFIVFYGAALLMPAGVYFLMFPKTRTFFMSLIKIMLGVMMKLYLVVVINGVILTTF
EVANSHAGGEDKTLLDFYSADFLYMGLSGWILIHLLRRISRRCKSDV

>gene_672|GeneMark.hmm|116_aa|+|2|352 >NVVL01000030.1 Rickettsiales bacterium isolate NORP64 Contig_source1382A_18417, whole genome shotgun sequence
KQIDKIGNNGEKVMKTIADGRREEGWKDGLAEGREEGREEGREEGREEGISIGEERGEKI
GEERGEKIGVEVERKKTVARMLKENFAPKIISSITGMSQRAISKLRSQLELQGKLV

>gene_673|GeneMark.hmm|98_aa|+|440|736 >NVVL01000030.1 Rickettsiales bacterium isolate NORP64 Contig_source1382A_18417, whole genome shotgun sequence
MLKQIDKIGNNGEKVMKTIADGRREEGWKDGLAEGREEGREEGREEGISIGEEVERKKTV
ISMLRENFAPKIISSITGMSQRAISKLRSQLELQEKLA

>gene_674|GeneMark.hmm|61_aa|+|760|945 >NVVL01000030.1 Rickettsiales bacterium isolate NORP64 Contig_source1382A_18417, whole genome shotgun sequence
LCFVICPPCGGSGAEKHGNSMPSGEFLDSAFRKLLLAGLEYCFEIRPSFWGDNSRPADVA
S

>gene_675|GeneMark.hmm|835_aa|-|942|3449 >NVVL01000030.1 Rickettsiales bacterium isolate NORP64 Contig_source1382A_18417, whole genome shotgun sequence
MYALQVLLLKTQVLLYNNCVIFIMLGIPMSNTENKPNADLYEADSIKVLKGLEAVRKRPG
MYIGDTDDGSGLHHMVYEVVDNAIDEALAGHCDLVRVIINKNGSVTVSDNGRGIPVDIHK

EEGISAAEVIMTQLHAGGKFDQNSYKISGGLHGVGVSVVNALSDWLELRIWRNGKEHLIR
FRNGDAEFPLRVEGDGNGKKGTEVTFMPSANIFKIMEFCYNTIEHRLRELAFLNSGVRIL
LVDNREEETKEIELLYEGGVQAYVEYINRSKTALHPCIALCSSDNKQGITVEFAMQWNDS
YHENILCFTNNIRQRDGGTHLSALKSALTRVINSYADKSGLMKKAKVSITGDDAREGLAC
ILSIKVPDPKFSSQTKDKLVSSEVRPVVESSVYAKLLEWFEEHPSEAKAIIGKVIEAASA
REAAKRARELTRRKSALEVSNLPGKLADCQEKDPALSELFIVEGDSAGGTAKQGRSRKTQ
AILPLRGKILNIERARFDKMLASEQVGTLITALGTSIGQDFNIEKLRYHKIVIMTDADVD
GSHIRTLLLTFFYRHMMPIIEKGYLYIAQPPLYKVKRGSSEVYLKNEQALQDHLVSGALQ
DASVKLTNGEEVSGAPLEEMTSQIVKFNSRLSNIAKRFDSNIAECLAINDLLKTESFTSS
KKDELVAALSPLNLGDAAPDKTDWEVSVSEGSIEFSRLVRGVKNTKTLYREQAESSEFIL
LNKIGQKLERFFLGKCELNVKSQSYQISLPSHMLQLITESGKKGLTVQRFKGLGEMNAEQ
LWETTLDSEKRTLLQVGVNNIDEAEEIISTLMGSVVEPRREFINKNALNVTNLDV

>gene_676|GeneMark.hmm|292_aa|+|3448|4326 >NVVL01000030.1 Rickettsiales bacterium isolate NORP64 Contig_source1382A_18417, whole genome shotgun sequence
MAGKFMTQLEFKQLSAAIDDYDLILFDIWGVIVEGGEVYDGVVDAINKVILQKEVIFLSN
APRPAFKVINTLRGWGISDLTEEMITTSGDLARKIILQKKESLGGRNPVIYHLGADRNDD
ILSDFDHDLTDDLDKAEVFLLSIYRDDNEDIHEFDELLKQAAKKPNLLNICSNPDTTIPK
RGSLRYCAGYFAEIIEQHGGEVVYTGKPKTTIYDEIFRKKPNIKKNRILMVGDTFETDIL
GANDSGIHSALVLSGNSEPFHNMHSAMEDKLVALQKRGMKVGSMPNFVTKLV

>gene_677|GeneMark.hmm|143_aa|+|4330|4761 >NVVL01000030.1 Rickettsiales bacterium isolate NORP64 Contig_source1382A_18417, whole genome shotgun sequence
MFSAPCEFILENEQDSKALAQKIATNLKLHDVLAFTGDLGAGKSFFCREIIKSLCGQQIN
VISPTFNLLQIYESKNFSIYHFDLYRLKHQSEVYELGIEEAFDSALSLIEWPEIIGEILP
KGSIFVDIKIISDSSRRVSIRKD

>gene_678|GeneMark.hmm|551_aa|+|4844|6496 >NVVL01000030.1 Rickettsiales bacterium isolate NORP64 Contig_source1382A_18417, whole genome shotgun sequence
MAVEDKGASLESGSRESGFGEARSREARSKENSTQRRRDPDDFDVLLGGRKADSPEKETP
SANEPDIYQTTATAPGGSRPKSEEKHAEPIHIEVAVPPNATSETLAAAQVQEVEPEPEPD
AVQEQEQEPEPAQNEEKEQDEKATLNAAPTVGLEDAPSPTDDEAKTEAIRAREDALFEAR
AKEQEQAEISASGGKQGDDDPGEERATDRFYSGGSGGDKAPPANELDQSVINASYINDAV
DDITQAIRDQIIAKQQEILQAAIAKTLESEARDKFLNLTRLEFRDYLNSKQGRGQAELAL
QNSGVQAEIQRIDLEGYRSVHYDFKDRFRNVEWEKKDTTSTSIVKDGDREICKLTETIVN
IPGQFVTANGEQVEVTSYRKINFPISLENGGPMHLSMAVKDQHGKNISKQDAVYFTAHYN
EEGKLMELSSPVPVKFAGEGGDAIGYIERGGKIYTLPVTRANYQAMMHEVGINNGLSTNL
SQTLPSPAIATPAQAAGQEQATGQAAGQAMGQDQGRERGQSQPKQRDPSNDRDAPPPPPA
PPPPPPPPTPG

>gene_679|GeneMark.hmm|518_aa|+|285|1841 >NVVL01000031.1 Rickettsiales bacterium isolate NORP64 Contig_source1382A_18590, whole genome shotgun sequence
VSLQQNTRGGKQNPTREVSLGIGNIVPHYWYKSIKAPSGKPDLVAITVLSELWFLHRKSG
GAEFNAGYANFERKFDFSRSQLKDAMVRLHDDGLLRRSFRALVINGRTFPNELHLALNIA
SLLAMLPNGASGGGKSGGSSFSQDMESSNSDDGEIFHGEVFGNSVQHSLETPEEHIRNRK
ISLRKNRSLRSEESSFKINDLKEELDSGSTLSATSNSVPSPSDYQKLKPANAGLGEFYPL

EREDIAALASSSGREFGMNAVNEILLKLSKSCPDHSFPNKAAFMSYMSKVLRYEKRDAVR
INNAHFRIRSNMSETELATVEQERFLAGIENIREVSPLMHLKKKLASVLAPDIAAPLLRA
FRSIRRDGDVCKIYLSRQVELTDLNKELILNQVKATHEGLTSGGAGMFETISRVEIISPQ
PSAVAPEALKPEMLASTTQEPGIWGQVRARLIEYFGADGVAIDQNWFSRLRAEIDDNSQS
LTLKTSSEFFRDWITSHYRDLIERFCKMFGFEFAGVVV

>gene_680|GeneMark.hmm|88_aa|+|1933|2199 >NVVL01000031.1 Rickettsiales bacterium isolate NORP64 Contig_source1382A_18590, whole genome shotgun sequence
MEKEFKAARKDNSAQDSLDLIVLEWVEEQFCGQKKILLDLSGQNIHKIALKNGKRIYVVP
EAWYMAAADLIREKKTQKAGGSEDLLKG

>gene_681|GeneMark.hmm|70_aa|+|2228|2440 >NVVL01000031.1 Rickettsiales bacterium isolate NORP64 Contig_source1382A_18590, whole genome shotgun sequence
LLKVSQTNLARSWDNATSESFFSTLKRELVELHNFADQQQAAAAIFEYIEVFYNKIRLHS
TTLISCSHTV

>gene_682|GeneMark.hmm|254_aa|+|2672|3436 >NVVL01000031.1 Rickettsiales bacterium isolate NORP64 Contig_source1382A_18590, whole genome shotgun sequence
MRQSLNLELRTETLELVKDYFTQLKMFKVDHIGYREFRKDGTSMAFCSHLDWYDVDTDVV
YKEEMANHYAKELIFLVKNQFDCITRSASSINNKFLQELSSKDMCNSFLIYKNTEDVIYM
YAFISSSLNPDALSYFVNQSGFLEELINKYKATFASVFQKSKYQELREQMFSEKVAQHIF
SDLTSSMQNSSFQQLTPKEIECTALVARGWNNKAIAKKLNISTRTVEFHLSNIRNKLNIN
SRNDIVDIAKHINY

>gene_683|GeneMark.hmm|451_aa|+|3399|4754 >NVVL01000031.1 Rickettsiales bacterium isolate NORP64 Contig_source1382A_18590, whole genome shotgun sequence
MILLILLNTLIIKRTMKDLSKKVMGSCFLAACLEMYDFAIFGFFSSVLHSNYLSFLDKKN
AMIIAYALFAVGFIFRPLGSLIFGYIGDVYGRRIALILSVSFMGSASLAMFLMPSYAMIG
ILSCYLIALIRIVQGISVGGEYSGALIYAVEHFNKKKSGLVGGVVICGCITGVLLASIVS
KILQNPELPEYSWRFAFLLGFGLSAIGYFIRKNLLETPEFKRLGSSRVKVPLIAGLKNYT
TECIGTIFAAASLGINFYFILIFLPGYINKLTGLDVKFYPTLTTVILIILSPMFGWLSDK
LNRAQMLKWSMLAISCYSFVGLQLVALYPTYSSALLFFCGHAIIFSIQASTANIFIIEVF
PPQCRFSCASVCYSLGMGVVGGTSPLLASLILDKFDNPTFYLSSYITMISFLGYVGVRVI
TSKKERVKEVEPLIAESDLVWSYSYEFENQQ

>gene_684|GeneMark.hmm|276_aa|+|4898|5728 >NVVL01000031.1 Rickettsiales bacterium isolate NORP64 Contig_source1382A_18590, whole genome shotgun sequence
MKNTIELRSGISLRESLAAVEGEFGIHASSVDISKLAEVVIPDQIKDEIPTLNRMGYMKT
AVDSYSQEFIDYASKSDLPVLEIGCAYGFVAGRVLENGGNIIATDLSAEHLAILLKKTNQ
EHLERLHLYEGSFPDNVDFPKKSVGAILISRVLHFLRGEVVEEGLDKMHGWLSDGGKLYF
TAVSPYHVAFKEKFLPSFQERSRAGDKWPGVIENLWEIAPQHKEYVHEFLNIFDIPQLEG
LLPKHGFTLDRISLFDFPNDTDSAGKGHVGFAATKV

>gene_685|GeneMark.hmm|181_aa|+|5886|6428 >NVVL01000031.1 Rickettsiales bacterium isolate NORP64 Contig_source1382A_18590, whole genome shotgun sequence
VTILQKARGGKKNPAREVSLGIGNIVPHCWYKSIKAPSGKPDLVAITILSELWFLHRKSG
GVEFNDGYSHFERKFDFSRSQLQDATLRLHETGLLRRSFRALVINGRTFPNELHLALNIV

ALMAMLPNGGGFVGGSSSGLDMESSNSDDGEIFHGEVFGNSVQHSLETPEEHIRNRKISL
R

>gene_686|GeneMark.hmm|244_aa|-|1|732 >NVVL01000032.1 Rickettsiales bacterium isolate NORP64 Contig_source1382A_19237, whole genome shotgun sequence
MGRHKLRIKDALKKIHCALESRGNLAAPIIESYCEMIESRMRGQIRSTIISPAISGMVSS
GFNMLGEQIISQMESGTLIEEARLLPDRDLSGISAGDAADEEIKHHANSDPDSSDDEAWS
QNNLASRDELGHFFPIEDREEPHEPATILNTEFGPVIIPSSAPMSFDPEQSPGLEFRFSS
VIEAEFLKMSDEDLQQEYSTLAGGMGALRPEFLYNAREIALSLAYKARFAEQIGQYNVAA
IADA

>gene_687|GeneMark.hmm|92_aa|-|835|1113 >NVVL01000032.1 Rickettsiales bacterium isolate NORP64 Contig_source1382A_19237, whole genome shotgun sequence
MNKDPVKEYRLLMTEFVNGNIADSQFEISYLDLFMDDKKQGMSKELYKIISDVFFDVEDY
CSDVTLRDERDIDETELLKRTKAALKKLNDFK

>gene_688|GeneMark.hmm|263_aa|-|1115|1906 >NVVL01000032.1 Rickettsiales bacterium isolate NORP64 Contig_source1382A_19237, whole genome shotgun sequence
VEYIVTREYLISLLKAWKNDKITDQNVYDTANFLYPNDEIYYIDWELEGGWDNDDNTSIA
QEVVSILEPMDVDGILKEDADAYIEFLHTPIGKYKEGAVKLGIYESKIDYTKRSEEFAAL
YPLSFPTVRNSSTETFVITREYLISLLEAWKKDQITAEDIYNTTNGLFNSKFDSFIDWEQ
KGMLGSKVSLTQEVACALKAMDKNLITKDDIDVYLEFLHTPIGSYSEGAAKFEKYKSKIN
YAEREAKLSDISPYSKSKNGIVV

>gene_689|GeneMark.hmm|246_aa|-|1912|2652 >NVVL01000032.1 Rickettsiales bacterium isolate NORP64 Contig_source1382A_19237, whole genome shotgun sequence
MLDQAAHTMHLIDPGSMAILNGATNAAGHIVSKTLQKLPESFTNAVAVSYASLMSDCRTK
NQRFAVETMLGVVLVEVGGAGIKAVMMNRGVGVQGVTSKIVDIQPQFKGGGRNPTISEMH
LHNAAVHDISSPARYSGKMFKFNTRDQARQFGDNAGVRKAARKFFGSATRGTVGRTNIPT
FAHFKARKVPNGDTVMEFFAKSKEPLYIKRYIQIVDRNGAVKLRRQETIIKETGRLVNLK
FNNFKE

>gene_690|GeneMark.hmm|96_aa|-|2799|3089 >NVVL01000032.1 Rickettsiales bacterium isolate NORP64 Contig_source1382A_19237, whole genome shotgun sequence
MYKIKDFGIFLEKVLKRTNKPSVIGNLAFKEYHNNLPDVEKEVQDIAITLSSMEDGIEFE
YSIDELHEIAKRLTEGDITVTRIGFDQKVNLKTSKT

>gene_691|GeneMark.hmm|607_aa|-|3092|4915 >NVVL01000032.1 Rickettsiales bacterium isolate NORP64 Contig_source1382A_19237, whole genome shotgun sequence
MSLVSSTLSKMASNSGSAAARAITSTVSVVAQIASAIDKIEAIAGEFCDHMEHTIHDISS
TLPTTEGILQHMLPSIRANDISNITRHLQNADIIQDNWNINASLLGFEGGIQQLTSAPAT
GTISPTRVSPTSLTTASIPRPFEFEELHSFAPPASTAASTRPATQRRNFDQISGMGRHKL
RIKDALKKIHHALESRGNLATPIIESYCEMIESRMRGQIRSTIISPAISGMVGSGFNMLG
EQIISQMESGALIEEARLLPDRNISGISAGDAVDEEIKHHANSDPNSSDDEAWSQSNLAG
RDELERFLPIEDRPEPHEPATILNTEFGPVIIPSTAPMSFDPEQSPGLEFRFSSVIEEEF
LKMSDQDLQQEYSTLAGGMGALRPEFLYNAREIALSLAYKARFAEQIGQYNVAAIADAIR

ATIGRMPVIGDIAAEGAISTIEHITGQPLHKPGELPAINMIGNRVAAGFGGNISLDSTAP
KPVNVNRVPRINQQPARDNVQNVNAQSALAKKMSSLQNAQTNAVKTRTLLDGRIRYYEKE
RLARKEGITRGSSYVTEYNPQTGRVSSWNECYDHSGKVIRVHPKTKNGMVLNSVHFPLTK
KELEESR

>gene_692|GeneMark.hmm|161_aa|-|5029|5514 >NVVL01000032.1 Rickettsiales bacterium isolate NORP64 Contig_source1382A_19237, whole genome shotgun sequence
MVVSAIVNSIKWTRKNKPITEEQLKLLQKIPFIFPTMFIQLLKHSNGGVLDYDFNYCDIE
DKRVMEGNIPCIYGVELKYDNLIKEYHAPPEFFSKNIVAFSDTGGGNYVCFDYRSNPKTD
NPPIVYWNHESCEGEDVSFIAKDFESFLRMLKEPEYDDDEL

>gene_693|GeneMark.hmm|223_aa|-|5496|6167 >NVVL01000032.1 Rickettsiales bacterium isolate NORP64 Contig_source1382A_19237, whole genome shotgun sequence
MPVIGDIAAEGAISTIEHITGQALRQPGELPAPNTIGSRMLGFGGNISLDSTAPKPVNVD
RVPRINQQPYDPRAAEALVQERFGGDGVKIVPRTVPRSNAPNVRLAGTRHPETGVVFDNR
GFPIFDDHVIFDTRIPSKVSRVENSKNHMRAATSDLRQKIDEGVVNPNMFNAEQLEAINN
CEPKIPNYTWHHHQDQSRMQLISTELHKTTSHIGGMKLWWFQR

>gene_694|GeneMark.hmm|82_aa|+|3|251 >NVVL01000033.1 Rickettsiales bacterium isolate NORP64 Contig_source1382A_19549, whole genome shotgun sequence
RKFRKSNALKKEQGNSRIFKSKSGKSILRRVGKNGIQKLYTLTDKAHIRPRFGFQKMAHK
VFIRHFDKIFQRQLDHTLKTAL

>gene_695|GeneMark.hmm|67_aa|+|412|615 >NVVL01000033.1 Rickettsiales bacterium isolate NORP64 Contig_source1382A_19549, whole genome shotgun sequence
LERGIKNNFIGSPVFFKRRKPLRILTVAQGSRRRKAKKTQVPQQIALNLEPTQKNEMPHL
PISVWGV

>gene_696|GeneMark.hmm|91_aa|+|694|969 >NVVL01000033.1 Rickettsiales bacterium isolate NORP64 Contig_source1382A_19549, whole genome shotgun sequence
MVLTIEMIMEHIYANLTASISELKKSPTALLNSSNGEPIALLNxNKPTAYLVPAxLYEQI
LDLLDDRELLQIAKSRLNEESKAIRVELDEL

>gene_697|GeneMark.hmm|82_aa|+|959|1207 >NVVL01000033.1 Rickettsiales bacterium isolate NORP64 Contig_source1382A_19549, whole genome shotgun sequence
MSYKLDFVPAAKKEWDKLDFTIKEQFMLALTIFFMRSLQKQKRRLESKVEREQKAIADLP
ELAITVLDYVKNHGRVTNRDMA

>gene_698|GeneMark.hmm|276_aa|+|1392|2222 >NVVL01000033.1 Rickettsiales bacterium isolate NORP64 Contig_source1382A_19549, whole genome shotgun sequence
MSFFLHPNKPKILRKDKLELMFHSNEEIIYQNRDDQLGDMIPIFRHIFVIYFCDLKQDTP
PNTTQNSYSLIVEQYKKDNEITYKAYFGYGDSLRVKSEIDFDISPKGFGIGSIIINHLIH
WGKQNYPEAKIKQLYLSAHDEEDKNNHIRRDRLYNKIGLIKYKGFKHLSDLKPSCDERDF
EVIDAYKYINQILIEQDTLRTSKSDLKGDLSYLEECSKQEKQEIKKYKKILDNPFRYTAY
YLCNTIDNIIGYIVHWLPNKIASILKGVANTKKQDD

>gene_699|GeneMark.hmm|93_aa|-|2330|2611 >NVVL01000033.1 Rickettsiales bacterium isolate NORP64 Contig_source1382A_19549, whole genome shotgun sequence
MSKLIQLIKFLKSCYRKVYVYYIFSTRKNWHISKNKNYYNPTYKATVYWYSGFGRNSEIK
EGWSIARFGVYYNGYKSREIAQEQAFKMWMRQR

>gene_700|GeneMark.hmm|86_aa|+|2666|2926 >NVVL01000033.1 Rickettsiales bacterium isolate NORP64 Contig_source1382A_19549, whole genome shotgun sequence
MLFSLCYKKLYIFSCIFTIKGGKGVINLSLKHATKPQIKKERALMKKSYPRLFSKGITTN
QRKTYTLPSNVLLFIGYKETIFDTLK

>gene_701|GeneMark.hmm|1056_aa|+|2923|6090 >NVVL01000033.1 Rickettsiales bacterium isolate NORP64 Contig_source1382A_19549, whole genome shotgun sequence
MSADKVTDILDYWIAVERFTPYEVEMNNKFGYVESIQKVCAAEDIAWKNKFKFKHKNTKS
HNWVYTVFLGIIKIDDINARIKDLLGSDVNDPSWLKSNSSSCIGCFQVNINGKPIKGTFE
VPDYFPNICSLAQRKSLPKTWLDNSCVFRRKITDRFENYCDALEQENNHNSLGYNDLQIL
LDQIIDDSLWPELTEKNLIKNIAVIHAHKLPIPKNKPQEDLVTLPDSAIHNSFYTKDLEN
IRMDGRFGKGFQEYMNMIDCGEKIDLFGKQGALKEYSHPNYLSGARWPVAGNYPLSTAQQ
IAVNLALKSNEGLFSINGPPGTGKSTLLKDIISGVLLERSRILLTLDNANDAFESSVSVS
VQGYNYKTWRLIPSLLGHEIVIASSNNAAVENISKEIPRLREIDINLEQDVDYFSEIASY
ILKEDSWGLAASALGNRKNCLDFFEKFWYKKPKKNPVVKEDYYYGLEYLLQNTTDIPDWD
KAKQSFKKVLNQYDEIISELNKLTYAMSQLSILSGNIFDYHVERLDLEEEKEQLTDKLED
ARNQLLYLHKSLCDQNEYHVRHVQERSSGIGYLIDLIFNRERIRYWLQEYRRSSCKLLSI
CKSINTAEDTVNQLEKELRNKASRIRILNEKTHIAETEYDQWLNIELEYRSKMGDNFPDD
DFWQKDDKEILLSSPWLFEELHEIRKQLFIESLKLHKAFIFNSSKRLLNNLRCMHRIISS
NDWPVSRTDLLPHIWGSFFMVVPAMSTTFASFSTLFRGMGRESIGWLLIDEAGQAPPQVA
SGAIYRAKRSIVVGDPFQIQPVVTIHEVINNALLNYYNISDQWNCLLESVQTIADRANIY
GTFVGNSENKKWIGCPLRVHRRCADPMFKVANEIAYNNLMIQATESVEPLPGAMASSQWI
HVPESVSNNNWIKEEGEVVQNLLSKITGELGTLPPIYIISPFRNVVFNMKQKLSHNLDKW
CHTQNTEQEIKLWINKSVGTIHTFQGRESSAVILLLGGDARKPGVFSWASKTPNLLNVAI
TRAKNTLIVVGNHDKWSKMPFFQVLARSIPKQNIKP

>gene_702|GeneMark.hmm|146_aa|-|1420|1860 >NVVL01000034.1 Rickettsiales bacterium isolate NORP64 Contig_source1382A_19779, whole genome shotgun sequence
MDQTLRLVQNVETVIIKEMQRAIQLAPHSLSDRPVVPRDGKCAVLIEGVRATHQLCERLR
DALRHLLQLSRQILGSGEGDVEGSGVVGVVCVDEGHPEGDSMGRRRSVGDECVWQIGISP
DLGTDEVVIVSLVALRLGLDRGIIIM

>gene_703|GeneMark.hmm|89_aa|+|2420|2689 >NVVL01000034.1 Rickettsiales bacterium isolate NORP64 Contig_source1382A_19779, whole genome shotgun sequence
MLECCINGDVMRPKLIGGKFVRGYHLQFMLELFKIEKIEHRMSIKVVVQSVSFASVFHNN
HDVMKICVDVSPSLYLTSENDNNSSIFQK

>gene_704|GeneMark.hmm|50_aa|+|4495|4647 >NVVL01000034.1 Rickettsiales bacterium isolate NORP64 Contig_source1382A_19779, whole genome shotgun sequence
MKARILDYQTVNQNTLNQLQFELRTIDGKLVGFEGSQNTFINLIFRLIIE

>gene_705|GeneMark.hmm|86_aa|+|5313|5573 >NVVL01000034.1 Rickettsiales bacterium isolate NORP64 Contig_source1382A_19779, whole genome shotgun sequence
MIYYYLFPLSCLKEFDNKKEIKVFSDLYIQCVLNSADIHIGGIRHIKLLLYFFIVIDAKK
KCIILLFYPKKTMLTYKCFSQTSLLV

>gene_706|GeneMark.hmm|104_aa|-|5700|6014 >NVVL01000034.1 Rickettsiales bacterium isolate NORP64 Contig_source1382A_19779, whole genome shotgun sequence
RETAGVSEVSLRPLERSARSREKPREMQLKHLHAQHFNETVLCSSRRRRELQLARNNQRD
IKMVVLVPARPHGEVREKPKRHKNNTAFYEKYKNILTVMNVIKP

>gene_707|GeneMark.hmm|110_aa|-|3|332 >NVVL01000035.1 Rickettsiales bacterium isolate NORP64 Contig_source1382A_20288, whole genome shotgun sequence
MQISISSNIKQIAKQLDHMQKRQLPFATSTALNKIAIAAQNSITKAIPFIFNNRKKWWGK
NQPTGIKVKFANKYELVSAVYTRAYFANIQEEGGIKTPRSGHKLAVPASG

>gene_708|GeneMark.hmm|148_aa|-|347|793 >NVVL01000035.1 Rickettsiales bacterium isolate NORP64 Contig_source1382A_20288, whole genome shotgun sequence
MAITREEKSNSRLMQLFAPALVGADQANIAAIDVSGFESVLVVANIGAAGDDLNQDNYIE
IELEESDDNAGFTHCSDESMQDAVAGTNTGTIAHLEAIDGAGISVLAAYKGRGRYIRPVI
RMTGNHGTGTIIGVSAILRGTKYRPVAA

>gene_709|GeneMark.hmm|172_aa|-|1032|1550 >NVVL01000035.1 Rickettsiales bacterium isolate NORP64 Contig_source1382A_20288, whole genome shotgun sequence
MKMKTVKLNTAKLNTLKLQTTKPQTSKLPSPNKNDKRPIAKFHRSWLQDTLSGTPLEKHR
GINQAQYAAADRLSSNYQRIFSNGGWSPEEVQVKQDYFRSNGVERKVQAIINHNRIFKQL
SKNSQAIIEHFCLNEQPIRAYELKQIPNWPKGAGTARLREGLDELVEIYRNS

>gene_710|GeneMark.hmm|769_aa|-|1835|4144 >NVVL01000035.1 Rickettsiales bacterium isolate NORP64 Contig_source1382A_20288, whole genome shotgun sequence
MSNIIQMVPNALPQRDQLQTFFYVLFGYSEGLIPCRSFPESGTDNSQRPHNIWIDADELV
MDKVVTFAKWTAREKVAFYVIPGIVSKQGQAGSDDIEQMQVLLIDIDSGDTEEKLKILTH
AMGQPTMIVESGGITPEGSAKLHIYWQLERPAAGDDLQRLVTLRHHIALAAGGDTHFKSA
HQPIRVAGSIYHKKGAHKLVKIRSYEPLEYNLEELIKSAEKLPLPAQTSTSEKITSPLDY
NNAARSFDHIITSKVYEGAEGEQSRFSHLQRVVGFWLRRLHEGAITQQQAWEEITGYNAA
NVVPAWDEKKLRQLADGLWRKHVKKHGEAAEAREAREIAEPVTNVNTNSNKINAIDPSTW
HGEPPKRQWLIEDWLPRGYVTALYGDGGVGKSLLAQQLMSALATGQSFLGKVQPKMRVYA
LMCEDDASELWRRQVAINKHYGIDMHDLSNIRLVSRVGNDNLLMTFANDDVGKLTKFFEE
LQTDILNYKPDLVILDTAADLFGGNENNRPQVRQFIQTACGQLARKIGGAVLLCAHPSES
GMQKGTGSGGSTAWNNTVRSRWYLKHPEAGELSSDHRILSKVKSNYSAKGQEEYFKWQDG
ALVGLDSAYISKPVIGQKQHNKNAAERDRKTQLMLRLIDLEAQQGNIYTMNQFAEAFEDQ
EDNNYGFGSKRSINERLSIASTQGLMKFFNDPKLYGIDIKKSNYGYLCCENTMLKIGEII
DSETGEITDKMIHIKPTHYKCRLNGELLVIKNSEIKEGENGENTTAKKQ

>gene_711|GeneMark.hmm|264_aa|-|4207|5001 >NVVL01000035.1 Rickettsiales bacterium isolate NORP64 Contig_source1382A_20288, whole genome shotgun sequence
MFGGRIMLDYNKIAKTKSSTPINDQLNILIDDALVKIRDTQPKRRYLGASRVGVSCSRLL

QYEQSGDSKDRGKGFSGQTLRIFAAGHVFEELATQWLRDAGFELLTNQEDGSQFGFSCAG
GNLAGHVDGIIIAAPKALGLAVPALWEMKSMNNKSWKETIRKGLVVSKPVYAAQIALYQA
YMEGAVVGISKNPALFTAINKDTAELYHELIPFDGELAQRMSDKAANIIRATKAGEMLPR
IASSSDYYECKMCAYQQRCWGNRQ

>gene_712|GeneMark.hmm|170_aa|-|4988|5500 >NVVL01000035.1 Rickettsiales bacterium isolate NORP64 Contig_source1382A_20288, whole genome shotgun sequence
MQFNDFNNAETSNFDLIRKGTLAKVEMKIKPGGYNDEERGWTGAYVTKNEVSDACYLNCE
FTVLNGEHQGRKVWSLIGLYSPKNDNRWGDIGRGFIRSILNSAKGYSDSDKSDKATGARI
IKGLGDLDYIRFMAKIDVEIDQDGNDKNVIKTAIDPSHRSYLASDQDVWG

>gene_713|GeneMark.hmm|111_aa|-|5490|5825 >NVVL01000035.1 Rickettsiales bacterium isolate NORP64 Contig_source1382A_20288, whole genome shotgun sequence
MQIDGSKTELELPGIVDQVITMAEITGDDERPYRAFVNHTINPYGYPAKDRSGCLEMLEE
AHLGRLMEKIKRARQDGKNLTYDMAKESANNLSEQSNKQSNKQNKENNYAI

>gene_714|GeneMark.hmm|171_aa|-|1|513 >NVVL01000036.1 Rickettsiales bacterium isolate NORP64 Contig_source1382A_20327, whole genome shotgun sequence
MRQDNKRFRGGRLGIFCRLAVLQIAIICSPISAFAGVDKLIKNVFPSGTMSNVTRGGVVE
HQSAGHLMGGSVMIRTPANPDLQMLHVQAPSCSMGGLPCGAQFEFFGGAASLMKLPAMLQ
HFQGLAKHLGIYAGITYIKTQCSFCEDVLEWMDNKADFLNQLANTNCQDMM

>gene_715|GeneMark.hmm|347_aa|-|510|1553 >NVVL01000036.1 Rickettsiales bacterium isolate NORP64 Contig_source1382A_20327, whole genome shotgun sequence
MIRNKRTLIRGMILLQLGLAATAPVYAVSSSYESSPSRANIPSSRVVGTSVMDTSVTGVG
AYSSTIDGISPSGASSSNSTTKGASAFFGERYRGWLWFEEKEALVPEGLAPEAAEADIDE
EAEFIPPTKEEMQRAKTENDVFKEELELMRHMMIRYPENLDYVVMYKKKEQEMHEGARKL
SRSWVMANFLNPDMTDELENPQNIYGRKLQKSLQKKQDKAALRALSSQIEIFVFRKEDCP
YCKTLEKHLSSFARMYGFKVEAVSADGSSSQHFKTHTQKELIEALSLDVMPMVIAVTNDS
SMRFELARGAVSILDLENKSLMLVRYLESLKESGSAAGQELNNGGRR

>gene_716|GeneMark.hmm|374_aa|-|1546|2670 >NVVL01000036.1 Rickettsiales bacterium isolate NORP64 Contig_source1382A_20327, whole genome shotgun sequence
MQVKMIARYMIKTVMVLMALCSIWAGSLASSFADTSFRSNYPCHDTGKRCVSSGTRMIDG
FEVHRDCWEYSYTKKCDYPSKDDCGRYAHCYVLGDRNCLLQDSAGKCVNMRKEFSCKEWD
AVNDEKHTVRMGLEEKEGARGVVCKGVPCMDGNCVDKSYETNGEMMDSLSKLYSTTQMNP
DKDGNFNLFPGSHDHCSKKAAGYSNCCRMGKQGWGSNLGAKCSEDEKSLMEQRSKNLCIY
IGKKTNKVAGVTTLVKHHFCCFGTMLDKVVQQGARVQLGMSFGNASSANCRGLTLEEITR
IDFSKIDFTEFIDDFRAKFAAKYSPPDAAGIGAHVESVMGDIAVFDENDPNPENNPENNM
TGWRQNIIDEEWDD

>gene_717|GeneMark.hmm|405_aa|-|2655|3872 >NVVL01000036.1 Rickettsiales bacterium isolate NORP64 Contig_source1382A_20327, whole genome shotgun sequence
MGVVNLGNIGSLVNSILLMSCISGAILFWCSPSYADHLDPTFRTNENEQTDLFEKNIPDL
RSFEEQIKSDRENAIHGIGNSSEDVKMQGIEALSGKSISEVEAETERLSKIPAMELESTG
RAKLLEDEILDDLWLDGSKPGNIRHKEDAKRLAKAQEGLLANMLGDLKDMGIECGQVRGN

TIREPAYYLQLETISRRPDTRYEQMFCESPRREYNCRQFFNVRCTNKVSKPLQAESFATN
MPVRYAPRTGILTFGWIDNLSHIGECGEVYDYNIEFDLNDLRNVTEFKLLNVSFDDHVRI
TVNDHQIFMAPEQGDRLELTGERSWRSRYFLVKVDESDRRYSAEYGTRHNHAPDIDIRRH
LKEGSNSIKVRLIVGGGGGIWLKFRAKVAECENWGISREEQCRLK

>gene_718|GeneMark.hmm|271_aa|-|3872|4687 >NVVL01000036.1 Rickettsiales bacterium isolate NORP64 Contig_source1382A_20327, whole genome shotgun sequence
MEEEKLLFYVENRIVKKSLVLLLLTLVSVMSSTSSFAKEEEAEVSMETKEWAKGLTEETR
RMSGETKKWVSELTTLTRDISMDAIKAKWQELQVMRMAKGEIDDTEIDIDNIDSSLTENI
GLRVFISSSMSPSLLRQYGQQAKKYGAVLVLRGLPTGASGRGSWRKLSELVMTISGTSPG
GTSAGDASIPDASAEGIGIQLDPASFEKYNITSVPSMVLAREEWSLDDGQEKETGEFDKV
IGNIGLRAALELFAEDGDLADEARSFLEEAG

>gene_719|GeneMark.hmm|342_aa|-|4657|5685 >NVVL01000036.1 Rickettsiales bacterium isolate NORP64 Contig_source1382A_20327, whole genome shotgun sequence
MKNNPGNILILVILLLVPFTSSASFTCHGHFVNPLSDVCWGCVPPLNIGGFSIGKGSSPK
KRDTKNPSSPICACTRAGVPMVPGISIGFWEPVRLVDVTRTPYCMVNLGGIELGSDKSKI
SAHSRGRGGNGGTHSSFYHVHYYVYPLIYWLELITDFMCLEAGTFDLAYMSEFDVTWNDP
KLQGLLNPESILFANPLAHASCALDCASATISMPIDSMFWCAGCLGSMYPFSGANGAHVG
GVKTSSLLVTRILAKMHRVGLAQETSTSSAxIDGxICRKSRAFKIKKSQYxxQMVNPKTT
SGGIGCWPLGxTDMMYSAFKEYPYDGEDWGYLIWRKKNCCFM

>gene_720|GeneMark.hmm|42_aa|-|5694|5822 >NVVL01000036.1 Rickettsiales bacterium isolate NORP64 Contig_source1382A_20327, whole genome shotgun sequence
MHPHDAPILYGMLLDCKAALQERPDLRKRWSHLGRELLVKVD

>gene_721|GeneMark.hmm|188_aa|-|1|564 >NVVL01000037.1 Rickettsiales bacterium isolate NORP64 Contig_source1382A_20496, whole genome shotgun sequence
MNFSKEEILIVTKATKTLIVPKGIKKLICTEIGLETLILNEEVVYVDxHNNKLAKLTVPN
SxTHLDCSHxxLTKIxVPDSVTHLYCSHNKLAKxxxxxSVTHLYCSHNKLAKLIVPDSVT
HLYCSHNQLTILIVPDSVSVIHLDCSYNQLTILIVPDSVTHLNCYNNKLAKLTVPNSVTR
LSCSYNKL

>gene_722|GeneMark.hmm|290_aa|+|817|1689 >NVVL01000037.1 Rickettsiales bacterium isolate NORP64 Contig_source1382A_20496, whole genome shotgun sequence
MKLRIVIILFISIIIAIITMTSWAVRHSLFAPDKHMIWAPDEYVDMMIEDVNGFDTINSW
YVKNNEKSTCMLFFHGNNGNMSWREYMVNMTKEIGVNLFMIDYRGFGKSRGNPTQRSLLE
DGESAYRYITKKYKNCDIIVWGESLGGAIASHVASKHKCRCLVLLSTFSSLDDVVLEYHG
NYAIRYLMSLLIKYIVEEMPIKSWIKCVKSPVVIIHSKSDETIPFEVSNTLFENISHEQK
THIKIKGKHSSPEMTRDNLMEILDICGISFNGKTITKALKCIDNIKFPIV

>gene_723|GeneMark.hmm|600_aa|-|1679|3481 >NVVL01000037.1 Rickettsiales bacterium isolate NORP64 Contig_source1382A_20496, whole genome shotgun sequence
MDPISHEKQRPSSNKGMENKKRISEDEILNDDRKALEREILNNTSTCSSGTIDLVSWRIS
PLNILMTKNGLRNNYYDPKVMESAACLVNSELDTNKNDVIGLRIKNWLTNLRIISEGVEG
YALSVGLGNTKDVFVMKTPKSGNNLLHEFFVGIVGLNKLRKIIPNFVYTLGGFGCPAPIV

QGEELVSWCGGDSDDSVDYILYENVDQSITLKEFILGGCSTDEYLNIYIQVLYALNIAGK
EIDFMHYDLHTGNVLVRTISEDEVYVPYKIGESVHYLKTKYLAVIIDYGFSSIVHKGMDY
GIYGFEDQGVGRHEPQLVDVTKLLSSSITDYLDKEQPNLGLGVAMGSISKLLDNNQDIHV
EEFTYKMLREFDPKFVTFYKPYNVRVIGCGDVGGTDDCIEYKNVPIMKDEEVIKPSSILD
LYDICFGLGKEEKYEQFDKMIESFDYKKHLMASMKEINSAHSKIEKSKRKYLKLKSIKRI
MEFSWEYIPIYFNTKTKDFVNAIRVFRDTKVLILLIKSVHYASTIYNDVDTTNRAARYIK
RTKTNNLRNASTISEMISGINIMSELSESYKYKPGYKDKYHKYFYFPLGELKLLMDSIKL

>gene_724|GeneMark.hmm|685_aa|+|3579|5636 >NVVL01000037.1 Rickettsiales bacterium isolate NORP64 Contig_source1382A_20496, whole genome shotgun sequence
MQVDPTSSLCRVVSSFAPKSEPTNIIPFRLGDVEVKVKIQSNKNQIIEYANLKSHSLLES
IKNRFDNPKFSKSRNLTNPFEEIGNSIFMNRAGVKIANVDAVLRLTNIQSGYLNRYSDHP
LTYAAIAEAPGAFVQYLQYRFNNSKGFGISLADSEITWNYDLIKCDNFTVYNGEDGTGDI
LNNYDSFSREVLAEHADGVDLVVADGAIDSVGRENKQEQDNMKLIYAELLIILTICKTGG
NCMFKVFDTYTKASVHLLLTAALSFEDIYIFKPISSRPANSEKYVVCINRKENIEKYISA
LVSCVKNGDNIYSIYTTIPKKFDEWVTKSNNFHLQRQITYGKKIIAHMRNTKIRVDLYDL
HKCNILWNIPTTPLSDVMYKRRDISSRLSKKYYTDDIEPIFVKGTHKHKGFVLIQEGIID
GGTIMRGLMPYISSINNNTILAWFMSDDTDIVSLSLCAKLNKKKVVIFVGYSNTSGELVK
ISRSYGAEVKVIHGVSENTAPSLDTVKKGALSYYKGNSNVHMINKSLGDIRFISKSYSES
LNDVIRFVPGRIWISSGNSMYHNIAKQFRNVKIIRVTTRHNPRASKEKNVETRGSKTKQN
YETRVIPPYSSNLMSDGKIWELFLKYGKDGDYIWNSYSPKPLNVGVNDAPTLSFEPIELK
ISDFGSTKSIGAMKYKVPILSFPGV

>gene_725|GeneMark.hmm|60_aa|+|3|185 >NVVL01000038.1 Rickettsiales bacterium isolate NORP64 Contig_source1382A_21498, whole genome shotgun sequence
AQEGPTPAQTRKRRLSQVNEEKEDHNRSTISPKHRKGLPALQEKSRREIQEGVRTNRRYL

>gene_726|GeneMark.hmm|678_aa|+|679|2715 >NVVL01000038.1 Rickettsiales bacterium isolate NORP64 Contig_source1382A_21498, whole genome shotgun sequence
MLAESTSLTVVYDCNHDKVTYQAIDLLEPQPCDDPDTDFMDPTERRVQILNTEKDRRIIAY
ACQVFVTKEIHFCGFDSISYGSKITENETPVDITPSVCREAVKGGKMHLTEWGKTYELGP
TQTYFDHFYSHGSLDNKGHCERETFTSGGIAYTGASESIFINIRLKKIRGILDVSTGLVQ
FSTGISVPYKDGVLRDQTEGTMVWDLEPPKCDSTLGELYQGKGLLHQKKTGLLGSILLIK
DNQTGQYTGLTLQMPTSICGMECRGTQIxGVVACLLREYDSPLPLVEPxFPFNSLxMDVL
SQVSYLHLSTNLKMHQNFRELYQQTCLLDREQLVLKLQILADEGSPYMLTDLFPPGHRTY
RAGTVVYIAKCVPLQAKRISYPNCTQEIPVEVDGVRRFADPYNWVLQDHPNEAPCSDLTP
IRWKLEGQWYCSTPTIRPCDAPDKLQLPPQYPALVPTEFTRGLGTGLFTKIQLQQHAEFV
LAYMARAPTLAKLVSSATRQMLGGSTLGSPIGITDLEELTDRLSYHLLPLFYLLGNLYHY
VVAIMIFWVIIKIVTGSSVRAYILWIEKGGGWWILAACWRTAYEVVRSPITLLETTTTTV
TEPVNQYPCVEQYYPLEEGTPDQAYRVSVIHTTIRRNQRQLAVLENLFPRHTPIMKRQLY
EDKGAGEDRDRDSKEESP

>gene_727|GeneMark.hmm|69_aa|+|3519|3728 >NVVL01000038.1 Rickettsiales bacterium isolate NORP64 Contig_source1382A_21498, whole genome shotgun sequence
MRKEGMPLLFRKKKRWRKMGKRQNILLPETSLSLPSRNLQQSGFDQQQACIHVYIQPGED
PPDEGTGIH

>gene_728|GeneMark.hmm|76_aa|+|4053|4283 >NVVL01000038.1 Rickettsiales bacterium isolate NORP64 Contig_source1382A_21498, whole genome shotgun sequence
MWANIFVDTMGGKPHYRERNRERSITIGKRNTQKVVKESGCKHWMTGHKEGSENMKFKSL
CKCQTTLKTRVGANIG

>gene_729|GeneMark.hmm|176_aa|+|4955|5482 >NVVL01000038.1 Rickettsiales bacterium isolate NORP64 Contig_source1382A_21498, whole genome shotgun sequence
MSEQQEKMTTGNKAASLEVQDSRKPGAQEESGPEPEVKKITPGQGPASGRQQEEKKETST
KPQEEGKKLAEVKAVEATRTGKPRGKRTVQASDAHRKNSPAGIILFAESRQDMSSLTDWY
ERVKNLYRLAYPSRGAYVDGSALLIRRFSVGLRNARLSHELMQTPETSLMGTMSRA

>gene_730|GeneMark.hmm|27_aa|-|421|504 >NVVL01000039.1 Rickettsiales bacterium isolate NORP64 Contig_source1382A_21554, whole genome shotgun sequence
MLKTALSDFQTKEAAAVKAYNALVITT

>gene_731|GeneMark.hmm|46_aa|-|679|819 >NVVL01000039.1 Rickettsiales bacterium isolate NORP64 Contig_source1382A_21554, whole genome shotgun sequence
VYDQRAREHPVAIAAVIDAQTKVAGLYIGFEAVNDQAMFLQTESRS

>gene_732|GeneMark.hmm|38_aa|-|1640|1756 >NVVL01000039.1 Rickettsiales bacterium isolate NORP64 Contig_source1382A_21554, whole genome shotgun sequence
MGLNVIKVIVFISNNLSKFKDVVTADISVVCKLVFSSG

>gene_733|GeneMark.hmm|94_aa|+|2300|2584 >NVVL01000039.1 Rickettsiales bacterium isolate NORP64 Contig_source1382A_21554, whole genome shotgun sequence
LIDNSTQAAVDFAKYIRNLNAELEDLEIELDRKREYETKLNGDVAEGAAALKNAQDELAE
AEKTLAAEIKRCDDLEAFNLKRIKEIEEELVIIG

>gene_734|GeneMark.hmm|63_aa|-|3263|3454 >NVVL01000039.1 Rickettsiales bacterium isolate NORP64 Contig_source1382A_21554, whole genome shotgun sequence
MSLVTLSTNADPASIDKILNLMEKIQSALTQSKTEDNTDENTAIADWNDEEISLNRSIGT
LTT

>gene_735|GeneMark.hmm|49_aa|+|3477|3626 >NVVL01000039.1 Rickettsiales bacterium isolate NORP64 Contig_source1382A_21554, whole genome shotgun sequence
LTDDFESKTLFTLDFSSATLLVSMKAFPPTLLILVNELIASLIAAMAAM

>gene_736|GeneMark.hmm|197_aa|+|4251|4844 >NVVL01000039.1 Rickettsiales bacterium isolate NORP64 Contig_source1382A_21554, whole genome shotgun sequence
MNKLFAVVCLAMLASVTMGTRLRAKSTYEIPEISAIEKSKWGAQLLDTVALQIQSKAPLD
KINALLQEIVDDLDRQQTDDDNQYAIDMLWCKDEKKRLNADITNAKAAIDVAENKIAKRT
KAIKEGVALESKLVKQIDTLESHIQHTHDERKKDSEDFAKRDTETNIAIDAIADAITEVQ
QLVGRVPAPIESAIQKL

>gene_737|GeneMark.hmm|64_aa|+|4941|5135 >NVVL01000039.1 Rickettsiales bacterium isolate NORP64 Contig_source1382A_21554, whole genome shotgun sequence
LKSSLEFSLSQDQADEKASVDAFDILLDNLNRNLTTANTNLVACKKQLKDDRKNLAIQEG
VKLE

>gene_738|GeneMark.hmm|219_aa|+|1|660 >NVVL01000040.1 Rickettsiales bacterium isolate NORP64 Contig_source1382A_21885, whole genome shotgun sequence
FPEIKAQKAEYRKQEAQAAEMRKDLPQVIKHRKSKIAAIDAELSEGKIPPEKVAEIMEIR
AGYVDQVSFDQKILDQEVGSVAEESVYLDNVINGAKRGNLSKDKAIVILSKQYPAMLECK
AVDQNHKGVEQCQQLAGLYGIELNQLGSSKQANQPIVRPEKRLTQKKKLPVAALLDDQGE
LKLEKIAAKVQVRLAKRTKLQHRLTPNVKGEGRGEGQVR

>gene_739|GeneMark.hmm|239_aa|+|696|1415 >NVVL01000040.1 Rickettsiales bacterium isolate NORP64 Contig_source1382A_21885, whole genome shotgun sequence
VAKSEYDLLEEAAERYAVVELVASGNVVYKLNHARVHEIHWNSIKFATRSDCSGGFLGVY
KTLRADGRNDDLGTTLGFLLVGLIVFCNEKVIQAVSDSYKYGYGVPVNDYLAKLTIAIGQ
YLSDDFDDEANQIIEEEMRMNQGGSLDQLSLKCVAWMQKHREFLDKSAYDVTDGEVGALL
KMFEAETHSGYLANHGALSRAWHLEAIEPLHLNMNPDDDEQVAVAGAGNQNSDCCCVIV

>gene_740|GeneMark.hmm|1006_aa|+|1687|4707 >NVVL01000040.1 Rickettsiales bacterium isolate NORP64 Contig_source1382A_21885, whole genome shotgun sequence
VQNKPCNPRAMGVLLVFGILLTVKCRYMLFGVEIGLDMGIVIGFLVLTLAVGMGHGKSVK
TIKDYALGGRNFSTGALVATIVATWASGSGFFVTMSKTYSDGLYYMFASFGLGVSFFITA
FLLVPRMGEFLGKVSIAEAMGDLYGQNVRIITAIAGTIGAAGSIAGQFKVFGNIFSYFLH
IPNYIAVITAGFIATLYSAFGGIRAVAFTDILQAVAFGIIIPMVGFIIWGEFYNEGFSFS
QAASDPKFNLDILFDSGNPEFLGMLALFFHFCKPTLSAPAFQRIAIGRNVAQVKKAFLIA
GACLIFIKIIIVWIPFLIYSMNPSLESSQLVSYIVDTYSYPGLRGLMIVAIIAFAMSTAD
SRINSASVLFTHDICKVLLPTIKRELLISRLFAFLLGGGAIMFSLVETDLLSIIILANSF
YYPIVLPPFLFTVFGFRSSSKSVLIGMMAGLVTNIIWKMSPIESVSLAQKIAGPQFALLM
NAVFLLGSHYLLKQKGGWVGIKDNSYLDKQKIIRRERWAAFMSKVRSFNPINYIKTHPMS
CDSTYVILGAYFLVFTFTSIYSTHAEMLKENKIIITTIYKTMIISSTMMGMYPIWPLSIK
TEIKQMVMRVWYPLSIFYMLIVFSTFFVLLNEVNNLHFVMFAVNMIVSVILLGWQMALGM
IVAGVYIGTQFYKYYIGVEYLDTSIGSPQFILLYSMLIVVMILIIFLKPKQEHHKKTEHK
VGELSDEVGTLVTVVSSRDSQIITLDNKVSGLEERIDHYTERTTNQAQEIERLGATAQRI
LNNVNHELRLPVGNVMNFAEILHDGLGxLxDEQLKMLSKEVYKNSNRLSTMIxNMLDLAT
xQAKKIELQIKLANFSELVEERLTACRKIYVEEKPLKFIIKIEKNVMVPMDENYIRQTID
NLVINAINFSEKGKILVIVARQTNFVTFTIKDQGIGIPLRELYDVFTPFKMGSNTETKAE
GRGVGLALCKAAVNAHGGEVKVESSGAGVLFRVVLPLASDRGRPRS

>gene_741|GeneMark.hmm|67_aa|+|4711|4914 >NVVL01000040.1 Rickettsiales bacterium isolate NORP64 Contig_source1382A_21885, whole genome shotgun sequence
MRARGSRCKKRQVAALSNNLAPMRINSEYHRPFEGEASAEEFLLKPVIGASPHPMPVLFF
GAISLQL

>gene_742|GeneMark.hmm|70_aa|+|5132|5344 >NVVL01000040.1 Rickettsiales bacterium isolate NORP64 Contig_source1382A_21885, whole genome shotgun sequence

VDRAGYEAPILPPNSPDMIHSTGFEEAVNAMTWLIEHYKADEGNESFAIYDIYGCTPLIE
GSFNSRLYRD

>gene_743|GeneMark.hmm|481_aa|-|2|1444 >NVVL01000041.1 Rickettsiales bacterium isolate NORP64 Contig_source1382A_22223, whole genome shotgun sequence
MQNKPCNPRMRGLLLVFRILLTVKCKYMLFDIDVIIVAVFLIATLVIGLGHGGDVKTIKD
YALGGRKFSTGALVATVVASWVSGSGFAITISRTYYDGLYYLVATFGMIISFLIIAFYLV
PRMGEFLGKTSIAEAMGDLYGTKVRIIVAISGIMASVGYIAVQFRVFGDIVSYFVDLDNT
LTIITAGLVVTIYSAYGGIKSVTFTDILQFFTFCFAVPLVGIMIWSKLGSIDSFSLSNAL
QVDKFNYKKVLNLGNPQFWEMIPLFLYFVLPKMAPSICQRIFMGNNLEQVKKVFIISAFL
IIFVKITIAWIPFLIFNVNPDLEHTQLISYIMDNYTYTGLKGLIIVCVIAMAMSSADTDI
NISSVLFSNDICSLFSVTSEKKLLLSKIFACVLGLVAIFFALSTTDLLDIILTTSSFYMP
IVSSPLILTIFGFRSTEKSVLIGMGAGAFTVVAWKAMGIQANPIAIAMLVNLIFLMGSHY
L

>gene_744|GeneMark.hmm|246_aa|-|1712|2452 >NVVL01000041.1 Rickettsiales bacterium isolate NORP64 Contig_source1382A_22223, whole genome shotgun sequence
LKRGVVAKSEYDLLVEEAAERLEAVELVVSKNVVYKLNYGRVDGRHWNQIKFATRSDCSG
AALGAYKTLREDGCNDDEGTMLSWLLVGMIIFGNEKVIQAVSDSYKHGYGVPVNDYLAKL
TIAIGRYLSDDFDDKANQIIAEESRMNGGVSSLDQLSSRCITWILDNLKFLKKSAYELTH
GEIGALLKMFEAETHSGYLANHDALSTEWHLYTIEPVHLNMNPDDDEQVAVAGAGNQDSG
CCCVIV

>gene_745|GeneMark.hmm|394_aa|-|2473|3657 >NVVL01000041.1 Rickettsiales bacterium isolate NORP64 Contig_source1382A_22223, whole genome shotgun sequence
MANNSEKTLKNIQHQLDTRLNDVQTDAVIAMIKSFQKDLANMNVQDLASLKSSVSSLNTP
NLPPEVAGALELLLASIAKHSAELEAEKMIEERIIQEELKHARNSGVSAEEVRKEYERNE
KLAAISDIxVxEYSEYKKALFxYRExDLAISERIRDGVELTPxDRAWVFDENxEQFPEIK
AQKAEYRKQEAQAAEMRKDLPLVIKHRKSKIAAIDAELSEGKLPPENVAALLEIRAGYAD
QVSFNQRILDQEVGSVAKKSVCLDHVISGAKRGNLSKDKAIAVLSAQYPAMLESKSLDQN
HKGVEQCQQLAKLCGIELDQIGSSKQANQPIPRPEKRLTQKKKLPVAALLDDQDELKLKR
IAAKVQALAAKRTKLQHHSTPNVKGHGQDEGQVR

>gene_746|GeneMark.hmm|87_aa|-|3797|4060 >NVVL01000041.1 Rickettsiales bacterium isolate NORP64 Contig_source1382A_22223, whole genome shotgun sequence
LEKLKGARGRMLFSQKHILPEIIRSKKLARSDINITSSITKELIQAYTREAGVRDLAKLP
KSLIQKPLLEKARTNKTPTLNKALLLS

>gene_747|GeneMark.hmm|92_aa|+|4149|4427 >NVVL01000041.1 Rickettsiales bacterium isolate NORP64 Contig_source1382A_22223, whole genome shotgun sequence
MANADVRLSRNSTTTYFTTSLTIKKLSMFAHIARGNFSIPTPPPIQKGGLCSGGNAAGAC
CEKRLKRAQEIMVDIHILIFTLQTYAIYTILV

>gene_748|GeneMark.hmm|91_aa|+|4466|4741 >NVVL01000041.1 Rickettsiales bacterium isolate NORP64 Contig_source1382A_22223, whole genome shotgun sequence
MEVRLRVGSKLKKNADSIFQEMGMTMSEAIRIFLTQAVNSGGLPFRPHLTNPNSTTIQAF

EDVDSGDFEEMSIEKFSNYLDGIEGEKSKNS

>gene_749|GeneMark.hmm|57_aa|+|5017|5190 >NVVL01000041.1 Rickettsiales bacterium isolate NORP64 Contig_source1382A_22223, whole genome shotgun sequence
MSNAQKKYTSEFKSQAISLVRDANLPASKVAKDLNINVNTLYNWLRVGSYRAFALRL

>gene_750|GeneMark.hmm|31_aa|-|2|94 >NVVL01000042.1 Rickettsiales bacterium isolate NORP64 Contig_source1382A_22761, whole genome shotgun sequence
MAKKSEITNESNIVNIAGKSNLAGRNAAIMF

>gene_751|GeneMark.hmm|282_aa|-|879|1727 >NVVL01000042.1 Rickettsiales bacterium isolate NORP64 Contig_source1382A_22761, whole genome shotgun sequence
MKDRVMINKLILLLLVLVASFPAGARPKIAVSITPVASILKMLTGDAAEIITINKSAGCP
HHYQMKPSDKSRFSDAEMLIFIDEKFDGFAGKLASQFQGEIVQISEIDDIDFSDDQGKIN
WHFWLDLDNVIILSEYLAKIIIEKFPELKGIVDINGKDALVELRTLAQVKKITLSSLSDL
VVLSDSLEHFFKNSDAKILKLYQRTHSSLRDVKKLSHALGKNKNQCIVIDAGQNPLTYVK
FDKKIIQLDSENWVVEKNDPVKESLFCVRYLRMLNQLQECSH

>gene_752|GeneMark.hmm|402_aa|-|1724|2932 >NVVL01000042.1 Rickettsiales bacterium isolate NORP64 Contig_source1382A_22761, whole genome shotgun sequence
MKILDQHLAINSKNFRTIIDHLVSFGGKARIVGGAVRDGLLDVPSSDIDIATDLPAEQVT
EALKAHNIRVIPTGIKFGTVTALIRGETFEITTLRRDISCDGRRAKVKYTKDFAEDAARR
DFTINALSYCPSEEKIYDYFDGIKDLKAGRVVFIGEPEQRIGEDYLRILRFFRFSCRYAK
EIDQEGLSACVRHKDMISILSGERIKSEIDSLLQLDGAPEIVHAMYESGILQQAFPVKTY
NHQMHVKALEVAKSFGVSLDVLVIYAILFMDCAFSLNQIVKLKFSKAEAKLVVSMIDLQN
DSTMMEDSLRKIWLEDRGFIQHSIFASVIKHENESDLESINKLYERLQLLERPLFPLRGR
DITPLGYSGKELGNAINLLKTKWIQSEFTASRDELINLVREK

>gene_753|GeneMark.hmm|391_aa|-|3084|4259 >NVVL01000042.1 Rickettsiales bacterium isolate NORP64 Contig_source1382A_22761, whole genome shotgun sequence
MTEVIEEIKETEEAVIWEEFVVGQEALVAEENTATKTPTPPADRPKSTSKNNKKRSPHDK
LFKAIFGNEASARGILKKHLPEDIKKLLDLDHLEIERDTFVSEDLKESMSDIVYKIKTKN
QKKDGDVILFLCEHQSSNDKKMMFRLWKYAISAWEPYLAKNEPLPIIAPLVLYNGKRKYS
SYRRFWDLFVNSELAEKIMAGKCQLIDLQSMSDEEILADKEFGLFKFFMKNIKRDNGIxL
WxECFKKFxEHIKLDKEQELFYTKLLWSYTxxKxPIERKDELVSFMLKQINKIGNNGEKV
MKTIADGYRDEGWKDGLAEGREEGREEGISIGEERGEKKGEKIGVEVERKKTVARMLKEN
FAPKIISSITGMSQRAISKLRSQLKLQGELA

>gene_754|GeneMark.hmm|202_aa|-|4535|5143 >NVVL01000042.1 Rickettsiales bacterium isolate NORP64 Contig_source1382A_22761, whole genome shotgun sequence
LKQYTKKHGDSLGPGNLVDFFWNARLHEHVGSAKAELIKSYNSHIIMNKTKLYAFNSIVS
LLKRSFCEREPHNNLFPHLLQFMDKMKYEFSFADYIKLELKILSEAGYELQLNRCAVTGE
SKDLYYVSPKSGKAVSKQVGAPYAEKLLKLPNFLKQDARGEAELTSIEKKTAAELTSYFF
NRYVMHGKPEPHARTCFFEMML

>gene_755|GeneMark.hmm|123_aa|+|2|373 >NVVL01000043.1 Rickettsiales bacterium isolate NORP64 Contig_source1382A_22818, whole genome shotgun sequence
LGLLLASVGITLVGGSIMNSSHKASKKTEIHLRVIREDVERANANGHVVKGYEDFLAKVE
RRVLREEVIGFKPYVSQPNDSWGRMIMFEYDKNKNQLIILSYGKDGVEGGDGEDADLSEV
VQF

>gene_756|GeneMark.hmm|325_aa|-|803|1780 >NVVL01000043.1 Rickettsiales bacterium isolate NORP64 Contig_source1382A_22818, whole genome shotgun sequence
MRHLQIKIYRRNTGHHILSFIHPKTKKRKRKKFPTKKEAKDYREEIEREFQTRGVHAFND
QTVAQLMQLHIEKCPATRVKERMNSFNSFLDNFGAYPVNEVGKNELSLWFEGLRAKHDYS
TRTLKHIKSELGHFFNFLVDEDIISSSPLLLVGFGTPSPPKRKRVVLSVDEVHGLLELVK
IWDSACLYPFMVALAHTGARRSEIARLKREDVDFSMGLIHLHETKNGEDRSVRMSDELTT
MFSIHLVSHTSAFAIPDPTGAKLGDSLIYKCMKKFKKLHPNGKEWTFHSFRHSFAFNYLK
SGGQMYQLQAILGHKTINMTIDLYG

>gene_757|GeneMark.hmm|128_aa|-|2302|2688 >NVVL01000043.1 Rickettsiales bacterium isolate NORP64 Contig_source1382A_22818, whole genome shotgun sequence
VSRTKHEKTSHGTLPTCEDRSSEQHKQAAPDPPAPELQKNLSNNLLKLLCQHGLTERELA
ERIGASQGHINKLKNGHARLSNIDYLVRIAEVFGVSLEQLLTSPGHAPPITHEGKQKLKK
ITVYVVEE

>gene_758|GeneMark.hmm|103_aa|-|3197|3508 >NVVL01000043.1 Rickettsiales bacterium isolate NORP64 Contig_source1382A_22818, whole genome shotgun sequence
MRLNKALERKNWGHQQLCDVVSKASNQKLRRQTLEHWLNHSKKCSKKFHFFLPFIADALG
TTVDQLLYGHELTVKNNLLEIQRLADLTLKIINADSLPKSLKR

>gene_759|GeneMark.hmm|102_aa|+|3863|4171 >NVVL01000043.1 Rickettsiales bacterium isolate NORP64 Contig_source1382A_22818, whole genome shotgun sequence
MWTVxYSRQAAKAIKAxxGxNKLSDEARGALDALHLDLEEDGPTQNTWPHYSKLKNQKKG
LDKRHCHLVRGNPTYVACWEVINTKKKVMEVYYAGTHEKAPY

>gene_760|GeneMark.hmm|128_aa|+|4152|4538 >NVVL01000043.1 Rickettsiales bacterium isolate NORP64 Contig_source1382A_22818, whole genome shotgun sequence
MKKHLTKNDITILVRGEAIELKDSVKSSLANIIGELVGDDSVSASEVHGDWLNSPRMRVA
KYLKGVRNKEGLTQGDVCKKLGIQQSNLSKMESGERSIPPTLVGKFSKLYKVSVKRLQET
EQKKKKAS

>gene_761|GeneMark.hmm|118_aa|+|450|806 >NVVL01000044.1 Rickettsiales bacterium isolate NORP64 Contig_source1382A_23026, whole genome shotgun sequence
LIFVFEKTDDRMSQFVSNILPALFGAFTATDANDKIREKVLMNVYLCLRTFAWADGVNDE
LVHTCFDDTFNTWMALFIQIIQSNPKSLFDLKRVSLKCLTVIFRDFVNYSKDSINLIL

>gene_762|GeneMark.hmm|29_aa|+|920|1009 >NVVL01000044.1 Rickettsiales bacterium isolate NORP64 Contig_source1382A_23026, whole genome shotgun sequence
MKNQTSKSQLQKKEALNQMTKKTNMEQKA

>gene_763|GeneMark.hmm|29_aa|+|1147|1236 >NVVL01000044.1 Rickettsiales bacterium isolate NORP64 Contig_source1382A_23026, whole genome shotgun sequence
MEEPNTYVADQDDEMSLRSIRNASLDLIS

>gene_764|GeneMark.hmm|53_aa|+|1960|2121 >NVVL01000044.1 Rickettsiales bacterium isolate NORP64 Contig_source1382A_23026, whole genome shotgun sequence
LIDLFRLWSGFTKCLDIMIAMFTPFIVEIIKNYHFSTKGGVVVDKAVVDSSIL

>gene_765|GeneMark.hmm|105_aa|+|2482|2799 >NVVL01000044.1 Rickettsiales bacterium isolate NORP64 Contig_source1382A_23026, whole genome shotgun sequence
MSVITKILKSRMPSIVQSLALVFARLIHTNPTEIFSFLSSTSIENRISLKIVLDKWLLHQ
PLFRGSYAKAVTLSALLNMFLSRDHRIENLMVIGYNPSHTNINSG

>gene_766|GeneMark.hmm|66_aa|+|2824|3024 >NVVL01000044.1 Rickettsiales bacterium isolate NORP64 Contig_source1382A_23026, whole genome shotgun sequence
VSAPFKILSVLLRFLDLNDAPKPSGDMREFRMKRDFEDERLDTMGEDDNEEDYGDEDAGG
QDEMQM

>gene_767|GeneMark.hmm|52_aa|+|3255|3413 >NVVL01000044.1 Rickettsiales bacterium isolate NORP64 Contig_source1382A_23026, whole genome shotgun sequence
LNDSYADINIKEHLGEKLFEFYQGNKDYVRYCVKQLPKEDQDIAKRLIFAKE

>gene_768|GeneMark.hmm|397_aa|-|3455|4648 >NVVL01000044.1 Rickettsiales bacterium isolate NORP64 Contig_source1382A_23026, whole genome shotgun sequence
MVPVGPECLGRILNVIGEPIDEKGPVNAKMKLPIHREAPPFDEQGSGDEILITGIKVVDL
LAPYQRGGKIGLFGGAGVGKTVLIMELINNVAKKHGGYSVFAGVGERTREGNDLYYEMID
SGVIDPVGNKSKAALVYGQMNEPPGARARVGLTGLAIAEYFRDEEGQDVLLFVDNIFRFT
QACSEVSALLGRIPSAVGYQPTLATDLGALQERITTTKKGSITSVQAVYVPADDLTDPAP
ATTFAHLDAQTVLSRALTELGIYPAVDPLDSNSRMLDPSKVGQEHYDIARGVQKLLQDYK
SLQDIIAILGIDELSEDDKLTVARARKVQRFLSQPFFMSEIFSGKPGVFVELTDTKEGFI
SLLEGGGDDYPEAAFYMVGDLKQAQDQGRKMAEAAEK

>gene_769|GeneMark.hmm|57_aa|-|4676|4849 >NVVL01000044.1 Rickettsiales bacterium isolate NORP64 Contig_source1382A_23026, whole genome shotgun sequence
VIGAVVDVQFDGNLPPILNALEVEDCDYRLVLEVAQHLGGNTVRTIALDSTEGLVRG

>gene_770|GeneMark.hmm|85_aa|+|2|259 >NVVL01000045.1 Rickettsiales bacterium isolate NORP64 Contig_source1382A_23472, whole genome shotgun sequence
GFIASGIVGLFRFPDFYTKIHAAGVIEVCGLPFCFIGLALMQSDYTSTFKLVAIAALIFL
LNPVSTYALARAAMPVKLDEKGRVK

>gene_771|GeneMark.hmm|193_aa|+|261|842 >NVVL01000045.1 Rickettsiales bacterium isolate NORP64 Contig_source1382A_23472, whole genome shotgun sequence
MAEFFELPFKLDFMMSEILLVISSIVVLATCIKLISSRNMLESIIIMSVFSLFISICYLL
MDAPDVAMTETALGACLSSCVLINVTNIVGENAGNPPQKMRIILSALLCAAFIACLTLAA
IDLPEFGIADSPLQTHLTAHYMENTKKDIAIPSVVAAVLASYRGFDTLGETVVILIAGLA

VLVITTRRRKKNA

>gene_772|GeneMark.hmm|160_aa|+|835|1317 >NVVL01000045.1 Rickettsiales bacterium isolate NORP64 Contig_source1382A_23472, whole genome shotgun sequence
MLKEFKNLRVIIAFIIPYILLYSVYIQLNGEISPGGGFQAGVIFGSGIIAFDMVFGKKAL
AMHFSTTGLTLCAILGVFIYAGTGVVSLIFNDNYLNYNAIANVIGNGVTVGNGIGDTVGN
TLAQGNIVGQHIGIFSIELGVGLTVAAVMSLIYSLLREDA

>gene_773|GeneMark.hmm|119_aa|+|1336|1695 >NVVL01000045.1 Rickettsiales bacterium isolate NORP64 Contig_source1382A_23472, whole genome shotgun sequence
MHNLIYFFAVIILTSGLFIMLTSDNYIRKIIGLAIFQSSVLIFYIALGKSAQGIVPIDIC
EKTSSCLHVFSSPLPHVLMLTAIVVGFATMAVGLALIMQIHTEYGTILESKVTQHEEDI

>gene_774|GeneMark.hmm|488_aa|+|1699|3165 >NVVL01000045.1 Rickettsiales bacterium isolate NORP64 Contig_source1382A_23472, whole genome shotgun sequence
MIIATHIPALQILIPLFAALFAALTYNRFAAWMIATISAALGFALSIYAMPFAHEGMSYA
FGGWNAPIGIEYRLDSLSQPIIVFINAILLLFLAFGRELIDSSITEYIEKKHQHLFYAIL
LFAHFGYLGVISTNDLFNIYVFIEISSLATYVLMSKGRTPRALVGAFDYLILGTIGATLI
LISIGFFLAMTGSLNITDVANILSKKAASPLITIAILFFLTGAILKMAFFPMHFWMRRAY
SSAAPFMLTYLATISSIFGVYLILRFVHFTVEQQNIQEMLSQILRPIAIGTIIICTLLAL
KARNFKDIVVYSTAAQIGYIFMLITIEPAKHLLFQFLILDALSKVALFTIIAHIQNKSDS
LNLESFASIEGAGFFKFLVALALMFSAGLPMASTFIVKIQIFDLLARNHMILEFVTVILG
SVFALHYHFKLAKAVFFGSKSKHTIQIKTKSYGLAFIVLIQILTIIYIHDLASFALNTES
FIQKIATI

>gene_775|GeneMark.hmm|512_aa|+|3162|4700 >NVVL01000045.1 Rickettsiales bacterium isolate NORP64 Contig_source1382A_23472, whole genome shotgun sequence
MTQFITPEFLVIGALLVGMMNLFTPFISKEDSPVRSLFLISVSVIFLINILLLDYLFLSG
IEVNFTLFEFGKYTIALHLEPLGMIFLTLLSVLWICALLYTIKFLAINEIENSDRFLFFV
NCCVLTGSIVALSANLFTMFAGYEILTLCTIPLIAHKRSQAVSDGLFKYLKILMTTGLLL
FLPAIIVIYSSIGHGNFTYGGFIAGHFSDANAIILLLLFIFGISKAAIYPFHSWLPAAMV
ASYPVSALLHAVVVVKTGLFCIYKILAYVFGLAYLQTLFADYNWLVLLPIFTIIYSSLQA
IRYTELKMILAYSTINQLSIALLSAFLLTPKGLMAAVIHMVSHSFTKICMFYAAGNMYSV
KNSYNIQELVGIKNTMPKTSFVMLIAGLSLIGMPPFAGFISKFYIMMAAAEQENLLVMLI
LLVSTAFSALYVIKIIIFIYRPTNDEFILHLKLKPYFNEPISHRSSKRIISSKHKAEKHL
PTFMILSIALCLSGVVGFFLIQQAIIKFLMFL

>gene_776|GeneMark.hmm|546_aa|+|4740|6380 >NVVL01000045.1 Rickettsiales bacterium isolate NORP64 Contig_source1382A_23472, whole genome shotgun sequence
MIYHPTIIILTSILVLILSYRKEKLFASASLIMPIIAAITLYTMPTQSGFEFFNLNLVCE
GTPYNKLIGFAFLLVLFSANLYALGQKKKLELMLGSAYAASTLCALFAGDFISMFVGIEL
MMIFSAAIIFIGGRRKSLRSAKKYFLTHLMSSNMIVIGIVHIITKEESIALIPVVDLLTS
PKHSHAMIIIMLTGMLINIAAFPFSGWMVNYYPKASPAGFLYLISFTTKLSIMLLIKLFT
GYESLKYIAAIMIIYASIKAVFEDRLQSLLCYLSIMAMGLMLLGISHGSPLAILATVSYL
FIHIIYKALLSISISSIIDHTGIKKCHDLQRINCKLTFFAIITGIAIMINIPILSSFYVK
STISHLYEGTIFYNLTIFLTAMTIITIPWKKYLHAKESMDINLNKHASLSILFMLIVALI
VGISASTMPIIGQLDNFRELLLFSQDTLNQGIIIIAALFVTLIYALPRKKTYPINLTEYL

GDVFFYCYWRLLKNRSKEDSSKEPWMIHSLERQITSKINAIHNQQTAIFIVFAGFVILLG
ASLALS

>gene_777|GeneMark.hmm|69_aa|+|6450|6659 >NVVL01000045.1 Rickettsiales bacterium isolate NORP64 Contig_source1382A_23472, whole genome shotgun sequence
MLAKAKVSKVRKTLHLHNGDKVSIKYSDSELVVSTFHSNVEKARNILKNYKEIDFQQELK
IMRKKEAGY

>gene_778|GeneMark.hmm|262_aa|-|6751|7539 >NVVL01000045.1 Rickettsiales bacterium isolate NORP64 Contig_source1382A_23472, whole genome shotgun sequence
MIIVDSHCHLDMLSEHDDIDAIVARAGEAGVKYLQTICTRLDNFENILKIAKKYDNVFAS
VGIHPSEVEGAADAKELVNLARDEKVIGLGETGLDYFYNKEPGHHALQQASFEQHIIASQ
ENKLPVIIHTRDAEQDTIDIVTAGKKHQEFPGLIHCFTASKEFAKAMLDLGVYISISGIV
TFKNAEAIREAIEYIPLDRILVETDSPYLAPVPKRGKTNEPSYTRYVAEFIADLKGVSLE
EVSLQTTQNFFDLFTKAKAQAS

>gene_779|GeneMark.hmm|510_aa|-|7536|9068 >NVVL01000045.1 Rickettsiales bacterium isolate NORP64 Contig_source1382A_23472, whole genome shotgun sequence
MSSKNFYITTPIFYVNDVPHIGHAYTSLACDVVARFMRLEGLHVKFLTGTDEHGQKVEAS
ALKAGVAPQEFTDKNSARFRDMMGVLGVSNDDFIRTTEEKHKKSVLAFWKKLEENGAIYL
GKYEGWYSVRDEAYYAESELTEDGLAPTGATVEWVEEPSYFFALSKWQDKLLAYYEENPE
FIWPNSRRNEVISFVSGGLHDLSVSRSSFKWGIPVPGDPAHVIYVWVDALVNYISALGYP
SADGDFADYWPAAAHVVGKDILRFHAVYWPAFLMAAGLELPKSIISHGWWTNDGQKISKS
LGNVIDPFGLVDEFGLDQVRYFLMREVNFGNDGNFSKENLVLRSNSELSNKIGNLLQRTS
AFVYKNCDAKIPDVDEAYVTEVYETDLFKEILSIVSANAGYMEKFDINKVLSNIIHITEA
ANIYIDKEAPWALRKTDPEKMKKVLYLLLEVLRYIAIMMQPFTPDSAKKMLDQLNIPEDA
RMNANLTMEFALSAGASIKEPTPIFPRLER

>gene_780|GeneMark.hmm|39_aa|+|9123|9242 >NVVL01000045.1 Rickettsiales bacterium isolate NORP64 Contig_source1382A_23472, whole genome shotgun sequence
MVLFQVVVLEIWTGQPCLAALKTLDTLEAMLAGLKYRVL

>gene_781|GeneMark.hmm|256_aa|-|9263|10033 >NVVL01000045.1 Rickettsiales bacterium isolate NORP64 Contig_source1382A_23472, whole genome shotgun sequence
MEKINWYGAYSLTLREVKRFCRVYNQTILTPVVSSLIFLSVFVLAIGQNRPDISGIKFIN
FMGYGLIAMSIIQNAFANSSSSFVMSKVLGYISDILLPPLGSVEILIAFTMGAVLRGVAV
GIAVTLALAPFIEYSLHHPVLLVFYVLASCTLLGMLGILTGMISNSFDQFSAVTSYIITP
LSFLSGTFYSVKALPFIFQQINLLNPFFYIIDGFRYCITNHADGNITAGISFLIASNIIM
YVVLIRLIDKGWRIKA

>gene_782|GeneMark.hmm|446_aa|+|10148|11488 >NVVL01000045.1 Rickettsiales bacterium isolate NORP64 Contig_source1382A_23472, whole genome shotgun sequence
MLKKLFIKTYGCQMNVYDSIKMQEILAPFGFESTEDMNEADMVILNTCHIREKAAEKVYS
ELGRVKKKIKDARKTQGLSDMIIVVAGCVGQAEGDEIFRRAHYVDIVVGPQSYHTLPDLLA
KLARSEKRLIDLDFVEEAKFDNLPQNYQGQGASAFVSIQEGCDKFCTYCVVPYTRGAEFS
RNVEEIYRESMVVASKGAKEVHLLGQNVNAYHGKTHEGKRHSIADLIRHIATLSDIERIR
YTTSHPSDMTDDLIALHGLEPKLMPFLHLPVQSGSNKVLKGMNRKHSIEEYLSIIDKLRV

ARPDIVMSSDFIVGFPGESDEDFADTLDLVKKVQYGQCYSFKYSPRPGTPAATMEQIPEE
IKTERLAILQKELSRQQFEFNESCIGRILPVLFEKNGKFDNHIVGKTPYMQSAYIENADK
SLIGRVVDVKITKGLAISVTGELVKN

>gene_783|GeneMark.hmm|788_aa|+|11603|13969	>NVVL01000045.1 Rickettsiales bacterium isolate NORP64 Contig_source1382A_23472, whole genome shotgun sequence
MLLRLLIFCSKLALFFGAIGVVVAGYVIYYYSRDLPDYSQLKLYHPPSITRMYSSDGKLI
EEYAREHRVFVPITSIPKPLIEAFIAAEDKNFYHHSGVDFVSILRAAITNVSHILNKRRV
EGGSTITQQVVKTFLLSSERSLERKIKEAILSYMMSQLFTKDEVMELYLNQIYLGKGAYG
VALGALNYFNKSIEELTLNEAAVLASLPKAPSKFNPAVNYKRAFVRKNYVLGRMFDDGYI
TEEQAREAIAQPITLAKFNKVTTIDADYYAARVREEVINMFGKEYFYTAGLTIMTCVDSN
IQKHAARALRAGIKRYDTKRGYRGAIKNIDLINWQDDLRAMPIPIGLRHYKLAVVLGVTD
VAAKVGLRDGSLKTLRIRDMKWAKTGLASVRELLKSGDIIAVERKGKKYILQQIPEVNGG
IMVVDHRTGRVLAAEGGYDFGFSKFDRTTQANRQPGSLIKPFVYLAAIENGAHPNDIFDD
APIELEQGPGLPLWMPKNHDDKFLGKMTLRKGLEKSRNLVTVRVGQFAGINKVAETIKRF
GINDDPLVVQSIVLGSIETTLSKMTMAFGALANNGRKIEPHYIEFIKDRKGNVIYKRDYA
ECSECKSYKLDEDDNAIVPKIEITKGAALTDEASSYQMTSLMMGGVQRGTSRKAKNLKHV
IAGKTGTTNDARDVWFMGYTPKIIVGTYVGYDQPRTLGKRAYGANIALPIFVDFMKKGYK
NAPLVDFIVPDSVALLSVDYETGKPSTGENSIIESFKADDYNPFLEKIKRRYKPLIKIQE
RDSSEELY

>gene_784|GeneMark.hmm|306_aa|-|14166|15086	>NVVL01000045.1 Rickettsiales bacterium isolate NORP64 Contig_source1382A_23472, whole genome shotgun sequence
MAGLLSRLKTALGKTSSKIGSSLDHLFIKKKLDDAALEELEDMLIFADVGVQVSAEIISL
LRARKFDKEVEQDEIKQDLSVIIEQILERQDHQFELKQGLNVILVCGVNGNGKTTTIGKM
AASYASAGKKVAIAACDTFRAAAVGQIETWAERSGARLFSGADKADPASVAHRAISESIS
SGVDILFIDTAGRLHNHKNLMDELAKIIRVIKKVDDSAPHHSLLVIDGTTGQNASVQVKE
FKALADISGLVITKLDGTAKAGALVGIVQKFELPVHFIGIGEAADDLKKFVAKDFARALV
GLSEEK

>gene_785|GeneMark.hmm|922_aa|-|15090|17858	>NVVL01000045.1 Rickettsiales bacterium isolate NORP64 Contig_source1382A_23472, whole genome shotgun sequence
MNNARKILLFLSCLLWVQSVCAVLPIQMEVKVFGKDVIFVFYRDKDQVTDFHIKDRSVSS
KFSIPSEFSLLNLSEFNKYASNLRVFPSKQKISFRANKNLQYQSIINGEKLDAIKFRLKD
KVKTQEDDLSKIGSANNDPGTISYAKNKQNHVLLFNLGNKKSKIASFFRGKYLWVFFDQK
KIFSFKKNKFFSKFKMIPSEHGSAMRIKIDPKFAHARLVRSKLGWNISLSESAHKKTGAG
AGSGSIITPKSLQGEDGIIIKGDFENDELISCEDPELGDMIVALPLKQPGRKVAVQKDAV
EFSLLKSLQGIAVVLYSDDVLVEKYEDGLRISSDATLPDDMMIEADAQPLRADEYINLPT
ILPYLDKGLDILDFNKQKSRLIMEASLAKNKIEAFQHNLALARFLFVQELYHESLDAVRV
AQKYSEDEYKSNLQARFLKAVNYTMVGEYLAAKELYEELLSYYDVRKIAEISIWNNYNNF
SLAISFDKIGLLENLPKTIHLYSDDKYWGLVFAEVEIALLENDLKTVERVFKEIRKTGRG
KYANSLKFYKANYYRKKGQINLAIQYYTDLTFQEKDIFNKVRAEFDLAKLKVREEKITHA
DAIKILNRLRYEWRGDRLEYDILTQLAAYYREAQDIMNSLRTYHYIQTAFNNKVSNFYIT
SEMARIFNEVFLPGGLSKEMDDFTVVALFYEFKELNPIGEQGDDVILSIAKRLVHLDLLE
NAAELLRHQVSFRLKGEKRVKNADNLAIILMMDKKPSEAMLVLDNTDKDNFSFKEHEYRV
RLRAKGFIHMEKYEEALDCLNDDVSEDAEIIRREALFQGKNWSRYAERISSDFDGLISRV
GNDDFAAQDILRLAISCYMLNIHDQLVVISNAVGDNNKRLKNVVDLLISGSGSVDYKNLD

ESLDIDQMKMLLDKYKNQFLGK

>gene_786|GeneMark.hmm|56_aa|+|17939|18109 >NVVL01000045.1 Rickettsiales bacterium isolate NORP64 Contig_source1382A_23472, whole genome shotgun sequence
LIYRAAFASNYKIMVIKHLLEAAPVGKYTGFLKALCATWGTPTSGCAQSFQACFDK

>gene_787|GeneMark.hmm|528_aa|-|18305|19891 >NVVL01000045.1 Rickettsiales bacterium isolate NORP64 Contig_source1382A_23472, whole genome shotgun sequence
MLDKIRKGSDSLLVRVLLAIIAFSFVGGGAAVFMGGNSQGDVISFSDTKSISMEDFQAAR
TREIDALQRQNGINLTDENIEELGINSSILRKLINDSMIKYLARYYDFDISDEKIIDFVK
MTPFFKDKNGEFDRAAFKAAFNSPRKEEEYLASIHKHLVTSTLVEVFMSSFNPPKIMTEN
MVDYMAETRIVDLLSINLGSKPAGYKAPNLSDEQLEDFYKKNQDQFVVPELRSFDYIKVD
RKFLSKKLKISAKELRKYFNENKEEFSSKHYAKVKKEVKELVSKAKLDELSSELVKNLEE
DASGGFTLKEIAKKYALKVKNVRGKSFDELSSGKVKDHAELVDNIFEMSEGEVSSPIERS
DNNEILMVALNSIAAQRQQEFVEVRKEIKKFLLKRELAFANVKKLEAIQKTYSPKKINRK
KLKSNSITTLVNKSLTRADLPLEKKLPANLLKGIFGAQKNTVTALVGDGKKVYFAYVKKV
TRSNAKAKKIRKTSSSHFADTIKEGLFNELIVFMTKKNKMKITGASEE

>gene_788|GeneMark.hmm|237_aa|+|19970|20683 >NVVL01000045.1 Rickettsiales bacterium isolate NORP64 Contig_source1382A_23472, whole genome shotgun sequence
MKPIFIANWKMNLSLQEATTKAKHIEAQNYPTQFLLAIPATYLAYFTKTFKKITFCAQDV
SVVSGFGAYTGECSADMIKSAGVNYSVIGHSEKRELVPESNRIIRKKAENCTRAGIVPII
CIGESLESRQSGHFQEFLLEQLNESIPGNAQEIIIAYEPLWAIGSGIIPTIEEISAIFHF
IKSSKDVSMVAKKARLVYGGSVNSGNYREILNIPDASGVMLGSASLKETELDLILNL

>gene_789|GeneMark.hmm|106_aa|+|20704|21024 >NVVL01000045.1 Rickettsiales bacterium isolate NORP64 Contig_source1382A_23472, whole genome shotgun sequence
MINTLLFVHMIISALLIVVILLQKTSKDGLSGIGGGGNNMGVVSGRSAATFLTRTTIILA
VAFFANALVLANLSAKRQESISAKIQKTERNATKADSKKSSLPMAK

>gene_790|GeneMark.hmm|273_aa|+|21046|21867 >NVVL01000045.1 Rickettsiales bacterium isolate NORP64 Contig_source1382A_23472, whole genome shotgun sequence
MNIVKVEDISIANNLPFALVAGPCQIETLDHALFMAESIKKITDKLGIPFIYKSSFDKAN
RTSAAAHRGAGIDDGLEILAKVKSQIGCAILTDVHTETQCAPVAEVVDVLQIPAFLCRQT
DLLKAAAETGKVVKVKKGQFLAPWDMKNVYEKLVSFNAKGIILTDRGTSFGYNTLVSDMR
GLPVMAETGAPVFFDATHSVQQPGGIGASSGGQRQYVELLARCAISAGVAGLFIETHQDP
DNAPSDGPCMLRLDSLEGTLSRLQRFDELRKSM

>gene_791|GeneMark.hmm|947_aa|+|22189|25032 >NVVL01000045.1 Rickettsiales bacterium isolate NORP64 Contig_source1382A_23472, whole genome shotgun sequence
MFLGFGIDAIIVVGFLIATLVVGLGHGRSITTIKGYALGGRNFSTGALVATLVATWIGGS
GFTINLSKTYSSGFLYIVPSFGMTLSFLIMAYLFVPRMGEFLGVTSIAEAMGDLYGKHIR
IITAVTGVMSEAGFVAVQFKVFGNLASYFLDLSPMTGIVAAAAIITTYSAFGGIRAVTFT
DILQLFTFGFAIPIIGVMVWNHAYYDGVSLANSIQDNRFSLEFILSKQNPDLGSMIALMC
YFALPGLKPAIFQRISMGSSVKQVQKAFIISAVIVMFITFASQWIPFLLFNIDSTLEPSE
LLSYMIENYTYIGFKGLVIAGIIAMTMSTADSNINSSAVLFANDICKPLNVGQNRELLIS
KLFSIALGIGGIVLAVSTKDLLDVVLTANSFYMPVVTVPVMLTILGFRSSTKSVLIGMGA

GFGTVVIWRLLSIQADCIAFSMLVNLIFLIGSHYVLKQKGGWVGIKGQAALDALREEKME
EQQKRAQKLADDKGFVDACQKHCPKDEMSFVGLGIYLIIYTMTTIYSTTEALKHSNQMIL
IIYQIMMVSGVVLAMYPIWPRGVARKTRETIAQVLWPMIIAYMLVMFNLFFVMVSNFSPL
QFAVFTLNLVIVVVLLGWRIGGIAIVFGSLFAMPLYKEYGAETNFDVAIGSPAFVLIYIL
LLVGATIVLFLKPKQEKLEQTEEKVGVLSGEVTSLDNKVVGLNEKVVHYSERTSDQAKEI
ERLGATAQKILNNVNHELRLPVGNVMNFAEMMRDGLGKFSDKQLKMISEEVYENSNRLST
MILNMLDLATLNAKKIELKKVKTNFSELVQERVESCRKIYVGDKPLEFKLNIEPNVMAVI
DANYIRQTIDNLVINAISFSDKGVIIIDVVKQDGFASFTIQDQGLGIPKNEIYDIFTPFK
MGSNTESKAEGRGVGLALCKAAVSAHGGEVTAESNGKGAMFEVMLPL

>gene_792|GeneMark.hmm|129_aa|-|25021|25410 >NVVL01000045.1 Rickettsiales bacterium isolate NORP64 Contig_source1382A_23472, whole genome shotgun sequence
MGFLHLHTLPIKPTLLLENTDTSGVHTLLLKNAHMLGNLSYLTVRGILKKKLNATPSNWS
YSSGIISPPILSFSNEKTSKMATSLEIREMAIYQKNSRRQPSQQTVKEQYRSPILKPHPP
LPRAGKLKR

>gene_793|GeneMark.hmm|581_aa|-|25604|27349 >NVVL01000045.1 Rickettsiales bacterium isolate NORP64 Contig_source1382A_23472, whole genome shotgun sequence
MSKIFKLFSTIVGYRLSLKELYFSLTTWLAGLVAFLGMATKFDSSLLLANIVIAFCVLGM
IVLITNRVLLSCFLTLLLAVLFDYVKLIKWQFFMQELLAADVFMLKLLINHGLLRVIYEY
STKEIYLIFFLLLINFMLMWNRSDTLLKKKELGNKHYYIFRLISFALAALISLKMFDLAI
DKKSYVHSAIESMKSTQKDYQRRTYGPFADVLFTLQDVYITPNHGEVDEALILDEVKKNN
FARSDKLSEMPDIVVILNESVFDPTKLDYDFAEKFDFSFFKDTKNTKYSGILNVNTFGGS
SWISEYEINTGVPHKSFSGPSYMPFITLVPATNNSIMSHLRSIGYRVEVVYPVDKNFSLA
FDAYTKLGAHEISDIYEFGFKPKSWGNIPDTMIGDMIIDALDRNPGKPKYIYASTMLNHG
PHSGFFPDTIGCSRVMHDGLCSKLNDYVSRLNRTSVDQSALIEKLMKRKKKTIIVNFGDH
MPSFEGHSTHLRFKREIKDYYKTFYNINANFKIASREKYPSLDISFIPSLVLDMAKVNSN
DFYKASSLVRKKCKGQMSSCASEKDMLLESYKSLTVKQLDF

>gene_794|GeneMark.hmm|161_aa|-|27681|28166 >NVVL01000045.1 Rickettsiales bacterium isolate NORP64 Contig_source1382A_23472, whole genome shotgun sequence
LFYPNLKIDIIKNNIDSLVMFKSIVIPVDLSDKQSIKAVFPPALNFVNAFGAKIHLVHIM
PDFGMKVIEDYLPRHWVRDQKEKYNKLFDDMVKKFVPEEVEVKIYIGRGAIYDEVINYSE
KVNADLIILSAVRPQLKNYMLGPNASKIVRHSSISVLVVRD

>gene_795|GeneMark.hmm|304_aa|-|28141|29055 >NVVL01000045.1 Rickettsiales bacterium isolate NORP64 Contig_source1382A_23472, whole genome shotgun sequence
MLRTIIILLLSITIAPLHSAVAGQKIVRIGVGSLVEGYHSMGSQICRYISESNEGVKCEL
VPTSDSREALWLLHREKIDFAFTLSNLALQSYSGKGYFVTSKPFKDMRQLLRLHDEYFTV
IVRDGADVKRFADLAGMRLSNGPPKSDSSVIYKALDSYYDFKNPPIDIELAYEDYAKKFC
GGAIDAIMIMTGHPSSLVNYIAHTCRSNFITIESDKIDLLLRNNPGFRKVVLESGVYPNI
NESHESVAVSTIFVVGKSVDEKIVANFLERFKKEVSKFKATNPVLYDLKDSHFMSEFVLP
KFKN

>gene_796|GeneMark.hmm|404_aa|-|29065|30279 >NVVL01000045.1 Rickettsiales bacterium isolate NORP64 Contig_source1382A_23472, whole genome shotgun sequence
MALWHKVFIGLILGLVFGVYLPEHVDIVKPIGDIFMRMITMVIVPLLFFSLVSGITGMRD

PNSVGRVAKKSVLAYFGTTCFAVIFGLGVAHIMKPGEGVVLDLGTAAPSNFKENGFDILN
FLIEIIPSNVFVAFADSNILQVVFFSIFTGMIINSMGTPLDPVRDFIHSAAKLVLKMISI
VVELSPYAAFALTAWVVGTQGLDIMFSLGKLVGAIVVAYILQYLIFGLFIVVFCRLSPIP
FYKKSIEYQMIAISTGSSKASLATTMQVCRHKLGVSESSTSFVLPLGAAINMDGLAIKLG
LSAMFFAQILGVELALSDYMVIILVGTVGSIGGAGIPGSFIIMLPIMLTSVGLPIEGVAL
LVGVDRVLDLLSTTINITGDATITLVIDSTEGTLDRDMYNSDQV

>gene_797|GeneMark.hmm|106_aa|-|30327|30647 >NVVL01000045.1 Rickettsiales bacterium isolate NORP64 Contig_source1382A_23472, whole genome shotgun sequence
LILGSAKYCVILTTTDCEETAILIAKTLTENKLAACVQMDRVESFFVYEAKNERQKEFRL
LIKARSDSYQAIEESILSKHNYQLPQIIMLDVAAGLPEYLNWIDGK

>gene_798|GeneMark.hmm|138_aa|-|30644|31060 >NVVL01000045.1 Rickettsiales bacterium isolate NORP64 Contig_source1382A_23472, whole genome shotgun sequence
VIECTRRIEFDAGHRVIGHKHKCKYLHGHRYVLEVTARSEELDELGMVVDFGVLKDVVKG
WIDENFDHNVILHKEDKELGAFITNHTGQKIYYLNANPTAENIALHLKSDIIPMLFVENS
FSIVKIRLFETPNSFVEV

>gene_799|GeneMark.hmm|132_aa|-|31070|31468 >NVVL01000045.1 Rickettsiales bacterium isolate NORP64 Contig_source1382A_23472, whole genome shotgun sequence
MSNTILVKIILPAQMILETEADMVNIPGSDGMFGVLPGHAKLTSGIDIGVITLVSRDFEQ
RYFVYGGVAQVTGEELNIITEFAVDLDKTNKVNVLDNITNLKSDLSDEEVGSVEANIIAG
QIERYETLLKFI

>gene_800|GeneMark.hmm|478_aa|-|31472|32908 >NVVL01000045.1 Rickettsiales bacterium isolate NORP64 Contig_source1382A_23472, whole genome shotgun sequence
MTKNEGRITQIISAVVDVKFDGGVQLPNISHALECLNNKQRLVLEVAQHIGDSTVRCIAM
DSTDGLTRGIVVTDTGKPIQVPVGKGTLGRIMNVIGESIDELGPIKTKEFSSIYRDAPAF
DEQSTERSILVTGIKVVDLLAPYAKGGKIGLFGGAGVGKTVIIMELINNVAKAHGGYTAF
AGVGERTREGNDLYHEMIDSGVINPKNLEESKVALIYGQMNEPPGARAKVALTGLTVAEH
FRDLDGGQDVLFFVDNIFRFTQAGSEISATLGRIPSAVGYQPTLATDMGILQERITSTKT
GSITSVQAIYVPADDLTDPAPATSFTHLDATTVLSRQIAELGIYPAVDPLDSSSQMLDPD
VVGERHYKIAREVQKVLQTYKSLQDIIAILGLDELSEEDKLIVSRARKIQKFLSQPFHVA
EVFTGLPGKFVDLEDTISGFEGLVEGKYDHLPEAAFYMVGTIEEAVEKAAKLAEKESI

>gene_801|GeneMark.hmm|290_aa|-|33041|33913 >NVVL01000045.1 Rickettsiales bacterium isolate NORP64 Contig_source1382A_23472, whole genome shotgun sequence
MSNLKQLRNRVRSIKSTQKITKAMHVVSASKLKKLKEKAEGLNHYTSVLGDIMHDISTSG
NLMDLPICDRKFFSEDLKKMSHLLVIMTSERGLCGGFNSSVIRSAKRDIARFQKEGKQVK
LLVIGKKGCDAFSRKYADMIEGTYHNNKASSACVSLEVKNKIMEMVENEEIGACYMYYNL
FKNAMTQILTTEQVLPAHTADEAESGGVSEYEYEGENVVHNVIDLYIGGQINYGLLQSRA
SEECSRMTAMDNATRNAGDLIDSLTLKLNRSRQAIITTELTEIISGAEAV

>gene_802|GeneMark.hmm|513_aa|-|33920|35461 >NVVL01000045.1 Rickettsiales bacterium isolate NORP64 Contig_source1382A_23472, whole genome shotgun sequence
MALNPSEISEILEREIGDIDQLGELKEVGYVITVGDGIAKVYGIDNVKAGEMVEFSSGVK
GMVLNLETDMVGVVIMGNDSSVRQGDKVERTGEILQTPVGPAMLGRVVDALGRPIDGKGE

IVTKHRSQVEVKAPGIISRKSVHEPVQTGIKAIDALIPIGRGQRELIIGDRQTGKTAIAI
DTIINQKSAHLAGEDADKIFCVYVAIGQKRSTVAQIVKKLEEAGAMEYTVVVSATASDPA
PLQFLAPYTGCSIAEYFRDKGQHALIIYDDLSKHAVAYRQISLLLRRPPGREAYPGDVFY
LHSRLLERAAKLSDKEGGGSLTALPIIETQAGDVSAYIPTNVISITDGQIFLESELFYKG
IRPAVNVGISVSRVGSAAQIKAMKQVAGSMKLELAQFREMEAFSQFGSDLDASTQRLISH
GRKLTELLKQAQYSPFLVEDQVVSLYAGVNGYLDKIDTSQIRDFEKKLLADIKLKGEDIL
ASIRKEKKVNEETEEKLKAYLEKFVKVFLGNNS

>gene_803|GeneMark.hmm|183_aa|-|35515|36066 >NVVL01000045.1 Rickettsiales bacterium isolate NORP64 Contig_source1382A_23472, whole genome shotgun sequence
MLLDLKVVKNYAQALFLKAKDLSKEDKILEQISIISDITEASPLAKEVLCSPIIDHSLKI
KLIDLLAKKYKFDKISQRFLYVLVKNTRCNFISKIVDEFGKMLAESKGIKAVNVSSAFKL
GKKELSFIKGFLETELNKKIELTTKEDASLIGGVVIEYDSNLIDCSVHGALEKIQKVALK
SKV

>gene_804|GeneMark.hmm|420_aa|-|36172|37434 >NVVL01000045.1 Rickettsiales bacterium isolate NORP64 Contig_source1382A_23472, whole genome shotgun sequence
VKLKERSSYIIAFCATIVRYYDYALFGLSASLISKHFMPGKDNSEQMLVFFAIFSVAVLA
RPVGSIIFGKIADRVSRIASVKIATVMAIISTSLIAVIPSFELISWFSVAALIFCRMVFM
MSLAGEVDAIKVYVAEKIGNEKRHFGAGIVSFSSQIGVLLASVMYHIAISYEEIKWLWKM
NFVIGGILGLAVFMMRGYFSESKVFLKEATTGSRLDADEGIISIIWNNKLKFVLSSIVNG
MIGGSYHFLVVFLGAFAANVINIIPPEQAASNNSKLIALYGVACILSGYIADKVKVIAQV
SIALTASIICVLVMEFMVEMNVFVIELHYTLAFIVPFYTIPCAIKVQSLFPIGIRMRMFS
LSHSIGSMIFSSTTPFICMLLWKSTELPSIVLSYFLAQLGILFFVMIYIANKNYVSMFKT

>gene_805|GeneMark.hmm|210_aa|+|37529|38161 >NVVL01000045.1 Rickettsiales bacterium isolate NORP64 Contig_source1382A_23472, whole genome shotgun sequence
MIGKLKGKIDSIDDGYFIIDVGGVGYMVYCSGKTMSKLVPDEYAEILVETHVREDHIHLY
GFYDMDEKSMFNILQSVKGVGTRMALGILSHLAPVEIQLALSAQDKTMFLSVSGVGKKLA
DRIITELKDKFPSTGVISPAAMAASAARGGASQAHSSVGMANDAISALANLGISKIDAQN
RVQTIIASNPDISINELIRLALKTGASSTI

>gene_806|GeneMark.hmm|343_aa|+|38158|39189 >NVVL01000045.1 Rickettsiales bacterium isolate NORP64 Contig_source1382A_23472, whole genome shotgun sequence
MTAEILSGNELENDADSSIRPGYLQEFVGQEHIKSNLGIFIQAAKNREEALDHTLFYGPP
GLGKTSLAHIVAREMEVNFKATSGPTLSKAADLAAILTNLQENDVLFIDEIHRLHIAIEE
ILYSAMEDFSLDIIIGEGPAARSVRIDLPKFTLIGATTRMGLLSNPLRDRFGIPLRLNFY
TTDELVSVINRAAKLFKTNITDKGAQEIAKRSRGTPRIALRLLKRVRDFATVDNKSEIDE
QIADSALNRLEVDKAGLDSNDYRYLKFIYDNYNGGPVGVETIAAALSEQRDTIEESIEPY
LIQIGLLQRTSRGRMITNNAYQHLGLNSPQASGTGTQETLFDE

>gene_807|GeneMark.hmm|412_aa|+|39640|40878 >NVVL01000045.1 Rickettsiales bacterium isolate NORP64 Contig_source1382A_23472, whole genome shotgun sequence
MKGNSSNFIAFDIGSSKIAAIASNITKQGRIKVNSQILHHAEGFKSGMITHMELAENTIV
TAIYALEKECDKSIKEVAISLSGTGVKSYYINHKIKLGNQTISKQDIKKLINKALSDFKV
KDQEIIHYFPMEFVIDEGQIVDNPVGMYARSVSCHLHIISADSLMLMNLSKCLDKCHVRI
NDVTVAIYASALACLSADEKELGSIIVDIGSHTTSFGVFLNGQIVYVGHVPIGSMHITTD

IAKQFSISLHEADKLKILYGNANPDLISKDTTIRLDDISSSENYHADLSITTIELSKMIH
SRIEEIFLNIKDQYNHISMDNLISRRMVITGGASSMPGIKNLAAEIFQKQIRIAKPENLP
GFAEDNNPYAYSTVVGMLKAESSKYQKDSIRSYQDDSGGIFKRMFSWLKENV

>gene_808|GeneMark.hmm|330_aa|+|40857|41849 >NVVL01000045.1 Rickettsiales bacterium isolate NORP64 Contig_source1382A_23472, whole genome shotgun sequence
VAQRKRLVVLISLVALILYVALIYPINAHASVWIPKTNEYKYYTSFSIIDKRSENKKIKW
ADLFVKDQEMIHKLTKRQNAIKAKADDENRSLFNHEIAELNDNAAFLKEFKEYKMILGSL
GEDKTSNFELEYGINDHQSLGMKIGYKTDNFLRSITPHYTEQKTGKDFELFYKYQLLKQE
GWIVAISPSLQYSTYTGCKPYRHFDLALMAGKSRLHKNNYTLFYEFGASVHAHSKENSLS
KTGFTLFMTDGIRFANNVIITNYMKYEQNRFKNYAHNKILYEQISIAKEIYFGNLSKQSF
TIQVGYFAKGSLVARGYNVSGFIFSLWFSL

>gene_809|GeneMark.hmm|585_aa|+|41834|43591 >NVVL01000045.1 Rickettsiales bacterium isolate NORP64 Contig_source1382A_23472, whole genome shotgun sequence
MVQPIINAAKCFSSIANSNRSKPGGDDCIINSLTANDWPGGDHVDNESIIESLIKNRQMP
FIDTHTKDINILHFSKINEYCRQGYLEFQDANGNRHYAINNLSVIKQKGLHLSEGHKFHL
IRKRDLTQILEKHFSHKQSIWASNHLNSLNKDACAKGVNYIKMVTGFSVIFFAIFFLFSN
LFNIVNNLLYLSQNILKAALFKNGFRKTDKSLAKRTGPLPIYSVLIPLYKEDIKAKFILK
SIELLDYPKDKLDVKLIIEADDILTIRALAVLDIPSYVQLIKVPYSLPRTKPKALNYAMG
FARGEFVTIYDAEDRPDSDQLLKALYAFETLTDDYACVQAKLNFYNAKENILTRFFSLEY
RIWFEYFLKGLSFLNIPIPLGGTSNHFKIDKLKEVGYWDAYNVTEDADLGIRLYLRGYKV
HIIDSVTTEEAPVNIYDWIAQRSRWIKGYLQTICVFMKAKKDRKVFSLQDSLSVYVFVGL
STYSFLCLPWLFTSLLLDVDPYIHYLLILNGVLSLSYLYATAFLALMRERSNKAPSLLGA
ASLLLWPLYFILHTIASYRAIYEIVVSPFKWNKTPHGVSIDEIEE

>gene_810|GeneMark.hmm|82_aa|+|43621|43869 >NVVL01000045.1 Rickettsiales bacterium isolate NORP64 Contig_source1382A_23472, whole genome shotgun sequence
MRKWQLHNAKDKLDDLIEEALSGSPQCITKHGEEAVIVISMVEYEKLKNEESDLREFLVN
KGPKFNNLEIERPHGVVRSYEV

>gene_811|GeneMark.hmm|59_aa|+|44054|44233 >NVVL01000045.1 Rickettsiales bacterium isolate NORP64 Contig_source1382A_23472, whole genome shotgun sequence
MKKLLALLQTVILLFLIPGVPIARWCKVEEDKMLFRMTINIIYYLTIAVFALIENISDL

>gene_812|GeneMark.hmm|719_aa|+|44394|46553 >NVVL01000045.1 Rickettsiales bacterium isolate NORP64 Contig_source1382A_23472, whole genome shotgun sequence
MHHDSNITDLNPPQIEAVNHTEGALLVLAGAGTGKTKVLTCRIANIIEKNLAYPSGILAV
TFTNKAAREMQERVSSMINADGLNIGTFHSIASRIVRSHVELLDINLNKTFTIIDQDDQV
KLVKNIAIQKNVDVKQYAPKMIHAIISRWKDQALLPAKLSSADTPSPAHSIAKMIYREYQ
IQMQASNVMDFGDLLLYCNQLLIQNPDILEHYQNKFKYVLVDEYQDTNAVQYIWLRMLAN
KHKNICCVGDDDQSIYSWRGAEVKNILRFEHDFPNAKVIKLEQNYRSTSYILNAASSIIQ
NNKSRHGKKLWTEQKSGEKINIISCWNDKEEARFISTKVEEIIHSKKYNPSQIAILVRAG
FQTRAFEETFISNALPYQIIGGLRFYERMEIRDLLSYIRLCINHNDNLALERIINTPKRS
IGNVTLKKIKDYASEGEISVFDALQRMLAENIFKGKAAASLEQFVTIILSAAAKYESGTA
IEATKFILEESKYIPLLKEEKTDESRGRIENINEMLRAIDEFDNIYEFVEHSSLVMDNAS
LEEGFGGTVKIMTLHAAKGLEFDLVFLPGWEENIFPHQKALSEEGGKGLEEERRIAYVGI

TRAKKELYITHAESRRVFAEFVNSLPSRFLKEIPEEYCNDRSSTNRLNYVASKHNFSMQT
NKPKHKIPIDNSDKKAGSRVLHQKFGSGIVVRKDGDTLEIAFEKIGLKTIKESFVEAAK

>gene_813|GeneMark.hmm|441_aa|+|46728|48053 >NVVL01000045.1 Rickettsiales bacterium isolate NORP64 Contig_source1382A_23472, whole genome shotgun sequence
MGKNRLAAEATVTRPTKEQLSHLSFAQEVKQGVSAVGDLIERAKKSAGVSSEQSPDTLLE
LSTGIFKRAMILGGSAGVQIYSTNDEGERIDKPASENGLPITYALSHGSRTLIKIPARSG
DQVINWLTSGNPAISGMSNTQTTQKGALADGKAVYNRPAATHAIGFTNDEPRELKSMLIG
VRDFLSSKILGRTTKHYGVDLGLCMEGSAAPDGKNGHLYIHYEPPTEARPGAILFGCESH
APSSSQHSKVGNSNPVSPTGCSKFKVLKHKKTIAGETEYTNTIIPDKYHGMIGALDDQGV
KALITIEADKFGKSLALTMPQNSPSDFIAQYVNSPETEKNLRTLPELPTSAQKTPAPLAK
PNLFIRIANKVSFGLLFHDTMKEYKSEKKRYKIQSAIGAENTNSPPKKSLRKRPHPPLTP
PPPLTPSRTPPTPTQEASRIR

>gene_814|GeneMark.hmm|140_aa|+|48299|48721 >NVVL01000045.1 Rickettsiales bacterium isolate NORP64 Contig_source1382A_23472, whole genome shotgun sequence
MIIKYPCNITKDQDRFLVTFPDFPEALTEGLNLEEALFNAIEALTLTLEARLDEKIDIPT
PSLKKHQYWITPSVQVQSALLIRFARAEKTISDLARILETSWPAASKLENPHHWPTLRQL
DKTAAALGKKLVISMEEIVR

>gene_815|GeneMark.hmm|333_aa|-|48787|49788 >NVVL01000045.1 Rickettsiales bacterium isolate NORP64 Contig_source1382A_23472, whole genome shotgun sequence
MHAFCVRIFSEDNQFMSKIMSKIYQDAILKMMSHSCATRQDIYNVVLKSAQGGFLHNIMD
SYKKMNGNDAIFSTSRLFRKIFLENNLEVLKAAAEKDILSEVLAHHLNDEVIHAVFYNSK
MFAQVFISVSDEQLSRVLNGYDAWGLGLLLSEATKANVLKEVIEKIGGKFTPDVASSLLS
ATCLAEVSSVLEAFASCSINFSDAWNKLKLDAQKNVLTDLVESGALPTMIELSSELSIDW
NTFFGKMNSNEIEEFLLKVVENGLYSQLIGAMGDNYVMIVANLSASFWDSFPKATHTHLQ
VVIGACEAIAECCDFFPVDVQNELAGFCENLPE

>gene_816|GeneMark.hmm|420_aa|+|50112|51374 >NVVL01000045.1 Rickettsiales bacterium isolate NORP64 Contig_source1382A_23472, whole genome shotgun sequence
MESEEKNKVVSMLVWLCITLFYCYQLVLRMLPNAIMTDIMTKYSIGASEFGSFAGIYYIG
YIIVHIPVGMLLSRFGGKIVLPIAIACTALGLIPLVYFDSWTSVIIGRVLTGVGSSAAIV
GALQIFRILYPAKFSRMLGFMVFFGLITAVYAGVPISQIIQSIGMDYTVSILLYSGIVLA
FVTYVLMPKSTNTASESSMLGDIKAIIFNYRLFFTSIFAGLMVGPVEGFADAWGSAFLIN
VYGIEKTIANSITLSVLLGMCAGCIILPYIAEKTKLYFGVTIFSGLVMILGFVYILNGKA
SADSLYYACIIIGVFCAYQVVIIAKIASFVSSDRSGMAAAIANMIIMFFGWVFHNSIGMR
LDSLWDGEMLGDMKIYSADAFISSISIIPAAISIAIVGLVIIAMSNIVRVRLAQKVNSLK

>gene_817|GeneMark.hmm|455_aa|+|51383|52750 >NVVL01000045.1 Rickettsiales bacterium isolate NORP64 Contig_source1382A_23472, whole genome shotgun sequence
MMRLIACFIICILPLTIQARDGVGDAIEQIITKTDPSLNIGIKITNLDKNKVVFAKNAAR
YFIPASTLKFIASVAFLEHFGKDYQFTSRVLKKGSSYYLDIHDPDFKTEDLAHLVKELAE
YSGKKIKGNIYILNREFSVPPAMGDKTCSDITYCMGAPVTLVHINKNCSRLPVSAKRVGK
KISVKPDEDFPYHIKNNTKTIPKGERDRLRVDVKNGQYIISGTLCMGRGKVEIGGVVSDN
NLSNVKYHLKKQLAKAGIQLKGKILYKKTLPKGRLVSSVSKSLQDIVAIALKQSDNFIMD
YLLAELATQNNQHEWRLAVSSLKKLVHEKLGVNLQNSVIKDVSGLSRSNLIAINQFDSLL

KQIAKQPNFAEIKGFMAIPGEDGTLQERFNGMKKLYAKTGTLGNVSNIIGYFYDKNNELH
SFAIFSNNFYGRRAKYRMLQEDIIRLVAGDNSFLE

>gene_818|GeneMark.hmm|184_aa|+|52773|53327 >NVVL01000045.1 Rickettsiales bacterium isolate NORP64 Contig_source1382A_23472, whole genome shotgun sequence
MNISFKPLCESDFPLMLKWLEAPHVKQWWDLDIVWTLELVQRKYSSYANGYKNLKLNDQI
IKKPIYAFIIFAGDVPIGYMQYYNSHDFPPEQSYNCSEFLEHCAGLDWYIGEVEFIGKGI
ASQIFLLFLEQFVFCKFDHVLVDPDSQNTKAIHIYEKIGFTVIKKTINKNLLMIKTKNNC
SRIS

>gene_819|GeneMark.hmm|98_aa|-|53459|53755 >NVVL01000045.1 Rickettsiales bacterium isolate NORP64 Contig_source1382A_23472, whole genome shotgun sequence
MLKQIDKIGNNGEKVMKTIADGRREEGWKDGLAEGREEGISIGEERGEKIGVEVERKKTV
ARMLKENFAPKIISSITGMSQRAISKLRSQLKLQGELA

>gene_820|GeneMark.hmm|266_aa|+|65|865 >NVVL01000046.1 Rickettsiales bacterium isolate NORP64 Contig_source1382A_23537, whole genome shotgun sequence
MLKARTFLRTKAEKATLEAETATKAKTNFLAFTAHEIRSPLGFIITGSEMMAKGFTGKLS
KQHQEYAEGIHENAQQILEFLSDILDESQIIEGKFKIRNTQTQLEDVINEAIKVNLARYH
KKKINVVSKMEENLPLLICDRRRILQVLSNLISNSLKYSDDHTTITLTGKVVNNKMEVQI
IDEGIGMDEKGINIALSSYGTLEHGRHYSDGSYGLGLPIVKMLLNAHDATLSIKSEKSKG
TVVKIVFPKYKLVYNAPNLDKTKGQS

>gene_821|GeneMark.hmm|367_aa|+|869|1972 >NVVL01000046.1 Rickettsiales bacterium isolate NORP64 Contig_source1382A_23537, whole genome shotgun sequence
MDYNRERFLKKLRNIPLIIPLLIILLCVYGFVLLYSAAGGSFHPWAYKQITVFCIFMPIS
ILIAMMDLRLIYRFSYLFYIVTLLLLIIVELTGKSAKGATRWLDFGLFTVQPSELVKISV
VLMLAKYFHESDKYNTSEYAIIIPIIAAAIPIVLVIKEPDLGTGIITIIVAAAIFFAAGV
RMLYFIVGGFLTLSAMPIIWFMMHNYQRTRVLTFLDPARDPLGAGYNIIQSKIAIGSGGL
FGKGLLSGTQSHLNFLPEHQTDFIFSFLAEELGFVGGMVLLILYSLLIISSLAVSINCRS
KFAKLIAVGITTLFFAHVFINIAMVMGLLPAVGVPLPLISYGRTMLASILIGFGFVMNAA
VNQRSNV

>gene_822|GeneMark.hmm|166_aa|+|1980|2480 >NVVL01000046.1 Rickettsiales bacterium isolate NORP64 Contig_source1382A_23537, whole genome shotgun sequence
MLKSIYNFLLRALSALYLALLCLLPFQWHKIGSYSEIFPAFDLIIVFYLNTYKNLQHWHL
FIIGLIVDRFYFLPVGSSSLLFILANMGLKVIARWLLLQNYLTNFAIFCVYSIFIMLSRE
LIVTIKSTYYIEGFALYFYILTTIFAYPIICMLINKPIKILTTHAE

>gene_823|GeneMark.hmm|599_aa|+|2470|4269 >NVVL01000046.1 Rickettsiales bacterium isolate NORP64 Contig_source1382A_23537, whole genome shotgun sequence
MLNKDTINKQIMTRRTFVIGAGKLGLLFLLAGRMFSMQFIKKDEYKTLSDKNRIKVIIIP
PDRGQIFDVNENIIAKNNTCFHLFLDKNVNPRFSKEISLVTEILELDDVHIKEVQKRVRN
AGRRIAAVIIDCLNWKQVAIIEERRSEFKSLFVDTGFNRFYRSGTATAHIVGHLGKVRKN
TKNQSLKLVGENFRVGVNGIESYYEESLRGKFGNKRIEVNARGKYVRELGNMAPTPGKDL
HLNIDIALQKKALSYLSSQGCSAIVMDCTNGDVLISASAPSYNPNEFYKLSNKYWNSLIN

NPYKPLIDKTIRSLYPPGSIFKIITVLAALESGVDPADKIICTGGPVIKGSKYFRCARSR
GHGALNMIDAVKHSCNTYIYAIARQIGADKIMETAKKLGLGAPTGVDLPGELSGFIPSKS
WKKERYGTKWRTGDTLNLSIGQGDLLSTPMQLVRLIAAIASNGKLFTPRIVKGEGPPEYT
QIDISPEHLGILKTALYHTMNSKGGTGYANRINYKSMRMAGKTGTAQVQSKKHTHDNLSR
EDIAWKSRNHAIFSGYAPFDNPKYAISIYYDHGGGGSKSATPIAKKIMLDVLSKYANKL

>gene_824|GeneMark.hmm|280_aa|-|4329|5171 >NVVL01000046.1 Rickettsiales bacterium isolate NORP64 Contig_source1382A_23537, whole genome shotgun sequence
MQIRQILKVAIKELSNQRAPSLDARLLLCATMEYTHEELLLNYNQELTPAEEKEFMALVS
RRKSSEPLAYILGQQEFYGLELRIDRNVLIPRPETELLIDLVLNDPLPHNKTIKILDLGT
GSGAISIALAKHIQAASITATDISKQALEIAKINAEIHDVSGQINFRISDWYDQLGEAKY
DYIISNPPYIAKSEQDQMAKETLLFEPELALYAPEEGLAAYKSIINAASQHLKPGGRLLL
EIGYSQKNAIISILSANGFKNTRTHQDLAGLDRVIMAKMR

>gene_825|GeneMark.hmm|249_aa|-|5174|5923 >NVVL01000046.1 Rickettsiales bacterium isolate NORP64 Contig_source1382A_23537, whole genome shotgun sequence
MGSACSRYKITLEYLGANYCGWQRQKESLSLQQVVEDAIFAFSKEHVTLHVSGRTDAGVN
AYGQVAHFDLEKSHDLARLMHSINHFCRPHTIAIVDIELVDSEFHARFSAKTRHYVYKIL
NRNAVNVIDSGKKCWIRHDLDVEEMQKAAKYLLGHHDFSSFRASACQSKSPFKTLDKIQI
IKNGDDIEIYFSALSFLHHMVRNIVGSLLMVGKGHWRAEKIKDVLEAKDRKAAGFTAPSE
GLYFLKVDY

>gene_826|GeneMark.hmm|684_aa|-|5924|7978 >NVVL01000046.1 Rickettsiales bacterium isolate NORP64 Contig_source1382A_23537, whole genome shotgun sequence
MNITTRQAVQAASNAKKLDNLVSKAKKKGFVTYDEINKSISANKDLSVTDLEGAISKFSD
AGIDIIEDDDNEDIKLDINVNQELTVSSRSSASSDEETPSDNISVDNGSTDDPVRLYLRD
MGGVELLSRENEIVIAKRIDEGKKELMLNSLCESPLAMKRFIKWFEDLVNENILLRDLIDL
DANLVTDTGMIEENDLIEKEEQEELAEQQVVERQVAEEDLEPKDGLKAGAGKKSNPDSDA
DSDADSMEKEDTPSLSVTAMEAELLPSITDKMEEIAGICEELLQKSREYFASSASTEKKS
SLKNDKAYTKTIATLVEAVTSVHFSSKRVEEILTDVYTANKALVSKEMELLKLAEKHKVK
RPSFLKEYVGAHLNEEWLEKLHKKKEAAWRNFLREEKEAIDKFLANIREAEEAAGLNVPE
FKRVVNAIQKGERQASRAKKEMIEANLRLVISIAKKYSNRGLQFLDLIQEGNIGLMKAVD
KFEYSRGFKFSTYATWWIRQAITRSIADQARTIRIPVHMIETINKIIRTSRQMMTELGYE
PTPAEIAARLAMPIDRVRKVMKIAKEPVSLENPVGDEDGSYLGDFIEDKNAVLPNEAAFQ
TNLRETTTRILSTLTPREERVLRMRFGIGVHTDHTLEEVGQQFNVTRERIRQIEAKALRK
LKHPTRSKKMISFLGENTTKNTTW

>gene_827|GeneMark.hmm|620_aa|-|8016|9878 >NVVL01000046.1 Rickettsiales bacterium isolate NORP64 Contig_source1382A_23537, whole genome shotgun sequence
MQISLEFYNHLRELVPVSEIVRKRLLLTRKGKEYLGICPFHNEKTPSFTVNDIKRFYHCF
GCSAHGDVIRFVSEINGISYKDAAIKIAGENGIELPKVSKAQEKRYEESEQIYNILELAS
QFFASNLNNDITKYLDKRGVSAESIKDFSIGYAPGGKQLEEFFARKSIDLKDLLKAGLVG
KKDDGRIYQIFNKRIMFPIRNIYNKVVGFGGRVIGDSLPKYINSPETLVFKKSDTLYGEN
IATGHSYKDNYSIIVEGYMDVIALHQAGFKQAVASLGTSVTENHLQKLWRSGDEIISCLD
GDTAGMRAAARLINNALVHIAHNKSVSFIELPKGSDPDDLLKASGSKGFSDLLQRRTQLS
EMIWKNEYAGKSFNSAESRSALEQKLEDYSAKIEDRTLKANFRRYFKDMIWNNLVRKKKS
NYNNNHGNNGNYSNNNSYGNNNGYNYNNNNKTISKELLASKEYSEMEFLEYSICAYMLKF

PEIIKKVHEDETLPNLMLENRELDEFKNWIFDIITREEGSADQLIRENVENTGFYDTYLV
LSASDTLFLDSLSLNKSNIDQEKVFEWLYKKHYLLLLKKEYMNTLNSKASEEQSKSSSYM
EEIQAISKHLAQLSDDFINN

>gene_828|GeneMark.hmm|204_aa|-|9882|10496 >NVVL01000046.1 Rickettsiales bacterium isolate NORP64 Contig_source1382A_23537, whole genome shotgun sequence
MNNTAFNFEIRRKAFHICAIAMPILYLLTSKIIMGIILTIIAGTTLYIDISRHYDKKIKG
VIDKFLGQFLRFSEKTGSFALSGASYMAFGFLITCLFFSQGLAITSWLILIISDSFAALI
GMKFGSPVINGKSYIGSAAFFLSAIFISILSYFTIGYTTSFMIIIISSFFATLVELFSKQ
IGIDDNFSIPVTYALSTFILGLML

>gene_829|GeneMark.hmm|162_aa|+|10578|11066 >NVVL01000046.1 Rickettsiales bacterium isolate NORP64 Contig_source1382A_23537, whole genome shotgun sequence
MEKFPITQKGYKKLEAEIKQLKYEERPSIIEAIATAREFGDLSENAEYHAAREKQSFVEG
RILDLGGKFSRAEIIDTSKLSTDSVKFGATVKLIDDKTEEECTYHVVGEYEADITKKMIS
VRSPLARAIIAKSVGDVVEVSTPKGTKTYEILEITFQELDLD

>gene_830|GeneMark.hmm|298_aa|+|11150|12046 >NVVL01000046.1 Rickettsiales bacterium isolate NORP64 Contig_source1382A_23537, whole genome shotgun sequence
MTTTTNLPVVTSASGFSGYLQEINKIPSLSKEEEFMLAKAYLEKHDLEAAHKLVTSHLKL
VAKIAIKYKNYGLPVTELVSEGNLGLMQAVKKYNPDLGHRLSTYAMWWIKASIQEYVLKS
WSLVKIGTTAAQKKLFFSLGKVKHKITNMYARSVNEGDYPEIANELGVTVKDVTEMSQRL
SGADLSLNRPVNRGDDSSSEMIELLPEARPSHEDTLSQSQSLTNQKDVLSRAMCVLNDRE
VKILTARKLLDSPSTLDTLSNEYNISKERVRQIENRAFEKVQNYVLGHMTEDGQVINC

>gene_831|GeneMark.hmm|271_aa|+|12106|12921 >NVVL01000046.1 Rickettsiales bacterium isolate NORP64 Contig_source1382A_23537, whole genome shotgun sequence
MTDRIPFVKMHGLGNDFVIILEDALPSNWEDSAFIPSISSRIIGIGCDQFITYKQTDSAR
FAMSIYNQDGGVALACGNASRCLSRLIFDETGLRNITLTVAGGRVVLCEYISKDAIKVNM
GVASFSESWMPESSDLWNLVEHYPIEPKEMICVDVANPHLVIFSQLSDQDKKLIGKNFQK
TDLFKGGVNVSFARVEDDKIHLKVWERGTGFTYACGSGAIATFAAAVKLGFAKDEAEIIF
KLGSLKMKTIDGMITICGSASYVFQGNYFYE

>gene_832|GeneMark.hmm|422_aa|+|12890|14158 >NVVL01000046.1 Rickettsiales bacterium isolate NORP64 Contig_source1382A_23537, whole genome shotgun sequence
MFFKGIISMNNSQEVVTFGCRLNIYESEIIKDNLAKSGTENVVVFNTCAVTKQAEKQARQ
AIRKLRKQDPDKKIIVTGCAAQNNPAMFASMPEVDKVIGNEEKLSDKYYDLNTERVIVND
IMSVKETANHMVKSFDGRARAFIQVQNGCDHRCTFCMIPFGRGNSRSVPIGVITQQVRAL
VDDGYKEIVLTGVDVTAYGADLPGTPTFAQMIKRLLSLVPDLARLRLSSIDVAEIDDDLF
DLMAHNTRLMPHFHISLQSGDNMILKRMKRRHNRDQVIEFCHKLRKIRPEVSFGADIIAG
FPTETDEMFENTRRLISEAAIQYLHVFPYSEREGTPAARMPQVPMEIRKKRAAILRAEGE
VELKKFFNQNIGRNVALLVEKNNIAHSESFIPVKLEGNFDSGQLVSGKLVEFDNEYMRAI
VA

>gene_833|GeneMark.hmm|316_aa|+|14164|15114 >NVVL01000046.1 Rickettsiales bacterium isolate NORP64 Contig_source1382A_23537, whole genome shotgun sequence
MDKLDKISIIGAGSWGTALGCLAARSAREVMIYSNDPAITYEINSSCRNTKYFNDTELHE

NLIATSQIKDTLKADIIILAIPSYAFSEILELLKSHGLKPSTILLIATKGLSENPVQFLS
DKIESEFENPYAFISGPNMALEVVRNKFTAMTISSKDLSLAAKLADVFKSDMLDVTISDD
IITIQIASIVKNIIAIRSGIMKSAGDGENAKAWLISTALAEISIISKALGGKPEANIALP
AVIGDLVLTCYSDISRNTRFGYEFHTHNYSKEFLTTYPLLVEGISSARLLGSLIKTYDLE
LPLISSIIDLLHVNYS

>gene_834|GeneMark.hmm|305_aa|-|15203|16120 >NVVL01000046.1 Rickettsiales bacterium isolate NORP64 Contig_source1382A_23537, whole genome shotgun sequence
MDSQFLSWGIFAVIIIIILALDLGVLNKNDHVIELRESLALTAFYIFVACLFGVYVFFKL
GAASAGDYFTGYLLEKAMSLDNIFVISMIFSFFKIPLKYQHRVLFWGILGVIMLRAIMIG
LGASLLARFEWILFVFAVFLIITGVKTFTASAENAIDVKNMYIYKLLASKLNVHPELAGN
KFIIKKQGKLFVTPLFMALITVEIMDVIFAIDSIPAIFAVTQDSYIVYTSNIFAILGLRA
LFFLLSDIINRFKYMKYSLAVILILIGVKIFVAHFIEIPNYIPLLVTITLLALGMIASNS
KKLDT

>gene_835|GeneMark.hmm|183_aa|-|16216|16767 >NVVL01000046.1 Rickettsiales bacterium isolate NORP64 Contig_source1382A_23537, whole genome shotgun sequence
LENMSNQKHIDVTTLHKWLDSKEAILVDVREIVEFSDEKIPGSINVPLGTVSAKNTLLSK
YKDKKIVISCRSGARSLSACTKILQDVPSAGLYHLDGGILAWKERGFKTIRKTEILPLER
QTQIALGGMIVLGNLLGYFVSFEWNLLSIFVGLGLINSGATGWCGLMKLIACLPWNRQSD
QQK

>gene_836|GeneMark.hmm|394_aa|-|16978|18162 >NVVL01000046.1 Rickettsiales bacterium isolate NORP64 Contig_source1382A_23537, whole genome shotgun sequence
MYKYKRYLELLIQQGQSIFLWGARKTGKSTYLKNLYSSSLYIDLLKTDLLVRYMKQPSLL
REEILALPDDKLELPIIIDEVQKVPALLDEVHWLIENTNATFVLCGSSARKLKQQGTNLL
GGRAIKYHFYPLVYPEYKKDFDLLKIFNHGLIPSHFMSPNPRKLLQAYVEDYLTNEIRSE
GYVRNIPAFSRFLDSTIFSDGEMLNYNNIARDVGIDAKTVKEYYQILVDTLVGYLIYPYK
KKMNRDIISHTPKFYYFDVGVATRIGKKHFENLAGAEAGRALEHFILMELMAFLNLTDSD
HKLYYWRTRTKLEVDFILSDNVSKPIPIEIKISKLVHKTEFKPMKAFMKEHEVNRGYVVC
LEETSRKIKLDEDKEIIILPVEQFLANLWEKKLI

>gene_837|GeneMark.hmm|46_aa|+|18433|18573 >NVVL01000046.1 Rickettsiales bacterium isolate NORP64 Contig_source1382A_23537, whole genome shotgun sequence
VPLEIISKRLGHSSIQTTMRYAHLADEQICNATNNLSEAFGKAMEI

>gene_838|GeneMark.hmm|344_aa|-|18608|19642 >NVVL01000046.1 Rickettsiales bacterium isolate NORP64 Contig_source1382A_23537, whole genome shotgun sequence
MKFHLHNSLNFIQLFVLLNIIFCCTNCLARNHTLEEYNTIKQAKHNSNNASKENFVPASC
SIVKATNGYPLLKNFNKVPNNSLYKYFNDISKKIPLGDFMLGYLHESGAGKKADGARAMK
YYNKALKRGSVDAMYLIALKKHSSITDHMVFGKTTFAFAKKEYKESLKLFLKAAKLNHDF
AQIMVATMYINGEIEYNNYKVAEKYIKKAIKTNEIDGYTALGAMYRDMGNFNKGTKYLEL
ASNKGSVVASIILFQDHLCGTGKDIDAILANKYKKLAIDNAKAQSVSIQDIDFIFVDKCK
KTFDLDKKQVFLYTSKDVLNDFLSKNRYDVGIIPSVFDSIIKNH

>gene_839|GeneMark.hmm|407_aa|-|20228|21451 >NVVL01000046.1 Rickettsiales bacterium isolate NORP64 Contig_source1382A_23537, whole genome shotgun sequence

MKQIGQIPQSLLYCLISLSVLTEAIYSVALPNIAEQLNTDGGIAQLSTTAYHLGFTVGIF
TLGRCSDIYGRRPVILFGISFYIIATYLITLSSNIETFIAFRFLQAYGASVGSVVAQAMA
RDSYKGWELTYIYASVALVMSIVPSFGSAVGGYIVEYSEEWEDVFRFLIVVSSILLLIYA
KFLPETNAYIGSGRSNRFSAVLRVALRDKLLLSSAFIVGAYNGIMFSFYIQMPFIFIERM
KMPPSEYGQLFLALTVANMAGGLFSRYLVKRFVSNSRMKIMGLTFSCIACLGLLGSTLWI
SETSSITFTAMMIFMPMSIHLMGHALIVPMMLKNALKDYYKVVGTAGAIFGALYYLVTAL
VSLMISLLHSNTINNYAYIYATLMVMCIGLLFMTFKWQNNATEPTFN

>gene_840|GeneMark.hmm|363_aa|-|21474|22565 >NVVL01000046.1 Rickettsiales bacterium isolate NORP64 Contig_source1382A_23537, whole genome shotgun sequence
MTLKCGIVGLPNVGKSTLFNALTASANAEAANYPFCTIEPNTGIVSVPDSRLANLAENAG
SAKIIPAFIEFVDIAGLVKGASKGEGLGNKFLSHIREVDAIIHVLRCFEDVDVTHVHDKI
DPIEDAEVIETELIIADLESVEKRVSGLTKKAKSDKALLEELELLKECQKILEQGKPIRS
MQNVDQKKLAGLQLITSKPVLYVCNVLESEATSGNAHSKNVEAMAKKEGANHVVISSKIE
AEISNLESEEEKQEFLGSIGLSETGLSKIIKSAYSLLDLMSFFTIGPKEAHAWTFEAGAL
APQAAGIIHTDFEKGFIRAEIISCMEYLEIGSEAKAKELGKMRVEGKDYVMQDGDVVHFR
FNV

>gene_841|GeneMark.hmm|33_aa|+|22647|22748 >NVVL01000046.1 Rickettsiales bacterium isolate NORP64 Contig_source1382A_23537, whole genome shotgun sequence
MVFLTKGRGVLISVQAILTLILENERVLIGLSI

>gene_842|GeneMark.hmm|504_aa|-|22812|24326 >NVVL01000046.1 Rickettsiales bacterium isolate NORP64 Contig_source1382A_23537, whole genome shotgun sequence
MGRFNKTKGKTRAEARAAAKKQKAEEQLRAALKTMNEQFSLNNDDASREEHFNAAKDLMQ
RCMTNLDLLVPRALANFVAVRAGLFEYCNNNPSNNMMDYSPLSLATNEDLSPELRTTLLH
KTGRYYFKRMKCSEDPIEQETNLQHSVTAFHQVIKLEGELGLNEDFSLAQIKIPNINKIL
AESFFILGCNYIEEARQNDHASKTETLETARDHLKRSLDIYKLTHSISTQIFDIHYNLGK
ALYHLAVAKQDTDDGLLTQAENHLREALEFYVRSSHTKNITRTLIYLKKISIALNDGENE
SPEKTIYGELLDEIEATQEVSSDFRGRFNEWKATQNAANKSQCIEGYATTAPALPLSDAE
EVKGALPEVPGLLSSEISFPTEYASSDDELDWDSDLSLWNEARKISDDLTPTISPELSGP
ETEADGAWEELQRPDLDPEDDTDSEPDSSGWSPNKASAIGSPSVDTGWGAWFFGSSNAPS
IAPAPELKNAGETTPLACDSSDLP

>gene_843|GeneMark.hmm|327_aa|-|24521|25504 >NVVL01000046.1 Rickettsiales bacterium isolate NORP64 Contig_source1382A_23537, whole genome shotgun sequence
MKFYSSGLADLIRKIEKGDVKSLLIHGVNHGFATTAIEQITKKLGLSVTNLTYKEVSAGK
LSLIANNQNFFGQKELIKISDTTTAINKEMKTLITDSQFYHFICFVSNDSLPASGIRKFF
EDKPNLASLGCYYDNEQTIAKMIVQQCKKRDKSIDEEALFYLKSHLKGDQQIIKSELQKL
FYYTHDKPNITKDDILKTLSPDLLASGDEMCIFFAQKEPMRFLNEIEKLKSQGKNEVLMI
RALIRYYLNIYNVAARIEDGSNIDIATRSLTPPIFFKYVDSFKRAARKTSSSDAIKILAA
LQDAEVSFKTNPSSFDLFETYIRVHGR

>gene_844|GeneMark.hmm|176_aa|-|25505|26035 >NVVL01000046.1 Rickettsiales bacterium isolate NORP64 Contig_source1382A_23537, whole genome shotgun sequence
MNMLVLGEILTKYRSIFLVFTLLATLTSCGFHPVLSSGGDACTRELLGQIELSSMQSIEG
ADFYNRLKNILPYGREKKYLLKTTLSFSKDFSIIQKNSDILREMVAARVSYSLRDKETGK

IRISGSFSRLSSFNTTFSPYSNQVQQYNVQKNLAIMSAEEVRNRIMLFLERENKEK

>gene_845|GeneMark.hmm|716_aa|-|26151|28301 >NVVL01000046.1 Rickettsiales bacterium isolate NORP64 Contig_source1382A_23537, whole genome shotgun sequence
MKEKIIIDANFPTEIRVARLDKHNNIEDIEFSSSNKTQVKGNIYLAKITRVEPSLQAAFV
DYGGGKSGFLPFSEIHSDYYHSFSTEKKSSNLVGEELKSPEITSEDLAEKPGGTKFNELA
DSDAIDIKAIEKMVDEKLQSDINIEASEDDVDAFSNDSAEQNKQYKIQEVIRKGQVLLVQ
VTKEERGNKGASMTTYISLAGKYCVLMPNKPSHNGISRKVSNRDERRRIKNVISSITELE
DSKTSSVIARTAAAGHTTLDIKRDYDYLVKLWNKIREITLKSQAPCFVHQEDGIILKTIR
DMFNRNVKEILVQGSEAYNSCIKFMGDIMPTHVRSVKEYKNKTPIFTKFGIEDQLIKLYQ
PVVHLPSGGYIVINPTEALVSIDVNSGRATSERNIEEMALKTNLEAAKEIAKQARIRDLS
GLLVLDFIDLNEARNRKILERSLWEYFGKDKARIQTSHLSAFGLLEMSRQRLRPSFLERN
SDMCSHCSGKGLVRSDDSNAMLILRTVENEIHNGKFDIVNVYGAASAVIFLLNSRREEIS
FIEKKYSIKLDLFIDRDATSDSYSIEKIKLSDKNKADTPEELQPILQDTSEIYNQNSSQQ
KTKRKIEKAKRPQEKKKPKAPEETASSNSTSSNNNSNTSNTNNSNKEAENQTQDKTQNQT
QDKNETPQAKEEQTAKPARSKKRIPRKPRRGREQNKPAPTVKAEADKTPQGQDETT

>gene_846|GeneMark.hmm|392_aa|+|28553|29731 >NVVL01000046.1 Rickettsiales bacterium isolate NORP64 Contig_source1382A_23537, whole genome shotgun sequence
MMRKRTILAFFAYSFCVVAYFVPIFANSIFIEGYLSRQSINFIEHDQIDAMHIYGYVVAT
ILLLVFFRYVAFKKLITFSVFLYILSTFAIVLAPLNEGVSWSWFFLHSASLIITPILMLW
YILCDDKINNNYSIAIYFSSILTAYLVVELVGYFIIYNQADFPLEGMVVSNLIPFGVFGS
ILGLSAVYEARIDGDDEQFYSIMRNMEIESLVGFTVFYVLILMLRGYDLYALINHLVILE
VRDIQFIMFIAMVIAIGVTIFLPFQVGEQKFNRHKLSISCIALMMILFTTLPYWGLYYIT
DIVCWFILGVLFYLLFSFHLLILSEKFDGINLFTALAIYALICCFGCYSGYLVGASSENT
IGENGFLISISFVLLGLLLYYMYFSKKHKLYK

>gene_847|GeneMark.hmm|444_aa|+|29731|31065 >NVVL01000046.1 Rickettsiales bacterium isolate NORP64 Contig_source1382A_23537, whole genome shotgun sequence
MTKLNIIGGGLAGAEAAWQAANRGVEVNLYEMRTASRKTPAHQTDKIAELVCSNSFRNDD
PASAIGLLHAEMRLMNSLIMKAADMTKVPAGSALAMDRDLFADTVSKELHDHPKINIIRE
EVQDISSEGHWIIATGPLTSDALSEQIQKLTGEDQLAFFDAIAPIVYKESIDFDIAWFQS
RYDKGDEVEGGGKDYINCPLNKAQYHNFIEALLASETTEFKEWEKDTPYFNGCLPIEVMA
ERGVETLRFGPMKPVGLTNQHDPQNKPYAVLQLRQDNKLGTLYNMVGFQTKLKYGSQKEI
FSCIPGLEKAEFARLGGLHRNTFINSPKLLGSKLELKCLPNVRFAGQITGVEGYVESAAM
GLLAGMFASCEINGKSVQIPPADTAIGSLLAHLTNGADSSTFQPMNINFGLVPQLEERIR
SKKERYTALSDRALASLQEWRKGG

>gene_848|GeneMark.hmm|610_aa|+|31090|32922 >NVVL01000046.1 Rickettsiales bacterium isolate NORP64 Contig_source1382A_23537, whole genome shotgun sequence
MDKISNDELLENLGKASAYYQEILQYILNNKSAEASFKYIDNKRNQEMVAKMAEQFLEHP
DKFINLNMEYVSNFQNLISDSLSKFTGSDKQEQSSQGSATDEKGAQEKASQTKDKYDKRF
QDPAWTENVYFDFIKQYYLITSDWMRKNVKQYDLDVNSKRYIEFATQHFIDALSPSNFAF
TNPEVLKESLDSRLENIVNGMSNFLKDIKKQGNLFDISTTDRSFFRVGKNLAATSGKVIY
QNDLIQLICYEPKEKTRSIPVFIVPPWINKYYILDLSEKNSMVKWLVDNNFQVFLVSWVN
PKKKLSGKDFEDYLKEGILDPYEQIKKLGFEKINAVGYCIGGTLLAAALSYLKENNKQYI
NSATFLTTLLDFANPGEIGALINQDSLAEIEKTVNAKGYLDGKYLSNSFSLIRANDLVWS

FFVNNYLLGKAPTAFDILYWNADSSNLPAKMYIYYLRNMYIENNLVKPNALEMLGTKIDL
SKIDTPSFWLAAQADHIALWKSVYDSHQHIGGEKIFCLTEAGHVAGVVNPEGNKKYSYML
GKDVGKDGVQNNWKDAEEWQKNATNHKGSWWKSWKKWLTQNSGKLVSSIDYEKLDSIQKA
PGNYVKKYNH

>gene_849|GeneMark.hmm|415_aa|+|33004|34251 >NVVL01000046.1 Rickettsiales bacterium isolate NORP64 Contig_source1382A_23537, whole genome shotgun sequence
MHHFTPDTSYLYAAVESNRSQMSPARFGLMMAHQWLEHSNNPLSNTHFGSTLKASIDILE
RVTRKYSKPEFGIKECVVNGNEEKIRQTTISKDTFCNLLHFSKPNFQKKQPKLLIVAPMS
GHHATLLRGTVRDTLPFFDVYITDWIDASQIPITDGSFDLDDFIDYIINYTKLLGPDLHI
LAVCQPTVPVLAATAIMSADKDLHVPKSMILIGGPIDGRKNPTKPNHLAMEKDLAWFDQA
LVTNVPPNYPGYRRRVYPGFLQLSSFMMMNMKKHVDSHVDLFKNLIIEDDEKTAKQKEFY
DEYLSVMDLPAEFYLQTIKEVFHDFSLAKGKFVSRGRKVDLTAITKCALMGIEGEHDDIA
AVGQTKAALKLCKNIPDKKKKYHLQKGVGHYGVFNGSKFRKMIVPEIKDFVYAND

>gene_850|GeneMark.hmm|245_aa|+|34335|35072 >NVVL01000046.1 Rickettsiales bacterium isolate NORP64 Contig_source1382A_23537, whole genome shotgun sequence
MSATEKAQQVVSTASSGADLSIISLVSSSDFVGKLVILTLVLASVWSWAIIASKLTHYSA
LKKRIRAFETLFWSGQILDELYERVKQSVDNPLSAVFVGAIKECKKSDASSSATRSSAAL
IGHKERVLGAMYLARNRELETLDEKLGFLATIGSSAPFIGLFGTVWGIMHSFQSIASSKN
TSLEVVAPGIAEALLATAIGLFVAIPAVMFYNYLSSEVDSINNKIDDFIGELNALISRAI
DEEKM

>gene_851|GeneMark.hmm|147_aa|+|35075|35518 >NVVL01000046.1 Rickettsiales bacterium isolate NORP64 Contig_source1382A_23537, whole genome shotgun sequence
MAAHLNQRGGGNIPGNSRRRRKPMSEINVTPMVDVMLVLLIIFMITSPMLVAGIDVDLPK
TDSAAISGQYKPLVISVNRKGELFLFETKLPKEKLEQKLKDVTKEKRDVKIFVKGDKNVS
YGTIIDVMSRIYNAGFTKVALISDIKH

>gene_852|GeneMark.hmm|287_aa|+|35518|36381 >NVVL01000046.1 Rickettsiales bacterium isolate NORP64 Contig_source1382A_23537, whole genome shotgun sequence
MRNPENNFFEKAVIFSVLVHIIVIGLFFFGLPSAFERLDNEKDIMTFEVLPMSAITNVKN
ERTIQKKAKIAKKSKQIKNSKTAAPPKPKKSAQKPKIKNKPKNIPAKKKKIKVVEKQKEM
KQAPPKQKAAPERYKLKEDVIDSILKNLESESEGDSSKTPVKSFSARDKGSKYARGMEYD
EDSPLSITENLLVKSQFEKHWRLPVGAINLQDVRVMMRIKVEKDGSITGIEIKDVVCPPN
SEATCKLVTESAVRAVKQASPLENLLPERYNTWKEFDLNFDPSLMAQ

>gene_853|GeneMark.hmm|439_aa|+|36403|37722 >NVVL01000046.1 Rickettsiales bacterium isolate NORP64 Contig_source1382A_23537, whole genome shotgun sequence
MKNFALFLICFFSLNAAAVSSVNVTKGHSDPIPLAINNFAGVDPYDVKLAKDIVKVISND
LKGSGMFRPISPAAFIEKKIGTRHKPLFAAWQQINASLLLNGDVSKLRSGKLQVNFILWD
SILEASMMSESFELPEHLWRQVAHKISDRVYKKITGYAGYFNSKIAYVSETGPYLKRIKR
LAIMDQDGENHRYLTDGKDLVLTPRFSPDAKKILYLSYKNELPQVHMLDLRTGRSSLIGN
FPGMSFAPKFSPDGKKALLSIAKDGSTHIYEINLRNKRMKQLTKGISINTSPSYSPDGKK
IAFNSDRLGMRQIFTMNRDGSNKQRISFGGGNYAEPNWSSRNYIAFTKITRDFGFTIGVM
SPNPHESQNRERLITRGYLVESPAWADNGRVLVFTKGMPPRGKRTKGLNRIYTIDFTGYN
ERIIPTPHDASGPDWSKNL

>gene_854|GeneMark.hmm|362_aa|+|38041|39129 >NVVL01000046.1 Rickettsiales bacterium isolate NORP64 Contig_source1382A_23537, whole genome shotgun sequence
MINNQTITLYLAKTFFRQFLVTSVVVLCVLFITNAFDTLQKFKASNLSSIEFWQFTSYKI
PYFFNKISSLVCFISTLMFLRTIVQCNELIIILNSGIPLWRVFIIPIIMAFLIGIVVLFV
SSPIGTHGLQEYKKLEAKLDGRENMNFTLTKSGIFFFENFDGVKRVIQTRSITPNKKILS
DVTIFLIDSENNLTQRIDAKKAILENGVFKLKSLTLISRKRSETLEELELPSSLSIENIM
QRFGSPDMIHLWNMNNVIDKFSSSGLVVTTYQIHLYKQIFNPLFMATMTFVACWFVGLNT
RSRSNAKMHVLGLTLGVCVYFFLEIVLRVLAYSAVPPIFATLLPMLFIILVSNFVILHFQ
EG

>gene_855|GeneMark.hmm|347_aa|+|39184|40227 >NVVL01000046.1 Rickettsiales bacterium isolate NORP64 Contig_source1382A_23537, whole genome shotgun sequence
MKLLSFLDRFSSDIAIDLGTANTLVYEKNKGIVLDEPSVVALINENGIFKPYAFGQEAKN
MRGRTPTDIEAIRPLRDGVIADFKGAEEMIKHFIRSVKDKRSFTGPLIIICVPSGSTPVE
RRAIQEAAESAGARDVYLIEEPMAAAIGAGLPVTEPTGSMIVDVGGGTTEVAVLSLGGIV
YARSVRVGGDKMDEAIISYIRRNFNLLIGESTAEKIKKQIGAAAVLEGEPLKEMEIKGRD
LINGIPKEMTLNEKQIAESLAEPVSKIVEEVKNALECTPPELSSDIVDKGIVMTGGGALL
KNFDHVLREATELPVFAAENPLACVVLGIGQVLEDFPKLKHVLFKQD

>gene_856|GeneMark.hmm|279_aa|+|40233|41072 >NVVL01000046.1 Rickettsiales bacterium isolate NORP64 Contig_source1382A_23537, whole genome shotgun sequence
VAILINRVKIRNNLLELGKFTLFSIRRFLVGTLIVVSCYLLYFSPVNIVTTSLLEVVGKT
LSAGVLIYNGSIDSVKLAYGRLSYFKDLESENLRLKLELTELKKIKQLALDARVENQALR
QMLRVPPKLRNNFVTAKIVGVSSTPFASSAVIQAGEADGVRVNDVVKGKSGLIGRITEIS
KNYSTVVLIDDHNSRIPLITSNSKARGLLAKQGDRLKMIHLKEDHTATIGEMIYTSGDGK
IYPKGIAVAKIERVTHEGVFVKLVEHFDDMEFVIIESTL

>gene_857|GeneMark.hmm|677_aa|+|41107|43140 >NVVL01000046.1 Rickettsiales bacterium isolate NORP64 Contig_source1382A_23537, whole genome shotgun sequence
MEEIEQTENDFIEIPRSGTENTVDGVYKIHLTKELSDLSNEFCKYYHTTNNATKENFFSI
VFENNFLAPLEYIDSLMRNPINGLNNIVTYSIAKLSSTKREHLVVIVDSYDEGSTLAAHL
ESAGTLSLEQIEAMVRKVTHLITRLQDNNIYGCSINPSNILMRDGKFFCIREFINSYPLF
HQEIPYIAPEIAECHEAARFNPTVKGDVYALGVTALYAYIKPGFLDDYEDAWDYNFARFE
HTTYKYLLNNEKIPERLRTFLKWTLHDDATIRWDIDNIDLWLGHENSKIHYDSLNEKKSS
IEFNGRSYSTMKSLAYGMFNHWSDAISVTKDSKLFKWASYIQTNTDILVDIKEILNTKSD
TSFIVTNAINSNAKIAKLLSVLDPNGPLRRDGLAVSAEAIPGFLYYLLVTNKKAMINSVI
TLMKDETWFDYADNKDIAGHLSLGSSDSFKAAADSTKSSSIARGVERLVYSLNPNASCHS
ALLKGMYITNIAELLVALDDFAKKNTKRFNIDRHITAFIAAKLDLKDDVKPITFQNFPKL
SENSVIKGLSIINLLHQHEPEIEIPNICKVIGNDLNNLLKEHLHNVEFKKELTSQIEEVA
QEGNIKGIIKLLSNQQQFINDYNGYREACQEAKNLKKKIKSLTNNRGIFDKSLLLGQKLT
VLLSYVLCFLVTVAVIV

>gene_858|GeneMark.hmm|151_aa|+|43165|43620 >NVVL01000046.1 Rickettsiales bacterium isolate NORP64 Contig_source1382A_23537, whole genome shotgun sequence
MSKAVSENNFQFATSLIKIIAIVISLMVIVVSWYAFIFFIGTMLPTIFAIFYDRNRHRCL
SATVCSFNLIGVLPYMIRIWESSSVDYLAKQLLTNIDTWMMIYGTAFIGQLLYVSMPVLI

VKLYEAKTKVHISNYQNQHKELCAKWDLDGC

>gene_859|GeneMark.hmm|394_aa|+|44031|45212 >NVVL01000046.1 Rickettsiales bacterium isolate NORP64 Contig_source1382A_23537, whole genome shotgun sequence
VRHGLTLGSGIAKMILETYPSKKEELYNEHLQEISGANFTFRELDIIACIIHNRGEKKIA
SLLSISYRTVGSHVRNIMSKLGYNSREYIIDAVEKSGKLQYVRQYYFHLVMEAAFEKYLR
KIRGLANRSGIVCATDFNNVTEEEKKSLELFTESLALANITLTDINLLKAEGSYEKARHN
LHIISMKSVNKLSGLYQNIKDAENNSEETTGENIEKNVKKTTQNENRKNIAVYFDKDIDL
LTVQDMEHVDFRSKSNYYFSVLELIGKLTGKPLVAEIIQEFKDAHQDLQKSWAGTGIAGG
EFSGNGSSYFTSKNAAIFAACVGSIIFFMYLLVWNNTPSENNNNSNGLQVASHNKTEIWN
LPKQLPTNKKVSNIITPVSYFVDHVRQLHQLKEN

>gene_860|GeneMark.hmm|323_aa|+|146|1117 >NVVL01000047.1 Rickettsiales bacterium isolate NORP64 Contig_source1382A_23569, whole genome shotgun sequence
MRQAGRYMPEYMAVRAGMDNFLDLCYDSDKASKVTLQPVERFGFDAAIIFSDILVLPHAL
GWDVAFKQGEGPVLRKFESEKDLSLISDNFSAKINNIYDTVSKVKGALPKNVSLIGFAGS
PWTVASYMLEGKGKQDFSVSKNFLYTKRSLAEKLIGIITEKTIEYLSGQIDAGAEVIQLF
DSWSGMLSGSEYHDFVIKPTKRIISALKQKYPHIPVIGFPRGAGYNYDQYIKHAGVDAVG
VDQFTPLSQMKKWQEEIVVQGNLDPVILLGSKENIARSVDEIFASMGGKGSKNFIFNLGH
GILQTTPVENVEYLVDYVKNCQK

>gene_861|GeneMark.hmm|378_aa|+|1074|2210 >NVVL01000047.1 Rickettsiales bacterium isolate NORP64 Contig_source1382A_23569, whole genome shotgun sequence
MLNILWIMSKTVKNKRTKIAEKSVADKLVNNKKTAIILFNLGGPDKPSSVKKFLFNLFND
KHIIPLPAIFRYVLAWFISFRREDAAKKNYAHMGGKSPIYEETKKQADALREELSGKIDS
EFEIFISMRHWHPMSDEVAARLRDYMPDEVILLPLYPQFSMTTSYSAIEDFKNSMHRNFS
KSAMSKIVLKTVCCYPDEEYFIKSHAELITESIGKIKNKDNYRILFSAHGLPVKNIKSGD
PYQWQIENTVKSITNLLSIKNLDYKITYQSKVGRLEWLTPSTEDEIKLAAEQGKEMIIVP
VAFVSEHIETLVELDIEYKEIANQYGVNYIRVPALGINKSFVKSLSRTILKFSKKEGEFL
SSASMSRICPKNCNKCPC

>gene_862|GeneMark.hmm|145_aa|+|2236|2673 >NVVL01000047.1 Rickettsiales bacterium isolate NORP64 Contig_source1382A_23569, whole genome shotgun sequence
MEDYFLWYKAIHIISVISWMAGMLYMPRLFAYHTKAETGSEMDKVFQLMELRLLRIIINP
AMILTYFFGLLTAYIYGCVALGMWFHIKMLMVLGLTIFHHFLARWRKDFAKGENKHSEKF
YRIINEVPTIMMVIAVVMVVVKPFE

>gene_863|GeneMark.hmm|111_aa|+|2708|3043 >NVVL01000047.1 Rickettsiales bacterium isolate NORP64 Contig_source1382A_23569, whole genome shotgun sequence
MYIFGVALHLISIVALTNESTLFSELLILCVIISLSFKAKELSEIDKTSIYNPFLKAVLV
RCLILSFPRELVVTYGDTALLYLLYMVIVVGMMYDLYQTIKIIQPFMKRAN

>gene_864|GeneMark.hmm|314_aa|+|3079|4023 >NVVL01000047.1 Rickettsiales bacterium isolate NORP64 Contig_source1382A_23569, whole genome shotgun sequence
MNMKTIIIQSSSPIRLDRYLRREFSNLTQGVIEKALRSGAVKLNDSKPKSSTRIQKDDIL
TIAEGAFPDKLAGNAGDTKHFSQNVIALAEKLLSKYLLFSSDEFIAIDKPCNLATQGGSK

ISLSVDDALAYLNSQENSNDGYKLAHRLDKETSGILLIAKGAENAARLGTAFQDKLIKKT
YIGLLSSCPAELEGKIVNNIGKDRSGIFEVVKELASGGKLAETHYKVLKKKGSSVLVQFN
PLTGRMHQLRFHSKHIGCPIIGDKKYGGKESARMFLHAQKIVISPAVFGAEIIIESPPAE
EFGFDADVAAQISG

>gene_865|GeneMark.hmm|219_aa|-|4024|4683 >NVVL01000047.1 Rickettsiales bacterium isolate NORP64 Contig_source1382A_23569, whole genome shotgun sequence
MAKLFSYVGVPKLVRTHTCKALPSNEASSNEPSSSELFYETFTMMHNSSLVVNAIREKSL
GSFLQRNPYIRIDNDTIKTISSILSCSSTEVTINIFTELRYLLEKIPKEHLNAYIAHLKT
LEVSDTVDILAESIYTALQTLQWLQTPPEIKGVVQESQKSPAPPEIDALIISYILPVETV
LDGSQKISFSFFKQPHIDAPSPPEIVDITGQEAGELAAS

>gene_866|GeneMark.hmm|547_aa|-|4859|6502 >NVVL01000047.1 Rickettsiales bacterium isolate NORP64 Contig_source1382A_23569, whole genome shotgun sequence
MFSVLSSGKVSLTKYIVTMENSKKTGFLAELRKIFWPIEWHENKKFIPMALMMACILFNY
ATLRSVKDGLVVTHIGPEAISFLKTYVVLPSAIMMMIAYAKLCNMMSQQKVFYTVTSFFL
GYIAFFAFVLYPNPEAFHPSASTISALIEEYPNFQWFIKIAGNWSYASFYTISELWGSMM
LSLLFWQFANQITKTDEAKRFYSMFGLLGNVALPLVALTLWFFMSEDTHIVPDDVKLIPV
LCITIVSGLTILFLYSWINRNVLTDPALCDQEKPNSTKKKKAKLSLGESFKMIFTSKYLG
LIAMLVLSYGVSINLVEGVWKAKIHELHPTVEAYTSYMGTFQAYQGFVAIFFMLVGSNIL
RRVSWQTAAIFTPIMILCTGLAFFSFIFFGDTMGLQVAAFLGTGPLAMVVMIGMAQNVLS
KGVKYSLFDSTKEMAYIPLDDEMKTKGKAAVDVIGGRFGKSGGGIIQSTFFILMPAASFT
EATPIFASIFFGIVVLWVFVVRALGKEYNKKLLRQEMLEALNADYEDLDDLDDLNKADKN
SKSDSKA

>gene_867|GeneMark.hmm|306_aa|-|6524|7444 >NVVL01000047.1 Rickettsiales bacterium isolate NORP64 Contig_source1382A_23569, whole genome shotgun sequence
MNNPFSHPRVTHNLHFITKYKFLEQFESFFPEDTLGISSSEVKSFTVDSQPNDLWSMEVY
LPAEPDLKLLASELSAFAEQHDLEIESELTLKQIEDKDWVAEYQSQLKPIIAGQFFITSS
SAKAACPKEKTPIFIEASRAFGTGDHATTSLCIEAMEALSDIKFKNIFDIGTGSGILSFA
ADKIWPGTQLLACDIEEISVKLAKENQIHNNSHIDFYQNTETDLAIPDSRVGKFDLIVSN
ILAKPLISMANSIRQITSQECRVILSGFLDYQENEVTNAYITEGFEVEEVLRRNSWVCLT
LKVKRY

>gene_868|GeneMark.hmm|124_aa|+|7519|7893 >NVVL01000047.1 Rickettsiales bacterium isolate NORP64 Contig_source1382A_23569, whole genome shotgun sequence
MNKIRNIAICLCLTLFANITIAADKSGVSKSKTKLRDPAEFLRVIDDYKAYVATVSPETR
DEIIVYRKEIAKLNKEKKLFYRKLSKNAQGYLKQEQKYKKKLPLNRKSLINIQTPGQKKS
RKKG

>gene_869|GeneMark.hmm|61_aa|+|8014|8199 >NVVL01000047.1 Rickettsiales bacterium isolate NORP64 Contig_source1382A_23569, whole genome shotgun sequence
MTGAGVIAQSVERLDGIQKVGGSIPPNSTSAPLELRLAGQPIHCYETSFGNIVIQNVQPI
F

>gene_870|GeneMark.hmm|193_aa|-|8263|8844 >NVVL01000047.1 Rickettsiales bacterium isolate NORP64 Contig_source1382A_23569, whole genome shotgun sequence

MKKILLTAMATTMLASPSAYAMEDVFYIKANAGLSQLSKIQTLKSKNKLFFGAGIGYNVI
DSVRIELMLEHFSDPLHKKGDVKLQGKANTLMFNGLVNIFDAHLVNIFVGAGAGFSEVSA
VKSGETDAANNGEIKKKHNLAFAGYLGASWELGGTASLEITYSYRDLGKTRQLNEKSFHY
KGHHLSAGLRFDI

>gene_871|GeneMark.hmm|82_aa|+|8961|9209 >NVVL01000047.1 Rickettsiales bacterium isolate NORP64 Contig_source1382A_23569, whole genome shotgun sequence
MLLPVDMDCHESEHKPGGGDRHECGARFESGHEREAGHEPHNNHEIFVADDLFSQPYYLH
LQGGRALLHAENAALECARYFS

>gene_872|GeneMark.hmm|103_aa|-|9332|9643 >NVVL01000047.1 Rickettsiales bacterium isolate NORP64 Contig_source1382A_23569, whole genome shotgun sequence
MQKPKPVTITKDKIAERLKGRLGLSSVICEEITSHIFSEILNITEQEGKLMLKNFGVWRI
NHKAARVGFNIKSGNSVRITPRSVLRFIPSKSFKEQINKSDAE

>gene_873|GeneMark.hmm|332_aa|-|9698|10696 >NVVL01000047.1 Rickettsiales bacterium isolate NORP64 Contig_source1382A_23569, whole genome shotgun sequence
MASKIIGSGGYLPTKVLSNSDLAKSIDTSDEWIRSRTGITQRHIAAEGEYSSHMAHKAAL
AAIENACINDACINDARINDAGIDASVIDLIIVCTNTPDNSFPSTANKLQGYLGLGNIPS
FDMQAICSGFIYGMQVADSMIKSGKYKTVLLVCAEKMSSLLNWQDRGTCILFGDGAGAVI
LQQTDSDSGIIDSNIYSDGSMYDMLYTDGGVSMNGKSGQVQMKGQEVFKKAIEKMAESTG
LLLESNNLKISDIDYFIPHQANLRIINSLAKKLNIDDKKIITTIQKHANCSAASIPLAFH
ELKSKGLFKPGDILAFTAFGAGATWGSLLVRW

>gene_874|GeneMark.hmm|65_aa|-|10704|10901 >NVVL01000047.1 Rickettsiales bacterium isolate NORP64 Contig_source1382A_23569, whole genome shotgun sequence
MAVPKKKTSTSKRNMRRSHDALSRINIIVDKDTGEYRLPHHVDQSSGSYNKRQIVIAKAD
STDSE

>gene_875|GeneMark.hmm|174_aa|+|10969|11493 >NVVL01000047.1 Rickettsiales bacterium isolate NORP64 Contig_source1382A_23569, whole genome shotgun sequence
LYYISLTKSRIFYIKNFMTQLNTYFLRLFSAILLLVLTGCQTIDVRGQFVSDTAIEKLSA
QKPTKQEVTDMIGSPTYIPDYSKNTWYYIQRSLARKAWFTPKVTEQRIIKITFNSKDKLK
KVQLLENLQNENISAKSQFTKTHGTEKSGIQTFVKNIGRFNKTSTGSNRSKKKK

>gene_876|GeneMark.hmm|100_aa|+|11651|11953 >NVVL01000047.1 Rickettsiales bacterium isolate NORP64 Contig_source1382A_23569, whole genome shotgun sequence
MINDDELKKLQKLSKLSFQPEELKTFSAKLAGVLDMIDEMQELDISGVKPLRSVLDGSQR
FREDKIENSDISEDLFKNTPEEIAEFAKEIKCFVVPKVIE

>gene_877|GeneMark.hmm|493_aa|+|11955|13436 >NVVL01000047.1 Rickettsiales bacterium isolate NORP64 Contig_source1382A_23569, whole genome shotgun sequence
MNDLIKLSITEALSAMKEKKFTPSELVEAHIKSAERTKNLNSYITETFDSARLLAEKADQ
HYNNGTARKLEGIPVAVKDLFCTNGVKTTAGSKMLSNFSPTYDSTVSKNIADSGSIMLGK
TNMDEFAMGSSNTTSYFGNVISPWKEIGSDKDLVPGGSSGGSAVSVSAFSSMAALGSDTG
GSIRQPAAFTGIFGMKPTYGRCSRWGMVAFASSLDQAGIFTRSTSDAALMLEAMMGFDEK
DSTSIDQSVPELVSACKKSVKGMRVGVPTDLMELDGINSEIVKMWQDSIAMMRAEGAEIV

DIKLPHAKYALSVYYVIAPAEASSNLSRYDGVRYGHRTDKTGLNLDDMYIQTRTEGFGEE
VKRRIMIGTYVLSSSHMDAYYTKAQRVRRLISNDFKDAYQKADVILLPSTPTAAFGIHDN
QDDPRAMYLNDLFTIPASLAGLPCASVPAALSVSGLPLGMQLVAPALDEYNVLRASAVIE
RATSDIDFTPKGF

>gene_878|GeneMark.hmm|481_aa|+|13437|14882 >NVVL01000047.1 Rickettsiales bacterium isolate NORP64 Contig_source1382A_23569, whole genome shotgun sequence
MAYITGNTGEWEYVIGLEVHAQISSNAKLFSATATKFGANPNTQVSLVDAAMPGMLPVLN
KYCVEQAIKTGLAINAEINKLSVFDRKNYFYPDLPQGYQISQFYLPIVQNGHLILTLEDG
SEKKIRINRIHLEQDAGKSIHDQHPDYSLIDLNRSGIALIEVVTEPDMSSPYEAAEYVKK
LRNIMRYVGSCDGNMDQGSLRCDANISVRRPGGELGTRCEIKNLNSIRNIVKAIEYEAMR
QVELLESGGVVAQETRLFNTETGETKTMRSKEDANDYRYFPDPDLLPVRVNDDMIERLRS
ELPELPDAKVGRYISELGLSKYDAEVIVADGAITDYFEEASKGASAKLIANWLTSELFGR
LNKNSMSLAECKITPSMLRDMVVLIEKGTISGKIAKTVFDDMFESGQAAEKIVKEKGLTQ
MSDRGSIEPIIDEIIKANPDSVEAYRGGKDKLFGFFVGQVMKKTGGKASPQMVNDILKEK
L

>gene_879|GeneMark.hmm|263_aa|+|14952|15743 >NVVL01000047.1 Rickettsiales bacterium isolate NORP64 Contig_source1382A_23569, whole genome shotgun sequence
MTIKIHTQNDFAKMRIAGKVAAETLDHITEHVQVGVTTDHIDKLCHDFMVSKGAIPATLG
YKGYTKSCCTSINHVVCHGIPSDKKLKNGDIVNIDVTAIVDGWHGDTSRMFYVGDVGVKQ
ARLTQVTYEAMMLAIDKVKPGAHLGDIGHAIQTHAEKHNYSVVRDYTGHGIGRVFHDEPS
VLHYGKAGKGVVLEEGMFFTIEPMINAGKYYTILSKFDGWTVTTRDKSLSAQFEHTIGVT
KDGAEIFTSSSKNWLYPPYGQKK

>gene_880|GeneMark.hmm|232_aa|+|15727|16425 >NVVL01000047.1 Rickettsiales bacterium isolate NORP64 Contig_source1382A_23569, whole genome shotgun sequence
MDKKSKDDKQPHYLGHRKRLKDKFLSNARSTLGDYELLELLLFQSIPRRDVKPLAKSLLQ
KFGGLRQLVHADKEMIMSVDDMTEGSFLQLKIIQEMLSRILYEEVQNKNVISSWGALLDY
LKFNMGCLKIEQFRVLFLNKRNALIADEVMATGTIDQTPVYPREIVKKALFYEAGAIILV
HNHPSGNPDPSKADIELTRQISTACKTINVTLHDHVIIASNNYYSFKTNRLL

>gene_881|GeneMark.hmm|101_aa|+|16422|16727 >NVVL01000047.1 Rickettsiales bacterium isolate NORP64 Contig_source1382A_23569, whole genome shotgun sequence
MKLVKDEVNKLIYKIFKRKHPILAEMIINWGRIVGIKFSDKSMPFKIVTSKEAGKKINIL
YITTESSTISMQMAYQQDLMIERIAVYLGYKGIHKIKILTN

>gene_882|GeneMark.hmm|102_aa|+|16741|17049 >NVVL01000047.1 Rickettsiales bacterium isolate NORP64 Contig_source1382A_23569, whole genome shotgun sequence
MKDHDIEFVQESERIITEIADAIEDQDEECLIDVDLNSSILTLTAEQGTFVINKQTAAQE
IWLSSPISGPYHFKKDKGYWRSRDGTELFEILAKELPINIKW

>gene_883|GeneMark.hmm|601_aa|+|17055|18860 >NVVL01000047.1 Rickettsiales bacterium isolate NORP64 Contig_source1382A_23569, whole genome shotgun sequence
MISNNVKQLAEYLALHIKSLIVVLVSLSIVAASLLSIGYVFRQLIDHGISNNQLDEIHGA
IYWIILLIAVFSIGSFFRSYFINLITVKVISKLKTDTHSNLLKIDLVAFEDLKVGDIISR
LGSDIELIGGLIINFLSFFIRNSIMLCGAITLMFIQSPKLSLLVLVSIPLLLFPLLRLSK

YVRRLSRSVLDEQGILASNIEENFTAIRTLYAYNQQEFIANKFNNHIAHYIKHAGLRFRA
RSLFFALAIAIIAGSITSVIWIGSVDILSGSMSSGQMISFIYYAVIVGTSAAGIAELFSE
IQAPLAALDRVLELKHLKVAQKSGVMQELSANGGGSKEIPTKVVPAKVVSSGDCSIRFEK
VRFAYPARADLVVLDGISLEIEHKKFTGIVGKSGSGKSTLMQLLLKFYNHQSGDIFIGSD
NINTLQDSLIRSKIAYVEQHPTIFSGTIKSNIAFSNPNATEAELREVAEICGILDFAKDM
KDGLDTPIGEKGVRISGGQKQRIAIARALIYRPEILLLDEATSALDNASEKKILQNIRSL
MKSKTIISIAHRISSIEDADIILVIDKGVLEGSGKHAELLRNSQIYNVLYKEQSDLKLAD
K

>gene_884|GeneMark.hmm|154_aa|+|19022|19486 >NVVL01000047.1 Rickettsiales bacterium isolate NORP64 Contig_source1382A_23569, whole genome shotgun sequence
MNKVICLIMNLILSLFIAQTSLASKPGAADANHSSVLEISDAWARKSMSPNNNSAAYMKI
HNPTDEQITIISASAMVVANNVELHKSFVDEGGISRMTAIDKIVVPAKSTIELKPGGIHI
MLIDLKYSLSEGNKFIIKVKIQNSDPIGVETMVK

>gene_885|GeneMark.hmm|328_aa|-|19811|20797 >NVVL01000047.1 Rickettsiales bacterium isolate NORP64 Contig_source1382A_23569, whole genome shotgun sequence
MQKLLTLIITAVILYMIFEQRLDNASTSHIIPAQENINTSQGQGIGQTHGEGQSKGRSPN
QASGTFLENTLSNVVSNVLKTDEGRIFMKSMFQPMNNGSFGDGFEMNNNVFLNSMFKIMS
FDEGVGMPVSCGHIVTVHYKILNMKNITLEERTATFPLGSEEIAPGLDAVIVGMRTGQTR
HATISNKYIPGTEQDKESSFKINVLLQEIMPKHFVGEDVKIFDSKLAYKRPLVCGNKTIY
DARVTKLSNGEVLYDSKKRGEKINMRIGNIVYPVIFSHALHNKIPVGTRSVIAKGHLFKS
YASNFSTIFPDKELPKDEYFMVEFFNFE

>gene_886|GeneMark.hmm|615_aa|+|20872|22719 >NVVL01000047.1 Rickettsiales bacterium isolate NORP64 Contig_source1382A_23569, whole genome shotgun sequence
MPSIFKTEKHYHDVLIIGSGGSGLAAGVFAANKNLDVAIISKVHPLNSHTVAAQGGINAS
LGNVTPDDWRWHMYDTLKASHWLADQDSVEEMCKGAGEVINMLDQLGVEFDRNKNGKIDQ
KIYGGHTTEFGQGGLAFRACYSKDKTGHSIMHKLFQHATEKQIAFYNYNFSLELIMQNER
CCGVLCLDMEKGILNVITAGNVIIASGGYSQIYATATSSAAGTGDGCGLVAGAGGALQDM
EFVQFHPTALRAGVLITEAARSAGGKLLNGDGERFMKKYAPKFMELAPRDIVARAISTEI
SQGRGCGAEKNHVFLDLTHLSREEIKENLPTIFENCSSFGGIDPSEEYIPIAPAAHYTMG
GIATNSKCQVISCTETEKPITGLYAIGEAACISVHGAGRLGCNSLLDLLVFAKKAVESLE
ELDNSGIGVDAGVSASNILTNFEQIFKGEIIDIDKMTRDLRNIMSMHVGIFREKKGLQSA
LVELMQLKEQYEQSALEYQPLQDKSSQDKSSQDESLQDESLQDKSSWDEFLQWNIELQHY
LELGNMLISAIATTESALWRCESRGAHWRQDFPETSDEFLGHTIFKPGQKENPSLRPIRK
SVNNVGFDEPNERNY

>gene_887|GeneMark.hmm|217_aa|+|22787|23440 >NVVL01000047.1 Rickettsiales bacterium isolate NORP64 Contig_source1382A_23569, whole genome shotgun sequence
MSKFKLLLISLLLSSPTIFAHIGTPLHLAHLNNLNYCTLTPETVNEYAPERFESTNNLLR
KSGQRALYCGEKIIIHGIVLDQNCAPVSDAKIHAWQVDCMGKYSYQPLKTRIDQKLININ
PDSTFTGNGTAMTNNKGKFHFVTTYPRAVHGRASHINIKIQHRMLGALETRLTLKGKRVR
HPSKIPELDSISEIAEENNIPVYSFKIVLPGIGMNSY

>gene_888|GeneMark.hmm|124_aa|-|23494|23868 >NVVL01000047.1 Rickettsiales bacterium isolate NORP64 Contig_source1382A_23569, whole genome shotgun sequence

MSDSVNIKEVDEFIGKRIYESRLAQGMHRQHLAKMIDVTQQQLIKYEKGINRISLGRLML
IAKALRKDMSYFHEWFEPGEEVIVTKQQTTCIEMSRNFMKIQSSEHQSAINSLIKSLLKA
EQAA

>gene_889|GeneMark.hmm|298_aa|+|24156|25052 >NVVL01000047.1 Rickettsiales bacterium isolate NORP64 Contig_source1382A_23569, whole genome shotgun sequence
MKPEDTIESYIDHYTTENGFTESAIKGVRLFRTDVPAARRPFTGSTCICFGIKGSVTCYY
SGNQFHYKSGNYMVVSMPMPIEWEMFTDEGEVSYAIVIDIDFIILQQILGRMDKCKSTPP
KEVIESNLRPAALNKDMKATLLRLLRCLDSPAKVKILAPGILKEIFYNVLCGSKSSNLCN
LANHNTPITRVAKALNYVNEHTNKPITVKMLAQEAGMSVSAFHTEFKKITAESPIQYVKK
MRLNKAELLLKQGKISANTVAQYVGYKSASQFSREFKQLFGVSPMGRLNKSWLMEKNL

>gene_890|GeneMark.hmm|296_aa|+|25049|25939 >NVVL01000047.1 Rickettsiales bacterium isolate NORP64 Contig_source1382A_23569, whole genome shotgun sequence
MKEGTLAYYVDRHIKENGIIDSPLKGVSFMRSDKHIPRAFFSYNPCIRILAQGRKRGYFA
GQEIQYNEGEYLTISLPVSIEHETFGTKEKPVLGIIIDIDLDMLNELIGKAKKDMIESDM
AIKNPSNTSCAPARNLQPIALDKCMHCAVLRLLHSFESKEKTRVMGNHFIKEILYNALCG
KDGAKLHDLVKYSQQAASIAKALNYVHEHINHRFSIEGLAHKANMSVSVFHRSFKEIMGD
SPLNHIKKIRLTKARAMVKQEDLAINVVAKSIGYENPSQFSREYKQLFGISPKGER

>gene_891|GeneMark.hmm|91_aa|-|26274|26549 >NVVL01000047.1 Rickettsiales bacterium isolate NORP64 Contig_source1382A_23569, whole genome shotgun sequence
VEVRLSKASGKYLKKLGSSKFAKQIAIKIKVLSTGHQNDTKKLKGNLSDYYRVDIGEYRI
IYQIEGPELYISLIGKRNDGEVYKLMERKQQ

>gene_892|GeneMark.hmm|95_aa|-|26554|26841 >NVVL01000047.1 Rickettsiales bacterium isolate NORP64 Contig_source1382A_23569, whole genome shotgun sequence
MLLSLDNINGVRVMISMSAKAAQSHFGEMLSLTIREPVAINRYGKRAAVVISNEEYEKFQ
KLEDLYWTAKAEEAAQDGFLSSKESDDLLNSILTS

>gene_893|GeneMark.hmm|960_aa|+|27516|30398 >NVVL01000047.1 Rickettsiales bacterium isolate NORP64 Contig_source1382A_23569, whole genome shotgun sequence
MGAIMHFNLDIAVISAFLSIIIVVGVVAGFGVKTIKDYALGGRNFSTATLTATIAATWIS
GNFFAIHTSGIYKDGIFYLVAGMGDVLYIFITGLVISSKMNEFLGSCSVAEIMGKLFGSK
VRLISAIASVLLSIGQLAIQVQILSMTLSHFLGLDAFLTTITSSVIIIGYSSFGGIRAVT
FTDVIQCITFGVFVPVFAMFIWQSLGDTTLIAATFNSNTLFDYTELVNTDNPKFWPFLFL
FLFYSIPGLNPTMVQRMLMAKDSKQIKSSFLIAGVVSLVLYISVALISIVMFSHNPSLPL
EGDNIIMHIIDNELPTWLKSVAIISIMAMVMSTADSWINASSVLFAHDICPALKIDKFDE
LKVSRIFAISISAASVVIALSFNNLLELMMLSATFYMPIVTAPLLVALFGFRTSGKVVMI
AMGVAAGTVIFWKWQLQAITGVDSVMIGVIAHIIALFAAHYLLGEDGGGTSPGTSPGAGS
SFGKRKNTRRERAEKILQEMKDLNILSYCNKRLPESRMTYIYFSIAAFLTIISIFSIEKD
LYNMHTALITSLQAIVFSIATAFLCKALWSDLIKEKYLGIIWYVSIFTGLALISSFLVLL
SKFSYVSLTILIMHLITISLLVGWRMSIIMIAACLWMSFSFYEGYIGEMVAGEIFDLKLK
MIYLLLMTSGLAFTFLRSKQEQMEKSECKVETLETEVTTQGTKITTLNKKVGHYSERVSD
QAREIERLGATAQKILNNVNHELRLPVGNVMNFAEMLRDGLEKYDKEHLKMLSEEVYNNS
TRLSSMILNMLDLATLNAKKIELKKEKINFSKLVHERFENCRNIYAQDKKALNFHLNIED
NVLVFADINYIRQMIDNLVINAINFSEQGTIAIIVNSEEYFVSFTIKDKGKGIPKTEIYD

IFTPFKMGSNTESKAEGRGVGLALCKAVMEAHGGKISAESRPIGALFRVCLPYGKLKGGK

>gene_894|GeneMark.hmm|1169_aa|-|30494|34003 >NVVL01000047.1 Rickettsiales bacterium isolate NORP64 Contig_source1382A_23569, whole genome shotgun sequence
MNRCLVIILTLFVLTGCSGDSCIEADDFGHAVYKVSARYAKSEIGGQPKNQVAPWRDSGY
KVNGRPLSITVKGWRYRYDRNNSSDLSAWCAWYGSSGDSRKLAKICARLRDCHFIDEIMC
TETVDAQIDNAPCLFRNGVGAYALIAKKDPNLTPQSQRSPKGLSFHLAKPVDRSYEVYDF
IRENGKLKKRILGGRIYNYKDQDANNYVNSTLYFKILDKFYDDNSGQYQIVVKSGIHRSN
ADPISHGIGLVKEYLFGDKKGLIKGIYLGIVNNPEYRTAVSALLTLYVMFTGLSYLAGNI
QITQMELVIRISKIAIVSALLSTEHSWNFFNDYLFVYFIGGVTQIIDMIMSAGATGPGSS
SIMALMLAPQTISKLLSLLFVDWAGFIYIILFFIALAFIMMVFFQAAVIYVTAIVAIGMI
ITMAPIFICFMLFGATRNLFDNWLKQLTSYAIQPIILFTGLALISMILRQEIYGSLGFRV
CKKDFPKMTNADDTPVFGHESEEILGFTMKHSIFYWWFPDPMKGGEFAKETSLIPIPLDN
FSKSRDGMRDRERNPESGFCEAYGCYDERYPDLPFLDVVKDRDRINAFQKGSFVQLDNLF
IIFIAIYLLHKFNRLSISVARFISDTSGNYAELESVEAGVRQDIKQHGPGLVTRAADITI
GKANRQKITALRDMAQAKIRSAGKKIYTKASAFVDKRRIRGLEQEAISSSPNKAVLSEIR
KKSGLDLSKIKIGSLKKYQARLANELRNMDSNLTAKQVSKLASQMSGKKYSALKNEFAQA
KYGMQYSALPENAKQQINTLHKDRALRDLAREAENVRMFQTEYIEAYARLSDKGIGLVGK
KSRLIRSIEEIRHDAKERKKHKQNKQQQIGREIISEIEGIKHSAYSKLSGGREDALSLGV
AGGAWHEINTDPKADNHRLQTQAEIIADQRLSTEREATARIIEDLSARHGTNVISPEFLA
EAERNGAADLEKYQNLEREDLNNRLHLALTAKTDDGPVLMGEKYMQEYASDEQMKEIIDR
IHETEEVFAQYDQFLSRATYYEDNLQFAEENLQENYAMLKDHYDRDNITAEEMPALLRSY
YDEADDISPEDAYSKIVQLEESISEFSSSQETLQQIDHRREELAEETDQHIENVNKHRTD
AGMEEYTPEARVELPVRRLRKIDDHLRNL

>gene_895|GeneMark.hmm|1136_aa|-|33996|37406 >NVVL01000047.1 Rickettsiales bacterium isolate NORP64 Contig_source1382A_23569, whole genome shotgun sequence
MRTIQLLLITICLAVHVDAYGSGDGSRYDWMNRDLSDRGWGCIEPPHFTSTKGKTTTLNL
DNKGVWVSSGISVEKDKLLKVNWSAGEVQSRPEKYTVLYRIDPRFAKPQIFIQRYDYKQQ
KYISNFHKAGLLKYQDSRTLNNNYTKRFADFNKFFNSQSITIKKNDVINVTLIGSGEYFS
NDSQMRTLGYENEPLVIRTNSALTDNRMIYSNASPWCMGAIGEDSSQYTDLCLKYPGYYT
DSAENIETLVGGIQNPSFARNLISIGKCPGSANGRNNTLCYYDNGRGMQFKLAGKTIKNT
PEKFVASSSSSKDFFYYKSDVNGKLEFVTQWDIAGMYNEMYTQNGKPVAKNWSWQFMTQW
PSFGNRSNFNSKLVDIRQNLSMNFLHFGRYFMRVEVGNSTASVSEADLNSINIEYTIMKN
GAPGSSAKGTSIGRGFSGNASKTGSLWVRVAGKNDNVTGVLHARTTSYTGSTWFSEKIYG
KLIKPLREKFARLTKTMYSKLVTDKNLQNIAMLMMVIYISIYGIMFLGGSVKITIKDIVT
RIVKISLVVTLLFSSSSWEFFNSYLFDALVSGSDYLMSSVIESTSQSGNVFGFIDPIFDK
YTDGSLWALLFIQLLQIHNGMTFFAIMTIYSILTYFRALIEVIVTYCLAFLGLAVLISIA
PLFIILILFERTKSMFDNYISTMFSYMIQPTILLIFFLMIDQIMSTHIANTVVRSCWSTL
IEIKLGLDLRHMGIPLSFSIGLPFLPNISFYVPQVTPIKTVSDFFIIGTISKVATSSLIF
FALAKLSSGLVNYVLLIVQFLTNVLAARREGILQETKNPVTDIMKDMERIIFPARFVARN
VRGFAKEKLIDQKIDSDNIRHRRGGDIDIDYSGMQKSNPDDPQNDSPDNNGGGGGGDGDN
AGGSGGSRGSGGGRSGGGSGGGRSSGSDGGSSSGNDGDGGGSGGNSSARSASTSRPSSGG
YDEHREYDESEFHASVEMTEFKSSTPAEPDDSKAASVSDDAPENARSRPGSDGALDNAPN
DTPDDAPENSRAASAPEDAPENSTDSAPGDASENSPENPSGQVERRDSEALHSPEESKAP
PSARDDAERRDVNESIEMTELAKKKRAPNDGSRGDGSRGDGSRGDGSQQGGDNSNE

>gene_896|GeneMark.hmm|153_aa|-|37637|38098 >NVVL01000047.1 Rickettsiales bacterium isolate NORP64 Contig_source1382A_23569, whole genome shotgun sequence
MITIDHKTQAMASASKLKKGAKSDKNRKTAFAPSKQTHEASSNESFAGVADVGGMLFLQE
FDQYAQDKQNLEEFGNKALKTLKELQLEILSGRINGAQLHSLKKEMDSNKFIISTPELAI
LADEIKIRIEVEIAKIEVNRSADIASKNLRPQI

>gene_897|GeneMark.hmm|449_aa|+|38226|39575 >NVVL01000047.1 Rickettsiales bacterium isolate NORP64 Contig_source1382A_23569, whole genome shotgun sequence
MFQTLTQNLTKIFDKMKGTGALSEAQIDSAMRDIRVALLEADVALPVVREFIDAVKKQAK
GQKVVKSVSPGQMVVKIINDEMVRLLSPNESESELNLSDAPPVNILIVGLQGSGKTTASA
KLALKLKNQNKKVLLVSLDTYRPAAQDQLEVLSRDIGADCLEIVKGQMPLEITKRAVKES
RLGGYDVVIYDSAGRLHIDEEMIEEVASVKKIINPTETLLVIDSMIGQDAVRVASAFEEK
LKITGVILSRIDGDSRGGAALSVRHVTGKPIKFLSTGEKPTDFEEFDAKRITSRILGMGD
VVSLVEKAAGLVDQEDAEKMAAKIKKGRFDLNDYISQIKMIKKMGGFGSMLSMIPGMSKF
SNKLGDAGDGDKLLSYQEAIVSSMTKKEKRNPDLMNASRKKRVASGSGISVQQINTMLKQ
FKQISTMMKKASKMDPKSLMRGDMGKLFS

>gene_898|GeneMark.hmm|69_aa|+|39642|39851 >NVVL01000047.1 Rickettsiales bacterium isolate NORP64 Contig_source1382A_23569, whole genome shotgun sequence
MGKAKIASKNNPEGRGRAKEYFHNGKKIKPVKMISLSQSFFAAEYDDSGEMVLDAAGKVL
PWGKAKYLG

>gene_899|GeneMark.hmm|418_aa|+|40219|41475 >NVVL01000047.1 Rickettsiales bacterium isolate NORP64 Contig_source1382A_23569, whole genome shotgun sequence
MIHHSEAFTIDYSNFSLNTALYPRLVAAVASIVPKILESIEDGTPAYKIFSIEHARQQAE
SCAHYAETIKANFSDLIVIGMGGAVLNPRTAISLTEFVPSSVKIHFLDNTDPFFLQKLLA
QIDLKNCAVLAISNSGQTLETLALTGVMINKFEQAGVRLSDRFFFITNKQSGRLRDVANK
IDATIIEHTSNISGRYSTLTNVTSLIAQIAGVNMKEYLAGAEMVMKDFFDKKENTPAALS
VSIMLNEPKSTLVNIGYLQQFDVFLEWYSQIIAESLGKEGKGVTPIRGLGPNDQHSMLQL
YLDGWKDKIYSLFYVENWKNVQTHKACDLAALGGIAGRELQEINTANFSATRAALLTRKL
PVRTITLKDLSARSIGALVTHSMLEIVIWSRVMHINPFNQPGIELIKTESTKIIDSKK

>gene_900|GeneMark.hmm|40_aa|+|41866|41985 >NVVL01000047.1 Rickettsiales bacterium isolate NORP64 Contig_source1382A_23569, whole genome shotgun sequence
LRDITKKIKFIQMFEKPLISFDYAIKFLLKNKEGYEIKEG

>gene_901|GeneMark.hmm|46_aa|+|1|141 >NVVL01000048.1 Rickettsiales bacterium isolate NORP64 Contig_source1382A_23573, whole genome shotgun sequence
YLAMINIITIALPETGSFFKYIVHSFFMGFMNLDFNYSYYASHIAS

>gene_902|GeneMark.hmm|251_aa|-|146|901 >NVVL01000048.1 Rickettsiales bacterium isolate NORP64 Contig_source1382A_23573, whole genome shotgun sequence
MLEHFTLEFVFHFMLVFARLGTAFRMFPGIGSPYLFERGKLAVALSISFVMMPVLTPYLP
PYTDNSALNLGYLVMEILIGLVISIAANLYFQSLHFVGQILSMQSGLSAAAFFDPLQKTQ
VAIFSNFMLLIAIVFIFTTNTHYLFIEAVADSYIKFPVGELLDSGDVSKFVWLVVNDSFI
LSFKLVSPFLVIGLAILTGSGLLARLMPNLQVFFVITPAQIIIMMGTMYLVIHSIIIKLV

GKIGASLSMSF

>gene_903|GeneMark.hmm|216_aa|+|1143|1793 >NVVL01000048.1 Rickettsiales bacterium isolate NORP64 Contig_source1382A_23573, whole genome shotgun sequence
MDDLAAMMPQLYFIVQGIGVTLKYSMSAVCIGLIIGTLLAICKMSDSKSLRLFAHAYTSI
FRGTPLLIQLTIIYFGLPGLLGIKMSVFTAGTLAFSMNSGAYVSEIIRAGINSVDKGQTE
AAKALGIPPVLRMKDIILPQAIRNILPALVNELINLVKESALISVIGGEDIMRRAQLISA
ETYSFFTPMLTAAATYYVLVLVISSFAMMLEKRLAL

>gene_904|GeneMark.hmm|240_aa|+|1790|2512 >NVVL01000048.1 Rickettsiales bacterium isolate NORP64 Contig_source1382A_23573, whole genome shotgun sequence
MIVLENVTKKFNDQTALKDINLTFDKKEAVVVLGSSGSGKSTLIRCINNLETPTSGSVFI
NGTQLTSKTRGKLCFKIGMVFQQFNLFPHMSVLQNLTYGAINVLGNTQHIAEEKAYKLLK
KFGITEKANSSPLDLSGGQKQRVAICRALMMDPETILFDEPTSALDPEIIKDIIGIINEL
KGQITMIIISHHIKFARVIADRIIFMDQGKILADQPAKEFFTKPSSHRARLFLENVGDLM

>gene_905|GeneMark.hmm|128_aa|-|2592|2978 >NVVL01000048.1 Rickettsiales bacterium isolate NORP64 Contig_source1382A_23573, whole genome shotgun sequence
MNRVILDASALLALINNEKGAEKVEPLIGRAVMSSVNVTEVAGKVYEILGNEEQCKLAIE
PFISSIIAFDKVQCYIAASLKSQTKHKGLSLGDCACLAAALHMGLPVYTADKVWAELNIP
NIEIHLIR

>gene_906|GeneMark.hmm|81_aa|-|2971|3216 >NVVL01000048.1 Rickettsiales bacterium isolate NORP64 Contig_source1382A_23573, whole genome shotgun sequence
MFAKAKISKGGKISIPSICRKALNVSDGDELLFDISDNQVVISPVKFTLQKIRKLLKDRN
PSKTSLVDELLQERKQELKNE

>gene_907|GeneMark.hmm|135_aa|-|3343|3750 >NVVL01000048.1 Rickettsiales bacterium isolate NORP64 Contig_source1382A_23573, whole genome shotgun sequence
MKAFEKYKNAQAYTSNSSHQLIFIFDELLKLLFTAQKALKEEDYETKFKALAKAIEVFYI
LKSGVEIDNPDESTKAIDMFYGATIVRLENINMTISSPPEDLVLMIDSIGEVRQALAQSI
APEKEEKKESYITSN

>gene_908|GeneMark.hmm|606_aa|-|3819|5639 >NVVL01000048.1 Rickettsiales bacterium isolate NORP64 Contig_source1382A_23573, whole genome shotgun sequence
MDISKGVSITGMLGPVDEFVAKATSQQELVLEDFNDQVSVRHDKLGAISELQMLLTDLKA
KATRLSDPMETGFNHKHCSTTINGESADKFLKNVRVDGTVSNGHVHVAVEQVASGASLTI
AFNAQHTGFTNDANPIGLDGDLTLTIGGNARIINVAAADNLGIIVSNINLNFENNGDAFE
AFLINGNPGGVPSSFIEIRAKNTGFEQIGIAWADIGNPAMTMLEHAHLDGQDAIVYVEGV
RYDNASNKYDNAIAGLSFELAGAVNVAAAVPNLAYNGFDYSAICTEEDHDPVKKMIIEFA
NSLDMLTYFVAKHGADSDVSTIDKYADPYADKSKDDQGGVLRGSMLLVEATQIVERFTGL
QHNGNGITSIAQMGMGLKTTVQDGISFETLYFSDEDLFVKKFEDDFESVRRFFINHVQVT
PTPGNLSSLQYIPTDMPGEVITPSIIGRDLPVSITYDPAGAVTAFSVTADGQVVNGAITY
NASLNRYSISFAGTALKGMAFSVDPKAANNVVENFTINYTRGISNLVRDEARTMLSDDGS
GGSTIAEAGRIQDRIKSLETSRDKIQAKLEKLKGDMQADYKRMILLETKSAIALSQADAL
LGLNDG

>gene_909|GeneMark.hmm|154_aa|-|5876|6340 >NVVL01000048.1 Rickettsiales bacterium isolate NORP64 Contig_source1382A_23573, whole genome shotgun sequence
MSLTHRKVCIDDLKIIVDLLSTDKLGKTREQISEELDGRYIDAFHKIDNDPNQYLMALCE
GKKIVGTSHCTLMPSLTFTGSTRMQIEAVRVCQSARGKNIGQQMIEFAIGWGKNHGATIF
QLSTNKERVDALRFYEKLGFNATHEGMKLYLEQE

>gene_910|GeneMark.hmm|165_aa|-|6337|6834 >NVVL01000048.1 Rickettsiales bacterium isolate NORP64 Contig_source1382A_23573, whole genome shotgun sequence
MYAGLKYQEFVRKMSNEIHTLARAVILDDEHILLCKTLDLPVNFYFLPGGHVEHGESASQ
ALMRELKEETGKDCTVKRFLGCLEYSFEPGHNSICHNHEYNFIFEAESDQLKVREKISSP
EKHIELIWVAVSDLSEIDFRADPLKKLLPEWLSRDAHNVFRSEMI

>gene_911|GeneMark.hmm|356_aa|-|6897|7967 >NVVL01000048.1 Rickettsiales bacterium isolate NORP64 Contig_source1382A_23573, whole genome shotgun sequence
MQAVIIAGGKGSRLWPASYKNHPKPFVRMNDGLSLIQKTLLRAGCVSLVRSIIIVTRHDY
VPRIKAEYEEIAHLMPKGVDLSFIEEPFGKNTAASIAAAALSLSAKNSEDEAMLVLPIDH
IIADQEGFNEAVSKAAEIAKNGQENSAGKGQLVTFGIKPTHAESGYGYIEFSGNEVLGFT
EKPSKETAEKYIKHGNFYWNSGMFCFTARTILQEMHDHCPSVLSTVRRALETSKRNVNGD
ISLDVSSFSLVSEVSIDYALMEKSSNISVIPCDIGWRDVGTWEHFSHQTQADAKGNRLRG
DVITHNASNCYVDSNGITIGVVGLNDVAIIHTERGLLVINKNSSEDASAIYDKTET

>gene_912|GeneMark.hmm|358_aa|-|8370|9446 >NVVL01000048.1 Rickettsiales bacterium isolate NORP64 Contig_source1382A_23573, whole genome shotgun sequence
MKQRITKLGTLGTQYFRSFTGSNLRFKAAGFSVTPYSGVNSRDSSNSINNWGHTTQWKEF
HTDSQILSGKTDPFSNQKTNSSSLATQQPAIDQTPKKPAADLSTVPDAQEQGRSNGGGGE
PYNWEPWLGLTALALGIFNVWQALKNRAESREYEEKLSKEKAREQLKSDLKELSKKCSNN
TIEDWPLSIENLLDFIKKNYSLDPSTLAKLLSDYDVDRLAYIIASYCNSGHPDRVEAREK
LEFARKLIASKCNCTLDEVKVPDLFRHHCFSELYIELTGMLSKTYIYPDNTESDIKHSIF
YVKKAYSLAQQHYKMTNEPKFWDKLLSEQNFQLLTIHGDQFVDNPTFLAGDDAVPNDS

>gene_913|GeneMark.hmm|159_aa|+|10089|10568 >NVVL01000048.1 Rickettsiales bacterium isolate NORP64 Contig_source1382A_23573, whole genome shotgun sequence
MTKDTAKASENINVKLVEGLLSKTDPSELFGKGGMFAQLKKQIVEKIPPSEMEHELGYSK
HSKISKLNSNRRNGSYEKTILDDEGYKLAAEIPRDRLGAYEPKLIPKGVRRFSGFDDKVI
SLYSRGMTMSEIQGHLEEIYSTEVSKELISTVTDSAIEE

>gene_914|GeneMark.hmm|109_aa|+|10599|10928 >NVVL01000048.1 Rickettsiales bacterium isolate NORP64 Contig_source1382A_23573, whole genome shotgun sequence
VYPILYLDCIHVKARDNHTIINKAVYLAIGVNMEGRKELLGIWVGKNEGSKFWMQVVTEL
KNRGVQKIYVASVDGLKGFPEAINSIFPDTIVQLCIVHMVRNCCEICVL

>gene_915|GeneMark.hmm|104_aa|-|11009|11323 >NVVL01000048.1 Rickettsiales bacterium isolate NORP64 Contig_source1382A_23573, whole genome shotgun sequence
LFKIAANKFELVQCHGPISDRHCPFFCSVSKCKKYYLFDRVIVWKYPLVFDDFSDLTIDR
FNCIRRINNLPDFSRKCKKWDYTSPIPFPYARDYWVFAIPLILK

>gene_916|GeneMark.hmm|154_aa|-|11403|11867 >NVVL01000048.1 Rickettsiales bacterium isolate NORP64 Contig_source1382A_23573, whole genome shotgun sequence
MANHSATKKAIRKTVSKTEINKTRKSRIKTYIKRVITAVEAGSSEESNKALVEAQSEIMR
GVANNLMKKNTAARKVSRLSKKVKAISTGAAVGTAKTTEQKATKPKTVSPEKATAEAGAT
GTKAKPAAVKKPATTKAKPAVKKEAEKKSDTASK

>gene_917|GeneMark.hmm|137_aa|-|11880|12293 >NVVL01000048.1 Rickettsiales bacterium isolate NORP64 Contig_source1382A_23573, whole genome shotgun sequence
MRHKIKGKKLNRTSSHKKAMFANMATSLVMNEQIKTTVPKAKALRPYIEALVTKARAATL
SARRDIISRIKDKKAAEKLMSVLAERYKERPGGYTRIIKAGFRYGDKAPIAYIEFVDRDL
SAKGCMTPKVEETENQE

>gene_918|GeneMark.hmm|339_aa|-|12449|13468 >NVVL01000048.1 Rickettsiales bacterium isolate NORP64 Contig_source1382A_23573, whole genome shotgun sequence
MLSLNKNWSSLIKPSKISYDGKEEKSNVANIIIEPLERGFGLTIGNALRRILLSSLQGAA
ITAVKINGAVHEFSAKSGVKEDIVDVVLNLKKISIKQHSTDRKTLRINVKGPCVVTAGMI
ETGTDVEVLNPNQKICTLSKDAELEMELYTEIGRGYIPAGSAKDHDFPLGVITIDALFSP
VKRVAYKVEDTRVGQVTDYDKLLMSIETDGTISPEMAVALAARIMQDQLQLFITFEEEEE
EKLESKEELEYNPILLKKVDELELSVRSRNCLQNDNIVYIGDLVNKTEAEMLRTQNFGRK
SLNEIKDVLQKFNLKFGMEVSDWPPENIEEIAKKYEEPY

>gene_919|GeneMark.hmm|127_aa|-|13487|13870 >NVVL01000048.1 Rickettsiales bacterium isolate NORP64 Contig_source1382A_23573, whole genome shotgun sequence
MTPINKVRKKKKNITLGIVHIKATFNNTIVTVTDVQGNVIAISTAGAHGFKGAKKATPYA
AQITVDKVSEMAKEHGLKTVSIKVKGAGSQREAAMRAVFSQNFVVISITDVSGVAHNGVR
APKRRRV

>gene_920|GeneMark.hmm|122_aa|-|13887|14255 >NVVL01000048.1 Rickettsiales bacterium isolate NORP64 Contig_source1382A_23573, whole genome shotgun sequence
VARIAGVNIPPNKRLVISLTYIHGIGLTSSQKICRSTKISESRRVKDLDDKELVALRAEI
DQNFQVEGDRRREVSLNIKKKKDIRTYEGLRHIRRLPVRGQNTHSNARTRKGKAVAIAGK
KK

>gene_921|GeneMark.hmm|215_aa|-|14316|14963 >NVVL01000048.1 Rickettsiales bacterium isolate NORP64 Contig_source1382A_23573, whole genome shotgun sequence
MTKKIIVLLGLPGSGKGTQGALLSEELVIPHISTGDVFRKMVLEDSEDSKTLAACMKEGR
LVPTGLVNKTIRKYILSDECRDGCILDGYPRTLDQAEYFIENIDATVNVIFFDLESDVVV
KRILGRISCSSCGRIYNEHFDKPKKKGVCDNCGSEEFSARSDDTESAILSRIAEYEKETL
PMVEYYQKKGRFFTVNAGKSKQKVVEEIASIVKKI

>gene_922|GeneMark.hmm|428_aa|-|14964|16250 >NVVL01000048.1 Rickettsiales bacterium isolate NORP64 Contig_source1382A_23573, whole genome shotgun sequence
MQKSKGELSSRILFTLSVLLVCRIGSFIPIPGIDSIALASLAERSQGGILGMFNMLSGGS
LERMSIFALAIMPYITASIVIQLMTIAYKPLENLKKDGEVGRRKINQLSRYLTVVFASFQ
AYGIAIGLGATVTPYGPIVIIDPFFFKVSAVTTLVVGTMFLMWLGEQISARGIGNGTSLI
IFIGIVSGLPSAVIGMFELSRKGAMSPVAVMAICAGVLAMITVIIYFERAQRKVLIQYPK
RQVGNKIYGGDATHMPLKLNTSGVIPPIFASSVLLFPATISEFSSKDSEIMGMVSRYLGH

GKPLFIFLYIALIMFFSFFYTAVVFNSEETAENLRKNGAYVPGRRPGKNTAEYFDYLLTR
LTVLGSLYLSVVCIVPELLMNKYSVAFALGGTSFLIVVNVVLDTFAQIQTHLFSARYEGL
VKKMKLRN

>gene_923|GeneMark.hmm|149_aa|-|16264|16713 >NVVL01000048.1 Rickettsiales bacterium isolate NORP64 Contig_source1382A_23573, whole genome shotgun sequence
MRLNTLFNVEGSKKPRKRVGRGIGSGTGKTCGRGVKGQKSRSGVAIKTEGGQMPLIKRMP
KRGFSCSKAVNYTAISIADIEVMIEEKRIDAKGSVNKELLVSIGYIKSDKVPVKLLGGLE
KASHKLTIELDACSASAKAVVEAAGGKVL

>gene_924|GeneMark.hmm|69_aa|-|16730|16939 >NVVL01000048.1 Rickettsiales bacterium isolate NORP64 Contig_source1382A_23573, whole genome shotgun sequence
MTEKKIKSKSKSLIKVTQIGSPIGRSSDQRKTLIGLGLNKLRKSRLLEDTPSVRGMAEKV
GHLLSVETV

>gene_925|GeneMark.hmm|172_aa|-|16974|17492 >NVVL01000048.1 Rickettsiales bacterium isolate NORP64 Contig_source1382A_23573, whole genome shotgun sequence
MSKYTRSNDSIIEDLVHVNRVTKVVKGGRNFSFAAIVIVGDEKGKVGYGNGKAKEVTEAR
AKANKDARKNMISVPLYKGRTIHHDVIGVSGAAKVVLRRAAPGTGVIAGGPLRSIFGRLG
LEDIVAKSLGSSNPQAMISATFNALKSLNSPKSIAKRRGKNIAELSVNAASA

>gene_926|GeneMark.hmm|118_aa|-|17505|17861 >NVVL01000048.1 Rickettsiales bacterium isolate NORP64 Contig_source1382A_23573, whole genome shotgun sequence
MSAKNHSFKRRKERVRVKLKKVSDRNRLSVFKSGRHIYAQIIDDSTSRTLVSASTLDKEI
RVTAKSNCNVEFAVKVGKLLADRAAEKSINLVAFDKGGHKYHGVVKALADEARKKLQF

>gene_927|GeneMark.hmm|177_aa|-|17868|18401 >NVVL01000048.1 Rickettsiales bacterium isolate NORP64 Contig_source1382A_23573, whole genome shotgun sequence
MSRVGKLPVSIPEGVKVDIKGLNIKIQGAKGALERTFAGQISIEQKDGVVIVKPLDDTTP
ARAMWGTARSIINGMMKGVTVGFQEELEVIGVGYRAAVKGKYLNLTLGKSHNTKIEIPDF
IKVSTPKQTIIMLESCDKEKLGQYVAVVRSQRPPEPYKGKGIRRKGEYVQRKEGKKG

>gene_928|GeneMark.hmm|130_aa|-|18414|18806 >NVVL01000048.1 Rickettsiales bacterium isolate NORP64 Contig_source1382A_23573, whole genome shotgun sequence
MSMSDNIADMLTRIRNGQKSKLLNVGLPLSNLKCAVLDVLEKEGYISGYTKDAESKTIDI
SLKYSRVGEGAICEIHRVSKPGKRMYSSIGDLPGYFNNMGIHILSTSHGVMSDREAKKLN
VGGEVICKVF

>gene_929|GeneMark.hmm|101_aa|-|18834|19139 >NVVL01000048.1 Rickettsiales bacterium isolate NORP64 Contig_source1382A_23573, whole genome shotgun sequence
MARTGSIETNNKRKRLAGSFKAKRDALSAKIKQKDLPLEERFELVMKLAKLPRNSAQIRV
RNRCALDGRPRGFCRKMGISRNKLRELAAKGLLPGIVKASW

>gene_930|GeneMark.hmm|180_aa|-|19166|19708 >NVVL01000048.1 Rickettsiales bacterium isolate NORP64 Contig_source1382A_23573, whole genome shotgun sequence
MLLRFKELYSKNIVKDLQEKFKYPNIHQMPKLTKIVVNMGVSDAVSDSKVINHAISDMTA
ITGQKPYETYAKQSIATFKLREGMKIGCKVTLRRARMFEFLERLVLIALPRVKEFKGLSV

KSFDGRGNITFGIKEQIVFPEINYDKIDKIRGMDITIVTTAKTDEEAKALLSGFQIPFYN

>gene_931|GeneMark.hmm|109_aa|-|19708|20037 >NVVL01000048.1 Rickettsiales bacterium isolate NORP64 Contig_source1382A_23573, whole genome shotgun sequence
MHKLKIKKGDTVQVTTGKNKGKVGDVTKIFPSDNKAIVAGVNLVKKHTKPSQTSEGGIIQ
KELPIHISNLSHIDPKTKEITKIGYKTLSDGKKVRFAKKSGEIISKEGK

>gene_932|GeneMark.hmm|122_aa|-|20039|20407 >NVVL01000048.1 Rickettsiales bacterium isolate NORP64 Contig_source1382A_23573, whole genome shotgun sequence
MIQMQSILTVADNSGAKKVMCIKVLGGSGHMVAGCSDVIVVSIKTAIPGGKVKKGEVHRA
LIVRTKKGVRRPDGSLIQFDSNSVVLVNKQNEPIGSRVFGAVPRELRGKGFMKIISLAEE
VI

>gene_933|GeneMark.hmm|76_aa|-|20480|20710 >NVVL01000048.1 Rickettsiales bacterium isolate NORP64 Contig_source1382A_23573, whole genome shotgun sequence
MPKRILQGTVVSAKSEKTVSVEVERRFRHPLYKKTVRKSTKYAVHDENSRCSEGDKVKIQ
ECRPLSKTKSWVIVEE

>gene_934|GeneMark.hmm|74_aa|-|20703|20927 >NVVL01000048.1 Rickettsiales bacterium isolate NORP64 Contig_source1382A_23573, whole genome shotgun sequence
MSKSEFTSKVLSQVTLEKLVEKLVFLKKELFNLRFQKTLGELTNTSRFLKVKKDIARVGT
ELTRRRKTGAKADA

>gene_935|GeneMark.hmm|136_aa|-|20920|21330 >NVVL01000048.1 Rickettsiales bacterium isolate NORP64 Contig_source1382A_23573, whole genome shotgun sequence
MLAPKKQKFRKAHKGRVASKSKAGTMLNFGSFGLKSLDGLRVTARQIEAARRAAVRHMKR
QGRFFIRIFPDLPVSKKPNEVRMGKGKGAPEYFAVRVSPGRIMFEIEGVDEVVARQALDL
ASAKLPVRTKMVQRYE

>gene_936|GeneMark.hmm|217_aa|-|21345|21998 >NVVL01000048.1 Rickettsiales bacterium isolate NORP64 Contig_source1382A_23573, whole genome shotgun sequence
MGQKVNPHGFRVGPTLFKDWDSVFYAERKDYSEKLIQDLKIRALIMKRYKIANVSSVVIK
RHSNNVVINISAKKPGIIGKSGSDIEQLKKDIQKISSTEVFINIHEIKKSGIDAMVIAQ
TIAQQLEKRSSFRKCMKTAIQNCFKQGGKGIKVACSGRLGGAEIARTEWYREGRVPLHTL
RANIDYGTEEANTTYGVIGIKVWVYKGDFAQKKDKVN

>gene_937|GeneMark.hmm|116_aa|-|22000|22350 >NVVL01000048.1 Rickettsiales bacterium isolate NORP64 Contig_source1382A_23573, whole genome shotgun sequence
MSDLLAKASIRRLRTSPQKLNLVAAMIRNMKASEALAQLAFSPKRIAVDVRKCLQSAVAN
AENNIGLDIDNLVVTSATVGRSLVMKRIRARARGRSARIMKPFSNLYITVSEREES

>gene_938|GeneMark.hmm|92_aa|-|22367|22645 >NVVL01000048.1 Rickettsiales bacterium isolate NORP64 Contig_source1382A_23573, whole genome shotgun sequence
MTRSVWKGPFVDGYLLKKVQKAMDSGRKEIIKTWSRRSTILPMFVGYTFAVHNGNKFIPV
AVSEEMVGHKLGAFAPTRTFYGHGADKKAKRK

>gene_939|GeneMark.hmm|274_aa|-|22660|23484 >NVVL01000048.1 Rickettsiales bacterium isolate NORP64 Contig_source1382A_23573, whole genome shotgun sequence
MALKKYNPTTPSQRGLVRVDRSELWKGAPHKPLTKGLSKSGGRNNLGRITSRHRGGGHKR
LYRMVDFKRCKLDSVATVERIEYDPNRTAYIAFIKYEDDTVSYILAPAKLVAGDKIISSA
DADIKIGNCLPLANIPVGTIVHNVEMKPGKGGQIARSAGTSVSIVGKDSGYAQIKLASSE
VRLVPLECRATIGTLSNAEKKNTIVGKAGRSRWMGKRPHVRGVAMNPVDHPHGGGEGKTS
GGRHPVTPWGKPTKGKKTRKNKLTSKFILKRRTK

>gene_940|GeneMark.hmm|98_aa|-|23486|23782 >NVVL01000048.1 Rickettsiales bacterium isolate NORP64 Contig_source1382A_23573, whole genome shotgun sequence
MKADKKYDLIRRPIITEKSTIAGEQGKYVFEIAANADKPSVKRSIEAIFEVKVKSVNILN
QNGKVKRFKGTMGRRSDVKKAIVTLEKDHTIDLAGGMK

>gene_941|GeneMark.hmm|206_aa|-|23782|24402 >NVVL01000048.1 Rickettsiales bacterium isolate NORP64 Contig_source1382A_23573, whole genome shotgun sequence
MKAELVSFEKKVVGEVSLDSSIFGLEARVDIVKRVIDWQRAKARAGTHSVKTVGEVSGTT
RKPFKQKGTGNARQGNARGVQRRGGGIAHGPQVRSHDTKLQKKVRRLGMRHALSIKFSEG
AVHFVDSVDMTKPKTSELVKTLSNFGKGRFFVIDGADLNANLKLSVASVENASAVPAIGA
NVYDIIRSDHILISKNALSLLEERLK

>gene_942|GeneMark.hmm|214_aa|-|24436|25080 >NVVL01000048.1 Rickettsiales bacterium isolate NORP64 Contig_source1382A_23573, whole genome shotgun sequence
MRTGLITKKLGMSSMFTDQGERITLTFLKLEDCLVVGQKTMDRDGYNALVLGAILRKVSR
VSKPMKAIFANAKIEPRAKLKEFRVSDSNMLAIGSELKPSHYEIGQYVDVSSRSIGKGFA
GVMKRHNFAGLEATHGVSISHRSHGSTGQCQDPGKVFKGKKMAGHMGDKNITIQNLQVVE
IDDVNGVIVVSGNVPGSKGGYVFIKDAVKKSVAA

>gene_943|GeneMark.hmm|105_aa|-|25082|25399 >NVVL01000048.1 Rickettsiales bacterium isolate NORP64 Contig_source1382A_23573, whole genome shotgun sequence
MNNQKIKIKLKSFDHRSLEHATSEIVSAVKRTGADISGPIPLPRRIERFTVIRGPHVDKK
SREQFEIRTQKRLVIINYPTPQTVEALRKIDLPAGVDVEIKLVGA

>gene_944|GeneMark.hmm|394_aa|-|25511|26695 >NVVL01000048.1 Rickettsiales bacterium isolate NORP64 Contig_source1382A_23573, whole genome shotgun sequence
MAKEKFDRSKPHVNIGTIGHVDHGKTSLTAAITKVLASKGKGINKQVKYDEIDGAPEERE
RGITISTAHVEYETEGRHYAHVDCPGHADYVKNMITGAAQMDGAILVVSAADGPMPQTRE
HILLAKQVGIEHMVVFLNKVDQVDDEELLELVEMEVRELLSAYKFPGDDIPIIRGSALRV
LEDKETECIDKLMEAVDSYIPTPKRETDKPFLMPIEDVFSISGRGTVVTGRIEQGIVNIN
DELEIVGIKDTEKTTCTGVEMFRKLLDSGEAGDNVGVLLRGTKREDVVRGQVLAKPGSIT
PHTEFEAEIYVLTKDEGGRHTPFFKNYRPQFYFRTTDVTGEVELPAGKEMIMPGDNTKIV
VKMISPVAMEAGLRFAIREGGRTVGAGVVSKVIK

>gene_945|GeneMark.hmm|252_aa|+|27190|27948 >NVVL01000048.1 Rickettsiales bacterium isolate NORP64 Contig_source1382A_23573, whole genome shotgun sequence
MKNNKKIGNSANNRKASSLNKGQYYMYGKHGVLAALKNENRTVRSVYCLEKQARELKNEL
HGTHVEIVQNNFITEKIGKDQAHQGIIALVSSIFKNNIEELTFTPGKDRIVILDQLSDPQ
NIGAIIRSAAAFGINKLILPSDNAPEESASIAKAACGCLELMQIAKVTNIKNSIDKLKKM

GFWIAGLDGQGKENMNDVMEIEKLAIIIGSEGKGMRRLTSETCDFLVKIPISEQVESLNA
SNAASIIFYLLR

>gene_946|GeneMark.hmm|237_aa|+|28108|28821	>NVVL01000048.1 Rickettsiales bacterium isolate NORP64 Contig_source1382A_23573, whole genome shotgun sequence
MIGNLLNKFKKQPELGALDAQNNQAPAGNAKNWYDERYDTIIVQRNIFLLLLVILLVLSI
ISIGVVAYVINTKRFDPFVIQIDDSTGIARIVNPLSSSVLSGNEALAQYFIKKYIIARET
YNPVDFDTLAKKTVRLLSENSVYWGYIGYLRNNDINPSIQYGQKNTTYLLVKSWSKLSGE
KYIVRFSINETSGARKSFSKIAVVDFVYTPMELSDDDRDVNPVGFQVTGYRVDDDSS

>gene_947|GeneMark.hmm|137_aa|+|28808|29221	>NVVL01000048.1 Rickettsiales bacterium isolate NORP64 Contig_source1382A_23573, whole genome shotgun sequence
MIVVRIILASMLILAASCGLAYSNDVPLTTDSRIKTYLYSPNEVYLLVLHYGFQSHIEFG
KNEEIETITVGDSYAWKITPLNNRLFIKPMERNIRTNMTIITNRKIYQFDIVAKELESGS
EKDLVYVVRFQYPKKSR

>gene_948|GeneMark.hmm|473_aa|+|29225|30646	>NVVL01000048.1 Rickettsiales bacterium isolate NORP64 Contig_source1382A_23573, whole genome shotgun sequence
MSKETNPASSAPPVKTSPKVEQDLSQVAINPKQNILILVGIVAVFIYLSFNLFAGDDGNE
QKPEPTPIPKEITRPVQLLANDDAPPIPTLPEPPKLEEPTPPPPPPPEPEALLPLPLPEL
PSVTTPQNAPPLPFLQPKTDESARRADEKRKSGIILMGGAPPAKTEDQLLEESNFKYRGN
MNLVLGRGKILSAIIETAVNTDFGGEIRALISRDVYSEWGKNILIPKGSRIFGSYATGIN
GAYGRISITWTRVDLATGYTLNLAGTGIDALGRTGNQGRVDNKFKERLSNAVLKSILDIS
LAKALDSIVKPQINSQTAAARSVGATNLTSIANAIFTQPGLTAKEKRAKICQDVLTAIAN
KTSNSFLKLSMTCNTLNTDPVASEPNKLLSLMGIINQLSDELIKDVTADSEKTQVQEASE
KSFEAISDSIKSMAEEQDFHPTITIPQGTAVKIYVNKDYKFPKKAVIKSRFLK

>gene_949|GeneMark.hmm|332_aa|+|30643|31641	>NVVL01000048.1 Rickettsiales bacterium isolate NORP64 Contig_source1382A_23573, whole genome shotgun sequence
MNYAALETYLLPFKNIFAEEGVNELMINKPGEVWVEKAGDQRLELIPEIDIDHMLGLGRL
VAQSTAQTISEETPLLSATLPNGYRIQIVFPPAVEPSLVAFAIRKGSTVHFSLDKYAEMG
AFDNTSTKEMTDENNAILSNHLKTGHFKEFIRHAVLCKKNLIISGGTSTGKTTFTNAALG
EIPSHERLITVEDAREVMLPNHPNKVHLLGSKGGQGRAKVTTQDLIEACLRLRPDRIIVG
ELRGAEAFSFLRAINTGHPGSISTLHADTPAMALEQLKLMVMQAGLGLPPEEVKKYIMAV
VDIVVQLKRGAGGKRYVSEIFFKGMEGGSDGI

>gene_950|GeneMark.hmm|586_aa|+|31631|33391	>NVVL01000048.1 Rickettsiales bacterium isolate NORP64 Contig_source1382A_23573, whole genome shotgun sequence
MEFSKLLRMARTLVGHVLIHPFVLFCTFWVSGAIIAMFTNEYELLSSEIFGGIIVLYKWG
IWLENSWGQLSFTSYDYIKIKLAAAITVPSFLVLVYYIKNFTRIRNLEFFLPKEAVYGSA
AWASPTEIERASLRAKHGMMLGKDAEGYFVADGFQHALLFAPTGSGKGVGFVIPNLLFWD
HSVIVHDIKLENHQITSGWRKKKGQEVFVWEPSNPDGITHCYNPIDWVSSKPGQMVDDVQ
KISNLIMPEKDFWNNEARSLFLGVVLYLLADDTKVKSFGEVVRTMRSDDVVYNLAVVLDT
LGDAIHPVAYMNIAAFLQKADKERSGVVSTMNSSLELWANPLIDTATASSDFSIMDIKKK
RTTIYVGLTPDNIHRLQKLMQVFYQQATEFLSRKMPDVKEEPYGVMFLMDEFPTLGKMEQ
FKAGIAFFRGYRVRLFLIIQDTQQLKGIYEDAGMNSFLSNSTYRITFAANNYETANLISQ
LCGNKTVEQTSHNKPLFFDLNISTRTQNVSKVQRALLLPQEVIQLPRDDQIVLIESFPPI

RSKKIKYYEDKFFTKRVLPPTFVPTQEAFVINNGTNEEEKNKKETE

>gene_951|GeneMark.hmm|467_aa|+|33460|34863 >NVVL01000048.1 Rickettsiales bacterium isolate NORP64 Contig_source1382A_23573, whole genome shotgun sequence
MKKKKMRSATIDIGYNAIRATVYEDNTLGAPEIFSSKFKSDLIGLLANQSIDMKHQTYLS
IQYLLHVFKRLNVTDIKCVATAVLRDHEKAEQFIQHIRDKYNFDIEVISGEKEANLTAFG
LVKGIRNCKGIAADLGGGSLELAEVGDGKISKLCSLALGTKILSMRNLADENEIIDIIKE
QYSSSEQESLYLIGGSLRFIGKSYMNSAFHYVKNLHNFEIEAGSFFEYLNAVQTSDGEIS
KLSSRRINNNAVLILKALIHLFNPKKIIVSTYGLKEGVRIDSMDEEEKQKDLILEKVKYL
CTYDTDATDFDSYYKIISPLFSAGVDLNEILKLAIMLLSMESQFDSTLPPTALSDSILSS
EIPFAYKMRVMLAMIIAYSTNHKPSVNLIKFSKKTLTQEEHNNAQIIGYFLYITKEIDGP
IFMTPSFDIKSCNYFLEIDTHEILPRAIFEKVCMKLKSIAFARRTGY

>gene_952|GeneMark.hmm|176_aa|+|34963|35493 >NVVL01000048.1 Rickettsiales bacterium isolate NORP64 Contig_source1382A_23573, whole genome shotgun sequence
MLKNTENSYGSITKIFHWVVALAIISLIAVGFTMHYMEPSPEKFELYGMHKASGVLVFML
VALRISWRFINQTVLPAKDLPNMLKLGAKAGHFMLYIFMLVMPISGITMSIFAGYDIPVF
GLFTIPAMEKNLQIAGIAHTLHEIAAWGFAIMIVLHILAALYHHFIRRDDTLKKMV

>gene_953|GeneMark.hmm|204_aa|+|35540|36154 >NVVL01000048.1 Rickettsiales bacterium isolate NORP64 Contig_source1382A_23573, whole genome shotgun sequence
MNFTINDIPESIAASMRSKAVWTPECPVPIEHLKLIEISHYDFAGDIKEGQMIVLDKISE
SVIKIFTELFARSLQVHSIKLMDEFNGDDNLSMAANNSSSFNFRKIEGSTLLSMHSYGLA
IDINPVQNPFIMIDEDTHNVRVLPKKSSMFLNRSNQRAGMAEPIIEIFKKHGLSVWGGNW
NNPIDYHHFQVPREDVAELMIGIS

>gene_954|GeneMark.hmm|375_aa|-|36200|37327 >NVVL01000048.1 Rickettsiales bacterium isolate NORP64 Contig_source1382A_23573, whole genome shotgun sequence
MKRHVFLYGFSIFSMFFGSGNLVFPLMVGVNNLDNWLAGFLGFFLTGIILPFLGLFVIKL
RKGSYVDFFAEAGVVAKYAIPLFTLSLLGGFGVIPRCITVAHGGLEFLFADLSLVLFSAI
FCIACFFICLNDKWMFTVLGKFLTPILLFFLIILITVGIYESQGLEGVGASFGDNFIDGF
FRGYATMDLFAAFFFSSLIFKQIEALVDETEGQSLIKAALAPSIFGGCLLSLVYLGFVYL
GSHYAFLATDLPGELILPAIAKHLLGTNGALVIAVIIIFSCLTTAVALNSIYARYLCSFE
IIGEKRFPILLFITTMIAFFISLFDFDGIAAMLAPLLTISYPGLIALTILSICTKDFKRL
KMVSFYGITILMLLL

>gene_955|GeneMark.hmm|340_aa|-|37679|38701 >NVVL01000048.1 Rickettsiales bacterium isolate NORP64 Contig_source1382A_23573, whole genome shotgun sequence
MCLAVIVMIFIGGLTRLTESGLSITEWNPVSGALPPQNGAEWGQEFEKYKKSPEYLQHNM
GMSISEFKSIYMLEFVHRLAGRFTGLLYLLPLMIFWLMGSINKRDSGTYLFILFLLALQG
AMGWYMVKSGLVADPCVCHYRLAAHLMLAVFMYILLFWQLMRNSFDILLIPGLAPGGVPG
EALGVVSLCRQKFWCTLSIFLLLIQIMLGAFVAGLDAGLVYNSFPLMGEGVIPHEVSLSS
VNLASFSEPVFIQFIHRMMAYMLFFVVCIFCFVGARLRHPKFTRSIVCVFAALSLQMIAG
VVTLLYSVPIPVALLHQLGAIILLSFLLWAYFLLKSTESE

>gene_956|GeneMark.hmm|344_aa|-|38800|39834 >NVVL01000048.1 Rickettsiales bacterium isolate NORP64 Contig_source1382A_23573, whole genome shotgun sequence

LNLIEEIHSYLSREISAMNAVIVENLAVEEELITLISNHLSKAGGKRMRPILTILSAKML
GDAGEPGSSGEPDDAGEVLGSESSAIKLAAAVEFIHMATLLHDDVVDGSKMRRFLPSANV
VWGSRASILVGDFLFSQSFKLIVSTGSIPALRALSSASAVIAEGEVSQLAHLEQKSMVSE
AEYLKIIGAKTAELFGASCEVGAIIAGKESSAKTMRDFGICLGKIFQITDDMLDYFSDSA
KVGKNVGDDFFEGKVTLPLILLSKKISADDNLSLQKMLQAESRTSEDFLRVKTLMQENDI
NTEITNYLAELEQTALVLLRSLSIQNQYCKFLENLVEFAINRTY

>gene_957|GeneMark.hmm|585_aa|-|39870|41627 >NVVL01000048.1 Rickettsiales bacterium isolate NORP64 Contig_source1382A_23573, whole genome shotgun sequence
MKLIRFIVLVLLLIAAAIGAGGYYVTHKVADELNEQYAGQKIAVTENGKASHFITFKEVV
PSGFPLKISWDVRGVIEETESSKTSFTSPVQFGYDLLLQKVFVSYAGDIMSSYKPAKFKF
GAKLKIEDYMLSVDLPVSMGLINTLRDLEDPVQIINHVGDINLSSDRVEIFDLIDNQKFY
DKKYENMKLSFVPQKTYVSLKDLMTNIPQRYVVRYAVETNEIKVPTRKLPDSLFYVFSAV
PSGMDMRANAVIKTAGNNSREITKNLEVKGDITCSGMFASVKNFKLDYKAGSDKLNRDYD
LAIDSKLHLKKGAFDHLFEQYKLISSKVALSPSAKMIDREINYIIANKQAFKFQDLENSD
YDFNLKMSNYHDQDKTYMKINNLDISSEGAGINLKHEIETNSKGFKTYLAKGDLLIRNYS
AVVEFTSGYIYRFGKFRFLNDAARSLYVDVNKTFLKSISDHPKSVSNDLSFNYEVNSRNL
KKTKLGSVTVDKISELYTLMLYKKLFDNVGSGGDVLERMQKILPGIDGKEPLLQKILPHI
SGGKSIEKSIKKEIDKVLPKNAQDLLKKIIPKDKLKKGFLKDLFK

>gene_958|GeneMark.hmm|87_aa|+|257|520 >NVVL01000049.1 Rickettsiales bacterium isolate NORP64 Contig_source1382A_23578, whole genome shotgun sequence
MFIHAGENWNTWQEEDKLHACGLATKGMGEAIALFHTYEKEVRQLCVGCGYSGRRVDILV
DALPTVVWICQPCLTLHERRVHTVDIR

>gene_959|GeneMark.hmm|49_aa|-|618|767 >NVVL01000049.1 Rickettsiales bacterium isolate NORP64 Contig_source1382A_23578, whole genome shotgun sequence
MSKGKKDIVICGKNYGDVIIGCNGTIMGRRRRKKSKPFTKAGKCKIVVG

>gene_960|GeneMark.hmm|155_aa|-|811|1278 >NVVL01000049.1 Rickettsiales bacterium isolate NORP64 Contig_source1382A_23578, whole genome shotgun sequence
MPVYIRVLYDAIPHQTRHNLKVVIMSKHGTILKKGEKESRTCMVVDNVVTLHHVTRHGQD
VDVVMDWGFDFTDVPTSKLMELASRSLVIDCRNAWKKQSEPIATGEEQTSQVFLVSDLLT
KQKRGKTVSEKVGALVEGMSPEDIAALLKQVTAAE

>gene_961|GeneMark.hmm|66_aa|-|1288|1488 >NVVL01000049.1 Rickettsiales bacterium isolate NORP64 Contig_source1382A_23578, whole genome shotgun sequence
MSKSELLKALATYPDDAQIVVSFDTRTIAKITHGMDYVTFGIDCVEDGWKPSNVADISIG
NDLIMG

>gene_962|GeneMark.hmm|52_aa|+|1978|2136 >NVVL01000049.1 Rickettsiales bacterium isolate NORP64 Contig_source1382A_23578, whole genome shotgun sequence
MASTEKHPRTPGDARKMLAQRKKARGMISKDQKLRSDMLRELLMLEKKGEAK

>gene_963|GeneMark.hmm|38_aa|+|2138|2254 >NVVL01000049.1 Rickettsiales bacterium isolate NORP64 Contig_source1382A_23578, whole genome shotgun sequence

MKRMRRAFTMTVMKRLKEYKDKVIVRKAKEDKKNATGE

>gene_964|GeneMark.hmm|57_aa|+|2238|2411 >NVVL01000049.1 Rickettsiales bacterium isolate NORP64 Contig_source1382A_23578, whole genome shotgun sequence
MPQVNEQLDKLWSNDIPEDTNINAEGLKLGLVKAEVADGVNNAGVHQVDNKGASCLK

>gene_965|GeneMark.hmm|89_aa|+|2399|2668 >NVVL01000049.1 Rickettsiales bacterium isolate NORP64 Contig_source1382A_23578, whole genome shotgun sequence
MPEVTLRGREKSGGGQAGEVFVQGKGILSAIWTGGGTGSAPDPEDYELADIGADLLGIAT
GVTYHIVGAVGSKSWVATGGTVQNLYGGA

>gene_966|GeneMark.hmm|92_aa|+|2668|2946 >NVVL01000049.1 Rickettsiales bacterium isolate NORP64 Contig_source1382A_23578, whole genome shotgun sequence
MHPIRAVRNYLDGRKSGKPCRINAKDYPRSDYSSYWVYFINSETFQWRVDFIDYSTGDTV
RLFGKAAKNANKARILAQKCVLKNIEQYRRVS

>gene_967|GeneMark.hmm|132_aa|+|2946|3344 >NVVL01000049.1 Rickettsiales bacterium isolate NORP64 Contig_source1382A_23578, whole genome shotgun sequence
MSLGFITALRNNMLDEITALIDAGADGLVRIYDGTQPATGGAVTTLLAEGAFSTNSAPAA
SGGVLTHSAIGDEASAPNGGTPTWYRIVDSTAAFVMDGTAGTSGTDMILDAATITAGQTV
SFNSSTITAGNV

>gene_968|GeneMark.hmm|142_aa|+|3344|3772 >NVVL01000049.1 Rickettsiales bacterium isolate NORP64 Contig_source1382A_23578, whole genome shotgun sequence
VSKLSDEITNDPLGRGYAGMDAEQVAFSLTEVKDRPVDNDESIDNIKMYLLRKRKYIVIK
DSPELAAREFMVLLTEFEQLLFDNPGKRKMFKDILSELVDAGLLTSPMRTALLGFVSFTG
TRSQELGLGRVRAGDVKESRNT

>gene_969|GeneMark.hmm|156_aa|+|3772|4242 >NVVL01000049.1 Rickettsiales bacterium isolate NORP64 Contig_source1382A_23578, whole genome shotgun sequence
MAIGTDATIIVLGTTDVADDTSTSAIVDGVMSVAADITAWTNDDDAPFANFILNWQSAAT
TLTGNIFLHVRPINIDGANDSPIPTTADPIGRAGVFEIDGAKAINTDHPYLASIDLRPFT
MKTSQEFEFYLFNNTGETIAANWDLDVVPLSFNGAA

>gene_970|GeneMark.hmm|266_aa|+|4242|5042 >NVVL01000049.1 Rickettsiales bacterium isolate NORP64 Contig_source1382A_23578, whole genome shotgun sequence
MPYALPDSRIENASLKSYDTVPVGRYEVDKRHWIAHKMTGFFLPGSLIQGVNGFGCENLM
KGYRDLTFTTASSADPVRVMSDKGRVVDLNDQFLASPEVDTPEFISADGVTIAALYRLDT
HQSSGGSPICRAVTSSSSSTYYATKDYSNRSQFAARIGGSNRTIVYNTGITLGQWVFRIG
LYDKASGRMELWDRELGLIANNSYAGDLSTATGSERNFRIACGKQDDANRGFDGVVAMAA
VWNGIGIDATTIEAFFNDYKQVLIPA

>gene_971|GeneMark.hmm|228_aa|+|5039|5725 >NVVL01000049.1 Rickettsiales bacterium isolate NORP64 Contig_source1382A_23578, whole genome shotgun sequence
MSLSLIRRKIGAGGGADVTADIVVTVDDFTGSISVAVKVGATVSPTLEDFTAGISVGVPV
QITIASTLDDATGSISAGVLAVATLNATVDDFIGAISASARFNANIASTLDDFIGAISAA
AKVQVTLSPTLDDITGSISASVPVESDFASTLDDFTAVIDVTAATNRTADIVATMDEMTG

SISASVLAQASMSTTLDDMGAVMNVVTGNTATSKMSPVMRMFIASGRM

>gene_972|GeneMark.hmm|132_aa|+|5747|6145 >NVVL01000049.1 Rickettsiales bacterium isolate NORP64 Contig_source1382A_23578, whole genome shotgun sequence
MKRLWLASLLLMTFNVYAQNFTLTWVNATEREDGSELLQSDIAHHKLRDSETGTIIIDNI
EGTLTSYIYNFPTGVYIASMTTVDSDGRESAYSPFLNFESVTDSETLPVIVEPPTSPQQV
IITITVDVETTP

>gene_973|GeneMark.hmm|165_aa|+|6155|6652 >NVVL01000049.1 Rickettsiales bacterium isolate NORP64 Contig_source1382A_23578, whole genome shotgun sequence
MNILQEHELMLKEDSPVYTSAQYEEEKASGDRKDDGSRKLLKLTPRHEEMIAMHLDGMRN
KDIASAYQITDSTISIFLKDPLVLAVISEARVQNEARFASLYGKVVDVIEDSVNGAESID
IRLKGASLYLKEVGNKPVGERKTAEDVVQQIINMQINGDIVVKGA

>gene_974|GeneMark.hmm|141_aa|+|6652|7077 >NVVL01000049.1 Rickettsiales bacterium isolate NORP64 Contig_source1382A_23578, whole genome shotgun sequence
MIRLEKNFILSELVHPALYKQFKGASYNMLNIRLSPTLQSIRDVFGQMAINGFFKGRLFK
DSGLRLPHGKVGAKRSGHKFGYTADAKFYDTTPIEVQRHILEHQEDFPYITRMEDANITK
TWLHIEVGTNNRVGAIYVFKP

>gene_975|GeneMark.hmm|104_aa|+|7061|7375 >NVVL01000049.1 Rickettsiales bacterium isolate NORP64 Contig_source1382A_23578, whole genome shotgun sequence
MCSSHNWRRLLESYLPEIIIAAALFIIGQLFILANRVKDLWDWHNINDEEGVKIWYVRKS
LEDAVKDMAASTNKIATVLDRMAERDILILRVLEKLENNANRSR

>gene_976|GeneMark.hmm|56_aa|+|7392|7562 >NVVL01000049.1 Rickettsiales bacterium isolate NORP64 Contig_source1382A_23578, whole genome shotgun sequence
MFKSMLIKWVLPSVVDLLIEALVSLSKKSSNTVDDAMAAMFVANKETIIIELKRNL

>gene_977|GeneMark.hmm|522_aa|+|7562|9130 >NVVL01000049.1 Rickettsiales bacterium isolate NORP64 Contig_source1382A_23578, whole genome shotgun sequence
MATRERLVIENMLMIADKDGNDVPFTLNSAQVKVDSRLTGRDIIPKARQEGVTSYFLARN
FIRCLSKRNTRAVIIAHEEDATKRMLRKVHYMIENIRGPKPVIKNASANEIVFSKTNSVF
YIGTAGSRQFGRGDTITDLHCSEVAFWKDPKSLLGGLFQAVPRSGNISIESTGNGTGNWF
HRSTMRAAAGSSRYRMHFLPWQDFKEYTVPLNDLEKSRILNNLSEEFEEVCMPARGFTAG
QIAFRREKLEELDYDLQLFKQEYPETLDECFQATGFSIFSKVNYEATDKWCNAGNGTHVL
EGHPKAGHQYSIGADVGAGVGKDYSVAEVWDLTDKEQVGEYRNNRIDPENFGFKIVSLGS
EFNDAFITCENNNHGILTVKVLKEKYPNHLQYSQVRLRRAANEIDRITDYGFRTTVKSKP
FAIGELRSSLAKEWLIHSPILKDELDTFIENDCGQLGAETGCHDDTVMASAMVAIGEPKA
IIMTMPRAILVHDTEPDPFLIENIINECVSKGSCNHAITVQV

>gene_978|GeneMark.hmm|416_aa|+|9137|10387 >NVVL01000049.1 Rickettsiales bacterium isolate NORP64 Contig_source1382A_23578, whole genome shotgun sequence
MRILIMSKSGDALGIAHRMVLEGNQVKMFIEDTRYADAGKGLVERVAAWRPHVLKSDLVM
CDMVGFGRYEETLEKFGKPVLMCNKFADMAELDRGKGMELFDKFGIETPETFIFNTPKEA
LDLLAVWEDPGFVIKPFGNIDTGKTYVIENQRDYEWALSTYKDNQKLLVQRTVEGIEIST
EGWYNGREWIKPFNHTFEEKRFMSGGVGRNTGCMGNVVIPASSDSKLVQETIMLIEPWLK

KINYRGCVDINTIVNATNAYALEFTIRIGYDAIEALAEGLMEPLGDFLFEVALGIKKSME
LKPGVFMAVRVSVPPWPMNEPGKDDAGRPIHGINEQALKHLFLTDVKMENGEYVYAAGDG
VVFKVTAQGSNVKEASNRINRSLEKVTVHDMQYRDDIGSRVKGDISQLKAWGWLNE

>gene_979|GeneMark.hmm|567_aa|+|10380|12083 >NVVL01000049.1 Rickettsiales bacterium isolate NORP64 Contig_source1382A_23578, whole genome shotgun sequence
MSNGYNSNGSVDITWWLDQIQEGEKFRKKMAHEDMWETWRKYYRGEWNNDVQPSNIYFKM
LRTTVPRIYFRDPVVSITPAKPGIENMLFAKILERIDNKLLRKMKIKRQMKRIVHDTFMF
GTGFGKLGYGAEFSPSDFNDAGEAPTGSVKRPNKVEYNSNIQPNQPWYLRTGPGSIILPS
GLEEFDEARWVVHKIMRPRSDVLDDARLKIKDKKSSRNRSFLMTSIDTVMGVQQSIDMVT
LFEVRDKKTGSVFIISPDFSEEVLLKAEDEFQVHGSVPIYSVNFNPDDMSTWGVPDSQIL
DPIQRELNETNTQIMWHRRMSLRKIFTRKNLMSESEKSRMVSEEIQAVIEVDGDPRTDIL
FKDGNNIPDDLFKAQDATRTDGRESLGFSRNQFGEYNPGSSDTTAREATIVNNSSEIRID
ERRDTMADMLTDVVNDVHHIIFNKWGTDEVIDIIGPAGVTMWVSFKGEMLKRGQYNVNVD
PDTAINQSRQQRQNTAIQVYNLLKQDPMVDQFQLRSYLMTEMAGTQFDNMLKLPQEQLNG
TQNNPLSLEEAGQQFRGPQVQATGGNQ

>gene_980|GeneMark.hmm|85_aa|+|12083|12340 >NVVL01000049.1 Rickettsiales bacterium isolate NORP64 Contig_source1382A_23578, whole genome shotgun sequence
MPLYDYKCERCHTTIEVFHKIKENPPLCDCGSKLVKLIQPARVRMFRAGIYEHIAHNPIQ
IDSMKQLKKECKKHGVTSAYAEDIC

>gene_981|GeneMark.hmm|136_aa|+|12375|12785 >NVVL01000049.1 Rickettsiales bacterium isolate NORP64 Contig_source1382A_23578, whole genome shotgun sequence
MKDDIRKITITITDLDFTNPKVEVEGEFNGAQSRRALAAGGKYIRVRATRAHYERQYAEE
AAAQAKEDAKTVAKQKAKEAADILTEERLANEIEEQKADTIIDSSDGVGWEDTDEEDDDH
LLNTHDTDNGVLNEDK

>gene_982|GeneMark.hmm|221_aa|+|12786|13451 >NVVL01000049.1 Rickettsiales bacterium isolate NORP64 Contig_source1382A_23578, whole genome shotgun sequence
MAEEIKNEDVFKEFTNKMVGAIDALGERIGTIETTVGAQSTSLQAIQDNAVAPAPIIKDE
ETVSLAEMDLESMSRGDFARAITAAVVSSMREEFKPINEQVGTLQNSNETQQLRTEFNDV
ANKHTDFGSYKDIMLGMVETHSHLSVQEMYTIAKANNPDIATQAASAEAEANPPANKGDA
FGGLTPTSGSTIQNKGEANTTDAAEEAWGETMTDTPESAVG

>gene_983|GeneMark.hmm|324_aa|+|13481|14455 >NVVL01000049.1 Rickettsiales bacterium isolate NORP64 Contig_source1382A_23578, whole genome shotgun sequence
MPASLTEQLDNLYTTTWQNMKSTVADSIFDSTPFWFWLKSKNKLRQERGGRFISEPLQYD
RNDNVQWIGKGGTVPLNDFEFLTIAKYDWRYLTASIVRFGVDDQQNRGKNEIINLMKAKM
ENSKNALVSELETRLFGASGAGDSAVDGLQLLVADDPTASATIGGIDQSLASSAWWRNKS
TNNGSFATNGTNNMRTMLNNVSNNLQSDTTDIIVAGQTPYELYENEIFDTHYRVTNNKLA
DAGFENQVYKGRPMVWSPSCSNSRMYFLNTNFLSFVFDPMMNFDMTEWKAIPDQVNDRAA
QVILAGSMTVSRRRCQGVLHSITA

>gene_984|GeneMark.hmm|154_aa|+|14468|14932 >NVVL01000049.1 Rickettsiales bacterium isolate NORP64 Contig_source1382A_23578, whole genome shotgun sequence
MPSGLKRVFLTPFDTVDTSDKEGVGTIRFEGSKIYKYIQYSEEAAAVASVVGEVAYYVAA

SGYANNDVTSDLSASDAVGAGVLQAILADNEYGWIQIKGYAVLTIALTAGADGDPLTPTG
SADGALDVSIAVSDNVCATAGDISAQEIMCNFVF

>gene_985|GeneMark.hmm|231_aa|+|14972|15667 >NVVL01000049.1 Rickettsiales bacterium isolate NORP64 Contig_source1382A_23578, whole genome shotgun sequence
MGTLTYLQIKEEVLGFLGNRDDFTSRIDTIVNIAQMRIARLSKFEELKTSLTDDLTLTGG
ITADKVYTLPTNLRNIFSIKVYEDTGDRYVKLVGLSSTQFDKKFTKPSKESRNMPVFYHR
WGSTIEMYPIIDKNYKIDIRYNKWPTVLAADGDLSDLQEKDDAIITLAASWMFMSLKKKE
DANFQWGVYKNMMKDILGEDRESSDITYGVDPRRPVAVGLGYDNPFVRGMN

>gene_986|GeneMark.hmm|127_aa|+|15667|16050 >NVVL01000049.1 Rickettsiales bacterium isolate NORP64 Contig_source1382A_23578, whole genome shotgun sequence
VANLISLNQPVLQLRQSNVKDADLYSELRTLLRDIEKQFLAIAIAINDNTAGGAVIILNG
ATSVVVTHGVSFIPLAQDINIISTENPTNDPGNFWLSSITNTQFTINCRQDPGASGLGIS
WNIHRTT

>gene_987|GeneMark.hmm|252_aa|+|16062|16820 >NVVL01000049.1 Rickettsiales bacterium isolate NORP64 Contig_source1382A_23578, whole genome shotgun sequence
MTVFTRTWNPAYEALPPDTENAKNGAERINETKVDTRERFEVDHSQAGDVDDGMHKKVTF
VDPLGAKPLQLNDETYLYSKDVDGSAELFFEDEAGNETQLTAVGKMVIDLVYPVGAIYAS
ASSTSPDTLFGGTWTALADKQTLVQQGTYAAGTSGGSSVAVNVDHTHSDTFSLSSDGAHA
HNVRYSSASLGGSSALTGLQSSSATGNTASSAAISNGSHSHTMSGGVSSAGVSGTDANMP
PYLSVHMWQRTA

>gene_988|GeneMark.hmm|592_aa|+|16828|18606 >NVVL01000049.1 Rickettsiales bacterium isolate NORP64 Contig_source1382A_23578, whole genome shotgun sequence
MAEIINNLPEEGWQYQIINELGGGLVQNVPKQQLEDGQFPFLENLIFKDGLVHVDTGVAL
FGNIVLGAPRIAFQFFLTNSTDFLVLLTNAVFYTWSSGTSSWLPYNDGTVTTVTNTTVPL
GAAQVSLVSTTGVLVGDIMCITLDNGHIQAVTVTIVASPLVDFTEPLQDAATSGNAVYET
TKLSGTDDIQPSVTVYPYNDWMVWTNGVDNPMYFDGAVLAVIPNLPSSGATLCKLVSTFN
NRLMLANTDEAGVPHPQRIRWSDAGDPTEWLLGDSGFQDLFDSEDFLIAVAPLGPFLMYY
RDRSIVRAEFVGSASKLFNFETVISGEGAISQDTVLDVGDFHMFFGSANIYEYKGGFDLE
PVGDVIYKKVFSSTGELVPNTKHRGFGLYIEELDESWFVYPHISSDAYPSRVIRFNAQGE
GWWQRTFPFQISGFGYFESTGNVTWNDWDGLTWNDLNFPWNAKNLQDNAPIILLCGATGQ
IYTYDYNAVTDAGTDISYVLETKAFGHPRVSYRFDEYEFYLSGADIKVEYSLDRGRTWFD
LIVTTSPGAQVISVRSYKQLVGNYISFRFSGSDAFTLESFSFQHSVESEHGG

>gene_989|GeneMark.hmm|302_aa|+|18608|19516 >NVVL01000049.1 Rickettsiales bacterium isolate NORP64 Contig_source1382A_23578, whole genome shotgun sequence
MANIKDVILGVAPKAVIEQRDTINKEQKSGLAALLQELQGDAPQVSVPVTEGQQTLIDQS
VGGNAVAQGEIDSSTEALKRLFDPAGDFDDQVQNPLIRDFNENILPQISRGFGNDFFSSE
RQRGDERATEDLLQTLVSEKSRFLRDDRATQLGAGNSLTANASNRGTILQQFLQSAGIGR
DIEEGGNLKDEGVRQELLKLLLGASTNPTKETIGIAQGGQKGVLGDIIKSVGSAFSKKSD
RRLKKNITRIGTFNCGAPKYMFQYVWGDWSIGCMAQEIGDYVSGAVLMDDDGYFSVDYSK
FS

>gene_990|GeneMark.hmm|407_aa|+|19552|20775 >NVVL01000049.1 Rickettsiales bacterium isolate NORP64 Contig_source1382A_23578, whole genome shotgun sequence
MAIVINVPRDDRFSDIGEGIGELVSALVDRRRDDKDSKTIEGIGNGSVTVSEGLATVSAR
NRAAAQQAGANYNRDNPKRSFLQLDDAGGAISERVGRFKQPGEVSAKVATFNKERKATDK
SAALSEIRSTQINDSPTFSDVSEAGRNRAARNINDQSVDLSRFNKNLYDKLGDLTGDNRI
RVDRRNIHYTDYIRGDNGKPITLDKNKAFTLAKQDAEKELQGFLSAQNSNDLITPEEYFG
RPQVLPKDKDDTTPIPTANQEVSPVDAFKSFASSASDIIGGMFNAQEPVVTDSDVSIVDP
EIRVSEATDSAINANYPLQDKEEWVDVGEVYPMVPQSMMDVVKAGNAGAVAPTYAAMANV
PVHVALQLLLQNQSKAPTITMLETKVIPDSVTDPEQDGNINTGTIQR

>gene_991|GeneMark.hmm|1278_aa|+|20780|24616 >NVVL01000049.1 Rickettsiales bacterium isolate NORP64 Contig_source1382A_23578, whole genome shotgun sequence
MAMSPEDMQVLIDESRIKLQEDKQRQNGYIKDVQANPPVTQEELREMITQPGREQRDDEG
VLDYVADEVMDIGSSAVGAVELGAEFTTGMFAAFAGTVVGTAKEVADDPLGFLPGPINIA
LSSLVEKDTMKKHGKFFMETFQDYTEAMTYQPRTVQGQALSHYAQQLFGKFQDWGEQAGD
AVLEETGSPLIATAVETAITGAPVYVPFIGGKILQRRADKAKLERRRELIDEGFDTPLDP
TAINQSMLPDPRKIASTPVDRLAPDVQSIQNKTAQAKQDKQLKDLEKQVRDPNQKTLDLE
EFPRINEDAATPHPDRGRKISTSEAVSRAKKVDQIQDASIIASGRRDIPSVRQVKDLIME
TTFDASAGLRRRILSGNDKTQNRLLELEHNKQSGATPKAQIDYIKSENKIYTNLAREDGI
LLDKFINSLRTIQVDLNAALKKQLPPKHSGNRTGVDHKIALAVTRKRIGEDKYNMLMKRA
DDYFRVHNRLLGDLFVEGLITKKQHNSMKGDIYSPRKFLENVDPLNESLTAGTVSFDIFG
KRVKLNDSGLRELNKGSLDLMEMNSKLLLQEVTVRITARIWRNQSNVMLLDWVKKNPDNG
WVRVAKEGEKAGKKGSIDAYIDGRKATMLVDKGMALQWVGNTYVMGHGFSSFLRTLSGQT
IMKPLTTGMNPEFALKLFGLDIVHAHFATDVFSPSAPIYSLQLGKNLAVTMKDAFFKGDR
FEAYINEGGGMNFLTHQGTDALGGKGVIRSNTTWRKTKTALSFLNEGFEIWVRLAIQERS
IAIGKSPSEAALIAREYIDFSQGGTFSKSIDTVFPYFNPVLQSVRSISAAATRGDKSQKV
LGLRKRFLYQVAELSAYMGAWAMYQELHFPEVMRQLPTEEKAKFFIFPVGRGIRNKQGEV
QYAYVKIRKDNVVAALINPAEMAVRNIITGEVPDDQTLAYFKSASPVLPAELSTPTGGML
HTFTYNHDFWGNPVWNNPDLDAGVSPEDEFTGHSVGKEQSSVFFRDVGKALGMAPERLEA
AMGNIAPSNGYLDFGGFAYKKIVDQMLMGNPEDPFLAEQVFEQQLTRAPFLRKMISLTHP
AYRVMEDSKEPRTKSQVLRKNQGDNLDSLIANNTSFSTVKQWVRQQKPEDQKRLLTSYIT
RTELHKIFNGKTEMSLNVPGPFFWNHVASEIKDNEEKAEVLYWEWKKMPTPQDRNKFDRI
MKRLSVDKQILPGTTLKHFRIMQQRWGTEGIMTSEDGGTFHMYGKRHYMKEMRVRMKQQE
DRKQQAGSIDQSLLPDIP

>gene_992|GeneMark.hmm|399_aa|-|24758|25957 >NVVL01000049.1 Rickettsiales bacterium isolate NORP64 Contig_source1382A_23578, whole genome shotgun sequence
MSNREISDWLDGYLKYTYETEPPVTYHTWVGLSLIAGALQRKVYFKWGHDTLYPNLYVVL
VGPSGRARKGTAMNIGKDIISSIDTIRVASESITREALIRDMKDAVNQYQDSTSGRMIFH
CSMTVMSEELSVFLRKNDNGFLSDLTDWFDSRDTWTYRTKGAGTDKIQGVCVNLLGATAP
DWLPSILPQEAVGGGFTSRIIFVVEEDKGKTVPEPKFTEEMHELRISLKKDLEKISLING
EVTFTPEAKQLYIDWYVDFERKISEGKTIIDDPRFSGYLDRRATHMRKVAMVCSMSRNSD
LFITTDDFNRADSLLHTVEAKMPKAFGGLGRASYSEMADKVITYIRDNKVVKRSDLLRKF
YRDVDPATMEMIEMVMNQMQLVSMSQDPLKGEVTYKYLG

>gene_993|GeneMark.hmm|510_aa|-|25978|27510 >NVVL01000049.1 Rickettsiales bacterium isolate NORP64 Contig_source1382A_23578, whole genome shotgun sequence

MLDESVLIYNGYDVANTLQCKEEIWKVTKDSAYTYDFTMRLLNPLMYMMTRGVKVDFKKL
DILRKSLHEQIDRKQLRLNELCGRELNVNSPKDCQTYFYVEKNIPPYYNSKRVGGQYKKS
VTTDDKAMQRIARGTAARKGLYEATLVQDIRGMRKMVGTYLDMEYDKDGRLRCSYNPRGT
KFGRLSSSKTIFGTGMNMQNLPLEFKGFIVPDPGNLMWEIDKKGAEWVAVAYLSGDANMI
KVVEEGLDPHAYTAHLMTGVPIELIQEEDAIIGHTNDPNLVSQGRAKLIKGSLANQHAYE
QAVFLPRTMTIRQCGKKSNHGLNYDEGYKMFALINEMLEGDAKRICHMYKHIVYPGVNLW
HKSIQDKLQQDRTLTNCFGRSMRFLGRWEEKLFKEAYAFLPQSTVVDIVNTGMEKFYYDR
RPDMCAPELLAQVHDSLLGQSPLDGDYAKMARVFKTIGDYMEPTIEYGGREFTIQNELKV
STDNWRDGVEVDIYAGDEELGRQIQNIVEK

>gene_994|GeneMark.hmm|60_aa|-|27500|27682 >NVVL01000049.1 Rickettsiales bacterium isolate NORP64 Contig_source1382A_23578, whole genome shotgun sequence
MLNNNEVPYEECNKIFISSNSGDTIPSWYYTTDCQYRVYSRITKSKFMGGKIKWGMYHVR

>gene_995|GeneMark.hmm|335_aa|-|27675|28682 >NVVL01000049.1 Rickettsiales bacterium isolate NORP64 Contig_source1382A_23578, whole genome shotgun sequence
MPFIPVQQERSYFGDGEKSSKICIVGESMSKWEIRSGRVFSGPVGTIYEQCLHGANLTRA
DVYATNIVKIEAKFTLYYREKKGFTDLGKEAVNALKKEISELDCNIVIAMGEAATFALTG
RMDVTKCRGYIFPCVFNPLLKVIPTIHPAKVQFGDYIARHYISHDMNKAKEHSSYKGLKY
DLTKVEIARTYLDAVTWLEEIATWKHVSIDIEVINYECSCIGFGKSIDTSIVIPFYGKDL
YTLEQEINLWRLVAIIMYDENIAKTGQNFIFDMQFLLARNRIVTRGTIYDTMIRHSIAYP
EFLKSLAFLTSIYCNRPYWKDMVSWKKKDQIKNDA

>gene_996|GeneMark.hmm|113_aa|-|28684|29025 >NVVL01000049.1 Rickettsiales bacterium isolate NORP64 Contig_source1382A_23578, whole genome shotgun sequence
MKPFIYVASPYSNQQASVRQQRFDMVCKYTANLICCGNIAFSPIAHSHNMAEALGIKAHG
FSFWEDLDVFLIERCDILHVLCLPDWEVSGGVAKEIEKANELGKPIVYIPWGV

>gene_997|GeneMark.hmm|69_aa|-|29022|29231 >NVVL01000049.1 Rickettsiales bacterium isolate NORP64 Contig_source1382A_23578, whole genome shotgun sequence
MRLVRKGTDKTVTQNTKLVIHEKMYQVFAIIIPNEEYPKGMIKVNLNTGGEIHSWQQFYP
NFLGLEFIE

>gene_998|GeneMark.hmm|442_aa|-|29215|30543 >NVVL01000049.1 Rickettsiales bacterium isolate NORP64 Contig_source1382A_23578, whole genome shotgun sequence
MSNRKLISIPIVDIIIGKRFRKKYEGIEELSTSIKETGLIQPVTINSKKELMAGGRRYMA
HVMGQLSHIDCIMLEDSSSEVDLREVELVENLYRQDMTWHERAELTDYIDQLKKANDPNW
SGRKTAELLGKGKSAVAKDLKLAEALKNVPELKNCKTEADANKVLVNISKKIDAQQAVDK
HKENPAILAAVTHYTIGDAFLGMEEMYDANPNSMVSLIEIDPPYAIDLQNIKKADLTKDG
SITNYNEVKTNDYEDFITRTCTDVFNCAGEDCWVIFWHAFQWQQLIRDSLEDVGFIVDPV
PGIWLKGEDGKEGSGQTNQPKTYLARCYEPFLIARKGKPEVQKQGRSNVFSFKPTPAAQK
YHPTQRPLALMNEIIKTFVHPNSIIMVPFLGSGITLRAAYQNHCTGFGWELNENNKPLFL
ASLLEDGFGADILEENTDEVST

>gene_999|GeneMark.hmm|96_aa|-|30536|30826 >NVVL01000049.1 Rickettsiales bacterium isolate NORP64 Contig_source1382A_23578, whole genome shotgun sequence
MALQIYVLQKPPPFMLFFNKYTKINTKLIKGKETSIIHMDEDLRVEFSEGSIVILFNDKN

KDTSSITHIHNIADQEKLLTLFALDVQYKDEERDHE

>gene_1000|GeneMark.hmm|100_aa|-|30826|31128 >NVVL01000049.1 Rickettsiales bacterium isolate NORP64 Contig_source1382A_23578, whole genome shotgun sequence
MLVDVYEAGNWEKTKWRIYTMARQIEDILPNFLDISANEQEQIVRACRYNKYVLKPSHQA
HKKKAGKKKTKVVSNKIKNLVKNMSEEEIEKLMANLAENT

>gene_1001|GeneMark.hmm|82_aa|-|31177|31425 >NVVL01000049.1 Rickettsiales bacterium isolate NORP64 Contig_source1382A_23578, whole genome shotgun sequence
LSTKWTQEVIKMSSDNDSTTRINIVIDSEQNTLLNKMLPWGAKSDIIRNIVEILIKDLEQ
DSSGDFYSALVKHNVKLVKIEE

>gene_1002|GeneMark.hmm|103_aa|-|31429|31740 >NVVL01000049.1 Rickettsiales bacterium isolate NORP64 Contig_source1382A_23578, whole genome shotgun sequence
LGLAPRVLFKEQDMSDQLNSEIVGVPMPTELNNAVNRMGQIQVQLSIVLSKISGNDNSIE
SQLIPQQDMCLREVLESAPTKLFEDQDIILKQIEDLDRILFCK

>gene_1003|GeneMark.hmm|141_aa|-|31843|32268 >NVVL01000049.1 Rickettsiales bacterium isolate NORP64 Contig_source1382A_23578, whole genome shotgun sequence
MSFIQVNLDETIKERSAVPAGAFDLVISDVSDKPANSGSAMLTITHAIDGDDDAMPVKHW
ITLPTKGDDADTVRQKSLGLKRYLEVAGIDYGEEGFDTDVLYGHAFSCSVTNELVDTDKD
GNPIDSPYEVNRLSIPFLASE

>gene_1004|GeneMark.hmm|287_aa|-|32291|33154 >NVVL01000049.1 Rickettsiales bacterium isolate NORP64 Contig_source1382A_23578, whole genome shotgun sequence
MASLLHAPLVVVTGKMIMMNRRAMMPNTKDITTNTPRMMVYSDPGGGKTTQFLTIPGRKF
AYLFDPNATQSLRGADITYEEFYPCDVSLRLTSLSKKNKAPSPKARTGAEAYNAWEKDFE
KKLESGFFNDFDVILFDSLTTFSDMVMDGVLAINGRGGQWPQQDDYGPQMLAIKNVFRAL
TGLGIPLYVTAHYKTLQDELTKKVYNQILVTGQLRQKLPLLFSEILYLEASSDGKGNVNY
LAQTKPDRMFPTVRCSTKGLDYKTDITIDYDKPLEGQGLGGLFFNTL

>gene_1005|GeneMark.hmm|86_aa|-|33220|33480 >NVVL01000049.1 Rickettsiales bacterium isolate NORP64 Contig_source1382A_23578, whole genome shotgun sequence
MSDSYNAHADDGRRKIKKENNVDQSIQILTDRGIELKRHTRYHYCITGNLGKIDFWPSTG
KYLTSYNTTIGRGVFNLIKEVDKARG

>gene_1006|GeneMark.hmm|325_aa|-|33640|34617 >NVVL01000049.1 Rickettsiales bacterium isolate NORP64 Contig_source1382A_23578, whole genome shotgun sequence
MALRYYDNTRISEARNCMRKFFLRHRLDWVREGLARPLLFGQCWHNAMDVVWLHADSDKT
DREIFELAGMAFNQQWLAEGGPDPLTMSPDMQAYWSPRTPGVGAEMLHNYIKTRREQIRQ
FEVIGIEVPFAVPLDPDDPELFFIGRLDKLFKNKQGDIIIGEHKTTTSYSKKDIFRADYI
SSWSPNSQIDGYLYATHMLYGSSVKSVWVDAALVHKTVHNGFKFIPVNRSKAHLKSWLYD
TLFWVNMIENEDFRYEQLKPDDEYLAAYPKNTGACSQWAGCTYKDICTMLPNPATQELPL
GFKKEHWEPFDVLKIEELGMVKGNG

>gene_1007|GeneMark.hmm|120_aa|-|34724|35086 >NVVL01000049.1 Rickettsiales bacterium isolate NORP64 Contig_source1382A_23578, whole genome shotgun sequence

MTTKVTAIGKTKDDKGAVTGENEGVIEFNFGENLEEAVKLYGKDAVFNNYRQASTIALQG
NMRRHLSQGKKGKELLAALKDWKPGQVSRQKKSAGEKLQAMLKGMTPEQVAATLKEAGIG

>gene_1008|GeneMark.hmm|62_aa|-|35204|35392 >NVVL01000049.1 Rickettsiales bacterium isolate NORP64 Contig_source1382A_23578, whole genome shotgun sequence
MARATASIDTIYIMQLSEKEAQYLLCITQNPIPSEPKTEEESEDTRDREAIFLALTTAGV
TF

>gene_1009|GeneMark.hmm|202_aa|-|35398|36006 >NVVL01000049.1 Rickettsiales bacterium isolate NORP64 Contig_source1382A_23578, whole genome shotgun sequence
MSKVVHLRISDEAVYTCLQVLKVNGVNTTNLPLATIVRRFIEGATDNYRIQKKLPLPEDL
SFIVSALSNDEITPIVMEDVGFVEPLPDGLDQDKMDNIRKAMAASSLGNNSPEVVVRDVV
EDEPVELPPPPWEAFHILRWATILDRAPKDVLVEEVVTNKSIPLQRAVQCIYSDLPIREW
GTEYTANLVQKILPTINKYFEE

>gene_1010|GeneMark.hmm|48_aa|-|36020|36166 >NVVL01000049.1 Rickettsiales bacterium isolate NORP64 Contig_source1382A_23578, whole genome shotgun sequence
MINKNIGDSSTPDTWKPTSDSIRDERIDAELQEAFDIVRQEELARERQ

>gene_1011|GeneMark.hmm|58_aa|-|36156|36332 >NVVL01000049.1 Rickettsiales bacterium isolate NORP64 Contig_source1382A_23578, whole genome shotgun sequence
MILEITNRTKSLALIALALVLLNLGLRSTDPFTQGLLFMYTLVSFVVAIAATGESHDK

>gene_1012|GeneMark.hmm|97_aa|-|36322|36615 >NVVL01000049.1 Rickettsiales bacterium isolate NORP64 Contig_source1382A_23578, whole genome shotgun sequence
LEKIMKKKLCVVKQVSLEYLQFISDVDYQLCYKDDENYIRITEVVEVDFPDIDKIELLNA
ELAALDTQATKIKAVAEAAITGIERKRQELLAITHDT

>gene_1013|GeneMark.hmm|89_aa|-|36741|37010 >NVVL01000049.1 Rickettsiales bacterium isolate NORP64 Contig_source1382A_23578, whole genome shotgun sequence
MNTTKIFKKTTWLAVCLSVLLVALTGCRNDNLPFELQDAKQPITIVLSNEAGVIVSDGDG
RIYAYKESYYFANMIMKSDLKKGDVITGN

>gene_1014|GeneMark.hmm|108_aa|-|37328|37654 >NVVL01000049.1 Rickettsiales bacterium isolate NORP64 Contig_source1382A_23578, whole genome shotgun sequence
MDKLKKESEAATGCEASAVERLVIPFGAKVHVTQKYRRKITGRKKEYVTQEFTANGIFLG
WRTISNGVTYCDHEEGCHWEADKYLQAALVAISPTTNPLYIPVDGIEV

>gene_1015|GeneMark.hmm|73_aa|-|37700|37921 >NVVL01000049.1 Rickettsiales bacterium isolate NORP64 Contig_source1382A_23578, whole genome shotgun sequence
MSKKNWNDLVSKYDKLIGGEYTDDEGMLFTFVGLIHSDDDFYYGLIPKGGSEKGMLIRLS
CVCALESFGYDRI

>gene_1016|GeneMark.hmm|84_aa|-|38310|38564 >NVVL01000049.1 Rickettsiales bacterium isolate NORP64 Contig_source1382A_23578, whole genome shotgun sequence
LLCAGLEYEMATVIINNVEYIEKPKDIKPPENIHNATHWAVLPDDSILFYRVDDEVVLWQ
PLSQLWIPPSTVPYTLHSYDDYST

>gene_1017|GeneMark.hmm|74_aa|-|38637|38861 >NVVL01000049.1 Rickettsiales bacterium isolate NORP64 Contig_source1382A_23578, whole genome shotgun sequence
MKRIDFLYALLACSIMICAYANGGEIYAFSASLTMIIFYALLAWAQFEENRLLRISGNFA
GDEFNEKYKDLSGT

>gene_1018|GeneMark.hmm|105_aa|-|38965|39282 >NVVL01000049.1 Rickettsiales bacterium isolate NORP64 Contig_source1382A_23578, whole genome shotgun sequence
MKTITFDMNKYTDSLLNFVSGTSGLDRAIDPPASSFRTEEQEQRIIDETKRLLANPIEIN
DACIEEVLELYRIHHNHDLLEFGESVHRYFTVLAKAQATELVDYK

>gene_1019|GeneMark.hmm|70_aa|-|39279|39491 >NVVL01000049.1 Rickettsiales bacterium isolate NORP64 Contig_source1382A_23578, whole genome shotgun sequence
MGDTMRKDVDKKLTNIVIEGADRNDYPDFSDAYIASADDADGRPLTEDELDKLNEEYPEI
AQQHAMESLL

>gene_1020|GeneMark.hmm|58_aa|-|39550|39726 >NVVL01000049.1 Rickettsiales bacterium isolate NORP64 Contig_source1382A_23578, whole genome shotgun sequence
MNKIEEYVILYHTNVLDLSFEVDRFMKMGWIPHGSLLATLSDSGGYEYFQAVIKVAQD

>gene_1021|GeneMark.hmm|82_aa|-|39723|39971 >NVVL01000049.1 Rickettsiales bacterium isolate NORP64 Contig_source1382A_23578, whole genome shotgun sequence
MRQTIVVLEIPTGRHYTRFNNALDAIVLIKKRISRLLFVIPTIDVPDGLTGGMFVALIDE
SIERGEGINKNSFLKMLGGGIT

>gene_1022|GeneMark.hmm|35_aa|-|39968|40075 >NVVL01000049.1 Rickettsiales bacterium isolate NORP64 Contig_source1382A_23578, whole genome shotgun sequence
MKTLKRIVEMIYPLPIVVILMIGWGFLNMYVEAQL

>gene_1023|GeneMark.hmm|64_aa|-|40072|40266 >NVVL01000049.1 Rickettsiales bacterium isolate NORP64 Contig_source1382A_23578, whole genome shotgun sequence
MAQFEIKTRRIRNLTEELNEFFSDNGLPPGSADEVLIDKEIELTTWQQAYLLNFIERWNK
ELLI

>gene_1024|GeneMark.hmm|66_aa|-|40271|40471 >NVVL01000049.1 Rickettsiales bacterium isolate NORP64 Contig_source1382A_23578, whole genome shotgun sequence
MSVLVQCIQDYDDAEHMSRLIKKGDICAVVDVREYILRVITSHYHGTDQWEVVSRFRYVT
NNKNRS

>gene_1025|GeneMark.hmm|71_aa|-|40471|40686 >NVVL01000049.1 Rickettsiales bacterium isolate NORP64 Contig_source1382A_23578, whole genome shotgun sequence
MIRFRYLPNISLTQIILTGKATSRCFFVTGKVSDINKVLLADIQVPEDLSDVCLNEALSS
RVGYTKAHEVR

>gene_1026|GeneMark.hmm|57_aa|-|40748|40921 >NVVL01000049.1 Rickettsiales bacterium isolate NORP64 Contig_source1382A_23578, whole genome shotgun sequence
MELSEMVTTLQKLLKDHGDLIVLDANDYPIEEISLCVSTYSAHGDHAIKLFSEGDEH

>gene_1027|GeneMark.hmm|251_aa|+|1|756	>NVVL01000050.1 Rickettsiales bacterium isolate NORP64 Contig_source1382A_23633, whole genome shotgun sequence
AMAMAMALALAMAMALAIAMAMAMALALAMAMAMAMALAMDKKTFIQNFVIQRTKTLPAE
AISDAVTAYDSIEALFPKFAKDSKEGFQTAKGRYITDHRLAWFDAIWTVWNVGISTGTKG
GKKQAGDAFLDQIKNKNDAKMAYASAREHAEKERPALEAKGSTPPYFNKWFNDGRWDTKL
IVSDEFEMDRPPPVSKEVIDLRCGIHECDNIIKQFGGYGATEGNTNIIKQAKERKEDLTA
KLMQASRNETV

>gene_1028|GeneMark.hmm|116_aa|+|743|1093	>NVVL01000050.1 Rickettsiales bacterium isolate NORP64 Contig_source1382A_23633, whole genome shotgun sequence
MKQFNSKEWSQGVEVVTGGMDSVRIICTDGPGHYPVVGFIGSDSEASQWNDIGCYCRDLG
TDCRDIFFKPKTITKYANILTRGNGDKYLSPNMFHNKNLVLNNAASIAIATIEWEE

>gene_1029|GeneMark.hmm|56_aa|+|1379|1549	>NVVL01000050.1 Rickettsiales bacterium isolate NORP64 Contig_source1382A_23633, whole genome shotgun sequence
MRCKERKDPKKDFGFRLNGKRKENCFVCEEERENNVVTSLPTRTLKTSQEWLLKRW

>gene_1030|GeneMark.hmm|83_aa|+|1551|1802	>NVVL01000050.1 Rickettsiales bacterium isolate NORP64 Contig_source1382A_23633, whole genome shotgun sequence
MDNLHNDILESFEKNTTSRNVSYTRVIRCNKGLWKVSSHDYGFAEEKAIREFLKYYFTGV
YDVDIDTDLIKFSNGWMQVSYGG

>gene_1031|GeneMark.hmm|137_aa|+|1792|2205	>NVVL01000050.1 Rickettsiales bacterium isolate NORP64 Contig_source1382A_23633, whole genome shotgun sequence
MGDEWLVNSDHSKAKFLEHVEKIYDEHKHVAFTIKTGKQRTNTQNASMHKYCELLASELN
EKGLDMDLVLSKGVSVPWNKILIKELMWRPVQLAMTDEQSTTKPKRQDYGEIYDVINKYI
AERFGIYVPWPSREEMK

>gene_1032|GeneMark.hmm|62_aa|+|2202|2390	>NVVL01000050.1 Rickettsiales bacterium isolate NORP64 Contig_source1382A_23633, whole genome shotgun sequence
MSVKIRNHKDDPDMLEVYSIDWRGEFTLWGSVHFDILTEFEVEITKDDLDNMERDLCIKE
GK

>gene_1033|GeneMark.hmm|37_aa|+|2387|2500	>NVVL01000050.1 Rickettsiales bacterium isolate NORP64 Contig_source1382A_23633, whole genome shotgun sequence
MKALELIESLGRIVSGLARADIRKDVDMQKIWIKVER

>gene_1034|GeneMark.hmm|58_aa|+|2573|2749	>NVVL01000050.1 Rickettsiales bacterium isolate NORP64 Contig_source1382A_23633, whole genome shotgun sequence
MSGYWDMGGSYGMSGEFSQDLNKELHRLLARFKNETEIIKITEPRQDRVYEKLVWLNE

>gene_1035|GeneMark.hmm|173_aa|+|2768|3289	>NVVL01000050.1 Rickettsiales bacterium isolate NORP64 Contig_source1382A_23633, whole genome shotgun sequence
MAKKCKECKSKIENPYSSTQIACSPLCAILLAQKARAKEDKKKHKEMKEKIKTKGKWLQE
AQQEFNKYIRKRDEGNSCISCGRSTGCKINAGHYRSVGACPELRFCEDNVHLQCEQCNCF

KSGNAIDYRIGLIKKIGLDQVEWLEGPHDLNNYTIDDIKEIKARYKIKVKGML

>gene_1036|GeneMark.hmm|67_aa|+|3302|3505 >NVVL01000050.1 Rickettsiales bacterium isolate NORP64 Contig_source1382A_23633, whole genome shotgun sequence
MIDLRDVRIDICATSPAISVGEVEIILTYEPTGQSVSGKGKSTYLLKEKLLYRLDQKVLW
GTGGDSI

>gene_1037|GeneMark.hmm|138_aa|+|3492|3908 >NVVL01000050.1 Rickettsiales bacterium isolate NORP64 Contig_source1382A_23633, whole genome shotgun sequence
VILFKKLNDLAIIPKVQTDGAAGFDLHCVEETSVWGGEVTKVMTGLACAIPDGYLGLIKP
RSGLAAKWGINVLGGVIDSDYRGELIVLLTKHTAMPFDIHPGERIAQLIVVPCMNQCMEV
GDLDSTTRGVNGFGSTGE

>gene_1038|GeneMark.hmm|62_aa|+|3901|4089 >NVVL01000050.1 Rickettsiales bacterium isolate NORP64 Contig_source1382A_23633, whole genome shotgun sequence
VNSYTHGIYKGKAAIFYNGLPISQEEAIDLLNQTVKLKQEFSILTSKLTALIKKRRLKQE
EI

>gene_1039|GeneMark.hmm|85_aa|+|4086|4343 >NVVL01000050.1 Rickettsiales bacterium isolate NORP64 Contig_source1382A_23633, whole genome shotgun sequence
LNIDELRNRFINSHKATINKKEFEILDKQFKQIMIESEASAISCDLVNSLLTKKINENKH
LTRLINTLKIQSNCFEQSIRELMVK

>gene_1040|GeneMark.hmm|126_aa|+|4450|4830 >NVVL01000050.1 Rickettsiales bacterium isolate NORP64 Contig_source1382A_23633, whole genome shotgun sequence
MPDSISNSSVVQALSAVLALVIGLLGSFAAWVGVRNIGRIDYLEKSSQYNKINLAKSNEK
FSMALTALGETVERNERQHKERIDSDRRETSRRLAYLEKMHEQSRMDLKELFDKHAQNLE
RIISNR

>gene_1041|GeneMark.hmm|136_aa|+|4827|5237 >NVVL01000050.1 Rickettsiales bacterium isolate NORP64 Contig_source1382A_23633, whole genome shotgun sequence
MKAHELIKLHEGLELKPYRCTAGELTIGYGINLDAGITEEEADYMLDNRVRSLTTQLHNI
DFWIELSGVRRAVLIDMAYNLGLSGLLKFRRMLAAMEVRDFDKAAIEMENSRWFNQVGNR
SKRLQKMMITGDWWTD

>gene_1042|GeneMark.hmm|205_aa|+|5728|6345 >NVVL01000050.1 Rickettsiales bacterium isolate NORP64 Contig_source1382A_23633, whole genome shotgun sequence
MRSAINQVPHQEIRVAEALLLPKRVMFVRHPLTRLASAFNHFWWLTLNNTRYHEFVPAGV
ILADGGRLEGRVGGNEHHFSGDRKKFYDDKLAEEIAKGGTTEEIAVRLKQEDWNRFIDHV
LSGNNDDHWAPQLDIAKHDGQIVANIAHRFEDVRTHWTKYVGGTLPELNSWQDVPKIDYR
LAELRSYYDETLTFWGAIDGTWNAG

>gene_1043|GeneMark.hmm|206_aa|+|6392|7012 >NVVL01000050.1 Rickettsiales bacterium isolate NORP64 Contig_source1382A_23633, whole genome shotgun sequence
VASFTPDADDFLLAYVAIEDGNSTEPTMSGQDDGISWARIGDIETATLQHGVFFGAAVGS
SPSSGVVTAAHGTTVGMTLQVTSISSVDVSGTVANALLQNDQGGGYASSLTLTLTDATAL
TVGGWSVFGNTVIPEGTEINGTSVDQLDTSMDYDASGDASPSASVSSGNTYFVGFAVELK

EAGGATYPLEIQEFTKFKNRQPILGM

>gene_1044|GeneMark.hmm|147_aa|+|7015|7458 >NVVL01000050.1 Rickettsiales bacterium isolate NORP64 Contig_source1382A_23633, whole genome shotgun sequence
MGRIVYGALSGVSISSDATQDIWNLLAGTNKRISLHGWELTSAAVAAALCSVSLQRVTTA
GTGGSAVTEQASDEDSGTIDSSMVTDVETPGTPDGVLMGYQWEQLGPVGHIWTPEMRPKS
VDSQGFALVCNTAIAMTVSGWVCWEEH

>gene_1045|GeneMark.hmm|147_aa|+|7537|7980 >NVVL01000050.1 Rickettsiales bacterium isolate NORP64 Contig_source1382A_23633, whole genome shotgun sequence
MFSAVLTGTALDTIDEDDITTGSKTIIVTLHNDTFKAAGTGPIGSTADTQALIDGFDAAS
SPTNGWNNEVRDKALTSEVVRTSSTIATWTVAAQSGYDVSAQEIITGTIPTDVLVIGFGA
ITATPNFTVDAVSSGLGSGHLLLLGTG

>gene_1046|GeneMark.hmm|606_aa|+|7992|9812 >NVVL01000050.1 Rickettsiales bacterium isolate NORP64 Contig_source1382A_23633, whole genome shotgun sequence
VADNIELSTGSGGATVKTDDDGSAHWQYVKVSFGADNTQTRVTSSVGLPVDILAGTATIG
EVTIGAATGAAGDLAKAEDAVHGSGDIGVMSLAVRNDTLAALAGTDGDYAPFQVNATGAL
YIQEGAALDVSAATLTVNAHAVTNAGTFVVQEDGAALTALQLLDDAVATLGTTTYTETTT
KGNVIGAVRNDTLAALAGTDNEIAPFQVNASGALYIQEGAAMDVSAATLTVNAHAVTNAG
TFVVQEDGAALTALQLIDNIVHVDDAAFTLGTHSGVMMMGFAGTQSVNANDAGAIAMETD
GAVHIHDGGNSITVDGTVTANPASGTIDTVTTVTAVTDITNTIDSTISGAALTALQLIDD
PVFADSAAFTLGSSKVNVQGGVFQSGTPTALTDNDAGAVLLNSTAGQMVELMASTAVIGS
VINAGTFATQNTAQASTNTQEMVGDVAHDAAAAGNPVLIGSRATAAIEALTEVAEADASY
IATDLQGAIITRSYCTAQEQVEFYIGNTDGAEDAVTGLDAGGAGVHNYVTSIIVHNAHAS
TNGFVTLLDGSGGSISAVFPAPATGGVVHRFDPPWKQPTANTALFVDVSAAITTMHITIS
GFQGQG

>gene_1047|GeneMark.hmm|46_aa|+|9812|9952 >NVVL01000050.1 Rickettsiales bacterium isolate NORP64 Contig_source1382A_23633, whole genome shotgun sequence
MSIDYIYKLLGMKEVELNATRAERDLIKAEVKKLKEELETLTKVAD

>gene_1048|GeneMark.hmm|59_aa|+|9952|10131 >NVVL01000050.1 Rickettsiales bacterium isolate NORP64 Contig_source1382A_23633, whole genome shotgun sequence
MPLLTIITNLGMGGTASTVRETRMRGENIITMPSQNRSLYMPNENRSIIMPPNRRKVDP

>gene_1049|GeneMark.hmm|100_aa|+|10128|10430 >NVVL01000050.1 Rickettsiales bacterium isolate NORP64 Contig_source1382A_23633, whole genome shotgun sequence
MTVKWHRDLIFKGDDAKDPSDVVDYTIDFGPMLQVDAISTITATGTNITIDSSSNSGNVV
TVWTSAGTDGTIATVKLLIVTTNATPRTFERSFKIEVRNQ

>gene_1050|GeneMark.hmm|125_aa|+|10427|10804 >NVVL01000050.1 Rickettsiales bacterium isolate NORP64 Contig_source1382A_23633, whole genome shotgun sequence
MTIRRQPQTLFTSAARTATIVSSVLSAEEVISSIFTIDCTAVTSTPSVVFSIEGKDPLSG
TFYNIIDSAAITATGTTTIQIGMNIIAAANIAANEMLPSEYRITATHGDGDSITYTVGAV
HMVQN

>gene_1051|GeneMark.hmm|99_aa|+|10830|11129 >NVVL01000050.1 Rickettsiales bacterium isolate NORP64 Contig_source1382A_23633, whole genome shotgun sequence
MPKTGVSVAAHNRQMRRDALREQLSNQKHVEHVIVLLDKLRDLDEELDNLEITRLSKVID
TKLKLINKYLPDDKEPSDLNLIAQVTTQTHEQWLESLNE

>gene_1052|GeneMark.hmm|512_aa|+|11122|12660 >NVVL01000050.1 Rickettsiales bacterium isolate NORP64 Contig_source1382A_23633, whole genome shotgun sequence
MNELELQKRKRLKSDFAFYARNCLFIRTKDKGLKPLQLNKAQLFIHQKLEEQKDTTGKVR
AIILKGRQQGASTYIEGRFMWRTTHEKGVRAYILTHEDDASQNLFNMAKRYFEHLPKFVK
PSTSAANAKELLFNLLDSGYQIGTAGNKSVGRSQTNQYFHGSEVAFWPNASEHAKGILQT
VPDADGTEVIYESTANGVGNFFHQQWKAAERGENDFIPVFVPWFWQEEYSKTPPKEFRAT
VEESELAEQYDITTDQIYWRRTKIADLSADGTDGERSFKQEYPMNAAEAFQLSGESGLIG
ADNVVKARKSEVNGNGSYIVGVDPSRGGDRFSIIKRQGRKAYDAKSWTGEAVNSLGKAVQ
KCKKVLDTACPTANKKPDMMFIDAGGGSDLVDRLHELGYDNVKAVAFGSSPLDADKYKNK
RGEMWGEMNLWLADENLDVEIPDDDEIQADLCASPYDRDSMDRIVLWKKEKIKKEYGFSP
DIGDALALTFAEPVSQGSKLSIPVSSGGWMNA

>gene_1053|GeneMark.hmm|685_aa|+|12653|14710 >NVVL01000050.1 Rickettsiales bacterium isolate NORP64 Contig_source1382A_23633, whole genome shotgun sequence
MPEKKLSTFSQAEKDKIIKQFMADFDLAFTAESENRADMVDDLKFAALDQWDEDVKNDRE
GRPMLTLDHIGQSIRKVTGGIRQNMPSIKVDPIDDGADKETASVLEDLTRQIEQTSKAQA
GAYMTAANFQVKMGYGVWRVNTVENTDDIFTQDIVIQRERNPFTWYFDPDATEPQKQDGR
FCIKSETMSADKFKMLYGKDVPSSTPSPSVGESMERWYSADSVRIGEYFVKKKKKRELTQ
LSNGVVIPTDEITQQDVQGYREQGIEPVKTREVEIDEIHWYKLSAFDILEHTIWPGKYFP
GIPVYGVEENIEGKTEYRGIVRPAKDPQRMYNYWNSAAAETIALQPKAPYLVTKDQIKGY
EEFWNQANQKNLPYLPYNPDAKAPPPQRIAPPSMQSGLLQQAQISAQDIQQATGVFEAST
GQLPEQRSGKAVIALQQEADLGTSLFMANLASAVEHTGRVILDLIPKYYDTQRMIRLRGE
DDSIKFVEINRPMLTPDGMRIQNDLTRGKYDVRVGVGPSYRTRRIEAASSMVELARVFPQ
ILEVSGDLVAKNLDWPGADEIAERLRKLLPPGIAEPTEEESEQMQQQQQQAQQQQQIQQA
AIQLEMAEKQAKIGNIEADTANKKASAVKGLTEAEQNDVENAVALAELARDQGNQQLMNQ
ALAEIVGLIQSAPAPQQAPTTGLVG

>gene_1054|GeneMark.hmm|65_aa|+|14740|14937 >NVVL01000050.1 Rickettsiales bacterium isolate NORP64 Contig_source1382A_23633, whole genome shotgun sequence
MHILIDGNEYRVDEQTKLGDYIKGKGEDVYFICTQAKMDEVDKLRQFAVARQYAHDQLGK
VAKLF

>gene_1055|GeneMark.hmm|267_aa|+|14986|15789 >NVVL01000050.1 Rickettsiales bacterium isolate NORP64 Contig_source1382A_23633, whole genome shotgun sequence
MSEEAESEVTQDAPIAESSPAPDATTEIENVDAIEPKKVNKVQERINQLTREKYEARQET
AALEERIKQLETNKPVTKEPEIVAPNEDDFDNHSKYQQANAKFVAETASNAAYDRISAEN
QVRDQANTETARQAELKTKKAGFDANLAEKRGNFEDFEDVAYGHQFMSIDMAERLFDMDK
GPEVAYHLGSNLDVAEKIFALPPIQQAIELTKIEFQVDALKPKLVSGAPDPITPLGSAEK
VSKDEDDMTDAEWLEARYAQINARNSQ

>gene_1056|GeneMark.hmm|397_aa|+|15799|16992 >NVVL01000050.1 Rickettsiales bacterium isolate NORP64 Contig_source1382A_23633, whole genome shotgun sequence

MANTLLTTDKILRESLRVLHAKLNFIGRVNRSYDSSFANKGAQIGDTLRIRLPAQYEVTD
GAVLTAQDTVEKNTSLAITSRKHVGAKFTDEELQMDIDSFSELHLEPAMSVLAAKAESDM
INSVYKQVYNQVNNVASDMTYKLIGNGRKLLVDNLVPQDNTVTACLNTSDNLDLNDALKG
LFNPQSTIGANFREGGIGRTSGYSFFENTHMPRHTTGTDDGTGDYLTDSATAQTGASLTI
DTGAGTMTAGDVFTVAGVNRVHPETKVDTGNLQQFVVTTAIGASATTITFTPEIIATGPY
QNVSNGAANNQALTKVGGNAAAHDVSLGFHRDAFAFATADLPVPGGMDMAGRKVMDGISM
RFVRGFDIVNSRFVSRFDITYGFNAIRPELAHRWANN

>gene_1057|GeneMark.hmm|160_aa|+|17005|17487 >NVVL01000050.1 Rickettsiales bacterium isolate NORP64 Contig_source1382A_23633, whole genome shotgun sequence
MALEQIGTNTDEGAVMPGRHLEVISGLAGGTTARQLLASESGSLCLFDDAAGQIYILPAP
VVGMVFDFLTTVAGTSNSHSIDTDASTTFMGGGVAAVSTAVAEGGDSFVATISSTVSLDM
DSDVTGRLVGSAIRAVALSSTTWGISGTTHGVGTLATPFA

>gene_1058|GeneMark.hmm|66_aa|+|17528|17728 >NVVL01000050.1 Rickettsiales bacterium isolate NORP64 Contig_source1382A_23633, whole genome shotgun sequence
MVCTECGHDNSAVAAKDPWCMYKFENGQVVNKLFDPDKIPKGWFDSPKAAKAGKKTSKAT
SEGLTV

>gene_1059|GeneMark.hmm|129_aa|+|17725|18114 >NVVL01000050.1 Rickettsiales bacterium isolate NORP64 Contig_source1382A_23633, whole genome shotgun sequence
MTTAQNMIDSSAKMAGILAEGQSLESGVNTDALNRLNRMFARWANIGIDLGLATLAASDT
VYVDVADEEAIELSLALRLMVKHRRQIAPGLSEAGDQMVTELQSKYSNLPVMALDAALSG
SQRYNINNG

>gene_1060|GeneMark.hmm|465_aa|+|18107|19504 >NVVL01000050.1 Rickettsiales bacterium isolate NORP64 Contig_source1382A_23633, whole genome shotgun sequence
MAKIPVPLVGPTYTNRSLPVGAQVTRNFYVEVNQGGDEAVSMQPFPGLKEWGSAGDGFDR
GLGVLNGILYKVSGDELYSVDRRGDAVLIGTILGSTRCTLVEDTAGNLVITTGHGKPYAY
NGATLTLGKDGDLPKASTAAYINDRVIYESTTGLAFADLDTPLTVNSANILKANTKADGM
MAVVAHRQQIFAFGAKSIEPNSFIGTGTPPYARVNNAVQQVGTSAKYSVSTNNNYIYFLD
NNKQPSRMSGITIQAIGNPAIGQAIAKYSDVSDCFTVCFSFDNQDFCLFSFPTGDETWLF
NEQSQLWTNLAFGVEDQHLISDYQFVYGKHLVSDRRNGNIYEIDMNTYTDNGLVIHRQRD
TISISSKDLGPPGHKLFMNSLQLVIEPGASLVTVQATVMMQYSDDNGRTWSSERWADIGE
QGDYTYHVEWLGLGSFYNRMFRFSMSDAINWVLISAHADVEMGLG

>gene_1061|GeneMark.hmm|178_aa|+|19497|20033 >NVVL01000050.1 Rickettsiales bacterium isolate NORP64 Contig_source1382A_23633, whole genome shotgun sequence
LAEKIAPYVQPIPKFVLETNNQEFINWFSYDNLWKQDINVNLGFGDAITNVTNSESFETS
SASSEYQAHKRELEQIEDLIPTTQVRELYSKIVTANYTAVNNDFCDIRNGSTVTLDSNAG
WNDQIITTNGDGSSVKVTSTIELRYKGQRGNTITIRQEGTSIHWYLFSDGTEKFWRAS

>gene_1062|GeneMark.hmm|50_aa|+|20030|20182 >NVVL01000050.1 Rickettsiales bacterium isolate NORP64 Contig_source1382A_23633, whole genome shotgun sequence
VSIEAESIDHERDIETSAEEQVRLLKAVIAGLALILDVEPELLIKIAEDL

>gene_1063|GeneMark.hmm|201_aa|+|20184|20789 >NVVL01000050.1 Rickettsiales bacterium isolate NORP64 Contig_source1382A_23633, whole genome shotgun sequence
MAGIEFLYQDDQTGFDARVKGSDNRFNASSRSDSRGYYNARDAGQAYSVAFEMTTPVAGE
YFAYWQNNHPNSLQLVISAIGVNAVQASRIKLHVVTGTAATGSGVTAITPLNMNRSSPNT
AASSGVTTAVQGDTGATGIGTLTSIGLVDFLYVAATGHEEFRLDDRLRLGVGDAIALEYD
EGTTGDVAGVMFGYYEKKGRA

>gene_1064|GeneMark.hmm|419_aa|+|20789|22048 >NVVL01000050.1 Rickettsiales bacterium isolate NORP64 Contig_source1382A_23633, whole genome shotgun sequence
MGIKVTPSGADQSGQDARASLHNRGGVVGSVNYVREYDNLRADIKFFFNETYGGEMNQAI
VSSEVATVIHDGGDTTNADSGTATGGTSGTNLEDTGQNFLTTVGVGMTVRNTTDTTYAVV
TAVVDNDNLTLDGSIMVDTETFTVGAVWVGTGDASFNFASGAKIVCTAANNNDAALFTNA
GTSDMQHFGVLTGKIDLDAYNSANHSVEFSFLSSSVLVGNAVNINDYINVIDVSEQSFSI
PLADMGITSQTVNELQAVVLRSGGSKPTFKLDDLQLGDASGSATWMALPDHGTTFLAKRL
RFVLADNITEIEYDKFMGLAALTNGVNLLIVSNGESLFQGIIRDMGGFYLAGIDEKTSSL
GATNSWTALEIDITSPVVLDGTSGNKAGLDDGFYLTISDDLSGLLRFQGIILGDTVLKE

>gene_1065|GeneMark.hmm|110_aa|+|22050|22382 >NVVL01000050.1 Rickettsiales bacterium isolate NORP64 Contig_source1382A_23633, whole genome shotgun sequence
MARNPFRDIQQIATTTLTTVYTAATGINSTISVVSLVNTTATALNIDVYHNDGATDFLKN
TIHIPGGAGRERLYYGFERGVLAAGDSIKVQADGATAFNLAIYGSEIESS

>gene_1066|GeneMark.hmm|156_aa|+|22357|22827 >NVVL01000050.1 Rickettsiales bacterium isolate NORP64 Contig_source1382A_23633, whole genome shotgun sequence
MGQKLRVARTFDIDVINAVLKHPKVKDCIGDDFSGDVEYPIVDNIYYLAVYDRELAGMFV
VYPLNAVTFDAHSAMLPGKYGNKAKEAGRLAIDWVFNHTGCLKINGSTPIYNKLALKYSE
QIGFIREGVNKRSIMKDGQLHDQVYFGLEREKWVLS

>gene_1067|GeneMark.hmm|239_aa|+|22812|23531 >NVVL01000050.1 Rickettsiales bacterium isolate NORP64 Contig_source1382A_23633, whole genome shotgun sequence
MGIELAIGGALLGSSLLGAREQRKGAQSAADAGERAGLAGIAEQRAAREAFEARTQPFLD
LGLSAGEQLQGFLQDPSAGLDQINPVASFLQEQGFRQIREGGSGGGRNVDQDLSRFQTGL
TSTLVPQFQQQRFNQLFNVLGLGQNAAVGQGQAALTTGGNISNLLGNIGQAQGQGAQQKA
AANQGVISNIAGLAGFGFGGGFGGAPAGGGAPSGGFNALNPNLFNQSQQPNLLQSTGTF

>gene_1068|GeneMark.hmm|368_aa|+|23531|24637 >NVVL01000050.1 Rickettsiales bacterium isolate NORP64 Contig_source1382A_23633, whole genome shotgun sequence
MARFNPLADFAGGKSSALGIKSQEQGIAREAAAAPVRNQLAQIQLQQAQTGQRREGQQFD
QEQALRRIKLLNQGAKAIGATDPSQWQQAIPRVVQFLGAQGVDAQELAQGVTPESLTSFI
AQTDEFLRDPSKLTSAQKEFASLTEGFSPEDKIKTRRVKAGIDPRAVGSAIQTISAKNMA
ADIAKTEGTIAEGKEFGKLTGSSRAKAIDSGFDKIQSISKNIDNINKAIEEIDAGASTGA
IESRFFPSIRASTVALEQIQSELALDVVGATTFGALSKGELDLAKEVALPTKLEPAELKK
FLVDKKSAQEKLMAYYQEQIDFIDQGGTKAGFLRSKERGVSTGQEGQIMVDAQGNRARVF
KDGTFEEL

>gene_1069|GeneMark.hmm|641_aa|+|24640|26565 >NVVL01000050.1 Rickettsiales bacterium isolate NORP64 Contig_source1382A_23633, whole genome shotgun sequence

MAFDLSTAKPVKRRFDLSTASPVVGAIDLEAFDPAGESVPSGEARPEFIGSGVLEPIAAI
GSGALASVAGGLAGLGAELTPGAEPGVGGDVVRQFQEAAFQPKTRAGQEGLENVGNVIQS
MVDRFNVPASGIGALLELVSGQGLDQAVQTIEGIQAQGVGPTVAERTFQQTGSPLAAATA
GTIPDLVGAILGTKGAGQALKTPQAQAIGREIQAVPETAKQVVEGVAGLQRPATRELARE
IQAGTADPAAARVELKPLRTFEGEPTGIERFFDTRGPKIQKDSLAAQAVKQGFDEGVIQP
LKQASRTDKKVMSRMAEVAERISKDKLFGMKNRPGDLAGDVLMNKVRAVRDANKRAGKAI
GIEARKLRGEKVNVANAGDKFLEDLDGIGIKLGDDGKLDFTGSIIEFQGGPKSTIRNIFK
RIGDIENPTAHDVHELKLLIDSEVTYGKGLKGLAGKAERILKDYRNNLDVILDESFTDYN
NANVAYSETIKALDALQDVAGKKMNLLGKNADKSTGTLMRRLMSNAQSRIRLLDAIEDIE
EAVKKHGGSDLLRIEGKAGKADNLQMLVLFADELDNVFGPAARTSLQGQFDQALQQGVRA
GTTKAGALDLGVSIVGKGIEKLRRINKPRAFKAIKELLKEP

>gene_1070|GeneMark.hmm|725_aa|+|26565|28742 >NVVL01000050.1 Rickettsiales bacterium isolate NORP64 Contig_source1382A_23633, whole genome shotgun sequence
MANQKMSEVPNPIIVDAAGTAGSGYVLKAYDVGTTDSKSITIDADATSPQTSVTANSNGV
WEVSGNEIVPHINGVYKWGIFANATDATANTPFYMGPFDSIQGSASTNNWFFAENYSTFA
LAVAAAAGKTLFISSVIAPGATETVAGLTIVILEAGAINPTTGITITLNCTVHNWSQSQT
VFGGAGTIAGWHNLVLGPEAGAALTTPVFAGHNTMIGAYAGQNTTTSRDNTFIGYECGKA
HIADASSFGGNTAVGANCFDVNTTGYQNTAIGNTALDDNVTGNFNTAVGYGALQKNLLSN
NTAVGIQALYLGNNNDDATGIGMFALANCTATTTKNTAIGFSAALNFGKASDGYLGGDTN
LFCGAQAGLASREGNRNTFVGGASATHGATTDMTGDDNTFVGAVSGDLNIVDGDKSTILG
AYSDVPILITGQTTSTTTDKLVDSTEHFTTNAYVGLTVKNLSDTEVTGNHDGSSGASVLT
DSGESWGVNDFLGRIIYNTTDGSSGTVTANTGTTITATLTGGTENDWDASDSYTLRVGRT
TVSAVDSATTLSLNHNIMITGENYEMFDESSTVSLCAGGSSNAPKPRFYSPSDGSATIGK
LGSSGRLYKNLSESVTTPGAASSFIIQVNIPSGAKIIGCQLKVDTALGSGETWSAAYSGG
STQSIVTTGQVITAGHIVDTFFDVNAATDIASAEVDVTITRDSGNFTSTGAIRAIVHYEA
FQSMS

>gene_1071|GeneMark.hmm|86_aa|-|28705|28965 >NVVL01000050.1 Rickettsiales bacterium isolate NORP64 Contig_source1382A_23633, whole genome shotgun sequence
MNIGAGNFIELGEHDNCVIIGDNLKATQDYHLIIEGVCDQVMTPEEHKLINAAVAVTKQK
CEAYYGNLLYQAKTLSSYSGTPRNER

>gene_1072|GeneMark.hmm|79_aa|-|29169|29408 >NVVL01000050.1 Rickettsiales bacterium isolate NORP64 Contig_source1382A_23633, whole genome shotgun sequence
MAQYLKKGSKVYIEGKLQTTKWQDQKGDDRYTTEIVVRDMQMLDSRNSGESTQTRSAQPP
QTQVDDGKQIDDFDEDLPF

>gene_1073|GeneMark.hmm|68_aa|-|29393|29599 >NVVL01000050.1 Rickettsiales bacterium isolate NORP64 Contig_source1382A_23633, whole genome shotgun sequence
MKGVNKAIIVGSLGADPESKTMPNGYMVCNISIATSEQWLDKNGQKNEKPSGTERSALDV
PPRSWPSI

>gene_1074|GeneMark.hmm|211_aa|-|29624|30259 >NVVL01000050.1 Rickettsiales bacterium isolate NORP64 Contig_source1382A_23633, whole genome shotgun sequence
VFEFHDIEQNTDEWLQLRCGRLTSSNLGKVMANFGKSFGEPAKRLAAQIAVQQITGKCES
NGFSNEHTERGHEQEPVARMLYEERFFCDVGNGGFFSTKDLGCSPDGLVLDSGAVEIKSV

IAATHFATVKRQNVDPAYRWQYIGNLKFTGRDWLDFISYCSEFPADKQLYIHRLNKSDMT
KEFDMIDSRVEQFMALVATTKETILESNYSN

>gene_1075|GeneMark.hmm|188_aa|-|30252|30818 >NVVL01000050.1 Rickettsiales bacterium isolate NORP64 Contig_source1382A_23633, whole genome shotgun sequence
LLWLGLQLNIGESKGKVMEGEEKTHYRKAFNSPYLSSADIVGPTVLTISRVGLQSDATNK
TKDSFNTAYFEEQELRKGERLKPMILNAHNSKVMKDLTGSHFLEDWSNVRVTIYVDPNVR
FGRETVEGLRISPQPPAESKATITPEKGQMWTNAKNAYLRDGDFEAVLKRAHISEENQAL
IVKECGSV

>gene_1076|GeneMark.hmm|61_aa|-|30873|31058 >NVVL01000050.1 Rickettsiales bacterium isolate NORP64 Contig_source1382A_23633, whole genome shotgun sequence
MRVSPTQTTVNAIHNKHVGHQINLDCKLMGMYLEGLSLIKVEQQRKEKQEKWAEFIKVLK
Q

>gene_1077|GeneMark.hmm|82_aa|-|31055|31303 >NVVL01000050.1 Rickettsiales bacterium isolate NORP64 Contig_source1382A_23633, whole genome shotgun sequence
MINKQLNPLIDFMINRTDGIPGSDNCDDIAVLIGESITLEIIEYFQDYINQPSTDNANAL
AKKLTQTFSDYVEKEDTFRIMQ

>gene_1078|GeneMark.hmm|57_aa|-|31300|31473 >NVVL01000050.1 Rickettsiales bacterium isolate NORP64 Contig_source1382A_23633, whole genome shotgun sequence
MLPEYIKRKYGTQRGALQAFADDIGRPYQSVQRWIRNKYRVEDGRVVKKQTVVVCVL

>gene_1079|GeneMark.hmm|76_aa|-|31626|31856 >NVVL01000050.1 Rickettsiales bacterium isolate NORP64 Contig_source1382A_23633, whole genome shotgun sequence
MDLTRFDAAKKALEFAGFNVCDDGGVLEISKDRICETFYQTGEHFNSLSFHKSVESVVAY
AEKSVILGKKYPLSEL

>gene_1080|GeneMark.hmm|95_aa|-|31877|32164 >NVVL01000050.1 Rickettsiales bacterium isolate NORP64 Contig_source1382A_23633, whole genome shotgun sequence
MAITINQILELDINSRNGLVDIGYNDSLRNFYRFYELDRLEKDITKALKISSLDQLQAVA
IKYKNSGLNAFNCDDLIEDLMGDDYYDWADANILD

>gene_1081|GeneMark.hmm|55_aa|-|32910|33077 >NVVL01000050.1 Rickettsiales bacterium isolate NORP64 Contig_source1382A_23633, whole genome shotgun sequence
MRPFDVSHLASKRICKQAEQLCNELKEAIHKYDNEMPIALVLGVLDIVKKEIVED

>gene_1082|GeneMark.hmm|62_aa|-|33064|33252 >NVVL01000050.1 Rickettsiales bacterium isolate NORP64 Contig_source1382A_23633, whole genome shotgun sequence
MEVIMCEMPKAFSTKTRKARKPHICCECNHAINPGEEYQYSSGIWDEPERHLLNKGEGNE
AI

>gene_1083|GeneMark.hmm|64_aa|-|33270|33464 >NVVL01000050.1 Rickettsiales bacterium isolate NORP64 Contig_source1382A_23633, whole genome shotgun sequence
MECNRGSVCKESDHQVKTLEKAVEWFYGVELEHLPIRVNQVTAHNGRVQITIFDCDGVPI
HKRW

>gene_1084|GeneMark.hmm|103_aa|-|33454|33765 >NVVL01000050.1 Rickettsiales bacterium isolate NORP64 Contig_source1382A_23633, whole genome shotgun sequence
MVITKVWIMANRTKKGAWTKSQIQVLGIDWPARRGWIQDIAGAEISEKNRVLFELKLPVK
KVPVDLAKSSYSSVMARIRDLTDQQISNLIRSLETEKWYRNGM

>gene_1085|GeneMark.hmm|27_aa|-|34272|34355 >NVVL01000050.1 Rickettsiales bacterium isolate NORP64 Contig_source1382A_23633, whole genome shotgun sequence
MVVIICAYCGLEAEKTEGEKKEGEKRK

>gene_1086|GeneMark.hmm|66_aa|-|34365|34565 >NVVL01000050.1 Rickettsiales bacterium isolate NORP64 Contig_source1382A_23633, whole genome shotgun sequence
MNKRQVGFVNIGLIFIVIVAVGGNVIANTKLESQRKLLNCIYAKPTYLDNQVNYCENLLK
TKWVVE

>gene_1087|GeneMark.hmm|188_aa|-|34562|35128 >NVVL01000050.1 Rickettsiales bacterium isolate NORP64 Contig_source1382A_23633, whole genome shotgun sequence
MAYSDYGGYAYRNNARVIERSDAIFTEEGLKSTPGQWPGFSFPEGRRGRSHHVILGDGPV
HIGMNKQSSQSVYLHGETFDIDPLIIARHPNTNTKWIGDNGEEHSYVDHESLLNTTVVEV
ILEGHKIEIFWADTDNYYMHIRLTQPDDNIWIGWSGYGVGAGLEDCGYGYSTEDIIGASE
DVWKVKLR

>gene_1088|GeneMark.hmm|59_aa|-|35239|35418 >NVVL01000050.1 Rickettsiales bacterium isolate NORP64 Contig_source1382A_23633, whole genome shotgun sequence
MTEQEYILAANLAKISTAVTIMRGVSPGNNYGISDDDYKLILLILRNSQTRLHELAKTD

>gene_1089|GeneMark.hmm|88_aa|-|35415|35681 >NVVL01000050.1 Rickettsiales bacterium isolate NORP64 Contig_source1382A_23633, whole genome shotgun sequence
MEIILIKALKYGYTRIIELDDFQYSRESHIVISKPLKIEFEMLPESDQVNAEVNVLRDIK
EKGLADAQVVANKIDEKIQSLLAIEDKR

>gene_1090|GeneMark.hmm|224_aa|-|35681|36355 >NVVL01000050.1 Rickettsiales bacterium isolate NORP64 Contig_source1382A_23633, whole genome shotgun sequence
MIINTTGELLDLRDKKGVITLEDDTVIKCDVPYSVGAEINGLHAKGNLDCKGYLYCEGNL
DCEGNLDCKGNLDCEGYLDCKGYLYCEGNLDCEVNLDCKGNLDCKGYLYCEGYLYCEGYL
DCEGDYLIITGTLYWSHASMPVLPAKHYINRVLPQKWQREYYQERLGIDISEGCYEEICI
TVLKQIINLLKQDKWTPTELWMLETLRDSFKKPPVWVSEIRGDK

>gene_1091|GeneMark.hmm|78_aa|+|36492|36728 >NVVL01000050.1 Rickettsiales bacterium isolate NORP64 Contig_source1382A_23633, whole genome shotgun sequence
MRKLDKSKGERQTTLIGFRANYTLKDIANQCDVSTVAIHKAVKDINDGVRPSISLYVDKK
DKIIYAFETDIRPVFGFK

>gene_1092|GeneMark.hmm|128_aa|+|36753|37139 >NVVL01000050.1 Rickettsiales bacterium isolate NORP64 Contig_source1382A_23633, whole genome shotgun sequence
MNIEDLTIKQVREIQGLTSGGTVSVAANNIGDMVGSKVIVRTYSAGVWFGTLYAKDGEEV
ILQSARRMWRWWAAESISLSAVAIHGIRCDKSEICEEVGKVWMKAIEIIPCAPASVNSIE

GAENAKAK

>gene_1093|GeneMark.hmm|65_aa|+|37123|37317 >NVVL01000050.1 Rickettsiales bacterium isolate NORP64 Contig_source1382A_23633, whole genome shotgun sequence
MQKPNKFDGDGSGDGYGYGDGSGSGDGSGSGDGYGYGDGSGSGYGDGSGDCYGYGDGSGS
GDGDG

>gene_1094|GeneMark.hmm|243_aa|+|409|1140 >NVVL01000051.1 Rickettsiales bacterium isolate NORP64 Contig_source1382A_23642, whole genome shotgun sequence
MYSDVAVIIPSRLGSTRLARKSLAQIGDKSLISHVVSKLVKLVKNGLYVATDSEEIAQEA
EKAGATAIMTDEDCPSGSERVFQAFEKIPNHHKINYIINVQGDMPFVDPNAVNDIIKKLK
TTDYDIVTPVVKVTADVAKNPSNVKVVVDLAGRAMYFSRSLVPYDATEFLYHVGIYGFKR
DALEKFVNLEQSEYEKIEKLEQLRALEHGMKIGICFSSEIPISVDTQEDLNKAREYYSWI
SET

>gene_1095|GeneMark.hmm|367_aa|+|1122|2225 >NVVL01000051.1 Rickettsiales bacterium isolate NORP64 Contig_source1382A_23642, whole genome shotgun sequence
MDIRNLIIIATFIICLPFSLAAKTRIKDITGIEGVRDNLLVGQGLVVGLNGTGDKLKNSP
FTEKGLTDFLERLGVNVQGAQIKTKNIAAVTVTANLPPFARMGTRINVKVSTIADAKSLN
GGTLLATPLIGADGTVYAVAQGSIVIPEFAPASGGVKTKSKAVETNAYIQNGAIVENEID
FTFTNMRRIKLFLYLPDFGTSVAIAAAINNHLSGNTARALDSATVEIVVPTYRTDDLVEF
IAEIESLEIKPDYKAKIVINESTGTVVIGDKVHIRPVAIAQGNLVVNVGHLEFDKKYGPE
TDEETRDLVNGFVEERRGRGIRQLEEGANLSDLVAGLNRLGVYPRDIINILHNMKSVGAL
DAVIEVR

>gene_1096|GeneMark.hmm|217_aa|+|2231|2884 >NVVL01000051.1 Rickettsiales bacterium isolate NORP64 Contig_source1382A_23642, whole genome shotgun sequence
MAGYFGGGIQNVEKPVEHYKIIDQRKNLSPERARGAGRFDEVMKKAASKLEAPLKAGLEP
GLEAGPSDKKVLPVTLRPQITRSNSAHPRSPGHLQTPGSEHLQTSNSKHLQTPEMHRKSS
LSTGMNPRETDDGTKIALKRLSKEMENQLMGMFWVMIDNARENDPEGGFAEKMFRTPYLH
EVVKSGADPELGEIGKAIYNNLVRNDRENVESVRAKK

>gene_1097|GeneMark.hmm|161_aa|+|2865|3350 >NVVL01000051.1 Rickettsiales bacterium isolate NORP64 Contig_source1382A_23642, whole genome shotgun sequence
VLGLKNNINYTNKLNQKQLIGDLSRVLKDLIDIIARENILLEGGRISQLDAVVAEKFAAL
EKFNQAEINIEIYKKQGGSFDQRKPDIVQAMELFQKFDKHTRRNEVLIQSNIEVSAKIVE
MYKESKKAQALRKFGYNKKGKIAVSKNVESIMPSIGLNDTI

>gene_1098|GeneMark.hmm|916_aa|+|3362|6112 >NVVL01000051.1 Rickettsiales bacterium isolate NORP64 Contig_source1382A_23642, whole genome shotgun sequence
MGNWTTIADTLKNTGRVLEEGPAYNLQRLQDPNARVVRASLGTDNIGSKGLLRTTFRMDR
PTNPLRERVYMSQVTELENRNTYTEFMDVMQTVIGGGGSSQSSRLVSVIDDFFKQAKILE
SNSGAAMRAAFVGKAENVATVLTQATDSITELRLEADTRLRDSVTSLNSTINALYALNQN
ILSAQTPLKLYDKRDELTRELAKAFDIKVSYGVGGVAKVQSKSSGEMLVSGDSFAQFSYE
GILTKTDILDHADYPPLKMHHYNQDQKQTNTSIFTDFKSSFSLFKGGKWEALIKLRDEIL
PEAWCCVDTLAQNFSGKINILHNDGSSCPAQGYFKSSGEHRGADGLDGLGAFSIYAVDQN

GNQLRGGAGLLNQVNIDPATILSSVEGKAPTMADLIRELNEKLDLAPSRDRAAMGAILDD
AGVQIDGQYLLNNVQLLARSQVSGVAPADSVTFELDLQGSSHFGSNIEILGVKTNDVGGG
NDLALAQNQLPSSFRLEKDTNAASGQFFTLQNSGAGREVTLEIRVIGDNGVVSRGEVSFV
VNPAITPGARVATNNVVAGDFAHPIGTNLSSHSGVARAKLVDSNGAEIDPLSGGSGKLVI
DTNNSSYRLVIQGGGFGGKFGFNDLFHYDAHTGFMEVNSDISANVGNLAVGRIQKDNGSD
SGRMVGDVAASAPMLFGGGAIAAGDVVSVNGIDFTFRNAPLPVPADPRDVDVAGTPLLNL
VNRINAHPALQNLVQATLNVNTITVTAKAAGTGGNAIAVGTNLVGGATASINGFAPAPGL
VISTLIGGTDKITINQEFRYDMDDNNLQILEAISNLQGQAIHVDCQGIVPESFVSLSNLA
TMVTGFFSNYVNDAEKKSDIAVTVLKQTDSAIKGSFGISREEEYIRSVDLAQYMNMLSHI
LGQMQQIEKTILDNIK

>gene_1099|GeneMark.hmm|316_aa|+|6144|7094 >NVVL01000051.1 Rickettsiales bacterium isolate NORP64 Contig_source1382A_23642, whole genome shotgun sequence
MALSITTTSSILESKPIRKGEVARLTSQLDDAMASITNPDRFSSRAKLDASGLNRLYDDA
QRAILAANGNKISAQVISVKLENENRGLAEFNAALVAFERDMGEMSDHAGTDAEKADRAL
EKMSLVLRRKDPGGRYIFGGSTPYIDPLSKLDNNGVRVAIDLKASTSLIGGQMSRNYSDT
SPNETVVTVSAFHEVKEGFLHPEMDAIYKTIGYINMVKDGQFTANQRDAAQLDARQARGA
AAMAIGLEEKKVEASLYVNDSEINSADSKNNYLFSSDLVESTGRMQSLMHSVQANISLDG
AKTKIFDNLMDRMRLH

>gene_1100|GeneMark.hmm|564_aa|-|7116|8810 >NVVL01000051.1 Rickettsiales bacterium isolate NORP64 Contig_source1382A_23642, whole genome shotgun sequence
MSSLKREGSEIDDAPGAKRVKLGPAMEPKTTIGVKRERSEEDTRPDSNGSAPPAEVKRAK
LESTACVTQPALLTGNPLTQVETKVFDAQHLSPEEIFTSRFHIPSEAAAPYTHDDLLSAL
KERKTSKTRQESYLENLPTEYNLKDLVKALGENNFAKGLVQLPVSSQKWLVKKYLSGENE
LNAAIMELDNNNFSTGFGKLRASIQTVILSQYTDTESLRDFILKLGNNDFAAGFEALRRP
AKAPIIKLYATKTKLNELVDLVSEESLHTAFTQLSDTNKKNLVGSDFASELCDHLPTKCM
ARMLCYMLPNAQKALLTNVLALDTLDRLTELMSCSNYFMLQTDHTETALIDEIIQRYLDY
NNLDVFLKIPLSLFRGLQPKVQILVIKIYFEEDKLHSLLFHLCVPGFSNKKQKEKAKMLE
EYLDCSAPDKLSYPLTPAFINMSEETKTAVIKACLEYGEFNFLLLLQPEYSLATLATLGN
HFVSTKDAILGLTPEDPTSRNQIPEDLCRLIVDFTYSEVAIPQSDDSAKYLPPSAIIAYL
QSLETPDTLPPVYLAGDATLMEID

>gene_1101|GeneMark.hmm|164_aa|-|8953|9447 >NVVL01000051.1 Rickettsiales bacterium isolate NORP64 Contig_source1382A_23642, whole genome shotgun sequence
MTYYSYIQISVGRVLLVSDGTNLTGVYVTGQRYFPEIKPEWEEKSDLQLFIKTEQQLIAY
LAGEIGEFDLPCKLEGSELQEHAWRAIGQVRRGETISYKDLASRTPYMPAIRAIASAVGQ
NPISIIVPCHRIVRHDDSLGGYAAGLEAKQRLLDLERKPVRGKG

>gene_1102|GeneMark.hmm|247_aa|+|9850|10593 >NVVL01000051.1 Rickettsiales bacterium isolate NORP64 Contig_source1382A_23642, whole genome shotgun sequence
MLKTFAKKYMVAFTLLFITALPLSSNAASASITRHDSYLDEDSYFFDSEGAKVFLERYEG
STVLLVFWATWCGACVNEIPVLDNLQKDFRKLPFKVIAVSEDYHGVQAVQKFFEEKEIRH
LEIFHDYQNQFFKALSISSLPTALLIDANGKMKTRFKGRIKWHDEKIRAMILSEIAGNPE
TPHNTYKPRELNMQVRPTPPANQKVPAKQVAPAGQEKQSAPMRQAAPAKQIDNKEVKKNE
QKSTKSK

>gene_1103|GeneMark.hmm|311_aa|+|10565|11500 >NVVL01000051.1 Rickettsiales bacterium isolate NORP64 Contig_source1382A_23642, whole genome shotgun sequence
MSKKALNQSNNTITDTRPILSRTAPTRLEQVLMALPFGFGKSQPVVAVLRLEGVIGKGGA
CRSGLNFGALNKLIEKTFKTDSLDAVCLVINSPGGSPVQSELIASRITSLAKEKDIPVYS
FVEDVAASGGYWLACAGDKIYASKSSIIGSIGVISSSFGFQDAIAKLGIERRVYTEGKTK
SVLDPFQPAKTGDVKIIKKLQKEIHNHFIDTVRTRRGGKLTQSDDLLFNGEFWCGQTALD
YGLIDGIDDLYSFIQTKYGDNVKIEYIENKTSWFKKKLGMHKIAKEFAQDLTETVLDSAE
NRLIQSKFDLK

>gene_1104|GeneMark.hmm|232_aa|+|11511|12209 >NVVL01000051.1 Rickettsiales bacterium isolate NORP64 Contig_source1382A_23642, whole genome shotgun sequence
MSNFRASILTIFPEMFPGTLGHSLAGAALERGDWLLDVINIRDFGLTKHKNVDDTMCGGG
HGMVMRPDVLGAALDKALEESPGAKIYYPSPRGIPLTQDVSKQISLEKNIIILCGRFEGI
DERVIDEYNANQISVGDYVLSGGELAAMTILDSVVRLLPGVLANQETLKEESFEVEEEGL
KLIEHPLYTKPSVWRGRGVPEILLSGNHAAIEEWRKAKSIQITKRRQRNLLK

>gene_1105|GeneMark.hmm|131_aa|+|12234|12629 >NVVL01000051.1 Rickettsiales bacterium isolate NORP64 Contig_source1382A_23642, whole genome shotgun sequence
MTNLIDQFEKNQIEKLTANKEIPTFRAGDTVRVSVRVIDGTTERLQSYEGVVIAKRNRGI
TSSFLVRKVSHGEGVERRFMTYSPIVAKIDVIKRGIVRRAKLFYLRELSGKAARIKERKT
YAPAAQKKKTA

>gene_1106|GeneMark.hmm|190_aa|+|13125|13697 >NVVL01000051.1 Rickettsiales bacterium isolate NORP64 Contig_source1382A_23642, whole genome shotgun sequence
MRVDSSDDKLFSWYFESIKEVLENKRGNDDFDVYNDFMMNLDLADKADVNIPKLATKLLC
QPFVVSTIIKQVLKTTCSQHVHVPSVEETSSSSDANLKPDDATITLEMRTNALAADAPVE
LDDLDHPVKRTHGANDTPRSIADDSETLDEVSKADQASSQLRFVLRLQGGDVEVSGEGSS
TGFTVEGDAS

>gene_1107|GeneMark.hmm|101_aa|-|13858|14163 >NVVL01000051.1 Rickettsiales bacterium isolate NORP64 Contig_source1382A_23642, whole genome shotgun sequence
MSKQGSNGTSEREETLSSRFRAWMKAERANSAQSIFELHKLEAETQIILKEIHSTQDKIE
RLQDEIEWLHGGIEGNLPRQSIHWMELSGESTRSMDTSSDG

>gene_1108|GeneMark.hmm|372_aa|+|14649|15767 >NVVL01000051.1 Rickettsiales bacterium isolate NORP64 Contig_source1382A_23642, whole genome shotgun sequence
MEPITATTITIKTRSAVKTVPVKTVPVKTVFAAEIDIKNDLAARLTTPVLSQIFLSFILF
LFALVASNTAFAKAPRKKLIAITQIVEHPSLEQAKRGILDELKNNGYEIGVNLKVIEKSA
QGSIANSAMIAKKFVSLSPDAIVAISTPSAQSVLKIARKAGLPVVFSSVTDPVAAGLVKD
LSSPVNGVTGAMDYPLIDEGVALIQTLLPKAKTIGFLYNVGEANSVKTVNLMKKAMDGKF
KYIDAQLANSNQIVQAVSALVGRVDAVYIPSDNMVFSAMPKLIQLSRKHKLPIFSSDPDS
VRSGVVACIGYSQYAVGKVAGKLLVRVLAGEREIAIEKPSKAEIFVNKKSAYIMNIEIPS
EILGIKTNIVGK

>gene_1109|GeneMark.hmm|304_aa|+|15853|16767 >NVVL01000051.1 Rickettsiales bacterium isolate NORP64 Contig_source1382A_23642, whole genome shotgun sequence
MMFVCVNVFCYANIASAEEKILIAVNQFVSHVALDKANSGLKKALTDRGILPDRARMIFG

NSQGNITNSVQISKHHASLEPAFMVSIGTPSAQTGLKAKSGKTILGFLAVTDPAAANLEQ
KQVIGVTDNPPIRELIDITLQVFPELKTVGVISNAGEVNSQKVIENLEKILAGHNIALKK
AVITNPSDIKAAMNKLAGSVDLIYIPQDNSVVSALSHIARLSNSLNIPLIANDPTLSASG
VMIALGANYFSSGMQLGNMIADLIEGKGVRQNIQGSDKKELKINHKIAKMLGVSVPKNIK
EGDK

>gene_1110|GeneMark.hmm|286_aa|+|16768|17628 >NVVL01000051.1 Rickettsiales bacterium isolate NORP64 Contig_source1382A_23642, whole genome shotgun sequence
MNQLQLLGAMEIGLIYALVAIGVYITFKIIDFPDLTVDGSFTLGAAVSSAMIFAGYDPYF
STLMAIIAGGLAGMLTGYLNVRWDILGLLAGILTMTALYSINLRIMGRPNIAMIDSETVF
GSWSIIVISLIIVIVAILALTRFFASEFGLAIRAVGINPKVSTSYGIRVGAMKVIALGLS
NAIVALAGSLFAQSQGFADISMGTGTIIVGLASVIIGEALVHPERVWVSLVTCTIGSILY
RIAIAFALNAHDIGLEASDLNLITALLVAVTMIATRWKKKNTVRVI

>gene_1111|GeneMark.hmm|260_aa|+|17595|18377 >NVVL01000051.1 Rickettsiales bacterium isolate NORP64 Contig_source1382A_23642, whole genome shotgun sequence
MEEKKYGQGNMIELKGIDVIFNKGTKLENHVLKSIDLKVETGEFITVIGGNGAGKSTLMN
VLSGNVAPASGQIFIDNLDVTRMPTEKRSSMVSRVFQDPMIGTFSDLTIEENMSIAIKRG
KCRGLAMSSNEPLREQFKAALADVGIGLEERLSDKVASLSGGQRQALSLIMATMIGSKIL
LLDEHTAALDPKIAKKIMQLTDKIVKRDNLTALMITHSMSQALEYGNRTIMMYHGSIARD
MHGADRSELSAADLVKYFDL

>gene_1112|GeneMark.hmm|500_aa|+|18389|19891 >NVVL01000051.1 Rickettsiales bacterium isolate NORP64 Contig_source1382A_23642, whole genome shotgun sequence
MTASTKDYDNLETELEQITHLQNLFKIAHWDSSTMLASGSAPSRQKEIATLLSVIHEKST
SDKISDLINGSLGEFEHLNDWQRSNLANAKKTYDMQVCVTSELQHEYNIASAESEFVWRE
ARLQNDFKKLEPYLDRVFDSVRNIASIQAKKLRRTPLDVLIDSYDPDRTSADVRSVFDVL
KAELPPLIERVMAKQESEKVIPLSETISEETQKAIGLRVMGAMGFDMNLGRLDKSVHPFC
IGSNDDLRITTRYDEKNFLSSLFGVIHETGHALYLQNLPAEYRSQPVGDYKGMAFHESQS
LIMENQVGTSRAFMEFLAKMLKDEFSFSGPEYSAENLYKLVTRVKPSFIRVEADEATYPL
HVIMRFEIEEAIINGNLKAMDLPDIWNSKMEEYLGIVPSSDREGCLQDVHWPSGMVGYFP
SYTNGALIASMLMKSAKEKYPEIDSELSTGNFTSLNRYLTDNLRKYGCSKNSADLLEAST
GHRSIAPGIFIDYLKGKYLQ

>gene_1113|GeneMark.hmm|110_aa|+|20337|20669 >NVVL01000051.1 Rickettsiales bacterium isolate NORP64 Contig_source1382A_23642, whole genome shotgun sequence
MYGVIKFQSPFEKLKKYPCSNEVKLYKATILQAVIDASNISSSVISRKNEIEAKHWIFGN
GAHFQEICYLADMEPSFIIKITRYVIKHQAIRKSQLFLSVLEGKVPKEDC

>gene_1114|GeneMark.hmm|154_aa|+|21064|21528 >NVVL01000051.1 Rickettsiales bacterium isolate NORP64 Contig_source1382A_23642, whole genome shotgun sequence
MNIFLSKYLNKIDKKGRVSVPAGYRSTLLKEDFNGVIIYPSFKNNCIEACSMSRLEELSK
IIQNLDPYSEERDAFETIVLGEAVQLPFDNEGRVILPAYLMEQVGISGQACFVGKGVVFE
IWHPPSFDKHLKSAREIAKGNKLILKNNQNNNVS

>gene_1115|GeneMark.hmm|331_aa|+|21532|22527 >NVVL01000051.1 Rickettsiales bacterium isolate NORP64 Contig_source1382A_23642, whole genome shotgun sequence

MNKKTDLQQQATSDSSSKNENISPHCSVLLAEVLEYMQIKDGGHYLDCTFGAGGYTKAML
AQAECCVTGLDQDGNVEKFADQVNEKFGNRFKFVQTNFAEAKAKLGGQKFDGIVLDLGVS
SMQIDSAERGFSFMRDGHLDMRMGSAGIDAAEFIESASEEEIANVIYKYGEEVQSRQIAK
KIVECRATEPIDTTLKLAQIIRDAMHYRKGKIDPATKSFQAIRIYINKELESLEKFLDQL
EDMLVPGGRILVVSFHSLEDSIVKAFFKEYSAPKVARSKYAKTPEIIEDGKWLKVITRKS
VTPGREEVLRNPRSRSSRLRVAEKIGGADVT

>gene_1116|GeneMark.hmm|125_aa|+|22517|22894 >NVVL01000051.1 Rickettsiales bacterium isolate NORP64 Contig_source1382A_23642, whole genome shotgun sequence
MLLNRVPVYIILILCLITVCTLFSIKSNVIIIRAELSEVNKQLQSERDTIHVLKAELAYL
SSPERLQKLNNVYLKLKDTKVSQMISDPLKQAEPVYIASNAPKARVRWNYKRGASKYLIR
VSSKK

>gene_1117|GeneMark.hmm|616_aa|+|22938|24788 >NVVL01000051.1 Rickettsiales bacterium isolate NORP64 Contig_source1382A_23642, whole genome shotgun sequence
MIFSLIKKLRKLRIIVASWAMFCVLYPRIILLKLRRAALFRIKKLSNSLHEFKWRKLYRS
LYIWDLSYSQSRMRMHIVIGVFAFVFFVISARLIMVATSEYTSYRRANEVASGSRLDIVD
RNNNLLAVNLPGASLYANPRKIIDQLDAANKLSRLIPELKKKELLRLFKKNKSFVWIKRD
ISPGEHEKIYNLGIAGLGFEREQKRIYPYGNLLSHVVGYVGRDLNGLAGLEKYFEKFLTG
QKEKEDRRKLGSSLKLSIDVRVQNILSAEIDKGIKKFSAKGASGIIVDPNNGEVIAMVSK
PDFNPHHPGDARPKQLFNTVTQGVYEMGSGMKALTVAIGLDSGTTSMHDVYDLSYMKVGG
KVIKDTHQMKGWHSVPHIFMKSSNIGVTQIMLEIGREKLSDYFRKIGFLDRLQIELPERA
RPLFQPFSRWSDLSLTTMSYGYGFSESPAHFMQAMIPVMNGGIIYPLTLLRRDEGEVLTG
ERVFKESTSNSMRQLMRLTVARGTGKKAEVKGYYVGGKTGTAYIAHAGKYDKTRRISSFF
GVMPASSPKYMIYVVVYNEPKGIKETFGWAGGGWTGAPTVGAVLSRMAALYGLSKLDKNSK
EVQELNNVEYKIENET

>gene_1118|GeneMark.hmm|508_aa|+|24778|26304 >NVVL01000051.1 Rickettsiales bacterium isolate NORP64 Contig_source1382A_23642, whole genome shotgun sequence
MKLKVQLQKYFSDKKIKRISFDSRLVKPGDAFFAIKGECFNGNDYIDNALALGASVVFTD
DESQKDNKIVHIEDARRGLAMAAGMLYSQMPENIIAVTGTNGKSSVVSYVHQIFTMLGKS
SASMGTLGVESTEELSKNAKEISSGMTSSDPLSFRKTLSLLARAGVSYLALEASSHGLHQ
FRLGEVKANGAAFTSFSQDHLEYHHTMEEYLLAKMMLFTEHLADGCEAVINSEMESAEFI
GKFLDERDIPYSSVGANGDLQIKNCQQSIGGQRVNFSFLGEDYSFSTEIIGSFQATNILI
AAKLVYNMGIEFGLIAPLLSKIKPVCGRLQRITEKNAEYQVFVDYAHTPDALEKSLIELE
KLKQGQGQLYVVFGCGGDRDSVKRPIMGKIATDIADYVVITDDNPRDENAEKIRGEILKG
LNYREAGNKEPGNKEETSKKFGNKEADKLKEIADRKLAINETVAKLKQNDILLIAGKGHE
DYQIIGDIVIDFSDIDVAFAAIRNRNMV

>gene_1119|GeneMark.hmm|461_aa|+|26310|27695 >NVVL01000051.1 Rickettsiales bacterium isolate NORP64 Contig_source1382A_23642, whole genome shotgun sequence
MTCWNSKQLEEALGIELSFKAKYNGIKFNSQDVKKGDLFIALKGTRDGHQFVDDAFSRGA
VAAIVSKISPGVDLDKIIQVEDTLAALHMLASYKRAHSKAKFIAITGSEGKTSTKEAIRM
MLTPYGKTYANYGTFNNHLGVSLTLASMPDDAEYVIVEMGMNARSEILELSKLAKPDIAV
ITSISEGHIGFFESLQDIVDAKCEIFESLDKEHGVGILNHDIPTYENCINNVKKIGLKNI
QTFGKSEEAVTRFVSYEELDSDSVRLKYMIADDEFEVAMHMLPMHLAENFAAAFAVVRAL
GLDPEAASDAISSFQPIIGRGKLVAVKDKKKSYRIICDYYNANPNSLKASLKYLEQIASD

SKIAVLSDMLELGAKEQELHESIVPYIIESGAGKVFLVGPAMARIKDKIPSSIEVRCYKD
TQHLIAEIDQHLHSKELILIKGSRSFALEEVAKHLGVKDAL

>gene_1120|GeneMark.hmm|362_aa|+|27685|28773 >NVVL01000051.1 Rickettsiales bacterium isolate NORP64 Contig_source1382A_23642, whole genome shotgun sequence
MLYNILASYAGTCDIANLFAYISSRSILATLLSASICFMIAPSLISYLRKVQKHGQPIRK
DGPETHFSKAGTPTMGGVMVLFSATISTLLLADLYNPYIWVCLFVMLSFGALGFMDDYTK
LSRNNHHGVSGRVKLAIQMIVSMIAFIVVQRYTEPEYATQLTFPFFKSLFLDLGYFYLPF
AMFVIVGSSNAMNLTDGLDGLAIGTGAIAISSFALISYLVGSSVYAESLQIIHIPEAGEL
AVLCGAIIGSSIGFLWYNSYPAQIFMGDTGSLALGGFLGIISVITKHELALSIIGGVLVI
ETLSVIIQVYYFKMSGGKRVFLMAPLHHHFEKKGWPESKVVIRFWILALICALVGLSSLK
LG

>gene_1121|GeneMark.hmm|225_aa|+|28777|29454 >NVVL01000051.1 Rickettsiales bacterium isolate NORP64 Contig_source1382A_23642, whole genome shotgun sequence
MLGDNPKRPPVSGDGNSLFVKKIFKTLQGEGVHAGVPAIFIRLGGCNLACSFCDTEFEDY
HSLDLSKILAEISSLRLNDEGRSVIDLVVITGGEPFRQQISPLCQGLLALGVKVQIETNG
TIYREIPEDAEIICSPKVAGGKYLQVRREMLPRVTAFKFLISCAAGGYALVPELGQREYD
IPVFVQPMDEYNDEVNLRNKQLAIDIAINNGYRLSYQLHKVLGIE

>gene_1122|GeneMark.hmm|228_aa|+|29564|30250 >NVVL01000051.1 Rickettsiales bacterium isolate NORP64 Contig_source1382A_23642, whole genome shotgun sequence
MKKAVILVSGGLDSSTVLAIVKNAGFEIYAISFNYGQNHLIEIEKIKQFIKNYDVAEHKI
VNLDLSAFSTSALINKSVDVPKHEKVGDIGSDVPVTYVPARNTIFMSYALGYAEVIGAQD
IFIGAHLTDRENYPDCRVEYLQSFEAMANLATKAGTEGAKISIHAPLIDMEKTEVVATGL
KLGVDYSGTISCYDPSPAAESCGACNACLIRLEAFRGNNKQDPIIYVR

>gene_1123|GeneMark.hmm|184_aa|+|30254|30808 >NVVL01000051.1 Rickettsiales bacterium isolate NORP64 Contig_source1382A_23642, whole genome shotgun sequence
VNIKYFFGQFPVLDLGSIVLRRMTEDDASHYMAYMNCPEMHDVLTKDNMPASLAKAKEEM
KYWSGLFDSKRSLYWAIAWKETDQIIGSIGFNHISFPHSRAEISYDLDPEYWGKGLMLKA
INGVLKFADGGLELVRIQGTVLISNERSIKVLDRCGFKREGVLKKYEIVAGEYRDYYMYA
RVVE

>gene_1124|GeneMark.hmm|163_aa|-|30940|31431 >NVVL01000051.1 Rickettsiales bacterium isolate NORP64 Contig_source1382A_23642, whole genome shotgun sequence
LSIQKKQLKQKALKGDLEMNNNTNPFDQMKSFMNNDNLNQTMQNMPNIDFSAMTDMMKNN
NDTATSSAQVATDGIQSILKKNSESLQKDTNDMCSSMKDAISAGDVSQITLCQQKFMKSA
IDNHVKNTKDILDISAKSILDSMNLFHNVVNDNVNKVSAKAKK

>gene_1125|GeneMark.hmm|44_aa|-|31535|31669 >NVVL01000051.1 Rickettsiales bacterium isolate NORP64 Contig_source1382A_23642, whole genome shotgun sequence
LDQKPQIIELFYYYKMKNLSINQTTTIVLESNANTELKSNTNAD

>gene_1126|GeneMark.hmm|132_aa|+|31782|32180 >NVVL01000051.1 Rickettsiales bacterium isolate NORP64 Contig_source1382A_23642, whole genome shotgun sequence
MDKIISVCIEIACSHCGNMLCYEYVDKTLIDTRIMRIIAIKRLKEFWESGYADSEQPLKV

WYQIFRQGSFDNPNKIKSLFSSCSFVGNNRVVFNISGNKYRLVIHIRYDLQIVYIRFIGT
HAQYDKINVGEV

>gene_1127|GeneMark.hmm|123_aa|+|32183|32554 >NVVL01000051.1 Rickettsiales bacterium isolate NORP64 Contig_source1382A_23642, whole genome shotgun sequence
MELHTIKTEKDYEKALNSIDLLLDSPAGSEQAEQLELLSLLVEDYENKHYKIDVPDPISA
IKFRAEQLGLTRKDLEKSIGSRGRVSEILNRKRSLTLVMIRKLHKNLNIPAEILISDIKR
KSA

>gene_1128|GeneMark.hmm|70_aa|-|32887|33099 >NVVL01000051.1 Rickettsiales bacterium isolate NORP64 Contig_source1382A_23642, whole genome shotgun sequence
MTQENIIKILAGMNSAARKAGEAVKESSSQYIEDNVIKGNYVSREEYNQLHKLVIKLSKE
VAELKSYKAT

>gene_1129|GeneMark.hmm|104_aa|-|33109|33423 >NVVL01000051.1 Rickettsiales bacterium isolate NORP64 Contig_source1382A_23642, whole genome shotgun sequence
MESEYSMIYLNFKILNELMSRETTIKNKLAILRPHILEVIDESTQHQGHSGNPDGAGESH
FSVKIASIELDDLTRIEKHRVINELLSREFASGLHALSIEIIPS

>gene_1130|GeneMark.hmm|408_aa|+|33493|34719 >NVVL01000051.1 Rickettsiales bacterium isolate NORP64 Contig_source1382A_23642, whole genome shotgun sequence
MAYAEKVAKILQREIKEAQSGILLQVLLLNMDEQNILIEDTTPLIAKFYDRISQACKQVP
DHKIVKTTDPAKVYIVLGKNTKDIKKIAHSIYAHMQEYTDSDFPETYLRCSVGSIKFRQT
SEDSNGKVIEFSASKLLSLLEYSMQRSGHPPYHCYEDHPVDIEKIRNGNINLNQLKKSIS
EGRVKFMYQPVIDRISGDIEYYECLLRAPDIHNKFVSAGEVIADAESKGLINIIDFAVIE
MVIGELSRDKDVTLSVNISNMGVLDKNLLKRIEELLQKHDVANRLIIEITETSLNRDFAN
TQKFINVLHGYGCLFAIDDFGSGFTSFKQMLGLSIDIIKIDGSYIRDMLVNNHSKAFVKA
LVGLAGELGIKTVAEFVENNKVAQLLVELKIDAMQGNFFAEAMKNRIS

>gene_1131|GeneMark.hmm|445_aa|+|34720|36057 >NVVL01000051.1 Rickettsiales bacterium isolate NORP64 Contig_source1382A_23642, whole genome shotgun sequence
MSFTTYLDALNSKIEDIKPVKLSGRVVSVKGLVIEARGISGFVSIGSRCRIKNNLQDSYI
LCEVVGFDVDSVLLMPFEDTEGVASGAEIEIDQHENAIYPDDKWLGRVINAFAEPIDELG
PLKSGQKAYPLKTSPPPPHKRQRIGKKIDLGVKAIDSFVSCCYGQRMGIFAGSGVGKSVL
ISMLTKYADTDVKVIGLIGERGREAKEFIEEYLGEEGLKKAVVILATGNESALLRKRAAY
VTMAIAEYFRDQGKEVLCIMDSVTRFAMAQREIGLAVGEPPTTKGYTPSVFSELPKLLER
AGPGLESANITGLFTVLVEGDDHNEPISDAVRGILDGHIVLDRAIAERGRFPAVDVLRSV
SRTLPRCNNDLENKQITYARRMLATYHDMAEMIRLGAYKQGTDREVDLSIGYFEQIEEFL
NQKPDESCSMEESYKRLGAILNITE

>gene_1132|GeneMark.hmm|147_aa|+|36095|36538 >NVVL01000051.1 Rickettsiales bacterium isolate NORP64 Contig_source1382A_23642, whole genome shotgun sequence
MGLEKSNKSLKPLKTLVKLNKNKMDTLLKEIKYRDSEKDRLEKKKQQIEDESQAEIARYS
GTKYAYMLDNYMQNARKSIKIVDAHIEQVVQILEKLREVLETQYSELKKFEIILEMKIKQ
QQEQEKIAETKAMDEFNSNKFIYEKEG

>gene_1133|GeneMark.hmm|131_aa|+|1|396 >NVVL01000052.1 Rickettsiales bacterium isolate NORP64 Contig_source1382A_23653, whole genome shotgun sequence
DRVEKAFLERKKMQYLYAEEHHLVFMDPESFEQLLLDKGLMGDKLPLLADGMNVDVEFYQ
EEPLSIKLPATIIVDVEMTDPVIKGSTVTSSYKPAILTNGLRVMVPPYLVSGEKIVIKTE
DLTFVERAKKC

>gene_1134|GeneMark.hmm|247_aa|+|390|1133 >NVVL01000052.1 Rickettsiales bacterium isolate NORP64 Contig_source1382A_23653, whole genome shotgun sequence
MLGDTGLVVDAMRKASRFLQRDYFELEHLQASGSKTSSFCQKACTKVMHTLHDELKRYYQ
TIIFSSDEVKSTNFSGKAALVETLDGLGNLERALPFFAIMVTIISNKDGEIVADKAIINL
PALNEIYYAEKGRGAWVERHSSNISGASRVRVSGAQSEQDAVVSGGREQMGKAFKSFKNV
RFFDSYTYALTLLVSGKIDALMLETRMISLAGIQLLLLEAGGSTHEKDGLLIASNFKLHD
KIKQLLL

>gene_1135|GeneMark.hmm|187_aa|+|1191|1754 >NVVL01000052.1 Rickettsiales bacterium isolate NORP64 Contig_source1382A_23653, whole genome shotgun sequence
MISAKDNSAEHMKKFTINCDFNGQMAPFAIYIGTPEKVHHPLHFQADWLMKVRGGTIPAD
VMDAVTQLNELAKKNSVPLEELCVYALGSESGNVLKSELEPAEASLPKKPSPELKPDAEA
LNKPKSQQEPQAKEQSESKEPPPDKAPTPEKHETNTHNQRNASEDAEDMSSEGGHVEDDS
SDSGKLK

>gene_1136|GeneMark.hmm|391_aa|+|1946|3121 >NVVL01000052.1 Rickettsiales bacterium isolate NORP64 Contig_source1382A_23653, whole genome shotgun sequence
MSRNLAVSRGVLREQRIAKVQLLIDAIRGVDIDKVTEILRQDARIINGVGSEGHTPLVLA
IMVGSSIELINTLINHGANPTLIVGGMIPLVCAIEKGNIELINTLIYHGADPTLVVGSFT
PLLYAIEVDSSIELIKTLMDNGAKPNQVVDIFTPLRCAIGKGSIELITMLIDHEANINLV
TGSFTPLSYAIEVDSSIELIKTLMDNGANPNQVVDIFTPLGCAIRKGSIELIIMLIDHGA
NPNLVAGSFTPLLHAIGVDSCIELITTLLDRGANPNQVVGSFTPLLQVAVGEGDNIELVK
GLLAYDANVFLTISFSLFGLLDPSKPVVKSLLCHASYLQLEAVTALYPAVLEDLLKSVLL
STLPEGASSNDIALIEPPLAGDAGAEEGTDF

>gene_1137|GeneMark.hmm|609_aa|+|3209|5038 >NVVL01000052.1 Rickettsiales bacterium isolate NORP64 Contig_source1382A_23653, whole genome shotgun sequence
MKPVHANSLTVLREIYGYTEFRGDQGEIINHIIDGNNTFVLMPTGGGKSLCYQIPSMCRP
GVGIVISPLIALMRDQVSALSQLGVSAAAINSTQSYAEISATIEKIENNQLDLLYIAPER
LLSEEFMGIIKDAKIALFAIDEAHCVSQWGHDFRPHYTQLEILAEKFPSVPRIALTATAD
APTRHDIIERLRLTNCRSFVAGFDRPNISYSITPKNNPKKQLLDFINGRRRENSGIIYCL
SRKMCEEVAKFLVDNGYDALVYHAGMQSNQRAQNQDRFLKGEGVIMVATIAFGMGIDKPD
VRFVVHMNMPKNIECYYQETGRAGRDGLASDALMFYGMQDSAMQRSFIENGDAPDNQKRI
EHQKLNALLGLCEASRCRRQILLEYFGDSADACGNCDICLNPPKTFDGTIAAQKAISCVY
RTDQRFGVVYLIDILLGKINDRMVNFRHDKISVFGIGTEHSKQEWQSIFRQLVASNLLKV
DIVGHGSILITEHGRKFLKNKETIALCEYKTQPKAKKASTRAASKAVDVIFTNDDQRELY
QSLKVKRLELAQQHNVPPYVIFQDKALKEMTMTKPSSHDEFKQINGVGASKLEKYADIFI
EVINIHDSE

>gene_1138|GeneMark.hmm|88_aa|-|5406|5672 >NVVL01000052.1 Rickettsiales bacterium isolate NORP64 Contig_source1382A_23653, whole genome shotgun sequence

MANTSLTLGIHWEKFIKNEIVGGRYASASEVVRCALRTLEEKAVNTHLELLRHALIQGEL
SGDAGELNMQTIRREAKSELSPNLSNDA

>gene_1139|GeneMark.hmm|955_aa|+|6027|8894 >NVVL01000052.1 Rickettsiales bacterium isolate NORP64 Contig_source1382A_23653, whole genome shotgun sequence
MFFGIDIDMAIVVTFLVATLIVGLKYSGNINTIKDYALGGRNFSTAALVATITATWASGS
GFFTTLSRTYTDGFFYLFATLFMGVQLLLVAFVFVPRMGEFLGSISVADAMGKLYGKEAR
VITAISGAVGSVGFIAVQFKAFGNIVSYFMGIPSEVSIITSGIIVTIYSSLGGIKAVTIT
DMLQIFAFCFTLPLVGIMIWNQLYFTDFSLTNAFQASKFDVGYVLDVGKPKFWEMILLML
YFMMPNMIPINFQRISIAKNLEQVKKAWAISAGLLIFLKLSIAWIPFLIFNIDENIPANQ
LLGYIIDNYTYTGLKGLIIVGVIAMAMSTADSLMNTGAVLFAHDICGVLKIWQRKELILA
KIFSFSIGGGAIMLALTELDFLGIILVAQSFYMPLVTVPLLFSILGFRSSSKSVLIGMGA
GFCTVVLWKISGIKADPIIFGMIANVVFLFGSHYLLGEKGGWVGIKDKEYLDKQKRIRQK
NKGAFLRWVQEFNFFNLCRKYSPKDDLTYTGFGVYSIICTITTMYSTQVELLAPNGQIVL
AIYQIMMVTGTMMAMYPVWPLSIKKIIKERVAQTWWNPAIFYMLAFFSTFFVLISEFGQL
QFVIFTVNTILLILLAGWRLASLMIITGFYGGIEFFKYYKGIDDIDMAIGSPLFVFMYSL
ILIGALLVIFLKPKQEHLEQTEDKVEDLESEVTTQGTKITTLNKEVGHYSQRVSNQAREI
ERLGATAQKILNNVNHELRLPVGNVMNFAEMLRDGLDKFSDDQLKMISEEVYNNSNRLSS
MILNMLDLAVLEAKKIELKKEEVNFSKLVRARAQNCRDIYAQDKKALEFRLNIEDDVMVF
ADANYICQMIDNLVINAINFSEKGIIAINVDREKYFVSFAIRDEGKGIPKDEIFDIFTPF
KMGSNMESSAEGRGVGLALCKSVIEAHGGEISAESRNVGALFRVCLPYGEVKKEK

>gene_1140|GeneMark.hmm|153_aa|+|9085|9546 >NVVL01000052.1 Rickettsiales bacterium isolate NORP64 Contig_source1382A_23653, whole genome shotgun sequence
MHKQELATIIDYLQPLLTKEDIDLYVENKRDSSSIEEYKYHATDFIATKLIEIEMPAFEG
WNSLYPILPAFNSKDLFVKSMVQRHIMKYFTPLAEILYDLQSSENLSIVNLKFKQELLNA
EENFHKANPEAQNEQIYYDKLCHMLEDAPKLDK

>gene_1141|GeneMark.hmm|49_aa|-|9668|9817 >NVVL01000052.1 Rickettsiales bacterium isolate NORP64 Contig_source1382A_23653, whole genome shotgun sequence
MAVLIISLAVLFVFFDKIIEHYAPNSDFTTFSNEIDTVILALDEQDDAR

>gene_1142|GeneMark.hmm|331_aa|-|10074|11069 >NVVL01000052.1 Rickettsiales bacterium isolate NORP64 Contig_source1382A_23653, whole genome shotgun sequence
MAKEPTKLDVDPYLDLESEISRLKKEMNAVILAHFYQDPDIQELADFVGDSLDLSKKAAE
TDADVIVFCGVKFMAEAALILNPSKKVLIPDLKAGCSLEDSCPAPEFKKFRDKHPNHISV
VYINCSAEVKALSDIIVTSSSAEKIINSIPEDKEILFAPDKHLGNYLIKKTGRDMTLWDG
TCVVHENFSERELVRLKTTHPEARIIAHPECPAALLSYADHIGSTSSLLRDVGAHREMDF
IVLTEPGIIHQMHKIAPSSKFYDVPSLDQAGCTSCSQCPYMRLNSLEKIYACMVNGAPQI
RLDPEVSKKAKIALDRMIAGKYTPLEALQEG

>gene_1143|GeneMark.hmm|957_aa|-|11189|14062 >NVVL01000052.1 Rickettsiales bacterium isolate NORP64 Contig_source1382A_23653, whole genome shotgun sequence
MSDLEPEDTLPLSKNILPISIEEEMKNSYMDYAMSVIVSRAIPDVRDGLKPVHRRILYSM
HEGGYVSSRPHRKSARIVGDVIGKYHPHGDSAVYDSLVRMAQDFSLRVPLVDGQGNFGSM
DGDSAAAMRYTESRLTKISHTLLEDIDKETVDFSPNYDGSESEPGVLPAMYPNLLVNGTG
GIAVGMATNIPPHNLGEVIDAACAYVDNNDIELTELISYIKGPDFPTGGTILGTSGIQSA

YLTGRGSIAFRGKCSIEDHNGREVIVVNEMPYMVNKAKLVEKIAELVREKRIEGISDLRD
ESNKDGVRVVVEVKRDAVADVVLNQIYSYTQLQTSFGVIMLALDDGMPKVMNLKEVIGAF
VRFREVVITKRTIFLLNKARDKAHILLGVRIAVSNIDEVIRIIRAAPNPVVAREQLMEKA
WDCSTITSLIKLVDDRTDISDDGTVHLSETQAKAILEMRLQRLTAMEKDKLEKDLGELTK
EITDFIDILSSRERLLSILKSELIRVRDEFATPRKTEIIEGNFDKDIEDLIQKEDMVVTV
THSGYIKRVPLVTYRAQRRGGKGRSGLSMRDEDITTQLFIGNTHTPLLFFSNLGQVYSLK
LYKLPLGNPQSKGRPIVNILPLQSGETIANVMPMPESQEEWDNMHIMFATAKGNIRRNDL
SDFKKIQANGKIAIRMDEDDSLVNVMACNEDEHVLIASKLGKAIRFPVNAIRVFKSRTSD
GVRAMRLADKDRVISMTILHGIKSSMEEREAYLSIPYEKRLAIAAGDHEFSAQELDVALT
KEQIIDMADAEEFILTVSENGFGKRSSAYLYRITNRGGSGIANMVLSEKTGEVVASMPAN
MNDELMLITNNGKLIRCKLDSVRITGRSTSGVILFKTDKGEQVVSASLIADSSDEEEDES
TADESTADESAADESAAGESGAGESGAGESADKSGTGDTNESDGPESDGSEANGSEE

>gene_1144|GeneMark.hmm|72_aa|-|14242|14460 >NVVL01000052.1 Rickettsiales bacterium isolate NORP64 Contig_source1382A_23653, whole genome shotgun sequence
MLSVRLGQSLENRLNVLSKKTHRPKSFYVKEALEKYISELEDTFIALNRSLSPNRKFYSS
KEVLNILQNETP

>gene_1145|GeneMark.hmm|676_aa|-|14460|16490 >NVVL01000052.1 Rickettsiales bacterium isolate NORP64 Contig_source1382A_23653, whole genome shotgun sequence
MRKLAAIWKTQKLSIIILLFSLSSCGDSTEIPSIEAIFAGGKSDEISSASSAEMAPIGGG
AIEGGATGETSIGSTSTETTSTAEQSAKIESRTTSTIVPPALLPLTGRGTTGLTENQSKK
IRDYIVKQTSSNRPIKLKKKFGTNLRGLKLTGVNLSGTDLSGTDLSGIDFRDVEFARVNL
SGAKLDNVILTGASMQECDFSGVNFTGLSAKGVDFHYSIFRGAVFNKVNLSKALLNEADL
TNAKFLNCQLDGVDLSKAVMKNVVIDNTNLQKANLSNANLSNAKITNSAFFGVDFRYVDL
FLATIESCSFESSDLSFIKARKTSFTNTDFTQTILEDISFTDSTKFDSSIFDYSSIKNSV
FDNVTINNCRFNDVSMAYTSFQDVDIQHTEINNVKFSSAKMSRVHMNNVKGRDNYFNKIT
MDGVKITDSDIRNSVFSNDNGKKISFLNVNLTNTLFDNYNLTDISFKKTSLSGTVFRNSK
ISKVSFLHSKLEKIAMVNCTLEEVTLNNSRVQRSLFNGVTAIAMLVVNSPSTRNIMINNN
GLEKIAGSQEITSTAQIAKIKNLSNMNMAYIDLSSMSIRQVDFSNSILLGANLAKASLQN
SLFVNVDFRYSNLSGTDFSNSTIANSSFLGAKLDNTILKNTKLKNLDFTRTFTRDVNFDN
AEMDNVFGMDAIEAIE

>gene_1146|GeneMark.hmm|475_aa|-|16504|17931 >NVVL01000052.1 Rickettsiales bacterium isolate NORP64 Contig_source1382A_23653, whole genome shotgun sequence
MALDILIIDDEADIRDLISDILKDEGFTSRVAANSTQAFNAISDKKPRAIILDIWLQGSD
LDGLGILEIVKKQYPLMPIIVISGHGTIETAVNAIKMGAYDYLEKPFTHDKLMIVLKRAC
ESAKLKRENIDLKSKVIDKTEFIGNTAITSKLKMEIEKVSPTSGRVMIHGAVGSGKELTA
RLMHKRSKRASGPFFIFNPTSMNPTKIHQELFGSDEKRNNQPLFDKRLSVLEAANNGTLY
IDEVGDLPLGSQNKLLKFLKDQTLMKPDGQSVSLDVRIITSTTKDMKKEIANGRFGQDLY
YRLNVIPLTIPQLAERKEDIPMLVEYFVKQLSKFSGLNEREFSEGAIAALQAYDWPGNVR
QLRNIVEWTLIMNPLTQNSTGMIKSEMLPQDIISGSVNISKPNSNVDMMSMPLREAREVF
ERQYLTAQMNRFNNNISKTSTFVGMERSALHRKLKTLNIHASSKPEIDEFAELEA

>gene_1147|GeneMark.hmm|645_aa|-|18174|20111 >NVVL01000052.1 Rickettsiales bacterium isolate NORP64 Contig_source1382A_23653, whole genome shotgun sequence
LLESLYANTNIYMLTQIWKNLSSYKLSNNAIYALISVISTSLVITLFTYADFFFNNPDPL

YILGFLLATLVLILCFSIIVVNNRVSSTFSFRKGGKKRVSKLRKRIIIAFSIGATLPTII
IAVFSTYFFNFGIEAWFSQKVSNVLDQSIIVGESYIEEHVMQLRETAISVSNDLSEMCYD
LIRDPESFSKVINAQAEMRSLDEAMVFQKDTQTILAQTKLSFALSFTTIPVHLITRADKG
GIVPIPAGSTKIRALIKLRGFNDAYLLIGRFVDSKIIDHIDKTNGAAEEYFRVKRQISEL
QIKFSMVFIIVSLILLVAATIWGRAFAERIVKPIRELVIAAERVKNGDLSTQVPMEGLKK
DEIKVLSAAFNQMVNQINRQQKDLVIAQRALAWSDVARRVAHEIKNPLTPIQLSADRLLK
KFAGEVSDPESFTKYVKNIIRHSNDIKSIVSEFVDFARLPTPSFAKCKIVSMISNLVESR
KLINDLISYSFVSDTEEFELVCDISQINRVMVNLLQNAEEAFGEQEKALGERTSGPKEQT
FQLEGQTSKSEEQKTDMQISVTLSASEDQFLIIIDDNGAGFSDKLLTSAKEAYVTTKSNG
TGLGLAIVDRIVIDHFGEVALSNNTHGGARITLTFSVKELTLKLK

>gene_1148|GeneMark.hmm|90_aa|-|20135|20407 >NVVL01000052.1 Rickettsiales bacterium isolate NORP64 Contig_source1382A_23653, whole genome shotgun sequence
MQNNIKNNDKTLTSAIEARMDEFFAMHEGDCPESGLYERVITQVEKILIKKTIDHVSGVQ
TKASKILGINRNTLRKKVKDLGISCPTPRK

>gene_1149|GeneMark.hmm|317_aa|-|20407|21360 >NVVL01000052.1 Rickettsiales bacterium isolate NORP64 Contig_source1382A_23653, whole genome shotgun sequence
LANPVILAPMSGVTDLPYRKLVKKFGAGLVVSEMVASRAMIARSDKSLKKCAIDNDDNDA
TKACVQLAGCDPDIIAESAKMNEDMGAKIIDLNFGCPAKKIVGGFAGSALMRDEELATKI
LYATVKAVNIPVTLKMRMGWDDNSKNAPVLAKIAEDAGVKMITIHGRTRCQFYKGSADWE
FISKVKEAVKIPVIANGDIVCIDSAVEALKQSNADGIMIGRGCYGKPWLISQIAHFLATG
EKLQDPSLEAQLGVVLDHYDAMVEHYGADTGVKMARKHLGWYSSGLKNSSEFRASINQLV
KIADVKDRINEFYGSLI

>gene_1150|GeneMark.hmm|57_aa|-|21363|21536 >NVVL01000052.1 Rickettsiales bacterium isolate NORP64 Contig_source1382A_23653, whole genome shotgun sequence
VQAAKVRKSPCAVKKSPGTGRAYLNLLNFDKISSKKYATMRVLKIQKHKKCKFESII

>gene_1151|GeneMark.hmm|158_aa|+|21740|22216 >NVVL01000052.1 Rickettsiales bacterium isolate NORP64 Contig_source1382A_23653, whole genome shotgun sequence
MQKTDTQKANTQKVVLPEGYKPSENEEYMNPMQIEYFKQKLLAWRDDLLDDSRETLDHLK
TENWNEPDLNDRASVETETTIELKTRSRYCKLISKIESTIERIEKGNYGYCEETGEEIGV
KRLEARPVATLCIEAQMRHENYEKTHIDEDNVDVHTAI

>gene_1152|GeneMark.hmm|1117_aa|+|22624|25977 >NVVL01000052.1 Rickettsiales bacterium isolate NORP64 Contig_source1382A_23653, whole genome shotgun sequence
MKLKNCISILGVSALSSSVALGGVSIVGGTAYVTGSYSGTIVDPTIYNSLNVDIDNTSSP
ILNNDVTGGDVDVRGSSSPIFQETVNGDYVNIRDDSTPTFEKSVTTSSPYFVAVRDNSSP
IFKGDVYSNHDLSINGTSTPNFMAKTESGRYVYVFGNATAAFTGDVTANYGLSLRENATG
TFDGKVSSNYYLFIFDNSKSTFNGEVTTGDYISISGAAEPEFKNTLTSTGSYIKIADSAK
PIFHDDVSVKGDVTISGTSESEFKKSITSTNGVILVQDDAKVIFYDVTSDGDITASGNSI
TEFNKATTGANSSLIVKDAAKATFHDDVVSDSNITISGTSESEFEKKLTSTSGTMDIQND
TKVTFHGDVLASGNVTISGTSESEFKQNLTSTAGSIFVQDDAKPIFNGSPSTTIAKGNSK
PIFNAPASNVEIRENSEAIFNDPTGDIYISDTPTVTFKGTSSSKITLLHSASPNTPILKL
EPGFTSSGDLIIDSIININLSEPTISGTVKLNKISMEADTKIKLDNDVTMDTPISTSVDK
TGSLEFLGDAKILQDIGSKTNWLKNVTFDTNPADISLEGNIYANSVSFSGSNISFVNDRS

ITTGQSSLTYHDSSLDLDTKKLTVSGKTLFTGKTTFKTTFNVDGGGKETGGHIVVDGANS
SFNFSGATPVEIILTADSPLSPTDTYSYKLLIEENGGTIIPANNITFTPNENNSLITWTY
DEATHTLNAKNNPAQLPVIVAKTGGSEDDVKVAATLASANSSNICDATNVVMSNIGRLKE
AQMLEAVQRVQTTPGLGARATEISSAILNELEARITSLVTGNQFADAGVAAGDSNNKYGV
WANGFYGRGYQGRLPHSPKYNSRLYGLTIGADAPINDDVIYGLSMTSVRSTTKFSDKKAN
DVVKVKSLVFSFYAHRDLGRNWFSDVIFSSGVNRVKGMEKRVSFNNPNQLAVSKYKASTY
SGQLMLGYNHHTKDNVIITPFGSLGYSWVDKIKYTETGAGAQNLSVQSGSSYVLEASFGG
DVAFQTLIKGVPVTPEIHAYISYNFKVKRPTVIAKLMGACTPIPARVAKGNKLFYNIGGE
INAISESGSIEYGLNYEIKLSKKYINQVGGLKLRVNF

>gene_1153|GeneMark.hmm|665_aa|-|25989|27986 >NVVL01000052.1 Rickettsiales bacterium isolate NORP64 Contig_source1382A_23653, whole genome shotgun sequence
MTKPLFEKVLVAGRGEIALRIMKTLRKMGIKSVAVYSEADTNSMHVQYADEAYYIGNSPA
TESYLSISNIIGAIRVSGAQAVHPGYGFLSENPQFANILKRNGIILIGPSAQVIKKMGDK
IEAKKVAIDSGVSTVPGYMGIINSLDQAIEIATEIGFPIIIKAAAGGGGRGMRVVESTDG
IQKAYESAKFEAENCFSDSRVFIEKLIKEPRHIEIQLLADQFGNAICLGERECSIQRYHQ
KVIEEAPSSFVDEETRQKMYNEVLALAKKVGYFSAGTVEFMMDKEKNFYFLEMNTRLQVE
HPVTELITGIDIVEQMIRIAAGEKLAYSQEDIKLTGWAIESRICAEDPSRGFLPSSGRIL
EYQEPAKTSHVRVDSGIGAGGEVSMFYDAMIAKLCTHADSRKQAIEVMKNSLSSFIIKGI
SHNISFLEAIISHQRFVDGNIHTGFISEEYPDGFSGANLTSEMSEVFLASTIFAYLTEQK
RAATLSNQMTDQSNKLATRWIVNINDEQFSVIIKQVEDGYNIRHGSNRLTIRSNWNIGSP
LITATINGRKANIKVERIITGYRLTHAGVTFDTYVRSPRMSELESLMPVRDSTDDQKELL
APLSGQIIALSVAVGDEVVPGQEILTLTAMKMENIITAERAGKIAKISVSELEQVNSGQI
LVEFE

>gene_1154|GeneMark.hmm|514_aa|-|28005|29549 >NVVL01000052.1 Rickettsiales bacterium isolate NORP64 Contig_source1382A_23653, whole genome shotgun sequence
MGKQSFWREKELEKNQSAARLGGGAKRMLTQHSKGKLTARERLEILLDPDSFEETGMFVE
HRCDNFGMKDKKSFGDGVVTGHGTINGRLVFVYSQDFTVLGGSLGEHHAKKICSLINSAI
EVGAPVIGINDSGGARIQEGVDSLAGYGELFHLNVKASGVIPQITLVMGPCAGGAVYSPA
LTDFIFMVKGSSYMFVTGPDVVKTVTGEEVSKEELGGAKVHTSKSGVADLAFKNDIELLL
ETRRFFNFLPLSNRTELPTRSTRDPADRVDMSLNTLVPSTPNKPYDMKELIERIVDEGEF
FEIQPDYAKNIIIGFAYMEGSPVGFVANQPLNLAGCLDINASCKAARFIRFCDAFSIPIV
TLVDVPGFLPGISQEYNGIIKHGAKLLYAYAEATVPKITVITRKAYGGAYIVMNSKHLRG
DVNYAWVNSEIAVMGAEGAAEIIFREESKDPEKKQAKIDEYKEKITSPFVAASRGYLDDI
IKPQNTRWRICKSLKYLRTKKTDTPWKKHDNLPL

>gene_1155|GeneMark.hmm|342_aa|-|29726|30754 >NVVL01000052.1 Rickettsiales bacterium isolate NORP64 Contig_source1382A_23653, whole genome shotgun sequence
MIENDNQNADLMVAEAEEDTAFVEILEPEPSEEEQYLSHDPLICGTLAIKSYAIEFLEDY
LPAEIQKSVDLSTINVEPESYVEDTLRKKMSDLVYSVKTKEGDDAFIYLLLEHQSSVDYW
ISFRLQKYMLLLCERHKKNKEPGKLPIIYPLVLYNGKAKYTASLNFWGLFDDPKRAKEVM
GGDYTLVDLHAMSDDDINYERYSSLVLSAMKHIHDRDTLKMLDLLFGRCKIALSLDKKED
YVLLRMIIEYTNPKVPAEKRKELEQVIKGHLSKKEGENFMKTVKDSYKEEGREEGAEQKS
LQIATNLLNQSLDQSFVASVTGLSISEVERLQSSAHNASSSL

>gene_1156|GeneMark.hmm|224_aa|+|31073|31747	>NVVL01000052.1 Rickettsiales bacterium isolate NORP64 Contig_source1382A_23653, whole genome shotgun sequence
VRRVKSLLLILIIFKSFAIFAHIDRELLESQKCSNIFGHFEKEYDLPKDTLYAISLQETQ
KTHSKHNIWFAWPWTINVGGKGYYFKNKEEAIKFARAKIKSGKKSIDVGCMQINLKYHAK
AFDSLEEAFTPAKNIAYAAKMLKDNHRRFGSWREAIGRYHSGIKKRAVRYHASVNRIISS
IDVYKKHLRDYIIKSQKRNEEFEPFSIQISVASYLSGSWFRASA

>gene_1157|GeneMark.hmm|294_aa|+|31796|32680	>NVVL01000052.1 Rickettsiales bacterium isolate NORP64 Contig_source1382A_23653, whole genome shotgun sequence
MFDLQKLERNRAKSYAFIKGSNFHQFVCDDILNRLEPVDRDFKDILVIGSALGEMVCADV
KKHYPNSNISLTSEVDNLEQQSKDHQSKDHQSKDHQSKDHQSKFDLIIFPFGLHWVNDVQ
IFLAKIKRMLTDDGVFICSFPAAGTLPKLRLHLVQLEEKHSTSHVPHISPFIQFEQVTQL
LQQAGFVENIIDTENLELEYDSPLALMRALKNHGESNALTCAPVYSITKAMHKELGKKKE
SDSEGISSGTGEEKDSAEEADMKEDSFIDFVNLTSFISSATKKTIKLKPKYFQG

>gene_1158|GeneMark.hmm|465_aa|+|32790|34187	>NVVL01000052.1 Rickettsiales bacterium isolate NORP64 Contig_source1382A_23653, whole genome shotgun sequence
MQEFDLVVIGGGPGGYTGAIRASQLGMRVACVELRKNLGGTCLNVGCIPSKALLNFSEKY
EEAREHFADIGIITNAKLDLAKMLAKKDGVVSDLCKGIGSLFAKNKITRFSGVGKILDDK
TVEISSGSKKEKIKAKNILIATGSEVIDIPGVEVDEESIVSSTGALTLSKVPKELVVIGG
GYIGLELGSVWRRLGSKVTIVEFADRIVPALDKEIGKSFTKILEKQGFNFMMSTKLVKAT
KKSGKVELVVSGVGDSKESKLSCDVVLVSVGRKPNTEGLGLVELGVILDDRGRISVNENF
QTNMPNIYAIGDVIKGAMLAHKAEEEAVAAVEIMNGQAGHVNYNLIPGVVYTWPEVASVG
ATEEELKQTSVNYKVGKFPFMANSRARSVGATDGMVKILACAETDRVLGAHIIGADAGTL
IGEIVAYMEFGGASEDIARTCHAHPTLNEAIKEAALAIEKRTINM

>gene_1159|GeneMark.hmm|296_aa|+|34312|35202	>NVVL01000052.1 Rickettsiales bacterium isolate NORP64 Contig_source1382A_23653, whole genome shotgun sequence
MSRVSVGGENYGSADNPIEASVSAREVMEVVDAWAHSKRSNSLYGLMADLASSFGKDQSK
LTNIMNILRGGEVVHILGNEGILFKCSCLNSIEKRESLERSEGMDGIKKGDPINSSKSIE
EFKSFLEPGARHSFEDIDAIRSVFADYFDEEGVERLIPIIEQGNKYAYIRGTSARVVLLK
VSKDGNDSPYILELETIKKGSQSNAISVGKTWQDFSDKISEFCRLKESARSFDAFKNLLD
SFLTGLTLENIVSILATGGPVYLKGYDEVNILLKTSHDNDVEITGSIMPWCQNLDA

>gene_1160|GeneMark.hmm|144_aa|+|35273|35707	>NVVL01000052.1 Rickettsiales bacterium isolate NORP64 Contig_source1382A_23653, whole genome shotgun sequence
MSRDNIPYIGLVGAGQVFSFSVERGGAKFLGLEFSGEHMNWVFALFDRIFQVLRISWWVE
ALLDEWRGDIAEEPVVDVRPEEARSIFRFSIDVYHIKYLHIDFSGECADWLCDVQDELAA
FVQDAQEMLGVLGGDAVAEAYQFG

>gene_1161|GeneMark.hmm|69_aa|-|35838|36047	>NVVL01000052.1 Rickettsiales bacterium isolate NORP64 Contig_source1382A_23653, whole genome shotgun sequence
KDEGKGIPKTEIYDIFTPFKMGSNTESKAEGRGVGLALCKSVIEAHDGEIEAESHAVGAL
FRVCLPYGK

>gene_1162|GeneMark.hmm|104_aa|-|90|404 >NVVL01000053.1 Rickettsiales bacterium isolate NORP64 Contig_source1382A_23664, whole genome shotgun sequence
MNNIENYYHLRKEGLIHHVSGLVTESAEDLGTNIAETLLYNELEAVKTQTPEEIEEMRAI
HDQISNNIDTAICKYRDSLIEEIKNEAMKIKVSNASITKEQKEC

>gene_1163|GeneMark.hmm|84_aa|+|856|1110 >NVVL01000053.1 Rickettsiales bacterium isolate NORP64 Contig_source1382A_23664, whole genome shotgun sequence
MVASKISRHSDKLESYDGGDIFSDIRYICSSTSLIVGSLQKGFDVAQLPSGDVIVTEVKT
INTQYSWDKKRKKMIKVGIVKLVE

>gene_1164|GeneMark.hmm|180_aa|-|1150|1692 >NVVL01000053.1 Rickettsiales bacterium isolate NORP64 Contig_source1382A_23664, whole genome shotgun sequence
MARERRSSAEIVETWNGQEEGVAYYDLVQEMSELLKEALGEEDFERAKTIIVNAKKCNPA
EVAWEGLGSLMLNMFDSISSQLPICEALQTFTPGIHTMTDIDFKAHPATPEQVRNFAALL
PNEEKLKLVYVHSVPLGMQNAMAQIWDAVPQTTTVQVDYIGGYSERGLPGAMETQEPTEG

>gene_1165|GeneMark.hmm|165_aa|-|1787|2284 >NVVL01000053.1 Rickettsiales bacterium isolate NORP64 Contig_source1382A_23664, whole genome shotgun sequence
LNHMLDSQLIAIVEKIKTAAQKGVTISAAESCTGGMLAAYLTAISGSSKYFHGGIVSYSN
SAKINLLSVNVGTLEKFGAVSEETAREMATGARKVLASDLAISITGIAGDTGGTRDKPIG
MVCFAIANNEGVQSYTHHFTGDRTEVRQQACKIALELLLAKMTRI

>gene_1166|GeneMark.hmm|201_aa|-|2281|2886 >NVVL01000053.1 Rickettsiales bacterium isolate NORP64 Contig_source1382A_23664, whole genome shotgun sequence
MDIRKKIAELFATIFYIGKIKYAPGTLGSLVAFPLCYMIVYLTSNSQFVFQISSLNFEES
QIFTLFTVAISTTLLIFIAGTYATKIYIEGAEEQDPSEVVIDELAGQMLTIILSSFSVFL
LHGTQIASMYDAQTIDFLLLFLLPFILFRFFDIKKPWPINWMDKNIKGALGVMLDDIAAA
LFATITHYAIIFIILDFYKMV

>gene_1167|GeneMark.hmm|108_aa|+|3006|3332 >NVVL01000053.1 Rickettsiales bacterium isolate NORP64 Contig_source1382A_23664, whole genome shotgun sequence
MKELIAKFDDFEERFAKDTSDPELIKFFDEFVGYVEKIDLDSEKHLQIKQRMHKLQQLFA
SRKEELTEESNNLMVKQKQFNRYITNFGYKNFSYKDGLTNKKSGAKWN

>gene_1168|GeneMark.hmm|92_aa|+|3340|3618 >NVVL01000053.1 Rickettsiales bacterium isolate NORP64 Contig_source1382A_23664, whole genome shotgun sequence
MELEIILKALLSLGFVFAVMYVLLKIMQKYSNVGSGSKLGGAESGLKIENILYIDGGTKI
INIANKSGSNYVIAVAKNNSFLIDKYKTKKEE

>gene_1169|GeneMark.hmm|249_aa|+|3615|4364 >NVVL01000053.1 Rickettsiales bacterium isolate NORP64 Contig_source1382A_23664, whole genome shotgun sequence
MNRKFALLNIYILLGTLIPELALASDSFATELEHLLSSSSMTSRLMQIFILLTVLGLAPS
ILIMVTSFVRIAIVLSIVRTALGLQQSPPNQVLVSLALFLTFFIMSPTLEESYEAGLKPM
LEESMTEEEAFPLIVQPFKNFMTANTRPKDLDLFCSIAKIEIPKEAASLPLRVIIPSFMI
SELKRGFEIGFLIFLPFLIIDIVVASILMAMGMMMMPPVMVALPFKIIFFVLIDGWYLLA
GSLVQSFAT

>gene_1170|GeneMark.hmm|105_aa|+|4385|4702	>NVVL01000053.1 Rickettsiales bacterium isolate NORP64 Contig_source1382A_23664, whole genome shotgun sequence
MTKEITDSEFEAEVVNSKEPVLVDFWAPWCGPCKMLTPILDQLSEEMAGSVKVVKMNIDD
NPEIPSKFNIRSIPTLMIFKDGKSVASKLGALPKNSIKAWIDSSI

>gene_1171|GeneMark.hmm|1046_aa|+|4706|7846	>NVVL01000053.1 Rickettsiales bacterium isolate NORP64 Contig_source1382A_23664, whole genome shotgun sequence
MTSLNWGIVFEDLSSLSGLKKLDERFLQYVQSHDSGLRLELLSNRTKALAGYWDKCTYQE
KSVRELEYSEFLLNLAPLLDDFIADLFNITEENLALKHQSSEFDIVYECRRKFIQRHALK
KYPKEKLDDSDFAVISLKMKELLGQISEQSIAKSVMKWQAEPEKFAPNLDIAAKYCAFMV
HKNSSLVLFDVPRRIDDNNHIRSHKIDQLSKDVCLGFDYRDKEQSMPRAMAHAKYCIYCH
KQNKDSCSKGIDSSINGCPLGQKISEMNQLKSRGFNIAALSVIVIDNPMIAATGHRICND
CMKACIYQKQDPVNIPLVESNILDQVLCLPWGAEIYALLTQWNPLNLAAPLPKPDSGHNV
LVTGLGPAGFALAHYLLKEGHSVAAIDGLKISPLHFDALRPIKYWSDVKLPLSQKSPTGF
GGVMEYGITNRWDKNNLSLVRLLLARRKAFRMYGGARLGSNITTKQAFDMGFDHIALCLG
AGKPRFMKSADYFAKGVRTASDFLMNLQQGGAYIHGSTSNLLVRMPGVVIGLGLTAIDAA
TEMLHYYPVQVENFLQKWESKENPEDGLGPEDKAIAHELVTHAKLLRAAKNDAERLKILA
GFGGVTICYRKTIKESPAYLLNHEEIEHAKAIGVKFEELMVPGEVKADEYGSASSLSFAN
GREIKAKSILIAIGTGSSEFQDIDGLDLEGAEIFRRKDNKISYFGDCNQKYAGSVVRALA
SAKNGYKSISETLNLETKQSLGSRRELCVDALGTQLKSYVKKVNILSDNIVELVVHAPLA
AENFKPGQFFKLQNHPKNHLKNQQGNPASEMEPLAITGAHVDKDKGLIWLIILEMGESSR
LCRTLQEGEEIVLMASGTATSIAQNKRVLLIGGGLGNAVLAPIAQALLANSCHVTYIAGY
KKLKDRFYPERIEQNASKVIWACEEGEASARADDLSIKGNIIAGIEYARKKNLLGGIDQV
ICIGSDRMMRAVSSQRKRLFGSAEMICSINSPMQCMMKGICGKCIQKTSDARGYIFTCEC
QDQNSDIIDFDALEKRLAQNSLLEKI

>gene_1172|GeneMark.hmm|253_aa|+|7980|8741	>NVVL01000053.1 Rickettsiales bacterium isolate NORP64 Contig_source1382A_23664, whole genome shotgun sequence
MTKSSEWQMILGSSSEDWRLNFVSVMNSLLVMCEEGFGSNAEKGVASSGLNARSNEILAK
SLDALPADMKPYCEQGFSSNILGFLAFNEKVKGFLLSQVFLLLSSPEAQQVDQFYDLRGQ
AIKLLKSLSLSKQVQYLVEKPVMVVLGDDDYAEAVDALGEEYLLTILKAPGAIEQLGDLG
AVIQDVQDITTLNKILWANLAVDLGIVQARKDELAREAAEELAAAEFVSDQELTELPPLG
EDYGYVAGTYNTD

>gene_1173|GeneMark.hmm|463_aa|-|8932|10323	>NVVL01000053.1 Rickettsiales bacterium isolate NORP64 Contig_source1382A_23664, whole genome shotgun sequence
MSKKPDGMDVSNNGSTTKRTAEEALGGGTESAKRVKLTTTDVAVDPADKHGHTALMRAVM
EGHVGVAMELIENGATVNLTDEQKTNIFMRATHNGHANVVAVLIENGTNVNLTDNDGRTA
LMFAIHNGQADVVAVLIEKGATVNLPDKQTTNIFMWAIHNGHANVVEVLIEKGATVNLTD
EQTTNIFMRAIHNGHANVVEVLIENGATVNLTDEQTTNIFMWAAQKGHADVVALLIKQGA
NIDQLDPNQDTALMFASQYGHANVVAVLLQRSANIHLVNDRDDTALGYAYRYGHDNIVKL
LREEGANIDLTTSAHDLEWVFNDFQMPVFILAAKYGKLEIARTLLQQGTNVDLADEDGGT
ALTWAAVNNHADVVAMLISGGSNIWVLSVEEFVNNPTSALLLRHIGSSMLKITAELYNET
LVDLLKLVVSTIMPQDYGVSNLDSSFTFDAHMIGNGAIDDDML

>gene_1174|GeneMark.hmm|1065_aa|-|10758|13955	>NVVL01000053.1 Rickettsiales bacterium isolate NORP64 Contig_source1382A_23664, whole genome shotgun sequence

MNILQIEASNPEISTWVSASAGTGKTKILTDRVLRLFLQGCPPNKILCLTFTNAAASEMR
ERVTSSLAKWSGLSEAGLKEELSKTLGRPPARGELTRARNLYSAYLSLEEKINIQTIHSF
CQKLLKKFPLEAGISPSFRIIDETKSYFILQQIKKTLLYKEDLAPIHEYLTANFHEMIID
EILSEIVGQRDKFLNLDIDNIHEKSLQIISELQKDFDQHYSAILRHQIVQDIVGFDVSVP
ELKSFFLTASGQKKKRIVPQKIAKPGSNLYSDLEHIQHETHQIDQANRSHQLEHHSKLLS
LLGASILRTYAEFKSNKGLLDYDDLIIYASKLLKSSDAKEWVLYKLDGGINHLLVDEAQD
TSSTQWQIIDAMIGEFYAGESGREENRTVFVVGDEKQSIFSFQGADVASFAHMNKLLKHK
MTSGGKEFKDINLSISYRSAAEIIESVHHVFSYIKNHMPDNFHPHLPKIEAFRTKHSGSV
ELWPICKTNEVPESFWPIELSEESKATSSHALAGKIADYIEKQIQSGRILPSTGAPVAYG
DFMILFRKRDEFTQEVIRALKNKKLEVSGLDRISLRENLAVRDLLAVAKFVINTHDDLNL
ASLLKSPLIGISEEELYDIATKRGENSIWEYIQNKLADNNSSSDNSSNIFTKLSVFIDLF
DQSNLNNFFQHIVDILDYRETLNAHCGADSNDAIDELLYASRNFAREHGTSLQSFIFWLE
NIDTSIKRDSASLNKIRIMTLHASKGLQAPFVILCDTTGTPVNSDRFLLGVDGKTLSSAK
SSSEPDYYKELKALKQSKAYGEYLRLLYVGMTRAEDHLVICGYQGSKKLPENCWYELARQ
AMQDFAQLSEDGRLIYGTEEAAARLDDSALKQASGVEFFYNRKQVLAQKENVIPKESDYH
KPDYSPLSKKDPMGYGLAFHKILEDSIKIADPGSMRSHPLIKTLDAKPQKRIKSSIEKIL
ANQEFMQLIQQDAKTEIGIGTRADGALKIGRIDLIVTRAQEVIIVDYKSDLLPPAADKPV
PENYYNQLLFYRETMQKIYPNKQIRTMILWLENGKLRELEDAQAT

>gene_1175|GeneMark.hmm|254_aa|-|14113|14877 >NVVL01000053.1 Rickettsiales bacterium isolate NORP64 Contig_source1382A_23664, whole genome shotgun sequence
MNIIHAQNKENFISYNRLVAAKKKAPFVIFHHGFMSDMQSKKCQLVESFCKKQGYNFIKF
DNYGHGESSGEFLNHTISSWTGGLELIVDNLAGDRPALLVGSSLGAWITALFAIKYPKKV
MGMVLVSSAFDFTEELIWNNLPQEQRDILERDGFYEVIGTDPSCDHTYPVSLDLILDGRK
NLLLTRDKINITCPTHLIHSMRDIDVPCSISKRAAEKIVSDDVVLKLIKNADHNMSGEKE
MHIVCNSIEELMEG

>gene_1176|GeneMark.hmm|386_aa|+|14906|16066 >NVVL01000053.1 Rickettsiales bacterium isolate NORP64 Contig_source1382A_23664, whole genome shotgun sequence
VTTTRKYHKRHYKKTILQVVPALISGGVERGTLEIAKKIVDSKNISFVISSGGPLVDALK
KAGSEHITMNVASKNPLTILRNVSKIIKVIRDNDVDIIHVRSRAPAWSCCMAAKATGATL
ITTFHGIYNFKSWIKKYYNSVMTEGKRIIAVSNFVKKHIMDNYRVDESKITVIHRGVNHK
AFEQKKLSAEKLSRFREKYNVPAGSKVILLPSRVTAWKGHMVLIDAINKIKDLDFYCIIA
GDLAKHPGYVARIKDKIKKYKVQHRVQLFGNEPDIMALYGISDIVLSTSVEPEAFGRTII
EAQSMEKMVIATNIGGACETIINDKTGFHVAVGDCEDLANKIRHCLSIIGTDEAKTITTN
ARKIAQEKFSLDCMLNSTLAVYDEMS

>gene_1177|GeneMark.hmm|280_aa|-|16199|17041 >NVVL01000053.1 Rickettsiales bacterium isolate NORP64 Contig_source1382A_23664, whole genome shotgun sequence
MRINNNFPAPSYSSRVNTIKYVIIHFTEMEFDGALSRLTDPAAEVSAHYLIKEGGEVFQL
VADENIAWHAGKSFWNGEESLNKTSIGIELDNLGNRAFDAEQIKACLELCGILQKKYDIP
SFNFLGHSDIAPDRKIDPGIFFDWELFYKNGFGMGARSRRNLAEREQDLGTRRQDIAKSR
QNLAKDEQGLEMGVFLSFGDVSEDVRSMQQRLQILGYKIDVTGIFDEQTNFVVRAFGAHY
FPEILQEKGLAAYQKLDSKYDWYHGADAFLNELIRIYKTA

>gene_1178|GeneMark.hmm|272_aa|+|17120|17938 >NVVL01000053.1 Rickettsiales bacterium isolate NORP64 Contig_source1382A_23664, whole genome shotgun sequence

LDNILTIEKTDKPHGAVKSEVLSLVLVIILALAVRTFIFELFYVPTGSMRATILEGDYIF
STKYNYGYSIYSIPFNPDLFEGRAMASQPEYGDIVIMRPPHNMEERYIKRLIGLPGDKIE
IVNDLTYINDKPVTRLEVGQFVSEDEIKYIKFRETLPNGLSFFSYKLKYSTKELGADQRN
YGPHIVEAGHYFFMGDNRDNSGDSRYQLGMVPFRNLIAKGRFIFFSTKESLWDSKAGFVE
QITRVWTWLSSIRFNRIFYKLYEPKSDSGNGK

>gene_1179|GeneMark.hmm|229_aa|+|17928|18617 >NVVL01000053.1 Rickettsiales bacterium isolate NORP64 Contig_source1382A_23664, whole genome shotgun sequence
MESNFSKESISPLEEAIGYQFKDIILAEEAFSHPSLKQVKESTLNYERLELLGDSILGFL
VTELIFKRFTSEEGILAKIKAHVVGAEILVKISESLGLADYIIMTNGEEKSGGRLNPNNL
ENTLEALIGAIYLDSNIDSVRAIVANLWAPYIENVDFSASDPKTFLQELVQRNGEDLPKY
TVIGREGSDHSPLFTVEVKTEEHTETGDGKSIKEAQKNAARMLLGKINN

>gene_1180|GeneMark.hmm|299_aa|+|18647|19546 >NVVL01000053.1 Rickettsiales bacterium isolate NORP64 Contig_source1382A_23664, whole genome shotgun sequence
MQKQKALSICIVGKPNAGKSTLLNRIIGTKLSIVTPKVQTTRSIITGVVTLGDTQLVLFD
TPGIFEPKKKLEKAMVRCAWSSFSSADIVVLIIDSSSKLDEMSETIVKRISSLNQKIVFL
MNKIDLRSKYFLDNIKFLEESVPKARIFNISATKGNGVDGFLNYLKEQATESGWSYGKDD
ITNLPSRFLGCEITREQLFLQLQQELPYNLTVECESWTEQKDGSVKVRQVIIVARDSHKN
MVIGKHGSRIKEVGSKARVNIEKLLGQKIHLFLFVKVRKDWENNPEFYQAMGLKYLKDE

>gene_1181|GeneMark.hmm|259_aa|-|19602|20381 >NVVL01000053.1 Rickettsiales bacterium isolate NORP64 Contig_source1382A_23664, whole genome shotgun sequence
MKESKLKVENIAILHNDSQDLVKIARSLEKMFNRTSPQEADLIIVIGGDGSMLHALHKYM
HLDIPFYGVNAGSIGFLMNDLHTENFLGNIHNSKITKLYPLEMHTIGEDGSESKALAINE
VSIFRKSNQVAKFKIEVDGVPRMELSADGALVSTPAGSSAYNLSAGGRIVPLAAKILCLT
PICPFRPRRWSGALLPIDSVVRFEILEPKKRPVNAVADFHEFQNIQSVTIKSTEEKAIRI
LCDGHHTFEDRVIKEQFTY

>gene_1182|GeneMark.hmm|558_aa|+|20496|22172 >NVVL01000053.1 Rickettsiales bacterium isolate NORP64 Contig_source1382A_23664, whole genome shotgun sequence
MSFNLKTNKDKLLFVPLGGSNEIGINVNLYHYKGKWIMVDCGSGFADEHLPGVDMVVADL
SFIEAHKKDLLGLIVTHAHEDHLGAIQYLWNELECPIYTTKFTKTFLKAKLSEYSFPDSM
KIHEVDPKKKFNLGDFSIEMIGLTHSAPEMQALMIRTDAGNILHTGDWKFDPKPIIGEPS
DEKALKACGDEGVLALVCDSTNVFNKGTSGSEGDVRESLVKIISECPKMVVVTTFASNLA
RLDTIIHAGQKAGRKVVLTGRSLHRMLTAAQESGYLNNIAPLVDERSIGSHKREELLIVA
TGCQGEEMAATSKIATGIHNSITLAPKDTVIFSSKIIPGNEKKIFKLFNIFVKKGIEVIT
EKDHFVHVSGHPAIDELKKMYKLVRPEICIPVHGEPVHIHEHARLARSEGIKHAIEVENG
SVVLLDKKSPKIVDKVENGYLAVDGNYLLPDNSHIFKMRRRMRDSGIVVVSVVLDENQRV
ALPPMLSLPGLLDHIEDAELISILKDEIIKGLKDQRKKNRGVLLVDQIESCIKTSLRRAI
KHEVKKSPIIIVNLEEID

>gene_1183|GeneMark.hmm|55_aa|+|22391|22558 >NVVL01000053.1 Rickettsiales bacterium isolate NORP64 Contig_source1382A_23664, whole genome shotgun sequence
MTCQDVYKEEGKEEGRVEGRVEGAEHKSLQIVTNILQQQFDLKQIVSITGFLLAR

>gene_1184|GeneMark.hmm|1360_aa|-|22861|26943 >NVVL01000053.1 Rickettsiales bacterium isolate NORP64 Contig_source1382A_23664, whole genome shotgun sequence
MSKIYPANKKRIAKKKATGKKLLSSSVLAGLFISSACYTPLANSQDYAFYNQTRTFSGNI
VASNNNPPAIELYNSTVIFKGTVEANSNRSFALYLRNNSTANFKKTMTANENSIGIFIND
NSTVKFKKAAEFNKNSTGVFLNDSTGNFNHTLTANENSLGMILNNSTGKFKKAVEFNKNS
TGMVLDNSTGKFKHTVTANENSLGMRLKNSTGNFDKNVTLTNNETGITTNTSTIIFKNKL
IVSESTITGISILNSDVTFDTVELNNNKLGLSVTDSTVIFNGPLTVTNDSPVSSTGITAS
NGELVFNDDITSHDLILSDNSKIILSDGITASVPITTNSNNKNSVICKGSVTINHNIGEV
GKLLEEVSFNSAGSRNSITLNAPIFASNALLDGTLGNFTNGIIISNNLKIKGASAANFIK
GVSTGKDLSITDNATPDFQDDVTVSETLNITADAKPVFHKAVSSKDLSIADNAVPDFQDS
VTTTGILNITVDAKPVFHKAVSSKDLSIADNAVPDFQDSVTATGILNITADAQPVFHKAV
SSKDLSIADNAVPDFQDSVTATRILNITADAKPVFHKAVSSKDLSIADNAVPDFQDSVTA
TGILNITADAKPVFHKAISADADITITDNSEPEFKETVSSTGGNITVDLSSKPVFNNLAS
SVIVQGNSEPIFNNNTNGDVTILGTPTVAFNGASGGGVILPSSASATAPTLNFAADFSSN
KELVIGSGAEVNLPEPTLNGTVRFAGLGIEADAKIKLGDNVILDTKISPLVDKEGRLEFL
GNATISQDIGSGGKWLKEINFNSADSNIELMGNIFSETMSFNASNIKLLNDTKITTGQNS
LNMHNNSLDLGSKSLLVSGKSVLTGKTTISTTFIADPGGNQGGHIIVDGAGSSFDFSGAN
PVEIILNADSPLPSASEYEYKLFTTQNGGTVIPANNITFTSAENNSLITWRYEPSTYILT
SANEPEQLPVIVAKAGGDIEDVKVATFISNADLSDSEDATASILSNIGRLSEAEILEAVK
RIQATANFGELADNIATQISNKLGERLTALTMENKFARAGVSSGDSGDDSGISMVNSADK
YGVWMNGFYGKGRQGQREHSPKYHSRMHGITLGADTALRQDIIIGLAVSGVQTNSKFSNN
KIDDKIKVNSLIFSLYAHKDLSNEWFIDGMISGGTNNITGKEQRILLGGKKAIATSKYKV
RTHNGQFTVGYNKNISKNIILTPLAGLGYSWAGATKYTETGAGKQNLSVTTDSTYVLEAI
FGGQIAFQKRIKNIAVTPELHAHVSYNFKTKRSLINAKLAGAKAPIPACLPRTNKLFYHI
GGEINAQSESGKFEYGINYEAKLSKKYINHVGGLKLRVNF

>gene_1185|GeneMark.hmm|179_aa|-|27137|27676 >NVVL01000053.1 Rickettsiales bacterium isolate NORP64 Contig_source1382A_23664, whole genome shotgun sequence
MVKFLSEFAPLIAFFYGYKTGGILEATLYMVVVSVIGIVVTYIVERRINKVNLVTTALLL
VSASLTLFSGNSIFIKMKPTVLYCLFALALFVTHFKWRPAIQYALGAAIKFKDQRAWYHL
NKRFMFFFLLMAAANETIWRNFSEDIWVNFKVFGALPITIIFIMMQVPFIMKHKIDEEH

>gene_1186|GeneMark.hmm|273_aa|-|27669|28490 >NVVL01000053.1 Rickettsiales bacterium isolate NORP64 Contig_source1382A_23664, whole genome shotgun sequence
MFKIAVIIFRECLEMAVLLGIIMAITKPIKNSKAYIALGSGVGVVFAAIFAMLASLIADS
FGGLGDEIFDAFVLLITTAIICWTVVWMQGYTTKLKRDLGDLSYQINAGTTSKIMLVMMV
AMAVLREGAEIILFVYSISSSENIAVSEYVIAILLGGFVGFSLGAMIYLGLIKCTGKYIF
SISTTLLILIAAGLSAQAAGMLTSSGIIEIYSDQVWDTSWFVENASVTGKVLNITVGYDS
KPNSLQIIFYLTTIIITLAMMKLRSILSNKSNG

>gene_1187|GeneMark.hmm|118_aa|-|28483|28839 >NVVL01000053.1 Rickettsiales bacterium isolate NORP64 Contig_source1382A_23664, whole genome shotgun sequence
MKKTAILYFMICALAAALPAYAGGFVEVDLIIKDHKFHPETLKLPAGKKIRITVHNQDST
IEEFESIDLKREKIVLGNSKARVILAPLKPGTYKFFGEFHEETAQGKIVVEQETGKDV

>gene_1188|GeneMark.hmm|522_aa|+|29319|30887 >NVVL01000053.1 Rickettsiales bacterium isolate NORP64 Contig_source1382A_23664, whole genome shotgun sequence

MLINIPLVLAIIVLLSLVGNLYVYRKLKFAANKTTKGILSGAEDLTYRIDALTRQNQALF
TMITEDLSSDPSKSDKLRKLCNVMIDDSHNPLEEAKGYQCLAKLHLADKNKGQLDRLKAI
KNTSYALNLSHDPEYVRLSYVAILGESVDELISLFVAQSDDALQKLLSSLNVADSTEEIK
LLEKIIQLADKDVKIRAKYRLSHIYQNGHSAAESTEIPIDYIKLLENRKYVFENVKTTSK
NIVNIALTFDDGYAAHASTNIASLLVNADPETHYKFYIVDGVDVQISQENREKLSSLNYI
KEYDINFITFPDEILGKIDEKYITARFPKIVLYRIFLSKILDISKIIYLDSDLVVLRDLW
DVFNTETSFISATLDPLSFRHAKLPRNNKAMCKSSVFYINSGMMLLNLDAMRGRDLAAEI
QDVIKNAKYPFNLPDQDMINFTYKDKINFISPKWNLNNLKDTGFAKHFVGTKPWVWPTSQ
DATETWKNAPQTVPEGHRRYWHYLDITPWTTKKDKLQAVISD

>gene_1189|GeneMark.hmm|72_aa|-|30923|31141 >NVVL01000053.1 Rickettsiales bacterium isolate NORP64 Contig_source1382A_23664, whole genome shotgun sequence
MAISKFKSHCLEILDKLEKSKSSIIITKRNKPIATISPFVRKKESLFGLLANKAEIKGDI
IAPIDDSWEANK

>gene_1190|GeneMark.hmm|363_aa|+|31364|32455 >NVVL01000053.1 Rickettsiales bacterium isolate NORP64 Contig_source1382A_23664, whole genome shotgun sequence
MWNWQRNDWPNFSYDSAVLEEFEAKFLEKAGENLGAFKHVSEEEKTQLRIDWISEEALKT
SEIEGEFLNRQSIQSSICNQFGLKTDNHRIPPAEQGIAEMMVDLYENFNEALDHDTLWSW
HKMLINTRRDIQAIGSYRTHKEPMQVVSGALHAPKVHFEAPPSKIVGRQMDAFIEWFTTS
SLPALTRAGIAHLYFVCIHPFEDGNGRIGRAISEKSLAQSLGRPTLIMLSYTIEQGRKNY
YQNLEQSNKHNEITQWLVYFAEMVLEAQDNTRKRIEFVIGKKKLYDRLQTLLNPRQEKLL
ARMFRENIEGFKGGLSVKNYINITKAPRTTATRDLQDLVEKGALVKTGQLRYTRYYLNIP
SFD

>gene_1191|GeneMark.hmm|643_aa|-|32571|34502 >NVVL01000053.1 Rickettsiales bacterium isolate NORP64 Contig_source1382A_23664, whole genome shotgun sequence
MSRTSLQSTQITPFFENELIQDQFHQYLLIMMGNDNTVAASALAEKGSSLGYEFQAKLRF
FKREIQLMLQQAITMDNEVVVNMDRITDLDERFKGLMDEGFLSCIKKANDQHSAFKGVID
GAENSLEIRIILVDKIYTQLISQMQIIPDLYAASYIVQQGLTTYQGDCFNGFMHFIATNE
TDPRVLELLTNSWLAAGRSLSENNGTLSTTTPLNIALLTDNVPFITALVERGVDITAPNE
DGTTALHLLIYQIHLGISDISFLQKWLDAGLEVDLRAKLLAQKLELTEVTNLIGEGSEIV
QQTLDELVASITQNYLDKMILNGVSDIILTNFGDPNAMLLHYLIENHNTVPLSVFKHFLD
SELLVRGIIDSRKTALQHAVEMGAVNFSVAMAEHGIGTTELDNGRHIIEYVHYIGGEHKA
AFVEAIKASQAIDMSGEQAAQLIADALIAKNGEFVATLVKAGADINHNDHELLKMFIKAG
ADYSDQIELAVLHGAEITGDMLELAAAHGLGELLTGLKQVQDQEAPVEMLQDGAITTIKA
LLVVMSEGSPSDEIMPLLVTLRMQGLYNVLQQKPALFEQFCKQEGIWQEMVSRADARNPR
EEKSKDKENVILPSHRAEDDDGNDEALLAGVSATAETSDYPLD

>gene_1192|GeneMark.hmm|253_aa|-|34752|35513 >NVVL01000053.1 Rickettsiales bacterium isolate NORP64 Contig_source1382A_23664, whole genome shotgun sequence
MADNLKFKVRSLYKSFGAHDVLKGVNLDVKKDSSLVILGGSGTGKSVLIKTMIGLLSPDS
GSILYEGTESVNMPQKERLKALQNFGYLFQAGALFDSLTVQENIVFFAEKLYSLNKNDKR
DLASKKLRSVGLSEKILDFYPSELSGGMQKRVSLARAICTDPKVIFFDEPTTGLDPIMSN
VINDLIIKVRDELGATTITITHDMQSARAIGKEAAFLYEGSIIWSGSIDEIDDSGNPHFH
QFINGNIEGPVIV

>gene_1193|GeneMark.hmm|64_aa|+|2|196 >NVVL01000054.1 Rickettsiales bacterium isolate NORP64 Contig_source1382A_23672, whole genome shotgun sequence
LFLIENMMPKAILMALIDLKEIELRLYNHGFNIKNGKSELTLKPCTAKGILRIKQSASNL
GLSI

>gene_1194|GeneMark.hmm|51_aa|+|307|462 >NVVL01000054.1 Rickettsiales bacterium isolate NORP64 Contig_source1382A_23672, whole genome shotgun sequence
MSADIIIFIGFIIFNLIFGLLSSKNVTTIKGYAVGDQNFSTATLVATIVAT

>gene_1195|GeneMark.hmm|81_aa|-|660|905 >NVVL01000054.1 Rickettsiales bacterium isolate NORP64 Contig_source1382A_23672, whole genome shotgun sequence
MATKKAGGSSKNGRDSAGRRLGAKKSDGQGVIPGNIIIRQRGTKIRPGRNVGMGKDHTLF
AMTAGKVEFVSKRTHKLVNVV

>gene_1196|GeneMark.hmm|105_aa|-|916|1233 >NVVL01000054.1 Rickettsiales bacterium isolate NORP64 Contig_source1382A_23672, whole genome shotgun sequence
MFAVVKTGGKQYKVAKDSVIKVEKIEGPLGGKVELDQVLMMSDSLKASFIGTPMVKGASV
TAEITSQFRDDKIIIFKKKRRQNYRRKNGHRQDLTELKILNIVKN

>gene_1197|GeneMark.hmm|405_aa|-|1451|2668 >NVVL01000054.1 Rickettsiales bacterium isolate NORP64 Contig_source1382A_23672, whole genome shotgun sequence
MYKRTSTKLINDISQTFPVLLITGPRQVGKTTLLKMCADETRNYVTLDNMEARQLAQTDP
ALFLQTYKAPLIIDEVQYAPQLFSYIKILVDREEKNGMFWLTGSQKFHLMKNVSETLAGR
IAILDLLGLSQSELNGYGDDAKPFMPTSEWLEQAKKVDTSNQLSDIYKNIWIGSFPRVVQ
NNDIRDVFYSSYVQTYIQRDIKNILNVTDETTFYRFLCSVAARTGQSVNYANLAKDVAID
VKTAKSWLSLLETSGLVYLLQPYHNNLTKRLVKAPKLYFLDTGLCCYLTKWPDAKSLEVG
AMSGAILETYIIAELLKSYWHNGKTPHFYYYRDLDQKEIDLIIETGDSLHPVEFKKTATP
SQTASKHFHMVGKFGKKIGHGGVVCFVEKPVPLSRAVTAIPVAYL

>gene_1198|GeneMark.hmm|151_aa|+|2828|3283 >NVVL01000054.1 Rickettsiales bacterium isolate NORP64 Contig_source1382A_23672, whole genome shotgun sequence
MDWSTRQLKAKQLEKHLKSLSNIRLPATGWVKTIRETLGMNVRQLGQRCEVSGERISRIE
ADELNSKLTMATLEKVAKAMNCKFVYGFVPNGGIKDTIEKQAEFKAKSQLRRVSHSMALE
DQKVSDSAMRNQIKILKEDMLQGNIKRIWEK

>gene_1199|GeneMark.hmm|193_aa|+|3283|3864 >NVVL01000054.1 Rickettsiales bacterium isolate NORP64 Contig_source1382A_23672, whole genome shotgun sequence
MLFTYAKGATPLSPDEVYNLIPSHLMTQKELDEWEQYNIIKAETWASQRKRADFLSTKFV
VDLHKKMFNETWKWAGKFRTRQTNIGIEAIHIPVELKLLFDDIEFWIENATYHPREIAVR
LHHRLVFIHPFPNGNGRLSRLVADLYMDYQGQARFSWGSCNLTSDSAVRQAYLSALREAD
LGDYMHLIEFSDS

>gene_1200|GeneMark.hmm|197_aa|+|4305|4898 >NVVL01000054.1 Rickettsiales bacterium isolate NORP64 Contig_source1382A_23672, whole genome shotgun sequence
MFIQTEETPNPNALKFLPGVDISPETPVFLNSPKEAKTKSLLAVKIFQIDGVGAVFYGAD
FVTVTKSDAADWGVIKPEILLTIMDHLVAGFPIFDEKQKETPKVNLDDMSDIEKQIVEII

ETRVRPSVAMDGGDIVYQGFENGVVYLQLRGACSGCPSASVTLKDGIESMLQHFVPEVKS
VEAVESADEEITPSIRD

>gene_1201|GeneMark.hmm|207_aa|-|4961|5584 >NVVL01000054.1 Rickettsiales bacterium isolate NORP64 Contig_source1382A_23672, whole genome shotgun sequence
LKEFDKEFSMRKEDYWKGRKEFLELNPASNLPVLVLGEPGESGGVLVGTYSIIEYMTESF
EDFYLMPKAPLERAEVRKYISWFNDKFHREVSKILIDEKMIRLLMRAGEPRSVFIRAAKS
NLHQHFVFLTNLLKKNSYIVSDRISYADIAAASHLSVVDYFGEINWDSWGVIKEWYSIIK
SRPAFQPILQDRVAGFAPPKDYSNLDF

>gene_1202|GeneMark.hmm|259_aa|+|6456|7235 >NVVL01000054.1 Rickettsiales bacterium isolate NORP64 Contig_source1382A_23672, whole genome shotgun sequence
MSSNMWGACPGITISENPQSKSMMVTLNRVTQMQSSDRCPPELSRIPQIEFVIQIYKSLW
QEALSYAAYNDFSRWDEYMNQVQGDLPHFILTSDGLQYIALRTGQVKIIAQCMWNVKKYG
DDETIMSLDTFLARILMVSSTKYHVNLIVDGLKIADQAMPNHDLSTDMLRAFCDLAPQHD
SPEILPKLYTYALKKAVKLYESSHPEEQAILDSDALSDETGSLLGGLLDNPECVSVEQTL
LGDASVILSNMPGSSCAIQ

>gene_1203|GeneMark.hmm|268_aa|+|7246|8052 >NVVL01000054.1 Rickettsiales bacterium isolate NORP64 Contig_source1382A_23672, whole genome shotgun sequence
LLAYIILPFNLKQINTMSSAGEYRLLNAQLPEHTNPNEADVELIQPGDFVADHNTNTVDE
ALWGQALAEAARGYPAKWKNLTRESDGIIPLHMLNGRNLPGIALETNDSSMLAQFMQSLW
LHEDTPGIMSFSSFLEKLLGVLSKKSHINLSIEAVKMADEYRPYCNLPENVLSELSNLEL
NLTDLNISKKNYEYTLQEAIGVYKLSYATDEFLATLTESLRNFPAELVQLTVTFTHIHAH
ADQALLGAAESSLDALASDFICFPCAIL

>gene_1204|GeneMark.hmm|113_aa|-|8127|8468 >NVVL01000054.1 Rickettsiales bacterium isolate NORP64 Contig_source1382A_23672, whole genome shotgun sequence
MVKVKYVPDRGDIVWLNFEPQKGREITKTRPALVISSRKYNSKTNLALFMPITSQVKGYP
FEVELNFAAISGAILCDQIRSLDWKERRATKITKLNKDFLDQAISKLRLLIED

>gene_1205|GeneMark.hmm|78_aa|-|8462|8698 >NVVL01000054.1 Rickettsiales bacterium isolate NORP64 Contig_source1382A_23672, whole genome shotgun sequence
MQAQIQKWGNSLGIRIPKLLSQKLNLHSGSQVEIDVARDHLIIVKSSSELDMLLDKINDD
NRHHEIFDDDKRVGNESW

>gene_1206|GeneMark.hmm|919_aa|-|8802|11561 >NVVL01000054.1 Rickettsiales bacterium isolate NORP64 Contig_source1382A_23672, whole genome shotgun sequence
MKLYQSSSKENFLEEVARFVIANFAGNFSKLNIILPSGLLCNHLQKLLITKLGSTMLPTI
TPLAGLVAESEEIFKIPSEKIGAISKLEEKITLSEVIHSYKKLGYDPAQSLLLAPSLANL
FFEFEVNNITLAELKNIPILDQPEHWHSIYDFLSYASDNWYVHMKSLHKVTRASYQKLTF
RAKLEQLEKAPLDNMLIAGIAGVNQMTDEFIQDASKLPNCYVILPPSGESSDSAPNRKFS
PEDALYKIDKLLHLINKEKAPIPALGTGEPTILNKLLTDSACGLLKQRIDYLEFDNIFHE
ANYIATQCMDALEQNPDSRIAIIVSNLQSKEQYCTFLAKYQLRYHDLLGTDIGKHNAMSL
ILLIAEHLCCEFSLKTFFAILSHPLVNNTNAQEVKNLIRKYDRLCSSLESISTTIKQHGE
EHLQEYIVKLHESLSAEVKSNKFASILRLVIKSVELLLLPDLWKQNPDIAASLSEIVHINW
SLALKKIDDFPELLKQIISGGRLAETTSDQNITICKSHDAALANYDLIIMADLNEGTHPA

PIHSSPWLSAHMQKELKLDSKPAEFGNALYDFYLNLQNKCVLLTRACKHGSMQMLPSPFI
LQLKHILGDKLNCKTMMQAPQAQMQTLESAALESAALESTALESTAAQQENSLYATSKIF
PKQISATDIETLLRSPYNFYAKKILNLRMEEEIEEKPNLAQFGNFFHEVAEEYTKNYNAS
EMDKPFAFKEIGQTILSSAPIPEHSKRSWGVKLSAIACEFVEWDEKRRQNITTIYTELKG
QITLDIKGTPVRLIAIADRIEIDKEGNAVILDYKTGAVPTKKDVLSGLSPQLLTEAIILS
GGGFNIGHKEISKLIYVKINSSLPYIKTTEIELSKADIHNHKQGLISLLEHYVTSKSFPL
EQNLMKYDNYTHLARRGAG

>gene_1207|GeneMark.hmm|140_aa|-|11558|11980 >NVVL01000054.1 Rickettsiales bacterium isolate NORP64 Contig_source1382A_23672, whole genome shotgun sequence
MDLPEIYALLFTDTLVSNFAFNASSEMVTASMKIFGTYNPYHVILIASAAFFIVSCVNYL
LGIICYKIVIPFNSKEADLSRITNLRKSRYLIIIMMLSAVPFFGKFVMLFAGFCGVRPGF
AIFTGSLAKLVYYSFFMLAQ

>gene_1208|GeneMark.hmm|420_aa|-|12037|13299 >NVVL01000054.1 Rickettsiales bacterium isolate NORP64 Contig_source1382A_23672, whole genome shotgun sequence
MNKRETKIFQYKRLKKILAHAYKNIPLYQEKWDKAGFHPDQFRSLDDLSKIPITTKEDLR
HLHVKSIIAKNNKNEHENLRLIGSSGSTGQKLVVASSESKWDYDVLLSSAHYINKRYNTN
IHKALIMLDFGNRSIESSVERMLGLIPFISKRIKTISYKTKPAKIIEILKEYQPDTIYSY
PSTMSLLCHYIEQNNIEVDELRYVVLTSEVIFKNLRRDIKSIFNATIIGGYGATEVGVIG
YQYGDNDYFDLVSWKNIVEIENDKFLKGKHLHTLGTVLVTDLIGFTSPVIRYSGLGDLGK
LIEPKRSGFPALSELHGRKSILLTSCGGAHLSPYSLTSYLQEIKSISRFQIVQSAQTTID
LNLEIHEGLKGDLPKSDKTKINAFFKKILGDKIVVKYHIVDKIKKDGSSHKTSVVISHLD

>gene_1209|GeneMark.hmm|290_aa|-|13507|14379 >NVVL01000054.1 Rickettsiales bacterium isolate NORP64 Contig_source1382A_23672, whole genome shotgun sequence
MFNKPLISFDYAIKFLLKNKEGYEIIEGFISALFAAENYKPVKITALLESESNKEKADFK
RSIADLVVEDTDGNKYIVEIERAFTPNFMHKACFNSSRLIADNLSVSQDYTKIKKIFHIS
LLYFENEEMQKPIYHGKTVIHEVDTAHPIDLRIMNQGMVTFENHNVFPEYFFISIPRFDD
QINQEIDEWLYVMKNAEVKKDFKSPYMAKVAERLSVLKMDDEERNQYYKYLKESVNKEDT
LTAALEKGRDEGRKLEKIEIAKTMLAQGLDISLISKISKLSIEEIKELKN

>gene_1210|GeneMark.hmm|571_aa|-|14485|16200 >NVVL01000054.1 Rickettsiales bacterium isolate NORP64 Contig_source1382A_23672, whole genome shotgun sequence
MIEKKLGLLREQLNKHGFGGYIVPSVDEYLSEFTPSFAKRLEYITGFTGSNGLAMILADT
VLFFTDGRYIEQAFTQLDDKLFEVFDQKLLANFPWEDYLESDCIVAYDPKLLTMRALQSF
NSISTKPYEENLIDQQWADQPAKPSSKIYDYPTKYAGADYEDKISQCREFLQENDAASLV
ITDPDSVCWLLNIRAHDIEFSPLLLANAIITMENLYLFTDAGRLDSELLRPDVTILDESE
FGDTLKQTSGLILFDVNTCSSYIAELIMAQKYKQVKNPCLLWKACKNDTEIMHMQQGHVQ
DAVAVCEILSFLASGDVSDLTEYDISEKLTELRAAREGYVFDSFPTICGYRENGAIIHYR
AQAGSAKKLEESGLLLIDSGGQYMGATTDITRTVAIGEKPSAEHIKYYTKVLKGHIALGQ
ATFPEKVITGANLDILARQYLWNDGRDYPHGTGHGVGSFLSVHEPPQSISLFSTKTALEA
GMVLSNEPGYYVPGAFGIRIENMMYVTKSEHAHFLKFKMLTLVPYAKELIDENMLTDAEK
LYLKSYYQEINHHVSPLLSSAAQQWLKGQID

>gene_1211|GeneMark.hmm|699_aa|-|16328|18427 >NVVL01000054.1 Rickettsiales bacterium isolate NORP64 Contig_source1382A_23672, whole genome shotgun sequence

MMNSMNNTAKIEPEELSKGDARTEIKELSSKLREYNKEYYMEDAPTISDAEYDQLFHRLK
TLEEKFSKLLKPDSPTQTVGSTLQEKFEKHTHIQPMLSLGNGFSEEDISDFIGKIKRFLG
QEDFLPIFCEPKIDGVSFSATYEKGKLSIGATRGDGYVGEDITQNIKSIAGLPNIIPDAP
DLLEVRGEIYIDKSDMEELNLEREANNKTKFANPRNAAAGSLRLLDYGVTASRPLKYFAY
AIGGASEKFAGTQEELLDALTKFGFRTNPRGALAENMEEIFKFYNELLKEREKLPYEIDG
IVYKVNDFALQERLGFVARSPRFAISHKFPAIIAETKLLDITIQVGRTGALTPVAELETI
NVGGVNVSRATLHNFQEIERLDIRIGDTVLLHRAGDVIPKISGINKDKRPSEALKFTLPE
ACPSCGSKPHIDPIDVIIRCDNGLNCPKQLSESIKHFVSKDAMNIDGLGKKQVEFFLERS
LIKNPADIFRLEPVNTASLSKIENMDGWGAKSVDKLFANITKSKQVPLARFIYALGIRHV
GVSNAKILAKEFISLEKFVESMVKLASADKATFELLDNLDGLGHKILIEIGNFFECEQNI
STLKELSEILKVSNYQERAAGGVLSGQNVIFTGSLSTLSRREAKSQAEKLGAKVASAVSG
STNLLIAGEKAGSKLKKAQELGVKVISEEEWVQMVNESK

>gene_1212|GeneMark.hmm|278_aa|-|18424|19260 >NVVL01000054.1 Rickettsiales bacterium isolate NORP64 Contig_source1382A_23672, whole genome shotgun sequence
MQLQKKLLKDSLDKALQEDYGIKGDITSNSVIDKDARIKFEIKARQELVVCGAAIVQYYF
DEYSSIEYKIHINDSQCAHPGDVIISGTGLAREVLMLERVCLNYLQHLSGISTLTEAFVK
KTHNTKAKIFDTRKTTPLHRQLQKYAVTCGGGHNHRLCLDSSILIKDNHIAICGGIQIAL
QKAKQNQPHYTKIEIECDTLEQVRIAASEGADIIMLDNMSLEQIREAIEIIQDRTIIEAS
GNVSLETVEALAQTGVDMISVGKITHSAPAVDIGLDII

>gene_1213|GeneMark.hmm|67_aa|+|19384|19587 >NVVL01000054.1 Rickettsiales bacterium isolate NORP64 Contig_source1382A_23672, whole genome shotgun sequence
MHWSDIEDIASKLEESYAEEEIPEYNLPYLQEMVLSLAGFDDHEVEVSDITLKQTLECWI
DIRNGIN

>gene_1214|GeneMark.hmm|379_aa|+|19651|20790 >NVVL01000054.1 Rickettsiales bacterium isolate NORP64 Contig_source1382A_23672, whole genome shotgun sequence
LKYHIRYFCTIFKILLDFIFQREYNNSESWLGARISHGELGMDKERALQAALSQIEKNFG
KGSVMKLGQRQDMNIESIPTGSVGLDIALGIGGLPKGRVVEIFGPESSGKTTLTLHAIAE
AQKQGGVCAFIDAEHALDPVYAKKLGVNIDNLIISQPDNGEQALEIADTLVRSGALSLLV
IDSVAALVPKAEIDGEMGDSHMGLQARLMSQALRKLTASISKTNCTIIFINQIRMKIGVM
FGNPETTTGGNALKFYASVRLDIRRIGAIKDKDEIVGNQTRVKVVKNKVSPPFKIVEFDI
IYGRGISKEGELIDLGVKHDFVEKAGAWFSYGEIRIGQGRENAKKYMIDHPDVASELEEK
IKTKLLKPEGDGETKEVEA

>gene_1215|GeneMark.hmm|241_aa|+|20835|21560 >NVVL01000054.1 Rickettsiales bacterium isolate NORP64 Contig_source1382A_23672, whole genome shotgun sequence
MTNLEGQNVFITGASGGIGSAITKQMHALGAHVYISGSNIEKLEALGTELGDNYTIKQCN
LADAALCGSLFDDIEKLDVLVCNAGITRDTLAIRMSDEKFSEVIDINLKANFILNRAAIK
KMMRARYGRIINISSVVATSGNPGQANYCASKAGLIGMSKSLAIEVASRGITINVVSPGF
IESSMTDILPDAVKDAIMQKIPLKTLGKPEDIANAVTFLASPLSSYVTGQTIHVNGGMLM
V

>gene_1216|GeneMark.hmm|79_aa|+|21670|21909 >NVVL01000054.1 Rickettsiales bacterium isolate NORP64 Contig_source1382A_23672, whole genome shotgun sequence
MNDIEQKVINTIAETLKMGEEEISLESKLADDLGADSLDAVEIMMAIEAKFDTEISDEDA

SKMATTKDIVEHIKKNLNQ

>gene_1217|GeneMark.hmm|415_aa|+|21956|23203 >NVVL01000054.1 Rickettsiales bacterium isolate NORP64 Contig_source1382A_23672, whole genome shotgun sequence
MTSRRVVITGIGIVSSVGLNANSSWSNILAGKSGVKLLTGFDSDFDINKLSCKIASTVPD
FNPEDYIPPRDVRKMDRFIHFGIAAANEAIKDSGWAPKDQEGKDRTGVMVGSGIGGFGTV
ENTAASLHTAERVRVSPYFIPSALINLLSGHISINHGFTGPNSAVVTACSTGAHAIGDAA
RMIKYGDADVMIAGGAEATVTPLGIAGFAAARTLATGFNDNPTEASRPWDKDHAGFVMGE
GAGIVVLEEYQHAVDRGAKIYAEIAGYGLTGDAHHITAPSGRGAYRAMENALKDAKINSS
QVDYINAHGTSTPVGDGIEVKTVQRLFEGNKSVKMSSTKSAIGHLLGAAGSVEAIFTALA
IRDQIAPPTLNLHNPIPEATMNLVPLVAQETKIDYALSNSFGFGGTNASLVLRKI

>gene_1218|GeneMark.hmm|128_aa|-|23353|23739 >NVVL01000054.1 Rickettsiales bacterium isolate NORP64 Contig_source1382A_23672, whole genome shotgun sequence
MLKNPLQLYSLAVCLIACIVIMITSGLMLNNLTDLTLTKYTYKSHLNNFVTNEKYISYKK
SSNGKDNDFPANLTTEEIQTERLLARDNYIENRQNSAISSLISSFTWFLTGFFFFIIHWR
IYKRSSII

>gene_1219|GeneMark.hmm|195_aa|-|24065|24652 >NVVL01000054.1 Rickettsiales bacterium isolate NORP64 Contig_source1382A_23672, whole genome shotgun sequence
MNNIASLPLLASGLLCSPRLAGKLLHLRLQFCAILLRPALTMLFCCLAFIIASNANAAKQ
LPVPRFAAIKFNEVNARTGPASDCPIEWVFVKKGEPVEIVAEYGHWRKIRDIASEGGWVH
SSVLSGKRSVVVTAKGTSPLLEGIAEYKKIVVYLEQNIRCVLQKCKKDWCKIDCKSHKGW
ISRKALWGVYPDEHP

>gene_1220|GeneMark.hmm|577_aa|-|24645|26378 >NVVL01000054.1 Rickettsiales bacterium isolate NORP64 Contig_source1382A_23672, whole genome shotgun sequence
MDIHPLVTVIVLLASAVFIVAIFRKLNLSPVLGYLVAGAIIGDNGMRVVTYAQTSLLAEL
GVVFLLFAIGLELSIERLKAMRRYVFGLGSLQVLITSAIIAGIVILLTNDYNAAIIISGG
LALSSTAIVMQVVAETKNKSTQIGRVALAILLLQDFVVVPLLVIVPILGGSSEVTGSLPY
IVGNSMLKAFVALVIIFIAGRVLLRPLFSFLVPENSEGGELPIAITLLVVLTASWGTEHF
GLSLALGAFVSGILVAETDFRGKAEESIHPFKSLLLGLFFMSVGMNIDVMEIYNNITTIV
SLALALIVLKAIIVTALCILFGFNIGVSVHAGLLMSQGGEFAFILFGLGKDYGVLDPSVA
TTLLLVVTFSMALTPALASIGQKFAENFERKRGRTPDQILEFGAQDLTNHVIIAGFGQVG
KMVASVLDAEGINYIALDTSDDLVKEETANGLPVFKGDATKIRTLKAIGADRALTFVITI
NNESMVKKISTIIHKKFTDLEMIVRLKNLKNSAELYAIGVNTIIPQDYETGLQLGGAVLK
SIGVSEYEINRVKGQFRAGNYVAAKREDTLLETEEDE

>gene_1221|GeneMark.hmm|119_aa|-|26416|26775 >NVVL01000054.1 Rickettsiales bacterium isolate NORP64 Contig_source1382A_23672, whole genome shotgun sequence
MKEDTENLKDFILECLKGKKATQIECMELNVAVPFADYMIFASGRSVKNIRAIAEHVTLQ
LKHEMNLNANFEGAEGSDWVLIDAGDVVVHLFHPETREQLKLEALWKRRNSDASSGSDG

>gene_1222|GeneMark.hmm|214_aa|-|26783|27427 >NVVL01000054.1 Rickettsiales bacterium isolate NORP64 Contig_source1382A_23672, whole genome shotgun sequence
LVSEIAILNFEYIFILMKLTQNITNLQGLSFMRHASLKVGILGGTFDPAHKGHLMISNLA
LKYYNFDYVIWLVANQNPFKQTNKRDIFARGCDAQKIVTNPRIIVSSAEHDLKTRYSYDS

LNKLTQYFPTVRFSWLMGIDNAMNFRKWYRAQDILELCNVMVFDRLVQKRLVNLQSIGLM
GKAMVAKNERKAIIIARDALCDISSTQIRTKLRA

>gene_1223|GeneMark.hmm|78_aa|+|27408|27644 >NVVL01000054.1 Rickettsiales bacterium isolate NORP64 Contig_source1382A_23672, whole genome shotgun sequence
MAISETKLKTILQQNFPNAKVKITDLAGDQDHYALDISCQSFEGLTLMQQHKLVKKALAE
VLLSGELHAISIKTRSDV

>gene_1224|GeneMark.hmm|306_aa|-|27708|28628 >NVVL01000054.1 Rickettsiales bacterium isolate NORP64 Contig_source1382A_23672, whole genome shotgun sequence
MLKRVSLATMFLLLVSVILSATEADARKRREKIIPAPTKTSLVVDGKSGRILHSRNARKK
IYPASLVKVMTVYLIFESLESGRLKLNQKLYVSKYATKARPLKLYLKPGRKISVRDAVLG
LIVKSANDAARVVAENIAGSERKFARLMTIRARQLGMKDTTFTNASGWHDPKQVTTAVDL
AKLSIAIKRDFPKYYRAFFSKTSFRFEGKRIKGHNKLTATYPGAEGLKTGFHTPAGCNLI
TTATRGNKSLVGIVTGRKNGTIRNRKMVQLLDKHFGVKHNTIKRKGGIKTRKMKLADRKK
RKSRRK

>gene_1225|GeneMark.hmm|808_aa|+|29063|31489 >NVVL01000054.1 Rickettsiales bacterium isolate NORP64 Contig_source1382A_23672, whole genome shotgun sequence
MTHSKVAQIALLTILFVYSCVASAEIPGLLDTEVSNGLSVEGDIFDFERRGSDDDDSDDY
DFSDDDFDDGFYHTNSNPPALPLHQGNWNTWRGILNFEGDIFRFEESSFRDNDNSDYDNI
QVAAGQTLQVVYDENLGIGYGIAEDVNLKPNNIKGRLHRLVDLRTGTRGVPRIGTRGIQG
AGNIIFEGKNRANCWLGSQEQSLGFIETRGDTSFVDEIYAQHMSFIGRTTMYSRDINTSS
IVLKNAEAAFLRGAYNPIEITGIGNVIYLRGSRLGKIIIQSTRGDFVENTISKDNNLSSS
GTLYLIAETGKETYEMTDNIIVSDYQTLGEGTVILNNNGYSLTIRGNDLAPLLVDHKSVS
ISAHGNTSQNIFQTTSQELAKEPSKEPTYKEPTYKERLAEAIRRSLTTEITDQEKRMASN
KIASEFMQLNLDTEEKVAEYSLTAEQVAELSPGAQEAAYYMGEATKSQQTLDDESATKSA
VLDANNKVASESSKVTLGAVSSRIGELFRSQHLANNTSGTSAGDDGFDIKKGVWVSGIFA
KNFQKSDENSSGYNSKITGVNIGADAQTTENILIGAFYSHVDALVKFNGSAKGSRLDAKS
NNWGFYTNISLTDNLSIQAIISAGFTKTNSQKKIGALIAFSKPRNKTYSTKLKLTYHKAL
NSSFAIIPKIGLKYSYSADAAYREIRSGVQDLMHAKKSSSNLSAILGTELLMQKRISDSI
TLLPSINASIHQTLSENRSKSKTIFAWGKDNFQFDHATRSKHSDRTTVNIGAGLLAKINN
IELSADYAVSLRRKYIGHQGIFKTRINF

>gene_1226|GeneMark.hmm|770_aa|-|31714|34026 >NVVL01000054.1 Rickettsiales bacterium isolate NORP64 Contig_source1382A_23672, whole genome shotgun sequence
MTVEKNTLPLLALRDVIIFPGIIAPVFIGREKSLKSLLCAKNTGDGQCILLVTQKDQASD
NPKIEDLYEVGVLAKIIQTIKLPNRNAKILVEAVSRVKLSNISGKDMFMADYVVLSDKGS
KDPKALEATLRTAMDSFRDYAKDSQKISTELLQILSEQKDPTYITNIISSHLGCKLPVKQ
GLLEETDILKRAEMLVEAITAENTYIHTENSVQKRLKKQIEKNQKDFYLTEQMKAIQNEM
TGDEGKSEFSDLENKIKKLNLPKEAKEKAMSELKKLKLMNGASAESSVVRNYIDTLCDMP
WGKKSPVKIDIKNTEDVLDKDHYGLEKVKERVVEYLAVLKRSDKIKGPILCLIGPPGVGK
TSLVKSIAKAMNRKYAKFALGGVRDEAEIRGHRRTYLGSMPGKIINLLKKSKVDNPVMLL
DEIDKMSSDFRGDPTSALLEVLDPEQNSHFTDHYLEIEYDLSDVVFIATANSYDLPRALL
DRMEVIDISGYIEEEKMQIAKNYLVAKQLENHAIKASELKITDETLLELIRYYTKESGVR
ALERSIGALARKVLTKILKNPDIKSVTIKPEDLEEYLGVKKYRFGLAEKSNQIGVTTGLA
YSEVGGDLLSIEAVSFAGKGDIKSTGTLGDVMKESAEAAHSCFLSRTESLGLKAKDYKDI

DIHLHVPAGATPKDGPSAGIAIYTTIVSLMTKKPVSKDVAMTGEITLRGKVLPIGGLKEK
LLAASRGGIKTVLIPEDNVKDLKDIPDNIKNQLKIIPVSTIEQVLENALV

>gene_1227|GeneMark.hmm|351_aa|+|34048|35103 >NVVL01000054.1 Rickettsiales bacterium isolate NORP64 Contig_source1382A_23672, whole genome shotgun sequence
LYIKSLINYIIAWHYVFKIDYKCNSNIICSNYKNKMTCTIQHPEIVAKAYARIKDYVHKT
PLWHSDSLNEKLGSQIYFKLDSMQKTGAFKIRGVLNNLLSLKELGKLPSKIVAYSTGNHA
IAMSYAAKLFDIKARVYLPKNVAPVKKRIAESYGAEIIEVATRKEAEQLSNRDGKAGYHY
LPPSDDDATIAGAGTMCYEALVDMIELGKKPDAIFAPCGGGGLLSGTYLAKELLSPNSEV
HGVEPKLANDAYLSLTSREIFKFKDAPETVADGLTTPSVPARTLHYLKKLDDFHLIEEEE
IRHWASRITEMTKVTCEPSAAISMAAAHSWIQTHGKDKVVLVLITGGNVDI

>gene_1228|GeneMark.hmm|30_aa|-|2|91 >NVVL01000055.1 Rickettsiales bacterium isolate NORP64 Contig_source1382A_23701, whole genome shotgun sequence
MATPQTALNSGQPTLVPDAMQATSMGDILA

>gene_1229|GeneMark.hmm|463_aa|-|122|1513 >NVVL01000055.1 Rickettsiales bacterium isolate NORP64 Contig_source1382A_23701, whole genome shotgun sequence
MAGQSLTSILPALGFLDAVVQDNTLAREFYDALFPELLYRAEAVPERWEANLGERMVFTR
SSLLSASTSPLTPGFDPAPESPAYEQWEVVAAQYGKSLDTNMPASRTALASLFARNAKTL
GLNAGQTLNRLARNKLFCNYVGGSTLTTAAGVAITALPVSSVNGFGTVIVNGQIVPVSGT
NPKSTTVSGVAGTVDVVAATPTNPLEPNGPGILTLAAVATWAIDAVVTASDAPTILRSGG
GTSVDSLTSTDILTVQNIRDAVATLRRNRVPPHEDGYYHCHLDPIAESQLFADNEFQRLL
QSLPDDFRYQRFAIGRLANCVFYSNNESPNSQNTNTLVATRGSAFASPSLGTEVINDAGV
AIIRTIITGGSAIMEKWVDETAEYASEAGYQGKVGAFSVVNNGIVVPIERARYIIRAPQD
RLQQIVSQSWSATLDFGVPADLLSGLTGARFKRAVTIESGSAL

>gene_1230|GeneMark.hmm|186_aa|-|1532|2092 >NVVL01000055.1 Rickettsiales bacterium isolate NORP64 Contig_source1382A_23701, whole genome shotgun sequence
LGDLMKDPVIARELAAATKRQVDAAVDAREKELTQAQERSKMEETERLRAEKADEEGKRI
KAENEASDLKIDSEFATCFFKSGVTLANPQSIDFIKAEARRTMDAEGLSMDACVADVLSK
HQYLVTQPQAPAAVAQQLPEVLATTVPPAQARHTETPAAPAPQIVVDTRGMTSQEYRKYR
QETHGF

>gene_1231|GeneMark.hmm|90_aa|+|2094|2366 >NVVL01000055.1 Rickettsiales bacterium isolate NORP64 Contig_source1382A_23701, whole genome shotgun sequence
VSGGSSGAVVGAGAATSATTGGWGCAGAATSTGGTSATGGGATGASGGASGLGFGVGVPG
TDMNGGYHPHWSGRNATYKLSAPVITPLGS

>gene_1232|GeneMark.hmm|141_aa|-|2638|3063 >NVVL01000055.1 Rickettsiales bacterium isolate NORP64 Contig_source1382A_23701, whole genome shotgun sequence
MAGITVTVDGTLSLQQADQVPGCPPPGADLITFPLALFEQGLTGIAGASGSEISQGCFTI
NSAASFVTLAYPASLRARIFYLRALPGSGALDVRLTHSVAGAVTYPDQYLMLVQFSAVEQ
LTAIEVQGVGSFAWAAVGVLV

>gene_1233|GeneMark.hmm|335_aa|-|3256|4263 >NVVL01000055.1 Rickettsiales bacterium isolate NORP64 Contig_source1382A_23701, whole genome shotgun sequence
MCSVVADCPTLGRLEKLDNVRLHTMHEFDHLLVDGRNMLYRAAWSNQMLTHRSLPTGGVF
GFLRTLLAAYRDFGCPTIQVCWEGKRNWRFKRFPAYKGNRSRGPRDDTGFDCGAAVASQQ
PLLRELLALAGIAQWRTPDGECDDVLYARGEELAADGETVAVLSHDRDMYQVTTNPLCSV
IRTDKDGDWHVVDRTVCEQELGGPPEKIPALKALAGDSSDNIPGVRGIGPKIAAKLMVPH
TSLSELLSSARSGERWAGSARQRSQVHDAAEDVSIFEEIVRLQMCELELSPEHHYPEKVD
LQAALEDLGILTLAEEGPLEALCSMGGTQLFGEKH

>gene_1234|GeneMark.hmm|67_aa|-|4559|4762 >NVVL01000055.1 Rickettsiales bacterium isolate NORP64 Contig_source1382A_23701, whole genome shotgun sequence
MFPKNTPKPPRLTVLTKTTLRLTSAEVRLALILAAERVLGHTIDSDASVSLYEEGATIQT
ETEDDGA

>gene_1235|GeneMark.hmm|172_aa|-|4780|5298 >NVVL01000055.1 Rickettsiales bacterium isolate NORP64 Contig_source1382A_23701, whole genome shotgun sequence
MRLVGICGYSGSGKSIAAAALRQDGFHVYPMAQVLKNMLRAFGLSEAQVCGDQKAVELDL
LGGKTPRHAMQTLGTEWGRDLISHDVWLKAWWTKVKHIPAVVCDDVRFGNEAEFIRAQGG
RIIKIHRPLGSGALPQHPSEQLAFRPDIEIVNVGTVPELEHKVRQALVNAGV

>gene_1236|GeneMark.hmm|125_aa|-|5286|5663 >NVVL01000055.1 Rickettsiales bacterium isolate NORP64 Contig_source1382A_23701, whole genome shotgun sequence
VNAEFLPKYLALVREHNKLHNPQPGEQHTRLLRSILGCAAESGELASAAMQSISNNCDID
PLEALDEFGDVLFNLVRGLDALGFDLELAMMANSSKLISRALRGKNKPSERRVLENLVSE
VACDS

>gene_1237|GeneMark.hmm|227_aa|-|5683|6366 >NVVL01000055.1 Rickettsiales bacterium isolate NORP64 Contig_source1382A_23701, whole genome shotgun sequence
VRSPFKLAAFLKYAGGKWATSHRYPKPLSRTIVEPFAGSAGYSVTHSNRRVVLSDTDERI
VGAWDWLIKARASDVAALPLLAQDERVSDLSLPQEAKWFLSYRVNIAAGPRNTATRWRMW
NARVRRRIALQVHRIDHWRVYHRGYADADEGPATYFVDPPYQEKSFYREGVADYAALGAW
VRSLSGQIIVCEGPGASWLPFRKLLDAKTHVNASAAKVPELIWYRCQ

>gene_1238|GeneMark.hmm|212_aa|-|6363|7001 >NVVL01000055.1 Rickettsiales bacterium isolate NORP64 Contig_source1382A_23701, whole genome shotgun sequence
MPRKSKPTTFDYIVPQLQSLAIPIADLEFDTKNTRRHGKKNVEGLRASLQRFKQYLPIIV
QEEGMVIRIGNCRTQAALDLGWTHIAAVVVPESGVDAVARAITDNRLGELGEWDWAVLTE
SLQEQELGLDELGWDPEELSALLETDFSVPEVDLDDEPPQDSSAPGESSELGAGSSDKQP
TLTLGVEASKVLRASAGRADLSPDAYVLSELA

>gene_1239|GeneMark.hmm|84_aa|-|6985|7239 >NVVL01000055.1 Rickettsiales bacterium isolate NORP64 Contig_source1382A_23701, whole genome shotgun sequence
MPHDNLKTKWPPGTSPELQLQQLFSALVTRIGLTNIKGVAVFVRYVGDDNDGGVSTQNAV
FTAGAEVDSVYDWMKEVTRDATQE

>gene_1240|GeneMark.hmm|94_aa|-|7221|7505 >NVVL01000055.1 Rickettsiales bacterium isolate NORP64 Contig_source1382A_23701, whole genome shotgun sequence

LITDPDFPGYPVTVIEYVDATELPNPDCVWAFHVLVLTRGWASTLTAHAVKAADDVTPQQ
PERACVLLQAAERIGRAVQDIEAAKGYVPCPTTT

>gene_1241|GeneMark.hmm|248_aa|-|7495|8241 >NVVL01000055.1 Rickettsiales bacterium isolate NORP64 Contig_source1382A_23701, whole genome shotgun sequence
VGCDIHLNLVKKVGDWELLSPPLNEESKALYPNHSDYWHAWPCDQRSYDCFSLMTDGQVR
RSDDPVLRGFTAPRGWAGVEAQFGLIPPDPDDPSWRGWEVGDHSFSYLTLGDFTSFRSAG
GWERFTQARNCTCTAAGVEAWWAADKTPSPWSYDHRLPTGVPQRGRVAEEFIRKYIDTTS
RQQRAVDNTQARLEGYTGGHLDRYVVVVTVSETVASRCSGLLELSELMAQYANEPGTDDV
LLVFSFDN

>gene_1242|GeneMark.hmm|76_aa|-|8587|8817 >NVVL01000055.1 Rickettsiales bacterium isolate NORP64 Contig_source1382A_23701, whole genome shotgun sequence
MTTTQTDMLDTLLPGYVFWYAPHVPHPYRWAVAQVPNGYEKRHGSHHGGASTQALALVAA
ETHASVVAADGFLHPA

>gene_1243|GeneMark.hmm|69_aa|-|8830|9039 >NVVL01000055.1 Rickettsiales bacterium isolate NORP64 Contig_source1382A_23701, whole genome shotgun sequence
MGLGIVGALIGEAIEQLGDLSRAALAAALRDVAGKVERGELVSDDEIEALRGSTGRIREM
LKRAGNNSS

>gene_1244|GeneMark.hmm|133_aa|-|9040|9441 >NVVL01000055.1 Rickettsiales bacterium isolate NORP64 Contig_source1382A_23701, whole genome shotgun sequence
MNHALHIVFALVAFTGCAGTQRSHAIRVGHIAKEVVDAGAVAVKAHIDARASACAPRVDD
GTKALTDCLGPIASHPDEVDGAFAAARAAQLGVYLAVSTDASPAEIKQARNDLEAALRAV
VALIQSVAAQEAE

>gene_1245|GeneMark.hmm|186_aa|-|9480|10040 >NVVL01000055.1 Rickettsiales bacterium isolate NORP64 Contig_source1382A_23701, whole genome shotgun sequence
MITPTIFGCDPALTTGFGLIDAAGDIATFGHWCNKAPHEGRKAEDRPRRDQIRAHTFAQQ
LRRILDGYPTIERLAYEWVWKHTSTAQSQLYAGWRALLLAECHARGIDVWPVAWSTLKSV
AYEYAEPSEIAQVKATKVKAERRLASKALIIVAAKRRWTNAPFTEDEADGLWVSEACRLG
RGGGKA

>gene_1246|GeneMark.hmm|536_aa|-|10037|11647 >NVVL01000055.1 Rickettsiales bacterium isolate NORP64 Contig_source1382A_23701, whole genome shotgun sequence
MRVKNLIVQGLTTHKATDIALPSQGLVVITGDNGSGKSSLVEAVSLCRFGKSLRGTDLWA
GPGELVTETYDGVTYRRKRTASGRMTFKTDGGPEYQTATRAQRELGGDHKLWMRSCVLSF
DDVVSFTRATDSERKKMLEGVLGLQRFDAALQQVRGELVTARSAHQLATHAVDVAQQQEH
AAAGVLSILQAQLGEGAPTSDPEVVARNVTSAVVDAEEARDDEEVTREQATKCREQSSAL
GARIALLGNQIGAEGDAECHACGRAFDNHRPDTIAELVEQRRELTAMRDEVIMEGKKWLS
DAEKHQKNAEVSNRKAECARDDLRAHGEYDARCSRLQTKIDEASVKNNDAGQHVATLRVA
EDSACADTSVLEAAEQVLGLRGFRSHVLAEALEGLEISANYWLERFSPGVQVRVRPTREL
KGGGTSDEISVEFTGVGGGHGYKGCSRGERRRQDVAITWALGELEAAASGHAESTVWCDE
PFDALDTQGVDALVSALTEVRERRCVVVISHSADLVEALRPDMHYHVEDGVVHRRR

>gene_1247|GeneMark.hmm|138_aa|-|11595|12011 >NVVL01000055.1 Rickettsiales bacterium isolate NORP64 Contig_source1382A_23701, whole genome shotgun sequence
MSTDQERLNHLAKVVVRQGERINQAIEQWVTQREAAAPHSDKAELNGRIQAAMALSNGLR
DDLESLMSISDVYVIAGKEVPEASLAGMSELDRTRRKLRITMAEAEMWQSKATELAERGG
RDESQKSDSPGSDDTQSN

>gene_1248|GeneMark.hmm|901_aa|-|12008|14713 >NVVL01000055.1 Rickettsiales bacterium isolate NORP64 Contig_source1382A_23701, whole genome shotgun sequence
MPETRLKLYNPSTSDPEDLLHEEERVERSSACARCDLHSKAQLVGASADGQAGGLLVLGE
YPHADEAKYCKGRPFWSKSGKYVRRQVAAHWSGPVVYDNAVRCCPQKVPTDTQTRKAADA
CRTFTAGILDSSQPERVLIMGQSGLQTLFGRGTYPRVRRGYQWLRFNGRAIPVFFLPKLN
PTFGNRLLMRNWQEDLKWALTATPEQPPWETSTTMVRTEEEARAAIEDLADSRCAIVDVE
TYGDPYSDDFVVLCISAGRLDNDTTYTWSTRRKDTEAIGPLLELLRNPSVGLGGHNFKFD
VVALEQYFGIECEGKMVDTKLLHRMLDCTQQADLNACSDQVGCGGHKAEAHFHVEAAVKL
IRKHRRRVKKDLGPDCLYPARLDLATGLVAAEPTIPDRDRAMFAHAVAMWDHHPKKYAYA
FMPRDVLHRYCARDVMATGRVWRGLWRELCGTPDVRLAWEALVQPATRTFSRIERNGIYV
DVPALDAFRDFLAVRETQTAIEISVHSSKYFPEGFNVRSAPQLQKLLFEDLQLPVVKRTP
KGSPSSDAEVLKELKGHHPIVPLLLEHRTITKLQGTYGDGLAPHVASDSRIHCNFNITGA
ATGRTSCTDPNMQTIPTKGEYAKQIKNVFAAPFGYKLVQFDFSQLELRVAAMLSKDPVMR
QIYVEGRDYHDETAAMIAPTAWDVVFEKATDEMWQTDPVRAQLMDDIRRAAKVVNFGLLY
GMGDVTLAKATKTSVEQAMAMRKAIYGKLRVLEAWIKQCVSTVRQRGYSSTWWLGKPARR
RYLWDIAMEGASDHASKARSNAENGAYNSPVQGTASDYCMASLNAIDAYLRDEGIDGKIV
MTVHDSIILEIADAQLREAVPAIERLMTQWYADGVPLTVDCEVGQTWGALKDWDTWLHEA
A

>gene_1249|GeneMark.hmm|396_aa|-|14706|15896 >NVVL01000055.1 Rickettsiales bacterium isolate NORP64 Contig_source1382A_23701, whole genome shotgun sequence
VKVAIIADVHVGNHRLFGGPNIGGLNERARDTVRVLGAAVQSAWRDGADKIIIAGDLFDT
DTPTPAVIAAVQLAITWSAAWDHKARMVLLNGNHDMTSTAPLHTALAPLQCMRGAIVVCE
PCSFHDADQGDVVLVPYQPGPACEWLPEAVEKALTTLSPTLTDYNDTSLAGTPRSRLLVL
HLGLEHKGTPPWLRGADDSIHIDRLRKICLEHSISNVVAGNWHGAWSENHGGITMRQVGA
LVPTGFDNPGVEPYGRVVWWTTQEGFTSDTVMKGPRFMKFTDIKELTAFETDVRTDPKYG
HDFYVHAQLPAHEWWGGKLILDRLSVESLIRGFKTSKQPAEVRARAAQAVGSVADLSSLG
AALFDYIAETPLPEGADLARVHQHCRRLLKLEAEDA

>gene_1250|GeneMark.hmm|332_aa|-|15893|16891 >NVVL01000055.1 Rickettsiales bacterium isolate NORP64 Contig_source1382A_23701, whole genome shotgun sequence
MKDMVGALNAGKDAPGASVLSKGAFRSEPSGFIPTGIEEVDKYVLGCGGLPLGRISEVYG
APGAGKSSFVYSCLRQCQLMGGIPVLIHTEEAAQKSRLVDTFHMDPDNILICEPRDMEGV
VDALQRVLQPILKSRAKNKPPVLVCWDSLAATKVRGHDEASKGEGPAKRARILNAALRVL
PQIIAEVQGHFMIVNQNREKVGVMFGSPKTQPGGKAPKYVSTFRLELTYVGKVGVPAHPT
GIEVKIEATKNKMAPPYRKLTSRFDFVKGWQDWGVLKLAKELKLVPATARSVDDARAKLD
EISWDPNAPVPEEKLGPAPDEVWDEEVEEESS

>gene_1251|GeneMark.hmm|300_aa|-|17052|17954 >NVVL01000055.1 Rickettsiales bacterium isolate NORP64 Contig_source1382A_23701, whole genome shotgun sequence
VLRELALFAGAGGGLLGSKLSGHRTVCAVELDPYCRRVLLQRQHDGWLDRFPIWDDVRTF

DGRPWGGRCDIVSGGFPCQDISSAGSGEGLAGERSGLWFDMLRIIGEVRPRFVFAENSPR
LRTLGLGTVIKGLASLGYDARWCVLGARHVGAPHRRDRMWILAYAQGERPQEGREVQRDR
EVQNLGCDGEAWLANANDTGQRVRAIDGETLRPLPPVARLGSEAVANTHSEILREQSGRR
SGASTAVDRGALQTGWTGPAPIARMDDGMAHRVDRTKATGNGQVPAVAALAWATLSAGLV

>gene_1252|GeneMark.hmm|73_aa|+|18047|18268	>NVVL01000055.1 Rickettsiales bacterium isolate NORP64 Contig_source1382A_23701, whole genome shotgun sequence
MQETRTITVELPLVVVVELEVTLDPDGFEATVHAVRQSTGAPNWSPCDVNESSVDVLEEI
DRLAREAFAKPES

>gene_1253|GeneMark.hmm|299_aa|-|18265|19164	>NVVL01000055.1 Rickettsiales bacterium isolate NORP64 Contig_source1382A_23701, whole genome shotgun sequence
MNALKNYADGWSEADVNKEVEDTKQEKSDESDFLAIAPDTELSLRAMPNAKGGSPFVKFA
KHFYQDPTTGKYVGFACPAKSRLAGFQQYACPTCEASARMRKSRIKADRDFGYKMKAKWR
ALINVYVRPNDAVVQAAMDDGVDPPKGQMKLWEISSWVGEKNGKNMHERLLALRNNPRVG
GNYVDPSAAGFDLLIQRQGENINTRYEVHALLSERKPLLPDPAAAQALIEDGQLDIAWYI
APPTLEELSQILQGEKRGGKSQAVATGASSNPQLPAGGGETAGEVIDVEDVGGDTGAGF

>gene_1254|GeneMark.hmm|167_aa|-|19161|19664	>NVVL01000055.1 Rickettsiales bacterium isolate NORP64 Contig_source1382A_23701, whole genome shotgun sequence
VPTIHDWQVEAYLHESVSLDRTDLNREFARVSSDLAYWGEKYAQAERCHAEAKAEHEQVQ
ARLYRQVRAVLEADAAAKASAAPTKKAPARVTDSHVESEVVLTADYAVAYQEVIDCESAK
TRVRMIIEAVKTKRDMLVSLGAQLRAELRGEPHIKDPDWDDWKKTTP

>gene_1255|GeneMark.hmm|326_aa|-|19668|20648	>NVVL01000055.1 Rickettsiales bacterium isolate NORP64 Contig_source1382A_23701, whole genome shotgun sequence
MKFNYDSDTGELCLEALLAPSQAATIFEMLSTMGAAKDPMSDALRPAGTPPTQRQRQMED
AADLAGGQLTVTHACGGEPVLVSKADEPLGVPATAAPVPKVPRRTKKKPPTNKPQAKPKS
KAVEETPPETPQAAPEKTPAKLAAEDEDRRAEHAQRWRGKNLFKDELVVGFAVIGADYTV
VLELESGEKVWLDDSDNEIKRVAPTSGLPEHESAAGDFRTGDTADAPPQAEPTTQPTAPP
ADMPQGVARAEKVRAIVQPFVDQAGGGRGKEFALGVVNAVVKCSPHIAKFEGMEASEVRK
RVVQVLAVLAVINHEELGEMKAEMGV

>gene_1256|GeneMark.hmm|316_aa|-|20660|21610	>NVVL01000055.1 Rickettsiales bacterium isolate NORP64 Contig_source1382A_23701, whole genome shotgun sequence
VTQGFRDGVKGNYREQLAAVQAAFEEQYDGGTGGEDARVRCPLCIDRTGKEDLRGCLSVN
LDNGFYNCFKCEAKGRLEGYSEWDYRDREPVVDRPVMKLPYDFVPLIKDYEHSILYRASA
LYAFSRVRDWQTIQDIGVGLAGGRLMVPVRNNGQHLGHVARSIPGQVPAYLPDTKYVYPK
GFKREHLLFNEDALDVPRSEYLMGEHKGLPVVVVEGVFDALPHWPLAVACLGKPTHAQER
ILERCQRPVVVMLDGDAWELGKSLAQRLFKLGIEASWVKLPPGQDPGNYDIKVMRRVLTT
AAGKPGQGFLIRTDDT

>gene_1257|GeneMark.hmm|464_aa|-|21607|23001	>NVVL01000055.1 Rickettsiales bacterium isolate NORP64 Contig_source1382A_23701, whole genome shotgun sequence
LVVSRAGMQDANASHLSVVPIGHTVRPYGLPKPLERSTVHWCTRSEDFWYSVGHLVEPAG
LGEACARTVLEHCIALHRADTVPTDAGVLSSMVMLRDRGRLTQEVMEDTVDYLDLIEGSA
MAEEATLQAIAPVLRRRKESEVVDALIKAHAAHQPLDDLVDALQTVQLIGTTDAHPGMFL

DQDVFARIDRLRHVERMSLGMKELDIAVEGGLGRSQLVVFMGKAGDGKSMALINLAAKAI
CSGMNVAMASLELEEEVWTARLLANLTGVPTNDILKGSWRDASQRLRELQEGVGLGRFAV
KWFPPRSTKWSEVKAWYARVREEMGGLDLFGSDYADKILSDGRHDGTYGAMGEVYEEHRL
AAVEWGCWAATASQKKSGVKGKADVDDASDSANKGRVTDMMIDLEALEDGNEMIARVIKN
RHGPSRMEIGPFVTDWARARISASDVHAGSHAIQAAQPDFGAGW

>gene_1258|GeneMark.hmm|254_aa|-|22995|23759 >NVVL01000055.1 Rickettsiales bacterium isolate NORP64 Contig_source1382A_23701, whole genome shotgun sequence
LALTTPPRAAVSGSERQARPRSTSADWDQLKAALKSPSEPIRPVSAELFYREVPKLRAED
LRLAQVPRPPKISLGTVSNTHAVECALWLKAWIYQHYGTAIRWTRRTRFDKRRNWKAMAD
GIVLLQKHHISAPRWISFVSQMPVVTESVTAPSCAQIFSRGIVTSQLDYCRRASTELGGR
TVMTPAARKLAHDHSMMVQDLRLTRASEPPAIRAIVARYFPGGSFARRVATVAAEADHER
EGMSKALTEVTWIW

>gene_1259|GeneMark.hmm|621_aa|-|23963|25828 >NVVL01000055.1 Rickettsiales bacterium isolate NORP64 Contig_source1382A_23701, whole genome shotgun sequence
MWLRVNNLYTTIAKAEVQEIQWLWNFLSYEDSARERAMGVGTVRLLSKRHRRFPTGLVPL
VEKHAKAAGFDVKRIDTRQRPHPRNWDEHLADAELGWLKTRLLDGRPYQFDAIVAAIKSG
RGIIRAPTGCHARGQKILMYDGSPRAVEDVRPGEYLMGPDGEPRRVAHLCRGRQKMVRVV
PKKGDPWVVNLDHVLTLVRTNDDTSLAGKLVDVRVRDWLDWSRTKKHVHKLVRCAGLPRK
SSVPGVFTEDLNQFGFTVEHLPEDDYFGFTLDGDGRYLLDDFTITHNSGKGEMVVGICKA
IPCKWLFLVHRSHLAEDIQQRWEARGDGSGHTWLGRGNWELGDRVSFCTFQTLYRALSNP
ECRNALQHVQGVVIDETHVLPAGSFMEVSEAMPNAYYRIGLSGTPLDRGDGKSLMAVAQT
GSVVYNVTPRSLMDAGFVARATVRVTEVKQKKGLASRTWVAVRKAYIAESAVRNAAVLRD
VLACQKPAMVFVDLLSHGRILRDAIEQAGLTVEFAWGAASKANRAELVQRVRDGDLDVLV
ANGVFREGVDIPSLASVVYACGGKSTIQVIQGAGRGSRVTANKTEFTIYDYLDRGHRWLE
GHSDARVRIYTRYGYSVLSST

>gene_1260|GeneMark.hmm|61_aa|-|26082|26267 >NVVL01000055.1 Rickettsiales bacterium isolate NORP64 Contig_source1382A_23701, whole genome shotgun sequence
VSIAAQMAAQGYLTISGAADRAGVPYHTMRNWMFKGLVASKKKAGKSYISLTSIEVFLAE
E

>gene_1261|GeneMark.hmm|223_aa|-|26264|26935 >NVVL01000055.1 Rickettsiales bacterium isolate NORP64 Contig_source1382A_23701, whole genome shotgun sequence
MTVDHITASSLPRLVTPLNTIRDAPRNSNVMPVHKYAALKKFIARNGLAQEIVVYREDPT
SSEWVITDGHHRVRALRELWDPSSPVPCLDASGLTTVERIELTLSLNNNRGQLDLSLVST
EIDFLLEAGRELVDLDVTGFTIDELEALSGATLELDEASLLDDAMATEVARDKPDKVFVL
EVTLATARELKLAKGRLRRAGGKGNRDLAKGFRAIAGLDGDDQ

>gene_1262|GeneMark.hmm|211_aa|-|26925|27560 >NVVL01000055.1 Rickettsiales bacterium isolate NORP64 Contig_source1382A_23701, whole genome shotgun sequence
VARVMTAIHIAAVGSVVRDIAEKFAIRSHRGAQGVEDLVQEGWLHALPAYQSYWVKCPEQ
SVKGLRGWVYRVVYVRVSMYMWKTGIPVSIPKKAIPPQVRYGRVRGTRLLSEDHPDWQSG
GVDTDRCGIEEEYARTQRRDAVDVAMGRVLMDYPLARLPLKQALSVRVAAKLLGKPELLP
ELRRQTAEARKALRRELEPYREPYRKTFNDC

>gene_1263|GeneMark.hmm|286_aa|-|27561|28421 >NVVL01000055.1 Rickettsiales bacterium isolate NORP64 Contig_source1382A_23701, whole genome shotgun sequence
MTDIHPSTTVRNDVSAVSDGQSTGCIVYWSLSGRVPATELLTALEDMADIPPESGPKLAG
EREMLARAVKIVAGRRREVLLDSCRGDFQLVYREEPDGGASTPVYSGKLRVHLGDDGEVV
YSDSAHPLCAEISNEYSARRGCFDTREISVWLTELLDKSHAVRMRPRGGFYYLPGARVLQ
FRAAVMALRACSEHVVYEIPAMSSRETAEAIISALRHEVEAATQRAQELMEGSPGKRARR
THLAELVKVKIKLGAYAEFMDGTLDGVITALDNASAVVSQTLLEGE

>gene_1264|GeneMark.hmm|410_aa|-|28459|29691 >NVVL01000055.1 Rickettsiales bacterium isolate NORP64 Contig_source1382A_23701, whole genome shotgun sequence
VGPTPPQSKKTMANQSSIVKASLFTPGLNGRWGLPLIAWGEPGVAKSGMIRDIAHECGLG
FNSTIASICEPADLAGYPIPNEERTMVNALPPPWIHKVNAMSGAVQLFDEATSCSNAAHA
ALMRITLEGKAGDVDVNPRVRFMMAANPVDCAANAQELAIPLANRFGHLQWDPGDARAWV
EWLLSGSEDNSPPVMSALAREKLVMEAWPEAFADAKGLVGAFISERGSVLHKMPLVDAGE
SSLAWPSRRTWEMATRAYAGGLVNGLTPAERHLFVTAFIGQGPAGEFIEFVKNVDLPKAL
DVLAGKVKFTHAPVRLDRTMAVLSSCAAYILSTHTEEGSPEHDQWLSHVDAYWALCLHVT
EAKAAELIIPSAKMMVLKRWDTRLFAGNKSNAFAVMSRIEPLITKVIKVA

>gene_1265|GeneMark.hmm|456_aa|-|30375|31745 >NVVL01000055.1 Rickettsiales bacterium isolate NORP64 Contig_source1382A_23701, whole genome shotgun sequence
MARRSRSRQDTSGPGFKMSVARAIVAEKAPYFGTVLYSMYPVKVDKIRRKVRKGGQLCVE
ESDTFGVTEKGLLIYTEKALNDWSEDNLVFVLAHETMHIMLEHPSRAATMGYDAALYNIA
ADIFINDQLRQGNWTDWPATICWPETWGFDSGLLADEYYHLLLQKRAEQQEKKDQRSAEG
PDSKPGEVDGEPDLTEMGEALNGDAFGHCGSGAGNPLENEGDLTEEARAAERSPAEMEAI
RQQAAEELADHIRRHPGTIPGGLERWAAAKLGPAKVSWREHLRRASRRAVQWVAGAQDYR
YGSVHRRQAGFGWGPRSIVMPTMVAPVPTVLLAVDTSGSMGADELGIALREADGLFKSMK
CQITVVSCDAEINELVECRNWKEAAKSLKGGGGTSFHPIFEMVDRMNPKPDIVVVATDGG
GPAPASAPRGYKVIWLLTGEYSQSPCDWGFHIKVGD

>gene_1266|GeneMark.hmm|98_aa|-|31745|32041 >NVVL01000055.1 Rickettsiales bacterium isolate NORP64 Contig_source1382A_23701, whole genome shotgun sequence
VPTVQHWLQMVTHTQAPPHSDAGRFDTTIGSETEAERIGKLVGARMLDGDEATKSILLEA
ARSQGLSIPSVMHTARYTVRVARIQAARARARVRLGGA

>gene_1267|GeneMark.hmm|78_aa|+|32113|32349 >NVVL01000055.1 Rickettsiales bacterium isolate NORP64 Contig_source1382A_23701, whole genome shotgun sequence
MDKKTPDEILLEARESLAPGYATDLLLAFVEYLPENRASHKELEPTDAGDLFRTLVLEEN
WSVKKARAVYDAAYEVVS

>gene_1268|GeneMark.hmm|289_aa|-|32467|33336 >NVVL01000055.1 Rickettsiales bacterium isolate NORP64 Contig_source1382A_23701, whole genome shotgun sequence
MVTHPTPDELLEAQHEHLAALSDKAARAARRAVARAMADLRRGIDSIPGADYGGWTTATR
QAMEIQLRAAMAQLTAGTAQGMATVHVHAVKRSYVDAKRWLGSLDRKFIGVAKPLRFDLA
AHVADENRFLIDRYKRSLSYWGQQTTNVMRELLADQALVGQNWADAAKHLKRKIPALAGK
KLWMVERVAATEMANAYNGTAWRVLMEQDKPHDRMSKKLVATFDKRTARDSKALNGQTVL
VDLPFINVDGRGYMHPPNRPHDREVVVGWRKSWGNHDDLLGEIASLDVT

>gene_1269|GeneMark.hmm|45_aa|-|33336|33473 >NVVL01000055.1 Rickettsiales bacterium isolate NORP64 Contig_source1382A_23701, whole genome shotgun sequence
SAKPPATGTVKTKKKPSPKPAGKKKAPGKGSSAQGSSEGGTSGDS

>gene_1270|GeneMark.hmm|362_aa|+|1|1089 >NVVL01000056.1 Rickettsiales bacterium isolate NORP64 Contig_source1382A_23747, whole genome shotgun sequence
NMKLISFLFTVLVNLNIAYGFDISKDILDKLDRAIHNNPNDIAAYNHAATELNDSHQFKE
SIKVCQKALKIDSKDAFCYMEISRSYLELGKKSEALKMVEKSIKLDPNNIFALYQKIFVL
DSIKPINKKELVKLGYKILEKNSEDKLAYIVLIKSLAGLGKKREMISAAEKYISYHASVV
NYLNLANAYAEIGKPDEYLLTIEKAINLDQSDLRPLWFKIQALFHLKRYVEVIPVSNKFL
KLQPDNTGVLAFKADALATLARYEEALNTYNIAIETKPKNPTALYLCKGLMLHKLGRLRE
SVQMYNKVLRLDPKNVSAYNNLGFVLSDMGQNKAAMEAYEKGLKIDPNNQLIQANKKILE
SK

>gene_1271|GeneMark.hmm|456_aa|-|1181|2551 >NVVL01000056.1 Rickettsiales bacterium isolate NORP64 Contig_source1382A_23747, whole genome shotgun sequence
MDTIFAQCSARGKAGVAVFRISGEKSIESLEKLIGAKTSKLKARQLYFRKIYHPVTKQLI
DEAMVVFFSGNSSFTGEDSVEIHTHGSLAVIKMLNEVLESMPDLRLAEGGEFSRRSFLNG
KMDLTAAEGLADLIDAETELQHKQAINQLGGGLEALYEQWRAELLALISLLEAYIDFPDE
DIPEETLQQVKKSVENLINKIKAHLDDNNRGERLRSGLKLAILGKPNVGKSSLLNYLMRR
DVAIISNIAGTTRDVIEGHLDIGGYPIILQDTAGIHDGSSDLIEIEGIERAKKISQNSDI
KIIMYDIAETAGLRALEEKDNVDYFSQLVDENTIILLNKTDISEINETTTPSQIHGKTPI
KVSVKKNMGMENLMNSIISIAEKIAKPSDSPQITRARHRVQLEQALESLTRFSLNDDLVL
ATEDIRISIRALCGITGKITVDEILGEIFSNFCIGK

>gene_1272|GeneMark.hmm|1603_aa|+|2645|7456 >NVVL01000056.1 Rickettsiales bacterium isolate NORP64 Contig_source1382A_23747, whole genome shotgun sequence
MKKNSNVKKSLYLSQLNCTIADISSEILDLVKGDKKGGIYTKFVRNFLKYIPIDYRSKDK
IDLFGDFTHEAFEFFKNKPAGEHKVKIFPNKYRNDPAITILIAAENRPFIIDSLNSLMSK
LALQAVFTFHPVIYTVRDDKGKLTDILGKREHGTAESLVYIKALGTFDKAAIDKITEEIN
VIIGLVNYTYNSWQPLLNKIISITTDVVHSTDIYESEDLPVEETLDFLNWLQKNNITFLG
TVDFDVESKALTNEDGVRDIWKDNLEEISTIIEFSKSEYYEKKLVMLGKINKLSPVHRNA
LVDYILVKRVDKDGVYRSGTIIFGLYGTAIYFQSIKSIPILRGKMNYVLDKSEFPVNGFN
SKKIKNIIESLPRDILIQIDEEDLYCMCIHMLSSMRSHRLKVFVQQDWSNSFINVIIFLP
REKLTPEVYNEISCYLKEKFGSEIIADNITVVAQDFSHLFATLAIKDILKLDFSHEEMEK
DLIQITTNWSESLLDRLCCELGEYDGASRHKDVAQSFPAEYRHKFDAGMTIDDIHHLEKA
SRQNELTFNLIQGEDNEYVIKFYSPEIKLTLSDTLPAIENLGFIAVDEQSFAIAKSSSFK
KSWIYEFKLSSPIKIGTPFDKLKENIEEALCKMSSGELASDVLSKLLVVSGFNWSKVKLI
KSLTRYLHQTGFIYGKGYVQKTLVSHHIFTEMLADLFEARFCPKAHSEKNVKSLSAKMGK
YLDEVSSSTEDKVLKNMMLIIEAITRTNFYQNSVCQDNEKMAKSYLSFKFDSSKVPDLPL
PIPYAEIFVYSNDFEGIHLRGGKVARGGLRWSDRGEDYRTEVLGLMKSQMTKNTVIVPVG
SKGTFYLSFAQGNMSREVYMKKVVACYQNFLRGLLDITDNLVNGKIMQPKDTIIYDAENP
YLVVAADKGTASFSDYANAVSAEYNFWLGDAFASGGSLGYDHKRMGITAKGAWISAQLHF
MEMGVDIQKEQFSVVAIGDMSGDVFGNGMLLSKYTNLVAAFNHQHIFIDPTPDHKTSYKE
RQRLFAMVRSSWTDYNLKLISKGGGIFSRSEKTINISAEIKELLGIKCSSLTPDELIRSI
LKAETDLIWNGGIGTYIKSSSENNIDIGDKANDNLRVNGNEVRAKVISEGGNLGLSQLGR

IEYALSGGRINTDFIDNSAGVDCSDHEVNIKITLNEAVASGKITTGQRNKILEQMTDGVE
ELVLADNHSQNLALTIAEHSPLMNIESFSQLIKEMEKAKLLDAENEFLPNKSELSRRSIA
GEGMTRAELAVLLSYSKMSVDTSLATSSLADDAYFQKYLFEYFPQVMREKFSKEIEAHPL
RQEIIRTVITNKMVNQLGGTEISSIKRETGASLCDIARAHSVVVEIFDLDNLWEKIAKLG
TSVDVGIKVEMFSDLGKIMRRGISWFVRNIKAPINVNNTIKEFSKQTADLTEIISKLLVG
TTKTRFFSRIEHYKHSCVDEEFARSVATLEVLVSAFDIIHIATTTENKNKDIANLYFESG
DVLSIDWLRQSCEAQVNESYWNRLSIQSLKDDFYDKQRRLVIEIASQAKPSASLESWLEE
HSTYASIFINFVEDIKMQETVDLNMIILANKKLEIFLRKLGAS

>gene_1273|GeneMark.hmm|130_aa|+|7456|7848 >NVVL01000056.1 Rickettsiales bacterium isolate NORP64 Contig_source1382A_23747, whole genome shotgun sequence
MTLSLEEARKKAGYSIADVAKKLKIRKQYILCLEKDDFDSLPGDVYIQGYKQIYYKFLGI
DLPKKKPISVLPPVAIYETNSINKGYIVFFAACMLGLVVLAYSMLNKSPLDEEEVVDENA
ISVHNIAMPK

>gene_1274|GeneMark.hmm|77_aa|+|7845|8078 >NVVL01000056.1 Rickettsiales bacterium isolate NORP64 Contig_source1382A_23747, whole genome shotgun sequence
MSEITKQDLIEIIHDISHKIDELVAKSAKNEKQIEDLKSQNKDLKQNNQDTLEQIKEYIQ
ELEQIRNHYVDSNNNSE

>gene_1275|GeneMark.hmm|102_aa|+|8050|8358 >NVVL01000056.1 Rickettsiales bacterium isolate NORP64 Contig_source1382A_23747, whole genome shotgun sequence
MSIVTITLNNKTFKLSCPEDSKEHLVLLAQHLDLEIESMKTVNPSASFELLLVMTALNLM
DEKQSGNEKTGGEILENANQNFQQLLSSLRSELNIVAKKVKK

>gene_1276|GeneMark.hmm|86_aa|-|8663|8923 >NVVL01000056.1 Rickettsiales bacterium isolate NORP64 Contig_source1382A_23747, whole genome shotgun sequence
MFKHVEEAKYIKNYRVWLAFNDGKEGEIDLYDKINSRTGVFEPLKDISNFKNFKVINDTL
SWENGADLAPESLYSMLSTHSKAMKY

>gene_1277|GeneMark.hmm|72_aa|-|8926|9144 >NVVL01000056.1 Rickettsiales bacterium isolate NORP64 Contig_source1382A_23747, whole genome shotgun sequence
MYYDEHNPPHFHAKYGEYEVAVEIKTGIVTGKFPRRALNAVIDWYVIHKEELMEDWELAQ
KDQKLNKIEPLE

>gene_1278|GeneMark.hmm|607_aa|+|9405|11228 >NVVL01000056.1 Rickettsiales bacterium isolate NORP64 Contig_source1382A_23747, whole genome shotgun sequence
MTSSIRNIAIIAHVDHGKTTIIDNLLKQSGTFRDNQKVEERAMDSNDLEKERGITILAKC
TSVVWKDTKINIIDTPGHADFGGEVERILSMVDGVVLLVDAAEGPMPQTKFVLGKALKLG
LKPIVVINKIDRSDAREEEVLDEIFELFMSLDANNEQLDFPVIYASGRSGWAVNDLEKDP
RENLDALFNMVVSHVEKPASDVDAPFSIIVTTREYDPYLGRILTGRIETGIAKINMPIKV
MDREGNVVETGRITKMLTFKGLERLPVEEAIAGDIIAIAGLEKANVSDTIADPEVKKPLE
AQPIDPPTLSMTFSINNSPLAGREGDKLTSRVLRARLMREVEGNVALKITETDKTDAFNV
AGRGELQLGVLIETMRREGFELSISRPSVLFKEDPETGARTEPMEEVQIDVDSEYVGSIV
ESMNLRKGQMKDMRETGAGKTRLIFIAPSRGLIGYHGPFLTETRGTGVMSRIFHSYGEYC
GKIEGRRNGVLISMEDGNSVAYALWNLEDRGDMFIGSGVSLYKGMIIGEHNRSNDLEINP
IKAKQLSNVRASGKDEAIKLSPPRLMSLEQAIAYIQDDELVEVTPKSIRLRKVYLDPNER

KRLSRQA

>gene_1279|GeneMark.hmm|153_aa|+|11326|11787 >NVVL01000056.1 Rickettsiales bacterium isolate NORP64 Contig_source1382A_23747, whole genome shotgun sequence
MISFMKIQIISVGKMSAELATLAKHYQKMIGWEVRSVELVHSKKLKDEQIKQYEAKLIKE
KLTKNAHIVALDLSGKHLTSHEFSKLFSSQMMRGGDIDFIIGGAFGLDESIISEASARLC
LSKMTFPHQIAKLILLEQIYRAQTILNGHPYHK

>gene_1280|GeneMark.hmm|89_aa|+|11800|12069 >NVVL01000056.1 Rickettsiales bacterium isolate NORP64 Contig_source1382A_23747, whole genome shotgun sequence
LRAANCYHCPIILIDWLKMAKEELIEFDGEVDELLPNATFRVTLENGYKIIAHTSGKMRK
NRIRILAGDKVRVEMTPYDLTKGRVILRY

>gene_1281|GeneMark.hmm|197_aa|+|12113|12706 >NVVL01000056.1 Rickettsiales bacterium isolate NORP64 Contig_source1382A_23747, whole genome shotgun sequence
MTTPIILASASPARLTLLRQVGIEPDRIIPADVDETEERGELPKKLAHRLAILKAQTVAS
TIDQGIIIGADTVPAMGRTPMRKAANADDVRSSLKSLSQRRHRVYTGICIIKKDSGGVRE
LSRVVQTTLRFKQISDAEIDYYCSTNEGIGKAGGYTLTGYAESFVAYISGSFSNVIGLPL
FDTMNMLNSCGVRLFGK

>gene_1282|GeneMark.hmm|239_aa|+|12847|13566 >NVVL01000056.1 Rickettsiales bacterium isolate NORP64 Contig_source1382A_23747, whole genome shotgun sequence
MSIHKFSLLHHAFASYLEGDDCDRTWIYTVERNHVGILRELLGIVNAQDIDIKLEILQNP
DLLRLATSSKTRNTDLFSQFLYNACGQSCEEEFLQSAFEFAIAKKDVARVTYIVEVAVYF
EVITPALVALNSLYTVGDRVYQHALYKGAYKLGLEQVYAKTLPTVVKIASLVDIPIDIAR
MIVLGFLGYPEGDQNFLDITEERISALDGNASEEWKALLDITEEDISELGSNASCCVIC

>gene_1283|GeneMark.hmm|364_aa|+|13593|14687 >NVVL01000056.1 Rickettsiales bacterium isolate NORP64 Contig_source1382A_23747, whole genome shotgun sequence
MSKRESETRDYSGRRTSQIDIYAAAGLVTSGRSASVPIIREDTESKVDINGFYRSNSILT
EVGLAASAPGSHRGSVVSFSAGVGEIDGLSRQSSRLSAFEPEGGNENAEEELFAARVSGM
VTRMPSMIKAANRESINNGSQNTLVFSDSLLLIDPNVAWDMVVASGKNNGNFSPWVGELQ
EASVDSITSVKIGQMLRLMVNNIDSATLGVARLHAKENPLEKLNLLSFTKKALIDSEQNL
FNTLVYLQRVDLWDKFLQSLAEPSAHLTKDGLTTIKVLLTALKQFIDSENDTMNGSNRNI
EIQGEYINLSNIPNVLIQLDEYIQNPSVYEASIVATSVELAIVAASDEEGTGCSCMGEEG
CVII

>gene_1284|GeneMark.hmm|196_aa|-|14777|15367 >NVVL01000056.1 Rickettsiales bacterium isolate NORP64 Contig_source1382A_23747, whole genome shotgun sequence
MFLSKRFSVFALIFTILLGALNPAQAAKSGSSSLRTFGDYAQIVNPLFVAGLASQEIGFG
HFAIIYGQSWVSMHGIKLISKSAKWSASKRPNTGNKKDRYEGMPSGHTNSAWVAASYVRM
FSEHKYLSIPLYATAIVTGCSRVKAKEHTTLQVIAGAALAELVTYINSRLDWSDEYQSTN
FYFGGDEVSARFEFRF

>gene_1285|GeneMark.hmm|403_aa|+|15578|16789 >NVVL01000056.1 Rickettsiales bacterium isolate NORP64 Contig_source1382A_23747, whole genome shotgun sequence
MRYLSKKDYSILLGNIIDHFDTSIYIFLAPMLVPIFFPHSDPEMGLIMAYGILATSIVTR

PLGTYLFGALARASSPPVALSYSLIGVGVTTLLVGLLPDYDLIGKYAPILLIIFRIFGGI
FAAGESAIAKLYLIQDKADKEAHSCSYLYQLSTVLGIFLASMTAALIHSIDTANAWRLCF
IFGGIASIIGFYIRKTSLPAPASGLQLFKFYDASGLRELWHHRRVVVRIAIVESFSQITY
VIPFVSMNYLIPLFTNISLSSMMYVNSFMLIFNIFAIIFIGRIVASFAPKYIMMRAGLML
ALTIIPLWYFIEGASIAYVTFVRLWIIILGVAFLCPLNLWYRDQIAGDEKYIIVGMGTTL
ASGIIGKLTPSICLVLYYSTGSHMSVIFYAACIFVAALFVINE

>gene_1286|GeneMark.hmm|522_aa|+|16807|18375 >NVVL01000056.1 Rickettsiales bacterium isolate NORP64 Contig_source1382A_23747, whole genome shotgun sequence
MAQNTTKKRDKDHVSTKVIIAYFAMVVGMFMAILDVQIVASSLSVIAAGLSASRDELSWI
QTSYLIAEVIVIPITGFITKAFSTRIIYLGATIGFTIMSVLCSLAWNIESMIFFRVMQGM
FGGAMIPITFGLAFVIFPKNIQVTVSMIIGLVVTIAPTIGPTLGGYITEISSWHFIFLIN
IIPGILVSIAVFLYVDFDKPNYTLLKNFDFLGIVLIAITLGGLQYVLEEGNKKGWMEDNH
LLFLSIVVAISFVWTLIQELTHKNPIVDFSAFQDRNFSLSCVFSFILGLGIYVVIYLLPL
FLFTVAGYSSLQIGIAMMVAGAFQFLSAPIAARMLSVGTDERIVLAIGFVMFAMGCYTNT
FLTAESRYWELFIPQALRGFSVMFCFISINGLAFDTMPKSRVQNASGLYNLTRNLGGAIG
LASINNLVISRTKIYAQAIKDNMPMTSPQLQETVGAISESMAGRINDPELAGLIFLDSLV
TREAFIIAINDAFAIIGLLFCSGLLLLPFLVLTKKGANSNVH

>gene_1287|GeneMark.hmm|150_aa|+|18365|18817 >NVVL01000056.1 Rickettsiales bacterium isolate NORP64 Contig_source1382A_23747, whole genome shotgun sequence
MFIKLLSLPIFILIWIGLAGADVPIFMIAAPLFVMLIALKMKVLPKKIAFRIGAIPYFMW
LLKEVIMSSIAVSKIAWRKNLKIQPMVEPVKSVQRTDVGIVTYANSVTLTPGTVTLSIDS
HALLVHAIDIRFMDDLQEGEMDRRIKKIIC

>gene_1288|GeneMark.hmm|90_aa|+|18811|19083 >NVVL01000056.1 Rickettsiales bacterium isolate NORP64 Contig_source1382A_23747, whole genome shotgun sequence
MLNICLVIILTFSSIVICGALWQKDVFTKLLFLNSGTSLAALFICFLGSFEVNSSYIDIA
LIYFLLSIVATSAYMKYFLQKHKGNKNAPS

>gene_1289|GeneMark.hmm|396_aa|+|19085|20275 >NVVL01000056.1 Rickettsiales bacterium isolate NORP64 Contig_source1382A_23747, whole genome shotgun sequence
MKNSSKITAETPNSPKITILGLGLGGMITALSLAKHNISTHIIEGRAAGGDGFFHDVRTT
ALTDSSKQFFQQIGVWEKLAELSCPINDIYVADNRAPNLLHFASSELKKGEKMGYLIENT
PFKKALFEEVSASSLINIMDDTSYEITENTPDGCSLLLNGKITHKCDLLLVCDGRGSAAR
RRYFSADLEKSYNQNAITFIAQHEKPHEGSAVEHFLPSGPFAILPLKDQHRSSIVWSVPS
DKNDMLMNLPKDELTHLVQRNFGEFLGEVQIKSEIASFPLKAYVTKQYYNKRIALVADTA
HIVHPLAGQGLNQGIKDVQALAELLLEHGIAERVLEKYQTMRKADNSNILEITDVINSVF
SNDSKLFHSARQLGFAMIESLPPLKNLLIKYAMGRR

>gene_1290|GeneMark.hmm|236_aa|+|20282|20992 >NVVL01000056.1 Rickettsiales bacterium isolate NORP64 Contig_source1382A_23747, whole genome shotgun sequence
MIKTLLTSLANYIMPYRCASCSELIEDSGGMCAECFAKLNFITSPYCQLCGVPFEFAMEG
QALCGKCIITPPKYDLGRSLLKFDQHSKNLIHAFKYNDKTSHAKMFAKLIIARYANDLQD
VEFIIPVPMHRIKRLVRNYNPAQVLSTELAKLLKKRMLPDGLIKTKWTKAQTTLSRSERM
KNLTGSIRINEKYDIKDRVILLIDDVRTTGTTSNTCSNLLKKAGAACVKLVTIGVA

>gene_1291|GeneMark.hmm|83_aa|-|21004|21255 >NVVL01000056.1 Rickettsiales bacterium isolate NORP64 Contig_source1382A_23747, whole genome shotgun sequence
LFYVNYFYGDFIMAKSGIDEWVMVKKGQRELTMDEQYAILDACRTFNYLIGDNLDDLLKS
GLELDYIEPSEPPLVGNSSDSEA

>gene_1292|GeneMark.hmm|84_aa|-|21283|21537 >NVVL01000056.1 Rickettsiales bacterium isolate NORP64 Contig_source1382A_23747, whole genome shotgun sequence
MVSAKRLALHKPSGIIAQKLGTQLLKAIRIINNTWIIRIDSYGLHTTEGLKKNNKNSSFC
VKKHHILLISGLFITVSNYFYDDC

>gene_1293|GeneMark.hmm|220_aa|-|21639|22301 >NVVL01000056.1 Rickettsiales bacterium isolate NORP64 Contig_source1382A_23747, whole genome shotgun sequence
LDGVSADLIQESLHETTNTEQPETGEMPRASSILPDLPPEMHMILPVFMDNLDGEHLKAI
YRTLASFDNLPPELSAKIFRVILVLTIQGSIRLKETCIYLAGLNNPNADSLENLYNILLE
CNNLSDELFKEIYDTLVGFSNLSDKFFEEIYDTLAELKNSSAEESLAETCYTFAETCYTF
EDSLSEAPSLDSAEAEEFVLFDIPDDENTSPAGALGPNDV

>gene_1294|GeneMark.hmm|77_aa|+|22938|23171 >NVVL01000056.1 Rickettsiales bacterium isolate NORP64 Contig_source1382A_23747, whole genome shotgun sequence
MTNNRVKYASTIRKNIGLSINKIRLEKRITLKKLSERTNVSINLLDKFELGKNKINIELI
AIIANALNVSAKDFLLK

>gene_1295|GeneMark.hmm|131_aa|-|23334|23729 >NVVL01000056.1 Rickettsiales bacterium isolate NORP64 Contig_source1382A_23747, whole genome shotgun sequence
MIVIDSNIILDIVTQDSRWFDWSSSQLKTLAEDYKLLINDVIYSEISISFEYIEELENIL
VDNFIIQPIPKEALFLAGKAFLKYKKLGGTKTSTLPDFFIGAHASILNVPLLTRDKKRYK
NYFPNLEIIAP

>gene_1296|GeneMark.hmm|74_aa|-|23726|23950 >NVVL01000056.1 Rickettsiales bacterium isolate NORP64 Contig_source1382A_23747, whole genome shotgun sequence
MKVSTKGQVTIPFDIRNKLGLLPHTEILMKTKGTQVIIEKKAGTVSRGQYVLNRMVGKAS
VKMSTDEIMKITRS

>gene_1297|GeneMark.hmm|298_aa|-|24060|24956 >NVVL01000056.1 Rickettsiales bacterium isolate NORP64 Contig_source1382A_23747, whole genome shotgun sequence
MRSRILIVTCLSLLIFAGGVALYLYPSKSTEIITLTADTSPTKKKPTDPGGMVLPNSDSL
VYEKLKTKVTNRKIRILPEPEAPIEIRRTLKTEAKFLDSIEEILDNIEYYEAKLANNKPD
ALGGSQDSRYGDSSYEDSSYRDSSYGDSRYLDSNYVMPNILIAKSEIPDDMMQDNPPQND
SSRGPHSILIAGTKLNITRAAEPEHKMAQFNLISKDSDGYKIQLSSAYSLASAETQWKAL
QKRHAKILKGAALITKRVEGKNERIFYLVMAGTYSSLSHAKSLCRKLGARKQNCIITR

>gene_1298|GeneMark.hmm|577_aa|-|25032|26765 >NVVL01000056.1 Rickettsiales bacterium isolate NORP64 Contig_source1382A_23747, whole genome shotgun sequence
MNIFKELKNDLKNIGLKLCADQDIWSQASLETPKDPLNGDISTNVAMIIASKEGGNPREI
AFKFKEHLSEIPYVAHIEIAGPGFINFTVKAEKWHASLRSILSDDVDFWEMNVGNGKKVN
VEYVSANPTGPMHIGHARGAVFGDALANILTKCGYMVTKEYYINDAGSQIDTLVDSVILR
YKEALTGQETQIPEGLYPGDYLKPLGKKLADEHGDALLSMDAGEMRGKIKRIAIDEMLIL

IKKDLADLGVNHDIFFSEQSLHDGGKIDEAIDVLRKKDLIYTGTLPPPKGKIMEDWEEKE
QMLFKSTNYGDDQDRPVQKGDGSWSYLAADFAYAKDKIDRGFDNLIYVLGADHNGYVKRI
KAVIEALGDGKVTSDIRTTQLVNFVKNGEPIKMSKRSGNFMTVSDVTRQVSKDIIRFIML
TRRNDMILDFDFDKTKEQSKDNPVFYVQYAHVRAKSILAKAQETIGSAYKNFIAEEFDVS
LLSSEEEIQLIKLLASWPKTLSAAAQHREPQRIAYYLIDVASKFHSIWNLGKENNNYRFN
IEDNIELTTARLALAEAVRKIIAGGFKVIGVTPMDKM

>gene_1299|GeneMark.hmm|386_aa|-|26762|27922 >NVVL01000056.1 Rickettsiales bacterium isolate NORP64 Contig_source1382A_23747, whole genome shotgun sequence
MLKQYACLPENSRGRIHPEDPTPYRNEFERDRDRIIHANAFKRLQYKTQVFVNHEGDHYR
NRMTHSMEVSSIARSLSKTLGLSEDLAESVALAHDLGHSPFGHAGEHALNECMKDFGGFS
HNAHSFKILTNLEHKYAAFDGLNLTWEVLEGIVKHNGPVINEKSYEYVYEYNDKHGLDLE
KYSSAEAQVASLADDIAYICHDLEDSIRAKIVSYRDLEELEFIDKYIHEVRAVYPNIADN
RLIYEIGRKLTHHLIDSLLLQTNRNLKSFKIETESDIRNHKRQIVEFTPSIFEEVKQIKD
FLMRKVYKHHEVAYVTLQSQNVVRKLFKLYVDNIELMPFSWRNMIAENNLDSKMSIIADY
IAGMTDRYAIKQYQSFFNLSFANKGL

>gene_1300|GeneMark.hmm|110_aa|+|27997|28329 >NVVL01000056.1 Rickettsiales bacterium isolate NORP64 Contig_source1382A_23747, whole genome shotgun sequence
MNLLISADASKRVAELIAKNDKKMVALRVSVDGGGCSGFQYQYQLAESINEDDFVLISDD
KNVKVIIDAVSQEFLDGCSIEFIQELGSSHFQITNPNASAKCGCGNSFAV

>gene_1301|GeneMark.hmm|752_aa|+|28769|31024 >NVVL01000056.1 Rickettsiales bacterium isolate NORP64 Contig_source1382A_23747, whole genome shotgun sequence
MLFGVEIGLDMVIVIGFLVLTLVVGMGHGKSVKTIKDYALGGRNFSTAALTATIVATWAS
GSGFFVTMSKTYSDGLPYMFARFGIGIYFLIVSFLLVPRMGEFLGKISIAEAMGDLYGRH
VRVITAIAGTIGAVGSVAVQLKVFGSIFSYFLDLSHYVTIIVAGVIVTVYSAFGGIRAVT
FTDMLQFFAFGIIIPVIGFIIWNDFYQGSADLTYAMSDPRFNITEIFSANNINSLEMTVL
FCYFAMPTISAAAFQRISIGNNIAQVKKAFLIAGTFLIVIQVITAWIPFLIHALDPSLAS
DQLLGYIIDTYTYPGLKGFIMVAILAFAMSTADSRINSASVLFTNDIYKLALPNLTNEIF
VSRLFAFILGGGSIMLALFETDILGILVLANSFYYPVVTPPFLLTVFGFRSSKKSVLIGM
AAGFSTVVIWKLLPVLFTSMSQKIVGVLFAMFVNAVFLMASHYLLRQKGGWVGVKDTTYI
DEQRIIRKKKWAAFMKKFQSFKLIKTIKEAPVPSESTCVMMGIYFILFTVTIAYSTDIDT
LKDNGNLVSIIYQIMMVTGTVMAMYPIWPMNIKQKTRDMIIRIWYPVTMFYMMVFFSAFF
VLLSDSNTLHYMIFTANIITLIILIGWQVGAVMIIVGTYMAVQFYKYYMGIEYLDISLGS
PQFILVYSLVFVGVILMIFLKPKQERLEQTEHRVGELSDEVGTLVTVVSSRDSQISTLDT
KVSGLEERVDHYTERTTNQAQEIERLGATAQR

>gene_1302|GeneMark.hmm|272_aa|+|330|1148 >NVVL01000057.1 Rickettsiales bacterium isolate NORP64 Contig_source1382A_23831, whole genome shotgun sequence
MKKIVIHLVSESSGQTVKHAANSSLSKFRDIEVKKYHWPMVRNLKMLEEVFEKVRRKPGM
ILYTISNEELRGALKKFCYDLKLPCVSVISKIVKEISEYIGTSADPAIGYANKFDDSYFD
KVEAIEYSLRHDDGQILEDLEEADIILIGASRTSKTPTSVYLAYNGFKTANIPFVRECPF
PDFLHEMTNPMIFGLVINPNRLIGIRESRMNLLQVTEKSAYTDIDSVREECRQVKKLCSE
NNWKIIDVSMRSIEESAAIIMKHYYDKKKMSR

>gene_1303|GeneMark.hmm|204_aa|+|1123|1737 >NVVL01000057.1 Rickettsiales bacterium isolate NORP64 Contig_source1382A_23831, whole genome shotgun sequence
MIKRKCRDNIGMMKNIAITGGFASGKSFLLKVAKSLGYDIFSCDDYVGMLYKDEKIQKII
ESEIDGLAKFNKQDLAKIIYDDDNARKKLENIVHPMVRAGIREFEQKNADKEMIFTEVPL
LFESGFDKYFAHNICVYCSDATRYLRAKSRGYDDDKIIERINKIQLTSDEKRKMADFTID
SEQSFGRMKSLLIGITSQIKNHRH

>gene_1304|GeneMark.hmm|221_aa|+|1760|2425 >NVVL01000057.1 Rickettsiales bacterium isolate NORP64 Contig_source1382A_23831, whole genome shotgun sequence
MTEREIILDTETTGLSPKDGHRIVEIGALEMVNKVLTGKKFHQYINPERDMPEGAYKVHG
ISYEFLQDKPLFSDIYKEFMDFIEGGKLVIHNAQFDIRFLNHELSLVKAAPLNLEDAIDT
LAMARRRFPGTRVNLDALCKRFKVDNSSREFHGALKDAALLSEVYVELTGGRQVTFSMDK
KDVKLARDSGDKFGARAIGNKIVIQPTEEEKNLHNKFLEKL

>gene_1305|GeneMark.hmm|209_aa|+|2441|3070 >NVVL01000057.1 Rickettsiales bacterium isolate NORP64 Contig_source1382A_23831, whole genome shotgun sequence
MQNKNNISAKSSKSNIELKKSLRKDILSRRKKLNEDDYFLANNIIIENVENLLACLKNET
PSKKRVAGLYWPMKGEPDLVKLIINSNWRIALPKVRGVKMDFMTYKIGEQLELSMGRGVR
QPQNNITLSPVVIIIPGLAFSIKGDRLGFGAGHYDRYFDKLGKEKDITKEKDVIKIGVCF
DENLYEHLPNEPHDVKLNYIITEKTIIAL

>gene_1306|GeneMark.hmm|240_aa|+|3067|3789 >NVVL01000057.1 Rickettsiales bacterium isolate NORP64 Contig_source1382A_23831, whole genome shotgun sequence
MTVTLFDSLIIVITVISSLFGVYKGMIHIIINFLGFIASIVFAMVIYPYALIMLSGYIES
ELVASITSGVASYVFSLVIFTFITAKTILLFSDSGGGIIDRILGLVAGFARGVFFSLIVF
GIAAIATTGTYSNVKHTEDMVLKLSSSKYPDWLADATTTPHLEKMLKDITSMILDRNRDR
GNIEDIEDMDDMMDLAEEKEEDLIDVIKRKKKKSFSSAISKSTTSSIAVPIEVGSENDFD

>gene_1307|GeneMark.hmm|246_aa|+|3806|4546 >NVVL01000057.1 Rickettsiales bacterium isolate NORP64 Contig_source1382A_23831, whole genome shotgun sequence
MQKYTFKEHLVELKNRLLKVVCAFIIAFGVSYWLKDDIYEIFLKPLSDLSSQGGRKIIYT
GLAEAFFSYIKLALFSALFIILPVICYQIYGFISSGLKESEKKIVSSVLAFSPILFYFGA
FFVFYFVMPRAWDFFLSYESSSMKIPLIMEARISEYLGLVMQLTLVFGMAFQLPIIMVIL
CVIGLVDSCGLRSKRRISIVIIFIVAAIFTPPDVISQIALAIPLMMLYETSIIMCKLVEN
RTEGSK

>gene_1308|GeneMark.hmm|427_aa|+|4548|5831 >NVVL01000057.1 Rickettsiales bacterium isolate NORP64 Contig_source1382A_23831, whole genome shotgun sequence
MLDIKWIRENETEFQELMQTRGLKIDSEKLIYLDEEKRKLTTMLQQFQQAKNQKSKRLGA
LKGRVSVEFDQLKKDVSHINEKLIEISSQLNEFDEFQSIMDNIPNLPANDVPYGIDESMN
RFVRDFKEPLEFPYAKEHFDLGRDLGMMDFEQTAKISGSRFVTLTGKLAKLERALISFMI
DTHNEDFAFEEVSPPSLVRSEAMYNVGQLPKFADDSYLTEGGDFRLIPTGEVPLTNMVAG
KILKRDELPLRYVAYTECFRSEAGSAGRDTRGMIRNHQFGKVELVTITTPDESKNEHEHM
LNASEEILKRLGLPYRVMLLCTGDMGFSARKTYDLEVWLPGQKKYREIASISNCESFQAR
RMKARYKETGENNTNFVHTLNGSALAVGRTIVAILENYQNEDGSITIPEVLRGYMGGCDK
IEKPNAS

>gene_1309|GeneMark.hmm|84_aa|+|5821|6075 >NVVL01000057.1 Rickettsiales bacterium isolate NORP64 Contig_source1382A_23831, whole genome shotgun sequence
MQVNMHLISDIVGMFGVALVILSYFLLQSEKVTSNSPFYLYSNLIGAIFLLFSLWYHWNL
ASVIIEVLWLLISIYGIVRNYRGT

>gene_1310|GeneMark.hmm|351_aa|+|6244|7299 >NVVL01000057.1 Rickettsiales bacterium isolate NORP64 Contig_source1382A_23831, whole genome shotgun sequence
VEEIMSDRIFYNNPTSHKLSEIAQAIGAELPSLPGGILAGDIDISTVKSLSEAGKGDITF
LSNKKYIAEFKATKASACIVPLGFGEDSNSDSELIMLKVKNPYSAYTHIIDMFYSSGKKL
GSRIMPTAYVSDSAKIGKNCYIGHNVVIEDEAEIGDNCIIESGSVIDYKVKIGNNARIDS
NVSISYSIIGDNFVALSGARLGTDGFGFSTNNGIHKKIYHIGRVVIGDDVEIGANSCIDR
GSMNDTIIEDLCRIDNLVQIGHNAHIKKGAILVSQVGVAGSSTIGSYCALGGQVGIAGHI
TIADGVQISAQGGVIQDIKEAGIMGGTPAVPIRDWHKQTIMMKKLIKGKIK

>gene_1311|GeneMark.hmm|149_aa|+|7296|7745 >NVVL01000057.1 Rickettsiales bacterium isolate NORP64 Contig_source1382A_23831, whole genome shotgun sequence
MTQGVINIEEIMSIIPHRYPFLLVDRVLEIELNESIIGIKNVTANEPQFTGHFPGRPVMP
GVLIVEAMAQLSAILVAKSMESVKDKEVYFMSIDECKFRKVVQPGDSLIMHANIVQNRGA
VWKFKARAEIGGQIAAESCFTAMVKDKTK

>gene_1312|GeneMark.hmm|260_aa|+|7755|8537 >NVVL01000057.1 Rickettsiales bacterium isolate NORP64 Contig_source1382A_23831, whole genome shotgun sequence
MIHKTAIIGENVKIGKNVKIGAYSVIGDGAVLGDNVELKSHVITEGRVTIGEGTVIYPFA
SLSYPQTLKYEGEDSEVIIGKNNTIREYVTIQHGTTEGGMATKVGDNCLFMVGVHIAHNC
IVGNNVIFANYASLAGHVEIGDHVIIGGLSAVQQFCRIGAHAMIGGVSAVTRDLVPYGLA
SSERAHFQGLNLIGMNRRGFDKKQSLEASKIVKDIFNGSSGGVFNKRVEIAKESYPENKV
LQEIIEFIQKDETRTYCKFK

>gene_1313|GeneMark.hmm|283_aa|+|8551|9402 >NVVL01000057.1 Rickettsiales bacterium isolate NORP64 Contig_source1382A_23831, whole genome shotgun sequence
MIPNLGIVAGEGELPLELAKLYKLSGGQCFIASISGAKTLDGFPCKPFALGAVGGMIQYF
KENKVENILLIGGINRPDFFTLRVDLTGGALIAKILKQKILGDDNLLRIVSDYIESKGFK
IISPAEILSLGAGTNAEIGTGAGAGFETRAGQITSKKVPSEQDMADIEIGKNIAKSLGAL
DVGQSVIVADGYVLGVEAAEGTDNLIQRCAELREKPTGGVLVKMSKSAQDMRLDVPAIGV
ETIRLLAECGFNGVAIEQNGVIVISPESTKQMLDKAGVFFSYI

>gene_1314|GeneMark.hmm|733_aa|-|9461|11662 >NVVL01000057.1 Rickettsiales bacterium isolate NORP64 Contig_source1382A_23831, whole genome shotgun sequence
MSSSKSEQLTAIEALQKYRLLYKSFTEEIVEFYTAQGAMDWNVLRYILTGKAMLDKELGD
NDSLFLMLYKIGVCYHELSKQTPELHHKALSYIQDAQQILNNYSINLSTEFRDGFFAFCN
KVQQAQPSGEVQQTPPSSKIHSAQSSLAPTQQKYEAFAQETVEFIEKYPTVQGSSVLLYV
LKGREFFSIATNDMAGLHHTLLSTAAWYLNLANRASTKEHTDDSIKIAKNCVAEAIKAFP
RESMTPAQQESFRSCMNKLVALKESAMNNSAVPNLPPQAPSEALGAMPISPEFENKLPIL
TPSLSSSSSSSQIAPPNVGIANSLAHSQFQLSPQLLAHLTICAEKNRNGYDQYVEATTPV
FNQLKQDVQNLSCCNLLLHLFIDAERLAKIWDNSESLFFITTNIAICYIMLSQVSANYKA
KATEYIAKARGMPYINNIPQNEINDFNAFTGEFEARISVNEPCNIAALQQQARISFPAAQ
INQSAYKQYGQITNLAIELTKKHPNDPSHHSVLFEILHGQRNLDCAHSNWNNMFSSLSNI

VVCCAKLSQVDASYRQEAMKYIDEAKNLLSQLSQDPTLAKTVYSFNKFVQRFEETRTDQD
MAPHISDDVPPLPLPPTGMQSSSSSSSSAPKRSLPPTDTPPAKRTKVTHDETNDSPTSLV
SPLPATMITIPKAPPTPPSDAFETDPFCPSNLETSSSPWGTPMSDIAGIDSDTNMFSMFG
EDGEDAGMSWMLT

>gene_1315|GeneMark.hmm|304_aa|-|12080|12994 >NVVL01000057.1 Rickettsiales bacterium isolate NORP64 Contig_source1382A_23831, whole genome shotgun sequence
MAITASLVKELREKTGAGMMDCKKALVETNGESEAAIDWLRTKGLAAAAKKAGRTASEGL
TGVCVDGTKGAAIEINSETDFVSKNEVFQNIVKEVSAIAIGHDSLEGLKAASTKSGKSVE
DEIIANVATIGENLNLRRMESVSVTDGVIASYIHNATTENMGRIAVLVALESTGDKAKLM
ELAKQIAMHVAAARPQCLNAESVDSALVQREKDIFTKQSKASGKPDNIIEKMIEGRIRKF
LEEIVLLNQTFVIDGKTKISELVASFAKELGTPVELKSYVRFELGEGVEKEETSFADEVA
AAAK

>gene_1316|GeneMark.hmm|302_aa|-|13051|13959 >NVVL01000057.1 Rickettsiales bacterium isolate NORP64 Contig_source1382A_23831, whole genome shotgun sequence
MSNLPKVSVGSLLDAGIHFGHKTSRWNPKMAPYIYGTRDGIHIIDLQQTVPLLQNALQKI
YETVKNNGKVLFVGSKVQATELIAEYAEKCGQFYVNNRWLGGTLTNWNTISKSIKKIDDI
EKIIEDEKKASSYTKKEILDLTRKREKLLKSLAGIRKIGGIPDLVVVIDTNKEHLAVNEA
TKLGVPIVAVVDSNSNPDNISYPIPGNDDAIRSIRLYCELFSEAALAGIKDALASSGVDV
GLSKKAEGDKSLDGVKKLEKTAKVSKAKPAPKTEAKSAAKPEPKAKAAPEKEAAKESASP
AK

>gene_1317|GeneMark.hmm|334_aa|-|14269|15273 >NVVL01000057.1 Rickettsiales bacterium isolate NORP64 Contig_source1382A_23831, whole genome shotgun sequence
LLKKVFKIKLLLIIVAAVFLLSIVNILGSFLFLPGNLQKEKTIIIKPNLSISQISALLED
AEIIKNKRLFEITCQIYSYHKKLKVGEYRFTQNITPYQIINKLTAGKSIVHRLFIPEGTL
VHEIIEQINANELLFGKKISHTIPEGYLMPSTYFYSYGDQREHIVNKMRTEMSEALDAVM
LKLSKNSPIKTRKDVLILASIVEKEAGNDRERPKVAAVFINRLNKKMKLQADPTTIYAIT
NGKYKLERSLTRKDLRIKSPYNTYHTYGLPVAPISCPGRASLEAVVAPAKTTALYFVVNG
RGGHSFSNSLKEHNKHVRKFKARVKRQKARRKRG

>gene_1318|GeneMark.hmm|79_aa|-|15276|15515 >NVVL01000057.1 Rickettsiales bacterium isolate NORP64 Contig_source1382A_23831, whole genome shotgun sequence
MANLKSIEKMSFEESLAELEGIVRKIDAGQENLADAVGSFERGVTLKNHCESMLKEAKLK
IEKITSTDNGEIKTTEVEL

>gene_1319|GeneMark.hmm|157_aa|-|15532|16005 >NVVL01000057.1 Rickettsiales bacterium isolate NORP64 Contig_source1382A_23831, whole genome shotgun sequence
MTHYFDESFVVAICFVAFIYLAYQPVRKAILASLDARIQEIKDKLEETETIRNDAKKLLD
DLEHKMSDFEARKQVILDSAEVTTKRLVETKTQEMELLLARKKESAVKFIENQKTKATAA
MRDEFTESVLNMVRTYLVETKNNSVSDEEIINHFIKK

>gene_1320|GeneMark.hmm|159_aa|-|16002|16481 >NVVL01000057.1 Rickettsiales bacterium isolate NORP64 Contig_source1382A_23831, whole genome shotgun sequence
MPQFDVSSFTSQLFWLALSFGCLYFLVSRFIAPKAESILTARNSCLEGNIHDANEYNNKT
KLLEITRKERSKEVHASVEEKHQQVLRALEANFNEQMEELAGVLQKKTEKALSEVNSYID

KFHADEPNSCAHLAAFIIQKVTNKQADLKLLEKIHGRSK

>gene_1321|GeneMark.hmm|75_aa|-|16510|16737 >NVVL01000057.1 Rickettsiales bacterium isolate NORP64 Contig_source1382A_23831, whole genome shotgun sequence
MEELMALKFIGAGLMAFGMLGAAIGVGNIFGSLLGAIARNPSASDQLQRMAFIGAGLAEA
MGLFSFVIAMLLIFT

>gene_1322|GeneMark.hmm|242_aa|-|16869|17597 >NVVL01000057.1 Rickettsiales bacterium isolate NORP64 Contig_source1382A_23831, whole genome shotgun sequence
MSHSPLDQFKIKELINIELFGHDVSITNSAAFMILASLSVFVYFMIALKDKKLVPSRLQL
SAEGTYGLIRDMLEQNVGSGGRKFVPLIFTLFMFILACNLLGMIPYGFTATSHISVTFAI
AMMIFLLVTVLGLFLHGFRFFSLFLPAGTPWWLAPLMVVIELFAYLARPVSLSLRLAANM
VAGHVLLKVMAGFVISMAIYLKFLPIPFIVILVGFEIFVAILQAYIFAILSCVYLSDAIN
LH

>gene_1323|GeneMark.hmm|103_aa|-|17587|17898 >NVVL01000057.1 Rickettsiales bacterium isolate NORP64 Contig_source1382A_23831, whole genome shotgun sequence
MRLWKLKNKMHNKKHQEIQNRIDKFKTVKKQASTQANTPLSSASRALNIGVELVAGTIVG
VVMGLFFDNLFDSKPLFLIICLIFAIIAAFKSIWNRYIKNNVT

>gene_1324|GeneMark.hmm|263_aa|-|17873|18664 >NVVL01000057.1 Rickettsiales bacterium isolate NORP64 Contig_source1382A_23831, whole genome shotgun sequence
MLQYDIANLNMQDSMNIIKKIFLTIAILGLSGCSEDAAISTNADQTMKDSATTSQAEGAS
PNEQGQEQGGDPKTETFEHVSPRSKYEVTDNDTMFGNKDAQVTIIEYFSPTCPHCVIYHK
KVFPEVKKNYIDTGKVLYVMREFIATKQDLDATILAKCLGDKESYAKFMDVILEQQINWA
YSKNYREILTNIGSLGGVSPEKYAACLNDESKIELLIQNTSLIYQDPEFGGTPIFFINRK
AVIGLYPVKKFLKEIDEALEAKK

>gene_1325|GeneMark.hmm|71_aa|-|18769|18984 >NVVL01000057.1 Rickettsiales bacterium isolate NORP64 Contig_source1382A_23831, whole genome shotgun sequence
VYTKYNINQVGGAYMANNSQEWGALFEEWLLPVYGSPIVSCGSGTVLPADPNAASEPATE
IFPGLIYTEPR

>gene_1326|GeneMark.hmm|175_aa|+|19278|19805 >NVVL01000057.1 Rickettsiales bacterium isolate NORP64 Contig_source1382A_23831, whole genome shotgun sequence
MSGIVTYEGVFKFGEGNVYLTVSFSMCIIGKKVEIMNLNVAYSKTQWGDERSLISTDSEL
NAWLEVVNNPLLSEYPNYTTKELAIFLVAMNCAAFYAQNNCSGISSSDRIEYKISPAKAE
SVADSLKAAGFAREDSKGHFDDSSIVLFTRAHDELPLGMELVDMQVAAEIIEQWE

>gene_1327|GeneMark.hmm|41_aa|+|19989|20114 >NVVL01000057.1 Rickettsiales bacterium isolate NORP64 Contig_source1382A_23831, whole genome shotgun sequence
MKVVKSLKSLKARDKNCRIVKRKGRVLVINKVNKRFKAKQA

>gene_1328|GeneMark.hmm|956_aa|+|20631|23501 >NVVL01000057.1 Rickettsiales bacterium isolate NORP64 Contig_source1382A_23831, whole genome shotgun sequence
MFGINLDLDISIVVGFLVVTLIVGMGHGRHIKTIEDYALGGRNFSTAALVATIVATWASG
SGFFITMSNTYSDGLYFIIAVMGMGISFLIEAAFIVPRMGEFLGKVSIAEAMGDLYGKEV

RIITAVAGTIGAAGMISVQFKVFGNMFSYFLHMPNYVAIISAGFIATLYSAFGGIRAVTF
TDILQFVAFGMIIPMIGFIIWSQFYYEGLSFTEAVSDPKFDLSVLFDPSNPKLIGMVIMF
CYFCIPSMSAPAFQRVAIGRNVAQVKKAFLISGICLIVIYMIIAWIPFLVYSMNPNLETT
HLLGYIVDTYSYAGLKGLIIVAVIALAMSTADSRINAASVLFTNDICKVIVGDLKRELLI
SRSFAFVLGIGSIILALSETNLIGIVVFANSFYYPVVTPLFLLTVFGFRSSSKAVLIGMG
AGFVTTVLWKVLPIEFSYFSQKMAGVLFAMLMNAVFLMGSHYLLSQKGGWVGIKDRTYID
MQKALRAERIATFINKVRNFNPVESIRKIPLAGESTYVTIGVYFLIFTFTTIYSTHMEML
KENGKMITTIYQIMMISGTMMGMYPIWPLSIKQATKEMAIRIWCPIAIFYMLIFLSTFFV
LVNKVNNLHFVMFAVNMIVSVVLLGWQVASAMIVVGAYMAVQFYKYYAGVEYLDTSIGSP
QFILLYSLVFIGTTLIIFFKPKQERLEQSEQKVDTLSYAIGDLTSKVTGLNEEVSHYSMR
VSNHEKEIERLGGTAQRIINNLNHELRLPVGNVVNFAEMLHDGLGKFSQDQLKEISKEVY
QNSSRLSSMILNMLDLATLSAKKIELNKKEIDFSRMVTERVHACRNIYAGDKPLEFKLEI
QPGIIAVVDENYLRQTIDNLLINAINFSEKGVITTILSRKKNLVSLVIQDQGLGIPKSEI
YDIFTPFKMGSNMETKAEGRGIGLALCKAAINAHGGEINAESQEVGTLFKVTLPLD

>gene_1329|GeneMark.hmm|117_aa|-|23586|23939 >NVVL01000057.1 Rickettsiales bacterium isolate NORP64 Contig_source1382A_23831, whole genome shotgun sequence
MSKHDLIEYKNYYGSVHFDAKEELFYGKIEYIRDLVNFEASDAKSLVQSFKDSVDDYLEE
CRALNKTPDASFKGSFNIRITPELHREIGLYALQHDSSINGVVKSALKEFMQQQGHA

>gene_1330|GeneMark.hmm|85_aa|-|23936|24193 >NVVL01000057.1 Rickettsiales bacterium isolate NORP64 Contig_source1382A_23831, whole genome shotgun sequence
MSRLEKLIVKLKRESKDFSWNELIKLLTGLDFTELSNGKTGGSRRKFYHEGHNIIINLHK
PHPSPYLKEYALKQIRNKLSEEQMI

>gene_1331|GeneMark.hmm|101_aa|-|24343|24648 >NVVL01000057.1 Rickettsiales bacterium isolate NORP64 Contig_source1382A_23831, whole genome shotgun sequence
MGAQNIENILDSGAFIFYLMNSNAQSEFGLIMAGVAQLVRAPDCDSGGRRFETGHSPHLQ
ETMRMVLEVWGLGFIIYASPCLIIFYKHQLIIFTRTCSSAG

>gene_1332|GeneMark.hmm|88_aa|+|24853|25119 >NVVL01000057.1 Rickettsiales bacterium isolate NORP64 Contig_source1382A_23831, whole genome shotgun sequence
MAANKILTVKLEAETKSKLQHLGKVKSRSTHWLMKKAIDDYVEKEEKKEALRQETLARWE
EAESGRVVGHDLVIQSLDSWGAGDKSDV

>gene_1333|GeneMark.hmm|96_aa|+|25112|25402 >NVVL01000057.1 Rickettsiales bacterium isolate NORP64 Contig_source1382A_23831, whole genome shotgun sequence
MFKIFWLESAVDDLARLKAFLEKASPKAASIAAKLIKNATNKLQEFPLIGQDVEGLEDFY
DIFIEFGASGYHLRYRVFNQKIYIIHIKHARELSFK

>gene_1334|GeneMark.hmm|348_aa|-|25584|26630 >NVVL01000057.1 Rickettsiales bacterium isolate NORP64 Contig_source1382A_23831, whole genome shotgun sequence
MSFSPKFQITNETTSALTAIERTRGFLDAATLSNEWIEQMQERALILEAYHTTHIEGTQL
TLEQSEQLLAGKKLLDTNPDDVKELLNYRKAFELVADYLNVGEPITNGLVREIHKELVID
VRGNSAAPGEYRKIQNYVVNSKTGKPVYTPPPAFEVPIMMSQLTDWINTEESINPILVAG
IAQFQFVHIHPFLDGNGRTGRLLSTLCLYRKGYDFKKLFSISEFYDRDRANYYKAIQSVR
ENDMDMTNWLEYFTRALSTQMQEIRSNGEQIIKLDILKNKYKLNPRQQNAVKHIMKHGSL

TIKDYETIFPKINRRTLQRELKAMVEYNVIKADGLTNQSVYKLTTNPL

>gene_1335|GeneMark.hmm|241_aa|-|26763|27488 >NVVL01000057.1 Rickettsiales bacterium isolate NORP64 Contig_source1382A_23831, whole genome shotgun sequence
MKKLAIVTGGIRGIGAATSIALHEGGYNVVANYNNNHEAARKFTAKTSIRAASWDVSSSK
ECRESVAKIEQEFEQTVSVLINNAGITRDGMLHKLSAENWAEVIDINLSSCFNMSSAVIG
NMRNQGFGRIVSISSVNAQLGQMGQTNYSASKAGIIGFTKALARESASKGITVNAIAPGY
IATDMVKQIPDKILAKIIEQIPVKRLGAPAEIARAIMFLIDEEASFITGETLSINGGHNM
V

>gene_1336|GeneMark.hmm|104_aa|+|27583|27894 >NVVL01000057.1 Rickettsiales bacterium isolate NORP64 Contig_source1382A_23831, whole genome shotgun sequence
MMSKIEKSDTIEISSVTYDYLMRIFCLLSICLLVNICIAKAGIIRDSEIEEAVDLIVAPL
REASGLKDLEVHIINNPIPNAFTAGGNKIFINTGLIINHPDPDI

>gene_1337|GeneMark.hmm|51_aa|-|3|155 >NVVL01000058.1 Rickettsiales bacterium isolate NORP64 Contig_source1382A_23838, whole genome shotgun sequence
MNLRKNGMMIFGLASAVSCGLLSSQTYAACDAGFTTDSADFALEAVEDFES

>gene_1338|GeneMark.hmm|179_aa|-|315|854 >NVVL01000058.1 Rickettsiales bacterium isolate NORP64 Contig_source1382A_23838, whole genome shotgun sequence
MRIKLAIIAVLAVILALLFWPSTQKILFHTESEFGPIWVYEEDNQRRLSFNGVPAHLIQS
QIHLDRPEHLVAPYNQMLMSSLFFTSNPKKILMIGLGGATTAKALNLLLPKTPIDIVEIN
PALPPIAAEYFDFREDARNKIFIEDGVGFMKNAAKNSYDLIILDAFDIDYIPPGFLTLS

>gene_1339|GeneMark.hmm|420_aa|-|862|2124 >NVVL01000058.1 Rickettsiales bacterium isolate NORP64 Contig_source1382A_23838, whole genome shotgun sequence
MIEDSDRIFTNLHGTESSGLQAAQKRGDWSKTKSLIQKGRDWIVQQVKDSGLRGRGGAGF
STGMKWSFMPKDSDKPSYLVVNADESEPGTCKDRDILRNDPHKLVEGCVLASVGMGANTC
YIYIRGEFYNEAIILQNAIDEAYAAKLIGKNACNSGYDIDIYLHRGAGAYICGEETALLE
SLEGKKGQPRLKPPFPAGFGLYGCPTTINNVESIAVVPTILRRGAEWFAGLGKPNNTGSK
LFCISGHVNNPCTIEEEMGIPLRELIEKHAGGIRGGWDNLKAIIPGGSSVPMIPKDVCET
VTMDFDSLRAVGSGLGTGGLIVMDKSTDIIYAIARLSKFYMHESCGQCTPCREGTGWMWR
VMMRLVKGDARPEEIDELLDVTKQVEGHTICALGDAAAWPIQGLIRHFRGEILDRMKLNQ

>gene_1340|GeneMark.hmm|242_aa|-|2111|2839 >NVVL01000058.1 Rickettsiales bacterium isolate NORP64 Contig_source1382A_23838, whole genome shotgun sequence
VSVRIRVRVRMRESEMRMKKLFGILLICIFSSLVASADSISGRFFVKGKSLDFDRSFHPI
KAGSYTKGKPTKCHGTIVYPELSHDDAGLFMTINRKIYDFVEIYGICNKDEQSNFSVSYD
VPRSRSKNFFSVLWMTKKDDKLWRIDSLNFNFESGNLLEVDDIFNLLSNHMMGEMVKLSA
GHLPKNTNWEQFLAKIASRDVQLYIKNREWHIVFNATANLDMVIDVKIPEYFLIGSRNND
RG

>gene_1341|GeneMark.hmm|189_aa|-|2841|3410 >NVVL01000058.1 Rickettsiales bacterium isolate NORP64 Contig_source1382A_23838, whole genome shotgun sequence
MSKMAKMDSKTEVFSFNKENLKKAQEIIAKYPEGRQKSAMLPLLDLAQRQNDGWLSVAAI

ECVANHISEPYIRAYEVASFYSMYNLKPVGKYHIQVCGTTPCMLRGAEDIMKSCEKHALA
KCGENSKDGLFTVSEVECLGACVNAPMIQINDDYYEDLDPKKVGDIIKALREEAQPSSGK
SPAKEKEKA

>gene_1342|GeneMark.hmm|405_aa|-|3407|4624 >NVVL01000058.1 Rickettsiales bacterium isolate NORP64 Contig_source1382A_23838, whole genome shotgun sequence
MRKQRNNKIVENMEAASEKKTTTINFGPQHPAAHGVLRLILEMDGEVVNKADPHIGLLHR
GTEKLIEHKTYMQATPYFDRLDYVSPMCQEHAYALAVEQLLKCDVPIRAQYIRVLFSELT
RILNHTMNIGTQAVDLGATTPLLWLFEEREKIMEFYERVSGSRMHANYFRPGGVVADLPK
GLLEDIATFSGQFLSRLADVETLLNNNRVWKQRLVGVGVVTQKQAMDWGFSGVMLRGSGI
EWDLRKSNPYEVYDKMDFDVPIGKNGDCYDRYLVRIEEMYQSIRIIDQCIKQMPEGEVKS
LDMKISPPKRKDMKDSMEAMIHHFKLYTEGYNVPKGESYGVVEAPKGEFGVYLYSDGSNK
PYRCKIKAPSFAHLQGLDFMTRGHLMADVVSVISSLDIVFGEVDR

>gene_1343|GeneMark.hmm|199_aa|-|4602|5201 >NVVL01000058.1 Rickettsiales bacterium isolate NORP64 Contig_source1382A_23838, whole genome shotgun sequence
MEYTHIIDEFAKDNDLSITPISIDGFAAYRTSAGDVLSLLKFALEDEELRFSVLTDLFAA
DFPEREKRFELVYSLLSLKLNKRLIFKIDLAEGETAHSVTSLYSAACWCEREVFDMFGIE
FSGSHDMRRILTDYGFKGHPLRKDFPLSGHLQVRYDEKLEKVIYEPVMLEQEYREFDFTS
DWKGPEGYVLPGDEKAKKQ

>gene_1344|GeneMark.hmm|170_aa|-|5207|5719 >NVVL01000058.1 Rickettsiales bacterium isolate NORP64 Contig_source1382A_23838, whole genome shotgun sequence
MKNNLQTGLKNNSFDEGLSNRGYVLTKIDDLTGWARSNSLWPMSFGLACCAVEMMQTASS
RYDMDRFGFLFRPSPRQADLMIVAGTLTNKMAPAMRRVYDQMAHPKYVLSMGSCANGGGY
YHYSYSVVRGCDRIVPVDVYVPGCPPTAEALLYGLMMLQRKIKRTSRFKG

>gene_1345|GeneMark.hmm|128_aa|-|5727|6113 >NVVL01000058.1 Rickettsiales bacterium isolate NORP64 Contig_source1382A_23838, whole genome shotgun sequence
MLNNMLQNLDFLSEYLPIAIFFCVATILSIVLVALPKILAKEKPQKDKLLSYECGFDAFG
DARGKFDIRFYLVAILFIIFDLEVAFLIPWAINLKAIGHFGFWSMMVFLFVLTVGFAYEW
KKGALEWE

>gene_1346|GeneMark.hmm|611_aa|-|6366|8201 >NVVL01000058.1 Rickettsiales bacterium isolate NORP64 Contig_source1382A_23838, whole genome shotgun sequence
MKKKILLTTALSLVPMFANGAGPVNTTYQGNNIFATDNQEFTGIIGVGQHLADAPIMIAS
DRNKYFRDMVQDNSILALMAGNIERLGRELKSNIPPEILQKATAFLAKVTKEKEKVLSAR
KMINENFDGDFSLIDAIQGVDPKNLTTAAIKAILQNAYDEKRRGEASVDCVVTPTTAPTA
IAASSATTSSAPASAEDIKSQKELAHLQQSEQRYLRQQGGEKREKMLKDVQTDIDHHPRI
EERRKSESTQEDFQDCLVDIADETDEAKKEALVKRLREDLRDYISPNIKRNTAPKRLVAA
QQPETAALEAMTKSVQTISRVVGGRMASLSHVGVSSGEAFESYGVWLKGTYSQGEQKAYD
ARSGYKFDQKAVTIGVDTGEESLIGAAYSFSSGKATSKNNKDNKDDITAHIATIYGKYAI
TDKTFVSAQGQIGTATIKKQRDTGTSTIAKANPKASILGGKIELGYVMTLADNVAFIPTI
GFDHASVKVKGYTIKGGTNKSVGKMTETRTSVIGGATMQYVHDTGHMKFSPEIHVNGKVA
LSAKNSDTKIKFGPLAPVTTPSEEAARASYNIGASLKVARCDMLEVSAGYDFDASEKFTS
HTGTLKLRVNM

>gene_1347|GeneMark.hmm|82_aa|+|8464|8712 >NVVL01000058.1 Rickettsiales bacterium isolate NORP64 Contig_source1382A_23838, whole genome shotgun sequence
MAREVSSFDAKTHLSNLLSQVQKGQEFVITKHKHAVARLIPIKKSAKIRNTKQIINEIQD
SSKSYSLNLAQGAPELKSIGRK

>gene_1348|GeneMark.hmm|708_aa|-|8727|10853 >NVVL01000058.1 Rickettsiales bacterium isolate NORP64 Contig_source1382A_23838, whole genome shotgun sequence
MQGIKKVCVIGSGVMGSGIAASVANSKTEVILLDIADENPTEDKNSNDASSIARAAVERM
FSAKPQPLLHPDFAKYITPGNLRDDLELIKECDLIIEVIPEKLPLKHKLYDSILPYLKES
AILASNTSTLPARELKKNLPENVQDRFVITHFFNPPRYMELLEFVSDDNTSADAIKRISE
FVTVKLGKTIVACNDTPGFIANRVGCFLLEMVVRKAIERKLDPVKIDQIFNKLLGFPKTG
IFGLYDLIGHDVMGLISESLKSSLPKGDKYHDISKGSPVLRKMATKGLLGRKSGAGFYKL
TKNNGKRTKEVIDFTRMKYSVVSTKTIPKTIAELLEGNNKYSKFFTEILADFFSYAISLV
GVVASDSGDIDKAMRLGYSLKYGIFELLEERIPSGMDWLRKASKNDALHYEPSSALRIDA
WYDIAKNVTSNDSAMLLDYKNQHIFVITSKMNSLNHDVFNLLIDSVARSEDTGKNLYICQ
SDAPNFSAGADLKFFYDNIEESNFKAIEDFVELGQKAMMALKYSKASIISCAHGVALGGG
CELLLHSDFVVAHQNLSAGLVELGVGLVPGWGGVKEMFLRSGGDEQKLLQNLSNILLQSK
SRSAEYFAQEYGVDSLINMNKSLILEEALAIKVPKKPKLNLVNLPPISLAASIDSTNFDE
LQLSVMDFFQQIIDLKIIDEFSLLELEKEKFLRLAAKPLCLKRLNRFV

>gene_1349|GeneMark.hmm|143_aa|-|10888|11319 >NVVL01000058.1 Rickettsiales bacterium isolate NORP64 Contig_source1382A_23838, whole genome shotgun sequence
MRNDLGVKLIAVLDINSLKLYKAQGLKIIDEVGCFLIHSDVNHKTEKHEGFKGQKSTPSS
FYDPHSSAKDIEYRESSKVAINHIKKIFASDREYKELFVVAEAKMLGHLRQYLTSGLKKC
LNKEIKKDLIHHKMKDVEKAVFA

>gene_1350|GeneMark.hmm|70_aa|+|11435|11647 >NVVL01000058.1 Rickettsiales bacterium isolate NORP64 Contig_source1382A_23838, whole genome shotgun sequence
MSGRIKIIPVFYKFCKQRGRFPKVSLIISLKLIDIFATVVNMRASAAWQRHFHPSIVNAL
TINLRFCNKS

>gene_1351|GeneMark.hmm|417_aa|+|11644|12897 >NVVL01000058.1 Rickettsiales bacterium isolate NORP64 Contig_source1382A_23838, whole genome shotgun sequence
MTNRIKETKETEATEVTEDAKIAKATAISLEDVAVSKASKRSKATKTPKKPKSYPKSSSN
NNKKMTPHDKLFKSVFDNEANGRAILEKHLPEDLKKHLDLDHLEIEKDAFIDEDLKAKMS
DIIYKVKTKNPKRDGDVILFLYEHQSSNDKKMMLRLWRYAISSWEPYLARGKKLPIVASL
VLYNGKGKYTSYRRFWDLFTNPELAEKIMSSECGLIDLQSMPDEEIFADKEFGLFKYFMK
NIRRKNVIELWKECFEKLPEHIEIDKKQKLFYIKRLWSYTEDKVPIEKKKELTSFILKQI
KKMGVNGVKTMKTIADGYREEGLEEGIFIGELRGQKIGEKIGEKIGEKIGQKIGEQIGQK
TGKKMGAEMERKKTVIRMLKGNLDPKFISSMTGMSQGAISKLRTQLKFEGKLEQDIA

>gene_1352|GeneMark.hmm|433_aa|-|12894|14195 >NVVL01000058.1 Rickettsiales bacterium isolate NORP64 Contig_source1382A_23838, whole genome shotgun sequence
MFYIPIISSAILAVILLYFIYSNVTLRAKMKFLEQVTAESSLKNTELESEKIRHIQKIEQ
LSGSLEHQKQFIADFEKLRAESHNSTKAALFEMGGELSKQLIDMHKKESKEHREVSDKSV
KDASVKFNSEFERIVNMVGALSKEVSQSKDTVDVIKNSLLSPSGAGCLAEITLENILNAS
GLRKSLDFIMQYSVVGEEKEILRPDAVVFLPSDKLMIIDAKASKFLVDDQGDLKNLARTM

NLHLRSLSSKSYSEVVQKSLKKNRIAVGNVVTLMFLPSEHAIEKLIEADTEFMTKAWKSN
IFPVGPAGLMNMLSFAKFQISETMMAHNHQQIIDEVKKLIASIGSMAEHSMRLGGSISSL
VSHYDKFAASFNRNFLSKARNIGKMGIEEALKKQNSLQRMQLVASQPELIEIEAENEIGS
EVGKIEEKLREQA

>gene_1353|GeneMark.hmm|271_aa|-|14213|15028 >NVVL01000058.1 Rickettsiales bacterium isolate NORP64 Contig_source1382A_23838, whole genome shotgun sequence
MSRTKKHRIFVVGNEKGGAGKTTCSMHLIVGLLDRGYKVASIDTDSRQHSLTTYINNRKE
YNQKNPKSRVAMPLHFHLKESVHKLLEEKNKHDQIQFDQAFNLAKKHADYIVIDTPGSHT
HLSCIAHSYADTVITPVNDSFVDLDVIAKIDTDSLEISKPSIYSQMLWEQKMERAERDKG
TIDWVVVVRNRLSNLDAKNKRNVSDALTKLAKRISFRVIPGFSERVVFRELFPYGLTVLDL
KKANFIKSLSISHVAARQELRAFLDSLDIRS

>gene_1354|GeneMark.hmm|504_aa|-|15015|16529 >NVVL01000058.1 Rickettsiales bacterium isolate NORP64 Contig_source1382A_23838, whole genome shotgun sequence
MLNISFENQDLYANEAIAVLIDDQLKMDSSAMEIDQKHHGLISKLIEDANKFKGETGQAL
TLTTSDKEGNLRNLIIVGIGDASKLKAYELEEIGGTIYSQAKACRASSAGVAVGGHIGEF
AESEASALLASGALLAAYKFDKYQTKQKDEDKCVTDEFNVVTGDHDAAYKAFEVKKSIAM
GVYFARDLVTEVPNVLYPESYAEQIATRLEPIGVDVEVLGEREMHNLGMGAMLGVGQGSA
RESKLVIMRYNGAADDVKPVCFVGKGVTFDTGGISIKPSANMGDMKYDMGGSASVVGAMK
ALALRSAKANVIGIVGLVENMPGGNAQRPGDVVTTMSGQTVEILNTDAEGRLVLADCVTY
LQEKFDPECVIDLATLTGAITVALASTYAGCFANDEELAKNLIASSVESNEKLWRMPLHK
DYDEMIKSPIADMANIGGARGQAGSSTAAHFIGRFIKDGVKWAHLDIAGMAWNSSDKNPI
CPKGAVGFGVKLLNQFVQDHYESD

>gene_1355|GeneMark.hmm|330_aa|+|16903|17895 >NVVL01000058.1 Rickettsiales bacterium isolate NORP64 Contig_source1382A_23838, whole genome shotgun sequence
MKEEIKKNEKSTKSNNLNLLKKAIQKEIIGQESLIENLLVCLIANGHMLIEGMPGLAKTT
AAKAIADGIEGEFHRVQFTPDLLPSDLTGTEVYTHETGEFSFREGPLFNNIVLADEINRA
PAKVQSALLEAMGERQVTIGHKTYKLPELYMVLATQNPIEQEGTYSLPEAQLDRFLMYVV
VDYPTFEEELQIIALDENKLNNKRKKDLLAVTTQEEIFSARACANDMHISDSLKSYAVSL
VMATRTPKEYDDMLAGWIMHGASPRATLALIRCSKALAWLRGDEYVTPSHIQTVAPFILA
HRVILTFEAEAKGISRNRIIDRLLEVVAVP

>gene_1356|GeneMark.hmm|297_aa|+|17909|18802 >NVVL01000058.1 Rickettsiales bacterium isolate NORP64 Contig_source1382A_23838, whole genome shotgun sequence
MLYPDFNELLQLRSKVTRSSLVSDQRVKSSQVGDHLSPFRGNGMEFEEVRQYVIGDDIRK
IDWLVTARLGLPHIKVFKEERQLNVTLCVDMNHTMRFGTRGTFKSVQAARVAALLGWCAN
VNNDSVGAYLFGDLPDGAEFFRPKSSRQSLWKMLKSLSDDSGAAGGEYVPISSSIEFLHK
ALQPGGLVFIISDFMNIDDEFRQQLCYLTRRSQVIFISVNDPSDMEIPNAGNILFAENDS
RKIHISTASKSGAEAYRKQWQENRSKLERIASDLGIRSINIRTNSDVFYDLFYGLKL

>gene_1357|GeneMark.hmm|185_aa|+|18806|19363 >NVVL01000058.1 Rickettsiales bacterium isolate NORP64 Contig_source1382A_23838, whole genome shotgun sequence
MEGITKNKELHGHLAKQVNETLLARLREIDGLDAANWWPLAPIWWVIIVAMLAGFAFLLI
RYLKLRAWRLSWKSDVFLELEQMQENLTIENAQNTATLLSEIIRRIVMHNHSREECASLE
GEEWLQWLAKHGDTMGAVNNTDNSTFDWRDNGRVLIEDIYAPSFSPDAMRRLANLINAIK

DWVKK

>gene_1358|GeneMark.hmm|337_aa|+|19364|20377 >NVVL01000058.1 Rickettsiales bacterium isolate NORP64 Contig_source1382A_23838, whole genome shotgun sequence
MFTFYWPLMVLLLPVPLIIWFFLPPDKNANSDSSPELLFPYVQRLLTAFERKEVAPSDSK
KYFIIMLSLLWASLVIALMRPQWADQMEYDHKKGYDIMLAVDVSASMEALDFSTLTKSIS
RLDVTKEVVSNFAGRRKGDRLGLILFGEHAYLQVPMSFDTLAVSKMLNNAVSGMAGASTA
IGDAIGIAVREIRSRPKKSRILILLTDGADTSSNIPPIEAAKIAKHYGIRVYVIGVGRSG
PVPYPNQFGQISMVEIGMDEEMLAKIAAITGGQYFKATDSTALEVIYDKIDKMEKIEINM
LGYKIRQPLYRYPLGFACILFLALCLMPLFKRSRYGV

>gene_1359|GeneMark.hmm|618_aa|+|20367|22223 >NVVL01000058.1 Rickettsiales bacterium isolate NORP64 Contig_source1382A_23838, whole genome shotgun sequence
MEFSQSMEFSQFHFAHSYYFLGILIIPIIWWLYLHYYENNANTKDLENFADSHLVPHLLR
SSKTGQISVVRSLVLGSVLWLLLMAAMAGPRWDFKEVETYKDDKSLVILLDLSKSMDASD
ITPSRLVRARQEIEDIINSSKGVKVGLVGFAAGAHMISPITDDMNNIRRLLPLLGTDLVF
VQGTSISPAFKTAKQMLLAEPGVNKSILVISDGGFQDDNHLSIVKNLAGLGVVTHTLGVG
TEKGVNFIGADGQPMMRGGQVLTSRLEKEKLEATARAGSGEYFNTHYSNENAKNILKLVE
GSMFGAGNKHANIHHWEERFYIFLAPLMLVLLLWFRKGFTFPILLLVLLTPAPQVLGASL
VDKIFLNKEQLAKKSFEEVDDIGSALAAFDDPYKRGVAYYKAGMFKKAAEKEFRKNKRPEV
SDSALYNLAGALAHQNKLDESIETYKKLLKKNPNHKKTRHNMGVVRSMVFVKEEKKEQEE
KKKPPKNEDDEKFPSGGGGGGDDEDDEGESGDESDDSGKGDEKDDSSGEGDDSDKDKGGS
DDDGDKDSDSKDKKDSDGADEKKGDKDEDTSGDEASGKDEAEIDQLLDLISNDHKNFLKN
QFQIESGNSKTKPTNDPW

>gene_1360|GeneMark.hmm|615_aa|+|22217|24064 >NVVL01000058.1 Rickettsiales bacterium isolate NORP64 Contig_source1382A_23838, whole genome shotgun sequence
MVKKLFLFITLLHCLCSLALAAEFTATIDRGTIALNQKLVLRLRLVDASPKSSPDFSGIR
KVFDILGTSRSTRSQWVNGQSSSLTEWQMSLAPKKIGSVSIPSISVKTGDGVLKTKQLAV
KIKKAKPTPAKDEKIGVFIENSVSRKKPYKYEPFIYSVKLYTADAAENIRFEPLKLEDVS
VKQNGSHSIYLSEYKGVEMNVIEVSYIITPLKHGPITIPKLKISGMHISMQGNRFDGIFG
SFFGNQAQGDLFTRKSREFLLQSKEIKLDIQPPQKGATPWLPAQSISLKESLSSDEFRVG
MPITRSITTIATGLTGKQLPEGGAGLRDNGDFQIYSETPEFTNNIKGSLISSSRKDTYSI
IPQKAGEITLPVIRLKWWNVLKSRPESASVPARTIKIAPSRMITAPEEDKAGKPGSSENA
IKIGDNSASNSAWGFGADGGFANVLAYLFLAIFGAGGVFLLYHFVDLSSLLKLLQKFRLT
PVNSKEMAGGQAVLAQGQAVSDIIAKPAIALKDIDSVSSARELYEFLNKYAHYHFQTPSN
SSLGKIFASMEKSCADIKEEQYKNIIQKLEDSLYAGKEIDLASVRLECAELLKLAKKKQP
SAKKRASSELPKLNP

>gene_1361|GeneMark.hmm|348_aa|+|24077|25123 >NVVL01000058.1 Rickettsiales bacterium isolate NORP64 Contig_source1382A_23838, whole genome shotgun sequence
MHIVNIIYNKSDGGLEKIFLDYNEYCLQLGHKVTAIAPPDAMINPAIRAQQVPIIPSNLI
ASSIHNKNPFAALYLRRIFKRIKPDLILIHNARTLELIQKAAKGICSVACVHHGGKTKRL
MRAKNVICVAKYMIEELRISGLENANFYYVPNSIKLTRNFTQAPLPKGNFPKGKCPVVIG
YIGRLSHEKGVDILLSALTKLEIPYKALIAGDGPLRVELEKSAKDHNLSAEFLGWISSAQ
KDDFFNKCDMLLVPSRQEPFGLVILEGWLYGVPVISSKSKGGCELIQHKADGLLFDICDE
KSLREAILSMDKTTHQRLINGGRAKLERLYTPDHARELFDSAIGQLLN

>gene_1362|GeneMark.hmm|63_aa|-|25152|25343 >NVVL01000058.1 Rickettsiales bacterium isolate NORP64 Contig_source1382A_23838, whole genome shotgun sequence
MTSNNKKYTIYAEEKEISSAFESDILAASDDSNIEKVHAMAAAKHFLKKSERINIRMNAF
RSY

>gene_1363|GeneMark.hmm|85_aa|-|25541|25798 >NVVL01000058.1 Rickettsiales bacterium isolate NORP64 Contig_source1382A_23838, whole genome shotgun sequence
MENEENADDINTVSRIAGAVAKGLFFALCAAYLFANMSRIFDGKIAASITGALLMLGVWA
MYNSCDPYEDDLLAGNLGNDDAEEV

>gene_1364|GeneMark.hmm|312_aa|-|25892|26830 >NVVL01000058.1 Rickettsiales bacterium isolate NORP64 Contig_source1382A_23838, whole genome shotgun sequence
MKNYTIIIALALILSSCNATKSPAPIEYNHPGSTLGGRPEASDDEIISRELASMMQPETI
TPYEGGARTGLQQSSIVDEAIIIYHEVQVGEELKDIAQKYKQSASDIARLNNLYPPYDLE
EFQIVKVKATREFTQNTSQYSGNQDLAEMKGDSLAAESKAASGPAKSASDLADGQKSVAT
KPADKLDFIAPVKGRVLTKFGKKTKYGINKGLNIAAKQGTKVLAATSGRVIYSDYDATFG
YLVIIKVHNKNIVTSYAHLEDLILSKGESVQQGNVVGYVGQTGKVAKSQLHFAIREGKIA
KDPLEFVSYSEK

>gene_1365|GeneMark.hmm|145_aa|+|26914|27351 >NVVL01000058.1 Rickettsiales bacterium isolate NORP64 Contig_source1382A_23838, whole genome shotgun sequence
MPNYDLAIASDHAGVKLKAKIMASLKAKAIKTLDLGANASDDRVDYPDYAKKMVEEILEG
SPESGILICGTGIGMSIAANRSSEIRAALCITEPMAELARLHNDANILVLGSKLIDDKLS
LKIVDKFLSTKFEGGRHARRIAKIS

>gene_1366|GeneMark.hmm|124_aa|+|1|375 >NVVL01000059.1 Rickettsiales bacterium isolate NORP64 Contig_source1382A_23852, whole genome shotgun sequence
DELVSFMLKQINKIGNNGEKVMKTIADGRREEGWKDGLNEGISIGEERGEERGEERGKKI
GEKIGEKIGEKIGEKKGVEVERKKTVARMLKENFAPKIIASVTGMNQRAISKLRSQLELQ
GKLV

>gene_1367|GeneMark.hmm|466_aa|-|456|1856 >NVVL01000059.1 Rickettsiales bacterium isolate NORP64 Contig_source1382A_23852, whole genome shotgun sequence
MKYDEQLLDAVLRRDFGSFIGKVFHSINPGASYHANWHIDLIADYLEAVRAGEIKRLIIN
MPPRALKSVCVSVAWPAWLLGQDPAARIMAASYSSVLSVKHSLDCRLILSSNWYRRVFSK
TILSKKHNQKSKFLTTQNGFRFATSVGGSATGEGGDVLIIDDPHNPTQINSLKMRSRAIE
WFEQTFVTRLNDKNKGAIVLVMQRLHEDDLSAHLCSSGGWEVLKIPAIAPSDMVYDIGKN
SYKYKQGELLNGKRDKLEFLHNIEQEVGSRNYAAQFLQEPLPAGFNLLKADDISYFETLP
VGFEYFVQSWDTAIKTSEKADYSVGTCWGVLDKKYYLAGMVRRKMSYPDLKREIEKQANK
YSPRHILIEDKASGQSIIQDLKLSGYNNIVPITPKLDKVTRFASIVALFQSGQVVLPEKA
GYKRELISELTSFPNVAHDDIADSISQFLNFSKNMRDSTKLRVRLL

>gene_1368|GeneMark.hmm|86_aa|-|1874|2134 >NVVL01000059.1 Rickettsiales bacterium isolate NORP64 Contig_source1382A_23852, whole genome shotgun sequence
MKPTIEKLYGFIARLVSMLEEELDELGANKSKSAITVKRNITDTLNKLVTLIIQLNKLSK

DDMTQNNESLPSEDAEIIERFLKKYQ

>gene_1369|GeneMark.hmm|397_aa|-|2136|3329 >NVVL01000059.1 Rickettsiales bacterium isolate NORP64 Contig_source1382A_23852, whole genome shotgun sequence
MLKTYFKNLTKRRSEKSKSFLDIPFLRGESDDRVGAESYKNNAIVHRCVSLIASSASHVP
WQIYKNNNGERKLQKNHPSVRLLKNPNPEKSGADFFTQSISTLLLYGSSYMLLGFDKKGK
PARMYNLHPNNVEFVHSKNHLVGYRHRAGGQEQIFHIDPATRMSHILHLKNYNPTNPEAG
SSSLSAAGKSINLHSKIMDWNKSLLRNAIRPSGALVFQDGNGYLTDEQFSRLQQQFYDNF
SGSSNSGKPLILEGGLKWQETSNAEKFEKFIDLKDSSAREIAIAFNVPPQLLGITGDNTY
SNMQEARFALWEENLMPLLDKYADALGSWFSCWFGEELIVDFDRDSISILGERRENLWAK
IASADFMTINEKREFVGLDPIQGGERLNSHVNNDNEQ

>gene_1370|GeneMark.hmm|424_aa|+|3577|4851 >NVVL01000059.1 Rickettsiales bacterium isolate NORP64 Contig_source1382A_23852, whole genome shotgun sequence
MNLQKIFADRKQRQIFILGIYSGMPLSIIYSTLPGWIKGAGVDIAIITTLALARIFYSLK
FLWAPFVDQIRLPILHKLGRRKSWMCLMSGIIAFIIFSYSRLSPEESFSEIYILTLALGF
CSATLDIAIDAFRIDTIKKEQQAIAAANAVFGYRVGMLVSNAGVFFMIEDYGWEVAFMMV
SSLYVAGLFFMMTLKEPKIEQEEFNILTAHSWKVMTINPFMDFLQRKGAVLILLAVVFYK
LGDAMLGVVATPFYLELGFSYKEIAAIAKVFGLIATLSGAYIGGFVMYRFGSFKGLIIGA
IAQSITNLSFIWLNHMGHDTSALMVTIVIENIASGMGDAALVGYLSHLCNKQYSATQYAL
LSSASGLFSHSIVTFGGTMVKAMGWNMYFLMTVFLAIPGIVLLLYLNKKDGFADYDKATI
KRSS

>gene_1371|GeneMark.hmm|59_aa|+|5018|5197 >NVVL01000059.1 Rickettsiales bacterium isolate NORP64 Contig_source1382A_23852, whole genome shotgun sequence
MKCCIRIILSIISLFVVLSCTPGAPYEVRSPCVSSGDSGHTYGVNPCVRRPLNMKYAIV

>gene_1372|GeneMark.hmm|236_aa|-|5234|5944 >NVVL01000059.1 Rickettsiales bacterium isolate NORP64 Contig_source1382A_23852, whole genome shotgun sequence
MIDTIMLFAAGLGNRMRHLTENSPKTLIPILGKPILHHALEMCKSYPFKKVVINTHYLHE
KIEQAIKDFQDSNPDFPEIVVIYEEELLETGGAVKNAQDILGDKPIFTLNTDIVMQSDDN
IFNYMNSHWKPKTMDFLLLMQPYDKSVGYSGHGDFDLDSDGRLIRPDKEEHYNYMYAGLQ
ILKPEKVARHPLKIFSLREYYLNSEKVIGLPAKNVRWYHATKPEDLIDIEMDMLAN

>gene_1373|GeneMark.hmm|369_aa|-|5952|7061 >NVVL01000059.1 Rickettsiales bacterium isolate NORP64 Contig_source1382A_23852, whole genome shotgun sequence
MPAYVAERGALLKEFIQDKFLQIDKIEGILGDAGLRSYSRIYSGGESYIIMDCPPDYCSV
QPFMDVAKYLAEQGFSVPKIIHHDVEHGFLALEDFGSVSVKDYLLQNANPEEHKRIYHLI
IDLLIDAQDKSDVGNAEIGKLVSGSVEPGKPGSGKPGSASLGKSNPALDSCAANKSESTF
GTFDNELLASELQIFIDYYIPYAYNRELSIGELEEFMEIWSNILASQDPMPDSFIFRDYH
VENMMYLEHRDSIKKIGLLDFQDAFLGSPIYDLVSVLEDARFNVPREDALAYIGYFTQKK
ELNLEHVLRNYHILGAQRNSRILGVFARKASRDGDDSYIKYIPRVLQYLDYDLSHSSLVV
LKAWLEKLK

>gene_1374|GeneMark.hmm|424_aa|-|7066|8340 >NVVL01000059.1 Rickettsiales bacterium isolate NORP64 Contig_source1382A_23852, whole genome shotgun sequence
MSKESSKKSSHCSFCGKGELEVKKLVAGPAVFICDECIVLCRDILKEGAKEEIEEKFSSI

PTPKEICKTLDDHVIGQDRAKKVLSVAVYNHYKRLESVLADDDDGVELNKSNIMLIGSTG
SGKTLLAQTLAKILDVPFTMADATTLTEAGYVGEDVENILLRLLQASDFNVEKAQKGIIY
IDEIDKIAKKSDNVSVTRDVSGEGVQQALLKIMEGTVAHVPPQGGRKHPQQDSIQIDTSN
ILFICGGAFMGLEGIITARGDNGSIGFAAKVQSKEEMRHSEILKDLQMEDLIKFGMIPEF
IGRLPVVASLKDLDKAALIQILTEPKNAIIKQYKKLFDINEANLEFTKDGLEAIAEKALK
RNTGARGLRAIIEEILLEDMYNFSELTNKTVTFNKESVKSSSSVIVNGRKKPANSSMSKK
PIAV

>gene_1375|GeneMark.hmm|202_aa|-|8405|9013 >NVVL01000059.1 Rickettsiales bacterium isolate NORP64 Contig_source1382A_23852, whole genome shotgun sequence
MQVGYVPIVVEQTAKGERSYDIYSRLLKERIVFVCGEFEDHMANLIVAQLLFLEADNPEK
DIYMYVNSPGGVVSAGLAIYDTMQYIKPKVATLCIGQACSMGATILLAGEKGMRYTLPHS
RVMIHQPLGGFRGQATDIEIHAKETMKVKATLQEIYAKHTGKTVAHMAKIMERDHFMSSA
EAKDLGLVDKIIEHRDLIKTEK

>gene_1376|GeneMark.hmm|139_aa|-|9162|9581 >NVVL01000059.1 Rickettsiales bacterium isolate NORP64 Contig_source1382A_23852, whole genome shotgun sequence
MLIVKDKLTISIELIQGVFMGRRIFLLIVITSLVWGGDWLYRAYEVKESEIPTSKISDLK
VILYSKEICAYCQSAKNLLDKKGINYEVVELTNNKDLHIKLINQTGQSTVPYVFINDKFI
GGYQNLLELDEAKGLECSD

>gene_1377|GeneMark.hmm|778_aa|+|9694|12030 >NVVL01000059.1 Rickettsiales bacterium isolate NORP64 Contig_source1382A_23852, whole genome shotgun sequence
MLSKNLERTLRKALSIATEHKHEYATYEHLLLAIMGDRDVRLVLVDRLSCITDIENKLKT
YLKNNLVDLVDKNIKDSMPTAGFQRIIQRSAMHNQTNGQLPLTAVHVLAEFFFEHDSYAL
LCLKEFNLNRKDVLDYAKRLDDIHKTLYPQNTPPHRALIANVAKDKNASPKQVKSDDAKE
SDLEKYCVNLNEQAGSNSIDLLIGRENEIQRTIEILCRRKKNNAILVGEPGVGKTAIAEG
LAVKIVNKDVPDILKDTVIYSLDIGSLVAGTKFRGDFEERIKSILNTLKANKNIVLFIDE
IHTIIGAGSTTSGAMDASNLLKPALAKGDLRCIGSITFKEYHNHFEKDMALVRRFQKIVV
EAPSEEESFNILQGVKGYYEKHHNVTYTDEALKAAVHLSERYINDRHLPDKAIDLIDEAG
ARSSMAHDPEDGTIITEKDIEVLLSAILNIPNLTIEADEIKQLKHLSSDLKRRIFGQDEA
IESLCGSIKLGVAGLRKGTRPTGCYLFTGPTGVGKTELAKQLSNCRNMKLLKFDMSEFVE
SSSISKLVGSSPGYVGFDQGGLLTAEVDKYPYSVILFDEIEKAHPDIFNLLLQVMDEGTL
TDNTGKHINFTHSMIILTSNLIAGDTKAPIGFSNKPAGQEEKKEPNMENINEHFSPEFRS
RLDKIIIFNPIDGIVEKIVSKNLKELASMLADKKVRLTISKSVIKYFVENCFSNASGARG
LDRMIDTKIKQKIADEILFGKLKQGGTAILDFSKKDKEITFKFSKVKKTTKNEQCETS

>gene_1378|GeneMark.hmm|137_aa|-|12176|12589 >NVVL01000059.1 Rickettsiales bacterium isolate NORP64 Contig_source1382A_23852, whole genome shotgun sequence
MKNKRNIDLTLPAEQLRNALSSLKSALDTPADEDRFLIDASIQRFEYSIELFWKFLKKIL
QREGRPVQLPREILKESFQAHLIDNEQIWIDMLIDRNQTSHTYNEDLADEIYQRIKTTHL
SEMLTSFESLVKKYEIF

>gene_1379|GeneMark.hmm|100_aa|-|12579|12881 >NVVL01000059.1 Rickettsiales bacterium isolate NORP64 Contig_source1382A_23852, whole genome shotgun sequence
MQLPHDISHYKFWQQLASLPFLDKIYLYGSRARGDNLERSDIDLAICCPNAKKYDWQKLV
YIIDNSDTLLKIDCVNLDNLNDDNQLKQNILKDGVVLYEK

>gene_1380|GeneMark.hmm|514_aa|-|13018|14562 >NVVL01000059.1 Rickettsiales bacterium isolate NORP64 Contig_source1382A_23852, whole genome shotgun sequence
VQKISKWKMMIAILVTITSIIYVTPNFTTTKSSWLPEDTVNLGLDLRGGAHLLLRVDFDK
YMDDVSQSVAESLRKNLRSDKIRYRNLHVSRNKIQFDLRNLDDQAKINKLARTIDANLSA
VKEEGKITLFYDEYAINQLQDKVIDQSIEIIRMRVDSTGTKEPSIQRQGTQDILLQVPGE
DNPAELQRVLGQTAKLTFHLVDEGANLERARSGHAPTGSMILKSGDDQEGVLVVKKKIIV
QGDQLNNAQAQFQEGEPVVHFSFNHLGAKKFAEATTKNRGRRLAIVLDGKILSAPVINGP
ITGGEGVVSGNFTVEAATELALMLRAGALPAPLSVIEKRTIGPNLGADSIESGKMASIVA
FGLVVLFMLLSYGILGLFANITLVLALLYIFAVLTMLQATLTLPGIAGIILTIGMAVDAN
VLIYERIKEESAKGCSNLYAVKVGFESAFATIMDSNVTTLIAAFLLYTFGVGAIKGFAVT
LTIGILASMYTALVITKLMIDIWLKLCNPKSLGF

>gene_1381|GeneMark.hmm|149_aa|-|14593|15042 >NVVL01000059.1 Rickettsiales bacterium isolate NORP64 Contig_source1382A_23852, whole genome shotgun sequence
MISSAHAREETDTITIQNSEGAETSMPSMESGFASLVPMLLIFAVFYFLLIRPQDKRRRL
QEALVLGVKKGEDILTNAGLFGKVMSINDSDNTIMVRVAKDVEMKMLKSSIADIVSRTKP
DGDKKDSKKGDKQEADKKNPATKAKPKKK

>gene_1382|GeneMark.hmm|587_aa|-|15124|16887 >NVVL01000059.1 Rickettsiales bacterium isolate NORP64 Contig_source1382A_23852, whole genome shotgun sequence
LILERKLGAKILITRLIKDHVSLYKKKIFTAIFFMVIVALCSAAVVKLVQPAIDLIFVEH
DREMLLLIPLVMLAIYAIKGVAEYFQNYLIKYIGQKILTNLQMRMYEHLLSADFLFIQSQ
SSGRLISRFTNDIMLMRGAVSNMLVGCAKHFLSVFFLILIMFSLDPFLSAFIFLAFPAAI
YPIQKLGRRMRKVTSEAQQELGNFTAMLDETFDSIKVIKSFCSEKIEVTKAQKITDTILE
LYKKTSKLDSLTSPIMEILTGFAITCVLWYGGSEVIEGRMTTGALMAFITAFVSAYRPFK
SLVALNVNLQEGLAAANRVFNILDSAPSVADAADAVSPEFAKAGIEFKDIEMNYGNRVGI
KSLDLKIKPGQTYAFVGKSGSGKTTLANLLVRFFDPTKGQILIGGHDIKQVSLQHLRNNI
SMVSQDTILFDASVAENISYGNKKASREDIINAAKAADAHEFIISLPNGYDTAVGTAGSS
LSGGQKQRLSIARAFLKDAPILLLDEITSALDPNSEQSIINSLERLRKDKTTLIITHRLG
GITNAHQIVVMKQGKIIEQGKHKELLAKGQEYYKLYNKELKDTTKAV

>gene_1383|GeneMark.hmm|252_aa|-|16968|17726 >NVVL01000059.1 Rickettsiales bacterium isolate NORP64 Contig_source1382A_23852, whole genome shotgun sequence
MTKFSVKNYREPIGKFKEINIVSDKLPPKILVIEPDLTLNANVCNAIERYWFDVIRTSDA
DAALRISEINPPNMAIISSRIKDMSAIEISVKLRKIAELQDLPLLFLIEKDEPESNYNLP
NSGYMELLHRPFTPNELMISIRSILRKSKPVFQDKIIGYDDIKMDLSTYQVFRGDKRVHL
GPTEFKILQLLVLKPKNIYSRRQIIDYVWGADKEIAPRTIDVHINRIRTMMKEEKDKHLL
IKTIRAAGYCLD

>gene_1384|GeneMark.hmm|129_aa|+|17921|18310 >NVVL01000059.1 Rickettsiales bacterium isolate NORP64 Contig_source1382A_23852, whole genome shotgun sequence
MAFYESIIIIRQDVSSTEVDRIANEFQQIIKDHNGRVIKTEYWGLRQLAYEINNNKKGHY
YFMGIEATKPLLDEMDRKIKLSESIIRSSLIKVDEVSKDPSPLLKVDNAGSEKTVDVTNK
TSDKKPSAV

>gene_1385|GeneMark.hmm|97_aa|+|18345|18638 >NVVL01000059.1 Rickettsiales bacterium isolate NORP64 Contig_source1382A_23852, whole genome shotgun sequence
MSREYSSNSRSAGAQTTDSRGSSKVFFRKNKGCSMCSTSAPAIDYKDPELLKGFISEGGR
MLPSRITSICSKHQRPLKKAIKHSRVLALLPFVLLNK

>gene_1386|GeneMark.hmm|207_aa|+|18680|19303 >NVVL01000059.1 Rickettsiales bacterium isolate NORP64 Contig_source1382A_23852, whole genome shotgun sequence
LEAMEVILVKPDRKLGKVGETVSVANGYGRNYLIPQELAIRATKENVEKFASLKKDLEAK
NDKNKVDAEKVAKTLEGKNLTFITQSASDGRLFGSVSAKGLAIELSKLTGTTLSYSNIKL
DDPIKFNGVYNIQLVLHAEVITNILVIVAKSESEAQDALREYLEGDKEEDDESEIEAAAL
AAAAAAPTTDAASDAEGTDADSGHAGE

>gene_1387|GeneMark.hmm|600_aa|+|19700|21502 >NVVL01000059.1 Rickettsiales bacterium isolate NORP64 Contig_source1382A_23852, whole genome shotgun sequence
MADLVEKDRSIPKLPKTLFPFIWYFLRSFKPAVIIFILLAILAGLWAPFNSILIKNVIDL
LPQAQNGDLSILLLPVSLIVLNFILFDNFTWRGITYIRCKFVPVIINRIIGQSMDYVLGQ
SHQFFQDNLSGKISKQITNLADSIEKIITSVIPNFLRGASLVLTAFVAAYFVNPVFCLIL
ITWFVCFVSTSVAMSKKLVALSDSQAAAESLVVGELVDVVSNQTNVRIFSKNSYENLRMT
PFFNAQQKTYHNTYFYALIMHSIQGVMIAIMMGFSGYFLLYLYGKNLVTIGDFALIFGLT
METGHMMWFTMSELDEFNKALGRCKQSFFALMTPLEIQDKSDAKLLESCGGQISFSKVKF
HYKGSEPIFQNKSINIKSGQKVGLVGYSGCGKSTFVNLILRLYDVTEGAILIDGQDIRDI
TQDSLHENIAMIPQDPNLFHRSIMDNIRYGRTRATDEEVIESAKKAHAHEFIIKLPQGYS
SLVGERGVKLSGGQRQRIAIARAILKNAPILILDEATSQLDSVTESLIQDSLWELMQGKT
TIVIAHRLSTLLHMDHVLVFDQGKIVEEGTHGSLLEKASLYKKLWDAQLGGFLGDHSKED

>gene_1388|GeneMark.hmm|360_aa|+|21670|22752 >NVVL01000059.1 Rickettsiales bacterium isolate NORP64 Contig_source1382A_23852, whole genome shotgun sequence
MDLQDYRINERLNLKPEIVEEVYSIIAEIDAVKNSWQITDKIFPQNIERLTRSVIVTSTG
ASNRIEGNRLTDDEVENLYKNLRVKKFKTRDEQEIAGYLECLEIIFNNYSDIKITESSIT
TLHHDMLTYSEKDKRHKGIYKFGSNRVEAKDNSGNIIGVIFDPTPPYLVRKEMQELVDWY
GKASKVKIKHPLITIANFVFEYLAIHPFQDGNGRTSRLLTNLMLLQQGYAFTQIVSHERL
IEAKKVDYYLSLNKTQSTWKTEKEDVSSWLLFFLNIVKSQAIQALSIIEGDDIEYLLSEK
QLAVWNWINDNKMEFSRKDIIAALGLAPRTAESIVKKFVDMKRLQSLGQGKATRYKLIHR

>gene_1389|GeneMark.hmm|559_aa|+|22844|24523 >NVVL01000059.1 Rickettsiales bacterium isolate NORP64 Contig_source1382A_23852, whole genome shotgun sequence
MKISLCQTAPRTGDLAGNFADIWQHYLAALNKEADICMFPELSTSGYLAEDLFLKESFIQ
DVWARARELIKNTGKTCLVLSMPIMQDEMLSDGAQLGSDENPIDNQKLSNHQKPGNNYSL
YNAVIVAQNGKIIGTTYKHQLPNGSVFDERRYFKPGVPAVISINGTSVGMPICEDIWSPE
VCAELKKQGAELFLVPNASPFEKGKMDERITHVQSRFKETQIPILYCNQALAQDGIVFDG
NSFCFDGGLQIVGKSFEVDSQIIEVRGNKFHPERSYDREENSYEEGSCEEIYKAMVLGTR
DYVRGNGFSKVILGLSGGIDSALVAAVAVSAVGRENVLAYMLPSNFTSQESMRDAQEMAD
SLGIDLMTISIMDAVGSLAGTLGLEEGSISHQNLQSRLRGTMLMAESNRLGALLLTTGNK
SEYATGYATIYGDMNGAFNPVKDLYKTELYKVAHHINAHHYAFPERVLCKAPSAELSHDQ
KDTDSLPEYPVLDQILERYIEQRQSQAEISKDFDPEIVERIIGLVNRAEFKRKQAAPGVK
ISSMNFEKDRGYPITNHYK

>gene_1390|GeneMark.hmm|96_aa|+|24625|24915 >NVVL01000059.1 Rickettsiales bacterium isolate NORP64 Contig_source1382A_23852, whole genome shotgun sequence
MNFRPLHDRIAIKAVATEEKTTGGIIIPDSAQEKPMQGEVVAVGTGARDQNGNIIPLDVQ
VGDIIVYGKWGGNELKVDGEDLIIMKESDVMGIIKK

>gene_1391|GeneMark.hmm|549_aa|+|24948|26597 >NVVL01000059.1 Rickettsiales bacterium isolate NORP64 Contig_source1382A_23852, whole genome shotgun sequence
MAKQIKYGTKAREEMIKGINILADTVKVTLGPKGRNVAIEQSFGAPRLTKDGVTVAKAIE
LKDPNQNLGAQLVKSVANKTADTAGDGTTTSTILTQAIVKEGNKAVAAGSNPMDLKRGID
LAVAHVLETVKKASKAISTQEEIAQVGTISANGDKQIGEKIALAMEKVGKEGVITVEEAK
NFGFEVDVVEGMMFDRGYLSPYFVTNSEKMIVELDNPYILIFEKKLSTLQPMLPVLEAVV
QSSRPLLIIAEDVEGEALATLVVNKIRGGLKVAAVKAPGFGDRRKAMLEDLAVLTGSQLV
SEDLGMKLDNVDLSMLGSAKKVKISKEDTVVIDGAGNAADLKARCAQMRQQIAESTSDYD
KEKLQERLAKLVGGVAVLKVGGATEVEVKERKDRVDDALNATRAAVEEGVVAGGGTALFY
AAKALDGVKGANEDQQAGINIIKKALQAPIRQIAENAGIDGAIIVGKLEDSKSTTQGFNA
QDMTYVDMIKEGIIDPTKVVRTALIDSASVASLIITTEAIITADPSAKEGAAPAGGGMGG
MGGMPGMGF

>gene_1392|GeneMark.hmm|129_aa|+|27002|27388 >NVVL01000059.1 Rickettsiales bacterium isolate NORP64 Contig_source1382A_23852, whole genome shotgun sequence
MSEVIEEIKRAEESVVTEENVATKTLKTNKETKATKETKTPKEADAAPAPPADRPKSTSK
NNKKRSPHDKLFKAIFGNEASARGILKKHLPEDIKKLLDLDHLKVERDTFVSEDLKESMS
DIVYKIKTK

>gene_1393|GeneMark.hmm|710_aa|+|146|2278 >NVVL01000060.1 Rickettsiales bacterium isolate NORP64 Contig_source1382A_23925, whole genome shotgun sequence
VSKKLVYFQSKGVDQLRPGSDINSALSEAVIQKNAVVVEQLLKQGADPNIMHSDGKPLLY
SAVSNKLTVIVRLLTTYGARIESVDDRIELFTDAAKHECFEMILILATPYKFDNQTMLAA
LNRVWYDDLDAATALLNLCDKTIQSYVMQLFAKPITREEFLCTLQTIKQCKINIPSQYRK
NALDLAFKYPDDQSIGALIECGISQAAIITLATRLLTSSTDRDTLKYALTTIENLVGYSG
VDFHKHGETPVFYAARQGYNWLIRMLKDYNVDIDACNIDGESPLFFAAKHGHVNVIKRLI
KYGANPNCVNSKEETPIFYAAKQRHTLIVERLIKYGADPNITNVVHKTASYYLLECDVRE
NLILAINVGYDNVVRKLLELNDTDIDYRSLLTLPDQHGKTIIYYIFRTDMVEKIKSNTQL
NQQMLELMQEYGDQLNIMLETCQIDLPTIFEYMQQHDIAFQWSVSSRYLGQQRQGSIKLD
TQHAHLGHRVAIEKFLVHECVHAVTSDYYSYNGRPYPYSSEDDPGKNSGFDILCKQVQST
IDTHKHTRLHSSNLILISFCATLCQGFEYDTESGKHTEVPARMAETMAVFGWRNTVIMMQ
YHFPKLMAYFEKLIPDQNNDFLGTLQPLLKDVFILQNIQKSEFNGYRSGTIPKLICGYSA
HEYSPQYVGTLLLDYNLSNPQNMYTDEIEMAVQWAAISGETIDVICDFVC

>gene_1394|GeneMark.hmm|53_aa|+|2771|2932 >NVVL01000060.1 Rickettsiales bacterium isolate NORP64 Contig_source1382A_23925, whole genome shotgun sequence
LHSFHKPEGALNLQGFQFKLQGARQGGVFLIDLPAACKSMFQKIVFILDKLCV

>gene_1395|GeneMark.hmm|411_aa|+|2987|4222 >NVVL01000060.1 Rickettsiales bacterium isolate NORP64 Contig_source1382A_23925, whole genome shotgun sequence
MNFLDEFKKRGYFYQCTNEEKLKKKTENQKITAYIGFDCTAKSLHVGNLMQIMILRLLQQ

HGHKPIVLIGGATTKIGDPTGKEEARKYLSDEDINANIAGIKKSLSKFIRFGTGESDAII
LNNSQWLESLGYIEFLRDMGKMFSVNRMLTMDSVRLRLEREQSLTFLEFNYMLLQAYDFY
HLNKEYNCDLQLGGSDQWGNIVTGVDLVRKVSSNESFGLTTPLLTTASGVKMGKSIGGAV
WINEEQLSPYDYYQYWRNTEDADVVKFSKLYCEFSDAEADEFENLAKDNINEAKKRLAYV
VTALCHGEDKADSAMETAIRVFENGTVGDDLPTITIDEARLASGVACYDLFLEVGLTKSK
GESRRLIRGKGAKINGEPVLDEDFVVDMSSMQEGVIKLSAGKKKHAIVKII

>gene_1396|GeneMark.hmm|342_aa|+|4235|5263 >NVVL01000060.1 Rickettsiales bacterium isolate NORP64 Contig_source1382A_23925, whole genome shotgun sequence
MKLSDFDFDLPRELIAQIPSEKRDCSDLLIPGEGGNKIVKFFNLVDCLSDGDLLVFNDSR
VVNAKLSLQKGSKAINVNLNKPSGKNSWLGFAKPSKKLEEGDEFAFGPHKLVISQKLEYG
EIEIEFRLDNISIFEFLDQYGEVPLPQYIKRPDANKEDLERYQTIYSKSKGSVAAPTAGL
HFTEELLEKIKAKGADLTFVTLHVGAGTFLPVKTENIHEHKMHSEWCEVSPDAAAKINLA
KKEGRRVIIVGTTAMRTVESCAVGGLVRSGVMETDIFITPGYEFQIADLLITNFHLPKST
LFMLICAFAGHQKMQKLYEYAINNKMRFFSYGDGMIIEKTNI

>gene_1397|GeneMark.hmm|428_aa|+|5284|6570 >NVVL01000060.1 Rickettsiales bacterium isolate NORP64 Contig_source1382A_23925, whole genome shotgun sequence
MSKKTISSKDPEFNDLSIEEQFEILTSEAEAEEEVEAAAKALDNPGSSDASGESGEGSND
ASDASDEWDAEIAELEAELAEISQNAANIPGKGSVSNSPSADKEEQTSKEAQIEDEHPAR
EEAAGSLRSNSHDTHNIEEARLANLPPAQASKQQAKQKTEPPLPEKEEEKKPGFIDRLVK
SFMDGLEWVKSKLSLTKEVHLNEGEGMPSKDGGSKKPGTTAAHQQNSEKSLAERASSLEG
VIEQRGTAINQRRETIAGLVGDRQQNEKQGRKKDRQFIEERPKDTDGVKRMVSQRLRHQR
QADLEIKKLQNLQRLEEKIQESLRQQLQQIAGLLEDEEKRKNLRHKLEQGAASTPALASA
SASPENAPRANAPRANAPRANAPRASARRVNISTQRKPMPTPHVPPSAPPKAQQPTGRSI
AGDNLRKK

>gene_1398|GeneMark.hmm|697_aa|+|6752|8845 >NVVL01000060.1 Rickettsiales bacterium isolate NORP64 Contig_source1382A_23925, whole genome shotgun sequence
MSRIATPEQRASYLESLFQGLHGDSDKIANLSRKDLYELLLHAMKQHGVEEIKSWLEAAQ
INVYTLYGQRQEDNRIEPIHVLLKAAIHAGKLKDTIECFGMDLRNPDDLFVLGQMVPCVE
SWLKRVQTRLAALDIKSLKQAIKNFSSDLTQENLANVLEIMSSDARDARDANQMTVKTLF
NFYNTFMLQEAMKAGNLLDTAEIIGGESVSDWDGELLFMLAEEAIKTGRTEELANLWGEV
DTNSLYDDGEGLLVAAIQAEDLEAVIQNLGIDVNQLITLRVCSILEEWVRDSNDNGYGRQ
VWAREIGDIGDFRTLPPELVQEVLNAKEVTKEQANTILGALGKSSEVAYNCLVHHILLAS
AAQGSLEADLAVLRVCGFPIEVGEFSDHQRLALLGSTRPDQLEDIARLLDIYLNTLPGPT
QVFILGTAIVNRDLETALNKEFITIKPSEISPDHLRELLVLAVRAKCLETAAGVLEENYL
ETAAGLLGQDLTALDSETQISVLGRAIKVKNLDLLSLFGREDVSDLGSKAISSFLHRAVS
SNVFYDIIRKWDINLLETFQGSIGDIYDNRLYTHHLAVGRILSAIPLHEFPQIEPGLLRY
VFPDDKHFMLVAREIEYRGELKTKAMYELLEPLDWMPFDLFRLLGNFLYRTKDNMNTSSL
DVEILDAAGAVALDEDGDPTFETISRIAGDLSDVDLA

>gene_1399|GeneMark.hmm|125_aa|-|9014|9391 >NVVL01000060.1 Rickettsiales bacterium isolate NORP64 Contig_source1382A_23925, whole genome shotgun sequence
VRKIMNTQHLEIINISIMNEAKELMGDRFVTMVEYFIEDSEGYLAEIEAGIKAKDEQALI
PPSHTVKSSAKQIGAERVSEVAKNIESFCREFDKSDPSSFLKLEDLAWQLKEELSMSNNE
FKKLL

>gene_1400|GeneMark.hmm|1090_aa|-|9393|12665 >NVVL01000060.1 Rickettsiales bacterium isolate NORP64 Contig_source1382A_23925, whole genome shotgun sequence
LVSYMSRKLLHIAILFLAYYIPGKLGFLLALPPDSSTAIWPSSGFASAGIVILGYAALPG
VFLGAFFLNLTHHLPILEVFSSEMFQYAPKASLISLGAMSESFAVAYIIKRFIGFPSIIS
HWRDVLILFIGAGFIGTIPSPTIGVTTLYFFKTIPLSSYVYTWWTWWIGNSLGVIAITPI
LVTMFSPSTYISNRRKLYIAIPLTTVLIIVSLAFLDTSKYEVEKLQHNLELEAKNSTLRL
KSHVYKHFKELSTLKSFYKASKFVSRSDFREFIEDIIDSCNGVYSVDWVPKVAFKDKSKI
INRAVNDGIKDFSIKEYDANGALKPAGKRSVYYPILYSESEKHSNPLGFDVFSNPKLKEL
LSIAIDTGKEVSTDLENKIDNGQSIKIFSIFKPIYINGSKTDTIENRRKNIKGFISGTFK
RGYLLDRYIKDLKSKGIEIVVFDVTKSLSGKSLDREGDTESKRNLERDLKKNPEKIDNKT
ILYESFSGKSKFLLSTQICMKFADKTWEINFQQTKKYLVANKEWHLWYLLFAGLIFEALT
GILTLIITGYSDTIENLVRKKTSDLKISETRFQLAVKGTRDGIWDWVDTSNDEQYWSPQF
FKLLGYEADEIPPKRSTFKTMIHENDLKAMQRALSDHINNGQQFDIEHRMQKKSGKYGWF
QSRGLLTIDPLTGIKRMTGSLSDISDRKNTEKKLKQAKEEAESATRMKSDFLATMSHEIR
TPMNGIIGITELILDTNLTQQQRGYLDNVLNSAETLLEILNDILDFSKIEAGQMDLERVP
FNLQEASQEVIDLLRPKAQQKKLEINLDFDKSVHKYFVGDSMRIRQILHNLVGNAIKFTE
KGSIDITVSKQDSASLPKNKAMMMISVQDSGIGLTKEQRRTIFNKFVQADSSTTRKFGGT
GLGLSICQMLVTMLEGEIGVESTDGKGSTFSFTMLLDSTTKESVAEKKMDNSVEQFNHNI
TSTVRVLMAEDNRINAEFAKEMLEKIRCEVTVVRNGQEAFEQVQKNREFDLIFMDCQMPI
MDGFEATKKICEYEKAESLEHIPIIALTANAMKGDREKCIAAGMDDYLSKPVRQKNFASM
VGKWFNKSIK

>gene_1401|GeneMark.hmm|308_aa|-|12659|13585 >NVVL01000060.1 Rickettsiales bacterium isolate NORP64 Contig_source1382A_23925, whole genome shotgun sequence
MIQKNKTSPLVLVVDDDKVMLMTLEEKVKQQKCRVLTATNGKEACEILTNTNEPINAILL
DRMMPVMDGMEVIEWLGKQTNIEKPPIIMVTGADSPEQIKEGIDAGVFYYLTKPVQTQVL
KSVLASAVEESIQQHALKNELKRHRISFKLMEKGLFYIQTLEEAENISCFLANCFPDSEK
ALPAIAELVINALEHGNLGITYDEKTTLIQNGTWRQEVAHRTNLPQNKDKKIKLLFEKKG
SEYLLKILDQGTGFEWSKFLKVNPARATDNHGTGIARANMIFSKLLYSDKGNQVTAIIDT
KNEKNINW

>gene_1402|GeneMark.hmm|454_aa|-|13605|14969 >NVVL01000060.1 Rickettsiales bacterium isolate NORP64 Contig_source1382A_23925, whole genome shotgun sequence
MLKPLIRFLSGKSKSYSKINQLRYLSQSALIEETKAPYMTRVTLVLVSFVIILLIIWAGI
TEVKEVAVTQGEVLPIRHIQRIQHLEGGVISKINAKEGELVKKGDTILVLDGASIKQDLS
ALRTKKISLEYQSLRLKSFISNTRPNFKKIVGYDHDSELEEEQIQAFDGMIEARDTEKKV
LTDQITQKKETLTSLMNRKMTLTKNIKLVKEERDLKQKLHKKGHLSKFKFLAAQKHFNDI
IGRLKKTESDIAQAKNMVSEYKNRVESLEARLIDDAHDELSQVEGDISQLDENITKFEQR
ITRLVIKSPSYGYIKVLNIKTIGGVIETGEVLAEIVPLRGGLVVEVRINPKDAGHIQTGQ
AVNVKISSYDFSRYGTLDGKLEYISATTFAADDGSRYYTGRVSIAKNYIGSNPKRNIIIP
GMTAQADIVTGSKSILIYLLKPIAVSIQTSFSER

>gene_1403|GeneMark.hmm|744_aa|-|14970|17204 >NVVL01000060.1 Rickettsiales bacterium isolate NORP64 Contig_source1382A_23925, whole genome shotgun sequence
MKGNTAGNMKENTISMMADTGSDDIIDRVNDILEMFNTHEVMPDDSKCDHVFAKALSMLM
VSLKWSGNKRHLFESLSHTKGREIDLLDILNSVANLGYTSHEITISMSEIDDRLMPCLFI

PAKRTNSPLVLLSKNEGIITAFDSRDKKIIEFAAGREKGEGYFFERINQEDLDEERKTQE
LAGMSWFDIIFSRLNPVVKEIILASFFINIFALAMPLFIMSIYDKVIGAGAIDTLVYLAF
GVSIAMISEFTLRIIRSKSVVWLGVRLDNIISNAIFERLLFMKAAYTEGASVSSQISRVK
SFESIRKFFTTPAFSVIVEIPFTIILLMAIWLIAGPLVYIPIVAVFLFILMLLYYQSKIR
VSMQAAAKASGECQRYGMETFMKINYLHHNGIANSWLQQYKEKLSKASMASFRSNITSSI
IESVAHSVTIASGLAVVTFGVHLIWAGEMSMGALVATMILIWRILGPLKTLCSMLPRIEQ
LRDSIRQINRLTNIETERSSTILKRPIEHLNGDIKLTNVGIRYSMDAEPLFVGLDLDIKA
GEIIAITGTNGSGKSSLLKLINGLYRPQTGTVRIDGTNILQMEPIELRNYIAYLPQIPNF
FHGSIKDNILLVNPLATEEDVVLALEKSGAMEMIKNLTHGIHTIIRANNPTLPNTFGYTL
NLARVFLKSGNILLLDELPNSSLNEKVGKAYKQLIIDSRGKKTVFFASQRDDFLKLADKV
ILFKPGNRPVTLKSNEFINKYGQD

>gene_1404|GeneMark.hmm|566_aa|-|17201|18901 >NVVL01000060.1 Rickettsiales bacterium isolate NORP64 Contig_source1382A_23925, whole genome shotgun sequence
MNLIDKYNLNDPACKFRLNKPELIVSSLAVNLISLALPVMVLQVVYDRVLSNNSIETLSML
TFGVFVAIILEGILRIARAYTTSWTGMMYEYTMSANAMRYYINADPAKLRKEGVGKQIQN
LTTFSKLRDFYGGQTLVTMVDIPFAFIFLGLIMYLTGKLVLIPIILILIFGYVTWLTGNK
LMDSMQQLDADDDKRYDFIIEALQGIHSIKSYGIESIFKRRYERMKEDSSRSNYKTALIS
TQGYNLGSLFNETMIVAVVAFGAPMVMNNEMSSGALIATVLLSGKLVQPIQKALFLWSGF
QDYRIATQKAEEIFSMHQIKRWGGELENEKIGLVELKDLGFTHGTKSVFSGLNLKVKLPE
AIAIHGAIYSGKTTLMSLIAGIFEPGEGEILVDNMPASSIPSAKLVHHVGFISPESVMFH
GTIMDNLTAFNEAKEADAIELAKLLGIDNEIALLPQGYETKINDGHTDTIPPGIKQRIAI
IRVLINKPKVILFNNADKGLDRDGYNCLVSFLSLLKGKVAMIIVTEDKNINRLADKHFLL
KDGSLSEIEVKDSNIFNVKPYKELKI

>gene_1405|GeneMark.hmm|1773_aa|-|19058|24379 >NVVL01000060.1 Rickettsiales bacterium isolate NORP64 Contig_source1382A_23925, whole genome shotgun sequence
MADSKSGETGNNSEASHSQEQFDRFDRLTNVDKKDLDARKMTAHEKASTLDFEEILVTSD
DITSSTEELTYSPPEKTEILDGAVDSRDIRTQHSPDELMINAQFEYNDDSSYNPPPETPI
ATFIEEDALPKEASGEIIEPSFHNAQKTEIAAGQKDANEDIIDQNEASKEQTGSEEAANK
KDIREIEDEDLLIQRNEEEVVTEDVPGDPPPAPATPPPPIPDPTNPDPTPDPTPDPDDPT
PDPDPTPNPDPDPDPEPLPDPPIPDQLGNGNYNIIPGYGISLVFDSLTSNTGYDNSFGHY
FVDGDGNPISGAIDFSNVGDTLGEGSEVIVRYESSDIPAGAEQIGFFLIPDGANLNQLSA
GDVVTFQNIGGIWTPFINGDPIQGESTPIYFSDQSLNPDGINHMNSSPDGKVGWEDMFGG
GDNDFNDAILNITVQSTTDNNGSDDIITGTAGEDVASGGKGDDILIGGAGNDLLRGGANN
DILIGNKGDDTLLGGAGNDILKGDKGSDTLKGGKGDDDLFGGSGADILKGGAGDDMLFGG
SGADTLKGGGGADTLKGGSGDDNLFGGAGDDTLLGGGGADTLKGGAGDDNLFGGAGDDTL
LGGGGADVLKGGGGADTLKGGAGDDNLFGGSGDDTLLGGGGADTLKGGGGADTLKGGAGD
DNLFGGSGKDILFGGSGADVLKGGGGADTIKGGAGNDILYGDQKSNNDTELVQLPNGNYL
IPFGVGFTISLDSIISHAGYNNSIGHYFANSNGDPIAGMIDFANVKDTLGVGEPSVSVYA
PENLPSGVVEVGFFLIPNGDRLNHQISDGDEVTFTLNNQGEWTPVLDGTELKGQGDPVFF
SDSALNVNGFDYTEIIDQNPLQIGFEDLKGGGDNDFNDVVVNVDIKQNSTPEDDILIGGK
GDDILFGGAGIDTAIFKGNADDYIITHNKDGSITVEDTVPDRDGTDIVHDVEKLEFKDTT
IDVDDIDPPAPPPPPPPLPDPSFSGTSDEYIFTSNGDGTFTASDTVPARDGDQTFQGGAD
LDFTDKTYTTTNDTIIGTDGDDVMRGGAGDEIFKGGKGNDNISGGAGNDTILGGKGDDTI
DGGDGADIIYSGDGDDIVDAGAGDDIVIGGLGNDNIQGGAGNDVINAGSGDDIVDGGIGD
DSIAGGAGADTIDGGDGDDWISGGDGNDAIIAGKGADVVFGNKGDDNISGNEGADTIYGN

EGTDIIDGGADNDTLHGGDGNDIIDGGDGDDLIVGDKYGYQDPQLLPNGNYGVPEEGGLH
ISIDSIYSTTTGSNNSLGYYFADENGEPISGHVAFADSKDSLWAGDPFTIDYNSEDIPAG
ATQIGLFIIPDGETENPTLTDGSAITFQNIAGQWTPMLGGAALKGDQGVPAFFSDSNINV
AGFDYTNSNYDGTRISFEDFLGGGDNDFNDVVADISIKTLYNGPDNNDTLTGGAGNDTIL
GGAGDDNISGGDGDDDIHGGIGDNIIAGNDGDDWIESGSGADNITGGLGADTIQSNAGDD
IIDSGDGNDKVFGGAGADDIKGGAGDDYISAGSGDDTVDAGDGNDIVLGNTGTNTLSGGK
GHDYLVSGDGDDSTLFGNEGDDHLYGGSSNNTLIGGEGDDVIVSSGKSGTFNALFGDEGD
DILKASDGSNFFDGGTGNDIAIFEGAYSEYIITIDFVGDIVVTDTVADRNGDNTLTSVET
LQFSDGTVLASDIIAGNPIVPNGLIVADVTDDWTDAISKDPNFVATDPGTWVEDLNLKSH
DHENDYDDLDENELDNNDIPDDGDLDPNDPPPI

>gene_1406|GeneMark.hmm|292_aa|-|24496|25374 >NVVL01000060.1 Rickettsiales bacterium isolate NORP64 Contig_source1382A_23925, whole genome shotgun sequence
MTTIVSNTVSFIKTLLTHLRMSLSSASFYKDVITSYKGYGVKYILTLSLIGSLFSTMFFL
GDVNTLQTYLEDSKIEQKIENIDHILTQMPTLDYDGKNISTKKNTPIFLLDKKNKKIVAI
DPNGKLKPSESAKIPVLMTGNKIVITLIHSDQAIRNSWPLQYTQIFGKEPQILTQEVIKS
NFASLLEKSSSVFIYMVFPIAAILLFINTILEKSFFILVIYFITRAIAPQFSMQACIRTV
LFASGSAVLIQPVLLLAIPTLSWLLGILQIWANLLMVLGILKASGRSRFSLK

>gene_1407|GeneMark.hmm|175_aa|+|108|635 >NVVL01000061.1 Rickettsiales bacterium isolate NORP64 Contig_source1382A_23964, whole genome shotgun sequence
VEKGYVSSEFIVKSGRKKNATITGYASVFGVVDFHNDIIVKGAFKKAKANKIRLLWQHES
KKPIGVITLLEEDEYGLKMEAEINSNTVAGAEAAELVRQKAICGLSIGFVIESSNYNKEG
IRVIDGINLMEVSIVTFPANRSAEIQAIKNRDKEKGSYEESLDTLQGLIKKLENY

>gene_1408|GeneMark.hmm|353_aa|+|685|1746 >NVVL01000061.1 Rickettsiales bacterium isolate NORP64 Contig_source1382A_23964, whole genome shotgun sequence
MEENKIEEMSEDLKSFLARESNTGVKINKIEGDISKMQNYFLNDNINNNPEEKAAFNSFI
REGVGSDFINKSFSGGAQEGGVLITPTLNNQIISAINAKSPMRQLASIESISTRALDIVI
EDGAFISGWVAESAARPDTNTPNLTKKTINAHELYAQPKATQSIIDDSEINIENWLSERL
VDSFVKLENEAFINGDGNNKPFGLLANAGIGRIDVEHEVSPASLLNLINGVGEGYLENAS
FLMNRSTLSSIQSLTDEAGRFIWQQSLSDPLKQTIFGVPVVISSNMPDIADNSLSIAIGD
FRSAYKIVDRSGMNLMRDPYTDKPFVKFYAVKRVGGDVVNPSAVKFARFAAAA

>gene_1409|GeneMark.hmm|175_aa|+|1832|2359 >NVVL01000061.1 Rickettsiales bacterium isolate NORP64 Contig_source1382A_23964, whole genome shotgun sequence
MIMIVNHKILNIRNIETWPLEEVKNYLRITSDYDDVLLIGLVETATNIAESFTGLSLHNR
HILCEIKNAKPIIKLKHVPVLRLTGVRLASKDEKEDISDDFGFVETSNSTLHLASHYAGK
DVEIEYESGYEEHIPREIQHGILMHVASMYEHSEDGTSINSEIKDLYLPYRVMKI

>gene_1410|GeneMark.hmm|109_aa|+|2376|2705 >NVVL01000061.1 Rickettsiales bacterium isolate NORP64 Contig_source1382A_23964, whole genome shotgun sequence
MKQNIISKLSHRIIFLENRAASELEEDLWEEVQTSFAEMKPICDNRFVSLEGLQFGNVIT
EGYFLFRIRSIKDVHSRMRINFQGRLFEIKRIIDEDEKGRMLSIVALEI

>gene_1411|GeneMark.hmm|140_aa|+|2723|3145 >NVVL01000061.1 Rickettsiales bacterium isolate NORP64 Contig_source1382A_23964, whole genome shotgun sequence
MSLTFIHDFQKQLYSALSADAWITNNINKIYFGVVQDGQSPFILINIQKAEDLSLHHNSI
YSVDFQIIGYAKDHNHQLLVSLGDRVVELLSNSERCFGGYIIAGIKANDVQFERARDLVL
NKLTINYKALIKKEVYDELS

>gene_1412|GeneMark.hmm|198_aa|+|3132|3728 >NVVL01000061.1 Rickettsiales bacterium isolate NORP64 Contig_source1382A_23964, whole genome shotgun sequence
MSFHDVNLPRHIEVFAIGSSEFSTSCAVSMSGREVRSLDAASPKRRYTLKDCRLSALQFE
AFNSFFKARGGRRFSFRLRDHFDYIAARQIIAIGDGSAIEFQLQKTYEDKVNPYVRTITK
PIAASVNLWSDMEDIIPNSIDANTGIVRLAQPLAGGASLSASFEFDVPVRFACDEFQYSL
SKDGSIILNDVELIEVYE

>gene_1413|GeneMark.hmm|260_aa|+|3728|4510 >NVVL01000061.1 Rickettsiales bacterium isolate NORP64 Contig_source1382A_23964, whole genome shotgun sequence
MGENFVYLFYIKTSRNKEYYLTSSSEVVVSAGVKYQPYSGLALTSGKFNDSGQNQVILSG
IFEKQGIDKDDDLTGALVKIMYMQGDKGDKGNKDDKAIHFVSYICTQHSKMDLDFEIKCE
PETIKYNQPVVQMFSKTCRANFGDDKCKVDMGDYSVLCDVVKVKKNILTCELGDVKSGYF
KCGKLVTLGGEVESCSFKIMSHSGNDIEVERGCNFDFAEQKQVTLIPGCDKSFRTCCYSF
KNAVNFRGEPAIPEFNFIKN

>gene_1414|GeneMark.hmm|43_aa|+|4561|4692 >NVVL01000061.1 Rickettsiales bacterium isolate NORP64 Contig_source1382A_23964, whole genome shotgun sequence
VFRFGAVSVPQTDQLQDSAEYFCRVEKDTLSAYEKLVKEYERK

>gene_1415|GeneMark.hmm|168_aa|+|4679|5185 >NVVL01000061.1 Rickettsiales bacterium isolate NORP64 Contig_source1382A_23964, whole genome shotgun sequence
MKESKKFFENNRLNKGYGRAPSNRVSDSALVNKKYNHALPPIDMMEEYEEMNPGSCAQLF
SMAKQEQNHRHSMDLVAMNRHNKAVHMGRMFSLLFVMIVAASTFALAFFGNVIVASVFAG
SAFACIVMVSYFYSKNTVNTVKREFNRPQQRNHRPSKGRGQRNNRPSR

>gene_1416|GeneMark.hmm|259_aa|-|5189|5968 >NVVL01000061.1 Rickettsiales bacterium isolate NORP64 Contig_source1382A_23964, whole genome shotgun sequence
MKNKVVVVTGSTSGIGLGIAKRFSKSGAHLVINGFADSGEIKKISEELMADGAAGVIHEG
ADLTKPAEIDQMFANILDKFGKIDVLINNAGIQFVSPIEEFPPEKWEAIIRIDLIASFYT
IKNVVPIMKKNGWGRIINIASAHGLVASPFKSAYVAAKHGMLGLTKTVALEVAEHGITVN
SICPGYVKTPLVLEQVADTAKARGISEESVIKDVLLAAQATKKFVEIEEIASLAHYLCSK
EAASITGTALSMDGGWTSQ

>gene_1417|GeneMark.hmm|105_aa|+|6408|6725 >NVVL01000061.1 Rickettsiales bacterium isolate NORP64 Contig_source1382A_23964, whole genome shotgun sequence
MIKTKEIHFITTENGKCPVKEWLKKLDIQTRARINVRLVRLEYGVYGDCKRIDKQLYELR
FFFGKGYRVYFTERDNKIILLLSAGDKSTQIKDINKAKDILKELS

>gene_1418|GeneMark.hmm|98_aa|+|6737|7033 >NVVL01000061.1 Rickettsiales bacterium isolate NORP64 Contig_source1382A_23964, whole genome shotgun sequence
MEKFNDYLTLQLQDQDELVAYINAALEQYSIDQNKELFLAAIKEAIIARGGVTKVAIQAH

INRQHIYRMLSSKGNPSFDNIGILLSVLGTRLMVEARA

>gene_1419|GeneMark.hmm|64_aa|-|7092|7286 >NVVL01000061.1 Rickettsiales bacterium isolate NORP64 Contig_source1382A_23964, whole genome shotgun sequence
VVYEESIANHAAGAFVAILEGLDVGNKHHGKQRFFKSVISVFIIAILSYILYYPIINDRM
NNYN

>gene_1420|GeneMark.hmm|510_aa|-|7666|9198 >NVVL01000061.1 Rickettsiales bacterium isolate NORP64 Contig_source1382A_23964, whole genome shotgun sequence
MSSSRSTRVNAKHLLSGDFSRNEGDWVRTQTWLRVFKILLQEDNLNTISEIDSTELKSLV
STHGVLGDSDFKKGRQGRQEKTSAQDSFKQVITTLLTRSINEETINSAKVSEFLKLFEGT
PLEEYVISSANTLFKAKCTEHARPYSRAFEALDLSESTGLFKMLSAFNPFFSFVDTSIIP
RNLFKMWVTPKTPDYDTIMSILSNTQETTIQNLKGDQVETKSIATLVATLNDTTDTDELD
FGSASETSESTESVVSSDLDTSFTVSERSTVDLEVKSHTPGTLSDESRIFSACTTGDIKT
VSTMLNGGYDVNSHETYDRPPLLLAVSVSDPNLALVSLLIDKGADVTARSTDDKGVLEHI
QEDSLDMHPGATKDIIAKIASKFLDMGRIGQFVDELPSYLIDAQLNLWINALPEKVTQAI
KSIKYSLHQDKLTSLQEALPLATSEEVPIELPLAEDLSQVDLLSLSLHDLFRKFLQDNPS
VLGLTAGELAGTFSEEHSTMGDSLDPHPAD

>gene_1421|GeneMark.hmm|75_aa|-|9195|9422 >NVVL01000061.1 Rickettsiales bacterium isolate NORP64 Contig_source1382A_23964, whole genome shotgun sequence
VIRKDVTYLQCCLIKIRKLFAFVLGNVNIFVIESARRREALSAKTIRFMPLVSAKNERSL
MCDKCLLIIKYRMML

>gene_1422|GeneMark.hmm|360_aa|+|9550|10632 >NVVL01000061.1 Rickettsiales bacterium isolate NORP64 Contig_source1382A_23964, whole genome shotgun sequence
MSIDRKTIVVAMSGGVDSSVVAAMLHKAGHKVIGITLQLYDYGEALQKKGACCAGQDIYD
AKMVADKLGFPHYIFDYESKFKEEVIDDFVDSYLRGETPLPCVRCNQSVKFKDLLRAAKE
LNADALATGHYVQKIVNNGMSELHKGVLEEKDQSYFLFSTTQEQLDYLMFPLGGYSKDKT
RELAREFGLEIADKPDSQDICFVPNGNYRDIIDKARPDAMKPGRFMHIDGFDLGGHKGIA
GYTVGQRKGLGIAFGKPIYVIKIDPQTNTVYVGAESALFKTKFLLKDINWIARDKNPEGL
EIIAKIRSTTPGVLARLNYKDGQLQVTLLEREKAVTPGQACVFYDGSRVLGGGWITREIE

>gene_1423|GeneMark.hmm|471_aa|+|10732|12147 >NVVL01000061.1 Rickettsiales bacterium isolate NORP64 Contig_source1382A_23964, whole genome shotgun sequence
MKFFKKKSFESVKDAGGTSGLDRTLDSLDLILLGLGGIVGTGVFALTGMVAAQYAGPAVT
ISYIIAGVTCIFVALVYTELATMLPTSGSVYTYSYVAFGEMVAWLVGSALIIELCFSASA
VAGSWSAYVQGIFESAGAPLPKMLTNSPFEGGIVNLPAFLIVVFVGCMLYRGTKESKKLN
NILVFIKMAAIFIFIFFAAPHFDAANWENFMPNGFDDVLYGSSILFFAFTGFGTLASAAE
ECKDPKRNLTIGIIGSLVLATIVYVVVAAVLTGIVPFGDLDNAQPLAYALKQTGNGIGST
LVATGAVAGMTTVIMMNIYGQSRIFYVIARDGLLPKSLAKLHHKYDSPYMTILVFTTLIA
VMGALIPYGILAQLSSMGALVDYMVVAVVVMMFRIQKPDVERPFKCPIVFVIAPLALMAS
FYLLFKQIISKHGEMLLTGKIFIYWFIIMFVLYVMKSSLFSAKEQIDNAAD

>gene_1424|GeneMark.hmm|450_aa|+|12243|13595 >NVVL01000061.1 Rickettsiales bacterium isolate NORP64 Contig_source1382A_23964, whole genome shotgun sequence
LYFKIKPIQVKLMSVMDSTFNAVIFLEDFFWAYIGWALIVSAGIYLTIISRGLQFRVLFN

FRSNIKNIYCEASKSKNGGVHPFKLYFASVGGMVGLGNIVIISMAVMIGGPGSIFWTVLA
SFSGMLLKYSEIYLGVKYRVQNPNGGFNGGPMYFLQKAFKPKIFAYIFAFLMCLYGVEVS
NFLIIVDRMEHSFEFNRYAVIFALLAVTIYSAIGGIERLANICTAIMPVFMIGYVILAIY
IIFINASLLPEFFKTVIVSAFTPHAQIGGFAGSSVILAAQLGISKAVYSGDIGIGYDSII
QSETRIVNPHDQATLAIYALFTDTFICLLTNVMIGVTGAWYTLNHLVPSDIVSKILSEYI
PYSDVFMTLLLFFAGFTTIIAYLAAGIKCSEFISQKYGKMIYLSYAIFAFIFVCNFSQTN
AIIIMSLLAGLLVLLNICGILRLRKDIEFK

>gene_1425|GeneMark.hmm|429_aa|+|13636|14925 >NVVL01000061.1 Rickettsiales bacterium isolate NORP64 Contig_source1382A_23964, whole genome shotgun sequence
MKDLLPDDFRIHKHIEEIAFNTAKLYGYEGFATPMLEYVSVFDRTLGEESDVVSKEMYSF
HDKKGRHIALRPEFTASVMRAFISNGLKQKLPLKLFSSGALFRYDRPQKGRQRQFHQLNF
EYLGASGAYSDAETIALAAHVLENLGLLADVTLELNSLGCRESKQNYQAALSAYFAQYVG
ELSEDSKKRLEKNPILILDSKDESDKKIAKDAPVITDYYTKDAASYFQEVRSNLDMLGVS
YVVNPRLARGLDYYSHTAFEFVTTSLGAQSTVLAGGRYDGLSELMGGDKMPAIGFAAGIE
RIALMGEFSPAKERRVYVIPIGAECADYASRVTNILRRGGVQTSLSFEGKIAKRMQSALA
ADASFVVFIGSEEKAKGTYKLKDLDAGSEIEVNSAELLEITQQQVVQQQIARQQIARQQV
APQKTRQGQ

>gene_1426|GeneMark.hmm|333_aa|+|14988|15989 >NVVL01000061.1 Rickettsiales bacterium isolate NORP64 Contig_source1382A_23964, whole genome shotgun sequence
VDYSDALALCGDEVINISSIGAKINSRHARKYSLPNLAPWCVFSKLYLKILTILLKPDII
ICHGNRAISFASEFRFGIFDKNIPIVGVAHNYSYKHLKKCDYVIALTENLQEHLVARGIA
SKQILRIPNMARVGREYKQKEFSSPAAGSPEAGLPVVIGSFGRFVAKKGFVYLIEAINIL
KQNGHDVRLVLGGSGQDREMLKEKVQELKLEGEVSIEGWVTDKDAFFQQMDIFCLPSVVE
PFGIILLEAMEHSKPIIATKSGGPEEVIRDNVDGLLARVESSEDLARKIEQLIMDQDLAH
RLARSGYLRLKENYDINIVAKNLSNSLNRLIKK

>gene_1427|GeneMark.hmm|425_aa|+|15986|17263 >NVVL01000061.1 Rickettsiales bacterium isolate NORP64 Contig_source1382A_23964, whole genome shotgun sequence
MSFNSSKLPNGLDVVTYSMEQVNSVAINIIVNVGSRYETEEEAGISHFLEHMAFKGTTNR
TALEIAKEFDAIGGQFNAYTSREHTVYCTKVLSQHTDQALDMLSDILQNSLFEEGDIKKE
LGVILQEIAGVNDSPDDLVYERFYELAYPNQPIGKSILGTGENIAKFDKNAFQKYMAQHY
RPDNIVISMAGKVTHEEAQAQVSKFFSNMGSSKQEPHMQTSHPLAQYVGGYHFTPKELEQ
TTIALGFSSVSYADLKLFYHAQILSIILGGGLSSRLFQRVREELGLAYSIGSWVNAFADS
GTFSISAAADHNNIAVLLENIVIEVNKIKTDISEEELKLAKAQIETNIYMAEERSEYKSE
EVGKNFALFRKYSPIEEVMDIVLSTTTDDLHAMAAEIFASNPTLSIVGAPQKDVSFNNIQ
DKLLK

>gene_1428|GeneMark.hmm|212_aa|+|17330|17968 >NVVL01000061.1 Rickettsiales bacterium isolate NORP64 Contig_source1382A_23964, whole genome shotgun sequence
MFSDKKHKMLNCILASESLEKIGQKRKNGKTKIAELMEDFYENGILLAMIFFSLPVAIPL
PYPPGFTTIMGVPLVILSIQLLIGSKKVRLPEKINNYEMKNATLKTISDKIVPIIKYVEK
YVKPRFEFAKSVYCEQFVGFVCLIAAIAVALPIPFTNAIPALGVTIMSLGLLNRDGLVII
AGFVITVVGVVIAISAIAVSYVAVQYLFHMIF

>gene_1429|GeneMark.hmm|236_aa|+|17991|18701 >NVVL01000061.1 Rickettsiales bacterium isolate NORP64 Contig_source1382A_23964, whole genome shotgun sequence
MQKDLIYKGSTKSLYHHEKEHMLKMSFEDTLKTSAKDLIEIPGKGIINNSISSYIMQQLD
LIGVATHFVKKENMRQQTIQHTDIYPIQIHIATVASGRYVTDFGMEPGLIFETPLIDFRV
KNKKIGYPPVNESQIMHFGWLYKEEIEDLKTRAMRVHDFLYGLFAGVQIRMIDVKLEFGR
VYDEENFMIMLADEISPDTCRLWDMNSNEKLSYEIAEEDPEKILPAYQEVLRRLEG

>gene_1430|GeneMark.hmm|704_aa|-|18753|20867 >NVVL01000061.1 Rickettsiales bacterium isolate NORP64 Contig_source1382A_23964, whole genome shotgun sequence
MSSKLTIFTLKDIRDMPESRVVIILNDQVYDVTDYLVCHPGGREVLLENNRKDATEAFSA
IGHSDRAEEILKEYKIGSLAEKERSGGPPLQSNALSSSPEPLKRVMHIKNKLVTEEDPYF
FHKIFGLIVLLHMAIRFIVLGLDAGEVIIQRDIIGFNSDNVFSDGTKLFFAFCHGILSVS
SFIFAVPKHSSQNKPMIHQMFRGHSVCFASRAVLCMVVDVLVQSPELKRFLISCIVLSCL
IAADQITKYLATEDDRYKTTNSMPYWRGCSVEREHLHKTFYAFAQFLASIICLFGSYTTI
FFTLPAIQGAALLMTLTRKNIITSHSYHQIYTFLLFYPIPLFLVLWPAKTIFAIILSGVL
YYLRSRNINKYLLWIPVLIMANMIELTPENYPNGLIIGAAIFLLTLYIVKSNAKIIETER
IDGNNRVVSHKQITPDAYELIIRTQTPIKLEIGQHVLIQINDSLNRKYTPIWTKYLPETD
QTLLCLRIKEYKSTERLTASSYLARCGEGSVLALHGPYGNKFYCPKNDAIIDKINKCQYN
LEDYQVYLFSAGSGITPIYQLAKNICKPGQKRGDSKNGGEKGDGKEKGREKGKKNEKLTL
ITCDQKHENQMMKDELTQLKEDFPNELDWLCFLSREGKNSPDIPATIFKERLTPGKLMNI
VKCDSPSLIIICGPDKWQEMIQQTINLIDFDLDKPPPKCKVLAW

>gene_1431|GeneMark.hmm|994_aa|-|21191|24175 >NVVL01000061.1 Rickettsiales bacterium isolate NORP64 Contig_source1382A_23964, whole genome shotgun sequence
MARERKAGISYLEQQKARYNIRINVVKSLYASKLKTNPETPDHDLELKLSFISILNLTNI
ASSRKLKAFYSLNLAFPDVIKSLFGLDAKFFSNLNKLTPEEKNSIRDFLYFATDAYISLD
KKESLFHIAAKFGAASVLESLMRTCSDEKGSINSNNQNFLYNLKLYGHNELHQTMLENHP
ELNELDTDHPLLLKSPPEMSPPSALDSMNKGVAYYYISIFGLTPEYNCNTRNSYSNKALK
HLIDYCNAPINTHNPLAITCLLDIYHASKNENLLTVLQSTCSNASDLSQYFIASCIKYAI
QTKDTDYLDKAYKFCKSSNSLEAHVIIAVYAVKHGDWTFYKAIRDDVSVEQPFRALQDVD
NNIARVFAAFNQNQEIIDAESDLEFKAKLKAASVTVQGLCARLVNVDDATALCSQEIVLP
SAIKKSPQLLIEACKLKALALQSTDIDGAIEIFNTVIHVMTRMPGISLRAKVEIFILKGQ
ALQEKSVRILKLWGQVPQSKDIDIDSEVRNTIDNAIMVYDKALNHIWEASGDAQALYNSV
LEYGVENAYPNELKIKLSELNIFGKFREILYHKFYCYIIQRDSDNALICASLLDYSPDEE
PKLYTLLARKAAGGDPSVFAEFLAIDPDTLSPKTKAVYKKIAPILIRCNEKDIADTETSI
GAMEAELTSVQEELKQTHPEQPTSTQLVQFTHPGKEGRFIKSEVKVRFMNLEDLPYEFFT
THIKALAGCKAGEVWLWLSRLKSHHRIFQSECQKIQDGRSNVLQKKGYVDKLLAEYDSIL
ENHENRELSCALITPKNHNILKEFHKEVQHFTLNLLQIVFGIQAIFLGRNEYARKLLESF
EANPSQKMLKICIEIGEEEDELLTPLEDLLSTSQGLASIPETEILKTRTHTTKCPYTSEN
FLGIYTDWLDALPSQLAVQTSALPNEEAERTLLQSAAPSSKSPSTVEVFEYFNFVLDVLL
MPDSDHDVDLYGKPISTHNTGEFVFAEVDTLSFF

>gene_1432|GeneMark.hmm|185_aa|-|174|731 >NVVL01000062.1 Rickettsiales bacterium isolate NORP64 Contig_source1382A_23967, whole genome shotgun sequence
MEESQKQELTAKMEKTMAVLDRELKGLRTGRASVNLLDPVVVEVYGSKMPLSQMGTVSTP
DAKTITVQVWDKGMVKTAEKAIADANLGLNPSSDGQLIRMSLPPLDEERRKDLVKIAYKC

AENTKIALRSVRRDGMDGLKKMEKANEISKDEHHSHGEEVQKLTDGFIGKVDSLVKSKEQ
EILTI

>gene_1433|GeneMark.hmm|239_aa|-|737|1456 >NVVL01000062.1 Rickettsiales bacterium isolate NORP64 Contig_source1382A_23967, whole genome shotgun sequence
MSLTEYKRVLFKVSGEALMGARSFGHDQETLKGISEDIKEVCELGVQVCAVVGGGNIYRG
ADAEILGMERASADYMGMLATVINALALQNALEQSGVYTRIQSAIPMTSICEPFIRRKAR
RHMQKGRVVIFAAGIGSPFFTTDSAAVLRAVEMECDCLLKGTNVDGVYDDDPKQNPNAKK
YDEVTYTDVLRDNLKVMDMAAIAVARENNLPIKVFSIKKRGNFAKVLQDQGIYTLIKGN

>gene_1434|GeneMark.hmm|724_aa|+|1691|3865 >NVVL01000062.1 Rickettsiales bacterium isolate NORP64 Contig_source1382A_23967, whole genome shotgun sequence
MPKLTIDDKEIEIEAGATVMQACQQAGIEVPHFCFHDRLKIAGNCRMCLVEMEKSPKPIA
SCAMPVSEGMNIKTNTPLVKKAREGVMEFLLINHPLDCPICDQGGECDLQDQAFKYGKGS
NRYTENKRSVKDKNLGPLVKTQMTRCIHCTRCIRFFNDVAGLQEMGAIGRGEHMEVVSYL
EKSLTSELSGNVIDLCPVGALTSKPYAFKARSWELTKTNSVDVMDAVGSNIRIDSRGSEV
MRILPRVNEDINEEWLSDKARFAYDGLKAQRLDVPYVKKNGKLKKASWDSAFAAITQKIL
NIKGKEIAAIAGSTASSEPMFLLKKLLAKLGSDAVDANQFGYSLDVSARGNYLFNTGIAS
IEKSDLCLLVGANPRQVAPILNARIGKAVRAGTMQIARVGEVDDQTYNIDELGCDAAILT
EIASGKGSFASLLKKAKNPAIIGDGALVREDGEAILVLVHKIVEKYKIVRSDWNGFNIL
HNHASMVGALDIGFHYGSNGTADILDKSQKNDVKMLYLLGADDFDIDKIGKDCFVVYQGH
HGDAAASRADVILPEAAYTEQDGIFVNMEGRPQYAKAAACRLGEAKESWDIILTLARKLD
VALPAKDLSSVRELMAKEHSVFANMDAVQTVDFTCFKSDQKLLKTPIGRVAINYYMTDPI
SRASVTMARCTKDLKEGLEAKKVPELKKDLDSKGSLDSKKDLSSKKDLGTKQSPGSKKDL
EAAL

>gene_1435|GeneMark.hmm|338_aa|+|3862|4878 >NVVL01000062.1 Rickettsiales bacterium isolate NORP64 Contig_source1382A_23967, whole genome shotgun sequence
MMDLFMDYAFLILIIIAKILLITVPLLLSVAYLTYAERRVIGLMQLRKGPNVVGPMGLLQ
PIADAVKLMFKETIVPTAANKVVFIMAPMVTFVLSLIGWAVIPFDAGMVLADINVGVLYI
LAVSALGVYGIIMAGWASNSKYAFLGAVRSSAQMISYEVSMGLVIVTVLLVTGTLNLSEI
VEYQRTMPFWIQLLLAPMAVVFFISVLAETNRLPFDLPEAEAELVAGYNVEYSSMAFALF
FLGEYANMILVSGITVTFFMGGYLPPFNIGFLNFVPGIIWFILKVEVLLFIFLWLRATLP
RYRYDQLMRIGWKVFLPFTLFWVVFVASILMLTDSLPG

>gene_1436|GeneMark.hmm|159_aa|+|4891|5370 >NVVL01000062.1 Rickettsiales bacterium isolate NORP64 Contig_source1382A_23967, whole genome shotgun sequence
MIKYIKSFVLYEILVGMALTLKYLFKPKVTINYPYEKSPVSPRFKGEHALRRYDNGEERC
IACKLCEAICPAQAIVIEAEVRKDGSRRTTRYDIDMTKCIYCGLCQEACPVDAIVEGANF
EFSTETHQELLYDKNRLLKNGEIWEKELSTKLKDDYLYR

>gene_1437|GeneMark.hmm|206_aa|+|5379|5999 >NVVL01000062.1 Rickettsiales bacterium isolate NORP64 Contig_source1382A_23967, whole genome shotgun sequence
MAVFFYGFAALLILSSVMVISSRNPVHAVLWLIFAFVNASGLMVLIGAEFLAMMLIIIYV
GAVAVLFLFVVMMLDIKFSEFKSATGSEMGISFLIAFFLFVDLSVVVLLGTKIITPHASF
SFNISPDIGSAYAIGKVLYTDFILPFQTSGIILFVAMIASIALTLRMREGVKRQNVSHQL
AQSKESNLTFAKLDGKSGLDNLNYDN

>gene_1438|GeneMark.hmm|105_aa|+|5989|6306 >NVVL01000062.1 Rickettsiales bacterium isolate NORP64 Contig_source1382A_23967, whole genome shotgun sequence
MTINEISLEHYLILSSLLFSIGVTGLFMNRKNVITILMSVELMLLAVNLNFIAFSAYLQD
VVGQVFSVIILSIAAAEISIGLAILVLYSRNRGSIEIDDINQMKG

>gene_1439|GeneMark.hmm|643_aa|+|6310|8241 >NVVL01000062.1 Rickettsiales bacterium isolate NORP64 Contig_source1382A_23967, whole genome shotgun sequence
MEFILKSLIFLPLFSGIINGLFCKRFTNKLASIISSGAITISALCALFIFIEAGIGQNTM
HVTLARWISIDSLSINWALYVDQLTAIMFLLVTAVSAVVHIYSLGYMADDKNLPKFLSYL
SLFTFFMLSLVSADNFVQLFFGWEGVGLCSYLLIGYYYHKDSANNAAIKAFIVNRVGDFA
FIIGIVMIIIYTGSIDFENVFGLSENLSKTEVTIWDTNFIVLDIICLMLFLGCMGKSAQI
GMHVWLPDAMEGPTPVSALIHAATMVTAGVFLIARCSFMFEYSPFVLQIITIVGAVTCLF
AALVAIAQTDIKKIIAYSTCSQLGYMFFACGVSAYQAGIFHLVTHGFFKALLFLSAGSVI
HSCHQQDLFKMGGLRKKMPITYANFWMGSLAIIGIFPFAGFYSKDLVLESAYAAGGVGAF
AFALGIIAAILTAIYSLKIIVLVFHGKTNLSKEAFDHAHESPRIMNLPLLILVAGTLFAG
MIGYYILSIDSPYGFFSGSIFNLHISESGHHVALGIQLLPLIVGLAGMLFGWYVYGNNLS
DAISGRMKFAYHILKNKFFFDEIFEALFIKPASRLSSLAKIFDSHCIDRLFCGGSAGAAG
IFNWCVCQVQTGYIFNYALYIVFAVVVAITAFVGNYLYGVWSI

>gene_1440|GeneMark.hmm|496_aa|+|8243|9733 >NVVL01000062.1 Rickettsiales bacterium isolate NORP64 Contig_source1382A_23967, whole genome shotgun sequence
MLNLPILSISILLPLLSALYIVFFISNSRSEKKQLYAMYVAILASSLTLIATTYLLFSFR
FKIAGFQFVERYSWVDSIGLEFHVGVDAMSVYFIFLSALLTLICIIASLFTIKKHIKEFL
LCFLLLESFCIGAFSAINLLLFYGFFEVILIPMYLIIGIWGGKDRVAAAFKFFLYTFFGS
VFLLIALIYIYSQTGTFSMVELANLAPEFSLEVQQWLWLATFISFAVKVPMVPFHTWLPD
AHVQAPTAGSVMLAGILLKLGGYAFLRVSLPMFPGASEYFAVYVLGISGFAVIYASFVAL
AQTDMKKMIAYSSIAHMGYVTGAIFSFTVKGISGAVFQMISHGLISSALFLIVGMLYERH
HTKEIAKYGGVAASMPVLATFFMIAMLGSVGLPGFSGFVGEFLSILSIFEVNPIVGIMCA
FGVIIGAVYMLKLYKCVMFGEVVDKNIINFTDLKLYEKAALVPLICLIIYIGLAPNGVLK
ALAVPSANLAQLYGAL

>gene_1441|GeneMark.hmm|466_aa|+|9771|11171 >NVVL01000062.1 Rickettsiales bacterium isolate NORP64 Contig_source1382A_23967, whole genome shotgun sequence
MLNQFAIILPEMMLALLAMTMQMAGVFLKNHTKHICATTYILGLAIVGYLLYFVPEYELG
FSDSFGVSPAIYFFKAVVLSLALMSLIIYQDLAKISQTHLRFEFMTLVLLSTLGVFISIS
AQDFMLLFCGLELQALSGYALAAFDTKRAKSSEAGLKYFVLGALMSALMLLGISFLYGFS
GSMNFIDIRVALGSEFNIGLVVGAVLMLSSILFKISAAPLHIWTPDVYEGAPISSVTYFA
VAQKIGMLVVLINVLDGVIGNYTAISASLIKIVAIFSMVIGGLGAITQHSLKRLMAYSTI
LNIGYVLVAVCLQSHEGSHAAFVYMLIYVVGVMGFFACIVALLGAKADEASFDDLKGIGS
QRKTIAAAISIIMFSMIGLPPLAGFFGKYYIFYNAVMQGEITLALIGVLSSVVAAYYYLK
IIKFMYFMEPDGEFIHIPTHAGLRFVGVITVSFILLFFSFAQSYLF

>gene_1442|GeneMark.hmm|250_aa|+|11193|11945 >NVVL01000062.1 Rickettsiales bacterium isolate NORP64 Contig_source1382A_23967, whole genome shotgun sequence
MTLNWLESYNLLTLDKIDSTSAEAQRLARSSAEEDYVIISKEQSAGKATKGREWDSKPGN
LYSSILISSKLGLERIPELSFLVANAVYDAITSLAISQEVELDIKLKWPNDVLINGKKTA

GILLESIFVKDRHYVMIGIGVNLLHAPNHLNCPVTSLFDEGVHPGGAREFLNSLMTNFHR
LYSGWLSDNNFAKTRSDWMGRAYNLNKVITIDDGLRRTSGVFKEISFDGSMVMQLASGQF
CSLASGEIEL

>gene_1443|GeneMark.hmm|248_aa|+|11935|12681 >NVVL01000062.1 Rickettsiales bacterium isolate NORP64 Contig_source1382A_23967, whole genome shotgun sequence
LNYNSKRRKNYNMKNISIILAEPQMGENIGAAARSMKNFGCTDLRIVNPRDGWPNDKAIS
MSVGAIDLIQNARLYDSVRDAIADLEYVYAATAAPRGMNKDYVLSRDLRNDMDPSCKIGI
MFGRENYGLNNQEISLANKIVTIDTDINFSSLNIAHAIAVICYEIFQTKEQPRTDLENKQ
QLATNVDLEYFYNHLFGELGNRSFFRTPEKKEHMSRKIRNIFSRIDGLSQTELQTLRGIV
TTLSRDEK

>gene_1444|GeneMark.hmm|104_aa|+|12730|13044 >NVVL01000062.1 Rickettsiales bacterium isolate NORP64 Contig_source1382A_23967, whole genome shotgun sequence
MQKIRLSEFYREAIIKAFKGHFGADDHLWIFGSRVDTQQKGGDIDLYIKTQEVDARIANK
MRAQYHQELWKILGEQRIDIVLNIYPFELKLPIYEIAENGVLLV

>gene_1445|GeneMark.hmm|152_aa|+|13064|13522 >NVVL01000062.1 Rickettsiales bacterium isolate NORP64 Contig_source1382A_23967, whole genome shotgun sequence
MISLLKEHLKIARIHALRIKEAREAINHLFPFTPAIIEELTCSDIAFLELYTNRFSKLQD
LIGAKMFPEILLLYGEDLQALFFIDRLNKMEKIGILNDAQEWLYMRKISKQLSHEYPENP
EFLADNLNIICKLEPELLACLANIEKLAKTMI

>gene_1446|GeneMark.hmm|169_aa|+|13519|14028 >NVVL01000062.1 Rickettsiales bacterium isolate NORP64 Contig_source1382A_23967, whole genome shotgun sequence
MNYFKLLELEQKYDIAPSLLEQQYLNKQLTYHPDKAESEPMRGKYLEISMQLNDAYKILK
DDYMRAEYMLKLSGQKFDDDTLKNKLSNSEIEEIIDEYELIDEIDQLPKLKEIQLAKNSD
KLKLTKELTDCFSNNETAKALDLTVRLKYLTNLVRNIKLKIKHADSRDQ

>gene_1447|GeneMark.hmm|605_aa|+|14006|15823 >NVVL01000062.1 Rickettsiales bacterium isolate NORP64 Contig_source1382A_23967, whole genome shotgun sequence
MQIVEISEPNSEDGANEAPEIVVGIDFGTTNSLIAISNDSSAKIIKMHDGAELLPSVIEI
IDDEIIIGNKATQNPDAIRSIKRLLAKTSKEIKSAYAGSALSGRLALEESEPKILAKGRK
FTITEMASEILKYLKSQAEASLETDVNKAVISVPAHFNNTQRGQVLLAAKLAGFSVMRLI
SEPTAAAYAYGLGKSARGAYLVYDFGGGTFDVTVLNMHQSVLQVVATGGDNMLGGDDIDM
ILAKYIAKETNSDLNNEIIALAKKTKEDFADKNEVALNFNSTNIVISKAKYEELITPIIN
KTIKIAKDTLHEAEDIDLNGIILVGGSTRVSLIKEKLNKAFALPIYSDLDPDKIVALGAA
LQAENLSSRSGSLLIDVVPLSIGLELYGGIAEKIILRNTPIPFSVTKEFTTHADNQTGIN
FHVVQGEREMVKDCKSLAKFELDSLSPMKAGKARIEVTFAMDADGILSITALDAESGRAQ
NIEINPGNDLNEKEINEALSIAFQNASADHLARLLAEARADAQSLISGIEKAMLDTPDIL
NEPEKNQIDIAISSLQKEINSDNRDEILLKIDELNKLAAGFIQKHLDKGADLLLKGRHID
NINSK

>gene_1448|GeneMark.hmm|112_aa|+|15831|16169 >NVVL01000062.1 Rickettsiales bacterium isolate NORP64 Contig_source1382A_23967, whole genome shotgun sequence
MAAETIKVIFIEDGTEKEIEALVGLSILEIAHENDIDLEGACEGSLACATCHVVLEEKTY
NSLEEPEEAEEDMLDLAFGLTHTSRLGCQIILTKELDGMRILLPSATRNINL

>gene_1449|GeneMark.hmm|355_aa|+|16174|17241 >NVVL01000062.1 Rickettsiales bacterium isolate NORP64 Contig_source1382A_23967, whole genome shotgun sequence
MIRKIALFTILILVTIWFSWAYLIKGNILKAIDNLASDNIKFSYQGSKISGFPFDLKLRI
ISPKVTIIDQTSLHEFSTKEIEFKFGYSFKSSVINLGKKIIYNKSNGNAKQEYTLLSEEN
IIIAAGFKRPFCFMSPSLGAVKDIASIHLTLPKLECRDRSNSSSSSDDTGSASGGGGGGG
GDSHNQPLFTASSIELLSLQETDNIEDRFSLKFTGEYDAPSQLSKIAKAHLSANLNYISK
REYKEPKEVNFEKKLEIQNIKLKLGDALLAINGALKLTKSALPQGRINVSMVKYQEIIDA
LIPENFIISPSRLKKTIDKTTTPQAINPASADFHIIFSEKGISMESSAKKLRPQT

>gene_1450|GeneMark.hmm|230_aa|-|17402|18094 >NVVL01000062.1 Rickettsiales bacterium isolate NORP64 Contig_source1382A_23967, whole genome shotgun sequence
MTDFQIYSELYSRLGIKDTVYLAFRDIPELINKHVSGRKTLDFGCGAGRSARFLKELGME
VNGVDSSIEMITQAKKIDDSISYEHISNNYFTQHSETYDLVFSSFVFFETSSLDLMLEIF
QLIHKTLKNDGIFILITGSMEMYSHNWLSLDANYPENKNPTSGSLVKIKLKNVDLELDDY
FWTDDDYKITAAKAGFSLLEMHNPIGTKHDGYKWKSEEIVSPYSIYVFKK

>gene_1451|GeneMark.hmm|247_aa|-|18102|18845 >NVVL01000062.1 Rickettsiales bacterium isolate NORP64 Contig_source1382A_23967, whole genome shotgun sequence
LADNLKINNLMNQVTKNNILAELMKECGIDDIKLSQYTNVPISSISRIRLAKNCNPTIST
LQPLADFFDVSIEQFLNYQPLDDDRIPGTHKPTLFTSSMLPVIKWSDVIDFIDNKETFFS
NKKIEWVASEQETSENSFAVEIPDASFALFLKKGSTILIDSEKTIKNGDFVLFKVKNKNL
IVLKQVLIEKDNVYIKSMNSKTNTTRTIPESYITLGNVIEIRCNLQSAPKQNNPRTNAIN
ILNPLKI

>gene_1452|GeneMark.hmm|312_aa|+|19103|20041 >NVVL01000062.1 Rickettsiales bacterium isolate NORP64 Contig_source1382A_23967, whole genome shotgun sequence
MFFKSLSSLFVDSISLNKSCKYKVLSIDEGQIPPEVICEIIADGTEIKFKLEDIIKENKL
KYFMSKDALQIANCFQKIKSNSNAIRERNNQDNKYLHTLSQMFTVLFIVSNIAAAKLSSF
QGIAFCGGIITFPMLYVLNDVITEVYGFRASRRVIWTALFYNLLAFSLLYLAIFLPHHDS
WSNHEAFEKVFSLSPRIFVASMTSYLVGEFFNSVLLANLKIKFNGRFFGLRALFSTLSAS
FVDTAIFFFILFWGVIPYTEMIPMVVILGLVKVLYEALLIPLTIRLVAFLKLKENSDVFE
KPSLSNMMLFTG

>gene_1453|GeneMark.hmm|74_aa|+|20046|20270 >NVVL01000062.1 Rickettsiales bacterium isolate NORP64 Contig_source1382A_23967, whole genome shotgun sequence
MILEDKDNTPELYAKYKITGTPYLPFRDMPNILNKYVEGKKALDFGSGAGESTIFLKSLG
FDTIGVDINEITRV

>gene_1454|GeneMark.hmm|60_aa|+|20367|20549 >NVVL01000062.1 Rickettsiales bacterium isolate NORP64 Contig_source1382A_23967, whole genome shotgun sequence
LQSGARVKIEFRNIDLTIFDYYWTQENYTEIFQKAGLHIIEIHNPLGMQVMGMNGGTRQC

>gene_1455|GeneMark.hmm|972_aa|-|20739|23657 >NVVL01000062.1 Rickettsiales bacterium isolate NORP64 Contig_source1382A_23967, whole genome shotgun sequence
MLFGVEIGLDMGIVIGFLLLTLIIGVFFCKGVNSIKQYAIGDRNFSTGVLAATITATWIG
GGFFSLTISKTYSDGINYIILGLCEVVAFLVLATLIAPRIKSFLGSLSVAESMGTLYGKN

AQIITALVGIARVTGTIAIQFKVCSTVLTYFFGISSTYSTIVSASIIMFYTALGGIRAVT
FTDVIQFITFTIFIPILSMTIWGTIDNPQIVLDTINTNPALNFTKLASLSGTELYSFIAL
IILFIIPGLDPAIFQRIIISKDITQAKHSLYIATGAFLFISLVTFWMGVLLLSNAPDLNP
VDLLGYVVDEYTYTGLKGLLLAGILAMLMSTADSYIHSASILFSHDFCVPLNLQWAQKNE
LLLSRIFAVVISIFALMLALSTANLFEIILVMAGFYMPLVTVPLLLAIFGFRTSVKSYSF
GIIVGFFSTLFPRIFDLGIDGAIIAVISIFLVALSILSFHYLLNQTGGWKLYQKEVLCTN
NTCSHSCKKLLWKVQNFNIVEFCKMNTPKNEITYTLVGLFFIVAVFSTMYSISFDIRHQY
PKLLEFIYHSVLTISSGFLTYPMWPPRFKNQNFGAIFWIMGLFYILVFVGCLQVIISNFG
QFQLMVFLLGIVILSTLVRWQAALLLIITGCISSVYFFKWYAGIEQLNGGFGGLQFKIMY
VLILLSSILIVFFKPKQEHLEQTEHRVGELSDEVGTLVAVVSSRDSQISTLDTKVSGLEE
RVDHYTERTTNQAQEIERLGATAQRILNNVNHELRLPVGNVMNFSEMLHDGLGKLSDEQL
KMLSKEVYQNSNRLSTMILNMLDLATLQAKKIELQIKLANFSELVEERLTACRKIYVEEK
PLKFIIKIEKNVMVPMDANYIRQTIDNLVINAINFSEKGKILVIVARQTNFVTFTIQDQG
IGIPLSELYDVFTPFKMGSNTETKAEGRGVGLALCKAAVNAHGGEVKVESSGAGVLFRVV
LPLESDRGRPRS

>gene_1456|GeneMark.hmm|91_aa|-|24062|24337 >NVVL01000062.1 Rickettsiales bacterium isolate NORP64 Contig_source1382A_23967, whole genome shotgun sequence
LVTVAKQTNFVTFTIKDQGIGIPLRELYDVFTPFKMGSNTETKAEGRGVGLALCKAAVNA
HGGEVKVESSGAGVLFRVVLPLASDRGRPRS

>gene_1457|GeneMark.hmm|468_aa|-|179|1585 >NVVL01000063.1 Rickettsiales bacterium isolate NORP64 Contig_source1382A_24304, whole genome shotgun sequence
MAKYEKNIMSFKDLPAFLKLLEKNGHLKRITIEVSTKLEITEISRRFLAKDGPALLFENV
IKEDGTKSEYPVLTNLFASKQRIAMGLGMKDASELREFGALLAFMKHPEPPTSLKETFSM
LPIAKRILSMPPKTVSKAPSQEVVIKNPDLSILPIQTCWPGDVGPLITWPLIVTKGAGSE
KTDNYNLGIYRLQPAGRNKLIMRWLKMRGGAQHHEKWKRTQQKPMPAAAVIGANPALILA
AVMPIPNTMSEYNFAGLLQNKAIDLVRCKTSDLKVPAHAEIIIEGEVSMSEYLPEGPFGD
HTGYYNDVELFPVFNVTAITMKKKPIYLSTYTGKPPDEPSILGEALNEIFVPLIQQQFPE
ITDFYLPPEGCSYRICIVSIKKTYAGQSKRVMMGIWSYLQQFTYTKYIIVVDDDINIRDW
KEVMWAISTRTDPKRDSTFIESAPIDYLDFASPESGMGSKMGIDATNK

>gene_1458|GeneMark.hmm|356_aa|-|1558|2628 >NVVL01000063.1 Rickettsiales bacterium isolate NORP64 Contig_source1382A_24304, whole genome shotgun sequence
MQTYKKYILSSILPPMAMLTLILTSLVWITQTLKYIFLIDKGIALATFFRLTILLVPYLL
YMILPIITVITIIYVYNHLQDGRQLIIFRNSGLSNYQLAKPALLVAICFTIITWYISMHL
MPFSYNLMKQEMANFKEGYISKIIDAKTFNQISKYHTVYVDKKNPDGTLEAVLLFDNKVS
ANRTMLFAKTGRITISDEQHIEFELNDGFRHSYDHLGNLTKLYFDNLAVIINGETNASHT
RKNSELFINEMLFPDASLPLKKRNRLIADGHSRLVWPLFNFAFVFLALSIFLNQPHNRRA
HIKPFILTFLPLLLASYLHFTLQKLAYKNLDYIFLCYANVFLCIICAIWQNMKKTL

>gene_1459|GeneMark.hmm|496_aa|+|2904|4394 >NVVL01000063.1 Rickettsiales bacterium isolate NORP64 Contig_source1382A_24304, whole genome shotgun sequence
MRGKLTVGETTIDLFDMNLQKVLNVLNTLTTNQEEVVFKKAEFTGRGFLDAILSVYSRAC
SNGEEADMLLMPLFVKRMKALFAKNELWGTAQEPGVLQVLGATELDRLLILNEDATIPDL
QARLNRLIQAPQAQLTVMQNLPEITNPAALAKQIRCLELGERIRNASDTSEGELSTLQFG

EASTVISSDSGGIAEIESKSLNLHLEGKRLGNASDLLFESACSNAPPTAPVLLAIIGAGE
KTWGYPMTDYRIQTMRQFFGTTFSYGDGVHEISKGQMSLAVLNTHLSCMQKESHDGGQYS
KANLGVVLFYAHGAESEDGRVTKLVEIVNTRLSSIFERTVVIIHACYSGQYHDLELGENV
FMITSASHKNPQSSLISSFNNALKDVAKYFPQGRADLPGFLELFASYEASQPAFSGKIGD
ELFKGIEIAELLSSSDIVAALEALESPDASPAASEELEQPHCTIAVHDFSNIHEDAPGNP
ALIGDDDGCALDVVAS

>gene_1460|GeneMark.hmm|112_aa|-|4424|4762 >NVVL01000063.1 Rickettsiales bacterium isolate NORP64 Contig_source1382A_24304, whole genome shotgun sequence
MTQKQIMSITDEAASQVKALLSQRGKPSVGIRIGVKSGGCSGLKYFVEYADEKNQFDEMI
CDKEITILIDPKALMYLLGTRMDYAEEEFKAGFTFTNPNEKAKCGCGSSFNV

>gene_1461|GeneMark.hmm|126_aa|-|4759|5139 >NVVL01000063.1 Rickettsiales bacterium isolate NORP64 Contig_source1382A_24304, whole genome shotgun sequence
MAYSEEVIDHYENPRNVGAMDKKDETVGTGLVGAPACGDVLKLQIKVNDSGIITDAKFKA
FGCGSAIASSSLVSEWVKGKNLDDAMAIKNTEIAKHLSLPPVKLHCSMLAEDAIKAAISD
YKQKQK

>gene_1462|GeneMark.hmm|418_aa|-|5164|6420 >NVVL01000063.1 Rickettsiales bacterium isolate NORP64 Contig_source1382A_24304, whole genome shotgun sequence
MHNYTELKQRIVDEGGRFPIYLDNQSTTKTDPRVVAEMMPYFTDKFGNPHSRSHSYAWET
EDACEIGRARVANLIGANPKEIIFTSGATESNNLALRGIAQFYGSKKKHIVTVVTEHKCI
LDTCRYLQQDGFKVTYLPVQANGIVDLNVLRDAITEDTMLVSVMAVNNEIGVIQPLKEIG
KICREKGTFFHSDIAQAFGKIPINVNECNIDLASISGHKIYAPNGIGALYIRRKPRVRVS
PMITGGGQERGIRSGTLSTPLVVGFGKAAEIAGLEMEKDQKHVRKLFDKFLTAIQSNAEE
VFLNGDKEQRYHGNLNLSFAHIEGESMILAIKDLAVSSGSACTSASLEPSYVLRALGVDE
ATAHTSIRFGFGRFTTEAEVDFAIEVILANINKLRELSPLWEMHQEGIDINSIKWSTH

>gene_1463|GeneMark.hmm|373_aa|-|6413|7534 >NVVL01000063.1 Rickettsiales bacterium isolate NORP64 Contig_source1382A_24304, whole genome shotgun sequence
MIYLDHNATTILHPFVKTKMDEIALFPINPSATHSYGRKGKGILENARRSIARLLCFDDC
HKDYQVTFTSSGTEANNLILFNFNDAEIFISATEHPSILSHKKFSSNIQTIKVDEDGILD
FDDLINKLQNSKSEKKLVSVMLANNETGVIQPIKKIAKIAHEHGAIMHSDCVQAIGKINV
NILDLDLDFVSISGHKFGGPIGVGALIGKASILLKPMILGGGQEKNLRAGTENVHGIAGL
GEAALLAKKELTSRNTHMRKLQKQLESALLESFPEIKIAGMKSERLPNTSLILNSRKKAE
IQLIAFDLKNIAISSGSACSSGKIGLSYVLEEMGCSEREARSAIRVSVGAGSTEEDIDNF
IKIYKEINKESYA

>gene_1464|GeneMark.hmm|129_aa|-|7537|7926 >NVVL01000063.1 Rickettsiales bacterium isolate NORP64 Contig_source1382A_24304, whole genome shotgun sequence
MAVTEVAESSDGNTPMKLADISTNQNIPLNYLEQIFLKLKKARLVNSVKGPGGGYYLAEP
SDKINIEQIVNAVEENLKMTRCSKDKSCRKNGVSCKTHNLWKGLGRQIRNYLASISIADV
VSGNVNLGE

>gene_1465|GeneMark.hmm|110_aa|+|8219|8551 >NVVL01000063.1 Rickettsiales bacterium isolate NORP64 Contig_source1382A_24304, whole genome shotgun sequence
MLYLLVGILFILGRLAIKLITKKGVIVMPKEACTNKSEIWVNKSGTVASKDDDDDAVATL

GQAVSSKVTGGNIEIVTVGESFDEGEGSNEGEGSNEIVAAGETANSEEGQ

>gene_1466|GeneMark.hmm|422_aa|+|8946|10214 >NVVL01000063.1 Rickettsiales bacterium isolate NORP64 Contig_source1382A_24304, whole genome shotgun sequence
MKTIKRLLQPLILEALQSFPAVYINGPRQAGKTTLARTLLAKEFKAKYITFDDPLERAAA
MRNPLGYVRESGIPLIIDEVQMAPNIFRSLKMLIDEQRMEALSSSSAAASSSANGHYLLT
GSANLMVIPELADAMVGRMATLTLLPLSASEVKNSTSHFLDYCFTKDFSKIKADNTKLTE
IMRQASFPELMQMPESMVDKWLGNYIQKVTLEDPRYIYNLEKAEFMPALLQSLAVRAGNL
INDASIGREIGLNVVTTRNYRALLNGTFVTNTLSPWYRNITKRLVKSDKIFFHDTMLLCY
LLQSTPEELVKNQPSRFGHVLENFVLSELCKANFASGEKVNISFYRTNDGREVDFILEKQ
NKLVAIEVKHAENINDKDLAGIKEFQAHTGNDFCCGIVLCNTPRVIAYGKDIYLVPFSAL
WQ

>gene_1467|GeneMark.hmm|603_aa|-|10600|12411 >NVVL01000063.1 Rickettsiales bacterium isolate NORP64 Contig_source1382A_24304, whole genome shotgun sequence
MHTFRTHHCNELKSQHVGQRVKLSGWVHRRRDHGNLLFIDLRDHYGITQLVFTDKDLSLK
DKATHLRYESVITVEGTVVARSNETANNSIETGQIEVDAEKYILESDSEMLPLIVNSDQD
APEDTRLKYRFLDLRREKLHSNIMLRSNIISEMRKLMTEAGFTEFQTPILTASSPEGARD
FLVPSRMHPGKFYALPQAPQQFKQMLMMSGFDKYFQIAPCFRDEDARADRSPGEFYQLDI
EMAFVTQEDVFAAIEPILFNIFNKFGNKKLSVSSFARIPYAEAMLKYGTDKPDTRNPLEI
TDVTDLFKDAEFTLFREAAKKGAVVRAIPAPGASTKSRKFFDGMTKFAINEGAGGLGYVN
FAEDGTAKGPIAKFLNDDQLAKLQELAKISAGDAVFFACDKELAAAKLAGKVRTKLGQEL
ELIPDDVFELCWVTDFPFYEMNEDTKKIDFSHNPFSMPQGGLEALENAKTEEELLAIKAF
QYDIVCNGIELSSGAIRNHKPDLMYKAFEIAGYSQEEVDKEFGGMIRAFKFGAPPHGGIA
PGVDRIVMLLAETANIREVIAFPMNQQAEDLLMEAPNHVSEAQLRELNIMLSPSVKSKLA
KKD

>gene_1468|GeneMark.hmm|286_aa|+|12488|13348 >NVVL01000063.1 Rickettsiales bacterium isolate NORP64 Contig_source1382A_24304, whole genome shotgun sequence
MQLIKNQEDFDKICEKLETEQTLFIDTEFQRKKTYYAKLSLVQIASKHHKVVIDVLLIDD
LTGLKNLLANEEILKVFHAPDQDLDIFLHIFKQLPKNIFDTQIAAWVVGLDECMGYSRLC
KALLNLELDKSLQNSNWLKRPLTDDLLEYAIKDVEYLIPLHRELSATLTKRKLWDTYNDK
SAKWLKEGTYKINPDKVIKRMKLLDYSDRYKHKIKSLILLREECAQTLDLPRFFCAADKD
LTRLCRELPTTEAELVKMNFLKKPIARRQFKKKLLELCAGLREFKL

>gene_1469|GeneMark.hmm|177_aa|+|13430|13963 >NVVL01000063.1 Rickettsiales bacterium isolate NORP64 Contig_source1382A_24304, whole genome shotgun sequence
MKIKFALLTKLVAPISVALISLVALVTSPAIASDYPQSRLEREIEETGSLLKGDGLTFRP
GKERSTATKATIGNVNKYLYQASIDVLKFAPLASSDSNAGVIVTDWYSPKGQKNTQFKVT
VYIRDKLISPEALEVVVFQRDKTGGKWPSDYKESPIAATFEDKIIRKARDLYLNSAK

>gene_1470|GeneMark.hmm|842_aa|+|13974|16502 >NVVL01000063.1 Rickettsiales bacterium isolate NORP64 Contig_source1382A_24304, whole genome shotgun sequence
MNILEIERKWQQRWDQDEAFVAKNDSDKPKYYVLEMFPYPSGKFHVGHLRNYAIGDVISR
YLQASGYNVLHPMGWDAFGLPAENAAIANDTHPADWTYANIDSMRAQLKSVGLSYDWNRE
LASCSPDYYKHEQKFFLDLLARGAAYQKESSVNWDPVDNTVLANEQVVDGRGWRSGALVE
KKYLKQWFLKITDYADELLTDIDSLSEWPENVKTMQRNWIGKSEGARFHFDVQNLDEKIE

VYSTRPEVIFGASFVAIAYNHPLTQKIAINDEMKAFIDKCAHHSTSESELEKDDKECVYT
GLYASHPFDKTIKLPIVIANFVLMDYGTGAVYGCPAHDQRDFELVQNTKDLEITQVVHSD
NHEIDLSKHAYIYGSDDVMINSDFLDGLQTDQARAKIIDKMEELGIGSRETNYRLRDWGI
SRQRYWGCPIPIIYCESCGAVPVPEEDLPVELPRDISFEKTGNPLDLHPTWKYVDCPKCQ
SKGVRETDTFDTFFESSWYFTRFCDNKAKDMADKKAADYWLPVDQYIGGVEHAILHLLYA
RFFTKAMSDVGYLNVREPFKRLLTQGMVLHATYKDKDGKWLYPEEVVEKDGKYLHAKTGD
EVFKGKIEKMSKSKLNIIDLETMLATHGADVIRMFVLSDSPAEKDLEWSMAGIDGCKKFI
SRLVSLVKKLPDLEGVNGGAEDKNLQASIHRTIADVSDDLMNYRLNKAIARIRELYNATQ
SALSAAKPDTASICEAARVMIQLLSPFIPHITEELWEMLGNKVPLYKTSWPKADKSKLVS
DSYVMAIQVKGKLRATHEFAVSATDAEIKEVAINLPAITKHIGDAAIRKIIVVPKKIINI
VI

>gene_1471|GeneMark.hmm|231_aa|+|16503|17198 >NVVL01000063.1 Rickettsiales bacterium isolate NORP64 Contig_source1382A_24304, whole genome shotgun sequence
MRIAKFIANAGICSRRAAERLIEDGEVSVDGEVIDSPALNISDENIIKVGGKLVGAIPKA
RLWVYYKPLGLITTNSDPQDRPTIFQALEGKLPRVISVGRLDINSEGLLLLTNNGDLSRY
LESPSTNCERIYKVRTFGKGREVAIRNKKIVIDGVNYHPKLISLTKKTVSNSWYKVVLTE
GKNREIRRLFEHFGFEVSRLIRTDFGKYSLGNMKPGDLLEVTIDQNYSWKT

>gene_1472|GeneMark.hmm|188_aa|+|17170|17736 >NVVL01000063.1 Rickettsiales bacterium isolate NORP64 Contig_source1382A_24304, whole genome shotgun sequence
LIRIIAGKHKNRVIPTLKKADYRPSTTKFREALFSILSSGEFAESQPVTGATLLDLFAGT
GALSFEALSRGAGSITLIDSNETHLKLAKEFAAKIGEEDNVTTLTANALLLPKSITKYDL
VFMDPPYYNDMVAKSLKCLIKRDWLANGALIAIEMSARERLDLLDGLILLKEKRYGNNKL
LILKYEQS

>gene_1473|GeneMark.hmm|445_aa|+|17723|19060 >NVVL01000063.1 Rickettsiales bacterium isolate NORP64 Contig_source1382A_24304, whole genome shotgun sequence
MSKVKKQYSCASCGSTSSKWSGQCPDCMEWGTISEEMSSASRIMVPKTGTPQQLQSLSDQ
VESTIRTKTPIEELNRVLGGGLVSGSAILIGGDPGIGKSTLLLQLSASLSQNNTGCLYIT
GEESINQIKLRAQRLDIKDTKTGLLAATNVEDIISTIDQNKKELDLVVIDSIQTISTSEL
SSAPGTVSQIRASAHMLINYAKQNSITLLIACHVNKDGQLAGPKVLEHMVDTVLYFEGDH
NNHFRILRSIKNRFGAVNEIGVFEMTAKGLIEIANPSELFLMERENNVSGSAVFAGIEGS
RPLLIEIQALIAPSNMATPRRSVVGWDHNRLSMIIAVLSVRFGLNLTSHEVYLSVAGGLR
ITEPAADLAVAAALISAASNAPLPQGSVFFGEVGLSGEVRKVSQTEARVKEASKLGFKKM
YCCSKEKLKSVEAIMHLRQLKDVIV

>gene_1474|GeneMark.hmm|597_aa|-|379|2172 >NVVL01000064.1 Rickettsiales bacterium isolate NORP64 Contig_source1382A_24651, whole genome shotgun sequence
MENSTRQVVLKDVQIDGYKRMKEILCYRSCVINTSPLGAGKTILTLKYAAETNSNLFIVA
PLTVHETWKRECRKYGVDYVDIITYDKLRGRGRSREGTSCSGFLERKAIFSHSLDRDEYF
YYPTEELEKYIEFGTLFVFDECHKIKNVNSIQHRAACKIVEAARGSASRCIFLSGSIADK
KIFTLSICRYLFLTQETRLGNLNSGGLSDVYNLAIGVNKKLANKIAKSYHDPEDVLFELY
VKIIRPIFSFYCPMGNSELKDFKNGKYNIENENYDDAVDALRDAMKNHTEWRSRFRTITK
SLVLLEKIKLHKIYKLALGTLNADSNNKVCIFLWYNDHVGDMIDFFDGKYTLAVLCGATK
SSDRQNAIDHFNQENNKCRVIISTTQTGGTGVSLDDTSGNYPRTAYCTPSYFSSDLYQLS

GRICRANTKSVSTLRVVYGNRTDEQYLIKSLSDKGNIMLKFLGYGKNIVDIPGSFGSYNE
PKERENEYETKPKVELKVILSDESKVELEETLEENLEEVLSDESEVELDEPLDENLEEVL
SDESEVELDEPLEEKLPYEPEGKYERENKIDNFTLAAQGRNIAECEVSEDLLKKLLG

>gene_1475|GeneMark.hmm|260_aa|-|2271|3053 >NVVL01000064.1 Rickettsiales bacterium isolate NORP64 Contig_source1382A_24651, whole genome shotgun sequence
MDKINVCVLTLKKESENVKRISSIFSDEIYNLILIPITNIVDSVSNVSDVESHLDTKRML
HALDMSKTIDPEAITIILKDTSIFSSKKDHVLEVIKTSLEVEDWDLVYLNRWLDRCDLYT
DDRHRVGNTFTEIIRTRSPNGTQAIMFSVIGRDRILGVEPLRDSTYFKLVNISIDTLLNI
SIENSSLFAYVIVPNLVEFDIGVSSTLSNLAKMSECRAPPDVSNKGLLPFTLFLGIVICT
FLLMWAYGKISKVSIKDSVV

>gene_1476|GeneMark.hmm|339_aa|+|3075|4094 >NVVL01000064.1 Rickettsiales bacterium isolate NORP64 Contig_source1382A_24651, whole genome shotgun sequence
MESKFIPLVLAMIMVFATIIVIVLAVSQKGRLQKLGENCSRYPCSRGLTCQSNKCKSNSG
EFCNTLTDCTSDNLSCLNNKCTNVGGSIGNICTELLPCNPELICEKGICKSKLNGSCDNN
SECIYPFICVEGTCREDIKVQTCDLLDIMTYGCIKLLLFSNKIKRINDNLGISMDITSSV
EISRFFMYNGLLHSLGVDGKLYALSVPIEHNYWEWEEQLWCVNEIDHVSVTLDEHNIWIQ
RKYVHISGTRGYLYEGNSLISTHSLNVNVIRNYGITTLEYVEINKKTMVMYATPSGVILN
NVSNARIGFDSKIVHSGGELIKVIFDRMVKVSIETYTTY

>gene_1477|GeneMark.hmm|260_aa|+|4106|4888 >NVVL01000064.1 Rickettsiales bacterium isolate NORP64 Contig_source1382A_24651, whole genome shotgun sequence
MSEFNEKSYKGSIALSLLKVKKTQIQMLIDRKIVDISLLPTHILDETYGIGDFLEEFQGG
GGFRENISNTYKKLNGDNLLIIYSNTSKKSKTTGNSTIKLIIDRVPEITDGSRNHLIVIS
ELKLSPQAKGIIKNIQYQMKMEHFIYSELETNPSSHFLTPKHEILTDSEVIELYSDHRIQ
ISNIPRIVKDDMMCRYIGACIGKTLKITYDNSFYDSYLKEYIEYRAVVSSVNIDDGEITT
DYQEKFEDEFSISEYYDEEF

>gene_1478|GeneMark.hmm|249_aa|+|5162|5911 >NVVL01000064.1 Rickettsiales bacterium isolate NORP64 Contig_source1382A_24651, whole genome shotgun sequence
MGESLESLICDNNNIRQIKCNSKLLSLKCANNNINFLSPNESLEILDCSDNNIRSIEANK
NLRVLICRKSNLNKIKLSNELIYLDCSYNIIYTIHLGYNLQYIDCSNNYISCITLHGDIQ
YANCSKNPLTSVRFTGKSNILYNDYIECALKHKRTKTHSVTKIGKICNISDKPIEFTRVV
KAQITLYSMCYKVLGKSNKRAIELNHLNWARCFYCLVSYPVDLLGLNETQTYSRAICYLC
HNSMHNLMI

>gene_1479|GeneMark.hmm|389_aa|+|5955|7124 >NVVL01000064.1 Rickettsiales bacterium isolate NORP64 Contig_source1382A_24651, whole genome shotgun sequence
MDVKETSNKLEDLIYDVKYSGLFGQYIFDIKLLEVSLEKLNSMVGAKKIKESVVKQIKYI
LMTKIEGRANEFPLHTILYGPPGVGKTTFGTILSNIWKSIGLGKLYTQTPLDSDSPNLTY
MKNLIPKISGFIASTSEVRTSVMKLGKESVHPLKLIYGLELDIIFAQIELEEYIIEIEEF
RKSQECPKIVSRSDLVAEYTGQSGPKTRNVLQSASDVLFIDEAYSLYNGRGDNFGEEVLV
EINRFMSESKDKCILIFSGYRDKMEEGIFKAQPGLKRRFSWIFELDGYTGKELSEIFILQ
TKGKLWDLGEIDIVTFFVNNLKYFERYGGDTLRLVFQIELLLLSTDFGDFLTSNQIKNKF
IGKEIFSNAFKIYKENIGPVKSPNLNYYI

>gene_1480|GeneMark.hmm|269_aa|-|7108|7917 >NVVL01000064.1 Rickettsiales bacterium isolate NORP64 Contig_source1382A_24651, whole genome shotgun sequence
MENSLEGLEKLRDGIIYDNVTNYIYSSPHTVPKIIHQTWKTNDIPNVWKSSRESWIKYHK
KYVYVLWTDEMNRKLILDYYPWFIEYYDKFPYNIQRADAIRPFILHRYGGLYSDLDIKPT
KSFEPLLKHKSSVILLSDNGRYTNMIMASNIGNPFWINVFDAMKSPNIPWYCGTRHFYIQ
ATTGPILIDKVARNFKGKGNIIDFPSKFVQPCSICESKPCTSKKAYVIMLEGSSWHYLDS
KVITFLSCKYKSLLFIFVSIIALIFIYNN

>gene_1481|GeneMark.hmm|62_aa|+|7986|8174 >NVVL01000064.1 Rickettsiales bacterium isolate NORP64 Contig_source1382A_24651, whole genome shotgun sequence
MNFYFYVDGFVTVNVNMSELILLNSLLDDDEFNDPTALASALRIIRGKINVFLPLIFDYH
EW

>gene_1482|GeneMark.hmm|57_aa|-|8586|8759 >NVVL01000064.1 Rickettsiales bacterium isolate NORP64 Contig_source1382A_24651, whole genome shotgun sequence
MVHIVIISLIILLVLINISAGAVAFIMRRDLIDCETNESPYCLKYICPNGKEATRDT

>gene_1483|GeneMark.hmm|469_aa|-|8760|10169 >NVVL01000064.1 Rickettsiales bacterium isolate NORP64 Contig_source1382A_24651, whole genome shotgun sequence
MIKDMYILLTDNDMKVSERKINKGGVGSSSKYLYVSIIASPSGSGISNIDILYSYGSHNN
RIEAKFSKPDPSRLISIEASNLLDKLKLIIITYSSKQLGNVLSNTEYIGITNLQKYIYKF
VGCYALTLALYLLMSHNDNDIGNPFTYLNTTTPLMEMSIDNIVNNNMNYYNNYYTKVISP
MKTGSIGKRLLEQSKVFYDTWGEFLPNGKVLDNSEALHSSIIGMSKYMDDKMDKLLSLLN
DTEIQMFRHKEEISDISIKSMDNITNQQNKTIDLQNIFLSNFNDLVAKATDEISNGIIDI
KNKCTSGKEMISIHSKSMISEIDSKVSTMMDAFDDQKNQSTEEILELKEKSIETISLYES
NHTSKQKDAFETLRKGIVKELDKHRDDGVLFYDSLMGKLTSRYETLTKNIDLVEVESTEK
IESLVSDLMGDMETLVENVIGKIQKENIEFNNAITFRINNAIKKISDLS

>gene_1484|GeneMark.hmm|143_aa|-|10192|10623 >NVVL01000064.1 Rickettsiales bacterium isolate NORP64 Contig_source1382A_24651, whole genome shotgun sequence
MDTHTKLVDVHVKYLRGGNQEKTYDNLREWIDDKDNVYIGRQGRVNILDKNKTSKVFGYG
RAIFANPNSGNPKLGEYRKHIENLIEEGTITIADIINLDGKNLGCWCVKGKRKGGKDDPE
RCHGNILMDILNDYRKMYPKYSN

>gene_1485|GeneMark.hmm|269_aa|+|10753|11562 >NVVL01000064.1 Rickettsiales bacterium isolate NORP64 Contig_source1382A_24651, whole genome shotgun sequence
MNKINKKRFSPEIEHKNFDVFDVYKGNDTRVVEVDVEELEEIIKSLRKVAKVKTSELKGD
DRKNLDKIISYVSVLYDTDSYKILYGIAMKHWSDLKIISPGTVGSYFAGCIRTSSSSDMQ
GCEVICAGSMPRPNESDEWKQCDKAVIWGKYHKGKYSFNVLQNNSDMSHSILHINCTNYN
TCPKFSPEDIDTLKSIGVETITLRGYKNNGRDHLDLIGKEISVDDLVTNENGKESSHTLT
FIVIGFLIFAFCLWQSKYFGLDIQKYLQG

>gene_1486|GeneMark.hmm|189_aa|+|11735|12304 >NVVL01000064.1 Rickettsiales bacterium isolate NORP64 Contig_source1382A_24651, whole genome shotgun sequence
MCAYVIDSSISLDELDQFLKSNACVYGLMNVIMKKGNETNSTLILLSDKYYRIIKSIKNL
KLYGIDTFVPMKRKNCNLSDKLRVTIPRNVSPGVCRKHITHLLMIMKSYNLVTDDVEIII
PLVSRMKSIHTGVCHIYLNSEKTQNTYIIKNLLDYSYWPMTEKYIRCFHVTPTGGVWVND

SKLTILRKR

>gene_1487|GeneMark.hmm|1091_aa|+|12642|15917 >NVVL01000064.1 Rickettsiales bacterium isolate NORP64 Contig_source1382A_24651, whole genome shotgun sequence
MSATHDAGIYKRPNEIEHVLLRPDSYIGSVNSVNREGRIFSGGKVVEKVISTPEGMVRLY
LELLTNASDNLYESMQKGVPCTFIDIKVDKYSVTVTNDGLMPPIEYSDKYECYIPEMIFG
MLRTSSNYTDDNKRVAGRNGFGAKLCNIFSSSFSLDLSNENGKKYTQQWENNMKTISEPN
IGLAKGKPSITISYTLDFKRFGCDGYTKEDIALFASFAIDTAVTCKIPVKFNGKMFNFKK
TSTYLNMVFGKCKMEGFVQGKSRVPDLEVYLMDTPYAGRIYSFVNGIPTKDDGVHVSSVL
NSIIRPIIRTINKNIKQSDIRSGVTMKNATSHISMYISYRCIKPEFTGQMKSKLNKPKPR
ISVPVGMVDKVGKWTFVTKLNNILKDKCQKKELKTDGKKKKHISVDTVEDANFAGHRTKY
RFCKLYVVEGKSAMAYAIKAISSMEGRRDFNGAFPMKGKPLNVMRHPNKVFENQEILNLK
KVLGLRENVDYKLKINFSKLRYGSMVIAADSDVDGKHILGLIINLFNCKYRSLLELGYVK
YMRTPIVRVSRGKITKKFYTMDQYKAWCLSTNPKQKWKHDYLKGLGTSTDAYIKDDTENQ
VIVIPFLDKDSEDNLELAFHPDKTMERKSWVTSDRMEIGSYEGKQNISEFINAELVEYSK
YNLTRSIPGEMDGLKISQRKILYGSMLIWKSNKNKVKVSELGSAVSSSMGYHHGVFSLGN
AIKSMASDYVGSNNLSYFSQEGQFGTRNMGGKDAADGRYSAVKPEWWWPYVYKEDDIPIL
SMVTDDGKVREPVTLLPIIPMSLVNGARGIATGYSTFIPCHSITDILSWYEKKLTGSVLF
ELCPWYRNYTGKIQLITLDNKSHRMVTSGSFEMVRKANKDVTRVTELPIGRWNHSYGLWL
KSKLEKKEITDFDNHSTHLVPSFDIKGFTNPTIANLKLYKTYTMDNMVLLSEGGMPRKYE
NVTEILEGFYVKRLGYYVKRKEYKLESLNIEINDLTSLSKFIMAVVNEEIIVFKQKIDDI
YKKMDTMGFNRDFLKRVPLHKCTKDYISELERKISTMKEYSNELTNTKESDMWLKDLLDF
RKKYLSVYGLD

>gene_1488|GeneMark.hmm|103_aa|-|34|345 >NVVL01000065.1 Rickettsiales bacterium isolate NORP64 Contig_source1382A_24665, whole genome shotgun sequence
MKDCETQTIPIQLHPTPPPLTTNQTQQTSPPSLPTPPQTDESIINYESDHNPHLPNPIPE
VIRAGQRSLSVSKYGISCSSNVEKTSVHSYNSGKMVHKHNIDI

>gene_1489|GeneMark.hmm|78_aa|+|886|1122 >NVVL01000065.1 Rickettsiales bacterium isolate NORP64 Contig_source1382A_24665, whole genome shotgun sequence
MSALTIRLPDEILDEVDKRSAKLHISRSEYIRLSIAKMNKGICEDERRAKLMETSHRVRK
ESMRINSEFAKVEHDPEA

>gene_1490|GeneMark.hmm|119_aa|+|1106|1465 >NVVL01000065.1 Rickettsiales bacterium isolate NORP64 Contig_source1382A_24665, whole genome shotgun sequence
MTRKLNLGEIWLADLGSKLGTELGKLRPVLIIQNQTLLDANHPSSLVIPLTTNLIDDAEP
LRIRINAQGKLDRDSDLLIDQIRAIDNTRLHIGALVLCNEDFMKKVYQAIAEVIGDKNS

>gene_1491|GeneMark.hmm|129_aa|+|1493|1882 >NVVL01000065.1 Rickettsiales bacterium isolate NORP64 Contig_source1382A_24665, whole genome shotgun sequence
LSNAILTILDLRIWLHLGCSEQERHQPQCVSFDISITFPKGLKGTSSDDINDSFCYGEAT
KLIKETISSKPYNLIEHVASDVHEVLLGSLREKGFPEASLLVKITKLSPPVLDIHGGVSF
TYAPAGGRA

>gene_1492|GeneMark.hmm|444_aa|+|1891|3225 >NVVL01000065.1 Rickettsiales bacterium isolate NORP64 Contig_source1382A_24665, whole genome shotgun sequence
MIYISLGSNMGNRLSALRDAARLIKTNILTHSYESIVIETKALLKEGAPASWNKPYLNMV
IAGKSDLSPVKLLEALKNIEEKIAGSRYKERWSPRVIDLDILIYNDQTIQAPGLNIPHAE
LLNRDFLLHLLALMPEEYMPLIQTNSSTAPSDGAMVRADEYAHGALEYAGLFDKSFSLAV
PIAGILNVTPDSFSDGGKNSSPEMAIENAKQMVLDGAEIIDIGAQSTRPGGIEIIGAEKE
YERLAPVLDGLKDLDAKISIDTYHPALIRRLLKEYKIDWINDVTGNLEDDILQEISAAGC
KILTMHSLSVPPSPANILPHAQEGGAISLLKEWGRGAFERMQRLGFSAEDIILDPGIGFG
KSIYQNLALIRQMQELQELQELGVSILVGHSRKSFIFGLSTAPAKERDIETMVMSGNLAS
KVDFIRVHNVYDHHRALIANQLSL

>gene_1493|GeneMark.hmm|179_aa|+|3203|3742 >NVVL01000065.1 Rickettsiales bacterium isolate NORP64 Contig_source1382A_24665, whole genome shotgun sequence
LLINCHYNYGIVVMSRKIIGIMACTNKGVIGLNGAIPWRYPEEFLHFKNTTDGQIVVMGR
RTFDELANLDLLSSRDNIVFTQNKSLLKSKMAENIRFISSLEEFEKLTLNPDKKIYMIGG
AKIVELFLKNDMIDEFLLTKIHKEYDGDTYFPLDLMKNWDSECVDTREGYSIYSYKHKN

>gene_1494|GeneMark.hmm|223_aa|+|3752|4423 >NVVL01000065.1 Rickettsiales bacterium isolate NORP64 Contig_source1382A_24665, whole genome shotgun sequence
MNFLDRLHKELDQYHLLKHPFYQAWNEGELSVDTLKIYAKEYYGHVAAFPRYISAIHSRC
ENIKDRQVLLENLIDEEQGEENHPELWLKFAEGLGCKREDCKTQELDSTHKLVDEYFDIT
RSSYASGLGALYAYERQTPAVAKSKIDGLKKFYAINDEDTLKFFTVHMVADEWHTEECAQ
LLEKMDAENQELAMNGAVNGAKLLWNFLDEMQEVHLGNRALAS

>gene_1495|GeneMark.hmm|302_aa|+|4676|5584 >NVVL01000065.1 Rickettsiales bacterium isolate NORP64 Contig_source1382A_24665, whole genome shotgun sequence
MAKKRQVHSDDIIDAAQTGKNNRLLSAFVFKRFQIDYDSDAPEDEDERYDSLMCDALFYA
ARNEQDEAIKILLNAGYTDISGLEHYSGYKKIRQLKDEMPLPLQSVDSRDVIYAAQDGGN
NELLLAFVSSTELQIEYDSDAPEDEDERYDSLMCEALWHAAFNEQDEAIKILLNGGYTDI
SGLEHYSGYKKIRAGLLEELSIKLFTAARSGRNLELLSELVQSELGFSESELRKVIEDMI
EIAAFNSQQGALTRLLEGSNIDVSSFGVLVENPAVGKTNPAVEKTLSTLGDASSDVDEDA
LG

>gene_1496|GeneMark.hmm|294_aa|+|5693|6577 >NVVL01000065.1 Rickettsiales bacterium isolate NORP64 Contig_source1382A_24665, whole genome shotgun sequence
MKNYLNQIKLSILIGIMVFIGSFVFKANAQIVQASSVNEIKKYTSQSQYKGEDTLVLFDI
DYVLLTPRDAVLRYAGENNNYRSKHFKAILKDFQGKEIVLDKSRMPMAEYLISQVLSSSK
VELVSQDMPSFVNRLNAQKTTVVGLTSNSSGKYGIVQNEAELHLSRLKSLGYNFKDNGNL
LKGDFPQYVSPVIFTNKTDKGKMLLGFLKQLSKSYKNIIFIDDRLKNLISVQNALKDRQI
NYIGINYTELKNKNELLNQDIADKQFQLLSQEHIWLSDEETQARLNAKSNQEAK

>gene_1497|GeneMark.hmm|188_aa|-|6622|7188 >NVVL01000065.1 Rickettsiales bacterium isolate NORP64 Contig_source1382A_24665, whole genome shotgun sequence
MQISVSGQQMSVGASLQEYVKERAVEVVSKYFENAPSARVSFSKQGRVFVCDIMVNDGTG
RGMVIKSNSSGDEVYNSFDAAIIKIEKQLRKYKSKLKDHHKRMKVSQITPESVKYIIAPT
SVAEELVEDNPVIVAEKPISVLPLSVSDAVMKMDLEDLPALMFENAKTGKMNVVYYRKDG
NISWVDSK

>gene_1498|GeneMark.hmm|450_aa|+|7399|8751 >NVVL01000065.1 Rickettsiales bacterium isolate NORP64 Contig_source1382A_24665, whole genome shotgun sequence
MTKSKTKKKPAKSKDLKHVQDHYLDYPYPERNPEEEKTRLMKVYGDYLGEINHWLFEGKE
DFKKGFRVLIAGGGTGDSTVYLAEQLKDTDADVVYLDFSKNSMKIAQERAKNRGLTNIKW
VNDSILNLPELGLGKFDYINCTGVLHHLESPDAGLKCLTDSLTERGGMNIMVYAEYGRTG
VYQIQNMMNLVNDGVKSRQDEVKNAWTMINSLPNTNWYSRGKDLLADHVKFGDIGMYDMF
LHKQDRAYTVPQLYEFVENAGLNFVEFNSPHSRVMLKIETYVKDPEMLKKIKAMDKSKQH
AICELMCGSIIKHSFYVSKKKKPIAKFSNLDNTPYFYTVKNVPTQIADYIKENPQIVGGV
VNFSWTSDFLGKVTINIPASKYTEHLFRHMAGETKSFKEIFKAIEKDLGEKIDSSVLMTE
VNRILPMMGESGSLLLRDKKVSIASMISSS

>gene_1499|GeneMark.hmm|591_aa|+|8771|10546 >NVVL01000065.1 Rickettsiales bacterium isolate NORP64 Contig_source1382A_24665, whole genome shotgun sequence
VSESTKNKSANGKYWSARSYDENFIQELSRFLGISDFLARLISVRVSSPADAQDFLEPKI
KSLLPDPFHLRDMEEAVTRTMLAINSGEKICIFADYDVDGATSSALLKNIFREMNVESSI
YVPDRISEGYGPTIQGMEKIHASGAKLLITVDCGSVAFEPLEHAAKLGMDVIVIDHHISM
DELPKSVAVINPNRLDETSECKNLAAVGVSFLFAVALCSRLKESGYFKEKNIPPPNLMKQ
LDIVALGTVCDVMLLSGLNRAFVTQGLKVARARKNIGYASLCDVAEISEALSCYHLGFIL
GPRINAGGRVGKASLGANLLSTGSKLEADKISAELDRYNEERKVIEMTMLEEASEMAKNQ
EDSPMLFITGQGWHPGVIGIVAGRLKEKYTKPVAVIALNDGIGKASCRSIKGVDFGCTVI
EAKQKELIIAGGGHSMAAGFTVNEEKLNDLQEFFNKEFAKKLAGSDAHLREHYDADLTSS
AATLELLEEISKLEPFGNGNPSPVFRFSNLYVLKADIVGTKHLRIMFSPSRDAYGSKPLT
AIAFNAAGTELAECILSRKPHTLSVFGTLKVNKWQDRETVQLQLRDVLIEE

>gene_1500|GeneMark.hmm|34_aa|-|10577|10681 >NVVL01000065.1 Rickettsiales bacterium isolate NORP64 Contig_source1382A_24665, whole genome shotgun sequence
MQIIIYIDKEFIDFYKLSPQGENIPYFYKLRPQG

>gene_1501|GeneMark.hmm|226_aa|+|10965|11645 >NVVL01000065.1 Rickettsiales bacterium isolate NORP64 Contig_source1382A_24665, whole genome shotgun sequence
MNTTDNTVDTTTEIETRPDGTRIEKRTVHLKNSGLSREEADSTMTKSGEPNQVASTLAGG
ARALGSIVEGITGAPDTVLSLAGKAMDLAMSSGKKDDPELPPEPESPGVPDFDSARSSLY
GNPFDSDSSPEIAPMPRAVVSRGESMFDIPDEVQVQAAQIRWGQAQHSRDQSQHVRPPAT
PSDRRGSIVEDAIFEIRDSLIGNIVGQMGGDLLGGSSDYAEPPEVT

>gene_1502|GeneMark.hmm|185_aa|+|11811|12368 >NVVL01000065.1 Rickettsiales bacterium isolate NORP64 Contig_source1382A_24665, whole genome shotgun sequence
MEPVSITLIILVAGAVISGCGWHHHHSRNQETHTHETTTTEKTTITTPGGTVTEKTAVTI
EIDTDITSEESSGEGAEGDPEVAKGLVNIAGNLLAGGGVFDAVAGVGELFGSGDDDDDSD
AEAPMLDAPFRSDSGEPGEPNAARARLTGIFQLVIDNPGVLDANGDSEGVMGDSTAALEP
SDAIV

>gene_1503|GeneMark.hmm|530_aa|+|12573|14165 >NVVL01000065.1 Rickettsiales bacterium isolate NORP64 Contig_source1382A_24665, whole genome shotgun sequence
MKIMKPFIALSCSLLLTLSAWGSANAKDDVSTKSHASTSAKASIKDATNINPGAITRAKP
KNIAKSEDKLEIDDYIAMCKQDLQFVRSQMKKNSAAYANKIDKEFHRWFKEGNKNTLKLI

SKIGDLDDCYYAIKYYINGFHNSHISIRGYIALPSEEYPGLLSAKKGKGHYIIYKHPALK
YLKNVNIGDELTHINGIKIDRYYKDFLLPFYANDKSELTLESASIYALIVDGNRFKPSPR
RITLKQKQNKDINIDLKYTELKGAAVEAAKKIKQPDRSAPFKVELVSNGVWIRIPSFFPA
RNEAIYYTGMLSMLKNDLAKEDYILFDMRGNRGGASKWSRPIIRNLWGDAFIKSLGKKHD
YNKKWQKNLRVSAENFPSFKKIYSPAASKSYASLIKRKKDFFLKKWSIYNDKENLYTNLD
SKPFNAKIYVLTDHFCRSTCWKFVNELKQIPGVTHIGEETTIQSIYSYAKRERASSEHFD
FFYPTQIRVKPSYKLGTALVPSEIYKGDFRDEASVIDWALSITEKEDEDF

>gene_1504|GeneMark.hmm|154_aa|-|14162|14626 >NVVL01000065.1 Rickettsiales bacterium isolate NORP64 Contig_source1382A_24665, whole genome shotgun sequence
MIISSIQEFYEFFQNGMPILSIDYGGKKLGVAISTPDHSFSMALQMISTESEKEKFRQVL
NIAEEKNICAIVIGWPVNMDGSKSDQTLIVERFAARLEGRTKLPIFLQDERLTSKAADNL
LKKFGVKRKDRNSRDDMTAASMILETTLESIKRL

>gene_1505|GeneMark.hmm|431_aa|+|14642|15934 >NVVL01000065.1 Rickettsiales bacterium isolate NORP64 Contig_source1382A_24665, whole genome shotgun sequence
LVYKKVSVFKFTKIQLDVKLIKASLVFALLYCILFNSAVFVYKFEFYQANLFTASLELTK
DFIYLTVTLFMFFFGLSTHRILFIVGSILLFVTGAIASYYLFFFSISPTPSMMPAVFCNP
AADASELISTSLIVWISFCLAICLYGIYYFKIQTSKSFITRFLSFLCILFVVSNILLPKF
SFLKTYFPVQYLHNSYIFLQGQLNEPLREDINTKFSFVDKSDDDVTGVLVIGETARYDKF
GINGYGRDTTPNLSELDLVSLKARACATSTYYSVPCMLSRYTEKDIDLVETESSFLSVLT
RLGINTTWLGTQTCTKYYKNTQNYSFYDEVNLRVIPGGSLIFKANCHDGKMLPYIKKNIE
SHNKNFLVLHTTGSHWNYARRYSEEFEKFTPSISKKTKDPFSCDLEKLKNSYDNSMLYTD
FFLSRVIEMLK

>gene_1506|GeneMark.hmm|954_aa|-|1|2862 >NVVL01000066.1 Rickettsiales bacterium isolate NORP64 Contig_source1382A_24681, whole genome shotgun sequence
MQNKPCNPHAIGVLLVFGILLTGKCRYMLFGVEIGLDMGIVIGFLVLTLVVGMGHGKSVK
TIKDYALGGRNFSTAALTATIVATWASGSGFFITMSKTYSDGLIYMFSRFGIGLSFLILA
FVLVPRMGEFLGKTSIAEAMGDLYGRHVRIITAIAGTLGALGSIAVQFKVFGSMFSYFLH
VPDYIATIAAGMIVTVYSAFGGIRAVTFTDVLQFGAFGMIIPIIGFIVWNDFYYSGFTIV
QTASDSSFNLVEIFSINNASLLEMGVMFFYFSIPTISAPAFQRIAIGNSISQVKKAFLIG
GVILILIQVMTAWIPFLLHTLDPSLEAKHILGHIITTYSYPGLKGLMIVAVLALAMSTAD
SRINASSVLFTNDICKLLIPNLKKEMFVSRSFSFALGIGGIALSLSESDLLAIMLLASSF
YYPIITPPFLLSVFGFRSSAKAVLIGIAAGFATTVIWKILPISFVSVSQKMVGIFFAMLM
NTAFLFASHYLLKQKGGWVGIKDSSYIDKQKIIRREKWAAFVSKAQNFNLINYIKTRPVV
EDSAYVTLGAYFLIFTFTSIYSTHAEMLKANGDIIGVIYQIMIVSSTMMGMYPIWPLSIK
TQIKQMVMRVWYPLSIFYMLIVFSTFFVLLNEVNNMHYIMFAVNMIVSVVLLGWRMALGM
IVVGVYMGVQFYKYYAGVEYLDISIGSPQFILIYSSMFVVVILLIFLKPKQEHQKQTEYK
VGELSDEVGTLVAVVSSRDGQISTLDTKVSGLEERVDHYTERATDHAKEVERLGATAQKI
LNNVNHELRLPVGNVMNFAEILHDGLGKFSDEQLKMLSKEVYKNSNRLSTMILNMLDLAT
LQAKKIELQIKLANFSELVEERLTVCRKIYVEEKPLKFITKIEKNVMVPMDANYIRQTID
NLMINAINFSEKGNISISVVEQTNFVTFTIQDQGIGIPLSELYDVFTPFKMGSN

>gene_1507|GeneMark.hmm|76_aa|+|2857|3087 >NVVL01000066.1 Rickettsiales bacterium isolate NORP64 Contig_source1382A_24681, whole genome shotgun sequence

LHKYEDASTNSDPGYSRESGLDGGFSLPETLDYSNSKNPFSGRALRELLQLFAARHGMRQ
GAYHRFQGKLSAEVSP

>gene_1508|GeneMark.hmm|203_aa|-|3320|3931 >NVVL01000066.1 Rickettsiales bacterium isolate NORP64 Contig_source1382A_24681, whole genome shotgun sequence
MIDIELLIDGHKRFRKKHFDKDKKLYQELATEGQTPKTLIIACSDSRVDPAIIFDADPGD
LFVIRNVANLVPPYQPMNESYHGTSAALEFGVNNLKVEHIIVLGHSGCAGIKSLCDHDDE
GSKKFSFISSWVNIAKEAKEMAQKGKPSSVEEMYSLCEKESILTSIENLLTFPWIKEKVK
NNTLALHGWYFSLNNGKLLRVKY

>gene_1509|GeneMark.hmm|459_aa|-|3928|5307 >NVVL01000066.1 Rickettsiales bacterium isolate NORP64 Contig_source1382A_24681, whole genome shotgun sequence
MNELSCFKAYDIRGKVPSQINENLAYKIGVALHTMLAPKTVIVGYDIRLESPKLAESLIQ
GLTDSGVGVINIGLCGTEEVYFHTFHKEQDGVCAGVMITASHNPKGHNGMKIVGKGSRPL
STEDLKKLQEHVRALGPDALPYSEKGLKGTETIDEDKSSYITHLLEYVDISKLKPLKIVV
NAGNSAAGVVIRLLEKHLPFEFIYMNDTPDGNFPNGVPNPMIVENRTATSEAVIAHKADF
GVAWDGDFDRCFLFDEQGKFIEGYYIVGLLAEIFLQKSSPEKIVHDPRLVWNSIDIVQAA
GGVPVMSKSGHSFIKEKMRQENAIYGGEMSAHHYFRDFAYCDSGMIPWLMVAELISSARE
NLSALVDERVAAYPCSGEINYRLDDIQAVIGKIEQYFLPKCKFVDYTDGLTIEFDDWRFN
IRSSNTEPLMRLNLESRGDVDIIKKRVEEIESLFQGEEK

>gene_1510|GeneMark.hmm|208_aa|-|5433|6059 >NVVL01000066.1 Rickettsiales bacterium isolate NORP64 Contig_source1382A_24681, whole genome shotgun sequence
MLLELKKRYAEKKAKKLALGGLLEFTERLSNELKILQVHGASPEKIQLFQEWMIEVYKVK
ATEAAGNKSYWFNNKKNITDEVLSMVSILLPLELEAAFTANLDIELASMEEQILKFFKEC
QEDLGLNNDYSKIFQKELHDILGVDRKLILESFREGLDLSLHKASPLPTPDWQSYLDAEF
GRLCESASLYLYKPHAPEVEIGGMDIDS

>gene_1511|GeneMark.hmm|89_aa|+|6357|6626 >NVVL01000066.1 Rickettsiales bacterium isolate NORP64 Contig_source1382A_24681, whole genome shotgun sequence
MIRKEPVMALRRNLGQVLNEVEYKHDTIIITRDGHASAAIINMNLFEKIRQMKNRFSQLT
RELQSAFSDLVEQEKDDLIDEALLEARIK

>gene_1512|GeneMark.hmm|831_aa|-|6676|9171 >NVVL01000066.1 Rickettsiales bacterium isolate NORP64 Contig_source1382A_24681, whole genome shotgun sequence
MKLVIVESPAKAKTINKYLGTDFKVIASFGHIRDVPSKKGSVLPEEDFSIKYEISERSER
YVDEIVKYAKQSDEIYLATDPDREGEAISWHITEVLKEKKIITDDSKFKRIAFNEITKKA
VLAAVASPRKIDLDLVNAQQARRSLDYLVGFTLSPILWRKLPGCKSAGRVQSVALRLICE
REDEIERFISQEYWDITLDMLSDAGKPFSAKLSHVDGKKLEKFSITNQEGADDIAARLKT
QEFSIASITKKQQKRNPAAPFITSSLQQEASRKLGFGAKKTMQIAQKLYEGIDIGGETTG
LITYMRTDGVTLAADAVTSIRELIEQKYGDKYLPAKARIYKSKSKNAQEAHEAIRPTDII
RTPEELKGRLDNDQLRLYDLVWKRTIACQMENVIIDMVGADLITADKSFTARATGSTIAF
DGFYKVYQEGLDDSKDEKSKMLPPLAEGEDLSTKKINPEQHFTEPAPRYSEASLVKSLEE
LGIGRPSTYASIISVLQERNYVTVEKKRFFPNDLGRLVTAFLVGFFEKYVQYDFTADLET
DLDKVAEGTIPWKNLMSNFWDGFNKNVEKVSEQKIGDVIAYVEKSLDFHLFGKEGTEEYK
KNKKCGTCPDGDLSLKLGKYGAFLACSNYPDCTFKKQVSKGDSNEEAQTIIDNTKDLGQD
KDGKTVYLKKGPYGWYVQAGEAESKKDKPKRSPLPATVKPDDLDLPMAIKLLSLPLHVGN

HTETGEEIVMGIGKFGPYLKYQNKFTSIPRAIDHFAVTLEQAVELIAEKAKKDAAKKGAP
KKVVAKKAVAKKVAPKKATPKKAGVKKVGAKVAKKAAPKKVAPKKAVAKKS

>gene_1513|GeneMark.hmm|379_aa|-|9177|10316 >NVVL01000066.1 Rickettsiales bacterium isolate NORP64 Contig_source1382A_24681, whole genome shotgun sequence
MLDNLFVRNKASTYSDETIDILRLIRSENVGSRTYTLLIKKFGSARNALENIGDFSLRGG
RSKPIKIFPKSSAEQELEKLRKNGAHLITHKDYNYSQLLAHIPDFPPVISYKGNISLLNS
QKIIAVVGARNSSLNSRSFAYKLSKDLVDAGYVTASGLARGIDTEVHKASGAKTIAVIAG
GIDHTYPPENKKLQEYIEKEGLLLAELAIGSKPLSQHFPQRNRIISGLALATIVIEASLK
SGSLITARFALEQGREVFAVPGFPLDPRCMGSNKLIKDGAQLLESSEDVIANVPLYKKSL
EESKTYSNNLKAPAIDDAANIASSDRENVRTLLSSMPVTYEMIADETKLSLPIIYTICLE
LELAGKIVRYPDNKIALLY

>gene_1514|GeneMark.hmm|200_aa|-|10334|10936 >NVVL01000066.1 Rickettsiales bacterium isolate NORP64 Contig_source1382A_24681, whole genome shotgun sequence
MNPFIGKKAPDFTAKAVMHNDIIEHDFNLKSHLKGQKGVLFFYPLDFTFVCPSEIIAFNN
RVAEFTERNTKIVAVSVDSHFSHHAWRSIPANKGGIGNVQFPLVADLNKEISTAYDVLNE
DSVSYRATFLIDEHFNIRHFLINDLPLGRNVDETLRMIDALDHHNQHGEVCPAGWQKGDQ
GMNPTHQGVADYLAVNAEKL

>gene_1515|GeneMark.hmm|335_aa|+|11197|12204 >NVVL01000066.1 Rickettsiales bacterium isolate NORP64 Contig_source1382A_24681, whole genome shotgun sequence
MQFIDESKIYIKAGNGGHGAVSFRREKYIDRGGPDGGDGGRGGSIIFRSNSHLNTLLKFR
YQRHFKADNGEYGKGRNKTGKSRDPVILEVPLGTQIFSEDGLLLIHDFVEDQEDFEILEG
GKGGLGNSHFKSSTNQAPRKRIDGETGAEMDVCLKLKLLSDAGLIGLPSAGKSTFLSTTT
SAKPKIAEYHFTTLKPGLGVVYVSEEEFVLADLPGLIKGAHLGSGLGDRFLKHIERCGVL
IHVIDGLRDDIAEDYKTIREELESYSPLLKDKYEIICLNKIDAMGEEEILEKMQILEELT
GKKINPISGYTKNGLDIVLKQALRLIKEDDIEDII

>gene_1516|GeneMark.hmm|387_aa|-|12586|13749 >NVVL01000066.1 Rickettsiales bacterium isolate NORP64 Contig_source1382A_24681, whole genome shotgun sequence
MSRSSNSENITISTVSVAYMVAQIRAKQLASKASQDELLRHIVEYLESEAAAEITGASFA
TIMAELVETELKHSYSFEDLELVGYPIKYNKTKLILEMVQSWVSTSDDSLMADITNNTLH
NGQGNTVLSELPRLQAPSQVSSWGNENPVVNTALLLIGASHFTQDDLTFTGAATFFPFFD
PTHVVDGYSNIFGASNGMILVGDYQYGGHRYFSNPAHGLKQQLFGAEDCSSAVAKATGLG
KYVVEVTTTSLREAYTSPKWGGHYTAITSTYQEEIDWAKVQPGDIYIRGGHTAIIASAPD
QHGFETLEFNRDIESNTKHKLLGGGMYHRTTDPQGKDIYFLCQPAKDFKESCSATDLLQL
IDQRYEEMYGDNVPVDVAGDCGIFLQL

>gene_1517|GeneMark.hmm|636_aa|-|13888|15798 >NVVL01000066.1 Rickettsiales bacterium isolate NORP64 Contig_source1382A_24681, whole genome shotgun sequence
MDVTVAAYYIKEEKISYSEYIDNMNIINKAVTEMQKNLMQDYGVYKKTRFGILVSSIQKI
IAIDQEFKDLLFFMGLINYKNISRDLLINFKNEKIVNNFIAYLKEFSIITEERKNKNNKI
DNFTILKSSNIIIRHYLQQLLFLGECRVIAARGAKILQQYLKFNEKKLSSIDRDNYYEHI
EAFLQNKDLLDNDILAYYSKILGIYYVGNENYDKAKKLLENALEYYNLNKKMYGKEIANI
SVNSGELYLFSGGYEKAKKSLEIARQFYSQNIEEYECVLSWTYGLMGYTCRALEQYEQAR
IYLDKSLAIHSGCSNFDKKTRLLTYLGSVHRNLGNYDLAITLLTSANNLIIDFYGKNHFK

KYWIDNYLGQTYLCKGEYLKAKELLEVAVIKYQNLYGKKDKRTAEISIWLGTLKSYIGEY
AESQAILSFAHKRFVKIYPENHLKIAWCATSLGEVYRQLKEYDLSQRYLEQAQAIFSLKL
AKNNIKIAWNTLNLGLLYRDQNKLVKAEQYFQKSFDIYMKNNGLQHKRTATVLAELGYIK
FLKGDSAAGSGMIKKALRIMVTKNYTRRYIALIQLAEINFSKLKNNLSDEEKRNITNQTL
LYMNQALEIVTNNFPSSSTCSEEIQYKMKQYQNRLP

>gene_1518|GeneMark.hmm|262_aa|+|45|833 >NVVL01000067.1 Rickettsiales bacterium isolate NORP64 Contig_source1382A_24823, whole genome shotgun sequence
MKKILLTTAAAAVLATSSAYASEADTFSVGVHAGMIKPTDEKFGGMKYKSSAAGFAELSV
NYAIMDNVRVGAAFSHFFNPKYKASWEVKHADIDGGTFKHILSKTKAAAASVKAAADLAE
PDAKFKTVLKAKFSTFLVNGFVDVADLGAAKIFVGAGVGLSRTSAKFKKTSTTVATKAIP
ANTATNQKEVKVGDVLTGPTKSGKIKSKTNFAYALYLGASTEVAPGINAELAYSFRGFGK
LKDVKKASLKGHNVSVGVRFDI

>gene_1519|GeneMark.hmm|96_aa|+|1954|2244 >NVVL01000067.1 Rickettsiales bacterium isolate NORP64 Contig_source1382A_24823, whole genome shotgun sequence
MNKFIKSLVNKEQDSFLTQILDILSPMYNLSTETSESGVVSIFKGGAKFAIIEGETMKLA
DKNGSFTEIDAPIKSADALLQVATQAYWIAIKNIAS

>gene_1520|GeneMark.hmm|168_aa|-|2502|3008 >NVVL01000067.1 Rickettsiales bacterium isolate NORP64 Contig_source1382A_24823, whole genome shotgun sequence
MSESPNFAIGDKVVYPSHGVGQIIEIESQDIAGMQIEVYVISFPQDKMTLRVPVSRGAAS
GLRTLVTKKDISEVYNILNSKPKRGNKMWSRRAQEYETKINSGEIDAVAEVVRDLYKNVD
NDRSYSERTIYESALGRLAAEIAVLEDISSQESTEKLVELLKEKLVAA

>gene_1521|GeneMark.hmm|644_aa|-|3095|5029 >NVVL01000067.1 Rickettsiales bacterium isolate NORP64 Contig_source1382A_24823, whole genome shotgun sequence
MLISSLFASAALNSIDFGSLPAKSGGIVGVNVEQFTLSFSQILPYKQFINPGYITLAVIF
FILALGVRLRVYKRIVFGFLGFLFRLISATGILQKLGSIDNGKAYYASDEFSYAGGDEEE
ASPFATRRSAKPQRRARQKSRGDSDNMYELPSVDLLDDEKHQGAKPQTQAMLNENAEQLL
AVLKDFGVKGQIIDVRQGPVVTMYEFEPSAGTKSSRVIGLADDIARSLSAIATRIAVIPG
RNALGIELPNKNRMFFRLKDLVKSDEFQTSDISLPLILGKDLAGVPLVADLAKMPHLLVA
GTTGSGKSVAINAMILSLLYKYSPDECKMIMIDPKMLELSAYDNIPHLMTPVVTEPGKAV
VALKWAVKEMENRYRLMSHLGVRGITNYNIRVQEAAKNGEVLERRIQTGFDPETGKPTHD
TVPIDMNKMPFIVVIVDEMADLMIVAGKDIESSIQRLAQMARAAGIHIIMATQRPSVDVI
TGVIKANFPSRISFKVTSKIDSRTILGEQGSEKLLGMGDMLYMGNSSKILRVHGPFVDDK
EVERVTNYLRSTGTPEYISAVTEADEECGDDSGENFSSDDGENDIYTQAVNIVRLERKAS
ISYIQRSLRIGYNRAANLIDEMEKNGIVSSPNHSGKREILLPED

>gene_1522|GeneMark.hmm|97_aa|+|5028|5321 >NVVL01000067.1 Rickettsiales bacterium isolate NORP64 Contig_source1382A_24823, whole genome shotgun sequence
MAIVLNVAQSSFSLRNKIHAQEMQHMDIINADQPQNWKSISARYDPKYNRRLLGSLSVNK
LNDGSCGLYEARETTLASASTNNIVDLDLLCAKNFFI

>gene_1523|GeneMark.hmm|119_aa|-|5432|5791 >NVVL01000067.1 Rickettsiales bacterium isolate NORP64 Contig_source1382A_24823, whole genome shotgun sequence

MSLFEFIVVLIVALLVVKPEDLPKIVKKFKQMQSFITNTKKEIISQISIDDELEEIMQDS
SSPNLNKEMEQVNYYLEKISNIGPEYEGEYSLAAIKKHYLKLVKEKMADSNSAKQDSNS

>gene_1524|GeneMark.hmm|555_aa|-|5837|7504	>NVVL01000067.1 Rickettsiales bacterium isolate NORP64 Contig_source1382A_24823, whole genome shotgun sequence
MSYQYVYVMKGLSKSINGKEILKETWLSFLPGAKIGIIGHNGAGKSTLLKIMAGVDKEYD
GEAFIADGVKVGYLEQEPQLDPTKNVFENIMEGLAEKKALIDEFNEVSAKFAEEMTDDEM
NDLLARQGDLQEKIDACDAWSMEREIEIAMDALKCPAKDADVTKISGGEKRRVALCKLLL
EKPGMILLDEPTNHLDAESVSWLENYLKEYKGAVVAITHDRYFLDNVAEWILEVDRGKCV
PWHSNYSAWLQEKQAKLASEEKEEGDHSKKLKRELEWIQQSAKGRHTKSKARINAYQELL
NKKRDVGNGSGQIIVPNGPRLGELVIEVKNISKKFGDKNLLTDFSFRVPRGAIVGIVGPN
GAGKSTLFNMITGKVKPDSGIINIGDTVKLGYVDQSRDHLDDSKNVWEEISEGLEVLDLG
GMSMKSRAYCSAFNFKGAQQQKKVGQLSGGERNRVHLAKLLKEGANVILLDEPSNDLDVD
TLRALEDAILDFAGCVFVISHDRWFLDRIATHIIAYDQDSNATWFEGNYDDYHNYMTNIV
GQNTKNPKYKHKRLA

>gene_1525|GeneMark.hmm|216_aa|-|7530|8180	>NVVL01000067.1 Rickettsiales bacterium isolate NORP64 Contig_source1382A_24823, whole genome shotgun sequence
MKLFLDSVDSAEIKKYSALGIIDGLTTNPSLMVKSKIGFYETVSELTSLVKEDVSIEVIA
KDFDDMVKEGERILDVAKNIVVKLPITWDGIRACKYFADKGRKVNMTLCFSANQALLAAK
VGATYVSPFIGRLEDAGEDGLGLIAEIREIYDNAKFETKILAASIRTVAHVTDAALCGAD
YATLPANIMDELVKHPLTDKGLAKFDEDWAKSGMRI

>gene_1526|GeneMark.hmm|282_aa|-|8185|9033	>NVVL01000067.1 Rickettsiales bacterium isolate NORP64 Contig_source1382A_24823, whole genome shotgun sequence
MRNKALGRGLSSLLKEEIVPIIVSQSENSLSLDLIEASDNQPRKYFDEGKIKELADSILS
HGLVQPIVVNRVTSGKYKIIAGERRFRACKIAGLENVPVIIKDLNNKDILEVALIENIQR
QDLTAIEEAEAFQKLMDEYGYTQSELGVAVGKSRSHVANLLRLNHLPDSIKLMVNHGQLS
MGHARCLIGLENAEELAKKIVANDLNVRQTEELVSSKRKNDRAPKMQGKESAPVDEDLVL
LGQSLSEKFGVKVVVENSWNGGKISFHYGNLEELDSILTKFN

>gene_1527|GeneMark.hmm|255_aa|-|9042|9809	>NVVL01000067.1 Rickettsiales bacterium isolate NORP64 Contig_source1382A_24823, whole genome shotgun sequence
MSAKIVSVVNQKGGVGKTTTAVNLATIFAIMDKKTLLIDIDSQGNSSSGLGIEQSQRKVT
SYHIFCSLDSVKNAVIQTEVPNLSVIASNTNLAGVELELFGQEGHEFILKNALESIKDDY
DYIIIDCPPSLNLLTLNALVATNEIIIPMLCDFYSLEGLSHLLKTVEIVEKKLNPQIKIG
GILFTMYDKRHKLTEQVERDVRSCLGAMVYNTTIPRNVRVSEAPSHGKAAVIYDHHCSGA
RAYISLAKEIMSKET

>gene_1528|GeneMark.hmm|194_aa|-|9806|10390	>NVVL01000067.1 Rickettsiales bacterium isolate NORP64 Contig_source1382A_24823, whole genome shotgun sequence
MIDVREYVSRGDVGAFVELLLKWNNTINLISSKTTANVMDRHVLDSLQILEFIGDKDVSI
IDFGSGGGFPGILLSIAGVRKVTLIESDARKAAFLRQASKISRHEVEVINDRIENVENLE
CDIITSRAFADLDSVFGYSRNIQVRDKYLLHKGESYSDEMLKAKKHWLFNTIVHDSITSN
RGKILEIRDVEYIS

>gene_1529|GeneMark.hmm|614_aa|-|10387|12231 >NVVL01000067.1 Rickettsiales bacterium isolate NORP64 Contig_source1382A_24823, whole genome shotgun sequence
VKRKYSVVVIGGGHAGCEAAAAAARVGADTLLITLKPENLGEMSCNPAIGGVAKGVLVKE
VDALDGLMGRVTDKAGIHYKMLNESKGPAVWGPRAQADRELYRAAMFDELTNYKNLTILY
DSVEDITTNSVVTKNNGEIECSKIILTTGTFLSGMIHIGQKQIPAGRDGEQASYGLSATL
KRFDFKLGRLKTGTPPRIDGSTIDYSKTERQPGDKVPRPFSEMTDNVEIPQIDCYITRTT
LKTHEIIKANLDKSAMYSGNIEGVGPRYCPSIEDKIVRFSDKESHQIFLEPEGLNISTVY
PNGISTSFPEDIQREWIKTIPGLENAKIIKPGYAIEYDYIDPRELKPTLESKKVSGLYFA
GQINGTTGYEEAAAQGVISGINAALSALGKSDFILTRADAYIGVMIDDLISFGTTEPYRM
FTSRSEYRLSLRADNADLRLTPKGIEAGVVSSKRQTLFENKLNTIESTKKLLLSCNLTTT
KLAKLGIAIAQDGTKKSAYQLLGLPNFGLTKTKEIFPEIASLNPSALNYLKIESQYSSYL
KRQSLDIKMFQEEESYTIPDDVNYSDIQSISTEIKEKLSYHRPTTIGAARRISGVTPTAL
TAIIIYLKTKYLKK

>gene_1530|GeneMark.hmm|140_aa|-|12243|12665 >NVVL01000067.1 Rickettsiales bacterium isolate NORP64 Contig_source1382A_24823, whole genome shotgun sequence
MTIEYTFSIIKPDATERNLTGKINSYLETAGLKVAAQKMTILTKEQAEEFYGEHKARPFF
GGLVKYMTSAPVILQILKGENAIAKNREIMGATNPEDAAPGTIRKDFAKDIEANSIHGSD
SADSAKREMEFFFSKDEIVA

>gene_1531|GeneMark.hmm|458_aa|-|12739|14115 >NVVL01000067.1 Rickettsiales bacterium isolate NORP64 Contig_source1382A_24823, whole genome shotgun sequence
MKLSLFNSLTRKREVFTPINPDLIGMYVCGPTVYDHPHIGNARSVVVYDVLYRVLTHLYS
KEKVRYVRNLTDIDDKIIARAIEENTSIDELTKRTTEYFHKDMNYLGCLEPNFEPKATEH
IAEMVDIINKLLSSQIAYQANNHVYFDVSKAKNYTKLSGRSFEDMLEGVRVENSTDKKHP
NDFVLWKPAHQNDDESAKFQSPFGEGRPGWHIECSAMSHRYLGETFDIHGGGADLIFPHH
TNEIAQSCSAFPGSEFAKTWVHNGFLTVNHEKMSKSLGNFITVDDLIQKGVRGEIIRLFL
LSNHYRKPLDYNDKAISDAAKMLDYWYRAIEDINPPLVQTPPSVFIETLLDDINTNNAIK
IINDFAKQAHAATNEDQKLEAVGNLLASARFLALLNTSPAKWFKCEKNEERINILIAKRA
QAKQLKDWALADSMRAELLSIGVVVEDKPDGSSSWRKV

>gene_1532|GeneMark.hmm|279_aa|+|14186|15022 >NVVL01000067.1 Rickettsiales bacterium isolate NORP64 Contig_source1382A_24823, whole genome shotgun sequence
MDEFLNKQETEQINKKATIQMVTFGMITILVIVTLVSCIRIYSNKKEQVLNEMRMESSLL
KTVITDQLNYSLYSVDIIGDHIKSEPRNLIHIHHTLKSHFASRDFNVLFGWRKYSWMDEN
FLEIVTSTQGVVSNPRYASFVKDIVENTKTIKKGGENKIVFYTSHNSLKGNSLKIIDNIF
NAKGKYVGSAVLSYDIKTMIRSLDRRKRNSATNFVLLDKNMEVVAQSQPIIENIIDNKGE
FSNYLKFSLKMHGRDKDLEYLDMFNGLNYFVSKLDELPF

>gene_1533|GeneMark.hmm|584_aa|-|198|1952 >NVVL01000068.1 Rickettsiales bacterium isolate NORP64 Contig_source1382A_24987, whole genome shotgun sequence
MFVSQSVAMLAKLREFARELNAGYIESFAYEEQEKESVFANYIVSALCKYKELLPFLNSV
YRHDLEDCLKTHAARYVKYCSDNNQSTVDVSKFESDVKKIVQLLEKPTKGTEKDKGKDAS
AQDLKLNLIRELTCTAVKNAQLLFAEQTQNPEAKASLQAFAIAFSFEHLDADDQNSLFSK
LLLTKEIWCIDSPEEASKNGIKYNFYHNASALLKLGKKTSADETLMAVIKYLVCESQVLP
HLSDEQLISLYDRLKNHEWSSDESFLLLSELHKESRFRYSYNRLSDSGYDLLNRFQGYDA

MAHQIENLEFSIVETYLITRRVFFSKLLKLLIKHADNIRGFNLNKNEDLLSVLKKMDIWV
RDKPEKEKQNYLVMLSGFNKKLKDSGGVRVQKRYTTLDKFTVRALHERYKTLFANIGHLL
EHDELGMEFLNKIFLQKLTKEFYLPNLANSVKKLRQMDEYQKEVKAQLLELNLIDIFQSK
GVINEIHSLNFSKVAMNSSIKLPSDYIGLLTQQIVAMCTPSQLEALYSPLATQDNLSNAQ
LYLKSEIKQEIHRGVSIKKMIYSIIRGKKKEYDHLKNLKERMLA

>gene_1534|GeneMark.hmm|103_aa|-|2125|2436 >NVVL01000068.1 Rickettsiales bacterium isolate NORP64 Contig_source1382A_24987, whole genome shotgun sequence
MAYVVTDECVKCKYTDCVEVCPVDCFYEGDLMLVINPDECIDCGVCVPECPIGAIKEESP
DLIKWLEINKELSAKWKNITVQKDPLPDADKYKDETNKFEKYM

>gene_1535|GeneMark.hmm|232_aa|-|2437|3135 >NVVL01000068.1 Rickettsiales bacterium isolate NORP64 Contig_source1382A_24987, whole genome shotgun sequence
MIKLLTPNFFIKIEKVLLPILTICLPITIGIGLYYALLASPADYQQGEMVRVMYVHVPSA
WMSLGVYSFIAICSFSALVWQTRMSYILAVAAAPIGASFALITLVTGSIWGKPIWGTWWV
WDARLTSMLVLFLLYISYIAITNSGSNILRAERPASVMAIIGFINVPIVKFSVDIWYSLH
QPASVLRIGGPSIHPSMMLPLMIMFAGFIMYFILMLIMRSRTLMNKIKNNGY

>gene_1536|GeneMark.hmm|243_aa|-|3135|3866 >NVVL01000068.1 Rickettsiales bacterium isolate NORP64 Contig_source1382A_24987, whole genome shotgun sequence
MELEFIKNKVYYKIFDRTYTGSTGVYSKHKHKDAAKLAEIQQNIDNIKNKYDAQGMVLLK
QVHGTNVVLAGSECSDYPEADGSVTCSKGVALSIMTADCVPVLFASADGAVIGAAHCGWR
SAKAGIVARIRENMEAQGGKDIKAIIAPSIQQASYEVDTDYHQAFIKDDETLKALFIPST
NKGRYMFDLAGYVKRMLEQENIELKLHIDEDTYSMQEKYPSYRRSHHKGEQYSQSILSTI
MIK

>gene_1537|GeneMark.hmm|267_aa|+|3865|4668 >NVVL01000068.1 Rickettsiales bacterium isolate NORP64 Contig_source1382A_24987, whole genome shotgun sequence
MTFSLDIYYDKLSDLGGEYNLLNHQLVQFKSKTKLIPCIGAEIEFYLDGQIDLKMLKTME
KATGYVLKKEKGKNQYEIELPPSNDLASYARQIVQVREGVIHAANKLGARADFSSKPYED
DYGNSMHIHLNFQRNEDSAENKGEDGNKDIEKYARILCHYLPETIEYYLPKPEDYRRLDS
KFMAPTHISYGGNNRTLLIRIPDSKPKRLEHRLPAANANPAHVIYAMLQSIDRGLQHPGE
ISSLKKTFGNAFDPQYGLERIGARKLC

>gene_1538|GeneMark.hmm|346_aa|-|4697|5737 >NVVL01000068.1 Rickettsiales bacterium isolate NORP64 Contig_source1382A_24987, whole genome shotgun sequence
MSDDRDQGDVIDAVDVIDTIEEQDASKAQGVRNVQGEQEYLSDKPLVSFDYAIKYLLKDK
GDYGVVEGFISTLLKTRGYKDVKIISLMESESNKENVKSKRSLADLVVQDEDNNKYIVEI
ERNVKDSFVHKSLFNTSRLIVDNLAQREDYTKILKVFHISLLYFPIGNGSIYHGKTVTHE
IDTNEKLSVNIKNRDTQEVFDATDIMPEYFYISVPMFNDRLEDEMDDWLHVLKYDEVPPN
YHSPYMAQIADKLSILKMTKEEREKYSYYQKILHNDRDELKAAEFRGESRGIDIGKAEGI
DIGKAEGIDAGKATLVKMMIAQGNSVNNIAKMTGLSEAEIIKLKDI

>gene_1539|GeneMark.hmm|962_aa|-|5831|8719 >NVVL01000068.1 Rickettsiales bacterium isolate NORP64 Contig_source1382A_24987, whole genome shotgun sequence
MQEFIKVRGAKEHNLKNINVDIPRDKFVVITGLSGSGKSSLAFDTIYAEGQRRYVESLSS
YARQFLHLQNKPNVESISGLSPAIAIDQKTTSKNPRSTVGTITEIYDYLRLLYARVGVPY

SPATGLPIKSQSVSEMVDIVSALPDGTKIYILSPMIRGQKGEFRREFVSLRKHGYQRLFL
NGELCELDEMPKIDKKKKHNIEVIIDRLVISDSLGNRVAESLEASLQLGEGLTYIEIMSL
PEKHGTKYKDGQRIMLSEKYSCPESGFQLEEVEPRIFSFNSPFGACPSCEGIGKESFFDE
KLIVPDETVSIREGAIVPWSKSSSKFFMDTLASLAKHYSFNLTSPFSTLSSKAKKVLFHG
SGNEIIKFEYHDDRQAEIIEQPFGGIIPSLEEKYQRSDTAWMRDELARFKSKAPCADCEG
YRLKKESLCVKIDDLNIGQVSVKTIADAHKWFENIDSKLDAKQKLIAKRVVKEIQERLKF
LMNVGLDYLSLSRESGTLSGGESQRIRLASQIGCGLSGVLYVLDEPSIGLHQRDNARLID
TLKNLRELGNTVIVVEHDDETMLASDHIIDVGPGAGIHGGHIIAEGTPEQIKNSEKSITG
QYLSGKKYIPVPDKVRIGSKDKEIVLSGAKSNNLQNLTLRLRLGTFIAITGVSGSGKSSL
LINTFYKAALKALEPKSKIYPGEYDSIKGLEHIDKIIDINQSPIGRTPRSNPATYTGAFT
PIRDWFTDLPESKIRGYKVGRFSFNVKGGRCEACQGDGLIKIEMHFLPDVYINCDMCNGQ
RYNRETLEVKYKGKNIADVLSMTVEDATDFFEKVPAIYEKLVTLKEVGLGYIKIGQSATT
LSGGEAQRVKLAKELSKRSTGKTLYILDEPTTGLHIDDIKKLLTVLHKLVNKGNTVVVIE
HNMDVIKTSDHIIDIGPEGGHRGGAIVVQGNPDTVANCKASHTGKYLKEYLKNSPKDSAF
WK

>gene_1540|GeneMark.hmm|224_aa|-|8913|9587 >NVVL01000068.1 Rickettsiales bacterium isolate NORP64 Contig_source1382A_24987, whole genome shotgun sequence
MYKMCLTLRAKESIFQVNIYIYKIINWFTMSKKTKYPLMPKATAVWMIDNTILTFDQIAV
FCGLHPLEVKGIADGEVATGIFGVDPIVSGQLSKEELERCSKNPKATLQIHLSAAYEGVM
KGAKKRAKYTPIARRQDKPDAIFWLLKNFPEINDATIIKLIGTTKLTVASLRDRSHWNIE
NIRQRDPVLLGICSQVDLDRVVEQAKLEAKTEAKNEAKTEEKKS

>gene_1541|GeneMark.hmm|132_aa|-|9675|10073 >NVVL01000068.1 Rickettsiales bacterium isolate NORP64 Contig_source1382A_24987, whole genome shotgun sequence
MIHYQFNKLIRSKLPERMTNEGVKLHITHLSKAEYIKELKNKIVEEASEVAETKTKENLV
TELADVMEVIRALSEANDITLEEIEAARVEKRSVNGCFLPENYIHYIEVAPDNHEVIEYM
ENKERPYKKLTV

>gene_1542|GeneMark.hmm|512_aa|-|10054|11592 >NVVL01000068.1 Rickettsiales bacterium isolate NORP64 Contig_source1382A_24987, whole genome shotgun sequence
MEAKRIYDQIGGVAPKKGYVLFETGYGPSGLPHIGTFAEVARTTMVMRAFQQICDIPTKL
ICFSDDMDGLRKIPSNLPNQEMIASHLGKPLTAIPDPFGEKESFGHYMNAKLRLFLDRYG
FEYSFYSATECYKSGKFDHMMVRMLEKYDEIMNLMLPTFREERRKTYSPFMPICPDTGVV
LQVPIEKIDASRQTVFYRDEDGELVETLVTGGHCKLQWKPDFGMRWSAMDVDYEMYGKDH
RPNAEIYSAICKILGGTPPVQFFYEMFLDAQGAKISKSKGNSISIDEWMQYATPESMSLF
MYQSPTKAKRLHFDILPKSVDEYITFNKKYHEEEDLQKKLANPVFHIHNANVPVINTYGI
SFSLLLNLTSVCNPDDKSVLWSFITQYAPEATPENAPYLDHMTGFAINYYNDFVKNTKSY
LEPTDAQKTLLRQIVDMLSALNGDETGAEIQNQIYAIGTEAKYENLRDFFKEIYQILLGQ
TSGPRLGSFFKLYGIKDTIQLIESKINDTLSI

>gene_1543|GeneMark.hmm|179_aa|+|11658|12197 >NVVL01000068.1 Rickettsiales bacterium isolate NORP64 Contig_source1382A_24987, whole genome shotgun sequence
MMKNINDNPEYYDIGIMSIKLNSTCLKNPYFLLLALSVFGLLFAYFVEYIMGLAACPLCI
YQRFPYLIFIMLSIISLSGGGQSYNKYYIIITLVAIMLAAYHTSIELGIFEPSALCKPLV
SIDADFSVSDFKKMLYSQKIGTCSKPALVIFGLSMTVWNLLLNVVLLPIFIKYRNYEYK

>gene_1544|GeneMark.hmm|179_aa|+|12199|12738 >NVVL01000068.1 Rickettsiales bacterium isolate NORP64 Contig_source1382A_24987, whole genome shotgun sequence
MPKPYFEDKDKKIQEIIMVDHAGEYGAQRIYAGQLKYTNVPAEKKMIKHMLDQELEHLEY
FENKIKAGKSKPTALLPLWDIFGYGFGAISAKLGSKTAMLVTENVEEVIVEHYQEQIDYL
EKTGKDAPLLQKIKKFKQDEADHINIALDHESKNAKFNKPLSALVQAICKSAIFLSKRI

>gene_1545|GeneMark.hmm|166_aa|+|12752|13252 >NVVL01000068.1 Rickettsiales bacterium isolate NORP64 Contig_source1382A_24987, whole genome shotgun sequence
MRLLLMRHAHTHPSEDKPDIDRLLTKRGMSEAKEAAVFLCEYQVDKLLVSYAKRTMQTSK
IIQTHLSVQESEIVTELYNEGIEAIIDLLAAQEDRNKHILVIGHNPLIYEVALLLSNPTS
AEYDQLIATLMPPARVIIIDFPDAVKWGDIRKMKGNIKKIFTPSVV

>gene_1546|GeneMark.hmm|257_aa|-|13328|14101 >NVVL01000068.1 Rickettsiales bacterium isolate NORP64 Contig_source1382A_24987, whole genome shotgun sequence
LCEHQSSNDKKMMFRLWKYAISAWEPYLAKNKSLPIIAPLVLYNGKRKYSSYRRFWDLFV
NSDLAEKIMAGKCQLIDLQSMPDEDILADKEFGLFKFFMKNIKKDNGIELWKECFKKFRE
HIELDKEQELFYTKLLWSYTGNKVPIERKQELVSLILEQMEKIGKNGEEEMKTVADSYRE
EGRKGALEEGIFIGEERGEKIGEERGEKIGVEVERKKTVARMLKENFAPKIISSITGMSQ
RAISKLRSQLKLQGELA

>gene_1547|GeneMark.hmm|41_aa|+|3|128 >NVVL01000069.1 Rickettsiales bacterium isolate NORP64 Contig_source1382A_24995, whole genome shotgun sequence
NLNLDKKISKNIEYSRPAHLASKVLTGIHLYQQKQYSDNIK

>gene_1548|GeneMark.hmm|968_aa|-|251|3157 >NVVL01000069.1 Rickettsiales bacterium isolate NORP64 Contig_source1382A_24995, whole genome shotgun sequence
MSKKSKNVVAVARIGLERRTNKLAANLEILRKQHLLHKENAERIQLEPDSNRSLMHELHL
SAIIQSKNLRAFHALSQAFPEIIKNLFGLDEVFCASLLKMSKDEIINTKGFLFFAVNQYL
TNNETESLFHLAAAFGANGVLNSLMISRQNDISILDSNGRSFIYILKLCQYNLLIEQMLS
RYPELDALNEETPDAASIISTSRTEEAKLAQLNQPLSPNTHFNEGIAYYIRYVFSPDTYN
DAKLAKKASHCLTQSQHQFMCYFLTLQEGENDSLMQSNPDRTTVTCMNYLLKIYQASRSA
DNMENPESDFLLNFIKNICINYPCLQQDFIIFFIDEFHKTLDDFYLEEAYQTVISSNSLP
IHLVLAVYMVKNDTRVLYDAIHDKIGDVKSLCSLEDLDLSKGSGDLRLEFNATIELIGLE
QNEHLKSKLTAAVFLLKGLAIQQTSPQKAISFFNDVIAPSNKVKTAPKLLVDAYRLKGLA
LKNTDPNKSIDTFAKAICVINRNPYISLKTKLEILLLQSLSTYQNNDAANALHILNIAFG
NIMKFPDIQALYDAATSQHADCADCNRDLTNLRSHLSQWNIFEEFCQIIYRKFWCHLKLE
DRALASEQIKALDHYFNDPNSMHMLLLQHVLNNNSEALATFQRINPELLSDTLQTIYQRI
NEAFIEHNSRMRDESETRVQELEYKLFIQYAQTLLEYGANNFYSWFSALKEHHKIFSAQP
SCLHNPKFINQLLDQYASILERHSDNSHSLIEWRHTPESLSKLPDDLLTKTLPSISSSFH
SEILKKPSDYLIRNLPLNLLQIVFGIEAVFLEKYEYARSCFKKFESNANLILLEICRQID
QDFWEYTCDDISRNAEGLYTDLLNQLLAQPLSDDQEEKIPPALQVSNATPPTDHIAFDDI
IEYFEYVLNQLLMPDGHDISFKQSVDYAVSIDIAPLLESTLEIEAISPHENTSCSDFLLS
GNTEPWYE

>gene_1549|GeneMark.hmm|172_aa|-|3287|3805 >NVVL01000069.1 Rickettsiales bacterium isolate NORP64 Contig_source1382A_24995, whole genome shotgun sequence

MSATRKPDLSAASSGNSGSTTIDSEVATVLGELIISTQATFASLSMLNHNITVSSGGAVT
VNGEFSVKNTLIILIEEGGSFSFKGSNITFLNDATLNYEKKCSDGTTTLIGTLNRENTHD
ENLITLTSDGMTLALDEDYALLTSGESSSQNDQESAQLSGASGAPEDESDAL

>gene_1550|GeneMark.hmm|73_aa|-|3941|4162 >NVVL01000069.1 Rickettsiales bacterium isolate NORP64 Contig_source1382A_24995, whole genome shotgun sequence
MRGLFLVYVSFISVYLNLIFIYMSFVFICTHCLYLDVFVYISTYSRGIIAYRYQDEKIIF
YMMLHYRVLTNLE

>gene_1551|GeneMark.hmm|1149_aa|+|4204|7653 >NVVL01000069.1 Rickettsiales bacterium isolate NORP64 Contig_source1382A_24995, whole genome shotgun sequence
MRKVEIPLSSSSFFAWEHFKKSGKNILLCCSDEENAAAAYKQLLFLAGAGRHADILHLPS
LDSVPYDRVSPNSAILSQRADSLTKLSASQSPKMLISSAQNLLAKIPPPSLFENRTLTIS
KGLSMGIEELSVFLVDNGFSRAASAIDSSEFAIRGEIIDIVTSNKQGYRINFGWEKIESI
REYDIYSQISKNHLPSLTLSSASEGVLNEETIASFKRNFLQKFGVNHIRSPLYESILSGR
KFQGYEHLMPLFYEEMSSIYDFLGEYSVIYDNLCVQAMLEHENTYDDFYQSRLLSNKANP
DSFYFAIPPEEFILPSSEVKDILAKDGNILLEPGEGKGSREKDKKDRDHERLGSFGNLRT
QAKIEEKTEFEKLFEVILEHKNKIPVILCSAPSSIERIKNIIEEYEYTAKIIEELPRAKK
NFINLAIAPMAITPMASGFVSDKYLLISEKGILGDKTATKSHKSSERKLKNILTELDNIS
EGALVTHREHGIGRFEKIQTIYADKIAHDCLKIIYENNDILYLPVLNIDQITKYGMEGAQ
LDRLGGANWQKRKAKLKNRIKDIAASLIKMTAKRQLAQAEPVLFEQASYEEFCNKFPYNE
TEDQLSSINDIKSDLEAGRLMDRLICGDVGFGKTEVAMRAAFMGACDINEDSPQIAIVSP
TTILCKQHYSNFIARFSGTKLRIAQLSRLVKPAEARKNLHDLANGKINIIIGTHALLASG
IKFKNLKMLIIDEEQHFGVAQKEHLKNLKTNVHVLSLSATPIPRTLQMSMVGIKDLSLIA
TPPIDRLPIKTTVMPFDAVVIREALMRERFRGGLSFYVSPRIKDIEFIEEQLNKFVPELK
YKVAHGQMPASRIDDIMSEFCDGKFDILLSTTIIESGIDIPIANTIIIHKADMLGMSQLY
QLRGRVGRGKVRGYAYLTLDHKKQITKHSMQRLEVLQNIDSLGAGFTIAGHDMDLRGFGN
LVGSEQSGHIKEVGSELYQEMLDEAIAELKQQETFEKQADFTPNINLSIAILIPADYIDD
SALRLAIYRRAGDLKTYDEIESFRDEMIDRFGALPEEFGNLLRMTAMKNTCSRLKIESLD
SGPNGFVLKFNKNFDVSNMVMGFVNKYPRHAKIKPEGKLVFLQSLKPEILLKEAEKLLQE
LGDCQQKAI

>gene_1552|GeneMark.hmm|188_aa|-|7797|8363 >NVVL01000069.1 Rickettsiales bacterium isolate NORP64 Contig_source1382A_24995, whole genome shotgun sequence
LIKALKLTIASIILVWCGGFFYFLQIINHTPINSVAITDAIVVFGGDSNRLRTGTLLLQK
GYAPLIFITCESSQEEYTDLIKKHVVIPEQFIVGTDVANCKKSHAADTELFLEQYQLRSL
RLVVDSVQLPRALMELSAKLPPDTIIVPHETDIEHKDYVKVLKEYIKYSTLVVMSVLGVQ
TDLKMSYS

>gene_1553|GeneMark.hmm|243_aa|-|8360|9091 >NVVL01000069.1 Rickettsiales bacterium isolate NORP64 Contig_source1382A_24995, whole genome shotgun sequence
MLKSYLAGDNHQMTEWISLPGLVTYNDGIELMEKRLFEVIESQKPEAIFLLEHEDVYTAG
TGYKDSELLAGTSSKETETELLAGTLLQETESELLRAPKIPVIYTGRGGKFTYHGAGQRV
IYPILDLRTREKDIRLYVKNLENWIINSLAHLGLKAYTIQGMVGIWVEQKGTHAKLGAIG
VRLKKWVAYHGISINITTDLSKYSGIIPCGISNFPVTSLKELGIEVSMQEFDSILKNEFR
KIF

>gene_1554|GeneMark.hmm|636_aa|-|9063|10973 >NVVL01000069.1 Rickettsiales bacterium isolate NORP64 Contig_source1382A_24995, whole genome shotgun sequence
MKIKLLSNTTINKISAGEVIERPASVVKELVENSIDAGATQIEITLEQAGKNLILVRDNG
IGMSKEELELAIQRHATSKLDEDDLLNINSFGFRGEAMPSIGAISKFKITSRKRGSESAY
SLSIIGGTQEATVGATSNEGTVIEVRDLFFATPARLKFLRTDRTELNASVNLIKRIALAH
PSVAISLIHDGKELIKVKPLEGTSAEALKNRIIEIIGAEFIENAVQIDSVRGDNLRGDIE
ITGYTSLPTYNRASAEDQFLFINNRPVKDKILNIALRVAYQDYLSRDRHPVSVLFLRVAP
HFVDVNVHPAKTEVRFHDPNLIRGMIISAIKNALSSSSYRVSTTASDAALSYMRGNSTQP
DMQGNTQPAGFAEKASTSASATSQGGFSMPSGGRGSANRYAHTRGASSHAPRVDYALAQA
DFLAEAKPESFIETQQEELPKSRFPLGAAKAQLHGTYIISQTDDSIIITDQHAAHERIGY
EKIKEQMEKNSMIKQRLLIPEIVELPDSARADILIDHQDALSKLGLTIEKFGEKSIIVSE
IPSLVGEINPASLICDLAEHLISMGENIALTELIEHVTETYACHYAIRAGKKLGGHEMNA
LLRQMESTPFSGQCNHGRQTYVELKLADVEKLFGRR

>gene_1555|GeneMark.hmm|416_aa|-|10970|12220 >NVVL01000069.1 Rickettsiales bacterium isolate NORP64 Contig_source1382A_24995, whole genome shotgun sequence
LLKKDFIISVLTVFVQYYDYHLFGFLAANIATHFFPADEAIMQLLNTYFLMSVAMAAKPV
GAVIFGKIGDVVGRSNSFKISLIGTAIASIILFITPSYESIGLLSAFILLIARMIICAFV
SSGSDGVRIYIYENIDKSKQCLGVGVTSLFAQAGSLTASVSAWFFTMNSMPDYSWRFAFL
IGGIIGFVILIAMSKTNLSDMKPVKNHPRFEEFRDLSIGTIVKSNKGLFFWCTLLAGGIG
STNQFILIFFGTYNFEILKIIDRALMQSYISLAIIAYMIFSIVGGYLADKFDRYKVTIYA
SIMVIILSIMLSISLNQMVMRAELFIAIAAVMPFITMPSAAILKQSIPVVIRYRLFALSH
ATGSIIFSAPTALLSTLLYHKTKLTWLPVCYFITIIIMISLSLYHLNKRIISEPEE

>gene_1556|GeneMark.hmm|360_aa|-|12272|13354 >NVVL01000069.1 Rickettsiales bacterium isolate NORP64 Contig_source1382A_24995, whole genome shotgun sequence
MKFKFNIKNTHKKARVGTIETAHGEIQTPAFMPVGTRGTVKAMMPESVKATNSDIVLGNT
YHLMLKPGAERIARLGGLRKFMNWSSPVLTDSGGFQVMSLSDLRKISEDGVQFRSHIDGS
KHMLTPEYSTEIQHLLDSTITMAFDECTPYPATFEEAKSSMELTSRWAQRSRDAFKIRDG
YAQFGIIQGGVYEELRAQSAKDLIPMDFDGYAIGGLAVGEGQEEMFSVLNYAPDLLPKEK
PRYLMGVGKPDDIIGAVKRGVDMFDCVIPTRSGRNGQAFTKSGTINIRNSHFADDNEPIE
ADCPCPACMNYSKAYLHHTVKVGEMIGSMLMTWHNIQYYQDLMKRIRAYIAAGKDFDFDC

>gene_1557|GeneMark.hmm|66_aa|+|13662|13862 >NVVL01000069.1 Rickettsiales bacterium isolate NORP64 Contig_source1382A_24995, whole genome shotgun sequence
MSEVIEETEDAAIWEESVVGQEALVAEENTATKTPAPPADRPKSTSKNNKKKTEPSRQAL
QGNLRQ

>gene_1558|GeneMark.hmm|64_aa|+|13882|14073 >NVVL01000069.1 Rickettsiales bacterium isolate NORP64 Contig_source1382A_24995, whole genome shotgun sequence
LKKHLPEDLKKHLDLDHLKVERDTFVSEDLKESMSDIVYKIKTKNQKKDGDVILFLCEHQ
SSND

>gene_1559|GeneMark.hmm|97_aa|-|3|293 >NVVL01000070.1 Rickettsiales bacterium isolate NORP64 Contig_source1382A_25018, whole genome shotgun sequence
VTFHDMLRKLLESYAATRTEGNFDSKALVANLRRAAKGNTYRIYAEPQSEQGGFRVAARI

EKDVDGEVKTHLMIRADKCQSMEEAMTTSVRKAKILI

>gene_1560|GeneMark.hmm|189_aa|+|287|856 >NVVL01000070.1 Rickettsiales bacterium isolate NORP64 Contig_source1382A_25018, whole genome shotgun sequence
MSHLYYRNLEKIGFSKAEIKEIADDHEVIDGPDNEGDMFERKALPSDNFVPPYPNEQAAR
ASNNGAFPADLSLVVKARPDGANYLFSLLTGYSDAPAGVTLMPGLSYNPYFEGGQIAMPP
PLASDQVEFIDGTPATLEQMARDLTIFLQWAAEPEMEHRKSMGLKVMMFLAIFTILFMIA
KKRTWQKLD

>gene_1561|GeneMark.hmm|298_aa|+|1033|1929 >NVVL01000070.1 Rickettsiales bacterium isolate NORP64 Contig_source1382A_25018, whole genome shotgun sequence
MNTHQPEAIAIIDSMENNMYHKLRYIPSKINSMSVTKIGETLIVDSALPSDTFNTAYGGH
ITLEQTKLVMEYYLNRKMPMAWWICSSSDDNKNTKDDMESAGFVHDEFDVGMYCDLSSAD
LDEYTAPEDLQIIECRSAQEFLDFGNVLSSIFDPTDEYVKIFYSKIQAIPQDAREDLILF
VGYANNQPVATSGLFITDVAGIYDVSTKPNEQRKGYGSAMFHTALAYAKNKGLKTSILQA
SPDGLNIYKRFGYKEICDFNTWSNKAKLELQEEKSFSKLADERIATTKKWVSHEDAWK

>gene_1562|GeneMark.hmm|70_aa|-|2007|2219 >NVVL01000070.1 Rickettsiales bacterium isolate NORP64 Contig_source1382A_25018, whole genome shotgun sequence
MDNSFTLTANLIESLQELDNKHVMISPVLNVGGAVSNYTKNTDLTFEEVRNAAWLNSRNN
QSTKKLRLQA

>gene_1563|GeneMark.hmm|329_aa|+|2507|3496 >NVVL01000070.1 Rickettsiales bacterium isolate NORP64 Contig_source1382A_25018, whole genome shotgun sequence
MHFWGYMRVENKFNLSLIVILLSVLIVSCANQISNKNTQSNQGYIQVQGGKIFYKSMGQG
TPILVVHGGPGLDQTYLQPQLFEFAENHQLIFYDQRGSGKSLESTDNSSNINIEQFIADI
EELRTKLGLKKIILMGHSWGGLLSMHYATKYSKNLHGLILLNTAPANHKGQKAFVDSFME
RTANIKKDITPLFSYKEFEKQNANEIADLYRKVFSVYFYNQSDVKSLNLTFNVNSAKSGF
KVNEKMSKTAWLETGINLLPQLKTLKVPTLILHGKQDVVPLWTAKEINSVIKGSKMITLE
NCGHFPYIEKPNEFKRAVNDFINTLKDTN

>gene_1564|GeneMark.hmm|59_aa|+|3543|3722 >NVVL01000070.1 Rickettsiales bacterium isolate NORP64 Contig_source1382A_25018, whole genome shotgun sequence
MERSKSFVVKKAVEQYIITTKEDAEDIKAALQALEAGGSICSLEEMMEELGIEHDRATN

>gene_1565|GeneMark.hmm|129_aa|+|4048|4437 >NVVL01000070.1 Rickettsiales bacterium isolate NORP64 Contig_source1382A_25018, whole genome shotgun sequence
MSKDTDNIKNSRPLSPHLGIYKPQITSMLSIGHRLSGVGLFFVLAATCWWFALWVFSRFD
PAYLDILDNNFVKTILIIASYGFFYHLCTGIRHLIWDTGRCFSICAVHYTGWLAIIGSLA
LTLVYWLYI

>gene_1566|GeneMark.hmm|127_aa|+|4449|4832 >NVVL01000070.1 Rickettsiales bacterium isolate NORP64 Contig_source1382A_25018, whole genome shotgun sequence
MENKFKSDLSLAKNLGSAAHGSMHWWHQRVTGLFLAIATIWFFCFSWEISASEMSGVVEV
IKKPWHVVMLSLFVLAAFYHSVLGMQVVIEDYVHSRAWRVGMLLVIQIFSIVTAAALIVA
ALYVMTL

>gene_1567|GeneMark.hmm|595_aa|+|4847|6634 >NVVL01000070.1 Rickettsiales bacterium isolate NORP64 Contig_source1382A_25018, whole genome shotgun sequence
MSKPYNVINHEFDVVVVGAGGAGLRATFGMAEAGLSTACISKVFPTRSHTVAAQGGISAS
LGNMGKDDWRWHMYDTVKGSDWLGDQDAIAYMCKNAADSVIELEHYGVPFSRNKEGKIYQ
RPFGGMTTEYGKGKAAQRTCAAADRTGHAILHTLYQQALKHQAQFFIEYFATDLIMEDGV
CKGVLAWNLDDGTLHSFRGHTVVLATGGYGRAYFSATSAHTCTGDGGGMCIRAGLAMQDM
EFVQFHPTGIYGSGCLITEGSRGEGGYLVNSEGERFMERYAPSAKDLASRDVVSRSMTME
IREGRGVGPEKDHIYLHLNHLPPEVLASKLPGISETAKIFAGVDVTKEPIPVLPTVHYNM
GGVPTNYKTEVITLKDGKQEIVPGLMAIGEAACVSVHGANRLGSNSLLDLVVFGRAAAHR
AAEIVEKGMSHSRLKEGTIDALIDRLDEVRSSKGKILVSDLRLKMQKTMQNYAAVFRTEE
TLEEGKRLIDEIREEYKDIKISDRSLIWNSDLVEALELGNLIDQAVLTMHAAANRKESRG
AHAREDFQDRDDKTWMKHTILWLNEDGKVTIDYKPVTLDTNTDEVDTVPPVKRVY

>gene_1568|GeneMark.hmm|261_aa|+|6652|7437 >NVVL01000070.1 Rickettsiales bacterium isolate NORP64 Contig_source1382A_25018, whole genome shotgun sequence
MAEMRLPKNSRVESGEFHVGIESAKTPKRLKIYRYNPDTAENPKLDTYEIDLDSCGPMVL
DALIKIKNELDSTLTFRRSCREGICGSCAMNIDGTNTLACTKPIEEISGDIKIYPLPHMK
VVKDLIPDMTHFYAQYASIKPWLKTDTPVARGEERLQSEEDREKLNGLYECILCACCSTS
CPSYWWNGDKYLGPAILLQAYRWLADSRDEADGERLHELEDPFKLYRCHTIMNCTSTCPK
GLNPAKAIAEIKKKIVERVGI

>gene_1569|GeneMark.hmm|290_aa|+|7610|8482 >NVVL01000070.1 Rickettsiales bacterium isolate NORP64 Contig_source1382A_25018, whole genome shotgun sequence
MFEKPLISFDFAIKFLLKSKEDYEIIEGFLSALFVANNYKPVKITALLESESTKEAAHLK
KSVADLIVEDTDGNKFIVEIERSFTPNFTHKACFNTARLISDSLSTSQDYSKIQKIFHIS
LLYFETAGMEKPIYHGKTIIHEVDTEHPIDIDIMNQGIATFESKNIFPEYFFISVPRFND
QIRSEMDEWLYMMKNSEVKKDFKSPYMARVAERLKVLHMTDDERAEYHKYLKESAVQEDI
LKAATDRGLEKGRKLEKIEMAKNMLTQSLDINIISKVTNLSINEIEQLRS

>gene_1570|GeneMark.hmm|306_aa|+|8512|9432 >NVVL01000070.1 Rickettsiales bacterium isolate NORP64 Contig_source1382A_25018, whole genome shotgun sequence
MNIRDSEISSIIEKWQEHLAVQRNYSMHTLQSYLGDLNHFLDFTTNYYSKSASLGQIESI
DIRFIRSWLSARHREEYSAGSNARALSAVRNFYKYLEKKHDIHSHVVFTVQTPKKPKALP
KALSKQDVTASLNNIDLIGNKDWIHCRNKALLTLIYATGLRISEALSLTKQHLKNHEFIK
ILGKGNKERVIPWIEESRLLLEKYIQLLPYPIAPDEPIFRGQKGGILQRAVFNRELINLR
RALGLPEYLSSHAFRHSFATHLLENGADLRSIQDLLGHQSLSTTQRYTKINKTHLESVYN
KAHPES

>gene_1571|GeneMark.hmm|737_aa|-|9608|11821 >NVVL01000070.1 Rickettsiales bacterium isolate NORP64 Contig_source1382A_25018, whole genome shotgun sequence
MSRTRLNEANDNLVTAVREGKWYSIPSCLEKDADINYTTKSGKTLLMMAAKNGHTYTVQE
LLKLGADVLCTTKKGNSALTLAARKGHVDTALLLLELGADTDDIHPNDLDDGLCEFRNAL
MKKAVQKESKYIVRILLVIPDVDCEAALIMAVDDGNLPMIDLLLDEGIGIHLITREALTR
VATNGRYDVVSSLLCAGVDPNYLGEFFYPVLRDAVQNYGIEVLLKLLEANPDSIYSRDDA
GYTLLMHAVQNGCLSAVNELLKVAPNIIHDMDNERNTALKIAKSLENNPISIHITFALLE
AGANPRDLTESEMGTTLINAAKKGKKDVIQILLEVGLDLNQDDEFGYNALTYAAQNGHLV
VVEMLLEADVNPDYNEFAPMREAVTNERTGVVTALLKADANPNCTIKFGNTPLMQYAKKG

DSAMVKMLLEAGANPNDVNKFGYNALSCAPRGTLGDEVRSILLEVSDDPIPIIELSLPAF
SDTKSDPESDSEEELGEFIDELWRKTVLGYIEERLLEVSDHRPSSTLPDFSDSEEEYRAE
QLGIGKRAQTIDLLSGDINLMRQQLLTQAAENGWHDTVSLCLNEGADPHDLSRSQFGATL
IKAAKNGQNDVMQILLDAGADLNHSDEFGSLLAYAAQSGNSAVVQMLLAAGYHPDNDLIT
ALKISLENDYNGIVGMLLEKAAGPDYVDQSGDNALMRCALSEVSNHRPSSFSYIDDPEEK
HLTVESSGESSEESDCD

>gene_1572|GeneMark.hmm|660_aa|-|11970|13952 >NVVL01000070.1 Rickettsiales bacterium isolate NORP64 Contig_source1382A_25018, whole genome shotgun sequence
LNEKQNNLIDNLTNCIKILSVDAIESAKSGHPGMPLGFAQVMTVLATEFLKFQPKHPKWF
NRDRLVLSAGHGSALLYSFLYLAGYQEFSLDDLKNFRQLHSKAAGHPERDMHDAIETTTG
PLGQGMANAVGMAIAAKKYKAELGKEICDHKIYTIVGDGCLMEGISYEAASVAGHLKLDN
LIILFDDNSISIDGKTNITVSEDHLKKFEAMGFNTTRIDGHDTTQIRAALAEAQNSDKPS
FIACKTVIGKGALTKEGSEKSHGAPLGEDEIKHLKSQIPFAKGKFYIPEELKLAWEDTSR
KSDKEYLAWQENFSKLSEDKKSYLPASVINIPDIESPNKPEATRVSSGKIIEALMGQERK
IICGSADLAGSNNLKNQWSRAISAQDFTGNTIHYGVRENAMGAIMNGLALSGFTPIGGTF
FVFSDYMRPSIRLSALMGLPVIYVMTHDSIGVGEDGSTHQPIEHLASFRAMPNINIFRPA
CFTEVKECYEIALKSKSTPSMMGTPSMMVLTRQAVPRIRSGDSGSSVDSGNMSSKGGYII
SEANDASNIDITIFASGSEVGLAIEVQEALELQGKSVRICSVPSMELLWAQGDDYLDDLR
GNAGMIAAIEAASSLGWHRIIGSKGRFFGMDSFGASAPAKDLYKHFGLNADAIVNGLLRS

>gene_1573|GeneMark.hmm|67_aa|-|12|215 >NVVL01000071.1 Rickettsiales bacterium isolate NORP64 Contig_source1382A_25058, whole genome shotgun sequence
MFRVFLIIIALITIILIASGSVPQLSMLAWIKDVFFSAARLIFSIVRGVWPLLILAACGY
LLIKKSR

>gene_1574|GeneMark.hmm|512_aa|-|303|1841 >NVVL01000071.1 Rickettsiales bacterium isolate NORP64 Contig_source1382A_25058, whole genome shotgun sequence
MITKISSLAFNGIDITDVDVQVQIEPGIPKFTIVGLADKTVAESKERVRAALLSIGLSLP
AKNILINLAPADLVKEGSHFDLAIGACILASMGALPAEQVANYLILGELSLDGAILPVNG
VLPVAIGASARGKGVICAKQNGSEAAWSGNDDILAPANLLELINHFKGTQTLEQPKPAVN
SKTVTYPDLKDIKGQKIAKRALEIAAAGGHNLLMFGPPGSGKSMLAQRMPGIMPELSPME
MLECSTISSIAGRIQDGCLTRERPFRSPHHSCTSAAMVGGGVGRKVKPGEISLAHHGVLF
LDELPEFNSAVIEALRQPLETREVLISRSGAHVKYPANFQLIAAMNPCKCGYLNDSYKSC
SRAPMCGSSYRSKISGPILDRFDLHIEVGNSEVDYNYDQILNVSGEESSQDVAARVKAAQ
LTQLERHDGHNIRLNNSLEGQLLIDYAVPADDGRDMLNEAAKKFRLSMRAYNRVLRVART
IADLGGVKSVRKIHIAEALSYRQFDFAEQKVA

>gene_1575|GeneMark.hmm|443_aa|-|1883|3214 >NVVL01000071.1 Rickettsiales bacterium isolate NORP64 Contig_source1382A_25058, whole genome shotgun sequence
MTSKNKMGLTPTEIVNELDRFIVGQKNAKKAVAVALRNRYRRACVAEPMRSEIAPKNILL
IGSTGVGKTEIARRVAKLEDSPFIKVEATKFTEVGYVGRDVDSIIRDLVESAVDFQKTQA
KKDIAEKAKDRAIDRILETVVGKTASEETKNRFKEKVLSGELDSTEIEISVKDTPAAGGN
FFDVPGMPGASMGVLNVTDMLGKVMGGEKTKQKKLSVKDAIKIIIAEESDKMIDEEKTIE
KAIKAVENDGIVFLDEIDKIASSSSETRRGQVSREGVQRDLLPLIEGTNVSTKYGTVKTD
HILFIASGAFHLSKPSDLLPELQGRLPIRVEMTALTKEDLVKILTEPESSLVKQYMALIA

TEGVVLEFSDEAIENIATYAARFNMEIEDIGARRLHTILEQLLEDISFDVTHSKKKKITI
DKKFVDERLSGFVKNRDLAKFIL

>gene_1576|GeneMark.hmm|187_aa|-|3211|3774 >NVVL01000071.1 Rickettsiales bacterium isolate NORP64 Contig_source1382A_25058, whole genome shotgun sequence
MSNSSTNGADLRGTTILCLKKGDDVVIAADGQVSLGNSIMKSTARKLRTLSDNKIVAGFA
GSTADAFTLFERLEAKLDQHSNQLVRSAVELAKDWRTDKYLRRLEAMLIVADKDNILIVT
GTGDVIEPEDNIAAIGSGGLFALSAAKALVSVEKNKMTAEEIAKKSMNIAADICVFSNHN
IIIEKVV

>gene_1577|GeneMark.hmm|122_aa|+|3850|4218 >NVVL01000071.1 Rickettsiales bacterium isolate NORP64 Contig_source1382A_25058, whole genome shotgun sequence
MNIYSIYTNPNKNDDAPVPIKQGFSLVAGLFNLFWALYHKMWSVALFMVLITTFIGAIDS
SHTGFSVNIMIMLTFAIFASDLMENRLTKRGFKLDDIILAANEEEAEIRYFMRKNSNKSE
QL

>gene_1578|GeneMark.hmm|112_aa|+|4277|4615 >NVVL01000071.1 Rickettsiales bacterium isolate NORP64 Contig_source1382A_25058, whole genome shotgun sequence
MYDKNNVFAKIIKNDIAGEKLYEDENLIAIKDINPEADIHLLVIPKGDYVDLADFTTNAS
AQEIEHYFKMIPIIAKENGAEEYRIVSNSGASAGQSVFHFHTHILSGSKLSM

>gene_1579|GeneMark.hmm|360_aa|+|4681|5763 >NVVL01000071.1 Rickettsiales bacterium isolate NORP64 Contig_source1382A_25058, whole genome shotgun sequence
MSKEYIVAIIGATGNVGREVLNSLSERNFPVKEVIAAASANSVGKEVSFGDDQTIKVLQM
DELDFAKIDIAFFCAGSEVSEKYAKKAASKNCIVIDKSSLYRLEKDVPLIVPEANINALK
DFSANAGDASTNGSAGTADASANGSRGGIISNPNCCVIPLAAALKPLDNEAKIKRIIVST
YQSVSGAGKSAMDELYLQTKAKFVFENTTPSVFPTQIAFNLIPQIGEIRDDGSCDEEYKI
EQEMQKIMGNHISLSVTCVRVPVFVSHSMSVNVEFENDMNAAEAEEILSEADGIIVYSRE
NEIQYATPVDVVEHDEVFVSRVRDDKSRKNSINMWICVDNLRKGAALNAVQIAEEIIKTA

>gene_1580|GeneMark.hmm|295_aa|+|5872|6759 >NVVL01000071.1 Rickettsiales bacterium isolate NORP64 Contig_source1382A_25058, whole genome shotgun sequence
MILSDEEIGMVQKNESQFVSFKYLDDKGVLKQIDTSFQNILDGKRFFSDDSINLKTIIGK
AFIDPFRSFPTTSFFCENLTSKDNQRGKAIKLIDEYSEPDFVFLGAEIRFWVFKKINDCD
KFTSGFVDPFANLRSDIIACMEKINIPTTTHNCGEIPTESVVGIEGNSIVDLADNVLLAK
FIIANVTDSYGMSALFSCDPSASNLALLARGNTERMTENARNITRDSAKIKEVTGLKTDN
LKDSSVLTIHKAELNDDLVNIKISMQTKGEFVPYLAFTELLLYNAVGSSLGNTVF

>gene_1581|GeneMark.hmm|585_aa|+|6908|8665 >NVVL01000071.1 Rickettsiales bacterium isolate NORP64 Contig_source1382A_25058, whole genome shotgun sequence
MAKNFNQVDKPLNPLKAALEKCRAAFKVVFVFAFFINLLMLVTPLYSLQVLDRVIGSGNL
STLLMLSIIIGVVYFVHMKLQIARSFTLIKVGEWLDNNVAPTIFGHSISASATKVNPASS
QLLRDFQTIKTFFTSTGINTVFDAPWSIVYITVIFLIHPYIGLITIFGAVLIVSLAFFNA
VATNRTLGEATEFSIKSMGQAEIANRNAEAVEAMGMIANVKKNWAKFNEASLAKQSTASY
RNGVIQNFSRFIRNIMQMAVTGTGAYVVVSTNGQDMTTGGMIASSIIVGRALAPFDNSIV
MWKSISGAMKSYKNINSSFDKHEERDQAMPIPHVDGHLTVENVYYAAPTPPGKPQPPMPK
HILKGVSFALQPGESLAIIGPSAAGKSTLAKLIVGVWHASSGSVRLDGGDVFTWNRENFG

QHVGYLPQGIELFGGTIKQNIARMMDDADPAKVIDTAKMCGAHEIILRFSDGYDTDIGPG
GSNLSGGQKQRIGLARAFFGDPKLVILDEPNANLDEAGEMALSDALKAAKARGIAVVLIS
HRPSVLSEVDKIMVLQDGSVASFGSREEVQGRIKTLKNGMIHIND

>gene_1582|GeneMark.hmm|528_aa|+|8687|10273 >NVVL01000071.1 Rickettsiales bacterium isolate NORP64 Contig_source1382A_25058, whole genome shotgun sequence
MSDNKEKLTPEKLKQIQQLQQMMAMAAKDGKVPGGLPPGGLPPGFPGMKKPSKWSLKGMF
IGTLQGMQGGVKFIDQFVNFITKQDGDKTNDVIKNARSPILFGTFVIFFFVLVGVVWSAT
APLDSAAVAIGTVISKSQLKQINHQEGGVLEKFYVEVGDRVEKGDKLLEFDDTKMHSEYE
IILNKYRIYLAIETRLTAEINDDPEITYPKFLLDDQSVPEVAKIIETQNSMFYSKHEITV
AEKASIEQKVKQTNLQIDAQNARKVALNKTREIVQDRLEASKKLSKKGIIKKTDLLNLEE
KEAGTLGELAIADIEIAKMHQEIIKSNIELINLDNQVSTRAHQELKEVQLSLAEAKERLV
LLKDALSRVIVRSPVDGVVNNLYHHTVRSVIQQGQPIVDISPTADSLVIEVKLEPRLISD
IKVGRLAKIRFSAFKSRTTPLFMGEVVSMSPDIVIDPRQQNNPTLANGYYLARIEIDMDA
FEEIAKTRNLELLPGMQAEVQIITGTRTMLQYLLDPVFDAMFKGFKEK

>gene_1583|GeneMark.hmm|51_aa|-|10476|10631 >NVVL01000071.1 Rickettsiales bacterium isolate NORP64 Contig_source1382A_25058, whole genome shotgun sequence
MAGSFCSANFKLACQLKLFEQKLVEMEGIEPTTFCLQSRRSTPELHPHFVE

>gene_1584|GeneMark.hmm|80_aa|-|10661|10903 >NVVL01000071.1 Rickettsiales bacterium isolate NORP64 Contig_source1382A_25058, whole genome shotgun sequence
MITSCGRICIGKKKINVSTVLAGQNVGIMQVEDNIWLVQFMDFDIGYFGMDSCRIEPTTD
PFGVKLLSMCSVYTAMSVIR

>gene_1585|GeneMark.hmm|61_aa|-|11022|11207 >NVVL01000071.1 Rickettsiales bacterium isolate NORP64 Contig_source1382A_25058, whole genome shotgun sequence
LSKLSVWWLRLGIKIERIKHGNPQQNGRHERMHRTLKQDAAKPAAFNFLQQQEKFDHFVK
V

>gene_1586|GeneMark.hmm|59_aa|-|11204|11383 >NVVL01000071.1 Rickettsiales bacterium isolate NORP64 Contig_source1382A_25058, whole genome shotgun sequence
LTITDNYSRYLLACEGQESTKAFGAFAVFEAVFKEFGLPEKLFVVIMAHHSLRAMLYMA

>gene_1587|GeneMark.hmm|466_aa|-|11729|13129 >NVVL01000071.1 Rickettsiales bacterium isolate NORP64 Contig_source1382A_25058, whole genome shotgun sequence
MSKDPMSDIEEVNARTKRNRRDVENDIDEPQAKRAKTSDEVNAGAKRNRSNAENDIDEPQ
AKRVKITIEEMIKMHVRNNMDAENILDVVGYFAESGYLERFVESLDDHSRSVLYDEITSL
DSSMNQEIIERAMNAGKVADLLDCQYADSIFEGRQDEIFQAITRENKLDKLTSGRHDALH
MFNRFSSSDDIISMYVKPNMSTENIQSVIGNVVESGCLERFVESLDDHSRSVLEANINNS
NIQLEIVERAIDAENVAALLDCRYANSIFRGMEQRVIKAIIKENKLDELLLHGDRSGKEY
FAKLMLEITSDIPEDIIVITDKLQNLDLLSSAKEALLERHRQPRNPRSRNPARPDDPIPQ
KHPLLQHIEYVFENETMRKDAAMHSLDERKNPEIPREVSRLVSEFTHGKDVGAHNFPLPP
EDQQQLQQSARTLRASRASTQTSSSSAPRGGQPKPRNVRGDGDLGR

>gene_1588|GeneMark.hmm|195_aa|-|82|669 >NVVL01000072.1 Rickettsiales bacterium isolate NORP64 Contig_source1382A_25088, whole genome shotgun sequence
MVEINWTFEEFFSAGGNTKFEDRLASVLGVHSSRIKIVGVKTGSSIIHYFITLNEEYAND
PDKQKLELSKINDMLASLYASGEFEKHLGMPILRMESYLSGPDGAGSTGNNSIEKSESNV
KYPVLGLLIFSAALLILGAIVAFMVMRRRSKYDMVQVRSTSDVAKDDGMFTSNKFDIDAT
LADEEKFNVSNAHLK

>gene_1589|GeneMark.hmm|70_aa|-|741|953 >NVVL01000072.1 Rickettsiales bacterium isolate NORP64 Contig_source1382A_25088, whole genome shotgun sequence
MNYKEKMMLSRDYRSEFNSLMLDIMQFTLNLISMENFNLNKSRNAMPSMMVTLNPVILLK
ILVENAHGPR

>gene_1590|GeneMark.hmm|102_aa|-|1315|1623 >NVVL01000072.1 Rickettsiales bacterium isolate NORP64 Contig_source1382A_25088, whole genome shotgun sequence
MFGYNTHMTDYQAITEVTGITIIDSNIANLHVAFDAPIEWVQLSICGDFHCTGPRNYFIS
ISESTIDESCTNDYGFDFSMTGGLVVPPNRIVDDFVSVDDCT

>gene_1591|GeneMark.hmm|60_aa|-|2308|2490 >NVVL01000072.1 Rickettsiales bacterium isolate NORP64 Contig_source1382A_25088, whole genome shotgun sequence
LEETEGAPLRQLIQYCSFNYGRGWAINIDEAFNVDILNNVVFHHVAIGFQVTISTNVLIS

>gene_1592|GeneMark.hmm|61_aa|-|2728|2913 >NVVL01000072.1 Rickettsiales bacterium isolate NORP64 Contig_source1382A_25088, whole genome shotgun sequence
MIFTEHNDRLYHVDRVLAKTDPQTISAGDLVKDIQGRYYTILHNAEEGYFYIEIKGDGWG
A

>gene_1593|GeneMark.hmm|189_aa|-|2956|3525 >NVVL01000072.1 Rickettsiales bacterium isolate NORP64 Contig_source1382A_25088, whole genome shotgun sequence
MTRLLESAEIGSTTITVDESEVSNWVIGDRIALAPSGFNAREGESFTILDISVDGNLATI
TVDTEISFYHFGAETGAGESDCSTDIRTEVIHLSRCVRIIGVEDQTLKVILKSPINDEDS
SSTKSASSTDGSSKYDSAKSSSSCSTKKSSKSSSKSKSKSSKSDSKSKSKSDDDEEERRV
RRLSLTGGR

>gene_1594|GeneMark.hmm|60_aa|-|3522|3704 >NVVL01000072.1 Rickettsiales bacterium isolate NORP64 Contig_source1382A_25088, whole genome shotgun sequence
MKILQLKMFKIIHSRENLISYCLVISKMLILLQEVLLKQAIKLLLTLEASQLILTITTNQ

>gene_1595|GeneMark.hmm|178_aa|-|3706|4242 >NVVL01000072.1 Rickettsiales bacterium isolate NORP64 Contig_source1382A_25088, whole genome shotgun sequence
LHAKVIICRDISKCIPGLIAAEKETFLRYWSNSQHWFKAEEPEPERRRLIREENDDEEDR
DGFVRYELPVNGDNVEILEGRNVIYDLFYSPTTNNTPALRTVIVNGNLIIPMKKTKHNGS
EEGGIIYIYQDEIDYDKPKTAYDKLASNNAEAEDFIGRIQAKHIFTRKGHVYVGLFPE

>gene_1596|GeneMark.hmm|68_aa|-|4312|4518 >NVVL01000072.1 Rickettsiales bacterium isolate NORP64 Contig_source1382A_25088, whole genome shotgun sequence
MTHFLHNTTAYREDIDLTAIGSMCTPLEFKLTLDSTIDNMLDGASHGDNYYDYAAEMWHF
VISGYYQN

>gene_1597|GeneMark.hmm|139_aa|-|4870|5289 >NVVL01000072.1 Rickettsiales bacterium isolate NORP64 Contig_source1382A_25088, whole genome shotgun sequence
VTPSLPEDGAEAAEEILFNMIFGNGTGVHNSREVRKLTFTKYNPYYTFRGAHLNIIRVTD
FLGEFDEEIESNLPCVEEEPEEGVRLLKKRDRKHGKKYYEQFKNHGKGSTSSSDDNDNES
GSKSKSKSKSHTHSHSHSH

>gene_1598|GeneMark.hmm|66_aa|-|5316|5516 >NVVL01000072.1 Rickettsiales bacterium isolate NORP64 Contig_source1382A_25088, whole genome shotgun sequence
MDYPSLMLLKEFILVHLEDKFSKIPITRSLIYQNSLSPKISLISYGLFIILGTLISQFTH
MEDLNI

>gene_1599|GeneMark.hmm|105_aa|-|5517|5834 >NVVL01000072.1 Rickettsiales bacterium isolate NORP64 Contig_source1382A_25088, whole genome shotgun sequence
MDILLTLSLLEELQHGWQEILKAMKSTVDILMSAKEKNLYGVSSLQELKTSKSTMFTSQT
STSANAITELSLMAQLIALIELLLSEPVHIVKHGIQHLKEGIKLL

>gene_1600|GeneMark.hmm|63_aa|-|5919|6110 >NVVL01000072.1 Rickettsiales bacterium isolate NORP64 Contig_source1382A_25088, whole genome shotgun sequence
MKLLELSVETEHIQMKCMDLEFTARCITLLMHSTSRDHKLSSLKTSLAIRMSDAELFTLM
LDI

>gene_1601|GeneMark.hmm|146_aa|-|6112|6552 >NVVL01000072.1 Rickettsiales bacterium isolate NORP64 Contig_source1382A_25088, whole genome shotgun sequence
MNTEFQYVGQAYQIGRYPLHFNKIGNARESIVSGCSIHNSYNRIVGIQGTNNLLIKDNVS
FRTKGHGYYFANGDETNNTFNNNLALIVERSWSLLNTDKIPSTFWIRHPMNHFIGNSAGG
SDGNGFWYDLESQPRGSTFGTSSARP

>gene_1602|GeneMark.hmm|151_aa|-|6637|7092 >NVVL01000072.1 Rickettsiales bacterium isolate NORP64 Contig_source1382A_25088, whole genome shotgun sequence
MTSRVDITLSGTKYDPAVPVFGKKVLAARNGLISFVGQDVVDHTFLNASSVPWESHISVA
GDAGWHVGDHIVVGTTSFSNYESEERTIVEISYDETSETTSITLDTALWYHHFGDLFSAG
KTGEEKDFEMRAEVALLTRTIRFRGNPEDSQ

>gene_1603|GeneMark.hmm|150_aa|-|7593|8045 >NVVL01000072.1 Rickettsiales bacterium isolate NORP64 Contig_source1382A_25088, whole genome shotgun sequence
MVMMVLSIFIDQKIVKFLSHSQMVIEFHMSLDLIIGFQMLMIVKQITLTEMMDLSFMPQK
MELKKKMLFGKQELRYNLDIMMLEFLLKDLILLFSEVKVTPVLLLRSNSKDILFMNLMNV
LLKLNTLIKEVEHFMIQEQLLHLIKPRHCQ

>gene_1604|GeneMark.hmm|65_aa|+|8484|8681 >NVVL01000072.1 Rickettsiales bacterium isolate NORP64 Contig_source1382A_25088, whole genome shotgun sequence
LLKAHNEQQVQYHQFQDQIQLMLQYLKQLRMFQLDYHLILHKQLMIIQHFLSKSTDLLSQ
LLISK

>gene_1605|GeneMark.hmm|140_aa|-|8890|9312 >NVVL01000072.1 Rickettsiales bacterium isolate NORP64 Contig_source1382A_25088, whole genome shotgun sequence

MTYNIQDNLDGSMTLSVYIAYIEPRHCPTGGHTGKIRVFLKATDEASCDYDLENGGCTKI
EWFEPDTDVWNITGLSFSDGNVVATGSGFETNPEFSLFVIDGQAMPVQTATATKLTARIT
NLKTNSHTEIFIFPQGGVPK

>gene_1606|GeneMark.hmm|62_aa|-|9433|9621 >NVVL01000072.1 Rickettsiales bacterium isolate NORP64 Contig_source1382A_25088, whole genome shotgun sequence
MNACRFIPGSNGKDAGEYGLLFPGGPSGVYNMRLYHPIARAFKYSKKFVVEALITDIVGP
DG

>gene_1607|GeneMark.hmm|151_aa|-|9736|10191 >NVVL01000072.1 Rickettsiales bacterium isolate NORP64 Contig_source1382A_25088, whole genome shotgun sequence
MRLTGIGLPETMEEATAFNMIVIITDSAKRERDVDSNNLIACEISDIIESGIIECSFIPS
EGCIDPESPGCSILEDESGTYIAVLFYGYDFLESFGDNLDNFVNSDIELVGTSKKAGPNI
RIFAFGGTFGSLETTSVSPVLKTEMIVQTTK

>gene_1608|GeneMark.hmm|90_aa|-|10234|10506 >NVVL01000072.1 Rickettsiales bacterium isolate NORP64 Contig_source1382A_25088, whole genome shotgun sequence
VTDQDWDVDDRILTIEGTFGTLEHTLRFAHRDGVILSWTDTEITAFVNNPLAGAWGVEVI
YPEGNAIIDTALLNCEIELFFHGNPNSKII

>gene_1609|GeneMark.hmm|91_aa|-|10546|10821 >NVVL01000072.1 Rickettsiales bacterium isolate NORP64 Contig_source1382A_25088, whole genome shotgun sequence
LIESSHDILSITFVSSLTNPLEGQSDTQWNAYMELEAVQSVTSFGHYDDSYFFRYVAIEN
LKAIHDGGSDHLLVKITHMGVVVDAYCTNIA

>gene_1610|GeneMark.hmm|162_aa|-|10984|11472 >NVVL01000072.1 Rickettsiales bacterium isolate NORP64 Contig_source1382A_25088, whole genome shotgun sequence
LIDIDPVTDNDEPFHFEFLLSRHGSWTESESYTNYPSAGTLRNEIYNFGTLEHWDGEFRV
YKNALTDGGFVYEFVLLRSVDISRTGDAEQLVIHGVRIVTDVEEGIVVLKNEEALVGRDL
PAEFDIPEGEEDELYGLYTISNRPVQGQFLTRITFLDSFGDE

>gene_1611|GeneMark.hmm|166_aa|-|11539|12039 >NVVL01000072.1 Rickettsiales bacterium isolate NORP64 Contig_source1382A_25088, whole genome shotgun sequence
LTDSPAITDSPPTGVTRKIWYGNDIDTWAEVDYENPQVEEYYGGAETIHYRNYYCEELDG
IFVNPLAGEYRFYTSCDGSVDFFFNKGSTAFDELAIVADNSNKNSGFRSYHHTGDGEGTP
FTFAASGEQSRFTFRHCHSTGDDYCSIGMEYQVLATEDALPTNPAL

>gene_1612|GeneMark.hmm|154_aa|-|12466|12930 >NVVL01000072.1 Rickettsiales bacterium isolate NORP64 Contig_source1382A_25088, whole genome shotgun sequence
MGGADVEFTGQMFNPQTENNFVKIKSRESDDRARTFAQTYTLSGKDIFIYYKDEDISHSN
AGLGKLVWRSPPLADLLGMTEPHHLNTSFSLWLEYYRNHATTDITCNGGQGGCSYTSSWK
TTPFNFGVIPHTFGYKQRVSAWLQGRHSASYKSD

>gene_1613|GeneMark.hmm|42_aa|-|13005|13133 >NVVL01000072.1 Rickettsiales bacterium isolate NORP64 Contig_source1382A_25088, whole genome shotgun sequence
MQRKKYQALWVSALIICVLLMAATGKIGSLIFQRDELLLLTE

>gene_1614|GeneMark.hmm|211_aa|+|46|681 >NVVL01000073.1 Rickettsiales bacterium isolate NORP64 Contig_source1382A_25202, whole genome shotgun sequence
MTYCDHSNQEEYPFKLPELPFAKDAFAPHFTPETFEYHHEKHHNAYVTNLNKLLDANKEL
TDLDLEGLIVRTNASNAAIFNNAAQIWNHSFFWYSIKPGGGGKPEGEMLEAIEKDFGSYE
NFAAEFKAAAIGQFGSGWVWLVYHNEKLKIMKTSNAETPITQGAFPLIACDVWEHAYYID
YRNKRPDYVSSFLDHMINWEFGLMHLKRATQ

>gene_1615|GeneMark.hmm|210_aa|+|933|1565 >NVVL01000073.1 Rickettsiales bacterium isolate NORP64 Contig_source1382A_25202, whole genome shotgun sequence
MSIKLQSVIAITLLFLIYVVINLIYWKNPGINSNNIECLQECVYKSPNRKTITLTSMNNL
LEIKFIANSARTCFNPSFPIIHIKTKSDHNAWIHIVRTDAAAEELRFFIDTDKKFTPFYN
FNEDFYDAPFWYYGIFNKPLSFWEGHAYAVKVDHDSKTITCMGGIKWGFKLQYFSLKPKM
IDPISLSHEDWKKDWLFFSKSLTGYTLKVN

>gene_1616|GeneMark.hmm|113_aa|+|1859|2200 >NVVL01000073.1 Rickettsiales bacterium isolate NORP64 Contig_source1382A_25202, whole genome shotgun sequence
MFEFIEFASAIRALYQYVNDELVEEDFWLITEEQRKRLPKEDQTGVWYMLNPDKQKKDQN
SVFLVDKAEKDRLIRAVAFIKSSAKKLPESASFLEKLLYCKKTLPPVLFKLES

>gene_1617|GeneMark.hmm|96_aa|-|2267|2557 >NVVL01000073.1 Rickettsiales bacterium isolate NORP64 Contig_source1382A_25202, whole genome shotgun sequence
MAKKQAEKENVELTLEALYDVPVQVSVVLGSTTMPLSNILKLSRGAVIELERTVGEPIDV
YVNNKKVAKGEIVIVDEKIGVTLTEVVVNEKDFQSA

>gene_1618|GeneMark.hmm|306_aa|-|2573|3493 >NVVL01000073.1 Rickettsiales bacterium isolate NORP64 Contig_source1382A_25202, whole genome shotgun sequence
VSKKHKKFEFEDLPKANVVNSNTHAKKTEADELINDAVTGHLNKIVDDKKEDSDPDDDDS
SGGSNLAEKTNPAGENAAQTPLPATGIQAPSPQLDGAASAPGTGAGAPSGASEGVASGTG
AGGSAAQPAAEGAPQEPSTAQAEPVQGAAEQTAPEGAGTAAPQVGVDFAKLLEEKLSAIL
PEVDIGAQIAKASAESISSIVKKLHLILPANFEEIIVNGLLEKLKKFYKEGSITLTIHPD
RYDFCTKALQAEGIKDRFKDSFTIIKDDTLGTDDCKLEWGETKLEYNQEQLASEIDRIIE
QLKSAT

>gene_1619|GeneMark.hmm|339_aa|-|3509|4528 >NVVL01000073.1 Rickettsiales bacterium isolate NORP64 Contig_source1382A_25202, whole genome shotgun sequence
MGIEVKDIAKLTATQKVAIVLLSLSDDNATRIFSLMSEEEITDISHAMSNLGSVAPEAVD
SVMDKLSTDMAGDSTFFGNLDNTERLLQKILDKDRVDALIDEIRGPQGKNTWEKLANVNE
ELLALYFRNEHPQTAALVLSKIAPDHAAKVLSNLPDSFAFEIISRILSMGSVKKEVLERV
EKILRAEFISSVGKTQKYDSIERLVGIFGNLDRSTEVKFMTMLESNLPEAATKIKDMMFT
FDDLLKIDSKGIQRLLRDVEKSRLTLALKGASDELKKMVFSSMSQRAAKIIEEDIMSLGS
VRMKDVDAARFDVVSVAKGLIENGEIDVSLDNEEEEFVT

>gene_1620|GeneMark.hmm|544_aa|-|4528|6162 >NVVL01000073.1 Rickettsiales bacterium isolate NORP64 Contig_source1382A_25202, whole genome shotgun sequence
MQVLIQFLKEMSPAKLAAIAVTILASIIMFILFLSKVGEDEFAVLYTDLDLQDSAKIAED
LDTRKIIYKAMFDGSTIKVHKSEVASTRLALAQSGLPNAGSIVGYEIFDKEDSIGATNFS

QNVKLIRALEGELSRTITSFDQVSRARVHLVMPQREIFSKERLEPKASVVLKFRGGRRLS
KSGIDAISHLVVTSVPGLEMKNITIVDTKGKPLKIGSSEENMDFASSKNEEMRIGSENRL
RGMIEELLSHSFGSGKVKAQVSLEMNFDRVVTNSEIYDPEGAVIRSVQSVDEREKTPVSS
GGGGDVSVANNIPGGDSGGGGAGGNFATTEKSDQTTNYEISKTIKNHISESGVVTKMSIG
ILVDGNYTTDPETQEVSYSDRSKEDLDKIVSLVKVATGFDDDRGDKIEVINIRFSSNLEP
FDDDEVDWVMEHLPNLFQVLIFAILVFLVLITVIKPVALKAFEVKNNQADMDSDIIDAAG
GIPGEGGVSGGVVAVSADGTPVEGAASETPVPVKISPVTKINELADSNPEDLVSVLRKWL
NEDH

>gene_1621|GeneMark.hmm|118_aa|-|6243|6599 >NVVL01000073.1 Rickettsiales bacterium isolate NORP64 Contig_source1382A_25202, whole genome shotgun sequence
MSDEIKEYLEPGKKNLILIYMLYLCGVLVPLLPIVGGVFAYANQNNEDEIWKSHYIFAFR
SAVLGVLGMLISMITAFIFIGVIFYMVVVIWFVVRSILALQYLFEGKAHPNPMSYWIK

>gene_1622|GeneMark.hmm|888_aa|-|6609|9275 >NVVL01000073.1 Rickettsiales bacterium isolate NORP64 Contig_source1382A_25202, whole genome shotgun sequence
MSINSESKYAKELELDGQSYHVFDIRQAARDSDFDLGSLPYSLRVLFENVVRNGAREKEM
GAFKTWLKNKSSQDEISFSPARVLMQDFTGVPAIVDLASMRGAMQTLGDSPSKINPVIPV
DLVIDHSVQVDSYGNKGSFNKNVEKELERNGERYKFLKWGQSSFSNFKVIPPGTGICHQV
NLEYLAQVVFIDEKDGKKTAYPDTLVGTDSHTTMVNALCVLGWGVGGIEAEAAMLGQSLP
MILPEVVGFKMVGSMGSGITATDLVLTVTQMLRAKGVVGKFVEFFGEGVKNLSLADRATI
SNMAPEYGATCGFFPVDEETLKYLRLTARDEHKVKLVEEYTKIQGLWLDDNTPEYSDVLE
LDISTLEPSLAGPKRPQDRVALSDMQNNFKEELPGFAGTGADISAKHKVSTGEFAIGHGD
VVIAAITSCTNTSNPSVMIAAGLVAQKACELGINVKPWVKTSLAPGSKIVTEYLNKSNLG
QYLDKLGFNLVGYGCTTCIGNSGPLDNIIDEVIREKKLVVASVLSGNRNFEGRVHPHVMA
NYLASPPLVVAYALAGTLNIDLDKDPIGQSESGVDVYLKDIWPSREEIQQYVEDCVDSQM
FAEKYKDVFKGDEHWSAIETSEGETYDWQESSTYIQNPPYFDNIDKPPLPLENVNNARIL
AMFGDSITTDHISPAGVISHGGAAAKFLISEGVSVGDFNSYGSRRGNHKVMMRGTFANVR
IKNQLCEGVEGGVTINHLSGKQETIYDAAMDYRANAVPLVIFAGKEYGMGSSRDWAAKGT
NLLGVKAVIAESFERIHRSNLVSMGVLPIAFNSGTSWKSLNLKGDEEITIMGMSDDITPY
QPLTCVIKRKNSDNNETINVTLQIYTANEVGYIKHGSILGAVIKTLTN

>gene_1623|GeneMark.hmm|199_aa|+|9450|10049 >NVVL01000073.1 Rickettsiales bacterium isolate NORP64 Contig_source1382A_25202, whole genome shotgun sequence
LGNREVFKDLSLSFLPSSIIYIKGANGSGKTSLMRMLAGIQTPSSGSITFDREALPISML
EKPYCTYIGHKVGIKPELTALEHLEFWAKLYDSAELIDAAIIYFKLEEIIDKKCYELSAG
MQKRVALARLIASPAKLWLLDEASSNLDSDNRQLLLNLIITHANNGGITFIACHNEPEIK
TAQVLDLAEFLPNLEENKS

>gene_1624|GeneMark.hmm|212_aa|+|10046|10684 >NVVL01000073.1 Rickettsiales bacterium isolate NORP64 Contig_source1382A_25202, whole genome shotgun sequence
MKSLILQEFRVQDKIYNLIKYSLIFFIFCSLSITLINSYENVQIFGVVFSVICIPLAFIN
LSAGLIKPDIEDGSLEILLTAKTPIKIVMAKYIALFTCFCLSFAMLLPLIYIIYNIPNTI
FILIAASGFLLISLCAALAVLIASIQGYFRANTNFLSIIIMPLVIPSIILSGIIIQNPDN
LHLLAIMIGINLIVIPPSIYLSGYLVENIYNI

>gene_1625|GeneMark.hmm|110_aa|+|10862|11194 >NVVL01000073.1 Rickettsiales bacterium isolate NORP64 Contig_source1382A_25202, whole genome shotgun sequence
MVLFVYGLMGKWLIAIMITALTVTYFIFKGLDEAGVIKGAEDVVFKVFNDTKSIAQHCTP
LITNLNDFWYCIEHPPTYTPSEEEAALRSGALNLIAPEENSNDIKDPYAE

>gene_1626|GeneMark.hmm|178_aa|+|11293|11829 >NVVL01000073.1 Rickettsiales bacterium isolate NORP64 Contig_source1382A_25202, whole genome shotgun sequence
MTDETSSDKPDKTTRRDFVTLTASGMTAIGAACAVWPLVDSFNPSADVLALSSIEVNLAP
VEEGQTLVVKWQGKPIFIKHRTEAEIEEAEMTPMKELIDPEADSDRVKPGQSKWLVTIGI
CTHLGCVPIANKGEYDGWFCPCHGSHYDTSGRIRKGPAPLNLAVPPYEFISDTKIKIG

>gene_1627|GeneMark.hmm|406_aa|+|11831|13051 >NVVL01000073.1 Rickettsiales bacterium isolate NORP64 Contig_source1382A_25202, whole genome shotgun sequence
MSTPESNDKQQSGIIAWIDYRLPIFSAMKHLGEYKTPKNLSYWWSLGSIAGIALVIQILT
GIVLAMHYTPHVDHAFDSVEKIMRNVNYGWLIRYMHAVGASMFFVAVYLHMFRGMYYGSY
KAPRELLWHFGLIIFLAMMATAFMGYVLPWGQMSYWGATVITNLFSAIPVVGDSIVTWLW
GGFSVDNPTLNRFFSLHYLLPFVIVGLVLLHIWALHVHGSNNPTGVKLKKKDMIPFHPYY
TIKDFVGFGVYFLIFAYFIFYKPNLLGHPDNYIPANPLVTPAHIVPEWYFLPFYAILRAV
PSKLGGVLLMFGSIIILFFLPWLDRSKVRSATFRPIYKWAFWVFVVDCLVLGYVGGMPAE
EPYLTISRLGAAYYFFHFLIVLPSIARFEKPLPLPHSVEEEDENPH

>gene_1628|GeneMark.hmm|318_aa|+|2|958 >NVVL01000074.1 Rickettsiales bacterium isolate NORP64 Contig_source1382A_25374, whole genome shotgun sequence
KHPRINTVTLCEYSEYISFFFKGIFRDYYSLITKIHVTSFFIGYYLNFYMKAIKLKSFVL
IIFLTTSLFALDSAFASKRRTLVIAGDYWCPYNCHPDSKMPGYLVELATRALHIYKIDIE
YRMMPWTKALKEVKKGNIDAVIGLTDFEGNGLQTTRVALEYTQSSAFTRTDSKWVYDGVR
SLTRKKLGIIGYAQSEEINNYIGMNYITKPDLFVMEGGKNAVIDSIANLIDGDIDVCIG
DRRVFNHYIKENGLEPYIREAGKIDKIKHPLYIAFSPKTLHIDQYIKCLEEGLAALKATG
EDDYLRQKYKISRKEHDL

>gene_1629|GeneMark.hmm|181_aa|+|948|1493 >NVVL01000074.1 Rickettsiales bacterium isolate NORP64 Contig_source1382A_25374, whole genome shotgun sequence
MTYKLLPYRQSTPRVDSTTFLADGCAIIGNVTIKSNSSIWFNTVIRGDVDRISIGSNTNI
QDLVVIHTSRKDGGYVDIGDNVTVGHSALLHACSIEDNAFIGMQSIIMDESIISEHAFIA
AGSLVPAGSIVRSKELWMGRPAKYVRMVTDAELKFMEENARHYVELSKEHQLARDEPLNE
I

>gene_1630|GeneMark.hmm|461_aa|+|1536|2921 >NVVL01000074.1 Rickettsiales bacterium isolate NORP64 Contig_source1382A_25374, whole genome shotgun sequence
LGGIQADINTIHLEKVFNLDMNFGLNIYLKREIGKMLDLLIGLSAPAELSGTLIEKVTSG
EDIRLINLDIVRKNYHELKKAFPYAVSYTAVKANSAEGILELLNEEGASFEIATIEELRK
SIKRGISPERIIFSHPAKDAIEIAESFKIGVNRFASDSQEDIALIAKHAPGSKVMVRIKT
SHEDKNHNSLTGFNERFGVTPAAATNLLRSLKSLGLEPYGVSFHVGTQEEDVNAWDWAIM
RAASIFKELKNEGTELEVLDIGGGFPSRYKSKIPALSEYGEAIHRSINKHFSGALPQKII
IEPGRAISAMAGITFGRVINVKPCEHDDSRYIVTLSTGKFSAGLFNVGNGMFFYRKTSSG
KMKNLSGASSKKADIHGKGGASFDRPIEGDDICMPGDLKSGDIVIFTGTGAYSGEMTTNW

CAKSPPRTITLSNNAEMEPLLHNTLSAESPTDETAPSPALA

>gene_1631|GeneMark.hmm|308_aa|-|2985|3911 >NVVL01000074.1 Rickettsiales bacterium isolate NORP64 Contig_source1382A_25374, whole genome shotgun sequence
MSAGRMDENSGILSDAINQPDAILLASLFDAIGDGDVEEVKSLLDNGANANGVGRGGSTP
LLKATGNRYLNREVSTIQEIVKLLLDHGADPTIADEEGRTPLHEVSVRSARNFQIRKEED
SQKVSDNMQAIQDMATSFIKLGASVNSPDKRSDTPLLEAVSQGSVGLVKMFLENGALVNQ
AGHNGETPLSIASEERHIEVVKLLKKAIAYIQVKALHELIPGVTVELLKLTVLTTTPWYI
KPNDIDISGDLDNYGTTPPQETEFRGSSETCSLITDLEEQAGFADNPAATMDTPPLGGLD
DGTTDAVS

>gene_1632|GeneMark.hmm|62_aa|+|4472|4660 >NVVL01000074.1 Rickettsiales bacterium isolate NORP64 Contig_source1382A_25374, whole genome shotgun sequence
MLYVFIIIFAILTTVTLLLGVLSMAAGEKFSKKYGTKLMSLRVFLQSLLVLSLAIAYLSQ
PL

>gene_1633|GeneMark.hmm|76_aa|-|4775|5005 >NVVL01000074.1 Rickettsiales bacterium isolate NORP64 Contig_source1382A_25374, whole genome shotgun sequence
LLSSYFIHFYVFFIANIPAGLSGLIFNNLLKSCENPLTYTQMLGLGVVVKFYFNNYGLVF
LPIHKRYAGFRGALIF

>gene_1634|GeneMark.hmm|392_aa|+|5209|6387 >NVVL01000074.1 Rickettsiales bacterium isolate NORP64 Contig_source1382A_25374, whole genome shotgun sequence
MFRQMPVILALIIVIIISFGDYIPQALASLLFGISLSIKSLIIFLLPVIIFSLLYKATIS
LAKDASKIITIIFLGVIGSNFLTTTLSQMIGMWVYGFDISLIMPSKESTLPASDLFLLPS
IIPNYLAMIGGIIAGVASTQMAPNLAINIGAKLERITGWLLQIVTLLIPIFVTGFVVKLQ
YDGIVMQIIKDYTVIFVIIASAQFGYIAFLYFALSGFRVKPAVSSITNMLPAAFSGFSTM
SSAASMPLTIAGTEKNAKDKDIAHLVVPASVNIHLIGDCVAIPCFAFAILKNYDMVQPDL
FAYFIFTCYFVLAKFSVAAIPGGGIIVMLPILEQYLGFNAEMLSLITALYILFDPVITSA
NVIGNGAFAKMIDLLLYKKAKYTKKLIHEELG

>gene_1635|GeneMark.hmm|149_aa|+|6384|6833 >NVVL01000074.1 Rickettsiales bacterium isolate NORP64 Contig_source1382A_25374, whole genome shotgun sequence
MTFKLDKTLEKDSKFVMDLELSQVRLINNSDFPWVILVPRREDIIEITDLEEDDYTKLME
EIRIVAGGVQEEFTPDKLNIATLGNVVSQMHFHIIARYQNDELYPKPVWGCKFTNYGDMQ
MQKRIRSLKNTIATRALREDGEIATNSEK

>gene_1636|GeneMark.hmm|393_aa|+|6871|8052 >NVVL01000074.1 Rickettsiales bacterium isolate NORP64 Contig_source1382A_25374, whole genome shotgun sequence
MIKNLPFLLVISLLTGCLEVDISVPSFPDISDYFDISDALTQMTIAINFLGFCISSIVYG
PLSDSFGRRRVMMVGNFIMLIGAVGCVVATSIEFLLFARFIQGIGASTSAVVVFAMIADT
YSADKSAKIIGTMNSLITVFMSLAPIVGGLINESIGWRGNFFTIAMLSIVSWIMLYFWLP
ETKKHFTPFSRKKILQDYKTLFFDRSFTYASLVPTLTFAGYISFIACASFLYMEAYDLPI
MHYALHQGAIIAVFSVVSLYSGDVSKFLGERNCVIYGMFLLTAGSSFMLMVALLSNKSPY
LTTLSMMIYGVGSAISYPVVFARSLEIFPDISGSASSVIMAIRSLMCAAFIALSSYLYAG
KLVSVALVILLAATIAAFLSSRLLKLISFDTNT

>gene_1637|GeneMark.hmm|185_aa|+|8065|8622 >NVVL01000074.1 Rickettsiales bacterium isolate NORP64 Contig_source1382A_25374, whole genome shotgun sequence
LRFEINYIFVSFVKKQIVYPDFIPRVFAMTIDIAILLYVWVFITPVLNIATHQVFVYIFQ
DFFLLYSIDMSDAKAVAATIYHPAFSEYVSAPKFLAYFGYLLFVNTVFLGSYFITFWYKF
GATPGKMAMKMKIVDANDYSKPPLCNLVKRFFGYITALIGMWSILFSRRGIALHDKIANT
VVIKS

>gene_1638|GeneMark.hmm|310_aa|+|8767|9699 >NVVL01000074.1 Rickettsiales bacterium isolate NORP64 Contig_source1382A_25374, whole genome shotgun sequence
MHNVFYSVLPIFLIALLGSVIRRKYLTSDEFWRGLEKLSFYILFPTVLFEYSLKVDLTSS
EFFKLTVALIAANLTITIMLVIYQMKTDYDGVQFTSVFQGATRYNNYIFFAIGAALFGES
GLSIIATISPYLLILTSVTSIMSFTHYVPNSAGKKTLRNNLVLMMKSIIASPIIIASVLG
MSFNYFQIELNIGILNTIHSLASSALAIGILIVGAGIKLKLKPEHLKQVVLASSVKLILM
PVVTFIALVALDITGPAKSIAILFSCLPCSSSSYILSRQLGGDPETMSSIITFTTIFSIL
SLSLLVYILG

>gene_1639|GeneMark.hmm|773_aa|+|9748|12069 >NVVL01000074.1 Rickettsiales bacterium isolate NORP64 Contig_source1382A_25374, whole genome shotgun sequence
MSDTKSADANTKSAEYIEALEYHQNGQPGKIALKPTKRLQSQKDLALAYSPGVAAPCLEI
QKNPDDIYKYTAKGNTVAVISNGTAVLGLGAIGAAASKPVMEGKAVLFKKFADIDCFDIE
VDCTDPEEFINIVKHLGHTWGGINLEDIKAPECFIIEAKLKELMDIPVFHDDQHGTAIIT
AAGLINAAFITNREMSEIKIVVNGAGAAAMSCINLAISLGIDKNNVILCDTRGVIYKGRT
EGMNKWKEEKAADTKHRTLEDAMKGADVFLGLSAKGAVTKEMVASMAPSPIIFAMANPDP
EITPEDIKLVRDDAIIATGRSDYDNQINNVMGFPYIFRGALDVRATCINEEMKIAAAKAI
AALARKPVPGEVYKAYGSGRKSFGADYIIPVPFDPRLITTIPVAVAEAAIATGVAKLKNI
DIKKYKAELASRLNPTSTYMHFVYEKIQHAELQRVVFAEGEEEEIIKSAMMMRDENYGTP
IIVGRSSKIDPIVESMGEEYNLDNIMVMNAAINKNLDKYIDYLYSKLQRKGYLYRDCARM
VKNDRNVFAACMIACGDADAMVTGITKSYYNSFDDICKVIKPKEGKRIMGYSIMLSSKHN
IIIADNTACELPDAQDLVEITLQTAKAAKSMGFKPRVALLSFSNFGNPHKEKTARVRDAV
SLLDKMDVDFEYDGEMTVDVALNSNLQKIYPFCRLTAPANVLIMPGLHSASISTNLLEEF
GGGIFIGPILNGFEYPVQIIPMGSSANDILRIAAFSAIEAINSKNEKTEEKQV

>gene_1640|GeneMark.hmm|64_aa|+|12041|12235 >NVVL01000074.1 Rickettsiales bacterium isolate NORP64 Contig_source1382A_25374, whole genome shotgun sequence
MRKPKRNRFERGEDNAGFEEAVNAMIWLIEHYKTGEGNESFAIYDVYGCAPIIEGSFNSR
LYRD

>gene_1641|GeneMark.hmm|109_aa|-|1|327 >NVVL01000075.1 Rickettsiales bacterium isolate NORP64 Contig_source1382A_25997, whole genome shotgun sequence
MGTIMHFNLDITVITVFLAIITVVGVVAGFGVRTIKDYALGGRNFSTPTLTATITATWIS
GIFFALHTSGIYKDGVWYLVAGVGDVLYLFIIALIISPKMAEFFDSCSV

>gene_1642|GeneMark.hmm|53_aa|+|869|1030 >NVVL01000075.1 Rickettsiales bacterium isolate NORP64 Contig_source1382A_25997, whole genome shotgun sequence
MAKNNIRLVAVLYSDKHRAYIIYFYQFWLKKQFTDLIIVHLSFQRIKLEKIKN

>gene_1643|GeneMark.hmm|137_aa|-|1299|1712 >NVVL01000075.1 Rickettsiales bacterium isolate NORP64 Contig_source1382A_25997, whole genome shotgun sequence
MYLLDSCAISDFMKGDKNTSEKLKSILPSEIYTSTITQMEIQYGLIRRFDHSHRYFGILE
DFLSAITVLPFDRSAASHSAKIRKTLDSQGTPIGAYDVLIAGIAMSANLTLVTSNEREFL
RVDGLEIENWRTKKCRT

>gene_1644|GeneMark.hmm|77_aa|-|1745|1978 >NVVL01000075.1 Rickettsiales bacterium isolate NORP64 Contig_source1382A_25997, whole genome shotgun sequence
MNINIYIEEPVGRQLSEYSKKFKRKRNSIIREAIKNWLTNHSTKQWPESILRWDGIEDFP
SIKELRSGLIEPNKKLF

>gene_1645|GeneMark.hmm|175_aa|-|2049|2576 >NVVL01000075.1 Rickettsiales bacterium isolate NORP64 Contig_source1382A_25997, whole genome shotgun sequence
MKKLASFITLSIIIILLSGCYAVETSHEYNHRFTWAKSTSKPIYLRFKDTKSKPFYFLLS
RNTKENGYKLIVRWKNAKSGDLLFNGNKTTLKFFIDKEIILTFKPIKRPKIVSYNMNSGT
HEEEGIFSLSEKEFTRIAYATSVMTELTGRYNTVMGEFSSRNTKKAFRNFAEKSH

>gene_1646|GeneMark.hmm|80_aa|+|2892|3134 >NVVL01000075.1 Rickettsiales bacterium isolate NORP64 Contig_source1382A_25997, whole genome shotgun sequence
MFYVYLIRSINFPDQVYVGITEDFKKRLKTHNSGGSVHTAKYRPWDLVVYMGFKNEIKAR
DFEKYLKSGSGRAFSKKRLW

>gene_1647|GeneMark.hmm|403_aa|-|3297|4508 >NVVL01000075.1 Rickettsiales bacterium isolate NORP64 Contig_source1382A_25997, whole genome shotgun sequence
MLILPIMNIIVQKFGGTSVADTARLEKVANIIKAELDSGNAVIAVVSAMVGVTNSLISKC
NALSPLNSAAHMREYDAALASGEVVTSALLALQLQKIGIKAQSLQGWQIPLKTDGTHGNA
QITEASPALLKDMLAEGITPVITGFQGIAPNGAITTLGKGGSDTSAAIIAASLEASRCDI
YTDVEGIYTADPRVVHDAKKIGVINIDELYALCINGAKILHPRAALAAKRYGFPIRILSS
FTDNTGTVTQIEKLDMENKIVKAITSNKNLLKVNVEYQPRQLSGILQKLSEESIEIEQIY
NSCERNSSIITNLTDKNKCKKLLDSLKSASALKEYSLNSNISTVTMVGYGIKSDTKLTWK
IMDVLMSNDVQVLSVHSSDVTISMLINDADNEKTIKLLHDYCF

>gene_1648|GeneMark.hmm|247_aa|+|4595|5338 >NVVL01000075.1 Rickettsiales bacterium isolate NORP64 Contig_source1382A_25997, whole genome shotgun sequence
MSEKSSINQHELNKFNKTEQEWWDLAGEFKLLHKINPVRIEYISSVIGKQFPLAEANSPF
SDLKILDVGSGGGLICAPLCKLGAKVTGLDANAHNTKASSEHAKRNNLDIEYIHKTAEEH
VKSSPKYDVVLCLEVVEHVANPKEFVLNLSKMVADGGVLILSTINRTVKSYANAIIMAEY
VLGWVPKQTHDHSKFVKPSEFASMLEGSCLRLSELKGLSLSMLSQEWHLSDDIDVNYFAS
FVDDKKL

>gene_1649|GeneMark.hmm|402_aa|-|5438|6646 >NVVL01000075.1 Rickettsiales bacterium isolate NORP64 Contig_source1382A_25997, whole genome shotgun sequence
MGLTMGNSLVGAASRQIGADNLKSDDLGQSMATGANKRADVVDLFLGNSLRDAEVVLKTL
ANSTNYSKNMLEITDKYLSTLAGSLQESLQTIGSAGPLSGEKRAILQQNLVDKKTQVGLL
IKTADFDGKSLLSGDIKDLGVQVGLASSDKLFLNATDISGGKLWRTSATNLIIAQMKTAN
GTAYYATQAEADADAGKNINLLSAALQNNNAVTGATGSGAVAMTDTELATLIDAVRTGNT
SFSSSLNEMAPITLAQIEIANGGDTFADSSIANLAIAIAAGGSRAELEALFGDDVTIDIS

DPALTVNLTVANNVFTTALNTIRIEQASIANQGSNIAEAADALLATINVTQRAADSYTMT
DYVTTATEYSETIRTITSAISALQAANKIPEAAQELVRSLAR

>gene_1650|GeneMark.hmm|79_aa|-|6913|7152 >NVVL01000075.1 Rickettsiales bacterium isolate NORP64 Contig_source1382A_25997, whole genome shotgun sequence
MSYLIANETQNTLNKLIDEVALSHKPQIIKGKRNSAVIISVEDWESIQETWMIVSNQKLS
DSLIKNMKTPFEECSKELY

>gene_1651|GeneMark.hmm|165_aa|+|7339|7836 >NVVL01000075.1 Rickettsiales bacterium isolate NORP64 Contig_source1382A_25997, whole genome shotgun sequence
LGAVYMENKISDIIEETISDLGFNLVKVSIHGLGTKIVEILIERQNGERVEVGDCQKISR
DIGALLDVEDVISGKYFLEVSSAGLERPLVKLDDFERFVDREIKICLRVAFNGNLKYKGK
LLGVDGDKVKLQSKNVELSFPYDNIRKANLVFTDEMFRALLNKKK

>gene_1652|GeneMark.hmm|512_aa|+|7853|9391 >NVVL01000075.1 Rickettsiales bacterium isolate NORP64 Contig_source1382A_25997, whole genome shotgun sequence
MDNNVAKAEILQIVESVSRERGIPKANLIEAMEEAVQMAGRKKYGIEHNISAKIDQNTGK
ISLFRVREVVEQAEDTFKQIELEEAMLIRPDVQLGEEILEPLPPIDLGRVAAQAAKQVII
HKVGEVERIRQYEDYKERKGDILNGVVKSIEFGHIIVDLSRAEGVLRRNQQIRGEVFNVG
DRIKAYVQDVRREPKGSQIFLSRTDDNFLKKLFEMEVPEIYDHVIEIRAIAREPGSKAKI
AVFSSDRSIDPVGSCVGVRGARVKSITNELAGEKIDVINWDDNVAQFVINCMTPAEISKI
VFDETQYKVETIVAEDQLSLAIGRRGQNVRLASKITGWRIDVMTEDQESTRRSEEFARTT
ELLVSTLEVEEMVAQLLSAEGYGSIEQIAAVEDSVLSAIDGLSEELVAEIKNRAITYIEA
KDEKVIEELEKLGVDQEIIDALDLPAEYLLKLAEYGVKTIEDLGEMSVHEFRRIVPKSVI
SKEDIEALIAFAKEQDQGENQGKDQGQEDASN

>gene_1653|GeneMark.hmm|377_aa|+|9487|10617 >NVVL01000075.1 Rickettsiales bacterium isolate NORP64 Contig_source1382A_25997, whole genome shotgun sequence
MTDNQDTKKIKKLTLGKPKISLRNKSAYPKRSAAAPGVVVVEVKRGRVSADGGMGIKDAK
SLEPDQAQLNRMLDTLKKSNDDAGPDKASISTLSKLAEMNQVPEPQADTKEDVAENTGSD
DAKKTGKDGEHAVKGGEKPAAGTDKTSSHKKEFTKQPPSVVAPAKLNEKEQEAKEAPSKP
KIIEPKKLKKTDIINMLDEASGAAPFKTRSLASIRRARSKDRRKRTGTVQAQKVYREVIL
PEEITVGELAIRMTERASDVTRELMKLGVMANNAQFIDADTAELIITSFGHKVKRVKDSD
IEDVLTKYEDKPEDLKPRAPIVTVMGHVDHGKTSLLDALKSTDMAAAEAGGITQHIGAYS
VTMSDDRMITFIDTPGH

>gene_1654|GeneMark.hmm|96_aa|+|3|293 >NVVL01000076.1 Rickettsiales bacterium isolate NORP64 Contig_source1382A_25999, whole genome shotgun sequence
KDVLLEEDIFKVRMLIFDVENIKYGELENLIVNCQFAIYEFCFEWEDANNITLLGEFSER
KEKYQELLNISRYFDDIIENKKSKIIENVIGARSGT

>gene_1655|GeneMark.hmm|437_aa|+|543|1856 >NVVL01000076.1 Rickettsiales bacterium isolate NORP64 Contig_source1382A_25999, whole genome shotgun sequence
MSKISYEKLGDGIALYKPVKSRFWWCRVRVDNHPAIKEIRKSLKTEDKNRAIYEAQKIAL
ETEIKIKENIDLMTSKPKAWKIAEKAKIRLERAKPAKPTYPGYIRILEKEIIPFLKELDK
NITEINRYTVEDFFDKYEKNSKTQVNMSKTCFKRVFEEGLREQWIEEKNIPKMPKVEIKE

TEEKRNHFNEEELATLHKRIDEYIGNSRKSVSKENRFLLKCMMLFLEASGARTGEECKIK
WDDLEVIKKDGIKAVMCKISKGKTKGKGGRKIILDGQAIEAIELVFNYQLKGIEKIEKVV
KTPFFEYYDYSNDRIDFFDFIDEAKKKLSGFHIFERKDRKEIDFSKTFELYIKWLREEEL
IGNKNHTLYSFRHTFITSKLMEKEEKRRWTAEQVAKHCGNSLQMIWDYYLHLQAEDTIFN
FDYYIELRNKTEKITGA

>gene_1656|GeneMark.hmm|122_aa|-|1889|2257 >NVVL01000076.1 Rickettsiales bacterium isolate NORP64 Contig_source1382A_25999, whole genome shotgun sequence
MRRNMSSYLKKSLLAEQLQKELEELSKNEDAKKEKQFFEDLKSVLDEHEYSFHQLHSMLS
DVLEFEKPTKKTTRKSAWQGLEIEYEGSIYKVNSIGRQPAQTKELLDSIEMTPTEFIERF
KK

>gene_1657|GeneMark.hmm|76_aa|+|2316|2546 >NVVL01000076.1 Rickettsiales bacterium isolate NORP64 Contig_source1382A_25999, whole genome shotgun sequence
MEDLQEEIERTNKILVLMLQELKNINMNLINNVLEYPEFGIKTNEKETKEERLKRFLSFI
DELENENGKKTNRPTK

>gene_1658|GeneMark.hmm|397_aa|-|2669|3862 >NVVL01000076.1 Rickettsiales bacterium isolate NORP64 Contig_source1382A_25999, whole genome shotgun sequence
MAINIILGTTGDGKSYTAVDKWIIPTITKQKRKVITNIPLHIDELKKSFPDINFDELLEI
RQGTNGEAYDAFQTADDFQDENWVNDKGQKPLIVVDEAHFCLGNDRPARELKPIEDFFAL
HRQMGYDILLLTQTTQKFSRRTLGLVKHFYHCSKLSVVGQSNRYALRIRQDGSKKSSIIQ
EEIRKYDPEIYKFYKSHAKSDGEVQEAVDTNVKSFLKHPVFYGIAFIPFFFIFLWWKGWL
NFNIFSATTKTSENTKNLERTQNSPILSNQSTVSSQEVVQKNKQIKALNGEVEELKNHNK
ELAKALEKIQSHPFTASNIIIKGSSWMPSNQSKFVFSAIVEGPNGNFSTNSKSLKKIGYD
IEYIDSCLYRWTFKEFNGYWTCGVSKDQKRLSENMKL

>gene_1659|GeneMark.hmm|103_aa|-|3886|4197 >NVVL01000076.1 Rickettsiales bacterium isolate NORP64 Contig_source1382A_25999, whole genome shotgun sequence
MFQDLMDWFEQVFDDVALTFFAMLSDLFSFLFDVITKAFLFVLDTLALGLDFMNPLQYIS
MIPDSTLNMMGVLGISEAMSIIISATGIKLLMQMIPFVGLGSK

>gene_1660|GeneMark.hmm|475_aa|-|4213|5640 >NVVL01000076.1 Rickettsiales bacterium isolate NORP64 Contig_source1382A_25999, whole genome shotgun sequence
MVRIISFLSLILFSSFSFSAEINRTDYYENIDITFSSGNTSLDQFNFNDPMIACSVALEY
THPQNEYSGASSINWALVIHREYDDQPDSWDLGCNYYNTRRGYSSSMYVRSNEFPGQDGK
FTSSFDSSNYIIFESAGSVSTEPEIPETDTDGDGIPNIEDLDIDGDGIPNEQDDDVDGDG
VPNYIDNTPYGEYTEVPGYDDMDGDGIPDSTDNDIDGDGIPNYIDSDPLSDINKDTDGDG
IPDNQDLDIDDDGFINEEDDDIDGDGILNGEDNDIDGDGTLNNQDSDIDGDGIPNYQDDD
IDSDGIPNYIDNSPNGWNVNSPEDGPTYCLIGGFYMGRGDCSNYATDENGDGEYTTDEVN
DPDSLIPDQVPIDSQPDSPAILETGYWTPIYPDGFSGVLLDFKKEIEKSKFMEFLDSFRF
TNDGTVPVFTFNFDIGIHDFGIMSIDPDQFLPGVFGFLKVIVLISATLFAFRIVF

>gene_1661|GeneMark.hmm|108_aa|-|5634|5960 >NVVL01000076.1 Rickettsiales bacterium isolate NORP64 Contig_source1382A_25999, whole genome shotgun sequence
MKRAILFFIIFAVGFAVGKARAFPVYHGYTDDPLTQKEYCEIYYPKSTVAKSEKDFLEKY
FIDYSNIPASPLYLQDMTIAFELPCFPEKTSDDLLQERIKLYEKGETW

>gene_1662|GeneMark.hmm|79_aa|-|6193|6432 >NVVL01000076.1 Rickettsiales bacterium isolate NORP64 Contig_source1382A_25999, whole genome shotgun sequence
MNSVKEKMLNKYNQAGIVAGSLILANSAHATETSNVEQLFNSVDMGGVATAVVTIGGTVL
GIFLAYKGITLVKRAISKA

>gene_1663|GeneMark.hmm|78_aa|-|6480|6716 >NVVL01000076.1 Rickettsiales bacterium isolate NORP64 Contig_source1382A_25999, whole genome shotgun sequence
MLCVELDVNNTLVANSQTVENCTTFLLMEKQDYNILVQPLTISPEQIGFVFAWGFSAVLT
FWFFGYVVGVIKSVIRQA

>gene_1664|GeneMark.hmm|116_aa|-|6716|7066 >NVVL01000076.1 Rickettsiales bacterium isolate NORP64 Contig_source1382A_25999, whole genome shotgun sequence
MELWARSPTFPPLTNLINWSIIMKYFIQGRFRGLVEQKRGKDKEYTFYYVVLESEQNITL
IKQIQYNPEKFQIDEDSFKGQTVLCPVYIDHVHKKSDSGDVKTFENIKLIDEIKVV

>gene_1665|GeneMark.hmm|180_aa|+|7660|8202 >NVVL01000076.1 Rickettsiales bacterium isolate NORP64 Contig_source1382A_25999, whole genome shotgun sequence
MNNRYTVYDKLTGKEVEAKDIEFKASKEKIRVPKEAIMLSQRDFEQLLEMRDKDDKLVFQ
GNDLRVLFKIIFKLDFENYVGINQKKLASVLRLNPSSVSRSLKKIKKIGVIEESDEESIH
TYYRLNPQFGWKGKGETWAIELKKRQVKKEVDYEKEKHEQREIDDILDNAEEVDDSSLYE

>gene_1666|GeneMark.hmm|224_aa|+|8310|8984 >NVVL01000076.1 Rickettsiales bacterium isolate NORP64 Contig_source1382A_25999, whole genome shotgun sequence
MKINKINSESIDSLRTISCLIVVLSHVYELSTNNYNNLVNELISFTAVAVFFYLSGYVNY
ISYKNKNNPLIFWKERIKRIYPAYFLAVLLSFTLAYILDIEKLSYLSNYIFLQSWATETI
ITNQALWSLAYEMFLYLIFPAFFVVRNKAFWGFLAICVVLIVFHKINALILLLCFLAGMI
KARKKKEFKEFYFFPKIGKWTYEIYIFHFPALFMLFELYSRFIA

>gene_1667|GeneMark.hmm|345_aa|-|8952|9989 >NVVL01000076.1 Rickettsiales bacterium isolate NORP64 Contig_source1382A_25999, whole genome shotgun sequence
MFIGNCKLPKLLYNNIEKNNKRRSMKRFVSFNIKYYKKTKVDVLSAHCERKIEDTENVID
KKLTPLNQYFFYGKNIKKNFNEKFEAYKKILGKNPRSDFNCYLEGVLAFSNEKFQKSYNE
NPLDFERALTESLERISKTFGFTGLGWSLHVDEGHKQEDGTIVLNPHAHLSFFNYDFENR
KSTFKKLKKKDFSKMQDICGEVFKELGFERGISKEITKKKHLEKSEFVAQKKKENLKHWK
ELERRNKSLEQKQSKLLELEYDLTSDPKPRPKFLKRFKVQFLSLFSKKQKEEIKHWDAYQ
AKMIEQRLLKEEIQKEKETIKQSYSIKMSDDRKSKSRYKPTIKLK

>gene_1668|GeneMark.hmm|143_aa|+|10090|10521 >NVVL01000076.1 Rickettsiales bacterium isolate NORP64 Contig_source1382A_25999, whole genome shotgun sequence
LIYKIYVINLNYKNNKGALMIDLIFILILTIIIIIMMFLLLTFPKKYYKIEEECEKNARK
NILIEEVKRLRFKKYDLNQRISLFCLEYSKQFNIFLEECRKNKIFPEDITEKMTTREFAT
MLRHSPWYLVKEHKSIKIKNEEE

>gene_1669|GeneMark.hmm|31_aa|+|10523|10615 >NVVL01000076.1 Rickettsiales bacterium isolate NORP64 Contig_source1382A_25999, whole genome shotgun sequence
MVYLFEGYNRDFRNEKLNDEFEKMYKENFNF

>gene_1670|GeneMark.hmm|361_aa|-|114|1199 >NVVL01000077.1 Rickettsiales bacterium isolate NORP64 Contig_source1382A_26108, whole genome shotgun sequence
MPIDLKIRNIIKEVGHIKIDDMMREALTTNNDSYYRSVQNLGEGGDFITAPEISQLFGEV
IALWAIEKWKELGSPSSFALVELGGGQGKLMRDFLRTAKLVPEFFKAAKICLFEVNPHFI
KKQQQHLDQYDKQTTWLSDLKNLPKLPTILIANEFFDALPIKQYLKSKDNWFEQVLVIDP
SDGMVKHHKLELHKTLKGQLLLDHTSAQDGAILEESVDAFNMMRLIAKNLSKYGGASLII
DYGYDIDVNVRTRKQYNATLQAIKNHKYESIIATMGEADLTAHVDFNALKKVSTEQGLEK
QTIFSQGEFLAKYGIDLRLDSLKQKLPKSDSEVLERQVYRLTAKSQMGELFKVLEVFNSA
V

>gene_1671|GeneMark.hmm|262_aa|-|1190|1978 >NVVL01000077.1 Rickettsiales bacterium isolate NORP64 Contig_source1382A_26108, whole genome shotgun sequence
MLDFPNIDPVIISFGTLAVTWYSISYVGGVLLGWYYALMLVKTTKTPLTKQHVDDFITWV
IIGIIIGGRLGYVIFYDPARFWAHPVDIFKTYEGGMSFHGGAAGFIAAAIFYARYNRVSI
FATLDLFATTAPIGLLLGRLANFINGELYGRVTDVPWSFIFPGSDGNPRHPSQLYEAALE
GGVLLFILMIAALRFNALERRGVISGLFLVFYSMFRIIAEFFREPDIHIGFLAGGFTMGQ
VLCIPMLFAGVILIFYSKKQCQ

>gene_1672|GeneMark.hmm|406_aa|-|2004|3224 >NVVL01000077.1 Rickettsiales bacterium isolate NORP64 Contig_source1382A_26108, whole genome shotgun sequence
MFDYNAAFESQIQQIKNEGRYREFVGLQRIAGSFPKAYWGEDKKEIIMWCTNDYLGMSQH
PKVLKAATQGILENGVGSGGTRNIGGNNYAIMDLEKEVADLHGSEAALVFTSGYISNDAT
LTTLAKIMPGLVFLSDESNHASMISGMKNSRAEKHIYRHLDMEHLEEILKTIDINRPKII
AFESAYSMNGLISPIDKICELAKKYNAMTYIDEVHSVGLYGDRGAGIANMMGVEDKIDII
QGTFAKAYGVIGGYIAGNSAIVDAVRLTASGFIFTTSLPPAVTAAATASIRHLKDSDVER
EKHHDSVENVKNYFKEAGIEFLKNQTHIIPIIIGDPKLTAKASKMLVEEHNIFVQHINYP
TVPKGTERLRITPTPAHTKQMAQQLTEALIDVFAKLGISLKNKEAA

>gene_1673|GeneMark.hmm|264_aa|-|3325|4119 >NVVL01000077.1 Rickettsiales bacterium isolate NORP64 Contig_source1382A_26108, whole genome shotgun sequence
MVNHDGVEHSGYMSFMVLVSIFPFFIFILALTSFLGASELGAKFIEFALENLPEQSIESI
RARISELIKAPPQELMTLAIFGSIWTSSSFVECLRTILNRVYEIKSPPNYIFRRLLSIIQ
FLAISLVISLAMLVLLIIPIGLAKLPQFVLLAKGYGETLNLARYALVFLSLFLSVCSLYY
IIPNTKLRFEEVVPGAFLTVILWTSSGYLLSKYIGHYHQLNVVYGSIGSIIITLIFFYIV
NMIFIIGAEFNYLTRRGRKKSSIR

>gene_1674|GeneMark.hmm|123_aa|+|4446|4817 >NVVL01000077.1 Rickettsiales bacterium isolate NORP64 Contig_source1382A_26108, whole genome shotgun sequence
MARKNEYIQEVDKFVGGEIYSLRLAKGLSRQQLAKNIGVTHQQLQKYEKGTNRISVGRLV
LIARALGKEIAFFYEGLDSNHKEPVTTKHQRMCIEVSRNFMKINSADHQSAVNTLVKSLS
KVA

>gene_1675|GeneMark.hmm|708_aa|-|4926|7052 >NVVL01000077.1 Rickettsiales bacterium isolate NORP64 Contig_source1382A_26108, whole genome shotgun sequence
LLTGKCRYMFFGVNIDLIIVVAFLTVTLVIGLGQGGRVKTIKDYALGGRNFSTAALVATI

VATWASASGFFIDLTEVYSHGVLFVVASLCMGIQLLIMAYILVPRMGEFLGSTSVAESMG
DLYGKKVRIITAIAGSIGSMGLIAVQFKAFGNMVSYFSDIPSYVAVIISGMVVTFYSAMG
GIRAVTVTDILQLFTFCFVLPLVGIMIWNQLYFTGFSIDTALESPKFNIQRALNIGNPKL
WEILPLMIYFTIPTMMPASFQRISMARTLEQIKKATIISAAVFATILLVMAWIPFLLFYV
NPDMESSQLFGFLIDNYTYTGLKGLLIIGIIAMSMSTADSNMNISAVMISHDICNVVAIK
KNNELVLAKVFSFLLGIGGIILALKGRDLLSIVLSANSFYMTVVTVPLLFSILGFRSSTN
SVLIGMGAGFCTVVVWKIFSIKADTIICGMLANIIFLFGSHYLLGSEGGWVGIKDRKYLD
AQELIRKKNKGSFLRWFQEFNFFDLCRKYSPKDDLTYTGFGIYCIICTITTMYSTKVELL
SPNGQIVLAIYQIMMVTGTMMAMYPIWPLSVPKIIKERVVQIWWNPAIFYMLAFFSTFFV
LISKFGQLQFVIFTVNNILIILLAGWRLSIPMIIGGFYAGIEFFKYYREIDSFDIVVGSP
QFIFMYSLVLIGALMVIFLKPKQEHLEHTEDKAPAQTKSEPEKLRPQA

>gene_1676|GeneMark.hmm|256_aa|-|7391|8161 >NVVL01000077.1 Rickettsiales bacterium isolate NORP64 Contig_source1382A_26108, whole genome shotgun sequence
MDKSIVAEIKEITTEAGAMAVNLRNKGLEISLKEDSSPVTNADKAISSFIFDRLTSIAPD
IPVICEERPPCAVDTSKQFWLIDPIDGTRSFIDNTDNFTVNIALIDNKSAAYGFIYQPTT
GLLYFTDAEQNFCVEENGIKTIRPAHGSEGLVAVVSSHHLNEKTESFLASNSFSEIVSIP
SSIKLCMLAEGRVDIYPKFGTTMEWDIAAGDALIRAAGGKVCTLSGEIMQYAKKDFRNNH
FIAMGKRWNKGVGESC

>gene_1677|GeneMark.hmm|296_aa|-|8172|9062 >NVVL01000077.1 Rickettsiales bacterium isolate NORP64 Contig_source1382A_26108, whole genome shotgun sequence
MGLLVTNKEAIIIDGKQVSEQILADLKLKIDEDKRQSKPPAMLAIILVGNDPASAIYVQN
KIKAAARVGIEVILKEFDDDIPEDVLLKEIDALNLDDNVSGIIVQIPLPAHISKAKILTA
INPEKDVDGFHPINVGRLYSPHKNGFVPCTARGCLALIKHCTNALAGLNVVVVGRSNIVG
RPLAALLLKEDCTVTICHSRTKNLAAITSKADIVISAIGKPAFLTTEYFNKDAIVIDVGI
SRVMGEGGKTRLSGDVDFQNVQKLVKYITPVPGGVGPMTVAYLLVNTYAAKIGLNS

>gene_1678|GeneMark.hmm|423_aa|-|9097|10368 >NVVL01000077.1 Rickettsiales bacterium isolate NORP64 Contig_source1382A_26108, whole genome shotgun sequence
MDSIIIRGGNPLVGEIFISGAKNASLPIMAASLLTEQTLELTNIPKLSDIVTMRSLLENH
GTEIDSAEQPDESLTLRLSAKSITSFLAPYDIVRKMRASIWVLAPLLARFGQAKVSLPGG
CAIGARQVDLHIAAMEAMGASIEIKEGYIHAEVKGRLKGCHFAFAKSSVGATVSAVLAAV
LADGETHLSNCAREPEIVDLCYCLNQMGAEITGIGTGELKIIGKEKLSGTSYRILPDRIE
AGTYMIAAAATRGDVKIRGIDYELVENLALRMKEAGIEVTPRDGYIHVKHKGEIKAVNIS
TQPYPGFSTDLQAQFMSLMTLSVGTSVVSENIFENRFMHVPELCRMGADITIRDHSAVVQ
GVDALTGAVVMASDLRASVSLVISGLCAKGETTIRRVYHLDRGYQSLEKKLANCGADILR
VAE

>gene_1679|GeneMark.hmm|1539_aa|-|3|4619 >NVVL01000078.1 Rickettsiales bacterium isolate NORP64 Contig_source1382A_26184, whole genome shotgun sequence
MRKARQAGALGAEIKDHGVTLTNASGQNMKIHAVFWLTMRIADRTITEPVIVVTHMSGDM
ILGQNVIDVHGLTFDHALSQFAFAKDPSSNPTDWATATVRTSRICSIKGSEALKVQVHLF
DDSTGLRMKGGVNFVADISGVPIVAKTNHNGVALLFVSNTDDGEVTIPRGTTIGTAEALD
RYYMDASPVSVDTADAVIHAVAVQPKSRRKPARPTKTADGGLQASASSKSQKPSSQGDST
PKLSAAKRILINEVVIRSKIPQQYRQRYTALLSKYSDIISESPHDLGHCAAVVHDIKLRS

DEPVYTKQYTLPEQEMRFIKDNVKDWLRTGVIERSTSKFNSAIFCVRKKEGQGLRVVLDY
RKLNANSLPDRYSIKSADALIRDIGEAGGKVFSTIDLRSGFWQMGLAEQARHMTAFTLLG
EGQFQWKRGAMGLSGCPSSFSRLIEIVTRGLENVVTYIDDILTFSKTHEEHLVHLEGLLM
RLRKHNLKINIAKCSFGTFETAYLGHTLTPEGVRPGIDKAKAIRDCVPPETPKQLKSFLG
VCNYFRNYLGHFARKAAPLYKLTRGDSVWKKGPLPPKAKRAFQLLQKEIAERPVLAFPNS
KGRFHLYIDGALGGESKEGGLGAVLMQEQPDGQMKPIGFASRQLQEHEKNYSAFLVELAA
ATFAIDFFQIYLKPNTFTLYSDHKPMTNLSKVHMKTLNRLQLMMTEFHFDIKWVRGEDNI
VADFLSRNAIAAINAVDTAPSTIAKAQAEDGPIAAVRRAVVTGDWREVGPQWKLSKESLH
LKDDILVIELRLRQGFSQRSKWHVVLPRALHKEVMEMAHASVIGGHSGLLKTGERIRADF
WWPNMLEDVATFLKECKVCQEFSDKNRPLQQPQQNFPLAPGPNVRVHVDLHGPFTDEKGD
KNMIVVLTDAFTKFVQLAIISHKSAAETASAILETWIYKFGVMQTLVSDGGREFCNSLQD
TLCDLLKIERKTTSPYWPRANGQVETFNKTMDHYLRTVLKEADKSPVDWRMYLGPLAFSY
NTAVNRAIKMSPFRALFGYPDRAPLWDDMDILLEKNVKSVKGKGDADYLHDFAEVQKTAR
RLAYTNNQHDRDLRAKTVAAQEEGKTADVTYHEGQAVWVKVMQHTGKNPKLQAKWETAEI
IKRVSNTTFSVQRHNRKRGRFVTLNTRYIKPRVGADVHTPDIPTAKPETTEDIDSDSVDE
EVPASNVPTEQSSLVAAIVQALETHKYLSRPELIEREINKEVSDIFAHWWHWQQKLAGMK
QPPFQLSFSRRGALTAPPGFPAAPGFVQPLPATPPVSRAPSEADQEEGSHTPVNSDWSSA
PSSNNSSTTDLVDLVERANRIKHNAVRAERGEPVSFSRSSSDTDISSDNAATTPKGYSSP
DSDEDFTDLFFTPTQDREPVLTRSESRRAANQADAPDTPEFQLAASSPPEGAAGHSASGP
TTRTPSPQPSTSAAVPRTPPPPAHTFLSYPLRDKLEQERLSLGIRVTNAFTHNKNKAIIP
QCMQFFGPAPLQVRKLGRQAEAAWHECQLRQSFPDVFPP

>gene_1680|GeneMark.hmm|363_aa|-|4895|5986 >NVVL01000078.1 Rickettsiales bacterium isolate NORP64 Contig_source1382A_26184, whole genome shotgun sequence
MAETIFKQESLFYGTDAKKSDLSAEEFLDRVTAHLNTIEDITQIQRIDAITARLRSQARL
WWKKSAKNSRTAADYRLLQTDWQAWVQCFTKQYCKGTSISDATLNWTGFKALSGESSTTF
LIRVMDAAYDFANFNKQEEEENYPAAHFQLQHTDEEDEEDDANVTHVRAAITGLNHARRT
LLTRAIHDHAQRYRDTFMTRYTVMMILKVTINGMNEPRLRPILLKALLETHLIGRVIDDL
LEAENTLPRKGKSASLHAIDVSDTEDQEEENLDAVKAKTNKKKKKGAPNKPKKTKPKADA
AKKDKVCTFCDKTGHLEADCFTRRHAMANAKAATKTSKDAKPASFEHADAIATGQQHFHS
GNY

>gene_1681|GeneMark.hmm|47_aa|+|6326|6469 >NVVL01000078.1 Rickettsiales bacterium isolate NORP64 Contig_source1382A_26184, whole genome shotgun sequence
MSEEMNEKLSEQISENFAVFSFLIKNFVPRFGWKVPGERNRRKLVEL

>gene_1682|GeneMark.hmm|389_aa|-|7460|8629 >NVVL01000078.1 Rickettsiales bacterium isolate NORP64 Contig_source1382A_26184, whole genome shotgun sequence
MQREGDEDIPEYSFKAVFREGDRIDLQAQITRSTITTNMRMTQGFHQMSHALCEVQRATR
ANKLIAILGTNNEYALFDLHGKGSKVISAGTSAYVAKCVPVEATRADYAECTEEVPVKIG
NKTFFADAVNWIIRELPTVIPCSEVMPMRWFINDRWLCSTPTTQLCAAPSRLNVSSFELT
NNNDYAIQLGRGLFTHAQLQQHRRFMANLESRRAAIAHVTLAAIFHADDSGALGIPISKE
ALKKLKHDIASSYFPMIAWLGWSYSYIYGAIAIVVALSVVAGALSRAFILYRERGCGWWI
FGAVSAAVFSIIRFQFQIVRTTRDLVVNNLDTTVNTTAYQEIRTENAAFRKDIERLALQV
DTLTHTQETSHKLYPSPVLPPQDFHKPSD

>gene_1683|GeneMark.hmm|61_aa|+|8624|8809 >NVVL01000078.1 Rickettsiales bacterium isolate NORP64 Contig_source1382A_26184, whole genome shotgun sequence
LHASDESVNLGTEALEAADRDGIRENETRSLFGVVVGHEEDGTVHGVAPGPMEQSGPEVD
L

>gene_1684|GeneMark.hmm|159_aa|+|8834|9313 >NVVL01000078.1 Rickettsiales bacterium isolate NORP64 Contig_source1382A_26184, whole genome shotgun sequence
LRCPDECANRIVLSDPVVVADDQVVEGGEGQRALGLGEVSGPVDGVEGEVKCCLLVGVVE
LAAGRDEGCCCAGAAEEAAVRVVLVALRLAGTRREDLPFEGEEVLPVNPAAVSWIDGNVP
LPHHSFATVVERREAAAANDLGNRYPALESGDWKCRVCV

>gene_1685|GeneMark.hmm|127_aa|-|9814|10197 >NVVL01000078.1 Rickettsiales bacterium isolate NORP64 Contig_source1382A_26184, whole genome shotgun sequence
PATTSAHLPLVPAKGQTRAGTALARDQGHERVHTQQEQSHHSAMHAVLRTSSVAGKAAGK
TGRSSLARVPTATVLPGRLSAGAQIQTRSDEADGVQAHTLNESATSAAINSLINGVISSK
LLGYPFT

>gene_1686|GeneMark.hmm|270_aa|-|52|864 >NVVL01000079.1 Rickettsiales bacterium isolate NORP64 Contig_source1382A_26257, whole genome shotgun sequence
MVKLSGFIIAKNEATRITKAINSIKNIVEEVIVIDSGSTDDTVKIAEKLGARVVYNEWKG
YVLQKSFGESLCKNDWILNIDADEEFTQKLQDEIAGIFAEERQDDCHAYEVNFVVMHRND
KKLRFLAPANRFIRVYNRNYCSFSNVKETTTHDAVMFNKDTPKDSYKLKGAAYHRSGTSI
TQLVAKANFYSSEQAKDWKKLGRKPSNFRIALELPLCFLKAFFIRRHCVFGFDGFVDSMV
FAFARFIRIAKTREIYRNEESEANTGFRKT

>gene_1687|GeneMark.hmm|284_aa|+|1192|2046 >NVVL01000079.1 Rickettsiales bacterium isolate NORP64 Contig_source1382A_26257, whole genome shotgun sequence
MPVLMQTTIQNPISCYGIGVHSGKNTQITLKPAKRDTGIVFVRTDVSSVDNEIYASYENV
FDTSLSTSIKNNANTQVHTIEHLMAAIWGCGISNLIVEIDGAEVPIMDGSSKPFVFMIEC
SGIQKSNVPQKRLKLLKEIDVSDNGCEIVGVPSDCLSIDLTIDFASPAIGKQQHIFTEQD
GFKDEIANSRTFGFLHELDYLQSKGLAKGASLDNAIGIDKDVILNHDGLRHQNEFARHKT
LDQLGDLFTAGNLIADLKSFKTSHYLNNQFLHKVFSDPYSYKWI

>gene_1688|GeneMark.hmm|135_aa|+|2305|2712 >NVVL01000079.1 Rickettsiales bacterium isolate NORP64 Contig_source1382A_26257, whole genome shotgun sequence
MPDYINIILLIILGFAALFMIRKMLTHQKPKHIVPKDPASQDLYEQQKHQGDEEIILSME
EKIELSWEFLLGIVRKIITIFSPEDKEQLHQAGGVLNKNGMQYQHDVNQEVIMMDNLSKV
SGVQKQKNDSDGRSR

>gene_1689|GeneMark.hmm|202_aa|+|2808|3416 >NVVL01000079.1 Rickettsiales bacterium isolate NORP64 Contig_source1382A_26257, whole genome shotgun sequence
MTKIKNSKAMIVILSAPSGGGKSSIAQKLLAQDENLSLSISVTTRAPRASESDGVDYFFK
TREEFQNMMNADMMLESAEIYGNLYGTPVQHVESTLAQGTDVLFDIDSQGAYQIAEKMKD
RIVSIFIIPPSIEALRARLESRAQDDKATIDKRMKLAKDEMEHAKNYDYMVVNDDFNQAV
TEIQQIIHTQRKQRDYKQRVSK

>gene_1690|GeneMark.hmm|412_aa|+|3413|4651 >NVVL01000079.1 Rickettsiales bacterium isolate NORP64 Contig_source1382A_26257, whole genome shotgun sequence
MKKPKKRIAGAFLGTIVEYYDYSLYGFSAGILAEKFFPTMSKIESLTYVFAIYAISYLAK
PFGSIFFSHIGDFYGRKISLRITMIGIAFPTLTIGLLPDYHSIGNLGAYILVICRFLQGF
FVAGEYDGAVIYVVEHIGKKHHYAASATARSTGVAGLLLGIAATNFFNSSIFPEWCWRIP
FLLSVPLTIVTIYYRKFLEETPEFMRSKAKNTEFSSTLSFIKKRWRTLLMVILLSGGFGV
TYQVSIIFMKQYLPMVVPYTGTIITTFSVIMVLGFGLAMPVAGILADRFGCERLVKYSLL
STLVASGLLIISINHQLINLTLISCLMLAVSVAPFNALAHGVIIKAFKPNERYRGVSLGH
TTGSMLMSGTANYVCLLFMQKFGFNLFPIFYIGFFAIVTFVTVMLFTRTYKA

>gene_1691|GeneMark.hmm|98_aa|-|4699|4995 >NVVL01000079.1 Rickettsiales bacterium isolate NORP64 Contig_source1382A_26257, whole genome shotgun sequence
MSFKLLFTKEAASNLEALENDPSKKKRLKAVRKSLGYLETNPRHPSLNTHEYNSLSRDLR
VKIFEAYAENKTPQAYRIFWHYGLGKSEITIIAITPHP

>gene_1692|GeneMark.hmm|89_aa|-|4996|5265 >NVVL01000079.1 Rickettsiales bacterium isolate NORP64 Contig_source1382A_26257, whole genome shotgun sequence
MRTQKILRPDAKGRICLGLLAKGISGYEAVINKANKEITLKPYTEIPLSENWLFKNEEAR
DSIKRGLKQSADNKTIYKGSFAKYLDEDN

>gene_1693|GeneMark.hmm|506_aa|+|5488|7008 >NVVL01000079.1 Rickettsiales bacterium isolate NORP64 Contig_source1382A_26257, whole genome shotgun sequence
MKFFRFFIIIFLILNSFTIFASDNDIECADSTLKQKLLSGWYLWEPYQFYKITPGGAAVL
TGMDVQLVKSLAKRVGVRIEYEEISWNQHQQNIREGTLDIASGATYTKERAEYAYFSDPY
RFEENSLFVLRHSEKNLSFETILEFLAQIRLQNFRLGITKGFIYSDPQINLFISDKLNRD
IIYQYNDDVEAIESLLKGEIDGVISDRVVGAATILNHKATSLIREVELNIKTPISLMFSK
KTVPVRLVNEFNKEIKRFSEGDERNNIVKTYLYPVMLMQTIESNWFYIIGVIGTIAFAIS
GVAIAAKDNTTLFGTFLFAMLPSVGGGIMRDIMINRDEVGIFLTPSYMYYIIIVVLVGFA
TIRLLEYYNQKANEDDVIIRFWDNLLILGDALGQASFIVTGVSIAIMSRIEPIILWGPFF
AFLTANGGGILRDLLRKKRGIICLNGEINAEISVFWGVVFSVYLDVTSHDPDSSGIRTAV
IIIATSAFITRLVTYYLKVPNLKFRK

>gene_1694|GeneMark.hmm|538_aa|+|7063|8679 >NVVL01000079.1 Rickettsiales bacterium isolate NORP64 Contig_source1382A_26257, whole genome shotgun sequence
VAKKRHKNCSNRFRDGNMLVKKRHKNCFNRFCDGNMLVKRRHKSYIYRLCDIKINFNMQD
FPFMGREYELKSLYDLTQKKSASLVVLRGRRRIGKSRLAKEFATKYKPVKCRFISFAGLV
PTNDTAATQKEEFARQLAKNLNLPPIKADDWGDIFTHLARETQSGRVVILFDEISWMGSK
DPSFLGKLKNAWDLEFKNNSNLILILCGSVSSWIEENILKSTGFMGRLSLVLDLQELKLD
ESNQFLSKIGFCDSVYERFKVLSVTGGVPRYLEEINPSKLADENIKDLCFKSSGILFREF
NDIFLDIFARRSKTYEKITRTLVTGRKELKDIAKAIGTQKSGHLSEYLSDLVKSGFIKRD
YVWRIKDNKESKLSYYRLSDNYLRFYLKYIEPNKSKIENGHFDNKALSTLPAFDSIMGLQ
FENLVLANRQLVLQKLNISPEDVVNDHPYFQRPQQRVEGCQIDYLIQLKQNILYACEVKF
SRKEAGMSVIQDMKQKLDKFILPKGFAIIPVLININGVSDSVRDSDYFRLIDFSDMLN

>gene_1695|GeneMark.hmm|354_aa|+|8968|10029 >NVVL01000079.1 Rickettsiales bacterium isolate NORP64 Contig_source1382A_26257, whole genome shotgun sequence
MGVLLVFGILLTVKCRYMLFGVEIGLDMGIVIGFLVLTLAVGMGHGKSVKTIKDYALGGR

NFSTAALTATIVATWASGSGFFILMSKTYSDGLVYMFASFGIGISFFIVAFLLVPRMGEF
LGSVSIAEAMGNLYGKKVRVITAIAGTIGAAGSIAGQFKVFGNMFSYFLHIPDYIAVITA
GFIATLYSAFGGIRAVTFTDILQSVAFGVIIPMVGFIVWGEFFNEGLSFSQAASNSKFNL
DILFDSGNPELLGMVVMFCYFCIPTVSAPAFQRIAIGRNVEQVKKAFLIAGAFLIFIKII
IAWIPFLVYSMNPSLESGQLVSYIVDTYSYPGLRGFIIVAIIALSMSTADSRIN

>gene_1696|GeneMark.hmm|30_aa|+|2|94 >NVVL01000080.1 Rickettsiales bacterium isolate NORP64 Contig_source1382A_26406, whole genome shotgun sequence
QSPKTPKPQNPKTPYLNQGKQLIIILITER

>gene_1697|GeneMark.hmm|43_aa|+|974|1105 >NVVL01000080.1 Rickettsiales bacterium isolate NORP64 Contig_source1382A_26406, whole genome shotgun sequence
VQLQPMEMLSARLVMLLLQVIATLAQSSEKSMLSDIREQLKQS

>gene_1698|GeneMark.hmm|91_aa|+|1102|1377 >NVVL01000080.1 Rickettsiales bacterium isolate NORP64 Contig_source1382A_26406, whole genome shotgun sequence
LTTDNKCKKMCGPKQIELMVGEKDNHILICQDCDAGCEHGCRGNTKSCKPDSTKTPADAD
FLSAHCATGKKVEKFPRRFKKNNRGPMPADQ

>gene_1699|GeneMark.hmm|72_aa|+|1409|1627 >NVVL01000080.1 Rickettsiales bacterium isolate NORP64 Contig_source1382A_26406, whole genome shotgun sequence
MQIWKTKLNSKSDVLPLAQMAMDSLLQRLLNLSVKCAKLKELLIVQPHGMQIVTVVKWFQ
ELLLAKKLKNSS

>gene_1700|GeneMark.hmm|48_aa|+|1624|1770 >NVVL01000080.1 Rickettsiales bacterium isolate NORP64 Contig_source1382A_26406, whole genome shotgun sequence
MTSEGRCKHIKTINCPPAKFFDKAAKACADCTGCAKCHGAADNCSECA

>gene_1701|GeneMark.hmm|27_aa|+|1837|1920 >NVVL01000080.1 Rickettsiales bacterium isolate NORP64 Contig_source1382A_26406, whole genome shotgun sequence
MTDKACTVIEGGRKERDILDKAEEEEE

>gene_1702|GeneMark.hmm|161_aa|+|2159|2644 >NVVL01000080.1 Rickettsiales bacterium isolate NORP64 Contig_source1382A_26406, whole genome shotgun sequence
MELVLQLLIVELASLLTPLKVNVHLAKPIIALNVNTEETVAVKSALLISSFTKENVSTLA
IPLKDYLRSHKMERKHVNTVTTIMVAKHVKLQIQTQQLVKSVTQLTLFSALNVSKIAHLR
LTERLQTMKPRVHLVKMMLKLSNADQEKTPQVLMYYKQWNH

>gene_1703|GeneMark.hmm|89_aa|+|2833|3102 >NVVL01000080.1 Rickettsiales bacterium isolate NORP64 Contig_source1382A_26406, whole genome shotgun sequence
VHPVTGECARDGPTSECKTDLACAFCKKSEAAGSGALCTSCPTGKVLKSGLCVDPADCDA
GQWGGKKRMGDVEISACVPCFEGCATCNK

>gene_1704|GeneMark.hmm|33_aa|+|3239|3340 >NVVL01000080.1 Rickettsiales bacterium isolate NORP64 Contig_source1382A_26406, whole genome shotgun sequence
MEIQQIRNAKLAVQTNSNALKQMEQQPAIEDLF

>gene_1705|GeneMark.hmm|119_aa|+|3365|3724 >NVVL01000080.1 Rickettsiales bacterium isolate NORP64 Contig_source1382A_26406, whole genome shotgun sequence
VQHAQPTNSTIESLLQMLKVNLKKFVLTVLVIVQNVNTKHHNVLSAQLISQWLLEFIDVG
LHPLHQKVMPLTRIHLNLSQIHKPLFNAQLHTKTALLVERLIHSKLLLFHVLNVMTDSN

>gene_1706|GeneMark.hmm|109_aa|+|3745|4074 >NVVL01000080.1 Rickettsiales bacterium isolate NORP64 Contig_source1382A_26406, whole genome shotgun sequence
MCYEKCPIGEFFSNKANMCRQCKGDDMACKEVPVGDPQTATEFDYKTIACKKGAIMRKGR
HGERSCVSKCGKNEFEAFEELEIKNEGGNMSKKPDQGGDKLDSEAKGDF

>gene_1707|GeneMark.hmm|98_aa|+|4124|4420 >NVVL01000080.1 Rickettsiales bacterium isolate NORP64 Contig_source1382A_26406, whole genome shotgun sequence
VKKICKTQIHKKMEPERLSWIKMMKEKEMLNLSKLTLNTKRNVMEQKKILMHHILPEMLK
VLTKPSLKERTKSQFSKRMVMKNQNSKKNFKTTQLIKR

>gene_1708|GeneMark.hmm|214_aa|+|4417|5061 >NVVL01000080.1 Rickettsiales bacterium isolate NORP64 Contig_source1382A_26406, whole genome shotgun sequence
MKECNDLDKTRKKNQKFKPMSIKVCRPCPDWCNDCNKFGQCKACKNSQDVLDKQGNCVCK
DLTSNIKVLDDKERGTVIIITYPGRIDVGKDDKREEKMDDLEERNGGQHKPDKEEMDGVM
HSWGIDRDQFEIFRGKAERIMNNEKCQLKWNMARSMGEFDKRLDEEFYEEKEEKNRKDKM
CKAYNHAEFLTAADGEAAGFSGEEQGDRQKIEGC

>gene_1709|GeneMark.hmm|71_aa|+|5048|5263 >NVVL01000080.1 Rickettsiales bacterium isolate NORP64 Contig_source1382A_26406, whole genome shotgun sequence
LKDVKPRHSKMSFSAQLPINAELLELNHCLRNKNHLEVRMRKISNNSVIKNLRTLKMHRP
YFQNNKEMMQD

>gene_1710|GeneMark.hmm|45_aa|+|5314|5451 >NVVL01000080.1 Rickettsiales bacterium isolate NORP64 Contig_source1382A_26406, whole genome shotgun sequence
MADIIKAFMSANKGSQKDILRSSENEKDDSCGKEPSMSQNDKENM

>gene_1711|GeneMark.hmm|88_aa|+|5432|5698 >NVVL01000080.1 Rickettsiales bacterium isolate NORP64 Contig_source1382A_26406, whole genome shotgun sequence
MIKKICKSNSMERLSNHLLQEKEMITLLSKEPKEMLDYLEKIDKKKLNVVIKCFKDLERL
LDLKHWEDVKYHQLTKNREKKKKEAKEK

>gene_1712|GeneMark.hmm|34_aa|+|5695|5799 >NVVL01000080.1 Rickettsiales bacterium isolate NORP64 Contig_source1382A_26406, whole genome shotgun sequence
VSGDANEISDRDGGDRTREEEQQMEQIVGMIDDA

>gene_1713|GeneMark.hmm|128_aa|+|5789|6175 >NVVL01000080.1 Rickettsiales bacterium isolate NORP64 Contig_source1382A_26406, whole genome shotgun sequence
MMHKKKCLESKEVMKLTVKTDKKDKNHFNKVRKMLKDLLHISKLVYQQKRLKIIEEVMER
ENLQKMLLDQEEKRMMKKPERTSNQIVQTKNKITDQVVERSCPMRIWNMFKTAELDILRT
ILEIGQQS

>gene_1714|GeneMark.hmm|313_aa|+|6304|7245 >NVVL01000080.1 Rickettsiales bacterium isolate NORP64 Contig_source1382A_26406, whole genome shotgun sequence
LKSRFDTKPMTGIYARGEQGELEKCWEHKFDFPHKDEVEEDFIKPCCQIKVLNAADNSID
EVNTPKCQAQFAATQSISKALSADLAGKTIVFNCEQENKRGGLCFQQVFKVNENDAVTYR
QLDRVPDRKGRCEIKGDTFIQVPRLVKNSCTVQESEEGLAISLVLKKKNAQGTFDSATAD
LPADFDIANVKMGSRAKFFNALPVGIYKATWAWDGVSGEEFVDIFCIRNKPIGLNCMDLR
IEQGNGGAMKCDAENLNSVDDISVSVAGGCADPVKTIVEGKLVLTFATIDAAKDCELLIT
DANTSEELAGNRV

>gene_1715|GeneMark.hmm|113_aa|+|7297|7638 >NVVL01000080.1 Rickettsiales bacterium isolate NORP64 Contig_source1382A_26406, whole genome shotgun sequence
VKQEQTLKLNIDEPNQFFLQCGRGTAVAGTTISLFDSEFTLITTFDVSKNGGFVIKGADA
NKFTEVDGATTGYLSLENGSIQVERDLELLGVGYDFSVENVNGDNIAFEANTY

>gene_1716|GeneMark.hmm|52_aa|+|7681|7839 >NVVL01000080.1 Rickettsiales bacterium isolate NORP64 Contig_source1382A_26406, whole genome shotgun sequence
LEKGGPKKGKINRGCKIETTSDGFTIINPLPKGKPSTGIAICVMEGDYKVAC

>gene_1717|GeneMark.hmm|60_aa|-|7836|8018 >NVVL01000080.1 Rickettsiales bacterium isolate NORP64 Contig_source1382A_26406, whole genome shotgun sequence
LHDFLVSLSKLAKSEHAALVNSTHRLASSLQIDIFSFRIAAPSSPKAAALFELSFVIDNI

>gene_1718|GeneMark.hmm|95_aa|+|8177|8464 >NVVL01000080.1 Rickettsiales bacterium isolate NORP64 Contig_source1382A_26406, whole genome shotgun sequence
MILFQIVKHFSPTLKKIKIHLLKKKREIIAKSFKKLEQLLPLLRQLMHLKQLWTRTGQHR
FSKEQNSPIRTKLLLRLKLLKAQSELNSQRADVKI

>gene_1719|GeneMark.hmm|59_aa|+|8669|8848 >NVVL01000080.1 Rickettsiales bacterium isolate NORP64 Contig_source1382A_26406, whole genome shotgun sequence
MMEQPQVSTMNHLLKMNLLAVLKKINSAFQLDNKLMQMEILFQEMLSKSTQKIQITLIK

>gene_1720|GeneMark.hmm|265_aa|+|8845|9642 >NVVL01000080.1 Rickettsiales bacterium isolate NORP64 Contig_source1382A_26406, whole genome shotgun sequence
MTSDTVADNIIATSCSASQYFFLETASSSISADRFYMGFILYTIIFLDLYLIFGVILGKK
FDNEGKSAVQIPSDDEKSVNANNTDMGLKGDIQEQNVRAKKNPTCCFALKQKHRCISVFS
AYHPLLSRPARTLLNVSGYFLALILTGGMLLAMEDIVGGIIGFAIAFLFYRLWIFLGEKM
FKANKCLGYIVGLILVVGGHGAGVAFTLTQIDEAFTFWGIFFWAGIVIELAFWEIISFCV
QNGIAKKANKEKRSWVTIPLWKAYN

>gene_1721|GeneMark.hmm|277_aa|-|40|873 >NVVL01000081.1 Rickettsiales bacterium isolate NORP64 Contig_source1382A_26629, whole genome shotgun sequence
MSLSACKGISIDGEQWFNSWANDKCYDIMYNLVYKDMGESSYNVIGYEMSQDIMVEVFEK
YTNTYNITSPGLEGYNSFQNTLRDVCSRLPGVCEKALESYCHSCPKCNRRDMSLSKPTLE
FCGCYIRNGKTTKQCDPLCNRVNTIQLPNGSGGTLKCNENICVINDVTIQAAKSDLSNKS
VTFKQLCNNCVGGCKCIISGINITSLLNSTGITKNIDQHCGAKSTCLKILPNGTDEIVNC
LTQLPNDKHKEIYYISLTFICVLLFAAIIFYFYKRNK

>gene_1722|GeneMark.hmm|66_aa|+|914|1114 >NVVL01000081.1 Rickettsiales bacterium isolate NORP64 Contig_source1382A_26629, whole genome shotgun sequence
MESKSENFRKNAVGFMYKFFGVFDKTEFRLHSVMNAEDNTTRFLENLDKSHNKHYSKRYK
GGSPKK

>gene_1723|GeneMark.hmm|401_aa|+|1152|2357 >NVVL01000081.1 Rickettsiales bacterium isolate NORP64 Contig_source1382A_26629, whole genome shotgun sequence
MEFGPNIYPIYSQTCKTKKNISFVISNGSVRASIDSGYLKIQDLLAYLIYSDVSNPNVIS
DDCLSAYTQICIYKYDQYMGNIHFCEIVNNEIYRMIYIECKCDLFLRSSTAGIILHFMTK
ALLLLREDSEECTKSKMSHLENIPKNSIPLAIRCCNSNVENNVEQIAKLCVFHDIGGYLE
TTDGYKYENYVLDKCLYLSSEHEEYWETTAKEIYKKLIYGVPGISMSTRASLSFIYGIGG
FDLFTLKSMRYLCMDKNILSYSLGIKFDTGIPTEKVMVDAVKKLELLGIDKYTEEVKSLN
ISKYDPLSMFSHLGVKSVVCNNKYDIHSYNNSDLVYFCDGGIVYVFIREDFKKLIETNIN
PFSGKEFPYRIIIEMYKKVNSSHRMGLPECSSIRKLLDMVN

>gene_1724|GeneMark.hmm|215_aa|-|2381|3028 >NVVL01000081.1 Rickettsiales bacterium isolate NORP64 Contig_source1382A_26629, whole genome shotgun sequence
MYNANEETVKATKNNVMFLQDVEQHEYIIPEGIIFFRTSRCKINRLVLSKEVLDVKCDSC
GLLEIILNEKVLSLDCSNNNLSGTLIVKHRMHKFHCNNNNIKNLVFKCTPAILSCNNNII
GNLTFNKTSPSIISCYGNELTRNKDIEISYTKPLNYTSIAYNMRTGRYPSKRKYMCISCN
SFVSSDLIYTKTRKNVTGYTTKTRICIKCFRRMIF

>gene_1725|GeneMark.hmm|89_aa|+|3240|3509 >NVVL01000081.1 Rickettsiales bacterium isolate NORP64 Contig_source1382A_26629, whole genome shotgun sequence
MKPIIIVYKMSDEYVFNVLSSESENKIKINVTGNIDNIFITLFLGKDSYIINTKDVNIMD
LNPHPKCKAEVMVPSVSSVATLPRNKIPH

>gene_1726|GeneMark.hmm|1098_aa|+|3547|6843 >NVVL01000081.1 Rickettsiales bacterium isolate NORP64 Contig_source1382A_26629, whole genome shotgun sequence
MSSKETKKNAKPESKVRGRINVNRRKRINVNRKRRSNKTSDRQGKISGGRSTKVNVNLTK
VREERKKNREYKNKGYRGRTKPEAIESELISDVTNDGILVTREAMYRATSKAKASSSKLP
MYVVRGVTIRVADQETLIRESVATISNSSARGKNSINDPRTGVTMSDVLCPTCNQGITCQ
GHPGLIKLSRPLLHPMYIREIIRILRIICNVCGEPLMGFEEMKNEGITSLLSDVRLKVLE
VKCIKRTCQSHAKFGIPEPCRVNPQYDVKKSDTARAIKMNAGRSTLSAGKVSEILGRLTN
KNAILLGFAYGMHPKNYVLKVLPVMVPRIRPSSVHSGTTMEDAQTQTYKKIINANNAILN
NLNSADADNKYAELVGYVNDFLWKNNSQSNNPHTQAANIPKKLGNKEGILRRNMQGKRVD
NSARTVIVGVSGIPFWVVMLPRVIAETLYAVETVGPGNITWLTFLLRNNKITYIIPYGGP
RKGISIKIDINSGAPNIGYTLKFGDRIRRNILDGDHIIVNRNPTLHKGGLLAYEVKIWDN
LTLGMTLPATTLHNADFDGDEVNIHMPTTPAAVAELEIIVTIKAQIGVGVIFDALTSVNL
LTFGNAFNDRSNDIDKSKEINVRIGKCNFDQLVNKIYVIGSLGDNITPSFNESFSFRDYR
VRVGELDKPSNGFSFKTYIEKLKIIDEFGIGKYTGKSLFSLLLPNDFNYERKDGDVIIRN
GVLLSGVIGKDDIGPMGSLVVKLRNSYGLKVRNNFVASATNVFESWISMRGFSVGYDDCH
PFNEKERLILENTVSEEVIKAEAHVMSLGPVKKDPMEEEERENEVISTLNIVKTIGQKIT
KEILTRIPKSEENEDDFVVIISNKFMEHDTIGYGKMVIEGDVLTVEYDNTIIASNDWFYV
DEILNRSLYVVADKTIIEIREHDEYICGIMKENSLVIMSDSKAKGNPVNVAQITSLLGQQ
YFEGKRPGSTVYFGKDSSNIRARGFCTRSYTQGSTPYGFFAVAKAGREGLVETATGTQDT

GTSQRSAVKALEDITIRNDGTVRASNGKIIQFHYGDNSVDTERIIFKKVGNKNVRTFIDI
SSEVNKLNTGYGIMKPIK

>gene_1727|GeneMark.hmm|304_aa|+|6887|7801 >NVVL01000081.1 Rickettsiales bacterium isolate NORP64 Contig_source1382A_26629, whole genome shotgun sequence
MFKLPKSFGNDKVIMFPDENDVLNGILIIEEGTQVCKCSNLNIKYIFCPKSLIYLDCSMN
KLKIFICSDNLSYLICNDNDIRSITFNNKLKHLNCSNNNIEQLCPNDELINLFCPGSHIS
HVEFTKSLLKVTCSHNNLKNIIVNKGLLHLDCRNNKINFILLPKSIKYLNVTSNPLANIS
IKGNNAYDKAITKCIKYPNVQHKIGGLYANRYKKNITPVISNSRKIPIDFYVSRRPNPTL
YSLCYRLTKPKNKSFRVLELSNCQTCWSKYPTNLIGDVKVVTVGNVKTAKVCIDCIMEQK
LIIM

>gene_1728|GeneMark.hmm|175_aa|-|7856|8383 >NVVL01000081.1 Rickettsiales bacterium isolate NORP64 Contig_source1382A_26629, whole genome shotgun sequence
VDIINTIQSNSISFIYLVYIMKYMYIVFLVILVYLIWSEYGSQDCIDQKCHNKVEEVNDL
KFETILGVIRNANDNNHCIVVWRRAMISAITTGIILNKIIYNSDYSLFIVITVVFGLSYI
FSMKTQKETRSKDIALDNELKKLNKNIVLAQIPFGILASTESPHERIKHVGYSLY

>gene_1729|GeneMark.hmm|294_aa|+|8340|9224 >NVVL01000081.1 Rickettsiales bacterium isolate NORP64 Contig_source1382A_26629, whole genome shotgun sequence
MKLILLLCIVFIISTSVLFLTFRGKGEEEITTPKNKPLDELNILTGSSKSGIEIVGGENS
GSYYVDSLSCNKSDGIWLHTHLGKHKCKCDFSLFGKKCNRVAYDDTYKCLGNIDKTKINL
DIISEQNVSNLSFVHNSCTSLCNDEDNCVGVHYEDKVCTLFSKISIEGTITYSHSVDGNL
YMKGNNKVIIIDRVFITKGKLPLRYYLKDRYSTKDGLDITWAIYQMTQYKIHFTPTFIIN
DPNWIGVYSNNPINLDNILQIISNGTTADYFVSYTLDGLPIWKITYLVYVEITP

>gene_1730|GeneMark.hmm|1382_aa|-|3|4148 >NVVL01000082.1 Rickettsiales bacterium isolate NORP64 Contig_source1382A_26648, whole genome shotgun sequence
MKRHVDPINPNIGINAVAAVDPILGIVSAKVIARQEVGYEDVRKLLKVATKTSLPIVKKN
AVDLLMAILDKRYPKPARSADLMAKNILNYFVSESNLENISLNDAAILTIASYLDNGRCR
NSATKALIAIKVNNPLPKQASDLLAQQKGGLAIGGPASSSAAPSPSGSLAQLNEQHMGTV
QSSSNQREAEQGEVGQREAEQRARILSRMIQGLAVRSGEGHYQLSKKTLGYSKLNPGDEG
LVNIAKGLPYEDAEKTQEILTYLKKLYLRNKHAPDFVIAKLIELEALPQIQAESIEEFIP
AALIATNQDGAKPRHVLIDEIGAANKNNHALSSWLSGASNELSRIDETITQDSQAFPQGK
AIDNWQAEDILAWSEAVSKEHINEDLLYELIAVASQGVRLHKGYAPRDIQTLSVLALLNK
QPDQGRLIQVKTGEGKTIICAMLACITAFRNKQPVDVITSSSVLAERDSAEQVAYFSLFG
LSCSNLTRGDSNAPKECYTADILYGSASDFQGDYLRDEFKRLGTRSNRVFGSVIVDEVDS
MLVDDGAKITKLSSPRPGTEYLAPIFMLTWKAIANLEITDEVLSNKGLRGELTEQIQARI
EAIIDGEYFSYPSYLLGYISSQIKNWANSALAASVMREDKHYVIAQDIDSGADIIAPVDY
INSGVVQNKTSWGNGVHQFLQVKHSLMMSAESVTTSYISNMSFFMKYGANIMGMTGTLGS
TEAKSLLGEVYHIDTAVIPKFKPDLFHEEEAIITSDEATHKAAILSAATEVANSGRAVLI
ISESIAFAKELTADLQAICPPNKVHIYSRTDMEEMNAVSNIQEAGNIIVATNLAGRGTDI
KLTDEVNENGGLHVIITFLPSNLRVEEQAFGRTARQGQPGSGRLVLNGAEEASKLSLEAD
SSANTDSYKAIRDLSEKIRLAEVKEVRLPKISFEDRLFERFNQEIYQHLRTTEENRYKLA
QLEESWGFWLKDLMVSHKNSENPETQSAAYLEFEAFKQEQIALYNSDVATSNPIYQIQMG
HSRLGRNNSYDESIRLLEQVAKPEDPYSFAGHYYLAYAYLRKNLAGKAKNDQGLVAAAYR

HMHIAKSHLEEVIIPQLQSMQLLLGTNSAGSPISTQITNKVDLFNHHLSYINHALKFIES
SNPKNIMTVGKTKRFEEFYAEGQAPMSEIRELNSLGAMQLYELDAKKPPKNILTSVVVII
IGVAQVAVGTMMAMASGGTLGIGLIASGLGDMYQGINAALKGEGIHLGEYFKAKAIEFAI
NVISQGIADKLRPAAKAGAVVKKGAELSTGQLLFKIGTRVGMRLAADQVVVLATKELSRG
ALKKAINRAIRSARRELESRLSSPRIAAHLENMLAIDNLYNRKYSRQLSSAARDFFTRRS
SE

>gene_1731|GeneMark.hmm|946_aa|-|4273|7113 >NVVL01000082.1 Rickettsiales bacterium isolate NORP64 Contig_source1382A_26648, whole genome shotgun sequence
MENAVSPPVSGSTLQNLQLSAAGHQRSLNALSAERNALNQSIAQLRNSNQAGQNQYKNDL
QKLDTVKKQILEHQREIIALNRDISSMEMGMDNISIFQDFMVEMKSILSKYSSVVAGRRI
DEAEMVVKTLPENELMHTDIAKATCERQSLGAFNELLSKLGNINAQDGEGRTLLMHALMH
GFFPALDLLLARPELDCNITDNKGQTALIYACTMPHLKYVPQILSRTADVNLKTLETGNT
AMHLVIAATRDKLFSDEYDDYDLTDGSGNVFFDASQFVGNLVITGGTFTMGSASPTGRTI
NQAKTMLLINMLCEGGLDFNAQNNQGQTPFMIACAHRLKYLVNTCLDQGDLVDTSKIDNN
GYNSLLWVLEVRDTAIVERFLNDGFSADYRDSEGRTSFFWMSIYNQPEMLQLLLRHGADP
SICGNSGSTALHAAAETGAAKVMPILLNHFDVNERMDTPHQITPLWMACQNGHLEAVKYL
LTHGANPALGRADMDISILRIAINKQHLAVVQELVARDDVDANAPDNGGIYPIHAAAELG
STDIVRLLLNYSDVNVRAIAPHQITALWLACQNGKAETIELLLQSGGDINLARADNGISP
ISMAMQKNHVYVVKKLLARDDVDLSAVDGANAGVIHYATVLDNLEIMQILVDKQIDINAQ
NAAGLTALYVATLKNSPEMAALLLAGGAEHDIPNVQGFYPIHGAVHHGHMGMMEILLPFI
NTQSSGGHTPLYYAIICQAEPNLELIQFLFDHGADSSISHAGGYFPIHGAVHNHHLAALE
LLLQNGVDINSHDLEKHSATPLYYSMGYGNQEIKLGTVDVLLAHGADTNIPEFDGDTPMH
MAGYRARVDLMQRLIAHGASVNAQNDNGETALYVCIKQPDFATDEQKLLAVQYLLANDAE
VAPESGLSALDAASQYLVSAMGFLEHPESVRPLEEFNIEVLGGDEV

>gene_1732|GeneMark.hmm|603_aa|-|7145|8956 >NVVL01000082.1 Rickettsiales bacterium isolate NORP64 Contig_source1382A_26648, whole genome shotgun sequence
MGSVGKGFCKIFGINHKKRIIIDHEEERRVNELTNIRANNKYEASGRAPGVFSDFEEALA
YKDWLSQSGADNRNSPYYSFARSDSKQRTKVLGFNGHPNIYVHWEDRFDHAAYNRDMNSA
ELAIRQCKWEIQREADDLRSIISQCSAQESPYKKQISKLKSVYERCKVEYTALCNKNRAL
TNVLNEQKKLLVVKKHDHDEALTHFRQLQESVKAQYREEFGANAQDAKSALRELITAEQQ
RVYHKLEASTIEERTKILFGIFDNPKYQQMFQMILSLGVDQDFLAYMAVKFEKMSAFEYA
LEHGATVDGYFVDDATTLERILSSGKEDYIARALQASDSVLCTALSAAQKHDLGILHQLY
DSDPGVFSKHDGVVGYSLTHFFALAGYIDALELAIQLDPDCLSVLTLAEESVFAVVLRSG
SDEVIRLIGNNIDIELALQAFIASDNDEFLIKAMSCIELSLEIKAELLESIIYENKTLLA
RELFQNPDEIKQIFAIVALSGNNILAEECFQTFMDQIGQEYLDQVLANPEISDEMRAYFE
ARNMAAEDMLADFVENEALDNLLLEFSELVGDEFAGEALMGGDLVEDGLAGDGLVGEELK
FDE

>gene_1733|GeneMark.hmm|269_aa|-|154|963 >NVVL01000083.1 Rickettsiales bacterium isolate NORP64 Contig_source1382A_26682, whole genome shotgun sequence
VVDCIQGPKGCTGEQGIPGPTGPQGIQGPLGPQGIQGQIGPQGIQGLPGTQDSSNTCAGE
WILTGEIDPPTLYTSPGAIFSLPNLVLTIGEWQSAHSSCKIIEGMPSNVTERVFDEIVVS
GTDKLSLRSGKEGLYNVSLTTISALVYNRDEVSVGNDNRFVYEIGLREDEIVNQTTPVIF
TVNSMDPVGLADQEVISKANTLGRMIVSSNTRLMVFKEGRTYGLVFRFTKAPASGTGMTK

LLIASSYMRVVATRISDIPDDYVSTLPPQ

>gene_1734|GeneMark.hmm|454_aa|-|1256|2620 >NVVL01000083.1 Rickettsiales bacterium isolate NORP64 Contig_source1382A_26682, whole genome shotgun sequence
MEGPGLKDILTGKYGDKAKIGALVEMAMNDKLPYPFIKNTIDPKLKFNNLRKFTPTILES
QYKISGHNNQNNLYHNWKGGDLVSDPENSYDDYNIILDYYTEIERSKSSASGSISSFDSW
SDRTIVKKVMTEIVKNHKKGGKMPTLRDFSDALYNQVKRPGLFRPTWLKGIFWILYKGKY
PKDLNILNMSSGWGSELMISLVLGYSYLGFDPNKKLKPGHDAMIVDFGNVKKQKVIYEPF
ENYDLGKLKFDIAVTSPPFYDKEIYSSGEQSIDTYSDFSSWMVGFLFQYISNAWYSIKVG
GYLIIHMSDIRGREINNPMNLYIEQFLKNSSWVKMVGVSFSKNAGYPVWIWQKKNGALNK
WNRKRKRKFSTLYPSLFDKIIMGYVRTLTTEPKKSTILELALADVSPDVVNYARDTVFRE
NYKVEYIVNHSKNTKTAKETIRKIALFAYYSENT

>gene_1735|GeneMark.hmm|337_aa|-|2801|3814 >NVVL01000083.1 Rickettsiales bacterium isolate NORP64 Contig_source1382A_26682, whole genome shotgun sequence
MLVLVVTLSLLSVIAISLLIILFGNISKKTDTDVSDNVTLIENERTGWGILSDLPNGLRN
ECNSYTFGALSDDEYGKFTLDVETLNRLVPTLISEDVCIDSGQIAAKQQIRECIGKDNDP
CVDSEGKVFVKGQKEILYVPCEAKRCKGTLSLISLNFDAKDPSNSSCIYDLSGTAISTKC
SLLDESQLILVERTNNKELKENDNGIYAKFLDRFNGKCIVPLDEIPVIGTKIGLGECKQN
SGYVWLLIPPTNIDGEMTSQQIVYTSTLENGPKLGNFRDFVSANELLSLNADGDIITLEK
FNTSSGQSSQILDYRLYNILNGKQSDGNVDYPYYKFS

>gene_1736|GeneMark.hmm|258_aa|+|3886|4662 >NVVL01000083.1 Rickettsiales bacterium isolate NORP64 Contig_source1382A_26682, whole genome shotgun sequence
MSGSTNTLNEFNSKVNDFVKDLIVTDVLGKLKTWLYTSQGLDVSIDDMLKGMEMPVAVKE
RKRSKTTTSKRSGGKTVTGQCDYVFKKGKFANNSCEKNIFVEGHKKCSSHMGRKSSAIQG
PMITTKPKVSRKKILPVKKYGSGGFITQGGIIVKKESGVPTVVGKLTDGKVSDLTDEDEK
HAVKLGLATKGGKIITNLSERTQIQPNKDEEDMSDEEDMSDEEDMSDEEEDRPGRPVSVS
VDSLLTEEEMSQFVSADV

>gene_1737|GeneMark.hmm|205_aa|-|4698|5315 >NVVL01000083.1 Rickettsiales bacterium isolate NORP64 Contig_source1382A_26682, whole genome shotgun sequence
MSTTKTNLSIVLDLDETLLHTITNVKSLIKMNILGNPNKLGVRERLYIINMNDGNNMWGI
YRPHLREFINFCFDYFKHVCVWSAGSYEYVHKIVHKIFSGINKPHIVYTRNDMKIVAGEM
RKPLSHLMVGCMNSKNTLILEDRYKSFSVTDPNNGLLIPAYLPSSDLLGVMEEDNHLEAL
ILWLSSDKISHCSDIREVDKISLFV

>gene_1738|GeneMark.hmm|627_aa|-|5347|7230 >NVVL01000083.1 Rickettsiales bacterium isolate NORP64 Contig_source1382A_26682, whole genome shotgun sequence
MNNPPAKVSRLSINLDDLIKSSSEIVYSYGEHYDKNFKVNQSYDVMMYKFKQNIVDRIAN
LVTLFKPLDTLLLSMGGVIPMAGINNIRTQLYKEKYADNHRKKINVNRFDVSLLCPGTHL
MRSVTEFIQTWISESAHSLPGEVILFSHLTPGSSIDKILHSVPINPKGSHIIKSSCSESI
IRAILSTSNNTVISREFVTRKRRKIQISTKYVSINNLRSLIHRSLSKRKTSLYDFSILLS
MLGNKYLPKCPSLDDSKLNNTIKGIDDLILSYNNANVSLVDVKNGNIKWDSLLILLGKLK
DKEEAMLSSQLKNRDKENSSSKISSMSVLVQSKNTTQTFSENRKLVDYNKYRNNWYSNAI
GPRSNTQSLGILNGMSVVYDGGIYGARREDVIAMCINYLNGIQWSIDYHSLGISGVSNEW
VYPKHYAPLFVDMYSVLRDVKVINEIYPKSKEAINTLHMLIMVLPMSSFSLLPVVIENKL

KEISELSYNLPVSYILDVNNGEEIHLIPKHKLREVISFMGSDDIIRVSDDFSIFNSENKE
IFVGPVKNGYYSTLNKNIIFKSKTNEKIKEDRDIDRQVKNFKNRRDSKTKKVKVKAYIPM
TYRDINSSNTKSNISRFDMESIDVPEF

>gene_1739|GeneMark.hmm|194_aa|-|7348|7932 >NVVL01000083.1 Rickettsiales bacterium isolate NORP64 Contig_source1382A_26682, whole genome shotgun sequence
MRLVFGCDILYIYALILFVTLFIMYLLVGGKKHAFVGLDENNKVTVCTKTVKVLHKETHK
NVGKIKRKYIKCSKGETECRNVLEDLFNVYFDRCRPDFLKNPETKRNLELDCFNGKLMLA
VEYNGVQHYVHPNFTNQSKEAFVKQMRRDIFKIEMCDKLHIYLITVPYTVKRKDIKEYII
SRIPRPILSKRKDI

>gene_1740|GeneMark.hmm|243_aa|-|8260|8991 >NVVL01000083.1 Rickettsiales bacterium isolate NORP64 Contig_source1382A_26682, whole genome shotgun sequence
MNFSKEEVLVVTKATKTLIVPKGIKKLVCIDIGLETLILNEGIVYVSCSHNELNELIIPS
SVTHLNCSYNEIANLMVPDGMTHLDCHGNKLTELIVPNGMTHLDCHGNKLTELIVPDSVV
RLHCHNNGLTELIVPNGVMHLICNGNGLTELIVPNGVTHLICHGNPLGDLSKYSELIKPK
LVSLYSMCYEIVDKSNIPKCFAGVDEVTCDGCKNKVIKDLVYVSYKGTVGGYIRKTIKCK
KCF

>gene_1741|GeneMark.hmm|55_aa|+|3|170 >NVVL01000084.1 Rickettsiales bacterium isolate NORP64 Contig_source1382A_26696, whole genome shotgun sequence
DNFVISEFNSVFCPTDINDFKKIKFHTLFKSIAVRAGGNSIDYSSNNNCRIGNIY

>gene_1742|GeneMark.hmm|42_aa|-|311|439 >NVVL01000084.1 Rickettsiales bacterium isolate NORP64 Contig_source1382A_26696, whole genome shotgun sequence
LLQGALLVGLEYHIFLDLSVVVFEETAIKTEEELKSLLEDIK

>gene_1743|GeneMark.hmm|83_aa|-|463|714 >NVVL01000084.1 Rickettsiales bacterium isolate NORP64 Contig_source1382A_26696, whole genome shotgun sequence
VGFQEVFRDVDLSQVDLSQLEAIGLDRFIEIAGFVDINIEGLDQIRLLHLLLENNDRDIV
LSLPLEEVANVPLALEVDAMGEV

>gene_1744|GeneMark.hmm|500_aa|-|748|2250 >NVVL01000084.1 Rickettsiales bacterium isolate NORP64 Contig_source1382A_26696, whole genome shotgun sequence
LFKAAIKRDNLEEVITAFGIDVNKSSMYQTLAFIHRFIREGEDEDLEELANDIDSFLEKA
SPEKLVSIACFVGYDIGYMPDIDEPREAIDLFLENASPKRLASIARFISHVSYIHFGRDD
RPDAASINKLRIDQLREAISLFLENASPEEIHLITSFISYDIDGLRGAIGLFLENALPEE
LDSIEKFVDGASERVLNVVDINQLREQIASLLGKATPRKLESIASCINSESEDGSSAVGF
TSDVVELRNAINLSFENATSGKLKSTIKSIFADILESKIALFLKCETPENLKSIVEFIGS
EKYSDPKFTQYFLTYSQFCLLKVAIAAGNLQQVSESIQANPSELAERQAHMLAIDAIKTN
QLEVASQFFRGAYLDLPQGLDLNLLYDKCEVSLLTMAAQRGNFAVTIQAFGIDLQKFVEY
QICAMLREWLEDLKRGNFPVRSSMKSLLAIELDPNGDYADLPGQTVETPEKVQAMLERLG
SSFEKSYNLHLYSLLHILAT

>gene_1745|GeneMark.hmm|142_aa|-|2265|2693 >NVVL01000084.1 Rickettsiales bacterium isolate NORP64 Contig_source1382A_26696, whole genome shotgun sequence

MKNLDYFLILKLNMRSIMSRTENPLELRNISRLPEELSILERSLLEDKLAAALESEGMTI
AELAQEKEEELYSLICFAIRNNHFAEIAALLKEADVDVSILYKYSLLTEDPQQDESAEQL
GQSQTMRLMTLSGMRRVEKTII

>gene_1746|GeneMark.hmm|262_aa|-|3046|3834 >NVVL01000084.1 Rickettsiales bacterium isolate NORP64 Contig_source1382A_26696, whole genome shotgun sequence
MTIRKTKNTILIKNETIGSNEIAELASLLQENPNLTTLMINESQMDAATIASFAELFKEN
NTLTTLNLNSNIGDNGAAILAEALSNNHTLTTLTFSSAGIGSAGIDHLANGLQNNYFLTN
LIFWGKQGHPEVQPITAIIDRNKAFLKNFAKMLASDADATTNNQITWSHDLINYLKLCDH
SKLSEYLIKLRVCDSNSDASNLINNYHTQIAEFQHEHANEVSDMLEECLALHAFPTDLTP
HVFSFLPDVDSGIESILGATND

>gene_1747|GeneMark.hmm|1186_aa|+|4044|7604 >NVVL01000084.1 Rickettsiales bacterium isolate NORP64 Contig_source1382A_26696, whole genome shotgun sequence
VRRELTLDSGLTKMILETYPSKKEELYNEHLQEISGASFTFRELDIIACIVHNRGEKKIA
SLLSISYRTVGSHVRNIMSKLGYSSREYIIDAVERSGKLQHVRQYYFHLVMEASFEKHLQ
KIGGVVNRSGIVCATNFSDVTEEEKKSLELFTESLTIANITLTDINLLKAEGSYEKARHN
LYIISTKSVNKTDLHQKTGDAGKAPQNESKKNIAVCFDQDIDLLTIQDLEHVDFRSKSNY
YFSVLELVGKLTGKPLAHGIIQEFKDEHQDLQKSWAGTGITGGEFSDNGSTHFMSKNAAI
LAGCAGIIIFLVYLLVWNNTPSGNNNNSNNMQVASHNQTEIWNLPKQLEHYTQRAELTKS
IWDKFRENKGSKRTKKTSILTGLYGLGGVGKTSLAKNVIHNPSSAQHYDFKGWFSAETKE
LLQADYFELGEKHYLFTKNISDKQKILRVKSWLERRGNILLVYDNAPDMSILRPYLPDNG
HIIITSRNYKLPGVIEVDVMTEKEATKLLDKLIPNKIKQDKAYDKDLKKLAKELGYLPLA
LSQAGAYIAENMLTIAGYLSLYNTERDRLLSDKTMPTMDKHAPAYITWDMSIKKLQQLKQ
EGEEALDLLDFIAYCYPENIPKKLLTQYLYKKADNETSVKLNTVLNLLRRYALIKITPHT
VSIHGLVHTWLKSKHSTNKKLTFLKRSIPIIKAIYPWENKTMEDVSLIKLILPHVTVIIS
QMQSLTAEIEYASLLSILGDSYYTLGDYAKSRQFFDKVLTINKKHFGDNSIEIARTSHDL
GKVYLKLGNYAKAKELLEKALRIKEKYYGESHIETIISLHHLGRVYLRLGKYTKAKELLE
KVLLVKEKYYGTKHINVIYSLHILGKVYLCLGNYTKAKELLERALVIKKMHYGSKHVEVA
YSLHHLGRTHFFQGDYEKAKKLLEKALIMKERYYGLKHVETARTLHYLGRVYSYSSNDMK
AKQSLEKALAIQELHYGTDHMEAAYILYHLGKVNLRLSNYVKAKELLEKALMINQTYHGS
NHNTLTINALGSMGIVYFALGEPRKGQELLGKASEMLTKLHKKESIFVANSLANIGNAYR
VLNNHHESKQLLKQSLTLLRKYYNYDKITIAKVLGNIGLLYGALGKPEKKKEYLENALLI
FKNHLQTEHPDIRKTIKYLNNSDNFAKRLNKNNNSKRLGYSIIVLP

>gene_1748|GeneMark.hmm|446_aa|+|7685|9025 >NVVL01000084.1 Rickettsiales bacterium isolate NORP64 Contig_source1382A_26696, whole genome shotgun sequence
MSASSDLEYQCSTGASHTDDGTGALRVEHQLPESKVLDLNFGGPVPIWDEAKVSEWENQE
GQILSESGSITPTTLGSEYLLSTDINSVIQFCEQLSIERQEQLKEERSKFEEDKRSFLLQ
IQRYPDVALSPLKNDSTYGLALLETEEIDLEEISKLFTKISKLLQHFNPNKAIMKALKKL
AEDERYFRLVEDYSGFALPDLSTPPLCLRTYQQEHNGFLSEKNPHTVVSRFVLLAENLST
CINAPLEENELHPLEQLIFQAAIAKLKESAAPSGVACDTFVRKNGVIHNDQGHPETHCVV
LWKVGDTVYLIDPNRHSLSKELYQPIEVILKATDGPNNHGNVEVDTQGVVYKRLQNQAIG
RGDNDARDCVDLAIKLILELVYQCSRTEETVMEAAVKQIASLGSRNAGELQGKNYKNMQT
PRNFATSDADTRHNARLLYIQNLHTN

>gene_1749|GeneMark.hmm|206_aa|-|2|619 >NVVL01000085.1 Rickettsiales bacterium isolate NORP64 Contig_source1382A_26742, whole genome shotgun sequence
MFGGLAKLSMIGALTCFVLSAAFTFLLFRMKEADAWVSFKSLFKLVLSLVTAVLFYVGIF
LFQSYWSLQEMHYSIYRQKDFNQEVWFEEERKYHPAPGRYAWIDCTRGRMYDDLHKNHLK
FLMSKKEIEDLLSHSSIKRKYGWLYKYSDCFQYMLGSCHKGPPSLLVFCSIPFTNSYRHY
LTPEKDRYTEIYEDNEERQKIINKLY

>gene_1750|GeneMark.hmm|120_aa|-|659|1021 >NVVL01000085.1 Rickettsiales bacterium isolate NORP64 Contig_source1382A_26742, whole genome shotgun sequence
VDAELMQFVEQVKTEHGIPHIDLFTGHSLGGSLATLGAIAQNSEVVAFDPAGVKRIVETR
FGLDVDESMVTNFLSKANVINGVNKQFGRVIQLRSDVGEYNTRKIHDLSFFSSMVANNGV

>gene_1751|GeneMark.hmm|285_aa|-|1033|1890 >NVVL01000085.1 Rickettsiales bacterium isolate NORP64 Contig_source1382A_26742, whole genome shotgun sequence
MKRVSLESRRIQGDNEEIFKQALEQQDVEAVRSLISKGNFNVNGSIFYQPLIEAIKLDLV
EVVGLLLGAKANPNQPSRHGNTILEEAVARGNHAMLSVLLEHGADPKQSSIFNSPLRQAI
EDGNHKAAKLLLDKGCDPNQGRAFHDKPLFIAQEVGDEDMIALLQKHGANLEISMNPPAL
VDTETYERDDGAPSLTSFPGGGVFAGGGIFAGGSITITARSFENTGTMSAGDEIKLNVES
FNDTGLINGQKVTLNLQPRNDLAAAESAEGSANLAGDSNGDDEVL

>gene_1752|GeneMark.hmm|242_aa|+|2624|3352 >NVVL01000085.1 Rickettsiales bacterium isolate NORP64 Contig_source1382A_26742, whole genome shotgun sequence
MFLFEILKSIFTKQKNQTSEIDNPHNIYLEVPYSQKEIAKNYGAKWDCKKKKWFIPQGIN
PNPFERWMPCEEENFRAKYFYLGQAYRNCYECNKATQVNAIILPEGFEAIDGEALDDLEE
LGIQPKTIPFCRQDYLSILSYLTYISSDALEEIHKYTGRAYFVKKHSKVTGYSYYRSLCQ
HCSAAQGDNFVISEFNSVFCPTDINDFKKIKFHTLFKSIAVRAGGNSIDYSSNNNCRTSN
IY

>gene_1753|GeneMark.hmm|121_aa|+|3306|3671 >NVVL01000085.1 Rickettsiales bacterium isolate NORP64 Contig_source1382A_26742, whole genome shotgun sequence
LTTVQIITVELVTYINYTAHGTIDFFECALDAQLALPHSTTEVCLNSSISRFDSLLNLLE
DVDTKWYEKVRSLWFDLDIIIAMQDGNVFSKTDLTEINQTVQELKNLVEDKVKIIPEDYM
L

>gene_1754|GeneMark.hmm|91_aa|-|3675|3950 >NVVL01000085.1 Rickettsiales bacterium isolate NORP64 Contig_source1382A_26742, whole genome shotgun sequence
LLATIGAEGFFILDITYTMFNFKIKKNLEFSLKFVKCFSIKSLLALKLEPVTPLIAGEII
VLRIFRGAPLMHSLQRPVWLPKFIFLSYFII

>gene_1755|GeneMark.hmm|1045_aa|+|3937|7074 >NVVL01000085.1 Rickettsiales bacterium isolate NORP64 Contig_source1382A_26742, whole genome shotgun sequence
MVASKSPEELLALKEAMLKRAISEGTLAEVLGEEGLTIAALYEQNEKVLYSLICFAVQEN
RFAEIAELLKNANVDVNILYKHSVLVETAKQNEYPEQSEDEGLFSEDPSNEVDSKWDVEH
EDGQEDSHITYDSVLVETAKQNEYFEQPEDEGRFSEDSSDEVDSELVEGHEGNEESEEDH
HITYANRLFKAAIETGNLPEVISAFSIDIDKSSMYQTLALIHRFIHKGDDGEVEDLKKDI
DSFLEKASPERLGLIASFVGYDTGNIPNIDEPRGAIDLFLENTSPKRLSSIASFIDHVSH
ISFGRNDRLDAASINKLRIDQLREAISSFLENASPEKLNLITRFISYDIDELRGAIGLFL

ENALPEELDSVEKFVDGASERVLNVVDINQLRENIASLLEKATPRELESIVSRIRDESED
GSRAVEFTLDVGELRNAINLSFENATPEKLKSTIKSIFADILESKIVLFLEHEEPEDLRS
IVEFVGREKYSDLKFTQWFFTYSNFCLLKVAIAAGNLQQVSESIQVNPHELPERQAHMLA
IDAIKTNQLGDAAQFFRGRDLNLLYDECEVSMLAIAAKSGNFAATIGAFGIDLQRFAEYR
ICSMIREWLEDINDTERGDFHVRSSMKSLLAIELDHEDNYADLPGQTVATQEEVRAILKR
LGSNFELSYNLHLYSLLHILATEGTLDQIRGLPGVEFHGVFRDVDLAQLEAIGLDGFIEI
AEFFDINIEELDQIRLLHLLLENNDTARALSFLGMELPENETIEMLKLKTAIAAGRLQDM
LSYVSRSYGQSDGYISRILSKQDLYSLAYFAVQENQLDGIKHLFNELRDNHCFHRESWDL
FDRKDKANLAQLFEVAIAAGNLAETMEAFKINITVLYAYKIRPVLEEWVNDVCASDSEFL
EALIYKVDYYMHELSESLSARLLRGEDLTPDDAQKIAQSFDLSSYSGGGIRALEISAERG
YLQEVLRFLNIDISTISENIQSLLLQAAGVNQLVNVAECLGVDLSALDMDTQTPLGFSLI
PMEHSERPDALSDVDITVAGDLAIL

>gene_1756|GeneMark.hmm|93_aa|+|7121|7402 >NVVL01000085.1 Rickettsiales bacterium isolate NORP64 Contig_source1382A_26742, whole genome shotgun sequence
MSNGQKKYTSEFKSQAISLVRDANLPASQVARDLNINVNTLYNWLNAANKMLNNPTDKDS
LSEIKRLKKELAQSNLERDILKKAAAYFAKSTL

>gene_1757|GeneMark.hmm|232_aa|+|7487|8185 >NVVL01000085.1 Rickettsiales bacterium isolate NORP64 Contig_source1382A_26742, whole genome shotgun sequence
MYGARKIKKRLEQCKIIISRTRIARLMSAAGLQIKTKRKYKTTTDSKHNLIISPNLLKRE
FYVAEPNSVWAGDITYISTQNGWLYLASVMDLYSRKIIGWSMASSMKASLVNDAITMAIW
QRRPAKGLIWHTDRGSQYCSKSHRQILQDHGIVQSMSRKGDCWDNATSESFFSTLKRELV
ELHNFADQQQAAAAIFEYIEVFYNKIRLHSTIGYLAPTQFEDNERVLKKSVL

>gene_1758|GeneMark.hmm|166_aa|+|8233|8733 >NVVL01000085.1 Rickettsiales bacterium isolate NORP64 Contig_source1382A_26742, whole genome shotgun sequence
VVKVGMHKVLYKKTKEQSIDRHEDGARDCVDLAVKLILELDYQYSSLQGFKELMQTIYKT
ENSTLDYGILELIYSCNRTQEDVMTVMKAASEQISSLGSDHAGKLIPLKHIPTPRDFASS
GTAARHAARNDYEEAHHLLKLICQYSGGKRNSVQVLYKLCHEALIF

>gene_1759|GeneMark.hmm|74_aa|+|8919|9140 >NVVL01000085.1 Rickettsiales bacterium isolate NORP64 Contig_source1382A_26742, whole genome shotgun sequence
MTKKRQVEPYEIITAAQTGENELLSKFVSKALEINYDSDAPKDEDERYDTLICDALWHAA
FNEQDESVKTLLNG

>gene_1760|GeneMark.hmm|267_aa|+|58|861 >NVVL01000086.1 Rickettsiales bacterium isolate NORP64 Contig_source1382A_26876, whole genome shotgun sequence
MNHTPRLNLPLIHSGQAQKEITHNEALSMLDVLINPVVQDIGIIHPPDAQEGQIFIVGTE
AQEEFKEHQNKLAQRIGQGWRFVTPSKWLEATLDSDGSKHRFNGEYWAPSSHDIRDSPQA
ISTEIDQAELKHPKVEPQLQGDMLINKDTGEYLRVGHLAEELEPQGTHIDTNIQIPRHSI
VIAITMRVTEELSGISNFSIGVADDVERYGQNLNADTDTTNIGLTNHPLAYWTDTPIRIT
ANDGSFTGGKINLTIQYLQPHGAWDWD

>gene_1761|GeneMark.hmm|87_aa|+|908|1171 >NVVL01000086.1 Rickettsiales bacterium isolate NORP64 Contig_source1382A_26876, whole genome shotgun sequence

MKLAQSFKTLTFNGIATIAAWLATNYDVDISEENQMAITTTIMAIINILLSLFTGKRLKN
KQSENHAESQNINPAPEKESIQTEQGN

>gene_1762|GeneMark.hmm|52_aa|+|1248|1406 >NVVL01000086.1 Rickettsiales bacterium isolate NORP64 Contig_source1382A_26876, whole genome shotgun sequence
MDNQQEVQKDFHYLRKLRQRAEDSGFLAMRAIISIIIGAMAVLLWEGVKILS

>gene_1763|GeneMark.hmm|140_aa|+|1438|1860 >NVVL01000086.1 Rickettsiales bacterium isolate NORP64 Contig_source1382A_26876, whole genome shotgun sequence
MINSTNQEKTTIKSPIPYFKHKELACPQTNQLILADGFAAKLLQLRIAFNQSMIITSCCR
SKAYNREVGGHPRSLHVYDKPYHNTDGTCAIDIAVTNTTTKAQLMQLAWNLGWSIGINEK
FLHLDRRTDHTNLKQMVFRY

>gene_1764|GeneMark.hmm|2077_aa|-|1884|8117 >NVVL01000086.1 Rickettsiales bacterium isolate NORP64 Contig_source1382A_26876, whole genome shotgun sequence
MSSTKWNNYELAVINRDIYKVLNFNEHNYGLEEITKLGYTIKSISGKGQNWNGFFAILLE
KNGKHIIAIQGTNASLGAPITSVGDFLADADLIAGKMPERQFSNLMEFVERVKTEYNIQH
IDLFTGHSLGGSLATLGAIAQNSEAVTFDPVGVKRIVETYFDFDVDDSMVTNFVSKANGI
NGINKQFGGAIQFQPNVGEYNVEGLDSMSVSASVPIFYPIGMPFSVPLSLLPNREHALIQ
KILIKAVKYKILFTHSIENMAKMFNKDGSIAVSYAELNSYGGNINEAIHSTVNYAQSQAF
WNHVTKRAHQNPAFKNLSFEELKAKLLNGYLLNEEIIKIVNKEISFYTQLSGTNITTDPE
YSIDMDSHLSRTQELLAPIAQKIKSGEELTDEEFYVGISVAQGYFIKKYHHSGNEDHEYN
YNFITGTGEVGKFTNAMLTRNCGTDGAPQAVRKCHYSKIMLAFRDVEASSANPNRALSLA
DHDQVRAQAFADVGVDQNADLLGFVCALSNNRSAKCGSVNEVLSELKPHINITLQADGTS
VVTPKALANFTSEIITAIAAFQETLTDFFGLTSQTRGASFVAESADSLAQSLGVDSNMID
RKYGSDDYSDAIRTSVGDTLPVFSVRSRNEEVRIPFLPGHGSEQKVVMADPEFTVRMAIS
PVSFLRPEQLETKSVLYSVVSAPSTDDSRLVVDISPEIHDRHEAKSSEELGSMWRDGVAA
YGGHHEVRMPAGILNGDGYVNVRPSSIVTGGWRPGAAQLSDFSTDSFLQQLPGAMMQESP
ECSRSVVMKSRLEFNRCRISAFDQLNIDPLVVDMSARGRSGIKLTHWQDNEVLFDIDGDG
YLEETGWIRDKTGLLVLDEDGSVTSVHQLLSEHFQGGSYSDGFEALRQFDINGDGIIDSS
DPVYHRLKVWFDLSKDGICREGELKSLTELGIKQIILNDLVEVNSERSGNLIKQKGVIVM
EDGSHRVIASVDFLANASGHKYEETEGGIRIASVGRNVRIVENKFFQKLKDEEVERETAT
FFAKEGSPTRLEADALKVDNICASSAGSVLVGGNGDNWLIGSDGADQYLGGSGNNTLVIN
EHDRQEDIQGGIGFNTVFINSVAGRYFSLDSSGIAVVYGSRAGDVLDARSTNYNCFVQAG
DGSSLMFGGSAASVLSGGAGDDIITSGSGGGILRANLGNNFLIARGGNTILESGLGFNYL
RGSNENNIFKCNAKGFSICDGSEGKFNALQLAGSIYDYSMEYNEEGHVVVRNKNYYSEFS
AVLINIHLVNFGDMRNYNFNLILPQKWVMQNDFSNLQLIDASELFSHEFGPRGQQIGFAK
VHKTEGCSIQDEQGRNLADIAEQELFFIERFSLAIDSDHKGPVFIQYTPRTRESRVCFSF
DTKDGEISNSAISLVLFRGDISEISNYYFDLLRIDYLRENNYLGQGVNVQVCEQRSDHAQ
GVWSVLKNILPEGRLYFTDQLFSAEHAKMDVLNFSISFHSPIRTNMEYNHAIGYTGGLNH
LDAAARKTVLEGRDGLGLAVIFAAGNERQDGGDANYDLIKSIPSIIVVGGISRKSVLGLG
EQPNEPFSSPGSNILTSAPANFIPAVTHGQGNKYGNYFKDSHALVQGTSFAAPIVSGIVG
AMLQVNKHLTIYDIQKILAYSSKQIESETEWQFNGASTWNGGGLHFSYDFGFGCIDAHNA
LLLAQNWLVGNRQYVERKWVFSNGTFHAKQSASFNSSATIDGEIVSTNVNFIIKEIDIFN
LKLSLAYDGQTFTILKPKVGDDLSFPTLSYAAMTPLTHFLMGMRIDSQIGCLFENTGAGD
MKFGVVNMTAGVQRDNNTSYIFTDECTKFYDEDRATLAAQNQFNTLNLAAVSYSSRVNLQ
EMVMYLAEQLYRLEGAFHNVISGSACTHITGNEENNIIVSNGSYSEVTLITGHNIVSSSS

QVNSTTKITSGDRADTFVIKRSAGSTIIIEGFKVLKFDTTPSEQMYSDVVNLAAFEEITV
TSDLILETEGSDRIICLPDNQKVVLKNVDSMRGYNFRFNKDFYIAHEFEEDFMHNNIIQN
LMFGPELSGVDAGNSILASITPEEVGHNLYDAFSGMA

>gene_1765|GeneMark.hmm|213_aa|-|8110|8751 >NVVL01000086.1 Rickettsiales bacterium isolate NORP64 Contig_source1382A_26876, whole genome shotgun sequence
MFGEFAKFSMVCAVVCFILVVAFSFSLSRMKEADSWVSFKALFKLVLFLVTIVLFFLGIF
LFRGYFSLQKMHYSIYRQKDFNQEVWFEEEKEYHPAPGVYGSLNCARGRMYDDLHKNHLK
FLMSKKEIENLLGHSTVKRKYGWLYKYSDCFQYELGFCHQGPPSLLTFCRVPFFTSYRYY
LVRQGDGGKAVYEDNEERQKIIDKLYNKGEKDE

>gene_1766|GeneMark.hmm|62_aa|+|2|190 >NVVL01000087.1 Rickettsiales bacterium isolate NORP64 Contig_source1382A_27198, whole genome shotgun sequence
FCFNTKRYNSLFSRNVSLKIPIVSAAMDTVTEYRLAIELAKLGGLGIIHKNLTNILQCIN
PL

>gene_1767|GeneMark.hmm|234_aa|+|172|876 >NVVL01000087.1 Rickettsiales bacterium isolate NORP64 Contig_source1382A_27198, whole genome shotgun sequence
MYKSSIRAFLVAFISFALSGCLPVLFTGAASSVAILAKDRSAGEAASDIKIATAIKASFV
RSNFTRLYTAIKVDVIKGRVLLTGMVEKEDDSIEAVKIVWAAKGVVEVINELKLNKKGRY
FDLIQYTRDMLTTSQIKSQTIINRDIKFINYTIITLNDVVYLFGLARSEAELASVASIAS
NVGRVQKVVSHVRIQPVAGKTKRGTKENSTKENRLLLEQKDEDLGQDEGAGDEW

>gene_1768|GeneMark.hmm|226_aa|+|866|1546 >NVVL01000087.1 Rickettsiales bacterium isolate NORP64 Contig_source1382A_27198, whole genome shotgun sequence
MSGNIRDSKFYQAAYSLCSILLVICLSLSLASCNSAKKKRLTYQTLSRDDPHNTHYKGHY
KIGKKYKIKRRGYRPREVRRYNKVGIASWYGSRYGFHGKKTANGDIYNKNLLTAAHKTLQ
LPCLVKVKNLENGKSVIVLVNDRGPYAKNRIIDLSEKAAKVIGMRKKGTAKVRVKYLHSE
SKQLLKTLRLKKKPGAKAKRSVRNKKCSVNCYVKLVNLKHRRKRIR

>gene_1769|GeneMark.hmm|91_aa|-|1598|1873 >NVVL01000087.1 Rickettsiales bacterium isolate NORP64 Contig_source1382A_27198, whole genome shotgun sequence
LLKIELALKLLTRGISWCTISLYKGINYIIMEKVSYSYTRQNLSKVLNQIGLDSEASCIE
TNYLLKSGGNAKELITGLNEANKCIGTKIDF

>gene_1770|GeneMark.hmm|300_aa|+|2080|2982 >NVVL01000087.1 Rickettsiales bacterium isolate NORP64 Contig_source1382A_27198, whole genome shotgun sequence
MSSARTDGVPTHSAENILPRLEQDLREDGLRLTPETLRKIIDNPHQFDDVLEPKMVTEFA
SMTAEQHDSLIDLLDKHPHILHTEESRDSHIVGKFKALMTKLWNKATDRVEDALDEAEDF
FDGLIAALKKLASDGVSSLGELADDAIAEHVPEGELRDALSGAADSLEDHLEDGVSKVAN
VAQNGVSKVADVVREEVSDGLDVAFQAAKGAIDDAAASMPELSLDREASVLRGEDSGNER
VTFPEGAPGAGDSELPLVFVLEGDAEPAHLSEPMALVPAPGAAADLLVAGDAVEGAADGA

>gene_1771|GeneMark.hmm|105_aa|+|3353|3670 >NVVL01000087.1 Rickettsiales bacterium isolate NORP64 Contig_source1382A_27198, whole genome shotgun sequence
MKNKSKLLATAFAVAAFSAGSVQAGGHGNMEKCHVVDSHGKGLIKAHKADCKSKHNSCSG

NNEAHDAEAWILVPKGECAKINHGDFSGVDKETKDKIETDHETDH

>gene_1772|GeneMark.hmm|279_aa|+|3786|4625 >NVVL01000087.1 Rickettsiales bacterium isolate NORP64 Contig_source1382A_27198, whole genome shotgun sequence
MSNTELGIGLRAQHLEQFVTERPSIAWVEVHSENYSTLGGLDFDLLIELRKHYPCSLHGI
GMSLGSASGINAGHLRQTKELVGAIAPFLISEHLSWNTHDKFLPDLLPIPFNDEAMEIFS
RNISVVQDALKRQILIENPSTYFSFTSSNYSEPEFLNLLAEKTGAGILLDVNNVYISGLN
NGWSAEEYINNINPLLVKEMHLSGHSVKNLAGGQALYIDSHNCRISDDVWDLYRKALAKF
GKVPTLIEWDENIPKLEVLLEEASKAQEYVNMQSGGVYA

>gene_1773|GeneMark.hmm|268_aa|+|4618|5424 >NVVL01000087.1 Rickettsiales bacterium isolate NORP64 Contig_source1382A_27198, whole genome shotgun sequence
MHNIAQIQEEMQNAILGREANLDFIEQSAGISTKERMQVHLDTVFSNLTNNLKIIYPGIW
QLIGEECASGVALAYSHDMKHLVGRERINNFGEHFPEFLRQFPSIKHLKYLPDYAQLEQL
RYKSYSAKRGKVVSLKELQQVFQSDEVESYKLTLNDSVLFLKSIYPLMSIQELLENKDSK
PLDLTETKCFLIICKTQKKLETLSAREAEWNFLRSLANSDAVGNSVESRVGNTIGKAMES
LPPENIEPELTKIIQLMLSKQMISKIYV

>gene_1774|GeneMark.hmm|165_aa|+|5417|5914 >NVVL01000087.1 Rickettsiales bacterium isolate NORP64 Contig_source1382A_27198, whole genome shotgun sequence
MFNLIHKLQITAPGRAYLAFTKFADDYLFSLLILFMRIWMAQIFWYSGLTKISSWKSTLY
LFEYEYAVPIIPSELAAILATATELSAPIFLVLGFMTRLVALPMLVMVAVIQFTYLDMRE
HLYWASFLGVMILRGPGIYSIDCIINHKTKIYKRNSGWSHLKKPK

>gene_1775|GeneMark.hmm|526_aa|-|6007|7587 >NVVL01000087.1 Rickettsiales bacterium isolate NORP64 Contig_source1382A_27198, whole genome shotgun sequence
MVERNKASKAIILSRVSSKEQEEGYSIDAQKFRLQEYCIRKGLEILKTFEFSESSTTGHR
EKFNEAINFAKEQKEIIAVVSDKVDRLQRSYKETPLLNDLIAREKIELHFYTENCIIHKY
STSQEKMTWNMFVMMAQSYVDSLRDNINRSIAQKLREGEWISKAPIGYAHIRAERKVGRS
KGKIVVDEDRAPLIKRLFEEYSTGAHTLGELVKKTKEWGLYNVAGNRGQLSLSHIHRLIQ
NPFYYGVMRVQRTGKEYPHIYQPIITKELFDRCQRVRLGWKNKPFKWYGKEYIFRGLIKC
GATGRVVTSDTKRKTYANGKTGEWTYLRVWNPDNPKKAMFVKEEKILKKVEKVFKSMHLE
SAELAKAISHIKTSSDAERSFHKNQIKELHSAHTKTKSRLDRLTDLFLDGDIDKVSYEEK
RKQLIQKRADIVREIENHNRTDDKRANYLIHTIELASRASEVFKGSTTSEKRKLINLVLS
NLELKDQKLSYTLHSPFDQFIKTAKTREWRPLRESNPCLHRERVAS

>gene_1776|GeneMark.hmm|63_aa|-|7812|8003 >NVVL01000087.1 Rickettsiales bacterium isolate NORP64 Contig_source1382A_27198, whole genome shotgun sequence
MNNATQDEIFNDIAAGIRKSSKTTMTEAESRQAARRLIEFCRIMIDVQRRIDNDKNDNNN
KVS

>gene_1777|GeneMark.hmm|81_aa|-|8024|8269 >NVVL01000087.1 Rickettsiales bacterium isolate NORP64 Contig_source1382A_27198, whole genome shotgun sequence
MKLELFMKITEMTIPTLSKFLGISQTQATQYLYKNTMPRPENMQKIHSKTIGLVAANDFY
GTTPEKLAEILLKDKKITIPK

>gene_1778|GeneMark.hmm|256_aa|+|1|771 >NVVL01000088.1 Rickettsiales bacterium isolate NORP64 Contig_source1382A_27253, whole genome shotgun sequence
INIMLKKALVTTLITTILALITSGCASDPGSPLKRSANNKLFDRKGFQGEKRAPLYNKKY
ISKAKRNILAGDFDEDDLDEDDNLYENTNISQENIEMYREMIEEDLANMKKSKKKRKKRK
IRPFPSISRANSKLTPSDHAANLELRAELQQIKALLHEARKEFSNNKCPTIADLKRESAK
LDKKAAQKPAAAPVPRSVQAGVIPPAPAPAAPPPAKQDPAMQASLKAPASLPSPSPSDAA
PSDTTTPQANNSVKAI

>gene_1779|GeneMark.hmm|27_aa|-|1228|1311 >NVVL01000088.1 Rickettsiales bacterium isolate NORP64 Contig_source1382A_27253, whole genome shotgun sequence
MVTSEFVDVIAGAGGFEVDVAGVGLVD

>gene_1780|GeneMark.hmm|209_aa|-|1379|2008 >NVVL01000088.1 Rickettsiales bacterium isolate NORP64 Contig_source1382A_27253, whole genome shotgun sequence
MGYSESYPNKEPATPFLNLDQLSALRAVFTEEVTSFKDGMTTLQNALRLFQEYMIGAGKE
DAQRQIQDYMHVLNQQMKYVEFFSEVGGAALPTHEILSSALTECREMLHANFAYQVKTVE
LEPGREVAYYEKAVDYLQQCPDSQVCKTKLAQAHVHMAKLANDDTTAAIEHYVAAIGYDK
TLAEVYEKLGQLFQQGGEYSKEASIAKQC

>gene_1781|GeneMark.hmm|365_aa|+|2203|3300 >NVVL01000088.1 Rickettsiales bacterium isolate NORP64 Contig_source1382A_27253, whole genome shotgun sequence
MAKASNSTIVQSIHPIVQSIQDGNPELFTQRLREYQQNGSFDCELTNSIISAFMESRNAK
FFSPIIENARSLGFFGSHEIISQVFDFLAKNPNIDFAISLIEETVETGVFQQIPTLFTTK
SLIKIQDQSFYEECVDASFKVLINPDIYLGTKINSSHLITLILREAISNDYFDLIAKIIS
SSVCLSNPTLILNSDTIGSIIEQIQQNGDQYLEFAHTIFIPLSKYIQENKPEQSLMELYK
NLKLLVQQSLPECTDLKTFEPNEFSTVYIVGNDSISDADLSEFANRLGLDSTLGVVVLGD
LTNVTTEGIKLLKDALGNHDAILQLSLDLDPALVERVKLISSTGEIDNFPEDELIEFHNL
LGNVE

>gene_1782|GeneMark.hmm|799_aa|-|3355|5754 >NVVL01000088.1 Rickettsiales bacterium isolate NORP64 Contig_source1382A_27253, whole genome shotgun sequence
MSGAQIALRQQNFLDSMRDLYQAGTNHYHEGKMDEAEQYFAQALKPVFSRYLMADSPFNI
EDIKEEIFAIIYRLGKTYLHFNNNSSDHYLKTAAIFQYCSAFVKKHDMDSNISGVHLSSD
DYPRYLSNIACFLEKQLLHTLCQEDITATLPRDYFYTNHPQRITRYKAGLEQVRLITKKE
LESISSIENISERAKRVEELYKNIATFFVDESNKGFIQQLLDECFDDLGQLPNGCEYAIS
AMGSLSLGTMTPWSDLEFIILINEDNEGYKEYFRKFTVLLNMKVILLGETFLAAAGIKIL
NDYKSENPSDICFFDNVTKGGFCFDGPVWYACKTPLGRQGYSNHLDYELILTPRQMFEFQ
SDSWFESDRHLVQALRSVSLIASSADGKKLFDDYRSLLRDMTAEKHCSLQARSLQLLQED
VGKFSLKITEEEEGSPLRVKQDIYRIVDRVIVALFNYYNLMPDNDQPALTIWQMVDRMVN
ETIIPYQAGQYLKEALSIAAELRLEVYSEKNAQNEMIPDSILLDTKLLHHFYCIILNMQD
VIEYVVSGQDSGELVFFDDSHYIKGQVHARFQEYEEAFREMRLAFNTMCIPQKTAPEICY
IVQNLVLLSSKIKDPKTALSMIDIQSFQHIDLRDFVTNSDAFYHLMLFEQLADLGSLFFR
CNMHAEELIIYKICWLLSNQYVYCNPDFHGTIKTNLLGMIKELANTDNHSVYAQEAIYCI
ANFQFILSKLYYSNGQYAESISRANQALQIFARLGGDLSAFDVDKIHYKLEAIYYCLEND
AELKSLGDTQTVGDDWLHE

>gene_1783|GeneMark.hmm|713_aa|-|5778|7919 >NVVL01000088.1 Rickettsiales bacterium isolate NORP64 Contig_source1382A_27253, whole genome shotgun sequence
MFMSLHNSLSDKYKLYTRYLEKITDIRFTIREIDVIACIIHNRGIQNISGLLSISARTVG
AHIYNIRAKLGYIPKDSIIDIIEKSGKLQYIQQYYLHLVAQSLFEKYLCKIAAVVNRTEI
ICNADYNNMNEQTKKLLSQLKYFLKLANITLTENKDTSGCMYNICVIDAPSTLENNHNLD
TKEFNIALLLDENVDKSTLVNLEYIDFINDHYSAILSLIAKIINKPELLQLTKKFKNEYQ
TLQSSWNGSKVQNIASENNTTSFRVTKKLKLAVAAIAICLIVTLSVFLLPKTQPNIVKLN
EQLADFAMHFSSDNISTQAKRKENYRLLSEVEKIAPLFDNQQVYTYFSSKEVPTYELMNC
LYVLHALANQYTNYQHNYKKARKLLEHIKIFAEQYVINKSSVGINFNELSKEEIYLELNI
IQDLPEIYTRIIYLLGRTYIYQGNVESSVQYFKLASYLGSKLKTFEGYLSQRSGLEVIRR
QEIDNDIENGDFAQAVKKLHAAIKSMSELREDNTAYKINYHPGHTNQTIVPKNDSYSRVY
CTEQIVKYYARLVMITSDARQNEKYTNKISSFFIGNKSLSDLMDFAEKNIPQRSAAVYIS
LGNTLLKLYEKHLSFATLKTAMSQKLKITPKTDLDFIEKLFKLAASKSRNTHYTKADSYD
GLARVYEIKLQQEEINVEQKLMLQNAIKDLRLKRDNINQKLTRMNQELRQHNL

>gene_1784|GeneMark.hmm|654_aa|-|2|1963 >NVVL01000089.1 Rickettsiales bacterium isolate NORP64 Contig_source1382A_28651, whole genome shotgun sequence
MTKATGTAIGIDLGTTYSCVGVWMNEKVEIIPNDQGNRTTPSYVAFTNDERLIGDAAKNQ
CARNPKNTIYDAKRLIGRKVGDKTVQSDMKLWPFKIEEGTAGKPQIVVQHKGEFKKFHAE
EISSMVLIKMKEIAESYLSKPIKNAVITVPAYFNDSQRQATKDAGAISGLNVLRIINEPT
AAAIAYGLDKKGAQERNVLIFDLGGGTFDVSLLTIDDGIFEVKATNGDTHLGGEDFDNRV
VEFCMEDFKKKSGIDIRGNDRALRRLRTQCEKAKRILSAALSAPIECDALAEGEDYNASI
SRAKFEELCIDLFRKCIAPVENVLKDSQLSKGQIHDVVLVGGSTRIPKVQQMIQDFFNGK
EPNRTINPDEAVAFGAAVQAAILTGEDDAQVKDLLLLDVTPLSLGIETAGGVMTTLIARN
TTIPTKKGQTFTTYADNQPGVEIKVYEGERTMTKDNNILGKFSLDGIPPAPRGVPQVEVT
FDLDANGIMNISAQDKGTQKEQKITITNDKGRLSQEEIKKMVDDAEKYKDADDLVKKKIE
SKNALENYCFSMKNTLNDEKLKDKFTEEDKTTITEGCEEGIKWLEANVEAEATEFEAKQK
ELEGKFNPIMMKVYQAAGGAPGGMPGGMPGGMPDMSGMGGMGGMGGMGGMG

>gene_1785|GeneMark.hmm|102_aa|-|2082|2390 >NVVL01000089.1 Rickettsiales bacterium isolate NORP64 Contig_source1382A_28651, whole genome shotgun sequence
LVFHNFTTRLGDRISDVLKYLFPVPKVDSKRTMTFANYSDYISFRHHVFKKDDHKTVKLK
EIGPRFELKPYQISLGTVDMVDAQKEWVLRPFMNTAKKRKAL

>gene_1786|GeneMark.hmm|37_aa|+|4122|4235 >NVVL01000089.1 Rickettsiales bacterium isolate NORP64 Contig_source1382A_28651, whole genome shotgun sequence
MKMKCMKMIIQHLQKKIRTILVIKKEEVNLLMKKLHQ

>gene_1787|GeneMark.hmm|88_aa|+|5409|5675 >NVVL01000089.1 Rickettsiales bacterium isolate NORP64 Contig_source1382A_28651, whole genome shotgun sequence
MMLLSKARLKVMLPHQSRIKKLKLQKKPVQRMIKFKMLKKIKPMLLQLLKHLIKLKMLNL
LKMNPKMNPKMNLKMNPRLSPKMSQRKM

>gene_1788|GeneMark.hmm|48_aa|+|5900|6046 >NVVL01000089.1 Rickettsiales bacterium isolate NORP64 Contig_source1382A_28651, whole genome shotgun sequence
VDAVKETAKESDVEEGKGESPAPASDKVPEASQDDIKADASGTGAAES

>gene_1789|GeneMark.hmm|68_aa|-|6194|6400 >NVVL01000089.1 Rickettsiales bacterium isolate NORP64 Contig_source1382A_28651, whole genome shotgun sequence
MTPRDLDAAVPLFPVKPAEDEDSDVRETKLHRLGYLLKYFARNFLSDKIVIFLLVMITIA
FIAIIVCS

>gene_1790|GeneMark.hmm|75_aa|-|217|444 >NVVL01000090.1 Rickettsiales bacterium isolate NORP64 Contig_source1382A_28652, whole genome shotgun sequence
MKKDIHPAYKELTISIGKDKFVTASTLKSGEILMDVDFRKHPAWNKDGAHSVNQSNKTVS
DFNKKFGGLSFGMKK

>gene_1791|GeneMark.hmm|100_aa|-|441|743 >NVVL01000090.1 Rickettsiales bacterium isolate NORP64 Contig_source1382A_28652, whole genome shotgun sequence
MSRKCELTGVGVQYGNNVSHSQRKTRRRFEPNVKSVVYSSELTGEKYRMKIVVKTMRTIE
KFGGFDAFMLGAKDSLMSIKAKKVKREIAKKQTEKTEASA

>gene_1792|GeneMark.hmm|468_aa|-|852|2258 >NVVL01000090.1 Rickettsiales bacterium isolate NORP64 Contig_source1382A_28652, whole genome shotgun sequence
MGIAFMSLPISKVAIMQNAILPDHQDRFAEQFAHITYLINNDRLGDAIDSMLDLHYADLA
DFLDNTPHKLYPVIFPAITSRLNPETIACLSDANKQPAIESIGIAKSAKLIDDLDIEDSI
EIAEALSPDLKDALLAQLSPAMRQQIIEGFLYPEDTVGRALEKDFVSFGQHWTAGQAIDF
IRRSDISADFHAAIIVSPRDKPVGTILLSTLLKSERNTPLEKIMSREFKSVDAHTALDDL
AFIFKQYALTIVPVITRQGKLLGTISIDNMLYIVEDQAESELLHLAGIHNTDIFHSLRTT
VRHRFPWLFVNLIFACLTSLVINQFSGTIAKLVTLATIMPIVASMGGSAGTQAMTVTIRA
LGSREITSSSTSRVVTKEVLSCALNGIILAFIGMMLTYLLFSDIYLSLVFAAAVVINFTL
AGLFGSSIPIMLNNMEIDPAAASGVFLTTCTDAIGFFSFLGLAYFFLV

>gene_1793|GeneMark.hmm|277_aa|-|2594|3427 >NVVL01000090.1 Rickettsiales bacterium isolate NORP64 Contig_source1382A_28652, whole genome shotgun sequence
MSNKQTNTIIILALMSFVLLVTSVFTGYRYFKWRLSYQRLAHEYSYRGEVIHPKTTQEII
DTTFSGDWMVQEIYQMLKDTTELFEKFEIKYAVESGTALGVIRHGGFIPWDDDVDIMLMQ
EDEHKLIELAPELEKLGYKILWDEDVYRIFLPNKLVHPEMSYTLPAIDINIMFENKEKNT
VEMVNWNMRKLYSKVWFPRDKFFPLKKYKFGPIEVYGPSDIEWHVKHYYGKNALKEIRMD
PKIWLGKHSNIITVPSPVPDVFFKPALPKKPLLDNVN

>gene_1794|GeneMark.hmm|291_aa|-|3467|4342 >NVVL01000090.1 Rickettsiales bacterium isolate NORP64 Contig_source1382A_28652, whole genome shotgun sequence
LYCLVIKTTTKYCIKMLKQTNIIRILSVVSFLLLVGCIISGYHYFKWKSLHQATIQEYRY
RDKLAYPKTNQEIIDVTFSNDKMVQELYQMLKDTTELFDKFEMSYAVEGDTALGIVRHGG
LLPWGNDAAIMLMQKDESKLISITSELEKLGYKIIWDKDLYRIFFKSKKGYVGRRYTLPV
INVSIMFENKKKNIVEPLNWKIKKALPTNWLPKDKFFPLKKYKFGAIEVYGANNIEWYVG
HRYGKSVLKELKIEPKFWLGKHSNVITVPSPAPDVFFKPALPKEALLDRVQ

>gene_1795|GeneMark.hmm|168_aa|+|4439|4945 >NVVL01000090.1 Rickettsiales bacterium isolate NORP64 Contig_source1382A_28652, whole genome shotgun sequence
MPQKTIGIYPGTFDPITNGHADIIARASKITDELVLAISESTVKNPMFSIEERCEMARIY

LDKYNIASNVKVMPFEGLLAKFASSVGAQFIVRGLRAVSDFEYEFQMACMNARMDSTIET
IFLPASEKNQFISSRLVKEIASLGGNVSEFVIPEIEEKLIKYYNISNE

>gene_1796|GeneMark.hmm|365_aa|+|4938|6035 >NVVL01000090.1 Rickettsiales bacterium isolate NORP64 Contig_source1382A_28652, whole genome shotgun sequence
MNNAKCSLQINLQTIKENYIALRKLCIGSEVGAAVKANSYGLGASRIAPVLQESGCKHFF
VATCEEGVDLRQTLRGDANIYVLNGVFQNELEYFINHNLTPVLNNPEQVEIWQHYAARHG
RRLPCFIHIDTGMHRLGFSSAEASELELDLLRNNLDILCVMSHLASAEEVDNPFNKTQLE
RFTQRSAKFGDIKRSLANSSGIFLGRDYHFNLARPGAALYGINPAPYLENLTLKNPVKLS
APIIQLRDLPPGESVGYNSTHTNRRGDSCSIATIAIGYADGLHRALSNKGVLYINGFEAP
IIGRVSMDLTAIDLSNIPKNDVFLGQMVEVIGENSSPDELAKFCGTNGYEILATLGKRFK
KIYTG

>gene_1797|GeneMark.hmm|241_aa|+|6129|6851 >NVVL01000090.1 Rickettsiales bacterium isolate NORP64 Contig_source1382A_28652, whole genome shotgun sequence
MLSSVRYIGNRSINGAQRLGVLTMFIAEVMAGMAKRPLYLKLIGRQLITIGYFSLPIVAM
TALFSGAVLALQSYTGFSRFSAESSIATVVVLSITRELGPVLAGLMVAGRVGASIAAEIA
TMRVTEQIDALYTLSTNPIKYLVVPRVIAAVVTLPCLVLIGDVIGVMGGYLVSVYKLGFN
GTNYLINTATYLESIDVISGLVKAAVFGFIISIMSCYHGYHSGKGARGVGAETTGAVVSS
S

>gene_1798|GeneMark.hmm|90_aa|+|147|419 >NVVL01000091.1 Rickettsiales bacterium isolate NORP64 Contig_source1382A_28992, whole genome shotgun sequence
MTALSINLPDSLTKESTRFAKELGMSRTEFIKRAIIHELADLKKKQEEAKIINSFAAMKK
SKTYIKQTIQTIEEFEDILPSEQVQWWKKK

>gene_1799|GeneMark.hmm|430_aa|+|871|2163 >NVVL01000091.1 Rickettsiales bacterium isolate NORP64 Contig_source1382A_28992, whole genome shotgun sequence
MIITKRKFFIVWLLGFMSGFTLMISGNTLNFWLAKENIDLQTIGVFALISIPYAINFTWA
PIFDTVKLPILNKLFGKRLSWLLLIQILLSSAIYVMSTLSPTYALELLAICGFVIAFLAS
AQDTILGAMRTEMVDKNRQGEIAGMYVFGYRIGMLASSSGAIYISQYVHWGLVYELFSVI
TIAFPLILLLFTNDLLPIKQEFTPKVEDPNTKPALQKSALHRKMIDFFSEILKPVGKPKY
IIFVLIFLALYRLPDNFISMMINPFLLHIGYDEFEISTAGKLFGTSAAMIGGFLASYIMK
KKNLYDSLLIFGSVHAIAHLFFVLQELYGKNLYMLFVVIGFEGVSGGMSMAAYIAFIASL
CQGKFRATQYSFFSSMMGFSRSIFPALSGYIVSSFGWTVFYLFTTIATLPSLMMILYLEK
LQKQNSSEEK

>gene_1800|GeneMark.hmm|459_aa|+|2160|3539 >NVVL01000091.1 Rickettsiales bacterium isolate NORP64 Contig_source1382A_28992, whole genome shotgun sequence
MIDIIIVFSYLMILLFIGVYQRSKLAGFAGFARIGGKLQKNRLILVATIFVSSIGGGTTF
GISEKAFAENIAYSYGLFFAIPVDILIAIYIIPRLAKHYGAETIGDIMEVYYGKPGRYIA
GFSAILVSVGLVAAQISVSGRIFEYILQIEYVWSVVFSYAIIVIYTTIGGFRSVLFANLL
QFFAILIAIPMISIFGIYQIGLDNFTQLVPASKVSFFDNPDLLSTTIAATLGFAVMNMFP
TFIQRALINKDAKATSRAIYIKSSIYAIFLVFITLNGLIAFIKYPDLEPSLALPYLIDHM
IPTGLQGLVVVGLLAAVMSTADSDLNITSITIVKDFLSPIFSVQNQDKMLMLARVINIIV
GSFAIIIALCFTRVVDLVIFIAGFWSPVILVPLVLALFGIVISKRGMICSCFSGVASFIL

WENYCASEHCFKGVFVGVMMHLFVFSIWRIYTKRNSVIS

>gene_1801|GeneMark.hmm|175_aa|-|3532|4059 >NVVL01000091.1 Rickettsiales bacterium isolate NORP64 Contig_source1382A_28992, whole genome shotgun sequence
MSGSDLELNKMAAAILVASLIAMIVGVVANALYKPKLEVAQRGYQVEVAEEGTVAEATKA
SAEIVDIKILMASANAEAGRKIFKKCVSCHTVNKNGSNKVGPNLWNIVNAQKGEKPGFAY
SKAMLASGGSWDEENMFAFLTKPSKYLPGTKMSFRGIKKPTQVADVIAYLQKEAS

>gene_1802|GeneMark.hmm|341_aa|-|4091|5116 >NVVL01000091.1 Rickettsiales bacterium isolate NORP64 Contig_source1382A_28992, whole genome shotgun sequence
LSIKILGLESSCDDSAAAIIDSKLNILSNIVISQNQEHELFKGVVPEIAARSHLANLETA
LQRSLTEASLTLGDIDVIAATAGPGLIGGVIVGSMYGKALASVLNKPFIAVNHLEGHALT
ARLTDKIEFPYLLLLASGGHCQFVAVMGMGKYKILGQTLDDAIGEAFDKVAKMMDLGFPG
GPAVEKMAKDGDSARFDFPRPLFSRQNCDMSFSGLKTAVRLQIEKLLPLKKQDIHDIAAS
FQQTVCDIITRKTEYAIREYEKLCTSKQIVISGGVAANNNIRSALTELSASLGYNFVAPP
TKLCTDNAAMIAFTGLERFKAGTINGMDFKPRARWSLEEVD

>gene_1803|GeneMark.hmm|394_aa|-|5125|6309 >NVVL01000091.1 Rickettsiales bacterium isolate NORP64 Contig_source1382A_28992, whole genome shotgun sequence
MKKYYSLFIAIILNVSLSYADSVLRKNVNDATSEYLSTHFMNGVYMFCDENGLITQGAKG
FYSLENKKKLSTSQLMPIASATKTMTAASIMKLRDKGLLNMKDTVAKHLSAESGIWKEGK
LADWASKITLHNLLTHRSGLPEYFMKVKVDLTKTHDEINKDILHFLASNELKFKPEAKYQ
YSNSNYVLLGLIIEQVSGQKLSEFMQQEIFTPLDMKDTKIPTLSEALEHQTTPESTIYPS
RYFVTPTGKKPMFNKAMSKYPMMPFSDGGVTSTTKDMTKWLKALHSGKVVSADSYKLMKT
RHYEIKHRTGVKNYMGYGMYIAELKNGDIAYHHPGNAVAIRSESGYIPAKNLYFTVLSNV
MDYIPPQMKGKIDKTKSVNQLDIRYFMQCIFNTI

>gene_1804|GeneMark.hmm|240_aa|-|2|721 >NVVL01000092.1 Rickettsiales bacterium isolate NORP64 Contig_source1382A_29265, whole genome shotgun sequence
MCDFCVSGKIETYEQLVSGSAEKKEVFKLFDRHIIHFDNVDLCVIDDIKNPTIRDVITKL
YPEGWKDIFNDNLYELKTICDYISSEAKETQIFPHFEDVFKVFDMCSPEDIKVIIIGQDP
YPRLGKLGLPLGTGLSFSVRVGENIPRSLSNIYNELDRSIDSFVKPKHGNLENWVNQGVF
LLNKSLTVENGKVNSHKRVWNNFTSNIIKYLSAFDEDIIYVLWGKDAQEMNKTYTKGVIF

>gene_1805|GeneMark.hmm|536_aa|-|750|2360 >NVVL01000092.1 Rickettsiales bacterium isolate NORP64 Contig_source1382A_29265, whole genome shotgun sequence
MDVLECNVNKITQMVGIPVVIKPVDAKRGNVLFIGTNDGHIFYVDESSKKKTLLDLRNVI
KTPSFCKMGLLGLAFHEDFDKNGKFYVHYSLDTSNGISNGIDVEDVDVNNLSSLFQEWSD
INEYNHIDVVEEYIQDCFCDNPKCVAEKSKTLLKIRQPFENNNGSDSLLYKEGKLLLTLG
DGGSSYDPFNLSQNNNSLHGKIISIDETIVWDETVSIGNIYELDETQCDKVTILSKGIQS
PSSLDILDDMMYLVDNGKVVNELNVFKKFGNNFGWRPFEGILPTLHMSNIKNISPTYNSG
INPVDFKVDEVVWDVECDKGGVIHLDIAPGDYLHFKSSNPCNVIETFSNYENKKDPIIDS
EISIINESILLECTGDFYFKSFVPGGDNTSLSVSVRNDILRKRKVNSIKLENYKDKLKSS
KEVPIFTVAYYNESLSLAPKLKQQCDTYILPSENRISSGKYYKGELGHIKDKFVFIDLLK
GIYTFSLCEPHIQKINVKIEGDFYTSLGVNDKRDTLYIGTSTILDSKAIGTVYSLK

>gene_1806|GeneMark.hmm|507_aa|-|2366|3889 >NVVL01000092.1 Rickettsiales bacterium isolate NORP64 Contig_source1382A_29265, whole genome shotgun sequence
MDRIGIVFVICTFIIISIFYIKIFSKRHLGKIVVQMGKCKSNSLNCRDLGTRTITSTCVS
DNQNDTCGNGMFVSDTITTSETCEPTCRKSFWLDHEVGKCTVKDNLTGCVDLGSVGNRTR
NVECVRGDDFGINTCTSLELLEHTGPLGLGKIHKIKLYNIGDVVSMEEECTNYTNKICGK
WKTNKNNIISPCNLGKQLSVHKNCITGVSKPFDSLVEGYVEEDMLCTKTIGNVEVIRGND
VCHLVKELISCSSGTTDPVDIRNGTLPLDFTPTICRPTDDIPKCVRVCRMYPNKINVGIF
SDITGAMFTLRIKNQGYFTAENLPIDVDGKLEDTKIVLIDDGTGISSLCNSKSLKYNTEY
NTSIMFCLGVRRYDGNTIKAQISCIIGGSYVGWLSVNKSNEIIWEQAYNIYGAPGSTTTD
AYMFTIKNYVKENDLGSNHMGFGNNSTYKGMCGIDLKSNTGGNLYIKDVRGKAHELNHIV
ALIINIESVLNRSDIFTECNLLYKNNN

>gene_1807|GeneMark.hmm|78_aa|-|3946|4182 >NVVL01000092.1 Rickettsiales bacterium isolate NORP64 Contig_source1382A_29265, whole genome shotgun sequence
MRGWKISHVLYFLAIIAALVGVILGVGVGIKNGSDLSENDTHNITRANLFFLASVAIGII
AMVQMSYDKHGHHRLVTA

>gene_1808|GeneMark.hmm|169_aa|-|4204|4713 >NVVL01000092.1 Rickettsiales bacterium isolate NORP64 Contig_source1382A_29265, whole genome shotgun sequence
MGACNIKSAFQSEQSRVTMIYENAVTLDTTSAYNIARSDIESILNSTSYTNLEVPLGDYP
TGMDRLVVVFPVVDSVVQKIVTIQILYKCDDTTNTHHIFHMTLLPGPNHEAITLIPHRNS
FEETKADDNLHDHTSLNTVSTLKRDVNTCYRPPKCKKYCSGCHKYHSNK

>gene_1809|GeneMark.hmm|205_aa|-|4725|5342 >NVVL01000092.1 Rickettsiales bacterium isolate NORP64 Contig_source1382A_29265, whole genome shotgun sequence
VKFGTTGKRKFRKTRKMTHTNIKMRKYINIKWEREKQYRAGVLPYYKSEKGIGSQKEVIF
IMGVDSMYGNYTDFGGAKKLGRNNKRIEKSNETAFRELEEETLGCIKRKDLDLFNALVVS
SDQMLIVLAEVKISDIQDRNNTKKIKHIFNSFDLKLANEGSPEVSSLCEIPKDLFIYSML
SDKSTVYPPVKNLLSGKITHISKIK

>gene_1810|GeneMark.hmm|58_aa|-|5344|5520 >NVVL01000092.1 Rickettsiales bacterium isolate NORP64 Contig_source1382A_29265, whole genome shotgun sequence
MKKITSVKEATSQETLFYILLGENYARIQGVPKEEIDTRSIGATLWHFVIKKQFEKNS

>gene_1811|GeneMark.hmm|133_aa|+|5873|6274 >NVVL01000092.1 Rickettsiales bacterium isolate NORP64 Contig_source1382A_29265, whole genome shotgun sequence
MPNEFKEMFGDSALVEWTSYGDCGPDVPLLVAEAGSDVAFGYKCGTGLTNPVLLSDCTIK
VSNGSGKFAIKYLADFAGWVGMIITAKCKGCDGVSTSRFFTLDGLPHTKHNRDFLCCKDS
PYGTELDLPIVVS

>gene_1812|GeneMark.hmm|126_aa|-|252|632 >NVVL01000093.1 Rickettsiales bacterium isolate NORP64 Contig_source1382A_29274, whole genome shotgun sequence
MIKHIVGMSIYQCIIIFTITLAGEYIIPEDPDYIVKNLDNPGFVHPGRLYKWNGDDLYNV
LLPIHGPSRNLTMVFNTFVFLQIFNMINARKINDEINPFADIFKNKMFIGIWLIIFLLQI
VLTQFT

>gene_1813|GeneMark.hmm|136_aa|-|663|1073 >NVVL01000093.1 Rickettsiales bacterium isolate NORP64 Contig_source1382A_29274, whole genome shotgun sequence
MVTGLKQFGDTVAVTGDGTNDAPALKKADVGFAMGIAGTDVAKHAADIILLDDNFASIVR
AVVWGRNIYDNIRRFLQFQLTVNVVALVVSFIGAAIMRESPLQAIQLLWVNLIMDSLASL
ALATEPPKDTLLDRPP

>gene_1814|GeneMark.hmm|44_aa|-|1176|1310 >NVVL01000093.1 Rickettsiales bacterium isolate NORP64 Contig_source1382A_29274, whole genome shotgun sequence
MVTGDNKITALAIAKECKIVEEGDNMEMAVMEGKDFYEYVGGIV

>gene_1815|GeneMark.hmm|49_aa|-|1329|1478 >NVVL01000093.1 Rickettsiales bacterium isolate NORP64 Contig_source1382A_29274, whole genome shotgun sequence
MGEGGATHEDDDVEGVQKVVEKDGLVCLALLGIRDIIRKEVPGAVAKCR

>gene_1816|GeneMark.hmm|132_aa|-|1479|1877 >NVVL01000093.1 Rickettsiales bacterium isolate NORP64 Contig_source1382A_29274, whole genome shotgun sequence
MFESISCNTVGTVDDSSATEKAMLKMLKKFSVNVEDSRATHLGESFVRFPFTSKRKRMSS
VASNISEQRYGYDKRLHIKGAAEIILACCSHYIDDNGAEQEMTASIKDGVLGVIEFFGTQ
ALRCICVAYKDI

>gene_1817|GeneMark.hmm|119_aa|-|1956|2315 >NVVL01000093.1 Rickettsiales bacterium isolate NORP64 Contig_source1382A_29274, whole genome shotgun sequence
MLFAALTVLVLFIRFFIETDYTDFGDKFGTYLGDWFGFLIIGITIIVVAVPEGLPLAVMI
SLAYSVRKMLAEKNFVKKLASCEIMGGANNICSDKTGTLTMNEMSVTNLWNGATQGTPD

>gene_1818|GeneMark.hmm|72_aa|-|2457|2675 >NVVL01000093.1 Rickettsiales bacterium isolate NORP64 Contig_source1382A_29274, whole genome shotgun sequence
MPVDGVCVRSRGDIMTDESAMTGESVELKKHTLDGCFRRKEERLKELEYSKAAQLDSHSF
PSPIIISGTQVQ

>gene_1819|GeneMark.hmm|75_aa|-|2943|3170 >NVVL01000093.1 Rickettsiales bacterium isolate NORP64 Contig_source1382A_29274, whole genome shotgun sequence
MQLTSKRGMAIMATTISLLHREPVRTKDVLHIAFCKFFCAAMNDLMLKVLIVCAIISIVV
SMIFEADHRSTGKFE

>gene_1820|GeneMark.hmm|54_aa|+|4633|4797 >NVVL01000093.1 Rickettsiales bacterium isolate NORP64 Contig_source1382A_29274, whole genome shotgun sequence
MQELEKTHQQFDSSQVASVKTSEISSPTVDSGVVPKIVNSNFHVEPKKADTLGS

>gene_1821|GeneMark.hmm|40_aa|+|5542|5664 >NVVL01000093.1 Rickettsiales bacterium isolate NORP64 Contig_source1382A_29274, whole genome shotgun sequence
MGDGVACRGGVMAEPEIFEFTITKADKMMIIASDGVWEFL

>gene_1822|GeneMark.hmm|46_aa|+|5713|5853 >NVVL01000093.1 Rickettsiales bacterium isolate NORP64 Contig_source1382A_29274, whole genome shotgun sequence
VYPFFEKRNAEGAAEALVRESFKAWRREEDVIDDITCIVIFLDVKM

>gene_1823|GeneMark.hmm|967_aa|+|329|3232 >NVVL01000094.1 Rickettsiales bacterium isolate NORP64 Contig_source1382A_29280, whole genome shotgun sequence
MFGAQISLDMIIVIGFLVLTLIVGMGHGKNVKTIKDYALGGRNFSTAALVATIVATWASG
SSFFITMSMTYSDGLYDTFAVIGGGISFLIEAFFIIPRMGEFLGKVSIAEAMGDLYGKKV
RVITAIAGTIGAAGIIAVQFKVFGNIFSYFLHIPAYVAIVAAGFIATLYSAFGGIRAVTF
TDILQFLAFGIIIPVVGFIIWNAFYAEGLSFDQAITDPKFNLGILLDTSNPGFVGMIFMF
CYFCVPTMSAPAFQRVAIGRDVAQVKKAFIIAGLIFIVIQIVSAWIPFLIYSINPSLETS
HLLGYIVDTYAYPGLKGLIIVAIIALSMSTADSRINAASVLFTNDICKVFVAKLKRELLI
TRSFAFLLGAISIVLALIETDLIGVVVFANSFYYPIVTPPFLLTVFGFRSTSKSVLIGMA
AGFATVIIWKMLPISFATISQKMVGVLFAMLVNVIFLIASHYLLRQEGGWVGVKDTSYID
KQRALRKKKWAVFMQKFQNFRLVKAITTAPLPSETRCVMTGLYFIFFTVTIAYSTDIDTL
KDNGNLVLIIYQIMIATGTVMAIYPIWPMNVKQKTKNMIIRIFYPIAMFYMMVFFSTFFV
LLSDTNTLHYMIFAANIITLIVVMSWQAGTVMVIVGAYLAIQFYKYYMEIEHLDSSIGSP
QFILIYSIVFIGSALMMFFKPKQEDLEQREREISMLSGKVGSLKTKIGTLDNELLGMSEQ
VTHYSQRASDQALEIERLGAISQKILNNVNHELRLPVGNVMNFAEMMRDGLGKLPDKQLR
MISDEVYDNSNRLSTMILNMLDLATLDANKIQLQKREVNFSDLVVERLQICRKIYVGEKL
LKFKLNIDPMVVLSIDVNYIRQMVDNLIINSINFSKIGLITIIVNRDGKFANFIIQDQGI
GIPITEIYDVFTPFKMGSNTEGKAEGRGVGLALCKAAIEAHDGTIQVESRGVGVVFRVKL
PFTQEQR

>gene_1824|GeneMark.hmm|135_aa|-|3253|3660 >NVVL01000094.1 Rickettsiales bacterium isolate NORP64 Contig_source1382A_29280, whole genome shotgun sequence
MGVVIDTSVLIAAERQEIDFNKWQKYDAAYISSITITELLIGVHRAKSSKIRVKRSAFVE
HIISQITALVFGEEEARVYAQILDDLYKKGITLGTHDMIIGASAIVNGYPVLTMNEKDFK
RIPGLEVLSLSKEIR

>gene_1825|GeneMark.hmm|92_aa|-|3651|3929 >NVVL01000094.1 Rickettsiales bacterium isolate NORP64 Contig_source1382A_29280, whole genome shotgun sequence
MTKLITVTDMIRSFSDIVARVYYKGEIFDIKKGHNIVARLSPAKTRATIGISELNNFFKE
APHLDSDDAEDFENTLKETRLLKDSGASMGWE

>gene_1826|GeneMark.hmm|649_aa|-|4044|5993 >NVVL01000094.1 Rickettsiales bacterium isolate NORP64 Contig_source1382A_29280, whole genome shotgun sequence
MHQIIKVLLPQVCLFPLDYSSDTDHNIGDLIVVPFRNKELTGIVWEIGCADSGKALKNIC
SPPLFPASINPPMIELITRASSYYLADLGTIAKLVLPVNINESPLKIKTHDEQASTPRAL
PPLTPSQQEALGIVKASILPVVIKGVTGSGKTELYFHAIQEQLALGKQALIMLPEIALGS
QIIARFTERFGFEPAIWNSKVTKAQKKQILRGIISGSVKIVIGARSSLFLPYKELGMIVV
DEEHDSSYKQGEGILYHARDMAVLRGAVEGCKILLGSATPSIESLYNVQIGKYQLATLND
RWSGAFLPDVKIVDMRNESLGHNCYLSEEATLAIGKNLAAKNQTLIFLNRRGYAPLMLCK
ACGYRFDCKSCSASMVVHKSSGRMECHHCGAVSRVHDKCPDCQEENTLTLCGAGIERVAE
EVSNLFPNSNTSLVSKEEFSCLEKMKELLSRMESGEIDILIGTQIVTKGYHFPNLTLVVV
ANSDIGFMGGDLRTAERTYQLLHQVGGRAGRADKKGSVLLQTYYPEHKVITAIASGTEDE
FIRQEIASREQAKVPPFAKMAAITITGKNPARTLIAAKDFVAKAPKSTAQILGPAEAAML
KLSGRYRYRILVIADKKFNLQKYLTLWREHAALRSTYQLKIDIDPQSTI

>gene_1827|GeneMark.hmm|118_aa|-|6037|6393 >NVVL01000094.1 Rickettsiales bacterium isolate NORP64 Contig_source1382A_29280, whole genome shotgun sequence
MKNSEVKEDFKSPYMARVAKRLRVLRMTDEERIEYHKYLKESAVQEDILHAATERGREEG
VEEGIEKGREEGLSEGAKLAKIEVVKTMLMQGLDVNLISQATKLSIDEVKALRSHKVT

>gene_1828|GeneMark.hmm|27_aa|-|3|83 >NVVL01000095.1 Rickettsiales bacterium isolate NORP64 Contig_source1382A_29500, whole genome shotgun sequence
MDLATYYLRIQMINLFYDNNKLGFWGF

>gene_1829|GeneMark.hmm|213_aa|-|250|891 >NVVL01000095.1 Rickettsiales bacterium isolate NORP64 Contig_source1382A_29500, whole genome shotgun sequence
MPTAGSASKYEVAGGGDKVTVEVADGDKDDKGEKDAEGADTKKGDDKGEEGTPDEKKLKK
SETNHYGGILDETEKEVTVVGPGACNAFLWKHRVCNPFATADPIFRRFLRAIFFGGQFWF
IFCVSGLFVWNDVFVSENEDGVPMPLEGGFKFLIVLIIFVLSRFFLWMVEGLNRLDAVSF
LMRGLVTFVTVITIGKSCYFNLKVFATMESFTA

>gene_1830|GeneMark.hmm|168_aa|-|1174|1680 >NVVL01000095.1 Rickettsiales bacterium isolate NORP64 Contig_source1382A_29500, whole genome shotgun sequence
MSQIAQSSCANAELVEGSSCTAFESTSRTVSSFLLDRSTVGETLINVKSPNLVAIADSGP
IENARRNLEGEQLQVILSENSIPAECDSPILTVIAVGNVISLLDKTLFDIEIYGKNYGPK
GVGVQASLSCIEFENSQYVITPSSIVDVTTIKLATLPTIDNYYTASAA

>gene_1831|GeneMark.hmm|63_aa|-|2232|2423 >NVVL01000095.1 Rickettsiales bacterium isolate NORP64 Contig_source1382A_29500, whole genome shotgun sequence
LLEILQEEQHKFTLHHKLQLTLVSAQSQENPGKQDSRLMLDTLSQRLIHVFMGIIQNLVS
KII

>gene_1832|GeneMark.hmm|102_aa|-|3268|3576 >NVVL01000095.1 Rickettsiales bacterium isolate NORP64 Contig_source1382A_29500, whole genome shotgun sequence
MTELNDVTLTDNINTVLGNAANISSVSLDSGISTSLSGRDVQFGAYTVDYLGRKVLASKL
VTFSNNSSPIVYIDAAYFVSNTQDFTLTPRVTPSECSGAKTQ

>gene_1833|GeneMark.hmm|53_aa|-|4233|4394 >NVVL01000095.1 Rickettsiales bacterium isolate NORP64 Contig_source1382A_29500, whole genome shotgun sequence
VKFATQITTWDKMASVMQHVLVITMVWYRLQDLSIVQTVQANAQHASMLVVHP

>gene_1834|GeneMark.hmm|32_aa|-|4416|4514 >NVVL01000095.1 Rickettsiales bacterium isolate NORP64 Contig_source1382A_29500, whole genome shotgun sequence
MKPIQLPRKLALHALPIPTSQNIAQLGAQAIV

>gene_1835|GeneMark.hmm|135_aa|-|4935|5342 >NVVL01000095.1 Rickettsiales bacterium isolate NORP64 Contig_source1382A_29500, whole genome shotgun sequence
LLRLEHAMLTIAQMAFMQMVKTVKPVLQHVKPVLPLAQLNAQNALRNIQMLTEIVFLKSN
AATFHKNSPIEIEFVLISPHVAPTKCLIYQGTYVLIHVTMDGSKVLIFVRFVTLHVDNVL
DQTLTNALAVTTTMP

>gene_1836|GeneMark.hmm|58_aa|-|5343|5519 >NVVL01000095.1 Rickettsiales bacterium isolate NORP64 Contig_source1382A_29500, whole genome shotgun sequence
VIFAQMDILDELTEPVPLLAHQELMTLTVKYVKDVMAIVKLARKTQLSAFSAVMLTNS

>gene_1837|GeneMark.hmm|27_aa|-|5676|5759 >NVVL01000095.1 Rickettsiales bacterium isolate NORP64 Contig_source1382A_29500, whole genome shotgun sequence
MVVQLVLEEGTVNALLAPLYGTLQISY

>gene_1838|GeneMark.hmm|63_aa|+|1|192 >NVVL01000096.1 Rickettsiales bacterium isolate NORP64 Contig_source1382A_29577, whole genome shotgun sequence
VLLPSLFSLLFYATFSLNVARYQMTALPGFSIAFAFFAVWVRGKAHNWSSGRRWTSAKEK
KNA

>gene_1839|GeneMark.hmm|332_aa|+|185|1183 >NVVL01000096.1 Rickettsiales bacterium isolate NORP64 Contig_source1382A_29577, whole genome shotgun sequence
MPNDNMIQPTAAYNHARIAILLPCYNEGKAISHVIASFRSIVPSAQIVVIDNASNDDTAE
KAMAADAIVIREDLPGKGNAVRRGFSEVEADIYVMADGDGTYDARALPQLINKLVEDRLD
MVVGTRAEKEQAAYRSGHRIGNRLFNTVFAKLFGTRFTDIFSGYRVFSRRFVKTFPAIST
GFEIETEISVHAIMLRAPVAEIPTSYGARHEGTESKLRTYRDGIRILSKILLFTATHKPF
VFYGLIAASLAILSVLLSIPLILEFLETGAVYRLPTAVLTVGIMISAILSFVTGAILSNI
GIGQWEVKRLSYLMHTDRLDAIRAALTVLREK

>gene_1840|GeneMark.hmm|132_aa|+|1180|1578 >NVVL01000096.1 Rickettsiales bacterium isolate NORP64 Contig_source1382A_29577, whole genome shotgun sequence
MINRSLIDKALRFAIVGSLGFLTDAGLLWVATNKFGLDPYSARLVSFSTALLTTWLLNSQ
FTFKSPKRRTKSQFTGYVVVQVTSFFLNYALYSLLVWQGVATPLVSLFFAAIISMFYSFT
TVNIWVFGDRRQ

>gene_1841|GeneMark.hmm|102_aa|-|1624|1932 >NVVL01000096.1 Rickettsiales bacterium isolate NORP64 Contig_source1382A_29577, whole genome shotgun sequence
LGPLNSAHRISAVQSIIGEELMTEVGGIAADQLASYVERIERLEEEKANLMADIKEVYGE
AKALGYDVKILRQIIRLRKMEDHERTEQEEILEVYKRALGMS

>gene_1842|GeneMark.hmm|389_aa|-|2248|3417 >NVVL01000096.1 Rickettsiales bacterium isolate NORP64 Contig_source1382A_29577, whole genome shotgun sequence
LAGASAREPGFSIGIEEEFWLVDRETRDLAKDPPAEFLKDAKAKLGDQVSSEFMRCQVET
GSQVCNSALEAREQLIHMRSTLSEVAAKYDMALLAASTHPFAQWDDQKHTDGERYNTIAR
DLRTVVDRLLICGMHVHVGIEDDDLRIELMAQASYFLPHLLALTTSSPFWRGKETGLQTF
RLSVFDNLPRTGLPEVFGSWAEYRRHVDMLINAGVIEDGTKLWWDLRPSDRWPTLEMRIA
DVCTDLEDAVCVASITRCLMRMLYRLRRNNQRWRIYANMLVRENRWRACRYGSDPGEKAG
LIDFGRGEIVSYADLLEEILELIRPDAEFFGCVDEVEHARTIVKRGTSARRQREIYDGAI
ELGATPKDALVAVADVLIERTDPHARSET

>gene_1843|GeneMark.hmm|258_aa|-|3419|4195 >NVVL01000096.1 Rickettsiales bacterium isolate NORP64 Contig_source1382A_29577, whole genome shotgun sequence
LGHDDPAPFEILNENGKGHSLFVCDHASREVPKSLGTLGLPDEERKRHIGWDIGARKVTE

ILVERFDCPAVLGGYSRLVIDCNRQLWDPTGMPEIADQTVVPGNKNVSPSEHMKRIREIF
LPYHWAIEEQIATFHAHKIAPALIAIHSFTPVFQGFERPWEIGVLWDQDDRIPVPLLETL
RAQNPDLTIGDNEPYSGQHLADYTIDHHGEAAGLPCVSIEIRQDLIDTDEGCEKWAKILG
DAFVDILADPNLYKIRRD

>gene_1844|GeneMark.hmm|249_aa|+|4515|5264 >NVVL01000096.1 Rickettsiales bacterium isolate NORP64 Contig_source1382A_29577, whole genome shotgun sequence
MPSAKTLQAQDDPETRRPKRVADQVADQILEKISDGIFLPGDRLPGERQLAESMSVSRVS
VRAALQKLKTRGYLRAVQGGGTEILSSTGIDMDTAMTDLVRDSISNLRDLQEIRCTLETW
AARRAAINASDEDIKLLRMAFRQAHDPRRAAKHRADDDHRLHMMIGRATGSIIYYHVMKM
LHDVLQAALTEIRFNGMAGPTFDRMVQEHHRAIIEAIAAHDPDAAEKAMSDHLQAVIDFF
KSASDKQAT

>gene_1845|GeneMark.hmm|300_aa|+|5308|6207 >NVVL01000096.1 Rickettsiales bacterium isolate NORP64 Contig_source1382A_29577, whole genome shotgun sequence
MTTSREKLLALMDAALKAADPGVQIPRNLPEPPKGKTIVIGAGKASANMARAFEKAWKGD
VEGLVVTRYGHAVECDQIEIVEASHPVPDAAGEKAAKRILEIVSDAGPEDLVVCMISGGG
SALLALPGPGLTLEDKQAISKALLRSGATISEMNCVRKHLSAIKGGRLAKACAPARMVTY
LVSDVPGDDPAIIASGPTVSDPTTGQDALDIIRHYQIEIPQAAKDLLQSAEDETIKPDDP
CFEGHETIMLARPQDSLEAAASVARDMGLNAVILGDAIEGEAREVAIVHAGIAHQVANHG

>gene_1846|GeneMark.hmm|757_aa|-|114|2387 >NVVL01000097.1 Rickettsiales bacterium isolate NORP64 Contig_source1382A_29601, whole genome shotgun sequence
MSLPIPRKLTDDEITEIVNGLPESPGLTDAAEKSREGVKLDLIFQLKDIKIVEEAIDDLK
NIIYTKYKKSLVKPGAPVGMIASSALIAHLTQATLNSFHSSGSVGEELSGIGAMMALMSG
SSNKKGIGTIISMKNKYLNISDILALRKDFVELNVSDITMEMLILSKQELMESEPKNYDL
YRSVIGKIPNSSNGLRLVLDTKILYNNRISSLDISKAILSKVNTGMAQLIECVYFPTKVG
IIDIYPDSKSILSTIHSSIACKDPVLFFIHSMLLPIIGDIGIMGIKGIKSMGPVSFDIWS
LILGEKKISNGETLLALDRNKMFMHGVSKNHVNRMLREFGMVIVERKVKIGLTMVNTIKS
DEYMVVKLTEKRSDGLTSPTKIIHHYRDETDELEEAFEEDKLSGVIEPNTRMEETPYQKV
SKYWYARTNGTNLNEIMRMDSVDPDHTHSKDVNEIINKLGICAAKNFIFLRTVEVASLGG
ANVDPKHILILADYMTSKGNLSKVTCQSKTQNSQFITGASSKSAADALKNAAAIGSSEKM
ECGPTLAIASGAVPRIGTGYAENVEVEARIDELIASIESSKEIDYNKQDFSDSISNTRRL
MSGSSVLSTGNNTPEFSFTNTPDVGVEVLYIPQVGKRIKKTHTKHNRKSVNVIFNMVNRS
LDEMKEKIETERKITDEIYINKESPVTQTDVEPSITFIPKITNFNDIPLILFKYLGEFLG
IDEYKMVLHDNGKVPLIDITQMSNILNNFNQKIGANF

>gene_1847|GeneMark.hmm|237_aa|-|2421|3134 >NVVL01000097.1 Rickettsiales bacterium isolate NORP64 Contig_source1382A_29601, whole genome shotgun sequence
MQTWKNNDIPDHWKESISSIKSKMPKWRHVLMTDEDNRNFISEHFPDFLEYYDKFPHNIQ
RADAIRPCWLYVHGGIYMDLDLVVQKNLEELLVNSGAHFVASGNISHTITNSFMASEPGN
PIWLEYIEQMKLAPPFWAMGKHMRVMTTTGPLCLTRVINESTQSHTILPNKLLMPCSVCN
ISRCDTTKSYIKPIKGQSWNDWTSHTLNLCMCKWRELLLILALFLLLLISYVLVPQL

>gene_1848|GeneMark.hmm|315_aa|+|3193|4140 >NVVL01000097.1 Rickettsiales bacterium isolate NORP64 Contig_source1382A_29601, whole genome shotgun sequence

MRSRKKCRNVFIRGAQGIQGDQGLTGDQGDQGLTGDQGDQGIQGIQGDQGIQGLTGDQGI
QGDQGDQGIQGIQGDQGIQGLTGDQGDQGDQGDQGIQGIQGLTGDQGDQGDQGDQGD
QGDQGDQGIQGIQGDQGDQGIQGIQGDQGIQGIQGIQGIQGDQGDQGDQGDQGDQGDQGD
EGDGAWDFSFNTSLSLGGRVYESDVVSTSGSSPFVNVCILEYPGSDLVGDINYWSVTCAI
TDDCCGEAELFVVSTNSVISSIEIKFGTSLLPVSALSGSFSNIPTSDSVLGIRMRITESV
NGRCLLTLYSSKITC

>gene_1849|GeneMark.hmm|259_aa|+|4169|4948 >NVVL01000097.1 Rickettsiales bacterium isolate NORP64 Contig_source1382A_29601, whole genome shotgun sequence
MRTLCVLIGFDYKNTKKNLVCIFIDLYIAYTSLKNMGHKKIMIITDIVETPDILLLKNAI
FDGLVGGNILSFMSEIRNKGKYIKYTGSISLKHLIMSLVKLNNQEKMLLYYTGHGENNSI
TLPTKFDVLSMNDIKDIIEESTKINKAIILMDCCFGSINLPYTLRGKTYRLSTNDMKFSK
KKILCISSTTKCGTSLMTNTGSVFTKSLFENIALSDVVEITNRINTEIWTKSNSKQKVTV
YSSHPNIKNITQILELGPN

>gene_1850|GeneMark.hmm|264_aa|-|4958|5752 >NVVL01000097.1 Rickettsiales bacterium isolate NORP64 Contig_source1382A_29601, whole genome shotgun sequence
MNRKIKYQKLLVLSIKAKGGKLLTPYVRSDVKVSVKCKQDHIFSCTPKSIKSGKWCSECP
IQKKINAKNIIEKKGGELLSKYSRETTIIRVRCGRGHIFKTTQKKVLLRKWCPMCSGSRG
EIECSVYLNSKGIEFFKEVKGKNLEKIDIHKVDIKNRRFDFYVPSHNLLIEYDGRQHFSD
IKFYNGTPFKERQKIDKDKTIVALECGYNVLRIDNNSSHVIRRILGKTLELIKLDINSSN
LYITSERYSYLDHNNKMVYVQSKF

>gene_1851|GeneMark.hmm|49_aa|-|1|147 >NVVL01000098.1 Rickettsiales bacterium isolate NORP64 Contig_source1382A_29766, whole genome shotgun sequence
MIMRIVKRWGKRVWLHFRLKRIWNNLFTSIINCRIFIKIIEDMSNRKIK

>gene_1852|GeneMark.hmm|80_aa|-|155|397 >NVVL01000098.1 Rickettsiales bacterium isolate NORP64 Contig_source1382A_29766, whole genome shotgun sequence
MSASQEDEFELNPLPPDIDPLVKAKMIKLKNNRFLQQNLPAWRPVSTFRSTLVTFIVIGS
VFLALGILLWMETLAVVEVE

>gene_1853|GeneMark.hmm|50_aa|+|482|634 >NVVL01000098.1 Rickettsiales bacterium isolate NORP64 Contig_source1382A_29766, whole genome shotgun sequence
MDLKEIMVSLVLGNDCLQDRIITIMLVTTNMIFANIKEKMLMEAGLKIII

>gene_1854|GeneMark.hmm|164_aa|+|1012|1506 >NVVL01000098.1 Rickettsiales bacterium isolate NORP64 Contig_source1382A_29766, whole genome shotgun sequence
MDIVLNPINKDLTRIPVTSITMAKTLTSAKRTLKEDLYDHRIKTAGVSLLTLLRPSLLLE
LLIEQEDITEAAMESDNTREQMTNIIKAEEVNIAEERGVEGAQILVTQVRQNVVVEEDKD
KEADIGDIVAIIDTLTEALNDSTEIEASTTDTRNVIKTASFLSP

>gene_1855|GeneMark.hmm|69_aa|+|1617|1826 >NVVL01000098.1 Rickettsiales bacterium isolate NORP64 Contig_source1382A_29766, whole genome shotgun sequence
MCDTDPPKELSQTNATLLEDSLNCQLSSQVATHNEEQWKIDSTILSEFAVQSILDKDRGS
AIQSRIFGD

>gene_1856|GeneMark.hmm|35_aa|+|1845|1952 >NVVL01000098.1 Rickettsiales bacterium isolate NORP64 Contig_source1382A_29766, whole genome shotgun sequence
MLRDLLEKDNISLTRTLQHRFEEVEKLNGIVDAEE

>gene_1857|GeneMark.hmm|89_aa|-|2408|2677 >NVVL01000098.1 Rickettsiales bacterium isolate NORP64 Contig_source1382A_29766, whole genome shotgun sequence
LIVSYGILTLPNSAGAFELECPTWRPMTGWKDEAFTFYLGGPPKLVNSDPISKDLGKRAQ
LTSISSGSVHVHCEVIMKNFKELGLSGNN

>gene_1858|GeneMark.hmm|97_aa|-|2704|2997 >NVVL01000098.1 Rickettsiales bacterium isolate NORP64 Contig_source1382A_29766, whole genome shotgun sequence
MKEQLPITNPRYISSVKSLVAQILIPLKVYFAKFRLSMENTGLLFLKLKVFKLILHFLMM
MECLYGLILLICISMLGVFMVGLGLYVKCGDLMISVN

>gene_1859|GeneMark.hmm|60_aa|-|3228|3410 >NVVL01000098.1 Rickettsiales bacterium isolate NORP64 Contig_source1382A_29766, whole genome shotgun sequence
MITRRGAISPKKLKPEVIENFPPEMPTATVGGGSMNHIKIVPDKAILPFNGLNQNYKKDE

>gene_1860|GeneMark.hmm|91_aa|-|4683|4958 >NVVL01000098.1 Rickettsiales bacterium isolate NORP64 Contig_source1382A_29766, whole genome shotgun sequence
MINPQINGGFAAPVPLTNEQNQRLQIARRKSTDLRDYNDGMQNNYRIGDDGMYAERKISD
FPYGYRNDTNHLDPSSAGNPFMHQMMSSPSF

>gene_1861|GeneMark.hmm|48_aa|-|4974|5120 >NVVL01000098.1 Rickettsiales bacterium isolate NORP64 Contig_source1382A_29766, whole genome shotgun sequence
MENSGIKSRGQSFDTMIDMEKGVDIHGVITAELAHDPMMVRNFLTQRQ

>gene_1862|GeneMark.hmm|64_aa|-|5453|5647 >NVVL01000098.1 Rickettsiales bacterium isolate NORP64 Contig_source1382A_29766, whole genome shotgun sequence
LEMKGHLHRCKIGSVISIKMSNLKKLFWLLSETNATSKIDKSNLKLHRNLLTRIKCFMLK
HQQK

>gene_1863|GeneMark.hmm|104_aa|-|5696|6010 >NVVL01000098.1 Rickettsiales bacterium isolate NORP64 Contig_source1382A_29766, whole genome shotgun sequence
VEAVPKEAIVNLIKAGLISVSKSLAMLSRDKMKKILINNAKLFYLEIVESANQVFHFAIV
KTCFRLRMKLLLGALTCNNGFNCPMEASYSCMFGIQEGRRGLEL

>gene_1864|GeneMark.hmm|643_aa|-|3|1931 >NVVL01000099.1 Rickettsiales bacterium isolate NORP64 Contig_source1382A_29788, whole genome shotgun sequence
MSRKQKKEEEEDLTELEIASRSWGGFKGHFTRVVKSAIKSLDFAPHVPGSLLVINNLKEH
MESVQKAFTRADSSLSVCALLDDPDVQGDYDGKAEELHARRDNIIAQLMDQIDELIAKAE
DRKNTAALEAAAAAPLHGQGAGPGPGPTVRPNAELKPPTLTRDFTPVEFRSWSEKFRAYH
SSSRMELGKVRDQQAYLRACLDSYLDSRLARKLEDDTPIFGRDSCIAILQTEWEVRYPIL
ERRLEFWRTKQATGQWFTDWATALHRMGDEAELDNMTINQMYIMRYIVGTNDIKLREKFL
AEIDPSLEDLQRIAGAYEVAAVSMKAVAPHQHQAQAHAMQTGQQGGRDKPRGQSGISRED

KMAEMRRKDLCFRCAKRKHENKSECPAINGSCNKCQKKGHYSPACLAGYEAKGYRQRVRD
DDRQKEKYKKTTSQTKTNQVTATTNAILVGTLTGENAATPRMMVQAKATSGTPFDFPALP
DTGATRTIIDADVARTYGIRPKKIKAELVTTASGQSMKCLGSAVMTITFVNRTIHVNALI
VEGIRAGFIICWRDLIRLGVLHDTFPQPIPQQAEVMAVDDKKPPSPPPSPSSPDARGAPC
GPSTGAIPKKKRRQDSEHDRDGTRQNDNTADTIDSLVAEFADV

>gene_1865|GeneMark.hmm|170_aa|+|2595|3107 >NVVL01000099.1 Rickettsiales bacterium isolate NORP64 Contig_source1382A_29788, whole genome shotgun sequence
VINSAHEVDMRLDVGIWFGLGSFFRLVCVSDNGGNLNALSRQCGHLADILSLSRNVVDLG
EAMRVERGRDPLLRRFVVTWWKKGWQRTSIPDDRMRCGESMRKVVRKRPLLHVASGGLLS
AAGALSYAEVQSPCSQGKQGNRHERDTHAGCVDLEQVEPAGGILFDRPVC

>gene_1866|GeneMark.hmm|952_aa|-|3229|6087 >NVVL01000099.1 Rickettsiales bacterium isolate NORP64 Contig_source1382A_29788, whole genome shotgun sequence
RRQDSEHDRDGTRQNDNTADTIDSLVAEFADVFDDKEVTPMAGAPMHIHLREGVNVRPTR
ISTARLIPLHYREEAEKAIELFVRSGVIEKVTGNPTDWVAPAFFVPKPNGKVRLVVDYKG
INNQIRRPIHPFPCPRDIIRGILPTSRYFIKLDAVQGYYQIPLDDDSKDLTTFLLPSGRY
RFARAPMGLNSSSDEWCSRSDAAFAPVPNLIKIVDDALIQAPTEREALRLLRIALVCSRE
HNITLSRKKIEAGNDISFAGYKITDKGVKPDESKLSAIRDFPQPKDITTVRSFLGLVNQL
GFFVPDLAHMTDMLRRLLKKNIAFQWLAEHQSAFEQIKALLVSDMIVKPFDTALRTEILT
DASRLHGLGYALVQRNEDDSLRLIQCGSRSLLPAESRYATNELEALAIAWAITDCKFYLA
GANFTVVTDHRPLVGTFAKPLGEIANARLLRIREKLVNFTFDIVWTPGKVHFIADALSRS
PIFHPDAINDALSDDEGIADICAVHTYTVDNSYVYETADVQSLQNFNMEVMQSAAREDEA
YSSTLRALREGKQLRDLPRDHPARAYRDQWEQMSICQDDILIVDFMRIIVPRSLRAKILQ
LIHQGHAGYVRTKALAQEYYFWPSMKRDIQKMIEQCEPCQRLRPSLPKEPLQLHKKATEP
MESISMDLFHHEGREFLVTVDRFSGYPFVHKLRSTSTAAIINILAEILNVWGFPVEIMTD
GGPQFRTEFNSYCLQHDIRHRKSSPYNPQSNGLAEAAVKNVKHLMMRTPSEDFPAALAIW
RATPRSHQEDSPSKLFLGRKPRIAIPTLSATSSCQGEKLQEQTPSTEKSGKHQLQTGDRV
RLQDPISRRWTETGIIQSACESGRSYFVKTENGSVRQRNRRFLKKIDHEASFEANPEAEE
RHEADKAECEKKHAPNTQLKRGRKMKTYADAAKRAISPPKRRSARLAGKKAD

>gene_1867|GeneMark.hmm|291_aa|-|2|874 >NVVL01000100.1 Rickettsiales bacterium isolate NORP64 Contig_source1382A_29869, whole genome shotgun sequence
LTYSSKQATLSVPNKFKNIQGNGMQWIKAIFIGLILIGLSVLAVFFIWLAPVGAAYSAKV
MCSAIFVNGLTSTRAREIDVLADNNPLLSLITTNVDLRNQAVSAHAFGFRKRFAIYRPNL
GCTLADSPEHIAKLRNSTPVMTPVEPRPLLTTSLPADVDRRALNSILFDAMDEPGLRPER
RTRAVVILHDGKVVAERYAAGITAETPLPGWSMTKSVFNAILGRMRFEGMISDLQEPVLI
NEWQAEPGDPRATINYDELLRMRSGLEFDESYANPLSDVVQMLFIEPAAAG

>gene_1868|GeneMark.hmm|391_aa|-|897|2072 >NVVL01000100.1 Rickettsiales bacterium isolate NORP64 Contig_source1382A_29869, whole genome shotgun sequence
MPPIAAVIILYGLGLTASMQVGLIGPIAPDLRASFGLSQAEFGLVASLITAAGALCAIPA
GVWAARAGLRKSLVLGCVMMILGALIFATASVSSLLYVGRLVTSVGYLLIVVGAPSWMAG
FRNPKLVAMAMSIWGTFIPVGIALGNWISAAVVTWIDWRLAVGACTLPVLILLPLLALMD
KPETGSARRSLLLGVIGVLKSRAALQVAMTFAAFAGATSAALAFMPAMLADNLDIGIALS
AGIIGTTAIAGNVVGSVAAGAVLGRSIPGRAILIVFLPIMAVAISTLFVSTSTSVPLSISML

IVFNICQGAVAGTCFALLPKIATEGLSMPALQGMLAQFAEIFVVIVPPFAGAMIDRDGWA
GAAAVLGGFYFIGFLVSLRKPGASAPIRQKS

>gene_1869|GeneMark.hmm|193_aa|-|2183|2764 >NVVL01000100.1 Rickettsiales bacterium isolate NORP64 Contig_source1382A_29869, whole genome shotgun sequence
MKPMDETAPDKEFGRLLRQWRRTSDIKQSRLADILGVTQATVSRWESGAQSPAPLQYDLI
HQMMTRKRNFRLDHAVKRLVMHSSQRVHLIEDRSHRLLCASRPRENEWQRDCTPLLGTSL
WRFATPEIASAEKSLHDLGWHEDQTDHLTFETSRRDGDPMRIIDTYILWERFFLSDGTAV
RLTTGFDQKPAHI

>gene_1870|GeneMark.hmm|189_aa|-|2833|3402 >NVVL01000100.1 Rickettsiales bacterium isolate NORP64 Contig_source1382A_29869, whole genome shotgun sequence
MRIVGGTHRGRLLSAPEGRDTRPTLDRVREALFSILSHASRWYEDDFNPLFDGVILDAFA
GSGALGLEAISRGADQAVFFDNDRKAVAIIEQNIDALRISDQASARRADATKPPRASRPA
SMVFLDPPYGKDLIEPSITALAKAGWIDGNTLIVAERDPRDPEIALEDFYLCDSRKYGRC
QIDIGRFIA

>gene_1871|GeneMark.hmm|437_aa|-|3408|4721 >NVVL01000100.1 Rickettsiales bacterium isolate NORP64 Contig_source1382A_29869, whole genome shotgun sequence
MSDKNTTPDDNKPERIAKRIARSGVCSRRDAERMIAEGLVRLNGKKLDTPAVTVTDEDEI
IVDGKPLPEKEKPRLWRLFKKRGLVTSHRDEQGRDTVFEHLPSYVPRVISVGRLDLNSEG
LLLLTNDGELARYLELPATGWARRYRVRVHGQIDEAKLKQLEDGVTVDGINYGSIHAEVD
VQKGTNAWMTVTLREGKNREIRRVMEHLGWPVTRLMRVSYGPFQLGNLAPGECEEVTGKV
MKEQLAAFFKGDYSKVSEKKSVARGGTSRPKAPRTVFGQPAQRKRPQGGPNATPQNAEAE
EQRETRERPSGGAARKGPRIGAAPRAGGRGKPATGGGGRQERDMREERGSSRPTNKGRGR
YSAEGKAASGRDGGRDGGRDGGRGTGGREGKPGFIEGRGRGKPNGKPSGKPAGRSGDTTH
SAPGRKPSGNRPRGKKD

>gene_1872|GeneMark.hmm|240_aa|-|4718|5440 >NVVL01000100.1 Rickettsiales bacterium isolate NORP64 Contig_source1382A_29869, whole genome shotgun sequence
MNTRKPIIGITLDSEEPGGYSKFPWYALRENYAGAVTEAGGIPMPLPHEVELVPDLLDLI
DGLVVTGGAFDVDPTLFGATETHDTVVTKDRRTKFEWAMAEGALERDMPVLGICGGQQLL
NVILGGSLIQHIPDAIENCLPHEQPNPRNEPGHDVTVEPNTLLAKIVGDVKTLSVNSAHH
QAAEGVGPDVIINSYAPDGVIEGIEHPKYRYCLGVQWHPEFHISSGDAKIFDALISEARK

>gene_1873|GeneMark.hmm|110_aa|+|5711|6040 >NVVL01000100.1 Rickettsiales bacterium isolate NORP64 Contig_source1382A_29869, whole genome shotgun sequence
MSADSYMDMALEEARAAARRGEVPVGAVLVDADTGEVLARAGNLTEEMNDPTAHAEILVI
RAAAGAKGEPRLPNCDLYVTLEPCPMCATAISFARLRRVYFGAYDPKGGG

>gene_1874|GeneMark.hmm|1142_aa|+|165|3593 >NVVL01000101.1 Rickettsiales bacterium isolate NORP64 Contig_source1382A_29891, whole genome shotgun sequence
VDTDMGKKYTKFEFYDQKLSHINDIRFTYREMDVLACLVNKLEEEEISLLLSIASQTIKT
YIRNIRIKIGNNSRISILRFVEKSQYLLLIIEYYKYINIEASFQKQLLNIGTKINPELIS
IKLSHGGLSKTEQNKLKILNDHLKLSNIILENLPHPDTNAPPNTRHPNISHSNTNHPEIC
HESVSVLGSSLPSRSLQDIIIMLESTDLDFDKNEIIDLENNYYESVFILIEKILKSPKIQ

DFFDKHVKNSKKLSFKRASKSPWNKLYLIVIFSVLLAAILAYNNLIFSFKTKTVIKSNLF
LSQVDSKLYRSGMTSLIKNKFAESSDIQKVVLAGIGGAGKTTLARQYAKNSNARIIWEIE
AITKEKTFLSLQQLASELCENNIESQDLKAILSRGNATSRYGKFMSFLTQKLKTHSNWLL
IYDNVKNFQHIKKFFPYDHNVWGTGKVIITTRNSNISSNIFIPDDNIIYVGELNPKDKRT
LFDNIIGDLGMMTEKEKIKINDFLAQVPSFPLDVSLAAYYIKETNISHERYLNQLLKRDK
KFVLKQEKLLSEIGEYSNTRFDIVAICTKQLIKNNPNFQELLFLISLIDPQTISREILLD
FKSNIIVDRFFHFLKKFSLITKRTLSIHNQNIDSISLHRSSGRIILSFLKSFMNEAEKQA
VYNQVNQSIAKYMLKVIDQCDITKIKLIIRHAESFIRNTKENPIDIKMGNLLSECYMIVG
EYKKSKQLLHEIISVNNLQDIPNSENLAETISHLGNVYRAMGKYQEAQEHLEESSKLLAQ
TYGLKNIITRKALINLSYTYRDLGKYHKAKNILKDMLNVSKELKNIAPIELANIKAELAD
IYKKLGMYDIALKYYQKAIATLKNHYGNNTTKIAWIYVQLGALYRNMGLYKKSKDLVDQG
SRFFIDNYGKHSVKIAWILIQKGNIYQDIGNTKNITELFKRAHRIYQDYYGENHIKLGWA
SCYLGRAYSKFGLHKESISFLKQAVKIHKMHYGLDHVKTAWAQSHLINSYIKNGNLTEAQ
HLLEQVNKIYKDFYGATHVKYAHILLAKGQISFAKGHLELAEMMYNDALDIYNNSSHAGR
YKCFEALGDLYLFRAQNNVSGNINISNILYKKAKYYFTESLDILLKDFPKDSPHVTRIQS
KI

>gene_1875|GeneMark.hmm|703_aa|+|3771|5882 >NVVL01000101.1 Rickettsiales bacterium isolate NORP64 Contig_source1382A_29891, whole genome shotgun sequence
LSRIKKLFLYFLGILILTIGYYIFFHKPKADLIRSELPLPCNEIILVRSDLINQIASSLK
EKSGIQTVVLHGIGGAGKTTLARQYASSQPYPIIWEVNAENSATLNASFERFIYSSCKSE
KLKNEFKLISKDNDAVRRERNLYLFFAKIVREYDNWLLIYDNVRQFHDIEKYYPYDTSVW
GNGKIIITTRNSRIKDNILIPDSNMIKVDELTELEKTALFKKILPNNINESLKNELANQE
FLKTIPSFPLDVITAAYYIREEKTPFAEYLKYIETHNNQFTKLQKELLLNIGGYDKSRYG
IISTSVKHLIENNDLLPQLLLLCLLDSRNIPYNLVTKSKDPYIIDQISNIQKLALISLED
KTNNFISIHRSTQAIMLSVILDIVDAERVKKVTNQVVLDFVKYLDNKLSSFSTAEIKLMI
NHMQRMLGRLEISSDKSRILLKQALGEYHLRIDQDKQAKELFRQVVSDNKKHRDQNSTET
ATSLVLFAITLVDFAENDKAQELITTALNIFEKNKEDDNNIAWAKVWLGVIQRNNGDYKK
SIDLLEQAVSIYDKNKKEQSLYWAYVYLGSSYVSIGNNEKAKYLLEKAQDFFIENNGPKH
FRIAWLSTYLGKVYTNMKEYKKAEDALNTSLTLHKNLFEDKHYKIAWSMENLAALYTELG
KYQKAKSLATRAIAIYNADESSNLLALSRTKALLNKINQALGE

>gene_1876|GeneMark.hmm|48_aa|+|173|319 >NVVL01000102.1 Rickettsiales bacterium isolate NORP64 Contig_source1382A_30088, whole genome shotgun sequence
MVIPGYQGFVPGVKADNIYGKTNTEASRKVLNKTLDGRIGFNSFVNIG

>gene_1877|GeneMark.hmm|57_aa|+|485|658 >NVVL01000102.1 Rickettsiales bacterium isolate NORP64 Contig_source1382A_30088, whole genome shotgun sequence
MISPVKLAKPNVRSKSVISYKMQLPEPTLGKDSGSGFAPNKPMRISNMMCLNIDYLY

>gene_1878|GeneMark.hmm|54_aa|+|954|1118 >NVVL01000102.1 Rickettsiales bacterium isolate NORP64 Contig_source1382A_30088, whole genome shotgun sequence
LEKKKAPHKLTVEDASNDDNSIVEMTQAKMDELKIFKGDTVLLKGKSMVGLISV

>gene_1879|GeneMark.hmm|420_aa|+|1151|2413 >NVVL01000102.1 Rickettsiales bacterium isolate NORP64 Contig_source1382A_30088, whole genome shotgun sequence

LVAEDSDNLDNSKLRMNKVVRKNLCVRLGDIVQVHPCPDVPNGNRVHILPIDDTIEGITG
NLTQIYLIPYFKDCYRPLRKGDTFLVRGGFKAVEFKVVETDPGEYCIVSPNTMIFDEGEP
IQREDEDQRDGIGYDDIGGCRQQMAKIREMIELPLRHPTLFKTLGVKPPRGVLLYGPPGS
GKTLIARAIANETGAFFFLINGPEIMSKMAGEAEGNLRKAFEEAEKNSPAIIFIDELDSV
APKREKTQGEVEKRVVSQMLTLMDGLKGRGQVVVIAATNRPNSIDTALRRFGRFDREIDI
GVPDEVGRMEIMRIHTRNMKLGEDVDLAAVAKETHGYVGADLAALCTESALQCIREKMDL
IDIEDETIDAEILDAMFVTQEHFTFAMGQTNPSSLRETVVEVPNVQWDDIGGLDETKKSL

>gene_1880|GeneMark.hmm|200_aa|+|2465|3067 >NVVL01000102.1 Rickettsiales bacterium isolate NORP64 Contig_source1382A_30088, whole genome shotgun sequence
MSPSKGVLFYGPPGCGKTLLAKAVANECSANFISIKGPELLTMWFGESEANVREIFDKAR
GAAPCVLFFDELDSVGAARGSSQGDAGGAGDRVMNQLLTEMDGVGGKKNLFFIGATNRPD
ILDEALIRPGRLDQMIYIPLPDLPSRFSILKAVLRKSPIAQNVSIEFLSQMTENFSGADI
TELCQRAIKAGIRESIEAEV

>gene_1881|GeneMark.hmm|126_aa|+|4273|4653 >NVVL01000102.1 Rickettsiales bacterium isolate NORP64 Contig_source1382A_30088, whole genome shotgun sequence
LLREEKRADDNFDPQTKFKILDTSQMEVVEKHAQALAEKEGTGCREMFKHKKLEELALMY
RVFSRVELTLKYILDEMQPYIQERGKILVMDKELEKNPVEFTKKLLELKREMDEMVESSF
NNNMKF

>gene_1882|GeneMark.hmm|138_aa|+|4684|5100 >NVVL01000102.1 Rickettsiales bacterium isolate NORP64 Contig_source1382A_30088, whole genome shotgun sequence
MNEQQVTAHFIASYCDNEFKKGLKGVSEAETDSRLDAIIRLFCCLHSRDVFVKQYTKFLA
GRLLNKSSISDDAEQLMLSKLKVECGLQTVNKMSHMFTDMTLSKELMNEFKKSPFGNDGF
INGVEIHCEVLTNGHWPE

>gene_1883|GeneMark.hmm|60_aa|+|5287|5469 >NVVL01000102.1 Rickettsiales bacterium isolate NORP64 Contig_source1382A_30088, whole genome shotgun sequence
LLLFNEKEVYTYEEVGKQLNLDAKVLHTQCMYMFSPKMRLLTKENAKKPMCTPDEKISIN

>gene_1884|GeneMark.hmm|70_aa|+|5599|5811 >NVVL01000102.1 Rickettsiales bacterium isolate NORP64 Contig_source1382A_30088, whole genome shotgun sequence
VVRIMKVSVFIVTLKARKEEKYNPLLEEVFRQIQMFRPQPKMIKERIESLIEREYLKRDE
NDRSRYIYLP

>gene_1885|GeneMark.hmm|280_aa|-|82|924 >NVVL01000103.1 Rickettsiales bacterium isolate NORP64 Contig_source1382A_30229, whole genome shotgun sequence
MIHAIIEIAPSGLLQGLILSLLVLGVMIPFRLLDFPDLSAEGSYPLGGAMCASLIVVGVP
PIFALILACLCSGMMGVGTAFVHLRYGVNTLLAGIILSTMIYSVNLRVMGKPNIALFEHP
NLFSDLSSGLSSQIIALLIINFVVMCGLFLFLNTEKGLRFRAVGLNAKFAERQGANLKKN
ITAGLFMGNALCGLAGALLVQIQGYADIGIGVGIVIHALAAMMIGEKMIGTHTTLRMVAA
PFIGAILYQQIQGIALAVGMAPSDMKLLTGVIVLGIIALR

>gene_1886|GeneMark.hmm|250_aa|-|921|1673 >NVVL01000103.1 Rickettsiales bacterium isolate NORP64 Contig_source1382A_30229, whole genome shotgun sequence

MLALKNISKSFPQITAKVLKNINLTIDEGEYCIILGSNGSGKSTLLKIISGEYSADHGQI
FLNDIDITKQAISHRAGHISSVAQDITKGVIQDMTLLENMSLSNLRGQDASYKFYKDQSA
RILESVRALGLGLEKFLHSNMSTLSGGQRQAVATLMAMEPRPSILLLDEHTSALDPVRQG
KIMHFTNEYIKQHNITSLMITHNIEDAAKYGNRLIIMHHGEIVFDVKGQEKQALSSGELL
DILYKMGGSL

>gene_1887|GeneMark.hmm|326_aa|-|1679|2659 >NVVL01000103.1 Rickettsiales bacterium isolate NORP64 Contig_source1382A_30229, whole genome shotgun sequence
MLRSPKIYAAFAVIALISLLSIFKTDPNRSAKTLVAIANYGPHSSLLQTIDGIKEELTKL
GYTEGENINYEISDVNFETSLIIQMLHKLKASKPDVIVAISTPIAQAAKNLIKDIPVIYA
NVTNPEEAGLVSDDIDTNITGASDRQDFSVMLDFAKKLLPRAKTVGVLYSLGEANDASMV
DALKKTAEILSINVLAVPIEHTRDVATRMRVFKGNVDFIYTGSSGAIQASLPAIASTAES
MKLPVFNFNSAEVMSHNVLASYGISHRQIGANAGQIIHRVLLGIKPVDIKPVYPDKADYA
GFISRKRAAKIELAIPADLKNVTIVD

>gene_1888|GeneMark.hmm|615_aa|+|2991|4838 >NVVL01000103.1 Rickettsiales bacterium isolate NORP64 Contig_source1382A_30229, whole genome shotgun sequence
MTQETKKFDAEIGKVLHLMIHSLYTNKEIFMRELISNSSDACDKLRYLSQTDAALTKGDA
DFKISVKVNKEARTVTIRDNGIGMNRDDLSENLGTIAKSGTQKFLEHLSGDNKKDSQLIG
QFGVGFYSSFMIADQITVTSRKAGEDKVYSWSSDGAGEYIIADSDKEFSRGTEIVLHIKK
DEDSFLDHFRLKHIVKSYSDHIAVPIFFEDEEGVQAQVNSSSALWRRAKSEIKEEQYTEF
YKTVSHAVDTPWLTMHNKNEGVVEFTNLLFIPSTKTFDLFHPDRKCRVKLYIKRVLIGDE
NIDIVPQYLRFLRGVVDSEDLPLNISRETLQHNAVLEKIKKSIVKRTLSELKKKKENNFD
EYALFWDNFGGALKEGLCEVTSDHEKLLEVCLFRSALHNKMISLDEYLANSKDKEKTIYF
LSGDNPEKLRNNPQIEGFLSKGIDVLLFTDAVDDFWINVNGTYKEAQIKSVTRADIDLGT
DKDAGDKKEENSVYEPLLSYFKEALGDLVKDVKISKKLTSSPACLTVADGAMDIRMERYL
IEQKQLSTLSAKILEINPGHKIVTKIANDLDKSDKHEENKQLIHLLHDQACIIEGEPVVD
AAAFSKRLNSLLEMV

>gene_1889|GeneMark.hmm|186_aa|+|5072|5632 >NVVL01000103.1 Rickettsiales bacterium isolate NORP64 Contig_source1382A_30229, whole genome shotgun sequence
MSRRFGSNGAFKPFVQQKSRPYPPTLAHPEGYIDTDEHPRPQSNEYPLSKKRKLNIDEPA
LEICQRFGQIISKLDSSNLDIDVAAEIEAKTFSQKLYDFTARSEYDPSIKEFTAWGVALL
DLEDFIYQHRKIQIYLDHLQANKSQYNSNNSYNLAVGYLTELSAIHPSDTIEIGIAGLEI
CMICYG

>gene_1890|GeneMark.hmm|37_aa|+|3|116 >NVVL01000104.1 Rickettsiales bacterium isolate NORP64 Contig_source1382A_30523, whole genome shotgun sequence
KAAVNAHGGEVKVESSGAGVLFRVVLPLASDRGRPRS

>gene_1891|GeneMark.hmm|247_aa|-|353|1096 >NVVL01000104.1 Rickettsiales bacterium isolate NORP64 Contig_source1382A_30523, whole genome shotgun sequence
MRFDMKNPSAAAIIIGNEILSGRTLDINTQEIALRLGEIGVTLVETRTIADDHDMIVTTV
QELSNKYDYVFTTGGIGPTHDDITAKAIAAAFNVGYVRNAEIYDILKRHYISIEEQRAVG
REKMAYIPDGAKLLYNDATNIPGFLTGNVFAMAGIPKIMKSMLKTAIPMLKKGKIVKSES
IDIMTGESVIASDFEALQKKYQNVEMGSYPFQKDGVHATSLVLRSSDYEALDLAFSEVKT

LCKKLGR

>gene_1892|GeneMark.hmm|524_aa|-|1197|2771 >NVVL01000104.1 Rickettsiales bacterium isolate NORP64 Contig_source1382A_30523, whole genome shotgun sequence
MRWVFYIISVFSLLFVLVGYQFWPPAIYFLIIIIPYIAIGIHDIFSVRHTILRNYPVVGH
LRYMLEFIRPEIQQYFVESDRDGTPYPREVRSLIYQKSKGVRETIAFGTKNDITSVGYQF
SYHSLSPKEVASENARVTIGGKDCTKPYEASRLNVSGMSFGALSPNAIKALNKGAKIGNF
AHNTGEGGLSPYHLDGGGDIIWQIGTAYFGCRDKEGNFSPEAFKATAEKEIVKMIEIKLS
QGAKPAHGGILPAVKVTKEISEIRMVEMGEDAISPPMHAECLTPIELLEFVAKLRKLSGG
KPVGFKLCLGHRTEFMSICKAMIKTGIKPDFITIDGAEGGTGAAPVAFTNKLGTPINEAI
TFVNNCLVGANLRDEIKLIASGKVATGYDMVTKIALGADLCNAARAMMFALGCIQSLHCN
TNACPTGIATQNKHRWSALNVKDKSIRVANFHKRTLHSFLELVGALGKDNPDELDPSHIR
RYADISTSKSYATIYPQLSPGELLAKDVTGIYADLWKNSQAERY

>gene_1893|GeneMark.hmm|739_aa|-|2850|5069 >NVVL01000104.1 Rickettsiales bacterium isolate NORP64 Contig_source1382A_30523, whole genome shotgun sequence
MSNKNDNIENVDFGDAISERYLSYALSTIMSRSLPDVRDGLKPVHRRLLYAMLKLNLDPA
SRYKKCARVVGDVIGKYHPHGDTAVYDTLVRLAQGFSLRYPLIDGQGNFGSIDGDNAAAM
RYTESKMTEICTLLMQDIDQDTVDFRATYDESDTEPVLMPAQFPNLLANGSEGIAVGMAT
SIPPHNLHELCDALIYLIDNQKEASCADMMRFIKGPDFPTGGTIIDSKEAIASAYNTGKG
SFRVRAKWEKESFSHGLYQIVITEIPYQVQKSKLIEHIATLFKDKRIPLISNIRDESTEE
IRVIIEPKGRNCSPEMIMESLFKLTALESRVHLNMNVLSARKAPKVMNILEVLQEFIAHR
QEVILRRTKYQIGKIDDRLEILHALKIAYLNIDEVIRIIREEDNPKQELIAKFDINAVQA
EAILNIRLRALRKLEEEHINAEHDELKKKHVELSKILGNSTVLWKVVKNELKMVQKKFGY
DTIIGARKTDFNEIEPSAQIIDISAFIEKEPLTIICSKMGWIRSMKGHNLDLAGLKYKEG
DTQAFVIESYTTDNILVCSTAGKFFTILADNIVRGKGGGESVKLMVDIENYDVLQILPHK
PAEKILLGSSNGKGFVVNSDDLITKARSGRQVMNVKGDVICQICKPVTGDMVAVIGTNRK
LLVFALDEMPEMKRGQGVMLQKYRDAKLGDVKTFNSEEGLSWNLGSKIRLEKEIMSWRAG
RGSIGKIPPTGFPKNNKFN

>gene_1894|GeneMark.hmm|108_aa|-|5316|5642 >NVVL01000104.1 Rickettsiales bacterium isolate NORP64 Contig_source1382A_30523, whole genome shotgun sequence
MARVAKRLRVLRMTDEERIEYHKYLKESAVQEDILHAATERGREEGVEEGMEKGREEGRE
EGREEGLSKGAKLANIKAAKAMLVKGLDIDLISQISELSIDEIMELKN

>gene_1895|GeneMark.hmm|39_aa|+|111|230 >NVVL01000105.1 Rickettsiales bacterium isolate NORP64 Contig_source1382A_30565, whole genome shotgun sequence
MKIAMINSAMRIVPNSHGQKENLDPVPIKITFALEISSS

>gene_1896|GeneMark.hmm|77_aa|-|446|679 >NVVL01000105.1 Rickettsiales bacterium isolate NORP64 Contig_source1382A_30565, whole genome shotgun sequence
MICKSPRSYSLPPTGQIPFSLPFGVAFNEDEFRPWTHSGHMYRFYDQPRLSKAVPYEVNL
NEVSEVYVYAARKSIFY

>gene_1897|GeneMark.hmm|62_aa|+|853|1041 >NVVL01000105.1 Rickettsiales bacterium isolate NORP64 Contig_source1382A_30565, whole genome shotgun sequence

MILRWEWSMNNYSKLLGQGKSHSLTAEGHRVNDISFGLRLEPQLLRKRGLLYNQVELKRN
LI

>gene_1898|GeneMark.hmm|264_aa|-|1241|2035 >NVVL01000105.1 Rickettsiales bacterium isolate NORP64 Contig_source1382A_30565, whole genome shotgun sequence
LDTSYSTNEIKIYQYTSSFVEYFYYKQPVVKEIFPHSGASHGGTQVQVAGAWFKHRPQYG
LIPYCKFGEKIVRGRFISTVRIVCEAPPKEGRGDRVRFEVSQNGIDFTETNYEFSYFDQP
DVLKIEPHSGPESGGTEVRIFGTNFSNHTFSKEFLCTFTPVEGNIPAKSIPARYENNTSV
LCLSPGGWGVGTAAFVRLTFNGQDASQSEHIFYFYNIVTAEPLSGPADGRGGLITVTGNG
FSNSTTTFCKLDNELYKPYSVSAN

>gene_1899|GeneMark.hmm|212_aa|-|2219|2857 >NVVL01000105.1 Rickettsiales bacterium isolate NORP64 Contig_source1382A_30565, whole genome shotgun sequence
VHYKATSKAIGYDGFAPEVKVFDILTDNNITIWYYAEPEYEIVQPTAVPANQEANLMIKT
DFKWEINNKEKIIAHGNFTCRFMSFDGKKVVFTNATILTYPVGDEGDPNTVSCKSPKWDL
SGGLLREENIRVDVSINGVDYSGDRTILISENLDVYKIVPLCGPNEGSTRVKIIGTGFKQ
KEEISVKWGVIITRPLDKQALFDFVYNEAEFE

>gene_1900|GeneMark.hmm|106_aa|-|3122|3442 >NVVL01000105.1 Rickettsiales bacterium isolate NORP64 Contig_source1382A_30565, whole genome shotgun sequence
VKVVPEGLTRSFIYYDSPQILAITPLFGPVRSKTDEILEISGTNFKCPVEGCDLLCRFGK
PPIAMYSKATRVSDTKVTCPIPKYAQPEIMDVELTLNGEDYTNNKH

>gene_1901|GeneMark.hmm|49_aa|-|3518|3667 >NVVL01000105.1 Rickettsiales bacterium isolate NORP64 Contig_source1382A_30565, whole genome shotgun sequence
MTIKGKGFSPLKDQNGTTIVGDIWVRFTLIKNDKKIVEEESKAKLISDE

>gene_1902|GeneMark.hmm|180_aa|-|3788|4330 >NVVL01000105.1 Rickettsiales bacterium isolate NORP64 Contig_source1382A_30565, whole genome shotgun sequence
VDYLYYETPIITRIEPICGPERGFTQITVFGKNFVNMGFGMTMCVFNNTIFTNATIMEHN
IIKCDSPPAENWTRFTEEKIVYYYVKITLNGIEISGPEQKFIYYKEADIYSVTPNLGPVD
GGTLSKIDGRGFNQIGVCNMTVRYATWQVKPLNYTYDYIFVESPPVETPDDVVVAVGMNG

>gene_1903|GeneMark.hmm|91_aa|-|4445|4720 >NVVL01000105.1 Rickettsiales bacterium isolate NORP64 Contig_source1382A_30565, whole genome shotgun sequence
LALVPATVLNSTKVICHSPASYILRQSIVEITLNNQEYTDNNVVFYYYRPPFVFDIEPRE
GPTKGNTTVYAIGSNFRNTKDIKCKFADIVV

>gene_1904|GeneMark.hmm|78_aa|-|5033|5269 >NVVL01000105.1 Rickettsiales bacterium isolate NORP64 Contig_source1382A_30565, whole genome shotgun sequence
MTLNKEEWCLQCDNNPDGEKAEIVPFTVSLEGDFSDCENSVAYRYYKDPKVWAIYPRYGK
KNGGTWVQVWGENFLNFD

>gene_1905|GeneMark.hmm|568_aa|-|3|1706 >NVVL01000106.1 Rickettsiales bacterium isolate NORP64 Contig_source1382A_30989, whole genome shotgun sequence
MTRPAGEPIGPSEDTALGPGILETIEANVRVMGIRSTARARRLMQCRAFYVGSHHDHLSR

RWDGSARDPGVGYLHERLKPQGFVPVQSIPYGQRKPDTGMPLARQIINRFSEMLLGQGRA
PGLSIPADVDTERYLEAVFSEAMCWDVLMEARDVTGSCGSAAIVPSVVNGVPTAEVLMPF
DIHVYRWKNGASWEPLEVVEQRLVDRVVPDPETGKLDVQQYWRTRMWTETHTVTFDDVPQ
NWPPEDPIPYRPENRVEHHAGRCPVVWYQNTRSSKSPDGAADCDGVWHLIDQTDRIMSQV
YKAARANVDPMLHIKEEERLKKKIGAVTKGGLVMTSAAGDAKYVEMEGTSISVGLEVVRT
LRQEVLQTAECIIVDPASAGAYKTGEALQLLWRAMEAKTNRLRVMLTQTIRELSRIWLSL
GRAVGVANLEDAKPGNGILLPPVVELVEQTTEEIVAGERPTPNLTTQSVGTGAHITFKWP
PYWNPTAKQVNEMALAMGAAKNASLLSSSTRVTEMAKLLGIDPGDEFVKVKREQLEAQQK
AEDDFEKEVDRMETQGTAAAAVLPPPLP

>gene_1906|GeneMark.hmm|231_aa|-|1703|2398 >NVVL01000106.1 Rickettsiales bacterium isolate NORP64 Contig_source1382A_30989, whole genome shotgun sequence
MRYLGGKSRIASKIVGLINSETPLDSPFYDVFAGGLSITCAAATTRTGPVYANDRCTPLI
TMYQRWSTGWRPKKLGYLDYLQLKRKQDPLDPHTALAGFGMSFAGIWFGSYARDSVNTSY
FDVSVRSLEKGMGPCQDVEFTCKDFSALSPPSESVVYLDPPYKGTASYRYHQGFSTRRLL
SWSANLPTPHVFMSEYESPGPNWEEVGSFDTQGSGLSRKQEKLFRLISSPP

>gene_1907|GeneMark.hmm|232_aa|-|2411|3109 >NVVL01000106.1 Rickettsiales bacterium isolate NORP64 Contig_source1382A_30989, whole genome shotgun sequence
MQYLGGKSRIAGAIVASINALTETGRPFYDVFCGGLSITVAAAATRSGSVLANDRCAPLI
TLYSRWAQGWRPARSIGYLDYKQLQAKQDPLDPRTALAGFGLSFGGKWFAGYARNKSGRD
YYSSAVRVLSDKIKVCGGGVKFTCLDYSVIRVAPGSVVYLDPPYAGTTGYGYHKGFSRAR
FLEWVTSVQNSGSDVFLSEYESPGDGWTCVEEFPLVHGRLATRPERLYHLPR

>gene_1908|GeneMark.hmm|495_aa|-|3132|4619 >NVVL01000106.1 Rickettsiales bacterium isolate NORP64 Contig_source1382A_30989, whole genome shotgun sequence
VNLHEDQRARFNARTALICKRARQCPVEFTRWVSRVEKGAAIEVAATHVKWHQHLDEHDR
VVLFAPVGHGKSNQITRWRVLWEIGRNPDIRIAICSATKELPAKVLNDIRKDIDGEGPGS
QWLRMIFPHLARINWQVWADDRISLVRQGNPTIATYSPGTQILGSRLDLIIADDLHDIKN
TLTPLNREKVHRWFRTEVLSRDSPSGTRVWVLGHVWHEDDALHRLVREQEFKKFKYSCYV
ENEVSGAREPLIPEIWTLEKLALREKNLGPVSAKLMLFNELVDSGTGRIKREYFFECLRR
GSTLGAKFATFWNPGDAPTVTGVDLGFGGGSLTCLFTVAVLPDMSLRVLDVRSGDWTGPQ
KIEQMEYVHQAFGSILCVESNGAQKMLEEFTTDMTALPIKGHHTGLNKHDMDFGVDSLAT
ELANRKWIIPCDDELRPNNEIAFWINEAVAYSRDTHAGDRLMASWIAREGVRLSGFRRRA
SDRRDLIVDADTLVR

>gene_1909|GeneMark.hmm|148_aa|-|4627|5073 >NVVL01000106.1 Rickettsiales bacterium isolate NORP64 Contig_source1382A_30989, whole genome shotgun sequence
MEEQVQTILEMATTGALSLLAMTVSGLLAWGARSIKHWLDSQAHMASFACASTKLASLAS
DAVLEVEQTLVKQLKNDEAWNADTAKTARDTAAAIVKRHLGTRGLAELRGCLGLALADIE
GLIRTQIEVSVQRAGTAGRHSPTVALGE

>gene_1910|GeneMark.hmm|108_aa|-|5091|5417 >NVVL01000106.1 Rickettsiales bacterium isolate NORP64 Contig_source1382A_30989, whole genome shotgun sequence
MLKVLYKFMDYSKMGDAFERVYERGGASAPGRLPKGAKVDFNIDPETVLPAVVSTQPAVV
AAQEQESEDFHQQLFRDFEAWSEEELEAYMSSGDMPASEKFTPGSGDE

>gene_1911|GeneMark.hmm|60_aa|-|3|182 >NVVL01000107.1 Rickettsiales bacterium isolate NORP64 Contig_source1382A_31424, whole genome shotgun sequence
MTKNGEGIDRDQFDKLKSMLDPKTKKMIEDETLEFIKVNMNHLPMYYIPGEEFPWNREFG

>gene_1912|GeneMark.hmm|144_aa|-|360|794 >NVVL01000107.1 Rickettsiales bacterium isolate NORP64 Contig_source1382A_31424, whole genome shotgun sequence
MDRYNGREMGSRLARSVFDIYDPSIQTGSGQSPHEQSSLQDEELPTFFLPQGLGTTIPGN
IIYYHINTTNKLIPKLDYEENKNRKDLTCDNLDEKGNGHFIKFTFNNIGLIDSVTYMGTE
EIVLQSLWAFVGLHENYLNMLTSR

>gene_1913|GeneMark.hmm|40_aa|-|1272|1394 >NVVL01000107.1 Rickettsiales bacterium isolate NORP64 Contig_source1382A_31424, whole genome shotgun sequence
VIPEKHCHVEENYDDEPEMLKDLPIINPDAFCDENVEEKV

>gene_1914|GeneMark.hmm|46_aa|-|1653|1793 >NVVL01000107.1 Rickettsiales bacterium isolate NORP64 Contig_source1382A_31424, whole genome shotgun sequence
LKRLMLETRITVINARMVDIDRGDKNVILHDETIVPYDTLILSMGI

>gene_1915|GeneMark.hmm|101_aa|-|1836|2141 >NVVL01000107.1 Rickettsiales bacterium isolate NORP64 Contig_source1382A_31424, whole genome shotgun sequence
LKKKWDHERAALEEEEENKDDIDGHDRDFLDENESGFALCFSSKRLLSEPKIVKNSRIVI
VGASDTGISFIEALLSISYLNFTNIILVAPGGLPHQHTDER

>gene_1916|GeneMark.hmm|84_aa|-|2361|2615 >NVVL01000107.1 Rickettsiales bacterium isolate NORP64 Contig_source1382A_31424, whole genome shotgun sequence
LHRDSLLDADILVRRAVAADLDLIKPLCESLDEKEEVYDDIYDATINPESKYLSFIAKVG
DNAIGAFVCSKDINLSYYKSHFHI

>gene_1917|GeneMark.hmm|43_aa|-|3294|3425 >NVVL01000107.1 Rickettsiales bacterium isolate NORP64 Contig_source1382A_31424, whole genome shotgun sequence
MICFGDIQYDLRIIEKMPEEENKKKEDDKKEGDEGAEPPKIEK

>gene_1918|GeneMark.hmm|47_aa|-|3444|3587 >NVVL01000107.1 Rickettsiales bacterium isolate NORP64 Contig_source1382A_31424, whole genome shotgun sequence
MDIAEERSILENIFVDEYGEPSFKKCVNIMDLGTKLADKGVAIVKET

>gene_1919|GeneMark.hmm|132_aa|-|3750|4148 >NVVL01000107.1 Rickettsiales bacterium isolate NORP64 Contig_source1382A_31424, whole genome shotgun sequence
MKCNLIAQRISLQEFLTEKKADIVTEIESYTTNDEEAKKLETKAPVMEIFMKWLEGFILN
QPSEYFVDHPTDDADLVCNIQNELNFLISTLEIFGLPMGYMSGEGHFPNWGKDNKDDKLA
AVRKRALASKMK

>gene_1920|GeneMark.hmm|46_aa|-|4197|4337 >NVVL01000107.1 Rickettsiales bacterium isolate NORP64 Contig_source1382A_31424, whole genome shotgun sequence
MGLTSEVDVKLLHQCFELESYDNLLKPEFMDAVRKRRETLLDERRT

>gene_1921|GeneMark.hmm|62_aa|-|4491|4679 >NVVL01000107.1 Rickettsiales bacterium isolate NORP64 Contig_source1382A_31424, whole genome shotgun sequence
MRGDADEEDSWYCFEPLKDFFEELDCRNKEVLKNVRGIHYNSKVFFSSRLLGLPFVEVRQ
AL

>gene_1922|GeneMark.hmm|67_aa|-|4842|5045 >NVVL01000107.1 Rickettsiales bacterium isolate NORP64 Contig_source1382A_31424, whole genome shotgun sequence
VEIGKDDVYNRVYSYPKILKLIETAYLSISVLDQNGQVIAFAAFEDYPHGMKGQYDEKHY
NIWEDWF

>gene_1923|GeneMark.hmm|42_aa|-|5058|5186 >NVVL01000107.1 Rickettsiales bacterium isolate NORP64 Contig_source1382A_31424, whole genome shotgun sequence
MEVNMNEGSPGSQNPGGLAVYNEDNFDMESIVLRRADPDDCD

>gene_1924|GeneMark.hmm|196_aa|-|99|689 >NVVL01000108.1 Rickettsiales bacterium isolate NORP64 Contig_source1382A_31592, whole genome shotgun sequence
MSGVDIIIGPMFSGKTTELFETLNRQADIQHLGGLENKTLLILHEDDVRKEQEKNRNAIS
KNVITIRCTRLSDLNVENFNIIGIDEGQFFPDINKVVRSWMLTKRVIVASLCGDHKTKTF
GEVYKLIPVCSSIKKKLAICLPHIQRKGIFNVGAQCSFRLSFIHGKPQKLIGGYDKYMPV
CIECYNEMKPGTLQSK

>gene_1925|GeneMark.hmm|197_aa|+|754|1347 >NVVL01000108.1 Rickettsiales bacterium isolate NORP64 Contig_source1382A_31592, whole genome shotgun sequence
MSVFILRGVSPKNVRNNYDNGHYAKMQPPGKISNTKLWKAPKVLVLKNDSRLTSFTTMTG
SDKKIISLNNIESKCHNCGCECPDSELGIPIDIKRCKGGYIVLVIGKHCSYQCCFSDIIR
ESNLPKSKRDNIYECSEQFIRIMFKKQHPGRELTKANNWKIMETVGTKIFSSGKLKFIET
GNVSIVRCEKEYVVMDT

>gene_1926|GeneMark.hmm|126_aa|+|1369|1749 >NVVL01000108.1 Rickettsiales bacterium isolate NORP64 Contig_source1382A_31592, whole genome shotgun sequence
MYSYDILTCIFVYTPAILLIFIYSYLKFKRINLSFVKSVIKSIEIRTLKSDIEIYDKYCV
IKFYHNGSEQSVMLPYNRSLKYVGNAKDIYLKYGNTDILVKHYGCIPFMVSAEDIGCDSI
EYVDPF

>gene_1927|GeneMark.hmm|209_aa|+|1839|2468 >NVVL01000108.1 Rickettsiales bacterium isolate NORP64 Contig_source1382A_31592, whole genome shotgun sequence
MNTHLGIDFAASVLAQSISNGILETQGKLSTLYGPNYLNDLDEMDNRWEERQKQKQKKAD
EFGSAYHEEEKPNRRRNISKYTKNVRNGVHIAERGDPIQIVGNSKESLLLYGLVDSEITI
IGKTNNIVMSKCENVVISLETSLTGIKIFKCKNVRLNIESIKYLGISTSNKIIVNGGVAA
NSMITLYTSDKCKVNGSNIPLDMFDVVTL

>gene_1928|GeneMark.hmm|416_aa|-|2505|3755 >NVVL01000108.1 Rickettsiales bacterium isolate NORP64 Contig_source1382A_31592, whole genome shotgun sequence
MKMVTQLLFFHIKKNNLIRMCEIVKASVLIGAALRAVDNDDIKLLNKIIEVISPESLDVV
TSDEMYITLISRAKEHNRKHTTIYLIGKWEELYPNSDKFSLYASLYINYRVPVSLIKYLS
SFYATYTFNEIVSDLIHQNDNQNTLLACIKALEVYGEQSYEIYKRLVDKSSSNSNIYNFM

VDKLKEVSHYAKTPPWINNSFDQIPNVMDLVDRVPNESFILPSDKEISLIIYPGVDVELL
PRRSKILKEISMMSDKEKAELVKPEMDIIRESKRQLDVELGRILGPSNVSEELTTESSVY
GGSRMFLCETYDYDEEDDRVYDWFTGSCEICFSKIKYKWYAVRMPNKGKLGGWEGQYCSW
NCCRKDIVGEDIVTYAMIAVYEKYINKIGIQERLPDKDPSIVWASGDGIIVDEYTF

>gene_1929|GeneMark.hmm|122_aa|-|3718|4086 >NVVL01000108.1 Rickettsiales bacterium isolate NORP64 Contig_source1382A_31592, whole genome shotgun sequence
MLQNIYNLSGGINSQYIFTITLAAIVIGWILVSLWTRVLENVAFGSFGYDSKSSLDSMIV
SLAVTVIFVAFVWMVDEYGVVGVESEVISSDRLKSTVDNLSKVHTDHMSKNENGNPIIIF
PH

>gene_1930|GeneMark.hmm|323_aa|+|4147|5118 >NVVL01000108.1 Rickettsiales bacterium isolate NORP64 Contig_source1382A_31592, whole genome shotgun sequence
MSLPENIKNFSLYPLNDEDKVRYDYYQKQLSSFWTSDEIDFTSDADDFLGLDEDIKTLVK
NVLSFFTVIDGIVIDGIFKTLLIGSVSLADMMFYTAQLNIESVHVVVYAKALRDIIPKDL
GDAILSNLDTMESIKRKTDWIDAHVSSGASREHMLVVFSCVEGIFLMSPIMMIYRLASLG
IFKGLSFANEQISKDEQLHSEYAEYNYSVSKKIPDEDVIKIVSECVELELLTLSDILPKT
SGVFHPKDFVDFIKHLGNIKLKGHGIDPIWDVEVENLASWVGSVSMTRKSNFHETKVGNY
STFSLKDDKPNVDVNVGYMLRDF

>gene_1931|GeneMark.hmm|68_aa|-|69|275 >NVVL01000109.1 Rickettsiales bacterium isolate NORP64 Contig_source1382A_31865, whole genome shotgun sequence
MATNNFSKITSKGQVTIPHNIREKLHLSTGSKIEFIIQDDAVLMIPINNKLSNLYGILPK
PKKLRPQA

>gene_1932|GeneMark.hmm|335_aa|-|465|1472 >NVVL01000109.1 Rickettsiales bacterium isolate NORP64 Contig_source1382A_31865, whole genome shotgun sequence
MITQSANQSTQPKIWVVLVDSRVGNANQAIELAEKLDESFEIKHIKYNHFAKLPNSLLALF
PIHVKRRTLKKLKKETIPDIIISSGRRTAALAIHLKKLSKGSAKIIQIMRPDFDPKEFAL
IILPQHDNFNYTLPNVVRIIGALTNIKEKIAKSQEDFETNYPAAKDFIAVLIGGSTKNYK
LTLENAKTLGDGILTISESHLLPVFISFSRRTPENVKKYFRDKFSWPNIIYDPSEGGANP
YPAILGKAEYIITTTDSISMCSEAASTGKPVYVFTPANFKLKKHKFFTQQLIDIGNVRRL
ETTTTYLDKFDYEPLSEINKIAKIIKERFLSDDGE

>gene_1933|GeneMark.hmm|140_aa|+|1795|2217 >NVVL01000109.1 Rickettsiales bacterium isolate NORP64 Contig_source1382A_31865, whole genome shotgun sequence
LSLISLRNQKEFDLVKNRGSEHSGRNLILLLATKCDLIKSDKENPVFFGMKVSRRLSKKA
VIRNKVRRRIRHLVQNIVKNPKVDTDQAAFIVIPKKGFDKTKFAQLNYDFRRTFSRALRD
RNAAEESESGSKASKSESAD

>gene_1934|GeneMark.hmm|203_aa|+|2601|3212 >NVVL01000109.1 Rickettsiales bacterium isolate NORP64 Contig_source1382A_31865, whole genome shotgun sequence
MSTKLNELKRHLHPGRVYRRADLAKWSSAVDRHVAQLLEDGVLTKLSAGIYHYPKKTTFG
AAPAEDDKLVAAFLKDHRFLLTSPNLYNALGLGTTQLYNEIVVYNHKRHGRFTLGGRTFD
FRMKPHFPKTVTQEFLLVDLVNNLDRLAENTDKLLKRVANKALELDSNKLLRMARDYGSV
KARKFFKEVLSGGVASEKRRCQV

>gene_1935|GeneMark.hmm|113_aa|+|3321|3662 >NVVL01000109.1 Rickettsiales bacterium isolate NORP64 Contig_source1382A_31865, whole genome shotgun sequence
MSRTVTLPIPVKKAMWKLGKDINNARRRRRVTMELMAERAGISRITLAKIEKGQSSVSIG
AYASALFVLGMTSRLENLADSEHDLVGRELEEENLPKRIRLPRSSRGKHNEEP

>gene_1936|GeneMark.hmm|411_aa|+|3662|4897 >NVVL01000109.1 Rickettsiales bacterium isolate NORP64 Contig_source1382A_31865, whole genome shotgun sequence
MSSNKIYVSLQLEKETCFVGTLWCHRRGARQSSSFEYNPDWLSHPEKFALDPALQLTKGV
FHTKSDHVLFGAIGDSAPDRWGRVLMRRAEVARAKELGEAPRSLSEIDYLLGVNDEARQG
ALQFSLGADGGYLAPKDKNTIPPLVNLPRLLSATERYLNDDETAEDLKILLAPGSSLGGA
RPKASIRDQDGHLALAKFSKKDDEFNVVVWEAVALTLAEKAGIKTAEWRLAQIIGKAVLI
TRRFDRQGGRRIPFLSAMSMLGAKDNEAHSYLEIAYALAQHGGAPNQDMEELWRRIVFTV
LISNTDDHLRNHGFLYEQNKGWRLSPAYDINPVPLEVKARILSTSINFDNPTASLDVALS
VIDDFRIKKPRALEIIKEVDSAVKSWRSVAKQFGLSQKEIQRMESAFRRGD

>gene_1937|GeneMark.hmm|52_aa|-|2|157 >NVVL01000110.1 Rickettsiales bacterium isolate NORP64 Contig_source1382A_32065, whole genome shotgun sequence
MNKYVLLLIILIMQNSIIIAKQLPTNKKVSNIITPVSYFVDHVRQLHQLKEN

>gene_1938|GeneMark.hmm|592_aa|-|367|2145 >NVVL01000110.1 Rickettsiales bacterium isolate NORP64 Contig_source1382A_32065, whole genome shotgun sequence
MANTTTNPDFVAAPQHSSARELTQEEFLSLKTKFAEPGNIIPSFWYHKIIDEKGRADITA
ITLLSEIFALYRFSSSSNPCAFISNPSCSSPTLVGRALRVPYEHFVKRFCISKEKARRAF
IKLEELCLITRDVCNIALKKGGRQNFLLLTLDHEFFKSCFRDPKQDVRVRMDAEHRINKA
DNTEFAEGDKYTTINLAKEINEGVTEDNSNSKLNFRKNDQIEEGIYAEERLDGDKSPSIQ
ICNHHIKKKNIIKNRSMKSNFKINDLKEKEKELEIEEILEPTKSNIPEEAGKEEVIRKKE
RKRWEKKTLTDYYPLTEEDCKILQKESGREFNLNAMNEILLDISGKMKSVSFWSKKGFMS
YMSKIYSYEKRNEMLINNASFRIRTNMTKEEAKCQLIEQYLGKIESRTEVTPEMTLQRKL
AVLLKPETAYEFLRAYSSGVLQGEKYVMYLRKHVELMEIEKEVITSQVQVVYGRLRVSED
CFFGTISGVEIKMPKKQTSKLPRASTGEEKKKEPELFKGIWGKVRRGLIECLGEDNDRNW
FSNMSCKVDEAKKELTVITSSDFYKDWANRNFLPELKQVSKDCGFELVGISC

>gene_1939|GeneMark.hmm|85_aa|-|2375|2632 >NVVL01000110.1 Rickettsiales bacterium isolate NORP64 Contig_source1382A_32065, whole genome shotgun sequence
MCYINYIKEVIMSKSVVSLSFAVPLELSIQMNVYMETKGLNRSQLIKAALRLFLEIQKKK
EDIPVKLEEIHQELLNIKKIVSKKA

>gene_1940|GeneMark.hmm|220_aa|+|3200|3862 >NVVL01000110.1 Rickettsiales bacterium isolate NORP64 Contig_source1382A_32065, whole genome shotgun sequence
MKKTKVLTIANRKGGAGKSTCAAHLSIEAAKNGLKTILIDLDPQKTLETWWKKREEENPF
LTDINPKKIEEVIASLNEYDFDLCIIDTPGDTSVNASAGLKVADLVLIPTKPTAPDLQAI
GRTIASIKKLEKEYIFVVTQIVLRTKLALQATAVLSEFGVVAPSSFVNRVAYANAMSVGD
SATAEDKGAKEEVELLWKFISSKLFDKGEKNAKEKVRLNI

>gene_1941|GeneMark.hmm|90_aa|+|3831|4103 >NVVL01000110.1 Rickettsiales bacterium isolate NORP64 Contig_source1382A_32065, whole genome shotgun sequence
MPKKKFDLTSDIINVDLGEANAEESLPAVLSQSRAHRQQEVEAKNKNVLLTFSVEEAFRK
EYKIWCARKGLKMNEAFIKGFELLKQVDYT

>gene_1942|GeneMark.hmm|169_aa|+|4503|5009 >NVVL01000110.1 Rickettsiales bacterium isolate NORP64 Contig_source1382A_32065, whole genome shotgun sequence
MPELPEVETLKCYLAGHIIGETIASLETRRDKLRYELSPYLEQHAVLGVVSGIKRKAKFL
IIELSNGYMLIFHLGMSGRLTMQSVDYQPQKHDHIIISFQSGDQLVFNDARRFGMVYSCR
ADMLSEQSFLNRAGREPLEEGFDACYLLKQLAAKKSPIKTAIMDNKIVV

>gene_1943|GeneMark.hmm|262_aa|+|305|1093 >NVVL01000111.1 Rickettsiales bacterium isolate NORP64 Contig_source1382A_32090, whole genome shotgun sequence
MTLKCGLAIRWLKACADILIKNYNQQGGYMAKTEAKQVTLSSAKYYNILTFIFVFCLLIS
NIAEMKIINIFNIAQIGAGTLFFPLLYVLNDVITEIYGFSASRRMIFLALIFNMSFSLLM
YLIIFLPEGADWQEKEAFNTIFALSPRIMLGSLLSYFIGELVNSAIISNLKLQFRGKFFA
IRATFSTLVSSFLESILFVLIAFYGRMPDQELIKMILMLTIIKVMYEILVMPATLALISF
LKETEKLDVYEKPLFKNIFPKW

>gene_1944|GeneMark.hmm|247_aa|-|1144|1887 >NVVL01000111.1 Rickettsiales bacterium isolate NORP64 Contig_source1382A_32090, whole genome shotgun sequence
MNKILAFDVSNNSCSVAISLDQEILAFEQELEPSMQAERLMVLIESVLKKARLQYQDIDY
LAVTTGPGSFTGIRIGIAAARGIIFASNGIASNIKGIGITNFEASYYRACEQVKDHDSAL
IMLNAYRGQLYVQEFAGGVPSAEARIIDDSELKSLLQNRSGVTICAGSGLAKSYAEIKEL
DDFITSDLIILPRFPVIRALHIARLAGQKIKRGEITPIAPLYIRKPDAIIPKPFAEILAK
KSRSKGG

>gene_1945|GeneMark.hmm|289_aa|+|1947|2816 >NVVL01000111.1 Rickettsiales bacterium isolate NORP64 Contig_source1382A_32090, whole genome shotgun sequence
MKIISGMSNPQLAARIAKEIGAELLDTKTDNFSDGELRVQVHGDMGNDVIIVQSTSTPVN
NHLMELLLLADTTKRAGAQNITAIVPYFGYSRQDRCTYKHGPISASLVIKMIESAGVTKL
ITLDLHSSQLEGMFNIPIINLATESIFFSAIENRENSIVVSPDIGGIARARNYSSLFGVD
LAIINKSRNPDNSCSMNGIIGDVSGKNCIIIDDIIDGASTLCLATELLLAAGALSVEAII
THAVLSGDAVSKVENSGIKQIYISDSICHNRLPPKFVTMPIHQLIGVVL

>gene_1946|GeneMark.hmm|274_aa|-|2956|3780 >NVVL01000111.1 Rickettsiales bacterium isolate NORP64 Contig_source1382A_32090, whole genome shotgun sequence
MIQKLFNFILVAIFVSLIALFVYKSMYKPEILSVSLDGTKIEKNSGASSTGAQGRSKSEL
NAIIKEYIINHPEDILKSLEGMQKKKILELTKKSTEYLKANNSAIENADSPPVIGNLEGD
ISIVVFYDYNCSFCQKANIHTNNLIESDAGIKVILRPIPILGGSSMYVAKVMLAVQRISP
AKFLAIHNDLMKMKLINKDSVKSLISKYEIDYSLVDNEVNSYSIRQQINKNFEFAKGLGI
KGAPSHVINGKFIPGMLTLDKLKSIVKHIRAAKK

>gene_1947|GeneMark.hmm|367_aa|-|3899|5002 >NVVL01000111.1 Rickettsiales bacterium isolate NORP64 Contig_source1382A_32090, whole genome shotgun sequence
GNKIFINTGLIINHPDPDILRGIIAHEIGHILGRHVVRNQEIIENYQKASIGATALGLVA

AMSGSADAGMAVVLGGSHFARRSIYAYSRTFESSADQTAIRLLEKSSHSVIGLIKFFEQV
RKLQATGIDNPYESTHPLSNDRVTILKSFNKRSKFQLSQNHDDIVHKYQRSSAKLAAFTL
EIDNIFKHSYVTQTKDITTYMNAIKSFRMGSFDDSLNYINQLIIKHPDDPYYHELKGQLY
FEAGKGDALVEYKIAIETRPDDELILLGKAIVGITKHIDRPGYLEQFHKDLERVLQKNPD
SVLALHYNAIYYNKMGMVGEGYLSAALIALKSGRIADARKMASAAKNALAKKSVDWYRAG
DILAATE
