# Supplementary material for: Deciphering viral presences: two novel partial giant viruses detected in marine metagenome and in a mine drainage metagenome
Source: Virol J. 2018 Apr 10;15:66. doi: 10.1186/s12985-018-0976-9 (PMC5891951; doi:10.1186/s12985-018-0976-9)
Supplement: Supplementary file 5 — List of NVVL00000000.1 contigs. We identified 15 and 11 contigs among 111 “Rickettsiales” contigs as viruses and as ciliate protist. (PDF 60 kb) [file 12985_2018_976_MOESM5_ESM.pdf]

Marine metagenome NVVL00000000.1:

List of 15 contigs identified as virus (Misidentifiedvirus):

- NVVL01000008.1 Rickettsiales bacterium isolate NORP64 Contig_source1382A_2665. GC content = 33,1 %.
- NVVL01000010.1 Rickettsiales bacterium isolate NORP64 Contig_source1382A_7217. GC content = 33,6 %.
- NVVL01000017.1 Rickettsiales bacterium isolate NORP64 Contig_source1382A_10295. GC content = 33,1 %.
- NVVL01000019.1 Rickettsiales bacterium isolate NORP64 Contig_source1382A_11027. GC content = 33,1 %.
- NVVL01000020.1 Rickettsiales bacterium isolate NORP64 Contig_source1382A_11070. GC content = 32,6 %.
- NVVL01000022.1 Rickettsiales bacterium isolate NORP64 Contig_source1382A_13955. GC content = 32,3 %.
- NVVL01000024.1 Rickettsiales bacterium isolate NORP64 Contig_source1382A_15885. GC content = 40,8 %.
- NVVL01000037.1 Rickettsiales bacterium isolate NORP64 Contig_source1382A_20496. GC content = 36,7 %.
- NVVL01000064.1 Rickettsiales bacterium isolate NORP64 Contig_source1382A_24651. GC content = 33,8 %.
- NVVL01000081.1 Rickettsiales bacterium isolate NORP64 Contig_source1382A_26629. GC content = 33,0 %.
- NVVL01000083.1 Rickettsiales bacterium isolate NORP64 Contig_source1382A_26682. GC content = 34,1%.
- NVVL01000092.1 Rickettsiales bacterium isolate NORP64 Contig_source1382A_29265. GC content = 34,2 %.
- NVVL01000097.1 Rickettsiales bacterium isolate NORP64 Contig_source1382A_29601. GC content = 34,1 %.
- NVVL01000108.1 Rickettsiales bacterium isolate NORP64 Contig_source1382A_31592. GC content = 33,1 %.

List of 11 contigs probable represent an undefined ciliate (*Stylonychia spp.*;*Sterkiella spp.* ; *Oxytricha spp.) :*

- NVVL01000013.1 Rickettsiales bacterium isolate NORP64 Contig_source1382A_8708. GC content = 36,7 %.
- NVVL01000044.1 Rickettsiales bacterium isolate NORP64 Contig_source1382A_23026. GC content =39,1%
- NVVL01000072.1 Rickettsiales bacterium isolate NORP64 Contig_source1382A_25088. GC content= 37,1%
- NVVL01000080.1 Rickettsiales bacterium isolate NORP64 Contig_source1382A_26406. GC content= 38,2%
- NVVL01000089.1 Rickettsiales bacterium isolate NORP64 Contig_source1382A_28651. GC content= 36,9%
- NVVL01000093.1 Rickettsiales bacterium isolate NORP64 Contig_source1382A_29274. GC content= 37,9%
- NVVL01000098.1 Rickettsiales bacterium isolate NORP64 Contig_source1382A_29766. GC content=38,5%
- NVVL01000101.1 Rickettsiales bacterium isolate NORP64 Contig_source1382A_29891. GC content= 32,2%
- NVVL01000102.1 Rickettsiales bacterium isolate NORP64 Contig_source1382A_30088. GC content= 38,3 %
- NVVL01000105.1 Rickettsiales bacterium isolate NORP64 Contig_source1382A_30565. GC content = 38,2 %
- NVVL01000107.1 Rickettsiales bacterium isolate NORP64 Contig_source1382A_31424. GC content=37,1%
