# Supplementary material for: Deciphering viral presences: two novel partial giant viruses detected in marine metagenome and in a mine drainage metagenome
Source: Virol J. 2018 Apr 10;15:66. doi: 10.1186/s12985-018-0976-9 (PMC5891951; doi:10.1186/s12985-018-0976-9)
Supplement: Supplementary file 6 — Protein alignment of Major capsid protein. This alignment was done MUSCLE with standard parameter and was used to build the tree in the Fig. 1. (PDF 104 kb) [file 12985_2018_976_MOESM6_ESM.pdf]

```
Reference sequence (1): Pithovirus_massiliensis_LC8
Identities normalised by aligned length.
Colored by: identity + property

                                                                 1 [        .         .         .         .         :         .         .         . 80
  1 Pithovirus_massiliensis_LC8                           100.0%    -MDGDQRDVLSRMVTEEETEYEKALRYSEE------GCQSLFDYAV------RKPSRNHHFPREM--------KRADTSG
  2 Armadillidium_vulgare_iridescent_virus_YP_009046748.1  17.6%    -MSISSSNVTSGFI--DIATKDEIEKYMYG------GKTST-AYFV---RETRKSTWFTQVPVSL--------TRANGFP
  3 Cedratvirus_A11_YP_009329068.1                         45.2%    --MQEQQCSLIRMITEDETEHEKIIRYKSGDDKKVDEVTSLFDFNP---KEYA---RNHHLPREM--------ARGERSG
  4 Cedratvirus_lausannensis_SOB74375.1                    45.5%    --MQEQQCSLIRMITEDETEHEKIIRYKSGDDKKVDEVTSLFDFNP---KEYA---RNHHLPREM--------ARGERSG
  5 Cherax_quadricarinatus_iridovirus_ASZ84981.1           16.7%    -KNCKMANIAGALQ--DMANLGAVERYQYG------TTNAV-TYFI---RETRKSTLFSQLPIQL--------SSKNGNP
  6 Invertebrate_iridescent_virus_30_YP_009010370.1        14.8%    -MSMSSSNITSGFI--DIATFDEIEKYMYG------GPTAT-AYFV---REIRKSTWFTQVPVPL--------SRNTGNA
  7 Invertebrate_iridovirus_22_YP_008357369.1              14.8%    -MSMSSSNITSGFI--DIATFDEIEKYMYG------GPTAT-AYFV---REIRKSTWFTQVPVPL--------SRNTGNA
  8 Invertebrate_iridovirus_25_YP_009010611.1              15.6%    -MSMSSSNITSGFI--DIATFDEIEKYMYG------GPTAT-AYFV---REIRKSTWFTQVPVPL--------SRNTGNA
  9 Orpheovirus_IHUMI-LCC2_YP_009449240.1                  14.4%    -MNSNDINNNERAI--QNALFYHLYKKHDN------ENSPISSFAPTPERSLIYSPSSHIVPHYLCVDKAKTEQNKTGLN
 10 Melbournevirus_YP_009094806.1                          14.9%    -ICGTRVTAASGFV--DLATFSDLEAYLYG------GCSAV-TYFV---RAIKKANWFSFLPVVL--------RNISGLP
 11 Marseillevirus_marseillevirus_YP_003407071.1           14.9%    -ICGTRVTAASGFV--DLATFSDLEAYLYG------GCSAV-TYFV---RAIKKANWFSFLPVVL--------RNISGLP
 12 Brazilian_marseillevirus_YP_009238902.1                14.9%    -VCGTRVTAASGFV--DLATFSDLEAYLYG------GCSAV-TYFV---RAIKKANWFSFLPVVL--------RNISGLP
 13 Golden_marseillevirus_YP_009310340.1                   12.3%    -VSGTRVTAASGFV--DLATFSDLEAYLYG------GCSAV-TYFV---RAIKKANWFSFLPVVL--------RNIAGLP
 14 mine_drainage_metagenome_EQD26795.1                    21.6%    -AHVDELNNLHMFY--DCAHTPQKSGEVLG------DISSF-LY-----QKYIPTTWNVIYSKKM--------DTI----
 15 Pithovirus_sibericum_YP_009001361.1                    95.1%    -MDGDQRDVLSQMVTEDETEYEKALRYSEE------GCQSLFDYAV------RKPSRNHHFPREM--------KRADTSG
 16 Shrimp_hemocyte_iridescent_virus_ATE87157.1            16.7%    -KNCKMANIAGALQ--DMANLGAVERYQYG------TTNAV-TYFI---RETRKSTLFSQLPIQL--------SSKNGNP
 17 Wiseana_iridescent_virus_YP_004732793.1                15.0%    -MSMSSSNITSGFI--DIATFDEIEKYMYG------GPTAT-AYFV---REIRKSTWFTQVPVPL--------SRNTGNA
 18 PCJ29163.1_COA94_02315_Rickettsiales                   22.3%    MTPGNVSNLESD----GWSSFQKRLHTIES------KGETIPSKFS---HQATKISWSTPVVREV----------ASRNT
    consensus/100%                                                  ....p..s........t.s.h.th.t...t......t.psh..h........h.......hsh.h...............
    consensus/90%                                                    ...p..s...th...p.sph.th.th..t......t.psh.sa..   ...h...h.p.hsh.h        tp.tt.s
    consensus/80%                                                    hshpp.s.htthh..D.sphtch.tY..u      tssuh.sahs   +ththssh.p.lPh.h        pp.st.s
    consensus/70%                                                    hshsp.shtuthl  DhApapcl.+Yh.G      ssouh.sYhl   +th+Kssh.s.lPh.l        pptss.s

                                                                81          .         1         .         .         .         .         :         . 160
  1 Pithovirus_massiliensis_LC8                           100.0%    SY--VYLADPEYS--------FLYDMQMYLELPKVVVKKKY---------RDNVQIAMCSYPGINVVTQATMYCGKKKSP
  2 Armadillidium_vulgare_iridescent_virus_YP_009046748.1  17.6%    NFGSEWSASVSRAGD------YLLYTWLRLRLPAVTLLPTNQFFNVPVAGANNGRIRWTKNFMHNLIRECSITF-NDLVA
  3 Cedratvirus_A11_YP_009329068.1                         45.2%    QY--EYNADSSYS--------YLYEMTLYQVLPQVTVKKKY---------RDRIEICWAHYPGLNVVKNAIMFGGEKTVI
  4 Cedratvirus_lausannensis_SOB74375.1                    45.5%    QY--EYSADSSYS--------YLYEMTMYQVLPKVTVKKKY---------RDRIEICWAHYPGLNVVKNAIMFGGEKTVI
  5 Cherax_quadricarinatus_iridovirus_ASZ84981.1           16.7%    DFDREWSVEPSKAFD------YLIHMWIRVTVPEVKLLAGNVY-------KEHGRIRWTRNFMHNLIKKVSFNV-NDLEI
  6 Invertebrate_iridescent_virus_30_YP_009010370.1        14.8%    AFGQEWSVSISRAGD------YLLQTWLRVNIPQVTLNPLL---------ANTFALRWTRNLMHNLIREATITF-NDLVA
  7 Invertebrate_iridovirus_22_YP_008357369.1              14.8%    AFGQEWSVSISRAGD------YLLQTWLRVNIPQVTLNPLL---------ANTFALRWTRNLMHNLIREATITF-NDLVA
  8 Invertebrate_iridovirus_25_YP_009010611.1              15.6%    AFGQEWSVSISRAGD------YLLQTWLRVNIPQVTLNPLL---------AATFSLRWTRNLMHNLIREATITF-NDLVA
  9 Orpheovirus_IHUMI-LCC2_YP_009449240.1                  14.4%    RLHELYDKTLSYNLNHISDADFIIAAILEQVLPRTEVKRKY---------VGMIEVCWMPNIGNNIPQSFYLKYASDVNF
 10 Melbournevirus_YP_009094806.1                          14.9%    GFGSEFSASVNRSGD------YVLNTWLRVRLPLVAIRPTNTGGAI----NANATIRWTRNFMHNLVEKVNITF-NDLIV
 11 Marseillevirus_marseillevirus_YP_003407071.1           14.9%    GFGSEFSASVNRSGD------YVLNTWLRVRLPLVAIRPTNTGGAI----NANATIRWTRNFMHNLVEKVNITF-NDLIV
 12 Brazilian_marseillevirus_YP_009238902.1                14.9%    GFGSEFSASVNRSGD------YVLNTWLRVRLPLIAIRPTNAGGAI----NANATIRWTRNFMHNLVEKVNITF-NDLIV
 13 Golden_marseillevirus_YP_009310340.1                   12.3%    GFGSEFSASVNRSGD------YVLNTWLRVRVPLIAIRPTNGAGAI----NANATIRWTRNFMHNLVEKINITF-NDLIV
 14 mine_drainage_metagenome_EQD26795.1                    21.6%    AVGSNVNYLIDSNSH------SLLSSRMVFKVPPIKVKEEF---------NDKIKIAWTHNLGHNRILDAEFIC-DGIIY
 15 Pithovirus_sibericum_YP_009001361.1                    95.1%    SF--MYAADSEYS--------FLYDMQMYLKLPKVVVKKKY---------RDNVQIAMCSYPGINVVTQAAMYCGKKKSP
 16 Shrimp_hemocyte_iridescent_virus_ATE87157.1            16.7%    DFDREWSVEPSKAFD------YLIHMWIRVTVPEVKLLAGNVY-------KEHGRIRWTRNFMHNLIKKVSFNV-NDLEI
 17 Wiseana_iridescent_virus_YP_004732793.1                15.0%    AFGQEWSVSISRAGD------YLLQTWLRVNIPQVTLNPLL---------AATFSLRWTRNLMHNLIREATITF-NDLVA
 18 PCJ29163.1_COA94_02315_Rickettsiales                   22.3%    RSGSTYEADMNCD--------FLSNSYMTLDLPQITVGDEY---------LESVQVCWTPNIFHNIIESGGLYM-NGYKY
    consensus/100%                                                  t....h.h..p.s.........l....h...lP.h.l..............t.htlthh...h.Nh..ph.h.h.pth..
    consensus/90%                                                   th...athp.phs..      alht..hh..lP.lhl.........    .tphtlths...hhNllpph.h.h.pth..
    consensus/80%                                                   ta..passs.shu..      alhphhhhhplP.ltlt.h......    tsphplpWsp..hhNllppsthhh.pch.h
    consensus/70%                                                   saspEauss.spu.p      YLlphhlplplPtVslp.t...       tsphplpWT+NhhHNllcpsshhh sclhh

                                                               161          .         .         .         2         .         .         .         . 240
  1 Pithovirus_massiliensis_LC8                           100.0%    YHFDQTWFNIYNQFY----NKKTEHFNRYCGHIPALTD---W-----GCSLPSHQ---LQVQQPWFFTRE-GFPFPLFRV
  2 Armadillidium_vulgare_iridescent_virus_YP_009046748.1  17.6%    ARFDNYHLDFWAAFT--TPASKAVGYDNMIGNISALIQPQPPPVAPATVSLPEID---LNLPLPFFFSRDSGVSLPTAAL
  3 Cedratvirus_A11_YP_009329068.1                         45.2%    QTSDSKWSNIDNQFY---GNKKIDHINHYLGHVPALIE---W-----THKLPKFP---LRVLQPWSFCK--GVPFPLFKA
  4 Cedratvirus_lausannensis_SOB74375.1                    45.5%    QTSDSKWSNIDNQLY---GNKKIDHINHYLGHVPALIE---W-----THKLPKFP---LRVLQPWSFCK--GVPFPLFKA
  5 Cherax_quadricarinatus_iridovirus_ASZ84981.1           16.7%    EKFDNYFLDFWNQFT--LSSSKKDGYNNMIGNDDDLLIPKSK-----DGKIESKS---LTLPIPFFFSRDSGLALPVGGV
  6 Invertebrate_iridescent_virus_30_YP_009010370.1        14.8%    ARFDNYHLDFWSAFT--VPASKRNGYDNMIGNVSALINPVAP-----GAALGPTGGTNLNLPLPFFFSRDTGVALPTAAL
  7 Invertebrate_iridovirus_22_YP_008357369.1              14.8%    ARFDNYHLDFWSAFT--VPASKRNGYDNMIGNVSALINPVAP-----GAALGPTGGTNLNLPLPFFFSRDTGVALPTAAL
  8 Invertebrate_iridovirus_25_YP_009010611.1              15.6%    ARFDNYHLDFWSAFT--VPASKRTGYDNMIGNVSALINPVAP-----GGSLGSTGGTNLNLPLPFFFSRDTGVALPTAAL
  9 Orpheovirus_IHUMI-LCC2_YP_009449240.1                  14.4%    SRKDSVDMDNFLSWC----IRNKHNYNKHIGNVPELTE---W-----NTILPRYE---LGILLPACFSDGGPTALPIFLG
 10 Melbournevirus_YP_009094806.1                          14.9%    HEFDSYWFDFNSQFN--IDASKRVGYRNMIGDIPAMINPVTT-----GNPLGTGE--FFNLPIPLFYTEDSGLALAVSAL
 11 Marseillevirus_marseillevirus_YP_003407071.1           14.9%    HEFDSYWFDFNSQFN--IDASKRVGYRNMIGDIPAMINPVTT-----GNPLGTGE--FFNLPIPLFYTEDSGLALAVSAL
 12 Brazilian_marseillevirus_YP_009238902.1                14.9%    HEFDSYWFDFNSQFN--IDASKRVGYRNMIGDIPAMINPVTT-----GNPLGTGE--FFNLPIPLFYSEDSGLALAVSAL
 13 Golden_marseillevirus_YP_009310340.1                   12.3%    HEFDNYWLDFNAQAN--IDASKRIGYRNMIGDIPAMINPVTT-----GNPLGTGD--FFNLPVPLFYSEDSGLALAVSAL
 14 mine_drainage_metagenome_EQD26795.1                    21.6%    TRFDNVWLDFESQFFQDGGAGKRKSRQNGIGNIPALEE---F-----SNKLPEFT---LDVWQPWFYSMHSFSAFPIFFR
 15 Pithovirus_sibericum_YP_009001361.1                    95.1%    YQFDQTWFNIYNQFY----NKKTEHFNRFCGHIPTLTD---W-----GYSLPSHQ---LQVQQPWFFTRE-GFPFPLFRV
 16 Shrimp_hemocyte_iridescent_virus_ATE87157.1            16.7%    EKFDNYFLDFWNQFT--LSSSKKDGYNNMIGNDDDLLIPKSK-----DGKIESKS---LTLPIPFFFSRDSGLALPVGGV
 17 Wiseana_iridescent_virus_YP_004732793.1                15.0%    ARFDNYHLDFWSAFT--VPASKRTGYDNMIGNVSSLINPVAP-----GGNLGSTGGTNLNLPLPFFFSRDTGVALPTAAL
 18 PCJ29163.1_COA94_02315_Rickettsiales                   22.3%    QNVDKYWLDIHSQLITTSSESKRNAYMKNIGNKAALCD---W-----SCNIEGDE---LFLFIPWFFTENSVLSLPLLLI
    consensus/100%                                                  .p.Dph..s...th......tph.th.p.hGp.sth...........s..l.......h.l..Ph.as.....shsh.hh
    consensus/90%                                                   .p.Dpha.sh.sth.....tpKh.thpphhGp.sshh.....     s..l..h....htl..Ph.asc..shshsh.hh
    consensus/80%                                                   tphDshahsh.sta.  .sspKp.tapphlGplsshhp....     ss.Lspht...hpl..PhFaocp.Ghshsh.th
    consensus/70%                                                   tcFDshahDh.spFh  .ssuK+suYpphIGslsALlp...h     sstLsstt  .Lsl.lPaFFoc-oGluLPlhtl

                                                               241          :         .         .         .         .         3         .         . 320
  1 Pithovirus_massiliensis_LC8                           100.0%    KE---SVQFEYTFKKSIFDLLRFKIRAE-GEDW-KEVPFHKNFIEFVSVT--DGGILSPPIMIGEYSKITIEEEQ-AIKN
  2 Armadillidium_vulgare_iridescent_virus_YP_009046748.1  17.6%    PYNEMRINFQFRDWDQLLILDNINSAAS-QS-V-VPVVGAAADIA-------QAPVISHAAVWGNYAIVSNEERR-RMGC
  3 Cedratvirus_A11_YP_009329068.1                         45.2%    KE---KIYFSYKTCSSIFELLRVRVLKD--GQWTYISPTKEANTISKYLNFPDKGSLPSPIMIAEYSKITHDEEM-SIKG
  4 Cedratvirus_lausannensis_SOB74375.1                    45.5%    KE---KIYFSYKTCSSIFELLRVRVLKD--GQWSCISPTKEANNISKYLNFPDKGSLPSPIMIAEYSKITHDEEM-SIKG
  5 Cherax_quadricarinatus_iridovirus_ASZ84981.1           16.7%    KWNKLRIDFEFRNWTELLILENVGAAHN-GEKNPCKVPQVGSDIA-------VAPSLSNVQCWVNGGLIPEAERA-RMGC
  6 Invertebrate_iridescent_virus_30_YP_009010370.1        14.8%    PYNEMQINFNFRDWTELLVLTNSALVPP-ASPY-VPIV-VPTHLT-------TAPVLGPVQVWANYAIVSNEERR-RMGC
  7 Invertebrate_iridovirus_22_YP_008357369.1              14.8%    PYNEMQINFNFRDWTELLVLTNSALVPP-ASPY-VPIV-VPTHLT-------TAPVLGPVQVWANYAIVSNEERR-RMGC
  8 Invertebrate_iridovirus_25_YP_009010611.1              15.6%    PYNEMQINFNFRDWTELLVLQNSALVAP-ASPY-VPIV-VPTHLT-------TAPVLGPVQVWANYAIVSNEERR-RMGC
  9 Orpheovirus_IHUMI-LCC2_YP_009449240.1                  14.4%    GKNS-NFELIINIRNRLSNLLRMRRKGK-DGVWQEIRANTKYLVGFK-----KEESISLPTLRCEYTTDIGGYKESILCQ
 10 Melbournevirus_YP_009094806.1                          14.9%    PFNDIKINYCLRRWQDLIVL-NVGVGGN-PPTY-DDIVQVSYDSTFTLIYSSNAPAITNVETWCHYAVVHNDERV-KMGK
 11 Marseillevirus_marseillevirus_YP_003407071.1           14.9%    PFNDIKINYCLRRWQDLIVL-NVGVGGN-PPTY-DDIVQVSYDSTFTLIYSSNAPAITNVETWCHYAVVHNDERV-KMGK
 12 Brazilian_marseillevirus_YP_009238902.1                14.9%    PFNDIKINYCLRRWQDLLVL-NVGVGAN-PPTL-DDVVQVSYDAAFTLIYSSNAPAITNVETWCHYAVVHNDERV-KMGK
 13 Golden_marseillevirus_YP_009310340.1                   12.3%    PFNDIKINYTLRRWQDLLVL-NTGVGAN-PPTY-DDVVQVSYDSTFTLIYSTSAPALTNVETWCHYAVVHNDERV-KMGK
 14 mine_drainage_metagenome_EQD26795.1                    21.6%    NSQS-RIVHKYTLQDKIGNLLRMKKYNEKKKKWIDEKTSEWNDHLKDYLEL-DSETLEVPELWGRYAMVTEQELNRRKSC
```

```
15 Pithovirus_sibericum_YP_009001361.1                 95.1%    KE---SVQFEYTFKKSIFDLLRFKIRAE-GEDW-EEVPFHKNFIEFVSVA--DGGILSPPIMIGEYSKITIEEEQ-AIKN
16 Shrimp_hemocyte_iridescent_virus_ATE87157.1         16.7%    KWNKLRIDFEFRNWTELLILENVGAAHN-GEKNPCKVPQVGSDIA-------VAPSLSNVQCWVNGGLIPEAERA-RMGC
17 Wiseana_iridescent_virus_YP_004732793.1             15.0%    PYNEMQINFNFRDWTELLVLQNSALVAP-ASPY-VPIV-VPTHLT-------VAPVLGPVQVWANYAIVSNEERR-RMGC
18 PCJ29163.1_COA94_02315_Rickettsiales                22.3%    KD---SVKFEFKFNLNFSSLIRMRIKGE-NGNWECIKYDSKYLKGIN-----DKQRIPSPQMFAKYLMILESERKWRLSE
   consensus/100%                                               .....ph.h.hp...ph..L.p.t.h.........................t..l..s.hhsph.h...t.....htt
   consensus/90%                                                .....pl.aphp..ppl..L.p.thh.. ........s..............t..ls.s.hhsphshl..tEc..thtt
   consensus/80%                                                .....plpaphp.hpplh.L.p.thht. ...h...hs.h....t......stsslsss.hhspYuhl.ppEc. phtp
   consensus/70%                                                ..pp.pIpapa+phpplhsL.phtlhss ss.a.s.ls.sthphs......susslsssphaspYuhlpp-E+h +hup

                                                            321       .         .         :         .         .         .         .        4 400
 1 Pithovirus_massiliensis_LC8                        100.0%    NDKH---------CAVEDILTFKTELVD--KVTPLAQVQIKTKYPLKGIFYVAENVDATRLN---------NFSNFSTCS
 2 Armadillidium_vulgare_iridescent_virus_YP_009046748.1 17.6%  SVRD---------ILIEQVQTAPRHVYN-PNTNDEPSYDIRFSHAIKALFFAVRN--TTFKN---------VWSNYTTAS
 3 Cedratvirus_A11_YP_009329068.1                      45.2%    DMDH---------YVVEDIKRFSSEIVN--KTNPKVTIEVDIEDSIKGIFYVAENLDATLTN---------NLSNFSTNA
 4 Cedratvirus_lausannensis_SOB74375.1                 45.5%    DMDH---------YVVEDIKRFSSEIVN--KTNPKVTIEVDIEDSIKGIFYVAENLDATLTN---------NLSNFSTNA
 5 Cherax_quadricarinatus_iridovirus_ASZ84981.1        16.7%    VHRD---------MLIESIQTSSKLNFN-PVLNPNPSYDIRFQRTVKALFFGVRN--TTNPN---------VWSNYTTAS
 6 Invertebrate_iridescent_virus_30_YP_009010370.1     14.8%    AIRD---------ILIEQVQTAPRQNYT-PLTNASPTFDIRFSHAIKALFFSVRN--KTGAS---------EWSNYATSS
 7 Invertebrate_iridovirus_22_YP_008357369.1           14.8%    AIRD---------ILIEQVQTAPRQNYT-PLTNASPTFDIRFSHAIKALFFSVRN--KTGAS---------EWSNYATSS
 8 Invertebrate_iridovirus_25_YP_009010611.1           15.6%    AIRD---------ILIEQVQTAPRQNYT-PLTNASPTFDIRFSHAIKALFFSVRN--KTSAS---------EWSNYATSS
 9 Orpheovirus_IHUMI-LCC2_YP_009449240.1               14.4%    GVLV---------QYIREVINLDRDETA--RLGSTVKIPINKPGLSQAIFVTSSAVSLHKPTSKDTSLVPLNYSNYTTNI
10 Melbournevirus_YP_009094806.1                       14.9%    NPRD---------MVIKQVQKVNETTINLSQLNALVPIDIRVSHAVVGYFYAIRN--SSTTG---------EWSNYTT--
11 Marseillevirus_marseillevirus_YP_003407071.1        14.9%    NPRD---------MVIKQVQKVNETTINLSQLNALVPIDIRVSHAVVGYFYAIRN--SSTTG---------EWSNYTT--
12 Brazilian_marseillevirus_YP_009238902.1             14.9%    NPRD---------MVIKQVQKVNETTINLSQLNALIPIDIRISHAVVGYFYAIRN--SSTPG---------EWSNYTT--
13 Golden_marseillevirus_YP_009310340.1                12.3%    NPRD---------MVIKQVQKVNETTINLSQLNALLPIDIRISHAVVAYFFAIQNSSDSRR----------VVQLHHRTP
14 mine_drainage_metagenome_EQD26795.1                 21.6%    FKIDKEGKYKDEVLFIRDVIACDDINPS--KFGSYSSIDLTTSNPCLAIFWSAENNTAKSYN---------YHSNYTTEA
15 Pithovirus_sibericum_YP_009001361.1                 95.1%    NDKH---------CAVEDILSFKTELVD--KVTPLAQVQIKTKYPLKGIFYVAENVDATRLN---------NFSNFSTCA
16 Shrimp_hemocyte_iridescent_virus_ATE87157.1         16.7%    VHRD---------MLIESIQTSSKLNFN-PVLNPNPSYDIRFQRTVKALFFGVRN--TTNPN---------VWSNYTTAS
17 Wiseana_iridescent_virus_YP_004732793.1             15.0%    AIRD---------ILIEQVQTAPRQNYT-PLTNASPTFDIRFSHAIKALFFSVRN--KTSAS---------EWSNYATSS
18 PCJ29163.1_COA94_02315_Rickettsiales                22.3%    SSHK---------IYARDMVRVSSMVVR--EGGDRVDININCSNPITHIYWTAENMEQNKLN---------NLSNYTTSG
   consensus/100%                                               .............hhcph.t..p...t...hss...h.lph.....thahs.ps...p.............hp.atp..
   consensus/90%                                                s..c         hhlcpl.p..p..hs...hss...hplphp.shhuhFashpN..tp..s         .hSNasT..
   consensus/80%                                                s.cc         hhlcpl.phsp..hs...hss.sshpIchppslhuhFashcN..tot.s         .hSNasTt.
   consensus/70%                                                s.+c         hlIcplppsscpshs ..hNs.ssh-I+hpcslpulFasscN..tophs         paSNYoTsu

                                                            401       .         .  ] 423
 1 Pithovirus_massiliensis_LC8                        100.0%    ----------EDMSEGVSPICSV
 2 Armadillidium_vulgare_iridescent_virus_YP_009046748.1 17.6%  QVVTPTTVIFEPSTGAFDPIDHT
 3 Cedratvirus_A11_YP_009329068.1                      45.2%    ----------TEILDGDKPIKGI
 4 Cedratvirus_lausannensis_SOB74375.1                 45.5%    ----------TEILDGDKPIKGI
 5 Cherax_quadricarinatus_iridovirus_ASZ84981.1        16.7%    PVPDADKIDFDPDQSAFDPIGTA
 6 Invertebrate_iridescent_virus_30_YP_009010370.1     14.8%    PVVIGSTVNFEPA-GSFDPIANT
 7 Invertebrate_iridovirus_22_YP_008357369.1           14.8%    PVVTGSTVNFEPA-GSFDPIANT
 8 Invertebrate_iridovirus_25_YP_009010611.1           15.6%    PVVTGATVNFEPA-GSFDPIANT
 9 Orpheovirus_IHUMI-LCC2_YP_009449240.1               14.4%    ----------YDVKSGYSPIEYI
10 Melbournevirus_YP_009094806.1                       14.9%    ----------EPAYAGLDPLEAA
11 Marseillevirus_marseillevirus_YP_003407071.1        14.9%    ----------EPAYAGLDPLEAA
12 Brazilian_marseillevirus_YP_009238902.1             14.9%    ----------EPAYAGLDPLEAA
13 Golden_marseillevirus_YP_009310340.1                12.3%    PMPVLTRSRLLSLCTSRLPVSAT
14 mine_drainage_metagenome_EQD26795.1                 21.6%    ----------GDSKIGYDPIKST
15 Pithovirus_sibericum_YP_009001361.1                 95.1%    ----------EDMSEGVSPICSV
16 Shrimp_hemocyte_iridescent_virus_ATE87157.1         16.7%    PVPDADKIDFDPDQSAFDPIGTA
17 Wiseana_iridescent_virus_YP_004732793.1             15.0%    PVVTGATVNFEPT-GSFDPIANT
18 PCJ29163.1_COA94_02315_Rickettsiales                22.3%    DESGKNPI--HKRSMSYGPLERD
   consensus/100%                                               ...............u..Plt..
   consensus/90%                                                ...............u.tPltth
   consensus/80%                                                ..........ps..tuhsPltss
   consensus/70%                                                ..........csh.suhcPItss
```
