# Supplementary material for: Deciphering viral presences: two novel partial giant viruses detected in marine metagenome and in a mine drainage metagenome
Source: Virol J. 2018 Apr 10;15:66. doi: 10.1186/s12985-018-0976-9 (PMC5891951; doi:10.1186/s12985-018-0976-9)
Supplement: Supplementary file 7 — Additional phylogenetic trees. Maximum likelihood trees were added based on VETF, mRNA capping enzyme and DNA topoisomerase II proteins. (PDF 744 kb) [file 12985_2018_976_MOESM7_ESM.pdf]

Phylogenetic trees based on the VETF, mRNA and DNA topoisomerase II proteins.

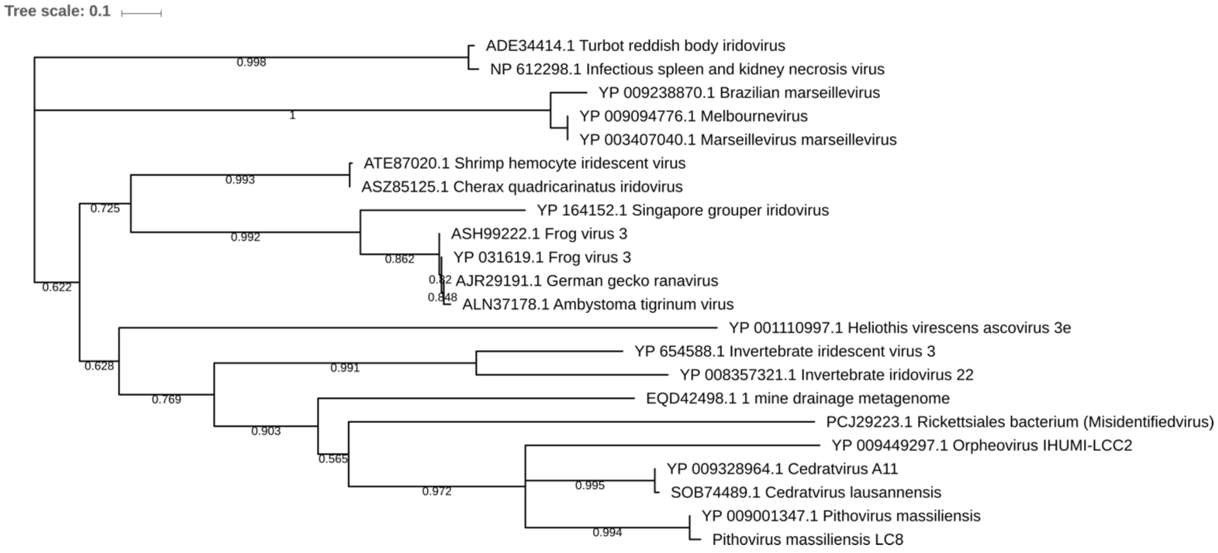

Figure S1: Phylogenetic tree based on the Very Early Transcription Factor protein.

Maximum-likelihood tree based on 454 positions. We deleted branches with a bootstrap value inferior at 0.5. Muscle program was used with standard parameter. The tree was built using FastTree program with JTT model.

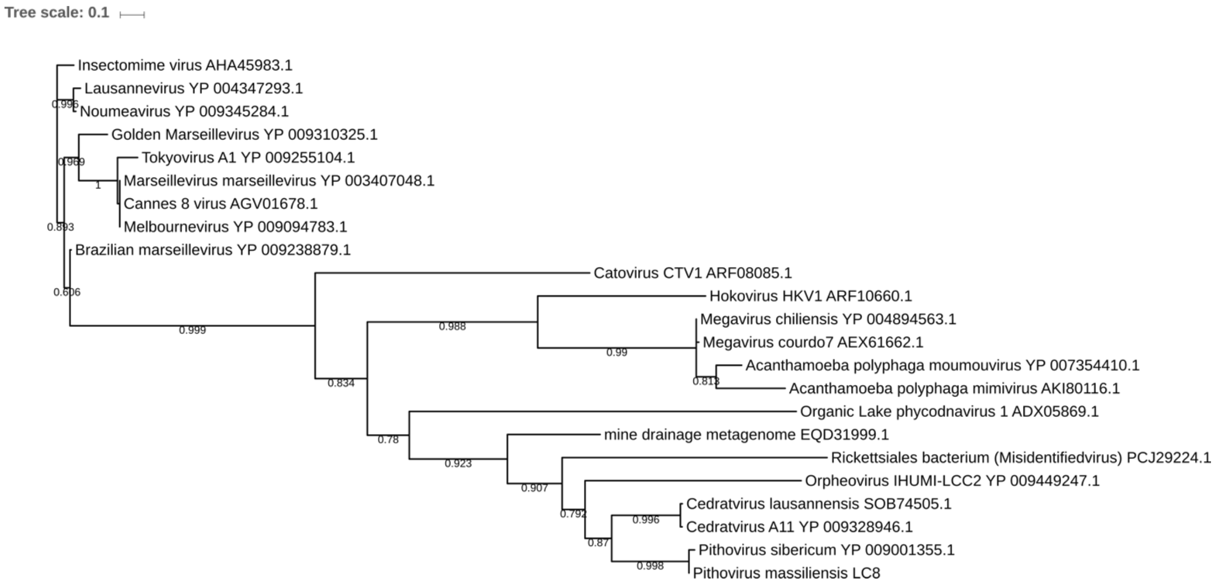

Figure S2: Phylogenetic tree based on the mRNA capping enzyme protein.

Maximum-likelihood tree based on 507 positions. We deleted branches with a bootstrap value inferior at 0.5. Muscle program was used with standard parameter. The tree was built using FastTree program with JTT model.

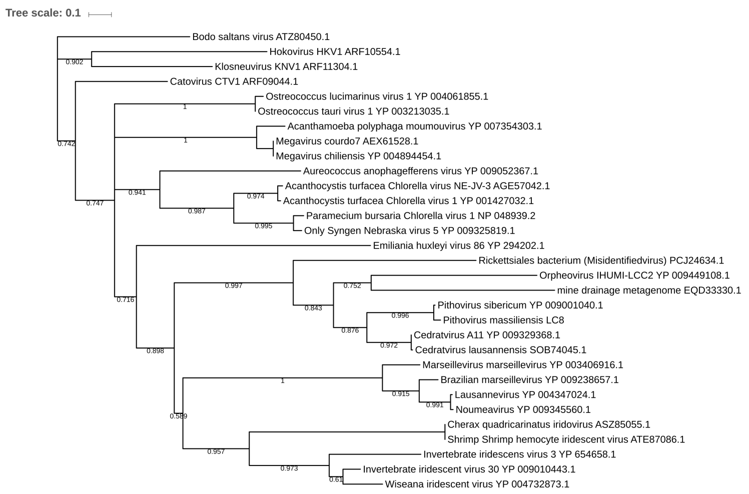

Figure S3: Phylogenetic tree based on the DNA topoisomerase IIA protein.

Maximun likelihood tree based on 280 positions. We deleted branches with a bootstrap value inferior at 0.5. Muscle program was used with standard parameter. The tree was built using FastTree program with JTT model.
